# Losing a Herd Mate: Negative Effects on Milk Yield and Udder Health Indicators in Loose-Housed Dairy Cattle

**DOI:** 10.3390/ani14233459

**Published:** 2024-11-29

**Authors:** Barbora Valníčková, Jitka Bartošová, Luděk Bartoš

**Affiliations:** Department of Ethology, Institute of Animal Science, 104 00 Praha, Czech Republic; valnickova.barbora@vuzv.cz (B.V.); bartos@vuzv.cz (L.B.)

**Keywords:** social environment, regrouping, dairy cow, milk yield, udder health, precision farming

## Abstract

Feral cattle (*Bos taurus*) live in socially stable herds. Due to farm management practices, intensively managed dairy cows experience frequent regrouping, which can disrupt affiliative bonds and induce social stress. This study examines how changes in herd composition affect milk yield and udder health traits in resident dairy cows exposed to the withdrawal of herd members or the introduction of new individuals. Milk yield, electrical conductivity, and blood presence in milk were measured in 798 cows using precision dairy sensors over 9 years. Milk yield decreased significantly (*p* < 0.001) one day after the removal of familiar herd members, while introducing cows had no effect. A higher number of cows being withdrawn (up to 10) was associated with reduced milk yield (*p* < 0.001), elevated milk electrical conductivity (*p* = 0.01), and an increased prevalence of blood presence in milk (*p* = 0.01), which can indicate potential udder health issues linked to social stress. Holstein Friesian, primiparous, and first-lactation-stage cows were more affected than Czech Simental and multiparous cows later in lactation. These findings underscore the influence of social stability on dairy cow welfare and productivity, emphasising the need for strategies to mitigate stress when herd changes are necessary.

## Simple Summary

Extensively managed and feral domesticated cattle (*Bos taurus*) live in a stable, complex social environment. Herds comprise members of various ages, genders, and sexual maturity. Under intensive farm management, dairy cows experience frequent and routine regrouping, which can disrupt affiliative bonds and induce social stress. This study explores the impact of changes in herd composition on milk yield and udder health traits in resident dairy cows that experience the removal of familiar herd members and the introduction of new individuals into the group. Data on milk yield (production trait), electrical conductivity, and blood presence in milk (indicators of udder health) were monitored over 9 years using precision dairy sensors in a sample of 798 cows. Milk yield dropped after the loss of familiar herd members while introducing new cows had no effect. The higher number of withdrawn cows (up to 10 at once) resulted in lower daily milk yield, increased milk conductivity, and a higher probability of blood presence in milk. Thus, besides lowering milk production, social stress is linked to potential udder health issues. Holstein Friesian cows and primiparous and early-lactation cows were more affected by herd changes than Czech Simental and multiparous cows in later lactation stages. These findings highlight the importance of social stability for dairy cow welfare and productivity, emphasising the need for strategies to reduce stress during necessary herd adjustments.

## 1. Introduction

In conditions without human intervention and management, cattle (*Bos Taurus*) females live in herds with a complex and stable social environment. New individuals are introduced to the herd within days after birth [1,2] without needing to establish a hierarchy immediately. New members enter the established herd hierarchy at the lowest level and gradually rise up the hierarchical ladder with time, which in a stable group is determined mainly by age [3,4]. Females spend their whole lives in their natal herd, and individuals usually leave the herd upon death. Foreign herds hardly ever merge [5]. Social stability in the cow herd allows females to form complex relationships [6,7] and build long-lasting affiliative relationships with their peers [8,9,10].

In contrast, the dairy industry has intensified through the years to meet growing food consumption demands and keep up with economic fluctuations [11,12]. Therefore, most dairy cattle are currently subjected to their farm’s limitations, needs, and procedures for optimal milk yield and economic revenue [13,14]. Housing dairy cattle on intensive farms without access to pasture (zero-grazing systems) presents numerous challenges for social animals kept in this manner. This impacts their welfare, namely, the fourth of the Five Freedoms (i.e., Freedom to express normal behaviour) that has been incorporated into farm animal welfare standards [15]. One of the challenges may be an unstable social environment that alters the establishment of affiliative relationships. At first, young calves are usually separated from their mothers within a few hours after birth, housed individually afterwards, and introduced to a group of peers only around 8 weeks [16]. In addition, female cattle are often regrouped and exposed to frequent social environment changes during their ontogeny and productive life [17,18].

However, despite these challenging changes in their social environment, cows create stable social relationships with other cows during their life on the farm to meet their natural social needs [9]. Although cows, after regrouping, find animals they know from the past and affiliate with them [9], it was documented that an unstable social environment takes its toll, and each group change results in agonistic interactions between the regrouped animals [18,19,20]. Regrouping might cause an association of individuals who do not suit each other or overstocking density. These factors can induce social instability and physical aggression, which might result in social stress [21]. Social stress may negatively affect emotions [22], as well as the behaviour, production, and health [23,24] of animals, such as an increase in agonistic interactions, decrease in social licking, reduced lying time, and decrease in dry matter intake, milk yield [25,26,27], or udder diseases [28]. Udder diseases can be detected by elevated milk conductivity, which is determined by the concentration of anions and cations. If the cow suffers from mastitis, the concentration of Na^+^ and Cl^−^ in the milk increases, which leads to the increased electrical conductivity of milk from the infected quarter [29,30]. These changes in behaviour, production, and health provide ample evidence regarding the disruptive effects of regrouping on animal welfare and performance [25,26,27]. Current research describes regrouping as a source of social stress in animals moved between groups, when unfamiliar groups are mixed [25,27,31], or when a group of cows receives new members [27]. There is a lack of studies examining resident animals’ reactions to the withdrawal of herd members from an established hierarchy, as well as comparisons of different types of social changes, such as the mate leaving the herd vs. incoming new herd mate, and their impact on resident animals’ responses.

This study aimed to determine if regrouping within herd turnover in a dairy farm affects milk production and health indicators of “resident” cows that are not moved but experience the loss of known animals or the addition of newcomers. Cows form long-term affiliative relationships in the environment of dairy farms with dynamic group management [9]. Therefore, we predicted that the withdrawal of an established group member and sudden cut of affiliative bonds, as a potentially stressful event, can negatively affect milk yield and indicators of mammary gland diseases such as milk electric conductivity and the presence of blood in milk in the resident animals which are exposed to a change in group structure but not moved.

## 2. Materials and Methods

### 2.1. Animals and Housing Management

All observations were conducted at the experimental farm of the Institute of Animal Science in Prague, Czech Republic, between 1 January 2013 and 26 May 2021. In total, 798 individual cows in the mixed group of two breeds (638 Holstein Friesian and 160 Czech Simental) were followed during this period. We obtained data from 1822 lactations in these cows: 645 times at 1st lactation and 1177 times from 2nd to 11th lactation. Cows were housed in a free-stall system that consisted of five barns for cows in different stages of lactation, i.e., delivery ward, early-lactation cow group, mid-lactation cow group, late-lactation cow group, and dry cow group. This study was conducted in a barn for early-lactation cows designed for up to 54 individuals. The barn operated below its maximal capacity, allowing sufficient space for all individuals.

All cows in our study were housed in groups from three months of age. They were inseminated between 13 and 18 months of age, and the insemination age depended on individual body weight and body condition. At 5 months of pregnancy, the heifers were moved to the group of dry cows, where they stayed until calving. Pregnant cows or heifers were isolated and placed in a straw-bedded calving pen right before the calving. After calving, primiparous and multiparous fresh cows were moved to the early-lactation cow group. Depending on their health status, the cows typically entered the group within the first week after parturition (median 6th day postpartum, modus 5th day postpartum). They stayed there usually until their milk yield dropped below 24 kg/day. After that, the cows were moved to the mid-lactation or late-lactation cows group, and visual and tactile contact with focal cows was lost. Cows were moved between barns by the decision of the farm manager, typically in pairs or small groups, but occasionally single individuals too.

On average, the cows stayed 76.19 ± 53.64 days in the observed group (median 65 days, range 1–295 days). During the observed period, the herd structure was changed 1014 times: 298 times, at least one animal was withdrawn from the group; 650 times, at least one cow was introduced to the group; and 66 times, both changes occurred within one day. Up to 10 cows were withdrawn or up to six were added during the focused social changes.

Cows were loosely housed in a zero-grazing system. The barn had net walls, a solid concrete floor, and cubicles bedded with straw. The interior space of the barn was divided into several functional zones: three rows of lying cubicles (54 in total, width 1.137 m, length 2.164 m, neck rail from the curb 1.066 m), centrally located drinking troughs, a feed bunk, and corridors. The barn management practices ensured good welfare conditions for the cows. The free-stall system enabled natural movement and social interactions among cows. Animals had unlimited access to a mechanical brush. Manure from the floor was removed twice daily during the milking time when cows were in the parlour. Straw in the cubicles was changed once a week. Cows had ad libitum access to water and were fed twice daily with a total mixed ration (TMR) based on corn silage, haylage, and straw with mineral and vitamin additives. Scattered TMR was pushed back to the feeding bunk nine times per day. Before every delivery of fresh feed, all remaining feed was removed. Cows were milked twice daily at 4 am and 4 pm in a tandem parlour with 5 × 5 stalls.

### 2.2. Data Collection

The experimental design fully respected farm management, and all focal animals were managed according to a commercial dairy farm’s standard practices and routine procedures. Milking data were automatically collected and processed by commercially available precision dairy monitoring systems created and produced by Afimilk Ltd. Kibbutz Afikim, Israel. The sensors in these systems have been designed to measure various variables for individual cows; AfiMilk MPCTM milk meters measure milk yield, electrical conductivity, and milking duration. To ensure accurate information on conductivity, 200 cubic centimetres of milk were checked while flowing through the milk meter. These sensors include AfiLab (Kibbutz Afikim, Israel), a tool that accurately estimates fat, protein, and lactose content and blood presence and concentration. The milk spectra for every 200 mL of milk passing through the machine are analysed during milking, and an average is calculated. Devices were installed in each milking stall, and data were collected during each milking session. Afitag sensors (Kibbutz Afikim, Israel) attached to the left hind legs of cows, with an ID number assigned to each cow, serve as identifiers for all cows in the group. Afitags were crucial in identifying the cow at the milking parlour, where milk production and components were measured. AfiLab and AfiMilk MPCTM milk meters collected data automatically during each milking session. Following each milking, each cow’s data and ID number were promptly scanned and transmitted to the central computer via the IDeal Afi radiofrequency antenna. Subsequently, the data were processed and stored using the dairy farm management software AfiFarm^TM^ version 4.1 [32].

The total amount of milk (grams), the highest values of electrical conductivity, the concentration of protein, fat, and lactose, blood presence, and the milking duration are noted in the AfiFarm^TM^ software after every milking. The software gives the final numbers of daily data after the last milking session every day. Information about calving, moving animals between the production groups, and entry and exit of the animal to/from the farm were added to the software manually by the farm manager according to the routine. The obtained dataset was processed with the software SAS 9.4 [33].

### 2.3. Statistical Analysis

The data were analysed using SAS for Windows version 9.4 (SAS Institute Inc., Cary, NC, USA). The UNIVARIATE procedure produced descriptive statistics for quantitative variables while FREQ was used for categorical variables. Three dependent variables were investigated: resident cows’ daily milk yield (kg/day), milk electrical conductivity (mmHO), and probability of blood presence in milk (yes/no). The effects of tested factors on resident cows’ daily milk yield and milk electrical conductivity were calculated via general linear mixed models (GLIMMIX procedure) with the normal distribution and identity as the link function. A generalised linear mixed model with binomial distribution and logit as link function was fitted for blood presence in milk. The identity of a cow on certain lactation entered the models as a random effect.

The predetermined models included the tested effects of the day (−1/0/1/2/3/4 days around the social change occurring at day 0) nested within the type of social change (withdrawal/arrival/both changes) and interactions between the type of social change and the cow’s parity (primiparous/ multiparous), lactation phase (up to 21 days/22+), and breed (Holstein Friesian/Czech Simental); furthermore, the number of added cows (up to 6) and number of withdrawn cows (up to 10) during the given social change were used as correlates. The decision to divide the lactation phase into 21 days postpartum and beyond was based on the consensus that cows are most susceptible to metabolic diseases and inflammation during the first three weeks post-calving [34,35]. Therefore, we assumed that cows might respond differently to social stress during this transition period than afterwards. The social changes occurred on day 0, between milking sessions or after the evening milking, so the effects were expected to occur on day 1 and beyond. Within-group least squares means were calculated for classes of tested categorical variables (LSMEANS statement). Tukey–Kramer adjustment was applied to correct multiple comparisons between means.

According to the high frequency of social changes in a herd of dairy cows on a commercial farm, the 6-day periods around a social change (days −1 to 4) were analysed when no other change appeared at least two days before and 4 days after. ’Resident cows’ fulfilled the criteria of being a member of the group for at least seven days and spending the whole observation period there.

## 3. Results

**Figure 1:**
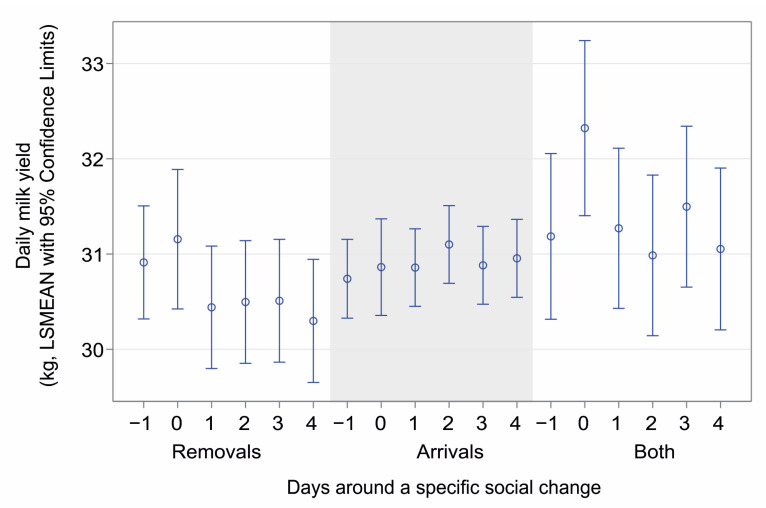
Daily milk yield in consecutive days around a social change (day 0) according to its type, i.e., removing cows from the group, introducing cows, or co-occurrence of removing and adding. Data are presented as LSMEANs with 95% confidence limits (general linear mixed model, PROC GLIMMIX, SAS).

None of the dependent variables statistically differed (*p* > 0.16) according to the type of social change on the day preceding the change (i.e., on the day −1). The type of social change (whether a cow or cows left the herd, a new cow/cows joined, or both occurred on the same day) had a significant impact on the daily milk yield of the resident cows (F_(15,28624)_ = 2.59, *p* < 0.001, GLMM, PROC GLIMMIX, SAS). The mean milk yield tended to decrease after the group had lost a member or members, while it was relatively stable when newcomers arrived (Figure 1). However, there was large individual variability among cows, especially when both changes had co-occurred.

**Figure 2:**
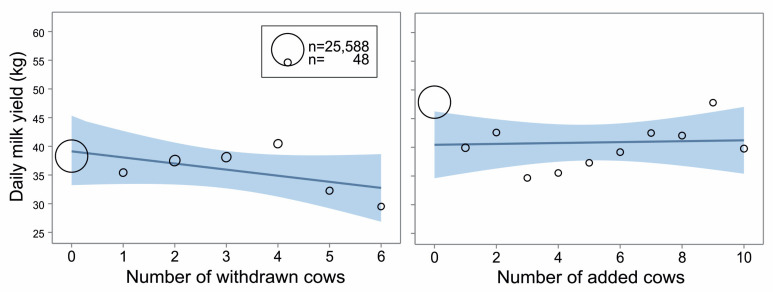
Daily milk yield over the observed period around a social change in the group of dairy cows according to the number of withdrawn (**left**) or added (**right**) cows during the change. The line and blue field show estimated daily milk yield ± 95% confidence limits (general linear mixed model, PROC GLIMMIX, SAS). The bubble positions represent the estimated milk yield for each number of cows, while their size is the number of observations.

Daily milk yield was not influenced by the number of cows being added to the herd (*p* = 0.60, Figure 2 right) but by the number of cows that were taken away from the group (F_(1,28624)_ = 8.01, *p* < 0.001, Figure 2 left); the more cows left, the lower the daily milk yield (slope = −0.12, *p* < 0.01).

**Figure 3:**
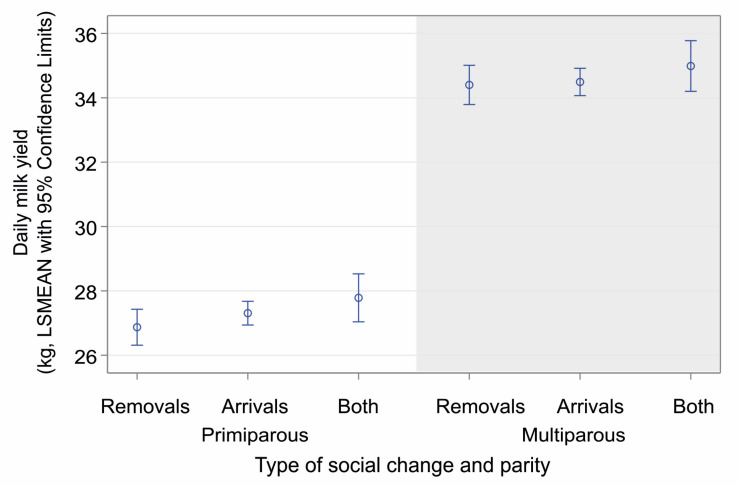
Daily milk yield in primiparous and multiparous cows according to the type of social change (only removals, arrivals, or both). Data are presented as LSMEANs with 95% confidence limits (general linear mixed model, PROC GLIMMIX, SAS).

**Figure 4:**
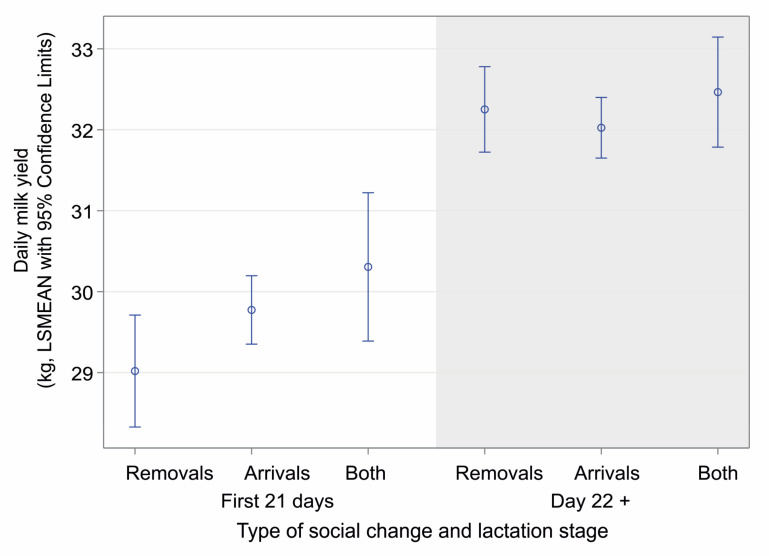
Daily milk yield in the postpartum stage (up to 21 days after giving birth) and later in lactation according to the type of social change. Data are presented as LSMEANs with 95% confidence limits (general linear mixed model, PROC GLIMMIX, SAS).

As expected, the parity, lactation phase, and breed factors fundamentally impacted milk yield, while the type of social change altered some of the mean levels. Primiparous cows (F_(3,28624)_ = 1571.72, *p* < 0.0001, Figure 3) and cows up to three weeks of lactation (F_(3,28624)_ = 179.18, *p* < 0.0001, Figure 4) gave less milk daily over the observed periods of 6 days than multiparous cows or cows at a later stage of lactation; cows always produced less milk after they experienced removal of a herd member compared to other types of social change (differences in LSMEANs were not statistically significant anymore after multiple comparison adjustment).

**Figure 5:**
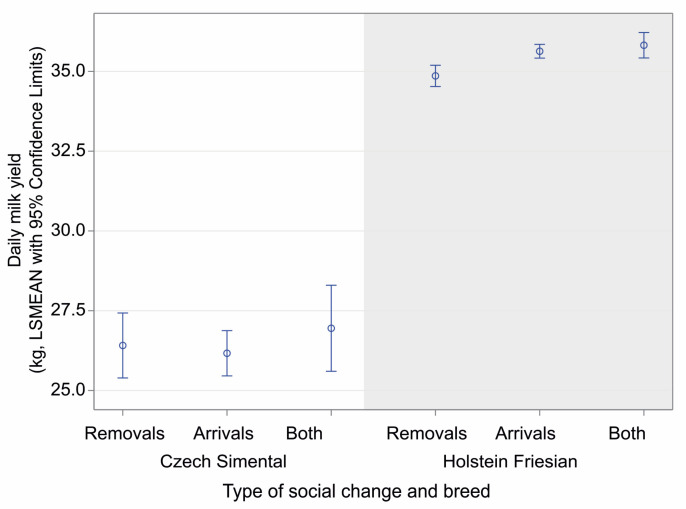
Daily milk yield over the observed period around a group change in the two observed cattle breeds, Czech Simental and Holstein Friesian, according to the type of social change. Data are presented as LSMEANs with 95% confidence limits (general linear mixed model, PROC GLIMMIX, SAS).

The breed also expressed other differences than higher daily milk yield in Holstein Friesian compared to Czech Simental cows (F_(3,28624)_ = 229.50, *p* < 0.0001, Figure 5). On average, Holstein Friesian cows showed 0.9 kg and 1.0 kg lower milk yield after the removal of a herd member compared to the arrival of new herd mates or mixed social change (34.78 ± 0.52 kg vs. 35.66 ± 0.11 kg, *p* < 0.0001, and 35.77 ± 0.20 kg, *p* < 0.001, respectively).

**Figure 6:**
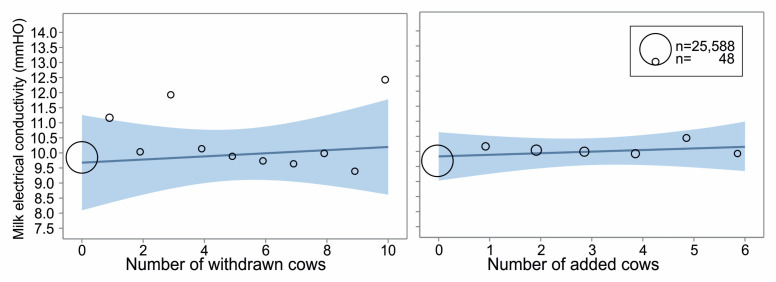
Milk electrical conductivity over the observed period around a social change in the group of dairy cows according to the number of withdrawn (**left**) or added (**right**) cows during the change. The line and blue field show estimated daily milk yield ± 95% confidence limits (general linear mixed model, PROC GLIMMIX, SAS). The bubble positions represent the estimated milk conductivity for each number of cows, while their size is the number of observations.

**Figure 7:**
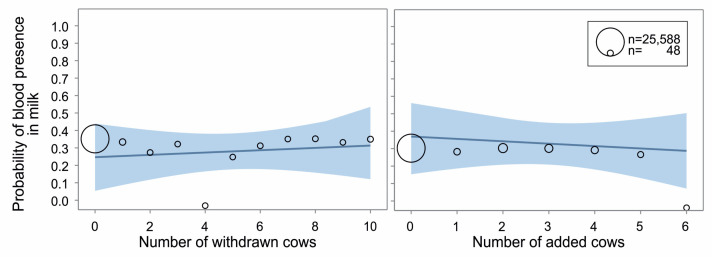
Probability of blood presence in milk over the observed period around a change in group structure according to the number of withdrawn (**left**) or added (**right**) cows during the change. The line and blue field show estimated probability ± 95% confidence limits (generalised linear mixed model, PROC GLIMMIX, SAS). The bubble positions represent the estimated probability of blood appearing in the milk for each number of cows, while their size is the number of observations.

Different types of social change did not directly induce statistically significant changes in milk electrical conductivity or the probability of blood in milk within consecutive days after the regrouping, not even in interaction with parity, lactation phase, or breed. Nevertheless, over the observed period, resident cows showed higher conductivity with an increasing number of withdrawn cows (slope = 0.012, F_(1,28580)_ = 6.56, *p* = 0.01, Figure 6 left), but not the number of added cows (*p* = 0.91, Figure 6 right). The probability of blood appearing in the milk revealed the same trend; an increased number of withdrawn cows induced a higher probability of blood in milk (slope = 0.054, F_(1,28753)_ = 6.47, *p* = 0.01, Figure 7 left), but not the number of added cows (*p* = 0.24, Figure 7 right).

## 4. Discussion

### 4.1. Effect of Social Changes on Milk Production and Udder Health Indicators

We found various effects of regrouping on milk yield and udder health parameters (milk conductivity and blood occurrence in milk) in resident cows, primarily associated with the withdrawal of a herd member or members. The milk yield in resident animals decreased a day after removing a conspecific from the group, but introducing new individuals did not affect it. On the contrary, previous studies described a decrease in milk yield after new introductions in resident cows [25,36], after cows were added to an established group [37,38], and after two groups merged [39,40]. All these studies induced a need for hierarchy reestablishment after regrouping that led to a more frequent agonistic behaviour [25,27,31], which resulted in social stress [21] and a subsequent decrease in milk yield [26]. On the other hand, regrouped cows often reconnect with familiar affiliates from the past if they find any in the new group [9,41]. In our study, primiparous and multiparous cows had the opportunity to interact before joining the focal group, as they were previously housed together in preparturient/dry cow groups or, for multiparous cows, in production groups during prior lactations. These potential pre-existing affiliative relationships may account for the absence of any negative impact on milk yield after adding new animals observed in this study. Additionally, differences between this and previous studies may be due to variations in sample size and experimental design. Our study (i) utilised the data collected over more than 8 years, (ii) included 1014 events of changes in group structure and 798 individual cows, and (iii) applied an experimental design that reflected routine farm practices and procedures. In contrast, other studies observed a significantly lower number of events from fewer animals selected on specific criteria and that implemented strictly controlled experimental designs and management practices.

The presence of affiliative relationships among the animals can also explain the decrease in resident cows’ milk yield following the removal of group members. So far, no published study has investigated milk performance and production traits in cows which remain in the group while others are shifted in/out. Only Walker et al. (2015) observed cattle remaining in the group after removing their conspecifics. They found reduced food intake and rumination, while elevated IgA concentrations in milk indicated a stress response to conspecific removal [42]. Decreased food intake [43,44,45] and rumination time decrease [46,47,48] can, among other factors, reduce milk yield.

In our study, the number of withdrawn but not added individuals mattered. The more animals left the group, the lower the milk yield, the higher the milk conductivity, and the more probable the presence of blood in the milk. Both elevated conductivity [49,50] and blood presence in milk [50] are indicators of udder illness, which can be linked to social stress [28]. Higher milk conductivity, among others, has been found to correlate with elevated plasma cortisol [51], a validated stress biomarker [52]. In feral cattle without human management, cows occasionally retreat from the herd. Such retraction is always temporary and driven by the need for isolation, typically for calving or when a cow becomes ill. It lasts a few hours to a few days, and retreated cows always keep visual contact with their herd [53]. Temporary and voluntary withdrawal of cows from their herd during sickness or calving differ from the motives and circumstances of group structure changes within farm cattle management. Many studies have suggested, in general, that artificial grouping and social environment changes during the cattle lifetime on farms are not in alignment with the social needs of the animals and are stressful [18,25,42]. According to our results, we can assume that losing a herd mate with whom the remaining animals had close relationships is stressful and compromises cows’ welfare. The resident cows in our study had known each other (they spent at least one week together), but we did not observe the strength of social bonds among the individuals. The social relationships should be subjected to detailed research; a new tool for automatic monitoring of social proximity among animal has been validated using ultra-wideband positioning system [54]. We suggest a more significant impact on resident cows, e.g., when losing a preferred social partner or partners (affiliates).

Further research should also be devoted to complex situations when both changes, i.e., withdrawal and addition of cows, coincide. Our data contained a lower number of such cases (66) than only removals (N = 298) or additions (N = 650), and it also revealed higher variability; the estimated means, however, mostly reached the highest values when both changes co-occurred. This may indicate the compensating potential of newcomers for resident cows experiencing social loss. Their social relationships, again, should be known and analysed to conclude.

### 4.2. Effect of Stage of Lactation, Parity, and Breed of Resident Cows on Reaction to a Social Change

As expected, primiparous cows and those within the first three weeks of lactation produced less milk daily over the observed six-day periods compared to multiparous cows or those later in lactation. Primiparous throughout first lactation and early-lactation cows in the transition period seemed more sensitive to removing a herd member than adding one. Primiparous and multiparous cows were differently affected when introduced to a lactating group after calving; according to the duration and synchronisation of lying behaviour, primiparas were strongly challenged by their entrance to the group while multiparas coped well with it [41]. After regrouping, new incomers found their affiliates from the juvenile period and showed physical closeness to the cows raised in the same age group [9]. Since cows form long-lasting affiliative relationships early in life, typically when they are calves under three months old [10,55], it is likely that primiparous cows—who are unaccustomed to group changes during lactation—lack familiar peers from their early years and older, recognisable cows in the production group. This absence may increase their stress and intensify their reactions to an unstable social environment, as they are without animals with whom they form affiliative bonds.

The intense reactions to social environment changes observed in resident cows at the beginning of lactation may be attributed to their elevated physiological reactivity to external factors and increased sensitivity to stress stimuli during the critical transition period of the first three weeks postpartum [35,56]. This high reactivity can influence their overall well-being. It may also affect various aspects of their production and health [56], as in this period, they are most sensitive to the negative energy balance and risk of subsequent metabolic diseases [35,57]. Understanding this sensitivity is crucial for developing management strategies that minimise stress during this vulnerable stage.

Compared to Czech Simental, Holstein Friesian cows reflected the removal of herd mates by lower milk yield. This may reflect lower stress resilience in Holsteins, as previous studies have shown higher plasma cortisol levels in Holsteins relative to other breeds [58] and lower milk yield under heat stress compared to Jersey and Simental cows [59].

## 5. Conclusions

This study highlights the significant impact of social dynamics within dairy herds on resident cows’ milk production and udder health indicators. The results indicate that removing established group members may result in a notable decline in milk yield. At the same time, the introduction of cows from the same farm does not significantly affect production. Additionally, the increase in milk conductivity and the likelihood of blood presence in milk following the removal of group members suggest a potential link between social stress and udder health. This aligns with the existing literature indicating that social stressors can negatively impact animal welfare and production. Furthermore, primiparous cows throughout the first lactation and those in the transition period exhibited heightened sensitivity to social changes, resulting in reduced milk yields compared to multiparous cows. This difference can be attributed to the lack of familiar peers or the stress of navigating an unstable social environment. All these findings underscore the importance of a stable social environment for dairy cattle, as familiar conspecifics might play a crucial role in maintaining productivity and good health.

Dairy producers need to consider the social structure of dairy herds when implementing management practices, as minimising group changes and fostering stable social relationships among dairy cows could lead to improved milk production and health. Future research should further explore the effects of social dynamics and the importance of affiliative relationships among cows on their welfare and production.

## Data Availability

The raw data presented in this study are included in the article/Supplementary Materials. Further inquiries can be directed to the corresponding author.

## Supplementary Materials

The following supporting information can be downloaded at https://www.mdpi.com/article/10.3390/ani14233459/s1, Supplementary Materials contain a file with raw data that entered the study. Table S1: Raw data table, including animal ID, date, age, lactation stage, parity, breed, and milk parameters.
