# Supplementary material for: Losing a Herd Mate: Negative Effects on Milk Yield and Udder Health Indicators in Loose-Housed Dairy Cattle
Source: Animals (Basel). 2024 Nov 29;14(23):3459. doi: 10.3390/ani14233459 (PMC11640356; doi:10.3390/ani14233459)
Supplement: Supplementary file 1 [file Table S1 Valnickova et al.pdf › Table S1 Valnickova et al.pdf]

Animal_ID	Group_ID	Date	Days_in_Milk	Age_Days	Lactation_Num	Conductivity	Blood_pct	yield	para	faze_lakt	plemeno	
2	1	12/23/2019	5	742	1	9.8	0.15	24.69	1	primi	1	H
2	1	12/24/2019	6	743	1	10	0.03	29.35	1	primi	1	H
2	1	12/25/2019	7	744	1	10	0	26.58	1	primi	1	H
2	1	12/26/2019	8	745	1	10	0.01	28.52	1	primi	1	H
2	1	12/27/2019	9	746	1	10	0	26.4	1	primi	1	H
2	1	12/28/2019	10	747	1	9.8	0.03	25.73	1	primi	1	H
2	1	12/29/2019	11	748	1	10.3	0.01	24.61	1	primi	1	H
2	1	12/30/2019	12	749	1	10.4	0.01	20.08	1	primi	1	H
2	1	12/31/2019	13	750	1	10.3	0	23.52	1	primi	1	H
2	1	01/01/2020	14	751	1	10	0.01	27.29	1	primi	1	H
2	1	01/02/2020	15	752	1	9.9	0	26.51	1	primi	1	H
2	1	01/03/2020	16	753	1	10.3	0.01	27.35	1	primi	1	H
2	1	01/04/2020	17	754	1	10	0	27.13	1	primi	1	H
2	1	01/05/2020	18	755	1	10.3	0.02	27.73	1	primi	1	H
2	1	01/06/2020	19	756	1	10	0	29.4	1	primi	1	H
2	1	01/07/2020	20	757	1	10	0.02	31.52	1	primi	1	H
2	1	01/08/2020	21	758	1	10.3	0	33.95	1	primi	1	H
2	1	01/09/2020	22	759	1	10.6	0	33.33	1	primi	2	H
2	1	01/10/2020	23	760	1	10.4	0.04	33.8	1	primi	2	H
2	1	01/11/2020	24	761	1	10.2	0	34.64	1	primi	2	H
2	1	01/12/2020	25	762	1	10.4	0.01	34.91	1	primi	2	H
2	1	01/13/2020	26	763	1	10	0.04	36.54	1	primi	2	H
2	1	01/14/2020	27	764	1	10	0.19	33.51	1	primi	2	H
2	1	01/15/2020	28	765	1	9.9	0	38.95	1	primi	2	H
2	1	01/16/2020	29	766	1	10	0.04	34.33	1	primi	2	H
2	1	01/17/2020	30	767	1	9.9	0.03	38.52				

1 primi	2	H						
2	1	01/18/2020	31	768	1	9.8	0	37.59
1 primi	2	H						
2	1	01/19/2020	32	769	1	10	0	37.78
1 primi	2	H						
2	1	01/20/2020	33	770	1	9.8	0	36.06
1 primi	2	H						
2	1	01/21/2020	34	771	1	9.9	0.06	41.61
1 primi	2	H						
2	1	01/22/2020	35	772	1	9.9	0.04	38.37
1 primi	2	H						
2	1	01/23/2020	36	773	1	9.9	0.06	38.5
1 primi	2	H						
2	1	01/24/2020	37	774	1	9.5	0.05	37.49
1 primi	2	H						
2	1	01/25/2020	38	775	1	9.8	0.06	38.18
1 primi	2	H						
2	1	01/26/2020	39	776	1	9.9	0.03	37.59
1 primi	2	H						
2	1	01/27/2020	40	777	1	9.6	0.07	39.2
1 primi	2	H						
2	1	01/28/2020	41	778	1	9.9	0.3	37.28
1 primi	2	H						
2	1	01/29/2020	42	779	1	9.8	0.01	40.42
1 primi	2	H						
2	1	01/30/2020	43	780	1	9.9	0.03	38.93
1 primi	2	H						
2	1	01/31/2020	44	781	1	10	0	40.49
1 primi	2	H						
2	1	02/01/2020	45	782	1	10.2	0.04	40.43
1 primi	2	H						
2	1	02/02/2020	46	783	1	9.9	0.03	39.83
1 primi	2	H						
2	1	02/03/2020	47	784	1	10.2	0.04	39.56
1 primi	2	H						
2	1	02/04/2020	48	785	1	10	0.01	39.52
1 primi	2	H						
2	1	02/05/2020	49	786	1	10.3	0.05	36.7
1 primi	2	H						
2	1	02/06/2020	50	787	1	10	0.04	42.82
1 primi	2	H						
2	1	02/07/2020	51	788	1	10.2	0.04	39.89
1 primi	2	H						
2	1	02/08/2020	52	789	1	10.2	0.05	39.68
1 primi	2	H						
2	1	02/09/2020	53	790	1	10	0.06	39.28
1 primi	2	H						
2	1	02/10/2020	54	791	1	10.2	0.02	39.02
1 primi	2	H						
2	1	02/11/2020	55	792	1	9.9	0.02	38.78
1 primi	2	H						
2	1	02/12/2020	56	793	1	9.9	0	36.08
1 primi	2	H						
2	1	02/13/2020	57	794	1	10.2	0	36.2

1 primi	2	H						
2	1	02/14/2020	58	795	1	10.3	0.04	38.37
1 primi	2	H						
2	1	02/15/2020	59	796	1	10	0.31	41.51
1 primi	2	H						
2	1	02/16/2020	60	797	1	10	0	40.57
1 primi	2	H						
2	1	02/17/2020	61	798	1	10	0.07	36.84
1 primi	2	H						
2	1	02/18/2020	62	799	1	10.2	0	37.76
1 primi	2	H						
2	1	02/19/2020	63	800	1	10.2	0	38.32
1 primi	2	H						
2	1	02/20/2020	64	801	1	10.2	0.02	38.62
1 primi	2	H						
2	1	02/21/2020	65	802	1	10.2	0	41.67
1 primi	2	H						
2	1	02/22/2020	66	803	1	10.4	0.02	37.98
1 primi	2	H						
2	1	02/23/2020	67	804	1	10.3	0.07	40.9
1 primi	2	H						
2	1	02/24/2020	68	805	1	10.3	0	39.63
1 primi	2	H						
2	1	02/25/2020	69	806	1	10	0.09	38.46
1 primi	2	H						
2	1	02/26/2020	70	807	1	10.2	0.08	39.1
1 primi	2	H						
2	1	02/27/2020	71	808	1	10.4	0	35.6
1 primi	2	H						
2	1	02/28/2020	72	809	1	10.6	0.09	36.01
1 primi	2	H						
2	1	02/29/2020	73	810	1	10.6	0.1	38.89
1 primi	2	H						
2	1	03/01/2020	74	811	1	10.6	0	40.03
1 primi	2	H						
2	1	03/02/2020	75	812	1	10.3	0.06	36.64
1 primi	2	H						
2	1	03/03/2020	76	813	1	10.3	0	38.43
1 primi	2	H						
2	1	03/04/2020	77	814	1	10.3	0.07	40.47
1 primi	2	H						
2	1	03/05/2020	78	815	1	10.3	0.07	39.03
1 primi	2	H						
2	1	03/06/2020	79	816	1	10.3	0.16	40.09
1 primi	2	H						
2	1	03/07/2020	80	817	1	10.6	0.06	41.18
1 primi	2	H						
2	1	03/08/2020	81	818	1	10.3	0.07	39.85
1 primi	2	H						
2	1	03/09/2020	82	819	1	10.4	0.1	41.15
1 primi	2	H						
2	1	03/10/2020	83	820	1	10.3	0	40.86
1 primi	2	H						
2	1	03/11/2020	84	821	1	10.4	0.07	40.14

1 primi	2	H						
2	1	03/12/2020	85	822	1	10.4	0	39.55
1 primi	2	H						
2	1	03/13/2020	86	823	1	10.4	0.06	39.9
1 primi	2	H						
2	1	03/14/2020	87	824	1	10.4	0	41.56
1 primi	2	H						
2	1	03/15/2020	88	825	1	10.3	0.06	41.39
1 primi	2	H						
2	1	03/16/2020	89	826	1	10.6	0	41.78
1 primi	2	H						
2	1	03/17/2020	90	827	1	10.3	0.06	38.94
1 primi	2	H						
2	1	03/18/2020	91	828	1	10.7	0	42.65
1 primi	2	H						
2	1	03/19/2020	92	829	1	10.4	0.07	39.38
1 primi	2	H						
2	1	03/20/2020	93	830	1	10.6	0	40.36
1 primi	2	H						
2	1	03/21/2020	94	831	1	10.4	0	39.87
1 primi	2	H						
2	1	03/22/2020	95	832	1	10.4	0.05	38.48
1 primi	2	H						
2	1	03/23/2020	96	833	1	10.2	0.16	39.3
1 primi	2	H						
2	1	03/24/2020	97	834	1	10.3	0	39.95
1 primi	2	H						
2	1	03/25/2020	98	835	1	10.4	0.06	40.42
1 primi	2	H						
2	1	06/03/2020	168	905	1	10.7	0.32	29.67
1 primi	2	H						
2	1	06/04/2020	169	906	1	11.1	0	31.22
1 primi	2	H						
2	1	06/05/2020	170	907	1	11.1	0	31.57
1 primi	2	H						
2	1	06/06/2020	171	908	1	11.1	0	33.02
1 primi	2	H						
2	1	06/07/2020	172	909	1	11.1	0	32.94
1 primi	2	H						
2	1	06/08/2020	173	910	1	10.7	0	35.66
1 primi	2	H						
2	1	06/09/2020	174	911	1	10.6	0	32.92
1 primi	2	H						
2	1	06/10/2020	175	912	1	10.9	0	35.76
1 primi	2	H						
2	1	06/11/2020	176	913	1	11.1	0.06	35.46
1 primi	2	H						
2	1	06/12/2020	177	914	1	10.7	0	35.17
1 primi	2	H						
2	1	06/13/2020	178	915	1	10.6	0	34.51
1 primi	2	H						
2	1	06/14/2020	179	916	1	10.7	0.09	36.53
1 primi	2	H						
2	1	06/15/2020	180	917	1	10.7	0	35.78

1 primi	2	H						
2	1	06/16/2020	181	918	1	10.4	0.04	32.91
1 primi	2	H						
3	1	12/22/2020	10	686	1	9.9	0.21	23.13
1 primi	1	H						
3	1	12/23/2020	11	687	1	9.9	0.06	22.02
1 primi	1	H						
3	1	12/24/2020	12	688	1	10	0.2	23.22
1 primi	1	H						
3	1	12/25/2020	13	689	1	9.8	0.19	23.51
1 primi	1	H						
3	1	12/26/2020	14	690	1	9.6	0.21	25.32
1 primi	1	H						
3	1	12/27/2020	15	691	1	9.1	0.12	21.38
1 primi	1	H						
3	1	12/28/2020	16	692	1	9.1	0.22	24.13
1 primi	1	H						
3	1	12/29/2020	17	693	1	9.2	0.04	27.48
1 primi	1	H						
3	1	12/30/2020	18	694	1	8.9	0.08	25.61
1 primi	1	H						
3	1	12/31/2020	19	695	1	8.8	0.15	27.25
1 primi	1	H						
3	1	01/01/2021	20	696	1	8.6	0.17	27.8
1 primi	1	H						
3	1	01/02/2021	21	697	1	8.9	0.16	26.92
1 primi	1	H						
3	1	01/03/2021	22	698	1	8.6	0.2	29.95
1 primi	2	H						
3	1	01/04/2021	23	699	1	8.7	0.09	30.37
1 primi	2	H						
3	1	01/05/2021	24	700	1	8.7	0.09	30.67
1 primi	2	H						
3	1	01/06/2021	25	701	1	8.7	0.03	31.25
1 primi	2	H						
3	1	01/07/2021	26	702	1	8.6	0.07	31.21
1 primi	2	H						
3	1	01/08/2021	27	703	1	8.6	0.15	31.12
1 primi	2	H						
3	1	01/09/2021	28	704	1	8.5	0.08	31.23
1 primi	2	H						
3	1	01/10/2021	29	705	1	8.7	0.21	32.55
1 primi	2	H						
3	1	01/11/2021	30	706	1	8.4	0.28	32.27
1 primi	2	H						
3	1	01/12/2021	31	707	1	8.5	0.06	32.67
1 primi	2	H						
3	1	01/13/2021	32	708	1	8.5	0.22	33.34
1 primi	2	H						
3	1	01/14/2021	33	709	1	8.5	0.25	32.89
1 primi	2	H						
3	1	01/15/2021	34	710	1	8.6	0.28	34.1
1 primi	2	H						
3	1	01/16/2021	35	711	1	8.4	0.03	31.48

1 primi 2		H						
3	1	01/17/2021	36	712	1	8.6	0.1	33.76
1 primi 2		H						
3	1	01/18/2021	37	713	1	8.7	0.14	34.15
1 primi 2		H						
3	1	01/19/2021	38	714	1	8.7	0.14	33.93
1 primi 2		H						
3	1	01/20/2021	39	715	1	8.5	0.14	29.73
1 primi 2		H						
3	1	01/21/2021	40	716	1	8.6	0.19	31.68
1 primi 2		H						
3	1	01/22/2021	41	717	1	8.6	0.05	33.13
1 primi 2		H						
3	1	01/23/2021	42	718	1	8.4	0.14	31.31
1 primi 2		H						
3	1	01/24/2021	43	719	1	8.6	0.1	33.68
1 primi 2		H						
3	1	01/25/2021	44	720	1	8.9	0.2	32.68
1 primi 2		H						
3	1	01/26/2021	45	721	1	8.7	0.12	34.57
1 primi 2		H						
3	1	01/27/2021	46	722	1	8.8	0.06	34.14
1 primi 2		H						
3	1	01/28/2021	47	723	1	8.7	0.19	36.66
1 primi 2		H						
3	1	01/29/2021	48	724	1	9.1	0.22	36.25
1 primi 2		H						
3	1	01/30/2021	49	725	1	8.7	0.16	35.75
1 primi 2		H						
3	1	01/31/2021	50	726	1	8.8	0.06	36.39
1 primi 2		H						
3	1	02/01/2021	51	727	1	8.8	0.02	36.05
1 primi 2		H						
3	1	02/02/2021	52	728	1	8.8	0.13	35.89
1 primi 2		H						
3	1	02/03/2021	53	729	1	8.7	0.06	35.96
1 primi 2		H						
3	1	02/04/2021	54	730	1	9.1	0.01	38.58
1 primi 2		H						
3	1	02/05/2021	55	731	1	8.8	0.04	36.57
1 primi 2		H						
3	1	02/06/2021	56	732	1	8.6	0.2	37.04
1 primi 2		H						
3	1	02/07/2021	57	733	1	8.9	0.25	36.82
1 primi 2		H						
3	1	02/08/2021	58	734	1	9.1	0.03	37.2
1 primi 2		H						
3	1	02/09/2021	59	735	1	8.6	0.18	34.46
1 primi 2		H						
3	1	02/10/2021	60	736	1	8.7	0.09	35.16
1 primi 2		H						
3	1	02/11/2021	61	737	1	8.6	0.27	37.7
1 primi 2		H						
3	1	02/12/2021	62	738	1	8.7	0.02	35.4

1 primi 2		H						
3	1	02/13/2021	63	739	1	8.6	0.21	36.34
1 primi 2		H						
3	1	02/14/2021	64	740	1	8.5	0.08	35.02
1 primi 2		H						
3	1	02/15/2021	65	741	1	8.7	0.01	36.04
1 primi 2		H						
3	1	02/16/2021	66	742	1	8.7	0.15	36.95
1 primi 2		H						
3	1	02/17/2021	67	743	1	8.7	0.18	37.5
1 primi 2		H						
3	1	02/18/2021	68	744	1	9.2	0.02	36.89
1 primi 2		H						
3	1	02/19/2021	69	745	1	8.7	0.18	36.8
1 primi 2		H						
3	1	02/20/2021	70	746	1	8.6	0.16	37.05
1 primi 2		H						
3	1	02/21/2021	71	747	1	8.8	0.05	36.62
1 primi 2		H						
3	1	02/22/2021	72	748	1	8.7	0.22	36.29
1 primi 2		H						
3	1	02/23/2021	73	749	1	8.9	0	36.97
1 primi 2		H						
3	1	02/24/2021	74	750	1	8.9	0.24	37.79
1 primi 2		H						
3	1	02/25/2021	75	751	1	8.5	0.08	36.99
1 primi 2		H						
3	1	02/26/2021	76	752	1	8.5	0.15	35.63
1 primi 2		H						
3	1	02/27/2021	77	753	1	8.6	0	36.38
1 primi 2		H						
3	1	02/28/2021	78	754	1	8.9	0.13	37.18
1 primi 2		H						
3	1	03/01/2021	79	755	1	8.9	0.15	37.14
1 primi 2		H						
3	1	03/02/2021	80	756	1	8.8	0.21	38.39
1 primi 2		H						
3	1	03/03/2021	81	757	1	8.8	0.05	36.04
1 primi 2		H						
3	1	03/04/2021	82	758	1	8.7	0.06	37.21
1 primi 2		H						
3	1	03/05/2021	83	759	1	9.1	0.46	35.36
1 primi 2		H						
3	1	03/06/2021	84	760	1	9.1	0.15	36.63
1 primi 2		H						
3	1	03/07/2021	85	761	1	8.8	0.05	35.7
1 primi 2		H						
3	1	03/08/2021	86	762	1	8.7	0.23	38.58
1 primi 2		H						
3	1	03/09/2021	87	763	1	8.4	0.24	35.2
1 primi 2		H						
3	1	03/10/2021	88	764	1	8.9	0.15	37.97
1 primi 2		H						
3	1	03/11/2021	89	765	1	8.9	0.15	33.19

1 primi 2		H						
3	1	03/12/2021	90	766	1	8.8	0.25	36.37
1 primi 2		H						
3	1	03/13/2021	91	767	1	8.7	0.26	34.51
1 primi 2		H						
3	1	03/14/2021	92	768	1	8.9	0.26	37.61
1 primi 2		H						
3	1	03/15/2021	93	769	1	8.7	0.27	34.45
1 primi 2		H						
3	1	03/16/2021	94	770	1	8.8	0.26	37.74
1 primi 2		H						
3	1	03/17/2021	95	771	1	8.7	0.27	36.4
1 primi 2		H						
3	1	03/18/2021	96	772	1	8.7	0.31	36.43
1 primi 2		H						
3	1	03/19/2021	97	773	1	8.9	0.27	37.19
1 primi 2		H						
3	1	03/20/2021	98	774	1	8.4	0.28	35.94
1 primi 2		H						
3	1	03/21/2021	99	775	1	8.7	0.27	36.16
1 primi 2		H						
3	1	03/22/2021	100	776	1	8.6	0.37	37.19
1 primi 2		H						
3	1	03/23/2021	101	777	1	8.8	0.34	38.23
1 primi 2		H						
3	1	03/24/2021	102	778	1	8.9	0.52	38.72
1 primi 2		H						
3	1	03/25/2021	103	779	1	9.1	0.13	38.78
1 primi 2		H						
3	1	03/26/2021	104	780	1	8.9	0.04	37.8
1 primi 2		H						
3	1	03/27/2021	105	781	1	9.1	0.05	39.12
1 primi 2		H						
3	1	03/28/2021	106	782	1	8.8	0.05	34.39
1 primi 2		H						
3	1	03/29/2021	107	783	1	8.9	0.17	37.79
1 primi 2		H						
3	1	03/30/2021	108	784	1	8.7	0.22	38.06
1 primi 2		H						
3	1	03/31/2021	109	785	1	9.3	0.19	38.92
1 primi 2		H						
3	1	04/01/2021	110	786	1	8.8	0.38	37.16
1 primi 2		H						
3	1	04/02/2021	111	787	1	9.1	0.19	38.55
1 primi 2		H						
3	1	04/03/2021	112	788	1	9.1	0.17	37.75
1 primi 2		H						
3	1	04/04/2021	113	789	1	8.6	0.28	35.31
1 primi 2		H						
3	1	04/05/2021	114	790	1	8.5	0.26	35.66
1 primi 2		H						
3	1	04/06/2021	115	791	1	8.8	0.21	37.11
1 primi 2		H						
3	1	04/07/2021	116	792	1	8.6	0.33	35.21

1 primi	2	H						
3	1	04/08/2021	117	793	1	8.7	0.25	36.32
1 primi	2	H						
3	1	04/09/2021	118	794	1	8.8	0.34	38.12
1 primi	2	H						
3	1	04/10/2021	119	795	1	9.1	0	41.91
1 primi	2	H						
3	1	04/11/2021	120	796	1	9.1	0.32	39.16
1 primi	2	H						
3	1	04/12/2021	121	797	1	9.1	0.36	40.25
1 primi	2	H						
3	1	04/13/2021	122	798	1	8.7	0.38	39.32
1 primi	2	H						
3	1	04/14/2021	123	799	1	8.5	0.31	38.6
1 primi	2	H						
3	1	04/15/2021	124	800	1	8.6	0.31	37.25
1 primi	2	H						
3	1	04/16/2021	125	801	1	8.5	0.26	38.05
1 primi	2	H						
3	1	04/17/2021	126	802	1	8.7	0.31	37.21
1 primi	2	H						
3	1	04/18/2021	127	803	1	8.9	0.28	37.93
1 primi	2	H						
3	1	04/19/2021	128	804	1	8.6	0.38	38.85
1 primi	2	H						
3	1	04/20/2021	129	805	1	8.8	0.38	40.02
1 primi	2	H						
3	1	04/21/2021	130	806	1	9.1	0.24	40.31
1 primi	2	H						
3	1	04/22/2021	131	807	1	8.7	0.31	39.05
1 primi	2	H						
3	1	04/23/2021	132	808	1	8.9	0.25	39.91
1 primi	2	H						
3	1	04/24/2021	133	809	1	8.8	0.39	37.82
1 primi	2	H						
3	1	04/25/2021	134	810	1	8.6	0.28	38.28
1 primi	2	H						
3	1	04/26/2021	135	811	1	8.8	0	39.51
1 primi	2	H						
3	1	04/27/2021	136	812	1	8.8	0	37.6
1 primi	2	H						
3	1	04/28/2021	137	813	1	8.8	0.31	38.76
1 primi	2	H						
3	1	04/29/2021	138	814	1	9.1	0.28	40.21
1 primi	2	H						
3	1	04/30/2021	139	815	1	9.1	0.36	40.07
1 primi	2	H						
3	1	05/01/2021	140	816	1	8.8	0.31	37.35
1 primi	2	H						
3	1	05/02/2021	141	817	1	8.9	0.27	37.73
1 primi	2	H						
3	1	05/03/2021	142	818	1	8.6	0.3	35.87
1 primi	2	H						
3	1	05/04/2021	143	819	1	8.9	0.28	37.01

1 primi	2	H						
3	1	05/05/2021	144	820	1	8.8	0.15	37.47
1 primi	2	H						
3	1	05/06/2021	145	821	1	8.9	0.16	37.53
1 primi	2	H						
3	1	05/07/2021	146	822	1	8.9	0.23	38.72
1 primi	2	H						
3	1	05/08/2021	147	823	1	9.2	0	39.73
1 primi	2	H						
3	1	05/09/2021	148	824	1	8.9	0.38	39.66
1 primi	2	H						
3	1	05/10/2021	149	825	1	9.3	0.25	41.89
1 primi	2	H						
3	1	05/11/2021	150	826	1	8.9	0.28	39.45
1 primi	2	H						
3	1	05/12/2021	151	827	1	8.9	0.28	41.21
1 primi	2	H						
3	1	05/13/2021	152	828	1	8.9	0.34	37.95
1 primi	2	H						
3	1	05/14/2021	153	829	1	9.2	0.22	40.48
1 primi	2	H						
3	1	05/15/2021	154	830	1	9.2	0.31	39.34
1 primi	2	H						
3	1	05/16/2021	155	831	1	8.9	0.3	36.91
1 primi	2	H						
3	1	05/17/2021	156	832	1	8.9	0.31	37.61
1 primi	2	H						
3	1	05/18/2021	157	833	1	8.9	0.06	38.73
1 primi	2	H						
3	1	05/19/2021	158	834	1	8.8	0.28	36.7
1 primi	2	H						
3	1	05/20/2021	159	835	1	9.1	0.33	37.79
1 primi	2	H						
3	1	05/21/2021	160	836	1	9.8	0.27	41.22
1 primi	2	H						
3	1	05/22/2021	161	837	1	8.9	0.26	37.46
1 primi	2	H						
3	1	05/23/2021	162	838	1	9.2	0.1	39.5
1 primi	2	H						
3	1	05/24/2021	163	839	1	9.1	0.27	40.27
1 primi	2	H						
3	1	05/25/2021	164	840	1	9.2	0.13	39.59
1 primi	2	H						
3	1	05/26/2021	165	841	1	9.1	0	20.08
1 primi	2	H						
4	1	05/11/2021	8	733	1	9.1	0.33	25.18
1 primi	1	C						
4	1	05/12/2021	9	734	1	10.2	0.08	26.89
1 primi	1	C						
4	1	05/13/2021	10	735	1	10	0.08	25.96
1 primi	1	C						
4	1	05/14/2021	11	736	1	9.9	0.06	26.25
1 primi	1	C						
4	1	05/15/2021	12	737	1	9.6	0.15	25.09

1 primi	1	C						
4	1	05/16/2021	13	738	1	9.6	0.13	26.12
1 primi	1	C						
4	1	05/17/2021	14	739	1	9.5	0	27.34
1 primi	1	C						
4	1	05/18/2021	15	740	1	10	0.13	26.4
1 primi	1	C						
4	1	05/19/2021	16	741	1	9.9	0.06	28.35
1 primi	1	C						
4	1	05/20/2021	17	742	1	9.6	0.07	29.15
1 primi	1	C						
4	1	05/21/2021	18	743	1	10	0.07	28.84
1 primi	1	C						
4	1	05/22/2021	19	744	1	9.8	0.07	28.19
1 primi	1	C						
4	1	05/23/2021	20	745	1	9.8	0.07	28.03
1 primi	1	C						
4	1	05/24/2021	21	746	1	9.9	0.07	27.59
1 primi	1	C						
4	1	05/25/2021	22	747	1	9.8	0	31.08
1 primi	2	C						
4	1	05/26/2021	23	748	1	9.8	0.06	14.08
1 primi	2	C						
5	1	03/01/2021	13	740	1	8.8	0.3	21.01
1 primi	1	C						
5	1	03/02/2021	14	741	1	9.3	0.03	22.88
1 primi	1	C						
5	1	03/03/2021	15	742	1	9.3	0.07	23.34
1 primi	1	C						
5	1	03/04/2021	16	743	1	9.3	0.08	23.29
1 primi	1	C						
5	1	03/05/2021	17	744	1	9.6	0.07	22.9
1 primi	1	C						
5	1	03/06/2021	18	745	1	9.3	0.16	23.4
1 primi	1	C						
7	1	12/23/2019	4	747	1	10	0.36	18.81
1 primi	1	H						
7	1	12/24/2019	5	748	1	11.1	0.16	22
1 primi	1	H						
7	1	12/25/2019	6	749	1	10.3	0.39	22.41
1 primi	1	H						
7	1	12/26/2019	7	750	1	10	0.01	23.48
1 primi	1	H						
7	1	12/27/2019	8	751	1	10	0	24.28
1 primi	1	H						
7	1	12/28/2019	9	752	1	10	0.02	24.83
1 primi	1	H						
7	1	12/29/2019	10	753	1	10	0.06	24.49
1 primi	1	H						
7	1	12/30/2019	11	754	1	10.7	0.05	25.77
1 primi	1	H						
7	1	12/31/2019	12	755	1	10.7	0.05	26.94
1 primi	1	H						
7	1	01/01/2020	13	756	1	10.2	0.12	27.55

1 primi	1	H						
7	1	01/02/2020	14	757	1	10	0.02	24.66
1 primi	1	H						
7	1	01/03/2020	15	758	1	10.3	0.12	29.1
1 primi	1	H						
7	1	01/04/2020	16	759	1	10.3	0.11	27.64
1 primi	1	H						
7	1	01/05/2020	17	760	1	9.8	0.09	29.01
1 primi	1	H						
7	1	01/06/2020	18	761	1	10	0.04	28.2
1 primi	1	H						
7	1	01/07/2020	19	762	1	10	0.01	29.43
1 primi	1	H						
7	1	01/08/2020	20	763	1	10	0	28.4
1 primi	1	H						
7	1	01/09/2020	21	764	1	10.2	0.1	28.31
1 primi	1	H						
7	1	01/10/2020	22	765	1	9.9	0.19	24.01
1 primi	2	H						
7	1	01/11/2020	23	766	1	10.3	0.09	26.92
1 primi	2	H						
7	1	01/12/2020	24	767	1	10.2	0.08	30.07
1 primi	2	H						
7	1	01/13/2020	25	768	1	10	0.04	28.87
1 primi	2	H						
7	1	01/14/2020	26	769	1	10.2	0	30.63
1 primi	2	H						
7	1	01/15/2020	27	770	1	10.4	0.03	33.17
1 primi	2	H						
7	1	01/16/2020	28	771	1	10.3	0.1	30.36
1 primi	2	H						
7	1	01/17/2020	29	772	1	10	0.13	32.25
1 primi	2	H						
7	1	01/18/2020	30	773	1	10.2	0.03	30.8
1 primi	2	H						
7	1	01/19/2020	31	774	1	10.2	0.01	30.77
1 primi	2	H						
7	1	01/20/2020	32	775	1	10.3	0.03	32.81
1 primi	2	H						
7	1	01/21/2020	33	776	1	10.3	0	26.06
1 primi	2	H						
7	1	01/22/2020	34	777	1	10.3	0.1	33.81
1 primi	2	H						
7	1	01/23/2020	35	778	1	10.4	0.09	28.46
1 primi	2	H						
7	1	01/24/2020	36	779	1	10.6	0.33	29.1
1 primi	2	H						
7	1	01/25/2020	37	780	1	10.3	0	30.09
1 primi	2	H						
7	1	01/26/2020	38	781	1	10.4	0.02	29.3
1 primi	2	H						
7	1	01/27/2020	39	782	1	10.4	0.12	29.78
1 primi	2	H						
7	1	01/28/2020	40	783	1	10	0.27	17.64

1 primi 2		H						
7	1	01/29/2020	41	784	1	10.2	0.05	33.55
1 primi 2		H						
7	1	01/30/2020	42	785	1	10.9	0.04	31.15
1 primi 2		H						
7	1	01/31/2020	43	786	1	10.3	0.08	26.14
1 primi 2		H						
7	1	02/01/2020	44	787	1	10.4	0.08	32.46
1 primi 2		H						
7	1	02/02/2020	45	788	1	10.9	0.03	30.56
1 primi 2		H						
7	1	02/03/2020	46	789	1	10.9	0.01	31.62
1 primi 2		H						
7	1	02/04/2020	47	790	1	10.7	0.01	17.12
1 primi 2		H						
7	1	02/05/2020	48	791	1	10.6	0.1	31.17
1 primi 2		H						
7	1	02/06/2020	49	792	1	10.4	0.05	30.15
1 primi 2		H						
7	1	02/07/2020	50	793	1	10.2	0.04	25.59
1 primi 2		H						
7	1	02/08/2020	51	794	1	10.7	0.08	28.84
1 primi 2		H						
7	1	02/09/2020	52	795	1	10.2	0.09	26.18
1 primi 2		H						
7	1	02/10/2020	53	796	1	10	0.11	29.95
1 primi 2		H						
7	1	02/11/2020	54	797	1	10	0	23.8
1 primi 2		H						
7	1	02/12/2020	55	798	1	10.4	0.01	24.04
1 primi 2		H						
7	1	02/13/2020	56	799	1	10.7	0.04	23.92
1 primi 2		H						
7	1	02/14/2020	57	800	1	11.1	0.03	36.68
1 primi 2		H						
7	1	02/15/2020	58	801	1	10.7	0.22	31.04
1 primi 2		H						
7	1	02/16/2020	59	802	1	10.6	0.01	32.98
1 primi 2		H						
7	1	02/17/2020	60	803	1	10.3	0.39	32.9
1 primi 2		H						
7	1	02/18/2020	61	804	1	10	0.01	32.97
1 primi 2		H						
8	1	06/22/2013	7	737	1	9.9	0	27.72
1 primi 1		H						
8	1	06/23/2013	8	738	1	10	0	28.68
1 primi 1		H						
8	1	06/24/2013	9	739	1	10	0	27.04
1 primi 1		H						
8	1	06/25/2013	10	740	1	9.9	0	29.93
1 primi 1		H						
8	1	06/26/2013	11	741	1	10	0	32.01
1 primi 1		H						
8	1	06/27/2013	12	742	1	10	0	35.78

1 primi	1	H						
8	1	06/28/2013	13	743	1	9.8	0	33.82
1 primi	1	H						
8	1	06/29/2013	14	744	1	9.9	0	36.01
1 primi	1	H						
8	1	06/30/2013	15	745	1	9.5	0	34.83
1 primi	1	H						
8	1	07/01/2013	16	746	1	9.9	0	36.9
1 primi	1	H						
8	1	07/02/2013	17	747	1	9.5	0	36.9
1 primi	1	H						
8	1	07/03/2013	18	748	1	10	0	37.16
1 primi	1	H						
8	1	07/04/2013	19	749	1	9.9	0	32.9
1 primi	1	H						
8	1	07/05/2013	20	750	1	9.6	0	35.49
1 primi	1	H						
8	1	07/06/2013	21	751	1	9.9	0	35.67
1 primi	1	H						
8	1	07/07/2013	22	752	1	9.8	0	37.31
1 primi	2	H						
8	1	07/08/2013	23	753	1	9.8	0	37.05
1 primi	2	H						
8	1	07/09/2013	24	754	1	9.9	0	39.01
1 primi	2	H						
8	1	07/10/2013	25	755	1	9.9	0	38.56
1 primi	2	H						
8	1	07/11/2013	26	756	1	9.6	0	39.25
1 primi	2	H						
8	1	07/12/2013	27	757	1	9.6	0	40.17
1 primi	2	H						
8	1	07/13/2013	28	758	1	9.8	0	38.48
1 primi	2	H						
8	1	07/14/2013	29	759	1	9.8	0	39.53
1 primi	2	H						
8	1	07/15/2013	30	760	1	9.3	0	40.21
1 primi	2	H						
8	1	07/16/2013	31	761	1	9.5	0	42.32
1 primi	2	H						
8	1	07/17/2013	32	762	1	9.2	0	37.14
1 primi	2	H						
8	1	07/18/2013	33	763	1	8.9	0	38.44
1 primi	2	H						
8	1	07/19/2013	34	764	1	9.5	0	42.11
1 primi	2	H						
8	1	07/20/2013	35	765	1	9.6	0	40.82
1 primi	2	H						
8	1	07/21/2013	36	766	1	9.6	0	39.44
1 primi	2	H						
8	1	07/22/2013	37	767	1	9.8	0	41.7
1 primi	2	H						
8	1	07/23/2013	38	768	1	9.8	0	43.87
1 primi	2	H						
8	1	07/24/2013	39	769	1	9.5	0	42.35

1 primi	2	H						
8	1	07/25/2013	40	770	1	9.9	0	43.67
1 primi	2	H						
8	1	07/26/2013	41	771	1	9.6	0	42.79
1 primi	2	H						
8	1	07/27/2013	42	772	1	9.6	0	44.86
1 primi	2	H						
8	1	07/28/2013	43	773	1	9.5	0	42.03
1 primi	2	H						
8	1	07/29/2013	44	774	1	9.6	0	44.69
1 primi	2	H						
8	1	07/30/2013	45	775	1	9.8	0	39.9
1 primi	2	H						
8	1	07/31/2013	46	776	1	9.9	0	41.77
1 primi	2	H						
8	1	08/01/2013	47	777	1	9.8	0	44.16
1 primi	2	H						
8	1	08/02/2013	48	778	1	9.5	0	44.43
1 primi	2	H						
8	1	08/03/2013	49	779	1	9.5	0	41.43
1 primi	2	H						
8	1	08/04/2013	50	780	1	9.3	0	46.05
1 primi	2	H						
8	1	08/05/2013	51	781	1	9.6	0	45.32
1 primi	2	H						
8	1	08/06/2013	52	782	1	9.3	0	42.68
1 primi	2	H						
8	1	08/07/2013	53	783	1	9.5	0	43.65
1 primi	2	H						
8	1	08/08/2013	54	784	1	9.9	0	43.2
1 primi	2	H						
8	1	08/09/2013	55	785	1	9.5	0	42.7
1 primi	2	H						
8	1	08/10/2013	56	786	1	9.9	0	44.51
1 primi	2	H						
8	1	08/11/2013	57	787	1	9.3	0	41.71
1 primi	2	H						
8	1	08/12/2013	58	788	1	8.9	0	38.05
1 primi	2	H						
8	1	08/13/2013	59	789	1	9.3	0	38.67
1 primi	2	H						
8	1	08/14/2013	60	790	1	9.3	0	39.38
1 primi	2	H						
8	1	08/15/2013	61	791	1	9.8	0	41.23
1 primi	2	H						
8	1	08/16/2013	62	792	1	10.2	0	44.91
1 primi	2	H						
8	1	08/17/2013	63	793	1	9.6	0	40.44
1 primi	2	H						
8	1	08/18/2013	64	794	1	9.8	0	41.78
1 primi	2	H						
8	1	08/19/2013	65	795	1	9.6	0	44.08
1 primi	2	H						
8	1	08/20/2013	66	796	1	9.8	0	42

1 primi	2	H						
8	1	08/21/2013	67	797	1	9.9	0	44.75
1 primi	2	H						
8	1	08/22/2013	68	798	1	9.8	0	45.86
1 primi	2	H						
8	1	08/23/2013	69	799	1	9.6	0	44.14
1 primi	2	H						
8	1	08/24/2013	70	800	1	9.8	0	46.36
1 primi	2	H						
8	1	08/25/2013	71	801	1	9.6	0	44.3
1 primi	2	H						
8	1	08/26/2013	72	802	1	9.3	0	44.66
1 primi	2	H						
8	1	08/27/2013	73	803	1	10	0	39.3
1 primi	2	H						
8	1	08/28/2013	74	804	1	10.2	0	46.13
1 primi	2	H						
8	1	08/29/2013	75	805	1	10.4	0	45.76
1 primi	2	H						
8	1	08/30/2013	76	806	1	10.3	0	44.95
1 primi	2	H						
8	1	08/31/2013	77	807	1	9.8	0	44.9
1 primi	2	H						
8	1	09/01/2013	78	808	1	9.9	0	44.34
1 primi	2	H						
8	1	09/02/2013	79	809	1	10	0	44.73
1 primi	2	H						
8	1	09/03/2013	80	810	1	10	0	43.57
1 primi	2	H						
8	1	09/04/2013	81	811	1	10	0	46.84
1 primi	2	H						
8	1	09/05/2013	82	812	1	9.3	0	45.7
1 primi	2	H						
8	1	09/06/2013	83	813	1	10.2	0	45.13
1 primi	2	H						
8	1	09/07/2013	84	814	1	10	0	44.47
1 primi	2	H						
8	1	09/08/2013	85	815	1	10	0	44.59
1 primi	2	H						
8	1	09/09/2013	86	816	1	9.6	0	44.76
1 primi	2	H						
8	1	09/10/2013	87	817	1	10	0	44.03
1 primi	2	H						
8	1	09/11/2013	88	818	1	9.8	0	43.53
1 primi	2	H						
8	1	09/12/2013	89	819	1	9.3	0	45.68
1 primi	2	H						
8	1	09/13/2013	90	820	1	9.2	0	42.98
1 primi	2	H						
8	1	09/14/2013	91	821	1	9.6	0	44.42
1 primi	2	H						
8	1	09/15/2013	92	822	1	9.8	0	43.9
1 primi	2	H						
8	1	09/16/2013	93	823	1	9.8	0	42.08

1 primi 2		H						
8	1	09/17/2013	94	824	1	9.8	0	43.54
1 primi 2		H						
8	1	09/18/2013	95	825	1	9.8	0	42.26
1 primi 2		H						
8	1	09/19/2013	96	826	1	9.8	0	43.61
1 primi 2		H						
8	1	09/20/2013	97	827	1	10	0	38.96
1 primi 2		H						
8	1	09/21/2013	98	828	1	10	0	37.69
1 primi 2		H						
8	1	09/22/2013	99	829	1	10	0	36.24
1 primi 2		H						
8	1	09/23/2013	100	830	1	9.5	0	38.72
1 primi 2		H						
8	1	09/24/2013	101	831	1	10	0	36.73
1 primi 2		H						
8	1	09/25/2013	102	832	1	9.8	0	37.74
1 primi 2		H						
8	1	09/26/2013	103	833	1	10.3	0	36.88
1 primi 2		H						
8	1	09/27/2013	104	834	1	10.4	0	36.94
1 primi 2		H						
8	1	09/28/2013	105	835	1	9.3	0	37.36
1 primi 2		H						
8	1	09/29/2013	106	836	1	9.6	0	34.59
1 primi 2		H						
8	1	09/30/2013	107	837	1	10.4	0	37.35
1 primi 2		H						
8	1	10/01/2013	108	838	1	9.9	0	36.65
1 primi 2		H						
8	1	10/02/2013	109	839	1	10.2	0	30.46
1 primi 2		H						
8	1	10/03/2013	110	840	1	10	0	34.16
1 primi 2		H						
8	1	10/04/2013	111	841	1	10.7	0	35
1 primi 2		H						
8	1	10/05/2013	112	842	1	10.6	0	33.58
1 primi 2		H						
8	1	10/06/2013	113	843	1	10.7	0	32.93
1 primi 2		H						
8	1	10/07/2013	114	844	1	10.4	0	33.63
1 primi 2		H						
8	1	10/08/2013	115	845	1	10.2	0	33.2
1 primi 2		H						
8	1	10/09/2013	116	846	1	9.9	0	35.17
1 primi 2		H						
8	1	10/10/2013	117	847	1	10.6	0	36.55
1 primi 2		H						
8	1	10/11/2013	118	848	1	10.7	0	35.75
1 primi 2		H						
8	1	10/12/2013	119	849	1	9.9	0	34.45
1 primi 2		H						
8	1	10/13/2013	120	850	1	10.3	0	36.92

1 primi 2 H
8 1 10/14/2013 121 851 1 10.4 0 36.01
1 primi 2 H
8 1 10/15/2013 122 852 1 10.4 0 37.21
1 primi 2 H
8 1 10/16/2013 123 853 1 11.7 0 31.97
1 primi 2 H
8 1 10/17/2013 124 854 1 10.6 0 32.56
1 primi 2 H
8 1 10/18/2013 125 855 1 11.1 0 31.66
1 primi 2 H
8 1 10/19/2013 126 856 1 10.9 0 32.55
1 primi 2 H
8 1 10/20/2013 127 857 1 10.4 0 33.5
1 primi 2 H
8 1 10/21/2013 128 858 1 10.4 0 34.01
1 primi 2 H
8 1 10/22/2013 129 859 1 10.7 0 34.6
1 primi 2 H
8 1 10/23/2013 130 860 1 10.9 0 36.28
1 primi 2 H
8 1 10/24/2013 131 861 1 11.1 0 34.79
1 primi 2 H
8 1 10/25/2013 132 862 1 10.7 0 36.17
1 primi 2 H
8 1 10/26/2013 133 863 1 10.6 0 33.86
1 primi 2 H
8 1 10/27/2013 134 864 1 10.6 0 37.03
1 primi 2 H
8 1 10/28/2013 135 865 1 10.9 0 37.07
1 primi 2 H
8 1 10/29/2013 136 866 1 10.6 0 38.41
1 primi 2 H
8 1 10/30/2013 137 867 1 10.4 0 36.22
1 primi 2 H
8 1 10/31/2013 138 868 1 10.4 0 38.72
1 primi 2 H
8 1 11/01/2013 139 869 1 10.4 0 37.35
1 primi 2 H
8 1 11/02/2013 140 870 1 10.4 0 38.89
1 primi 2 H
8 1 11/03/2013 141 871 1 10.7 0 38.04
1 primi 2 H
8 1 11/04/2013 142 872 1 10.3 0 38.83
1 primi 2 H
8 1 11/05/2013 143 873 1 10.7 0 37.92
1 primi 2 H
8 1 11/06/2013 144 874 1 10.4 0 37.98
1 primi 2 H
8 1 11/07/2013 145 875 1 10.9 0 36.48
1 primi 2 H
8 1 11/08/2013 146 876 1 10.3 0 35.01
1 primi 2 H
8 1 11/09/2013 147 877 1 10.7 0 34.39

1 primi	2	H						
8	1	11/10/2013	148	878	1	10.7	0	35.14
1 primi	2	H						
8	1	11/11/2013	149	879	1	10.9	0	37.01
1 primi	2	H						
8	1	11/12/2013	150	880	1	10.4	0	35.8
1 primi	2	H						
8	1	11/13/2013	151	881	1	10.9	0	35.09
1 primi	2	H						
8	1	11/14/2013	152	882	1	10.7	0	35.56
1 primi	2	H						
8	1	11/15/2013	153	883	1	10.9	0	33.45
1 primi	2	H						
8	1	11/16/2013	154	884	1	10.7	0	33.18
1 primi	2	H						
8	1	11/17/2013	155	885	1	10.3	0	34.1
1 primi	2	H						
8	1	11/18/2013	156	886	1	10.6	0	31.3
1 primi	2	H						
8	1	11/19/2013	157	887	1	10.3	0	33.31
1 primi	2	H						
8	1	11/20/2013	158	888	1	10.4	0	34.72
1 primi	2	H						
8	1	11/21/2013	159	889	1	10.7	0	35.83
1 primi	2	H						
8	1	11/22/2013	160	890	1	10.7	0	33.25
1 primi	2	H						
8	1	11/23/2013	161	891	1	10.6	0	37.45
1 primi	2	H						
8	1	11/24/2013	162	892	1	10.4	0	36.55
1 primi	2	H						
8	1	11/25/2013	163	893	1	10.7	0	35.17
1 primi	2	H						
8	1	11/26/2013	164	894	1	10.4	0	38.73
1 primi	2	H						
8	1	11/27/2013	165	895	1	10.6	0	39.15
1 primi	2	H						
8	1	11/28/2013	166	896	1	10.6	0	38.38
1 primi	2	H						
8	1	11/29/2013	167	897	1	10.4	0	36.51
1 primi	2	H						
8	1	11/30/2013	168	898	1	10.7	0	38.81
1 primi	2	H						
8	1	12/01/2013	169	899	1	10.4	0	38.36
1 primi	2	H						
8	1	12/02/2013	170	900	1	10.7	0	37.17
1 primi	2	H						
8	1	12/03/2013	171	901	1	10.6	0	38.51
1 primi	2	H						
8	1	12/04/2013	172	902	1	10.4	0	37.38
1 primi	2	H						
8	1	12/05/2013	173	903	1	10.6	0	38.3
1 primi	2	H						
8	1	12/06/2013	174	904	1	10.7	0	35.89

1 primi 2		H						
8	1	12/07/2013	175	905	1	10.6	0	35.97
1 primi 2		H						
8	1	12/08/2013	176	906	1	10.7	0	36.04
1 primi 2		H						
8	1	12/09/2013	177	907	1	10.4	0	38.65
1 primi 2		H						
8	1	12/10/2013	178	908	1	10.7	0	38.96
1 primi 2		H						
8	1	12/11/2013	179	909	1	10.7	0	37.65
1 primi 2		H						
8	1	12/12/2013	180	910	1	10.2	0	38.24
1 primi 2		H						
8	1	12/13/2013	181	911	1	10.3	0	39.16
1 primi 2		H						
8	1	12/14/2013	182	912	1	10.6	0	39.65
1 primi 2		H						
8	1	12/15/2013	183	913	1	10.6	0	37.44
1 primi 2		H						
8	1	12/16/2013	184	914	1	10.7	0	37.52
1 primi 2		H						
8	1	12/17/2013	185	915	1	10.6	0.03	39.27
1 primi 2		H						
8	1	12/18/2013	186	916	1	10.2	0.03	37.93
1 primi 2		H						
8	1	12/19/2013	187	917	1	10.4	0.02	37.21
1 primi 2		H						
8	1	12/20/2013	188	918	1	10.4	0.04	38.51
1 primi 2		H						
8	1	12/21/2013	189	919	1	10.3	0.01	38.05
1 primi 2		H						
8	1	12/22/2013	190	920	1	10.7	0	37.76
1 primi 2		H						
8	1	12/23/2013	191	921	1	10.4	0.04	37.96
1 primi 2		H						
8	1	12/24/2013	192	922	1	10.3	0.03	35.87
1 primi 2		H						
8	1	12/25/2013	193	923	1	10.6	0.02	35.15
1 primi 2		H						
8	1	12/26/2013	194	924	1	10.2	0	35
1 primi 2		H						
8	1	12/27/2013	195	925	1	10.4	0.04	36.35
1 primi 2		H						
8	1	12/28/2013	196	926	1	10.3	0.02	35.14
1 primi 2		H						
8	1	12/29/2013	197	927	1	10.4	0.02	38.89
1 primi 2		H						
8	1	12/30/2013	198	928	1	9.9	0.17	42.33
1 primi 2		H						
8	1	12/31/2013	199	929	1	10.4	0.01	36.98
1 primi 2		H						
8	1	01/01/2014	200	930	1	10.4	0.03	35.23
1 primi 2		H						
8	1	01/02/2014	201	931	1	10.2	0.02	34.33

1 primi	2	H						
8	1	01/03/2014	202	932	1	9.9	0	35.54
1 primi	2	H						
8	1	01/04/2014	203	933	1	9.9	0.02	36.92
1 primi	2	H						
8	1	01/05/2014	204	934	1	10.3	0	36.47
1 primi	2	H						
8	1	01/06/2014	205	935	1	10.3	0	36.31
1 primi	2	H						
8	1	01/07/2014	206	936	1	10.2	0	35.52
1 primi	2	H						
8	1	01/08/2014	207	937	1	10.3	0.02	36.19
1 primi	2	H						
8	1	01/09/2014	208	938	1	10.3	0	35.18
1 primi	2	H						
8	1	01/10/2014	209	939	1	10.2	0	27.7
1 primi	2	H						
8	1	01/11/2014	210	940	1	9.9	0.02	35.44
1 primi	2	H						
8	1	01/12/2014	211	941	1	10.4	0	36.31
1 primi	2	H						
8	1	01/13/2014	212	942	1	10.3	0.01	34.11
1 primi	2	H						
8	1	01/14/2014	213	943	1	10.6	0.01	36.88
1 primi	2	H						
8	1	01/15/2014	214	944	1	10.2	0	35.25
1 primi	2	H						
8	1	01/16/2014	215	945	1	9.9	0.03	34.88
1 primi	2	H						
8	1	01/17/2014	216	946	1	10.2	0.03	36.89
1 primi	2	H						
8	1	01/18/2014	217	947	1	10	0	36.01
1 primi	2	H						
8	1	01/19/2014	218	948	1	10.2	0.02	34.89
1 primi	2	H						
8	1	01/20/2014	219	949	1	10.6	0.02	34.71
1 primi	2	H						
8	1	01/21/2014	220	950	1	10.2	0.02	32.17
1 primi	2	H						
8	1	01/22/2014	221	951	1	10.3	0	33.26
1 primi	2	H						
8	1	01/23/2014	222	952	1	10.2	0.02	32.55
1 primi	2	H						
8	1	01/24/2014	223	953	1	9.9	0.02	32.5
1 primi	2	H						
8	1	01/25/2014	224	954	1	10.4	0.02	33.21
1 primi	2	H						
8	1	01/26/2014	225	955	1	10.7	0.03	33.69
1 primi	2	H						
8	1	01/27/2014	226	956	1	10.3	0.02	31.06
1 primi	2	H						
8	1	01/28/2014	227	957	1	9.9	0.01	31.21
1 primi	2	H						
8	1	01/29/2014	228	958	1	10.6	0	31.6

1	primi	2	H						
8		1	01/30/2014	229	959	1	11.4	0	31.78
1	primi	2	H						
8		1	05/18/2014	7	1067	2	9.2	0	30.73
2	multi	1	H						
8		1	05/19/2014	8	1068	2	10	0	31.46
2	multi	1	H						
8		1	05/20/2014	9	1069	2	10.3	0	34.98
2	multi	1	H						
8		1	05/21/2014	10	1070	2	9.9	0	37.7
2	multi	1	H						
8		1	05/22/2014	11	1071	2	9.8	0	42.24
2	multi	1	H						
8		1	05/23/2014	12	1072	2	9.6	0	39.76
2	multi	1	H						
8		1	05/24/2014	13	1073	2	9.8	0	42.33
2	multi	1	H						
8		1	05/25/2014	14	1074	2	9.8	0	42.69
2	multi	1	H						
8		1	05/26/2014	15	1075	2	9.9	0	45.63
2	multi	1	H						
8		1	05/27/2014	16	1076	2	9.8	0	42.92
2	multi	1	H						
8		1	05/28/2014	17	1077	2	9.9	0	45.36
2	multi	1	H						
8		1	05/29/2014	18	1078	2	9.9	0	46.92
2	multi	1	H						
8		1	05/30/2014	19	1079	2	9.9	0	43.51
2	multi	1	H						
8		1	05/31/2014	20	1080	2	9.9	0	46.97
2	multi	1	H						
8		1	06/01/2014	21	1081	2	10.2	0	51.43
2	multi	1	H						
8		1	06/02/2014	22	1082	2	10.2	0	51.46
2	multi	2	H						
8		1	06/03/2014	23	1083	2	9.9	0	45.32
2	multi	2	H						
8		1	06/04/2014	24	1084	2	9.6	0	49.06
2	multi	2	H						
8		1	06/05/2014	25	1085	2	9.8	0	42.59
2	multi	2	H						
8		1	06/06/2014	26	1086	2	9.5	0	44.33
2	multi	2	H						
8		1	06/07/2014	27	1087	2	9.5	0	44.45
2	multi	2	H						
8		1	06/08/2014	28	1088	2	9.6	0	49.01
2	multi	2	H						
8		1	06/09/2014	29	1089	2	9.5	0	47.04
2	multi	2	H						
8		1	06/10/2014	30	1090	2	9.8	0	50.46
2	multi	2	H						
8		1	06/11/2014	31	1091	2	9.9	0	46.72
2	multi	2	H						
8		1	06/12/2014	32	1092	2	9.9	0	48.34

2 multi 2		H						
8	1	06/13/2014	33	1093	2	9.8	0	41.06
2 multi 2		H						
8	1	06/14/2014	34	1094	2	10.2	0	48.33
2 multi 2		H						
8	1	06/15/2014	35	1095	2	10.2	0	48.71
2 multi 2		H						
8	1	06/16/2014	36	1096	2	10.3	0	51.35
2 multi 2		H						
8	1	06/17/2014	37	1097	2	10.2	0	47.63
2 multi 2		H						
8	1	06/18/2014	38	1098	2	10.3	0	53.41
2 multi 2		H						
8	1	06/19/2014	39	1099	2	10.3	0	53.22
2 multi 2		H						
8	1	06/20/2014	40	1100	2	10	0	51.23
2 multi 2		H						
8	1	06/21/2014	41	1101	2	10.2	0	51.79
2 multi 2		H						
8	1	06/22/2014	42	1102	2	10	0	50.25
2 multi 2		H						
8	1	06/23/2014	43	1103	2	10.4	0	54
2 multi 2		H						
8	1	06/24/2014	44	1104	2	9.9	0	49.43
2 multi 2		H						
8	1	06/25/2014	45	1105	2	10.3	0	52.58
2 multi 2		H						
8	1	06/26/2014	46	1106	2	10.3	0	55.8
2 multi 2		H						
8	1	06/27/2014	47	1107	2	10.2	0	53.44
2 multi 2		H						
8	1	06/28/2014	48	1108	2	10.2	0	54.97
2 multi 2		H						
8	1	06/29/2014	49	1109	2	10.2	0	54.45
2 multi 2		H						
8	1	06/30/2014	50	1110	2	10	0	53.85
2 multi 2		H						
8	1	07/01/2014	51	1111	2	9.9	0	51.02
2 multi 2		H						
8	1	07/02/2014	52	1112	2	10.3	0	52.28
2 multi 2		H						
8	1	07/03/2014	53	1113	2	10.2	0	51.53
2 multi 2		H						
8	1	07/04/2014	54	1114	2	9.9	0	54.23
2 multi 2		H						
8	1	07/05/2014	55	1115	2	10	0	54.73
2 multi 2		H						
8	1	07/06/2014	56	1116	2	10.3	0	52.71
2 multi 2		H						
8	1	07/07/2014	57	1117	2	10.3	0	51.12
2 multi 2		H						
8	1	07/08/2014	58	1118	2	10	0	50.81
2 multi 2		H						
8	1	07/09/2014	59	1119	2	9.9	0.07	45.75

2 multi	2	H						
8	1	07/10/2014	60	1120	2	10	0	46.99
2 multi	2	H						
8	1	07/11/2014	61	1121	2	10.2	0	46.72
2 multi	2	H						
8	1	07/12/2014	62	1122	2	10.4	0	54.19
2 multi	2	H						
8	1	07/13/2014	63	1123	2	10.4	0	54.39
2 multi	2	H						
8	1	07/14/2014	64	1124	2	10	0	51.27
2 multi	2	H						
8	1	07/15/2014	65	1125	2	10.2	0	52.1
2 multi	2	H						
8	1	07/16/2014	66	1126	2	10.4	0	49.18
2 multi	2	H						
8	1	07/17/2014	67	1127	2	10	0	53
2 multi	2	H						
8	1	07/18/2014	68	1128	2	10.2	0.04	48.9
2 multi	2	H						
8	1	07/19/2014	69	1129	2	10.7	0	46.61
2 multi	2	H						
8	1	07/20/2014	70	1130	2	10.7	0.01	47.44
2 multi	2	H						
8	1	07/21/2014	71	1131	2	10.4	0.48	44.63
2 multi	2	H						
8	1	07/22/2014	72	1132	2	10.4	0	49.29
2 multi	2	H						
8	1	07/23/2014	73	1133	2	10.7	0.02	46.3
2 multi	2	H						
8	1	07/24/2014	74	1134	2	11.1	0	48.22
2 multi	2	H						
8	1	07/25/2014	75	1135	2	10.9	0	47.91
2 multi	2	H						
8	1	07/26/2014	76	1136	2	10.3	0	47.45
2 multi	2	H						
8	1	07/27/2014	77	1137	2	10.3	0	50.79
2 multi	2	H						
8	1	07/28/2014	78	1138	2	10.6	0	49.13
2 multi	2	H						
8	1	07/29/2014	79	1139	2	10.6	0	44.42
2 multi	2	H						
8	1	07/30/2014	80	1140	2	10.9	0	48.63
2 multi	2	H						
8	1	07/31/2014	81	1141	2	10.7	0	43.79
2 multi	2	H						
8	1	08/01/2014	82	1142	2	10.4	0	43.77
2 multi	2	H						
8	1	08/02/2014	83	1143	2	10.4	0	44.97
2 multi	2	H						
8	1	08/03/2014	84	1144	2	10.6	0	32.48
2 multi	2	H						
8	1	08/04/2014	85	1145	2	10.4	0	29.13
2 multi	2	H						
8	1	08/05/2014	86	1146	2	11.3	0	28.51

2 multi	2	H						
8	1	08/06/2014	87	1147	2	11.9	0	25.67
2 multi	2	H						
8	1	08/07/2014	88	1148	2	12.3	0	30.44
2 multi	2	H						
8	1	08/08/2014	89	1149	2	10.7	0	28.16
2 multi	2	H						
8	1	09/11/2015	4	1548	3	9.8	0.01	36.15
2 multi	1	H						
8	1	09/12/2015	5	1549	3	9.9	0	32.74
2 multi	1	H						
8	1	09/13/2015	6	1550	3	8.8	0.17	34.09
2 multi	1	H						
8	1	09/14/2015	7	1551	3	9.5	0	33.72
2 multi	1	H						
8	1	09/15/2015	8	1552	3	9.2	0	34.46
2 multi	1	H						
8	1	09/16/2015	9	1553	3	9.5	0	37.66
2 multi	1	H						
8	1	09/17/2015	10	1554	3	8.9	0.08	35.36
2 multi	1	H						
8	1	09/18/2015	11	1555	3	9.1	0	38.88
2 multi	1	H						
8	1	09/19/2015	12	1556	3	10.3	0	41.1
2 multi	1	H						
8	1	09/20/2015	13	1557	3	9.3	0	42.5
2 multi	1	H						
8	1	09/21/2015	14	1558	3	9.6	0	44.93
2 multi	1	H						
8	1	09/22/2015	15	1559	3	9.8	0	41.08
2 multi	1	H						
8	1	09/23/2015	16	1560	3	9.8	0	44.28
2 multi	1	H						
8	1	09/24/2015	17	1561	3	9.6	0	48.94
2 multi	1	H						
8	1	09/25/2015	18	1562	3	9.5	0	47.79
2 multi	1	H						
8	1	09/26/2015	19	1563	3	9.6	0	47.05
2 multi	1	H						
8	1	09/27/2015	20	1564	3	9.5	0.02	52.24
2 multi	1	H						
8	1	09/28/2015	21	1565	3	9.5	0	44.83
2 multi	1	H						
8	1	09/29/2015	22	1566	3	9.3	0	46.69
2 multi	2	H						
8	1	09/30/2015	23	1567	3	9.3	0.05	48.94
2 multi	2	H						
8	1	10/01/2015	24	1568	3	9.5	0	49.36
2 multi	2	H						
8	1	10/02/2015	25	1569	3	9.5	0	50.45
2 multi	2	H						
8	1	10/03/2015	26	1570	3	9.3	0	50.27
2 multi	2	H						
8	1	10/04/2015	27	1571	3	10	0	49.76

2 multi	2	H						
8	1	10/05/2015	28	1572	3	9.6	0	52.15
2 multi	2	H						
8	1	10/06/2015	29	1573	3	9.6	0.52	52.5
2 multi	2	H						
8	1	10/07/2015	30	1574	3	9.5	0	52.92
2 multi	2	H						
8	1	10/08/2015	31	1575	3	9.6	0	50.95
2 multi	2	H						
8	1	10/09/2015	32	1576	3	9.5	0	53.08
2 multi	2	H						
8	1	10/10/2015	33	1577	3	9.3	0	52.37
2 multi	2	H						
8	1	10/11/2015	34	1578	3	9.8	0.02	49.2
2 multi	2	H						
8	1	10/12/2015	35	1579	3	9.2	0	52.04
2 multi	2	H						
8	1	10/13/2015	36	1580	3	9.1	0	53.02
2 multi	2	H						
8	1	10/14/2015	37	1581	3	9.2	0.01	51.77
2 multi	2	H						
8	1	10/15/2015	38	1582	3	9.2	0	55.05
2 multi	2	H						
8	1	10/16/2015	39	1583	3	9.5	0	52.89
2 multi	2	H						
8	1	10/17/2015	40	1584	3	9.1	0	53.76
2 multi	2	H						
8	1	10/18/2015	41	1585	3	9.5	0	52.87
2 multi	2	H						
8	1	10/19/2015	42	1586	3	9.3	0	53.07
2 multi	2	H						
8	1	10/20/2015	43	1587	3	9.5	0	54.29
2 multi	2	H						
8	1	10/21/2015	44	1588	3	9.1	0	51.76
2 multi	2	H						
8	1	10/22/2015	45	1589	3	9.2	0	50.23
2 multi	2	H						
8	1	10/23/2015	46	1590	3	9.3	0	47.53
2 multi	2	H						
8	1	10/24/2015	47	1591	3	9.2	0	48.97
2 multi	2	H						
8	1	10/25/2015	48	1592	3	9.5	0	48.81
2 multi	2	H						
8	1	10/26/2015	49	1593	3	9.2	0	52.62
2 multi	2	H						
8	1	10/27/2015	50	1594	3	9.2	0	48.5
2 multi	2	H						
8	1	10/28/2015	51	1595	3	9.5	0	51.52
2 multi	2	H						
8	1	10/29/2015	52	1596	3	9.2	0	51.32
2 multi	2	H						
8	1	10/30/2015	53	1597	3	9.5	0	51.84
2 multi	2	H						
8	1	10/31/2015	54	1598	3	9.6	0	46.94

2 multi	2	H						
8	1	11/01/2015	55	1599	3	9.8	0	56
2 multi	2	H						
8	1	11/02/2015	56	1600	3	9.6	0	52.87
2 multi	2	H						
8	1	11/03/2015	57	1601	3	9.5	0	52.7
2 multi	2	H						
8	1	11/04/2015	58	1602	3	9.5	0	51.51
2 multi	2	H						
8	1	11/05/2015	59	1603	3	9.6	0	54.66
2 multi	2	H						
8	1	11/06/2015	60	1604	3	9.3	0	54.55
2 multi	2	H						
8	1	11/07/2015	61	1605	3	9.1	0	50.86
2 multi	2	H						
8	1	11/08/2015	62	1606	3	9.6	0	49.2
2 multi	2	H						
8	1	11/09/2015	63	1607	3	9.3	0	47.56
2 multi	2	H						
8	1	11/10/2015	64	1608	3	9.5	0	50.06
2 multi	2	H						
8	1	11/11/2015	65	1609	3	9.8	0	48.74
2 multi	2	H						
8	1	11/12/2015	66	1610	3	10.2	0	53.02
2 multi	2	H						
8	1	11/13/2015	67	1611	3	10	0	49.7
2 multi	2	H						
8	1	11/14/2015	68	1612	3	9.8	0	48.3
2 multi	2	H						
8	1	11/15/2015	69	1613	3	9.9	0.47	49.57
2 multi	2	H						
8	1	11/16/2015	70	1614	3	9.8	0	49.69
2 multi	2	H						
8	1	11/17/2015	71	1615	3	10.6	0	42.61
2 multi	2	H						
8	1	11/18/2015	72	1616	3	10.9	0	46.15
2 multi	2	H						
8	1	11/19/2015	73	1617	3	10.3	0	47.62
2 multi	2	H						
8	1	11/20/2015	74	1618	3	10.4	0.02	50.02
2 multi	2	H						
8	1	11/21/2015	75	1619	3	10.6	0	44.57
2 multi	2	H						
8	1	11/22/2015	76	1620	3	10.6	0	46.24
2 multi	2	H						
8	1	11/23/2015	77	1621	3	10.6	0	47.71
2 multi	2	H						
8	1	11/24/2015	78	1622	3	10.6	0	26.69
2 multi	2	H						
8	1	11/25/2015	79	1623	3	10.7	0	50.84
2 multi	2	H						
8	1	11/26/2015	80	1624	3	10.2	0	50.38
2 multi	2	H						
8	1	11/27/2015	81	1625	3	10.4	0.04	47.29

2 multi	2	H						
8	1	11/28/2015	82	1626	3	10.2	0	49.97
2 multi	2	H						
8	1	11/29/2015	83	1627	3	10.4	0	52.5
2 multi	2	H						
8	1	11/30/2015	84	1628	3	10.3	0	53.64
2 multi	2	H						
8	1	12/01/2015	85	1629	3	10.4	0	56.14
2 multi	2	H						
8	1	12/02/2015	86	1630	3	10.4	0	45.57
2 multi	2	H						
8	1	12/03/2015	87	1631	3	10.2	0	51.06
2 multi	2	H						
8	1	12/04/2015	88	1632	3	9.6	0	50.47
2 multi	2	H						
8	1	12/05/2015	89	1633	3	10.3	0	51.39
2 multi	2	H						
8	1	12/06/2015	90	1634	3	10	0	49.91
2 multi	2	H						
8	1	12/07/2015	91	1635	3	10.2	0.13	52.24
2 multi	2	H						
8	1	12/08/2015	92	1636	3	10.2	0	51.95
2 multi	2	H						
8	1	12/09/2015	93	1637	3	10	0	51.18
2 multi	2	H						
8	1	12/10/2015	94	1638	3	10.4	0	52.72
2 multi	2	H						
8	1	12/11/2015	95	1639	3	10.2	0	49.79
2 multi	2	H						
8	1	12/12/2015	96	1640	3	10.2	0	52.4
2 multi	2	H						
8	1	12/13/2015	97	1641	3	10.2	0	51.83
2 multi	2	H						
8	1	12/14/2015	98	1642	3	10.3	0	51.15
2 multi	2	H						
8	1	12/15/2015	99	1643	3	9.8	0	47.97
2 multi	2	H						
8	1	12/16/2015	100	1644	3	10.6	0	52.15
2 multi	2	H						
8	1	12/17/2015	101	1645	3	10.6	0	51.89
2 multi	2	H						
8	1	12/18/2015	102	1646	3	10.4	0	53.08
2 multi	2	H						
8	1	12/19/2015	103	1647	3	10.2	0	49.83
2 multi	2	H						
8	1	12/20/2015	104	1648	3	10.3	0.06	51.12
2 multi	2	H						
8	1	12/21/2015	105	1649	3	10.2	0	51.67
2 multi	2	H						
8	1	12/22/2015	106	1650	3	10.6	0	52.12
2 multi	2	H						
8	1	12/23/2015	107	1651	3	10.2	0	51.86
2 multi	2	H						
8	1	12/24/2015	108	1652	3	10.4	0	50.35

2 multi 2		H						
8	1	12/25/2015	109	1653	3	10	0	48.88
2 multi 2		H						
8	1	12/26/2015	110	1654	3	10.2	0	37.96
2 multi 2		H						
8	1	12/27/2015	111	1655	3	10.6	0.01	36.28
2 multi 2		H						
8	1	12/28/2015	112	1656	3	11.4	0	40.95
2 multi 2		H						
8	1	12/29/2015	113	1657	3	11.7	0	38.43
2 multi 2		H						
8	1	12/30/2015	114	1658	3	10.7	0	43.63
2 multi 2		H						
8	1	12/31/2015	115	1659	3	10.7	0	39.38
2 multi 2		H						
8	1	01/01/2016	116	1660	3	10.9	0	44.54
2 multi 2		H						
8	1	01/02/2016	117	1661	3	10	0	40.37
2 multi 2		H						
8	1	01/03/2016	118	1662	3	9.8	0	38.82
2 multi 2		H						
8	1	01/04/2016	119	1663	3	10.9	0	41.37
2 multi 2		H						
8	1	01/05/2016	120	1664	3	10.3	0	38.74
2 multi 2		H						
8	1	01/06/2016	121	1665	3	10.9	0	35.57
2 multi 2		H						
8	1	01/07/2016	122	1666	3	10.6	0	38.09
2 multi 2		H						
8	1	01/08/2016	123	1667	3	10.9	0	38.1
2 multi 2		H						
8	1	01/09/2016	124	1668	3	10.7	0	40.33
2 multi 2		H						
8	1	01/10/2016	125	1669	3	10.6	0	39.77
2 multi 2		H						
8	1	01/11/2016	126	1670	3	10.2	0	38.68
2 multi 2		H						
8	1	01/12/2016	127	1671	3	10.4	0	42.52
2 multi 2		H						
8	1	01/13/2016	128	1672	3	10.3	0	42.62
2 multi 2		H						
8	1	01/14/2016	129	1673	3	9.9	0	41.49
2 multi 2		H						
8	1	01/15/2016	130	1674	3	10	0	39.65
2 multi 2		H						
8	1	01/16/2016	131	1675	3	10.6	0	43.96
2 multi 2		H						
8	1	01/17/2016	132	1676	3	10.7	0	43.06
2 multi 2		H						
8	1	01/18/2016	133	1677	3	10.7	0	40.54
2 multi 2		H						
8	1	01/19/2016	134	1678	3	10.7	0	43.8
2 multi 2		H						
8	1	01/20/2016	135	1679	3	10.7	0	45.87

2 multi	2	H						
8	1	01/21/2016	136	1680	3	10.4	0	40.53
2 multi	2	H						
8	1	01/22/2016	137	1681	3	10.2	0	42.84
2 multi	2	H						
8	1	01/23/2016	138	1682	3	10.7	0	42.61
2 multi	2	H						
8	1	01/24/2016	139	1683	3	11.1	0	40.82
2 multi	2	H						
8	1	01/25/2016	140	1684	3	10.7	0	40.64
2 multi	2	H						
8	1	01/26/2016	141	1685	3	11.3	0	45.1
2 multi	2	H						
8	1	01/27/2016	142	1686	3	10.7	0	43.25
2 multi	2	H						
8	1	01/28/2016	143	1687	3	11.1	0	44.1
2 multi	2	H						
8	1	01/29/2016	144	1688	3	10.9	0	41.04
2 multi	2	H						
8	1	01/30/2016	145	1689	3	10.7	0	44.42
2 multi	2	H						
8	1	01/31/2016	146	1690	3	10.7	0	40.97
2 multi	2	H						
8	1	02/01/2016	147	1691	3	10.9	0	46.36
2 multi	2	H						
8	1	02/02/2016	148	1692	3	10.7	0	45.54
2 multi	2	H						
8	1	02/03/2016	149	1693	3	10.7	0	44.57
2 multi	2	H						
8	1	02/04/2016	150	1694	3	10.4	0.15	43.33
2 multi	2	H						
8	1	02/05/2016	151	1695	3	10.7	0.16	43.91
2 multi	2	H						
8	1	02/06/2016	152	1696	3	10.9	0	44.37
2 multi	2	H						
8	1	02/07/2016	153	1697	3	10.7	0	45.49
2 multi	2	H						
8	1	02/08/2016	154	1698	3	10.7	0	43.7
2 multi	2	H						
8	1	02/09/2016	155	1699	3	10.9	0	41.72
2 multi	2	H						
8	1	02/10/2016	156	1700	3	11.1	0	45.01
2 multi	2	H						
8	1	02/11/2016	157	1701	3	10.7	0	44.53
2 multi	2	H						
8	1	02/12/2016	158	1702	3	10.7	0	44.46
2 multi	2	H						
8	1	02/13/2016	159	1703	3	10.7	0	43.88
2 multi	2	H						
8	1	02/14/2016	160	1704	3	11.1	0	43.51
2 multi	2	H						
8	1	02/15/2016	161	1705	3	10.7	0	44
2 multi	2	H						
8	1	02/16/2016	162	1706	3	10.6	0	44.39

2 multi	2	H						
8	1	02/17/2016	163	1707	3	11.7	0	43.38
2 multi	2	H						
8	1	02/18/2016	164	1708	3	10.9	0	41.07
2 multi	2	H						
8	1	02/19/2016	165	1709	3	11.1	0	42.49
2 multi	2	H						
8	1	02/20/2016	166	1710	3	11.7	0	41.26
2 multi	2	H						
8	1	02/21/2016	167	1711	3	11.4	0	42.57
2 multi	2	H						
8	1	02/22/2016	168	1712	3	11.7	0	41.9
2 multi	2	H						
8	1	02/23/2016	169	1713	3	11.1	0	42.26
2 multi	2	H						
8	1	02/24/2016	170	1714	3	10.7	0	42.98
2 multi	2	H						
8	1	02/25/2016	171	1715	3	11.4	0	42.29
2 multi	2	H						
8	1	02/26/2016	172	1716	3	11.1	0.01	42.64
2 multi	2	H						
8	1	02/27/2016	173	1717	3	10.9	0	41.41
2 multi	2	H						
8	1	02/28/2016	174	1718	3	11.9	0	42.86
2 multi	2	H						
8	1	02/29/2016	175	1719	3	11.3	0	42.47
2 multi	2	H						
8	1	03/01/2016	176	1720	3	10.7	0	42.36
2 multi	2	H						
8	1	03/02/2016	177	1721	3	10.9	0	40.89
2 multi	2	H						
8	1	03/03/2016	178	1722	3	11.7	0	42.21
2 multi	2	H						
8	1	03/04/2016	179	1723	3	11.4	0	38.9
2 multi	2	H						
8	1	03/05/2016	180	1724	3	11.4	0	42.5
2 multi	2	H						
8	1	03/06/2016	181	1725	3	11.1	0	43.26
2 multi	2	H						
8	1	03/07/2016	182	1726	3	11.4	0	42.23
2 multi	2	H						
8	1	03/08/2016	183	1727	3	11.4	0	41.32
2 multi	2	H						
8	1	03/09/2016	184	1728	3	11.1	0	43.38
2 multi	2	H						
8	1	03/10/2016	185	1729	3	11.3	0	41.58
2 multi	2	H						
8	1	03/11/2016	186	1730	3	10.7	0	41.74
2 multi	2	H						
8	1	03/12/2016	187	1731	3	11.4	0	41.9
2 multi	2	H						
8	1	03/13/2016	188	1732	3	11.4	0	39.5
2 multi	2	H						
8	1	03/14/2016	189	1733	3	11.3	0	38.57

2 multi	2	H						
8	1	03/15/2016	190	1734	3	11.3	0	39.87
2 multi	2	H						
8	1	03/16/2016	191	1735	3	11.1	0	37.96
2 multi	2	H						
8	1	03/17/2016	192	1736	3	11.3	0	39.37
2 multi	2	H						
8	1	03/18/2016	193	1737	3	12.6	0	39.95
2 multi	2	H						
8	1	03/19/2016	194	1738	3	12.3	0	36.97
2 multi	2	H						
8	1	03/20/2016	195	1739	3	11.9	0	37.41
2 multi	2	H						
8	1	03/21/2016	196	1740	3	11.7	0	39.28
2 multi	2	H						
8	1	03/22/2016	197	1741	3	11.7	0	38.68
2 multi	2	H						
8	1	03/23/2016	198	1742	3	11.3	0	37.89
2 multi	2	H						
8	1	03/24/2016	199	1743	3	12	0	38.4
2 multi	2	H						
8	1	01/26/2017	142	2051	4	10.2	0	49.88
2 multi	2	H						
8	1	01/27/2017	143	2052	4	11.3	0	49.27
2 multi	2	H						
8	1	01/28/2017	144	2053	4	10.2	0	48.81
2 multi	2	H						
8	1	01/29/2017	145	2054	4	9.9	0	48.26
2 multi	2	H						
8	1	01/30/2017	146	2055	4	9.9	0	47.89
2 multi	2	H						
8	1	01/31/2017	147	2056	4	10.2	0	45.02
2 multi	2	H						
8	1	02/01/2017	148	2057	4	10.3	0	66.63
2 multi	2	H						
8	1	02/02/2017	149	2058	4	10.7	0	44.24
2 multi	2	H						
8	1	02/03/2017	150	2059	4	11.1	0	44.37
2 multi	2	H						
8	1	02/04/2017	151	2060	4	11.9	0	45.95
2 multi	2	H						
8	1	02/05/2017	152	2061	4	11.4	0	45.55
2 multi	2	H						
8	1	02/06/2017	153	2062	4	11.1	0	45.65
2 multi	2	H						
8	1	02/07/2017	154	2063	4	11.1	0	46.01
2 multi	2	H						
8	1	02/08/2017	155	2064	4	11.3	0	44.88
2 multi	2	H						
8	1	02/09/2017	156	2065	4	11.3	0	43.98
2 multi	2	H						
8	1	02/10/2017	157	2066	4	11.3	0	47.37
2 multi	2	H						
8	1	02/11/2017	158	2067	4	11.1	0	45.08

2 multi	2	H						
8	1	02/12/2017	159	2068	4	10.7	0	45.52
2 multi	2	H						
8	1	02/13/2017	160	2069	4	10.4	0	44.94
2 multi	2	H						
8	1	02/14/2017	161	2070	4	11.9	0	48.8
2 multi	2	H						
8	1	02/15/2017	162	2071	4	12.3	0	40.84
2 multi	2	H						
8	1	02/16/2017	163	2072	4	11.1	0	42.95
2 multi	2	H						
8	1	02/17/2017	164	2073	4	11.3	0	40.15
2 multi	2	H						
8	1	02/18/2017	165	2074	4	11.4	0	43.79
2 multi	2	H						
8	1	02/19/2017	166	2075	4	11.7	0	44.01
2 multi	2	H						
8	1	02/20/2017	167	2076	4	11.3	0	40.64
2 multi	2	H						
8	1	02/21/2017	168	2077	4	11.3	0	43.02
2 multi	2	H						
8	1	02/22/2017	169	2078	4	11.3	0	40.64
2 multi	2	H						
8	1	02/23/2017	170	2079	4	10.4	0	42.62
2 multi	2	H						
8	1	02/24/2017	171	2080	4	11.1	0.14	43.57
2 multi	2	H						
8	1	02/25/2017	172	2081	4	10.6	0	41.69
2 multi	2	H						
8	1	02/26/2017	173	2082	4	10.2	0	44.23
2 multi	2	H						
8	1	02/27/2017	174	2083	4	12	0	43.43
2 multi	2	H						
8	1	02/28/2017	175	2084	4	11.7	0	43.02
2 multi	2	H						
8	1	03/01/2017	176	2085	4	12	0.03	41.26
2 multi	2	H						
8	1	03/02/2017	177	2086	4	11.9	0	42.12
2 multi	2	H						
8	1	03/03/2017	178	2087	4	12	0	40.79
2 multi	2	H						
8	1	03/04/2017	179	2088	4	11.9	0	39.79
2 multi	2	H						
8	1	03/05/2017	180	2089	4	11.9	0	41.21
2 multi	2	H						
8	1	03/06/2017	181	2090	4	11.7	0	40.25
2 multi	2	H						
8	1	03/07/2017	182	2091	4	11.7	0	43.3
2 multi	2	H						
8	1	03/08/2017	183	2092	4	11.7	0.02	38.92
2 multi	2	H						
8	1	03/09/2017	184	2093	4	11.3	0.06	41.09
2 multi	2	H						
8	1	03/10/2017	185	2094	4	11.4	0	41.21

2 multi	2	H						
8	1	03/11/2017	186	2095	4	11.7	0.11	41.93
2 multi	2	H						
8	1	03/12/2017	187	2096	4	10	0	40.56
2 multi	2	H						
8	1	03/13/2017	188	2097	4	11.1	0	39.95
2 multi	2	H						
8	1	03/14/2017	189	2098	4	11.4	0.11	40.99
2 multi	2	H						
8	1	03/15/2017	190	2099	4	12.5	0	41.85
2 multi	2	H						
8	1	03/16/2017	191	2100	4	12	0.13	40.13
2 multi	2	H						
8	1	03/17/2017	192	2101	4	11.3	0	39.91
2 multi	2	H						
8	1	03/18/2017	193	2102	4	12.5	0	41.85
2 multi	2	H						
8	1	03/19/2017	194	2103	4	12.5	0	40.99
2 multi	2	H						
8	1	03/20/2017	195	2104	4	11.9	0	43.94
2 multi	2	H						
8	1	03/21/2017	196	2105	4	11.7	0	41
2 multi	2	H						
8	1	03/22/2017	197	2106	4	11.4	0	40.49
2 multi	2	H						
8	1	03/23/2017	198	2107	4	11.9	0	42.42
2 multi	2	H						
8	1	03/24/2017	199	2108	4	13.5	0	41.84
2 multi	2	H						
8	1	03/25/2017	200	2109	4	13.5	0	38.54
2 multi	2	H						
8	1	03/26/2017	201	2110	4	10.7	0	34.01
2 multi	2	H						
8	1	03/27/2017	202	2111	4	11.7	0	38.19
2 multi	2	H						
8	1	03/28/2017	203	2112	4	13.1	0.01	37.5
2 multi	2	H						
8	1	03/29/2017	204	2113	4	11.9	0	39.08
2 multi	2	H						
8	1	03/30/2017	205	2114	4	13.1	0	38.1
2 multi	2	H						
8	1	03/31/2017	206	2115	4	12.5	0.07	37.8
2 multi	2	H						
8	1	04/01/2017	207	2116	4	12.5	0	40.05
2 multi	2	H						
8	1	04/02/2017	208	2117	4	13.5	0	40.58
2 multi	2	H						
8	1	04/03/2017	209	2118	4	13.1	0	38.9
2 multi	2	H						
8	1	04/04/2017	210	2119	4	12.5	0	34.99
2 multi	2	H						
8	1	04/05/2017	211	2120	4	10.3	0	32.09
2 multi	2	H						
8	1	04/06/2017	212	2121	4	13.6	0	38.01

2	multi	2	H						
8		1	04/07/2017	213	2122	4	13.1	0	37.23
2	multi	2	H						
8		1	04/08/2017	214	2123	4	10.3	0	36.71
2	multi	2	H						
8		1	04/09/2017	215	2124	4	10.2	0	38.48
2	multi	2	H						
8		1	04/10/2017	216	2125	4	12.5	0	37.35
2	multi	2	H						
8		1	04/11/2017	217	2126	4	12.5	0.68	39.31
2	multi	2	H						
8		1	04/12/2017	218	2127	4	13.1	0	38.37
2	multi	2	H						
8		1	11/08/2017	7	2337	5	10.4	0	41.1
2	multi	1	H						
8		1	11/09/2017	8	2338	5	10.6	0	42.96
2	multi	1	H						
8		1	11/10/2017	9	2339	5	10.4	0	36.07
2	multi	1	H						
8		1	11/11/2017	10	2340	5	10.3	0	43.99
2	multi	1	H						
8		1	11/12/2017	11	2341	5	10.9	0	40.36
2	multi	1	H						
8		1	11/13/2017	12	2342	5	11.3	0	41.69
2	multi	1	H						
8		1	11/14/2017	13	2343	5	10.7	0	44.03
2	multi	1	H						
8		1	03/19/2018	138	2468	5	12	0	49.92
2	multi	2	H						
8		1	03/20/2018	139	2469	5	11.7	0.02	48.89
2	multi	2	H						
8		1	03/21/2018	140	2470	5	11.3	0.23	48.81
2	multi	2	H						
8		1	03/22/2018	141	2471	5	11.3	0	49.79
2	multi	2	H						
8		1	03/23/2018	142	2472	5	11.3	0.21	46.75
2	multi	2	H						
8		1	03/24/2018	143	2473	5	11.4	0	46.73
2	multi	2	H						
8		1	03/25/2018	144	2474	5	11.4	0	49.37
2	multi	2	H						
8		1	03/26/2018	145	2475	5	10.9	0	48.21
2	multi	2	H						
8		1	03/27/2018	146	2476	5	11.4	0	45.33
2	multi	2	H						
8		1	03/28/2018	147	2477	5	11.4	0	51.39
2	multi	2	H						
8		1	03/29/2018	148	2478	5	11.3	0	47.78
2	multi	2	H						
8		1	03/30/2018	149	2479	5	11.1	0	46.23
2	multi	2	H						
8		1	03/31/2018	150	2480	5	10.3	0	47.31
2	multi	2	H						
8		1	04/01/2018	151	2481	5	10.4	0	44.27

2 multi	2	H						
8	1	04/02/2018	152	2482	5	11.7	0	50.43
2 multi	2	H						
8	1	04/03/2018	153	2483	5	11.7	0	49.41
2 multi	2	H						
8	1	04/04/2018	154	2484	5	11.7	0	47.77
2 multi	2	H						
8	1	04/05/2018	155	2485	5	11.9	0	44.67
2 multi	2	H						
8	1	04/06/2018	156	2486	5	11.7	0	44.7
2 multi	2	H						
8	1	04/07/2018	157	2487	5	12	0.03	44.52
2 multi	2	H						
8	1	04/08/2018	158	2488	5	12.3	0.05	49.27
2 multi	2	H						
8	1	04/09/2018	159	2489	5	11.9	0	47.03
2 multi	2	H						
8	1	04/10/2018	160	2490	5	11.4	0	43.68
2 multi	2	H						
8	1	04/11/2018	161	2491	5	11.4	0	40.68
2 multi	2	H						
8	1	04/12/2018	162	2492	5	11.9	0	45.22
2 multi	2	H						
8	1	04/13/2018	163	2493	5	12.3	0	39.27
2 multi	2	H						
8	1	04/14/2018	164	2494	5	11.4	0	37.8
2 multi	2	H						
8	1	04/15/2018	165	2495	5	12	0.05	38.21
2 multi	2	H						
8	1	04/16/2018	166	2496	5	13.5	0	39.1
2 multi	2	H						
8	1	04/17/2018	167	2497	5	12.6	0.03	42.22
2 multi	2	H						
8	1	04/18/2018	168	2498	5	0	0.06	20.9
2 multi	2	H						
8	1	04/19/2018	169	2499	5	0	0	20.83
2 multi	2	H						
8	1	04/20/2018	170	2500	5	11.9	0.19	42.28
2 multi	2	H						
8	1	04/21/2018	171	2501	5	11.9	0	43.93
2 multi	2	H						
8	1	04/22/2018	172	2502	5	12.9	0.02	44.97
2 multi	2	H						
8	1	04/23/2018	173	2503	5	12.3	0	44.15
2 multi	2	H						
8	1	04/24/2018	174	2504	5	11.9	0.01	43.39
2 multi	2	H						
8	1	04/25/2018	175	2505	5	11.4	0	42.1
2 multi	2	H						
8	1	04/26/2018	176	2506	5	12.3	0	44.46
2 multi	2	H						
8	1	04/27/2018	177	2507	5	12.5	0.16	43.42
2 multi	2	H						
8	1	04/28/2018	178	2508	5	10.9	0	42.34

2	multi	2	H						
8		1	04/29/2018	179	2509	5	10.7	0.05	45.43
2	multi	2	H						
8		1	04/30/2018	180	2510	5	11.4	0	42.61
2	multi	2	H						
8		1	05/01/2018	181	2511	5	12.5	0.25	44.49
2	multi	2	H						
8		1	05/02/2018	182	2512	5	13.1	0	44.4
2	multi	2	H						
8		1	05/03/2018	183	2513	5	12.6	0	67.45
2	multi	2	H						
8		1	05/04/2018	184	2514	5	12.5	0	42.03
2	multi	2	H						
8		1	05/05/2018	185	2515	5	12.6	0	42.19
2	multi	2	H						
8		1	05/06/2018	186	2516	5	12.6	0	41.87
2	multi	2	H						
8		1	05/07/2018	187	2517	5	12.6	0	42.43
2	multi	2	H						
8		1	05/08/2018	188	2518	5	12.9	0	41.3
2	multi	2	H						
8		1	05/09/2018	189	2519	5	12.9	0.05	40.99
2	multi	2	H						
8		1	05/10/2018	190	2520	5	13.5	0	40.84
2	multi	2	H						
8		1	05/11/2018	191	2521	5	13.6	0	43.03
2	multi	2	H						
8		1	05/12/2018	192	2522	5	11.4	0	42.83
2	multi	2	H						
8		1	05/13/2018	193	2523	5	12.3	0.01	44.11
2	multi	2	H						
8		1	05/14/2018	194	2524	5	12.5	0	44.18
2	multi	2	H						
8		1	05/15/2018	195	2525	5	12.6	0	42.19
2	multi	2	H						
8		1	05/16/2018	196	2526	5	12.9	0	45.45
2	multi	2	H						
8		1	05/17/2018	197	2527	5	12.9	0.05	41.4
2	multi	2	H						
8		1	05/18/2018	198	2528	5	13.6	0	41.57
2	multi	2	H						
8		1	05/19/2018	199	2529	5	13.1	0	39.62
2	multi	2	H						
8		1	05/20/2018	200	2530	5	10.9	0	42.6
2	multi	2	H						
8		1	05/21/2018	201	2531	5	13.1	0	39.95
2	multi	2	H						
8		1	05/22/2018	202	2532	5	11.1	0	40
2	multi	2	H						
8		1	05/23/2018	203	2533	5	13.6	0.01	39.88
2	multi	2	H						
8		1	05/24/2018	204	2534	5	11.3	0	43.52
2	multi	2	H						
8		1	05/25/2018	205	2535	5	12.6	0.05	44.22

2 multi	2	H						
8	1	05/26/2018	206	2536	5	12.6	0	39.55
2 multi	2	H						
8	1	05/27/2018	207	2537	5	13.1	0	42.26
2 multi	2	H						
8	1	05/28/2018	208	2538	5	12.9	0.03	40.68
2 multi	2	H						
8	1	05/29/2018	209	2539	5	12.5	0	39.28
2 multi	2	H						
8	1	05/30/2018	210	2540	5	13.5	0.06	40.47
2 multi	2	H						
8	1	05/31/2018	211	2541	5	13.5	0.01	39.47
2 multi	2	H						
8	1	06/01/2018	212	2542	5	13.5	0.06	39.78
2 multi	2	H						
8	1	06/02/2018	213	2543	5	13.8	0.01	40.42
2 multi	2	H						
8	1	06/03/2018	214	2544	5	13.5	0	38.91
2 multi	2	H						
8	1	06/04/2018	215	2545	5	13.1	0	39.09
2 multi	2	H						
8	1	06/05/2018	216	2546	5	13.8	0.14	38.73
2 multi	2	H						
8	1	06/06/2018	217	2547	5	13.6	0	37.73
2 multi	2	H						
8	1	06/07/2018	218	2548	5	13.5	0.07	41.3
2 multi	2	H						
8	1	06/08/2018	219	2549	5	12.3	0.05	39.77
2 multi	2	H						
8	1	06/09/2018	220	2550	5	11.3	0.02	41.64
2 multi	2	H						
8	1	06/10/2018	221	2551	5	11.3	0	38.57
2 multi	2	H						
8	1	06/11/2018	222	2552	5	13.8	0.05	38.87
2 multi	2	H						
8	1	06/12/2018	223	2553	5	12.5	0	39.06
2 multi	2	H						
8	1	06/13/2018	224	2554	5	12.9	0.17	40.86
2 multi	2	H						
8	1	06/14/2018	225	2555	5	12.5	0.15	41.14
2 multi	2	H						
8	1	06/15/2018	226	2556	5	13.6	0.12	39.22
2 multi	2	H						
8	1	06/16/2018	227	2557	5	12.6	0.25	39.14
2 multi	2	H						
8	1	06/17/2018	228	2558	5	12.6	0	38.36
2 multi	2	H						
8	1	06/18/2018	229	2559	5	12.9	0	37.02
2 multi	2	H						
8	1	06/19/2018	230	2560	5	12.5	0	38.89
2 multi	2	H						
8	1	06/20/2018	231	2561	5	12.3	0	37.42
2 multi	2	H						
8	1	06/21/2018	232	2562	5	12.9	0.01	37.56

2 multi	2	H						
8	1	06/22/2018	233	2563	5	12.5	0.23	36.13
2 multi	2	H						
8	1	06/23/2018	234	2564	5	12.6	0	35.97
2 multi	2	H						
8	1	06/24/2018	235	2565	5	12.9	0.01	38.89
2 multi	2	H						
8	1	06/25/2018	236	2566	5	12.5	0.05	41.36
2 multi	2	H						
8	1	06/26/2018	237	2567	5	12	0	37.99
2 multi	2	H						
8	1	06/27/2018	238	2568	5	12.5	0.08	41.08
2 multi	2	H						
8	1	06/28/2018	239	2569	5	12.6	0.25	37.02
2 multi	2	H						
8	1	06/29/2018	240	2570	5	12	0.06	38.29
2 multi	2	H						
8	1	06/30/2018	241	2571	5	13.1	0.17	34.27
2 multi	2	H						
8	1	07/01/2018	242	2572	5	12.9	0.02	35.02
2 multi	2	H						
8	1	07/02/2018	243	2573	5	12.9	0.01	35.25
2 multi	2	H						
8	1	07/03/2018	244	2574	5	12.9	0.07	34.47
2 multi	2	H						
8	1	07/04/2018	245	2575	5	12.6	0.01	28.99
2 multi	2	H						
8	1	07/05/2018	246	2576	5	12	0.16	35.5
2 multi	2	H						
8	1	07/06/2018	247	2577	5	12.3	0.03	30.14
2 multi	2	H						
8	1	07/07/2018	248	2578	5	10.6	0	30.61
2 multi	2	H						
8	1	07/08/2018	249	2579	5	11.9	0.09	32.78
2 multi	2	H						
8	1	07/09/2018	250	2580	5	13.1	0.01	34.08
2 multi	2	H						
8	1	07/10/2018	251	2581	5	12.6	0	33.43
2 multi	2	H						
8	1	07/11/2018	252	2582	5	12.5	0.01	32.38
2 multi	2	H						
8	1	07/12/2018	253	2583	5	11.9	0.25	30.64
2 multi	2	H						
8	1	12/08/2018	9	2732	6	9.3	0.01	33.06
2 multi	1	H						
8	1	12/09/2018	10	2733	6	9.1	0.01	31.48
2 multi	1	H						
8	1	12/10/2018	11	2734	6	9.5	0.02	33.97
2 multi	1	H						
8	1	12/11/2018	12	2735	6	9.8	0	41.4
2 multi	1	H						
8	1	12/12/2018	13	2736	6	9.6	0.01	42.33
2 multi	1	H						
8	1	12/13/2018	14	2737	6	9.6	0.03	42.63

2 multi	1	H						
8	1	12/14/2018	15	2738	6	9.8	0	42.68
2 multi	1	H						
8	1	12/15/2018	16	2739	6	9.6	0	42.76
2 multi	1	H						
8	1	12/16/2018	17	2740	6	9.5	0	44.95
2 multi	1	H						
8	1	12/17/2018	18	2741	6	9.5	0	45.36
2 multi	1	H						
8	1	12/18/2018	19	2742	6	9.5	0	48.3
2 multi	1	H						
8	1	12/19/2018	20	2743	6	9.5	0	49.11
2 multi	1	H						
8	1	12/20/2018	21	2744	6	9.5	0	47.56
2 multi	1	H						
8	1	12/21/2018	22	2745	6	9.5	0.01	48.73
2 multi	2	H						
8	1	12/22/2018	23	2746	6	9.3	0	47.2
2 multi	2	H						
8	1	12/23/2018	24	2747	6	8.8	0	47.23
2 multi	2	H						
8	1	12/24/2018	25	2748	6	9.3	0	47.17
2 multi	2	H						
8	1	12/25/2018	26	2749	6	9.8	0	53.66
2 multi	2	H						
8	1	12/26/2018	27	2750	6	9.8	0	51.72
2 multi	2	H						
8	1	12/27/2018	28	2751	6	9.8	0	52.81
2 multi	2	H						
8	1	12/28/2018	29	2752	6	9.5	0	49.43
2 multi	2	H						
8	1	12/29/2018	30	2753	6	9.8	0	53.04
2 multi	2	H						
8	1	12/30/2018	31	2754	6	9.2	0.17	50.56
2 multi	2	H						
8	1	12/31/2018	32	2755	6	9.5	0	52.63
2 multi	2	H						
8	1	01/01/2019	33	2756	6	9.5	0.15	51.58
2 multi	2	H						
8	1	01/02/2019	34	2757	6	9.3	0	49.34
2 multi	2	H						
8	1	01/03/2019	35	2758	6	9.3	0	55.01
2 multi	2	H						
8	1	01/04/2019	36	2759	6	9.5	0	53.61
2 multi	2	H						
8	1	01/05/2019	37	2760	6	9.3	0	51.19
2 multi	2	H						
8	1	01/06/2019	38	2761	6	9.2	0	52.66
2 multi	2	H						
8	1	01/07/2019	39	2762	6	9.9	0	54.18
2 multi	2	H						
8	1	01/08/2019	40	2763	6	9.2	0	49.97
2 multi	2	H						
8	1	01/09/2019	41	2764	6	9.2	0	54.77

2 multi	2	H						
8	1	01/10/2019	42	2765	6	9.5	0	54.41
2 multi	2	H						
8	1	01/11/2019	43	2766	6	9.5	0	48.83
2 multi	2	H						
8	1	01/12/2019	44	2767	6	9.8	0	51.19
2 multi	2	H						
8	1	01/13/2019	45	2768	6	9.3	0	50.45
2 multi	2	H						
8	1	01/14/2019	46	2769	6	9.3	0	47.4
2 multi	2	H						
8	1	01/15/2019	47	2770	6	9.2	0	47.64
2 multi	2	H						
8	1	01/16/2019	48	2771	6	9.3	0	52.68
2 multi	2	H						
8	1	01/17/2019	49	2772	6	9.8	0	51.15
2 multi	2	H						
8	1	01/18/2019	50	2773	6	9.9	0	52.59
2 multi	2	H						
8	1	01/19/2019	51	2774	6	9.9	0	50.31
2 multi	2	H						
8	1	01/20/2019	52	2775	6	9.8	0	55.74
2 multi	2	H						
8	1	01/21/2019	53	2776	6	9.6	0	56.87
2 multi	2	H						
8	1	01/22/2019	54	2777	6	9.6	0	52.7
2 multi	2	H						
8	1	01/23/2019	55	2778	6	9.3	0	55.92
2 multi	2	H						
8	1	01/24/2019	56	2779	6	9.5	0.12	53.48
2 multi	2	H						
8	1	01/25/2019	57	2780	6	9.3	0	56.21
2 multi	2	H						
8	1	01/26/2019	58	2781	6	9.5	0	53.31
2 multi	2	H						
8	1	01/27/2019	59	2782	6	9.5	0	49.34
2 multi	2	H						
8	1	01/28/2019	60	2783	6	9.2	0	54.5
2 multi	2	H						
8	1	01/29/2019	61	2784	6	9.5	0	51.98
2 multi	2	H						
8	1	01/30/2019	62	2785	6	9.1	0	54.62
2 multi	2	H						
8	1	01/31/2019	63	2786	6	9.2	0	55.51
2 multi	2	H						
8	1	02/01/2019	64	2787	6	9.3	0	49.46
2 multi	2	H						
8	1	02/02/2019	65	2788	6	9.3	0	53.87
2 multi	2	H						
8	1	02/03/2019	66	2789	6	9.5	0.42	51.91
2 multi	2	H						
8	1	02/04/2019	67	2790	6	9.6	0	54.29
2 multi	2	H						
8	1	02/05/2019	68	2791	6	9.3	0	55.3

2 multi	2	H						
8	1	02/06/2019	69	2792	6	9.5	0	52.07
2 multi	2	H						
8	1	02/07/2019	70	2793	6	9.3	0	55.55
2 multi	2	H						
8	1	02/08/2019	71	2794	6	9.6	0	54.23
2 multi	2	H						
8	1	02/09/2019	72	2795	6	9.6	0	53.21
2 multi	2	H						
8	1	02/10/2019	73	2796	6	9.6	0	52.97
2 multi	2	H						
8	1	02/11/2019	74	2797	6	9.6	0	53.21
2 multi	2	H						
8	1	02/12/2019	75	2798	6	9.8	0	53.91
2 multi	2	H						
8	1	02/13/2019	76	2799	6	9.6	0.13	52.2
2 multi	2	H						
8	1	02/14/2019	77	2800	6	9.6	0	52.7
2 multi	2	H						
8	1	02/15/2019	78	2801	6	9.8	0	53.4
2 multi	2	H						
8	1	02/16/2019	79	2802	6	9.3	0	51.22
2 multi	2	H						
8	1	02/17/2019	80	2803	6	9.3	0	49.62
2 multi	2	H						
8	1	02/18/2019	81	2804	6	9.6	0	52.93
2 multi	2	H						
8	1	02/19/2019	82	2805	6	9.9	0	50.1
2 multi	2	H						
8	1	02/20/2019	83	2806	6	9.8	0	54.24
2 multi	2	H						
8	1	02/21/2019	84	2807	6	9.8	0	52.05
2 multi	2	H						
8	1	02/22/2019	85	2808	6	10	0	51.76
2 multi	2	H						
8	1	02/23/2019	86	2809	6	10	0	49.11
2 multi	2	H						
8	1	02/24/2019	87	2810	6	10	0	53.98
2 multi	2	H						
8	1	02/25/2019	88	2811	6	10	0	51.51
2 multi	2	H						
8	1	02/26/2019	89	2812	6	9.9	0	51.56
2 multi	2	H						
8	1	02/27/2019	90	2813	6	9.8	0	55.01
2 multi	2	H						
8	1	02/28/2019	91	2814	6	9.8	0	55.98
2 multi	2	H						
8	1	03/01/2019	92	2815	6	9.9	0	54.35
2 multi	2	H						
8	1	03/02/2019	93	2816	6	10	0	54.13
2 multi	2	H						
8	1	03/03/2019	94	2817	6	9.9	0.14	54.27
2 multi	2	H						
8	1	03/04/2019	95	2818	6	9.9	0	57.47

2	multi	2	H						
8		1	03/05/2019	96	2819	6	9.9	0	54.6
2	multi	2	H						
8		1	03/06/2019	97	2820	6	10	0	57.4
2	multi	2	H						
8		1	03/07/2019	98	2821	6	10	0	50.55
2	multi	2	H						
8		1	03/08/2019	99	2822	6	10	0	57.84
2	multi	2	H						
8		1	03/09/2019	100	2823	6	10.4	0	54.74
2	multi	2	H						
8		1	03/10/2019	101	2824	6	9.9	0	51.41
2	multi	2	H						
8		1	03/11/2019	102	2825	6	9.9	0	50.65
2	multi	2	H						
8		1	03/12/2019	103	2826	6	9.8	0	52.33
2	multi	2	H						
8		1	03/13/2019	104	2827	6	9.9	0	51.1
2	multi	2	H						
8		1	03/14/2019	105	2828	6	10	0	55.87
2	multi	2	H						
8		1	03/15/2019	106	2829	6	9.8	0	52.44
2	multi	2	H						
8		1	03/16/2019	107	2830	6	9.6	0	54.07
2	multi	2	H						
8		1	03/17/2019	108	2831	6	9.8	0	53.78
2	multi	2	H						
8		1	03/18/2019	109	2832	6	9.8	0	51.08
2	multi	2	H						
8		1	03/19/2019	110	2833	6	9.8	0	53.22
2	multi	2	H						
8		1	03/20/2019	111	2834	6	9.9	0	54.99
2	multi	2	H						
8		1	03/21/2019	112	2835	6	9.8	0	52.45
2	multi	2	H						
8		1	03/22/2019	113	2836	6	10.9	0	53.11
2	multi	2	H						
8		1	03/23/2019	114	2837	6	10.2	0	51.76
2	multi	2	H						
8		1	03/24/2019	115	2838	6	10.2	0	52.38
2	multi	2	H						
8		1	03/25/2019	116	2839	6	10.6	0	50.82
2	multi	2	H						
8		1	03/26/2019	117	2840	6	10.3	0	49.94
2	multi	2	H						
8		1	03/27/2019	118	2841	6	10	0	50.4
2	multi	2	H						
8		1	01/27/2020	5	3147	7	9.2	0.11	28.33
2	multi	1	H						
8		1	01/28/2020	6	3148	7	9.9	0.03	26.57
2	multi	1	H						
8		1	01/29/2020	7	3149	7	9.6	0.04	30.62
2	multi	1	H						
8		1	01/30/2020	8	3150	7	9.9	0.05	35.7

2 multi 1		H						
8	1	01/31/2020	9	3151	7	9.8	0.05	36.9
2 multi 1		H						
8	1	02/01/2020	10	3152	7	9.8	0.03	40.51
2 multi 1		H						
8	1	02/02/2020	11	3153	7	9.8	0.41	37.26
2 multi 1		H						
8	1	02/03/2020	12	3154	7	9.6	0.02	38.9
2 multi 1		H						
8	1	02/04/2020	13	3155	7	9.8	0.28	41.02
2 multi 1		H						
8	1	02/05/2020	14	3156	7	9.8	0.12	40.69
2 multi 1		H						
8	1	02/06/2020	15	3157	7	9.6	0	38.61
2 multi 1		H						
8	1	02/07/2020	16	3158	7	9.6	0	42.21
2 multi 1		H						
8	1	02/08/2020	17	3159	7	9.6	0	45.37
2 multi 1		H						
8	1	02/09/2020	18	3160	7	9.8	0	49.48
2 multi 1		H						
8	1	02/10/2020	19	3161	7	10	0.1	46.18
2 multi 1		H						
8	1	02/11/2020	20	3162	7	10	0.02	51.33
2 multi 1		H						
8	1	02/12/2020	21	3163	7	9.9	0.02	52.46
2 multi 1		H						
8	1	02/13/2020	22	3164	7	10	0.41	51.33
2 multi 2		H						
8	1	02/14/2020	23	3165	7	10	0	48.77
2 multi 2		H						
8	1	02/15/2020	24	3166	7	10	0	51.9
2 multi 2		H						
8	1	02/16/2020	25	3167	7	10	0	56.71
2 multi 2		H						
8	1	02/17/2020	26	3168	7	9.9	0.08	53.19
2 multi 2		H						
8	1	02/18/2020	27	3169	7	9.9	0	58.5
2 multi 2		H						
8	1	02/19/2020	28	3170	7	9.9	0	56.67
2 multi 2		H						
8	1	02/20/2020	29	3171	7	10	0	51.65
2 multi 2		H						
8	1	02/21/2020	30	3172	7	9.6	0	56.22
2 multi 2		H						
8	1	02/22/2020	31	3173	7	9.5	0	56.65
2 multi 2		H						
8	1	02/23/2020	32	3174	7	9.3	0	59.59
2 multi 2		H						
8	1	02/24/2020	33	3175	7	9.6	0	57.71
2 multi 2		H						
8	1	02/25/2020	34	3176	7	9.6	0	60.01
2 multi 2		H						
8	1	02/26/2020	35	3177	7	9.9	0	59.33

2 multi	2	H						
8	1	02/27/2020	36	3178	7	9.9	0	57.98
2 multi	2	H						
8	1	02/28/2020	37	3179	7	9.9	0	58.79
2 multi	2	H						
8	1	02/29/2020	38	3180	7	9.8	0	58.08
2 multi	2	H						
8	1	03/01/2020	39	3181	7	10	0.36	57.12
2 multi	2	H						
8	1	03/02/2020	40	3182	7	10	0	61.14
2 multi	2	H						
8	1	03/03/2020	41	3183	7	10	0.05	65.07
2 multi	2	H						
8	1	03/04/2020	42	3184	7	10.2	0	58.47
2 multi	2	H						
8	1	03/05/2020	43	3185	7	10.2	0	59.98
2 multi	2	H						
8	1	03/06/2020	44	3186	7	9.9	0	64.04
2 multi	2	H						
8	1	03/07/2020	45	3187	7	9.8	0	62.2
2 multi	2	H						
8	1	03/08/2020	46	3188	7	9.6	0	62.45
2 multi	2	H						
8	1	03/09/2020	47	3189	7	9.8	0	62.5
2 multi	2	H						
8	1	03/10/2020	48	3190	7	9.3	0	56.72
2 multi	2	H						
8	1	03/11/2020	49	3191	7	9.8	0	59.41
2 multi	2	H						
8	1	03/12/2020	50	3192	7	9.8	0	59.49
2 multi	2	H						
8	1	03/13/2020	51	3193	7	9.8	0	57.24
2 multi	2	H						
8	1	03/14/2020	52	3194	7	9.8	0	57.52
2 multi	2	H						
8	1	03/15/2020	53	3195	7	9.9	0	58.66
2 multi	2	H						
8	1	03/16/2020	54	3196	7	9.9	0.02	63.04
2 multi	2	H						
8	1	03/17/2020	55	3197	7	9.8	0	63.41
2 multi	2	H						
8	1	03/18/2020	56	3198	7	10.4	0	57.46
2 multi	2	H						
8	1	03/19/2020	57	3199	7	10	0	63.31
2 multi	2	H						
8	1	03/20/2020	58	3200	7	9.9	0	66.21
2 multi	2	H						
8	1	03/21/2020	59	3201	7	10	0	61.15
2 multi	2	H						
8	1	03/22/2020	60	3202	7	9.6	0	54.69
2 multi	2	H						
8	1	03/23/2020	61	3203	7	10	0.55	57.01
2 multi	2	H						
8	1	03/24/2020	62	3204	7	9.8	0.14	59.25

2 multi	2	H						
8	1	03/25/2020	63	3205	7	10	0	60.08
2 multi	2	H						
8	1	03/26/2020	64	3206	7	9.9	0	64.41
2 multi	2	H						
8	1	03/27/2020	65	3207	7	10	0.06	63.59
2 multi	2	H						
8	1	03/28/2020	66	3208	7	10.2	0	63.9
2 multi	2	H						
8	1	03/29/2020	67	3209	7	10.2	0.47	59.41
2 multi	2	H						
8	1	03/30/2020	68	3210	7	10	0	61.29
2 multi	2	H						
8	1	03/31/2020	69	3211	7	9.9	0.52	63.78
2 multi	2	H						
8	1	04/01/2020	70	3212	7	10	0.33	62.71
2 multi	2	H						
8	1	04/02/2020	71	3213	7	10	0	61.36
2 multi	2	H						
8	1	04/03/2020	72	3214	7	10.3	0	64.75
2 multi	2	H						
8	1	04/04/2020	73	3215	7	10	0	60.38
2 multi	2	H						
8	1	04/05/2020	74	3216	7	10	0	63.09
2 multi	2	H						
8	1	04/06/2020	75	3217	7	9.9	0	59.31
2 multi	2	H						
8	1	04/07/2020	76	3218	7	9.9	0.03	57.92
2 multi	2	H						
8	1	04/08/2020	77	3219	7	10	0	63.49
2 multi	2	H						
8	1	04/09/2020	78	3220	7	9.9	0	56.32
2 multi	2	H						
8	1	04/10/2020	79	3221	7	10.2	0	60.42
2 multi	2	H						
8	1	04/11/2020	80	3222	7	10	0.08	59.18
2 multi	2	H						
8	1	04/12/2020	81	3223	7	10	0	64.98
2 multi	2	H						
8	1	04/13/2020	82	3224	7	10.3	0	64.76
2 multi	2	H						
8	1	04/14/2020	83	3225	7	10.2	0	61.32
2 multi	2	H						
8	1	04/15/2020	84	3226	7	10.2	0	60.74
2 multi	2	H						
8	1	04/16/2020	85	3227	7	10.3	0	62.05
2 multi	2	H						
8	1	04/17/2020	86	3228	7	9.8	0	54.36
2 multi	2	H						
8	1	04/18/2020	87	3229	7	10	0	56.27
2 multi	2	H						
8	1	04/19/2020	88	3230	7	10.2	0	58.84
2 multi	2	H						
8	1	04/20/2020	89	3231	7	9.9	0	61.71

2 multi	2	H						
8	1	04/21/2020	90	3232	7	10.2	0	53.23
2 multi	2	H						
8	1	04/22/2020	91	3233	7	9.9	0.22	56.75
2 multi	2	H						
8	1	04/23/2020	92	3234	7	10.2	0	54.48
2 multi	2	H						
8	1	04/24/2020	93	3235	7	10.3	0	56.33
2 multi	2	H						
8	1	04/25/2020	94	3236	7	10	0	59.41
2 multi	2	H						
8	1	04/26/2020	95	3237	7	9.8	0	56.92
2 multi	2	H						
8	1	04/27/2020	96	3238	7	10	0	52.44
2 multi	2	H						
8	1	04/28/2020	97	3239	7	10.2	0	60.17
2 multi	2	H						
8	1	04/29/2020	98	3240	7	9.9	0.13	53.89
2 multi	2	H						
8	1	04/30/2020	99	3241	7	9.8	0	61.09
2 multi	2	H						
8	1	05/01/2020	100	3242	7	10	0	52.18
2 multi	2	H						
8	1	05/02/2020	101	3243	7	10	0	58.61
2 multi	2	H						
8	1	05/03/2020	102	3244	7	10	0	54.28
2 multi	2	H						
8	1	05/04/2020	103	3245	7	10.2	0.01	55.2
2 multi	2	H						
8	1	05/05/2020	104	3246	7	10	0	57.61
2 multi	2	H						
8	1	05/06/2020	105	3247	7	10.2	0	53.68
2 multi	2	H						
8	1	05/07/2020	106	3248	7	9.9	0	59.63
2 multi	2	H						
8	1	05/08/2020	107	3249	7	10	0	57.42
2 multi	2	H						
8	1	05/09/2020	108	3250	7	10.2	0.16	48.35
2 multi	2	H						
8	1	05/10/2020	109	3251	7	10.2	0	54.76
2 multi	2	H						
8	1	05/11/2020	110	3252	7	10	0	49.67
2 multi	2	H						
8	1	05/12/2020	111	3253	7	10	0	51.28
2 multi	2	H						
8	1	05/13/2020	112	3254	7	10.3	0	52.79
2 multi	2	H						
8	1	05/14/2020	113	3255	7	10.2	0	52.85
2 multi	2	H						
8	1	05/15/2020	114	3256	7	10.2	0	47.82
2 multi	2	H						
8	1	05/16/2020	115	3257	7	10.2	0	54.61
2 multi	2	H						
8	1	05/17/2020	116	3258	7	10	0	52.89

2 multi	2	H						
8	1	05/18/2020	117	3259	7	10.2	0	52.07
2 multi	2	H						
8	1	05/19/2020	118	3260	7	9.9	0	48.67
2 multi	2	H						
8	1	05/20/2020	119	3261	7	10	0	49.66
2 multi	2	H						
8	1	05/21/2020	120	3262	7	10	0	53.8
2 multi	2	H						
8	1	05/22/2020	121	3263	7	10	0	49.49
2 multi	2	H						
8	1	05/23/2020	122	3264	7	10	0	48.06
2 multi	2	H						
8	1	05/24/2020	123	3265	7	10	0.03	48.44
2 multi	2	H						
8	1	05/25/2020	124	3266	7	10.2	0	49.47
2 multi	2	H						
8	1	05/26/2020	125	3267	7	9.9	0	49.65
2 multi	2	H						
8	1	05/27/2020	126	3268	7	10	0	49.62
2 multi	2	H						
8	1	05/28/2020	127	3269	7	10	0	54.16
2 multi	2	H						
8	1	05/29/2020	128	3270	7	10.3	0	51.81
2 multi	2	H						
8	1	05/30/2020	129	3271	7	10	0	47.38
2 multi	2	H						
8	1	05/31/2020	130	3272	7	10.3	0	49.8
2 multi	2	H						
8	1	06/01/2020	131	3273	7	10	0	47.43
2 multi	2	H						
8	1	06/02/2020	132	3274	7	10.2	0.01	49.55
2 multi	2	H						
8	1	06/03/2020	133	3275	7	10.2	0	50.72
2 multi	2	H						
8	1	06/04/2020	134	3276	7	10.4	0	51.27
2 multi	2	H						
8	1	06/05/2020	135	3277	7	10.2	0	51.64
2 multi	2	H						
8	1	06/06/2020	136	3278	7	10.3	0	49.16
2 multi	2	H						
8	1	06/07/2020	137	3279	7	10.2	0	47.22
2 multi	2	H						
8	1	06/08/2020	138	3280	7	9.9	0	45.94
2 multi	2	H						
8	1	06/09/2020	139	3281	7	10	0	49.04
2 multi	2	H						
8	1	06/10/2020	140	3282	7	10.4	0.15	41.24
2 multi	2	H						
8	1	06/11/2020	141	3283	7	10	0	43.44
2 multi	2	H						
8	1	06/12/2020	142	3284	7	10	0	49.53
2 multi	2	H						
8	1	06/13/2020	143	3285	7	10.2	0.53	49.86

2 multi	2	H						
8	1	06/14/2020	144	3286	7	10.3	0	49.92
2 multi	2	H						
8	1	06/15/2020	145	3287	7	10.3	0	46.22
2 multi	2	H						
8	1	06/16/2020	146	3288	7	10	0	46.19
2 multi	2	H						
8	1	06/17/2020	147	3289	7	10.3	0	48.22
2 multi	2	H						
8	1	06/18/2020	148	3290	7	10.4	0.09	42.72
2 multi	2	H						
8	1	06/19/2020	149	3291	7	10.4	0	46.2
2 multi	2	H						
8	1	06/20/2020	150	3292	7	10.3	0	44.71
2 multi	2	H						
8	1	06/21/2020	151	3293	7	10.3	0.08	49.3
2 multi	2	H						
8	1	06/22/2020	152	3294	7	10	0	46.96
2 multi	2	H						
8	1	06/23/2020	153	3295	7	10	0.5	45.56
2 multi	2	H						
8	1	06/24/2020	154	3296	7	10	0	45.85
2 multi	2	H						
8	1	06/25/2020	155	3297	7	10.2	0	44.7
2 multi	2	H						
8	1	06/26/2020	156	3298	7	10.2	0	44.78
2 multi	2	H						
8	1	06/27/2020	157	3299	7	10.2	0	44.12
2 multi	2	H						
8	1	06/28/2020	158	3300	7	10.2	0	44.99
2 multi	2	H						
8	1	06/29/2020	159	3301	7	10.2	0	43.49
2 multi	2	H						
8	1	06/30/2020	160	3302	7	10.2	0	47.32
2 multi	2	H						
8	1	07/01/2020	161	3303	7	10.2	0	42.33
2 multi	2	H						
8	1	07/02/2020	162	3304	7	10.4	0	42.18
2 multi	2	H						
8	1	07/03/2020	163	3305	7	10.3	0.14	42.28
2 multi	2	H						
8	1	07/04/2020	164	3306	7	10.3	0.2	50.36
2 multi	2	H						
8	1	07/05/2020	165	3307	7	10.4	0.04	41.66
2 multi	2	H						
8	1	07/06/2020	166	3308	7	10	0	38.75
2 multi	2	H						
8	1	07/07/2020	167	3309	7	10.3	0.18	44.59
2 multi	2	H						
8	1	07/08/2020	168	3310	7	10.3	0.01	45.58
2 multi	2	H						
8	1	07/09/2020	169	3311	7	10.2	0	48.84
2 multi	2	H						
8	1	07/10/2020	170	3312	7	10.2	0.02	46.16

2 multi	2	H						
8	1	07/11/2020	171	3313	7	10	0	50.19
2 multi	2	H						
8	1	07/12/2020	172	3314	7	10.3	0.12	47.43
2 multi	2	H						
8	1	07/13/2020	173	3315	7	10.2	0.01	47.98
2 multi	2	H						
8	1	07/14/2020	174	3316	7	9.9	0.02	49.19
2 multi	2	H						
8	1	07/15/2020	175	3317	7	10.2	0	42.32
2 multi	2	H						
8	1	07/16/2020	176	3318	7	10	0.66	47.03
2 multi	2	H						
8	1	07/17/2020	177	3319	7	9.9	0.03	50.82
2 multi	2	H						
8	1	07/18/2020	178	3320	7	9.9	0.01	44.95
2 multi	2	H						
8	1	07/19/2020	179	3321	7	10.3	0	45.34
2 multi	2	H						
8	1	07/20/2020	180	3322	7	10	0	47.51
2 multi	2	H						
8	1	07/21/2020	181	3323	7	10.2	0.01	46.72
2 multi	2	H						
8	1	07/22/2020	182	3324	7	10.2	0.01	45.29
2 multi	2	H						
8	1	07/23/2020	183	3325	7	10	0	44.11
2 multi	2	H						
8	1	07/24/2020	184	3326	7	10.2	0	40.49
2 multi	2	H						
8	1	07/25/2020	185	3327	7	10.2	0	45.03
2 multi	2	H						
8	1	07/26/2020	186	3328	7	10.3	0	42.88
2 multi	2	H						
8	1	07/27/2020	187	3329	7	10.2	0.47	47.05
2 multi	2	H						
8	1	07/28/2020	188	3330	7	10.4	0	41.13
2 multi	2	H						
8	1	07/29/2020	189	3331	7	10.4	0	42.4
2 multi	2	H						
8	1	07/30/2020	190	3332	7	10.3	0	41.87
2 multi	2	H						
8	1	07/31/2020	191	3333	7	10.4	0	43.14
2 multi	2	H						
8	1	08/01/2020	192	3334	7	10.4	0.05	41.88
2 multi	2	H						
8	1	08/02/2020	193	3335	7	10.2	0.06	45.43
2 multi	2	H						
8	1	08/03/2020	194	3336	7	10.2	0	39.58
2 multi	2	H						
8	1	08/04/2020	195	3337	7	10.3	0.12	44.05
2 multi	2	H						
8	1	08/05/2020	196	3338	7	10.3	0.01	43.25
2 multi	2	H						
8	1	08/06/2020	197	3339	7	10.4	0	45.58

2	multi	2	H						
8		1	08/07/2020	198	3340	7	10.2	0	41.94
2	multi	2	H						
8		1	08/08/2020	199	3341	7	10.2	0	39.5
2	multi	2	H						
8		1	08/09/2020	200	3342	7	10.2	0	43.9
2	multi	2	H						
8		1	08/10/2020	201	3343	7	10.3	0	42.79
2	multi	2	H						
8		1	08/11/2020	202	3344	7	10.2	0	39.44
2	multi	2	H						
8		1	08/12/2020	203	3345	7	10.3	0.01	41.1
2	multi	2	H						
8		1	08/13/2020	204	3346	7	10.3	0.02	40.42
2	multi	2	H						
8		1	08/14/2020	205	3347	7	10.4	0.17	43.4
2	multi	2	H						
8		1	08/15/2020	206	3348	7	10.3	0.07	37.32
2	multi	2	H						
8		1	08/16/2020	207	3349	7	10.3	0.3	39.87
2	multi	2	H						
8		1	08/17/2020	208	3350	7	10.3	0.02	41.68
2	multi	2	H						
8		1	08/18/2020	209	3351	7	10.6	0	40.84
2	multi	2	H						
8		1	08/19/2020	210	3352	7	10.7	0.04	37.56
2	multi	2	H						
8		1	08/20/2020	211	3353	7	10.3	0	39.11
2	multi	2	H						
8		1	08/21/2020	212	3354	7	10.6	0	41.49
2	multi	2	H						
8		1	08/22/2020	213	3355	7	10	0	38.08
2	multi	2	H						
8		1	08/23/2020	214	3356	7	10.3	0	40.45
2	multi	2	H						
8		1	08/24/2020	215	3357	7	10.3	0	39.12
2	multi	2	H						
8		1	08/25/2020	216	3358	7	10.3	0.03	39.77
2	multi	2	H						
8		1	08/26/2020	217	3359	7	10.2	0.08	39.23
2	multi	2	H						
8		1	08/27/2020	218	3360	7	10.4	0.03	43.7
2	multi	2	H						
8		1	08/28/2020	219	3361	7	10	0.12	43.19
2	multi	2	H						
8		1	08/29/2020	220	3362	7	10	0.05	41.74
2	multi	2	H						
8		1	08/30/2020	221	3363	7	10	0.6	42.55
2	multi	2	H						
8		1	08/31/2020	222	3364	7	9.9	0	38.23
2	multi	2	H						
8		1	09/01/2020	223	3365	7	9.9	0.09	39.45
2	multi	2	H						
8		1	09/02/2020	224	3366	7	10	0.23	43.65

2 multi	2	H						
8	1	09/03/2020	225	3367	7	10.2	0.05	41.44
2 multi	2	H						
8	1	09/04/2020	226	3368	7	10.3	0	40.69
2 multi	2	H						
8	1	09/05/2020	227	3369	7	9.9	0	38.15
2 multi	2	H						
8	1	09/06/2020	228	3370	7	10.2	0	36.09
2 multi	2	H						
8	1	09/07/2020	229	3371	7	10.4	0.03	38.12
2 multi	2	H						
8	1	09/08/2020	230	3372	7	10.3	0.01	38
2 multi	2	H						
8	1	09/09/2020	231	3373	7	10.3	0	34.36
2 multi	2	H						
8	1	09/10/2020	232	3374	7	10.6	0.05	38.05
2 multi	2	H						
8	1	09/11/2020	233	3375	7	10.4	0.09	32.22
2 multi	2	H						
8	1	09/12/2020	234	3376	7	10.4	0.04	25.91
2 multi	2	H						
8	1	09/13/2020	235	3377	7	11.1	0.05	31.76
2 multi	2	H						
8	1	09/14/2020	236	3378	7	10.9	0.1	31.27
2 multi	2	H						
8	1	09/15/2020	237	3379	7	10.7	0.01	30.59
2 multi	2	H						
8	1	09/16/2020	238	3380	7	10.9	0.05	32.59
2 multi	2	H						
8	1	09/17/2020	239	3381	7	10.9	0	25.3
2 multi	2	H						
8	1	09/18/2020	240	3382	7	10.7	0.1	29.92
2 multi	2	H						
8	1	09/19/2020	241	3383	7	10.9	0	34.61
2 multi	2	H						
8	1	09/20/2020	242	3384	7	11.1	0.01	32.92
2 multi	2	H						
8	1	09/21/2020	243	3385	7	10.9	0	32.79
2 multi	2	H						
8	1	09/22/2020	244	3386	7	10.6	0.06	34.76
2 multi	2	H						
8	1	09/23/2020	245	3387	7	10.7	0.03	34.78
2 multi	2	H						
8	1	09/24/2020	246	3388	7	10.4	0.03	31.97
2 multi	2	H						
8	1	09/25/2020	247	3389	7	10.6	0.01	34.99
2 multi	2	H						
8	1	09/26/2020	248	3390	7	10.4	0	36.96
2 multi	2	H						
8	1	09/27/2020	249	3391	7	10.4	0.01	33.48
2 multi	2	H						
8	1	09/28/2020	250	3392	7	10.2	0.04	35.79
2 multi	2	H						
8	1	09/29/2020	251	3393	7	10.4	0.05	32.64

2 multi	2	H						
8	1	09/30/2020	252	3394	7	10.9	0.54	32.98
2 multi	2	H						
8	1	10/01/2020	253	3395	7	10.7	0.32	35.58
2 multi	2	H						
8	1	10/02/2020	254	3396	7	10.7	0	33.7
2 multi	2	H						
8	1	10/03/2020	255	3397	7	10.9	0	33.46
2 multi	2	H						
8	1	10/04/2020	256	3398	7	10.9	0	32.74
2 multi	2	H						
8	1	10/05/2020	257	3399	7	10.6	0.01	36.28
2 multi	2	H						
8	1	10/06/2020	258	3400	7	10.6	0.03	32.92
2 multi	2	H						
8	1	10/07/2020	259	3401	7	10.4	0.02	28.99
2 multi	2	H						
8	1	10/08/2020	260	3402	7	10.7	0.04	35.46
2 multi	2	H						
8	1	10/09/2020	261	3403	7	10.7	0	30.11
2 multi	2	H						
8	1	10/10/2020	262	3404	7	10.3	0.04	31.39
2 multi	2	H						
8	1	10/11/2020	263	3405	7	10.3	0.03	33.4
2 multi	2	H						
8	1	10/12/2020	264	3406	7	10.2	0.03	34.01
2 multi	2	H						
8	1	10/13/2020	265	3407	7	10.6	0	29.7
2 multi	2	H						
8	1	10/14/2020	266	3408	7	10.9	0.04	33.74
2 multi	2	H						
8	1	10/15/2020	267	3409	7	10.7	0.05	30.55
2 multi	2	H						
8	1	10/16/2020	268	3410	7	10.7	0.05	33.02
2 multi	2	H						
8	1	10/17/2020	269	3411	7	10.6	0.06	33.72
2 multi	2	H						
8	1	10/18/2020	270	3412	7	10.7	0.03	33.49
2 multi	2	H						
8	1	10/19/2020	271	3413	7	10.3	0.05	33.56
2 multi	2	H						
8	1	10/20/2020	272	3414	7	11.1	0.07	32.02
2 multi	2	H						
9	1	05/11/2021	7	822	1	9.2	0.22	30.37
1 primi	1	H						
9	1	05/12/2021	8	823	1	10.2	0	33.19
1 primi	1	H						
9	1	05/13/2021	9	824	1	10.7	0	32.49
1 primi	1	H						
9	1	05/14/2021	10	825	1	11.1	0.02	31.63
1 primi	1	H						
9	1	05/15/2021	11	826	1	10.2	0	29.58
1 primi	1	H						
9	1	05/16/2021	12	827	1	10.2	0	31.46

1 primi	1	H						
9	1	05/17/2021	13	828	1	10.7	0	34.69
1 primi	1	H						
9	1	05/18/2021	14	829	1	10.3	0.01	40.46
1 primi	1	H						
9	1	05/19/2021	15	830	1	10.2	0.23	36.88
1 primi	1	H						
9	1	05/20/2021	16	831	1	10.3	0	36.72
1 primi	1	H						
9	1	05/21/2021	17	832	1	10.3	0	33.02
1 primi	1	H						
9	1	05/22/2021	18	833	1	10.9	0.04	37.19
1 primi	1	H						
9	1	05/23/2021	19	834	1	10.4	0.03	37.26
1 primi	1	H						
9	1	05/24/2021	20	835	1	11.3	0	33.58
1 primi	1	H						
9	1	05/25/2021	21	836	1	10.2	0.04	39.52
1 primi	1	H						
9	1	05/26/2021	22	837	1	9.6	0	19.05
1 primi	2	H						
10	1	03/13/2021	7	775	1	9.8	0.32	26.25
1 primi	1	H						
10	1	03/14/2021	8	776	1	10.4	0.11	30.5
1 primi	1	H						
10	1	03/15/2021	9	777	1	10.2	0.18	30.93
1 primi	1	H						
10	1	03/16/2021	10	778	1	0	0	32.41
1 primi	1	H						
10	1	03/17/2021	11	779	1	10.4	0	34.88
1 primi	1	H						
10	1	03/18/2021	12	780	1	10.3	0.12	32.13
1 primi	1	H						
10	1	03/19/2021	13	781	1	10	0	33.41
1 primi	1	H						
10	1	03/20/2021	14	782	1	10	0.32	31.79
1 primi	1	H						
10	1	03/21/2021	15	783	1	10.3	0.07	34.23
1 primi	1	H						
10	1	03/22/2021	16	784	1	10.3	0	36.28
1 primi	1	H						
10	1	03/23/2021	17	785	1	10.3	0.1	34.42
1 primi	1	H						
10	1	03/24/2021	18	786	1	10.2	0.11	34.98
1 primi	1	H						
10	1	03/25/2021	19	787	1	10	0	37.05
1 primi	1	H						
10	1	03/26/2021	20	788	1	10	0.1	38.25
1 primi	1	H						
10	1	03/27/2021	21	789	1	10.4	0	40.85
1 primi	1	H						
10	1	03/28/2021	22	790	1	10.3	0.04	38.49
1 primi	2	H						
10	1	03/29/2021	23	791	1	10	0	38.42

1 primi 2		H						
10	1	03/30/2021	24	792	1	10.3	0.1	38.3
1 primi 2		H						
10	1	03/31/2021	25	793	1	9.9	0.03	41.2
1 primi 2		H						
10	1	04/01/2021	26	794	1	10.2	0	40.01
1 primi 2		H						
10	1	04/02/2021	27	795	1	9.8	0.05	39.76
1 primi 2		H						
10	1	04/03/2021	28	796	1	9.9	0	40.17
1 primi 2		H						
10	1	04/04/2021	29	797	1	9.8	0.02	39.7
1 primi 2		H						
10	1	04/05/2021	30	798	1	9.5	0.27	40.95
1 primi 2		H						
10	1	04/06/2021	31	799	1	9.6	0.28	40.55
1 primi 2		H						
10	1	04/07/2021	32	800	1	9.9	0	40.67
1 primi 2		H						
10	1	04/08/2021	33	801	1	9.8	0	36.98
1 primi 2		H						
10	1	04/09/2021	34	802	1	9.8	0.23	37.2
1 primi 2		H						
10	1	04/10/2021	35	803	1	10	0.02	40.64
1 primi 2		H						
10	1	04/11/2021	36	804	1	10.3	0.33	41.35
1 primi 2		H						
10	1	04/12/2021	37	805	1	10.2	0	42.73
1 primi 2		H						
10	1	04/13/2021	38	806	1	10.2	0	41.86
1 primi 2		H						
10	1	04/14/2021	39	807	1	9.8	0	39.45
1 primi 2		H						
10	1	04/15/2021	40	808	1	10	0	40.42
1 primi 2		H						
10	1	04/16/2021	41	809	1	10	0	40.27
1 primi 2		H						
10	1	04/17/2021	42	810	1	10	0.03	37.93
1 primi 2		H						
10	1	04/18/2021	43	811	1	9.9	0.04	39.95
1 primi 2		H						
10	1	04/19/2021	44	812	1	10.2	0.24	41.56
1 primi 2		H						
10	1	04/20/2021	45	813	1	10.4	0.37	40.47
1 primi 2		H						
10	1	04/21/2021	46	814	1	9.8	0.32	38.3
1 primi 2		H						
10	1	04/22/2021	47	815	1	10	0.23	41.68
1 primi 2		H						
10	1	04/23/2021	48	816	1	9.9	0.34	41.02
1 primi 2		H						
10	1	04/24/2021	49	817	1	9.8	0.03	37.49
1 primi 2		H						
10	1	04/25/2021	50	818	1	10.2	0.34	41.91

1 primi 2		H						
10	1	04/26/2021	51	819	1	10	0.03	42.75
1 primi 2		H						
10	1	04/27/2021	52	820	1	10	0	42.69
1 primi 2		H						
10	1	04/28/2021	53	821	1	10.2	0.08	41.21
1 primi 2		H						
10	1	04/29/2021	54	822	1	10.3	0.27	38.46
1 primi 2		H						
10	1	04/30/2021	55	823	1	10.3	0	42.69
1 primi 2		H						
10	1	05/01/2021	56	824	1	10	0.08	38.16
1 primi 2		H						
10	1	05/02/2021	57	825	1	10.2	0.12	40.45
1 primi 2		H						
10	1	05/03/2021	58	826	1	10.4	0.12	37.82
1 primi 2		H						
10	1	05/04/2021	59	827	1	11.1	0.01	40.07
1 primi 2		H						
10	1	05/05/2021	60	828	1	10.4	0.04	39.67
1 primi 2		H						
10	1	05/06/2021	61	829	1	10.6	0.1	38.44
1 primi 2		H						
10	1	05/07/2021	62	830	1	10.6	0.26	39.52
1 primi 2		H						
10	1	05/08/2021	63	831	1	10.4	0.34	37.48
1 primi 2		H						
10	1	05/09/2021	64	832	1	10.4	0.02	39.47
1 primi 2		H						
10	1	05/10/2021	65	833	1	10.6	0.23	38.61
1 primi 2		H						
10	1	05/11/2021	66	834	1	10.4	0.46	40.7
1 primi 2		H						
10	1	05/12/2021	67	835	1	10.4	0.13	41.69
1 primi 2		H						
10	1	05/13/2021	68	836	1	10	0.1	39.31
1 primi 2		H						
10	1	05/14/2021	69	837	1	10.4	0	39.85
1 primi 2		H						
10	1	05/15/2021	70	838	1	11.1	0.03	39.47
1 primi 2		H						
10	1	05/16/2021	71	839	1	10.6	0.1	39.38
1 primi 2		H						
10	1	05/17/2021	72	840	1	10.4	0.03	39.92
1 primi 2		H						
10	1	05/18/2021	73	841	1	10.4	0.23	40.6
1 primi 2		H						
10	1	05/19/2021	74	842	1	10.4	0.1	39.72
1 primi 2		H						
10	1	05/20/2021	75	843	1	10.2	0.1	39.14
1 primi 2		H						
10	1	05/21/2021	76	844	1	9.9	0.03	38.22
1 primi 2		H						
10	1	05/22/2021	77	845	1	10	0.32	40.5

1 primi	2	H						
10	1	05/23/2021	78	846	1	10	0.31	41.75
1 primi	2	H						
10	1	05/24/2021	79	847	1	10.3	0.54	42.07
1 primi	2	H						
10	1	05/25/2021	80	848	1	10.3	0.1	39.67
1 primi	2	H						
10	1	05/26/2021	81	849	1	10	0.12	18.79
1 primi	2	H						
13	1	08/15/2013	5	702	1	9.9	0	13.77
1 primi	1	H						
13	1	08/16/2013	6	703	1	9.5	0	15.55
1 primi	1	H						
13	1	08/17/2013	7	704	1	9.6	0	15.11
1 primi	1	H						
13	1	08/18/2013	8	705	1	9.3	0	17.08
1 primi	1	H						
13	1	08/19/2013	9	706	1	9.9	0	17.68
1 primi	1	H						
13	1	08/20/2013	10	707	1	10	0	20.15
1 primi	1	H						
13	1	08/21/2013	11	708	1	9.6	0	19.17
1 primi	1	H						
13	1	08/22/2013	12	709	1	9.5	0	21.26
1 primi	1	H						
13	1	08/23/2013	13	710	1	9.5	0	20.72
1 primi	1	H						
13	1	08/24/2013	14	711	1	9.3	0	20.66
1 primi	1	H						
13	1	08/25/2013	15	712	1	9.5	0	20.65
1 primi	1	H						
13	1	08/26/2013	16	713	1	9.3	0	22.05
1 primi	1	H						
13	1	08/27/2013	17	714	1	9.5	0	19.85
1 primi	1	H						
13	1	08/28/2013	18	715	1	9.5	0	23.93
1 primi	1	H						
13	1	08/29/2013	19	716	1	9.1	0	23.41
1 primi	1	H						
13	1	08/30/2013	20	717	1	9.1	0	20.41
1 primi	1	H						
13	1	08/31/2013	21	718	1	9.3	0	20.61
1 primi	1	H						
13	1	09/01/2013	22	719	1	9.2	0	21.21
1 primi	2	H						
13	1	09/02/2013	23	720	1	9.2	0	20.26
1 primi	2	H						
13	1	09/03/2013	24	721	1	9.5	0	20.8
1 primi	2	H						
13	1	09/04/2013	25	722	1	8.9	0	22.51
1 primi	2	H						
13	1	09/05/2013	26	723	1	8.7	0	23.01
1 primi	2	H						
13	1	09/06/2013	27	724	1	9.3	0	33.74

1 primi 2		H						
13	1	09/07/2013	28	725	1	8.7	0	20.63
1 primi 2		H						
13	1	09/08/2013	29	726	1	8.9	0	22.11
1 primi 2		H						
13	1	09/09/2013	30	727	1	8.8	0	22.47
1 primi 2		H						
13	1	09/10/2013	31	728	1	8.8	0	23.27
1 primi 2		H						
13	1	09/11/2013	32	729	1	8.8	0	22.77
1 primi 2		H						
13	1	09/12/2013	33	730	1	8.5	0	24.35
1 primi 2		H						
13	1	09/13/2013	34	731	1	8.3	0	22.73
1 primi 2		H						
13	1	09/14/2013	35	732	1	10.4	0	35.66
1 primi 2		H						
13	1	09/15/2013	36	733	1	8.6	0	22.77
1 primi 2		H						
13	1	09/16/2013	37	734	1	8.9	0	23.84
1 primi 2		H						
13	1	09/17/2013	38	735	1	8.7	0	23.19
1 primi 2		H						
13	1	09/18/2013	39	736	1	8.6	0	21.93
1 primi 2		H						
13	1	09/19/2013	40	737	1	8.3	0	23.18
1 primi 2		H						
13	1	09/20/2013	41	738	1	8.1	0	21.6
1 primi 2		H						
13	1	09/21/2013	42	739	1	8.5	0	23.62
1 primi 2		H						
13	1	09/22/2013	43	740	1	8.6	0	22.62
1 primi 2		H						
13	1	09/23/2013	44	741	1	8.7	0	21.87
1 primi 2		H						
13	1	09/24/2013	45	742	1	8.6	0	22.65
1 primi 2		H						
13	1	09/25/2013	46	743	1	8.9	0	23.23
1 primi 2		H						
13	1	09/26/2013	47	744	1	8.8	0	23.49
1 primi 2		H						
13	1	09/27/2013	48	745	1	8.7	0	23.05
1 primi 2		H						
13	1	09/28/2013	49	746	1	8.6	0	23.34
1 primi 2		H						
13	1	09/29/2013	50	747	1	8.6	0	22.31
1 primi 2		H						
13	1	09/30/2013	51	748	1	8.5	0	23.15
1 primi 2		H						
13	1	10/01/2013	52	749	1	8.8	0	23
1 primi 2		H						
13	1	10/02/2013	53	750	1	8.4	0	22.08
1 primi 2		H						
13	1	10/03/2013	54	751	1	8.5	0	23.32

1 primi	2	H						
13	1	10/04/2013	55	752	1	8.5	0	23.94
1 primi	2	H						
13	1	10/05/2013	56	753	1	8.8	0	24.55
1 primi	2	H						
13	1	10/06/2013	57	754	1	8.6	0	23.63
1 primi	2	H						
13	1	10/07/2013	58	755	1	8.4	0	21.11
1 primi	2	H						
13	1	10/08/2013	59	756	1	8.7	0	21.13
1 primi	2	H						
13	1	10/09/2013	60	757	1	8.7	0	23.13
1 primi	2	H						
13	1	10/10/2013	61	758	1	8.9	0	19.99
1 primi	2	H						
13	1	10/11/2013	62	759	1	8.8	0	22.44
1 primi	2	H						
13	1	10/12/2013	63	760	1	8.5	0	20.8
1 primi	2	H						
13	1	10/13/2013	64	761	1	8.7	0	20.92
1 primi	2	H						
13	1	10/14/2013	65	762	1	8.9	0	23.51
1 primi	2	H						
13	1	10/15/2013	66	763	1	10.6	0	32.16
1 primi	2	H						
13	1	10/16/2013	67	764	1	9.1	0	23.83
1 primi	2	H						
13	1	10/17/2013	68	765	1	8.8	0	24.23
1 primi	2	H						
13	1	10/18/2013	69	766	1	8.8	0	24.54
1 primi	2	H						
13	1	10/19/2013	70	767	1	9.1	0	25.9
1 primi	2	H						
13	1	10/20/2013	71	768	1	8.9	0	27.35
1 primi	2	H						
13	1	10/21/2013	72	769	1	8.8	0	25.73
1 primi	2	H						
13	1	10/22/2013	73	770	1	8.8	0	25.5
1 primi	2	H						
13	1	10/23/2013	74	771	1	9.2	0	26.59
1 primi	2	H						
13	1	10/24/2013	75	772	1	9.1	0	24.08
1 primi	2	H						
13	1	10/25/2013	76	773	1	10	0	40.51
1 primi	2	H						
13	1	10/26/2013	77	774	1	8.7	0	24.45
1 primi	2	H						
13	1	10/27/2013	78	775	1	8.8	0	26.29
1 primi	2	H						
13	1	10/28/2013	79	776	1	8.8	0	24.51
1 primi	2	H						
13	1	10/29/2013	80	777	1	8.7	0	25.63
1 primi	2	H						
13	1	10/30/2013	81	778	1	8.8	0	26.31

1 primi	2	H						
13	1	10/31/2013	82	779	1	9.2	0	28.07
1 primi	2	H						
13	1	11/01/2013	83	780	1	9.1	0	26.32
1 primi	2	H						
13	1	11/02/2013	84	781	1	8.6	0	25.58
1 primi	2	H						
13	1	11/03/2013	85	782	1	9.8	0	42.91
1 primi	2	H						
13	1	11/04/2013	86	783	1	8.8	0	26.75
1 primi	2	H						
13	1	11/05/2013	87	784	1	8.9	0	26.61
1 primi	2	H						
13	1	11/06/2013	88	785	1	8.9	0	26.16
1 primi	2	H						
13	1	11/07/2013	89	786	1	9.2	0	25.48
1 primi	2	H						
13	1	11/08/2013	90	787	1	8.7	0	24.71
1 primi	2	H						
13	1	11/09/2013	91	788	1	8.9	0	25.71
1 primi	2	H						
13	1	11/10/2013	92	789	1	9.1	0	26.28
1 primi	2	H						
13	1	07/22/2014	4	1043	2	9.5	0.09	28.17
2 multi	1	H						
13	1	07/23/2014	5	1044	2	9.1	0.04	26.67
2 multi	1	H						
13	1	07/24/2014	6	1045	2	8.9	0	22.07
2 multi	1	H						
13	1	07/25/2014	7	1046	2	9.5	0	32.9
2 multi	1	H						
13	1	07/26/2014	8	1047	2	9.5	0	33.24
2 multi	1	H						
13	1	07/27/2014	9	1048	2	9.1	0	34.49
2 multi	1	H						
13	1	07/28/2014	10	1049	2	9.3	0	33.85
2 multi	1	H						
13	1	07/29/2014	11	1050	2	9.5	0.01	36.07
2 multi	1	H						
13	1	07/30/2014	12	1051	2	9.5	0	38.22
2 multi	1	H						
13	1	07/31/2014	13	1052	2	9.2	0	36.5
2 multi	1	H						
13	1	08/01/2014	14	1053	2	9.5	0	38.5
2 multi	1	H						
13	1	08/02/2014	15	1054	2	9.6	0	39.28
2 multi	1	H						
13	1	08/03/2014	16	1055	2	9.8	0	39.51
2 multi	1	H						
13	1	08/04/2014	17	1056	2	9.9	0	42.18
2 multi	1	H						
13	1	08/05/2014	18	1057	2	9.8	0	40.98
2 multi	1	H						
13	1	08/06/2014	19	1058	2	9.6	0	39.62

2 multi	1	H						
13	1	08/07/2014	20	1059	2	9.8	0	43.3
2 multi	1	H						
13	1	08/08/2014	21	1060	2	9.9	0	39.43
2 multi	1	H						
13	1	08/09/2014	22	1061	2	9.5	0	43.28
2 multi	2	H						
13	1	08/10/2014	23	1062	2	9.5	0	42.81
2 multi	2	H						
13	1	08/11/2014	24	1063	2	9.3	0	43.12
2 multi	2	H						
13	1	08/12/2014	25	1064	2	9.5	0	40.58
2 multi	2	H						
13	1	08/13/2014	26	1065	2	9.5	0	44.16
2 multi	2	H						
13	1	08/14/2014	27	1066	2	9.1	0.02	40.96
2 multi	2	H						
13	1	08/15/2014	28	1067	2	8.9	0	39.28
2 multi	2	H						
13	1	08/16/2014	29	1068	2	9.3	0	40.38
2 multi	2	H						
13	1	08/17/2014	30	1069	2	9.2	0	41.76
2 multi	2	H						
13	1	08/18/2014	31	1070	2	9.3	0	40.76
2 multi	2	H						
13	1	08/19/2014	32	1071	2	9.3	0.01	58.33
2 multi	2	H						
13	1	08/20/2014	33	1072	2	9.2	0.03	37.25
2 multi	2	H						
13	1	08/21/2014	34	1073	2	9.1	0	41.62
2 multi	2	H						
13	1	08/22/2014	35	1074	2	9.3	0	42.76
2 multi	2	H						
13	1	08/23/2014	36	1075	2	9.1	0	36.82
2 multi	2	H						
13	1	08/24/2014	37	1076	2	9.2	0	39.55
2 multi	2	H						
13	1	08/25/2014	38	1077	2	9.1	0	40.99
2 multi	2	H						
13	1	08/26/2014	39	1078	2	9.3	0	43.75
2 multi	2	H						
13	1	08/27/2014	40	1079	2	9.3	0	43.39
2 multi	2	H						
13	1	08/28/2014	41	1080	2	9.3	0	40.32
2 multi	2	H						
13	1	08/29/2014	42	1081	2	9.1	0.21	41.08
2 multi	2	H						
13	1	08/30/2014	43	1082	2	9.2	0	43.56
2 multi	2	H						
13	1	08/31/2014	44	1083	2	9.2	0.12	42.3
2 multi	2	H						
13	1	09/01/2014	45	1084	2	9.2	0	41.89
2 multi	2	H						
13	1	09/02/2014	46	1085	2	8.9	0	39.95

2 multi	2	H						
13	1	09/03/2014	47	1086	2	9.3	0	40.24
2 multi	2	H						
13	1	09/04/2014	48	1087	2	9.1	0	41.63
2 multi	2	H						
13	1	09/05/2014	49	1088	2	9.1	0	40.52
2 multi	2	H						
13	1	09/06/2014	50	1089	2	8.8	0	40.03
2 multi	2	H						
13	1	09/07/2014	51	1090	2	8.7	0	38.95
2 multi	2	H						
13	1	09/08/2014	52	1091	2	8.8	0	37.49
2 multi	2	H						
13	1	09/09/2014	53	1092	2	8.6	0	38.36
2 multi	2	H						
13	1	09/10/2014	54	1093	2	8.7	0	34.83
2 multi	2	H						
13	1	09/11/2014	55	1094	2	9.1	0	36.54
2 multi	2	H						
13	1	09/12/2014	56	1095	2	9.3	0.32	35.9
2 multi	2	H						
13	1	09/13/2014	57	1096	2	9.3	0	37.63
2 multi	2	H						
13	1	09/14/2014	58	1097	2	9.1	0.05	37.66
2 multi	2	H						
13	1	09/15/2014	59	1098	2	9.3	0	35.62
2 multi	2	H						
13	1	09/16/2014	60	1099	2	9.1	0.11	37.39
2 multi	2	H						
13	1	09/17/2014	61	1100	2	8.8	0	35.84
2 multi	2	H						
13	1	09/18/2014	62	1101	2	8.9	0	35.4
2 multi	2	H						
13	1	09/19/2014	63	1102	2	8.9	0	33.26
2 multi	2	H						
13	1	09/20/2014	64	1103	2	8.9	0	34.32
2 multi	2	H						
13	1	09/21/2014	65	1104	2	8.8	0	35.38
2 multi	2	H						
13	1	09/22/2014	66	1105	2	8.8	0	35.02
2 multi	2	H						
13	1	09/23/2014	67	1106	2	8.8	0	34.67
2 multi	2	H						
13	1	09/24/2014	68	1107	2	8.8	0	37.08
2 multi	2	H						
13	1	09/25/2014	69	1108	2	8.9	0	36.69
2 multi	2	H						
13	1	09/26/2014	70	1109	2	9.1	0	35.46
2 multi	2	H						
13	1	09/27/2014	71	1110	2	9.5	0	37.64
2 multi	2	H						
13	1	09/28/2014	72	1111	2	9.3	0	38.72
2 multi	2	H						
13	1	09/29/2014	73	1112	2	9.2	0.01	36.98

2 multi	2	H						
13	1	09/30/2014	74	1113	2	9.2	0	37.18
2 multi	2	H						
13	1	10/01/2014	75	1114	2	9.3	0	36.52
2 multi	2	H						
13	1	10/02/2014	76	1115	2	9.1	0	38.11
2 multi	2	H						
13	1	10/03/2014	77	1116	2	9.2	0	38
2 multi	2	H						
13	1	10/04/2014	78	1117	2	8.9	0	34.04
2 multi	2	H						
13	1	10/05/2014	79	1118	2	8.8	0.26	35.48
2 multi	2	H						
13	1	10/06/2014	80	1119	2	9.1	0	38.22
2 multi	2	H						
13	1	10/07/2014	81	1120	2	8.9	0	37.9
2 multi	2	H						
13	1	10/08/2014	82	1121	2	9.5	0	37.24
2 multi	2	H						
13	1	10/09/2014	83	1122	2	9.1	0.05	37.12
2 multi	2	H						
13	1	07/01/2015	6	1387	3	9.2	0	28.93
2 multi	1	H						
13	1	07/02/2015	7	1388	3	9.1	0.1	29.17
2 multi	1	H						
13	1	07/03/2015	8	1389	3	10	0	36.8
2 multi	1	H						
13	1	07/04/2015	9	1390	3	9.3	0	39.43
2 multi	1	H						
13	1	07/05/2015	10	1391	3	9.9	0	37.55
2 multi	1	H						
13	1	07/06/2015	11	1392	3	9.6	0	38.32
2 multi	1	H						
13	1	07/07/2015	12	1393	3	9.8	0	39.37
2 multi	1	H						
13	1	07/08/2015	13	1394	3	9.8	0	39.97
2 multi	1	H						
13	1	07/09/2015	14	1395	3	9.6	0	38.66
2 multi	1	H						
13	1	07/10/2015	15	1396	3	9.8	0	41.01
2 multi	1	H						
13	1	07/11/2015	16	1397	3	9.8	0	41.42
2 multi	1	H						
13	1	07/12/2015	17	1398	3	9.8	0	39.42
2 multi	1	H						
13	1	07/13/2015	18	1399	3	9.9	0	44.66
2 multi	1	H						
13	1	07/14/2015	19	1400	3	9.6	0	38.6
2 multi	1	H						
13	1	07/15/2015	20	1401	3	9.5	0	43.15
2 multi	1	H						
13	1	07/16/2015	21	1402	3	9.5	0	44.9
2 multi	1	H						
13	1	07/17/2015	22	1403	3	9.6	0	46.79

2 multi	2	H						
13	1	07/18/2015	23	1404	3	9.2	0	42.92
2 multi	2	H						
13	1	07/19/2015	24	1405	3	9.1	0	40.83
2 multi	2	H						
13	1	07/20/2015	25	1406	3	9.2	0	36.16
2 multi	2	H						
13	1	07/21/2015	26	1407	3	9.2	0	32.28
2 multi	2	H						
13	1	07/22/2015	27	1408	3	9.1	0	31.26
2 multi	2	H						
13	1	07/23/2015	28	1409	3	9.3	0.09	32.26
2 multi	2	H						
13	1	07/24/2015	29	1410	3	9.6	0	31.72
2 multi	2	H						
13	1	07/25/2015	30	1411	3	9.5	0	37.75
2 multi	2	H						
13	1	07/26/2015	31	1412	3	9.1	0.15	34.92
2 multi	2	H						
13	1	07/27/2015	32	1413	3	8.9	0	37.99
2 multi	2	H						
13	1	07/28/2015	33	1414	3	9.6	0	38.93
2 multi	2	H						
13	1	07/29/2015	34	1415	3	9.8	0.09	44.67
2 multi	2	H						
13	1	07/30/2015	35	1416	3	9.8	0	46.44
2 multi	2	H						
13	1	07/31/2015	36	1417	3	9.6	0.11	44.57
2 multi	2	H						
13	1	08/01/2015	37	1418	3	9.8	0	48.38
2 multi	2	H						
13	1	08/02/2015	38	1419	3	9.6	0	47.48
2 multi	2	H						
13	1	08/03/2015	39	1420	3	9.3	0.01	43.99
2 multi	2	H						
13	1	08/04/2015	40	1421	3	9.6	0	48.54
2 multi	2	H						
13	1	08/05/2015	41	1422	3	9.6	0	42.79
2 multi	2	H						
13	1	08/06/2015	42	1423	3	9.6	0	46.87
2 multi	2	H						
13	1	08/07/2015	43	1424	3	9.9	0.49	40.96
2 multi	2	H						
13	1	08/08/2015	44	1425	3	9.6	0	40.22
2 multi	2	H						
13	1	08/09/2015	45	1426	3	9.6	0	43.29
2 multi	2	H						
13	1	08/10/2015	46	1427	3	9.3	0	41.17
2 multi	2	H						
13	1	08/11/2015	47	1428	3	9.5	0	39.74
2 multi	2	H						
13	1	08/12/2015	48	1429	3	9.6	0	39.82
2 multi	2	H						
13	1	08/13/2015	49	1430	3	9.6	0	41.35

2 multi	2	H						
13	1	08/14/2015	50	1431	3	10.2	0	47.2
2 multi	2	H						
13	1	08/15/2015	51	1432	3	9.8	0.09	40.02
2 multi	2	H						
13	1	08/16/2015	52	1433	3	10	0	63.38
2 multi	2	H						
13	1	08/17/2015	53	1434	3	10	0	43.19
2 multi	2	H						
13	1	08/18/2015	54	1435	3	9.8	0	42.96
2 multi	2	H						
13	1	08/19/2015	55	1436	3	9.6	0	43.94
2 multi	2	H						
13	1	08/20/2015	56	1437	3	9.6	0	46.26
2 multi	2	H						
13	1	08/21/2015	57	1438	3	9.6	0	40.8
2 multi	2	H						
13	1	08/22/2015	58	1439	3	9.8	0	41.64
2 multi	2	H						
13	1	08/23/2015	59	1440	3	9.8	0	41.63
2 multi	2	H						
13	1	08/24/2015	60	1441	3	9.8	0	42.55
2 multi	2	H						
13	1	08/25/2015	61	1442	3	10.2	0	44.87
2 multi	2	H						
13	1	08/26/2015	62	1443	3	10	0	45.23
2 multi	2	H						
13	1	08/27/2015	63	1444	3	10	0	45.4
2 multi	2	H						
13	1	08/28/2015	64	1445	3	10.2	0	46.06
2 multi	2	H						
13	1	08/29/2015	65	1446	3	10	0.06	42.82
2 multi	2	H						
13	1	08/30/2015	66	1447	3	10.2	0	46.03
2 multi	2	H						
13	1	08/31/2015	67	1448	3	10	0.01	46.09
2 multi	2	H						
13	1	09/01/2015	68	1449	3	10	0	43.29
2 multi	2	H						
13	1	09/02/2015	69	1450	3	9.6	0.07	37.78
2 multi	2	H						
13	1	09/03/2015	70	1451	3	9.6	0.45	40.13
2 multi	2	H						
13	1	09/04/2015	71	1452	3	9.5	0	41.52
2 multi	2	H						
13	1	09/05/2015	72	1453	3	9.5	0.03	41.23
2 multi	2	H						
13	1	09/06/2015	73	1454	3	9.6	0	46.07
2 multi	2	H						
13	1	09/07/2015	74	1455	3	9.6	0	46.85
2 multi	2	H						
13	1	09/08/2015	75	1456	3	9.3	0.15	42.6
2 multi	2	H						
13	1	09/09/2015	76	1457	3	9.5	0	44.27

2 multi	2	H						
13	1	09/10/2015	77	1458	3	9.5	0	39.38
2 multi	2	H						
13	1	09/11/2015	78	1459	3	9.6	0.09	43.48
2 multi	2	H						
13	1	09/12/2015	79	1460	3	9.8	0	45.25
2 multi	2	H						
13	1	09/13/2015	80	1461	3	9.6	0.2	48.14
2 multi	2	H						
13	1	09/14/2015	81	1462	3	9.8	0	47.42
2 multi	2	H						
13	1	09/15/2015	82	1463	3	9.6	0	44.59
2 multi	2	H						
13	1	09/16/2015	83	1464	3	9.8	0	45.56
2 multi	2	H						
13	1	09/17/2015	84	1465	3	9.5	0.03	51.29
2 multi	2	H						
13	1	09/18/2015	85	1466	3	9.5	0	47.55
2 multi	2	H						
13	1	09/19/2015	86	1467	3	9.5	0	42
2 multi	2	H						
13	1	09/20/2015	87	1468	3	9.1	0.01	41.84
2 multi	2	H						
13	1	09/21/2015	88	1469	3	9.2	0	42.5
2 multi	2	H						
13	1	09/22/2015	89	1470	3	9.6	0	46.77
2 multi	2	H						
13	1	09/23/2015	90	1471	3	9.3	0	40.3
2 multi	2	H						
13	1	09/24/2015	91	1472	3	9.3	0	41
2 multi	2	H						
13	1	09/25/2015	92	1473	3	9.5	0	37.93
2 multi	2	H						
13	1	09/26/2015	93	1474	3	9.6	0	47.35
2 multi	2	H						
13	1	09/27/2015	94	1475	3	9.6	0	41.76
2 multi	2	H						
13	1	09/28/2015	95	1476	3	9.6	0	38.19
2 multi	2	H						
13	1	09/29/2015	96	1477	3	9.8	0	43.32
2 multi	2	H						
13	1	09/30/2015	97	1478	3	9.6	0	46.73
2 multi	2	H						
13	1	10/01/2015	98	1479	3	9.5	0	42.24
2 multi	2	H						
13	1	10/02/2015	99	1480	3	9.6	0	43.69
2 multi	2	H						
13	1	10/03/2015	100	1481	3	9.6	0	43.96
2 multi	2	H						
13	1	10/04/2015	101	1482	3	9.5	0	42.13
2 multi	2	H						
13	1	10/05/2015	102	1483	3	9.5	0	43.1
2 multi	2	H						
13	1	10/06/2015	103	1484	3	9.5	0	43.12

2 multi 2		H						
13	1	10/07/2015	104	1485	3	9.8	0.12	46.1
2 multi 2		H						
13	1	10/08/2015	105	1486	3	9.3	0	40.28
2 multi 2		H						
13	1	10/09/2015	106	1487	3	9.5	0.15	44.51
2 multi 2		H						
13	1	10/10/2015	107	1488	3	9.6	0	44.88
2 multi 2		H						
13	1	10/11/2015	108	1489	3	9.8	0.27	49.6
2 multi 2		H						
13	1	10/12/2015	109	1490	3	9.6	0	37.63
2 multi 2		H						
13	1	10/13/2015	110	1491	3	9.3	0	41.42
2 multi 2		H						
13	1	10/14/2015	111	1492	3	9.6	0.07	40.29
2 multi 2		H						
13	1	10/15/2015	112	1493	3	9.8	0	43.97
2 multi 2		H						
13	1	10/16/2015	113	1494	3	9.3	0.01	40.71
2 multi 2		H						
13	1	10/17/2015	114	1495	3	9.2	0	43.44
2 multi 2		H						
13	1	10/18/2015	115	1496	3	9.2	0.02	40.62
2 multi 2		H						
13	1	10/19/2015	116	1497	3	9.3	0	43.44
2 multi 2		H						
13	1	10/20/2015	117	1498	3	9.3	0	41.62
2 multi 2		H						
13	1	10/21/2015	118	1499	3	9.2	0	38.91
2 multi 2		H						
13	1	10/22/2015	119	1500	3	9.5	0	40.65
2 multi 2		H						
13	1	10/23/2015	120	1501	3	9.3	0	38.88
2 multi 2		H						
13	1	10/24/2015	121	1502	3	9.3	0	39.6
2 multi 2		H						
13	1	10/25/2015	122	1503	3	9.5	0.07	37.69
2 multi 2		H						
13	1	10/26/2015	123	1504	3	9.6	0	40.89
2 multi 2		H						
13	1	10/27/2015	124	1505	3	9.8	0.12	40.56
2 multi 2		H						
13	1	10/28/2015	125	1506	3	9.8	0	40.79
2 multi 2		H						
13	1	10/29/2015	126	1507	3	10	0.36	41.39
2 multi 2		H						
13	1	10/30/2015	127	1508	3	9.8	0	42.36
2 multi 2		H						
13	1	10/31/2015	128	1509	3	9.8	0	42.32
2 multi 2		H						
13	1	11/01/2015	129	1510	3	9.5	0	42.86
2 multi 2		H						
13	1	11/02/2015	130	1511	3	9.2	0	41.11

2 multi 2		H						
13	1	11/03/2015	131	1512	3	9.5	0	42.95
2 multi 2		H						
13	1	11/04/2015	132	1513	3	9.5	0	41.94
2 multi 2		H						
13	1	11/05/2015	133	1514	3	9.3	0	42.48
2 multi 2		H						
13	1	11/06/2015	134	1515	3	9.8	0	39.57
2 multi 2		H						
13	1	11/07/2015	135	1516	3	9.1	0	36.32
2 multi 2		H						
13	1	11/08/2015	136	1517	3	9.8	0	37.07
2 multi 2		H						
13	1	11/09/2015	137	1518	3	9.5	0	37.94
2 multi 2		H						
13	1	11/10/2015	138	1519	3	9.5	0	36.19
2 multi 2		H						
13	1	11/11/2015	139	1520	3	9.5	0	36.7
2 multi 2		H						
13	1	11/12/2015	140	1521	3	9.3	0	32.71
2 multi 2		H						
13	1	11/13/2015	141	1522	3	9.5	0	31.94
2 multi 2		H						
13	1	11/14/2015	142	1523	3	9.5	0	35.83
2 multi 2		H						
13	1	11/15/2015	143	1524	3	9.5	0	35.42
2 multi 2		H						
13	1	11/16/2015	144	1525	3	9.5	0	36.6
2 multi 2		H						
13	1	11/17/2015	145	1526	3	9.3	0.02	37.24
2 multi 2		H						
13	1	11/18/2015	146	1527	3	9.6	0	40.72
2 multi 2		H						
13	1	11/19/2015	147	1528	3	9.6	0	38.24
2 multi 2		H						
13	1	11/20/2015	148	1529	3	9.6	0.37	40.07
2 multi 2		H						
13	1	11/21/2015	149	1530	3	9.6	0	41.12
2 multi 2		H						
13	1	11/22/2015	150	1531	3	9.8	0	41.41
2 multi 2		H						
13	1	11/23/2015	151	1532	3	9.9	0	39.72
2 multi 2		H						
13	1	11/24/2015	152	1533	3	9.3	0	20.17
2 multi 2		H						
13	1	11/25/2015	153	1534	3	10	0	41.54
2 multi 2		H						
13	1	11/26/2015	154	1535	3	9.6	0	41.06
2 multi 2		H						
13	1	11/27/2015	155	1536	3	9.6	0	36.34
2 multi 2		H						
13	1	11/28/2015	156	1537	3	9.8	0	39.8
2 multi 2		H						
13	1	11/29/2015	157	1538	3	9.5	0	38.66

2 multi	2	H							
13	1	11/30/2015	158	1539	3	9.9	0.12	40.35	
2 multi	2	H							
13	1	12/01/2015	159	1540	3	9.8	0.11	38.77	
2 multi	2	H							
13	1	12/02/2015	160	1541	3	9.6	0.36	37.02	
2 multi	2	H							
13	1	12/03/2015	161	1542	3	9.6	0	37.76	
2 multi	2	H							
13	1	12/04/2015	162	1543	3	9.8	0	40.74	
2 multi	2	H							
13	1	12/05/2015	163	1544	3	9.9	0	38.31	
2 multi	2	H							
13	1	12/06/2015	164	1545	3	9.9	0	31.22	
2 multi	2	H							
13	1	12/07/2015	165	1546	3	9.6	0	29.78	
2 multi	2	H							
13	1	12/08/2015	166	1547	3	9.6	0	35.11	
2 multi	2	H							
13	1	12/09/2015	167	1548	3	9.8	0	34.28	
2 multi	2	H							
13	1	12/10/2015	168	1549	3	9.8	0	33.49	
2 multi	2	H							
13	1	12/11/2015	169	1550	3	9.5	0	34.35	
2 multi	2	H							
13	1	12/12/2015	170	1551	3	9.8	0	35.81	
2 multi	2	H							
13	1	12/13/2015	171	1552	3	9.9	0	36.11	
2 multi	2	H							
13	1	12/14/2015	172	1553	3	9.8	0	37.76	
2 multi	2	H							
13	1	12/15/2015	173	1554	3	10.7	0	36.4	
2 multi	2	H							
13	1	12/16/2015	174	1555	3	10.4	0	36.45	
2 multi	2	H							
13	1	12/17/2015	175	1556	3	10	0	36.79	
2 multi	2	H							
13	1	12/18/2015	176	1557	3	11.3	0	35.38	
2 multi	2	H							
13	1	12/19/2015	177	1558	3	11.1	0	34.46	
2 multi	2	H							
13	1	12/20/2015	178	1559	3	10.3	0	37.94	
2 multi	2	H							
13	1	12/21/2015	179	1560	3	11.4	0	28.71	
2 multi	2	H							
13	1	12/22/2015	180	1561	3	11.3	0	9.66	
2 multi	2	H							
13	1	12/23/2015	181	1562	3	10.7	0.02	25.66	
2 multi	2	H							
13	1	12/24/2015	182	1563	3	11.4	0	33.22	
2 multi	2	H							
13	1	12/25/2015	183	1564	3	10.7	0	35.77	
2 multi	2	H							
13	1	12/26/2015	184	1565	3	10.6	0.03	31.91	

2 multi	2	H						
13	1	12/27/2015	185	1566	3	9.8	0	36.71
2 multi	2	H						
13	1	12/28/2015	186	1567	3	9.5	0	31.13
2 multi	2	H						
13	1	12/29/2015	187	1568	3	9.5	0	35.64
2 multi	2	H						
13	1	01/26/2017	119	1962	4	9.6	0	48.13
2 multi	2	H						
13	1	01/27/2017	120	1963	4	9.9	0.07	52.16
2 multi	2	H						
13	1	01/28/2017	121	1964	4	9.5	0	49.15
2 multi	2	H						
13	1	01/29/2017	122	1965	4	9.5	0.56	44.53
2 multi	2	H						
13	1	01/30/2017	123	1966	4	9.3	0	43.15
2 multi	2	H						
13	1	01/31/2017	124	1967	4	9.2	0	40.68
2 multi	2	H						
13	1	02/01/2017	125	1968	4	9.2	0	44.7
2 multi	2	H						
13	1	02/02/2017	126	1969	4	9.1	0.06	44.52
2 multi	2	H						
13	1	02/03/2017	127	1970	4	9.2	0	40.45
2 multi	2	H						
13	1	02/04/2017	128	1971	4	9.6	0	41.43
2 multi	2	H						
13	1	02/05/2017	129	1972	4	9.8	0	45.06
2 multi	2	H						
13	1	02/06/2017	130	1973	4	9.5	0	38.2
2 multi	2	H						
13	1	02/07/2017	131	1974	4	9.8	0	41.44
2 multi	2	H						
13	1	02/08/2017	132	1975	4	9.6	0	41.89
2 multi	2	H						
13	1	02/09/2017	133	1976	4	9.6	0	41.18
2 multi	2	H						
13	1	02/10/2017	134	1977	4	9.6	0	42.34
2 multi	2	H						
13	1	02/11/2017	135	1978	4	9.8	0	47.22
2 multi	2	H						
13	1	02/12/2017	136	1979	4	9.8	0	43.87
2 multi	2	H						
13	1	02/13/2017	137	1980	4	9.8	0	47.42
2 multi	2	H						
13	1	02/14/2017	138	1981	4	10.4	0	46.03
2 multi	2	H						
13	1	02/15/2017	139	1982	4	9.9	0	44.74
2 multi	2	H						
13	1	02/16/2017	140	1983	4	10.6	0	45.89
2 multi	2	H						
13	1	02/17/2017	141	1984	4	11.7	0	44.86
2 multi	2	H						
13	1	02/18/2017	142	1985	4	9.9	0	43.05

2 multi 2		H						
13	1	02/19/2017	143	1986	4	10	0	41.94
2 multi 2		H						
13	1	02/20/2017	144	1987	4	10.3	0.03	44.16
2 multi 2		H						
13	1	02/21/2017	145	1988	4	10.2	0	40.29
2 multi 2		H						
13	1	02/22/2017	146	1989	4	9.9	0	41.82
2 multi 2		H						
13	1	02/23/2017	147	1990	4	10.3	0	42.24
2 multi 2		H						
13	1	02/24/2017	148	1991	4	10	0	42.69
2 multi 2		H						
13	1	02/25/2017	149	1992	4	9.8	0	45.55
2 multi 2		H						
13	1	02/26/2017	150	1993	4	10.2	0	45.24
2 multi 2		H						
13	1	02/27/2017	151	1994	4	10.3	0	42.7
2 multi 2		H						
13	1	02/28/2017	152	1995	4	9.9	0.02	47.03
2 multi 2		H						
13	1	03/01/2017	153	1996	4	10.4	0	43.82
2 multi 2		H						
13	1	03/02/2017	154	1997	4	9.9	0	42.62
2 multi 2		H						
13	1	03/03/2017	155	1998	4	10.9	0	42.66
2 multi 2		H						
13	1	03/04/2017	156	1999	4	10.4	0	41.43
2 multi 2		H						
13	1	03/05/2017	157	2000	4	10.2	0.03	37.48
2 multi 2		H						
13	1	03/06/2017	158	2001	4	10.2	0	44.46
2 multi 2		H						
13	1	03/07/2017	159	2002	4	10.7	0	42.52
2 multi 2		H						
13	1	03/08/2017	160	2003	4	10.3	0	40.39
2 multi 2		H						
13	1	03/09/2017	161	2004	4	10.4	0	39.32
2 multi 2		H						
13	1	03/10/2017	162	2005	4	10.3	0	41.39
2 multi 2		H						
13	1	03/11/2017	163	2006	4	11.3	0	40.26
2 multi 2		H						
13	1	03/12/2017	164	2007	4	10.6	0	37.44
2 multi 2		H						
13	1	03/13/2017	165	2008	4	10.6	0	40.18
2 multi 2		H						
13	1	03/14/2017	166	2009	4	10.4	0	41.92
2 multi 2		H						
13	1	03/15/2017	167	2010	4	10	0	43.04
2 multi 2		H						
13	1	03/16/2017	168	2011	4	10.7	0	44.41
2 multi 2		H						
13	1	03/17/2017	169	2012	4	11.3	0	41.29

2 multi 2		H						
13	1	03/18/2017	170	2013	4	10.6	0	44.28
2 multi 2		H						
13	1	03/19/2017	171	2014	4	10.3	0	43.35
2 multi 2		H						
13	1	03/20/2017	172	2015	4	10.3	0	41.58
2 multi 2		H						
13	1	03/21/2017	173	2016	4	10.3	0	42.58
2 multi 2		H						
13	1	03/22/2017	174	2017	4	10.2	0	43.62
2 multi 2		H						
13	1	03/23/2017	175	2018	4	10.3	0.02	44.23
2 multi 2		H						
13	1	03/24/2017	176	2019	4	10.2	0.1	41.38
2 multi 2		H						
13	1	03/25/2017	177	2020	4	10.2	0	42.67
2 multi 2		H						
13	1	03/26/2017	178	2021	4	10.3	0	41.22
2 multi 2		H						
13	1	03/27/2017	179	2022	4	10.4	0	37.6
2 multi 2		H						
13	1	03/28/2017	180	2023	4	10.6	0	41.4
2 multi 2		H						
13	1	03/29/2017	181	2024	4	10.6	0.13	41.06
2 multi 2		H						
13	1	03/30/2017	182	2025	4	10.2	0.09	37.64
2 multi 2		H						
13	1	03/31/2017	183	2026	4	10.2	0	41.74
2 multi 2		H						
13	1	04/01/2017	184	2027	4	10.2	0	39.74
2 multi 2		H						
13	1	04/02/2017	185	2028	4	10.6	0	40.84
2 multi 2		H						
13	1	04/03/2017	186	2029	4	10.4	0	41.88
2 multi 2		H						
13	1	04/04/2017	187	2030	4	10.7	0	40.43
2 multi 2		H						
13	1	04/05/2017	188	2031	4	10.2	0	40.5
2 multi 2		H						
13	1	04/06/2017	189	2032	4	9.5	0	42.11
2 multi 2		H						
13	1	04/07/2017	190	2033	4	10.7	0.33	40.27
2 multi 2		H						
13	1	04/08/2017	191	2034	4	10.3	0	40.02
2 multi 2		H						
13	1	04/09/2017	192	2035	4	10.4	0	40.75
2 multi 2		H						
13	1	04/10/2017	193	2036	4	10.3	0	39.47
2 multi 2		H						
13	1	04/11/2017	194	2037	4	10.6	0	39.47
2 multi 2		H						
13	1	04/12/2017	195	2038	4	10.3	0	39.92
2 multi 2		H						
13	1	04/13/2017	196	2039	4	10.6	0	39.54

2 multi	2	H						
13	1	04/14/2017	197	2040	4	10.4	0	37.82
2 multi	2	H						
13	1	04/15/2017	198	2041	4	10	0	38.16
2 multi	2	H						
13	1	04/16/2017	199	2042	4	10.6	0	40.01
2 multi	2	H						
13	1	04/17/2017	200	2043	4	10.2	0	40.18
2 multi	2	H						
13	1	04/18/2017	201	2044	4	10.2	0	38.52
2 multi	2	H						
13	1	04/19/2017	202	2045	4	10	0	38.86
2 multi	2	H						
13	1	04/20/2017	203	2046	4	9.9	0	35.42
2 multi	2	H						
13	1	04/21/2017	204	2047	4	10.6	0	37.21
2 multi	2	H						
13	1	04/22/2017	205	2048	4	10.4	0	39.99
2 multi	2	H						
13	1	04/23/2017	206	2049	4	10.4	0	40.83
2 multi	2	H						
13	1	04/24/2017	207	2050	4	10.4	0	38.09
2 multi	2	H						
13	1	04/25/2017	208	2051	4	10.4	0	38.98
2 multi	2	H						
13	1	04/26/2017	209	2052	4	10.6	0.01	38.18
2 multi	2	H						
13	1	04/27/2017	210	2053	4	9.3	0	36.44
2 multi	2	H						
13	1	04/28/2017	211	2054	4	9.6	0	38.56
2 multi	2	H						
13	1	04/29/2017	212	2055	4	10.9	0	39.67
2 multi	2	H						
13	1	04/30/2017	213	2056	4	10.6	0	38.84
2 multi	2	H						
13	1	05/01/2017	214	2057	4	10.3	0	39.34
2 multi	2	H						
13	1	05/02/2017	215	2058	4	10.7	0	37.62
2 multi	2	H						
13	1	05/03/2017	216	2059	4	9.9	0	38.48
2 multi	2	H						
13	1	05/04/2017	217	2060	4	10.4	0	40.29
2 multi	2	H						
13	1	05/05/2017	218	2061	4	10.6	0	38.89
2 multi	2	H						
13	1	05/06/2017	219	2062	4	10.7	0	39.16
2 multi	2	H						
13	1	05/07/2017	220	2063	4	10.6	0	41.81
2 multi	2	H						
13	1	05/08/2017	221	2064	4	11.4	0	39.28
2 multi	2	H						
13	1	05/09/2017	222	2065	4	10.7	0	38.78
2 multi	2	H						
13	1	05/10/2017	223	2066	4	10.3	0	38.72

2 multi	2	H						
13	1	05/11/2017	224	2067	4	10.3	0	37.15
2 multi	2	H						
13	1	05/12/2017	225	2068	4	10.2	0	39.53
2 multi	2	H						
13	1	05/13/2017	226	2069	4	9.8	0	36.92
2 multi	2	H						
13	1	05/14/2017	227	2070	4	10.6	0	39.85
2 multi	2	H						
13	1	05/15/2017	228	2071	4	10.4	0	38.16
2 multi	2	H						
13	1	05/16/2017	229	2072	4	10.6	0	37.96
2 multi	2	H						
13	1	05/17/2017	230	2073	4	10.6	0	38.18
2 multi	2	H						
13	1	12/01/2017	16	2271	5	9.1	0	36.39
2 multi	1	H						
13	1	12/02/2017	17	2272	5	9.1	0.09	37.09
2 multi	1	H						
13	1	12/03/2017	18	2273	5	8.9	0.1	39.93
2 multi	1	H						
13	1	12/04/2017	19	2274	5	9.2	0.02	38.45
2 multi	1	H						
13	1	12/05/2017	20	2275	5	9.1	0.03	36.67
2 multi	1	H						
13	1	12/06/2017	21	2276	5	9.2	0.01	43.39
2 multi	1	H						
13	1	12/07/2017	22	2277	5	9.2	0.1	44.56
2 multi	2	H						
13	1	12/08/2017	23	2278	5	8.9	0	42.07
2 multi	2	H						
13	1	12/09/2017	24	2279	5	9.2	0.24	41.57
2 multi	2	H						
13	1	12/10/2017	25	2280	5	9.3	0.02	44.81
2 multi	2	H						
13	1	12/11/2017	26	2281	5	9.2	0	41.53
2 multi	2	H						
13	1	12/12/2017	27	2282	5	8.6	0	45.01
2 multi	2	H						
13	1	12/13/2017	28	2283	5	9.1	0	43.62
2 multi	2	H						
13	1	12/14/2017	29	2284	5	8.6	0	42.3
2 multi	2	H						
13	1	12/15/2017	30	2285	5	9.1	0	41.34
2 multi	2	H						
13	1	12/16/2017	31	2286	5	8.8	0	44.09
2 multi	2	H						
13	1	12/17/2017	32	2287	5	8.9	0	44.36
2 multi	2	H						
13	1	12/18/2017	33	2288	5	9.3	0	43.77
2 multi	2	H						
13	1	12/19/2017	34	2289	5	9.8	0	37.94
2 multi	2	H						
13	1	12/20/2017	35	2290	5	9.8	0.02	40.93

2 multi	2	H						
13	1	12/21/2017	36	2291	5	10	0.01	37.16
2 multi	2	H						
13	1	12/22/2017	37	2292	5	10.6	0.18	36.96
2 multi	2	H						
13	1	12/23/2017	38	2293	5	9.9	0.02	32.37
2 multi	2	H						
13	1	12/24/2017	39	2294	5	9.6	0	33.92
2 multi	2	H						
13	1	12/25/2017	40	2295	5	9.6	0.13	37.56
2 multi	2	H						
13	1	12/26/2017	41	2296	5	9.9	0.01	42.23
2 multi	2	H						
13	1	12/27/2017	42	2297	5	10.9	0.11	30.21
2 multi	2	H						
13	1	12/28/2017	43	2298	5	10.3	0.26	30.3
2 multi	2	H						
13	1	12/29/2017	44	2299	5	10.2	0.1	35.21
2 multi	2	H						
13	1	12/30/2017	45	2300	5	10	0.52	37.3
2 multi	2	H						
13	1	12/31/2017	46	2301	5	9.9	0.09	36.56
2 multi	2	H						
13	1	01/01/2018	47	2302	5	9.8	0	40.8
2 multi	2	H						
13	1	01/02/2018	48	2303	5	9.6	0	38.44
2 multi	2	H						
13	1	01/03/2018	49	2304	5	10	0	45.08
2 multi	2	H						
13	1	01/04/2018	50	2305	5	10	0	40.99
2 multi	2	H						
13	1	01/05/2018	51	2306	5	10.3	0	42.54
2 multi	2	H						
13	1	01/06/2018	52	2307	5	10.3	0.3	40.65
2 multi	2	H						
13	1	01/07/2018	53	2308	5	10.3	0.06	42.05
2 multi	2	H						
13	1	01/08/2018	54	2309	5	10	0.02	27.03
2 multi	2	H						
13	1	01/09/2018	55	2310	5	11.9	0	45.74
2 multi	2	H						
13	1	01/10/2018	56	2311	5	10.7	0.06	39.37
2 multi	2	H						
13	1	01/11/2018	57	2312	5	9.8	0.05	43.3
2 multi	2	H						
13	1	01/12/2018	58	2313	5	10.3	0.38	43.78
2 multi	2	H						
13	1	01/13/2018	59	2314	5	9.6	0.52	40.68
2 multi	2	H						
13	1	01/14/2018	60	2315	5	10.4	0.06	48.83
2 multi	2	H						
13	1	01/15/2018	61	2316	5	9.1	0	45.44
2 multi	2	H						
13	1	01/16/2018	62	2317	5	9.6	0	41.99

2 multi 2		H						
13	1	01/17/2018	63	2318	5	9.8	0.06	45.64
2 multi 2		H						
13	1	01/18/2018	64	2319	5	9.3	0.04	45.48
2 multi 2		H						
13	1	01/19/2018	65	2320	5	9.6	0.17	44.01
2 multi 2		H						
13	1	01/20/2018	66	2321	5	9.5	0	43.77
2 multi 2		H						
13	1	01/21/2018	67	2322	5	9.8	0	46
2 multi 2		H						
13	1	01/22/2018	68	2323	5	9.6	0	45.96
2 multi 2		H						
13	1	01/23/2018	69	2324	5	9.8	0.35	46.24
2 multi 2		H						
13	1	01/24/2018	70	2325	5	9.3	0	50.75
2 multi 2		H						
13	1	01/25/2018	71	2326	5	9.6	0.27	44.41
2 multi 2		H						
13	1	01/26/2018	72	2327	5	9.3	0.45	48.02
2 multi 2		H						
13	1	01/27/2018	73	2328	5	9.8	0	43.93
2 multi 2		H						
13	1	01/28/2018	74	2329	5	9.5	0.03	41.71
2 multi 2		H						
13	1	01/29/2018	75	2330	5	9.5	0	45.42
2 multi 2		H						
13	1	01/30/2018	76	2331	5	9.6	0.04	43.17
2 multi 2		H						
13	1	01/31/2018	77	2332	5	9.8	0.57	48.97
2 multi 2		H						
13	1	02/01/2018	78	2333	5	9.3	0	43.96
2 multi 2		H						
13	1	02/02/2018	79	2334	5	9.6	0	47.26
2 multi 2		H						
13	1	02/03/2018	80	2335	5	9.3	0	45.78
2 multi 2		H						
13	1	02/04/2018	81	2336	5	9.6	0.04	48.14
2 multi 2		H						
13	1	02/05/2018	82	2337	5	9.6	0.04	45.46
2 multi 2		H						
13	1	02/06/2018	83	2338	5	9.3	0	45.23
2 multi 2		H						
13	1	02/07/2018	84	2339	5	8.9	0.16	42.72
2 multi 2		H						
13	1	02/08/2018	85	2340	5	9.8	0.04	46.6
2 multi 2		H						
13	1	02/09/2018	86	2341	5	9.5	0.04	46.45
2 multi 2		H						
13	1	02/10/2018	87	2342	5	9.5	0.05	47.14
2 multi 2		H						
13	1	02/11/2018	88	2343	5	9.6	0	46.24
2 multi 2		H						
13	1	02/12/2018	89	2344	5	9.3	0	42.33

2 multi	2	H						
13	1	02/13/2018	90	2345	5	9.3	0	46.06
2 multi	2	H						
13	1	02/14/2018	91	2346	5	9.3	0.05	48.54
2 multi	2	H						
13	1	02/15/2018	92	2347	5	9.2	0	42
2 multi	2	H						
13	1	02/16/2018	93	2348	5	9.5	0	43.72
2 multi	2	H						
13	1	02/17/2018	94	2349	5	9.2	0.41	43.46
2 multi	2	H						
13	1	02/18/2018	95	2350	5	8.9	0	43.78
2 multi	2	H						
13	1	02/19/2018	96	2351	5	9.3	0	45.88
2 multi	2	H						
13	1	02/20/2018	97	2352	5	8.9	0.22	43.28
2 multi	2	H						
13	1	02/21/2018	98	2353	5	9.1	0.18	42.92
2 multi	2	H						
13	1	02/22/2018	99	2354	5	9.3	0.05	45.56
2 multi	2	H						
13	1	02/23/2018	100	2355	5	9.5	0	46.24
2 multi	2	H						
13	1	02/24/2018	101	2356	5	9.8	0.06	48.14
2 multi	2	H						
13	1	02/25/2018	102	2357	5	9.8	0.06	47.76
2 multi	2	H						
13	1	02/26/2018	103	2358	5	9.3	0.35	44.83
2 multi	2	H						
13	1	02/27/2018	104	2359	5	9.5	0	44.91
2 multi	2	H						
13	1	02/28/2018	105	2360	5	9.3	0	44.69
2 multi	2	H						
13	1	03/01/2018	106	2361	5	9.1	0	45.1
2 multi	2	H						
13	1	03/02/2018	107	2362	5	9.1	0.12	45.45
2 multi	2	H						
13	1	03/03/2018	108	2363	5	8.8	0.09	45.57
2 multi	2	H						
13	1	03/04/2018	109	2364	5	9.2	0.05	44.29
2 multi	2	H						
13	1	03/05/2018	110	2365	5	9.2	0.06	44
2 multi	2	H						
13	1	03/06/2018	111	2366	5	9.3	0.09	41.84
2 multi	2	H						
13	1	03/07/2018	112	2367	5	9.8	0	45.01
2 multi	2	H						
13	1	03/08/2018	113	2368	5	9.6	0	41.09
2 multi	2	H						
13	1	03/09/2018	114	2369	5	9.9	0.05	44.61
2 multi	2	H						
13	1	03/10/2018	115	2370	5	9.9	0	44.04
2 multi	2	H						
13	1	03/11/2018	116	2371	5	9.3	0.09	44.09

2 multi	2	H						
13	1	03/12/2018	117	2372	5	9.5	0	42
2 multi	2	H						
13	1	03/13/2018	118	2373	5	9.9	0	42.74
2 multi	2	H						
13	1	03/14/2018	119	2374	5	9.9	0	44.46
2 multi	2	H						
13	1	03/15/2018	120	2375	5	9.3	0	45.4
2 multi	2	H						
13	1	03/16/2018	121	2376	5	9.2	0	46.47
2 multi	2	H						
13	1	03/17/2018	122	2377	5	9.1	0	45.21
2 multi	2	H						
13	1	03/18/2018	123	2378	5	8.9	0.06	43.61
2 multi	2	H						
13	1	11/30/2018	9	2635	6	9.1	0.4	28.63
2 multi	1	H						
13	1	12/01/2018	10	2636	6	9.3	0	28.75
2 multi	1	H						
13	1	12/02/2018	11	2637	6	9.3	0	29.13
2 multi	1	H						
13	1	12/03/2018	12	2638	6	9.2	0.02	31.29
2 multi	1	H						
13	1	12/04/2018	13	2639	6	9.2	0.14	31.88
2 multi	1	H						
13	1	12/05/2018	14	2640	6	9.2	0.44	32.48
2 multi	1	H						
13	1	12/06/2018	15	2641	6	8.8	0	33.37
2 multi	1	H						
13	1	12/07/2018	16	2642	6	9.3	0.05	31.38
2 multi	1	H						
13	1	12/08/2018	17	2643	6	8.8	0.17	34.6
2 multi	1	H						
13	1	12/09/2018	18	2644	6	9.1	0.13	35.83
2 multi	1	H						
13	1	12/10/2018	19	2645	6	8.9	0.47	31.2
2 multi	1	H						
13	1	12/11/2018	20	2646	6	9.2	0	32.15
2 multi	1	H						
13	1	12/12/2018	21	2647	6	9.1	0.26	33.06
2 multi	1	H						
13	1	12/13/2018	22	2648	6	8.6	0.3	29.94
2 multi	2	H						
13	1	12/14/2018	23	2649	6	9.3	0.56	33.89
2 multi	2	H						
13	1	12/15/2018	24	2650	6	9.5	0	34.09
2 multi	2	H						
13	1	12/16/2018	25	2651	6	9.5	0.43	33.91
2 multi	2	H						
13	1	12/17/2018	26	2652	6	9.3	0.08	36.78
2 multi	2	H						
13	1	12/18/2018	27	2653	6	9.3	0.1	38.33
2 multi	2	H						
13	1	12/19/2018	28	2654	6	9.2	0	36.15

2 multi 2		H						
13	1	12/20/2018	29	2655	6	9.3	0	39.36
2 multi 2		H						
13	1	12/21/2018	30	2656	6	9.5	0.11	36.92
2 multi 2		H						
13	1	12/22/2018	31	2657	6	9.1	0	38.83
2 multi 2		H						
13	1	12/23/2018	32	2658	6	9.2	0	40.41
2 multi 2		H						
13	1	12/24/2018	33	2659	6	9.1	0	37.21
2 multi 2		H						
13	1	12/25/2018	34	2660	6	9.3	0.12	38.55
2 multi 2		H						
13	1	12/26/2018	35	2661	6	8.9	0.1	34.31
2 multi 2		H						
13	1	12/27/2018	36	2662	6	9.1	0.1	39.14
2 multi 2		H						
13	1	12/28/2018	37	2663	6	9.2	0.39	38.71
2 multi 2		H						
13	1	12/29/2018	38	2664	6	9.5	0.11	36.71
2 multi 2		H						
13	1	12/30/2018	39	2665	6	9.3	0.01	27.23
2 multi 2		H						
13	1	12/31/2018	40	2666	6	8.9	0	28.61
2 multi 2		H						
13	1	01/01/2019	41	2667	6	8.9	0.01	29
2 multi 2		H						
13	1	01/02/2019	42	2668	6	9.3	0.11	33.46
2 multi 2		H						
13	1	01/03/2019	43	2669	6	9.1	0.01	30.09
2 multi 2		H						
13	1	01/04/2019	44	2670	6	9.2	0.43	29.66
2 multi 2		H						
13	1	01/05/2019	45	2671	6	8.9	0.3	31.56
2 multi 2		H						
13	1	01/06/2019	46	2672	6	9.5	0.79	33.98
2 multi 2		H						
13	1	01/07/2019	47	2673	6	9.5	0	33.64
2 multi 2		H						
13	1	01/08/2019	48	2674	6	9.5	0.12	36.89
2 multi 2		H						
13	1	01/09/2019	49	2675	6	9.3	0.12	37.42
2 multi 2		H						
13	1	01/10/2019	50	2676	6	9.6	0.12	37.88
2 multi 2		H						
13	1	01/11/2019	51	2677	6	9.5	0.27	36.83
2 multi 2		H						
13	1	01/12/2019	52	2678	6	9.6	0.12	36.7
2 multi 2		H						
13	1	01/13/2019	53	2679	6	9.2	0.13	34.82
2 multi 2		H						
13	1	01/14/2019	54	2680	6	9.3	0	37.12
2 multi 2		H						
13	1	01/15/2019	55	2681	6	9.8	0.36	38.45

2 multi	2	H						
13	1	01/16/2019	56	2682	6	9.6	0	36.13
2 multi	2	H						
13	1	01/17/2019	57	2683	6	9.6	0.26	39.92
2 multi	2	H						
13	1	01/18/2019	58	2684	6	9.5	0	35.21
2 multi	2	H						
13	1	01/19/2019	59	2685	6	9.5	0.65	34.33
2 multi	2	H						
13	1	01/20/2019	60	2686	6	9.3	0	35.65
2 multi	2	H						
13	1	01/21/2019	61	2687	6	9.5	0.11	38.69
2 multi	2	H						
13	1	01/22/2019	62	2688	6	9.8	0.11	39.84
2 multi	2	H						
13	1	01/23/2019	63	2689	6	9.5	0.34	36.5
2 multi	2	H						
13	1	01/24/2019	64	2690	6	9.3	0.49	36.53
2 multi	2	H						
13	1	01/25/2019	65	2691	6	9.8	0	35.6
2 multi	2	H						
13	1	01/26/2019	66	2692	6	9.5	0.26	34.31
2 multi	2	H						
13	1	01/27/2019	67	2693	6	9.5	0	33.8
2 multi	2	H						
13	1	01/28/2019	68	2694	6	9.3	0.12	38.02
2 multi	2	H						
13	1	01/29/2019	69	2695	6	9.1	0.36	38.42
2 multi	2	H						
13	1	01/30/2019	70	2696	6	9.3	0	32.49
2 multi	2	H						
13	1	01/31/2019	71	2697	6	9.2	0	34.1
2 multi	2	H						
13	1	02/01/2019	72	2698	6	9.3	0.09	37.8
2 multi	2	H						
13	1	02/02/2019	73	2699	6	9.2	0.42	36.99
2 multi	2	H						
13	1	02/03/2019	74	2700	6	9.3	0	34.95
2 multi	2	H						
13	1	02/04/2019	75	2701	6	9.5	0.01	35.95
2 multi	2	H						
13	1	02/05/2019	76	2702	6	9.3	0.02	33.93
2 multi	2	H						
13	1	02/06/2019	77	2703	6	9.2	0.2	33.18
2 multi	2	H						
13	1	02/07/2019	78	2704	6	9.5	0	33.59
2 multi	2	H						
13	1	02/08/2019	79	2705	6	9.3	0	35.27
2 multi	2	H						
13	1	02/09/2019	80	2706	6	9.3	0.49	33.88
2 multi	2	H						
13	1	02/10/2019	81	2707	6	9.3	0.61	34.68
2 multi	2	H						
13	1	03/28/2019	127	2753	6	9.5	0.42	30.17

2 multi 2		H						
13	1	03/29/2019	128	2754	6	9.8	0	31.1
2 multi 2		H						
13	1	03/30/2019	129	2755	6	9.5	0.15	29.61
2 multi 2		H						
13	1	03/31/2019	130	2756	6	9.2	0	32.07
2 multi 2		H						
13	1	04/01/2019	131	2757	6	9.6	0.04	34.11
2 multi 2		H						
13	1	04/02/2019	132	2758	6	9.8	0.11	34.13
2 multi 2		H						
13	1	04/03/2019	133	2759	6	9.8	0.1	34.1
2 multi 2		H						
13	1	04/04/2019	134	2760	6	9.5	0.22	31.37
2 multi 2		H						
13	1	04/05/2019	135	2761	6	9.9	0.53	34.2
2 multi 2		H						
13	1	04/06/2019	136	2762	6	9.6	0.12	33.39
2 multi 2		H						
13	1	04/07/2019	137	2763	6	9.6	0.11	31.16
2 multi 2		H						
13	1	04/08/2019	138	2764	6	9.3	0.27	30.37
2 multi 2		H						
13	1	04/09/2019	139	2765	6	9.5	0	30.31
2 multi 2		H						
13	1	04/10/2019	140	2766	6	9.3	0.09	27.78
2 multi 2		H						
13	1	04/11/2019	141	2767	6	9.9	0	28.93
2 multi 2		H						
13	1	04/12/2019	142	2768	6	10.4	0.12	30.22
2 multi 2		H						
13	1	04/13/2019	143	2769	6	10.2	0.14	31.87
2 multi 2		H						
13	1	04/14/2019	144	2770	6	10.6	0.09	30.83
2 multi 2		H						
13	1	04/15/2019	145	2771	6	10.7	0.49	33.33
2 multi 2		H						
13	1	04/16/2019	146	2772	6	12	0.31	30.34
2 multi 2		H						
13	1	04/17/2019	147	2773	6	11.4	0.14	32.69
2 multi 2		H						
13	1	04/18/2019	148	2774	6	12.3	0	31.61
2 multi 2		H						
13	1	04/19/2019	149	2775	6	11.3	0	32.1
2 multi 2		H						
13	1	04/20/2019	150	2776	6	10.2	0.12	32.89
2 multi 2		H						
13	1	04/21/2019	151	2777	6	10.2	0.12	32.75
2 multi 2		H						
13	1	04/22/2019	152	2778	6	10.4	0.02	34.16
2 multi 2		H						
13	1	04/23/2019	153	2779	6	10.4	0.11	34.36
2 multi 2		H						
13	1	04/24/2019	154	2780	6	9.9	0	35.45

2 multi 2		H						
13	1	04/25/2019	155	2781	6	10.3	0	36.05
2 multi 2		H						
13	1	04/26/2019	156	2782	6	10.4	0.1	36.72
2 multi 2		H						
13	1	04/27/2019	157	2783	6	10.6	0.01	35.1
2 multi 2		H						
13	1	04/28/2019	158	2784	6	9.9	0	29.64
2 multi 2		H						
13	1	04/29/2019	159	2785	6	10.6	0	34.21
2 multi 2		H						
13	1	04/30/2019	160	2786	6	10.6	0	33.31
2 multi 2		H						
13	1	05/01/2019	161	2787	6	10.4	0	33.55
2 multi 2		H						
13	1	05/02/2019	162	2788	6	10.7	0.01	34.98
2 multi 2		H						
13	1	05/03/2019	163	2789	6	10.4	0.39	33.68
2 multi 2		H						
13	1	05/04/2019	164	2790	6	10.3	0	32.92
2 multi 2		H						
13	1	05/05/2019	165	2791	6	10.2	0	34.85
2 multi 2		H						
13	1	05/06/2019	166	2792	6	10.2	0.1	29.96
2 multi 2		H						
13	1	05/07/2019	167	2793	6	10.4	0.09	34.24
2 multi 2		H						
13	1	05/08/2019	168	2794	6	10.2	0	35.1
2 multi 2		H						
13	1	05/09/2019	169	2795	6	10.9	0.38	30.56
2 multi 2		H						
13	1	05/10/2019	170	2796	6	10.4	0.3	31.41
2 multi 2		H						
13	1	05/11/2019	171	2797	6	10	0.14	32.66
2 multi 2		H						
13	1	05/12/2019	172	2798	6	10.6	0.23	31.25
2 multi 2		H						
13	1	05/13/2019	173	2799	6	10	0.12	34.62
2 multi 2		H						
13	1	05/14/2019	174	2800	6	10.3	0.15	34.74
2 multi 2		H						
13	1	05/15/2019	175	2801	6	10.2	0.1	35.01
2 multi 2		H						
13	1	05/16/2019	176	2802	6	10.2	0.44	33.43
2 multi 2		H						
13	1	05/17/2019	177	2803	6	10.7	0.09	36.28
2 multi 2		H						
13	1	05/18/2019	178	2804	6	10.3	0.01	34.59
2 multi 2		H						
13	1	05/19/2019	179	2805	6	10.4	0	35.01
2 multi 2		H						
13	1	05/20/2019	180	2806	6	10.3	0	34.01
2 multi 2		H						
13	1	05/21/2019	181	2807	6	10.7	0	35.75

2 multi	2	H						
13	1	05/22/2019	182	2808	6	10.3	0	34.23
2 multi	2	H						
13	1	05/23/2019	183	2809	6	10	0	34.72
2 multi	2	H						
13	1	05/24/2019	184	2810	6	10	0.51	34.37
2 multi	2	H						
13	1	05/25/2019	185	2811	6	9.9	0.02	33.08
2 multi	2	H						
13	1	05/26/2019	186	2812	6	10.2	0.09	32.61
2 multi	2	H						
13	1	05/27/2019	187	2813	6	9.9	0.09	31.6
2 multi	2	H						
13	1	05/28/2019	188	2814	6	9.8	0.08	30.25
2 multi	2	H						
13	1	05/29/2019	189	2815	6	9.8	0	32.55
2 multi	2	H						
13	1	05/30/2019	190	2816	6	9.6	0.1	32.48
2 multi	2	H						
13	1	05/31/2019	191	2817	6	10.2	0	32.84
2 multi	2	H						
13	1	06/01/2019	192	2818	6	10.2	0	32.25
2 multi	2	H						
13	1	06/02/2019	193	2819	6	10	0	31.34
2 multi	2	H						
13	1	06/03/2019	194	2820	6	10.2	0.23	30.46
2 multi	2	H						
13	1	06/04/2019	195	2821	6	10	0	31.59
2 multi	2	H						
13	1	06/05/2019	196	2822	6	10	0	33.68
2 multi	2	H						
13	1	06/06/2019	197	2823	6	9.9	0.06	28.28
2 multi	2	H						
13	1	06/07/2019	198	2824	6	10.2	0.42	32.44
2 multi	2	H						
13	1	06/08/2019	199	2825	6	9.6	0.16	29.24
2 multi	2	H						
13	1	06/09/2019	200	2826	6	9.8	0	30.2
2 multi	2	H						
13	1	03/01/2020	5	3092	7	10.2	0.26	29.32
2 multi	1	H						
13	1	03/02/2020	6	3093	7	10.2	0.28	32.14
2 multi	1	H						
13	1	03/03/2020	7	3094	7	9.9	0.06	36.3
2 multi	1	H						
13	1	03/04/2020	8	3095	7	10	0.01	34.84
2 multi	1	H						
13	1	03/05/2020	9	3096	7	10.3	0.13	33.89
2 multi	1	H						
13	1	03/06/2020	10	3097	7	10	0.49	35.1
2 multi	1	H						
13	1	03/07/2020	11	3098	7	10.4	0.12	37.34
2 multi	1	H						
13	1	03/08/2020	12	3099	7	10	0.1	36.06

2 multi	1	H						
13	1	03/09/2020	13	3100	7	10.2	0.11	40.5
2 multi	1	H						
13	1	03/10/2020	14	3101	7	10	0.1	39.19
2 multi	1	H						
13	1	03/11/2020	15	3102	7	10.2	0	45.1
2 multi	1	H						
13	1	03/12/2020	16	3103	7	10.3	0.36	41.99
2 multi	1	H						
13	1	03/13/2020	17	3104	7	10.2	0	44.18
2 multi	1	H						
13	1	03/14/2020	18	3105	7	10	0.54	42.23
2 multi	1	H						
13	1	03/15/2020	19	3106	7	10.2	0.47	44.03
2 multi	1	H						
13	1	03/16/2020	20	3107	7	9.9	0	43
2 multi	1	H						
13	1	03/17/2020	21	3108	7	10.2	0.02	46.67
2 multi	1	H						
13	1	03/18/2020	22	3109	7	10	0.06	46.7
2 multi	2	H						
13	1	03/19/2020	23	3110	7	9.9	0	48.34
2 multi	2	H						
13	1	03/20/2020	24	3111	7	10.2	0	49.14
2 multi	2	H						
13	1	03/21/2020	25	3112	7	10.3	0.28	49.39
2 multi	2	H						
13	1	03/22/2020	26	3113	7	10	0	50.21
2 multi	2	H						
13	1	03/23/2020	27	3114	7	10	0.26	48.25
2 multi	2	H						
13	1	03/24/2020	28	3115	7	9.8	0.02	46.6
2 multi	2	H						
13	1	03/25/2020	29	3116	7	9.6	0.43	46.72
2 multi	2	H						
13	1	03/26/2020	30	3117	7	9.9	0.42	48.08
2 multi	2	H						
13	1	03/27/2020	31	3118	7	9.8	0.09	44.99
2 multi	2	H						
13	1	03/28/2020	32	3119	7	9.9	0.06	47.7
2 multi	2	H						
13	1	03/29/2020	33	3120	7	9.9	0.15	47.83
2 multi	2	H						
13	1	03/30/2020	34	3121	7	9.9	0.16	48.65
2 multi	2	H						
13	1	03/31/2020	35	3122	7	10	0.12	47.23
2 multi	2	H						
13	1	04/01/2020	36	3123	7	10.2	0	49.67
2 multi	2	H						
13	1	04/02/2020	37	3124	7	10	0	50.79
2 multi	2	H						
13	1	04/03/2020	38	3125	7	10.2	0.07	47.7
2 multi	2	H						
13	1	04/04/2020	39	3126	7	10.2	0.06	50.93

2 multi	2	H						
13	1	04/05/2020	40	3127	7	10	0.16	51.56
2 multi	2	H						
13	1	04/06/2020	41	3128	7	10	0.1	48.06
2 multi	2	H						
13	1	04/07/2020	42	3129	7	9.9	0.02	49.38
2 multi	2	H						
13	1	04/08/2020	43	3130	7	9.8	0.2	48.15
2 multi	2	H						
13	1	04/09/2020	44	3131	7	9.6	0.35	44.35
2 multi	2	H						
13	1	04/10/2020	45	3132	7	9.8	0.01	50.83
2 multi	2	H						
13	1	04/11/2020	46	3133	7	9.8	0	48.93
2 multi	2	H						
13	1	04/12/2020	47	3134	7	9.8	0.61	49.84
2 multi	2	H						
13	1	04/13/2020	48	3135	7	9.9	0	55.47
2 multi	2	H						
13	1	04/14/2020	49	3136	7	9.9	0	48.93
2 multi	2	H						
13	1	04/15/2020	50	3137	7	9.9	0.06	49.8
2 multi	2	H						
13	1	04/16/2020	51	3138	7	9.8	0.06	49.19
2 multi	2	H						
13	1	04/17/2020	52	3139	7	9.8	0.33	49.37
2 multi	2	H						
13	1	04/18/2020	53	3140	7	9.9	0	48.01
2 multi	2	H						
13	1	04/19/2020	54	3141	7	10.2	0	37.71
2 multi	2	H						
13	1	04/20/2020	55	3142	7	9.8	0	37.32
2 multi	2	H						
13	1	04/21/2020	56	3143	7	9.8	0	38.85
2 multi	2	H						
13	1	04/22/2020	57	3144	7	9.8	0	41.93
2 multi	2	H						
13	1	04/23/2020	58	3145	7	9.8	0.15	42.97
2 multi	2	H						
13	1	04/24/2020	59	3146	7	9.6	0.06	36.5
2 multi	2	H						
13	1	04/25/2020	60	3147	7	9.6	0	34.23
2 multi	2	H						
13	1	04/26/2020	61	3148	7	10	0	37.89
2 multi	2	H						
13	1	04/27/2020	62	3149	7	9.8	0	41.59
2 multi	2	H						
13	1	04/28/2020	63	3150	7	9.9	0.06	40.37
2 multi	2	H						
13	1	04/29/2020	64	3151	7	9.9	0	40.09
2 multi	2	H						
13	1	04/30/2020	65	3152	7	9.8	0	39.1
2 multi	2	H						
13	1	05/01/2020	66	3153	7	9.9	0	40.36

2 multi	2	H						
13	1	05/02/2020	67	3154	7	10	0.09	41.12
2 multi	2	H						
13	1	05/03/2020	68	3155	7	10.3	0.05	42.32
2 multi	2	H						
13	1	05/04/2020	69	3156	7	10.3	0.06	42.59
2 multi	2	H						
13	1	05/05/2020	70	3157	7	10.2	0.02	40.91
2 multi	2	H						
13	1	05/06/2020	71	3158	7	10	0	40.3
2 multi	2	H						
13	1	05/07/2020	72	3159	7	10	0.19	41.8
2 multi	2	H						
13	1	05/08/2020	73	3160	7	9.9	0.07	42.53
2 multi	2	H						
13	1	05/09/2020	74	3161	7	10.3	0.24	44.68
2 multi	2	H						
13	1	05/10/2020	75	3162	7	10	0.03	43.84
2 multi	2	H						
13	1	05/11/2020	76	3163	7	9.9	0.11	41.57
2 multi	2	H						
13	1	05/12/2020	77	3164	7	9.5	0.07	38.83
2 multi	2	H						
13	1	05/13/2020	78	3165	7	10	0.07	41.61
2 multi	2	H						
13	1	06/30/2020	126	3213	7	10	0	28.52
2 multi	2	H						
13	1	07/01/2020	127	3214	7	10.6	0	35.15
2 multi	2	H						
13	1	07/02/2020	128	3215	7	9.9	0	33
2 multi	2	H						
13	1	07/03/2020	129	3216	7	10.3	0.23	37.07
2 multi	2	H						
13	1	07/04/2020	130	3217	7	9.9	0.22	36.9
2 multi	2	H						
13	1	07/05/2020	131	3218	7	9.9	0.33	41.01
2 multi	2	H						
13	1	07/06/2020	132	3219	7	9.9	0.01	41.14
2 multi	2	H						
13	1	07/07/2020	133	3220	7	10.2	0	41.39
2 multi	2	H						
13	1	07/08/2020	134	3221	7	10	0.11	42.39
2 multi	2	H						
13	1	07/09/2020	135	3222	7	10.2	0	40.89
2 multi	2	H						
13	1	07/10/2020	136	3223	7	10	0.07	42.06
2 multi	2	H						
13	1	07/11/2020	137	3224	7	9.9	0.15	39.3
2 multi	2	H						
13	1	07/12/2020	138	3225	7	9.6	0.11	35.93
2 multi	2	H						
13	1	07/13/2020	139	3226	7	9.9	0	40.39
2 multi	2	H						
13	1	07/14/2020	140	3227	7	9.3	0.24	39.71

2 multi 2		H						
13	1	07/15/2020	141	3228	7	9.8	0	38.49
2 multi 2		H						
13	1	07/16/2020	142	3229	7	9.8	0	40.03
2 multi 2		H						
13	1	07/17/2020	143	3230	7	10	0.75	38.26
2 multi 2		H						
13	1	07/18/2020	144	3231	7	9.1	0	35.45
2 multi 2		H						
13	1	07/19/2020	145	3232	7	9.1	0.04	37.23
2 multi 2		H						
13	1	07/20/2020	146	3233	7	10	0.07	39.46
2 multi 2		H						
13	1	07/21/2020	147	3234	7	9.9	0.16	37.48
2 multi 2		H						
13	1	07/22/2020	148	3235	7	10	0.09	39.38
2 multi 2		H						
13	1	07/23/2020	149	3236	7	9.8	0.18	35.35
2 multi 2		H						
13	1	07/24/2020	150	3237	7	9.8	0.09	36.27
2 multi 2		H						
13	1	07/25/2020	151	3238	7	9.9	0.08	36.93
2 multi 2		H						
13	1	07/26/2020	152	3239	7	10.2	0.1	38.31
2 multi 2		H						
13	1	07/27/2020	153	3240	7	9.8	0	33.74
2 multi 2		H						
13	1	07/28/2020	154	3241	7	9.8	0.21	36.59
2 multi 2		H						
13	1	07/29/2020	155	3242	7	9.8	0.18	36.8
2 multi 2		H						
13	1	07/30/2020	156	3243	7	9.6	0.01	36.25
2 multi 2		H						
13	1	07/31/2020	157	3244	7	10.2	0	38.09
2 multi 2		H						
13	1	08/01/2020	158	3245	7	9.5	0.08	37.24
2 multi 2		H						
13	1	08/02/2020	159	3246	7	9.9	0.11	39.26
2 multi 2		H						
13	1	08/03/2020	160	3247	7	9.8	0.59	37.8
2 multi 2		H						
13	1	08/04/2020	161	3248	7	9.8	0.24	39.33
2 multi 2		H						
13	1	08/05/2020	162	3249	7	9.9	0.21	43.84
2 multi 2		H						
13	1	08/06/2020	163	3250	7	9.9	0	40.68
2 multi 2		H						
13	1	08/07/2020	164	3251	7	9.6	0.18	39.51
2 multi 2		H						
13	1	08/08/2020	165	3252	7	9.9	0.01	40.79
2 multi 2		H						
13	1	08/09/2020	166	3253	7	9.8	0	39.81
2 multi 2		H						
13	1	08/10/2020	167	3254	7	9.6	0.13	39.69

2 multi 2		H						
13	1	08/11/2020	168	3255	7	9.5	0.09	38.31
2 multi 2		H						
13	1	08/12/2020	169	3256	7	9.8	0	39.69
2 multi 2		H						
13	1	08/13/2020	170	3257	7	9.9	0.11	37.83
2 multi 2		H						
13	1	08/14/2020	171	3258	7	9.9	0	37.48
2 multi 2		H						
13	1	08/15/2020	172	3259	7	9.5	0.11	36.97
2 multi 2		H						
13	1	08/16/2020	173	3260	7	9.3	0.1	37.25
2 multi 2		H						
13	1	08/17/2020	174	3261	7	9.6	0.25	35.48
2 multi 2		H						
13	1	08/18/2020	175	3262	7	10	0.12	37.72
2 multi 2		H						
13	1	08/19/2020	176	3263	7	10.2	0.07	39.31
2 multi 2		H						
13	1	08/20/2020	177	3264	7	10	0.31	39.84
2 multi 2		H						
13	1	08/21/2020	178	3265	7	9.9	0.04	38.42
2 multi 2		H						
13	1	08/22/2020	179	3266	7	9.9	0.03	35.49
2 multi 2		H						
13	1	08/23/2020	180	3267	7	9.8	0.51	36.83
2 multi 2		H						
13	1	08/24/2020	181	3268	7	10	0.08	35.85
2 multi 2		H						
13	1	08/25/2020	182	3269	7	10	0.12	35.4
2 multi 2		H						
13	1	08/26/2020	183	3270	7	10.2	0.11	37.85
2 multi 2		H						
13	1	08/27/2020	184	3271	7	10	0.1	37.63
2 multi 2		H						
13	1	08/28/2020	185	3272	7	9.9	0	31.08
2 multi 2		H						
13	1	08/29/2020	186	3273	7	9.2	0	33.13
2 multi 2		H						
13	1	08/30/2020	187	3274	7	9.5	0.03	37.61
2 multi 2		H						
13	1	08/31/2020	188	3275	7	9.8	0	35.49
2 multi 2		H						
13	1	09/01/2020	189	3276	7	9.8	0.04	36.36
2 multi 2		H						
13	1	09/02/2020	190	3277	7	10.2	0.13	36.66
2 multi 2		H						
13	1	09/03/2020	191	3278	7	10	0.11	35.22
2 multi 2		H						
13	1	09/04/2020	192	3279	7	10	0	34.78
2 multi 2		H						
13	1	09/05/2020	193	3280	7	10	0.59	33.75
2 multi 2		H						
14	1	09/09/2020	5	735	1	10.7	0.1	13.32

1 primi	1	C						
14	1	09/10/2020	6	736	1	10.3	0.24	12.38
1 primi	1	C						
14	1	09/11/2020	7	737	1	10.2	0.08	15.61
1 primi	1	C						
14	1	09/12/2020	8	738	1	10.4	0.16	16.4
1 primi	1	C						
14	1	09/13/2020	9	739	1	10.6	0.06	18.5
1 primi	1	C						
14	1	09/14/2020	10	740	1	10.4	0.11	18.64
1 primi	1	C						
14	1	09/15/2020	11	741	1	9.8	0	18.78
1 primi	1	C						
14	1	09/16/2020	12	742	1	9.6	0.12	20.23
1 primi	1	C						
14	1	09/17/2020	13	743	1	9.9	0.06	20.44
1 primi	1	C						
14	1	09/18/2020	14	744	1	9.6	0	19.79
1 primi	1	C						
14	1	09/19/2020	15	745	1	9.9	0.13	23.43
1 primi	1	C						
14	1	09/20/2020	16	746	1	9.9	0	23.22
1 primi	1	C						
14	1	09/21/2020	17	747	1	9.6	0	23.65
1 primi	1	C						
14	1	09/22/2020	18	748	1	9.8	0.01	24.83
1 primi	1	C						
14	1	09/23/2020	19	749	1	9.5	0	23.92
1 primi	1	C						
14	1	09/24/2020	20	750	1	9.5	0.25	26.6
1 primi	1	C						
14	1	09/25/2020	21	751	1	9.5	0.01	25.22
1 primi	1	C						
14	1	09/26/2020	22	752	1	9.5	0.06	27.1
1 primi	2	C						
14	1	09/27/2020	23	753	1	9.6	0.16	26.1
1 primi	2	C						
14	1	09/28/2020	24	754	1	9.5	0.15	25.81
1 primi	2	C						
14	1	09/29/2020	25	755	1	9.5	0.08	27.36
1 primi	2	C						
14	1	09/30/2020	26	756	1	9.5	0.01	27.89
1 primi	2	C						
14	1	10/01/2020	27	757	1	9.3	0.02	28.35
1 primi	2	C						
14	1	10/02/2020	28	758	1	9.3	0.07	27.06
1 primi	2	C						
14	1	10/03/2020	29	759	1	9.6	0.08	29.02
1 primi	2	C						
14	1	10/04/2020	30	760	1	9.5	0.19	27.83
1 primi	2	C						
14	1	10/05/2020	31	761	1	9.5	0.2	27.81
1 primi	2	C						
14	1	10/06/2020	32	762	1	9.1	0.1	27.36

1 primi	2	C						
14	1	10/07/2020	33	763	1	9.5	0.27	27.64
1 primi	2	C						
14	1	10/08/2020	34	764	1	9.2	0	29.01
1 primi	2	C						
14	1	10/09/2020	35	765	1	9.2	0.15	29.29
1 primi	2	C						
14	1	10/10/2020	36	766	1	9.3	0.02	30.5
1 primi	2	C						
14	1	10/11/2020	37	767	1	8.9	0.15	28.31
1 primi	2	C						
14	1	10/12/2020	38	768	1	9.5	0.01	30.73
1 primi	2	C						
14	1	10/13/2020	39	769	1	9.3	0.14	28.6
1 primi	2	C						
15	1	02/15/2021	5	654	1	9.2	0.3	17.85
1 primi	1	H						
15	1	02/16/2021	6	655	1	9.8	0.14	20.29
1 primi	1	H						
15	1	02/17/2021	7	656	1	9.6	0.05	20.46
1 primi	1	H						
15	1	02/18/2021	8	657	1	9.6	0.03	21.62
1 primi	1	H						
15	1	02/19/2021	9	658	1	9.6	0	21.84
1 primi	1	H						
15	1	02/20/2021	10	659	1	9.5	0	24.01
1 primi	1	H						
15	1	02/21/2021	11	660	1	9.2	0.05	23.41
1 primi	1	H						
15	1	02/22/2021	12	661	1	8.7	0	22.22
1 primi	1	H						
15	1	02/23/2021	13	662	1	8.8	0.14	24.46
1 primi	1	H						
15	1	02/24/2021	14	663	1	8.7	0.02	25.97
1 primi	1	H						
15	1	02/25/2021	15	664	1	8.7	0.01	25.55
1 primi	1	H						
15	1	02/26/2021	16	665	1	8.7	0.64	24.43
1 primi	1	H						
15	1	02/27/2021	17	666	1	8.5	0.17	27.23
1 primi	1	H						
15	1	02/28/2021	18	667	1	8.5	0	26
1 primi	1	H						
15	1	03/01/2021	19	668	1	9.1	0.01	28.11
1 primi	1	H						
15	1	03/02/2021	20	669	1	9.9	0.1	27.98
1 primi	1	H						
15	1	03/03/2021	21	670	1	9.1	0.02	29.1
1 primi	1	H						
15	1	03/04/2021	22	671	1	9.3	0	26.02
1 primi	2	H						
15	1	03/05/2021	23	672	1	9.1	0	27.65
1 primi	2	H						
15	1	03/06/2021	24	673	1	9.1	0	28.08

1 primi 2		H						
15	1	03/07/2021	25	674	1	8.9	0.02	29.17
1 primi 2		H						
15	1	03/08/2021	26	675	1	9.2	0.05	29.21
1 primi 2		H						
15	1	03/09/2021	27	676	1	9.2	0	29.94
1 primi 2		H						
15	1	03/10/2021	28	677	1	9.1	0	28.11
1 primi 2		H						
15	1	03/11/2021	29	678	1	8.9	0.01	27.49
1 primi 2		H						
15	1	03/12/2021	30	679	1	8.7	0.02	26.68
1 primi 2		H						
15	1	03/13/2021	31	680	1	8.7	0	28.52
1 primi 2		H						
15	1	03/14/2021	32	681	1	8.6	0	30.69
1 primi 2		H						
15	1	03/15/2021	33	682	1	8.9	0	28.44
1 primi 2		H						
15	1	03/16/2021	34	683	1	9.2	0.01	30.21
1 primi 2		H						
15	1	03/17/2021	35	684	1	9.1	0.31	31.01
1 primi 2		H						
15	1	03/18/2021	36	685	1	9.1	0	32.78
1 primi 2		H						
15	1	03/19/2021	37	686	1	9.5	0	31.2
1 primi 2		H						
15	1	03/20/2021	38	687	1	9.3	0	31.33
1 primi 2		H						
15	1	03/21/2021	39	688	1	9.1	0	31.47
1 primi 2		H						
15	1	03/22/2021	40	689	1	8.6	0.25	31.86
1 primi 2		H						
15	1	03/23/2021	41	690	1	9.5	0	30.82
1 primi 2		H						
15	1	03/24/2021	42	691	1	9.1	0.21	31.52
1 primi 2		H						
15	1	03/25/2021	43	692	1	9.1	0.2	31.4
1 primi 2		H						
15	1	03/26/2021	44	693	1	9.1	0.2	30.7
1 primi 2		H						
15	1	03/27/2021	45	694	1	9.3	0	32.51
1 primi 2		H						
15	1	03/28/2021	46	695	1	8.9	0.16	31.94
1 primi 2		H						
15	1	03/29/2021	47	696	1	9.3	0.16	33.86
1 primi 2		H						
15	1	03/30/2021	48	697	1	9.3	0.33	32.91
1 primi 2		H						
15	1	03/31/2021	49	698	1	9.2	0.06	31.7
1 primi 2		H						
15	1	04/01/2021	50	699	1	8.8	0.34	29.79
1 primi 2		H						
15	1	04/02/2021	51	700	1	9.2	0.16	32.89

1 primi 2 H
15 1 04/03/2021 52 701 1 9.5 0.02 34.86
1 primi 2 H
15 1 04/04/2021 53 702 1 9.3 0.25 33.58
1 primi 2 H
15 1 04/05/2021 54 703 1 9.1 0.18 31.89
1 primi 2 H
15 1 04/06/2021 55 704 1 9.6 0 32.78
1 primi 2 H
15 1 04/07/2021 56 705 1 9.3 0 30.06
1 primi 2 H
15 1 04/08/2021 57 706 1 9.1 0 29.53
1 primi 2 H
15 1 04/09/2021 58 707 1 9.3 0.01 33
1 primi 2 H
15 1 04/10/2021 59 708 1 9.5 0 33.5
1 primi 2 H
15 1 04/11/2021 60 709 1 9.5 0 33.76
1 primi 2 H
15 1 04/12/2021 61 710 1 9.3 0.09 34.87
1 primi 2 H
15 1 04/13/2021 62 711 1 9.1 0 32.93
1 primi 2 H
15 1 04/14/2021 63 712 1 9.2 0.06 33.5
1 primi 2 H
15 1 04/15/2021 64 713 1 9.3 0.03 34.42
1 primi 2 H
16 1 11/10/2016 8 771 1 9.8 0.03 26.56
1 primi 1 H
16 1 11/11/2016 9 772 1 10.3 0 27.53
1 primi 1 H
16 1 11/12/2016 10 773 1 10.4 0 28.25
1 primi 1 H
16 1 11/13/2016 11 774 1 11.3 0 29.74
1 primi 1 H
16 1 11/14/2016 12 775 1 10.3 0 27.93
1 primi 1 H
16 1 11/15/2016 13 776 1 10.2 0 29.92
1 primi 1 H
16 1 11/16/2016 14 777 1 9.9 0 28.97
1 primi 1 H
16 1 11/17/2016 15 778 1 9.9 0 29.05
1 primi 1 H
16 1 11/18/2016 16 779 1 9.6 0 28.32
1 primi 1 H
16 1 11/19/2016 17 780 1 9.6 0 29.09
1 primi 1 H
16 1 11/20/2016 18 781 1 9.6 0 29.47
1 primi 1 H
16 1 11/21/2016 19 782 1 9.9 0 29.29
1 primi 1 H
16 1 11/22/2016 20 783 1 9.3 0 29.39
1 primi 1 H
16 1 11/23/2016 21 784 1 9.6 0 27.74

1 primi	1	H						
16	1	11/24/2016	22	785	1	9.6	0	30.85
1 primi	2	H						
16	1	11/25/2016	23	786	1	10	0	25.81
1 primi	2	H						
16	1	11/26/2016	24	787	1	10.4	0	24.07
1 primi	2	H						
16	1	11/27/2016	25	788	1	10.6	0.02	25.73
1 primi	2	H						
16	1	11/28/2016	26	789	1	9.9	0	27.5
1 primi	2	H						
16	1	11/29/2016	27	790	1	10.2	0	26.42
1 primi	2	H						
16	1	11/30/2016	28	791	1	10	0	28.59
1 primi	2	H						
16	1	12/01/2016	29	792	1	0	0	31.84
1 primi	2	H						
16	1	12/02/2016	30	793	1	9.8	0	28.23
1 primi	2	H						
16	1	12/03/2016	31	794	1	9.9	0.17	28.71
1 primi	2	H						
16	1	12/04/2016	32	795	1	9.9	0	31.45
1 primi	2	H						
16	1	12/05/2016	33	796	1	9.8	0	28.94
1 primi	2	H						
16	1	12/06/2016	34	797	1	9.6	0	26.55
1 primi	2	H						
16	1	12/07/2016	35	798	1	9.5	0	29.7
1 primi	2	H						
16	1	12/08/2016	36	799	1	9.6	0	28.53
1 primi	2	H						
16	1	12/09/2016	37	800	1	9.5	0	29
1 primi	2	H						
16	1	12/10/2016	38	801	1	10	0	31.07
1 primi	2	H						
16	1	12/11/2016	39	802	1	10	0	31.89
1 primi	2	H						
16	1	12/12/2016	40	803	1	10	0	31.68
1 primi	2	H						
16	1	12/13/2016	41	804	1	9.9	0	32.52
1 primi	2	H						
16	1	12/14/2016	42	805	1	10	0	32.06
1 primi	2	H						
16	1	12/15/2016	43	806	1	9.9	0	33.22
1 primi	2	H						
16	1	12/16/2016	44	807	1	10	0	33.5
1 primi	2	H						
16	1	12/17/2016	45	808	1	10	0	36.29
1 primi	2	H						
16	1	12/18/2016	46	809	1	10.2	0	35.58
1 primi	2	H						
16	1	12/19/2016	47	810	1	10	0	36.25
1 primi	2	H						
16	1	12/20/2016	48	811	1	9.6	0	37.38

1 primi	2	H						
16	1	12/21/2016	49	812	1	9.6	0	37.62
1 primi	2	H						
16	1	12/22/2016	50	813	1	9.3	0	34.86
1 primi	2	H						
16	1	12/23/2016	51	814	1	9.8	0	36.79
1 primi	2	H						
16	1	12/24/2016	52	815	1	9.9	0	36.92
1 primi	2	H						
16	1	12/25/2016	53	816	1	9.5	0	35.64
1 primi	2	H						
16	1	12/26/2016	54	817	1	9.6	0	36.32
1 primi	2	H						
16	1	12/27/2016	55	818	1	9.6	0	38.38
1 primi	2	H						
16	1	12/28/2016	56	819	1	9.8	0	37.95
1 primi	2	H						
16	1	12/29/2016	57	820	1	9.5	0	34.8
1 primi	2	H						
16	1	12/30/2016	58	821	1	9.8	0	36.99
1 primi	2	H						
16	1	12/31/2016	59	822	1	9.8	0	37.21
1 primi	2	H						
16	1	01/01/2017	60	823	1	9.8	0	37.77
1 primi	2	H						
16	1	01/02/2017	61	824	1	9.6	0	36.97
1 primi	2	H						
16	1	01/03/2017	62	825	1	9.8	0	35.64
1 primi	2	H						
16	1	01/04/2017	63	826	1	9.5	0	37.01
1 primi	2	H						
16	1	01/05/2017	64	827	1	9.6	0	37.42
1 primi	2	H						
16	1	01/06/2017	65	828	1	9.5	0	36.7
1 primi	2	H						
16	1	01/07/2017	66	829	1	9.8	0	38.42
1 primi	2	H						
16	1	01/08/2017	67	830	1	9.6	0	37.03
1 primi	2	H						
16	1	01/09/2017	68	831	1	9.3	0	38.01
1 primi	2	H						
16	1	01/10/2017	69	832	1	9.6	0	38.77
1 primi	2	H						
16	1	01/11/2017	70	833	1	9.3	0	37.49
1 primi	2	H						
16	1	01/12/2017	71	834	1	9.6	0	39.45
1 primi	2	H						
16	1	01/13/2017	72	835	1	9.6	0	38.63
1 primi	2	H						
16	1	01/14/2017	73	836	1	9.5	0	38.61
1 primi	2	H						
16	1	01/15/2017	74	837	1	9.3	0	37.01
1 primi	2	H						
16	1	01/16/2017	75	838	1	9.5	0	38.56

1 primi 2		H						
16	1	01/17/2017	76	839	1	9.6	0	39.71
1 primi 2		H						
16	1	01/18/2017	77	840	1	9.5	0	38.53
1 primi 2		H						
16	1	01/19/2017	78	841	1	9.6	0	38.86
1 primi 2		H						
16	1	01/20/2017	79	842	1	9.5	0	37.91
1 primi 2		H						
16	1	01/21/2017	80	843	1	9.8	0	39.51
1 primi 2		H						
16	1	01/22/2017	81	844	1	9.8	0	38.39
1 primi 2		H						
16	1	01/23/2017	82	845	1	9.6	0	39
1 primi 2		H						
16	1	01/24/2017	83	846	1	9.6	0	36.75
1 primi 2		H						
16	1	01/25/2017	84	847	1	9.5	0	37.35
1 primi 2		H						
16	1	01/26/2017	85	848	1	9.5	0	37.26
1 primi 2		H						
16	1	01/27/2017	86	849	1	9.8	0	37.06
1 primi 2		H						
16	1	01/28/2017	87	850	1	9.3	0	35.74
1 primi 2		H						
16	1	01/29/2017	88	851	1	9.3	0	36.28
1 primi 2		H						
16	1	01/30/2017	89	852	1	9.8	0	35.6
1 primi 2		H						
16	1	01/31/2017	90	853	1	8.9	0	35.41
1 primi 2		H						
16	1	02/01/2017	91	854	1	9.6	0	35.11
1 primi 2		H						
16	1	02/02/2017	92	855	1	9.6	0	36.39
1 primi 2		H						
16	1	02/03/2017	93	856	1	9.8	0	37.68
1 primi 2		H						
16	1	02/04/2017	94	857	1	9.9	0	37.57
1 primi 2		H						
16	1	02/05/2017	95	858	1	9.8	0	36.6
1 primi 2		H						
16	1	02/06/2017	96	859	1	9.6	0	39.03
1 primi 2		H						
16	1	02/07/2017	97	860	1	9.6	0.24	39.19
1 primi 2		H						
16	1	02/08/2017	98	861	1	9.5	0	40.56
1 primi 2		H						
16	1	02/09/2017	99	862	1	9.5	0	39.22
1 primi 2		H						
16	1	02/10/2017	100	863	1	9.5	0	37.36
1 primi 2		H						
16	1	02/11/2017	101	864	1	9.8	0	39.2
1 primi 2		H						
16	1	02/12/2017	102	865	1	9.1	0	38.75

1 primi 2 H
16 1 02/13/2017 103 866 1 9.8 0 37.95
1 primi 2 H
16 1 02/14/2017 104 867 1 9.8 0 37.51
1 primi 2 H
16 1 02/15/2017 105 868 1 9.8 0.28 34.92
1 primi 2 H
16 1 02/16/2017 106 869 1 9.8 0 34.57
1 primi 2 H
16 1 02/17/2017 107 870 1 9.6 0 34.76
1 primi 2 H
16 1 02/18/2017 108 871 1 9.6 0 38.4
1 primi 2 H
16 1 02/19/2017 109 872 1 9.6 0 35.67
1 primi 2 H
16 1 02/20/2017 110 873 1 10 0 38.48
1 primi 2 H
16 1 02/21/2017 111 874 1 9.5 0 38.25
1 primi 2 H
16 1 02/22/2017 112 875 1 9.6 0 40.27
1 primi 2 H
16 1 02/23/2017 113 876 1 9.6 0 38.03
1 primi 2 H
16 1 02/24/2017 114 877 1 9.3 0 36.18
1 primi 2 H
16 1 02/25/2017 115 878 1 9.5 0 38.45
1 primi 2 H
16 1 02/26/2017 116 879 1 9.6 0 38.75
1 primi 2 H
16 1 02/27/2017 117 880 1 9.6 0 37.94
1 primi 2 H
16 1 02/28/2017 118 881 1 9.8 0 39.02
1 primi 2 H
16 1 03/01/2017 119 882 1 9.8 0 37.98
1 primi 2 H
16 1 03/02/2017 120 883 1 9.6 0 38.47
1 primi 2 H
16 1 03/03/2017 121 884 1 9.3 0.04 33.77
1 primi 2 H
16 1 03/04/2017 122 885 1 9.5 0 35.92
1 primi 2 H
16 1 03/05/2017 123 886 1 9.6 0.19 36.13
1 primi 2 H
16 1 03/06/2017 124 887 1 9.6 0 37.39
1 primi 2 H
16 1 03/07/2017 125 888 1 9.6 0 39.96
1 primi 2 H
16 1 03/08/2017 126 889 1 9.6 0 39.01
1 primi 2 H
16 1 03/09/2017 127 890 1 9.6 0 37.66
1 primi 2 H
16 1 03/10/2017 128 891 1 9.8 0 35.53
1 primi 2 H
16 1 03/11/2017 129 892 1 9.5 0 37.66

1 primi 2		H						
16	1	03/12/2017	130	893	1	9.8	0	37.58
1 primi 2		H						
16	1	03/13/2017	131	894	1	9.9	0	39.93
1 primi 2		H						
16	1	03/14/2017	132	895	1	9.8	0	34.61
1 primi 2		H						
16	1	03/15/2017	133	896	1	9.6	0	38.03
1 primi 2		H						
16	1	03/16/2017	134	897	1	11.7	0.02	55.85
1 primi 2		H						
16	1	03/17/2017	135	898	1	9.6	0	38.38
1 primi 2		H						
16	1	03/18/2017	136	899	1	9.8	0.03	37.14
1 primi 2		H						
16	1	03/19/2017	137	900	1	9.9	0	37.27
1 primi 2		H						
16	1	03/20/2017	138	901	1	9.3	0	38.67
1 primi 2		H						
16	1	03/21/2017	139	902	1	9.6	0	37.61
1 primi 2		H						
16	1	03/22/2017	140	903	1	9.6	0	39.36
1 primi 2		H						
16	1	03/23/2017	141	904	1	9.6	0	39.73
1 primi 2		H						
16	1	03/24/2017	142	905	1	9.9	0	36.5
1 primi 2		H						
16	1	03/25/2017	143	906	1	9.6	0.01	39.01
1 primi 2		H						
16	1	03/26/2017	144	907	1	9.6	0	38.57
1 primi 2		H						
16	1	03/27/2017	145	908	1	9.6	0	38.8
1 primi 2		H						
16	1	03/28/2017	146	909	1	9.8	0	37.56
1 primi 2		H						
16	1	03/29/2017	147	910	1	9.6	0.34	37.31
1 primi 2		H						
16	1	03/30/2017	148	911	1	9.8	0	37.34
1 primi 2		H						
16	1	03/31/2017	149	912	1	9.9	0	40.35
1 primi 2		H						
16	1	04/01/2017	150	913	1	9.6	0	38.45
1 primi 2		H						
16	1	04/02/2017	151	914	1	9.8	0	38.77
1 primi 2		H						
16	1	04/03/2017	152	915	1	9.8	0	39.08
1 primi 2		H						
16	1	04/04/2017	153	916	1	9.8	0	38.47
1 primi 2		H						
16	1	04/05/2017	154	917	1	9.9	0	39.84
1 primi 2		H						
16	1	04/06/2017	155	918	1	9.8	0	40.22
1 primi 2		H						
16	1	04/07/2017	156	919	1	9.6	0	39.41

1 primi 2		H						
16	1	04/08/2017	157	920	1	9.9	0.21	39.31
1 primi 2		H						
16	1	04/09/2017	158	921	1	9.6	0	38.16
1 primi 2		H						
16	1	04/10/2017	159	922	1	10.2	0	37.4
1 primi 2		H						
16	1	04/11/2017	160	923	1	10	0	39.29
1 primi 2		H						
16	1	04/12/2017	161	924	1	9.6	0	37.4
1 primi 2		H						
16	1	04/13/2017	162	925	1	9.5	0	38.83
1 primi 2		H						
16	1	04/14/2017	163	926	1	9.8	0	39.38
1 primi 2		H						
16	1	04/15/2017	164	927	1	10	0	43.77
1 primi 2		H						
16	1	04/16/2017	165	928	1	9.9	0	43.51
1 primi 2		H						
16	1	04/17/2017	166	929	1	9.6	0	40.53
1 primi 2		H						
16	1	04/18/2017	167	930	1	9.6	0	40.66
1 primi 2		H						
16	1	04/19/2017	168	931	1	9.6	0	37.55
1 primi 2		H						
16	1	04/20/2017	169	932	1	9.6	0	38.52
1 primi 2		H						
16	1	04/21/2017	170	933	1	9.6	0	36.4
1 primi 2		H						
16	1	04/22/2017	171	934	1	9.5	0	37.03
1 primi 2		H						
16	1	04/23/2017	172	935	1	9.8	0	37.14
1 primi 2		H						
16	1	04/24/2017	173	936	1	9.8	0	36.15
1 primi 2		H						
16	1	04/25/2017	174	937	1	9.9	0	34.77
1 primi 2		H						
16	1	04/26/2017	175	938	1	9.8	0	38.27
1 primi 2		H						
16	1	12/29/2017	10	1185	2	9.5	0.07	37.51
2 multi 1		H						
16	1	12/30/2017	11	1186	2	9.5	0	39.47
2 multi 1		H						
16	1	12/31/2017	12	1187	2	9.5	0	35.27
2 multi 1		H						
16	1	01/01/2018	13	1188	2	9.5	0	42.11
2 multi 1		H						
16	1	01/02/2018	14	1189	2	9.5	0	42.74
2 multi 1		H						
16	1	01/03/2018	15	1190	2	9.8	0	44.17
2 multi 1		H						
16	1	01/04/2018	16	1191	2	9.6	0	40.94
2 multi 1		H						
16	1	01/05/2018	17	1192	2	9.6	0	40.48

2 multi 1 H
16 1 01/06/2018 18 1193 2 9.6 0 41.8
2 multi 1 H
16 1 01/07/2018 19 1194 2 9.6 0.33 43.38
2 multi 1 H
16 1 01/08/2018 20 1195 2 10.4 0.41 45.64
2 multi 1 H
16 1 01/09/2018 21 1196 2 10.2 0 34.67
2 multi 1 H
16 1 01/10/2018 22 1197 2 10.2 0 45.24
2 multi 2 H
16 1 01/11/2018 23 1198 2 10.2 0.26 47.07
2 multi 2 H
16 1 01/12/2018 24 1199 2 10.2 0.34 52.2
2 multi 2 H
16 1 01/13/2018 25 1200 2 10.6 0.19 48.12
2 multi 2 H
16 1 01/14/2018 26 1201 2 9.9 0.3 44.73
2 multi 2 H
16 1 01/15/2018 27 1202 2 10 0.02 46.83
2 multi 2 H
16 1 01/16/2018 28 1203 2 10 0 48.24
2 multi 2 H
16 1 01/17/2018 29 1204 2 9.9 0 47.41
2 multi 2 H
16 1 01/18/2018 30 1205 2 10 0 55.6
2 multi 2 H
16 1 01/19/2018 31 1206 2 10.2 0 47.2
2 multi 2 H
16 1 01/20/2018 32 1207 2 10 0 51.41
2 multi 2 H
16 1 01/21/2018 33 1208 2 9.9 0 50.74
2 multi 2 H
16 1 01/22/2018 34 1209 2 9.8 0.3 44.75
2 multi 2 H
16 1 01/23/2018 35 1210 2 10.4 0.2 42.83
2 multi 2 H
16 1 01/24/2018 36 1211 2 10.2 0.06 44.43
2 multi 2 H
16 1 01/25/2018 37 1212 2 10.3 0 48.98
2 multi 2 H
16 1 01/26/2018 38 1213 2 10.2 0 47.75
2 multi 2 H
16 1 01/27/2018 39 1214 2 10.2 0 48.26
2 multi 2 H
16 1 01/28/2018 40 1215 2 10.3 0.32 48.63
2 multi 2 H
16 1 01/29/2018 41 1216 2 10.2 0 54
2 multi 2 H
16 1 01/30/2018 42 1217 2 10.2 0 62.02
2 multi 2 H
16 1 01/31/2018 43 1218 2 10.3 0 61.55
2 multi 2 H
16 1 02/01/2018 44 1219 2 10.2 0 49.66

2 multi	2	H						
16	1	02/02/2018	45	1220	2	10	0.05	60.32
2 multi	2	H						
16	1	02/03/2018	46	1221	2	9.8	0	50.91
2 multi	2	H						
16	1	02/04/2018	47	1222	2	9.9	0	50.3
2 multi	2	H						
16	1	02/05/2018	48	1223	2	9.9	0.42	56.49
2 multi	2	H						
16	1	02/06/2018	49	1224	2	9.8	0	50.27
2 multi	2	H						
16	1	02/07/2018	50	1225	2	10.3	0	55.05
2 multi	2	H						
16	1	02/08/2018	51	1226	2	9.9	0	49.23
2 multi	2	H						
16	1	02/09/2018	52	1227	2	10.4	0	53.39
2 multi	2	H						
16	1	02/10/2018	53	1228	2	10.2	0	46.62
2 multi	2	H						
16	1	02/11/2018	54	1229	2	10	0	49.81
2 multi	2	H						
16	1	02/12/2018	55	1230	2	9.8	0	46.33
2 multi	2	H						
16	1	02/13/2018	56	1231	2	9.6	0	54.65
2 multi	2	H						
16	1	02/14/2018	57	1232	2	9.8	0.47	52.34
2 multi	2	H						
16	1	02/15/2018	58	1233	2	9.6	0.43	53.97
2 multi	2	H						
16	1	02/16/2018	59	1234	2	9.9	0.12	62.96
2 multi	2	H						
16	1	02/17/2018	60	1235	2	10.3	0	61.29
2 multi	2	H						
16	1	02/18/2018	61	1236	2	9.9	0	60.21
2 multi	2	H						
16	1	02/19/2018	62	1237	2	10	0.56	64.82
2 multi	2	H						
16	1	02/20/2018	63	1238	2	9.8	0	47.16
2 multi	2	H						
16	1	02/21/2018	64	1239	2	10.3	0	45.1
2 multi	2	H						
16	1	02/22/2018	65	1240	2	10.4	0	48.91
2 multi	2	H						
16	1	02/23/2018	66	1241	2	10	0	47.26
2 multi	2	H						
16	1	02/24/2018	67	1242	2	10	0	51.07
2 multi	2	H						
16	1	02/25/2018	68	1243	2	10.2	0	56.68
2 multi	2	H						
16	1	02/26/2018	69	1244	2	10.6	0	49.3
2 multi	2	H						
16	1	02/27/2018	70	1245	2	9.8	0	50.19
2 multi	2	H						
16	1	02/28/2018	71	1246	2	9.8	0	51.18

2 multi	2	H						
16	1	03/01/2018	72	1247	2	9.5	0	45.72
2 multi	2	H						
16	1	03/02/2018	73	1248	2	9.6	0	48.4
2 multi	2	H						
16	1	03/03/2018	74	1249	2	9.6	0	49.04
2 multi	2	H						
16	1	03/04/2018	75	1250	2	10.6	0	48.47
2 multi	2	H						
16	1	03/05/2018	76	1251	2	11.1	0.53	50.71
2 multi	2	H						
16	1	03/06/2018	77	1252	2	10.6	0	50.67
2 multi	2	H						
16	1	03/07/2018	78	1253	2	10.3	0.08	57.63
2 multi	2	H						
16	1	03/08/2018	79	1254	2	10	0.27	47.62
2 multi	2	H						
16	1	03/09/2018	80	1255	2	10.2	0.26	54.65
2 multi	2	H						
16	1	03/10/2018	81	1256	2	10.2	0.14	60.16
2 multi	2	H						
16	1	03/11/2018	82	1257	2	10.2	0.07	51.14
2 multi	2	H						
16	1	03/12/2018	83	1258	2	10.6	0.66	46.54
2 multi	2	H						
16	1	03/13/2018	84	1259	2	10.7	0	45.8
2 multi	2	H						
16	1	03/14/2018	85	1260	2	10.6	0	48.51
2 multi	2	H						
16	1	03/15/2018	86	1261	2	9.8	0.13	49.78
2 multi	2	H						
16	1	03/16/2018	87	1262	2	10.2	0	53.84
2 multi	2	H						
16	1	03/17/2018	88	1263	2	9.5	0	54.65
2 multi	2	H						
16	1	03/18/2018	89	1264	2	9.5	0	58.83
2 multi	2	H						
16	1	08/31/2018	255	1430	2	11.1	0.14	39.83
2 multi	2	H						
16	1	09/01/2018	256	1431	2	10.2	0	41.01
2 multi	2	H						
16	1	09/02/2018	257	1432	2	11.4	0.03	41.38
2 multi	2	H						
16	1	09/03/2018	258	1433	2	11.4	0	42.74
2 multi	2	H						
16	1	09/04/2018	259	1434	2	11.1	0	42.47
2 multi	2	H						
16	1	09/05/2018	260	1435	2	12.5	0	40.79
2 multi	2	H						
16	1	09/06/2018	261	1436	2	12.5	0.53	36.13
2 multi	2	H						
16	1	09/07/2018	262	1437	2	10.9	0.64	40.99
2 multi	2	H						
16	1	09/08/2018	263	1438	2	11.3	0.5	37.7

2 multi 2		H						
16	1	09/09/2018	264	1439	2	11.9	0	36.95
2 multi 2		H						
16	1	09/10/2018	265	1440	2	11.4	0	37.01
2 multi 2		H						
16	1	09/11/2018	266	1441	2	11.7	0	39.15
2 multi 2		H						
16	1	09/12/2018	267	1442	2	10.7	0.34	37.88
2 multi 2		H						
16	1	09/13/2018	268	1443	2	12.9	0.08	37.76
2 multi 2		H						
16	1	09/14/2018	269	1444	2	11.7	0	37.16
2 multi 2		H						
16	1	09/15/2018	270	1445	2	10.7	0.6	38.02
2 multi 2		H						
16	1	09/16/2018	271	1446	2	10.3	0	41.89
2 multi 2		H						
16	1	09/17/2018	272	1447	2	10.9	0.34	38.76
2 multi 2		H						
16	1	09/18/2018	273	1448	2	11.7	0.1	37.81
2 multi 2		H						
16	1	09/19/2018	274	1449	2	11.1	0	40.45
2 multi 2		H						
16	1	09/20/2018	275	1450	2	11.1	0	38.67
2 multi 2		H						
16	1	09/21/2018	276	1451	2	11.3	0.04	38.48
2 multi 2		H						
16	1	09/22/2018	277	1452	2	10.6	0.38	39.94
2 multi 2		H						
16	1	09/23/2018	278	1453	2	11.3	0	38.88
2 multi 2		H						
16	1	09/24/2018	279	1454	2	10.4	0	40.7
2 multi 2		H						
16	1	09/25/2018	280	1455	2	10	0	33.26
2 multi 2		H						
16	1	09/26/2018	281	1456	2	12.5	0	38.99
2 multi 2		H						
16	1	09/27/2018	282	1457	2	10.9	0.57	39.94
2 multi 2		H						
16	1	09/28/2018	283	1458	2	10.4	0.11	35.64
2 multi 2		H						
16	1	09/29/2018	284	1459	2	10.3	0	40.52
2 multi 2		H						
16	1	09/30/2018	285	1460	2	10	0.3	38.3
2 multi 2		H						
16	1	10/01/2018	286	1461	2	10.2	0.59	35.19
2 multi 2		H						
16	1	10/02/2018	287	1462	2	11.9	0	37.16
2 multi 2		H						
16	1	10/03/2018	288	1463	2	11.3	0	40.19
2 multi 2		H						
16	1	10/04/2018	289	1464	2	11.4	0	38.19
2 multi 2		H						
16	1	10/05/2018	290	1465	2	11.1	0	41.07

2 multi	2	H						
16	1	10/06/2018	291	1466	2	10.9	0	36.39
2 multi	2	H						
16	1	10/07/2018	292	1467	2	13.1	0.41	39.66
2 multi	2	H						
16	1	10/08/2018	293	1468	2	12.5	0	36.6
2 multi	2	H						
16	1	10/09/2018	294	1469	2	10.4	0	37.16
2 multi	2	H						
16	1	10/10/2018	295	1470	2	10.6	0	36.95
2 multi	2	H						
16	1	10/11/2018	296	1471	2	0	0.15	36.9
2 multi	2	H						
16	1	10/12/2018	297	1472	2	11.1	0.5	43.6
2 multi	2	H						
16	1	10/13/2018	298	1473	2	10.4	0	32.68
2 multi	2	H						
16	1	10/14/2018	299	1474	2	10.6	0.21	37.92
2 multi	2	H						
16	1	03/06/2019	2	1617	3	9.8	0.54	28.36
2 multi	1	H						
16	1	03/07/2019	3	1618	3	9.3	0.14	33.41
2 multi	1	H						
16	1	03/08/2019	4	1619	3	9.2	0.94	33.39
2 multi	1	H						
16	1	03/09/2019	5	1620	3	9.1	0.18	33.43
2 multi	1	H						
16	1	03/10/2019	6	1621	3	9.5	0.02	35.42
2 multi	1	H						
16	1	03/11/2019	7	1622	3	9.2	0.35	39.49
2 multi	1	H						
16	1	03/12/2019	8	1623	3	9.5	0.4	41.71
2 multi	1	H						
16	1	03/13/2019	9	1624	3	8.8	0	38.08
2 multi	1	H						
16	1	03/14/2019	10	1625	3	9.1	0.12	42.72
2 multi	1	H						
16	1	03/15/2019	11	1626	3	9.3	0	45.39
2 multi	1	H						
16	1	03/16/2019	12	1627	3	9.2	0	48.52
2 multi	1	H						
16	1	03/17/2019	13	1628	3	9.6	0	46.05
2 multi	1	H						
16	1	03/18/2019	14	1629	3	9.5	0.17	48.21
2 multi	1	H						
16	1	03/19/2019	15	1630	3	9.3	0	50.63
2 multi	1	H						
16	1	03/20/2019	16	1631	3	9.3	0	50.95
2 multi	1	H						
16	1	03/21/2019	17	1632	3	9.5	0	50.86
2 multi	1	H						
16	1	03/22/2019	18	1633	3	9.3	0.05	51.64
2 multi	1	H						
16	1	03/23/2019	19	1634	3	9.5	0	53.79

2 multi	1	H						
16	1	03/24/2019	20	1635	3	9.8	0.54	50.69
2 multi	1	H						
16	1	03/25/2019	21	1636	3	9.3	0.07	55.51
2 multi	1	H						
16	1	03/26/2019	22	1637	3	9.3	0	56.36
2 multi	2	H						
16	1	03/27/2019	23	1638	3	9.5	0	51.56
2 multi	2	H						
16	1	09/13/2019	193	1808	3	10.2	0.01	37.77
2 multi	2	H						
16	1	09/14/2019	194	1809	3	9.9	0.26	40.52
2 multi	2	H						
16	1	09/15/2019	195	1810	3	10.7	0	38.44
2 multi	2	H						
16	1	09/16/2019	196	1811	3	10.7	0.37	35.8
2 multi	2	H						
16	1	09/17/2019	197	1812	3	11.1	0	32.91
2 multi	2	H						
16	1	09/18/2019	198	1813	3	10.3	0.15	41.15
2 multi	2	H						
16	1	09/19/2019	199	1814	3	10.3	0.09	40.1
2 multi	2	H						
16	1	09/20/2019	200	1815	3	10.3	0.25	40.36
2 multi	2	H						
16	1	09/21/2019	201	1816	3	10.7	0.36	40.72
2 multi	2	H						
16	1	09/22/2019	202	1817	3	10.9	0.18	42.58
2 multi	2	H						
16	1	09/23/2019	203	1818	3	10.4	0.64	36.47
2 multi	2	H						
16	1	09/24/2019	204	1819	3	10.6	0.2	32.41
2 multi	2	H						
16	1	09/25/2019	205	1820	3	10.4	0.34	39.71
2 multi	2	H						
16	1	09/26/2019	206	1821	3	10	0	37.22
2 multi	2	H						
16	1	09/27/2019	207	1822	3	11.1	0.17	28.92
2 multi	2	H						
16	1	09/28/2019	208	1823	3	10.9	0.02	33.85
2 multi	2	H						
16	1	09/29/2019	209	1824	3	10.7	0	33.24
2 multi	2	H						
16	1	09/30/2019	210	1825	3	9.9	0.66	36.14
2 multi	2	H						
16	1	10/01/2019	211	1826	3	10.3	0	34.35
2 multi	2	H						
16	1	10/02/2019	212	1827	3	10.6	0.03	33.37
2 multi	2	H						
16	1	10/03/2019	213	1828	3	10.4	0.02	41.19
2 multi	2	H						
16	1	10/04/2019	214	1829	3	11.4	0	30.99
2 multi	2	H						
16	1	10/05/2019	215	1830	3	10	0	32.13

2 multi 2		H						
16	1	10/06/2019	216	1831	3	10.9	0.01	36.06
2 multi 2		H						
16	1	10/07/2019	217	1832	3	10.7	0.01	37.82
2 multi 2		H						
16	1	10/08/2019	218	1833	3	10.6	0.03	40.08
2 multi 2		H						
16	1	10/09/2019	219	1834	3	10.3	0	21.35
2 multi 2		H						
16	1	10/10/2019	220	1835	3	10.4	0.66	40.96
2 multi 2		H						
16	1	10/11/2019	221	1836	3	11.7	0.46	40.13
2 multi 2		H						
16	1	10/12/2019	222	1837	3	10.9	0	41.11
2 multi 2		H						
16	1	10/13/2019	223	1838	3	10.2	0.2	40.81
2 multi 2		H						
16	1	10/14/2019	224	1839	3	10	0	40.53
2 multi 2		H						
16	1	10/15/2019	225	1840	3	10.4	0	40.38
2 multi 2		H						
16	1	10/16/2019	226	1841	3	11.3	0.47	38.81
2 multi 2		H						
16	1	10/17/2019	227	1842	3	10.9	0.13	38.1
2 multi 2		H						
16	1	10/18/2019	228	1843	3	10.3	0	36.4
2 multi 2		H						
16	1	10/19/2019	229	1844	3	10.4	0.01	36.96
2 multi 2		H						
16	1	10/20/2019	230	1845	3	11.7	0.24	36.2
2 multi 2		H						
16	1	10/21/2019	231	1846	3	11.3	0.01	38.84
2 multi 2		H						
16	1	10/22/2019	232	1847	3	10.7	0	37.98
2 multi 2		H						
16	1	10/23/2019	233	1848	3	10.4	0.21	36.02
2 multi 2		H						
16	1	10/24/2019	234	1849	3	10.3	0	36.82
2 multi 2		H						
16	1	10/25/2019	235	1850	3	10	0	35.13
2 multi 2		H						
16	1	10/26/2019	236	1851	3	10.3	0.28	38.85
2 multi 2		H						
16	1	10/27/2019	237	1852	3	10.2	0	35.08
2 multi 2		H						
16	1	10/28/2019	238	1853	3	9.8	0	33.58
2 multi 2		H						
16	1	10/29/2019	239	1854	3	10.4	0.01	37.09
2 multi 2		H						
16	1	10/30/2019	240	1855	3	10.9	0.35	34.9
2 multi 2		H						
16	1	10/31/2019	241	1856	3	10.3	0.31	36.83
2 multi 2		H						
16	1	11/01/2019	242	1857	3	10.3	0.02	36.4

2 multi	2	H						
16	1	11/02/2019	243	1858	3	10.6	0	39.96
2 multi	2	H						
16	1	11/03/2019	244	1859	3	11.7	0.03	32.66
2 multi	2	H						
16	1	11/04/2019	245	1860	3	11.9	0.18	34.93
2 multi	2	H						
16	1	11/05/2019	246	1861	3	10.9	0	38.49
2 multi	2	H						
16	1	11/06/2019	247	1862	3	10.7	0.01	37.01
2 multi	2	H						
16	1	10/19/2020	4	2210	4	10	0.25	27.32
2 multi	1	H						
16	1	10/20/2020	5	2211	4	10.3	0.04	31.71
2 multi	1	H						
17	1	09/28/2018	7	1215	2	10	0.23	34.16
2 multi	1	H						
17	1	09/29/2018	8	1216	2	10	0.07	38.24
2 multi	1	H						
17	1	09/30/2018	9	1217	2	9.3	0.09	34.08
2 multi	1	H						
17	1	10/01/2018	10	1218	2	10	0.07	38.96
2 multi	1	H						
17	1	10/02/2018	11	1219	2	9.8	0.16	37.15
2 multi	1	H						
17	1	10/03/2018	12	1220	2	9.5	0.31	33.23
2 multi	1	H						
17	1	10/04/2018	13	1221	2	9.9	0.06	38.89
2 multi	1	H						
17	1	10/05/2018	14	1222	2	9.9	0.19	39.58
2 multi	1	H						
17	1	10/06/2018	15	1223	2	9.9	0.13	38.68
2 multi	1	H						
17	1	10/07/2018	16	1224	2	9.5	0.14	42.18
2 multi	1	H						
17	1	10/08/2018	17	1225	2	9.6	0.41	39.83
2 multi	1	H						
17	1	10/09/2018	18	1226	2	9.6	0.11	41.04
2 multi	1	H						
17	1	10/10/2018	19	1227	2	9.5	0.01	42.44
2 multi	1	H						
17	1	10/11/2018	20	1228	2	9.5	0.15	41.1
2 multi	1	H						
17	1	10/12/2018	21	1229	2	9.3	0.12	41.67
2 multi	1	H						
17	1	10/13/2018	22	1230	2	9.1	0	41.68
2 multi	2	H						
17	1	10/14/2018	23	1231	2	9.2	0.04	38.95
2 multi	2	H						
17	1	10/15/2018	24	1232	2	9.5	0.14	42.83
2 multi	2	H						
17	1	10/16/2018	25	1233	2	9.5	0	41.26
2 multi	2	H						
17	1	10/17/2018	26	1234	2	9.5	0.61	45.58

2 multi 2		H						
17	1	10/18/2018	27	1235	2	9.3	0.12	38.57
2 multi 2		H						
17	1	10/19/2018	28	1236	2	9.5	0.09	44.75
2 multi 2		H						
17	1	10/20/2018	29	1237	2	9.2	0.12	39.87
2 multi 2		H						
17	1	10/21/2018	30	1238	2	9.2	0	42.35
2 multi 2		H						
17	1	10/22/2018	31	1239	2	8.6	0.48	36.04
2 multi 2		H						
17	1	10/23/2018	32	1240	2	9.1	0	40.39
2 multi 2		H						
17	1	10/24/2018	33	1241	2	9.5	0	45.08
2 multi 2		H						
17	1	10/25/2018	34	1242	2	10	0	46.02
2 multi 2		H						
17	1	10/26/2018	35	1243	2	8.9	0.69	43.73
2 multi 2		H						
17	1	10/27/2018	36	1244	2	8.9	0.28	45.22
2 multi 2		H						
17	1	10/28/2018	37	1245	2	9.2	0.46	48.5
2 multi 2		H						
17	1	10/29/2018	38	1246	2	8.9	0	43.4
2 multi 2		H						
17	1	10/30/2018	39	1247	2	9.6	0.08	46.73
2 multi 2		H						
17	1	10/31/2018	40	1248	2	9.5	0	48.21
2 multi 2		H						
17	1	11/01/2018	41	1249	2	9.5	0.09	48
2 multi 2		H						
17	1	11/02/2018	42	1250	2	9.5	0.1	45.07
2 multi 2		H						
17	1	11/03/2018	43	1251	2	9.5	0.1	47.46
2 multi 2		H						
17	1	11/04/2018	44	1252	2	9.6	0	42.99
2 multi 2		H						
17	1	11/05/2018	45	1253	2	9.8	0	44.13
2 multi 2		H						
17	1	11/06/2018	46	1254	2	9.2	0.07	44.07
2 multi 2		H						
17	1	11/07/2018	47	1255	2	9.8	0.11	47.51
2 multi 2		H						
17	1	11/08/2018	48	1256	2	10	0	48.9
2 multi 2		H						
17	1	11/09/2018	49	1257	2	9.5	0.1	46.83
2 multi 2		H						
17	1	11/10/2018	50	1258	2	9.3	0	47.64
2 multi 2		H						
17	1	11/11/2018	51	1259	2	9.5	0.1	45.95
2 multi 2		H						
17	1	11/12/2018	52	1260	2	9.8	0	47.56
2 multi 2		H						
17	1	11/13/2018	53	1261	2	9.2	0.11	47.18

2 multi 2		H						
17	1	11/14/2018	54	1262	2	9.2	0.1	47.09
2 multi 2		H						
17	1	11/15/2018	55	1263	2	9.2	0	48.95
2 multi 2		H						
17	1	11/16/2018	56	1264	2	9.5	0	47.31
2 multi 2		H						
17	1	11/17/2018	57	1265	2	9.6	0.08	46
2 multi 2		H						
17	1	11/18/2018	58	1266	2	9.3	0	44.28
2 multi 2		H						
17	1	11/19/2018	59	1267	2	9.2	0.26	46.76
2 multi 2		H						
17	1	11/20/2018	60	1268	2	8.9	0	45.89
2 multi 2		H						
17	1	11/21/2018	61	1269	2	9.1	0.1	44.33
2 multi 2		H						
17	1	11/22/2018	62	1270	2	9.8	0.11	43.26
2 multi 2		H						
17	1	11/23/2018	63	1271	2	9.2	0.16	46.35
2 multi 2		H						
17	1	11/24/2018	64	1272	2	9.5	0.1	47.14
2 multi 2		H						
17	1	11/25/2018	65	1273	2	9.1	0.16	43.17
2 multi 2		H						
17	1	11/26/2018	66	1274	2	9.9	0.1	46.07
2 multi 2		H						
17	1	11/27/2018	67	1275	2	9.3	0.23	38.9
2 multi 2		H						
17	1	11/28/2018	68	1276	2	10	0.02	30.67
2 multi 2		H						
17	1	11/29/2018	69	1277	2	9.6	0.17	27.43
2 multi 2		H						
17	1	11/30/2018	70	1278	2	9.1	0	34.44
2 multi 2		H						
17	1	12/01/2018	71	1279	2	9.2	0	35.74
2 multi 2		H						
17	1	12/02/2018	72	1280	2	9.6	0.09	39.57
2 multi 2		H						
17	1	12/03/2018	73	1281	2	9.9	0	39.93
2 multi 2		H						
17	1	12/04/2018	74	1282	2	9.8	0.09	39.76
2 multi 2		H						
17	1	12/05/2018	75	1283	2	10	0.01	39.34
2 multi 2		H						
17	1	03/28/2019	188	1396	2	10.6	0.11	28.91
2 multi 2		H						
17	1	03/29/2019	189	1397	2	10.4	0	32.43
2 multi 2		H						
17	1	03/30/2019	190	1398	2	9.3	0	36.15
2 multi 2		H						
17	1	03/31/2019	191	1399	2	9.3	0	32.79
2 multi 2		H						
17	1	04/01/2019	192	1400	2	10.6	0	36.37

2 multi	2	H						
17	1	04/02/2019	193	1401	2	10.4	0.28	31.09
2 multi	2	H						
17	1	04/03/2019	194	1402	2	10.4	0.01	30.55
2 multi	2	H						
17	1	04/04/2019	195	1403	2	10.3	0.1	30.5
2 multi	2	H						
17	1	04/05/2019	196	1404	2	10.6	0.01	30.43
2 multi	2	H						
17	1	04/06/2019	197	1405	2	10.9	0	32.53
2 multi	2	H						
17	1	04/07/2019	198	1406	2	10.3	0.32	34.76
2 multi	2	H						
17	1	04/08/2019	199	1407	2	10.9	0.11	33.7
2 multi	2	H						
17	1	04/09/2019	200	1408	2	11.1	0.01	33.37
2 multi	2	H						
17	1	04/10/2019	201	1409	2	10.3	0	32.69
2 multi	2	H						
17	1	04/11/2019	202	1410	2	10.6	0.1	29.67
2 multi	2	H						
17	1	04/12/2019	203	1411	2	10	0.28	29.85
2 multi	2	H						
17	1	04/13/2019	204	1412	2	9.6	0.11	32.75
2 multi	2	H						
17	1	04/14/2019	205	1413	2	9.3	0	29.03
2 multi	2	H						
17	1	04/15/2019	206	1414	2	10.7	0	32.63
2 multi	2	H						
17	1	04/16/2019	207	1415	2	10.9	0.09	31.54
2 multi	2	H						
17	1	04/17/2019	208	1416	2	10.2	0	32.45
2 multi	2	H						
17	1	11/05/2019	4	1618	3	10.2	0.37	28.95
2 multi	1	H						
17	1	11/06/2019	5	1619	3	10.4	0.26	31.63
2 multi	1	H						
17	1	11/07/2019	6	1620	3	10.4	0.09	31.97
2 multi	1	H						
17	1	11/08/2019	7	1621	3	10.6	0.12	37.26
2 multi	1	H						
17	1	11/09/2019	8	1622	3	9.6	0.08	35.73
2 multi	1	H						
17	1	11/10/2019	9	1623	3	9.9	0	38.35
2 multi	1	H						
17	1	11/11/2019	10	1624	3	10	0	37.94
2 multi	1	H						
17	1	11/12/2019	11	1625	3	9.6	0	41.36
2 multi	1	H						
17	1	11/13/2019	12	1626	3	9.5	0.15	38.9
2 multi	1	H						
17	1	11/14/2019	13	1627	3	9.8	0.41	42.78
2 multi	1	H						
17	1	11/15/2019	14	1628	3	9.6	0.02	41.82

2 multi	1	H						
17	1	11/16/2019	15	1629	3	9.5	0.06	45.77
2 multi	1	H						
17	1	11/17/2019	16	1630	3	9.5	0.05	42.74
2 multi	1	H						
17	1	11/18/2019	17	1631	3	9.3	0.16	45.81
2 multi	1	H						
17	1	11/19/2019	18	1632	3	9.2	0.18	43.82
2 multi	1	H						
17	1	11/20/2019	19	1633	3	9.3	0.15	47.24
2 multi	1	H						
17	1	11/21/2019	20	1634	3	9.6	0.07	44.97
2 multi	1	H						
17	1	11/22/2019	21	1635	3	9.6	0.41	46.4
2 multi	1	H						
17	1	11/23/2019	22	1636	3	9.5	0.04	46.76
2 multi	2	H						
17	1	11/24/2019	23	1637	3	9.5	0.07	47.64
2 multi	2	H						
17	1	11/25/2019	24	1638	3	9.6	0.07	47.87
2 multi	2	H						
17	1	11/26/2019	25	1639	3	9.6	0.06	48.14
2 multi	2	H						
17	1	11/27/2019	26	1640	3	9.6	0.08	48.21
2 multi	2	H						
17	1	11/28/2019	27	1641	3	9.5	0.01	49.69
2 multi	2	H						
17	1	11/29/2019	28	1642	3	9.6	0.17	47.84
2 multi	2	H						
17	1	11/30/2019	29	1643	3	9.6	0.03	48.99
2 multi	2	H						
17	1	12/01/2019	30	1644	3	9.6	0.19	46.16
2 multi	2	H						
17	1	12/02/2019	31	1645	3	9.3	0.14	47.35
2 multi	2	H						
17	1	12/03/2019	32	1646	3	9.3	0.05	49.08
2 multi	2	H						
17	1	12/04/2019	33	1647	3	9.3	0.3	50.71
2 multi	2	H						
17	1	12/05/2019	34	1648	3	9.3	0	48.29
2 multi	2	H						
17	1	12/06/2019	35	1649	3	9.2	0.44	50.66
2 multi	2	H						
17	1	12/07/2019	36	1650	3	9.3	0.13	49.46
2 multi	2	H						
17	1	12/08/2019	37	1651	3	9.3	0.26	49.08
2 multi	2	H						
17	1	12/09/2019	38	1652	3	9.9	0.05	50.51
2 multi	2	H						
17	1	12/10/2019	39	1653	3	9.5	0.13	49.39
2 multi	2	H						
17	1	12/11/2019	40	1654	3	9.5	0	48.12
2 multi	2	H						
17	1	12/12/2019	41	1655	3	9.5	0.19	50.17

2 multi	2	H						
17	1	12/13/2019	42	1656	3	9.5	0.1	49.92
2 multi	2	H						
17	1	12/14/2019	43	1657	3	9.8	0.13	49.95
2 multi	2	H						
17	1	12/15/2019	44	1658	3	9.5	0.04	51.71
2 multi	2	H						
17	1	12/16/2019	45	1659	3	9.3	0.26	49.06
2 multi	2	H						
17	1	12/17/2019	46	1660	3	9.6	0.01	48.88
2 multi	2	H						
17	1	12/18/2019	47	1661	3	8.9	0.12	46.74
2 multi	2	H						
17	1	12/19/2019	48	1662	3	9.5	0.11	48.8
2 multi	2	H						
17	1	12/20/2019	49	1663	3	9.6	0.5	49.04
2 multi	2	H						
17	1	12/21/2019	50	1664	3	9.5	0.13	47.51
2 multi	2	H						
17	1	12/22/2019	51	1665	3	9.2	0.04	50.09
2 multi	2	H						
17	1	12/23/2019	52	1666	3	9.3	0.36	49.27
2 multi	2	H						
17	1	12/24/2019	53	1667	3	9.3	0.24	49.68
2 multi	2	H						
17	1	12/25/2019	54	1668	3	9.8	0.11	49.08
2 multi	2	H						
17	1	12/26/2019	55	1669	3	9.3	0.17	48.59
2 multi	2	H						
17	1	12/27/2019	56	1670	3	9.5	0.01	47.24
2 multi	2	H						
17	1	12/28/2019	57	1671	3	9.5	0.12	48.49
2 multi	2	H						
17	1	12/29/2019	58	1672	3	9.8	0.14	44.56
2 multi	2	H						
17	1	12/30/2019	59	1673	3	9.9	0.22	44.81
2 multi	2	H						
17	1	12/31/2019	60	1674	3	9.8	0.02	45.41
2 multi	2	H						
17	1	01/01/2020	61	1675	3	9.5	0.41	46.26
2 multi	2	H						
17	1	01/02/2020	62	1676	3	9.3	0.03	43.66
2 multi	2	H						
17	1	01/03/2020	63	1677	3	8.9	0.28	44.37
2 multi	2	H						
17	1	01/04/2020	64	1678	3	9.2	0.07	44.8
2 multi	2	H						
17	1	01/05/2020	65	1679	3	9.5	0.42	47.71
2 multi	2	H						
17	1	01/06/2020	66	1680	3	9.8	0	45.82
2 multi	2	H						
17	1	01/07/2020	67	1681	3	9.8	0	46.6
2 multi	2	H						
17	1	01/08/2020	68	1682	3	10	0.39	48.96

2 multi	2	H						
17	1	01/09/2020	69	1683	3	9.6	0.02	46.76
2 multi	2	H						
17	1	01/10/2020	70	1684	3	9.9	0.34	49.64
2 multi	2	H						
17	1	01/11/2020	71	1685	3	10	0.02	49.46
2 multi	2	H						
17	1	01/12/2020	72	1686	3	9.6	0	48.54
2 multi	2	H						
17	1	01/13/2020	73	1687	3	9.6	0.62	46.72
2 multi	2	H						
17	1	01/14/2020	74	1688	3	10	0.53	49.25
2 multi	2	H						
17	1	01/15/2020	75	1689	3	9.6	0.6	50.86
2 multi	2	H						
17	1	01/16/2020	76	1690	3	10.2	0.1	46.49
2 multi	2	H						
17	1	01/17/2020	77	1691	3	9.6	0.19	49.53
2 multi	2	H						
17	1	01/18/2020	78	1692	3	9.9	0.03	50.04
2 multi	2	H						
17	1	01/19/2020	79	1693	3	9.2	0	47.24
2 multi	2	H						
17	1	01/20/2020	80	1694	3	9.5	0.03	47.57
2 multi	2	H						
17	1	01/21/2020	81	1695	3	9.6	0.02	46.89
2 multi	2	H						
17	1	01/22/2020	82	1696	3	9.5	0.18	46.98
2 multi	2	H						
17	1	01/23/2020	83	1697	3	9.6	0.49	44.98
2 multi	2	H						
17	1	01/24/2020	84	1698	3	9.8	0.32	47.88
2 multi	2	H						
17	1	01/25/2020	85	1699	3	9.9	0	48.57
2 multi	2	H						
17	1	01/26/2020	86	1700	3	9.5	0.52	46.77
2 multi	2	H						
17	1	01/27/2020	87	1701	3	9.8	0.02	50.23
2 multi	2	H						
17	1	01/28/2020	88	1702	3	9.3	0.35	44.34
2 multi	2	H						
17	1	01/29/2020	89	1703	3	9.6	0	51.7
2 multi	2	H						
17	1	01/30/2020	90	1704	3	9.5	0.1	46.52
2 multi	2	H						
17	1	01/31/2020	91	1705	3	9.9	0.08	49.78
2 multi	2	H						
17	1	02/01/2020	92	1706	3	9.8	0	51.8
2 multi	2	H						
17	1	02/02/2020	93	1707	3	9.5	0.36	49.03
2 multi	2	H						
17	1	02/03/2020	94	1708	3	9.6	0.28	47.43
2 multi	2	H						
17	1	02/04/2020	95	1709	3	9.9	0.01	48.68

2 multi	2	H						
17	1	02/05/2020	96	1710	3	10	0.17	46.23
2 multi	2	H						
17	1	02/06/2020	97	1711	3	9.6	0.09	44.84
2 multi	2	H						
17	1	02/07/2020	98	1712	3	9.5	0.48	47.41
2 multi	2	H						
17	1	02/08/2020	99	1713	3	10	0.09	49.78
2 multi	2	H						
17	1	02/09/2020	100	1714	3	9.8	0.01	45.83
2 multi	2	H						
17	1	06/30/2020	242	1856	3	10.3	0	36.65
2 multi	2	H						
17	1	07/01/2020	243	1857	3	10.6	0	37.41
2 multi	2	H						
17	1	07/02/2020	244	1858	3	10	0	33.94
2 multi	2	H						
17	1	07/03/2020	245	1859	3	10.4	0.23	39.28
2 multi	2	H						
17	1	07/04/2020	246	1860	3	9.8	0.21	35.22
2 multi	2	H						
17	1	07/05/2020	247	1861	3	9.8	0.02	41.66
2 multi	2	H						
17	1	07/06/2020	248	1862	3	10.2	0.13	36.13
2 multi	2	H						
17	1	07/07/2020	249	1863	3	10.2	0.17	38.47
2 multi	2	H						
17	1	07/08/2020	250	1864	3	10.4	0	39.61
2 multi	2	H						
17	1	07/09/2020	251	1865	3	10.3	0.1	38.07
2 multi	2	H						
17	1	07/10/2020	252	1866	3	10.3	0.09	37.19
2 multi	2	H						
17	1	07/11/2020	253	1867	3	10.4	0.6	38.11
2 multi	2	H						
17	1	07/12/2020	254	1868	3	10	0.11	34.85
2 multi	2	H						
17	1	07/13/2020	255	1869	3	10	0	37.36
2 multi	2	H						
17	1	07/14/2020	256	1870	3	10.4	0.02	41.56
2 multi	2	H						
17	1	07/15/2020	257	1871	3	9.9	0.1	39.5
2 multi	2	H						
17	1	07/16/2020	258	1872	3	10	0.12	39.55
2 multi	2	H						
17	1	07/17/2020	259	1873	3	9.8	0.14	39.93
2 multi	2	H						
17	1	07/18/2020	260	1874	3	9.3	0.1	39.35
2 multi	2	H						
17	1	07/19/2020	261	1875	3	10	0.02	41.15
2 multi	2	H						
17	1	07/20/2020	262	1876	3	10.2	0	37.66
2 multi	2	H						
17	1	07/21/2020	263	1877	3	10.3	0.01	39.69

2 multi	2	H						
17	1	07/22/2020	264	1878	3	9.9	0.09	38.79
2 multi	2	H						
17	1	07/23/2020	265	1879	3	9.8	0.15	40.45
2 multi	2	H						
17	1	07/24/2020	266	1880	3	10	0.03	36.11
2 multi	2	H						
17	1	07/25/2020	267	1881	3	10	0.19	34.2
2 multi	2	H						
17	1	07/26/2020	268	1882	3	10.2	0.08	38.7
2 multi	2	H						
17	1	07/27/2020	269	1883	3	9.9	0.11	39.9
2 multi	2	H						
17	1	07/28/2020	270	1884	3	10.2	0.04	36.43
2 multi	2	H						
17	1	07/29/2020	271	1885	3	10.3	0	38.12
2 multi	2	H						
17	1	07/30/2020	272	1886	3	10	0.1	36.82
2 multi	2	H						
17	1	07/31/2020	273	1887	3	10.4	0.02	36.72
2 multi	2	H						
17	1	08/01/2020	274	1888	3	9.5	0.1	35.88
2 multi	2	H						
17	1	08/02/2020	275	1889	3	9.6	0.11	39.01
2 multi	2	H						
17	1	08/03/2020	276	1890	3	9.8	0.02	38.14
2 multi	2	H						
17	1	08/04/2020	277	1891	3	10.2	0.08	39.29
2 multi	2	H						
17	1	08/05/2020	278	1892	3	10.2	0.05	39.24
2 multi	2	H						
17	1	08/06/2020	279	1893	3	9.9	0.05	38.72
2 multi	2	H						
17	1	08/07/2020	280	1894	3	9.8	0.02	39.4
2 multi	2	H						
17	1	08/08/2020	281	1895	3	9.9	0.11	39.34
2 multi	2	H						
17	1	08/09/2020	282	1896	3	10.2	0	33.99
2 multi	2	H						
17	1	08/10/2020	283	1897	3	10	0.12	36.71
2 multi	2	H						
17	1	08/11/2020	284	1898	3	10	0.12	34.37
2 multi	2	H						
17	1	08/12/2020	285	1899	3	10.2	0	34.62
2 multi	2	H						
17	1	08/13/2020	286	1900	3	10.3	0	34.64
2 multi	2	H						
17	1	08/14/2020	287	1901	3	10.2	0.02	34.69
2 multi	2	H						
17	1	08/15/2020	288	1902	3	10.2	0.03	34.3
2 multi	2	H						
17	1	08/16/2020	289	1903	3	9.6	0.17	32.31
2 multi	2	H						
17	1	11/29/2020	7	2008	4	10.3	0.52	30.61

2 multi	1	H						
17	1	11/30/2020	8	2009	4	10.3	0.11	34.39
2 multi	1	H						
17	1	12/01/2020	9	2010	4	10.2	0.13	36.39
2 multi	1	H						
17	1	12/02/2020	10	2011	4	10	0.67	39.53
2 multi	1	H						
17	1	12/03/2020	11	2012	4	9.6	0.39	38.14
2 multi	1	H						
17	1	12/04/2020	12	2013	4	10.2	0.44	40.19
2 multi	1	H						
17	1	12/05/2020	13	2014	4	9.9	0.19	47.89
2 multi	1	H						
17	1	12/06/2020	14	2015	4	9.9	0.12	45.99
2 multi	1	H						
17	1	12/07/2020	15	2016	4	9.8	0.06	48.87
2 multi	1	H						
17	1	12/08/2020	16	2017	4	10	0.21	48.88
2 multi	1	H						
17	1	12/09/2020	17	2018	4	9.9	0.45	52.39
2 multi	1	H						
17	1	12/10/2020	18	2019	4	9.8	0.1	50.33
2 multi	1	H						
17	1	12/11/2020	19	2020	4	9.6	0.06	53.36
2 multi	1	H						
17	1	12/12/2020	20	2021	4	9.6	0.19	51.62
2 multi	1	H						
17	1	12/13/2020	21	2022	4	9.8	0.1	52.24
2 multi	1	H						
17	1	12/14/2020	22	2023	4	9.5	0.04	52.73
2 multi	2	H						
17	1	12/15/2020	23	2024	4	9.6	0	52.99
2 multi	2	H						
17	1	12/16/2020	24	2025	4	9.5	0	52.26
2 multi	2	H						
17	1	12/17/2020	25	2026	4	9.5	0.07	53.05
2 multi	2	H						
17	1	12/18/2020	26	2027	4	9.3	0.08	49.14
2 multi	2	H						
17	1	12/19/2020	27	2028	4	9.5	0.11	46.04
2 multi	2	H						
17	1	12/20/2020	28	2029	4	9.3	0.11	47.01
2 multi	2	H						
17	1	12/21/2020	29	2030	4	9.6	0.67	51.57
2 multi	2	H						
17	1	12/22/2020	30	2031	4	9.8	0.12	52.7
2 multi	2	H						
17	1	12/23/2020	31	2032	4	10	0.19	54.24
2 multi	2	H						
17	1	12/24/2020	32	2033	4	9.9	0.12	51.92
2 multi	2	H						
17	1	12/25/2020	33	2034	4	9.8	0.16	50.26
2 multi	2	H						
17	1	12/26/2020	34	2035	4	10	0.14	56.19

2 multi	2	H						
17	1	12/27/2020	35	2036	4	9.8	0.13	53.58
2 multi	2	H						
17	1	12/28/2020	36	2037	4	9.8	0.13	54.44
2 multi	2	H						
17	1	12/29/2020	37	2038	4	9.8	0.04	55.19
2 multi	2	H						
17	1	12/30/2020	38	2039	4	9.9	0.32	52.17
2 multi	2	H						
17	1	12/31/2020	39	2040	4	9.6	0.15	50.64
2 multi	2	H						
17	1	01/01/2021	40	2041	4	9.3	0.6	54.29
2 multi	2	H						
17	1	01/02/2021	41	2042	4	9.5	0.08	51.05
2 multi	2	H						
17	1	01/03/2021	42	2043	4	9.6	0.35	55.5
2 multi	2	H						
17	1	01/04/2021	43	2044	4	9.6	0.16	51.63
2 multi	2	H						
17	1	01/05/2021	44	2045	4	9.8	0.23	50.01
2 multi	2	H						
17	1	01/06/2021	45	2046	4	9.6	0.37	56.78
2 multi	2	H						
17	1	01/07/2021	46	2047	4	9.6	0.4	52.9
2 multi	2	H						
17	1	01/08/2021	47	2048	4	9.9	0.82	49.32
2 multi	2	H						
17	1	01/09/2021	48	2049	4	9.8	0.55	53.13
2 multi	2	H						
17	1	01/10/2021	49	2050	4	9.9	0.24	54.38
2 multi	2	H						
17	1	01/11/2021	50	2051	4	9.5	0.26	54.24
2 multi	2	H						
17	1	01/12/2021	51	2052	4	9.8	0.31	51.6
2 multi	2	H						
17	1	01/13/2021	52	2053	4	9.6	0.69	53.71
2 multi	2	H						
17	1	01/14/2021	53	2054	4	9.6	0.17	51.05
2 multi	2	H						
17	1	01/15/2021	54	2055	4	9.3	0.36	51.4
2 multi	2	H						
17	1	01/16/2021	55	2056	4	8.9	0.32	49.14
2 multi	2	H						
17	1	01/17/2021	56	2057	4	9.2	0.21	53.83
2 multi	2	H						
17	1	01/18/2021	57	2058	4	9.6	0.23	50.67
2 multi	2	H						
17	1	01/19/2021	58	2059	4	9.6	0.07	43.92
2 multi	2	H						
17	1	01/20/2021	59	2060	4	9.6	0.06	47.92
2 multi	2	H						
17	1	01/21/2021	60	2061	4	9.6	0.13	47.55
2 multi	2	H						
17	1	01/22/2021	61	2062	4	9.9	0.07	52.25

2 multi 2		H						
17	1	01/23/2021	62	2063	4	9.9	0.16	51.18
2 multi 2		H						
17	1	01/24/2021	63	2064	4	9.9	0.43	53.89
2 multi 2		H						
17	1	01/25/2021	64	2065	4	10	0.16	52.13
2 multi 2		H						
17	1	01/26/2021	65	2066	4	9.9	0.09	53.75
2 multi 2		H						
17	1	01/27/2021	66	2067	4	9.8	0.11	50.43
2 multi 2		H						
17	1	01/28/2021	67	2068	4	9.6	0.31	47.95
2 multi 2		H						
17	1	01/29/2021	68	2069	4	9.6	0.33	53.82
2 multi 2		H						
17	1	01/30/2021	69	2070	4	9.6	0.02	54.37
2 multi 2		H						
17	1	01/31/2021	70	2071	4	9.6	0.05	51.17
2 multi 2		H						
17	1	02/01/2021	71	2072	4	9.5	0.15	54.43
2 multi 2		H						
17	1	02/02/2021	72	2073	4	9.6	0.07	51.65
2 multi 2		H						
17	1	02/03/2021	73	2074	4	9.9	0.17	53.95
2 multi 2		H						
17	1	02/04/2021	74	2075	4	9.8	0.15	52.36
2 multi 2		H						
17	1	02/05/2021	75	2076	4	9.6	0.04	54.84
2 multi 2		H						
17	1	02/06/2021	76	2077	4	9.8	0.02	51.78
2 multi 2		H						
17	1	02/07/2021	77	2078	4	9.9	0.17	52.54
2 multi 2		H						
17	1	02/08/2021	78	2079	4	9.8	0.05	46.44
2 multi 2		H						
17	1	02/09/2021	79	2080	4	9.3	0.18	53.07
2 multi 2		H						
17	1	02/10/2021	80	2081	4	9.8	0.74	52.45
2 multi 2		H						
17	1	02/11/2021	81	2082	4	9.5	0.02	51.99
2 multi 2		H						
17	1	02/12/2021	82	2083	4	9.5	0.08	51.41
2 multi 2		H						
17	1	02/13/2021	83	2084	4	9.5	0.2	49.9
2 multi 2		H						
17	1	02/14/2021	84	2085	4	9.9	0.02	50.22
2 multi 2		H						
17	1	02/15/2021	85	2086	4	9.9	0.19	43.73
2 multi 2		H						
17	1	02/16/2021	86	2087	4	9.9	0.46	42.39
2 multi 2		H						
17	1	02/17/2021	87	2088	4	10.3	0.02	49.75
2 multi 2		H						
17	1	02/18/2021	88	2089	4	9.9	0.43	49.34

2 multi	2	H						
17	1	02/19/2021	89	2090	4	9.6	0.34	48.67
2 multi	2	H						
17	1	02/20/2021	90	2091	4	9.8	0.02	51.03
2 multi	2	H						
17	1	02/21/2021	91	2092	4	9.8	0.05	51.85
2 multi	2	H						
17	1	02/22/2021	92	2093	4	9.8	0.17	51.6
2 multi	2	H						
17	1	02/23/2021	93	2094	4	9.6	0.06	49.87
2 multi	2	H						
17	1	02/24/2021	94	2095	4	9.8	0.05	50.08
2 multi	2	H						
17	1	02/25/2021	95	2096	4	9.6	0.05	51.19
2 multi	2	H						
17	1	02/26/2021	96	2097	4	9.6	0.09	49.86
2 multi	2	H						
17	1	02/27/2021	97	2098	4	9.6	0.4	48.64
2 multi	2	H						
17	1	02/28/2021	98	2099	4	9.6	0.01	52.23
2 multi	2	H						
17	1	03/01/2021	99	2100	4	9.9	0.09	48.99
2 multi	2	H						
17	1	03/02/2021	100	2101	4	10	0.07	51.32
2 multi	2	H						
17	1	03/03/2021	101	2102	4	9.9	0.5	52.11
2 multi	2	H						
17	1	03/04/2021	102	2103	4	10	0.14	48.47
2 multi	2	H						
17	1	03/05/2021	103	2104	4	9.9	0.01	49.38
2 multi	2	H						
17	1	03/06/2021	104	2105	4	9.5	0.15	46.78
2 multi	2	H						
17	1	03/07/2021	105	2106	4	10	0.18	53.8
2 multi	2	H						
17	1	03/08/2021	106	2107	4	9.9	0.04	49.41
2 multi	2	H						
17	1	03/09/2021	107	2108	4	9.8	0.06	48.78
2 multi	2	H						
17	1	03/10/2021	108	2109	4	9.9	0.08	50.45
2 multi	2	H						
17	1	03/11/2021	109	2110	4	9.9	0.08	49.34
2 multi	2	H						
17	1	03/12/2021	110	2111	4	10.3	0.05	47.37
2 multi	2	H						
17	1	03/13/2021	111	2112	4	9.8	0.15	47.2
2 multi	2	H						
17	1	03/14/2021	112	2113	4	10	0.14	49.45
2 multi	2	H						
17	1	03/15/2021	113	2114	4	10	0.07	50.11
2 multi	2	H						
17	1	03/16/2021	114	2115	4	10	0.12	50.3
2 multi	2	H						
17	1	03/17/2021	115	2116	4	10.3	0.06	48.78

2 multi 2		H						
17	1	03/18/2021	116	2117	4	9.9	0.06	46.98
2 multi 2		H						
17	1	03/19/2021	117	2118	4	9.8	0.07	45.5
2 multi 2		H						
17	1	03/20/2021	118	2119	4	10	0.15	46.88
2 multi 2		H						
17	1	03/21/2021	119	2120	4	10	0.07	47.45
2 multi 2		H						
17	1	03/22/2021	120	2121	4	9.9	0	49.07
2 multi 2		H						
17	1	03/23/2021	121	2122	4	10.3	0.15	47.26
2 multi 2		H						
17	1	03/24/2021	122	2123	4	10.3	0.03	47.42
2 multi 2		H						
17	1	03/25/2021	123	2124	4	10.2	0.06	48.16
2 multi 2		H						
17	1	03/26/2021	124	2125	4	9.9	0.03	46.79
2 multi 2		H						
17	1	03/27/2021	125	2126	4	10.3	0.15	46.89
2 multi 2		H						
17	1	03/28/2021	126	2127	4	9.9	0.07	45.2
2 multi 2		H						
17	1	03/29/2021	127	2128	4	10.2	0.07	48.27
2 multi 2		H						
17	1	03/30/2021	128	2129	4	10	0.12	47.32
2 multi 2		H						
17	1	03/31/2021	129	2130	4	10.2	0.06	48.65
2 multi 2		H						
17	1	04/01/2021	130	2131	4	9.8	0.06	46.37
2 multi 2		H						
17	1	04/02/2021	131	2132	4	10	0	49.19
2 multi 2		H						
17	1	04/03/2021	132	2133	4	9.9	0.01	45.72
2 multi 2		H						
17	1	04/04/2021	133	2134	4	10	0.13	44.87
2 multi 2		H						
17	1	04/05/2021	134	2135	4	10.3	0	47.39
2 multi 2		H						
17	1	04/06/2021	135	2136	4	9.5	0.13	46.14
2 multi 2		H						
17	1	04/07/2021	136	2137	4	9.8	0.22	43.26
2 multi 2		H						
17	1	04/08/2021	137	2138	4	9.8	0.07	45.18
2 multi 2		H						
17	1	04/09/2021	138	2139	4	9.8	0.01	46.68
2 multi 2		H						
17	1	04/10/2021	139	2140	4	10	0.05	46.43
2 multi 2		H						
17	1	04/11/2021	140	2141	4	10.2	0.12	43.89
2 multi 2		H						
17	1	04/12/2021	141	2142	4	10.3	0	48.2
2 multi 2		H						
17	1	04/13/2021	142	2143	4	9.8	0.08	43.45

2 multi 2		H						
17	1	04/14/2021	143	2144	4	10	0.06	44.95
2 multi 2		H						
17	1	04/15/2021	144	2145	4	9.9	0.07	44.78
2 multi 2		H						
17	1	04/16/2021	145	2146	4	10.2	0.11	45.18
2 multi 2		H						
17	1	04/17/2021	146	2147	4	9.9	0.07	41.68
2 multi 2		H						
17	1	04/18/2021	147	2148	4	9.9	0.3	44.13
2 multi 2		H						
17	1	04/19/2021	148	2149	4	9.9	0.11	43.61
2 multi 2		H						
17	1	04/20/2021	149	2150	4	9.9	0.03	44.85
2 multi 2		H						
17	1	04/21/2021	150	2151	4	9.6	0.1	43.26
2 multi 2		H						
17	1	04/22/2021	151	2152	4	9.6	0.15	43.88
2 multi 2		H						
17	1	04/23/2021	152	2153	4	10.2	0.33	44.38
2 multi 2		H						
17	1	04/24/2021	153	2154	4	10	0.02	43.45
2 multi 2		H						
17	1	04/25/2021	154	2155	4	10	0.01	43.84
2 multi 2		H						
17	1	04/26/2021	155	2156	4	9.8	0	44.39
2 multi 2		H						
17	1	04/27/2021	156	2157	4	10	0	44.11
2 multi 2		H						
17	1	04/28/2021	157	2158	4	9.9	0.02	39.21
2 multi 2		H						
17	1	04/29/2021	158	2159	4	10	0.02	43.87
2 multi 2		H						
18	1	05/09/2018	11	746	1	10.4	0	17.91
1 primi 1		H						
18	1	05/10/2018	12	747	1	10.3	0	19.44
1 primi 1		H						
18	1	05/11/2018	13	748	1	10.4	0.02	22.54
1 primi 1		H						
18	1	05/12/2018	14	749	1	10.3	0	21.44
1 primi 1		H						
18	1	05/13/2018	15	750	1	10.2	0.07	22.42
1 primi 1		H						
18	1	05/14/2018	16	751	1	10.2	0.13	22.94
1 primi 1		H						
18	1	05/15/2018	17	752	1	10	0.1	23.45
1 primi 1		H						
18	1	05/16/2018	18	753	1	10.6	0.09	23.96
1 primi 1		H						
18	1	05/17/2018	19	754	1	10.2	0.4	23.06
1 primi 1		H						
18	1	05/18/2018	20	755	1	9.8	0	23.02
1 primi 1		H						
18	1	05/19/2018	21	756	1	9.9	0.12	24.93

1 primi 1	H
18	1	05/20/2018	22	757	1	10.2	0	25.36
1 primi 2	H
18	1	05/21/2018	23	758	1	10.2	0.31	24.82
1 primi 2	H
18	1	05/22/2018	24	759	1	10.2	0.02	26.01
1 primi 2	H
18	1	05/23/2018	25	760	1	10	0	26.49
1 primi 2	H
18	1	05/24/2018	26	761	1	10	0.18	26.45
1 primi 2	H
18	1	05/25/2018	27	762	1	10	0	26.53
1 primi 2	H
18	1	05/26/2018	28	763	1	9.9	0.04	24.01
1 primi 2	H
18	1	05/27/2018	29	764	1	10	0	25.81
1 primi 2	H
18	1	05/28/2018	30	765	1	10.3	0	28.6
1 primi 2	H
18	1	05/29/2018	31	766	1	9.9	0.1	27.5
1 primi 2	H
18	1	05/30/2018	32	767	1	9.5	0	26.44
1 primi 2	H
18	1	05/31/2018	33	768	1	10	0.05	26.87
1 primi 2	H
18	1	06/01/2018	34	769	1	10	0	28.37
1 primi 2	H
18	1	06/02/2018	35	770	1	10.4	0.09	28.03
1 primi 2	H
18	1	06/03/2018	36	771	1	10	0	26.41
1 primi 2	H
18	1	06/04/2018	37	772	1	10.2	0	27.02
1 primi 2	H
18	1	06/05/2018	38	773	1	10	0	26.9
1 primi 2	H
18	1	06/06/2018	39	774	1	10.2	0	28.17
1 primi 2	H
18	1	06/07/2018	40	775	1	10	0	28.87
1 primi 2	H
18	1	06/08/2018	41	776	1	9.9	0.23	29.39
1 primi 2	H
18	1	06/09/2018	42	777	1	9.8	0.26	29.53
1 primi 2	H
18	1	06/10/2018	43	778	1	9.5	0	29.1
1 primi 2	H
18	1	06/11/2018	44	779	1	9.3	0.28	29.51
1 primi 2	H
18	1	06/12/2018	45	780	1	10	0.07	28.28
1 primi 2	H
18	1	06/13/2018	46	781	1	9.8	0.19	28.95
1 primi 2	H
18	1	06/14/2018	47	782	1	9.8	0.03	29.8
1 primi 2	H
18	1	06/15/2018	48	783	1	9.3	0.14	29.46

1 primi	2	H						
18	1	06/16/2018	49	784	1	9.8	0.24	30.2
1 primi	2	H						
18	1	06/17/2018	50	785	1	9.6	0.09	30.4
1 primi	2	H						
18	1	05/03/2019	6	1105	2	10.4	0.22	32.47
2 multi	1	H						
18	1	05/04/2019	7	1106	2	10	0.15	33.53
2 multi	1	H						
18	1	05/05/2019	8	1107	2	10	0.57	34.5
2 multi	1	H						
18	1	05/06/2019	9	1108	2	9.8	0.26	34.83
2 multi	1	H						
18	1	05/07/2019	10	1109	2	10.2	0.22	34.57
2 multi	1	H						
18	1	05/08/2019	11	1110	2	9.9	0.26	34.32
2 multi	1	H						
18	1	05/09/2019	12	1111	2	10	0.14	36.06
2 multi	1	H						
18	1	05/10/2019	13	1112	2	10.4	0.17	34.99
2 multi	1	H						
18	1	05/11/2019	14	1113	2	10	0	38.44
2 multi	1	H						
18	1	05/12/2019	15	1114	2	9.6	0	31.69
2 multi	1	H						
18	1	05/13/2019	16	1115	2	9.8	0.22	36.82
2 multi	1	H						
18	1	05/14/2019	17	1116	2	9.8	0.28	36.83
2 multi	1	H						
18	1	05/15/2019	18	1117	2	10.2	0.28	36.6
2 multi	1	H						
18	1	05/16/2019	19	1118	2	10.2	0.23	36.93
2 multi	1	H						
18	1	05/17/2019	20	1119	2	9.9	0	37.72
2 multi	1	H						
18	1	05/18/2019	21	1120	2	9.9	0.26	36.28
2 multi	1	H						
18	1	05/19/2019	22	1121	2	9.9	0.28	34.85
2 multi	2	H						
18	1	05/20/2019	23	1122	2	10.2	0.04	34.63
2 multi	2	H						
18	1	05/21/2019	24	1123	2	10	0.14	38.91
2 multi	2	H						
18	1	05/22/2019	25	1124	2	9.9	0.25	34.29
2 multi	2	H						
18	1	05/23/2019	26	1125	2	9.8	0	40.57
2 multi	2	H						
18	1	05/24/2019	27	1126	2	10.2	0.27	33.88
2 multi	2	H						
18	1	05/25/2019	28	1127	2	10.3	0.08	38.2
2 multi	2	H						
18	1	05/26/2019	29	1128	2	10.2	0.22	39.36
2 multi	2	H						
18	1	05/27/2019	30	1129	2	10.9	0.22	35.59

2 multi	2	H						
18	1	05/28/2019	31	1130	2	10.4	0.09	39.81
2 multi	2	H						
18	1	05/29/2019	32	1131	2	10.4	0.2	37.95
2 multi	2	H						
18	1	05/30/2019	33	1132	2	10.4	0.16	41.43
2 multi	2	H						
18	1	05/31/2019	34	1133	2	10	0.19	40.27
2 multi	2	H						
18	1	06/01/2019	35	1134	2	10	0.33	41.86
2 multi	2	H						
18	1	06/02/2019	36	1135	2	9.8	0.2	41.08
2 multi	2	H						
18	1	06/03/2019	37	1136	2	9.9	0.27	39.65
2 multi	2	H						
18	1	06/04/2019	38	1137	2	10.3	0.15	41.39
2 multi	2	H						
18	1	06/05/2019	39	1138	2	10.3	0.15	40.45
2 multi	2	H						
18	1	06/06/2019	40	1139	2	10.2	0.11	39.73
2 multi	2	H						
18	1	06/07/2019	41	1140	2	10	0.13	41.85
2 multi	2	H						
18	1	06/08/2019	42	1141	2	10.2	0.16	39.62
2 multi	2	H						
18	1	06/09/2019	43	1142	2	10	0	42.36
2 multi	2	H						
18	1	06/10/2019	44	1143	2	10.4	0.04	39.25
2 multi	2	H						
18	1	06/11/2019	45	1144	2	10.4	0.26	41.08
2 multi	2	H						
18	1	06/12/2019	46	1145	2	10.2	0.12	40.45
2 multi	2	H						
18	1	06/13/2019	47	1146	2	10.2	0.2	40.42
2 multi	2	H						
18	1	06/14/2019	48	1147	2	10	0.24	38.71
2 multi	2	H						
18	1	06/15/2019	49	1148	2	10	0.24	38.09
2 multi	2	H						
18	1	06/16/2019	50	1149	2	10.6	0.3	39.11
2 multi	2	H						
18	1	06/17/2019	51	1150	2	10.3	0.08	40.37
2 multi	2	H						
18	1	06/18/2019	52	1151	2	10.4	0	40.07
2 multi	2	H						
18	1	06/19/2019	53	1152	2	10.2	0.1	42.16
2 multi	2	H						
18	1	06/20/2019	54	1153	2	10	0.16	39.75
2 multi	2	H						
18	1	06/21/2019	55	1154	2	10.4	0.68	40.1
2 multi	2	H						
18	1	06/22/2019	56	1155	2	10.3	0.18	39.09
2 multi	2	H						
18	1	06/23/2019	57	1156	2	10.3	0.12	40.32

2 multi	2	H						
18	1	06/24/2019	58	1157	2	9.9	0.02	42.97
2 multi	2	H						
18	1	06/25/2019	59	1158	2	10	0	41.83
2 multi	2	H						
18	1	06/26/2019	60	1159	2	9.9	0.02	44.04
2 multi	2	H						
18	1	06/27/2019	61	1160	2	10.3	0.15	43.61
2 multi	2	H						
18	1	06/28/2019	62	1161	2	10.3	0	40.31
2 multi	2	H						
18	1	06/29/2019	63	1162	2	10.3	0	40.99
2 multi	2	H						
18	1	06/30/2019	64	1163	2	10.2	0	39.43
2 multi	2	H						
18	1	07/01/2019	65	1164	2	10	0.1	39.81
2 multi	2	H						
18	1	07/02/2019	66	1165	2	10.3	0.46	41.09
2 multi	2	H						
18	1	07/03/2019	67	1166	2	10.2	0.28	40.82
2 multi	2	H						
18	1	07/04/2019	68	1167	2	10	0.33	40.87
2 multi	2	H						
18	1	07/05/2019	69	1168	2	10.3	0.19	41.9
2 multi	2	H						
18	1	07/06/2019	70	1169	2	10.2	0.18	40.37
2 multi	2	H						
18	1	07/07/2019	71	1170	2	10.6	0.17	42.57
2 multi	2	H						
18	1	07/08/2019	72	1171	2	10	0.15	40.9
2 multi	2	H						
18	1	07/09/2019	73	1172	2	9.9	0.2	40.08
2 multi	2	H						
18	1	07/10/2019	74	1173	2	9.8	0.06	37.21
2 multi	2	H						
18	1	07/11/2019	75	1174	2	9.9	0.21	39.46
2 multi	2	H						
18	1	07/12/2019	76	1175	2	10	0.4	36.66
2 multi	2	H						
18	1	07/13/2019	77	1176	2	10.3	0.23	38.62
2 multi	2	H						
18	1	07/14/2019	78	1177	2	10	0.05	41.67
2 multi	2	H						
18	1	07/15/2019	79	1178	2	10.2	0.13	41.78
2 multi	2	H						
18	1	07/16/2019	80	1179	2	9.9	0.01	40.9
2 multi	2	H						
18	1	07/17/2019	81	1180	2	9.9	0.05	39.55
2 multi	2	H						
18	1	07/18/2019	82	1181	2	10.3	0	39.8
2 multi	2	H						
18	1	07/19/2019	83	1182	2	10.3	0.2	41.06
2 multi	2	H						
18	1	07/20/2019	84	1183	2	10.2	0	42.44

2 multi	2	H						
18	1	07/21/2019	85	1184	2	10.2	0	40.01
2 multi	2	H						
18	1	07/22/2019	86	1185	2	9.9	0.22	39.71
2 multi	2	H						
18	1	07/23/2019	87	1186	2	10.6	0.15	41.62
2 multi	2	H						
18	1	07/24/2019	88	1187	2	10.4	0.04	41.91
2 multi	2	H						
18	1	07/25/2019	89	1188	2	9.9	0	42.14
2 multi	2	H						
18	1	07/26/2019	90	1189	2	10	0.07	40.29
2 multi	2	H						
18	1	07/27/2019	91	1190	2	9.9	0.22	39.98
2 multi	2	H						
18	1	07/28/2019	92	1191	2	10.6	0.11	38.16
2 multi	2	H						
18	1	07/29/2019	93	1192	2	10.4	0.35	40.11
2 multi	2	H						
18	1	07/30/2019	94	1193	2	10.2	0.24	34.7
2 multi	2	H						
18	1	07/31/2019	95	1194	2	10.2	0.24	37.8
2 multi	2	H						
18	1	08/01/2019	96	1195	2	9.8	0.21	35.45
2 multi	2	H						
18	1	08/02/2019	97	1196	2	10	0.08	36.31
2 multi	2	H						
18	1	08/03/2019	98	1197	2	10.4	0.15	37.61
2 multi	2	H						
18	1	08/04/2019	99	1198	2	10.2	0.13	36.65
2 multi	2	H						
18	1	08/05/2019	100	1199	2	10.3	0.2	38.96
2 multi	2	H						
18	1	08/06/2019	101	1200	2	10	0	38.83
2 multi	2	H						
18	1	08/07/2019	102	1201	2	10.2	0.18	37.22
2 multi	2	H						
18	1	08/08/2019	103	1202	2	10.2	0.2	36.67
2 multi	2	H						
18	1	08/09/2019	104	1203	2	10	0.21	38.97
2 multi	2	H						
18	1	08/10/2019	105	1204	2	10	0.14	37.82
2 multi	2	H						
18	1	08/11/2019	106	1205	2	10	0.24	36.5
2 multi	2	H						
18	1	04/05/2020	7	1443	3	10.4	0.24	33.81
2 multi	1	H						
18	1	04/06/2020	8	1444	3	10.4	0.12	33.42
2 multi	1	H						
18	1	04/07/2020	9	1445	3	10.2	0.24	35.12
2 multi	1	H						
18	1	04/08/2020	10	1446	3	10	0.19	36.54
2 multi	1	H						
18	1	04/09/2020	11	1447	3	9.6	0.6	37.52

2 multi	1	H						
18	1	04/10/2020	12	1448	3	9.6	0.17	36
2 multi	1	H						
18	1	04/11/2020	13	1449	3	10	0.06	33.75
2 multi	1	H						
18	1	04/12/2020	14	1450	3	10.3	0.31	34.3
2 multi	1	H						
18	1	04/13/2020	15	1451	3	10.2	0.04	38.45
2 multi	1	H						
18	1	04/14/2020	16	1452	3	10	0.41	37.77
2 multi	1	H						
18	1	04/15/2020	17	1453	3	9.8	0.12	36.75
2 multi	1	H						
18	1	04/16/2020	18	1454	3	11.3	0.06	37.56
2 multi	1	H						
18	1	04/17/2020	19	1455	3	10.2	0.01	39.87
2 multi	1	H						
18	1	04/18/2020	20	1456	3	10.4	0.26	36.34
2 multi	1	H						
18	1	04/19/2020	21	1457	3	9.9	0.02	40.97
2 multi	1	H						
18	1	04/20/2020	22	1458	3	9.9	0	40.64
2 multi	2	H						
18	1	04/21/2020	23	1459	3	10	0.01	39.2
2 multi	2	H						
18	1	04/22/2020	24	1460	3	10	0	41.87
2 multi	2	H						
18	1	04/23/2020	25	1461	3	10.4	0.07	40.2
2 multi	2	H						
18	1	04/24/2020	26	1462	3	10.3	0.08	37.71
2 multi	2	H						
18	1	04/25/2020	27	1463	3	10.4	0.51	42.85
2 multi	2	H						
18	1	04/26/2020	28	1464	3	10.4	0.33	41.56
2 multi	2	H						
18	1	04/27/2020	29	1465	3	10.3	0	40.97
2 multi	2	H						
18	1	04/28/2020	30	1466	3	10.6	0.33	42.18
2 multi	2	H						
18	1	04/29/2020	31	1467	3	10.3	0	44.05
2 multi	2	H						
18	1	04/30/2020	32	1468	3	10.4	0.1	36.49
2 multi	2	H						
18	1	05/01/2020	33	1469	3	10.6	0.14	38.99
2 multi	2	H						
18	1	05/02/2020	34	1470	3	10.3	0.08	38.47
2 multi	2	H						
18	1	05/03/2020	35	1471	3	10.6	0.35	46.56
2 multi	2	H						
18	1	05/04/2020	36	1472	3	10.4	0.03	43
2 multi	2	H						
18	1	05/05/2020	37	1473	3	10.3	0	41.03
2 multi	2	H						
18	1	05/06/2020	38	1474	3	10.6	0.06	41.84

2 multi	2	H						
18	1	05/07/2020	39	1475	3	10.4	0.06	43.65
2 multi	2	H						
18	1	05/08/2020	40	1476	3	10.4	0.03	43.2
2 multi	2	H						
18	1	05/09/2020	41	1477	3	10.6	0.17	43.7
2 multi	2	H						
18	1	05/10/2020	42	1478	3	10.2	0.02	44.36
2 multi	2	H						
18	1	05/11/2020	43	1479	3	9.8	0	45.6
2 multi	2	H						
18	1	05/12/2020	44	1480	3	9.8	0.09	40.86
2 multi	2	H						
18	1	05/13/2020	45	1481	3	10.3	0	44.39
2 multi	2	H						
18	1	05/14/2020	46	1482	3	10.3	0.13	42.57
2 multi	2	H						
18	1	05/15/2020	47	1483	3	10.3	0.6	42.39
2 multi	2	H						
18	1	05/16/2020	48	1484	3	10.4	0	45.16
2 multi	2	H						
18	1	05/17/2020	49	1485	3	10.3	0.15	45.2
2 multi	2	H						
18	1	05/18/2020	50	1486	3	10	0.11	41.81
2 multi	2	H						
18	1	05/19/2020	51	1487	3	10	0.03	44.42
2 multi	2	H						
18	1	05/20/2020	52	1488	3	10.2	0.07	41.28
2 multi	2	H						
18	1	05/21/2020	53	1489	3	10.2	0	41.09
2 multi	2	H						
18	1	05/22/2020	54	1490	3	10	0	42.66
2 multi	2	H						
18	1	05/23/2020	55	1491	3	10	0	42.67
2 multi	2	H						
18	1	05/24/2020	56	1492	3	10.3	0.24	44.12
2 multi	2	H						
18	1	05/25/2020	57	1493	3	10.3	0.17	46.85
2 multi	2	H						
18	1	05/26/2020	58	1494	3	10	0.43	41.09
2 multi	2	H						
18	1	05/27/2020	59	1495	3	9.9	0.02	45.32
2 multi	2	H						
18	1	05/28/2020	60	1496	3	10.2	0.05	40.52
2 multi	2	H						
18	1	05/29/2020	61	1497	3	10	0	42.81
2 multi	2	H						
18	1	05/30/2020	62	1498	3	9.8	0.01	38.91
2 multi	2	H						
18	1	05/31/2020	63	1499	3	10	0.01	41.62
2 multi	2	H						
18	1	06/01/2020	64	1500	3	10.4	0.04	40.06
2 multi	2	H						
18	1	06/02/2020	65	1501	3	10.2	0	41.72

2 multi	2	H						
18	1	06/03/2020	66	1502	3	10.4	0.01	42.29
2 multi	2	H						
18	1	06/04/2020	67	1503	3	10.4	0	38.67
2 multi	2	H						
18	1	06/05/2020	68	1504	3	10.4	0.31	41.85
2 multi	2	H						
18	1	06/06/2020	69	1505	3	10.4	0.05	39.83
2 multi	2	H						
18	1	06/07/2020	70	1506	3	10.6	0.01	43.5
2 multi	2	H						
18	1	06/08/2020	71	1507	3	10	0.01	43.73
2 multi	2	H						
18	1	06/09/2020	72	1508	3	10.3	0.05	40.73
2 multi	2	H						
18	1	06/10/2020	73	1509	3	10.3	0	41.46
2 multi	2	H						
18	1	06/11/2020	74	1510	3	10	0.12	39.69
2 multi	2	H						
18	1	06/12/2020	75	1511	3	9.9	0	43.45
2 multi	2	H						
18	1	06/13/2020	76	1512	3	10	0.02	42.16
2 multi	2	H						
18	1	06/14/2020	77	1513	3	10.3	0	39.79
2 multi	2	H						
18	1	06/15/2020	78	1514	3	10	0	43.07
2 multi	2	H						
18	1	06/16/2020	79	1515	3	10	0	39.4
2 multi	2	H						
18	1	06/17/2020	80	1516	3	9.8	0.15	35.7
2 multi	2	H						
18	1	06/18/2020	81	1517	3	10.4	0	41.49
2 multi	2	H						
18	1	06/19/2020	82	1518	3	10.2	0.05	37.93
2 multi	2	H						
18	1	06/20/2020	83	1519	3	9.6	0	42.36
2 multi	2	H						
18	1	06/21/2020	84	1520	3	10	0	40.32
2 multi	2	H						
18	1	06/22/2020	85	1521	3	9.8	0	43.15
2 multi	2	H						
18	1	06/23/2020	86	1522	3	10.2	0	43.12
2 multi	2	H						
18	1	06/24/2020	87	1523	3	9.5	0	41.2
2 multi	2	H						
18	1	06/25/2020	88	1524	3	10	0	36.96
2 multi	2	H						
18	1	06/26/2020	89	1525	3	10	0	40.61
2 multi	2	H						
18	1	06/27/2020	90	1526	3	9.9	0	38.51
2 multi	2	H						
18	1	06/28/2020	91	1527	3	9.6	0	36.39
2 multi	2	H						
18	1	06/29/2020	92	1528	3	10	0	39.52

2 multi 2		H						
19	1	01/01/2020	4	886	1	9.9	0.26	24.99
1 primi 1		H						
19	1	01/02/2020	5	887	1	9.3	0.03	26.02
1 primi 1		H						
19	1	01/03/2020	6	888	1	9.8	0	26.8
1 primi 1		H						
19	1	01/04/2020	7	889	1	8.4	0	26.78
1 primi 1		H						
19	1	01/05/2020	8	890	1	9.3	0	26.63
1 primi 1		H						
19	1	01/06/2020	9	891	1	9.3	0	26.02
1 primi 1		H						
19	1	01/07/2020	10	892	1	9.6	0	27.88
1 primi 1		H						
19	1	01/08/2020	11	893	1	9.1	0.23	28.54
1 primi 1		H						
19	1	01/09/2020	12	894	1	9.5	0	29.77
1 primi 1		H						
19	1	01/10/2020	13	895	1	9.1	0.22	30.77
1 primi 1		H						
19	1	01/11/2020	14	896	1	9.2	0.03	30.53
1 primi 1		H						
19	1	01/12/2020	15	897	1	8.6	0.23	30.24
1 primi 1		H						
19	1	01/13/2020	16	898	1	9.1	0.04	32.16
1 primi 1		H						
19	1	01/14/2020	17	899	1	8.6	0.16	32.02
1 primi 1		H						
19	1	01/15/2020	18	900	1	8.7	0.12	32.01
1 primi 1		H						
19	1	01/16/2020	19	901	1	8.9	0	32.37
1 primi 1		H						
19	1	01/17/2020	20	902	1	8.9	0.32	32.99
1 primi 1		H						
19	1	01/18/2020	21	903	1	8.5	0	32.42
1 primi 1		H						
19	1	01/19/2020	22	904	1	8.8	0	33.94
1 primi 2		H						
19	1	01/20/2020	23	905	1	8.9	0.11	33.4
1 primi 2		H						
19	1	01/21/2020	24	906	1	8.9	0.21	33.11
1 primi 2		H						
19	1	01/22/2020	25	907	1	8.9	0	33.93
1 primi 2		H						
19	1	01/23/2020	26	908	1	8.6	0.13	34.48
1 primi 2		H						
19	1	01/24/2020	27	909	1	8.9	0.06	33.56
1 primi 2		H						
19	1	01/25/2020	28	910	1	9.2	0	34.5
1 primi 2		H						
19	1	01/26/2020	29	911	1	9.1	0	33.52
1 primi 2		H						
19	1	01/27/2020	30	912	1	9.1	0.1	34.42

1 primi 2 H
19 1 01/28/2020 31 913 1 8.7 0 32.56
1 primi 2 H
19 1 01/29/2020 32 914 1 9.3 0.03 33.49
1 primi 2 H
19 1 01/30/2020 33 915 1 8.9 0.26 33.79
1 primi 2 H
19 1 01/31/2020 34 916 1 8.9 0.28 34.66
1 primi 2 H
19 1 02/01/2020 35 917 1 8.9 0.22 35.39
1 primi 2 H
19 1 02/02/2020 36 918 1 8.7 0 35.37
1 primi 2 H
19 1 02/03/2020 37 919 1 8.9 0.21 35.84
1 primi 2 H
19 1 02/04/2020 38 920 1 8.9 0.15 34.9
1 primi 2 H
19 1 02/05/2020 39 921 1 8.8 0 35.84
1 primi 2 H
19 1 02/06/2020 40 922 1 8.9 0.16 33.73
1 primi 2 H
19 1 02/07/2020 41 923 1 8.4 0.03 35.32
1 primi 2 H
19 1 02/08/2020 42 924 1 8.8 0.2 34.81
1 primi 2 H
19 1 02/09/2020 43 925 1 8.7 0.26 34.17
1 primi 2 H
19 1 02/10/2020 44 926 1 8.7 0.31 34.44
1 primi 2 H
19 1 02/11/2020 45 927 1 8.8 0 35.64
1 primi 2 H
19 1 02/12/2020 46 928 1 9.2 0.32 35.18
1 primi 2 H
19 1 02/13/2020 47 929 1 8.6 0 34.79
1 primi 2 H
19 1 02/14/2020 48 930 1 9.1 0.34 35.36
1 primi 2 H
19 1 02/15/2020 49 931 1 8.6 0.26 32.24
1 primi 2 H
19 1 02/16/2020 50 932 1 8.7 0.22 34.19
1 primi 2 H
19 1 02/17/2020 51 933 1 9.1 0.25 35.57
1 primi 2 H
19 1 02/18/2020 52 934 1 8.5 0 33.42
1 primi 2 H
19 1 02/19/2020 53 935 1 9.2 0.23 34.63
1 primi 2 H
19 1 02/20/2020 54 936 1 9.5 0 33.99
1 primi 2 H
19 1 02/21/2020 55 937 1 9.5 0.2 36.46
1 primi 2 H
19 1 02/22/2020 56 938 1 9.3 0 36.49
1 primi 2 H
19 1 02/23/2020 57 939 1 9.3 0.02 36.24

1 primi	2	H						
19	1	02/24/2020	58	940	1	9.1	0.17	34.5
1 primi	2	H						
19	1	02/25/2020	59	941	1	9.5	0	36.28
1 primi	2	H						
19	1	02/26/2020	60	942	1	9.1	0.2	36
1 primi	2	H						
19	1	11/12/2020	3	1202	2	8.9	0.34	23.1
2 multi	1	H						
19	1	11/13/2020	4	1203	2	9.2	0.12	27.38
2 multi	1	H						
19	1	11/14/2020	5	1204	2	9.3	0.32	24.66
2 multi	1	H						
19	1	11/15/2020	6	1205	2	9.3	0.18	29.39
2 multi	1	H						
19	1	11/16/2020	7	1206	2	8.9	0.28	30.2
2 multi	1	H						
19	1	11/17/2020	8	1207	2	0	0.1	28.51
2 multi	1	H						
19	1	11/18/2020	9	1208	2	8.9	0.19	33.11
2 multi	1	H						
19	1	11/19/2020	10	1209	2	9.1	0.05	33.33
2 multi	1	H						
19	1	11/20/2020	11	1210	2	8.9	0.04	32.53
2 multi	1	H						
19	1	11/21/2020	12	1211	2	8.9	0.08	34.86
2 multi	1	H						
19	1	11/22/2020	13	1212	2	8.7	0.1	34.92
2 multi	1	H						
19	1	11/23/2020	14	1213	2	8.8	0.26	35.75
2 multi	1	H						
19	1	11/24/2020	15	1214	2	8.8	0.04	34.82
2 multi	1	H						
19	1	11/25/2020	16	1215	2	9.1	0.08	33.87
2 multi	1	H						
19	1	11/26/2020	17	1216	2	8.9	0.64	37.12
2 multi	1	H						
19	1	11/27/2020	18	1217	2	9.2	0.08	38.43
2 multi	1	H						
19	1	11/28/2020	19	1218	2	9.2	0.21	35.68
2 multi	1	H						
19	1	11/29/2020	20	1219	2	8.8	0	37.47
2 multi	1	H						
19	1	11/30/2020	21	1220	2	8.6	0.17	36.75
2 multi	1	H						
19	1	12/01/2020	22	1221	2	9.3	0.01	38.8
2 multi	2	H						
19	1	12/02/2020	23	1222	2	8.9	0.07	39.08
2 multi	2	H						
19	1	12/03/2020	24	1223	2	8.8	0.07	38.85
2 multi	2	H						
19	1	12/04/2020	25	1224	2	8.8	0.19	40.14
2 multi	2	H						
19	1	12/05/2020	26	1225	2	8.9	0.08	38.48

2 multi	2	H						
19	1	12/06/2020	27	1226	2	9.2	0.07	40.89
2 multi	2	H						
19	1	12/07/2020	28	1227	2	9.5	0.08	41.12
2 multi	2	H						
19	1	12/08/2020	29	1228	2	8.9	0.33	40.84
2 multi	2	H						
19	1	12/09/2020	30	1229	2	9.2	0.22	39.55
2 multi	2	H						
19	1	12/10/2020	31	1230	2	8.8	0.32	38.77
2 multi	2	H						
19	1	12/11/2020	32	1231	2	9.2	0.22	39.5
2 multi	2	H						
19	1	12/12/2020	33	1232	2	9.2	0.03	40.61
2 multi	2	H						
19	1	12/13/2020	34	1233	2	9.3	0.04	40.5
2 multi	2	H						
19	1	12/14/2020	35	1234	2	9.1	0.2	41.63
2 multi	2	H						
19	1	12/15/2020	36	1235	2	9.5	0	43.37
2 multi	2	H						
19	1	12/16/2020	37	1236	2	9.2	0	40.16
2 multi	2	H						
19	1	12/17/2020	38	1237	2	8.8	0.34	40.07
2 multi	2	H						
19	1	12/18/2020	39	1238	2	8.7	0.38	39.29
2 multi	2	H						
19	1	12/19/2020	40	1239	2	8.6	0	36.88
2 multi	2	H						
19	1	12/20/2020	41	1240	2	9.1	0	39.84
2 multi	2	H						
19	1	12/21/2020	42	1241	2	9.2	0.01	39.11
2 multi	2	H						
19	1	12/22/2020	43	1242	2	9.3	0.25	40.58
2 multi	2	H						
19	1	12/23/2020	44	1243	2	9.5	0.25	39.39
2 multi	2	H						
19	1	12/24/2020	45	1244	2	9.1	0.31	40.22
2 multi	2	H						
19	1	12/25/2020	46	1245	2	9.3	0.22	41.33
2 multi	2	H						
19	1	12/26/2020	47	1246	2	9.3	0.19	39.42
2 multi	2	H						
19	1	12/27/2020	48	1247	2	0	0	39.31
2 multi	2	H						
19	1	12/28/2020	49	1248	2	10.4	0.2	45.7
2 multi	2	H						
19	1	12/29/2020	50	1249	2	10.3	0.22	34.51
2 multi	2	H						
19	1	12/30/2020	51	1250	2	10.3	0.07	37.94
2 multi	2	H						
19	1	12/31/2020	52	1251	2	9.9	0.21	39.69
2 multi	2	H						
19	1	01/01/2021	53	1252	2	9.9	0.26	43.23

2 multi 2		H						
19	1	01/02/2021	54	1253	2	9.3	0.33	37.25
2 multi 2		H						
19	1	01/03/2021	55	1254	2	9.8	0.04	38.45
2 multi 2		H						
19	1	01/04/2021	56	1255	2	9.8	0.3	38.54
2 multi 2		H						
19	1	01/05/2021	57	1256	2	9.3	0.28	32.71
2 multi 2		H						
19	1	01/06/2021	58	1257	2	9.8	0	33.7
2 multi 2		H						
19	1	01/07/2021	59	1258	2	9.3	0.32	36.62
2 multi 2		H						
19	1	01/08/2021	60	1259	2	9.5	0.01	35.27
2 multi 2		H						
19	1	01/09/2021	61	1260	2	9.5	0.06	33.07
2 multi 2		H						
19	1	01/10/2021	62	1261	2	9.2	0.06	35.15
2 multi 2		H						
19	1	01/11/2021	63	1262	2	9.3	0.04	39.29
2 multi 2		H						
19	1	01/12/2021	64	1263	2	9.8	0.04	39.76
2 multi 2		H						
19	1	01/13/2021	65	1264	2	9.9	0.03	40.11
2 multi 2		H						
19	1	01/14/2021	66	1265	2	9.6	0.03	37.79
2 multi 2		H						
19	1	01/15/2021	67	1266	2	9.1	0.03	37.38
2 multi 2		H						
19	1	01/16/2021	68	1267	2	9.3	0.03	36.24
2 multi 2		H						
19	1	01/17/2021	69	1268	2	9.5	0	39.83
2 multi 2		H						
19	1	01/18/2021	70	1269	2	9.3	0.16	34.15
2 multi 2		H						
19	1	01/19/2021	71	1270	2	9.2	0.04	33.6
2 multi 2		H						
19	1	01/20/2021	72	1271	2	9.2	0	33.4
2 multi 2		H						
19	1	01/21/2021	73	1272	2	9.3	0	31.72
2 multi 2		H						
19	1	01/22/2021	74	1273	2	9.2	0.42	33.44
2 multi 2		H						
19	1	01/23/2021	75	1274	2	9.2	0.16	35.33
2 multi 2		H						
19	1	01/24/2021	76	1275	2	9.3	0.14	36.01
2 multi 2		H						
19	1	01/25/2021	77	1276	2	9.9	0	38.93
2 multi 2		H						
19	1	01/26/2021	78	1277	2	9.8	0.06	37.49
2 multi 2		H						
19	1	01/27/2021	79	1278	2	9.3	0.2	37.12
2 multi 2		H						
19	1	01/28/2021	80	1279	2	9.6	0.03	38.54

2 multi 2		H						
19	1	01/29/2021	81	1280	2	10.2	0	38.79
2 multi 2		H						
19	1	01/30/2021	82	1281	2	9.6	0.59	36.38
2 multi 2		H						
19	1	01/31/2021	83	1282	2	9.1	0.15	33.7
2 multi 2		H						
19	1	02/01/2021	84	1283	2	9.2	0	36.59
2 multi 2		H						
19	1	02/02/2021	85	1284	2	9.5	0.03	39.36
2 multi 2		H						
19	1	02/03/2021	86	1285	2	9.6	0.04	40.69
2 multi 2		H						
19	1	02/04/2021	87	1286	2	9.8	0.02	36.24
2 multi 2		H						
19	1	02/05/2021	88	1287	2	9.6	0.31	39.81
2 multi 2		H						
19	1	02/06/2021	89	1288	2	9.6	0.02	39.09
2 multi 2		H						
19	1	02/07/2021	90	1289	2	9.5	0.13	39.67
2 multi 2		H						
19	1	02/08/2021	91	1290	2	9.6	0.06	41.42
2 multi 2		H						
19	1	02/09/2021	92	1291	2	9.5	0.09	40.68
2 multi 2		H						
19	1	02/10/2021	93	1292	2	9.6	0.04	39.63
2 multi 2		H						
19	1	02/11/2021	94	1293	2	9.8	0.05	39.06
2 multi 2		H						
19	1	02/12/2021	95	1294	2	9.6	0	40.76
2 multi 2		H						
19	1	02/13/2021	96	1295	2	9.5	0.05	38.78
2 multi 2		H						
19	1	02/14/2021	97	1296	2	9.5	0.05	37.7
2 multi 2		H						
19	1	02/15/2021	98	1297	2	9.8	0.04	38.64
2 multi 2		H						
19	1	02/16/2021	99	1298	2	9.8	0.11	39.43
2 multi 2		H						
19	1	02/17/2021	100	1299	2	9.8	0.04	40.79
2 multi 2		H						
19	1	02/18/2021	101	1300	2	9.6	0.08	39.19
2 multi 2		H						
19	1	02/19/2021	102	1301	2	9.8	0	39.07
2 multi 2		H						
19	1	02/20/2021	103	1302	2	9.8	0	40.36
2 multi 2		H						
19	1	02/21/2021	104	1303	2	9.9	0.04	41.27
2 multi 2		H						
19	1	02/22/2021	105	1304	2	9.9	0	37.31
2 multi 2		H						
19	1	02/23/2021	106	1305	2	10	0.04	39.17
2 multi 2		H						
19	1	02/24/2021	107	1306	2	9.6	0	38.03

2 multi	2	H						
19	1	02/25/2021	108	1307	2	9.5	0.35	41.1
2 multi	2	H						
19	1	02/26/2021	109	1308	2	9.9	0	40.45
2 multi	2	H						
19	1	02/27/2021	110	1309	2	10	0.02	39.53
2 multi	2	H						
19	1	02/28/2021	111	1310	2	9.5	0.33	39.55
2 multi	2	H						
19	1	03/01/2021	112	1311	2	9.6	0.32	40.05
2 multi	2	H						
19	1	03/02/2021	113	1312	2	10.2	0	39.96
2 multi	2	H						
19	1	03/03/2021	114	1313	2	10.2	0.06	40.57
2 multi	2	H						
19	1	03/04/2021	115	1314	2	10	0.03	39.16
2 multi	2	H						
19	1	03/05/2021	116	1315	2	9.9	0.04	38.65
2 multi	2	H						
19	1	03/06/2021	117	1316	2	10	0.03	43.33
2 multi	2	H						
19	1	03/07/2021	118	1317	2	9.9	0.01	40.29
2 multi	2	H						
19	1	03/08/2021	119	1318	2	10	0.01	40.69
2 multi	2	H						
19	1	03/09/2021	120	1319	2	9.8	0.08	39.37
2 multi	2	H						
19	1	03/10/2021	121	1320	2	9.9	0.15	40.25
2 multi	2	H						
19	1	03/11/2021	122	1321	2	10.2	0.03	39.22
2 multi	2	H						
19	1	03/12/2021	123	1322	2	9.5	0.04	35.21
2 multi	2	H						
19	1	03/13/2021	124	1323	2	9.3	0.3	37.69
2 multi	2	H						
19	1	03/14/2021	125	1324	2	10.2	0.03	42.15
2 multi	2	H						
19	1	03/15/2021	126	1325	2	10	0.05	39.23
2 multi	2	H						
19	1	03/16/2021	127	1326	2	9.8	0.03	37.9
2 multi	2	H						
19	1	03/17/2021	128	1327	2	10.2	0.11	39.25
2 multi	2	H						
19	1	03/18/2021	129	1328	2	9.8	0.01	36.46
2 multi	2	H						
19	1	03/19/2021	130	1329	2	10.2	0.03	38.61
2 multi	2	H						
19	1	03/20/2021	131	1330	2	10.2	0.02	41.78
2 multi	2	H						
19	1	03/21/2021	132	1331	2	10.4	0.03	40.69
2 multi	2	H						
19	1	03/22/2021	133	1332	2	10.3	0	36.41
2 multi	2	H						
19	1	03/23/2021	134	1333	2	9.8	0	39.34

2 multi	2	H						
19	1	03/24/2021	135	1334	2	10.6	0.35	40.89
2 multi	2	H						
19	1	03/25/2021	136	1335	2	10.2	0	42.19
2 multi	2	H						
19	1	03/26/2021	137	1336	2	10.4	0.01	40.87
2 multi	2	H						
19	1	03/27/2021	138	1337	2	9.6	0	37.65
2 multi	2	H						
19	1	03/28/2021	139	1338	2	10.4	0	40.11
2 multi	2	H						
19	1	03/29/2021	140	1339	2	10.7	0.27	41.67
2 multi	2	H						
19	1	03/30/2021	141	1340	2	10.6	0.02	40.14
2 multi	2	H						
19	1	03/31/2021	142	1341	2	10.2	0.02	39.15
2 multi	2	H						
19	1	04/01/2021	143	1342	2	10.4	0.02	41.72
2 multi	2	H						
19	1	04/02/2021	144	1343	2	10.3	0	40.2
2 multi	2	H						
19	1	04/03/2021	145	1344	2	10.4	0.02	41.8
2 multi	2	H						
19	1	04/04/2021	146	1345	2	10.6	0.03	41.08
2 multi	2	H						
19	1	04/05/2021	147	1346	2	10.3	0.04	41.41
2 multi	2	H						
19	1	04/06/2021	148	1347	2	10.7	0	42.16
2 multi	2	H						
19	1	04/07/2021	149	1348	2	10.4	0.14	42.34
2 multi	2	H						
19	1	04/08/2021	150	1349	2	10.6	0.03	39.97
2 multi	2	H						
19	1	04/09/2021	151	1350	2	10.4	0.04	41.12
2 multi	2	H						
19	1	04/10/2021	152	1351	2	10.2	0.01	43.39
2 multi	2	H						
19	1	04/11/2021	153	1352	2	10.3	0	41.51
2 multi	2	H						
19	1	04/12/2021	154	1353	2	10	0	38.16
2 multi	2	H						
19	1	04/13/2021	155	1354	2	9.5	0.21	35.6
2 multi	2	H						
19	1	04/14/2021	156	1355	2	9.8	0.02	38
2 multi	2	H						
19	1	04/15/2021	157	1356	2	10	0.02	37.77
2 multi	2	H						
19	1	04/16/2021	158	1357	2	10.3	0	41.8
2 multi	2	H						
19	1	04/17/2021	159	1358	2	10.2	0.08	40.7
2 multi	2	H						
19	1	04/18/2021	160	1359	2	9.8	0.03	35.99
2 multi	2	H						
19	1	04/19/2021	161	1360	2	9.9	0	41.16

2 multi 2		H						
19	1	04/20/2021	162	1361	2	10	0	38.34
2 multi 2		H						
19	1	04/21/2021	163	1362	2	10.3	0	43.08
2 multi 2		H						
19	1	04/22/2021	164	1363	2	10.3	0.03	42.99
2 multi 2		H						
19	1	04/23/2021	165	1364	2	10.3	0.03	43.62
2 multi 2		H						
19	1	04/24/2021	166	1365	2	10.3	0.02	38.61
2 multi 2		H						
19	1	04/25/2021	167	1366	2	10.3	0.05	42.43
2 multi 2		H						
19	1	04/26/2021	168	1367	2	10.2	0.04	44.02
2 multi 2		H						
19	1	04/27/2021	169	1368	2	10.4	0	39.32
2 multi 2		H						
19	1	04/28/2021	170	1369	2	10.3	0	41.16
2 multi 2		H						
19	1	04/29/2021	171	1370	2	10.4	0.04	40.33
2 multi 2		H						
19	1	04/30/2021	172	1371	2	10.3	0	39.99
2 multi 2		H						
19	1	05/01/2021	173	1372	2	9.9	0.03	38.45
2 multi 2		H						
19	1	05/02/2021	174	1373	2	10.4	0.04	39.98
2 multi 2		H						
19	1	05/03/2021	175	1374	2	10.2	0.03	39.75
2 multi 2		H						
19	1	05/04/2021	176	1375	2	10.3	0.04	39.52
2 multi 2		H						
19	1	05/05/2021	177	1376	2	10.3	0	38.01
2 multi 2		H						
19	1	05/06/2021	178	1377	2	10.4	0.05	40.49
2 multi 2		H						
19	1	05/07/2021	179	1378	2	10.4	0.05	40.18
2 multi 2		H						
19	1	05/08/2021	180	1379	2	9.9	0	39.2
2 multi 2		H						
19	1	05/09/2021	181	1380	2	10	0.03	42.95
2 multi 2		H						
19	1	05/10/2021	182	1381	2	10	0	43.43
2 multi 2		H						
19	1	05/11/2021	183	1382	2	10	0	39.25
2 multi 2		H						
19	1	05/12/2021	184	1383	2	9.9	0.04	42.06
2 multi 2		H						
19	1	05/13/2021	185	1384	2	10.3	0.26	38.44
2 multi 2		H						
19	1	05/14/2021	186	1385	2	10.2	0.04	39.71
2 multi 2		H						
19	1	05/15/2021	187	1386	2	10.2	0	35.65
2 multi 2		H						
19	1	05/16/2021	188	1387	2	10	0.34	38.94

2 multi	2	H						
19	1	05/17/2021	189	1388	2	10	0.03	40.99
2 multi	2	H						
19	1	05/18/2021	190	1389	2	10.2	0.22	43.37
2 multi	2	H						
19	1	05/19/2021	191	1390	2	10	0.3	41.62
2 multi	2	H						
19	1	05/20/2021	192	1391	2	10	0.03	41.4
2 multi	2	H						
19	1	05/21/2021	193	1392	2	9.6	0.21	41.63
2 multi	2	H						
19	1	05/22/2021	194	1393	2	10.3	0.28	41.52
2 multi	2	H						
19	1	05/23/2021	195	1394	2	9.9	0	43.29
2 multi	2	H						
19	1	05/24/2021	196	1395	2	9.6	0	36.49
2 multi	2	H						
19	1	05/25/2021	197	1396	2	10	0.03	35.21
2 multi	2	H						
19	1	05/26/2021	198	1397	2	9.9	0	21.23
2 multi	2	H						
21	1	01/25/2020	4	725	1	10.2	0.16	24.57
1 primi	1	H						
21	1	01/26/2020	5	726	1	11.4	0.15	25.41
1 primi	1	H						
21	1	01/27/2020	6	727	1	10.2	0.08	25.75
1 primi	1	H						
21	1	01/28/2020	7	728	1	10.3	0.15	24.71
1 primi	1	H						
21	1	01/29/2020	8	729	1	10	0.05	24.75
1 primi	1	H						
21	1	01/30/2020	9	730	1	10.7	0.04	27.99
1 primi	1	H						
21	1	01/31/2020	10	731	1	10.2	0	28.91
1 primi	1	H						
21	1	02/01/2020	11	732	1	9.5	0.05	29.37
1 primi	1	H						
21	1	02/02/2020	12	733	1	10.4	0	30.68
1 primi	1	H						
21	1	02/03/2020	13	734	1	10	0.04	29.89
1 primi	1	H						
21	1	02/04/2020	14	735	1	10	0.02	27.32
1 primi	1	H						
21	1	02/05/2020	15	736	1	10.4	0.01	30.17
1 primi	1	H						
21	1	02/06/2020	16	737	1	10.7	0.02	27.7
1 primi	1	H						
21	1	02/07/2020	17	738	1	10	0	30.39
1 primi	1	H						
21	1	02/08/2020	18	739	1	10	0.01	30.24
1 primi	1	H						
21	1	02/09/2020	19	740	1	10	0.01	30.15
1 primi	1	H						
21	1	02/10/2020	20	741	1	10.3	0	28.74

1 primi	1	H						
21	1	02/11/2020	21	742	1	9.9	0.01	29.09
1 primi	1	H						
21	1	02/12/2020	22	743	1	10.2	0	29.12
1 primi	2	H						
21	1	02/13/2020	23	744	1	9.8	0	30.39
1 primi	2	H						
21	1	02/14/2020	24	745	1	10.3	0	31.06
1 primi	2	H						
21	1	02/15/2020	25	746	1	10.3	0	32.42
1 primi	2	H						
21	1	02/16/2020	26	747	1	10.4	0	32.77
1 primi	2	H						
21	1	02/17/2020	27	748	1	9.9	0.1	31.56
1 primi	2	H						
21	1	02/18/2020	28	749	1	10.2	0	33.77
1 primi	2	H						
21	1	02/19/2020	29	750	1	10.4	0	33.05
1 primi	2	H						
21	1	02/20/2020	30	751	1	10.4	0	32
1 primi	2	H						
21	1	02/21/2020	31	752	1	10	0	33.54
1 primi	2	H						
21	1	02/22/2020	32	753	1	10.3	0.11	31.89
1 primi	2	H						
21	1	02/23/2020	33	754	1	9.6	0	31.43
1 primi	2	H						
21	1	02/24/2020	34	755	1	9.8	0	32.33
1 primi	2	H						
21	1	02/25/2020	35	756	1	10	0	32.95
1 primi	2	H						
21	1	02/26/2020	36	757	1	9.9	0	33.13
1 primi	2	H						
21	1	02/27/2020	37	758	1	10.2	0	34.07
1 primi	2	H						
21	1	02/28/2020	38	759	1	10.3	0	31.6
1 primi	2	H						
21	1	02/29/2020	39	760	1	10	0	32.76
1 primi	2	H						
21	1	03/01/2020	40	761	1	10.2	0	32.26
1 primi	2	H						
21	1	03/02/2020	41	762	1	10	0	32.79
1 primi	2	H						
21	1	03/03/2020	42	763	1	10	0.13	34.27
1 primi	2	H						
21	1	03/04/2020	43	764	1	10	0	32.95
1 primi	2	H						
21	1	03/05/2020	44	765	1	10.2	0	31.51
1 primi	2	H						
21	1	03/06/2020	45	766	1	10.3	0	35.73
1 primi	2	H						
21	1	03/07/2020	46	767	1	9.8	0	32.2
1 primi	2	H						
21	1	03/08/2020	47	768	1	10	0.23	31.12

1 primi	2	H						
21	1	03/09/2020	48	769	1	10.2	0	35.87
1 primi	2	H						
21	1	03/10/2020	49	770	1	10.2	0	30.43
1 primi	2	H						
21	1	03/11/2020	50	771	1	10	0	31.37
1 primi	2	H						
21	1	03/12/2020	51	772	1	10	0	33.08
1 primi	2	H						
21	1	03/13/2020	52	773	1	9.8	0.05	32.88
1 primi	2	H						
21	1	03/14/2020	53	774	1	9.8	0	33.05
1 primi	2	H						
21	1	03/15/2020	54	775	1	10	0	33.34
1 primi	2	H						
21	1	03/16/2020	55	776	1	10.3	0	34.26
1 primi	2	H						
21	1	03/17/2020	56	777	1	10.3	0	34.65
1 primi	2	H						
21	1	03/18/2020	57	778	1	10.4	0.28	35.33
1 primi	2	H						
21	1	03/19/2020	58	779	1	10.3	0	36.08
1 primi	2	H						
21	1	03/20/2020	59	780	1	10.3	0.09	34.01
1 primi	2	H						
21	1	03/21/2020	60	781	1	10.2	0	34.4
1 primi	2	H						
21	1	03/22/2020	61	782	1	10	0	35.73
1 primi	2	H						
21	1	03/23/2020	62	783	1	10.3	0	34.29
1 primi	2	H						
21	1	03/24/2020	63	784	1	10.2	0	33.75
1 primi	2	H						
21	1	03/25/2020	64	785	1	10.2	0	33.64
1 primi	2	H						
21	1	03/26/2020	65	786	1	10.2	0	35.08
1 primi	2	H						
21	1	03/27/2020	66	787	1	10.4	0.01	35.78
1 primi	2	H						
21	1	03/28/2020	67	788	1	10.2	0.01	32.57
1 primi	2	H						
21	1	03/29/2020	68	789	1	10	0.08	32.63
1 primi	2	H						
21	1	03/30/2020	69	790	1	9.8	0.07	34.01
1 primi	2	H						
21	1	12/22/2020	6	1057	2	12.6	0.28	27.54
2 multi	1	H						
21	1	12/23/2020	7	1058	2	11.4	0.15	31.38
2 multi	1	H						
21	1	12/24/2020	8	1059	2	10.6	0.19	31.05
2 multi	1	H						
21	1	12/25/2020	9	1060	2	10.4	0.16	31.29
2 multi	1	H						
21	1	12/26/2020	10	1061	2	10.7	0.13	30.02

2 multi	1	H						
21	1	12/27/2020	11	1062	2	10.4	0.06	31.5
2 multi	1	H						
21	1	12/28/2020	12	1063	2	10.3	0.12	32.43
2 multi	1	H						
21	1	12/29/2020	13	1064	2	10.2	0.11	35.03
2 multi	1	H						
21	1	12/30/2020	14	1065	2	10	0	30.48
2 multi	1	H						
21	1	12/31/2020	15	1066	2	10.2	0.11	35.64
2 multi	1	H						
21	1	01/01/2021	16	1067	2	10.2	0.1	34.84
2 multi	1	H						
21	1	01/02/2021	17	1068	2	10.3	0.08	38.64
2 multi	1	H						
21	1	01/03/2021	18	1069	2	10.2	0	38.47
2 multi	1	H						
21	1	01/04/2021	19	1070	2	10.3	0	35.82
2 multi	1	H						
21	1	01/05/2021	20	1071	2	10	0.1	34.35
2 multi	1	H						
21	1	01/06/2021	21	1072	2	9.9	0.09	38.76
2 multi	1	H						
21	1	01/07/2021	22	1073	2	9.9	0	34.35
2 multi	2	H						
21	1	01/08/2021	23	1074	2	10	0.02	35.93
2 multi	2	H						
21	1	01/09/2021	24	1075	2	9.9	0.08	38.93
2 multi	2	H						
21	1	01/10/2021	25	1076	2	10.2	0.07	34.21
2 multi	2	H						
21	1	01/11/2021	26	1077	2	9.8	0.05	38.63
2 multi	2	H						
21	1	01/12/2021	27	1078	2	10.2	0.07	37.03
2 multi	2	H						
21	1	01/13/2021	28	1079	2	9.9	0.89	38.38
2 multi	2	H						
21	1	01/14/2021	29	1080	2	10	0.05	36.63
2 multi	2	H						
21	1	01/15/2021	30	1081	2	9.9	0.21	37.37
2 multi	2	H						
21	1	01/16/2021	31	1082	2	9.8	0.19	33.89
2 multi	2	H						
21	1	01/17/2021	32	1083	2	9.6	0.07	28.34
2 multi	2	H						
21	1	01/18/2021	33	1084	2	9.9	0.07	31.45
2 multi	2	H						
21	1	01/19/2021	34	1085	2	9.6	0.06	33.46
2 multi	2	H						
21	1	01/20/2021	35	1086	2	10.2	0.07	37.48
2 multi	2	H						
21	1	01/21/2021	36	1087	2	10.7	0.05	34.51
2 multi	2	H						
21	1	01/22/2021	37	1088	2	10.2	0.05	33.15

2 multi	2	H						
21	1	01/23/2021	38	1089	2	10.9	0.05	36.32
2 multi	2	H						
21	1	01/24/2021	39	1090	2	10.2	0	32.35
2 multi	2	H						
21	1	01/25/2021	40	1091	2	10.3	0.07	35.86
2 multi	2	H						
21	1	01/26/2021	41	1092	2	10	0	37.74
2 multi	2	H						
21	1	01/27/2021	42	1093	2	10	0	36.37
2 multi	2	H						
21	1	01/28/2021	43	1094	2	9.9	0.06	35.29
2 multi	2	H						
21	1	01/29/2021	44	1095	2	10.2	0.05	38.1
2 multi	2	H						
21	1	01/30/2021	45	1096	2	10	0	36.69
2 multi	2	H						
21	1	01/31/2021	46	1097	2	10.3	0.06	40.87
2 multi	2	H						
21	1	02/01/2021	47	1098	2	12.5	0.14	25.11
2 multi	2	H						
21	1	02/02/2021	48	1099	2	12.3	0.16	10.45
2 multi	2	H						
21	1	02/03/2021	49	1100	2	11.9	0	23.93
2 multi	2	H						
21	1	02/04/2021	50	1101	2	11.4	0.1	28.36
2 multi	2	H						
21	1	02/05/2021	51	1102	2	11.3	0.08	32.59
2 multi	2	H						
21	1	02/06/2021	52	1103	2	10.6	0.05	31
2 multi	2	H						
21	1	02/07/2021	53	1104	2	10.3	0	33.26
2 multi	2	H						
21	1	02/08/2021	54	1105	2	10.6	0.05	32.09
2 multi	2	H						
21	1	02/09/2021	55	1106	2	10.9	0.04	32.91
2 multi	2	H						
21	1	02/10/2021	56	1107	2	11.1	0.05	30.01
2 multi	2	H						
21	1	02/11/2021	57	1108	2	10.9	0	30.5
2 multi	2	H						
21	1	02/12/2021	58	1109	2	10.9	0.06	33.14
2 multi	2	H						
21	1	02/13/2021	59	1110	2	10.9	0	31.68
2 multi	2	H						
21	1	02/14/2021	60	1111	2	10.7	0.06	29.81
2 multi	2	H						
21	1	02/15/2021	61	1112	2	10.9	0.06	36.78
2 multi	2	H						
21	1	02/16/2021	62	1113	2	10.7	0.06	36.85
2 multi	2	H						
21	1	02/17/2021	63	1114	2	10.6	0.06	38.96
2 multi	2	H						
21	1	02/18/2021	64	1115	2	10.7	0.04	36.08

2 multi	2	H						
21	1	02/19/2021	65	1116	2	10.6	0.06	34.53
2 multi	2	H						
21	1	02/20/2021	66	1117	2	10.7	0.05	33.84
2 multi	2	H						
21	1	02/21/2021	67	1118	2	11.1	0.06	34.33
2 multi	2	H						
21	1	02/22/2021	68	1119	2	10.7	0.08	36.47
2 multi	2	H						
21	1	02/23/2021	69	1120	2	10.2	0.05	33.5
2 multi	2	H						
21	1	02/24/2021	70	1121	2	10.7	0.06	34.28
2 multi	2	H						
21	1	02/25/2021	71	1122	2	10.6	0	35.6
2 multi	2	H						
21	1	02/26/2021	72	1123	2	10.6	0.14	36.51
2 multi	2	H						
21	1	02/27/2021	73	1124	2	10.4	0.08	33.71
2 multi	2	H						
21	1	02/28/2021	74	1125	2	10.3	0.08	35.36
2 multi	2	H						
21	1	03/01/2021	75	1126	2	10.4	0.06	33.46
2 multi	2	H						
21	1	03/02/2021	76	1127	2	10.6	0.09	36.69
2 multi	2	H						
21	1	03/03/2021	77	1128	2	10	0.09	35.7
2 multi	2	H						
21	1	03/04/2021	78	1129	2	10.2	0.07	37.7
2 multi	2	H						
21	1	03/05/2021	79	1130	2	10.2	0.07	34.67
2 multi	2	H						
21	1	03/06/2021	80	1131	2	10.3	0	38.64
2 multi	2	H						
21	1	03/07/2021	81	1132	2	10.3	0.06	35.57
2 multi	2	H						
21	1	03/08/2021	82	1133	2	10.2	0	36.7
2 multi	2	H						
21	1	03/09/2021	83	1134	2	10.4	0.05	37.21
2 multi	2	H						
21	1	03/10/2021	84	1135	2	10.2	0.05	33.77
2 multi	2	H						
21	1	03/11/2021	85	1136	2	10.4	0.05	32.84
2 multi	2	H						
21	1	03/12/2021	86	1137	2	10.3	0.02	35.12
2 multi	2	H						
21	1	03/13/2021	87	1138	2	10.3	0.07	34.11
2 multi	2	H						
21	1	03/14/2021	88	1139	2	10.3	0.05	35.78
2 multi	2	H						
21	1	03/15/2021	89	1140	2	10.2	0.04	37.42
2 multi	2	H						
21	1	03/16/2021	90	1141	2	10.4	0.27	35.92
2 multi	2	H						
21	1	03/17/2021	91	1142	2	10.3	0.05	33.46

2 multi	2	H						
21	1	03/18/2021	92	1143	2	10.2	0.08	34.47
2 multi	2	H						
21	1	03/19/2021	93	1144	2	10.2	0.24	35.17
2 multi	2	H						
21	1	03/20/2021	94	1145	2	10.2	0.04	31.04
2 multi	2	H						
21	1	03/21/2021	95	1146	2	10	0	36.5
2 multi	2	H						
21	1	03/22/2021	96	1147	2	10.4	0.05	38.68
2 multi	2	H						
21	1	03/23/2021	97	1148	2	10	0	34.98
2 multi	2	H						
21	1	03/24/2021	98	1149	2	10	0.05	37.68
2 multi	2	H						
21	1	03/25/2021	99	1150	2	9.9	0	37.78
2 multi	2	H						
21	1	03/26/2021	100	1151	2	10.2	0	39.14
2 multi	2	H						
21	1	03/27/2021	101	1152	2	9.9	0.04	34.72
2 multi	2	H						
21	1	03/28/2021	102	1153	2	10	0.04	32.8
2 multi	2	H						
21	1	03/29/2021	103	1154	2	10.2	0.04	35.3
2 multi	2	H						
21	1	03/30/2021	104	1155	2	10.3	0.03	37.94
2 multi	2	H						
21	1	03/31/2021	105	1156	2	10.3	0	39.02
2 multi	2	H						
21	1	04/01/2021	106	1157	2	10	0.13	38.74
2 multi	2	H						
21	1	04/02/2021	107	1158	2	9.9	0	35.88
2 multi	2	H						
21	1	04/03/2021	108	1159	2	9.9	0.17	39.46
2 multi	2	H						
22	1	02/20/2017	8	745	1	9.1	0.07	31.09
1 primi	1	H						
22	1	02/21/2017	9	746	1	9.9	0	27.61
1 primi	1	H						
22	1	02/22/2017	10	747	1	8.9	0.12	31.09
1 primi	1	H						
22	1	02/23/2017	11	748	1	10	0	29.93
1 primi	1	H						
22	1	02/24/2017	12	749	1	9.8	0.03	33.53
1 primi	1	H						
22	1	02/25/2017	13	750	1	9.3	0.09	31.3
1 primi	1	H						
22	1	02/26/2017	14	751	1	8.7	0.02	32.38
1 primi	1	H						
22	1	02/27/2017	15	752	1	9.5	0.01	33.78
1 primi	1	H						
22	1	02/28/2017	16	753	1	8.9	0	34.23
1 primi	1	H						
22	1	03/01/2017	17	754	1	9.3	0	35.41

1 primi	1	H						
22	1	03/02/2017	18	755	1	8.8	0.07	35.92
1 primi	1	H						
22	1	03/03/2017	19	756	1	9.3	0	35.34
1 primi	1	H						
22	1	03/04/2017	20	757	1	9.2	0	33.84
1 primi	1	H						
22	1	03/05/2017	21	758	1	8.9	0.3	35.25
1 primi	1	H						
22	1	03/06/2017	22	759	1	9.2	0	33.79
1 primi	2	H						
22	1	03/07/2017	23	760	1	9.6	0	38.84
1 primi	2	H						
22	1	03/08/2017	24	761	1	9.5	0.11	37.77
1 primi	2	H						
22	1	03/09/2017	25	762	1	9.2	0.06	36.05
1 primi	2	H						
22	1	03/10/2017	26	763	1	8.7	0.21	38.37
1 primi	2	H						
22	1	03/11/2017	27	764	1	8.8	0.18	36.85
1 primi	2	H						
22	1	03/12/2017	28	765	1	8.7	0.09	35.8
1 primi	2	H						
22	1	03/13/2017	29	766	1	9.1	0.14	38.66
1 primi	2	H						
22	1	03/14/2017	30	767	1	8.7	0.08	38.89
1 primi	2	H						
22	1	03/15/2017	31	768	1	9.2	0.26	36.59
1 primi	2	H						
22	1	03/16/2017	32	769	1	8.5	0.12	38.78
1 primi	2	H						
22	1	03/17/2017	33	770	1	9.1	0.11	37.54
1 primi	2	H						
22	1	03/18/2017	34	771	1	8.8	0.09	38.61
1 primi	2	H						
22	1	03/19/2017	35	772	1	8.7	0	37.76
1 primi	2	H						
22	1	03/20/2017	36	773	1	9.1	0.22	37.2
1 primi	2	H						
22	1	03/21/2017	37	774	1	9.6	0.45	35.81
1 primi	2	H						
22	1	03/22/2017	38	775	1	8.8	0.09	34.81
1 primi	2	H						
22	1	03/23/2017	39	776	1	8.8	0.23	36.78
1 primi	2	H						
22	1	03/24/2017	40	777	1	8.8	0.02	38.08
1 primi	2	H						
22	1	03/25/2017	41	778	1	8.6	0.18	34.29
1 primi	2	H						
22	1	03/26/2017	42	779	1	8.9	0.09	35.99
1 primi	2	H						
22	1	03/27/2017	43	780	1	8.9	0.22	36.66
1 primi	2	H						
22	1	03/28/2017	44	781	1	8.7	0.25	36.41

1 primi	2	H						
22	1	03/29/2017	45	782	1	8.8	0.26	34.97
1 primi	2	H						
22	1	03/30/2017	46	783	1	8.6	0	34.78
1 primi	2	H						
22	1	03/31/2017	47	784	1	8.8	0.12	36.56
1 primi	2	H						
22	1	04/01/2017	48	785	1	9.2	0.18	35.37
1 primi	2	H						
22	1	04/02/2017	49	786	1	9.8	0	36.53
1 primi	2	H						
22	1	04/03/2017	50	787	1	9.8	0.2	31.09
1 primi	2	H						
22	1	04/04/2017	51	788	1	9.3	0.11	40.44
1 primi	2	H						
22	1	04/05/2017	52	789	1	9.5	0.05	34.87
1 primi	2	H						
22	1	04/06/2017	53	790	1	8.6	0.2	30.17
1 primi	2	H						
22	1	04/07/2017	54	791	1	8.3	0.11	32.23
1 primi	2	H						
22	1	04/08/2017	55	792	1	8.6	0.2	34.22
1 primi	2	H						
22	1	04/09/2017	56	793	1	8.9	0.19	33.95
1 primi	2	H						
22	1	04/10/2017	57	794	1	9.3	0.25	32.66
1 primi	2	H						
22	1	04/11/2017	58	795	1	8.9	0.12	34.34
1 primi	2	H						
22	1	04/12/2017	59	796	1	9.5	0.11	36.74
1 primi	2	H						
22	1	04/13/2017	60	797	1	9.5	0.01	35.26
1 primi	2	H						
22	1	04/14/2017	61	798	1	9.2	0.01	35.23
1 primi	2	H						
22	1	04/15/2017	62	799	1	9.2	0.24	34.33
1 primi	2	H						
22	1	04/16/2017	63	800	1	9.1	0.15	34.94
1 primi	2	H						
22	1	04/17/2017	64	801	1	8.9	0.18	35.12
1 primi	2	H						
22	1	04/18/2017	65	802	1	9.2	0	34.31
1 primi	2	H						
22	1	04/19/2017	66	803	1	8.4	0.2	31.54
1 primi	2	H						
22	1	04/20/2017	67	804	1	8.7	0.03	33.39
1 primi	2	H						
22	1	04/21/2017	68	805	1	8.5	0.12	32.03
1 primi	2	H						
22	1	04/22/2017	69	806	1	8.9	0.26	33.96
1 primi	2	H						
22	1	04/23/2017	70	807	1	9.3	0.06	35.02
1 primi	2	H						
22	1	04/24/2017	71	808	1	8.8	0.11	32.79

1 primi	2	H						
22	1	04/25/2017	72	809	1	9.1	0.06	33.89
1 primi	2	H						
22	1	04/26/2017	73	810	1	9.3	0	35.83
1 primi	2	H						
22	1	04/27/2017	74	811	1	9.5	0.12	35.23
1 primi	2	H						
22	1	04/28/2017	75	812	1	9.3	0.08	38.98
1 primi	2	H						
22	1	04/29/2017	76	813	1	9.5	0.1	39.74
1 primi	2	H						
22	1	04/30/2017	77	814	1	9.5	0.3	38.99
1 primi	2	H						
22	1	05/01/2017	78	815	1	9.3	0.22	36.42
1 primi	2	H						
22	1	05/02/2017	79	816	1	9.1	0	38.45
1 primi	2	H						
22	1	05/03/2017	80	817	1	9.6	0.07	34.91
1 primi	2	H						
22	1	05/04/2017	81	818	1	9.3	0.07	37.64
1 primi	2	H						
22	1	05/05/2017	82	819	1	9.6	0	39.54
1 primi	2	H						
22	1	05/06/2017	83	820	1	9.1	0.16	35.19
1 primi	2	H						
22	1	05/07/2017	84	821	1	8.6	0.15	36.19
1 primi	2	H						
22	1	05/08/2017	85	822	1	9.5	0	35.35
1 primi	2	H						
22	1	05/09/2017	86	823	1	9.1	0	37.1
1 primi	2	H						
22	1	05/10/2017	87	824	1	8.9	0.06	35.94
1 primi	2	H						
22	1	05/11/2017	88	825	1	9.5	0.07	35.37
1 primi	2	H						
22	1	05/12/2017	89	826	1	9.3	0.16	37.62
1 primi	2	H						
22	1	05/13/2017	90	827	1	9.5	0.05	38.44
1 primi	2	H						
22	1	05/14/2017	91	828	1	9.5	0.09	38.81
1 primi	2	H						
22	1	05/15/2017	92	829	1	9.5	0.12	37.35
1 primi	2	H						
22	1	05/16/2017	93	830	1	9.3	0.11	35.99
1 primi	2	H						
22	1	05/17/2017	94	831	1	9.5	0	37.58
1 primi	2	H						
22	1	05/18/2017	95	832	1	9.2	0.11	32.93
1 primi	2	H						
22	1	05/19/2017	96	833	1	9.6	0.11	38.59
1 primi	2	H						
22	1	05/20/2017	97	834	1	9.1	0.07	36.17
1 primi	2	H						
22	1	05/21/2017	98	835	1	9.6	0	38.01

1 primi 2		H						
22	1	05/22/2017	99	836	1	9.6	0.36	36.75
1 primi 2		H						
22	1	05/23/2017	100	837	1	9.5	0.07	38.11
1 primi 2		H						
22	1	05/24/2017	101	838	1	9.6	0.05	37.02
1 primi 2		H						
22	1	05/25/2017	102	839	1	9.5	0	37.55
1 primi 2		H						
22	1	05/26/2017	103	840	1	9.3	0.08	38.48
1 primi 2		H						
22	1	05/27/2017	104	841	1	8.9	0.19	37.94
1 primi 2		H						
22	1	05/28/2017	105	842	1	9.5	0	37.8
1 primi 2		H						
22	1	05/29/2017	106	843	1	9.6	0.14	37.39
1 primi 2		H						
22	1	05/30/2017	107	844	1	9.5	0.04	39.67
1 primi 2		H						
22	1	05/31/2017	108	845	1	9.3	0	39.03
1 primi 2		H						
22	1	06/01/2017	109	846	1	9.3	0	35.7
1 primi 2		H						
22	1	06/02/2017	110	847	1	10.2	0.17	33.79
1 primi 2		H						
22	1	06/03/2017	111	848	1	9.6	0.37	36.4
1 primi 2		H						
22	1	06/04/2017	112	849	1	9.3	0	36.93
1 primi 2		H						
22	1	06/05/2017	113	850	1	9.3	0.05	37.29
1 primi 2		H						
22	1	06/06/2017	114	851	1	9.2	0.12	36.76
1 primi 2		H						
22	1	06/07/2017	115	852	1	9.1	0.2	39.21
1 primi 2		H						
22	1	06/08/2017	116	853	1	9.8	0.02	35.02
1 primi 2		H						
22	1	06/09/2017	117	854	1	10.3	0.08	38.62
1 primi 2		H						
22	1	06/10/2017	118	855	1	9.5	0.03	38.38
1 primi 2		H						
22	1	06/11/2017	119	856	1	9.3	0.21	38.66
1 primi 2		H						
22	1	06/12/2017	120	857	1	8.7	0.12	35.89
1 primi 2		H						
22	1	06/13/2017	121	858	1	8.6	0.06	34.83
1 primi 2		H						
22	1	06/14/2017	122	859	1	9.5	0	36.07
1 primi 2		H						
22	1	06/15/2017	123	860	1	9.5	0.15	36.6
1 primi 2		H						
22	1	06/16/2017	124	861	1	9.2	0.11	38.05
1 primi 2		H						
22	1	06/17/2017	125	862	1	9.3	0	36.78

1 primi	2	H						
22	1	06/18/2017	126	863	1	9.1	0.31	33.63
1 primi	2	H						
22	1	06/19/2017	127	864	1	9.5	0.41	37.36
1 primi	2	H						
22	1	06/20/2017	128	865	1	9.5	0.07	36.79
1 primi	2	H						
22	1	06/21/2017	129	866	1	9.3	0.11	32.57
1 primi	2	H						
22	1	06/22/2017	130	867	1	8.9	0.1	37.18
1 primi	2	H						
22	1	06/23/2017	131	868	1	8.9	0.12	35.08
1 primi	2	H						
22	1	06/24/2017	132	869	1	9.5	0.13	36.08
1 primi	2	H						
22	1	06/25/2017	133	870	1	9.5	0.28	36.3
1 primi	2	H						
22	1	06/26/2017	134	871	1	9.5	0	36.65
1 primi	2	H						
22	1	06/27/2017	135	872	1	9.6	0.23	35.24
1 primi	2	H						
22	1	06/28/2017	136	873	1	8.8	0.07	34.92
1 primi	2	H						
22	1	06/29/2017	137	874	1	8.9	0.08	34.23
1 primi	2	H						
22	1	06/30/2017	138	875	1	8.9	0.49	34.31
1 primi	2	H						
22	1	07/01/2017	139	876	1	8.8	0.21	33.1
1 primi	2	H						
22	1	07/02/2017	140	877	1	9.2	0.1	35.26
1 primi	2	H						
22	1	07/03/2017	141	878	1	8.9	0.37	34.94
1 primi	2	H						
22	1	07/04/2017	142	879	1	9.5	0.32	34.09
1 primi	2	H						
22	1	07/05/2017	143	880	1	9.5	0.21	32.29
1 primi	2	H						
22	1	07/06/2017	144	881	1	9.3	0.21	33.35
1 primi	2	H						
22	1	07/07/2017	145	882	1	9.2	0.21	31.52
1 primi	2	H						
22	1	07/08/2017	146	883	1	9.5	0.13	31.73
1 primi	2	H						
22	1	07/09/2017	147	884	1	9.6	0	33.36
1 primi	2	H						
22	1	07/10/2017	148	885	1	9.5	0.05	33
1 primi	2	H						
22	1	07/11/2017	149	886	1	9.8	0.21	36.17
1 primi	2	H						
22	1	01/14/2018	9	1073	2	8.4	0.12	23.95
2 multi	1	H						
22	1	01/15/2018	10	1074	2	8.6	0.18	27.68
2 multi	1	H						
22	1	01/16/2018	11	1075	2	8.4	0.47	27

2 multi	1	H						
22	1	01/17/2018	12	1076	2	8.9	0.09	30.77
2 multi	1	H						
22	1	01/18/2018	13	1077	2	8.6	0.34	27.54
2 multi	1	H						
22	1	01/19/2018	14	1078	2	8.7	0.26	27.44
2 multi	1	H						
22	1	01/20/2018	15	1079	2	8.1	0.08	24.49
2 multi	1	H						
22	1	01/21/2018	16	1080	2	8.7	0.31	28.48
2 multi	1	H						
22	1	01/22/2018	17	1081	2	9.3	0.1	35.45
2 multi	1	H						
22	1	01/23/2018	18	1082	2	8.9	0.09	31.88
2 multi	1	H						
22	1	01/24/2018	19	1083	2	9.3	0.11	36.45
2 multi	1	H						
22	1	01/25/2018	20	1084	2	9.2	0.07	34.1
2 multi	1	H						
22	1	01/26/2018	21	1085	2	8.9	0	32.17
2 multi	1	H						
22	1	01/27/2018	22	1086	2	9.1	0	37.24
2 multi	2	H						
22	1	01/28/2018	23	1087	2	9.3	0	41.79
2 multi	2	H						
22	1	01/29/2018	24	1088	2	9.6	0	41.69
2 multi	2	H						
22	1	01/30/2018	25	1089	2	9.2	0	41.94
2 multi	2	H						
22	1	01/31/2018	26	1090	2	8.7	0.13	35.22
2 multi	2	H						
22	1	02/01/2018	27	1091	2	8.7	0.23	30.58
2 multi	2	H						
22	1	02/02/2018	28	1092	2	8.6	0.09	35.03
2 multi	2	H						
22	1	02/03/2018	29	1093	2	8.8	0.01	38.41
2 multi	2	H						
22	1	02/04/2018	30	1094	2	9.1	0	39.66
2 multi	2	H						
22	1	02/05/2018	31	1095	2	9.1	0.05	34.5
2 multi	2	H						
22	1	02/06/2018	32	1096	2	8.5	0	32.9
2 multi	2	H						
22	1	02/07/2018	33	1097	2	9.1	0.04	42.9
2 multi	2	H						
22	1	02/08/2018	34	1098	2	9.2	0.03	42.85
2 multi	2	H						
22	1	02/09/2018	35	1099	2	9.3	0.08	41.4
2 multi	2	H						
22	1	02/10/2018	36	1100	2	8.8	0.13	36.25
2 multi	2	H						
22	1	02/11/2018	37	1101	2	9.1	0.03	37.74
2 multi	2	H						
22	1	02/12/2018	38	1102	2	9.1	0.61	32.49

2 multi	2	H						
22	1	02/13/2018	39	1103	2	8.8	0.61	35.01
2 multi	2	H						
22	1	02/14/2018	40	1104	2	8.9	0.09	33.85
2 multi	2	H						
22	1	02/15/2018	41	1105	2	8.8	0.26	32.95
2 multi	2	H						
22	1	02/16/2018	42	1106	2	9.3	0	35.05
2 multi	2	H						
22	1	02/17/2018	43	1107	2	8.8	0	26.25
2 multi	2	H						
22	1	02/18/2018	44	1108	2	9.5	0.26	25.5
2 multi	2	H						
22	1	02/19/2018	45	1109	2	8.8	0.28	27.19
2 multi	2	H						
22	1	02/20/2018	46	1110	2	9.1	0.01	33.35
2 multi	2	H						
22	1	02/21/2018	47	1111	2	9.3	0.16	28.32
2 multi	2	H						
22	1	02/22/2018	48	1112	2	9.5	0.31	32.49
2 multi	2	H						
22	1	02/23/2018	49	1113	2	9.8	0.1	32.27
2 multi	2	H						
22	1	02/24/2018	50	1114	2	9.2	0	31.09
2 multi	2	H						
22	1	02/25/2018	51	1115	2	9.2	0	24.35
2 multi	2	H						
22	1	02/26/2018	52	1116	2	8.8	0.28	19.98
2 multi	2	H						
22	1	02/27/2018	53	1117	2	9.2	0.13	15.99
2 multi	2	H						
22	1	02/28/2018	54	1118	2	9.1	0.06	18.16
2 multi	2	H						
22	1	03/01/2018	55	1119	2	8.8	0.26	16.46
2 multi	2	H						
22	1	03/02/2018	56	1120	2	9.1	0.15	18.38
2 multi	2	H						
22	1	03/27/2018	81	1145	2	9.8	0.12	29.17
2 multi	2	H						
22	1	03/28/2018	82	1146	2	9.5	0.14	21.83
2 multi	2	H						
22	1	03/29/2018	83	1147	2	9.8	0.36	28.65
2 multi	2	H						
22	1	03/30/2018	84	1148	2	9.9	0.19	30.81
2 multi	2	H						
22	1	03/31/2018	85	1149	2	9.6	0.23	26.93
2 multi	2	H						
22	1	04/01/2018	86	1150	2	9.5	0.08	28.15
2 multi	2	H						
22	1	04/02/2018	87	1151	2	9.2	0.21	25.71
2 multi	2	H						
22	1	04/03/2018	88	1152	2	9.3	0.18	24.74
2 multi	2	H						
22	1	04/04/2018	89	1153	2	9.3	0.06	27.26

2 multi	2	H						
22	1	04/05/2018	90	1154	2	9.6	0	26.78
2 multi	2	H						
22	1	04/06/2018	91	1155	2	9.3	0.22	27.65
2 multi	2	H						
22	1	04/07/2018	92	1156	2	9.3	0.01	27.12
2 multi	2	H						
22	1	04/08/2018	93	1157	2	9.3	0	28.15
2 multi	2	H						
22	1	04/09/2018	94	1158	2	9.8	0	30.26
2 multi	2	H						
22	1	04/10/2018	95	1159	2	9.5	0.03	28.86
2 multi	2	H						
22	1	04/11/2018	96	1160	2	9.5	0.01	31.33
2 multi	2	H						
22	1	04/12/2018	97	1161	2	9.5	0.17	28.69
2 multi	2	H						
22	1	04/13/2018	98	1162	2	9.5	0	30.61
2 multi	2	H						
22	1	04/14/2018	99	1163	2	9.1	0.06	27.58
2 multi	2	H						
22	1	04/15/2018	100	1164	2	9.3	0.1	32.03
2 multi	2	H						
22	1	04/16/2018	101	1165	2	9.6	0.12	30.96
2 multi	2	H						
22	1	04/17/2018	102	1166	2	9.3	0.32	32.9
2 multi	2	H						
22	1	04/18/2018	103	1167	2	0	0.28	14.78
2 multi	2	H						
22	1	04/19/2018	104	1168	2	0	0	14.69
2 multi	2	H						
22	1	04/20/2018	105	1169	2	9.5	0.31	30.62
2 multi	2	H						
22	1	04/21/2018	106	1170	2	9.3	0.13	32.88
2 multi	2	H						
22	1	04/22/2018	107	1171	2	9.2	0.28	26.9
2 multi	2	H						
22	1	04/23/2018	108	1172	2	9.6	0.14	31.28
2 multi	2	H						
22	1	04/24/2018	109	1173	2	9.6	0.02	34.25
2 multi	2	H						
22	1	04/25/2018	110	1174	2	9.2	0.2	26.91
2 multi	2	H						
22	1	04/26/2018	111	1175	2	9.5	0.25	31.32
2 multi	2	H						
22	1	04/27/2018	112	1176	2	9.1	0.15	29.92
2 multi	2	H						
22	1	04/28/2018	113	1177	2	9.2	0.22	32.2
2 multi	2	H						
22	1	04/29/2018	114	1178	2	9.3	0.18	35.29
2 multi	2	H						
22	1	04/30/2018	115	1179	2	9.6	0.12	34.66
2 multi	2	H						
22	1	05/01/2018	116	1180	2	9.3	0.22	34.07

2 multi 2		H						
22	1	05/02/2018	117	1181	2	9.2	0.11	32.74
2 multi 2		H						
22	1	05/03/2018	118	1182	2	9.2	0.18	33
2 multi 2		H						
22	1	05/04/2018	119	1183	2	9.3	0	37.07
2 multi 2		H						
22	1	05/05/2018	120	1184	2	9.2	0	32.32
2 multi 2		H						
22	1	05/06/2018	121	1185	2	9.3	0.33	35.42
2 multi 2		H						
22	1	05/07/2018	122	1186	2	9.3	0	35.36
2 multi 2		H						
22	1	05/08/2018	123	1187	2	9.2	0.24	35.04
2 multi 2		H						
22	1	05/09/2018	124	1188	2	9.3	0.08	35.3
2 multi 2		H						
22	1	05/10/2018	125	1189	2	9.2	0	35.34
2 multi 2		H						
22	1	05/11/2018	126	1190	2	9.3	0.12	34.1
2 multi 2		H						
22	1	05/12/2018	127	1191	2	9.3	0.68	37.49
2 multi 2		H						
22	1	05/13/2018	128	1192	2	9.6	0.14	37.07
2 multi 2		H						
22	1	05/14/2018	129	1193	2	9.1	0.01	35.9
2 multi 2		H						
22	1	05/15/2018	130	1194	2	8.7	0.17	31.02
2 multi 2		H						
22	1	05/16/2018	131	1195	2	9.2	0.04	38.47
2 multi 2		H						
22	1	05/17/2018	132	1196	2	9.5	0.12	38.12
2 multi 2		H						
22	1	05/18/2018	133	1197	2	9.2	0	38.26
2 multi 2		H						
22	1	05/19/2018	134	1198	2	9.2	0.19	36.58
2 multi 2		H						
22	1	05/20/2018	135	1199	2	9.1	0	39.41
2 multi 2		H						
22	1	05/21/2018	136	1200	2	9.1	0	38.5
2 multi 2		H						
22	1	05/22/2018	137	1201	2	8.7	0.11	35.81
2 multi 2		H						
22	1	05/23/2018	138	1202	2	8.8	0.05	39.94
2 multi 2		H						
22	1	05/24/2018	139	1203	2	8.9	0	40.04
2 multi 2		H						
22	1	05/25/2018	140	1204	2	8.9	0.11	39.15
2 multi 2		H						
22	1	05/26/2018	141	1205	2	9.1	0.18	37.98
2 multi 2		H						
22	1	05/27/2018	142	1206	2	9.1	0	39.86
2 multi 2		H						
22	1	05/28/2018	143	1207	2	8.7	0.17	36.77

2 multi 2		H						
22	1	05/29/2018	144	1208	2	8.8	0.21	37.56
2 multi 2		H						
22	1	05/30/2018	145	1209	2	8.9	0	37.16
2 multi 2		H						
22	1	05/31/2018	146	1210	2	9.2	0.14	36.57
2 multi 2		H						
22	1	06/01/2018	147	1211	2	9.1	0.13	37.22
2 multi 2		H						
22	1	06/02/2018	148	1212	2	9.5	0	37.34
2 multi 2		H						
22	1	06/03/2018	149	1213	2	9.2	0.16	34.32
2 multi 2		H						
22	1	06/04/2018	150	1214	2	8.9	0.28	35.63
2 multi 2		H						
22	1	06/05/2018	151	1215	2	9.1	0.47	35.92
2 multi 2		H						
22	1	06/06/2018	152	1216	2	9.1	0	36.07
2 multi 2		H						
22	1	06/07/2018	153	1217	2	9.1	0.18	34.05
2 multi 2		H						
22	1	06/08/2018	154	1218	2	8.9	0.02	36.15
2 multi 2		H						
22	1	06/09/2018	155	1219	2	9.1	0	36.95
2 multi 2		H						
22	1	06/10/2018	156	1220	2	9.2	0.06	35.65
2 multi 2		H						
22	1	06/11/2018	157	1221	2	9.2	0.23	36.97
2 multi 2		H						
22	1	06/12/2018	158	1222	2	9.3	0.12	35.41
2 multi 2		H						
22	1	06/13/2018	159	1223	2	9.2	0.31	34.04
2 multi 2		H						
22	1	06/14/2018	160	1224	2	9.1	0.09	32.94
2 multi 2		H						
22	1	06/15/2018	161	1225	2	9.3	0.16	35.44
2 multi 2		H						
22	1	06/16/2018	162	1226	2	9.5	0.12	35.63
2 multi 2		H						
22	1	06/17/2018	163	1227	2	9.2	0.22	34.49
2 multi 2		H						
22	1	06/18/2018	164	1228	2	9.2	0	34.81
2 multi 2		H						
22	1	06/19/2018	165	1229	2	9.1	0.17	33.02
2 multi 2		H						
22	1	06/20/2018	166	1230	2	9.3	0.3	34.64
2 multi 2		H						
22	1	06/21/2018	167	1231	2	9.3	0.18	34.86
2 multi 2		H						
22	1	06/22/2018	168	1232	2	9.1	0.02	34.68
2 multi 2		H						
22	1	06/23/2018	169	1233	2	9.1	0.23	33.66
2 multi 2		H						
22	1	06/24/2018	170	1234	2	9.2	0.04	37.54

2 multi 2		H						
22	1	06/25/2018	171	1235	2	9.5	0	37.95
2 multi 2		H						
22	1	06/26/2018	172	1236	2	9.3	0.23	36.65
2 multi 2		H						
22	1	06/27/2018	173	1237	2	9.2	0	39.9
2 multi 2		H						
22	1	06/28/2018	174	1238	2	9.1	0.12	33.29
2 multi 2		H						
22	1	06/29/2018	175	1239	2	9.3	0	39.48
2 multi 2		H						
22	1	06/30/2018	176	1240	2	9.5	0.21	36.65
2 multi 2		H						
22	1	07/01/2018	177	1241	2	9.3	0	35.66
2 multi 2		H						
22	1	07/02/2018	178	1242	2	9.1	0.15	35.83
2 multi 2		H						
22	1	07/03/2018	179	1243	2	9.2	0.03	35.85
2 multi 2		H						
22	1	07/04/2018	180	1244	2	9.1	0.08	37.76
2 multi 2		H						
22	1	07/05/2018	181	1245	2	9.2	0	34.69
2 multi 2		H						
22	1	07/06/2018	182	1246	2	9.3	0.1	35.87
2 multi 2		H						
22	1	07/07/2018	183	1247	2	8.9	0.15	32.9
2 multi 2		H						
22	1	07/08/2018	184	1248	2	9.1	0	34.74
2 multi 2		H						
22	1	07/09/2018	185	1249	2	9.1	0.17	31.06
2 multi 2		H						
22	1	07/10/2018	186	1250	2	9.6	0.17	33.29
2 multi 2		H						
22	1	07/11/2018	187	1251	2	9.9	0.4	33.92
2 multi 2		H						
22	1	07/12/2018	188	1252	2	9.2	0.27	35.65
2 multi 2		H						
22	1	07/13/2018	189	1253	2	9.3	0.01	37.46
2 multi 2		H						
22	1	07/14/2018	190	1254	2	9.2	0.22	32.81
2 multi 2		H						
22	1	07/15/2018	191	1255	2	9.5	0.15	34.82
2 multi 2		H						
22	1	07/16/2018	192	1256	2	9.3	0.14	34.77
2 multi 2		H						
22	1	07/17/2018	193	1257	2	9.2	0.07	33.87
2 multi 2		H						
22	1	07/18/2018	194	1258	2	9.3	0	35.61
2 multi 2		H						
22	1	07/19/2018	195	1259	2	10.6	0.26	36.47
2 multi 2		H						
22	1	07/20/2018	196	1260	2	8.9	0	33.99
2 multi 2		H						
22	1	07/21/2018	197	1261	2	9.2	0.15	33.02

2 multi	2	H						
22	1	07/22/2018	198	1262	2	9.2	0.07	34.35
2 multi	2	H						
22	1	07/23/2018	199	1263	2	9.2	0.08	36.92
2 multi	2	H						
22	1	07/24/2018	200	1264	2	9.2	0.16	34
2 multi	2	H						
22	1	07/25/2018	201	1265	2	9.2	0.05	34.7
2 multi	2	H						
22	1	07/26/2018	202	1266	2	9.5	0.12	32.95
2 multi	2	H						
22	1	02/22/2019	7	1477	3	8.9	0.01	24.92
2 multi	1	H						
22	1	02/23/2019	8	1478	3	8.9	0.12	34.45
2 multi	1	H						
22	1	02/24/2019	9	1479	3	8.9	0.23	34.11
2 multi	1	H						
22	1	02/25/2019	10	1480	3	8.6	0	19.18
2 multi	1	H						
22	1	02/26/2019	11	1481	3	8.9	0.21	30.02
2 multi	1	H						
22	1	02/27/2019	12	1482	3	8.4	0	32.92
2 multi	1	H						
22	1	02/28/2019	13	1483	3	8.6	0.17	34.93
2 multi	1	H						
22	1	03/01/2019	14	1484	3	8.7	0	29
2 multi	1	H						
22	1	03/02/2019	15	1485	3	8.6	0.22	33.9
2 multi	1	H						
22	1	03/03/2019	16	1486	3	8.7	0	39.3
2 multi	1	H						
22	1	03/04/2019	17	1487	3	8.8	0	37.83
2 multi	1	H						
22	1	03/05/2019	18	1488	3	8.9	0	37.01
2 multi	1	H						
22	1	03/06/2019	19	1489	3	8.5	0	29.97
2 multi	1	H						
22	1	03/07/2019	20	1490	3	8.9	0.32	35.6
2 multi	1	H						
22	1	03/08/2019	21	1491	3	8.8	0.02	37.28
2 multi	1	H						
22	1	03/09/2019	22	1492	3	8.6	0	35.76
2 multi	2	H						
22	1	03/10/2019	23	1493	3	8.6	0	31.91
2 multi	2	H						
22	1	03/11/2019	24	1494	3	8.9	0	28.4
2 multi	2	H						
22	1	03/12/2019	25	1495	3	9.2	0	34.44
2 multi	2	H						
22	1	03/13/2019	26	1496	3	8.9	0.02	39.38
2 multi	2	H						
22	1	03/14/2019	27	1497	3	9.3	0.25	41.85
2 multi	2	H						
22	1	03/15/2019	28	1498	3	9.1	0	37.2

2 multi	2	H							
22	1	03/16/2019	29	1499	3	9.1	0	40.38	
2 multi	2	H							
22	1	03/17/2019	30	1500	3	8.7	0.23	34.04	
2 multi	2	H							
22	1	03/18/2019	31	1501	3	9.1	0.33	41.67	
2 multi	2	H							
22	1	03/19/2019	32	1502	3	9.1	0.17	39.42	
2 multi	2	H							
22	1	03/20/2019	33	1503	3	8.8	0.03	37.67	
2 multi	2	H							
22	1	03/21/2019	34	1504	3	9.3	0.1	40.33	
2 multi	2	H							
22	1	03/22/2019	35	1505	3	9.6	0.17	44.92	
2 multi	2	H							
22	1	03/23/2019	36	1506	3	9.6	0	40.81	
2 multi	2	H							
22	1	03/24/2019	37	1507	3	8.9	0.28	39.93	
2 multi	2	H							
22	1	03/25/2019	38	1508	3	9.1	0.28	38.36	
2 multi	2	H							
22	1	03/26/2019	39	1509	3	9.2	0.05	42.96	
2 multi	2	H							
22	1	03/27/2019	40	1510	3	9.1	0.28	40.86	
2 multi	2	H							
22	1	06/22/2020	6	1963	4	9.5	0.07	35.01	
2 multi	1	H							
22	1	06/23/2020	7	1964	4	9.3	0.13	39.1	
2 multi	1	H							
22	1	06/24/2020	8	1965	4	9.1	0	42.91	
2 multi	1	H							
22	1	06/25/2020	9	1966	4	9.2	0	47.75	
2 multi	1	H							
22	1	06/26/2020	10	1967	4	9.3	0	47.1	
2 multi	1	H							
22	1	06/27/2020	11	1968	4	9.3	0	47.93	
2 multi	1	H							
22	1	06/28/2020	12	1969	4	8.8	0	47.26	
2 multi	1	H							
22	1	06/29/2020	13	1970	4	8.7	0	47.18	
2 multi	1	H							
22	1	10/14/2020	120	2077	4	9.3	0.12	45.79	
2 multi	2	H							
22	1	10/15/2020	121	2078	4	9.2	0.41	48.41	
2 multi	2	H							
22	1	10/16/2020	122	2079	4	9.3	0	48.06	
2 multi	2	H							
22	1	10/17/2020	123	2080	4	9.5	0.12	46.26	
2 multi	2	H							
22	1	10/18/2020	124	2081	4	9.1	0.32	47.55	
2 multi	2	H							
22	1	10/19/2020	125	2082	4	9.5	0.09	46.19	
2 multi	2	H							
22	1	10/20/2020	126	2083	4	9.1	0.24	43.88	

2 multi	2	H						
22	1	10/21/2020	127	2084	4	9.3	0.1	46.58
2 multi	2	H						
22	1	10/22/2020	128	2085	4	9.6	0.08	45.32
2 multi	2	H						
22	1	10/23/2020	129	2086	4	9.3	0.11	38.45
2 multi	2	H						
22	1	10/24/2020	130	2087	4	9.6	0.14	42.72
2 multi	2	H						
22	1	10/25/2020	131	2088	4	9.6	0.12	43.3
2 multi	2	H						
22	1	10/26/2020	132	2089	4	9.6	0.05	48.69
2 multi	2	H						
22	1	10/27/2020	133	2090	4	9.6	0.06	45.82
2 multi	2	H						
22	1	10/28/2020	134	2091	4	9.3	0.36	36.85
2 multi	2	H						
22	1	10/29/2020	135	2092	4	9.6	0.13	47.75
2 multi	2	H						
22	1	10/30/2020	136	2093	4	9.2	0.2	43.76
2 multi	2	H						
22	1	10/31/2020	137	2094	4	9.6	0.52	46.75
2 multi	2	H						
22	1	11/01/2020	138	2095	4	9.5	0	42.87
2 multi	2	H						
22	1	11/02/2020	139	2096	4	9.6	0.64	47.45
2 multi	2	H						
22	1	11/03/2020	140	2097	4	9.2	0.2	44.21
2 multi	2	H						
22	1	11/04/2020	141	2098	4	8.9	0.43	41.38
2 multi	2	H						
22	1	11/05/2020	142	2099	4	9.2	0.27	43.85
2 multi	2	H						
22	1	11/06/2020	143	2100	4	9.1	0.23	43.86
2 multi	2	H						
22	1	11/07/2020	144	2101	4	9.2	0.21	44.09
2 multi	2	H						
22	1	11/08/2020	145	2102	4	9.1	0.17	42.29
2 multi	2	H						
22	1	11/09/2020	146	2103	4	9.2	0.37	43.11
2 multi	2	H						
22	1	11/10/2020	147	2104	4	9.3	0.52	44.72
2 multi	2	H						
22	1	11/11/2020	148	2105	4	9.1	0.54	43.33
2 multi	2	H						
22	1	11/12/2020	149	2106	4	9.2	0.6	41.29
2 multi	2	H						
22	1	11/13/2020	150	2107	4	9.2	0.08	41.59
2 multi	2	H						
22	1	11/14/2020	151	2108	4	9.1	0.45	42.24
2 multi	2	H						
22	1	11/15/2020	152	2109	4	8.9	0.22	38.85
2 multi	2	H						
22	1	11/16/2020	153	2110	4	9.1	0.35	42.22

2 multi	2	H						
22	1	11/17/2020	154	2111	4	9.2	0.24	42.1
2 multi	2	H						
22	1	11/18/2020	155	2112	4	9.2	0.22	39.78
2 multi	2	H						
22	1	11/19/2020	156	2113	4	9.9	0.86	40.53
2 multi	2	H						
22	1	11/20/2020	157	2114	4	9.3	0.34	42.16
2 multi	2	H						
22	1	11/21/2020	158	2115	4	9.2	0.64	42.85
2 multi	2	H						
22	1	11/22/2020	159	2116	4	9.2	0.14	41.72
2 multi	2	H						
22	1	11/23/2020	160	2117	4	9.1	0.14	44.27
2 multi	2	H						
22	1	11/24/2020	161	2118	4	8.8	0.07	43.22
2 multi	2	H						
22	1	11/25/2020	162	2119	4	8.8	0.18	40.58
2 multi	2	H						
22	1	11/26/2020	163	2120	4	9.2	0.16	40.47
2 multi	2	H						
22	1	11/27/2020	164	2121	4	9.6	0.45	41.23
2 multi	2	H						
22	1	11/28/2020	165	2122	4	8.9	0.11	36.23
2 multi	2	H						
22	1	11/29/2020	166	2123	4	9.1	0.49	38.8
2 multi	2	H						
22	1	11/30/2020	167	2124	4	8.9	0.37	36.13
2 multi	2	H						
22	1	12/01/2020	168	2125	4	9.2	0.04	39.63
2 multi	2	H						
22	1	12/02/2020	169	2126	4	9.8	0.24	38.24
2 multi	2	H						
22	1	12/03/2020	170	2127	4	9.2	0	38.8
2 multi	2	H						
22	1	12/04/2020	171	2128	4	9.1	0.11	36.43
2 multi	2	H						
22	1	12/05/2020	172	2129	4	9.5	0.08	40.8
2 multi	2	H						
22	1	12/06/2020	173	2130	4	9.3	0.12	41.59
2 multi	2	H						
22	1	12/07/2020	174	2131	4	9.2	0.54	41.69
2 multi	2	H						
22	1	12/08/2020	175	2132	4	9.3	0.14	41.53
2 multi	2	H						
22	1	12/09/2020	176	2133	4	9.6	0.36	41.3
2 multi	2	H						
22	1	12/10/2020	177	2134	4	9.6	0	42.21
2 multi	2	H						
22	1	12/11/2020	178	2135	4	9.3	0.11	41.24
2 multi	2	H						
22	1	12/12/2020	179	2136	4	9.2	0.16	39.52
2 multi	2	H						
22	1	12/13/2020	180	2137	4	9.3	0.18	41.71

2 multi 2		H						
22	1	12/14/2020	181	2138	4	9.3	0	39.96
2 multi 2		H						
22	1	12/15/2020	182	2139	4	9.5	0	42.06
2 multi 2		H						
22	1	12/16/2020	183	2140	4	9.3	0	40.52
2 multi 2		H						
24	1	04/02/2018	7	814	1	8.1	0.2	18.07
1 primi 1		H						
24	1	04/03/2018	8	815	1	8.5	0.22	18.86
1 primi 1		H						
24	1	04/04/2018	9	816	1	8.3	0	20.14
1 primi 1		H						
24	1	04/05/2018	10	817	1	9.1	0	19.78
1 primi 1		H						
24	1	04/06/2018	11	818	1	9.2	0.1	20.89
1 primi 1		H						
24	1	04/07/2018	12	819	1	8.5	0.23	19.2
1 primi 1		H						
24	1	04/08/2018	13	820	1	8.1	0.22	22.15
1 primi 1		H						
24	1	04/09/2018	14	821	1	8.9	0.15	21.32
1 primi 1		H						
24	1	04/10/2018	15	822	1	9.1	0	21.79
1 primi 1		H						
24	1	04/11/2018	16	823	1	8.9	0.07	22.55
1 primi 1		H						
24	1	04/12/2018	17	824	1	8.9	0.05	22.89
1 primi 1		H						
24	1	04/13/2018	18	825	1	8.9	0.15	23.54
1 primi 1		H						
24	1	04/14/2018	19	826	1	8.9	0	23.2
1 primi 1		H						
24	1	04/15/2018	20	827	1	8.9	0.07	24.88
1 primi 1		H						
24	1	04/16/2018	21	828	1	8.7	0.19	25.35
1 primi 1		H						
24	1	04/17/2018	22	829	1	8.7	0	23.95
1 primi 2		H						
24	1	04/18/2018	23	830	1	0	0.05	25.19
1 primi 2		H						
24	1	04/19/2018	24	831	1	8.7	0	26.71
1 primi 2		H						
24	1	04/20/2018	25	832	1	8.7	0.19	27.21
1 primi 2		H						
24	1	04/21/2018	26	833	1	9.1	0.05	26.95
1 primi 2		H						
24	1	04/22/2018	27	834	1	8.7	0.16	27.41
1 primi 2		H						
24	1	04/23/2018	28	835	1	9.1	0.05	28.01
1 primi 2		H						
24	1	04/24/2018	29	836	1	8.8	0.05	27.83
1 primi 2		H						
24	1	04/25/2018	30	837	1	8.8	0	28.21

1 primi 2 H
24 1 04/26/2018 31 838 1 8.7 0 28.89
1 primi 2 H
24 1 04/27/2018 32 839 1 8.6 0 27.49
1 primi 2 H
24 1 04/28/2018 33 840 1 8.7 0.06 27.93
1 primi 2 H
24 1 04/29/2018 34 841 1 8.8 0 28.74
1 primi 2 H
24 1 04/30/2018 35 842 1 8.9 0.05 28.74
1 primi 2 H
24 1 05/01/2018 36 843 1 8.8 0 28.34
1 primi 2 H
24 1 05/02/2018 37 844 1 8.7 0 27.63
1 primi 2 H
24 1 05/03/2018 38 845 1 8.8 0 28.39
1 primi 2 H
24 1 05/04/2018 39 846 1 8.8 0 28.29
1 primi 2 H
24 1 05/05/2018 40 847 1 8.9 0.06 31.03
1 primi 2 H
24 1 05/06/2018 41 848 1 8.4 0 29.43
1 primi 2 H
24 1 05/07/2018 42 849 1 8.8 0.07 31.27
1 primi 2 H
24 1 05/08/2018 43 850 1 8.9 0.03 28.96
1 primi 2 H
24 1 05/09/2018 44 851 1 8.6 0 28.84
1 primi 2 H
24 1 05/10/2018 45 852 1 8.7 0 29.49
1 primi 2 H
24 1 05/11/2018 46 853 1 8.7 0.18 30.33
1 primi 2 H
24 1 05/12/2018 47 854 1 8.9 0.03 28.78
1 primi 2 H
24 1 05/13/2018 48 855 1 8.9 0 30.4
1 primi 2 H
24 1 05/14/2018 49 856 1 8.8 0 30.52
1 primi 2 H
24 1 05/15/2018 50 857 1 8.6 0 28.67
1 primi 2 H
24 1 05/16/2018 51 858 1 8.7 0 31.95
1 primi 2 H
24 1 05/17/2018 52 859 1 8.5 0.04 29.12
1 primi 2 H
24 1 05/18/2018 53 860 1 8.8 0 31.53
1 primi 2 H
24 1 05/19/2018 54 861 1 8.5 0 30.29
1 primi 2 H
24 1 05/20/2018 55 862 1 8.7 0.05 32.45
1 primi 2 H
24 1 05/21/2018 56 863 1 8.7 0 28.3
1 primi 2 H
24 1 05/22/2018 57 864 1 8.9 0.07 30.48

1 primi 2		H						
24	1	05/23/2018	58	865	1	8.7	0.02	28.7
1 primi 2		H						
24	1	05/24/2018	59	866	1	8.7	0	32.88
1 primi 2		H						
24	1	05/25/2018	60	867	1	8.7	0.09	32.17
1 primi 2		H						
24	1	05/26/2018	61	868	1	8.7	0.06	29
1 primi 2		H						
24	1	05/27/2018	62	869	1	8.8	0	31.69
1 primi 2		H						
24	1	05/28/2018	63	870	1	8.7	0.05	33.37
1 primi 2		H						
24	1	05/29/2018	64	871	1	8.9	0.05	30.43
1 primi 2		H						
24	1	05/30/2018	65	872	1	8.8	0.04	29.93
1 primi 2		H						
24	1	05/31/2018	66	873	1	8.8	0.07	31.98
1 primi 2		H						
24	1	06/01/2018	67	874	1	8.8	0.12	29.71
1 primi 2		H						
24	1	06/02/2018	68	875	1	8.9	0	31.64
1 primi 2		H						
24	1	06/03/2018	69	876	1	8.8	0.07	28.87
1 primi 2		H						
24	1	06/04/2018	70	877	1	8.5	0.06	27.98
1 primi 2		H						
24	1	06/05/2018	71	878	1	8.9	0.07	29.24
1 primi 2		H						
24	1	06/06/2018	72	879	1	8.7	0	29.3
1 primi 2		H						
24	1	06/07/2018	73	880	1	8.9	0.08	28.66
1 primi 2		H						
24	1	06/08/2018	74	881	1	8.8	0.04	29.8
1 primi 2		H						
24	1	06/09/2018	75	882	1	9.1	0.04	30.45
1 primi 2		H						
24	1	06/10/2018	76	883	1	9.2	0.1	30.17
1 primi 2		H						
24	1	06/11/2018	77	884	1	9.1	0.07	28.86
1 primi 2		H						
24	1	06/12/2018	78	885	1	8.7	0.11	28.7
1 primi 2		H						
24	1	06/13/2018	79	886	1	8.8	0.09	30.67
1 primi 2		H						
24	1	06/14/2018	80	887	1	8.8	0.06	31.06
1 primi 2		H						
24	1	06/15/2018	81	888	1	9.1	0	32.33
1 primi 2		H						
24	1	06/16/2018	82	889	1	8.8	0	32.13
1 primi 2		H						
24	1	06/17/2018	83	890	1	8.9	0	31.23
1 primi 2		H						
24	1	02/26/2019	6	1144	2	8.8	0.27	24.25

2 multi 1 H
24 1 02/27/2019 7 1145 2 8.8 0.19 26.8
2 multi 1 H
24 1 02/28/2019 8 1146 2 8.5 0.17 25.46
2 multi 1 H
24 1 03/01/2019 9 1147 2 8.7 0.08 24.53
2 multi 1 H
24 1 03/02/2019 10 1148 2 8.5 0.14 26.26
2 multi 1 H
24 1 03/03/2019 11 1149 2 9.1 0.08 29.33
2 multi 1 H
24 1 03/04/2019 12 1150 2 9.1 0 30.48
2 multi 1 H
24 1 03/05/2019 13 1151 2 9.1 0 30.55
2 multi 1 H
24 1 03/06/2019 14 1152 2 8.6 0 30.78
2 multi 1 H
24 1 03/07/2019 15 1153 2 8.6 0 29.68
2 multi 1 H
24 1 03/08/2019 16 1154 2 8.6 0.11 29.07
2 multi 1 H
24 1 03/09/2019 17 1155 2 8.8 0 34.22
2 multi 1 H
24 1 03/10/2019 18 1156 2 8.8 0.06 34.02
2 multi 1 H
24 1 03/11/2019 19 1157 2 8.7 0.06 33.71
2 multi 1 H
24 1 03/12/2019 20 1158 2 8.8 0.19 35.36
2 multi 1 H
24 1 03/13/2019 21 1159 2 8.8 0 33.12
2 multi 1 H
24 1 03/14/2019 22 1160 2 8.8 0 36.11
2 multi 2 H
24 1 03/15/2019 23 1161 2 8.8 0 31.88
2 multi 2 H
24 1 03/16/2019 24 1162 2 8.5 0.07 34.99
2 multi 2 H
24 1 03/17/2019 25 1163 2 8.5 0.05 33.03
2 multi 2 H
24 1 03/18/2019 26 1164 2 8.9 0.03 37.29
2 multi 2 H
24 1 03/19/2019 27 1165 2 8.7 0 33.29
2 multi 2 H
24 1 03/20/2019 28 1166 2 8.5 0.05 34.45
2 multi 2 H
24 1 03/21/2019 29 1167 2 8.5 0.06 34.18
2 multi 2 H
24 1 03/22/2019 30 1168 2 8.9 0 37.19
2 multi 2 H
24 1 03/23/2019 31 1169 2 8.6 0.05 35.42
2 multi 2 H
24 1 03/24/2019 32 1170 2 8.6 0 36.47
2 multi 2 H
24 1 03/25/2019 33 1171 2 8.3 0.1 31.69

2 multi	2	H						
24	1	03/26/2019	34	1172	2	8.1	0.34	34.73
2 multi	2	H						
24	1	03/27/2019	35	1173	2	8.5	0.06	36.68
2 multi	2	H						
24	1	01/20/2020	5	1472	3	8.9	0.28	24.24
2 multi	1	H						
24	1	01/21/2020	6	1473	3	9.1	0.14	27.89
2 multi	1	H						
24	1	01/22/2020	7	1474	3	9.2	0.15	26.31
2 multi	1	H						
24	1	01/23/2020	8	1475	3	9.2	0.04	27.42
2 multi	1	H						
24	1	01/24/2020	9	1476	3	9.1	0.02	27.88
2 multi	1	H						
24	1	01/25/2020	10	1477	3	9.3	0.03	30.4
2 multi	1	H						
24	1	01/26/2020	11	1478	3	9.3	0	30.53
2 multi	1	H						
24	1	01/27/2020	12	1479	3	9.5	0.08	29.74
2 multi	1	H						
24	1	01/28/2020	13	1480	3	8.6	0.07	31.17
2 multi	1	H						
24	1	01/29/2020	14	1481	3	8.6	0	31.23
2 multi	1	H						
24	1	01/30/2020	15	1482	3	8.6	0.03	31.36
2 multi	1	H						
24	1	01/31/2020	16	1483	3	8.7	0	28.25
2 multi	1	H						
24	1	02/01/2020	17	1484	3	8.7	0.02	21.58
2 multi	1	H						
24	1	02/02/2020	18	1485	3	8.8	0	21.51
2 multi	1	H						
24	1	02/03/2020	19	1486	3	8.9	0	25.33
2 multi	1	H						
24	1	02/04/2020	20	1487	3	8.2	0.2	22.31
2 multi	1	H						
24	1	02/05/2020	21	1488	3	8.5	0.07	29.73
2 multi	1	H						
24	1	02/06/2020	22	1489	3	8.6	0.09	27.77
2 multi	2	H						
24	1	02/07/2020	23	1490	3	8.5	0	28.18
2 multi	2	H						
24	1	02/08/2020	24	1491	3	8.6	0.08	31.37
2 multi	2	H						
24	1	02/09/2020	25	1492	3	8.8	0.07	33.31
2 multi	2	H						
24	1	02/10/2020	26	1493	3	8.8	0.09	31.87
2 multi	2	H						
24	1	02/11/2020	27	1494	3	8.8	0.09	33.58
2 multi	2	H						
24	1	02/12/2020	28	1495	3	8.7	0.08	33.04
2 multi	2	H						
24	1	02/13/2020	29	1496	3	8.7	0	35.62

2 multi 2		H						
24	1	02/14/2020	30	1497	3	8.8	0.09	34.29
2 multi 2		H						
24	1	02/15/2020	31	1498	3	8.8	0	33.18
2 multi 2		H						
24	1	02/16/2020	32	1499	3	9.1	0.01	36.09
2 multi 2		H						
24	1	02/17/2020	33	1500	3	8.7	0.21	34.57
2 multi 2		H						
24	1	02/18/2020	34	1501	3	8.8	0.14	33.68
2 multi 2		H						
24	1	02/19/2020	35	1502	3	8.3	0.28	34.21
2 multi 2		H						
24	1	02/20/2020	36	1503	3	8.6	0	33.84
2 multi 2		H						
24	1	02/21/2020	37	1504	3	8.7	0.01	37.21
2 multi 2		H						
24	1	02/22/2020	38	1505	3	8.5	0.21	35.36
2 multi 2		H						
24	1	02/23/2020	39	1506	3	8.5	0.07	37.12
2 multi 2		H						
24	1	02/24/2020	40	1507	3	8.8	0	35.82
2 multi 2		H						
24	1	02/25/2020	41	1508	3	8.9	0.08	34.96
2 multi 2		H						
24	1	02/26/2020	42	1509	3	9.1	0	37.49
2 multi 2		H						
24	1	02/27/2020	43	1510	3	8.8	0.2	40.43
2 multi 2		H						
24	1	02/28/2020	44	1511	3	8.7	0	39.96
2 multi 2		H						
24	1	02/29/2020	45	1512	3	8.8	0.14	37.18
2 multi 2		H						
24	1	03/01/2020	46	1513	3	8.5	0.24	35.21
2 multi 2		H						
24	1	03/02/2020	47	1514	3	8.4	0.22	38.12
2 multi 2		H						
24	1	03/03/2020	48	1515	3	8.7	0.12	38.83
2 multi 2		H						
24	1	03/04/2020	49	1516	3	8.5	0.15	36.56
2 multi 2		H						
24	1	03/05/2020	50	1517	3	8.7	0.24	41.75
2 multi 2		H						
24	1	03/06/2020	51	1518	3	8.8	0	38.55
2 multi 2		H						
24	1	03/07/2020	52	1519	3	8.9	0	39.16
2 multi 2		H						
24	1	03/08/2020	53	1520	3	8.6	0.19	37.17
2 multi 2		H						
24	1	03/09/2020	54	1521	3	8.8	0.18	40.46
2 multi 2		H						
24	1	03/10/2020	55	1522	3	8.6	0.22	40.2
2 multi 2		H						
24	1	03/11/2020	56	1523	3	8.8	0.22	38.89

2 multi	2	H						
24	1	03/12/2020	57	1524	3	8.8	0.11	41.83
2 multi	2	H						
24	1	03/13/2020	58	1525	3	8.8	0	41.76
2 multi	2	H						
24	1	03/14/2020	59	1526	3	8.5	0.13	42.5
2 multi	2	H						
24	1	03/15/2020	60	1527	3	8.6	0.05	41.49
2 multi	2	H						
24	1	03/16/2020	61	1528	3	8.6	0	40.72
2 multi	2	H						
24	1	03/17/2020	62	1529	3	8.6	0.18	40.27
2 multi	2	H						
24	1	03/18/2020	63	1530	3	8.5	0.27	15.16
2 multi	2	H						
24	1	03/19/2020	64	1531	3	8.3	0.16	11.52
2 multi	2	H						
24	1	03/29/2020	74	1541	3	8.9	0.27	21.74
2 multi	2	H						
24	1	03/30/2020	75	1542	3	9.1	0.25	24.46
2 multi	2	H						
24	1	03/31/2020	76	1543	3	9.3	0.09	26.08
2 multi	2	H						
24	1	04/01/2020	77	1544	3	9.3	0.01	27.03
2 multi	2	H						
24	1	04/02/2020	78	1545	3	9.2	0.19	26.85
2 multi	2	H						
24	1	04/03/2020	79	1546	3	8.8	0.28	30.05
2 multi	2	H						
24	1	04/04/2020	80	1547	3	9.3	0	31.29
2 multi	2	H						
24	1	04/05/2020	81	1548	3	8.8	0.25	31.22
2 multi	2	H						
24	1	04/06/2020	82	1549	3	9.2	0	32.05
2 multi	2	H						
24	1	04/07/2020	83	1550	3	8.9	0	32.02
2 multi	2	H						
24	1	04/08/2020	84	1551	3	9.1	0.08	33.76
2 multi	2	H						
24	1	04/09/2020	85	1552	3	8.8	0	29.56
2 multi	2	H						
26	1	09/18/2019	6	766	1	9.9	0.12	13.6
1 primi	1	H						
26	1	09/19/2019	7	767	1	9.8	0.04	16.38
1 primi	1	H						
26	1	09/20/2019	8	768	1	9.3	0.06	14.33
1 primi	1	H						
26	1	09/21/2019	9	769	1	9.5	0.02	14.93
1 primi	1	H						
26	1	09/22/2019	10	770	1	9.1	0	14.74
1 primi	1	H						
26	1	09/23/2019	11	771	1	9.1	0.15	16.12
1 primi	1	H						
26	1	09/24/2019	12	772	1	8.8	0.13	14.52

1 primi 1		H						
26	1	09/25/2019	13	773	1	10.2	0.21	16.07
1 primi 1		H						
26	1	09/26/2019	14	774	1	9.9	0.15	18.35
1 primi 1		H						
26	1	09/27/2019	15	775	1	9.6	0.04	18.64
1 primi 1		H						
26	1	09/28/2019	16	776	1	9.6	0	18.96
1 primi 1		H						
26	1	09/29/2019	17	777	1	9.3	0.14	19.38
1 primi 1		H						
26	1	09/30/2019	18	778	1	9.3	0.01	20.02
1 primi 1		H						
26	1	10/01/2019	19	779	1	9.5	0.03	19.52
1 primi 1		H						
26	1	10/02/2019	20	780	1	9.2	0.16	18.28
1 primi 1		H						
26	1	10/03/2019	21	781	1	9.3	0.17	18.83
1 primi 1		H						
26	1	10/04/2019	22	782	1	9.1	0.06	18.82
1 primi 2		H						
26	1	10/05/2019	23	783	1	9.1	0.06	19.41
1 primi 2		H						
26	1	10/06/2019	24	784	1	8.5	0.04	17.76
1 primi 2		H						
26	1	10/07/2019	25	785	1	9.2	0.09	21.32
1 primi 2		H						
26	1	10/08/2019	26	786	1	9.1	0.02	21.26
1 primi 2		H						
26	1	10/09/2019	27	787	1	9.1	0.05	21.45
1 primi 2		H						
26	1	10/10/2019	28	788	1	9.1	0.01	20.85
1 primi 2		H						
26	1	10/11/2019	29	789	1	9.5	0.06	21.03
1 primi 2		H						
26	1	11/20/2020	6	1195	2	9.9	0.32	24.66
2 multi 1		H						
26	1	11/21/2020	7	1196	2	9.2	0.36	28.06
2 multi 1		H						
26	1	11/22/2020	8	1197	2	9.6	0.28	27.63
2 multi 1		H						
26	1	11/23/2020	9	1198	2	8.8	0.3	31.41
2 multi 1		H						
26	1	11/24/2020	10	1199	2	10	0.2	30.29
2 multi 1		H						
26	1	11/25/2020	11	1200	2	8.8	0.22	29.17
2 multi 1		H						
26	1	11/26/2020	12	1201	2	9.8	0.12	30.17
2 multi 1		H						
26	1	11/27/2020	13	1202	2	10	0.21	32.11
2 multi 1		H						
26	1	11/28/2020	14	1203	2	9.5	0.08	30.57
2 multi 1		H						
26	1	11/29/2020	15	1204	2	9.6	0.19	28.97

2 multi	1	H						
26	1	11/30/2020	16	1205	2	9.6	0.11	29.99
2 multi	1	H						
26	1	12/01/2020	17	1206	2	9.3	0.18	32.11
2 multi	1	H						
26	1	12/02/2020	18	1207	2	9.5	0.17	33.5
2 multi	1	H						
26	1	12/03/2020	19	1208	2	9.6	0.18	34.67
2 multi	1	H						
26	1	12/04/2020	20	1209	2	9.9	0.06	36.17
2 multi	1	H						
26	1	12/05/2020	21	1210	2	9.9	0.19	34.39
2 multi	1	H						
26	1	12/06/2020	22	1211	2	8.5	0.21	32.64
2 multi	2	H						
26	1	12/07/2020	23	1212	2	9.6	0.08	34.78
2 multi	2	H						
26	1	12/08/2020	24	1213	2	9.5	0.03	35.2
2 multi	2	H						
26	1	12/09/2020	25	1214	2	9.6	0.15	33.97
2 multi	2	H						
26	1	12/10/2020	26	1215	2	9.9	0.03	37.46
2 multi	2	H						
26	1	12/11/2020	27	1216	2	10	0.37	36.47
2 multi	2	H						
26	1	12/12/2020	28	1217	2	9.6	0.14	36.34
2 multi	2	H						
26	1	12/13/2020	29	1218	2	9.9	0.01	36.98
2 multi	2	H						
26	1	12/14/2020	30	1219	2	9.8	0.09	35.79
2 multi	2	H						
26	1	12/15/2020	31	1220	2	9.8	0	34.98
2 multi	2	H						
26	1	12/16/2020	32	1221	2	9.6	0	34.45
2 multi	2	H						
26	1	12/17/2020	33	1222	2	9.9	0.06	38.3
2 multi	2	H						
26	1	12/18/2020	34	1223	2	10	0.17	38.07
2 multi	2	H						
26	1	12/19/2020	35	1224	2	9.9	0.15	35.9
2 multi	2	H						
26	1	12/20/2020	36	1225	2	9.9	0.17	36.27
2 multi	2	H						
26	1	12/21/2020	37	1226	2	10	0.05	36.51
2 multi	2	H						
26	1	12/22/2020	38	1227	2	9.6	0.24	34.57
2 multi	2	H						
26	1	12/23/2020	39	1228	2	9.9	0.02	34.62
2 multi	2	H						
26	1	12/24/2020	40	1229	2	9.5	0.24	34.33
2 multi	2	H						
26	1	12/25/2020	41	1230	2	9.6	0.25	36.13
2 multi	2	H						
26	1	12/26/2020	42	1231	2	9.9	0.14	36.62

2 multi 2		H						
27	1	09/09/2020	6	724	1	10.3	0	26.36
1 primi 1		H						
27	1	09/10/2020	7	725	1	10	0.05	28.89
1 primi 1		H						
27	1	09/11/2020	8	726	1	9.9	0	27.43
1 primi 1		H						
27	1	09/12/2020	9	727	1	10.3	0	27.49
1 primi 1		H						
27	1	09/13/2020	10	728	1	9.9	0	29.55
1 primi 1		H						
27	1	09/14/2020	11	729	1	9.8	0.19	31.84
1 primi 1		H						
27	1	09/15/2020	12	730	1	9.8	0.17	32
1 primi 1		H						
27	1	09/16/2020	13	731	1	9.8	0.21	31.36
1 primi 1		H						
27	1	09/17/2020	14	732	1	9.8	0.28	32.72
1 primi 1		H						
27	1	09/18/2020	15	733	1	9.5	0	33.22
1 primi 1		H						
27	1	09/19/2020	16	734	1	9.8	0.26	34.27
1 primi 1		H						
27	1	09/20/2020	17	735	1	9.8	0.22	35.8
1 primi 1		H						
27	1	09/21/2020	18	736	1	9.8	0.23	35.94
1 primi 1		H						
27	1	09/22/2020	19	737	1	9.5	0.25	36.54
1 primi 1		H						
27	1	09/23/2020	20	738	1	9.5	0.31	37.99
1 primi 1		H						
27	1	09/24/2020	21	739	1	9.5	0.34	36.44
1 primi 1		H						
27	1	09/25/2020	22	740	1	9.3	0.2	38.13
1 primi 2		H						
27	1	09/26/2020	23	741	1	9.6	0.32	38.4
1 primi 2		H						
27	1	09/27/2020	24	742	1	9.6	0.06	36.99
1 primi 2		H						
27	1	09/28/2020	25	743	1	10	0.23	37.55
1 primi 2		H						
27	1	09/29/2020	26	744	1	8.9	0.21	33.42
1 primi 2		H						
27	1	09/30/2020	27	745	1	9.1	0.27	36.08
1 primi 2		H						
27	1	10/01/2020	28	746	1	9.3	0.25	37.98
1 primi 2		H						
27	1	10/02/2020	29	747	1	9.5	0.25	37.58
1 primi 2		H						
27	1	10/03/2020	30	748	1	9.8	0.32	36.77
1 primi 2		H						
27	1	10/04/2020	31	749	1	9.8	0.06	36.91
1 primi 2		H						
27	1	10/05/2020	32	750	1	9.6	0.2	37.78

1 primi 2		H						
27	1	10/06/2020	33	751	1	9.3	0.32	35.59
1 primi 2		H						
27	1	10/07/2020	34	752	1	9.8	0.3	39.03
1 primi 2		H						
27	1	10/08/2020	35	753	1	9.8	0.24	37.18
1 primi 2		H						
27	1	10/09/2020	36	754	1	9.9	0.33	37.15
1 primi 2		H						
27	1	10/10/2020	37	755	1	9.9	0.01	40.59
1 primi 2		H						
27	1	10/11/2020	38	756	1	10	0	39.55
1 primi 2		H						
27	1	10/12/2020	39	757	1	9.6	0.25	38.65
1 primi 2		H						
27	1	10/13/2020	40	758	1	9.8	0.27	40.26
1 primi 2		H						
29	1	06/26/2018	14	799	1	9.3	0.13	27.96
1 primi 1		H						
29	1	06/27/2018	15	800	1	9.9	0	29.35
1 primi 1		H						
29	1	06/28/2018	16	801	1	9.5	0	28.38
1 primi 1		H						
29	1	06/29/2018	17	802	1	9.8	0.01	29.25
1 primi 1		H						
29	1	06/30/2018	18	803	1	9.3	0	28.67
1 primi 1		H						
29	1	07/01/2018	19	804	1	9.9	0.23	29.83
1 primi 1		H						
29	1	07/02/2018	20	805	1	9.9	0	29.03
1 primi 1		H						
29	1	07/03/2018	21	806	1	9.5	0	32.01
1 primi 1		H						
29	1	07/04/2018	22	807	1	9.6	0.07	32.11
1 primi 2		H						
29	1	07/05/2018	23	808	1	9.2	0	31.6
1 primi 2		H						
29	1	07/06/2018	24	809	1	9.8	0	31.75
1 primi 2		H						
29	1	07/07/2018	25	810	1	9.8	0	32.4
1 primi 2		H						
29	1	07/08/2018	26	811	1	9.1	0	32.33
1 primi 2		H						
29	1	07/09/2018	27	812	1	9.6	0	31.93
1 primi 2		H						
29	1	07/10/2018	28	813	1	9.6	0.03	30.41
1 primi 2		H						
29	1	07/11/2018	29	814	1	9.8	0	32.15
1 primi 2		H						
29	1	07/12/2018	30	815	1	9.6	0	30.35
1 primi 2		H						
29	1	07/13/2018	31	816	1	10	0	34.79
1 primi 2		H						
29	1	07/14/2018	32	817	1	9.8	0.08	33.42

1 primi 2 H
29 1 07/15/2018 33 818 1 9.8 0.63 33.96
1 primi 2 H
29 1 07/16/2018 34 819 1 9.8 0 32.28
1 primi 2 H
29 1 07/17/2018 35 820 1 10.7 0.07 33.86
1 primi 2 H
29 1 07/18/2018 36 821 1 10.4 0 32.99
1 primi 2 H
29 1 07/19/2018 37 822 1 10.2 0 35.68
1 primi 2 H
29 1 07/20/2018 38 823 1 10 0 36.06
1 primi 2 H
29 1 07/21/2018 39 824 1 9.6 0 34.12
1 primi 2 H
29 1 07/22/2018 40 825 1 9.3 0 35.63
1 primi 2 H
29 1 07/23/2018 41 826 1 9.3 0 36.14
1 primi 2 H
29 1 07/24/2018 42 827 1 9.8 0 35.38
1 primi 2 H
29 1 07/25/2018 43 828 1 10.2 0 36.78
1 primi 2 H
29 1 07/26/2018 44 829 1 9.6 0 38.41
1 primi 2 H
29 1 07/27/2018 45 830 1 9.5 0 36.15
1 primi 2 H
29 1 07/28/2018 46 831 1 9.8 0.08 34.98
1 primi 2 H
29 1 07/29/2018 47 832 1 9.6 0 39.29
1 primi 2 H
29 1 07/30/2018 48 833 1 9.6 0 35.65
1 primi 2 H
29 1 07/31/2018 49 834 1 9.9 0 36.45
1 primi 2 H
29 1 08/01/2018 50 835 1 9.8 0 34.98
1 primi 2 H
29 1 08/02/2018 51 836 1 9.8 0 36.07
1 primi 2 H
29 1 08/03/2018 52 837 1 10.6 0 34.29
1 primi 2 H
29 1 08/04/2018 53 838 1 10.3 0 34.41
1 primi 2 H
29 1 08/05/2018 54 839 1 9.9 0 34.77
1 primi 2 H
29 1 08/06/2018 55 840 1 9.9 0 35.37
1 primi 2 H
29 1 08/07/2018 56 841 1 10 0 36.2
1 primi 2 H
29 1 08/08/2018 57 842 1 9.3 0.11 34.7
1 primi 2 H
29 1 08/09/2018 58 843 1 9.9 0 38.45
1 primi 2 H
29 1 08/10/2018 59 844 1 10.3 0 37.17

1 primi	2	H						
29	1	08/11/2018	60	845	1	10.2	0	36.43
1 primi	2	H						
29	1	08/12/2018	61	846	1	9.5	0	35.93
1 primi	2	H						
29	1	08/13/2018	62	847	1	9.6	0	37.73
1 primi	2	H						
29	1	08/14/2018	63	848	1	9.9	0	35.53
1 primi	2	H						
29	1	08/15/2018	64	849	1	9.8	0	36.77
1 primi	2	H						
29	1	08/16/2018	65	850	1	9.8	0	37.88
1 primi	2	H						
29	1	08/17/2018	66	851	1	9.9	0	37.15
1 primi	2	H						
29	1	08/18/2018	67	852	1	10	0	37.15
1 primi	2	H						
29	1	08/19/2018	68	853	1	10.6	0	35.52
1 primi	2	H						
29	1	08/20/2018	69	854	1	10	0.09	37.6
1 primi	2	H						
29	1	08/21/2018	70	855	1	10.2	0	36.21
1 primi	2	H						
29	1	08/22/2018	71	856	1	10.3	0.04	36.8
1 primi	2	H						
29	1	08/23/2018	72	857	1	9.9	0	38.46
1 primi	2	H						
29	1	08/24/2018	73	858	1	11.9	0	31.39
1 primi	2	H						
29	1	08/25/2018	74	859	1	10	0	37.49
1 primi	2	H						
29	1	08/26/2018	75	860	1	10	0	36.72
1 primi	2	H						
29	1	08/27/2018	76	861	1	10	0	38.58
1 primi	2	H						
29	1	08/28/2018	77	862	1	10.2	0	37.82
1 primi	2	H						
29	1	08/29/2018	78	863	1	10.6	0	37.56
1 primi	2	H						
29	1	08/30/2018	79	864	1	10.4	0	37.4
1 primi	2	H						
29	1	08/31/2018	80	865	1	10.3	0	36.47
1 primi	2	H						
29	1	09/01/2018	81	866	1	10	0	38.11
1 primi	2	H						
29	1	09/02/2018	82	867	1	10.6	0	35.22
1 primi	2	H						
29	1	09/03/2018	83	868	1	11.9	0.13	37.36
1 primi	2	H						
29	1	09/04/2018	84	869	1	10.7	0.15	36.93
1 primi	2	H						
29	1	09/05/2018	85	870	1	10.7	0	35.95
1 primi	2	H						
29	1	09/06/2018	86	871	1	10.6	0	38.28

1 primi	2	H						
29	1	09/07/2018	87	872	1	10.4	0	36.53
1 primi	2	H						
29	1	09/08/2018	88	873	1	10.6	0.39	33.52
1 primi	2	H						
29	1	09/09/2018	89	874	1	11.1	0	35.2
1 primi	2	H						
29	1	09/10/2018	90	875	1	10.7	0	34.59
1 primi	2	H						
29	1	09/11/2018	91	876	1	10.6	0	35.42
1 primi	2	H						
29	1	09/12/2018	92	877	1	10.4	0	34.81
1 primi	2	H						
29	1	09/13/2018	93	878	1	10.6	0.15	34.63
1 primi	2	H						
29	1	09/14/2018	94	879	1	10.7	0	35.34
1 primi	2	H						
29	1	09/15/2018	95	880	1	10.4	0.11	34.34
1 primi	2	H						
29	1	09/16/2018	96	881	1	10.3	0	36.23
1 primi	2	H						
29	1	09/17/2018	97	882	1	10.6	0	35.23
1 primi	2	H						
29	1	09/18/2018	98	883	1	10.6	0	36.18
1 primi	2	H						
29	1	09/19/2018	99	884	1	10.4	0	35.46
1 primi	2	H						
29	1	09/20/2018	100	885	1	10.4	0	34.81
1 primi	2	H						
29	1	09/21/2018	101	886	1	10.3	0	35.54
1 primi	2	H						
29	1	09/22/2018	102	887	1	11.4	0	32.26
1 primi	2	H						
29	1	09/23/2018	103	888	1	10.7	0	35.86
1 primi	2	H						
29	1	09/24/2018	104	889	1	10.7	0	33.84
1 primi	2	H						
29	1	09/25/2018	105	890	1	10.6	0	32.77
1 primi	2	H						
29	1	09/26/2018	106	891	1	10.3	0.06	35.89
1 primi	2	H						
29	1	09/27/2018	107	892	1	10.2	0	34.49
1 primi	2	H						
29	1	09/28/2018	108	893	1	10.4	0	34.28
1 primi	2	H						
29	1	09/29/2018	109	894	1	10.9	0	33.24
1 primi	2	H						
29	1	09/30/2018	110	895	1	10.4	0	34.12
1 primi	2	H						
29	1	10/01/2018	111	896	1	10.7	0	35.17
1 primi	2	H						
29	1	10/02/2018	112	897	1	10.7	0	35.79
1 primi	2	H						
29	1	10/03/2018	113	898	1	10.4	0	36.01

1 primi	2	H						
29	1	10/04/2018	114	899	1	10.3	0	34.41
1 primi	2	H						
29	1	10/05/2018	115	900	1	10.7	0	34.98
1 primi	2	H						
29	1	10/06/2018	116	901	1	10	0	33.54
1 primi	2	H						
29	1	10/07/2018	117	902	1	11.3	0	34.7
1 primi	2	H						
29	1	10/08/2018	118	903	1	10.6	0	34.69
1 primi	2	H						
29	1	10/09/2018	119	904	1	10.3	0.22	33.65
1 primi	2	H						
29	1	10/10/2018	120	905	1	10.2	0	34.76
1 primi	2	H						
29	1	10/11/2018	121	906	1	10.3	0	36.4
1 primi	2	H						
29	1	10/12/2018	122	907	1	10	0	34.92
1 primi	2	H						
29	1	10/13/2018	123	908	1	10.4	0	33.01
1 primi	2	H						
29	1	10/14/2018	124	909	1	10.4	0	36.3
1 primi	2	H						
29	1	10/15/2018	125	910	1	10.3	0	34.8
1 primi	2	H						
29	1	10/16/2018	126	911	1	10.2	0	34.55
1 primi	2	H						
29	1	10/17/2018	127	912	1	10.3	0	36.84
1 primi	2	H						
29	1	10/18/2018	128	913	1	11.1	0	30.64
1 primi	2	H						
29	1	10/19/2018	129	914	1	11.1	0	35.01
1 primi	2	H						
29	1	10/20/2018	130	915	1	10.7	0	34.45
1 primi	2	H						
29	1	10/21/2018	131	916	1	10.4	0	36.72
1 primi	2	H						
29	1	10/22/2018	132	917	1	10.3	0	34.66
1 primi	2	H						
29	1	10/23/2018	133	918	1	10.3	0	36.06
1 primi	2	H						
29	1	10/24/2018	134	919	1	10.4	0	36.79
1 primi	2	H						
29	1	10/25/2018	135	920	1	10.3	0	34.68
1 primi	2	H						
29	1	10/26/2018	136	921	1	10.3	0	36.58
1 primi	2	H						
29	1	10/27/2018	137	922	1	11.9	0.07	34.23
1 primi	2	H						
29	1	10/28/2018	138	923	1	10.3	0	35.47
1 primi	2	H						
29	1	10/29/2018	139	924	1	10.2	0	37.19
1 primi	2	H						
29	1	10/30/2018	140	925	1	10.6	0	37.5

1 primi	2	H						
29	1	10/31/2018	141	926	1	10.4	0	34.42
1 primi	2	H						
29	1	11/01/2018	142	927	1	12	0	34.78
1 primi	2	H						
29	1	11/02/2018	143	928	1	10.6	0	34.09
1 primi	2	H						
29	1	11/03/2018	144	929	1	10.6	0	38.63
1 primi	2	H						
29	1	11/04/2018	145	930	1	10.7	0	35.4
1 primi	2	H						
29	1	11/05/2018	146	931	1	10.2	0	37.51
1 primi	2	H						
29	1	11/06/2018	147	932	1	10.6	0	34
1 primi	2	H						
29	1	11/07/2018	148	933	1	10	0	35.9
1 primi	2	H						
29	1	11/08/2018	149	934	1	10.3	0	34.59
1 primi	2	H						
29	1	11/09/2018	150	935	1	10.3	0	35.95
1 primi	2	H						
29	1	11/10/2018	151	936	1	10.3	0	35.81
1 primi	2	H						
29	1	11/11/2018	152	937	1	10.3	0	36.36
1 primi	2	H						
29	1	11/12/2018	153	938	1	10.4	0	33.34
1 primi	2	H						
29	1	11/13/2018	154	939	1	10	0	31.36
1 primi	2	H						
29	1	11/14/2018	155	940	1	10.6	0	33.49
1 primi	2	H						
29	1	11/15/2018	156	941	1	10.4	0	32.92
1 primi	2	H						
29	1	09/09/2019	6	1239	2	10.3	0.02	27.11
2 multi	1	H						
29	1	09/10/2019	7	1240	2	9.9	0.03	27.07
2 multi	1	H						
29	1	09/11/2019	8	1241	2	9.9	0.07	28.47
2 multi	1	H						
29	1	09/12/2019	9	1242	2	9.6	0.17	29.47
2 multi	1	H						
29	1	10/19/2020	5	1645	3	10.9	0.28	33.65
2 multi	1	H						
29	1	10/20/2020	6	1646	3	10.7	0.1	35.43
2 multi	1	H						
29	1	10/21/2020	7	1647	3	10.3	0	34.22
2 multi	1	H						
29	1	10/22/2020	8	1648	3	10.2	0.08	40.86
2 multi	1	H						
29	1	10/23/2020	9	1649	3	9.8	0.05	40.91
2 multi	1	H						
29	1	10/24/2020	10	1650	3	10	0.13	43.31
2 multi	1	H						
29	1	10/25/2020	11	1651	3	10.2	0.06	41.14

2 multi	1	H						
29	1	10/26/2020	12	1652	3	9.9	0.09	32.81
2 multi	1	H						
29	1	10/27/2020	13	1653	3	9.9	0.06	41.66
2 multi	1	H						
29	1	10/28/2020	14	1654	3	9.9	0.05	38.07
2 multi	1	H						
29	1	10/29/2020	15	1655	3	10.2	0.13	37.95
2 multi	1	H						
29	1	10/30/2020	16	1656	3	10.2	0.07	39.37
2 multi	1	H						
29	1	10/31/2020	17	1657	3	9.8	0.06	40.47
2 multi	1	H						
29	1	11/01/2020	18	1658	3	9.9	0	38.86
2 multi	1	H						
29	1	11/02/2020	19	1659	3	9.5	0.67	45.42
2 multi	1	H						
29	1	11/03/2020	20	1660	3	9.9	0.19	47.37
2 multi	1	H						
29	1	11/04/2020	21	1661	3	10.2	0.13	45.59
2 multi	1	H						
29	1	11/05/2020	22	1662	3	10.2	0.03	47.55
2 multi	2	H						
29	1	11/06/2020	23	1663	3	9.9	0.04	46.85
2 multi	2	H						
29	1	11/07/2020	24	1664	3	9.6	0	47.68
2 multi	2	H						
29	1	11/08/2020	25	1665	3	10.2	0	47.96
2 multi	2	H						
29	1	11/09/2020	26	1666	3	10.2	0.02	47.78
2 multi	2	H						
29	1	11/10/2020	27	1667	3	9.9	0	45.99
2 multi	2	H						
29	1	11/11/2020	28	1668	3	10.3	0.04	48.17
2 multi	2	H						
29	1	11/12/2020	29	1669	3	10.2	0	44.77
2 multi	2	H						
29	1	11/13/2020	30	1670	3	10	0.18	42.99
2 multi	2	H						
29	1	11/14/2020	31	1671	3	10	0.03	45.87
2 multi	2	H						
29	1	11/15/2020	32	1672	3	10	0.82	46.03
2 multi	2	H						
29	1	11/16/2020	33	1673	3	10.3	0.07	47.8
2 multi	2	H						
29	1	11/17/2020	34	1674	3	10.2	0.1	50.72
2 multi	2	H						
29	1	11/18/2020	35	1675	3	10.7	0.04	47.93
2 multi	2	H						
29	1	11/19/2020	36	1676	3	10.7	0.21	53.56
2 multi	2	H						
29	1	11/20/2020	37	1677	3	10.4	0.85	50.98
2 multi	2	H						
29	1	11/21/2020	38	1678	3	10.4	0.03	49.17

2 multi	2	H						
29	1	11/22/2020	39	1679	3	10.2	0	50.5
2 multi	2	H						
29	1	11/23/2020	40	1680	3	10.2	0.49	53.77
2 multi	2	H						
29	1	11/24/2020	41	1681	3	10.4	0	48.07
2 multi	2	H						
29	1	11/25/2020	42	1682	3	11.1	0.03	49.72
2 multi	2	H						
29	1	11/26/2020	43	1683	3	10.3	0.15	51.01
2 multi	2	H						
29	1	11/27/2020	44	1684	3	10.7	0	50.14
2 multi	2	H						
29	1	11/28/2020	45	1685	3	9.9	0.05	56.51
2 multi	2	H						
29	1	11/29/2020	46	1686	3	10.2	0.04	51.18
2 multi	2	H						
29	1	11/30/2020	47	1687	3	10.2	0.04	47.51
2 multi	2	H						
29	1	12/01/2020	48	1688	3	10	0.06	52.3
2 multi	2	H						
29	1	12/02/2020	49	1689	3	9.9	0.04	51.99
2 multi	2	H						
29	1	12/03/2020	50	1690	3	10	0.12	52.64
2 multi	2	H						
29	1	12/04/2020	51	1691	3	10	0.63	50.74
2 multi	2	H						
29	1	12/05/2020	52	1692	3	10.2	0.37	52.77
2 multi	2	H						
29	1	12/06/2020	53	1693	3	10.6	0.08	51.99
2 multi	2	H						
29	1	12/07/2020	54	1694	3	10.2	0.02	46.88
2 multi	2	H						
29	1	12/08/2020	55	1695	3	10.2	0.09	48.65
2 multi	2	H						
29	1	12/09/2020	56	1696	3	10	0	50.16
2 multi	2	H						
29	1	12/10/2020	57	1697	3	10.9	0.03	47.44
2 multi	2	H						
29	1	12/11/2020	58	1698	3	10.3	0.05	52.24
2 multi	2	H						
29	1	12/12/2020	59	1699	3	10.3	0.05	49.57
2 multi	2	H						
29	1	12/13/2020	60	1700	3	10.9	0.04	47.82
2 multi	2	H						
29	1	12/14/2020	61	1701	3	10.7	0	46.56
2 multi	2	H						
29	1	12/15/2020	62	1702	3	11.1	0	50.35
2 multi	2	H						
29	1	12/16/2020	63	1703	3	10.4	0	48.78
2 multi	2	H						
29	1	12/17/2020	64	1704	3	10.2	0.04	50.09
2 multi	2	H						
29	1	12/18/2020	65	1705	3	11.3	0.1	49.16

2 multi 2		H						
29	1	12/19/2020	66	1706	3	11.7	0.04	45.58
2 multi 2		H						
29	1	12/20/2020	67	1707	3	10.7	0.08	49.5
2 multi 2		H						
29	1	12/21/2020	68	1708	3	11.1	0.05	48.22
2 multi 2		H						
29	1	12/22/2020	69	1709	3	10.4	0	47.62
2 multi 2		H						
29	1	12/23/2020	70	1710	3	11.3	0	47.4
2 multi 2		H						
29	1	12/24/2020	71	1711	3	10.7	0.1	50.7
2 multi 2		H						
29	1	12/25/2020	72	1712	3	10.9	0	46.9
2 multi 2		H						
29	1	12/26/2020	73	1713	3	11.1	0.04	49.22
2 multi 2		H						
29	1	12/27/2020	74	1714	3	11.1	0	43.21
2 multi 2		H						
29	1	12/28/2020	75	1715	3	10.6	0.01	47.7
2 multi 2		H						
29	1	12/29/2020	76	1716	3	10.6	0.17	50.14
2 multi 2		H						
29	1	12/30/2020	77	1717	3	10.3	0	45.87
2 multi 2		H						
29	1	12/31/2020	78	1718	3	11.1	0.05	46.84
2 multi 2		H						
29	1	01/01/2021	79	1719	3	10.7	0.2	51.09
2 multi 2		H						
29	1	01/02/2021	80	1720	3	10.7	0.04	46.02
2 multi 2		H						
29	1	01/03/2021	81	1721	3	10.7	0.08	50.98
2 multi 2		H						
29	1	01/04/2021	82	1722	3	11.7	0	49.15
2 multi 2		H						
29	1	01/05/2021	83	1723	3	10.9	0.07	49.29
2 multi 2		H						
29	1	01/06/2021	84	1724	3	10.7	0.05	49.86
2 multi 2		H						
29	1	01/07/2021	85	1725	3	10.6	0.05	47.57
2 multi 2		H						
29	1	01/08/2021	86	1726	3	12	0.06	46.55
2 multi 2		H						
29	1	01/09/2021	87	1727	3	11.7	0.24	46.46
2 multi 2		H						
29	1	01/10/2021	88	1728	3	11.4	0	43.85
2 multi 2		H						
29	1	01/11/2021	89	1729	3	11.3	0	47.74
2 multi 2		H						
29	1	01/12/2021	90	1730	3	11.4	0.05	49.54
2 multi 2		H						
29	1	01/13/2021	91	1731	3	11.3	0.08	49.2
2 multi 2		H						
29	1	01/14/2021	92	1732	3	10.9	0	47.81

2 multi 2		H						
29	1	01/15/2021	93	1733	3	10.9	0.09	49.22
2 multi 2		H						
29	1	01/16/2021	94	1734	3	10.9	0.04	45.29
2 multi 2		H						
29	1	01/17/2021	95	1735	3	10.7	0	48.13
2 multi 2		H						
29	1	01/18/2021	96	1736	3	10.6	0.02	46.04
2 multi 2		H						
29	1	01/19/2021	97	1737	3	10.4	0.03	50.74
2 multi 2		H						
29	1	01/20/2021	98	1738	3	10.6	0.03	47.21
2 multi 2		H						
29	1	01/21/2021	99	1739	3	11.4	0.03	47.93
2 multi 2		H						
29	1	01/22/2021	100	1740	3	11.4	0.04	46.19
2 multi 2		H						
29	1	01/23/2021	101	1741	3	11.1	0.1	46.04
2 multi 2		H						
29	1	01/24/2021	102	1742	3	11.3	0.05	46.09
2 multi 2		H						
29	1	01/25/2021	103	1743	3	10.9	0	46.76
2 multi 2		H						
29	1	01/26/2021	104	1744	3	10.6	0.02	44.45
2 multi 2		H						
29	1	01/27/2021	105	1745	3	11.4	0.05	44.48
2 multi 2		H						
29	1	01/28/2021	106	1746	3	11.9	0.05	42.76
2 multi 2		H						
29	1	01/29/2021	107	1747	3	11.1	0	47.18
2 multi 2		H						
29	1	01/30/2021	108	1748	3	10.6	0.05	45.68
2 multi 2		H						
29	1	01/31/2021	109	1749	3	10.6	0.02	44.63
2 multi 2		H						
29	1	02/01/2021	110	1750	3	11.1	0.01	45.91
2 multi 2		H						
29	1	02/02/2021	111	1751	3	11.3	0.02	46.78
2 multi 2		H						
29	1	02/03/2021	112	1752	3	10.7	0.45	45.57
2 multi 2		H						
29	1	02/04/2021	113	1753	3	10.7	0	47.46
2 multi 2		H						
29	1	02/05/2021	114	1754	3	11.4	0	44.81
2 multi 2		H						
29	1	02/06/2021	115	1755	3	11.3	0.06	45.33
2 multi 2		H						
29	1	02/07/2021	116	1756	3	10.7	0	45.66
2 multi 2		H						
29	1	02/08/2021	117	1757	3	10.9	0	46.16
2 multi 2		H						
29	1	02/09/2021	118	1758	3	10.7	0.04	43.66
2 multi 2		H						
29	1	02/10/2021	119	1759	3	11.7	0	46.19

2 multi 2		H						
29	1	02/11/2021	120	1760	3	10.9	0.05	42.88
2 multi 2		H						
29	1	02/12/2021	121	1761	3	10.7	0.04	45.44
2 multi 2		H						
29	1	02/13/2021	122	1762	3	10.4	0.05	47.79
2 multi 2		H						
29	1	02/14/2021	123	1763	3	10.6	0	42.75
2 multi 2		H						
29	1	02/15/2021	124	1764	3	11.1	0.02	45.15
2 multi 2		H						
29	1	02/16/2021	125	1765	3	10.7	0.33	47.74
2 multi 2		H						
29	1	02/17/2021	126	1766	3	10.4	0	48.13
2 multi 2		H						
29	1	02/18/2021	127	1767	3	11.3	0	48.16
2 multi 2		H						
29	1	02/19/2021	128	1768	3	10.9	0.02	44.08
2 multi 2		H						
29	1	02/20/2021	129	1769	3	10.9	0.22	45.35
2 multi 2		H						
29	1	02/21/2021	130	1770	3	10.6	0.03	46.36
2 multi 2		H						
29	1	02/22/2021	131	1771	3	10.6	0.13	44.36
2 multi 2		H						
29	1	02/23/2021	132	1772	3	11.1	0.02	47.06
2 multi 2		H						
29	1	02/24/2021	133	1773	3	10.6	0.42	46.21
2 multi 2		H						
29	1	02/25/2021	134	1774	3	10.9	0.04	47.75
2 multi 2		H						
29	1	02/26/2021	135	1775	3	11.3	0.03	47.34
2 multi 2		H						
29	1	02/27/2021	136	1776	3	11.3	0.09	45.1
2 multi 2		H						
29	1	02/28/2021	137	1777	3	10.7	0.02	46.46
2 multi 2		H						
29	1	03/01/2021	138	1778	3	10.2	0	47.46
2 multi 2		H						
29	1	03/02/2021	139	1779	3	11.7	0.05	47.55
2 multi 2		H						
29	1	03/03/2021	140	1780	3	11.9	0.03	43.62
2 multi 2		H						
29	1	03/04/2021	141	1781	3	11.1	0	41.28
2 multi 2		H						
29	1	03/05/2021	142	1782	3	10.7	0	47.17
2 multi 2		H						
29	1	03/06/2021	143	1783	3	10.7	0.02	46.75
2 multi 2		H						
29	1	03/07/2021	144	1784	3	11.1	0	43.95
2 multi 2		H						
29	1	03/08/2021	145	1785	3	12	0.03	44.49
2 multi 2		H						
29	1	03/09/2021	146	1786	3	10.6	0	42.47

2 multi	2	H						
29	1	03/10/2021	147	1787	3	11.3	0.02	46.07
2 multi	2	H						
29	1	03/11/2021	148	1788	3	10.9	0.02	44.75
2 multi	2	H						
29	1	03/12/2021	149	1789	3	10.7	0.02	37.27
2 multi	2	H						
30	1	10/05/2020	6	817	1	10.3	0.26	25.67
1 primi	1	H						
30	1	10/06/2020	7	818	1	10.4	0.25	22.91
1 primi	1	H						
30	1	10/07/2020	8	819	1	10	0.3	29.02
1 primi	1	H						
30	1	10/08/2020	9	820	1	9.9	0.08	24.09
1 primi	1	H						
30	1	10/09/2020	10	821	1	9.9	0.03	28.27
1 primi	1	H						
30	1	10/10/2020	11	822	1	9.9	0.04	27.56
1 primi	1	H						
30	1	10/11/2020	12	823	1	9.9	0	28.4
1 primi	1	H						
30	1	10/12/2020	13	824	1	9.8	0	27.9
1 primi	1	H						
30	1	10/13/2020	14	825	1	9.8	0.02	29.37
1 primi	1	H						
32	1	04/14/2016	5	677	1	9.6	0.02	25.82
1 primi	1	H						
32	1	04/15/2016	6	678	1	9.6	0.2	21.38
1 primi	1	H						
32	1	04/16/2016	7	679	1	9.6	0.02	25.66
1 primi	1	H						
32	1	04/17/2016	8	680	1	9.5	0	26.13
1 primi	1	H						
32	1	04/18/2016	9	681	1	9.2	0	26.17
1 primi	1	H						
32	1	04/19/2016	10	682	1	9.1	0	27.74
1 primi	1	H						
32	1	04/20/2016	11	683	1	9.2	0	26.68
1 primi	1	H						
32	1	04/21/2016	12	684	1	9.2	0.04	24
1 primi	1	H						
32	1	04/22/2016	13	685	1	9.1	0.27	26
1 primi	1	H						
32	1	04/23/2016	14	686	1	9.2	0.04	25.76
1 primi	1	H						
32	1	04/24/2016	15	687	1	9.2	0.03	25.64
1 primi	1	H						
32	1	04/25/2016	16	688	1	9.3	0	27.57
1 primi	1	H						
32	1	04/26/2016	17	689	1	9.3	0.04	30.12
1 primi	1	H						
32	1	04/27/2016	18	690	1	9.3	0.01	28.32
1 primi	1	H						
32	1	04/28/2016	19	691	1	9.6	0.08	33.2

1 primi	1	H						
32	1	04/29/2016	20	692	1	9.6	0.11	28.95
1 primi	1	H						
32	1	04/30/2016	21	693	1	9.8	0.24	32.53
1 primi	1	H						
32	1	05/01/2016	22	694	1	9.5	0	31.18
1 primi	2	H						
32	1	05/02/2016	23	695	1	9.5	0.12	33.2
1 primi	2	H						
32	1	05/03/2016	24	696	1	9.5	0	32.81
1 primi	2	H						
32	1	05/04/2016	25	697	1	9.5	0.03	33.53
1 primi	2	H						
32	1	05/05/2016	26	698	1	9.2	0.05	31.32
1 primi	2	H						
32	1	05/06/2016	27	699	1	9.2	0.03	31.4
1 primi	2	H						
32	1	05/07/2016	28	700	1	9.1	0	32.05
1 primi	2	H						
32	1	05/08/2016	29	701	1	9.3	0	30.39
1 primi	2	H						
32	1	05/09/2016	30	702	1	9.3	0	30.8
1 primi	2	H						
32	1	05/10/2016	31	703	1	9.6	0.01	32.48
1 primi	2	H						
32	1	05/11/2016	32	704	1	9.5	0.08	31.78
1 primi	2	H						
32	1	05/12/2016	33	705	1	9.3	0.31	32.88
1 primi	2	H						
32	1	05/13/2016	34	706	1	9.3	0.33	33.22
1 primi	2	H						
32	1	05/14/2016	35	707	1	9.2	0.31	31.82
1 primi	2	H						
32	1	05/15/2016	36	708	1	8.9	0.21	32.07
1 primi	2	H						
32	1	05/16/2016	37	709	1	8.9	0.23	32.28
1 primi	2	H						
32	1	05/17/2016	38	710	1	8.9	0.06	33
1 primi	2	H						
32	1	05/18/2016	39	711	1	8.9	0.14	32.1
1 primi	2	H						
32	1	05/19/2016	40	712	1	9.2	0.06	33.32
1 primi	2	H						
32	1	05/20/2016	41	713	1	9.1	0.15	34.54
1 primi	2	H						
32	1	05/21/2016	42	714	1	9.2	0.21	33.46
1 primi	2	H						
32	1	05/22/2016	43	715	1	9.3	0	33.81
1 primi	2	H						
32	1	05/23/2016	44	716	1	9.3	0.03	33.85
1 primi	2	H						
32	1	05/24/2016	45	717	1	9.2	0.04	32.96
1 primi	2	H						
32	1	05/25/2016	46	718	1	9.6	0.13	29.34

1 primi 2		H						
32	1	05/26/2016	47	719	1	9.9	0	19.74
1 primi 2		H						
32	1	05/27/2016	48	720	1	8.9	0.14	22.69
1 primi 2		H						
32	1	05/28/2016	49	721	1	8.8	0.28	26.99
1 primi 2		H						
32	1	05/29/2016	50	722	1	8.7	0	26.67
1 primi 2		H						
32	1	05/30/2016	51	723	1	8.6	0.25	26.83
1 primi 2		H						
32	1	05/31/2016	52	724	1	9.5	0.17	27.48
1 primi 2		H						
32	1	06/01/2016	53	725	1	9.2	0	29.41
1 primi 2		H						
32	1	06/02/2016	54	726	1	9.3	0.26	28.77
1 primi 2		H						
32	1	06/03/2016	55	727	1	9.6	0.08	30.98
1 primi 2		H						
32	1	06/04/2016	56	728	1	9.8	0.06	33.5
1 primi 2		H						
32	1	06/05/2016	57	729	1	9.8	0.04	29.8
1 primi 2		H						
32	1	06/06/2016	58	730	1	10	0.2	30.14
1 primi 2		H						
32	1	06/07/2016	59	731	1	9.6	0	31.57
1 primi 2		H						
32	1	06/08/2016	60	732	1	9.6	0.09	29.24
1 primi 2		H						
32	1	06/09/2016	61	733	1	9.5	0.17	29.85
1 primi 2		H						
32	1	06/10/2016	62	734	1	9.5	0.1	28.47
1 primi 2		H						
32	1	06/11/2016	63	735	1	9.5	0	29.3
1 primi 2		H						
32	1	06/12/2016	64	736	1	9.5	0	31.03
1 primi 2		H						
32	1	06/13/2016	65	737	1	9.6	0	32.03
1 primi 2		H						
32	1	06/14/2016	66	738	1	9.6	0.03	31.47
1 primi 2		H						
32	1	06/15/2016	67	739	1	9.9	0.05	32.79
1 primi 2		H						
32	1	06/16/2016	68	740	1	9.5	0.05	30.1
1 primi 2		H						
32	1	06/17/2016	69	741	1	9.2	0.19	31.23
1 primi 2		H						
32	1	06/18/2016	70	742	1	9.8	0.19	31.57
1 primi 2		H						
32	1	06/19/2016	71	743	1	9.9	0.11	31.75
1 primi 2		H						
32	1	06/20/2016	72	744	1	9.6	0.16	30.69
1 primi 2		H						
32	1	06/21/2016	73	745	1	9.8	0.03	31.69

1 primi 2		H						
32	1	06/22/2016	74	746	1	9.6	0.12	31.03
1 primi 2		H						
32	1	06/23/2016	75	747	1	10	0	32.93
1 primi 2		H						
32	1	06/24/2016	76	748	1	9.6	0.04	34.13
1 primi 2		H						
32	1	06/25/2016	77	749	1	9.6	0	31.35
1 primi 2		H						
32	1	06/26/2016	78	750	1	9.3	0.04	31.28
1 primi 2		H						
32	1	06/27/2016	79	751	1	9.8	0	31.34
1 primi 2		H						
32	1	06/28/2016	80	752	1	9.8	0	31.16
1 primi 2		H						
32	1	06/29/2016	81	753	1	9.6	0.03	34.73
1 primi 2		H						
32	1	06/30/2016	82	754	1	9.8	0.1	32.78
1 primi 2		H						
32	1	07/01/2016	83	755	1	10.2	0.12	32.33
1 primi 2		H						
32	1	07/02/2016	84	756	1	10	0.08	33.68
1 primi 2		H						
32	1	07/03/2016	85	757	1	9.9	0.06	31.71
1 primi 2		H						
32	1	07/04/2016	86	758	1	9.8	0.14	33.01
1 primi 2		H						
32	1	07/05/2016	87	759	1	9.6	0	33.56
1 primi 2		H						
32	1	07/06/2016	88	760	1	9.8	0.04	33.73
1 primi 2		H						
32	1	07/07/2016	89	761	1	9.8	0.02	32.64
1 primi 2		H						
32	1	07/08/2016	90	762	1	9.9	0.09	33.06
1 primi 2		H						
32	1	07/09/2016	91	763	1	9.9	0	34.3
1 primi 2		H						
32	1	07/10/2016	92	764	1	9.6	0.15	33.12
1 primi 2		H						
32	1	07/11/2016	93	765	1	9.8	0	30.86
1 primi 2		H						
32	1	07/12/2016	94	766	1	9.5	0.02	32.93
1 primi 2		H						
32	1	07/13/2016	95	767	1	9.5	0.12	32.33
1 primi 2		H						
32	1	07/14/2016	96	768	1	9.3	0.11	31.35
1 primi 2		H						
32	1	07/15/2016	97	769	1	9.8	0.11	35.5
1 primi 2		H						
32	1	07/16/2016	98	770	1	9.3	0.06	31.93
1 primi 2		H						
32	1	07/17/2016	99	771	1	9.9	0.12	32.12
1 primi 2		H						
32	1	07/18/2016	100	772	1	9.6	0.09	33.19

1 primi	2	H						
32	1	07/19/2016	101	773	1	9.6	0.01	34.3
1 primi	2	H						
32	1	07/20/2016	102	774	1	9.6	0	33.45
1 primi	2	H						
32	1	07/21/2016	103	775	1	9.5	0.05	32.29
1 primi	2	H						
32	1	07/22/2016	104	776	1	9.5	0.1	33.51
1 primi	2	H						
32	1	07/23/2016	105	777	1	9.6	0	32.77
1 primi	2	H						
32	1	07/24/2016	106	778	1	9.8	0.09	33.34
1 primi	2	H						
32	1	07/25/2016	107	779	1	9.8	0	31.54
1 primi	2	H						
32	1	04/21/2017	4	1049	2	9.6	0.13	29.71
2 multi	1	H						
32	1	04/22/2017	5	1050	2	8.8	0.52	32.22
2 multi	1	H						
32	1	04/23/2017	6	1051	2	8.9	0.09	32.14
2 multi	1	H						
32	1	04/24/2017	7	1052	2	9.5	0	31.71
2 multi	1	H						
32	1	04/25/2017	8	1053	2	9.6	0	36.31
2 multi	1	H						
32	1	04/26/2017	9	1054	2	9.9	0.01	38.22
2 multi	1	H						
32	1	04/27/2017	10	1055	2	9.8	0.04	37.94
2 multi	1	H						
32	1	04/28/2017	11	1056	2	9.8	0.08	39.26
2 multi	1	H						
32	1	04/29/2017	12	1057	2	9.9	0.07	41.2
2 multi	1	H						
32	1	04/30/2017	13	1058	2	9.9	0	38.26
2 multi	1	H						
32	1	05/01/2017	14	1059	2	9.9	0.06	41.28
2 multi	1	H						
32	1	05/02/2017	15	1060	2	9.9	0	43.24
2 multi	1	H						
32	1	05/03/2017	16	1061	2	9.5	0.04	41.38
2 multi	1	H						
32	1	05/04/2017	17	1062	2	10	0.04	46.06
2 multi	1	H						
32	1	05/05/2017	18	1063	2	9.9	0.05	46.13
2 multi	1	H						
32	1	05/06/2017	19	1064	2	9.6	0.08	44.54
2 multi	1	H						
32	1	05/07/2017	20	1065	2	9.6	0.23	44.23
2 multi	1	H						
32	1	05/08/2017	21	1066	2	9.8	0.06	43.83
2 multi	1	H						
32	1	05/09/2017	22	1067	2	9.6	0.19	44.89
2 multi	2	H						
32	1	05/10/2017	23	1068	2	9.5	0.09	42.5

2 multi	2	H						
32	1	05/11/2017	24	1069	2	9.5	0.06	47.52
2 multi	2	H						
32	1	05/12/2017	25	1070	2	9.9	0.16	48.11
2 multi	2	H						
32	1	05/13/2017	26	1071	2	9.8	0	48.58
2 multi	2	H						
32	1	05/14/2017	27	1072	2	9.8	0.09	49.75
2 multi	2	H						
32	1	05/15/2017	28	1073	2	10	0	48.47
2 multi	2	H						
32	1	05/16/2017	29	1074	2	9.9	0.2	47.69
2 multi	2	H						
32	1	05/17/2017	30	1075	2	9.5	0.08	44.54
2 multi	2	H						
32	1	05/18/2017	31	1076	2	9.3	0.01	45.82
2 multi	2	H						
32	1	05/19/2017	32	1077	2	8.9	0.03	42.74
2 multi	2	H						
32	1	05/20/2017	33	1078	2	8.9	0.07	41.4
2 multi	2	H						
32	1	05/21/2017	34	1079	2	9.5	0.13	46.05
2 multi	2	H						
32	1	05/22/2017	35	1080	2	9.6	0.09	47.52
2 multi	2	H						
32	1	05/23/2017	36	1081	2	9.8	0.13	48.56
2 multi	2	H						
32	1	05/24/2017	37	1082	2	9.8	0.19	49.11
2 multi	2	H						
32	1	05/25/2017	38	1083	2	9.8	0.1	49
2 multi	2	H						
32	1	05/26/2017	39	1084	2	9.9	0.19	49.16
2 multi	2	H						
32	1	05/27/2017	40	1085	2	10	0	50.9
2 multi	2	H						
32	1	05/28/2017	41	1086	2	10.2	0	49.82
2 multi	2	H						
32	1	05/29/2017	42	1087	2	10.9	0.76	49.75
2 multi	2	H						
32	1	05/30/2017	43	1088	2	10.2	0.02	50.84
2 multi	2	H						
32	1	05/31/2017	44	1089	2	10	0.05	48.51
2 multi	2	H						
32	1	06/01/2017	45	1090	2	9.9	0	49.75
2 multi	2	H						
32	1	06/02/2017	46	1091	2	9.8	0.01	51.51
2 multi	2	H						
32	1	06/03/2017	47	1092	2	9.9	0.04	49.12
2 multi	2	H						
32	1	06/04/2017	48	1093	2	9.5	0.11	48.01
2 multi	2	H						
32	1	06/05/2017	49	1094	2	9.5	0	49.95
2 multi	2	H						
32	1	06/06/2017	50	1095	2	9.8	0	51.13

2 multi 2		H						
32	1	06/07/2017	51	1096	2	9.6	0.14	46.87
2 multi 2		H						
32	1	06/08/2017	52	1097	2	9.8	0	49.32
2 multi 2		H						
32	1	06/09/2017	53	1098	2	9.5	0.32	46.48
2 multi 2		H						
32	1	06/10/2017	54	1099	2	9.1	0.06	42.54
2 multi 2		H						
32	1	06/11/2017	55	1100	2	9.2	0	47.83
2 multi 2		H						
32	1	06/12/2017	56	1101	2	9.5	0	49.33
2 multi 2		H						
32	1	06/13/2017	57	1102	2	9.2	0.1	46.38
2 multi 2		H						
32	1	06/14/2017	58	1103	2	9.6	0.1	47.01
2 multi 2		H						
32	1	06/15/2017	59	1104	2	9.6	0.06	46.16
2 multi 2		H						
32	1	06/16/2017	60	1105	2	9.8	0.04	47.95
2 multi 2		H						
32	1	06/17/2017	61	1106	2	9.9	0.17	48.49
2 multi 2		H						
32	1	06/18/2017	62	1107	2	9.6	0.16	47.74
2 multi 2		H						
32	1	06/19/2017	63	1108	2	9.6	0	49.29
2 multi 2		H						
32	1	06/20/2017	64	1109	2	9.3	0	44.48
2 multi 2		H						
32	1	06/21/2017	65	1110	2	9.2	0.02	41.07
2 multi 2		H						
32	1	06/22/2017	66	1111	2	9.8	0.05	43.45
2 multi 2		H						
32	1	06/23/2017	67	1112	2	9.9	0.03	46.62
2 multi 2		H						
32	1	06/24/2017	68	1113	2	9.8	0	46.53
2 multi 2		H						
32	1	06/25/2017	69	1114	2	9.9	0	48.25
2 multi 2		H						
32	1	06/26/2017	70	1115	2	9.8	0.01	46.18
2 multi 2		H						
32	1	06/27/2017	71	1116	2	9.6	0	48.34
2 multi 2		H						
32	1	06/28/2017	72	1117	2	9.9	0.18	47.59
2 multi 2		H						
32	1	06/29/2017	73	1118	2	9.9	0	46.22
2 multi 2		H						
32	1	06/30/2017	74	1119	2	9.9	0	48.3
2 multi 2		H						
32	1	07/01/2017	75	1120	2	9.8	0.06	47.06
2 multi 2		H						
32	1	07/02/2017	76	1121	2	9.9	0.08	48.5
2 multi 2		H						
32	1	07/03/2017	77	1122	2	9.8	0	47.17

2 multi 2		H						
32	1	07/04/2017	78	1123	2	9.3	0.17	43.77
2 multi 2		H						
32	1	07/05/2017	79	1124	2	9.5	0.14	46.8
2 multi 2		H						
32	1	07/06/2017	80	1125	2	9.8	0.14	47.7
2 multi 2		H						
32	1	07/07/2017	81	1126	2	9.3	0.1	43.3
2 multi 2		H						
32	1	07/08/2017	82	1127	2	9.6	0.13	44.82
2 multi 2		H						
32	1	07/09/2017	83	1128	2	9.8	0	51.51
2 multi 2		H						
32	1	07/10/2017	84	1129	2	9.9	0.1	51.88
2 multi 2		H						
32	1	07/11/2017	85	1130	2	9.8	0.11	45.46
2 multi 2		H						
32	1	07/12/2017	86	1131	2	9.9	0.15	45.67
2 multi 2		H						
32	1	07/13/2017	87	1132	2	10	0.05	45.21
2 multi 2		H						
32	1	07/14/2017	88	1133	2	10	0.06	45.48
2 multi 2		H						
32	1	07/15/2017	89	1134	2	9.9	0	45.63
2 multi 2		H						
32	1	07/16/2017	90	1135	2	9.9	0.04	46.12
2 multi 2		H						
32	1	07/17/2017	91	1136	2	9.9	0	50.23
2 multi 2		H						
32	1	07/18/2017	92	1137	2	10	0	44.79
2 multi 2		H						
32	1	07/19/2017	93	1138	2	9.9	0.12	45.21
2 multi 2		H						
32	1	07/20/2017	94	1139	2	9.9	0	45.75
2 multi 2		H						
32	1	07/21/2017	95	1140	2	9.9	0.1	45.69
2 multi 2		H						
32	1	07/22/2017	96	1141	2	9.8	0.14	42.9
2 multi 2		H						
32	1	07/23/2017	97	1142	2	9.8	0	41.18
2 multi 2		H						
32	1	07/24/2017	98	1143	2	9.9	0.17	44.21
2 multi 2		H						
32	1	07/25/2017	99	1144	2	9.9	0	43.83
2 multi 2		H						
32	1	07/26/2017	100	1145	2	10.2	0	47.32
2 multi 2		H						
32	1	07/27/2017	101	1146	2	9.9	0.05	46.4
2 multi 2		H						
32	1	07/28/2017	102	1147	2	9.8	0.05	46.15
2 multi 2		H						
32	1	07/29/2017	103	1148	2	9.9	0.04	47.58
2 multi 2		H						
32	1	07/30/2017	104	1149	2	9.9	0.02	46.28

2 multi 2		H						
32	1	07/31/2017	105	1150	2	9.9	0.14	46.88
2 multi 2		H						
32	1	08/01/2017	106	1151	2	10	0	45.1
2 multi 2		H						
32	1	08/02/2017	107	1152	2	9.6	0	40.72
2 multi 2		H						
32	1	08/03/2017	108	1153	2	9.8	0	40.37
2 multi 2		H						
32	1	08/04/2017	109	1154	2	9.6	0	38
2 multi 2		H						
32	1	08/05/2017	110	1155	2	9.8	0	38.64
2 multi 2		H						
32	1	08/06/2017	111	1156	2	9.8	0.11	38.17
2 multi 2		H						
32	1	08/07/2017	112	1157	2	9.6	0.57	38.82
2 multi 2		H						
32	1	08/08/2017	113	1158	2	9.9	0.03	37.98
2 multi 2		H						
32	1	08/09/2017	114	1159	2	9.9	0	38.21
2 multi 2		H						
32	1	08/10/2017	115	1160	2	9.9	0.1	39.95
2 multi 2		H						
32	1	08/11/2017	116	1161	2	10.2	0.17	40.29
2 multi 2		H						
32	1	08/12/2017	117	1162	2	10	0.09	41.45
2 multi 2		H						
32	1	08/13/2017	118	1163	2	10.2	0.14	44.11
2 multi 2		H						
32	1	08/14/2017	119	1164	2	10	0.12	41.32
2 multi 2		H						
32	1	08/15/2017	120	1165	2	9.8	0.05	43.66
2 multi 2		H						
32	1	08/16/2017	121	1166	2	10.2	0.03	45.61
2 multi 2		H						
32	1	08/17/2017	122	1167	2	9.9	0	46
2 multi 2		H						
32	1	08/18/2017	123	1168	2	9.8	0	43.03
2 multi 2		H						
32	1	08/19/2017	124	1169	2	9.3	0.17	39.62
2 multi 2		H						
32	1	08/20/2017	125	1170	2	9.3	0	41.96
2 multi 2		H						
32	1	08/21/2017	126	1171	2	9.5	0.17	40.75
2 multi 2		H						
32	1	08/22/2017	127	1172	2	9.6	0.01	41.63
2 multi 2		H						
32	1	08/23/2017	128	1173	2	9.8	0	40.28
2 multi 2		H						
32	1	08/24/2017	129	1174	2	9.8	0.2	41.83
2 multi 2		H						
32	1	08/25/2017	130	1175	2	9.9	0.11	40.04
2 multi 2		H						
32	1	08/26/2017	131	1176	2	9.5	0	39.68

2 multi	2	H						
32	1	08/27/2017	132	1177	2	9.9	0.2	40.94
2 multi	2	H						
32	1	08/28/2017	133	1178	2	9.9	0.16	39.55
2 multi	2	H						
32	1	08/29/2017	134	1179	2	9.9	0.23	40.66
2 multi	2	H						
32	1	08/30/2017	135	1180	2	9.8	0	38.51
2 multi	2	H						
32	1	08/31/2017	136	1181	2	9.6	0.15	40.13
2 multi	2	H						
32	1	09/01/2017	137	1182	2	9.2	0	37.19
2 multi	2	H						
32	1	09/02/2017	138	1183	2	12	0.43	36.86
2 multi	2	H						
32	1	09/03/2017	139	1184	2	9.5	0.05	37.94
2 multi	2	H						
32	1	09/04/2017	140	1185	2	9.3	0	39.81
2 multi	2	H						
32	1	09/05/2017	141	1186	2	9.5	0.06	38.35
2 multi	2	H						
32	1	09/06/2017	142	1187	2	9.6	0.38	39.73
2 multi	2	H						
32	1	09/07/2017	143	1188	2	9.6	0	39.88
2 multi	2	H						
32	1	09/08/2017	144	1189	2	9.5	0	38.14
2 multi	2	H						
32	1	09/09/2017	145	1190	2	9.5	0	37.14
2 multi	2	H						
32	1	09/10/2017	146	1191	2	9.9	0	37.73
2 multi	2	H						
32	1	09/11/2017	147	1192	2	9.9	0.02	39.61
2 multi	2	H						
32	1	09/12/2017	148	1193	2	9.9	0	41.36
2 multi	2	H						
32	1	09/13/2017	149	1194	2	9.9	0.04	38.2
2 multi	2	H						
32	1	09/14/2017	150	1195	2	9.9	0.02	40.99
2 multi	2	H						
32	1	09/15/2017	151	1196	2	9.6	0.01	39.92
2 multi	2	H						
32	1	09/16/2017	152	1197	2	9.8	0.17	41.72
2 multi	2	H						
32	1	09/17/2017	153	1198	2	9.5	0.01	40.75
2 multi	2	H						
32	1	09/18/2017	154	1199	2	9.6	0	43.99
2 multi	2	H						
32	1	09/19/2017	155	1200	2	9.6	0	40.93
2 multi	2	H						
32	1	09/20/2017	156	1201	2	9.5	0.1	42.24
2 multi	2	H						
32	1	09/21/2017	157	1202	2	9.6	0.03	41.46
2 multi	2	H						
32	1	09/22/2017	158	1203	2	9.8	0	40.08

2 multi	2	H						
32	1	09/23/2017	159	1204	2	9.3	0	37.95
2 multi	2	H						
32	1	09/24/2017	160	1205	2	9.1	0	34.06
2 multi	2	H						
32	1	09/25/2017	161	1206	2	9.5	0	37.58
2 multi	2	H						
32	1	09/26/2017	162	1207	2	9.6	0	40.05
2 multi	2	H						
32	1	09/27/2017	163	1208	2	9.5	0	37.05
2 multi	2	H						
32	1	09/28/2017	164	1209	2	9.2	0.11	36.79
2 multi	2	H						
32	1	09/29/2017	165	1210	2	9.3	0	36.02
2 multi	2	H						
32	1	09/30/2017	166	1211	2	9.3	0.05	36.07
2 multi	2	H						
32	1	10/01/2017	167	1212	2	9.2	0.04	36.62
2 multi	2	H						
32	1	10/02/2017	168	1213	2	9.3	0	34.57
2 multi	2	H						
32	1	10/03/2017	169	1214	2	9.6	0	35.88
2 multi	2	H						
32	1	10/04/2017	170	1215	2	9.5	0.19	38.13
2 multi	2	H						
32	1	10/05/2017	171	1216	2	9.3	0	36.35
2 multi	2	H						
32	1	10/06/2017	172	1217	2	9.3	0.09	33.39
2 multi	2	H						
32	1	10/07/2017	173	1218	2	9.5	0.02	35.13
2 multi	2	H						
32	1	10/08/2017	174	1219	2	9.2	0	34.05
2 multi	2	H						
32	1	10/09/2017	175	1220	2	9.1	0.05	31.61
2 multi	2	H						
32	1	10/10/2017	176	1221	2	9.2	0	35.27
2 multi	2	H						
32	1	03/24/2018	6	1386	3	10	0.16	36.74
2 multi	1	H						
32	1	03/25/2018	7	1387	3	9.3	0.21	31.1
2 multi	1	H						
32	1	03/26/2018	8	1388	3	9.3	0.01	35.99
2 multi	1	H						
32	1	03/27/2018	9	1389	3	9.5	0.11	36.93
2 multi	1	H						
32	1	03/28/2018	10	1390	3	11.1	0.01	39.59
2 multi	1	H						
32	1	03/29/2018	11	1391	3	9.9	0.11	37.31
2 multi	1	H						
32	1	03/30/2018	12	1392	3	9.9	0.07	43.53
2 multi	1	H						
32	1	03/31/2018	13	1393	3	9.3	0.12	42.18
2 multi	1	H						
32	1	04/01/2018	14	1394	3	8.9	0.07	40.39

2 multi	1	H						
32	1	04/02/2018	15	1395	3	9.6	0.11	41.86
2 multi	1	H						
32	1	04/03/2018	16	1396	3	9.5	0.08	43.17
2 multi	1	H						
32	1	04/04/2018	17	1397	3	9.5	0.1	44.89
2 multi	1	H						
32	1	04/05/2018	18	1398	3	9.6	0.08	44.09
2 multi	1	H						
32	1	04/06/2018	19	1399	3	9.5	0	43.27
2 multi	1	H						
32	1	04/07/2018	20	1400	3	9.3	0	48.23
2 multi	1	H						
32	1	04/08/2018	21	1401	3	9.6	0.08	46.37
2 multi	1	H						
32	1	04/09/2018	22	1402	3	9.6	0.09	48.05
2 multi	2	H						
32	1	04/10/2018	23	1403	3	9.6	0.08	50.61
2 multi	2	H						
32	1	04/11/2018	24	1404	3	9.8	0.08	47.22
2 multi	2	H						
32	1	04/12/2018	25	1405	3	9.6	0	53.2
2 multi	2	H						
32	1	04/13/2018	26	1406	3	9.6	0.12	49.37
2 multi	2	H						
32	1	04/14/2018	27	1407	3	9.8	0	48.54
2 multi	2	H						
32	1	04/15/2018	28	1408	3	9.1	0	48.47
2 multi	2	H						
32	1	04/16/2018	29	1409	3	9.8	0.19	53.61
2 multi	2	H						
32	1	04/17/2018	30	1410	3	9.8	0.2	49.83
2 multi	2	H						
32	1	04/18/2018	31	1411	3	0	0.12	24.82
2 multi	2	H						
32	1	04/19/2018	32	1412	3	0	0.07	22.8
2 multi	2	H						
32	1	04/20/2018	33	1413	3	9.6	0.25	49.71
2 multi	2	H						
32	1	04/21/2018	34	1414	3	9.8	0.04	51.17
2 multi	2	H						
32	1	04/22/2018	35	1415	3	9.6	0.06	52.03
2 multi	2	H						
32	1	04/23/2018	36	1416	3	9.5	0.22	51.97
2 multi	2	H						
32	1	04/24/2018	37	1417	3	9.8	0.25	54.05
2 multi	2	H						
32	1	04/25/2018	38	1418	3	9.8	0.04	52.4
2 multi	2	H						
32	1	04/26/2018	39	1419	3	9.8	0.05	51.27
2 multi	2	H						
32	1	04/27/2018	40	1420	3	9.5	0.22	43.5
2 multi	2	H						
32	1	04/28/2018	41	1421	3	9.5	0.27	50.71

2 multi	2	H						
32	1	04/29/2018	42	1422	3	9.6	0.05	47.73
2 multi	2	H						
32	1	04/30/2018	43	1423	3	9.5	0.21	48.7
2 multi	2	H						
32	1	05/01/2018	44	1424	3	9.3	0.18	46.91
2 multi	2	H						
32	1	05/02/2018	45	1425	3	9.8	0.15	53.35
2 multi	2	H						
32	1	05/03/2018	46	1426	3	9.5	0.03	49.92
2 multi	2	H						
32	1	05/04/2018	47	1427	3	9.5	0	47.93
2 multi	2	H						
32	1	05/05/2018	48	1428	3	9.6	0.08	48.04
2 multi	2	H						
32	1	05/06/2018	49	1429	3	9.6	0.2	51.58
2 multi	2	H						
32	1	05/07/2018	50	1430	3	9.5	0.09	48.28
2 multi	2	H						
32	1	05/08/2018	51	1431	3	9.9	0.15	49.07
2 multi	2	H						
32	1	05/09/2018	52	1432	3	9.9	0.21	51.07
2 multi	2	H						
32	1	05/10/2018	53	1433	3	9.6	0.16	50.82
2 multi	2	H						
32	1	05/11/2018	54	1434	3	9.8	0.04	50.87
2 multi	2	H						
32	1	05/12/2018	55	1435	3	9.9	0.16	53.14
2 multi	2	H						
32	1	05/13/2018	56	1436	3	9.8	0.08	52.42
2 multi	2	H						
32	1	05/14/2018	57	1437	3	9.6	0.14	49.89
2 multi	2	H						
32	1	05/15/2018	58	1438	3	9.6	0.14	48.76
2 multi	2	H						
32	1	05/16/2018	59	1439	3	9.6	0.17	47.92
2 multi	2	H						
32	1	05/17/2018	60	1440	3	9.6	0.14	44.28
2 multi	2	H						
32	1	05/18/2018	61	1441	3	9.9	0.07	47.18
2 multi	2	H						
32	1	05/19/2018	62	1442	3	9.9	0.16	47.88
2 multi	2	H						
32	1	05/20/2018	63	1443	3	10	0.17	50.32
2 multi	2	H						
32	1	05/21/2018	64	1444	3	9.8	0.11	48.55
2 multi	2	H						
32	1	05/22/2018	65	1445	3	9.6	0	44.89
2 multi	2	H						
32	1	05/23/2018	66	1446	3	9.6	0.14	48.95
2 multi	2	H						
32	1	05/24/2018	67	1447	3	9.6	0.21	48.98
2 multi	2	H						
32	1	05/25/2018	68	1448	3	9.9	0.19	53.03

2 multi 2		H						
32	1	05/26/2018	69	1449	3	9.5	0.07	47.75
2 multi 2		H						
32	1	05/27/2018	70	1450	3	9.6	0.05	50.92
2 multi 2		H						
32	1	05/28/2018	71	1451	3	9.9	0	46.67
2 multi 2		H						
32	1	05/29/2018	72	1452	3	9.9	0.03	46.56
2 multi 2		H						
32	1	05/30/2018	73	1453	3	9.8	0.05	49.07
2 multi 2		H						
32	1	05/31/2018	74	1454	3	9.9	0.13	49.77
2 multi 2		H						
32	1	06/01/2018	75	1455	3	9.8	0.2	45.99
2 multi 2		H						
32	1	06/02/2018	76	1456	3	9.8	0.02	48.57
2 multi 2		H						
32	1	06/03/2018	77	1457	3	9.8	0	44.96
2 multi 2		H						
32	1	06/04/2018	78	1458	3	9.8	0.08	42.07
2 multi 2		H						
32	1	06/05/2018	79	1459	3	9.8	0.06	47.09
2 multi 2		H						
32	1	06/06/2018	80	1460	3	9.6	0.05	44.21
2 multi 2		H						
32	1	06/07/2018	81	1461	3	9.8	0.16	45.62
2 multi 2		H						
32	1	06/08/2018	82	1462	3	9.8	0.2	45.28
2 multi 2		H						
32	1	06/09/2018	83	1463	3	10	0.07	44.35
2 multi 2		H						
32	1	06/10/2018	84	1464	3	9.9	0.06	45.24
2 multi 2		H						
32	1	06/11/2018	85	1465	3	10	0.12	45.63
2 multi 2		H						
32	1	06/12/2018	86	1466	3	9.9	0.13	43.62
2 multi 2		H						
32	1	06/13/2018	87	1467	3	10.2	0.13	46.67
2 multi 2		H						
32	1	06/14/2018	88	1468	3	10.3	0.28	45.9
2 multi 2		H						
32	1	06/15/2018	89	1469	3	10	0.06	44.3
2 multi 2		H						
32	1	06/16/2018	90	1470	3	10	0.17	42.73
2 multi 2		H						
32	1	06/17/2018	91	1471	3	10	0.09	42.51
2 multi 2		H						
32	1	06/18/2018	92	1472	3	10	0.19	40.79
2 multi 2		H						
32	1	06/19/2018	93	1473	3	10.2	0	41.29
2 multi 2		H						
32	1	06/20/2018	94	1474	3	9.9	0.12	40.52
2 multi 2		H						
32	1	06/21/2018	95	1475	3	10	0.03	38.82

2 multi 2		H						
32	1	06/22/2018	96	1476	3	10.2	0.24	40.35
2 multi 2		H						
32	1	06/23/2018	97	1477	3	10.2	0.08	41.96
2 multi 2		H						
32	1	06/24/2018	98	1478	3	10	0.1	42.54
2 multi 2		H						
32	1	06/25/2018	99	1479	3	10	0.16	43.84
2 multi 2		H						
32	1	06/26/2018	100	1480	3	9.9	0.09	42.27
2 multi 2		H						
32	1	06/27/2018	101	1481	3	10	0	44.46
2 multi 2		H						
32	1	06/28/2018	102	1482	3	10.2	0.24	42.75
2 multi 2		H						
32	1	06/29/2018	103	1483	3	10.2	0	46.11
2 multi 2		H						
32	1	06/30/2018	104	1484	3	10	0.06	42.12
2 multi 2		H						
32	1	07/01/2018	105	1485	3	9.9	0.16	39.03
2 multi 2		H						
32	1	07/02/2018	106	1486	3	10.2	0.15	41.44
2 multi 2		H						
32	1	07/03/2018	107	1487	3	10.2	0.07	40.33
2 multi 2		H						
32	1	07/04/2018	108	1488	3	10	0.15	39.95
2 multi 2		H						
32	1	07/05/2018	109	1489	3	9.9	0.05	38.5
2 multi 2		H						
32	1	07/06/2018	110	1490	3	10.2	0.19	37.29
2 multi 2		H						
32	1	07/07/2018	111	1491	3	10	0.03	38.27
2 multi 2		H						
32	1	07/08/2018	112	1492	3	10.2	0.23	38.3
2 multi 2		H						
32	1	07/09/2018	113	1493	3	10.2	0.21	42.31
2 multi 2		H						
32	1	07/10/2018	114	1494	3	10.3	0.2	40.41
2 multi 2		H						
32	1	07/11/2018	115	1495	3	9.9	0.01	42.6
2 multi 2		H						
32	1	07/12/2018	116	1496	3	10.2	0.18	41.39
2 multi 2		H						
32	1	07/13/2018	117	1497	3	10	0	40.48
2 multi 2		H						
32	1	07/14/2018	118	1498	3	10.2	0.14	40.68
2 multi 2		H						
32	1	07/15/2018	119	1499	3	10.2	0.12	37.04
2 multi 2		H						
32	1	07/16/2018	120	1500	3	10.2	0.15	39.92
2 multi 2		H						
32	1	07/17/2018	121	1501	3	10.3	0.05	39.47
2 multi 2		H						
32	1	07/18/2018	122	1502	3	10.3	0.03	38.65

2 multi 2		H						
32	1	07/19/2018	123	1503	3	9.9	0.14	40.2
2 multi 2		H						
32	1	07/20/2018	124	1504	3	9.8	0.16	38.42
2 multi 2		H						
32	1	07/21/2018	125	1505	3	10.2	0	39.65
2 multi 2		H						
32	1	07/22/2018	126	1506	3	10.3	0.03	38.2
2 multi 2		H						
32	1	07/23/2018	127	1507	3	10	0	38.64
2 multi 2		H						
32	1	07/24/2018	128	1508	3	10.2	0	36.67
2 multi 2		H						
32	1	07/25/2018	129	1509	3	10.3	0.06	39.36
2 multi 2		H						
32	1	07/26/2018	130	1510	3	9.9	0.08	33.22
2 multi 2		H						
32	1	08/31/2018	166	1546	3	9.9	0.13	32.92
2 multi 2		H						
32	1	09/01/2018	167	1547	3	10.2	0.02	40.47
2 multi 2		H						
32	1	09/02/2018	168	1548	3	9.8	0.13	41.51
2 multi 2		H						
32	1	09/03/2018	169	1549	3	9.9	0.04	43.51
2 multi 2		H						
32	1	09/04/2018	170	1550	3	10.2	0.13	40.27
2 multi 2		H						
32	1	09/05/2018	171	1551	3	9.9	0.2	37.52
2 multi 2		H						
32	1	09/06/2018	172	1552	3	9.9	0.08	38.14
2 multi 2		H						
32	1	09/07/2018	173	1553	3	10.2	0.13	37.76
2 multi 2		H						
32	1	09/08/2018	174	1554	3	10	0.02	40.52
2 multi 2		H						
32	1	09/09/2018	175	1555	3	10	0.28	36.16
2 multi 2		H						
32	1	09/10/2018	176	1556	3	9.9	0.19	35.75
2 multi 2		H						
32	1	09/11/2018	177	1557	3	10	0	37.4
2 multi 2		H						
32	1	09/12/2018	178	1558	3	10	0.15	35.42
2 multi 2		H						
32	1	09/13/2018	179	1559	3	9.9	0.07	33.42
2 multi 2		H						
32	1	09/14/2018	180	1560	3	9.8	0.1	31.18
2 multi 2		H						
32	1	09/15/2018	181	1561	3	10	0.06	34.11
2 multi 2		H						
32	1	09/16/2018	182	1562	3	10	0.07	35.38
2 multi 2		H						
32	1	09/17/2018	183	1563	3	10	0.18	36.36
2 multi 2		H						
32	1	09/18/2018	184	1564	3	10	0	37.24

2 multi 2		H						
32	1	09/19/2018	185	1565	3	10	0	36.09
2 multi 2		H						
32	1	09/20/2018	186	1566	3	9.9	0	33.8
2 multi 2		H						
32	1	09/21/2018	187	1567	3	9.6	0.05	34.76
2 multi 2		H						
32	1	09/22/2018	188	1568	3	9.9	0.19	32.93
2 multi 2		H						
32	1	09/23/2018	189	1569	3	9.9	0.2	33.5
2 multi 2		H						
32	1	09/24/2018	190	1570	3	9.9	0.1	32.66
2 multi 2		H						
32	1	09/25/2018	191	1571	3	9.8	0.12	32.09
2 multi 2		H						
32	1	09/26/2018	192	1572	3	9.8	0.21	33.3
2 multi 2		H						
32	1	09/27/2018	193	1573	3	9.6	0.2	33.88
2 multi 2		H						
32	1	09/28/2018	194	1574	3	9.9	0.22	33.88
2 multi 2		H						
32	1	09/29/2018	195	1575	3	9.8	0.14	34.58
2 multi 2		H						
32	1	09/30/2018	196	1576	3	9.8	0	34.21
2 multi 2		H						
32	1	10/01/2018	197	1577	3	9.9	0	32.87
2 multi 2		H						
32	1	10/02/2018	198	1578	3	9.9	0	34.89
2 multi 2		H						
32	1	10/03/2018	199	1579	3	9.8	0.14	34.69
2 multi 2		H						
32	1	10/04/2018	200	1580	3	9.8	0.13	33.47
2 multi 2		H						
32	1	10/05/2018	201	1581	3	9.5	0.19	31.86
2 multi 2		H						
32	1	10/06/2018	202	1582	3	9.8	0	33.4
2 multi 2		H						
32	1	10/07/2018	203	1583	3	9.8	0.16	33.6
2 multi 2		H						
32	1	03/04/2019	6	1731	4	9.8	0.31	41.1
2 multi 1		H						
32	1	03/05/2019	7	1732	4	9.5	0.05	43.77
2 multi 1		H						
32	1	03/06/2019	8	1733	4	9.1	0.04	36.19
2 multi 1		H						
32	1	03/07/2019	9	1734	4	9.5	0.03	37.56
2 multi 1		H						
32	1	03/08/2019	10	1735	4	9.5	0.17	43.41
2 multi 1		H						
32	1	03/09/2019	11	1736	4	9.5	0.07	45.56
2 multi 1		H						
32	1	03/10/2019	12	1737	4	9.8	0	48.72
2 multi 1		H						
32	1	03/11/2019	13	1738	4	9.8	0.02	45.79

2 multi	1	H						
32	1	03/12/2019	14	1739	4	9.8	0	52.6
2 multi	1	H						
32	1	03/13/2019	15	1740	4	9.6	0.09	49.64
2 multi	1	H						
32	1	03/14/2019	16	1741	4	9.6	0	51.51
2 multi	1	H						
32	1	03/15/2019	17	1742	4	9.8	0.14	50.18
2 multi	1	H						
32	1	03/16/2019	18	1743	4	9.6	0.02	51.08
2 multi	1	H						
32	1	03/17/2019	19	1744	4	9.5	0.07	49.33
2 multi	1	H						
32	1	03/18/2019	20	1745	4	9.8	0.07	51.35
2 multi	1	H						
32	1	03/19/2019	21	1746	4	9.5	0.09	55.73
2 multi	1	H						
32	1	03/20/2019	22	1747	4	9.5	0.14	53.41
2 multi	2	H						
32	1	03/21/2019	23	1748	4	9.6	0.17	52.82
2 multi	2	H						
32	1	03/22/2019	24	1749	4	9.5	0.01	52.98
2 multi	2	H						
32	1	03/23/2019	25	1750	4	9.3	0	51.28
2 multi	2	H						
32	1	03/24/2019	26	1751	4	9.6	0.2	56.8
2 multi	2	H						
32	1	03/25/2019	27	1752	4	9.6	0.11	52.9
2 multi	2	H						
32	1	03/26/2019	28	1753	4	9.3	0.15	54.38
2 multi	2	H						
32	1	03/27/2019	29	1754	4	9.3	0.1	58.23
2 multi	2	H						
32	1	09/13/2019	199	1924	4	9.9	0.01	32.5
2 multi	2	H						
32	1	09/14/2019	200	1925	4	9.8	0	35.25
2 multi	2	H						
32	1	09/15/2019	201	1926	4	10	0.01	35.4
2 multi	2	H						
32	1	09/16/2019	202	1927	4	10	0.09	35.17
2 multi	2	H						
32	1	09/17/2019	203	1928	4	9.8	0.23	36.55
2 multi	2	H						
32	1	09/18/2019	204	1929	4	9.6	0.1	35.75
2 multi	2	H						
32	1	09/19/2019	205	1930	4	9.9	0.16	34.18
2 multi	2	H						
32	1	09/20/2019	206	1931	4	9.8	0.09	35.52
2 multi	2	H						
32	1	09/21/2019	207	1932	4	9.9	0.25	38.24
2 multi	2	H						
32	1	09/22/2019	208	1933	4	10	0.01	37.4
2 multi	2	H						
32	1	09/23/2019	209	1934	4	9.9	0.19	35.75

2 multi 2		H						
32	1	09/24/2019	210	1935	4	9.8	0.11	35.53
2 multi 2		H						
32	1	09/25/2019	211	1936	4	10	0.02	36.99
2 multi 2		H						
32	1	09/26/2019	212	1937	4	9.8	0	33.76
2 multi 2		H						
32	1	09/27/2019	213	1938	4	10	0.01	35.31
2 multi 2		H						
32	1	09/28/2019	214	1939	4	9.9	0.02	34.13
2 multi 2		H						
32	1	09/29/2019	215	1940	4	9.9	0	35.75
2 multi 2		H						
32	1	09/30/2019	216	1941	4	9.9	0.08	36.65
2 multi 2		H						
32	1	10/01/2019	217	1942	4	9.8	0.11	36.76
2 multi 2		H						
32	1	10/02/2019	218	1943	4	9.8	0.04	35.39
2 multi 2		H						
32	1	10/03/2019	219	1944	4	10	0	34.56
2 multi 2		H						
32	1	10/04/2019	220	1945	4	10.7	0.41	45.18
2 multi 2		H						
32	1	10/05/2019	221	1946	4	10.2	0.24	34.44
2 multi 2		H						
32	1	10/06/2019	222	1947	4	9.9	0.21	34.03
2 multi 2		H						
32	1	10/07/2019	223	1948	4	10.2	0.13	36.4
2 multi 2		H						
32	1	10/08/2019	224	1949	4	10.2	0.12	37.18
2 multi 2		H						
32	1	10/09/2019	225	1950	4	10.3	0	35.81
2 multi 2		H						
32	1	10/10/2019	226	1951	4	9.6	0.06	33.83
2 multi 2		H						
32	1	10/11/2019	227	1952	4	9.9	0	32.61
2 multi 2		H						
32	1	01/27/2020	3	2060	5	10.2	0.24	34.29
2 multi 1		H						
32	1	01/28/2020	4	2061	5	9.8	0.14	39.69
2 multi 1		H						
32	1	01/29/2020	5	2062	5	10	0.03	39.93
2 multi 1		H						
32	1	01/30/2020	6	2063	5	9.8	0.04	43.55
2 multi 1		H						
32	1	01/31/2020	7	2064	5	9.8	0.06	41.22
2 multi 1		H						
32	1	02/01/2020	8	2065	5	9.8	0	42.9
2 multi 1		H						
32	1	02/02/2020	9	2066	5	9.8	0.02	45.34
2 multi 1		H						
32	1	02/03/2020	10	2067	5	9.6	0.01	48.26
2 multi 1		H						
32	1	02/04/2020	11	2068	5	9.6	0.02	43.66

2 multi	1	H						
32	1	02/05/2020	12	2069	5	9.6	0	50.79
2 multi	1	H						
32	1	02/06/2020	13	2070	5	9.5	0	48.64
2 multi	1	H						
32	1	02/07/2020	14	2071	5	9.6	0	52.31
2 multi	1	H						
32	1	02/08/2020	15	2072	5	9.3	0.02	52.07
2 multi	1	H						
32	1	02/09/2020	16	2073	5	9.5	0.1	52.55
2 multi	1	H						
32	1	02/10/2020	17	2074	5	9.5	0.11	49.26
2 multi	1	H						
32	1	02/11/2020	18	2075	5	9.5	0	52.38
2 multi	1	H						
32	1	02/12/2020	19	2076	5	9.5	0	52.35
2 multi	1	H						
32	1	02/13/2020	20	2077	5	9.3	0.17	52.85
2 multi	1	H						
32	1	02/14/2020	21	2078	5	9.3	0	55.8
2 multi	1	H						
32	1	02/15/2020	22	2079	5	9.5	0	53.22
2 multi	2	H						
32	1	02/16/2020	23	2080	5	9.5	0.07	56.51
2 multi	2	H						
32	1	02/17/2020	24	2081	5	9.5	0	54.04
2 multi	2	H						
32	1	02/18/2020	25	2082	5	9.5	0.09	57.36
2 multi	2	H						
32	1	02/19/2020	26	2083	5	0	0.23	54.34
2 multi	2	H						
32	1	02/20/2020	27	2084	5	9.6	0.02	55.23
2 multi	2	H						
32	1	02/21/2020	28	2085	5	9.5	0	59.77
2 multi	2	H						
32	1	02/22/2020	29	2086	5	9.5	0	55.34
2 multi	2	H						
32	1	02/23/2020	30	2087	5	9.6	0.11	54.3
2 multi	2	H						
32	1	02/24/2020	31	2088	5	9.8	0.01	54.63
2 multi	2	H						
32	1	02/25/2020	32	2089	5	9.9	0.01	58.97
2 multi	2	H						
32	1	02/26/2020	33	2090	5	9.8	0	58.45
2 multi	2	H						
32	1	02/27/2020	34	2091	5	9.9	0.03	57.07
2 multi	2	H						
32	1	02/28/2020	35	2092	5	9.6	0	54.3
2 multi	2	H						
32	1	02/29/2020	36	2093	5	9.6	0.51	60.64
2 multi	2	H						
32	1	03/01/2020	37	2094	5	9.6	0.13	56.58
2 multi	2	H						
32	1	03/02/2020	38	2095	5	9.5	0	55.25

2 multi 2		H						
32	1	03/03/2020	39	2096	5	9.9	0.19	61.62
2 multi 2		H						
32	1	03/04/2020	40	2097	5	9.5	0.07	56.36
2 multi 2		H						
32	1	03/05/2020	41	2098	5	9.8	0.34	56.49
2 multi 2		H						
32	1	03/06/2020	42	2099	5	9.5	0	51.58
2 multi 2		H						
32	1	03/07/2020	43	2100	5	9.5	0.23	51.84
2 multi 2		H						
32	1	03/08/2020	44	2101	5	9.6	0.06	55.07
2 multi 2		H						
32	1	03/09/2020	45	2102	5	9.3	0.13	55.58
2 multi 2		H						
32	1	03/10/2020	46	2103	5	9.5	0	56.85
2 multi 2		H						
32	1	03/11/2020	47	2104	5	10	0	54.74
2 multi 2		H						
32	1	03/12/2020	48	2105	5	9.8	0.17	49.8
2 multi 2		H						
32	1	03/13/2020	49	2106	5	9.9	0	59.41
2 multi 2		H						
32	1	03/14/2020	50	2107	5	10.3	0	58.82
2 multi 2		H						
32	1	03/15/2020	51	2108	5	9.8	0	55.46
2 multi 2		H						
32	1	03/16/2020	52	2109	5	9.6	0.2	56.71
2 multi 2		H						
32	1	03/17/2020	53	2110	5	9.8	0.06	59.49
2 multi 2		H						
32	1	03/18/2020	54	2111	5	9.8	0.08	59.93
2 multi 2		H						
32	1	03/19/2020	55	2112	5	10	0.01	58.39
2 multi 2		H						
32	1	03/20/2020	56	2113	5	9.5	0.13	57.46
2 multi 2		H						
32	1	03/21/2020	57	2114	5	9.9	0.12	56.89
2 multi 2		H						
32	1	03/22/2020	58	2115	5	9.8	0.04	57.3
2 multi 2		H						
32	1	03/23/2020	59	2116	5	9.6	0.06	56.77
2 multi 2		H						
32	1	03/24/2020	60	2117	5	9.8	0.13	57.2
2 multi 2		H						
32	1	03/25/2020	61	2118	5	9.6	0	57.25
2 multi 2		H						
32	1	03/26/2020	62	2119	5	9.6	0	56.26
2 multi 2		H						
32	1	03/27/2020	63	2120	5	9.9	0	53.3
2 multi 2		H						
32	1	03/28/2020	64	2121	5	10	0.08	55.53
2 multi 2		H						
32	1	03/29/2020	65	2122	5	9.8	0	52.01

2 multi 2		H						
32	1	03/30/2020	66	2123	5	9.6	0	56.23
2 multi 2		H						
32	1	03/31/2020	67	2124	5	9.8	0.16	54.3
2 multi 2		H						
32	1	04/01/2020	68	2125	5	9.5	0.15	51.13
2 multi 2		H						
32	1	04/02/2020	69	2126	5	9.5	0.19	52.26
2 multi 2		H						
32	1	04/03/2020	70	2127	5	9.5	0.06	51.33
2 multi 2		H						
32	1	04/04/2020	71	2128	5	9.3	0	45.17
2 multi 2		H						
32	1	04/05/2020	72	2129	5	9.8	0	47.24
2 multi 2		H						
32	1	04/06/2020	73	2130	5	9.2	0	48.22
2 multi 2		H						
32	1	04/07/2020	74	2131	5	9.5	0	47.39
2 multi 2		H						
32	1	04/08/2020	75	2132	5	9.3	0.05	49.3
2 multi 2		H						
32	1	04/09/2020	76	2133	5	9.6	0	46.75
2 multi 2		H						
32	1	04/10/2020	77	2134	5	9.9	0	49.62
2 multi 2		H						
32	1	04/11/2020	78	2135	5	10	0	46.06
2 multi 2		H						
32	1	04/12/2020	79	2136	5	10	0.28	48.49
2 multi 2		H						
32	1	04/13/2020	80	2137	5	10.2	0.28	53.32
2 multi 2		H						
32	1	04/14/2020	81	2138	5	9.9	0.06	51.08
2 multi 2		H						
32	1	04/15/2020	82	2139	5	10	0.04	50.91
2 multi 2		H						
32	1	04/16/2020	83	2140	5	9.8	0.21	47.24
2 multi 2		H						
32	1	04/17/2020	84	2141	5	9.6	0.3	46.18
2 multi 2		H						
32	1	04/18/2020	85	2142	5	10	0.01	49.48
2 multi 2		H						
32	1	04/19/2020	86	2143	5	10	0	49.79
2 multi 2		H						
32	1	04/20/2020	87	2144	5	10	0	47.47
2 multi 2		H						
32	1	04/21/2020	88	2145	5	9.6	0	37.09
2 multi 2		H						
32	1	04/22/2020	89	2146	5	9.8	0	41.57
2 multi 2		H						
32	1	04/23/2020	90	2147	5	9.9	0.14	44.37
2 multi 2		H						
32	1	04/24/2020	91	2148	5	10	0	44.75
2 multi 2		H						
32	1	04/25/2020	92	2149	5	9.8	0.11	42.77

2 multi 2		H						
32	1	04/26/2020	93	2150	5	9.9	0	45.75
2 multi 2		H						
32	1	04/27/2020	94	2151	5	9.9	0	46.6
2 multi 2		H						
32	1	04/28/2020	95	2152	5	10	0	47.02
2 multi 2		H						
32	1	04/29/2020	96	2153	5	10	0	46.25
2 multi 2		H						
32	1	04/30/2020	97	2154	5	10.2	0	45.19
2 multi 2		H						
32	1	05/01/2020	98	2155	5	9.8	0	44.51
2 multi 2		H						
32	1	05/02/2020	99	2156	5	9.9	0.22	46.32
2 multi 2		H						
32	1	05/03/2020	100	2157	5	10	0.14	45.24
2 multi 2		H						
32	1	05/04/2020	101	2158	5	9.9	0	45.34
2 multi 2		H						
32	1	05/05/2020	102	2159	5	9.9	0.14	46.46
2 multi 2		H						
32	1	05/06/2020	103	2160	5	10	0	47.31
2 multi 2		H						
32	1	05/07/2020	104	2161	5	10.2	0.06	45.76
2 multi 2		H						
32	1	05/08/2020	105	2162	5	9.8	0.13	47.71
2 multi 2		H						
32	1	05/09/2020	106	2163	5	9.9	0.14	44.21
2 multi 2		H						
32	1	05/10/2020	107	2164	5	9.9	0.01	48.24
2 multi 2		H						
32	1	05/11/2020	108	2165	5	9.8	0.17	42.83
2 multi 2		H						
32	1	05/12/2020	109	2166	5	10	0.25	44.46
2 multi 2		H						
32	1	05/13/2020	110	2167	5	9.9	0.06	46.69
2 multi 2		H						
32	1	05/14/2020	111	2168	5	10	0.02	45.33
2 multi 2		H						
32	1	05/15/2020	112	2169	5	9.9	0	45.28
2 multi 2		H						
32	1	05/16/2020	113	2170	5	9.8	0.28	46.4
2 multi 2		H						
32	1	05/17/2020	114	2171	5	9.8	0.07	44.03
2 multi 2		H						
32	1	05/18/2020	115	2172	5	9.9	0.03	42.77
2 multi 2		H						
32	1	05/19/2020	116	2173	5	9.9	0.07	42.88
2 multi 2		H						
32	1	05/20/2020	117	2174	5	9.8	0.09	44.6
2 multi 2		H						
32	1	05/21/2020	118	2175	5	10	0	43.58
2 multi 2		H						
32	1	05/22/2020	119	2176	5	10	0.03	45.22

2 multi 2		H						
32	1	05/23/2020	120	2177	5	9.9	0	40.99
2 multi 2		H						
32	1	05/24/2020	121	2178	5	9.9	0	43.25
2 multi 2		H						
32	1	05/25/2020	122	2179	5	9.9	0	46.45
2 multi 2		H						
32	1	05/26/2020	123	2180	5	9.6	0	41.53
2 multi 2		H						
32	1	05/27/2020	124	2181	5	10	0	44.15
2 multi 2		H						
32	1	05/28/2020	125	2182	5	9.8	0	43.89
2 multi 2		H						
32	1	05/29/2020	126	2183	5	9.9	0.06	43.1
2 multi 2		H						
32	1	05/30/2020	127	2184	5	10	0.32	43.59
2 multi 2		H						
32	1	05/31/2020	128	2185	5	9.9	0	44.15
2 multi 2		H						
32	1	06/01/2020	129	2186	5	9.8	0	42.9
2 multi 2		H						
32	1	06/02/2020	130	2187	5	9.8	0	39.79
2 multi 2		H						
32	1	06/03/2020	131	2188	5	9.8	0	39.84
2 multi 2		H						
32	1	06/04/2020	132	2189	5	9.8	0	39.3
2 multi 2		H						
32	1	06/05/2020	133	2190	5	9.6	0.06	37.89
2 multi 2		H						
32	1	06/06/2020	134	2191	5	9.8	0	39.2
2 multi 2		H						
32	1	06/07/2020	135	2192	5	9.5	0.07	36.93
2 multi 2		H						
32	1	06/08/2020	136	2193	5	9.2	0.07	36.2
2 multi 2		H						
32	1	06/09/2020	137	2194	5	9.6	0.07	35.05
2 multi 2		H						
32	1	06/10/2020	138	2195	5	9.5	0	34.06
2 multi 2		H						
32	1	06/11/2020	139	2196	5	9.3	0	37.87
2 multi 2		H						
32	1	06/12/2020	140	2197	5	9.5	0	35.89
2 multi 2		H						
32	1	06/13/2020	141	2198	5	9.9	0.01	37.27
2 multi 2		H						
32	1	06/14/2020	142	2199	5	9.9	0.32	40.12
2 multi 2		H						
32	1	06/15/2020	143	2200	5	9.9	0.2	36.56
2 multi 2		H						
32	1	06/16/2020	144	2201	5	9.8	0.17	40.22
2 multi 2		H						
32	1	06/17/2020	145	2202	5	10	0.28	39.16
2 multi 2		H						
32	1	06/18/2020	146	2203	5	9.9	0.21	40.54

2 multi	2	H						
32	1	06/19/2020	147	2204	5	9.9	0.22	41.27
2 multi	2	H						
32	1	06/20/2020	148	2205	5	9.9	0	36.58
2 multi	2	H						
32	1	06/21/2020	149	2206	5	9.9	0.11	37.91
2 multi	2	H						
32	1	06/22/2020	150	2207	5	10	0	37.12
2 multi	2	H						
32	1	06/23/2020	151	2208	5	9.9	0.22	38.99
2 multi	2	H						
32	1	06/24/2020	152	2209	5	9.9	0	36.3
2 multi	2	H						
32	1	06/25/2020	153	2210	5	10	0	41.06
2 multi	2	H						
32	1	06/26/2020	154	2211	5	10	0	39.39
2 multi	2	H						
32	1	06/27/2020	155	2212	5	10	0	42.81
2 multi	2	H						
32	1	06/28/2020	156	2213	5	9.9	0	40.19
2 multi	2	H						
32	1	06/29/2020	157	2214	5	10	0	38.75
2 multi	2	H						
32	1	06/30/2020	158	2215	5	9.8	0	36.62
2 multi	2	H						
32	1	07/01/2020	159	2216	5	10	0	39.7
2 multi	2	H						
32	1	07/02/2020	160	2217	5	9.8	0	39.23
2 multi	2	H						
32	1	07/03/2020	161	2218	5	9.8	0.21	36.47
2 multi	2	H						
32	1	07/04/2020	162	2219	5	9.8	0.13	38.85
2 multi	2	H						
32	1	07/05/2020	163	2220	5	9.9	0.11	38.91
2 multi	2	H						
32	1	07/06/2020	164	2221	5	9.9	0.16	42.14
2 multi	2	H						
32	1	07/07/2020	165	2222	5	10	0	41.23
2 multi	2	H						
32	1	07/08/2020	166	2223	5	9.8	0.22	38.38
2 multi	2	H						
32	1	07/09/2020	167	2224	5	9.8	0	36.98
2 multi	2	H						
32	1	07/10/2020	168	2225	5	10	0.2	38.12
2 multi	2	H						
32	1	07/11/2020	169	2226	5	10	0	35.9
2 multi	2	H						
32	1	07/12/2020	170	2227	5	10.2	0.07	36.98
2 multi	2	H						
32	1	07/13/2020	171	2228	5	9.9	0	37.83
2 multi	2	H						
32	1	07/14/2020	172	2229	5	10.2	0	39.74
2 multi	2	H						
32	1	07/15/2020	173	2230	5	10	0.21	38.41

2 multi	2	H						
32	1	07/16/2020	174	2231	5	9.9	0.16	38.67
2 multi	2	H						
32	1	07/17/2020	175	2232	5	9.8	0	35.51
2 multi	2	H						
32	1	07/18/2020	176	2233	5	9.5	0.12	32.58
2 multi	2	H						
32	1	07/19/2020	177	2234	5	9.6	0.13	37.14
2 multi	2	H						
32	1	07/20/2020	178	2235	5	10	0.14	39.37
2 multi	2	H						
32	1	07/21/2020	179	2236	5	10.2	0.03	37.33
2 multi	2	H						
32	1	07/22/2020	180	2237	5	9.8	0.24	39.21
2 multi	2	H						
32	1	07/23/2020	181	2238	5	9.6	0.16	33.2
2 multi	2	H						
32	1	07/24/2020	182	2239	5	9.9	0.26	38.19
2 multi	2	H						
32	1	07/25/2020	183	2240	5	10	0.22	35.39
2 multi	2	H						
32	1	07/26/2020	184	2241	5	9.9	0.22	34.46
2 multi	2	H						
32	1	07/27/2020	185	2242	5	9.6	0.23	36.4
2 multi	2	H						
32	1	07/28/2020	186	2243	5	10	0.2	35.01
2 multi	2	H						
32	1	07/29/2020	187	2244	5	10.2	0.23	39.14
2 multi	2	H						
32	1	07/30/2020	188	2245	5	9.8	0.18	34.55
2 multi	2	H						
32	1	07/31/2020	189	2246	5	9.6	0.02	34.17
2 multi	2	H						
32	1	08/01/2020	190	2247	5	9.5	0	34.57
2 multi	2	H						
32	1	08/02/2020	191	2248	5	9.8	0.07	36.46
2 multi	2	H						
32	1	08/03/2020	192	2249	5	9.6	0	33.98
2 multi	2	H						
32	1	08/04/2020	193	2250	5	9.9	0.12	39.38
2 multi	2	H						
32	1	08/05/2020	194	2251	5	10	0	38.27
2 multi	2	H						
32	1	08/06/2020	195	2252	5	9.9	0	34.42
2 multi	2	H						
32	1	08/07/2020	196	2253	5	9.8	0.09	37.48
2 multi	2	H						
32	1	08/08/2020	197	2254	5	10.2	0	35.35
2 multi	2	H						
32	1	08/09/2020	198	2255	5	10.2	0.01	38.81
2 multi	2	H						
32	1	08/10/2020	199	2256	5	10	0.17	34.15
2 multi	2	H						
32	1	08/11/2020	200	2257	5	9.6	0.01	34.68

2 multi	2	H						
32	1	08/12/2020	201	2258	5	10	0	36.51
2 multi	2	H						
32	1	08/13/2020	202	2259	5	9.6	0.15	36.31
2 multi	2	H						
32	1	08/14/2020	203	2260	5	9.9	0.25	36.69
2 multi	2	H						
32	1	08/15/2020	204	2261	5	10.2	0.24	34.5
2 multi	2	H						
32	1	08/16/2020	205	2262	5	10.2	0.03	34.95
2 multi	2	H						
32	1	01/01/2021	7	2400	6	9.9	0.06	43.03
2 multi	1	H						
32	1	01/02/2021	8	2401	6	10	0.04	33.7
2 multi	1	H						
32	1	01/03/2021	9	2402	6	9.8	0.27	43.74
2 multi	1	H						
32	1	01/04/2021	10	2403	6	9.6	0.06	43.51
2 multi	1	H						
32	1	01/05/2021	11	2404	6	9.9	0.02	45.52
2 multi	1	H						
32	1	01/06/2021	12	2405	6	9.8	0.1	50.69
2 multi	1	H						
32	1	01/07/2021	13	2406	6	9.8	0.02	46.33
2 multi	1	H						
32	1	01/08/2021	14	2407	6	10	0.11	50.13
2 multi	1	H						
32	1	01/09/2021	15	2408	6	10	0.05	48.84
2 multi	1	H						
32	1	01/10/2021	16	2409	6	10.2	0	52.15
2 multi	1	H						
32	1	01/11/2021	17	2410	6	9.8	0	51.34
2 multi	1	H						
32	1	01/12/2021	18	2411	6	9.6	0	50.35
2 multi	1	H						
32	1	01/13/2021	19	2412	6	9.9	0.04	55.27
2 multi	1	H						
32	1	01/14/2021	20	2413	6	9.9	0.04	55.11
2 multi	1	H						
32	1	01/15/2021	21	2414	6	9.6	0	56.65
2 multi	1	H						
32	1	01/16/2021	22	2415	6	9.6	0.22	53.58
2 multi	2	H						
32	1	01/17/2021	23	2416	6	9.3	0.05	51.8
2 multi	2	H						
32	1	01/18/2021	24	2417	6	9.5	0	53.79
2 multi	2	H						
32	1	01/19/2021	25	2418	6	9.5	0	50.3
2 multi	2	H						
32	1	01/20/2021	26	2419	6	9.8	0.11	50.82
2 multi	2	H						
32	1	01/21/2021	27	2420	6	9.6	0.11	49.97
2 multi	2	H						
32	1	01/22/2021	28	2421	6	9.8	0.03	53.24

2 multi 2		H						
32	1	01/23/2021	29	2422	6	9.8	0.04	54.13
2 multi 2		H						
32	1	01/24/2021	30	2423	6	9.8	0.04	55.3
2 multi 2		H						
32	1	01/25/2021	31	2424	6	9.9	0.07	55.82
2 multi 2		H						
32	1	01/26/2021	32	2425	6	9.8	0.04	54.8
2 multi 2		H						
32	1	01/27/2021	33	2426	6	9.8	0.12	53.09
2 multi 2		H						
32	1	01/28/2021	34	2427	6	9.6	0.06	52.44
2 multi 2		H						
32	1	01/29/2021	35	2428	6	9.8	0.04	53.45
2 multi 2		H						
32	1	01/30/2021	36	2429	6	9.8	0	56.6
2 multi 2		H						
32	1	01/31/2021	37	2430	6	9.3	0.15	54.66
2 multi 2		H						
32	1	02/01/2021	38	2431	6	9.6	0.04	54.76
2 multi 2		H						
32	1	02/02/2021	39	2432	6	9.5	0.01	56.18
2 multi 2		H						
32	1	02/03/2021	40	2433	6	9.5	0	48.79
2 multi 2		H						
32	1	02/04/2021	41	2434	6	9.5	0.12	55.18
2 multi 2		H						
32	1	02/05/2021	42	2435	6	9.8	0.03	54.94
2 multi 2		H						
32	1	02/06/2021	43	2436	6	9.6	0.03	54.31
2 multi 2		H						
32	1	02/07/2021	44	2437	6	9.2	0.01	26.52
2 multi 2		H						
32	1	02/08/2021	45	2438	6	9.8	0.04	49.91
2 multi 2		H						
32	1	02/09/2021	46	2439	6	9.9	0.03	53.02
2 multi 2		H						
32	1	02/10/2021	47	2440	6	9.8	0	47.97
2 multi 2		H						
32	1	02/11/2021	48	2441	6	9.8	0.26	56.2
2 multi 2		H						
32	1	02/12/2021	49	2442	6	9.8	0.01	53.13
2 multi 2		H						
32	1	02/13/2021	50	2443	6	10	0.04	53.59
2 multi 2		H						
32	1	02/14/2021	51	2444	6	9.8	0.08	53.11
2 multi 2		H						
32	1	02/15/2021	52	2445	6	9.5	0.04	52.38
2 multi 2		H						
32	1	02/16/2021	53	2446	6	9.8	0	51.13
2 multi 2		H						
32	1	02/17/2021	54	2447	6	9.9	0.12	54.2
2 multi 2		H						
32	1	02/18/2021	55	2448	6	10	0.03	55.14

2 multi 2		H						
32	1	02/19/2021	56	2449	6	9.9	0.1	54.03
2 multi 2		H						
32	1	02/20/2021	57	2450	6	9.8	0	57.6
2 multi 2		H						
32	1	02/21/2021	58	2451	6	9.6	0	54.5
2 multi 2		H						
32	1	02/22/2021	59	2452	6	9.6	0.03	53.67
2 multi 2		H						
32	1	02/23/2021	60	2453	6	9.6	0.03	48.12
2 multi 2		H						
32	1	02/24/2021	61	2454	6	9.6	0.03	52.51
2 multi 2		H						
32	1	02/25/2021	62	2455	6	10	0.04	55.25
2 multi 2		H						
32	1	02/26/2021	63	2456	6	9.6	0	53.03
2 multi 2		H						
32	1	02/27/2021	64	2457	6	9.2	0.11	48.78
2 multi 2		H						
32	1	02/28/2021	65	2458	6	9.6	0	55.26
2 multi 2		H						
32	1	03/01/2021	66	2459	6	9.6	0.02	51.96
2 multi 2		H						
32	1	03/02/2021	67	2460	6	9.5	0	50.86
2 multi 2		H						
32	1	03/03/2021	68	2461	6	9.6	0	47.61
2 multi 2		H						
32	1	03/04/2021	69	2462	6	9.6	0.11	47.84
2 multi 2		H						
32	1	03/05/2021	70	2463	6	9.6	0.19	50.78
2 multi 2		H						
32	1	03/06/2021	71	2464	6	9.6	0.14	49.75
2 multi 2		H						
32	1	03/07/2021	72	2465	6	9.6	0.17	48.51
2 multi 2		H						
32	1	03/08/2021	73	2466	6	9.8	0	47.25
2 multi 2		H						
32	1	03/09/2021	74	2467	6	9.9	0.04	51.4
2 multi 2		H						
32	1	03/10/2021	75	2468	6	9.6	0.04	52.67
2 multi 2		H						
32	1	03/11/2021	76	2469	6	9.8	0.01	51.7
2 multi 2		H						
32	1	03/12/2021	77	2470	6	9.9	0	51.22
2 multi 2		H						
32	1	03/13/2021	78	2471	6	10	0.1	47.82
2 multi 2		H						
32	1	03/14/2021	79	2472	6	9.9	0	50.59
2 multi 2		H						
32	1	03/15/2021	80	2473	6	10	0.03	54.3
2 multi 2		H						
32	1	03/16/2021	81	2474	6	9.9	0.02	52.07
2 multi 2		H						
32	1	03/17/2021	82	2475	6	9.8	0	48.97

2 multi 2		H						
32	1	03/18/2021	83	2476	6	9.8	0	49.5
2 multi 2		H						
32	1	03/19/2021	84	2477	6	9.5	0.1	50.13
2 multi 2		H						
32	1	03/20/2021	85	2478	6	9.6	0.06	47.09
2 multi 2		H						
32	1	03/21/2021	86	2479	6	9.6	0	50.03
2 multi 2		H						
32	1	03/22/2021	87	2480	6	9.8	0	48.06
2 multi 2		H						
32	1	03/23/2021	88	2481	6	9.5	0	47.55
2 multi 2		H						
32	1	03/24/2021	89	2482	6	9.6	0.02	46.29
2 multi 2		H						
32	1	03/25/2021	90	2483	6	9.8	0	51.36
2 multi 2		H						
32	1	03/26/2021	91	2484	6	9.6	0.19	52.05
2 multi 2		H						
32	1	03/27/2021	92	2485	6	9.8	0	50.83
2 multi 2		H						
32	1	03/28/2021	93	2486	6	9.3	0.01	47.1
2 multi 2		H						
32	1	03/29/2021	94	2487	6	9.6	0.01	49.34
2 multi 2		H						
32	1	03/30/2021	95	2488	6	9.9	0	50.36
2 multi 2		H						
32	1	03/31/2021	96	2489	6	9.8	0.02	50.72
2 multi 2		H						
32	1	04/01/2021	97	2490	6	9.8	0.03	48.8
2 multi 2		H						
32	1	04/02/2021	98	2491	6	9.6	0.11	48.27
2 multi 2		H						
32	1	04/03/2021	99	2492	6	9.9	0	51.99
2 multi 2		H						
32	1	04/04/2021	100	2493	6	9.3	0.06	44.99
2 multi 2		H						
32	1	04/05/2021	101	2494	6	9.8	0.26	48.59
2 multi 2		H						
32	1	04/06/2021	102	2495	6	9.8	0.23	47.66
2 multi 2		H						
32	1	04/07/2021	103	2496	6	9.5	0.23	44.93
2 multi 2		H						
32	1	04/08/2021	104	2497	6	9.5	0	46.19
2 multi 2		H						
32	1	04/09/2021	105	2498	6	9.6	0.01	44.4
2 multi 2		H						
32	1	04/10/2021	106	2499	6	9.6	0.01	52.85
2 multi 2		H						
32	1	04/11/2021	107	2500	6	9.6	0.28	44.96
2 multi 2		H						
32	1	04/12/2021	108	2501	6	9.5	0.28	46.93
2 multi 2		H						
32	1	04/13/2021	109	2502	6	9.6	0.09	47.63

2 multi 2		H						
32	1	04/14/2021	110	2503	6	9.6	0.1	51.14
2 multi 2		H						
32	1	04/15/2021	111	2504	6	9.6	0.26	46.05
2 multi 2		H						
32	1	04/16/2021	112	2505	6	9.5	0	44.59
2 multi 2		H						
32	1	04/17/2021	113	2506	6	9.8	0.05	46.14
2 multi 2		H						
32	1	04/18/2021	114	2507	6	9.8	0	47.28
2 multi 2		H						
32	1	04/19/2021	115	2508	6	9.6	0	45.22
2 multi 2		H						
32	1	04/20/2021	116	2509	6	10	0	46.7
2 multi 2		H						
32	1	04/21/2021	117	2510	6	9.6	0	47.85
2 multi 2		H						
32	1	04/22/2021	118	2511	6	9.9	0	49.07
2 multi 2		H						
32	1	04/23/2021	119	2512	6	9.8	0	45.44
2 multi 2		H						
32	1	04/24/2021	120	2513	6	9.8	0.04	48.06
2 multi 2		H						
32	1	04/25/2021	121	2514	6	9.8	0.03	50.26
2 multi 2		H						
32	1	04/26/2021	122	2515	6	9.6	0	48.14
2 multi 2		H						
32	1	04/27/2021	123	2516	6	9.9	0	48.3
2 multi 2		H						
32	1	04/28/2021	124	2517	6	9.6	0.27	48.02
2 multi 2		H						
32	1	04/29/2021	125	2518	6	9.8	0.22	48.16
2 multi 2		H						
32	1	04/30/2021	126	2519	6	9.8	0.13	46.21
2 multi 2		H						
32	1	05/01/2021	127	2520	6	9.5	0.05	46.78
2 multi 2		H						
32	1	05/02/2021	128	2521	6	9.6	0.04	43.25
2 multi 2		H						
32	1	05/03/2021	129	2522	6	9.8	0	43.53
2 multi 2		H						
32	1	05/04/2021	130	2523	6	9.8	0	45.61
2 multi 2		H						
32	1	05/05/2021	131	2524	6	9.6	0.12	41.07
2 multi 2		H						
32	1	05/06/2021	132	2525	6	9.8	0.15	45.26
2 multi 2		H						
32	1	05/07/2021	133	2526	6	9.8	0.22	45.14
2 multi 2		H						
32	1	05/08/2021	134	2527	6	9.8	0.05	43.38
2 multi 2		H						
32	1	05/09/2021	135	2528	6	9.8	0.14	47.84
2 multi 2		H						
32	1	05/10/2021	136	2529	6	10	0	49.47

2 multi	2	H						
32	1	05/11/2021	137	2530	6	10	0.08	45.52
2 multi	2	H						
32	1	05/12/2021	138	2531	6	9.9	0	46.8
2 multi	2	H						
32	1	05/13/2021	139	2532	6	9.9	0.23	44.48
2 multi	2	H						
32	1	05/14/2021	140	2533	6	10.2	0	47.66
2 multi	2	H						
32	1	05/15/2021	141	2534	6	9.9	0.25	43.79
2 multi	2	H						
32	1	05/16/2021	142	2535	6	9.8	0.04	42.32
2 multi	2	H						
32	1	05/17/2021	143	2536	6	9.9	0.32	45.25
2 multi	2	H						
32	1	05/18/2021	144	2537	6	9.9	0.24	45.72
2 multi	2	H						
32	1	05/19/2021	145	2538	6	10	0.22	43.76
2 multi	2	H						
32	1	05/20/2021	146	2539	6	9.8	0	42.87
2 multi	2	H						
32	1	05/21/2021	147	2540	6	9.9	0.17	44.69
2 multi	2	H						
32	1	05/22/2021	148	2541	6	9.9	0.13	44.03
2 multi	2	H						
32	1	05/23/2021	149	2542	6	9.9	0.28	43.51
2 multi	2	H						
32	1	05/24/2021	150	2543	6	9.8	0.05	43.52
2 multi	2	H						
32	1	05/25/2021	151	2544	6	9.8	0.06	51.32
2 multi	2	H						
32	1	05/26/2021	152	2545	6	9.6	0	22.87
2 multi	2	H						
34	1	03/22/2014	7	682	1	8.6	0	21.63
1 primi	1	H						
34	1	03/23/2014	8	683	1	8.5	0.03	23.12
1 primi	1	H						
34	1	03/24/2014	9	684	1	8.3	0	21.92
1 primi	1	H						
34	1	03/25/2014	10	685	1	9.6	0	24.58
1 primi	1	H						
34	1	03/26/2014	11	686	1	8.7	0	27.09
1 primi	1	H						
34	1	03/27/2014	12	687	1	8.6	0	26.4
1 primi	1	H						
34	1	03/28/2014	13	688	1	8.3	0	26.73
1 primi	1	H						
34	1	03/29/2014	14	689	1	8.4	0	27.43
1 primi	1	H						
34	1	03/30/2014	15	690	1	8.4	0	27.86
1 primi	1	H						
34	1	03/31/2014	16	691	1	8.2	0	30.59
1 primi	1	H						
34	1	04/01/2014	17	692	1	8.3	0	28.43

1 primi	1	H						
34	1	04/02/2014	18	693	1	8.4	0	29.71
1 primi	1	H						
34	1	04/03/2014	19	694	1	8.4	0	31.06
1 primi	1	H						
34	1	04/04/2014	20	695	1	8.2	0.04	30.73
1 primi	1	H						
34	1	04/05/2014	21	696	1	8.5	0	33.71
1 primi	1	H						
34	1	04/06/2014	22	697	1	8.4	0	31.51
1 primi	2	H						
34	1	04/07/2014	23	698	1	8.2	0	32.41
1 primi	2	H						
34	1	04/08/2014	24	699	1	8.3	0	31.32
1 primi	2	H						
34	1	04/09/2014	25	700	1	8.4	0	32.13
1 primi	2	H						
34	1	04/10/2014	26	701	1	8.3	0	33.37
1 primi	2	H						
34	1	04/11/2014	27	702	1	8.2	0	32.28
1 primi	2	H						
34	1	04/12/2014	28	703	1	8	0	30.96
1 primi	2	H						
34	1	04/13/2014	29	704	1	8.3	0	32.47
1 primi	2	H						
34	1	04/14/2014	30	705	1	8.3	0	33
1 primi	2	H						
34	1	04/15/2014	31	706	1	8.1	0	31.26
1 primi	2	H						
34	1	04/16/2014	32	707	1	8.2	0	33.82
1 primi	2	H						
34	1	04/17/2014	33	708	1	8.1	0	33.44
1 primi	2	H						
34	1	04/18/2014	34	709	1	8.4	0	34.99
1 primi	2	H						
34	1	04/19/2014	35	710	1	8.2	0	34.48
1 primi	2	H						
34	1	04/20/2014	36	711	1	8	0	32.39
1 primi	2	H						
34	1	04/21/2014	37	712	1	8.3	0	34.25
1 primi	2	H						
34	1	04/22/2014	38	713	1	10.2	0	38.28
1 primi	2	H						
34	1	04/23/2014	39	714	1	8.8	0.07	29.98
1 primi	2	H						
34	1	04/24/2014	40	715	1	9.2	0	33.59
1 primi	2	H						
34	1	04/25/2014	41	716	1	8.5	0.03	33.74
1 primi	2	H						
34	1	04/26/2014	42	717	1	8.5	0	34.06
1 primi	2	H						
34	1	04/27/2014	43	718	1	8.4	0.1	32.86
1 primi	2	H						
34	1	04/28/2014	44	719	1	8.5	0	34.33

1 primi 2		H						
34	1	04/29/2014	45	720	1	8.4	0	35.59
1 primi 2		H						
34	1	04/30/2014	46	721	1	8.2	0.07	33.73
1 primi 2		H						
34	1	05/01/2014	47	722	1	8.4	0	35.27
1 primi 2		H						
34	1	05/02/2014	48	723	1	8.5	0.05	30.83
1 primi 2		H						
34	1	05/03/2014	49	724	1	7.9	0	32.39
1 primi 2		H						
34	1	05/04/2014	50	725	1	8.1	0.09	31.95
1 primi 2		H						
34	1	05/05/2014	51	726	1	8.3	0	34.33
1 primi 2		H						
34	1	05/06/2014	52	727	1	8.3	0	33.5
1 primi 2		H						
34	1	05/07/2014	53	728	1	8.4	0.03	33.44
1 primi 2		H						
34	1	05/08/2014	54	729	1	8.3	0	33.38
1 primi 2		H						
34	1	05/09/2014	55	730	1	8.3	0	34.15
1 primi 2		H						
34	1	05/10/2014	56	731	1	8.3	0.06	34.06
1 primi 2		H						
34	1	05/11/2014	57	732	1	8.5	0	33.69
1 primi 2		H						
34	1	05/12/2014	58	733	1	8.3	0.07	33.86
1 primi 2		H						
34	1	05/13/2014	59	734	1	8.4	0	32.72
1 primi 2		H						
34	1	05/14/2014	60	735	1	8.4	0	32.58
1 primi 2		H						
34	1	05/15/2014	61	736	1	8.6	0	35.35
1 primi 2		H						
34	1	05/16/2014	62	737	1	8.5	0	32.36
1 primi 2		H						
34	1	05/17/2014	63	738	1	8.3	0.07	34.78
1 primi 2		H						
34	1	05/18/2014	64	739	1	8.3	0.06	32.78
1 primi 2		H						
34	1	05/19/2014	65	740	1	8.2	0.04	33.23
1 primi 2		H						
34	1	05/20/2014	66	741	1	8.2	0.06	33.32
1 primi 2		H						
34	1	05/21/2014	67	742	1	8.4	0	33.33
1 primi 2		H						
34	1	05/22/2014	68	743	1	8.5	0	32.65
1 primi 2		H						
34	1	05/23/2014	69	744	1	8.5	0	30.42
1 primi 2		H						
34	1	05/24/2014	70	745	1	10.2	0.19	27.94
1 primi 2		H						
34	1	05/25/2014	71	746	1	8.8	0	27.17

1 primi 2		H						
34	1	05/26/2014	72	747	1	9.6	0.12	32.25
1 primi 2		H						
34	1	05/27/2014	73	748	1	9.3	0	31.01
1 primi 2		H						
34	1	05/28/2014	74	749	1	9.1	0	34.9
1 primi 2		H						
34	1	05/29/2014	75	750	1	9.1	0	32.65
1 primi 2		H						
34	1	05/30/2014	76	751	1	8.9	0	31.68
1 primi 2		H						
34	1	05/31/2014	77	752	1	8.4	0	33.75
1 primi 2		H						
34	1	06/01/2014	78	753	1	8.8	0	34.02
1 primi 2		H						
34	1	06/02/2014	79	754	1	8.7	0	32.89
1 primi 2		H						
34	1	06/03/2014	80	755	1	8.7	0	31.75
1 primi 2		H						
34	1	06/04/2014	81	756	1	10.2	0	30.49
1 primi 2		H						
34	1	06/05/2014	82	757	1	9.5	0	31.92
1 primi 2		H						
34	1	06/06/2014	83	758	1	8.9	0	29.94
1 primi 2		H						
34	1	06/07/2014	84	759	1	9.2	0	35.22
1 primi 2		H						
34	1	06/08/2014	85	760	1	8.8	0	33.28
1 primi 2		H						
34	1	06/09/2014	86	761	1	8.9	0	35.43
1 primi 2		H						
34	1	06/10/2014	87	762	1	8.9	0	34.62
1 primi 2		H						
34	1	06/11/2014	88	763	1	9	0	31.97
1 primi 2		H						
34	1	06/12/2014	89	764	1	8.5	0	28.46
1 primi 2		H						
34	1	06/13/2014	90	765	1	8.5	0	33.09
1 primi 2		H						
34	1	06/14/2014	91	766	1	8.3	0	30.91
1 primi 2		H						
34	1	06/15/2014	92	767	1	8.4	0	33.35
1 primi 2		H						
34	1	06/16/2014	93	768	1	8.5	0	33.13
1 primi 2		H						
34	1	06/17/2014	94	769	1	8.7	0	34.33
1 primi 2		H						
34	1	06/18/2014	95	770	1	8.8	0	36.52
1 primi 2		H						
34	1	06/19/2014	96	771	1	8.7	0	33.24
1 primi 2		H						
34	1	06/20/2014	97	772	1	8.7	0	34.5
1 primi 2		H						
34	1	06/21/2014	98	773	1	9.1	0	36.33

1 primi	2	H						
34	1	06/22/2014	99	774	1	8.8	0	36.29
1 primi	2	H						
34	1	06/23/2014	100	775	1	8.8	0	36.2
1 primi	2	H						
34	1	06/24/2014	101	776	1	8.8	0	37.12
1 primi	2	H						
34	1	06/25/2014	102	777	1	8.7	0	35.6
1 primi	2	H						
34	1	06/26/2014	103	778	1	8.8	0.6	36.05
1 primi	2	H						
34	1	06/27/2014	104	779	1	9.2	0	36.61
1 primi	2	H						
34	1	06/28/2014	105	780	1	9.1	0	36.76
1 primi	2	H						
34	1	06/29/2014	106	781	1	9.1	0	37.47
1 primi	2	H						
34	1	06/30/2014	107	782	1	8.8	0.09	34.31
1 primi	2	H						
34	1	07/01/2014	108	783	1	8.9	0.06	37.75
1 primi	2	H						
34	1	07/02/2014	109	784	1	8.8	0	36.56
1 primi	2	H						
34	1	07/03/2014	110	785	1	8.1	0.05	35.74
1 primi	2	H						
34	1	07/04/2014	111	786	1	8.8	0.08	33.62
1 primi	2	H						
34	1	07/05/2014	112	787	1	8.9	0	36.41
1 primi	2	H						
34	1	07/06/2014	113	788	1	8.6	0	37.79
1 primi	2	H						
34	1	07/07/2014	114	789	1	8.8	0	35.02
1 primi	2	H						
34	1	07/08/2014	115	790	1	9.1	0	36.29
1 primi	2	H						
34	1	07/09/2014	116	791	1	8.7	0.04	31.46
1 primi	2	H						
34	1	07/10/2014	117	792	1	8.8	0	35.45
1 primi	2	H						
34	1	07/11/2014	118	793	1	8.8	0	34.22
1 primi	2	H						
34	1	07/12/2014	119	794	1	8.5	0	35.94
1 primi	2	H						
34	1	07/13/2014	120	795	1	9.1	0	35.43
1 primi	2	H						
34	1	07/14/2014	121	796	1	9.2	0	36.52
1 primi	2	H						
34	1	07/15/2014	122	797	1	9.1	0	36.08
1 primi	2	H						
34	1	07/16/2014	123	798	1	9.1	0.53	35.72
1 primi	2	H						
34	1	07/17/2014	124	799	1	9.2	0	36.86
1 primi	2	H						
34	1	07/18/2014	125	800	1	8.9	0	33.22

1 primi 2		H							
34	1	07/19/2014	126	801	1	8.9	0	32.47	
1 primi 2		H							
34	1	07/20/2014	127	802	1	8.8	0	32.86	
1 primi 2		H							
34	1	07/21/2014	128	803	1	9.2	0	31.08	
1 primi 2		H							
34	1	07/22/2014	129	804	1	9.2	0	33.08	
1 primi 2		H							
34	1	07/23/2014	130	805	1	9.1	0.07	30.81	
1 primi 2		H							
34	1	07/24/2014	131	806	1	9.5	0	32.86	
1 primi 2		H							
34	1	07/25/2014	132	807	1	9.3	0	33.62	
1 primi 2		H							
34	1	07/26/2014	133	808	1	8.9	0	33.65	
1 primi 2		H							
34	1	07/27/2014	134	809	1	9.2	0	35.92	
1 primi 2		H							
34	1	07/28/2014	135	810	1	8.8	0.04	33.11	
1 primi 2		H							
34	1	07/29/2014	136	811	1	9.2	0.03	32.48	
1 primi 2		H							
34	1	07/30/2014	137	812	1	9.1	0	34.44	
1 primi 2		H							
34	1	07/31/2014	138	813	1	8.8	0.07	31.95	
1 primi 2		H							
34	1	08/01/2014	139	814	1	9.3	0.02	34.85	
1 primi 2		H							
34	1	08/02/2014	140	815	1	9.3	0	34.69	
1 primi 2		H							
34	1	08/03/2014	141	816	1	9.3	0	35.52	
1 primi 2		H							
34	1	08/04/2014	142	817	1	8.9	0	33.87	
1 primi 2		H							
34	1	08/05/2014	143	818	1	9.1	0	35.39	
1 primi 2		H							
34	1	08/06/2014	144	819	1	9.2	0	34.47	
1 primi 2		H							
34	1	08/07/2014	145	820	1	9.1	0	35.6	
1 primi 2		H							
34	1	08/08/2014	146	821	1	8.9	0	35.99	
1 primi 2		H							
34	1	08/09/2014	147	822	1	9.2	0	35.29	
1 primi 2		H							
34	1	08/10/2014	148	823	1	9.2	0	33.7	
1 primi 2		H							
34	1	08/11/2014	149	824	1	9.1	0	34.59	
1 primi 2		H							
34	1	08/12/2014	150	825	1	9.1	0	32.71	
1 primi 2		H							
34	1	08/13/2014	151	826	1	8.8	0	32.69	
1 primi 2		H							
34	1	08/14/2014	152	827	1	8.9	0	35.27	

1 primi	2	H						
34	1	08/15/2014	153	828	1	8.9	0	34.76
1 primi	2	H						
34	1	08/16/2014	154	829	1	8.9	0	35.66
1 primi	2	H						
34	1	08/17/2014	155	830	1	8.9	0	35.94
1 primi	2	H						
34	1	08/18/2014	156	831	1	9.1	0	35.43
1 primi	2	H						
34	1	08/19/2014	157	832	1	9.2	0	35.08
1 primi	2	H						
34	1	08/20/2014	158	833	1	8.8	0.01	35.59
1 primi	2	H						
34	1	08/21/2014	159	834	1	9.1	0	36.19
1 primi	2	H						
34	1	08/22/2014	160	835	1	8.7	0.06	34.04
1 primi	2	H						
34	1	08/23/2014	161	836	1	8.8	0	35.32
1 primi	2	H						
34	1	08/24/2014	162	837	1	9.1	0	33.37
1 primi	2	H						
34	1	08/25/2014	163	838	1	8.8	0	34.09
1 primi	2	H						
34	1	08/26/2014	164	839	1	9.1	0	33.97
1 primi	2	H						
34	1	05/11/2015	5	1097	2	9.6	0	30.44
2 multi	1	H						
34	1	05/12/2015	6	1098	2	9.1	0	27.51
2 multi	1	H						
34	1	05/13/2015	7	1099	2	9.1	0	30.24
2 multi	1	H						
34	1	05/14/2015	8	1100	2	8.8	0.28	35.57
2 multi	1	H						
34	1	05/15/2015	9	1101	2	8.9	0	39.64
2 multi	1	H						
34	1	05/16/2015	10	1102	2	8.8	0	40.1
2 multi	1	H						
34	1	05/17/2015	11	1103	2	8.9	0	37.32
2 multi	1	H						
34	1	05/18/2015	12	1104	2	9.1	0	42.99
2 multi	1	H						
34	1	05/19/2015	13	1105	2	8.8	0	44.72
2 multi	1	H						
34	1	05/20/2015	14	1106	2	9.1	0	46.74
2 multi	1	H						
34	1	05/21/2015	15	1107	2	9.2	0	45.91
2 multi	1	H						
34	1	05/22/2015	16	1108	2	9.3	0	49.15
2 multi	1	H						
34	1	05/23/2015	17	1109	2	9.2	0	49.67
2 multi	1	H						
34	1	05/24/2015	18	1110	2	9.1	0.07	49.2
2 multi	1	H						
34	1	05/25/2015	19	1111	2	9.3	0	52.48

2 multi	1	H						
34	1	05/26/2015	20	1112	2	9.2	0	56.13
2 multi	1	H						
34	1	05/27/2015	21	1113	2	8.9	0	51.41
2 multi	1	H						
34	1	05/28/2015	22	1114	2	8.8	0.45	53.91
2 multi	2	H						
34	1	05/29/2015	23	1115	2	8.7	0.07	52.8
2 multi	2	H						
34	1	05/30/2015	24	1116	2	8.9	0	54.37
2 multi	2	H						
34	1	05/31/2015	25	1117	2	8.5	0	48.7
2 multi	2	H						
34	1	06/01/2015	26	1118	2	8.7	0	52.69
2 multi	2	H						
34	1	06/02/2015	27	1119	2	8.4	0	51.97
2 multi	2	H						
34	1	06/03/2015	28	1120	2	8.9	0	51.27
2 multi	2	H						
34	1	06/04/2015	29	1121	2	8.6	0	51.66
2 multi	2	H						
34	1	06/05/2015	30	1122	2	8.9	0	53.41
2 multi	2	H						
34	1	06/06/2015	31	1123	2	8.8	0	52.27
2 multi	2	H						
34	1	06/07/2015	32	1124	2	10.9	0.01	50.64
2 multi	2	H						
34	1	06/08/2015	33	1125	2	8.9	0	51.1
2 multi	2	H						
34	1	06/09/2015	34	1126	2	8.8	0.08	54.83
2 multi	2	H						
34	1	06/10/2015	35	1127	2	9.2	0.34	55.38
2 multi	2	H						
34	1	06/11/2015	36	1128	2	8.7	0	55.05
2 multi	2	H						
34	1	06/12/2015	37	1129	2	8.8	0.08	54.31
2 multi	2	H						
34	1	06/13/2015	38	1130	2	8.5	0	47.02
2 multi	2	H						
34	1	06/14/2015	39	1131	2	8.6	0	54.27
2 multi	2	H						
34	1	06/15/2015	40	1132	2	8.6	0	46.95
2 multi	2	H						
34	1	06/16/2015	41	1133	2	8.7	0	49.69
2 multi	2	H						
34	1	06/17/2015	42	1134	2	8.7	0	48.41
2 multi	2	H						
34	1	06/18/2015	43	1135	2	8.9	0	47.01
2 multi	2	H						
34	1	06/19/2015	44	1136	2	9.3	0	49.46
2 multi	2	H						
34	1	06/20/2015	45	1137	2	9.3	0	53.82
2 multi	2	H						
34	1	06/21/2015	46	1138	2	9.2	0	54.56

2 multi 2		H						
34	1	06/22/2015	47	1139	2	9.3	0	52.61
2 multi 2		H						
34	1	06/23/2015	48	1140	2	9.2	0.13	51.43
2 multi 2		H						
34	1	06/24/2015	49	1141	2	9.1	0.06	52.47
2 multi 2		H						
34	1	06/25/2015	50	1142	2	9.2	0	53.14
2 multi 2		H						
34	1	06/26/2015	51	1143	2	8.8	0	49.37
2 multi 2		H						
34	1	06/27/2015	52	1144	2	9.2	0	53.4
2 multi 2		H						
34	1	06/28/2015	53	1145	2	8.9	0	51.8
2 multi 2		H						
34	1	06/29/2015	54	1146	2	8.9	0.19	50.8
2 multi 2		H						
34	1	06/30/2015	55	1147	2	8.8	0.09	51.1
2 multi 2		H						
34	1	07/01/2015	56	1148	2	9.1	0	51.71
2 multi 2		H						
34	1	07/02/2015	57	1149	2	8.6	0.52	49.56
2 multi 2		H						
34	1	07/03/2015	58	1150	2	8.9	0	51.25
2 multi 2		H						
34	1	07/04/2015	59	1151	2	8.8	0	49.83
2 multi 2		H						
34	1	07/05/2015	60	1152	2	8.7	0.01	46.29
2 multi 2		H						
34	1	07/06/2015	61	1153	2	8.7	0	45.11
2 multi 2		H						
34	1	07/07/2015	62	1154	2	8.8	0.13	43.38
2 multi 2		H						
34	1	07/08/2015	63	1155	2	8.7	0	44.17
2 multi 2		H						
34	1	07/09/2015	64	1156	2	9.3	0.04	44.22
2 multi 2		H						
34	1	07/10/2015	65	1157	2	9.3	0.07	41.55
2 multi 2		H						
34	1	07/11/2015	66	1158	2	9.3	0	42.06
2 multi 2		H						
34	1	07/12/2015	67	1159	2	9.2	0	48.46
2 multi 2		H						
34	1	07/13/2015	68	1160	2	9.1	0	43.42
2 multi 2		H						
34	1	07/14/2015	69	1161	2	8.9	0.08	46.85
2 multi 2		H						
34	1	07/15/2015	70	1162	2	9.1	0	48.52
2 multi 2		H						
34	1	07/16/2015	71	1163	2	9.1	0.03	47.72
2 multi 2		H						
34	1	07/17/2015	72	1164	2	9.2	0.1	56.14
2 multi 2		H						
34	1	07/18/2015	73	1165	2	8.9	0	47.91

2 multi 2		H						
34	1	07/19/2015	74	1166	2	9.1	0	48.38
2 multi 2		H						
34	1	07/20/2015	75	1167	2	8.9	0	48.29
2 multi 2		H						
34	1	07/21/2015	76	1168	2	8.8	0	47.39
2 multi 2		H						
34	1	07/22/2015	77	1169	2	9.2	0.01	44.13
2 multi 2		H						
34	1	07/23/2015	78	1170	2	9.3	0	47.3
2 multi 2		H						
34	1	07/24/2015	79	1171	2	9.6	0	45.34
2 multi 2		H						
34	1	07/25/2015	80	1172	2	9.1	0.04	36.08
2 multi 2		H						
34	1	07/26/2015	81	1173	2	8.8	0	41.63
2 multi 2		H						
34	1	07/27/2015	82	1174	2	9.2	0.05	39.32
2 multi 2		H						
34	1	07/28/2015	83	1175	2	9.2	0.05	43.3
2 multi 2		H						
34	1	07/29/2015	84	1176	2	9.2	0.23	45.25
2 multi 2		H						
34	1	07/30/2015	85	1177	2	9.6	0.12	43.7
2 multi 2		H						
34	1	07/31/2015	86	1178	2	9.3	0	52.04
2 multi 2		H						
34	1	08/01/2015	87	1179	2	9.5	0	49.92
2 multi 2		H						
34	1	08/02/2015	88	1180	2	9.1	0	47.62
2 multi 2		H						
34	1	08/03/2015	89	1181	2	8.9	0	42.17
2 multi 2		H						
34	1	08/04/2015	90	1182	2	8.7	0	49.23
2 multi 2		H						
34	1	08/05/2015	91	1183	2	8.7	0.11	45.32
2 multi 2		H						
34	1	08/06/2015	92	1184	2	9.3	0	47.62
2 multi 2		H						
34	1	08/07/2015	93	1185	2	9.1	0.01	43.87
2 multi 2		H						
34	1	08/08/2015	94	1186	2	9.2	0	38.97
2 multi 2		H						
34	1	08/09/2015	95	1187	2	8.9	0	42.76
2 multi 2		H						
34	1	08/10/2015	96	1188	2	8.9	0	45.49
2 multi 2		H						
34	1	08/11/2015	97	1189	2	9.3	0	46.24
2 multi 2		H						
34	1	08/12/2015	98	1190	2	9.3	0.02	42.81
2 multi 2		H						
34	1	08/13/2015	99	1191	2	9.3	0	44.47
2 multi 2		H						
34	1	08/14/2015	100	1192	2	9.3	0	46.93

2 multi	2	H						
34	1	08/15/2015	101	1193	2	9.3	0.1	41.98
2 multi	2	H						
34	1	08/16/2015	102	1194	2	9.2	0.08	64.86
2 multi	2	H						
34	1	08/17/2015	103	1195	2	9.2	0	42.09
2 multi	2	H						
34	1	08/18/2015	104	1196	2	9.3	0.02	44.36
2 multi	2	H						
34	1	08/19/2015	105	1197	2	9.1	0	41.51
2 multi	2	H						
34	1	08/20/2015	106	1198	2	9.5	0.13	45.84
2 multi	2	H						
34	1	08/21/2015	107	1199	2	8.9	0	41.54
2 multi	2	H						
34	1	08/22/2015	108	1200	2	8.9	0	43
2 multi	2	H						
34	1	08/23/2015	109	1201	2	8.8	0.04	37.75
2 multi	2	H						
34	1	08/24/2015	110	1202	2	8.8	0	34.05
2 multi	2	H						
34	1	08/25/2015	111	1203	2	9.1	0	38.32
2 multi	2	H						
34	1	08/26/2015	112	1204	2	9.1	0	41.16
2 multi	2	H						
34	1	08/27/2015	113	1205	2	9.3	0	42.04
2 multi	2	H						
34	1	08/28/2015	114	1206	2	9.3	0	42.27
2 multi	2	H						
34	1	08/29/2015	115	1207	2	9.3	0	39.19
2 multi	2	H						
34	1	08/30/2015	116	1208	2	9.1	0	40.63
2 multi	2	H						
34	1	08/31/2015	117	1209	2	9.1	0	35.86
2 multi	2	H						
34	1	09/01/2015	118	1210	2	9.5	0	36.82
2 multi	2	H						
34	1	09/02/2015	119	1211	2	9.1	0.02	38.92
2 multi	2	H						
34	1	12/19/2017	6	2050	4	8.7	0	27.9
2 multi	1	H						
34	1	12/20/2017	7	2051	4	8.6	0.04	31.14
2 multi	1	H						
34	1	12/21/2017	8	2052	4	8.3	0	31.06
2 multi	1	H						
34	1	12/22/2017	9	2053	4	8.7	0.02	33.24
2 multi	1	H						
34	1	12/23/2017	10	2054	4	8.3	0	31.45
2 multi	1	H						
34	1	12/24/2017	11	2055	4	8.6	0.03	36.44
2 multi	1	H						
34	1	12/25/2017	12	2056	4	8.6	0	35.53
2 multi	1	H						
34	1	12/26/2017	13	2057	4	8.4	0.14	37.09

2 multi	1	H						
34	1	12/27/2017	14	2058	4	8.4	0.11	37.13
2 multi	1	H						
34	1	12/28/2017	15	2059	4	8.8	0.41	33.07
2 multi	1	H						
34	1	12/29/2017	16	2060	4	8.4	0	35.54
2 multi	1	H						
34	1	12/30/2017	17	2061	4	8.7	0	35.9
2 multi	1	H						
34	1	12/31/2017	18	2062	4	8.6	0.39	42.53
2 multi	1	H						
34	1	01/01/2018	19	2063	4	8.5	0	39.29
2 multi	1	H						
34	1	01/02/2018	20	2064	4	8.4	0	37.96
2 multi	1	H						
34	1	01/03/2018	21	2065	4	8.5	0	39.13
2 multi	1	H						
34	1	01/04/2018	22	2066	4	8.4	0.05	36.47
2 multi	2	H						
34	1	01/05/2018	23	2067	4	8.3	0.04	40.18
2 multi	2	H						
34	1	01/06/2018	24	2068	4	8.6	0.04	40.68
2 multi	2	H						
34	1	01/07/2018	25	2069	4	8.6	0	41.91
2 multi	2	H						
34	1	01/08/2018	26	2070	4	8.8	0.06	41.41
2 multi	2	H						
34	1	01/09/2018	27	2071	4	8.5	0	43.39
2 multi	2	H						
34	1	01/10/2018	28	2072	4	8.7	0	42.78
2 multi	2	H						
34	1	01/11/2018	29	2073	4	8.6	0	45.08
2 multi	2	H						
34	1	01/12/2018	30	2074	4	8.3	0.01	38.86
2 multi	2	H						
34	1	01/13/2018	31	2075	4	8.6	0	44.38
2 multi	2	H						
34	1	01/14/2018	32	2076	4	8.4	0	40.98
2 multi	2	H						
34	1	01/15/2018	33	2077	4	8.2	0	39.56
2 multi	2	H						
34	1	01/16/2018	34	2078	4	8.6	0	44.07
2 multi	2	H						
34	1	01/17/2018	35	2079	4	8.5	0	42.15
2 multi	2	H						
34	1	01/18/2018	36	2080	4	8.5	0.1	39.34
2 multi	2	H						
34	1	01/19/2018	37	2081	4	8.3	0	40.11
2 multi	2	H						
34	1	01/20/2018	38	2082	4	8.4	0	41.23
2 multi	2	H						
34	1	01/21/2018	39	2083	4	8.5	0	43.13
2 multi	2	H						
34	1	01/22/2018	40	2084	4	8.6	0	43.91

2 multi 2		H						
34	1	01/23/2018	41	2085	4	8.6	0	43.89
2 multi 2		H						
34	1	01/24/2018	42	2086	4	8.6	0	42.63
2 multi 2		H						
34	1	01/25/2018	43	2087	4	8.5	0	42.35
2 multi 2		H						
34	1	01/26/2018	44	2088	4	8.5	0	41.48
2 multi 2		H						
34	1	01/27/2018	45	2089	4	8.6	0	46
2 multi 2		H						
34	1	01/28/2018	46	2090	4	8.6	0.04	41.37
2 multi 2		H						
34	1	01/29/2018	47	2091	4	8.7	0.05	46.84
2 multi 2		H						
34	1	01/30/2018	48	2092	4	8.6	0	42.5
2 multi 2		H						
34	1	01/31/2018	49	2093	4	8.4	0	46.92
2 multi 2		H						
34	1	02/01/2018	50	2094	4	8.4	0	44.8
2 multi 2		H						
34	1	02/02/2018	51	2095	4	8.4	0.2	42.89
2 multi 2		H						
34	1	02/03/2018	52	2096	4	8.3	0.09	42.31
2 multi 2		H						
34	1	02/04/2018	53	2097	4	8.2	0	40.05
2 multi 2		H						
34	1	02/05/2018	54	2098	4	8.5	0	43.76
2 multi 2		H						
34	1	02/06/2018	55	2099	4	8.4	0.04	40.69
2 multi 2		H						
34	1	02/07/2018	56	2100	4	8.5	0	45.75
2 multi 2		H						
34	1	02/08/2018	57	2101	4	8.6	0.14	45.28
2 multi 2		H						
34	1	02/09/2018	58	2102	4	8.6	0.06	45.36
2 multi 2		H						
34	1	02/10/2018	59	2103	4	8.2	0.1	38.96
2 multi 2		H						
34	1	02/11/2018	60	2104	4	8.8	0.04	43.57
2 multi 2		H						
34	1	02/12/2018	61	2105	4	8.2	0.06	35.23
2 multi 2		H						
34	1	02/13/2018	62	2106	4	8.6	0.82	41.82
2 multi 2		H						
34	1	02/14/2018	63	2107	4	8.4	0	41.84
2 multi 2		H						
34	1	02/15/2018	64	2108	4	8.7	0.04	43.73
2 multi 2		H						
34	1	02/16/2018	65	2109	4	8.8	0.05	44.92
2 multi 2		H						
34	1	02/17/2018	66	2110	4	8.5	0	42.16
2 multi 2		H						
34	1	02/18/2018	67	2111	4	8.4	0	46.32

2 multi 2		H						
34	1	02/19/2018	68	2112	4	8.7	0.62	44.95
2 multi 2		H						
34	1	02/20/2018	69	2113	4	8.6	0	42.13
2 multi 2		H						
34	1	02/21/2018	70	2114	4	8.5	0.05	44.44
2 multi 2		H						
34	1	02/22/2018	71	2115	4	8.4	0	43.14
2 multi 2		H						
34	1	02/23/2018	72	2116	4	8.5	0	43.83
2 multi 2		H						
34	1	02/24/2018	73	2117	4	8.7	0.05	47.72
2 multi 2		H						
34	1	02/25/2018	74	2118	4	8.6	0.11	44.76
2 multi 2		H						
34	1	02/26/2018	75	2119	4	8.8	0	45.76
2 multi 2		H						
34	1	02/27/2018	76	2120	4	8.6	0.06	42.23
2 multi 2		H						
34	1	02/28/2018	77	2121	4	8.4	0.05	42.69
2 multi 2		H						
34	1	03/01/2018	78	2122	4	8.6	0.06	43.8
2 multi 2		H						
34	1	03/02/2018	79	2123	4	8.6	0.06	43.66
2 multi 2		H						
34	1	03/03/2018	80	2124	4	8.3	0	44.8
2 multi 2		H						
34	1	03/04/2018	81	2125	4	8.5	0	45.66
2 multi 2		H						
34	1	03/05/2018	82	2126	4	8.4	0	40.54
2 multi 2		H						
34	1	03/06/2018	83	2127	4	8.4	0.04	41.8
2 multi 2		H						
34	1	03/07/2018	84	2128	4	8.5	0.05	43.3
2 multi 2		H						
34	1	03/08/2018	85	2129	4	8.4	0	45.02
2 multi 2		H						
34	1	03/09/2018	86	2130	4	8.5	0	43.35
2 multi 2		H						
34	1	03/10/2018	87	2131	4	8.7	0.15	40.01
2 multi 2		H						
34	1	03/11/2018	88	2132	4	8.2	0.18	36.92
2 multi 2		H						
34	1	03/12/2018	89	2133	4	8.7	0.05	41
2 multi 2		H						
34	1	03/13/2018	90	2134	4	8.5	0	42.16
2 multi 2		H						
34	1	03/14/2018	91	2135	4	8.7	0	41.53
2 multi 2		H						
34	1	03/15/2018	92	2136	4	8.2	0	43.02
2 multi 2		H						
34	1	03/16/2018	93	2137	4	8.7	0	43.07
2 multi 2		H						
34	1	03/17/2018	94	2138	4	8.2	0	42.94

2 multi 2		H						
34	1	03/18/2018	95	2139	4	8.7	0	43.12
2 multi 2		H						
34	1	12/19/2018	5	2415	5	9.1	0.16	32.81
2 multi 1		H						
34	1	12/20/2018	6	2416	5	9.1	0	34.11
2 multi 1		H						
34	1	12/21/2018	7	2417	5	8.9	0.18	31.58
2 multi 1		H						
34	1	12/22/2018	8	2418	5	8.9	0	33.78
2 multi 1		H						
34	1	12/23/2018	9	2419	5	8.8	0	33.85
2 multi 1		H						
34	1	12/24/2018	10	2420	5	9.2	0.05	36.7
2 multi 1		H						
34	1	12/25/2018	11	2421	5	8.9	0.06	41.07
2 multi 1		H						
34	1	12/26/2018	12	2422	5	9.1	0.02	36.44
2 multi 1		H						
34	1	12/27/2018	13	2423	5	8.9	0.03	42.89
2 multi 1		H						
34	1	12/28/2018	14	2424	5	8.8	0.08	39.33
2 multi 1		H						
34	1	12/29/2018	15	2425	5	8.8	0	42.75
2 multi 1		H						
34	1	12/30/2018	16	2426	5	8.7	0	40.34
2 multi 1		H						
34	1	12/31/2018	17	2427	5	8.9	0.06	40.28
2 multi 1		H						
34	1	01/01/2019	18	2428	5	9.1	0	44.75
2 multi 1		H						
34	1	01/02/2019	19	2429	5	8.7	0	41.74
2 multi 1		H						
34	1	01/03/2019	20	2430	5	8.9	0.08	43.8
2 multi 1		H						
34	1	01/04/2019	21	2431	5	9.1	0.07	42.55
2 multi 1		H						
34	1	01/05/2019	22	2432	5	8.6	0	36.98
2 multi 2		H						
34	1	01/06/2019	23	2433	5	8.6	0	41.78
2 multi 2		H						
34	1	01/07/2019	24	2434	5	8.9	0	43.02
2 multi 2		H						
34	1	01/08/2019	25	2435	5	8.9	0	42.61
2 multi 2		H						
34	1	01/09/2019	26	2436	5	9.1	0	43.91
2 multi 2		H						
34	1	01/10/2019	27	2437	5	8.9	0.08	40.58
2 multi 2		H						
34	1	01/11/2019	28	2438	5	8.9	0.07	44.09
2 multi 2		H						
34	1	01/12/2019	29	2439	5	8.8	0	44.65
2 multi 2		H						
34	1	01/13/2019	30	2440	5	8.9	0	42.59

2 multi	2	H						
34	1	01/14/2019	31	2441	5	9.1	0	41.99
2 multi	2	H						
34	1	01/15/2019	32	2442	5	8.7	0	39.86
2 multi	2	H						
34	1	01/16/2019	33	2443	5	8.8	0	39.92
2 multi	2	H						
34	1	01/17/2019	34	2444	5	8.9	0.28	40.14
2 multi	2	H						
34	1	01/18/2019	35	2445	5	9.2	0	44.09
2 multi	2	H						
34	1	01/19/2019	36	2446	5	9.1	0	40.09
2 multi	2	H						
34	1	01/20/2019	37	2447	5	9.3	0.06	47.32
2 multi	2	H						
34	1	01/21/2019	38	2448	5	9.2	0.04	45.04
2 multi	2	H						
34	1	01/22/2019	39	2449	5	8.8	0	41.62
2 multi	2	H						
34	1	01/23/2019	40	2450	5	8.8	0	41.44
2 multi	2	H						
34	1	01/24/2019	41	2451	5	9.5	0	46.26
2 multi	2	H						
34	1	01/25/2019	42	2452	5	9.2	0	43
2 multi	2	H						
34	1	01/26/2019	43	2453	5	9.1	0	37.49
2 multi	2	H						
34	1	01/27/2019	44	2454	5	9.3	0	42.62
2 multi	2	H						
34	1	01/28/2019	45	2455	5	9.3	0.05	45.1
2 multi	2	H						
34	1	01/29/2019	46	2456	5	9.3	0	45.21
2 multi	2	H						
34	1	01/30/2019	47	2457	5	9.2	0.1	43.3
2 multi	2	H						
34	1	01/31/2019	48	2458	5	9.2	0	45.2
2 multi	2	H						
34	1	02/01/2019	49	2459	5	9.5	0	40.25
2 multi	2	H						
34	1	02/02/2019	50	2460	5	9.2	0.05	41.13
2 multi	2	H						
34	1	02/03/2019	51	2461	5	8.9	0	42.39
2 multi	2	H						
34	1	02/04/2019	52	2462	5	9.3	0.05	44.91
2 multi	2	H						
34	1	02/05/2019	53	2463	5	8.8	0	37.86
2 multi	2	H						
34	1	02/06/2019	54	2464	5	9.2	0.07	46.94
2 multi	2	H						
34	1	02/07/2019	55	2465	5	9.3	0.2	43.9
2 multi	2	H						
34	1	02/08/2019	56	2466	5	9.1	0	42.37
2 multi	2	H						
34	1	02/09/2019	57	2467	5	9.1	0	47.13

2 multi 2		H						
34	1	02/10/2019	58	2468	5	9.3	0	43.9
2 multi 2		H						
34	1	02/11/2019	59	2469	5	9.1	0.23	45.63
2 multi 2		H						
34	1	02/12/2019	60	2470	5	8.9	0	40.77
2 multi 2		H						
34	1	02/13/2019	61	2471	5	9.2	0.05	42.33
2 multi 2		H						
34	1	02/14/2019	62	2472	5	9.5	0	41.52
2 multi 2		H						
34	1	02/15/2019	63	2473	5	9.2	0.21	43.06
2 multi 2		H						
34	1	02/16/2019	64	2474	5	8.9	0	40.32
2 multi 2		H						
34	1	02/17/2019	65	2475	5	9.3	0.11	42.17
2 multi 2		H						
34	1	02/18/2019	66	2476	5	9.6	0	44.13
2 multi 2		H						
34	1	02/19/2019	67	2477	5	9.2	0	37.64
2 multi 2		H						
34	1	02/20/2019	68	2478	5	9.2	0	39.72
2 multi 2		H						
34	1	02/21/2019	69	2479	5	9.6	0.05	44.17
2 multi 2		H						
34	1	02/22/2019	70	2480	5	9.5	0.05	43.74
2 multi 2		H						
34	1	02/23/2019	71	2481	5	9.3	0	38.87
2 multi 2		H						
34	1	02/24/2019	72	2482	5	9.8	0	39.74
2 multi 2		H						
34	1	02/25/2019	73	2483	5	9.6	0.05	42.12
2 multi 2		H						
34	1	02/26/2019	74	2484	5	9.6	0	30.68
2 multi 2		H						
34	1	02/27/2019	75	2485	5	9.9	0	49.62
2 multi 2		H						
34	1	02/28/2019	76	2486	5	9.9	0	42.65
2 multi 2		H						
34	1	03/01/2019	77	2487	5	9.8	0	42.29
2 multi 2		H						
34	1	03/02/2019	78	2488	5	9.5	0.15	41.81
2 multi 2		H						
34	1	03/03/2019	79	2489	5	9.5	0.13	42.36
2 multi 2		H						
34	1	03/04/2019	80	2490	5	9.5	0	37.95
2 multi 2		H						
34	1	03/05/2019	81	2491	5	9.5	0	38.94
2 multi 2		H						
34	1	03/06/2019	82	2492	5	9.2	0.27	38.36
2 multi 2		H						
34	1	03/07/2019	83	2493	5	9.6	0	39.5
2 multi 2		H						
34	1	03/08/2019	84	2494	5	9.5	0.05	41.82

2 multi	2	H						
34	1	03/09/2019	85	2495	5	9.3	0	39.85
2 multi	2	H						
34	1	03/10/2019	86	2496	5	9.3	0.1	36.24
2 multi	2	H						
34	1	03/11/2019	87	2497	5	9.5	0.04	39.13
2 multi	2	H						
34	1	03/12/2019	88	2498	5	9.5	0.04	41.8
2 multi	2	H						
34	1	03/13/2019	89	2499	5	9.3	0	34.48
2 multi	2	H						
34	1	03/14/2019	90	2500	5	9.3	0	42.18
2 multi	2	H						
34	1	03/15/2019	91	2501	5	9.6	0.04	37.36
2 multi	2	H						
34	1	03/16/2019	92	2502	5	8.9	0.05	37.25
2 multi	2	H						
34	1	03/17/2019	93	2503	5	9.3	0.06	39.53
2 multi	2	H						
34	1	03/18/2019	94	2504	5	9.8	0.16	41.6
2 multi	2	H						
34	1	03/19/2019	95	2505	5	9.6	0	37.46
2 multi	2	H						
34	1	03/20/2019	96	2506	5	9.6	0.2	42.2
2 multi	2	H						
34	1	03/21/2019	97	2507	5	9.5	0.15	42.29
2 multi	2	H						
34	1	03/22/2019	98	2508	5	9.5	0.06	41.36
2 multi	2	H						
34	1	03/23/2019	99	2509	5	9.8	0	36.8
2 multi	2	H						
34	1	03/24/2019	100	2510	5	9.3	0	33.76
2 multi	2	H						
34	1	03/25/2019	101	2511	5	9.6	0	41.31
2 multi	2	H						
34	1	03/26/2019	102	2512	5	9.1	0.14	38.06
2 multi	2	H						
34	1	03/27/2019	103	2513	5	9.2	0.07	33.19
2 multi	2	H						
34	1	01/01/2020	5	2793	6	9.1	0.1	31.27
2 multi	1	H						
34	1	01/02/2020	6	2794	6	9.2	0.03	33.65
2 multi	1	H						
34	1	01/03/2020	7	2795	6	9.1	0.02	33.51
2 multi	1	H						
34	1	01/04/2020	8	2796	6	9.2	0	30.69
2 multi	1	H						
34	1	01/05/2020	9	2797	6	8.8	0.12	35.14
2 multi	1	H						
34	1	01/06/2020	10	2798	6	8.8	0.01	33.59
2 multi	1	H						
34	1	01/07/2020	11	2799	6	9.1	0	36.72
2 multi	1	H						
34	1	01/08/2020	12	2800	6	9.2	0	36.97

2 multi	1	H						
34	1	01/09/2020	13	2801	6	8.8	0.15	37.4
2 multi	1	H						
34	1	01/10/2020	14	2802	6	8.8	0	37.68
2 multi	1	H						
34	1	01/11/2020	15	2803	6	8.9	0	39.24
2 multi	1	H						
34	1	01/12/2020	16	2804	6	8.7	0	38.26
2 multi	1	H						
34	1	01/13/2020	17	2805	6	8.6	0.09	37.96
2 multi	1	H						
34	1	01/14/2020	18	2806	6	8.7	0	39.03
2 multi	1	H						
34	1	01/15/2020	19	2807	6	8.7	0.04	40.5
2 multi	1	H						
34	1	01/16/2020	20	2808	6	8.6	0.08	40.14
2 multi	1	H						
34	1	01/17/2020	21	2809	6	8.6	0.06	41.75
2 multi	1	H						
34	1	01/18/2020	22	2810	6	8.6	0.08	39.71
2 multi	2	H						
34	1	01/19/2020	23	2811	6	8.7	0.08	42.28
2 multi	2	H						
34	1	01/20/2020	24	2812	6	8.7	0.07	42.67
2 multi	2	H						
34	1	01/21/2020	25	2813	6	8.8	0	44.64
2 multi	2	H						
34	1	01/22/2020	26	2814	6	8.7	0	42.07
2 multi	2	H						
34	1	01/23/2020	27	2815	6	8.8	0	44.87
2 multi	2	H						
34	1	01/24/2020	28	2816	6	8.7	0.08	44.46
2 multi	2	H						
34	1	01/25/2020	29	2817	6	8.9	0.07	43.03
2 multi	2	H						
34	1	01/26/2020	30	2818	6	8.7	0	47.26
2 multi	2	H						
34	1	01/27/2020	31	2819	6	8.9	0	46.33
2 multi	2	H						
34	1	01/28/2020	32	2820	6	8.7	0.08	45.8
2 multi	2	H						
34	1	01/29/2020	33	2821	6	8.8	0	46.51
2 multi	2	H						
34	1	01/30/2020	34	2822	6	8.6	0	49.5
2 multi	2	H						
34	1	01/31/2020	35	2823	6	8.7	0	49.23
2 multi	2	H						
34	1	02/01/2020	36	2824	6	8.6	0.05	46.32
2 multi	2	H						
34	1	02/02/2020	37	2825	6	8.6	0.06	44.62
2 multi	2	H						
34	1	02/03/2020	38	2826	6	8.8	0	48.73
2 multi	2	H						
34	1	02/04/2020	39	2827	6	8.8	0	48.33

2 multi 2		H						
34	1	02/05/2020	40	2828	6	8.9	0	47.72
2 multi 2		H						
34	1	02/06/2020	41	2829	6	8.8	0	47.07
2 multi 2		H						
34	1	02/07/2020	42	2830	6	8.8	0.08	45.32
2 multi 2		H						
34	1	02/08/2020	43	2831	6	8.9	0.08	49.2
2 multi 2		H						
34	1	02/09/2020	44	2832	6	8.9	0	47.51
2 multi 2		H						
34	1	02/10/2020	45	2833	6	8.8	0	47.72
2 multi 2		H						
34	1	02/11/2020	46	2834	6	8.7	0	46.11
2 multi 2		H						
34	1	02/12/2020	47	2835	6	8.8	0	45.88
2 multi 2		H						
34	1	02/13/2020	48	2836	6	8.8	0.7	48.16
2 multi 2		H						
34	1	02/14/2020	49	2837	6	8.9	0	45.7
2 multi 2		H						
34	1	02/15/2020	50	2838	6	8.9	0	49.44
2 multi 2		H						
34	1	02/16/2020	51	2839	6	8.9	0.06	43.99
2 multi 2		H						
34	1	02/17/2020	52	2840	6	9.1	0.09	47.15
2 multi 2		H						
34	1	02/18/2020	53	2841	6	8.9	0	45.05
2 multi 2		H						
34	1	02/19/2020	54	2842	6	9.2	0	49.61
2 multi 2		H						
34	1	02/20/2020	55	2843	6	9.1	0	48.86
2 multi 2		H						
34	1	02/21/2020	56	2844	6	9.1	0	48.19
2 multi 2		H						
34	1	02/22/2020	57	2845	6	9.2	0	45.69
2 multi 2		H						
34	1	02/23/2020	58	2846	6	9.1	0	44.97
2 multi 2		H						
34	1	02/24/2020	59	2847	6	9.2	0	47.28
2 multi 2		H						
34	1	02/25/2020	60	2848	6	8.9	0.04	50.12
2 multi 2		H						
34	1	02/26/2020	61	2849	6	9.3	0	48.91
2 multi 2		H						
34	1	02/27/2020	62	2850	6	9.2	0.05	47.83
2 multi 2		H						
34	1	02/28/2020	63	2851	6	9.1	0	49.06
2 multi 2		H						
34	1	02/29/2020	64	2852	6	8.9	0.08	44.87
2 multi 2		H						
34	1	03/01/2020	65	2853	6	8.7	0.06	43.01
2 multi 2		H						
34	1	03/02/2020	66	2854	6	9.2	0.06	43.85

2 multi 2		H						
34	1	03/03/2020	67	2855	6	9.1	0.09	45.42
2 multi 2		H						
34	1	03/04/2020	68	2856	6	9.1	0	44.07
2 multi 2		H						
34	1	03/05/2020	69	2857	6	9.1	0.16	43.8
2 multi 2		H						
34	1	03/06/2020	70	2858	6	9.1	0.07	39.02
2 multi 2		H						
34	1	03/07/2020	71	2859	6	9.1	0	39.44
2 multi 2		H						
34	1	03/08/2020	72	2860	6	9.2	0	42.54
2 multi 2		H						
34	1	03/09/2020	73	2861	6	9.1	0	40.04
2 multi 2		H						
34	1	03/10/2020	74	2862	6	9.1	0	40.94
2 multi 2		H						
34	1	03/11/2020	75	2863	6	9.5	0	42.77
2 multi 2		H						
34	1	03/12/2020	76	2864	6	9.2	0	39.95
2 multi 2		H						
34	1	03/13/2020	77	2865	6	9.5	0	43.74
2 multi 2		H						
34	1	03/14/2020	78	2866	6	9.8	0	41.93
2 multi 2		H						
34	1	03/15/2020	79	2867	6	9.6	0.04	40.63
2 multi 2		H						
34	1	03/16/2020	80	2868	6	9.8	0	45.88
2 multi 2		H						
34	1	03/17/2020	81	2869	6	9.6	0.06	42.25
2 multi 2		H						
34	1	03/18/2020	82	2870	6	9.9	0.05	46.97
2 multi 2		H						
34	1	03/19/2020	83	2871	6	9.9	0	46.36
2 multi 2		H						
34	1	03/20/2020	84	2872	6	9.6	0	45.49
2 multi 2		H						
34	1	03/21/2020	85	2873	6	9.6	0	46.12
2 multi 2		H						
34	1	03/22/2020	86	2874	6	9.6	0	45.49
2 multi 2		H						
34	1	03/23/2020	87	2875	6	9.6	0	45.87
2 multi 2		H						
34	1	03/24/2020	88	2876	6	9.5	0	45.56
2 multi 2		H						
34	1	03/25/2020	89	2877	6	9.5	0.05	44.49
2 multi 2		H						
34	1	03/26/2020	90	2878	6	9.5	0	48.06
2 multi 2		H						
34	1	03/27/2020	91	2879	6	9.8	0.05	48.95
2 multi 2		H						
34	1	03/28/2020	92	2880	6	9.8	0	47.83
2 multi 2		H						
34	1	03/29/2020	93	2881	6	9.5	0.04	45.05

2 multi 2		H						
34	1	03/30/2020	94	2882	6	9.6	0	46.32
2 multi 2		H						
34	1	03/31/2020	95	2883	6	9.6	0	45.59
2 multi 2		H						
34	1	04/01/2020	96	2884	6	9.5	0.08	45.15
2 multi 2		H						
34	1	04/02/2020	97	2885	6	9.3	0	42.91
2 multi 2		H						
34	1	04/03/2020	98	2886	6	9.6	0	44.06
2 multi 2		H						
34	1	04/04/2020	99	2887	6	9.8	0	46.86
2 multi 2		H						
34	1	04/05/2020	100	2888	6	9.8	0.08	46.64
2 multi 2		H						
34	1	04/06/2020	101	2889	6	9.8	0	46.09
2 multi 2		H						
34	1	04/07/2020	102	2890	6	9.6	0	46.87
2 multi 2		H						
34	1	04/08/2020	103	2891	6	9.6	0	47.16
2 multi 2		H						
34	1	04/09/2020	104	2892	6	9.9	0.06	45.61
2 multi 2		H						
34	1	04/10/2020	105	2893	6	9.8	0.02	46.17
2 multi 2		H						
34	1	04/11/2020	106	2894	6	9.8	0.08	46.75
2 multi 2		H						
34	1	04/12/2020	107	2895	6	9.8	0	47.97
2 multi 2		H						
34	1	04/13/2020	108	2896	6	9.9	0.06	49.08
2 multi 2		H						
34	1	04/14/2020	109	2897	6	9.9	0.14	45.53
2 multi 2		H						
34	1	04/15/2020	110	2898	6	9.9	0	46.57
2 multi 2		H						
34	1	04/16/2020	111	2899	6	9.6	0	46.51
2 multi 2		H						
34	1	04/17/2020	112	2900	6	10	0	48.14
2 multi 2		H						
34	1	04/18/2020	113	2901	6	9.8	0	46.11
2 multi 2		H						
34	1	04/19/2020	114	2902	6	9.8	0	44.13
2 multi 2		H						
34	1	04/20/2020	115	2903	6	9.9	0.06	44.61
2 multi 2		H						
34	1	04/21/2020	116	2904	6	9.9	0	46.62
2 multi 2		H						
34	1	04/22/2020	117	2905	6	9.9	0	46.17
2 multi 2		H						
34	1	04/23/2020	118	2906	6	9.8	0	44.84
2 multi 2		H						
34	1	04/24/2020	119	2907	6	9.3	0.07	40.9
2 multi 2		H						
34	1	04/25/2020	120	2908	6	9.6	0.06	44.64

2 multi 2		H						
34	1	04/26/2020	121	2909	6	9.8	0.05	43.5
2 multi 2		H						
34	1	04/27/2020	122	2910	6	9.9	0	44.25
2 multi 2		H						
34	1	04/28/2020	123	2911	6	9.9	0	44.2
2 multi 2		H						
34	1	04/29/2020	124	2912	6	9.9	0	45.6
2 multi 2		H						
34	1	04/30/2020	125	2913	6	9.9	0.04	42.91
2 multi 2		H						
34	1	05/01/2020	126	2914	6	9.8	0.04	44.37
2 multi 2		H						
34	1	05/02/2020	127	2915	6	9.9	0	45.44
2 multi 2		H						
34	1	05/03/2020	128	2916	6	9.9	0	44.9
2 multi 2		H						
34	1	05/04/2020	129	2917	6	9.9	0.05	46.51
2 multi 2		H						
34	1	05/05/2020	130	2918	6	9.9	0	36.64
2 multi 2		H						
34	1	05/06/2020	131	2919	6	9.8	0.06	45.83
2 multi 2		H						
34	1	05/07/2020	132	2920	6	10	0	44
2 multi 2		H						
34	1	05/08/2020	133	2921	6	10	0	45.14
2 multi 2		H						
34	1	05/09/2020	134	2922	6	9.9	0	45.57
2 multi 2		H						
34	1	05/10/2020	135	2923	6	9.9	0	41.7
2 multi 2		H						
34	1	05/11/2020	136	2924	6	9.9	0.05	40.86
2 multi 2		H						
34	1	05/12/2020	137	2925	6	9.9	0	41.44
2 multi 2		H						
34	1	05/13/2020	138	2926	6	10.2	0	41.48
2 multi 2		H						
34	1	05/14/2020	139	2927	6	10.3	0.04	40.27
2 multi 2		H						
34	1	05/15/2020	140	2928	6	9.8	0	40.67
2 multi 2		H						
34	1	05/16/2020	141	2929	6	10	0.05	37.45
2 multi 2		H						
34	1	05/17/2020	142	2930	6	10	0.05	39.47
2 multi 2		H						
34	1	05/18/2020	143	2931	6	9.9	0.06	36.29
2 multi 2		H						
34	1	05/19/2020	144	2932	6	9.9	0.07	36.79
2 multi 2		H						
34	1	05/20/2020	145	2933	6	9.9	0.08	36.08
2 multi 2		H						
34	1	05/21/2020	146	2934	6	10	0.07	37.75
2 multi 2		H						
34	1	06/30/2020	186	2974	6	10.4	0	32.18

2 multi 2		H						
34	1	07/01/2020	187	2975	6	10.6	0	31.5
2 multi 2		H						
34	1	07/02/2020	188	2976	6	10.3	0	32.55
2 multi 2		H						
34	1	07/03/2020	189	2977	6	9.9	0.16	29.84
2 multi 2		H						
34	1	07/04/2020	190	2978	6	9.9	0.21	31.01
2 multi 2		H						
34	1	07/05/2020	191	2979	6	10.3	0	35.93
2 multi 2		H						
34	1	07/06/2020	192	2980	6	9.9	0.19	33.46
2 multi 2		H						
34	1	07/07/2020	193	2981	6	10.2	0	32.85
2 multi 2		H						
34	1	07/08/2020	194	2982	6	9.9	0	35.32
2 multi 2		H						
34	1	12/04/2020	10	3131	7	9.8	0.3	31.98
2 multi 1		H						
34	1	12/05/2020	11	3132	7	10	0.09	35.96
2 multi 1		H						
34	1	12/06/2020	12	3133	7	9.3	0.14	39.49
2 multi 1		H						
34	1	12/07/2020	13	3134	7	9.3	0.13	39.76
2 multi 1		H						
34	1	12/08/2020	14	3135	7	9.2	0.15	37.36
2 multi 1		H						
34	1	12/09/2020	15	3136	7	10.3	0.13	39.12
2 multi 1		H						
34	1	12/10/2020	16	3137	7	9.9	0.01	39.48
2 multi 1		H						
34	1	12/11/2020	17	3138	7	9.8	0.12	42.82
2 multi 1		H						
34	1	12/12/2020	18	3139	7	9.3	0	43.55
2 multi 1		H						
34	1	12/13/2020	19	3140	7	9.2	0.04	42.61
2 multi 1		H						
34	1	12/14/2020	20	3141	7	10.2	0	43.19
2 multi 1		H						
34	1	12/15/2020	21	3142	7	10	0	40.83
2 multi 1		H						
34	1	12/16/2020	22	3143	7	9.8	0	39.55
2 multi 2		H						
34	1	12/17/2020	23	3144	7	9.1	0.08	38.37
2 multi 2		H						
34	1	12/18/2020	24	3145	7	9.3	0.04	40.9
2 multi 2		H						
34	1	12/19/2020	25	3146	7	9.8	0.39	40.19
2 multi 2		H						
34	1	12/20/2020	26	3147	7	9.8	0.02	47.41
2 multi 2		H						
34	1	12/21/2020	27	3148	7	9.8	0.06	45.87
2 multi 2		H						
34	1	12/22/2020	28	3149	7	10.3	0.12	48.41

2 multi 2		H						
34	1	12/23/2020	29	3150	7	9.9	0.12	45.74
2 multi 2		H						
34	1	12/24/2020	30	3151	7	9.8	0.03	47.3
2 multi 2		H						
34	1	12/25/2020	31	3152	7	9.3	0.05	46.3
2 multi 2		H						
34	1	12/26/2020	32	3153	7	9.5	0.05	47.23
2 multi 2		H						
34	1	12/27/2020	33	3154	7	9.8	0.05	50.33
2 multi 2		H						
34	1	12/28/2020	34	3155	7	9.8	0	48.42
2 multi 2		H						
34	1	12/29/2020	35	3156	7	9.5	0.05	50.38
2 multi 2		H						
34	1	12/30/2020	36	3157	7	9.3	0.06	49.43
2 multi 2		H						
34	1	12/31/2020	37	3158	7	9.5	0.06	47.99
2 multi 2		H						
34	1	01/01/2021	38	3159	7	9.3	0	49.33
2 multi 2		H						
34	1	01/02/2021	39	3160	7	9.3	0.05	48.56
2 multi 2		H						
34	1	01/03/2021	40	3161	7	9.3	0	51.31
2 multi 2		H						
34	1	01/04/2021	41	3162	7	9.9	0.11	50.03
2 multi 2		H						
34	1	01/05/2021	42	3163	7	9.6	0.06	46.97
2 multi 2		H						
34	1	01/06/2021	43	3164	7	9.2	0.09	50.19
2 multi 2		H						
34	1	01/07/2021	44	3165	7	9.3	0.1	48.15
2 multi 2		H						
34	1	01/08/2021	45	3166	7	9.9	0.11	48.05
2 multi 2		H						
34	1	01/09/2021	46	3167	7	9.6	0.05	51.36
2 multi 2		H						
34	1	01/10/2021	47	3168	7	9.2	0.11	48.1
2 multi 2		H						
34	1	01/11/2021	48	3169	7	9.2	0.46	47.95
2 multi 2		H						
34	1	01/12/2021	49	3170	7	9.5	0.11	46.08
2 multi 2		H						
34	1	01/13/2021	50	3171	7	9.2	0.1	46.54
2 multi 2		H						
34	1	01/14/2021	51	3172	7	9.5	0.02	47.15
2 multi 2		H						
34	1	01/15/2021	52	3173	7	9.6	0.04	44.95
2 multi 2		H						
34	1	01/16/2021	53	3174	7	8.9	0	41.25
2 multi 2		H						
34	1	01/17/2021	54	3175	7	8.8	0.15	41.37
2 multi 2		H						
34	1	01/18/2021	55	3176	7	10.2	0.03	38.9

2 multi 2		H						
34	1	01/19/2021	56	3177	7	10	0.11	33.68
2 multi 2		H						
34	1	01/20/2021	57	3178	7	9.9	0.02	31.93
2 multi 2		H						
34	1	01/21/2021	58	3179	7	9.8	0	37.09
2 multi 2		H						
34	1	01/22/2021	59	3180	7	9.6	0.09	36.66
2 multi 2		H						
34	1	01/23/2021	60	3181	7	9.6	0.01	37.53
2 multi 2		H						
34	1	01/24/2021	61	3182	7	10	0.09	36.98
2 multi 2		H						
34	1	01/25/2021	62	3183	7	10.3	0.03	36.34
2 multi 2		H						
34	1	01/26/2021	63	3184	7	0	0	34.26
2 multi 2		H						
34	1	01/27/2021	64	3185	7	9.8	0.08	37.43
2 multi 2		H						
34	1	01/28/2021	65	3186	7	9.6	0.1	37.96
2 multi 2		H						
34	1	01/29/2021	66	3187	7	10.6	0	41.16
2 multi 2		H						
34	1	01/30/2021	67	3188	7	10.3	0.28	40.99
2 multi 2		H						
34	1	01/31/2021	68	3189	7	10.3	0	40.15
2 multi 2		H						
34	1	02/01/2021	69	3190	7	9.6	0.07	40.17
2 multi 2		H						
34	1	02/02/2021	70	3191	7	9.5	0.09	40.45
2 multi 2		H						
34	1	02/03/2021	71	3192	7	9.6	0	39.13
2 multi 2		H						
34	1	02/04/2021	72	3193	7	10.6	0	38.21
2 multi 2		H						
34	1	02/05/2021	73	3194	7	10.2	0.09	42.08
2 multi 2		H						
34	1	02/06/2021	74	3195	7	9.9	0.02	37.11
2 multi 2		H						
34	1	02/07/2021	75	3196	7	9.8	0	33.23
2 multi 2		H						
34	1	02/08/2021	76	3197	7	9.6	0.02	38.37
2 multi 2		H						
34	1	02/09/2021	77	3198	7	10	0.04	37.05
2 multi 2		H						
34	1	02/10/2021	78	3199	7	10.3	0.03	39.54
2 multi 2		H						
34	1	02/11/2021	79	3200	7	10	0	40.07
2 multi 2		H						
34	1	02/12/2021	80	3201	7	9.5	0.01	40.61
2 multi 2		H						
34	1	02/13/2021	81	3202	7	9.8	0.03	40.48
2 multi 2		H						
34	1	02/14/2021	82	3203	7	9.6	0.03	38.75

2 multi 2		H						
34	1	02/15/2021	83	3204	7	9.5	0.68	40.51
2 multi 2		H						
34	1	02/16/2021	84	3205	7	10.4	0.1	39
2 multi 2		H						
34	1	02/17/2021	85	3206	7	10.3	0	41.61
2 multi 2		H						
34	1	02/18/2021	86	3207	7	9.8	0	37.15
2 multi 2		H						
34	1	02/19/2021	87	3208	7	9.6	0	37.28
2 multi 2		H						
34	1	02/20/2021	88	3209	7	10.4	0.02	39.57
2 multi 2		H						
34	1	02/21/2021	89	3210	7	10.2	0.14	38.6
2 multi 2		H						
34	1	02/22/2021	90	3211	7	9.9	0	37.05
2 multi 2		H						
34	1	02/23/2021	91	3212	7	9.6	0.02	37.41
2 multi 2		H						
34	1	02/24/2021	92	3213	7	10.3	0.01	36.68
2 multi 2		H						
34	1	02/25/2021	93	3214	7	10.6	0	39.95
2 multi 2		H						
34	1	02/26/2021	94	3215	7	10	0.02	39.4
2 multi 2		H						
34	1	02/27/2021	95	3216	7	9.9	0.02	38.97
2 multi 2		H						
34	1	02/28/2021	96	3217	7	9.6	0.01	40.66
2 multi 2		H						
34	1	03/01/2021	97	3218	7	10.3	0.11	41.14
2 multi 2		H						
34	1	03/02/2021	98	3219	7	10.6	0	39.67
2 multi 2		H						
34	1	03/03/2021	99	3220	7	10.3	0.02	42.68
2 multi 2		H						
34	1	03/04/2021	100	3221	7	9.9	0.09	41.36
2 multi 2		H						
34	1	03/05/2021	101	3222	7	9.9	0.09	42.94
2 multi 2		H						
34	1	03/06/2021	102	3223	7	9.9	0.09	44.4
2 multi 2		H						
34	1	03/07/2021	103	3224	7	12.9	0.19	31.39
2 multi 2		H						
34	1	03/08/2021	104	3225	7	10.7	0	40.6
2 multi 2		H						
34	1	03/09/2021	105	3226	7	10.2	0	38.98
2 multi 2		H						
34	1	03/10/2021	106	3227	7	10.2	0	42.29
2 multi 2		H						
34	1	03/11/2021	107	3228	7	9.6	0.09	38.97
2 multi 2		H						
34	1	03/12/2021	108	3229	7	9.8	0.09	43.01
2 multi 2		H						
34	1	03/13/2021	109	3230	7	9.5	0	40.05

2 multi	2	H						
34	1	03/14/2021	110	3231	7	10.2	0.08	41.63
2 multi	2	H						
34	1	03/15/2021	111	3232	7	9.9	0.08	42.27
2 multi	2	H						
34	1	03/16/2021	112	3233	7	9.9	0	38.57
2 multi	2	H						
34	1	03/17/2021	113	3234	7	9.8	0.08	40.22
2 multi	2	H						
34	1	03/18/2021	114	3235	7	10.6	0	42.98
2 multi	2	H						
34	1	03/19/2021	115	3236	7	10.3	0.09	37.47
2 multi	2	H						
34	1	03/20/2021	116	3237	7	10.2	0	40.83
2 multi	2	H						
34	1	03/21/2021	117	3238	7	10	0.02	39.99
2 multi	2	H						
34	1	03/22/2021	118	3239	7	9.8	0.09	40.15
2 multi	2	H						
34	1	03/23/2021	119	3240	7	9.6	0	40.34
2 multi	2	H						
34	1	03/24/2021	120	3241	7	10.4	0.01	42.93
2 multi	2	H						
34	1	03/25/2021	121	3242	7	10	0	38.58
2 multi	2	H						
34	1	03/26/2021	122	3243	7	10.3	0	41.86
2 multi	2	H						
34	1	03/27/2021	123	3244	7	9.9	0.09	42.99
2 multi	2	H						
34	1	03/28/2021	124	3245	7	9.8	0.1	40.29
2 multi	2	H						
34	1	03/29/2021	125	3246	7	9.8	0.02	42.4
2 multi	2	H						
34	1	03/30/2021	126	3247	7	9.6	0.07	42.59
2 multi	2	H						
34	1	03/31/2021	127	3248	7	10.3	0.01	36.24
2 multi	2	H						
34	1	04/01/2021	128	3249	7	10.4	0.08	38.85
2 multi	2	H						
34	1	04/02/2021	129	3250	7	10	0	40.89
2 multi	2	H						
34	1	04/03/2021	130	3251	7	10.2	0.01	39.85
2 multi	2	H						
34	1	04/04/2021	131	3252	7	10	0.02	39.16
2 multi	2	H						
34	1	04/05/2021	132	3253	7	9.8	0.31	39.49
2 multi	2	H						
34	1	04/06/2021	133	3254	7	10.2	0	37.63
2 multi	2	H						
34	1	04/07/2021	134	3255	7	10.2	0	35.17
2 multi	2	H						
34	1	04/08/2021	135	3256	7	10.3	0.09	36.99
2 multi	2	H						
34	1	04/09/2021	136	3257	7	10	0.08	37.97

2 multi	2	H						
34	1	04/10/2021	137	3258	7	9.6	0.11	35.3
2 multi	2	H						
34	1	04/11/2021	138	3259	7	10.7	0	33.95
2 multi	2	H						
34	1	04/12/2021	139	3260	7	10.7	0	33.07
2 multi	2	H						
34	1	04/13/2021	140	3261	7	10.4	0.42	32.95
2 multi	2	H						
34	1	04/14/2021	141	3262	7	10.3	0.02	34.51
2 multi	2	H						
34	1	04/15/2021	142	3263	7	10.2	0.08	33.91
2 multi	2	H						
35	1	03/26/2020	4	874	1	9.2	0.25	19.11
1 primi	1	H						
35	1	03/27/2020	5	875	1	9.2	0.27	21.75
1 primi	1	H						
35	1	03/28/2020	6	876	1	8.8	0.15	22.6
1 primi	1	H						
35	1	03/29/2020	7	877	1	8.6	0.28	22.44
1 primi	1	H						
35	1	03/30/2020	8	878	1	8.9	0	22.4
1 primi	1	H						
35	1	03/31/2020	9	879	1	8.8	0	24.45
1 primi	1	H						
35	1	04/01/2020	10	880	1	8.8	0.04	25.6
1 primi	1	H						
35	1	04/02/2020	11	881	1	8.7	0	25.74
1 primi	1	H						
35	1	04/03/2020	12	882	1	8.8	0.04	25.83
1 primi	1	H						
35	1	04/04/2020	13	883	1	9.1	0.03	22.35
1 primi	1	H						
35	1	04/05/2020	14	884	1	9.1	0	28.97
1 primi	1	H						
35	1	04/06/2020	15	885	1	8.9	0.24	26.57
1 primi	1	H						
35	1	04/07/2020	16	886	1	9.5	0.01	28.24
1 primi	1	H						
35	1	04/08/2020	17	887	1	9.3	0	30.27
1 primi	1	H						
35	1	04/09/2020	18	888	1	9.5	0	27.59
1 primi	1	H						
35	1	04/10/2020	19	889	1	9.3	0.02	31.42
1 primi	1	H						
35	1	04/11/2020	20	890	1	9.1	0.23	29.96
1 primi	1	H						
35	1	04/12/2020	21	891	1	9.2	0.19	29.77
1 primi	1	H						
35	1	04/13/2020	22	892	1	8.9	0.25	30.76
1 primi	2	H						
35	1	04/14/2020	23	893	1	8.9	0.2	32.45
1 primi	2	H						
35	1	04/15/2020	24	894	1	8.8	0.24	30.08

1 primi 2		H						
35	1	04/16/2020	25	895	1	8.8	0.27	29.88
1 primi 2		H						
35	1	04/17/2020	26	896	1	8.4	0.31	27.45
1 primi 2		H						
35	1	04/18/2020	27	897	1	8.5	0.3	31.33
1 primi 2		H						
35	1	04/19/2020	28	898	1	8.2	0.33	28.21
1 primi 2		H						
35	1	04/20/2020	29	899	1	8.3	0.27	29.56
1 primi 2		H						
35	1	04/21/2020	30	900	1	8.5	0.23	29.21
1 primi 2		H						
35	1	04/22/2020	31	901	1	8.8	0.28	30.46
1 primi 2		H						
35	1	04/23/2020	32	902	1	9.2	0.32	30.13
1 primi 2		H						
35	1	04/24/2020	33	903	1	9.2	0.28	30.01
1 primi 2		H						
35	1	04/25/2020	34	904	1	9.3	0.32	30.87
1 primi 2		H						
35	1	04/26/2020	35	905	1	9.2	0.28	30.8
1 primi 2		H						
35	1	04/27/2020	36	906	1	9.3	0.21	32.45
1 primi 2		H						
35	1	04/28/2020	37	907	1	9.5	0.27	31.67
1 primi 2		H						
35	1	04/29/2020	38	908	1	10.3	0.05	32.02
1 primi 2		H						
35	1	04/30/2020	39	909	1	9.8	0.05	30.72
1 primi 2		H						
35	1	05/01/2020	40	910	1	9.5	0	34.68
1 primi 2		H						
35	1	05/02/2020	41	911	1	9.2	0.32	32.52
1 primi 2		H						
35	1	05/03/2020	42	912	1	9.1	0.28	34.15
1 primi 2		H						
35	1	05/04/2020	43	913	1	9.1	0.17	32.44
1 primi 2		H						
35	1	05/05/2020	44	914	1	9.2	0.31	34.52
1 primi 2		H						
35	1	05/06/2020	45	915	1	9.1	0.44	30.9
1 primi 2		H						
35	1	05/07/2020	46	916	1	9.1	0.25	32.65
1 primi 2		H						
35	1	05/08/2020	47	917	1	9.2	0.28	30.49
1 primi 2		H						
35	1	05/09/2020	48	918	1	9.3	0.44	32.2
1 primi 2		H						
35	1	05/10/2020	49	919	1	9.1	0.17	32.29
1 primi 2		H						
35	1	05/11/2020	50	920	1	9.6	0.15	33.2
1 primi 2		H						
35	1	05/12/2020	51	921	1	9.1	0.28	31.05

1 primi	2	H						
35	1	05/13/2020	52	922	1	9.5	0.21	34.07
1 primi	2	H						
36	1	03/16/2019	5	692	1	9.2	0.36	19.6
1 primi	1	H						
36	1	03/17/2019	6	693	1	10	0.1	21.59
1 primi	1	H						
36	1	03/18/2019	7	694	1	9.6	0	19.26
1 primi	1	H						
36	1	03/19/2019	8	695	1	9.2	0.1	21.59
1 primi	1	H						
36	1	03/20/2019	9	696	1	9.3	0	23.01
1 primi	1	H						
36	1	03/21/2019	10	697	1	9.3	0.09	23.74
1 primi	1	H						
36	1	03/22/2019	11	698	1	10.2	0	22.64
1 primi	1	H						
36	1	03/23/2019	12	699	1	9.8	0	24.26
1 primi	1	H						
36	1	03/24/2019	13	700	1	9.5	0	24.3
1 primi	1	H						
36	1	03/25/2019	14	701	1	9.2	0	24.4
1 primi	1	H						
36	1	03/26/2019	15	702	1	9.2	0.08	24.39
1 primi	1	H						
36	1	03/27/2019	16	703	1	9.5	0.08	24.32
1 primi	1	H						
36	1	03/28/2019	17	704	1	9.2	0.08	27.69
1 primi	1	H						
36	1	03/29/2019	18	705	1	9.2	0	25.72
1 primi	1	H						
36	1	03/30/2019	19	706	1	9.1	0	28.04
1 primi	1	H						
36	1	03/31/2019	20	707	1	9.5	0	27.94
1 primi	1	H						
36	1	04/01/2019	21	708	1	9.2	0	29.91
1 primi	1	H						
36	1	04/02/2019	22	709	1	8.9	0.23	30.1
1 primi	2	H						
36	1	04/03/2019	23	710	1	8.9	0.07	30.05
1 primi	2	H						
36	1	04/04/2019	24	711	1	9.2	0.07	28.09
1 primi	2	H						
36	1	04/05/2019	25	712	1	8.9	0	28.43
1 primi	2	H						
36	1	04/06/2019	26	713	1	9.1	0	29.21
1 primi	2	H						
36	1	04/07/2019	27	714	1	8.9	0.1	27.11
1 primi	2	H						
36	1	04/08/2019	28	715	1	9.1	0	29.91
1 primi	2	H						
36	1	04/09/2019	29	716	1	9.1	0.05	29.65
1 primi	2	H						
36	1	04/10/2019	30	717	1	8.9	0.15	30.03

1 primi 2		H						
36	1	04/11/2019	31	718	1	9.2	0	28.31
1 primi 2		H						
36	1	04/12/2019	32	719	1	9.1	0.05	30.47
1 primi 2		H						
36	1	04/13/2019	33	720	1	8.7	0	28.13
1 primi 2		H						
36	1	04/14/2019	34	721	1	8.9	0.05	32.45
1 primi 2		H						
36	1	04/15/2019	35	722	1	8.8	0.05	30.46
1 primi 2		H						
36	1	04/16/2019	36	723	1	8.9	0	33.46
1 primi 2		H						
36	1	04/17/2019	37	724	1	8.7	0	29.97
1 primi 2		H						
36	1	04/18/2019	38	725	1	8.9	0	32.62
1 primi 2		H						
36	1	04/19/2019	39	726	1	9.1	0.06	32.97
1 primi 2		H						
36	1	04/20/2019	40	727	1	8.8	0	34.82
1 primi 2		H						
36	1	04/21/2019	41	728	1	9.1	0.07	34.17
1 primi 2		H						
36	1	04/22/2019	42	729	1	8.9	0	35.18
1 primi 2		H						
36	1	04/23/2019	43	730	1	8.9	0.2	33.64
1 primi 2		H						
36	1	04/24/2019	44	731	1	8.9	0	32.46
1 primi 2		H						
36	1	04/25/2019	45	732	1	9.1	0.23	34.63
1 primi 2		H						
36	1	04/26/2019	46	733	1	9.3	0	35.84
1 primi 2		H						
36	1	04/27/2019	47	734	1	9.1	0	35.18
1 primi 2		H						
36	1	04/28/2019	48	735	1	8.9	0	31.38
1 primi 2		H						
36	1	04/29/2019	49	736	1	8.8	0.28	34.05
1 primi 2		H						
36	1	04/30/2019	50	737	1	8.8	0	30.4
1 primi 2		H						
36	1	05/01/2019	51	738	1	8.8	0	33.95
1 primi 2		H						
36	1	05/02/2019	52	739	1	9.1	0.06	31.77
1 primi 2		H						
36	1	05/03/2019	53	740	1	9.2	0	36.14
1 primi 2		H						
36	1	05/04/2019	54	741	1	9.2	0	35.95
1 primi 2		H						
36	1	05/05/2019	55	742	1	9.2	0	33.36
1 primi 2		H						
36	1	05/06/2019	56	743	1	9.2	0.25	32.67
1 primi 2		H						
36	1	05/07/2019	57	744	1	9.1	0	35.88

1 primi	2	H						
36	1	05/08/2019	58	745	1	9.1	0.21	33.98
1 primi	2	H						
36	1	05/09/2019	59	746	1	9.2	0	36.42
1 primi	2	H						
36	1	05/10/2019	60	747	1	9.2	0.21	34.81
1 primi	2	H						
36	1	05/11/2019	61	748	1	8.7	0.08	32.62
1 primi	2	H						
36	1	05/12/2019	62	749	1	8.8	0	29.05
1 primi	2	H						
36	1	05/13/2019	63	750	1	8.9	0	31.54
1 primi	2	H						
36	1	05/14/2019	64	751	1	8.8	0.06	31.92
1 primi	2	H						
36	1	05/15/2019	65	752	1	8.8	0	29.38
1 primi	2	H						
36	1	05/16/2019	66	753	1	8.9	0	29.31
1 primi	2	H						
36	1	05/17/2019	67	754	1	9.1	0.05	32.47
1 primi	2	H						
36	1	05/18/2019	68	755	1	8.9	0	30.33
1 primi	2	H						
36	1	05/19/2019	69	756	1	9.1	0	33.4
1 primi	2	H						
36	1	05/20/2019	70	757	1	9.1	0.05	33.39
1 primi	2	H						
36	1	05/21/2019	71	758	1	9.1	0	33.98
1 primi	2	H						
36	1	05/22/2019	72	759	1	9.2	0	35.68
1 primi	2	H						
36	1	05/23/2019	73	760	1	8.9	0	30.78
1 primi	2	H						
36	1	05/24/2019	74	761	1	8.9	0	31.49
1 primi	2	H						
36	1	05/25/2019	75	762	1	8.9	0	30.84
1 primi	2	H						
36	1	05/26/2019	76	763	1	8.9	0	34.36
1 primi	2	H						
36	1	05/27/2019	77	764	1	9.2	0.05	32.82
1 primi	2	H						
36	1	05/28/2019	78	765	1	9.1	0	32.2
1 primi	2	H						
36	1	05/29/2019	79	766	1	9.2	0.01	33.38
1 primi	2	H						
36	1	05/30/2019	80	767	1	9.1	0	33.3
1 primi	2	H						
36	1	05/31/2019	81	768	1	9.2	0.18	34.95
1 primi	2	H						
36	1	06/01/2019	82	769	1	9.2	0.23	36.69
1 primi	2	H						
36	1	06/02/2019	83	770	1	9.3	0.27	34.81
1 primi	2	H						
36	1	06/03/2019	84	771	1	9.1	0.26	35.22

1 primi 2		H						
36	1	06/04/2019	85	772	1	9.1	0.13	35.34
1 primi 2		H						
36	1	06/05/2019	86	773	1	9.2	0.13	34.78
1 primi 2		H						
36	1	06/06/2019	87	774	1	9.3	0.24	33.52
1 primi 2		H						
36	1	06/07/2019	88	775	1	9.6	0.26	32.9
1 primi 2		H						
36	1	06/08/2019	89	776	1	9.2	0.28	35.75
1 primi 2		H						
36	1	06/09/2019	90	777	1	9.3	0.18	36.48
1 primi 2		H						
36	1	06/10/2019	91	778	1	9.3	0.12	36.08
1 primi 2		H						
36	1	06/11/2019	92	779	1	9.5	0.28	36.9
1 primi 2		H						
36	1	06/12/2019	93	780	1	9.9	0.17	37.96
1 primi 2		H						
36	1	06/13/2019	94	781	1	9.5	0.09	35.3
1 primi 2		H						
36	1	06/14/2019	95	782	1	9.5	0.32	36.54
1 primi 2		H						
36	1	06/15/2019	96	783	1	9.8	0.16	36.45
1 primi 2		H						
36	1	06/16/2019	97	784	1	9.5	0.25	34.41
1 primi 2		H						
36	1	06/17/2019	98	785	1	9.5	0.26	34.99
1 primi 2		H						
36	1	06/18/2019	99	786	1	9.3	0.22	36.33
1 primi 2		H						
36	1	06/19/2019	100	787	1	9.6	0	35.84
1 primi 2		H						
36	1	06/20/2019	101	788	1	9.6	0.04	34.98
1 primi 2		H						
36	1	06/21/2019	102	789	1	9.6	0.12	34.09
1 primi 2		H						
36	1	06/22/2019	103	790	1	9.5	0	37.5
1 primi 2		H						
36	1	06/23/2019	104	791	1	10.7	0	34.12
1 primi 2		H						
36	1	06/24/2019	105	792	1	9.5	0.1	36.07
1 primi 2		H						
36	1	06/25/2019	106	793	1	9.5	0.36	38.36
1 primi 2		H						
36	1	06/26/2019	107	794	1	9.6	0.12	37.06
1 primi 2		H						
36	1	06/27/2019	108	795	1	9.6	0.3	31.48
1 primi 2		H						
36	1	06/28/2019	109	796	1	9.2	0.13	33.3
1 primi 2		H						
36	1	06/29/2019	110	797	1	9.6	0.04	32.78
1 primi 2		H						
36	1	06/30/2019	111	798	1	9.5	0.13	34.74

1 primi 2		H						
36	1	07/01/2019	112	799	1	9.5	0.21	35.61
1 primi 2		H						
36	1	07/02/2019	113	800	1	9.2	0.04	34.31
1 primi 2		H						
36	1	07/03/2019	114	801	1	9.6	0	37.13
1 primi 2		H						
36	1	07/04/2019	115	802	1	9.5	0	33.55
1 primi 2		H						
36	1	07/05/2019	116	803	1	9.6	0	36.04
1 primi 2		H						
36	1	07/06/2019	117	804	1	9.1	0.11	35.22
1 primi 2		H						
36	1	07/07/2019	118	805	1	9.8	0.01	33.07
1 primi 2		H						
36	1	07/08/2019	119	806	1	9.2	0	28.52
1 primi 2		H						
36	1	07/09/2019	120	807	1	9.2	0	33.15
1 primi 2		H						
36	1	07/10/2019	121	808	1	9.1	0	30.83
1 primi 2		H						
36	1	07/11/2019	122	809	1	9.3	0.19	31.02
1 primi 2		H						
36	1	07/12/2019	123	810	1	9.3	0	31
1 primi 2		H						
36	1	07/13/2019	124	811	1	9.3	0	33.05
1 primi 2		H						
36	1	07/14/2019	125	812	1	9.6	0	36.66
1 primi 2		H						
36	1	07/15/2019	126	813	1	9.6	0.09	35.85
1 primi 2		H						
36	1	07/16/2019	127	814	1	9.5	0.05	39.07
1 primi 2		H						
36	1	07/17/2019	128	815	1	9.5	0	37.55
1 primi 2		H						
36	1	07/18/2019	129	816	1	9.3	0.1	39.2
1 primi 2		H						
36	1	07/19/2019	130	817	1	9.3	0	36.42
1 primi 2		H						
36	1	07/20/2019	131	818	1	9.5	0.09	39.91
1 primi 2		H						
36	1	07/21/2019	132	819	1	9.5	0	38.33
1 primi 2		H						
36	1	07/22/2019	133	820	1	9.5	0.13	35.82
1 primi 2		H						
36	1	07/23/2019	134	821	1	9.8	0.15	33.95
1 primi 2		H						
36	1	07/24/2019	135	822	1	9.3	0	38.18
1 primi 2		H						
36	1	07/25/2019	136	823	1	9.3	0	36.75
1 primi 2		H						
36	1	07/26/2019	137	824	1	9.3	0.2	34.5
1 primi 2		H						
36	1	07/27/2019	138	825	1	9.8	0	36.4

1 primi	2	H						
36	1	07/28/2019	139	826	1	9.5	0.13	37.16
1 primi	2	H						
36	1	07/29/2019	140	827	1	9.5	0.12	36.63
1 primi	2	H						
36	1	07/30/2019	141	828	1	9.5	0	36.95
1 primi	2	H						
36	1	07/31/2019	142	829	1	10.6	0	37.43
1 primi	2	H						
36	1	08/01/2019	143	830	1	9.8	0	34.38
1 primi	2	H						
36	1	08/02/2019	144	831	1	9.5	0	30.8
1 primi	2	H						
36	1	08/03/2019	145	832	1	9.3	0	26.78
1 primi	2	H						
36	1	08/04/2019	146	833	1	9.1	0	27.85
1 primi	2	H						
36	1	08/05/2019	147	834	1	11.3	0.06	29.94
1 primi	2	H						
36	1	08/06/2019	148	835	1	9.8	0	31.7
1 primi	2	H						
36	1	08/07/2019	149	836	1	9.9	0	31.79
1 primi	2	H						
36	1	08/08/2019	150	837	1	9.9	0.08	35.02
1 primi	2	H						
36	1	08/09/2019	151	838	1	9.6	0.1	34.56
1 primi	2	H						
36	1	08/10/2019	152	839	1	9.3	0.06	34.82
1 primi	2	H						
36	1	08/11/2019	153	840	1	9.2	0.18	34.22
1 primi	2	H						
36	1	05/19/2020	6	1122	2	9.8	0.31	32.46
2 multi	1	H						
36	1	05/20/2020	7	1123	2	9.5	0.28	34.39
2 multi	1	H						
36	1	05/21/2020	8	1124	2	10	0.14	34.24
2 multi	1	H						
36	1	05/22/2020	9	1125	2	9.2	0.2	35.04
2 multi	1	H						
36	1	05/23/2020	10	1126	2	9.9	0.09	38.85
2 multi	1	H						
36	1	05/24/2020	11	1127	2	9.9	0	35.11
2 multi	1	H						
36	1	05/25/2020	12	1128	2	9.8	0.27	37.42
2 multi	1	H						
36	1	05/26/2020	13	1129	2	9.6	0.09	38.98
2 multi	1	H						
36	1	05/27/2020	14	1130	2	9.8	0.02	41.26
2 multi	1	H						
36	1	05/28/2020	15	1131	2	9.9	0	40.04
2 multi	1	H						
36	1	05/29/2020	16	1132	2	9.8	0.01	46.01
2 multi	1	H						
36	1	05/30/2020	17	1133	2	9.5	0.09	40.81

2 multi 1 H
36 1 05/31/2020 18 1134 2 9.6 0 41.3
2 multi 1 H
36 1 06/01/2020 19 1135 2 9.5 0 44.06
2 multi 1 H
36 1 06/02/2020 20 1136 2 9.6 0.26 43.22
2 multi 1 H
36 1 06/03/2020 21 1137 2 9.8 0 47.36
2 multi 1 H
36 1 06/04/2020 22 1138 2 9.8 0.26 44.64
2 multi 2 H
36 1 06/05/2020 23 1139 2 9.5 0.16 43.8
2 multi 2 H
36 1 06/06/2020 24 1140 2 9.6 0 49.03
2 multi 2 H
36 1 06/07/2020 25 1141 2 9.8 0.06 44.27
2 multi 2 H
36 1 06/08/2020 26 1142 2 9.9 0 48.22
2 multi 2 H
36 1 06/09/2020 27 1143 2 9.8 0 49.9
2 multi 2 H
36 1 06/10/2020 28 1144 2 9.9 0 47.39
2 multi 2 H
36 1 06/11/2020 29 1145 2 9.9 0.07 45.37
2 multi 2 H
36 1 06/12/2020 30 1146 2 9.6 0 47.96
2 multi 2 H
36 1 06/13/2020 31 1147 2 9.5 0.07 46.69
2 multi 2 H
36 1 06/14/2020 32 1148 2 9.6 0.06 46.13
2 multi 2 H
36 1 06/15/2020 33 1149 2 9.5 0.01 48.14
2 multi 2 H
36 1 06/16/2020 34 1150 2 9.5 0 49.48
2 multi 2 H
36 1 06/17/2020 35 1151 2 9.6 0.2 44.12
2 multi 2 H
36 1 06/18/2020 36 1152 2 9.6 0.23 50.6
2 multi 2 H
36 1 06/19/2020 37 1153 2 9.3 0.14 47
2 multi 2 H
36 1 06/20/2020 38 1154 2 9.5 0 44.39
2 multi 2 H
36 1 06/21/2020 39 1155 2 9.6 0.23 47.42
2 multi 2 H
36 1 06/22/2020 40 1156 2 9.6 0.14 48.75
2 multi 2 H
36 1 06/23/2020 41 1157 2 9.6 0.18 48.61
2 multi 2 H
36 1 06/24/2020 42 1158 2 10.4 0.18 47.6
2 multi 2 H
36 1 06/25/2020 43 1159 2 9.6 0 47.21
2 multi 2 H
36 1 06/26/2020 44 1160 2 9.5 0 50.61

2 multi 2		H						
36	1	06/27/2020	45	1161	2	9.6	0	52.87
2 multi 2		H						
36	1	06/28/2020	46	1162	2	9.6	0	50.37
2 multi 2		H						
36	1	06/29/2020	47	1163	2	9.3	0	45.06
2 multi 2		H						
36	1	10/14/2020	154	1270	2	9.3	0.08	37.29
2 multi 2		H						
36	1	10/15/2020	155	1271	2	10.6	0.05	38.27
2 multi 2		H						
36	1	10/16/2020	156	1272	2	10.4	0.07	35.78
2 multi 2		H						
36	1	10/17/2020	157	1273	2	10.6	0.09	35.94
2 multi 2		H						
36	1	10/18/2020	158	1274	2	10.6	0.02	38.14
2 multi 2		H						
36	1	10/19/2020	159	1275	2	10	0.11	42.64
2 multi 2		H						
36	1	10/20/2020	160	1276	2	10.2	0.44	40.18
2 multi 2		H						
36	1	10/21/2020	161	1277	2	10.4	0.06	42.52
2 multi 2		H						
36	1	10/22/2020	162	1278	2	10.2	0.07	40.98
2 multi 2		H						
36	1	10/23/2020	163	1279	2	10	0.27	42.19
2 multi 2		H						
36	1	10/24/2020	164	1280	2	9.9	0.07	38.4
2 multi 2		H						
36	1	10/25/2020	165	1281	2	10	0.06	40.68
2 multi 2		H						
36	1	10/26/2020	166	1282	2	10.4	0.53	39.03
2 multi 2		H						
36	1	10/27/2020	167	1283	2	10.2	0.24	39.8
2 multi 2		H						
36	1	10/28/2020	168	1284	2	10.2	0.31	37.41
2 multi 2		H						
36	1	10/29/2020	169	1285	2	10.3	0	38.89
2 multi 2		H						
36	1	10/30/2020	170	1286	2	9.9	0	37.38
2 multi 2		H						
36	1	10/31/2020	171	1287	2	10.3	0	40.66
2 multi 2		H						
36	1	11/01/2020	172	1288	2	10	0.05	42.07
2 multi 2		H						
36	1	11/02/2020	173	1289	2	10	0.06	40.87
2 multi 2		H						
36	1	11/03/2020	174	1290	2	10.2	0	39.24
2 multi 2		H						
36	1	11/04/2020	175	1291	2	10	0.06	37.7
2 multi 2		H						
36	1	11/05/2020	176	1292	2	9.9	0.16	39.57
2 multi 2		H						
36	1	11/06/2020	177	1293	2	9.9	0.06	39.03

2 multi	2	H						
36	1	11/07/2020	178	1294	2	9.2	0	36.42
2 multi	2	H						
36	1	11/08/2020	179	1295	2	10.3	0.09	38.99
2 multi	2	H						
36	1	11/09/2020	180	1296	2	10.3	0.01	39.21
2 multi	2	H						
36	1	11/10/2020	181	1297	2	10.3	0.79	39.39
2 multi	2	H						
36	1	11/11/2020	182	1298	2	10.2	0.06	40.96
2 multi	2	H						
36	1	11/12/2020	183	1299	2	10.3	0.28	39.73
2 multi	2	H						
36	1	11/13/2020	184	1300	2	10.3	0.07	38.94
2 multi	2	H						
36	1	11/14/2020	185	1301	2	10	0.05	40.31
2 multi	2	H						
36	1	11/15/2020	186	1302	2	10	0.08	39.26
2 multi	2	H						
36	1	11/16/2020	187	1303	2	9.9	0.06	42.12
2 multi	2	H						
36	1	11/17/2020	188	1304	2	9.5	0.09	40.36
2 multi	2	H						
36	1	11/18/2020	189	1305	2	10.2	0.1	41.54
2 multi	2	H						
36	1	11/19/2020	190	1306	2	9.9	0.19	38.86
2 multi	2	H						
36	1	11/20/2020	191	1307	2	10.2	0.23	41.41
2 multi	2	H						
36	1	11/21/2020	192	1308	2	9.3	0.13	36.97
2 multi	2	H						
36	1	11/22/2020	193	1309	2	9.8	0.05	39.1
2 multi	2	H						
36	1	11/23/2020	194	1310	2	9.1	0.06	43.03
2 multi	2	H						
36	1	11/24/2020	195	1311	2	9.8	0.14	37.42
2 multi	2	H						
39	1	04/16/2020	8	708	1	8.9	0.4	16.67
1 primi	1	C						
39	1	04/17/2020	9	709	1	9.2	0.05	20.86
1 primi	1	C						
39	1	04/18/2020	10	710	1	8.8	0.19	20.39
1 primi	1	C						
39	1	04/19/2020	11	711	1	9.1	0.06	21.77
1 primi	1	C						
39	1	04/20/2020	12	712	1	9.1	0.19	21.65
1 primi	1	C						
39	1	04/21/2020	13	713	1	10.7	0.05	23.25
1 primi	1	C						
39	1	04/22/2020	14	714	1	9.1	0.06	24.49
1 primi	1	C						
39	1	04/23/2020	15	715	1	8.9	0.06	21.92
1 primi	1	C						
39	1	04/24/2020	16	716	1	9.3	0.08	24.29

1 primi 1 C
39 1 04/25/2020 17 717 1 8.9 0.13 24.7
1 primi 1 C
39 1 04/26/2020 18 718 1 8.9 0.05 25.78
1 primi 1 C
39 1 04/27/2020 19 719 1 9.1 0.17 24.42
1 primi 1 C
39 1 04/28/2020 20 720 1 8.8 0.16 24.96
1 primi 1 C
39 1 04/29/2020 21 721 1 8.8 0.08 26.3
1 primi 1 C
39 1 04/30/2020 22 722 1 9.1 0.15 25.33
1 primi 2 C
39 1 05/01/2020 23 723 1 9.2 0.08 24.99
1 primi 2 C
39 1 05/02/2020 24 724 1 9.5 0 25.46
1 primi 2 C
39 1 05/03/2020 25 725 1 9.3 0.06 26.97
1 primi 2 C
39 1 05/04/2020 26 726 1 8.9 0.26 26.5
1 primi 2 C
39 1 05/05/2020 27 727 1 9.1 0.09 26.17
1 primi 2 C
39 1 05/06/2020 28 728 1 8.7 0.14 25.24
1 primi 2 C
39 1 05/07/2020 29 729 1 9.1 0 26.59
1 primi 2 C
39 1 05/08/2020 30 730 1 9.1 0.06 25.86
1 primi 2 C
39 1 05/09/2020 31 731 1 8.7 0 25.76
1 primi 2 C
39 1 05/10/2020 32 732 1 8.9 0.07 26.41
1 primi 2 C
39 1 05/11/2020 33 733 1 8.6 0.04 25.2
1 primi 2 C
39 1 05/12/2020 34 734 1 8.7 0 25.55
1 primi 2 C
39 1 05/13/2020 35 735 1 8.8 0 27.38
1 primi 2 C
39 1 05/14/2020 36 736 1 8.8 0 26.19
1 primi 2 C
39 1 05/15/2020 37 737 1 8.7 0.12 26.97
1 primi 2 C
39 1 05/16/2020 38 738 1 8.8 0 29.67
1 primi 2 C
39 1 05/17/2020 39 739 1 8.7 0 27.12
1 primi 2 C
39 1 05/18/2020 40 740 1 8.7 0 28.29
1 primi 2 C
39 1 05/19/2020 41 741 1 8.7 0.14 28.39
1 primi 2 C
39 1 05/20/2020 42 742 1 8.3 0.08 23.3
1 primi 2 C
39 1 05/21/2020 43 743 1 8.5 0.12 26.46

1 primi	2	C						
39	1	05/22/2020	44	744	1	8.7	0.11	28.26
1 primi	2	C						
39	1	05/23/2020	45	745	1	8.4	0	26.14
1 primi	2	C						
39	1	05/24/2020	46	746	1	8.4	0.11	27.2
1 primi	2	C						
39	1	05/25/2020	47	747	1	8.8	0.02	27.7
1 primi	2	C						
39	1	05/26/2020	48	748	1	8.7	0.11	25.45
1 primi	2	C						
39	1	05/27/2020	49	749	1	8.5	0.11	25.87
1 primi	2	C						
39	1	05/28/2020	50	750	1	9.1	0.03	29.83
1 primi	2	C						
39	1	05/29/2020	51	751	1	9.1	0	26.57
1 primi	2	C						
39	1	05/30/2020	52	752	1	8.7	0	27.75
1 primi	2	C						
39	1	05/31/2020	53	753	1	8.8	0	27.86
1 primi	2	C						
39	1	06/01/2020	54	754	1	9.1	0	28.3
1 primi	2	C						
39	1	06/02/2020	55	755	1	8.8	0	28.38
1 primi	2	C						
39	1	06/03/2020	56	756	1	8.8	0.09	27.77
1 primi	2	C						
39	1	06/04/2020	57	757	1	9.2	0	26.37
1 primi	2	C						
39	1	06/05/2020	58	758	1	8.5	0.01	29.51
1 primi	2	C						
39	1	06/06/2020	59	759	1	8.7	0.02	27.81
1 primi	2	C						
39	1	06/07/2020	60	760	1	8.8	0.07	30.21
1 primi	2	C						
39	1	06/08/2020	61	761	1	8.4	0.08	27.27
1 primi	2	C						
39	1	06/09/2020	62	762	1	8.6	0.09	30.08
1 primi	2	C						
39	1	06/10/2020	63	763	1	8.6	0	27.89
1 primi	2	C						
39	1	06/11/2020	64	764	1	8.6	0.13	27.67
1 primi	2	C						
39	1	06/12/2020	65	765	1	8.6	0.14	28.01
1 primi	2	C						
39	1	06/13/2020	66	766	1	8.8	0.08	28.51
1 primi	2	C						
39	1	06/14/2020	67	767	1	8.6	0.2	27.15
1 primi	2	C						
39	1	06/15/2020	68	768	1	8.7	0.06	28.49
1 primi	2	C						
39	1	06/16/2020	69	769	1	8.6	0.24	26.56
1 primi	2	C						
40	1	02/22/2021	9	753	1	8.7	0.35	22.33

1 primi 1		H						
40	1	02/23/2021	10	754	1	9.1	0.04	23.61
1 primi 1		H						
40	1	02/24/2021	11	755	1	8.9	0.34	24.09
1 primi 1		H						
40	1	02/25/2021	12	756	1	8.9	0.04	26.09
1 primi 1		H						
40	1	02/26/2021	13	757	1	9.1	0.11	27.06
1 primi 1		H						
40	1	02/27/2021	14	758	1	8.9	0.05	26.68
1 primi 1		H						
40	1	02/28/2021	15	759	1	8.7	0.05	27.68
1 primi 1		H						
40	1	03/01/2021	16	760	1	9.1	0.04	28.14
1 primi 1		H						
40	1	03/02/2021	17	761	1	8.6	0.67	28.58
1 primi 1		H						
40	1	03/03/2021	18	762	1	8.3	0	27.83
1 primi 1		H						
40	1	03/04/2021	19	763	1	8.7	0.12	29.56
1 primi 1		H						
40	1	03/05/2021	20	764	1	8.9	0	31.12
1 primi 1		H						
40	1	03/06/2021	21	765	1	8.7	0.05	31.21
1 primi 1		H						
40	1	03/07/2021	22	766	1	8.7	0	30.33
1 primi 2		H						
40	1	03/08/2021	23	767	1	8.5	0.02	29.85
1 primi 2		H						
40	1	03/09/2021	24	768	1	8.5	0.04	28.97
1 primi 2		H						
40	1	03/10/2021	25	769	1	8.6	0	30.91
1 primi 2		H						
40	1	03/11/2021	26	770	1	8.7	0.03	31.52
1 primi 2		H						
40	1	03/12/2021	27	771	1	8.9	0.48	32.76
1 primi 2		H						
40	1	03/13/2021	28	772	1	8.9	0.01	33.17
1 primi 2		H						
40	1	03/14/2021	29	773	1	8.8	0	32.62
1 primi 2		H						
40	1	03/15/2021	30	774	1	8.9	0	31.99
1 primi 2		H						
40	1	03/16/2021	31	775	1	10.3	0.09	32.55
1 primi 2		H						
40	1	03/17/2021	32	776	1	8.8	0	31.76
1 primi 2		H						
40	1	03/18/2021	33	777	1	8.6	0	33.5
1 primi 2		H						
40	1	03/19/2021	34	778	1	8.8	0.51	30.83
1 primi 2		H						
40	1	03/20/2021	35	779	1	8.6	0.09	31.18
1 primi 2		H						
40	1	03/21/2021	36	780	1	8.7	0.07	32.94

1 primi	2	H						
40	1	03/22/2021	37	781	1	8.7	0.07	33.74
1 primi	2	H						
40	1	03/23/2021	38	782	1	8.7	0.07	33.01
1 primi	2	H						
40	1	03/24/2021	39	783	1	8.8	0.22	29.67
1 primi	2	H						
40	1	03/25/2021	40	784	1	8.7	0	31.32
1 primi	2	H						
40	1	03/26/2021	41	785	1	9.1	0.04	32.37
1 primi	2	H						
40	1	03/27/2021	42	786	1	9.2	0.36	32.08
1 primi	2	H						
40	1	03/28/2021	43	787	1	9.3	0.4	32.44
1 primi	2	H						
40	1	03/29/2021	44	788	1	9.1	0	33.21
1 primi	2	H						
40	1	03/30/2021	45	789	1	8.9	0.09	34.65
1 primi	2	H						
40	1	03/31/2021	46	790	1	8.9	0	32.23
1 primi	2	H						
40	1	04/01/2021	47	791	1	8.9	0.17	33.55
1 primi	2	H						
40	1	04/02/2021	48	792	1	8.8	0.02	28.85
1 primi	2	H						
40	1	04/03/2021	49	793	1	8.8	0	33.75
1 primi	2	H						
40	1	04/04/2021	50	794	1	8.9	0	32.84
1 primi	2	H						
40	1	04/05/2021	51	795	1	8.9	0.07	32.49
1 primi	2	H						
40	1	04/06/2021	52	796	1	8.7	0.34	31.27
1 primi	2	H						
40	1	04/07/2021	53	797	1	8.7	0.06	32.07
1 primi	2	H						
40	1	04/08/2021	54	798	1	8.8	0.06	33.37
1 primi	2	H						
40	1	04/09/2021	55	799	1	8.6	0.11	32.31
1 primi	2	H						
40	1	04/10/2021	56	800	1	8.6	0.12	32.55
1 primi	2	H						
40	1	04/11/2021	57	801	1	8.7	0.13	30.46
1 primi	2	H						
40	1	04/12/2021	58	802	1	8.7	0.31	33.52
1 primi	2	H						
40	1	04/13/2021	59	803	1	8.7	0.25	33.07
1 primi	2	H						
40	1	04/14/2021	60	804	1	8.9	0.04	32.55
1 primi	2	H						
40	1	04/15/2021	61	805	1	9.1	0	31.03
1 primi	2	H						
40	1	04/16/2021	62	806	1	9.2	0.08	34.46
1 primi	2	H						
40	1	04/17/2021	63	807	1	8.9	0	33.04

1 primi 2 H
40 1 04/18/2021 64 808 1 8.7 0 34.08
1 primi 2 H
40 1 04/19/2021 65 809 1 8.9 0.08 34.35
1 primi 2 H
40 1 04/20/2021 66 810 1 8.9 0.07 34.29
1 primi 2 H
40 1 04/21/2021 67 811 1 9.1 0 34.23
1 primi 2 H
40 1 04/22/2021 68 812 1 8.9 0.5 33.7
1 primi 2 H
40 1 04/23/2021 69 813 1 9.1 0 34.66
1 primi 2 H
40 1 04/24/2021 70 814 1 8.9 0.09 32.51
1 primi 2 H
40 1 04/25/2021 71 815 1 9.1 0.05 35.95
1 primi 2 H
40 1 04/26/2021 72 816 1 9.1 0 35.29
1 primi 2 H
40 1 04/27/2021 73 817 1 9.1 0 34.54
1 primi 2 H
40 1 04/28/2021 74 818 1 8.8 0 32.59
1 primi 2 H
40 1 04/29/2021 75 819 1 9.3 0 34.79
1 primi 2 H
40 1 04/30/2021 76 820 1 9.3 0.07 34.27
1 primi 2 H
40 1 05/01/2021 77 821 1 9.1 0.1 34.11
1 primi 2 H
40 1 05/02/2021 78 822 1 9.2 0 34.34
1 primi 2 H
40 1 05/03/2021 79 823 1 9.2 0 34.81
1 primi 2 H
40 1 05/04/2021 80 824 1 9.1 0 34.49
1 primi 2 H
40 1 05/05/2021 81 825 1 8.9 0.06 34.57
1 primi 2 H
40 1 05/06/2021 82 826 1 9.1 0 31.67
1 primi 2 H
40 1 05/07/2021 83 827 1 9.2 0.07 33.37
1 primi 2 H
40 1 05/08/2021 84 828 1 8.9 0.22 33.93
1 primi 2 H
40 1 05/09/2021 85 829 1 8.9 0.06 34.52
1 primi 2 H
40 1 05/10/2021 86 830 1 9.2 0 32.71
1 primi 2 H
40 1 05/11/2021 87 831 1 8.8 0.06 33.03
1 primi 2 H
40 1 05/12/2021 88 832 1 9.1 0 34.2
1 primi 2 H
40 1 05/13/2021 89 833 1 8.8 0.3 33.82
1 primi 2 H
40 1 05/14/2021 90 834 1 9.2 0.05 35.03

1 primi	2	H						
40	1	05/15/2021	91	835	1	9.3	0.04	35.71
1 primi	2	H						
40	1	05/16/2021	92	836	1	9.2	0	35.18
1 primi	2	H						
40	1	05/17/2021	93	837	1	9.1	0.06	35.2
1 primi	2	H						
40	1	05/18/2021	94	838	1	9.2	0.02	35.05
1 primi	2	H						
40	1	05/19/2021	95	839	1	8.9	0	34.99
1 primi	2	H						
40	1	05/20/2021	96	840	1	9.8	0.06	35.45
1 primi	2	H						
40	1	05/21/2021	97	841	1	8.9	0.33	32.45
1 primi	2	H						
40	1	05/22/2021	98	842	1	9.2	0.06	37.06
1 primi	2	H						
40	1	05/23/2021	99	843	1	9.1	0.05	34.4
1 primi	2	H						
40	1	05/24/2021	100	844	1	9.2	0	34.57
1 primi	2	H						
40	1	05/25/2021	101	845	1	8.9	0.44	34.84
1 primi	2	H						
40	1	05/26/2021	102	846	1	9.1	0.05	19.41
1 primi	2	H						
41	1	03/08/2020	6	766	1	9.3	0.2	17.25
1 primi	1	H						
41	1	03/09/2020	7	767	1	8.8	0.03	18.53
1 primi	1	H						
41	1	03/10/2020	8	768	1	8.6	0.07	17.47
1 primi	1	H						
41	1	03/11/2020	9	769	1	9.3	0.06	18.37
1 primi	1	H						
41	1	03/12/2020	10	770	1	9.1	0.07	18.84
1 primi	1	H						
41	1	03/13/2020	11	771	1	9.1	0.04	20.88
1 primi	1	H						
41	1	03/14/2020	12	772	1	8.6	0	19.34
1 primi	1	H						
41	1	03/15/2020	13	773	1	8.9	0.05	19.54
1 primi	1	H						
41	1	03/16/2020	14	774	1	9.1	0.04	19.62
1 primi	1	H						
41	1	03/17/2020	15	775	1	8.8	0.27	20.06
1 primi	1	H						
41	1	03/18/2020	16	776	1	9.8	0.74	19.78
1 primi	1	H						
41	1	03/19/2020	17	777	1	9.5	0.3	19.4
1 primi	1	H						
41	1	03/20/2020	18	778	1	9.5	0.04	20.51
1 primi	1	H						
41	1	03/21/2020	19	779	1	9.3	0	20.97
1 primi	1	H						
41	1	03/22/2020	20	780	1	9.1	0.12	17.55

1 primi	1	H						
41	1	03/23/2020	21	781	1	8.7	0.03	20.09
1 primi	1	H						
41	1	03/24/2020	22	782	1	8.6	0	21.43
1 primi	2	H						
41	1	03/25/2020	23	783	1	8.7	0.02	20.19
1 primi	2	H						
41	1	03/26/2020	24	784	1	8.7	0.03	22.18
1 primi	2	H						
41	1	03/27/2020	25	785	1	8.6	0.28	23.34
1 primi	2	H						
41	1	03/28/2020	26	786	1	8.7	0	22.89
1 primi	2	H						
41	1	03/29/2020	27	787	1	8.7	0.04	21.37
1 primi	2	H						
41	1	03/30/2020	28	788	1	8.7	0.14	22.63
1 primi	2	H						
41	1	03/31/2020	29	789	1	8.4	0.1	22.34
1 primi	2	H						
41	1	04/01/2020	30	790	1	8.4	0.18	23.37
1 primi	2	H						
41	1	04/02/2020	31	791	1	8.5	0.24	23.75
1 primi	2	H						
41	1	04/03/2020	32	792	1	8.7	0	22.4
1 primi	2	H						
41	1	04/04/2020	33	793	1	8.8	0	22.31
1 primi	2	H						
41	1	04/05/2020	34	794	1	8.7	0.22	19.59
1 primi	2	H						
41	1	04/06/2020	35	795	1	8.9	0.22	19.36
1 primi	2	H						
41	1	04/07/2020	36	796	1	8.6	0.19	18.05
1 primi	2	H						
41	1	04/08/2020	37	797	1	8.5	0.19	20.27
1 primi	2	H						
41	1	04/09/2020	38	798	1	8.6	0.25	20.05
1 primi	2	H						
41	1	04/10/2020	39	799	1	9.1	0	22.18
1 primi	2	H						
41	1	04/11/2020	40	800	1	8.8	0	22.37
1 primi	2	H						
41	1	04/12/2020	41	801	1	8.7	0.02	23.68
1 primi	2	H						
41	1	04/13/2020	42	802	1	8.8	0	23.32
1 primi	2	H						
41	1	04/14/2020	43	803	1	9.1	0	21.55
1 primi	2	H						
41	1	04/15/2020	44	804	1	9.2	0	25.41
1 primi	2	H						
42	1	01/27/2020	5	747	1	9.3	0.31	19.38
1 primi	1	H						
42	1	01/28/2020	6	748	1	10.2	0.26	23.74
1 primi	1	H						
42	1	01/29/2020	7	749	1	9.5	0.19	25.13

1 primi	1	H						
42	1	01/30/2020	8	750	1	9.8	0.08	25.49
1 primi	1	H						
42	1	01/31/2020	9	751	1	9.8	0.03	25.45
1 primi	1	H						
42	1	02/01/2020	10	752	1	9.3	0	25.91
1 primi	1	H						
42	1	02/02/2020	11	753	1	9.6	0.08	26.94
1 primi	1	H						
42	1	02/03/2020	12	754	1	9.3	0.02	26.59
1 primi	1	H						
42	1	02/04/2020	13	755	1	9.2	0	25.1
1 primi	1	H						
42	1	02/05/2020	14	756	1	8.9	0	26.42
1 primi	1	H						
42	1	02/06/2020	15	757	1	9.3	0.13	27.74
1 primi	1	H						
42	1	02/07/2020	16	758	1	9.3	0.04	29.16
1 primi	1	H						
42	1	02/08/2020	17	759	1	9.2	0.12	27.29
1 primi	1	H						
42	1	02/09/2020	18	760	1	9.5	0.04	28.78
1 primi	1	H						
42	1	02/10/2020	19	761	1	9.3	0.13	28.45
1 primi	1	H						
42	1	02/11/2020	20	762	1	9.5	0.03	29.84
1 primi	1	H						
42	1	02/12/2020	21	763	1	9.5	0	30.24
1 primi	1	H						
42	1	02/13/2020	22	764	1	9.5	0.08	29.86
1 primi	2	H						
42	1	02/14/2020	23	765	1	9.8	0	28.87
1 primi	2	H						
42	1	02/15/2020	24	766	1	9.6	0.01	30.81
1 primi	2	H						
42	1	02/16/2020	25	767	1	9.5	0	30.35
1 primi	2	H						
42	1	02/17/2020	26	768	1	9.5	0.11	29.37
1 primi	2	H						
42	1	02/18/2020	27	769	1	9.5	0.08	29.62
1 primi	2	H						
42	1	02/19/2020	28	770	1	9.6	0	28.58
1 primi	2	H						
42	1	02/20/2020	29	771	1	9.6	0	28.78
1 primi	2	H						
42	1	02/21/2020	30	772	1	9.9	0	28.93
1 primi	2	H						
42	1	02/22/2020	31	773	1	9.6	0.02	31.48
1 primi	2	H						
42	1	02/23/2020	32	774	1	9.6	0.1	29.05
1 primi	2	H						
42	1	02/24/2020	33	775	1	9.6	0.1	27.15
1 primi	2	H						
42	1	02/25/2020	34	776	1	9.8	0.23	28.68

1 primi 2		H						
42	1	02/26/2020	35	777	1	9.8	0.34	32.12
1 primi 2		H						
42	1	02/27/2020	36	778	1	9.9	0.01	31.02
1 primi 2		H						
42	1	02/28/2020	37	779	1	9.9	0.06	28.79
1 primi 2		H						
42	1	02/29/2020	38	780	1	9.5	0.04	32.59
1 primi 2		H						
42	1	03/01/2020	39	781	1	9.6	0.22	27.46
1 primi 2		H						
42	1	03/02/2020	40	782	1	9.5	0.07	34.2
1 primi 2		H						
42	1	03/03/2020	41	783	1	9.6	0	32.08
1 primi 2		H						
42	1	03/04/2020	42	784	1	9.6	0.07	29.74
1 primi 2		H						
42	1	03/05/2020	43	785	1	9.6	0.06	31.66
1 primi 2		H						
42	1	03/06/2020	44	786	1	9.6	0.02	33.64
1 primi 2		H						
42	1	03/07/2020	45	787	1	9.6	0.08	31.52
1 primi 2		H						
42	1	03/08/2020	46	788	1	9.1	0	29.69
1 primi 2		H						
42	1	03/09/2020	47	789	1	9.2	0	31.05
1 primi 2		H						
42	1	03/10/2020	48	790	1	9.2	0.02	30.76
1 primi 2		H						
42	1	03/11/2020	49	791	1	9.2	0	31.78
1 primi 2		H						
42	1	03/12/2020	50	792	1	9.3	0	30.75
1 primi 2		H						
42	1	03/13/2020	51	793	1	9.6	0	31.21
1 primi 2		H						
42	1	03/14/2020	52	794	1	9.3	0.09	29.88
1 primi 2		H						
42	1	03/15/2020	53	795	1	9.3	0	32.77
1 primi 2		H						
42	1	03/16/2020	54	796	1	9.5	0.06	30.84
1 primi 2		H						
42	1	03/17/2020	55	797	1	9.6	0.28	31.48
1 primi 2		H						
42	1	03/18/2020	56	798	1	9.6	0	30.66
1 primi 2		H						
42	1	05/24/2021	7	1230	2	10	0.37	34.05
2 multi 1		H						
42	1	05/25/2021	8	1231	2	9.6	0.46	38.49
2 multi 1		H						
42	1	05/26/2021	9	1232	2	9.8	0.17	21.37
2 multi 1		H						
43	1	11/29/2020	6	723	1	9.1	0.24	26.11
1 primi 1		H						
43	1	11/30/2020	7	724	1	9.2	0.21	26.71

1 primi	1	H						
43	1	12/01/2020	8	725	1	9.2	0.28	26.66
1 primi	1	H						
43	1	12/02/2020	9	726	1	9.1	0.57	31.14
1 primi	1	H						
43	1	12/03/2020	10	727	1	8.9	0.25	30.23
1 primi	1	H						
43	1	12/04/2020	11	728	1	9.5	0.1	29.69
1 primi	1	H						
43	1	12/05/2020	12	729	1	8.8	0.11	30.85
1 primi	1	H						
43	1	12/06/2020	13	730	1	9.1	0.13	31.8
1 primi	1	H						
43	1	12/07/2020	14	731	1	9.6	0.03	30.63
1 primi	1	H						
43	1	12/08/2020	15	732	1	9.2	0.1	32.04
1 primi	1	H						
43	1	12/09/2020	16	733	1	9.5	0	31.58
1 primi	1	H						
43	1	12/10/2020	17	734	1	9.1	0.12	32.56
1 primi	1	H						
43	1	12/11/2020	18	735	1	8.8	0.45	30.16
1 primi	1	H						
43	1	12/12/2020	19	736	1	8.8	0.12	34.07
1 primi	1	H						
43	1	12/13/2020	20	737	1	8.8	0	29.78
1 primi	1	H						
43	1	12/14/2020	21	738	1	8.8	0.04	30.61
1 primi	1	H						
43	1	12/15/2020	22	739	1	8.9	0	32.61
1 primi	2	H						
43	1	12/16/2020	23	740	1	8.5	0	32.75
1 primi	2	H						
43	1	12/17/2020	24	741	1	8.6	0	31.95
1 primi	2	H						
43	1	12/18/2020	25	742	1	8.8	0.13	31.91
1 primi	2	H						
43	1	12/19/2020	26	743	1	8.8	0.11	30.32
1 primi	2	H						
43	1	12/20/2020	27	744	1	8.9	0	31.21
1 primi	2	H						
43	1	12/21/2020	28	745	1	8.9	0.03	28.15
1 primi	2	H						
43	1	12/22/2020	29	746	1	8.9	0.08	29.7
1 primi	2	H						
43	1	12/23/2020	30	747	1	8.8	0.03	30.61
1 primi	2	H						
43	1	12/24/2020	31	748	1	8.8	0.11	31.18
1 primi	2	H						
43	1	12/25/2020	32	749	1	8.9	0.06	30.14
1 primi	2	H						
43	1	12/26/2020	33	750	1	8.8	0	33.99
1 primi	2	H						
44	1	03/13/2021	9	740	1	9.6	0.31	24.2

1 primi 1	H
44	1	03/14/2021	10	741	1	9.9	0.14	21.42
1 primi 1	H
44	1	03/15/2021	11	742	1	9.2	0	18.63
1 primi 1	H
44	1	03/16/2021	12	743	1	9.9	0.12	19.67
1 primi 1	H
44	1	03/17/2021	13	744	1	9.9	0.01	21.91
1 primi 1	H
44	1	03/18/2021	14	745	1	9.9	0.06	22.93
1 primi 1	H
44	1	03/19/2021	15	746	1	10	0.13	23.74
1 primi 1	H
44	1	03/20/2021	16	747	1	10.3	0.04	24.68
1 primi 1	H
44	1	03/21/2021	17	748	1	10	0.16	24.73
1 primi 1	H
44	1	03/22/2021	18	749	1	10	0.1	25.26
1 primi 1	H
44	1	03/23/2021	19	750	1	10.2	0.07	25.08
1 primi 1	H
44	1	03/24/2021	20	751	1	10.2	0	25.61
1 primi 1	H
44	1	03/25/2021	21	752	1	9.9	0.06	25.67
1 primi 1	H
44	1	03/26/2021	22	753	1	10	0.05	25.23
1 primi 2	H
44	1	03/27/2021	23	754	1	9.8	0.17	24.89
1 primi 2	H
44	1	03/28/2021	24	755	1	9.9	0	25.65
1 primi 2	H
44	1	03/29/2021	25	756	1	10	0.08	27.17
1 primi 2	H
44	1	03/30/2021	26	757	1	9.9	0.01	28.2
1 primi 2	H
44	1	03/31/2021	27	758	1	9.8	0.13	29
1 primi 2	H
44	1	04/01/2021	28	759	1	9.9	0.15	30.5
1 primi 2	H
44	1	04/02/2021	29	760	1	9.8	0.04	30.81
1 primi 2	H
44	1	04/03/2021	30	761	1	9.9	0	33.33
1 primi 2	H
44	1	04/04/2021	31	762	1	9.6	0	31.29
1 primi 2	H
44	1	04/05/2021	32	763	1	9.8	0.13	32.54
1 primi 2	H
44	1	04/06/2021	33	764	1	9.6	0.03	30.78
1 primi 2	H
44	1	04/07/2021	34	765	1	10.2	0.12	29.5
1 primi 2	H
44	1	04/08/2021	35	766	1	9.8	0.01	30.06
1 primi 2	H
44	1	04/09/2021	36	767	1	9.6	0.01	31.44

1 primi 2		H						
44	1	04/10/2021	37	768	1	9.8	0	33.83
1 primi 2		H						
44	1	04/11/2021	38	769	1	9.9	0.14	31.17
1 primi 2		H						
44	1	04/12/2021	39	770	1	9.8	0.13	32.08
1 primi 2		H						
44	1	04/13/2021	40	771	1	9.8	0	32.91
1 primi 2		H						
44	1	04/14/2021	41	772	1	9.3	0.06	33.25
1 primi 2		H						
44	1	04/15/2021	42	773	1	9.3	0.02	32.02
1 primi 2		H						
44	1	04/16/2021	43	774	1	9.3	0.14	33.1
1 primi 2		H						
44	1	04/17/2021	44	775	1	9.3	0.04	32.74
1 primi 2		H						
44	1	04/18/2021	45	776	1	9.5	0.13	33.31
1 primi 2		H						
44	1	04/19/2021	46	777	1	9.6	0.12	34.52
1 primi 2		H						
44	1	04/20/2021	47	778	1	9.3	0.13	33.4
1 primi 2		H						
44	1	04/21/2021	48	779	1	9.5	0.01	34.18
1 primi 2		H						
44	1	04/22/2021	49	780	1	9.8	0.06	34.38
1 primi 2		H						
44	1	04/23/2021	50	781	1	9.5	0	34.62
1 primi 2		H						
44	1	04/24/2021	51	782	1	9.5	0.13	30.81
1 primi 2		H						
44	1	04/25/2021	52	783	1	9.8	0	29.61
1 primi 2		H						
44	1	04/26/2021	53	784	1	9.8	0	28.78
1 primi 2		H						
44	1	04/27/2021	54	785	1	9.3	0	23.35
1 primi 2		H						
44	1	04/28/2021	55	786	1	9.2	0.16	26.02
1 primi 2		H						
44	1	04/29/2021	56	787	1	9.5	0.06	28.42
1 primi 2		H						
44	1	04/30/2021	57	788	1	9.6	0	33.08
1 primi 2		H						
44	1	05/01/2021	58	789	1	10	0.13	32.89
1 primi 2		H						
44	1	05/02/2021	59	790	1	9.6	0.12	33.08
1 primi 2		H						
44	1	05/03/2021	60	791	1	9.6	0	32.6
1 primi 2		H						
44	1	05/04/2021	61	792	1	9.3	0	32.26
1 primi 2		H						
44	1	05/05/2021	62	793	1	9.3	0.11	32.12
1 primi 2		H						
44	1	05/06/2021	63	794	1	9.6	0.03	32.57

1 primi	2	H						
44	1	05/07/2021	64	795	1	9.6	0.09	32.54
1 primi	2	H						
44	1	05/08/2021	65	796	1	9.5	0.05	30.61
1 primi	2	H						
44	1	05/09/2021	66	797	1	9.8	0.12	34.26
1 primi	2	H						
44	1	05/10/2021	67	798	1	9.6	0	32.33
1 primi	2	H						
44	1	05/11/2021	68	799	1	10	0	34.56
1 primi	2	H						
44	1	05/12/2021	69	800	1	10	0	35.71
1 primi	2	H						
44	1	05/13/2021	70	801	1	9.8	0.02	35.3
1 primi	2	H						
44	1	05/14/2021	71	802	1	9.8	0.1	33.41
1 primi	2	H						
44	1	05/15/2021	72	803	1	10	0.03	36.02
1 primi	2	H						
44	1	05/16/2021	73	804	1	9.9	0.02	34.75
1 primi	2	H						
44	1	05/17/2021	74	805	1	9.9	0	36.12
1 primi	2	H						
44	1	05/18/2021	75	806	1	9.8	0	35.52
1 primi	2	H						
44	1	05/19/2021	76	807	1	9.9	0	35.11
1 primi	2	H						
44	1	05/20/2021	77	808	1	9.8	0.02	35.66
1 primi	2	H						
44	1	05/21/2021	78	809	1	9.9	0.02	35.2
1 primi	2	H						
44	1	05/22/2021	79	810	1	9.9	0.09	36.4
1 primi	2	H						
44	1	05/23/2021	80	811	1	9.8	0.03	35.8
1 primi	2	H						
44	1	05/24/2021	81	812	1	10.2	0	37.26
1 primi	2	H						
44	1	05/25/2021	82	813	1	10	0.02	36.9
1 primi	2	H						
44	1	05/26/2021	83	814	1	9.8	0	18.72
1 primi	2	H						
45	1	04/24/2013	9	795	1	9.2	0	25.78
1 primi	1	H						
45	1	04/25/2013	10	796	1	9.2	0	27.09
1 primi	1	H						
45	1	04/26/2013	11	797	1	9.2	0	26.37
1 primi	1	H						
45	1	04/27/2013	12	798	1	9.3	0	28.51
1 primi	1	H						
45	1	04/28/2013	13	799	1	9.3	0	31.01
1 primi	1	H						
45	1	04/29/2013	14	800	1	9.2	0	27.17
1 primi	1	H						
45	1	04/30/2013	15	801	1	8.7	0	29.22

1 primi	1	H						
45	1	05/01/2013	16	802	1	8.9	0	27.37
1 primi	1	H						
45	1	05/02/2013	17	803	1	8.9	0	30.45
1 primi	1	H						
45	1	05/03/2013	18	804	1	9.5	0	27.17
1 primi	1	H						
45	1	05/04/2013	19	805	1	11.4	0	27.4
1 primi	1	H						
45	1	05/05/2013	20	806	1	8.9	0	18.37
1 primi	1	H						
45	1	05/06/2013	21	807	1	10.3	0	32.43
1 primi	1	H						
45	1	05/07/2013	22	808	1	9.9	0	31.42
1 primi	2	H						
45	1	05/08/2013	23	809	1	9.6	0	31.8
1 primi	2	H						
45	1	05/09/2013	24	810	1	9.5	0	30.07
1 primi	2	H						
45	1	05/10/2013	25	811	1	10.3	0	30.2
1 primi	2	H						
45	1	05/11/2013	26	812	1	9.8	0	32.73
1 primi	2	H						
45	1	03/26/2014	4	1131	2	8.9	0.02	27.6
2 multi	1	H						
45	1	03/27/2014	5	1132	2	9.2	0	29.55
2 multi	1	H						
45	1	03/28/2014	6	1133	2	9.2	0.01	35.69
2 multi	1	H						
45	1	03/29/2014	7	1134	2	9.2	0.17	30.34
2 multi	1	H						
45	1	03/30/2014	8	1135	2	8.9	0	32
2 multi	1	H						
45	1	03/31/2014	9	1136	2	9.3	0	32.67
2 multi	1	H						
45	1	04/01/2014	10	1137	2	9.2	0	34.31
2 multi	1	H						
45	1	04/02/2014	11	1138	2	9.2	0	35.93
2 multi	1	H						
45	1	04/03/2014	12	1139	2	8.8	0	33.47
2 multi	1	H						
45	1	04/04/2014	13	1140	2	9.1	0	35.13
2 multi	1	H						
45	1	04/05/2014	14	1141	2	9.1	0	36.98
2 multi	1	H						
45	1	04/06/2014	15	1142	2	9.1	0	36.63
2 multi	1	H						
45	1	04/07/2014	16	1143	2	8.7	0	41.38
2 multi	1	H						
45	1	04/08/2014	17	1144	2	8.7	0	33.82
2 multi	1	H						
45	1	04/09/2014	18	1145	2	8.9	0.06	40.53
2 multi	1	H						
45	1	04/10/2014	19	1146	2	9.1	0	38.43

2 multi	1	H						
45	1	04/11/2014	20	1147	2	9.1	0	42.89
2 multi	1	H						
45	1	04/12/2014	21	1148	2	8.9	0	41.63
2 multi	1	H						
45	1	04/13/2014	22	1149	2	8.9	0.11	40.92
2 multi	2	H						
45	1	04/14/2014	23	1150	2	9.3	0	40.32
2 multi	2	H						
45	1	04/15/2014	24	1151	2	9.2	0	42.16
2 multi	2	H						
45	1	04/16/2014	25	1152	2	9.5	0	41.94
2 multi	2	H						
45	1	04/17/2014	26	1153	2	9.3	0	40.54
2 multi	2	H						
45	1	04/18/2014	27	1154	2	8.9	0.01	43.33
2 multi	2	H						
45	1	04/19/2014	28	1155	2	9.5	0	43.12
2 multi	2	H						
45	1	04/20/2014	29	1156	2	9.2	0.49	41.39
2 multi	2	H						
45	1	04/21/2014	30	1157	2	8.9	0	46.04
2 multi	2	H						
45	1	04/22/2014	31	1158	2	9.2	0.03	46.62
2 multi	2	H						
45	1	04/23/2014	32	1159	2	9.2	0	42.9
2 multi	2	H						
45	1	04/24/2014	33	1160	2	9.2	0	43.87
2 multi	2	H						
45	1	04/25/2014	34	1161	2	9.2	0	41.55
2 multi	2	H						
45	1	04/26/2014	35	1162	2	9.3	0	44.28
2 multi	2	H						
45	1	04/27/2014	36	1163	2	9.3	0	44.09
2 multi	2	H						
45	1	04/28/2014	37	1164	2	9.1	0	42.67
2 multi	2	H						
45	1	04/29/2014	38	1165	2	9.1	0	49.57
2 multi	2	H						
45	1	05/26/2014	65	1192	2	9.5	0	43.48
2 multi	2	H						
45	1	05/27/2014	66	1193	2	9.6	0.02	38.92
2 multi	2	H						
45	1	05/28/2014	67	1194	2	9.8	0.03	45.89
2 multi	2	H						
45	1	05/29/2014	68	1195	2	9.6	0	47.81
2 multi	2	H						
45	1	05/30/2014	69	1196	2	9.3	0	43.76
2 multi	2	H						
45	1	05/31/2014	70	1197	2	9.5	0.08	45.5
2 multi	2	H						
45	1	06/01/2014	71	1198	2	9.3	0	40.29
2 multi	2	H						
45	1	06/02/2014	72	1199	2	9.5	0	45.46

2 multi	2	H						
45	1	06/03/2014	73	1200	2	9.3	0.09	43.35
2 multi	2	H						
45	1	06/04/2014	74	1201	2	9.6	0	44.79
2 multi	2	H						
45	1	06/05/2014	75	1202	2	9.3	0	41.6
2 multi	2	H						
45	1	06/06/2014	76	1203	2	9.8	0	41.9
2 multi	2	H						
45	1	06/07/2014	77	1204	2	9.6	0	43.71
2 multi	2	H						
45	1	06/08/2014	78	1205	2	10	0	43.16
2 multi	2	H						
45	1	06/09/2014	79	1206	2	9.8	0	37.8
2 multi	2	H						
45	1	06/10/2014	80	1207	2	9.9	0.16	43.11
2 multi	2	H						
45	1	06/11/2014	81	1208	2	9.8	0	41.76
2 multi	2	H						
45	1	06/12/2014	82	1209	2	9.3	0	39.46
2 multi	2	H						
45	1	06/13/2014	83	1210	2	10.9	0	45.33
2 multi	2	H						
45	1	06/14/2014	84	1211	2	10.2	0	40.59
2 multi	2	H						
45	1	06/15/2014	85	1212	2	10.2	0	39.65
2 multi	2	H						
45	1	06/16/2014	86	1213	2	10	0	42.9
2 multi	2	H						
45	1	06/17/2014	87	1214	2	10	0.05	43.45
2 multi	2	H						
45	1	06/18/2014	88	1215	2	9.9	0	41.51
2 multi	2	H						
45	1	06/19/2014	89	1216	2	9.6	0	42.52
2 multi	2	H						
45	1	06/20/2014	90	1217	2	9.6	0	40.18
2 multi	2	H						
45	1	06/21/2014	91	1218	2	9.6	0	41.91
2 multi	2	H						
45	1	06/22/2014	92	1219	2	9.6	0	42.53
2 multi	2	H						
45	1	06/23/2014	93	1220	2	9.6	0	42.5
2 multi	2	H						
45	1	06/24/2014	94	1221	2	9.5	0	42.34
2 multi	2	H						
45	1	06/25/2014	95	1222	2	9.2	0	42.18
2 multi	2	H						
45	1	06/26/2014	96	1223	2	9.6	0	39.46
2 multi	2	H						
45	1	06/27/2014	97	1224	2	9.5	0	41.86
2 multi	2	H						
45	1	06/28/2014	98	1225	2	9.5	0	43.34
2 multi	2	H						
45	1	06/29/2014	99	1226	2	9.5	0.15	41.68

2 multi 2		H						
45	1	06/30/2014	100	1227	2	9.5	0	38.3
2 multi 2		H						
45	1	07/01/2014	101	1228	2	9.5	0.14	40.73
2 multi 2		H						
45	1	07/02/2014	102	1229	2	9.5	0	39.75
2 multi 2		H						
45	1	07/03/2014	103	1230	2	9.6	0	37.72
2 multi 2		H						
45	1	07/04/2014	104	1231	2	9.3	0.05	44.15
2 multi 2		H						
45	1	07/05/2014	105	1232	2	9.9	0	36.77
2 multi 2		H						
45	1	07/06/2014	106	1233	2	9.9	0	36.91
2 multi 2		H						
45	1	07/07/2014	107	1234	2	9.6	0	28.92
2 multi 2		H						
45	1	07/08/2014	108	1235	2	9.9	0	28.52
2 multi 2		H						
45	1	07/09/2014	109	1236	2	9.1	0	32.9
2 multi 2		H						
45	1	07/10/2014	110	1237	2	10.2	0.18	39.58
2 multi 2		H						
45	1	07/11/2014	111	1238	2	10	0.01	38.59
2 multi 2		H						
45	1	07/12/2014	112	1239	2	9.9	0	38.79
2 multi 2		H						
45	1	07/13/2014	113	1240	2	9.8	0	39.06
2 multi 2		H						
45	1	07/14/2014	114	1241	2	10	0	39.22
2 multi 2		H						
45	1	07/15/2014	115	1242	2	9.9	0	41.9
2 multi 2		H						
45	1	07/16/2014	116	1243	2	10	0	39.1
2 multi 2		H						
45	1	07/17/2014	117	1244	2	9.9	0	38.99
2 multi 2		H						
45	1	07/18/2014	118	1245	2	9.6	0	38.75
2 multi 2		H						
45	1	07/19/2014	119	1246	2	9.9	0	38.91
2 multi 2		H						
45	1	07/20/2014	120	1247	2	9.9	0	39.38
2 multi 2		H						
45	1	07/21/2014	121	1248	2	9.8	0	39.69
2 multi 2		H						
45	1	07/22/2014	122	1249	2	9.8	0.04	39.55
2 multi 2		H						
45	1	07/23/2014	123	1250	2	10	0	41.54
2 multi 2		H						
45	1	07/24/2014	124	1251	2	9.8	0.01	33.33
2 multi 2		H						
45	1	07/25/2014	125	1252	2	9.9	0	28.28
2 multi 2		H						
45	1	07/26/2014	126	1253	2	9.6	0	38.04

2 multi 2		H						
45	1	07/27/2014	127	1254	2	10.2	0	36.72
2 multi 2		H						
45	1	07/28/2014	128	1255	2	9.9	0.02	37.92
2 multi 2		H						
45	1	07/29/2014	129	1256	2	9.9	0	35.14
2 multi 2		H						
45	1	07/30/2014	130	1257	2	9.9	0	38.93
2 multi 2		H						
45	1	07/31/2014	131	1258	2	9.8	0.04	35.75
2 multi 2		H						
45	1	08/01/2014	132	1259	2	9.8	0	39.7
2 multi 2		H						
45	1	08/02/2014	133	1260	2	9.8	0	37.92
2 multi 2		H						
45	1	08/03/2014	134	1261	2	9.6	0	39.98
2 multi 2		H						
45	1	08/04/2014	135	1262	2	9.9	0	35.59
2 multi 2		H						
45	1	08/05/2014	136	1263	2	9.6	0.01	37.82
2 multi 2		H						
45	1	08/06/2014	137	1264	2	9.6	0	39.61
2 multi 2		H						
45	1	08/07/2014	138	1265	2	10	0	38.02
2 multi 2		H						
45	1	08/08/2014	139	1266	2	10	0	34.09
2 multi 2		H						
45	1	08/09/2014	140	1267	2	9.9	0	39.26
2 multi 2		H						
45	1	08/10/2014	141	1268	2	10	0	39.55
2 multi 2		H						
45	1	08/11/2014	142	1269	2	9.9	0	38.61
2 multi 2		H						
45	1	08/12/2014	143	1270	2	9.8	0	36.12
2 multi 2		H						
45	1	08/13/2014	144	1271	2	9.5	0	37.11
2 multi 2		H						
45	1	08/14/2014	145	1272	2	9.8	0	38.03
2 multi 2		H						
45	1	08/15/2014	146	1273	2	10.2	0.61	36.74
2 multi 2		H						
45	1	08/16/2014	147	1274	2	9.8	0	36.24
2 multi 2		H						
45	1	08/17/2014	148	1275	2	9.9	0	41.77
2 multi 2		H						
45	1	08/18/2014	149	1276	2	9.6	0	38.55
2 multi 2		H						
45	1	08/19/2014	150	1277	2	9.8	0	38.31
2 multi 2		H						
45	1	08/20/2014	151	1278	2	9.8	0	35.84
2 multi 2		H						
45	1	08/21/2014	152	1279	2	9.8	0	37.53
2 multi 2		H						
45	1	08/22/2014	153	1280	2	9.5	0	33.01

2 multi 2		H						
45	1	08/23/2014	154	1281	2	9.6	0	36.75
2 multi 2		H						
45	1	08/24/2014	155	1282	2	9.2	0	39.32
2 multi 2		H						
45	1	08/25/2014	156	1283	2	9.5	0	37.74
2 multi 2		H						
45	1	08/26/2014	157	1284	2	9.8	0	40.74
2 multi 2		H						
45	1	08/27/2014	158	1285	2	9.6	0	38
2 multi 2		H						
45	1	08/28/2014	159	1286	2	9.8	0.33	38.27
2 multi 2		H						
45	1	08/29/2014	160	1287	2	9.8	0.65	37.45
2 multi 2		H						
45	1	08/30/2014	161	1288	2	9.5	0	35.9
2 multi 2		H						
45	1	08/31/2014	162	1289	2	9.6	0	44.02
2 multi 2		H						
45	1	09/01/2014	163	1290	2	9.8	0	39.17
2 multi 2		H						
45	1	09/02/2014	164	1291	2	9.5	0	29.57
2 multi 2		H						
45	1	09/03/2014	165	1292	2	9.6	0	38.22
2 multi 2		H						
45	1	09/04/2014	166	1293	2	9.8	0	38.06
2 multi 2		H						
45	1	09/05/2014	167	1294	2	9.9	0	43.3
2 multi 2		H						
45	1	09/06/2014	168	1295	2	10.4	0	33.9
2 multi 2		H						
45	1	09/07/2014	169	1296	2	9.9	0.03	34.16
2 multi 2		H						
45	1	09/08/2014	170	1297	2	10.3	0	36.65
2 multi 2		H						
45	1	09/09/2014	171	1298	2	10.2	0	34.43
2 multi 2		H						
45	1	09/10/2014	172	1299	2	10	0	37
2 multi 2		H						
45	1	09/11/2014	173	1300	2	10.2	0	36.03
2 multi 2		H						
45	1	09/12/2014	174	1301	2	9.9	0	37.89
2 multi 2		H						
45	1	09/13/2014	175	1302	2	9.9	0	32.36
2 multi 2		H						
45	1	09/14/2014	176	1303	2	10	0	35.4
2 multi 2		H						
45	1	09/15/2014	177	1304	2	10.2	0	36.36
2 multi 2		H						
45	1	09/16/2014	178	1305	2	10.2	0	36.16
2 multi 2		H						
45	1	09/17/2014	179	1306	2	10	0.22	29.54
2 multi 2		H						
45	1	09/18/2014	180	1307	2	10	0	30.9

2 multi 2 H
45 1 09/19/2014 181 1308 2 10 0.32 32.76
2 multi 2 H
45 1 09/20/2014 182 1309 2 10.2 0 31.07
2 multi 2 H
45 1 09/21/2014 183 1310 2 9.6 0 36.69
2 multi 2 H
45 1 09/22/2014 184 1311 2 9.6 0.05 35.05
2 multi 2 H
45 1 01/27/2016 9 1803 3 8.6 0 32.27
2 multi 1 H
45 1 01/28/2016 10 1804 3 9.3 0 28.19
2 multi 1 H
45 1 01/29/2016 11 1805 3 8.6 0 30.49
2 multi 1 H
45 1 01/30/2016 12 1806 3 8.6 0 30.47
2 multi 1 H
45 1 01/31/2016 13 1807 3 8.8 0 32.97
2 multi 1 H
45 1 02/01/2016 14 1808 3 10.2 0 32.72
2 multi 1 H
45 1 02/02/2016 15 1809 3 9.2 0 33.07
2 multi 1 H
45 1 02/03/2016 16 1810 3 9.3 0 32.76
2 multi 1 H
45 1 02/04/2016 17 1811 3 8.9 0 33.99
2 multi 1 H
45 1 02/05/2016 18 1812 3 8.8 0 34.37
2 multi 1 H
45 1 02/06/2016 19 1813 3 8.9 0 35.29
2 multi 1 H
45 1 02/07/2016 20 1814 3 9.3 0 37.23
2 multi 1 H
45 1 02/08/2016 21 1815 3 9.5 0.28 36.79
2 multi 1 H
45 1 02/09/2016 22 1816 3 9.2 0 35.91
2 multi 2 H
45 1 02/10/2016 23 1817 3 8.7 0 38.75
2 multi 2 H
45 1 02/11/2016 24 1818 3 8.9 0.1 34.73
2 multi 2 H
45 1 02/12/2016 25 1819 3 9.6 0 38.55
2 multi 2 H
45 1 02/13/2016 26 1820 3 9.3 0 36.41
2 multi 2 H
45 1 02/14/2016 27 1821 3 9.6 0 38.2
2 multi 2 H
45 1 02/15/2016 28 1822 3 8.8 0.03 39.16
2 multi 2 H
45 1 02/16/2016 29 1823 3 8.9 0 40.74
2 multi 2 H
45 1 02/17/2016 30 1824 3 9.9 0 39.43
2 multi 2 H
45 1 02/18/2016 31 1825 3 9.6 0 36.74

2 multi 2		H						
45	1	02/19/2016	32	1826	3	9.2	0.14	38.46
2 multi 2		H						
45	1	02/20/2016	33	1827	3	8.8	0	38.76
2 multi 2		H						
45	1	02/21/2016	34	1828	3	9.1	0	41.48
2 multi 2		H						
45	1	02/22/2016	35	1829	3	8.8	0	39.78
2 multi 2		H						
45	1	02/23/2016	36	1830	3	8.8	0.11	39.42
2 multi 2		H						
45	1	02/24/2016	37	1831	3	8.7	0.03	44.19
2 multi 2		H						
45	1	02/25/2016	38	1832	3	8.9	0	40.24
2 multi 2		H						
45	1	02/26/2016	39	1833	3	9.3	0	40.02
2 multi 2		H						
45	1	02/27/2016	40	1834	3	9.5	0.08	42.47
2 multi 2		H						
45	1	02/28/2016	41	1835	3	9.3	0.01	34.64
2 multi 2		H						
45	1	02/29/2016	42	1836	3	9.2	0	42.61
2 multi 2		H						
45	1	03/01/2016	43	1837	3	9.2	0.08	39.36
2 multi 2		H						
45	1	03/02/2016	44	1838	3	9.2	0.05	33.66
2 multi 2		H						
45	1	03/03/2016	45	1839	3	9.2	0	35.78
2 multi 2		H						
45	1	03/04/2016	46	1840	3	9.6	0.14	38.68
2 multi 2		H						
45	1	03/05/2016	47	1841	3	9.3	0.01	38.34
2 multi 2		H						
45	1	03/06/2016	48	1842	3	9.3	0	48.04
2 multi 2		H						
45	1	03/07/2016	49	1843	3	9.5	0	36.37
2 multi 2		H						
45	1	03/08/2016	50	1844	3	9.5	0	38.42
2 multi 2		H						
45	1	03/09/2016	51	1845	3	9.5	0.08	38.34
2 multi 2		H						
45	1	03/10/2016	52	1846	3	9.8	0	39.35
2 multi 2		H						
45	1	03/11/2016	53	1847	3	9.1	0.06	40.49
2 multi 2		H						
45	1	03/12/2016	54	1848	3	8.7	0.02	39.34
2 multi 2		H						
45	1	03/13/2016	55	1849	3	9.1	0	46
2 multi 2		H						
45	1	03/14/2016	56	1850	3	9.2	0.35	42.01
2 multi 2		H						
45	1	03/15/2016	57	1851	3	8.9	0	38.96
2 multi 2		H						
45	1	03/16/2016	58	1852	3	8.9	0	39.6

2 multi 2		H						
45	1	03/17/2016	59	1853	3	8.9	0	41.19
2 multi 2		H						
45	1	03/18/2016	60	1854	3	9.1	0	40.48
2 multi 2		H						
45	1	03/19/2016	61	1855	3	8.9	0	40.65
2 multi 2		H						
45	1	03/20/2016	62	1856	3	8.9	0	39.31
2 multi 2		H						
45	1	03/21/2016	63	1857	3	9.2	0.16	43.05
2 multi 2		H						
45	1	03/22/2016	64	1858	3	9.5	0.02	39.4
2 multi 2		H						
45	1	03/23/2016	65	1859	3	9.3	0	40.83
2 multi 2		H						
45	1	03/24/2016	66	1860	3	8.8	0	36.99
2 multi 2		H						
45	1	03/25/2016	67	1861	3	8.7	0	37.18
2 multi 2		H						
45	1	03/26/2016	68	1862	3	9.9	0	37.41
2 multi 2		H						
45	1	03/27/2016	69	1863	3	9.9	0	35.84
2 multi 2		H						
45	1	03/28/2016	70	1864	3	8.8	0	34.45
2 multi 2		H						
45	1	03/29/2016	71	1865	3	8.6	0	34.28
2 multi 2		H						
45	1	03/30/2016	72	1866	3	8.7	0	37.85
2 multi 2		H						
45	1	03/31/2016	73	1867	3	9.3	0	38.07
2 multi 2		H						
45	1	04/01/2016	74	1868	3	9.8	0.01	41.67
2 multi 2		H						
45	1	04/02/2016	75	1869	3	9.6	0	41.88
2 multi 2		H						
45	1	04/03/2016	76	1870	3	9.5	0	40.17
2 multi 2		H						
45	1	04/04/2016	77	1871	3	9.6	0	42.53
2 multi 2		H						
45	1	04/05/2016	78	1872	3	9.3	0	42.08
2 multi 2		H						
45	1	04/06/2016	79	1873	3	9.3	0.37	42.04
2 multi 2		H						
45	1	04/07/2016	80	1874	3	9.1	0.09	41.91
2 multi 2		H						
45	1	04/08/2016	81	1875	3	9.6	0	41.12
2 multi 2		H						
45	1	04/09/2016	82	1876	3	9.2	0	40.41
2 multi 2		H						
45	1	04/10/2016	83	1877	3	9.6	0	41.79
2 multi 2		H						
45	1	04/11/2016	84	1878	3	9.5	0.03	40.18
2 multi 2		H						
45	1	04/12/2016	85	1879	3	9.6	0.03	43.5

2 multi	2	H						
45	1	04/13/2016	86	1880	3	9.5	0	41.13
2 multi	2	H						
45	1	04/14/2016	87	1881	3	9.5	0	42.33
2 multi	2	H						
45	1	04/15/2016	88	1882	3	9.2	0.09	40.54
2 multi	2	H						
45	1	04/16/2016	89	1883	3	9.6	0	45.16
2 multi	2	H						
45	1	04/17/2016	90	1884	3	9.6	0	40.44
2 multi	2	H						
45	1	04/18/2016	91	1885	3	9.3	0.02	40.96
2 multi	2	H						
45	1	04/19/2016	92	1886	3	9.6	0	39.74
2 multi	2	H						
45	1	04/20/2016	93	1887	3	9.5	0.02	39.07
2 multi	2	H						
45	1	04/21/2016	94	1888	3	9.6	0	37.6
2 multi	2	H						
45	1	04/22/2016	95	1889	3	9.5	0	40.37
2 multi	2	H						
45	1	04/23/2016	96	1890	3	9.3	0.09	39.74
2 multi	2	H						
45	1	04/24/2016	97	1891	3	9.5	0.01	41.22
2 multi	2	H						
45	1	04/25/2016	98	1892	3	9.3	0	40.59
2 multi	2	H						
45	1	04/26/2016	99	1893	3	9.3	0	38.55
2 multi	2	H						
45	1	04/27/2016	100	1894	3	9.2	0	38.28
2 multi	2	H						
45	1	04/28/2016	101	1895	3	9.5	0	40.22
2 multi	2	H						
45	1	04/29/2016	102	1896	3	9.3	0	39.24
2 multi	2	H						
45	1	04/30/2016	103	1897	3	9.5	0.04	42.21
2 multi	2	H						
45	1	05/01/2016	104	1898	3	9.5	0	39.35
2 multi	2	H						
45	1	05/02/2016	105	1899	3	9.3	0.18	35.62
2 multi	2	H						
45	1	05/03/2016	106	1900	3	9.8	0	39.59
2 multi	2	H						
45	1	05/04/2016	107	1901	3	9.3	0	38.61
2 multi	2	H						
45	1	05/05/2016	108	1902	3	9.5	0	39.34
2 multi	2	H						
45	1	05/06/2016	109	1903	3	9.6	0	40.35
2 multi	2	H						
45	1	05/07/2016	110	1904	3	9.1	0	39.06
2 multi	2	H						
45	1	05/08/2016	111	1905	3	0	0	49.85
2 multi	2	H						
45	1	05/09/2016	112	1906	3	10.3	0	37.78

2 multi	2	H						
45	1	05/10/2016	113	1907	3	10.3	0.03	38.94
2 multi	2	H						
45	1	05/11/2016	114	1908	3	9.8	0	39.28
2 multi	2	H						
45	1	05/12/2016	115	1909	3	9.6	0	37.59
2 multi	2	H						
45	1	05/13/2016	116	1910	3	9.5	0	40.85
2 multi	2	H						
45	1	05/14/2016	117	1911	3	9.5	0	40.99
2 multi	2	H						
45	1	05/15/2016	118	1912	3	9.6	0	38.78
2 multi	2	H						
45	1	05/16/2016	119	1913	3	9.2	0.02	39.3
2 multi	2	H						
45	1	05/17/2016	120	1914	3	9.2	0	39.46
2 multi	2	H						
45	1	05/18/2016	121	1915	3	9.5	0	40.51
2 multi	2	H						
45	1	05/19/2016	122	1916	3	9.3	0	39.73
2 multi	2	H						
45	1	05/20/2016	123	1917	3	9.5	0	38.52
2 multi	2	H						
45	1	05/21/2016	124	1918	3	9.2	0.11	38.56
2 multi	2	H						
45	1	05/22/2016	125	1919	3	9.3	0.08	40.13
2 multi	2	H						
45	1	05/23/2016	126	1920	3	9.5	0.07	39.87
2 multi	2	H						
45	1	05/24/2016	127	1921	3	9.8	0	39.12
2 multi	2	H						
45	1	05/25/2016	128	1922	3	9.6	0	38.24
2 multi	2	H						
45	1	05/26/2016	129	1923	3	9.6	0	38.92
2 multi	2	H						
45	1	05/27/2016	130	1924	3	9.5	0	39.55
2 multi	2	H						
45	1	05/28/2016	131	1925	3	9.5	0	39.02
2 multi	2	H						
45	1	05/29/2016	132	1926	3	9.6	0	39.03
2 multi	2	H						
45	1	05/30/2016	133	1927	3	9.5	0.08	38.63
2 multi	2	H						
45	1	05/31/2016	134	1928	3	9.2	0.04	37.1
2 multi	2	H						
45	1	06/01/2016	135	1929	3	9.8	0.04	45.9
2 multi	2	H						
45	1	06/02/2016	136	1930	3	9.3	0	40.24
2 multi	2	H						
45	1	06/03/2016	137	1931	3	9.9	0	44.82
2 multi	2	H						
45	1	06/04/2016	138	1932	3	0	0.04	29.09
2 multi	2	H						
45	1	06/05/2016	139	1933	3	10.2	0	38.1

2 multi 2		H						
45	1	06/06/2016	140	1934	3	9.8	0	36.49
2 multi 2		H						
45	1	06/07/2016	141	1935	3	9.8	0	40.88
2 multi 2		H						
45	1	06/08/2016	142	1936	3	9.6	0.24	35.85
2 multi 2		H						
45	1	06/09/2016	143	1937	3	9.6	0	35.9
2 multi 2		H						
45	1	06/10/2016	144	1938	3	9.5	0	38.44
2 multi 2		H						
45	1	06/11/2016	145	1939	3	9.6	0	37.69
2 multi 2		H						
45	1	06/12/2016	146	1940	3	9.6	0	38.27
2 multi 2		H						
45	1	06/13/2016	147	1941	3	9.6	0	35.9
2 multi 2		H						
45	1	06/14/2016	148	1942	3	9.8	0	36.12
2 multi 2		H						
45	1	06/15/2016	149	1943	3	9.6	0	37.87
2 multi 2		H						
45	1	06/16/2016	150	1944	3	9.8	0	35.27
2 multi 2		H						
45	1	06/17/2016	151	1945	3	9.5	0.32	34.48
2 multi 2		H						
45	1	06/18/2016	152	1946	3	10	0	39.08
2 multi 2		H						
45	1	06/19/2016	153	1947	3	9.3	0.3	35.49
2 multi 2		H						
45	1	06/20/2016	154	1948	3	9.6	0.16	39.06
2 multi 2		H						
45	1	06/21/2016	155	1949	3	9.9	0	36.63
2 multi 2		H						
45	1	06/22/2016	156	1950	3	9.9	0.11	34.66
2 multi 2		H						
45	1	06/23/2016	157	1951	3	9.5	0.05	34.02
2 multi 2		H						
45	1	06/24/2016	158	1952	3	9.8	0.07	37
2 multi 2		H						
45	1	06/25/2016	159	1953	3	9.8	0	34.87
2 multi 2		H						
45	1	06/26/2016	160	1954	3	9.6	0	37.3
2 multi 2		H						
45	1	06/27/2016	161	1955	3	9.8	0	35.85
2 multi 2		H						
45	1	06/28/2016	162	1956	3	9.8	0	36.66
2 multi 2		H						
45	1	06/29/2016	163	1957	3	9.8	0.04	36.78
2 multi 2		H						
45	1	06/30/2016	164	1958	3	9.6	0.19	36.53
2 multi 2		H						
45	1	07/01/2016	165	1959	3	9.8	0	37.87
2 multi 2		H						
45	1	07/02/2016	166	1960	3	9.8	0	39.5

2 multi	2	H						
45	1	07/03/2016	167	1961	3	9.6	0.06	37.74
2 multi	2	H						
45	1	07/04/2016	168	1962	3	9.6	0	37.14
2 multi	2	H						
45	1	07/05/2016	169	1963	3	9.8	0.17	38.1
2 multi	2	H						
45	1	07/06/2016	170	1964	3	9.6	0	34.96
2 multi	2	H						
45	1	12/24/2016	5	2135	4	8.7	0	24.85
2 multi	1	H						
45	1	12/25/2016	6	2136	4	8.6	0.05	24.45
2 multi	1	H						
45	1	12/26/2016	7	2137	4	8.3	0.11	26.08
2 multi	1	H						
45	1	12/27/2016	8	2138	4	8.4	0	28.47
2 multi	1	H						
45	1	12/28/2016	9	2139	4	8.6	0	26.46
2 multi	1	H						
45	1	12/29/2016	10	2140	4	8.5	0.01	27.37
2 multi	1	H						
45	1	12/30/2016	11	2141	4	8.6	0	28.11
2 multi	1	H						
45	1	12/31/2016	12	2142	4	8.6	0	26.88
2 multi	1	H						
45	1	01/01/2017	13	2143	4	8.7	0	29.74
2 multi	1	H						
45	1	01/02/2017	14	2144	4	9.1	0	28.72
2 multi	1	H						
45	1	01/03/2017	15	2145	4	9.1	0.06	33.76
2 multi	1	H						
45	1	01/04/2017	16	2146	4	9.2	0	35.77
2 multi	1	H						
45	1	01/05/2017	17	2147	4	8.8	0	34.27
2 multi	1	H						
45	1	01/06/2017	18	2148	4	8.9	0.08	34.72
2 multi	1	H						
45	1	01/07/2017	19	2149	4	9.2	0	36.35
2 multi	1	H						
45	1	01/08/2017	20	2150	4	8.9	0	35.85
2 multi	1	H						
45	1	01/09/2017	21	2151	4	9.1	0	37.97
2 multi	1	H						
45	1	01/10/2017	22	2152	4	8.9	0	36.44
2 multi	2	H						
45	1	01/11/2017	23	2153	4	9.1	0	38.9
2 multi	2	H						
45	1	01/12/2017	24	2154	4	9.1	0	39.26
2 multi	2	H						
45	1	01/13/2017	25	2155	4	8.9	0	39.62
2 multi	2	H						
45	1	01/14/2017	26	2156	4	8.8	0	37.56
2 multi	2	H						
45	1	01/15/2017	27	2157	4	8.6	0	33.84

2 multi	2	H						
45	1	01/16/2017	28	2158	4	8.7	0.26	35.11
2 multi	2	H						
45	1	01/17/2017	29	2159	4	8.9	0	36.75
2 multi	2	H						
45	1	01/18/2017	30	2160	4	8.7	0	36.09
2 multi	2	H						
45	1	01/19/2017	31	2161	4	8.9	0	36.64
2 multi	2	H						
45	1	01/20/2017	32	2162	4	8.7	0	35.47
2 multi	2	H						
45	1	01/21/2017	33	2163	4	9.1	0	36.24
2 multi	2	H						
45	1	01/22/2017	34	2164	4	9.2	0	37.46
2 multi	2	H						
45	1	01/23/2017	35	2165	4	8.7	0	39.78
2 multi	2	H						
45	1	01/24/2017	36	2166	4	8.9	0	40.32
2 multi	2	H						
45	1	01/25/2017	37	2167	4	8.9	0.07	38.51
2 multi	2	H						
45	1	01/12/2018	5	2519	5	9.8	0.53	29.6
2 multi	1	H						
45	1	01/13/2018	6	2520	5	10.3	0.01	27.21
2 multi	1	H						
45	1	01/14/2018	7	2521	5	9.8	0	26.03
2 multi	1	H						
45	1	01/15/2018	8	2522	5	9.3	0.07	31.07
2 multi	1	H						
45	1	01/16/2018	9	2523	5	9.9	0	31.95
2 multi	1	H						
45	1	01/17/2018	10	2524	5	9.5	0.06	30.92
2 multi	1	H						
45	1	01/18/2018	11	2525	5	9.6	0	32.38
2 multi	1	H						
45	1	01/19/2018	12	2526	5	9.6	0.01	29.21
2 multi	1	H						
45	1	01/20/2018	13	2527	5	9.5	0.02	30.33
2 multi	1	H						
45	1	01/21/2018	14	2528	5	9.2	0	30.83
2 multi	1	H						
45	1	01/22/2018	15	2529	5	9.6	0	33.2
2 multi	1	H						
45	1	01/23/2018	16	2530	5	9.5	0	34.18
2 multi	1	H						
45	1	01/24/2018	17	2531	5	9.3	0.06	34
2 multi	1	H						
45	1	01/25/2018	18	2532	5	9.5	0	35.94
2 multi	1	H						
45	1	01/26/2018	19	2533	5	8.9	0	35.34
2 multi	1	H						
45	1	01/27/2018	20	2534	5	9.2	0	38.79
2 multi	1	H						
45	1	01/28/2018	21	2535	5	9.5	0	39.8

2 multi 1		H						
45	1	01/29/2018	22	2536	5	9.1	0	34.51
2 multi 2		H						
45	1	01/30/2018	23	2537	5	9.1	0	34.44
2 multi 2		H						
45	1	01/31/2018	24	2538	5	9.1	0	36.14
2 multi 2		H						
45	1	02/01/2018	25	2539	5	9.1	0	40.19
2 multi 2		H						
45	1	02/02/2018	26	2540	5	9.1	0	36.05
2 multi 2		H						
45	1	02/03/2018	27	2541	5	9.2	0	40.85
2 multi 2		H						
45	1	02/04/2018	28	2542	5	9.3	0	41.89
2 multi 2		H						
45	1	02/05/2018	29	2543	5	9.3	0	38.67
2 multi 2		H						
45	1	02/06/2018	30	2544	5	8.9	0	31.34
2 multi 2		H						
45	1	02/07/2018	31	2545	5	9.1	0	37.72
2 multi 2		H						
45	1	02/08/2018	32	2546	5	9.6	0	39.62
2 multi 2		H						
45	1	02/09/2018	33	2547	5	9.5	0	42.12
2 multi 2		H						
45	1	02/10/2018	34	2548	5	9.2	0	36.44
2 multi 2		H						
45	1	02/11/2018	35	2549	5	9.5	0	38.83
2 multi 2		H						
45	1	02/12/2018	36	2550	5	8.8	0.35	36.69
2 multi 2		H						
45	1	02/13/2018	37	2551	5	9.2	0.15	37.63
2 multi 2		H						
45	1	02/14/2018	38	2552	5	8.9	0	35.58
2 multi 2		H						
45	1	02/15/2018	39	2553	5	9.2	0	41.68
2 multi 2		H						
45	1	02/16/2018	40	2554	5	9.5	0	41.33
2 multi 2		H						
45	1	02/17/2018	41	2555	5	8.9	0.01	37.97
2 multi 2		H						
45	1	02/18/2018	42	2556	5	8.9	0	36.51
2 multi 2		H						
45	1	02/19/2018	43	2557	5	9.1	0	37.77
2 multi 2		H						
45	1	02/20/2018	44	2558	5	9.1	0	44.56
2 multi 2		H						
45	1	02/21/2018	45	2559	5	9.5	0	45.88
2 multi 2		H						
45	1	02/22/2018	46	2560	5	9.6	0	31.97
2 multi 2		H						
45	1	02/23/2018	47	2561	5	8.9	0	40.19
2 multi 2		H						
45	1	02/24/2018	48	2562	5	9.2	0	38.45

2 multi	2	H						
45	1	02/25/2018	49	2563	5	9.5	0	38.18
2 multi	2	H						
45	1	02/26/2018	50	2564	5	9.3	0	42.51
2 multi	2	H						
45	1	02/27/2018	51	2565	5	9.5	0	47.28
2 multi	2	H						
45	1	02/28/2018	52	2566	5	9.1	0	38.3
2 multi	2	H						
45	1	03/01/2018	53	2567	5	8.9	0.01	39.6
2 multi	2	H						
45	1	03/02/2018	54	2568	5	9.2	0	40.01
2 multi	2	H						
45	1	03/03/2018	55	2569	5	9.1	0	39.74
2 multi	2	H						
45	1	03/04/2018	56	2570	5	9.1	0	39.45
2 multi	2	H						
45	1	03/05/2018	57	2571	5	8.7	0	38.31
2 multi	2	H						
45	1	03/06/2018	58	2572	5	8.9	0	41.94
2 multi	2	H						
45	1	03/07/2018	59	2573	5	9.2	0	41.11
2 multi	2	H						
45	1	03/08/2018	60	2574	5	8.9	0	35.33
2 multi	2	H						
45	1	03/09/2018	61	2575	5	9.1	0	44.99
2 multi	2	H						
45	1	03/10/2018	62	2576	5	9.1	0.11	38.16
2 multi	2	H						
45	1	03/11/2018	63	2577	5	9.3	0	38.16
2 multi	2	H						
45	1	12/12/2018	4	2853	6	9.2	0.47	19.88
2 multi	1	H						
45	1	12/13/2018	5	2854	6	9.2	0.46	19.97
2 multi	1	H						
45	1	12/14/2018	6	2855	6	9.2	0.08	23.51
2 multi	1	H						
45	1	12/15/2018	7	2856	6	9.2	0.02	25.83
2 multi	1	H						
45	1	12/16/2018	8	2857	6	9.1	0.19	26.52
2 multi	1	H						
45	1	12/17/2018	9	2858	6	9.5	0.54	26.7
2 multi	1	H						
45	1	12/18/2018	10	2859	6	9.1	0.09	29.73
2 multi	1	H						
45	1	12/19/2018	11	2860	6	8.9	0.02	28.3
2 multi	1	H						
45	1	12/20/2018	12	2861	6	8.9	0.01	29.97
2 multi	1	H						
45	1	12/21/2018	13	2862	6	8.9	0.21	31.24
2 multi	1	H						
45	1	12/22/2018	14	2863	6	8.6	0.02	30.99
2 multi	1	H						
45	1	12/23/2018	15	2864	6	8.8	0	34.45

2 multi	1	H						
45	1	12/24/2018	16	2865	6	8.8	0	33.7
2 multi	1	H						
45	1	12/25/2018	17	2866	6	8.9	0	35.07
2 multi	1	H						
45	1	12/26/2018	18	2867	6	8.9	0	31.83
2 multi	1	H						
45	1	12/27/2018	19	2868	6	8.8	0	34.92
2 multi	1	H						
45	1	12/28/2018	20	2869	6	9.2	0	34.36
2 multi	1	H						
45	1	12/29/2018	21	2870	6	9.2	0.01	34.88
2 multi	1	H						
45	1	12/30/2018	22	2871	6	8.7	0.01	33.26
2 multi	2	H						
45	1	12/31/2018	23	2872	6	8.9	0	33.41
2 multi	2	H						
45	1	01/01/2019	24	2873	6	8.9	0.31	33.6
2 multi	2	H						
45	1	01/02/2019	25	2874	6	8.9	0.11	33.75
2 multi	2	H						
45	1	01/03/2019	26	2875	6	8.9	0	33.85
2 multi	2	H						
45	1	01/04/2019	27	2876	6	8.9	0.02	35.1
2 multi	2	H						
45	1	01/05/2019	28	2877	6	8.9	0	30.26
2 multi	2	H						
45	1	01/06/2019	29	2878	6	9.1	0.59	34.8
2 multi	2	H						
45	1	01/07/2019	30	2879	6	9.1	0	36.43
2 multi	2	H						
45	1	01/08/2019	31	2880	6	8.8	0	33.5
2 multi	2	H						
45	1	12/11/2019	5	3217	7	8.9	0.21	22.27
2 multi	1	H						
45	1	12/12/2019	6	3218	7	9.3	0.05	25.38
2 multi	1	H						
45	1	12/13/2019	7	3219	7	9.1	0.08	23.35
2 multi	1	H						
45	1	12/14/2019	8	3220	7	9.6	0.22	26.19
2 multi	1	H						
45	1	12/15/2019	9	3221	7	9.1	0.24	26.19
2 multi	1	H						
45	1	12/16/2019	10	3222	7	9.8	0.24	29.54
2 multi	1	H						
45	1	12/17/2019	11	3223	7	9.2	0.08	28.75
2 multi	1	H						
45	1	12/18/2019	12	3224	7	8.7	0.03	28.6
2 multi	1	H						
45	1	12/19/2019	13	3225	7	8.9	0	28.12
2 multi	1	H						
45	1	12/20/2019	14	3226	7	8.8	0.05	30.58
2 multi	1	H						
45	1	12/21/2019	15	3227	7	8.8	0	29.97

2 multi	1	H						
45	1	12/22/2019	16	3228	7	8.8	0.04	31.24
2 multi	1	H						
45	1	12/23/2019	17	3229	7	9.1	0.05	24.97
2 multi	1	H						
45	1	12/24/2019	18	3230	7	8.6	0	20.23
2 multi	1	H						
45	1	12/25/2019	19	3231	7	8.8	0.08	23.25
2 multi	1	H						
45	1	12/26/2019	20	3232	7	8.4	0	29.3
2 multi	1	H						
45	1	12/27/2019	21	3233	7	8.2	0.03	27.18
2 multi	1	H						
45	1	12/28/2019	22	3234	7	8.7	0.03	28.99
2 multi	2	H						
45	1	12/29/2019	23	3235	7	8.9	0.02	30.17
2 multi	2	H						
45	1	12/30/2019	24	3236	7	8.7	0	31.79
2 multi	2	H						
45	1	12/31/2019	25	3237	7	8.8	0.02	29.79
2 multi	2	H						
45	1	01/01/2020	26	3238	7	8.8	0.05	29.95
2 multi	2	H						
45	1	01/02/2020	27	3239	7	8.6	0.17	29.32
2 multi	2	H						
45	1	01/03/2020	28	3240	7	8.9	0.19	30.41
2 multi	2	H						
45	1	01/04/2020	29	3241	7	9.6	0.06	30.42
2 multi	2	H						
45	1	01/05/2020	30	3242	7	9.2	0	27.11
2 multi	2	H						
45	1	01/06/2020	31	3243	7	8.7	0.53	27.34
2 multi	2	H						
45	1	01/07/2020	32	3244	7	8.9	0	31.35
2 multi	2	H						
45	1	01/08/2020	33	3245	7	9.1	0	33.55
2 multi	2	H						
45	1	01/09/2020	34	3246	7	8.9	0.01	28.15
2 multi	2	H						
45	1	01/10/2020	35	3247	7	8.7	0	25.21
2 multi	2	H						
45	1	01/11/2020	36	3248	7	9.1	0.25	31.78
2 multi	2	H						
45	1	01/12/2020	37	3249	7	8.6	0.22	30.88
2 multi	2	H						
45	1	01/13/2020	38	3250	7	8.5	0.23	31.54
2 multi	2	H						
45	1	01/14/2020	39	3251	7	8.7	0.22	31.38
2 multi	2	H						
45	1	01/15/2020	40	3252	7	8.7	0.07	34.08
2 multi	2	H						
45	1	01/16/2020	41	3253	7	8.7	0.07	30.99
2 multi	2	H						
45	1	01/17/2020	42	3254	7	8.9	0.13	33.76

2 multi	2	H						
45	1	01/18/2020	43	3255	7	8.7	0.15	32.39
2 multi	2	H						
45	1	01/19/2020	44	3256	7	8.8	0.08	33.19
2 multi	2	H						
45	1	01/20/2020	45	3257	7	8.8	0	35.29
2 multi	2	H						
45	1	01/21/2020	46	3258	7	8.7	0	38.36
2 multi	2	H						
45	1	01/22/2020	47	3259	7	8.7	0.02	34.32
2 multi	2	H						
45	1	01/23/2020	48	3260	7	8.8	0.03	30.48
2 multi	2	H						
45	1	12/08/2020	5	3580	8	9.2	0.26	24.07
2 multi	1	H						
45	1	12/09/2020	6	3581	8	9.2	0.74	26.18
2 multi	1	H						
45	1	12/10/2020	7	3582	8	9.2	0.62	27.6
2 multi	1	H						
45	1	12/11/2020	8	3583	8	9.1	0.51	28.25
2 multi	1	H						
45	1	12/12/2020	9	3584	8	8.9	0.09	29.19
2 multi	1	H						
45	1	12/13/2020	10	3585	8	8.8	0.25	30.03
2 multi	1	H						
45	1	12/14/2020	11	3586	8	8.8	0.13	31.16
2 multi	1	H						
45	1	12/15/2020	12	3587	8	8.8	0	31.89
2 multi	1	H						
45	1	12/16/2020	13	3588	8	8.9	0	34.18
2 multi	1	H						
45	1	12/17/2020	14	3589	8	8.9	0.08	31.65
2 multi	1	H						
45	1	12/18/2020	15	3590	8	8.8	0.18	31.62
2 multi	1	H						
45	1	12/19/2020	16	3591	8	8.6	0.03	32.28
2 multi	1	H						
45	1	12/20/2020	17	3592	8	8.7	0.09	36.09
2 multi	1	H						
45	1	12/21/2020	18	3593	8	8.7	0.04	32.07
2 multi	1	H						
45	1	12/22/2020	19	3594	8	8.7	0.07	34.98
2 multi	1	H						
45	1	12/23/2020	20	3595	8	9.1	0.01	34.86
2 multi	1	H						
45	1	12/24/2020	21	3596	8	8.6	0	32.4
2 multi	1	H						
45	1	12/25/2020	22	3597	8	8.8	0.05	37.07
2 multi	2	H						
45	1	12/26/2020	23	3598	8	8.8	0.06	34.49
2 multi	2	H						
45	1	12/27/2020	24	3599	8	8.8	0	34.61
2 multi	2	H						
45	1	12/28/2020	25	3600	8	8.9	0.01	37.92

2 multi 2		H						
45	1	12/29/2020	26	3601	8	8.9	0.09	37.36
2 multi 2		H						
45	1	12/30/2020	27	3602	8	9.2	0	33.17
2 multi 2		H						
45	1	12/31/2020	28	3603	8	9.1	0	35.05
2 multi 2		H						
45	1	01/01/2021	29	3604	8	8.8	0.05	42
2 multi 2		H						
45	1	01/02/2021	30	3605	8	9.1	0.12	37.58
2 multi 2		H						
45	1	01/03/2021	31	3606	8	8.9	0.08	40.89
2 multi 2		H						
45	1	01/04/2021	32	3607	8	8.6	0.06	38.35
2 multi 2		H						
45	1	01/05/2021	33	3608	8	8.9	0.07	37.32
2 multi 2		H						
45	1	01/06/2021	34	3609	8	9.1	0	39.11
2 multi 2		H						
45	1	01/07/2021	35	3610	8	9.1	0.01	39.26
2 multi 2		H						
45	1	01/08/2021	36	3611	8	8.9	0.03	39.49
2 multi 2		H						
45	1	01/09/2021	37	3612	8	8.8	0	37.75
2 multi 2		H						
45	1	01/10/2021	38	3613	8	8.9	0.07	36.55
2 multi 2		H						
45	1	01/11/2021	39	3614	8	8.9	0.01	39.85
2 multi 2		H						
45	1	01/12/2021	40	3615	8	8.8	0.06	40.04
2 multi 2		H						
45	1	01/13/2021	41	3616	8	8.8	0.02	40.02
2 multi 2		H						
45	1	01/14/2021	42	3617	8	8.9	0	36.58
2 multi 2		H						
45	1	01/15/2021	43	3618	8	8.7	0.08	39.33
2 multi 2		H						
45	1	01/16/2021	44	3619	8	9.1	0.13	38.98
2 multi 2		H						
45	1	01/17/2021	45	3620	8	8.9	0.07	41.79
2 multi 2		H						
45	1	01/18/2021	46	3621	8	9.2	0.06	37.52
2 multi 2		H						
45	1	01/19/2021	47	3622	8	9.2	0.06	38.28
2 multi 2		H						
45	1	01/20/2021	48	3623	8	9.1	0.04	38.47
2 multi 2		H						
45	1	01/21/2021	49	3624	8	8.8	0.09	36.72
2 multi 2		H						
45	1	01/22/2021	50	3625	8	9.1	0.06	39.09
2 multi 2		H						
45	1	01/23/2021	51	3626	8	9.1	0.05	38.6
2 multi 2		H						
45	1	01/24/2021	52	3627	8	9.1	0.13	38.61

2 multi	2	H						
45	1	01/25/2021	53	3628	8	9.2	0	35.74
2 multi	2	H						
45	1	01/26/2021	54	3629	8	9.1	0	39.14
2 multi	2	H						
45	1	01/27/2021	55	3630	8	8.9	0.06	39.05
2 multi	2	H						
45	1	01/28/2021	56	3631	8	9.3	0.05	41.52
2 multi	2	H						
45	1	01/29/2021	57	3632	8	9.3	0.06	37.6
2 multi	2	H						
45	1	01/30/2021	58	3633	8	9.1	0.07	38.15
2 multi	2	H						
45	1	01/31/2021	59	3634	8	9.1	0.04	41.07
2 multi	2	H						
45	1	02/01/2021	60	3635	8	9.1	0.01	40.69
2 multi	2	H						
45	1	02/02/2021	61	3636	8	9.1	0.04	40.84
2 multi	2	H						
45	1	02/03/2021	62	3637	8	9.2	0.05	38.41
2 multi	2	H						
45	1	02/04/2021	63	3638	8	9.1	0.06	38.39
2 multi	2	H						
45	1	02/05/2021	64	3639	8	9.2	0	39.71
2 multi	2	H						
45	1	02/06/2021	65	3640	8	9.3	0	41.96
2 multi	2	H						
45	1	02/07/2021	66	3641	8	9.3	0	27.59
2 multi	2	H						
45	1	02/08/2021	67	3642	8	9.2	0.07	38.37
2 multi	2	H						
45	1	02/09/2021	68	3643	8	9.2	0.05	38.11
2 multi	2	H						
45	1	02/10/2021	69	3644	8	9.2	0.06	36.19
2 multi	2	H						
45	1	02/11/2021	70	3645	8	9.1	0	35.05
2 multi	2	H						
45	1	02/12/2021	71	3646	8	9.3	0.05	34.21
2 multi	2	H						
45	1	02/13/2021	72	3647	8	8.9	0	32.83
2 multi	2	H						
45	1	02/14/2021	73	3648	8	9.1	0.05	32.93
2 multi	2	H						
45	1	02/15/2021	74	3649	8	9.1	0.03	33.86
2 multi	2	H						
45	1	02/16/2021	75	3650	8	9.2	0.02	33.74
2 multi	2	H						
45	1	02/17/2021	76	3651	8	9.5	0.01	36.26
2 multi	2	H						
45	1	02/18/2021	77	3652	8	9.3	0.04	36.96
2 multi	2	H						
45	1	02/19/2021	78	3653	8	9.5	0.04	36.35
2 multi	2	H						
45	1	02/20/2021	79	3654	8	9.5	0.03	33.32

2 multi	2	H						
45	1	02/21/2021	80	3655	8	9.3	0.02	36.92
2 multi	2	H						
46	1	03/12/2021	8	737	1	9.8	0.26	23.45
1 primi	1	C						
46	1	03/13/2021	9	738	1	10.7	0	25.36
1 primi	1	C						
46	1	03/14/2021	10	739	1	9.2	0	24.67
1 primi	1	C						
46	1	03/15/2021	11	740	1	9.9	0.05	25.16
1 primi	1	C						
46	1	03/16/2021	12	741	1	10	0.25	25.48
1 primi	1	C						
46	1	03/17/2021	13	742	1	10.7	0.24	27.24
1 primi	1	C						
46	1	03/18/2021	14	743	1	10	0.3	25.92
1 primi	1	C						
46	1	03/19/2021	15	744	1	10.3	0	25.89
1 primi	1	C						
46	1	03/20/2021	16	745	1	10.2	0.37	25.84
1 primi	1	C						
46	1	03/21/2021	17	746	1	10.4	0	25.21
1 primi	1	C						
46	1	03/22/2021	18	747	1	10.2	0.36	26.86
1 primi	1	C						
46	1	03/23/2021	19	748	1	9.5	0	22.68
1 primi	1	C						
46	1	03/24/2021	20	749	1	10	0.04	26.09
1 primi	1	C						
46	1	03/25/2021	21	750	1	10.3	0.19	25.65
1 primi	1	C						
46	1	03/26/2021	22	751	1	10.3	0.18	27.55
1 primi	2	C						
46	1	03/27/2021	23	752	1	10.4	0.24	28.52
1 primi	2	C						
46	1	03/28/2021	24	753	1	10	0.03	26.44
1 primi	2	C						
46	1	03/29/2021	25	754	1	9.6	0.05	28.6
1 primi	2	C						
46	1	03/30/2021	26	755	1	10.4	0	27.73
1 primi	2	C						
46	1	03/31/2021	27	756	1	10.3	0	28.17
1 primi	2	C						
46	1	04/01/2021	28	757	1	10.2	0.33	29.2
1 primi	2	C						
46	1	04/02/2021	29	758	1	10.2	0.14	27.54
1 primi	2	C						
46	1	04/03/2021	30	759	1	10.3	0.06	28.19
1 primi	2	C						
46	1	04/04/2021	31	760	1	10.6	0.07	28.09
1 primi	2	C						
46	1	04/05/2021	32	761	1	10.2	0.09	27.79
1 primi	2	C						
46	1	04/06/2021	33	762	1	10.2	0.05	28.07

1 primi 2		C						
46	1	04/07/2021	34	763	1	10.2	0	27.43
1 primi 2		C						
46	1	04/08/2021	35	764	1	9.9	0	26.56
1 primi 2		C						
46	1	04/09/2021	36	765	1	10.4	0	29.88
1 primi 2		C						
46	1	04/10/2021	37	766	1	10.2	0	29.86
1 primi 2		C						
46	1	04/11/2021	38	767	1	10.4	0.05	29.97
1 primi 2		C						
46	1	04/12/2021	39	768	1	10.4	0	29.31
1 primi 2		C						
46	1	04/13/2021	40	769	1	10	0.2	27.37
1 primi 2		C						
46	1	04/14/2021	41	770	1	9.9	0.25	27.8
1 primi 2		C						
46	1	04/15/2021	42	771	1	10.9	0.27	26.17
1 primi 2		C						
46	1	04/16/2021	43	772	1	10.3	0.28	28.46
1 primi 2		C						
46	1	04/17/2021	44	773	1	10.9	0	30.47
1 primi 2		C						
46	1	04/18/2021	45	774	1	10.7	0	27.83
1 primi 2		C						
46	1	04/19/2021	46	775	1	10.3	0	28.13
1 primi 2		C						
46	1	04/20/2021	47	776	1	10.6	0.11	27
1 primi 2		C						
46	1	04/21/2021	48	777	1	10.7	0.37	28.36
1 primi 2		C						
46	1	04/22/2021	49	778	1	10.4	0.24	28.57
1 primi 2		C						
46	1	04/23/2021	50	779	1	11.1	0.06	28.67
1 primi 2		C						
46	1	04/24/2021	51	780	1	10.6	0.09	28.59
1 primi 2		C						
46	1	04/25/2021	52	781	1	10.6	0	23.64
1 primi 2		C						
46	1	04/26/2021	53	782	1	10.6	0	22.23
1 primi 2		C						
46	1	04/27/2021	54	783	1	10.2	0	23.96
1 primi 2		C						
46	1	04/28/2021	55	784	1	10.6	0	16.37
1 primi 2		C						
46	1	04/29/2021	56	785	1	10.4	0	22.06
1 primi 2		C						
47	1	11/21/2019	7	758	1	9.2	0.26	20.7
1 primi 1		H						
47	1	11/22/2019	8	759	1	9.5	0.07	23.44
1 primi 1		H						
47	1	11/23/2019	9	760	1	9.2	0.19	22.67
1 primi 1		H						
47	1	11/24/2019	10	761	1	8.7	0.03	24.54

1 primi	1	H						
47	1	11/25/2019	11	762	1	8.6	0.05	24.67
1 primi	1	H						
47	1	11/26/2019	12	763	1	9.1	0.04	25.88
1 primi	1	H						
47	1	11/27/2019	13	764	1	9.3	0.13	26.78
1 primi	1	H						
47	1	11/28/2019	14	765	1	8.9	0.01	27.8
1 primi	1	H						
47	1	11/29/2019	15	766	1	8.9	0	27.31
1 primi	1	H						
47	1	11/30/2019	16	767	1	9.2	0.01	28.64
1 primi	1	H						
47	1	12/01/2019	17	768	1	9.1	0.01	28.78
1 primi	1	H						
47	1	12/02/2019	18	769	1	9.1	0	29.51
1 primi	1	H						
47	1	12/03/2019	19	770	1	8.9	0.01	29.74
1 primi	1	H						
47	1	12/04/2019	20	771	1	9.5	0.1	31.15
1 primi	1	H						
47	1	12/05/2019	21	772	1	9.5	0.08	31.02
1 primi	1	H						
47	1	12/06/2019	22	773	1	9.3	0.03	32.63
1 primi	2	H						
47	1	12/07/2019	23	774	1	9.3	0	32.98
1 primi	2	H						
47	1	12/08/2019	24	775	1	9.2	0.22	31.54
1 primi	2	H						
47	1	12/09/2019	25	776	1	9.3	0.02	32.96
1 primi	2	H						
47	1	12/10/2019	26	777	1	9.5	0	32.84
1 primi	2	H						
47	1	12/11/2019	27	778	1	9.2	0	32.71
1 primi	2	H						
47	1	12/12/2019	28	779	1	9.3	0	34.31
1 primi	2	H						
47	1	12/13/2019	29	780	1	9.3	0	33.43
1 primi	2	H						
47	1	12/14/2019	30	781	1	9.2	0	33.59
1 primi	2	H						
47	1	12/15/2019	31	782	1	9.3	0	34.66
1 primi	2	H						
47	1	12/16/2019	32	783	1	9.5	0.36	36.36
1 primi	2	H						
47	1	12/17/2019	33	784	1	9.3	0	34.04
1 primi	2	H						
47	1	12/18/2019	34	785	1	9.1	0.07	33.7
1 primi	2	H						
47	1	12/19/2019	35	786	1	9.2	0.31	33.18
1 primi	2	H						
47	1	12/20/2019	36	787	1	9.3	0.26	34.55
1 primi	2	H						
47	1	12/21/2019	37	788	1	9.5	0.05	32.28

1 primi 2 H
47 1 12/22/2019 38 789 1 9.3 0 33.56
1 primi 2 H
47 1 12/23/2019 39 790 1 9.1 0 35.03
1 primi 2 H
47 1 12/24/2019 40 791 1 9.6 0 35.6
1 primi 2 H
47 1 12/25/2019 41 792 1 9.2 0 33.49
1 primi 2 H
47 1 12/26/2019 42 793 1 9.3 0 35.47
1 primi 2 H
47 1 12/27/2019 43 794 1 9.2 0 32.69
1 primi 2 H
47 1 12/28/2019 44 795 1 9.3 0.06 34.04
1 primi 2 H
47 1 12/29/2019 45 796 1 9.5 0 35.31
1 primi 2 H
47 1 12/30/2019 46 797 1 9.3 0 37.15
1 primi 2 H
47 1 12/31/2019 47 798 1 9.5 0.06 35.1
1 primi 2 H
47 1 01/01/2020 48 799 1 9.3 0 35.14
1 primi 2 H
47 1 01/02/2020 49 800 1 9.1 0.02 34.14
1 primi 2 H
47 1 01/03/2020 50 801 1 9.1 0.05 31.93
1 primi 2 H
47 1 01/04/2020 51 802 1 9.3 0.06 33.96
1 primi 2 H
47 1 01/05/2020 52 803 1 9.3 0 33.64
1 primi 2 H
47 1 01/06/2020 53 804 1 9.5 0.14 33.17
1 primi 2 H
47 1 01/07/2020 54 805 1 9.3 0 29.81
1 primi 2 H
47 1 01/08/2020 55 806 1 9.5 0.05 35.6
1 primi 2 H
47 1 01/09/2020 56 807 1 9.6 0.18 33.09
1 primi 2 H
47 1 01/10/2020 57 808 1 9.5 0.2 33.38
1 primi 2 H
47 1 01/11/2020 58 809 1 9.8 0.07 33.18
1 primi 2 H
47 1 01/12/2020 59 810 1 9.9 0 35.72
1 primi 2 H
47 1 01/13/2020 60 811 1 9.6 0 35.73
1 primi 2 H
47 1 01/14/2020 61 812 1 9.6 0 35.88
1 primi 2 H
47 1 01/15/2020 62 813 1 9.6 0 36.74
1 primi 2 H
47 1 01/16/2020 63 814 1 9.6 0.07 34.02
1 primi 2 H
47 1 01/17/2020 64 815 1 9.6 0 35.77

1 primi 2 H
47 1 01/18/2020 65 816 1 9.6 0.06 35.07
1 primi 2 H
47 1 01/19/2020 66 817 1 9.3 0.52 34.18
1 primi 2 H
47 1 01/20/2020 67 818 1 9.3 0.13 36.13
1 primi 2 H
47 1 01/21/2020 68 819 1 9.6 0.25 34.58
1 primi 2 H
47 1 01/22/2020 69 820 1 9.6 0.06 35.97
1 primi 2 H
47 1 12/17/2020 7 1150 2 9.9 0.03 34.61
2 multi 1 H
47 1 12/18/2020 8 1151 2 10 0.04 34.98
2 multi 1 H
47 1 12/19/2020 9 1152 2 9.9 0.16 35.96
2 multi 1 H
47 1 12/20/2020 10 1153 2 10.3 0.01 37.26
2 multi 1 H
47 1 12/21/2020 11 1154 2 10.2 0.07 35.57
2 multi 1 H
47 1 12/22/2020 12 1155 2 10.3 0 39.21
2 multi 1 H
47 1 12/23/2020 13 1156 2 10 0.05 37.67
2 multi 1 H
47 1 12/24/2020 14 1157 2 10.3 0.12 38.09
2 multi 1 H
47 1 12/25/2020 15 1158 2 10 0.03 39.23
2 multi 1 H
47 1 12/26/2020 16 1159 2 10 0.07 39.96
2 multi 1 H
47 1 12/27/2020 17 1160 2 10.2 0 41.43
2 multi 1 H
47 1 12/28/2020 18 1161 2 10 0.03 40.42
2 multi 1 H
47 1 12/29/2020 19 1162 2 10.6 0.1 42.21
2 multi 1 H
47 1 12/30/2020 20 1163 2 10.4 0.04 42.35
2 multi 1 H
47 1 12/31/2020 21 1164 2 10.4 0.01 40.93
2 multi 1 H
47 1 01/01/2021 22 1165 2 10.9 0.11 44.08
2 multi 2 H
47 1 01/02/2021 23 1166 2 10.7 0.15 43.87
2 multi 2 H
47 1 01/03/2021 24 1167 2 10.6 0.32 43.61
2 multi 2 H
47 1 01/04/2021 25 1168 2 10.6 0.1 46.06
2 multi 2 H
47 1 01/05/2021 26 1169 2 10.4 0.34 44.75
2 multi 2 H
47 1 01/06/2021 27 1170 2 10.3 0.04 47.05
2 multi 2 H
47 1 01/07/2021 28 1171 2 10.4 0 47.99

2 multi	2	H						
47	1	01/08/2021	29	1172	2	10.6	0	42.74
2 multi	2	H						
47	1	01/09/2021	30	1173	2	10.6	0	48
2 multi	2	H						
47	1	01/10/2021	31	1174	2	10.6	0	47.27
2 multi	2	H						
47	1	01/11/2021	32	1175	2	10.6	0.12	44.32
2 multi	2	H						
47	1	01/12/2021	33	1176	2	10.7	0.14	46.2
2 multi	2	H						
47	1	01/13/2021	34	1177	2	10.9	0.17	47
2 multi	2	H						
47	1	01/14/2021	35	1178	2	11.1	0	45.92
2 multi	2	H						
47	1	01/15/2021	36	1179	2	10.6	0.26	42.38
2 multi	2	H						
47	1	01/16/2021	37	1180	2	10.6	0.01	46.4
2 multi	2	H						
47	1	01/17/2021	38	1181	2	10.6	0.06	45.85
2 multi	2	H						
47	1	01/18/2021	39	1182	2	11.4	0.2	32.65
2 multi	2	H						
47	1	01/19/2021	40	1183	2	10.6	0.09	40.85
2 multi	2	H						
47	1	01/20/2021	41	1184	2	10.7	0	41.87
2 multi	2	H						
47	1	01/21/2021	42	1185	2	10.7	0	41.85
2 multi	2	H						
47	1	01/22/2021	43	1186	2	10.3	0.09	43.27
2 multi	2	H						
47	1	01/23/2021	44	1187	2	10.3	0.08	42.69
2 multi	2	H						
47	1	01/24/2021	45	1188	2	10.4	0.03	44.12
2 multi	2	H						
47	1	01/25/2021	46	1189	2	10.4	0.01	49.11
2 multi	2	H						
47	1	01/26/2021	47	1190	2	10.6	0	44.86
2 multi	2	H						
47	1	01/27/2021	48	1191	2	11.1	0.1	45.13
2 multi	2	H						
47	1	01/28/2021	49	1192	2	11.1	0.09	47.18
2 multi	2	H						
47	1	01/29/2021	50	1193	2	10.6	0.11	49.47
2 multi	2	H						
47	1	01/30/2021	51	1194	2	11.4	0.24	44.7
2 multi	2	H						
47	1	01/31/2021	52	1195	2	11.3	0	45.82
2 multi	2	H						
47	1	02/01/2021	53	1196	2	11.1	0.11	47.5
2 multi	2	H						
47	1	02/02/2021	54	1197	2	11.1	0.02	46.05
2 multi	2	H						
47	1	02/03/2021	55	1198	2	11.4	0	45.36

2 multi 2		H						
47	1	02/04/2021	56	1199	2	11.4	0.42	51.08
2 multi 2		H						
47	1	02/05/2021	57	1200	2	12	0	47.14
2 multi 2		H						
47	1	02/06/2021	58	1201	2	12.6	0	47.57
2 multi 2		H						
47	1	02/07/2021	59	1202	2	11.7	0.01	44.3
2 multi 2		H						
47	1	02/08/2021	60	1203	2	11.9	0.37	45.48
2 multi 2		H						
47	1	02/09/2021	61	1204	2	10.9	0.03	44.97
2 multi 2		H						
47	1	02/10/2021	62	1205	2	11.4	0.08	45.15
2 multi 2		H						
47	1	02/11/2021	63	1206	2	10.7	0.1	49.79
2 multi 2		H						
47	1	02/12/2021	64	1207	2	11.1	0.02	45.15
2 multi 2		H						
47	1	02/13/2021	65	1208	2	10.7	0.21	44.93
2 multi 2		H						
47	1	02/14/2021	66	1209	2	10.7	0	46.43
2 multi 2		H						
47	1	02/15/2021	67	1210	2	10.6	0.23	45.04
2 multi 2		H						
47	1	02/16/2021	68	1211	2	10.4	0.07	35.11
2 multi 2		H						
47	1	02/17/2021	69	1212	2	11.3	0.09	48.69
2 multi 2		H						
47	1	02/18/2021	70	1213	2	11.3	0.1	44.03
2 multi 2		H						
47	1	02/19/2021	71	1214	2	11.9	0.02	44.92
2 multi 2		H						
47	1	02/20/2021	72	1215	2	11.7	0.03	47.49
2 multi 2		H						
47	1	02/21/2021	73	1216	2	10.9	0	46.4
2 multi 2		H						
47	1	02/22/2021	74	1217	2	11.3	0.28	44.29
2 multi 2		H						
47	1	02/23/2021	75	1218	2	10.9	0	46.09
2 multi 2		H						
47	1	02/24/2021	76	1219	2	11.1	0	45.07
2 multi 2		H						
47	1	02/25/2021	77	1220	2	11.1	0.13	49.05
2 multi 2		H						
47	1	02/26/2021	78	1221	2	10.7	0.1	46.16
2 multi 2		H						
47	1	02/27/2021	79	1222	2	11.1	0	45.44
2 multi 2		H						
47	1	02/28/2021	80	1223	2	10.7	0.2	48.12
2 multi 2		H						
47	1	03/01/2021	81	1224	2	10.9	0.1	46.75
2 multi 2		H						
47	1	03/02/2021	82	1225	2	10.9	0.1	46.48

2 multi 2		H						
47	1	03/03/2021	83	1226	2	11.3	0	46.01
2 multi 2		H						
47	1	03/04/2021	84	1227	2	10.6	0.08	42.5
2 multi 2		H						
47	1	03/05/2021	85	1228	2	11.1	0	48.88
2 multi 2		H						
47	1	03/06/2021	86	1229	2	11.4	0	47.52
2 multi 2		H						
47	1	03/07/2021	87	1230	2	11.4	0	44.89
2 multi 2		H						
47	1	03/08/2021	88	1231	2	10.9	0	45.53
2 multi 2		H						
47	1	03/09/2021	89	1232	2	11.4	0.11	43.11
2 multi 2		H						
47	1	03/10/2021	90	1233	2	11.1	0.09	45.95
2 multi 2		H						
47	1	03/11/2021	91	1234	2	11.4	0	42.7
2 multi 2		H						
47	1	03/12/2021	92	1235	2	11.3	0	47.6
2 multi 2		H						
47	1	03/13/2021	93	1236	2	11.3	0	45.06
2 multi 2		H						
47	1	03/14/2021	94	1237	2	11.7	0	46.45
2 multi 2		H						
47	1	03/15/2021	95	1238	2	11.4	0.02	45.71
2 multi 2		H						
47	1	03/16/2021	96	1239	2	11.3	0.02	46.8
2 multi 2		H						
47	1	03/17/2021	97	1240	2	11.7	0.02	42.28
2 multi 2		H						
47	1	03/18/2021	98	1241	2	11.1	0.01	44.53
2 multi 2		H						
47	1	03/19/2021	99	1242	2	11.1	0	41.34
2 multi 2		H						
47	1	03/20/2021	100	1243	2	11.3	0.01	44.79
2 multi 2		H						
47	1	03/21/2021	101	1244	2	11.1	0.08	46.42
2 multi 2		H						
47	1	03/22/2021	102	1245	2	11.1	0.01	46.16
2 multi 2		H						
47	1	03/23/2021	103	1246	2	11.3	0	44.55
2 multi 2		H						
47	1	03/24/2021	104	1247	2	11.7	0	45.96
2 multi 2		H						
47	1	03/25/2021	105	1248	2	11.4	0.09	46.46
2 multi 2		H						
47	1	03/26/2021	106	1249	2	11.4	0.08	45.78
2 multi 2		H						
47	1	03/27/2021	107	1250	2	11.3	0.1	44.98
2 multi 2		H						
47	1	03/28/2021	108	1251	2	11.1	0.02	43.35
2 multi 2		H						
47	1	03/29/2021	109	1252	2	10.6	0	48.22

2 multi	2	H						
47	1	03/30/2021	110	1253	2	11.7	0.02	47.84
2 multi	2	H						
47	1	03/31/2021	111	1254	2	10.9	0.08	46.3
2 multi	2	H						
47	1	04/01/2021	112	1255	2	11.3	0	46.47
2 multi	2	H						
47	1	04/02/2021	113	1256	2	10.9	0.09	42.66
2 multi	2	H						
47	1	04/03/2021	114	1257	2	11.7	0	44.23
2 multi	2	H						
47	1	04/04/2021	115	1258	2	11.4	0	42.46
2 multi	2	H						
47	1	04/05/2021	116	1259	2	11.3	0.09	41.65
2 multi	2	H						
47	1	04/06/2021	117	1260	2	11.4	0.08	43.69
2 multi	2	H						
47	1	04/07/2021	118	1261	2	11.4	0	44.64
2 multi	2	H						
47	1	04/08/2021	119	1262	2	10.9	0.09	43.19
2 multi	2	H						
47	1	04/09/2021	120	1263	2	10.9	0.17	41.21
2 multi	2	H						
47	1	04/10/2021	121	1264	2	10.6	0.24	48.61
2 multi	2	H						
47	1	04/11/2021	122	1265	2	11.1	0	44.22
2 multi	2	H						
47	1	04/12/2021	123	1266	2	11.3	0	45.56
2 multi	2	H						
47	1	04/13/2021	124	1267	2	11.1	0.09	42.68
2 multi	2	H						
47	1	04/14/2021	125	1268	2	10.4	0.08	45.78
2 multi	2	H						
47	1	04/15/2021	126	1269	2	10.9	0	41.23
2 multi	2	H						
47	1	04/16/2021	127	1270	2	11.1	0.09	46.09
2 multi	2	H						
47	1	04/17/2021	128	1271	2	11.7	0.01	44.49
2 multi	2	H						
47	1	04/18/2021	129	1272	2	10.9	0.02	44.68
2 multi	2	H						
47	1	04/19/2021	130	1273	2	11.1	0.28	45.31
2 multi	2	H						
47	1	04/20/2021	131	1274	2	10.9	0.25	42.25
2 multi	2	H						
47	1	04/21/2021	132	1275	2	11.1	0.01	46.03
2 multi	2	H						
47	1	04/22/2021	133	1276	2	11.3	0.02	45.14
2 multi	2	H						
47	1	04/23/2021	134	1277	2	10.9	0.14	46.63
2 multi	2	H						
47	1	04/24/2021	135	1278	2	11.4	0.09	43.12
2 multi	2	H						
47	1	04/25/2021	136	1279	2	10.9	0.07	46.47

2 multi 2		H						
47	1	04/26/2021	137	1280	2	11.3	0	45.7
2 multi 2		H						
47	1	04/27/2021	138	1281	2	10.9	0	43.52
2 multi 2		H						
47	1	04/28/2021	139	1282	2	10.9	0	45.84
2 multi 2		H						
47	1	04/29/2021	140	1283	2	11.1	0.01	45.51
2 multi 2		H						
47	1	04/30/2021	141	1284	2	11.4	0.03	46.28
2 multi 2		H						
47	1	05/01/2021	142	1285	2	11.7	0.03	42.93
2 multi 2		H						
47	1	05/02/2021	143	1286	2	11.1	0	40.87
2 multi 2		H						
47	1	05/03/2021	144	1287	2	11.1	0.04	42.11
2 multi 2		H						
47	1	05/04/2021	145	1288	2	11.3	0.03	43.84
2 multi 2		H						
47	1	05/05/2021	146	1289	2	10.9	0	42.02
2 multi 2		H						
47	1	05/06/2021	147	1290	2	11.1	0.1	42.82
2 multi 2		H						
48	1	07/25/2018	5	750	1	9.3	0.15	17.71
1 primi 1		H						
48	1	07/26/2018	6	751	1	9.8	0.07	16.86
1 primi 1		H						
48	1	07/27/2018	7	752	1	9.6	0	18.85
1 primi 1		H						
48	1	07/28/2018	8	753	1	9.5	0.04	19.98
1 primi 1		H						
48	1	07/29/2018	9	754	1	9.6	0.16	19.76
1 primi 1		H						
48	1	07/30/2018	10	755	1	9.2	0.04	19.53
1 primi 1		H						
48	1	07/31/2018	11	756	1	10	0.01	19.92
1 primi 1		H						
48	1	08/01/2018	12	757	1	9.6	0.01	21.18
1 primi 1		H						
48	1	08/02/2018	13	758	1	9.6	0.01	20.93
1 primi 1		H						
48	1	08/03/2018	14	759	1	9.6	0.07	22.62
1 primi 1		H						
48	1	08/04/2018	15	760	1	9.8	0	22.27
1 primi 1		H						
48	1	08/05/2018	16	761	1	9.5	0	21.82
1 primi 1		H						
48	1	08/06/2018	17	762	1	9.5	0	22.57
1 primi 1		H						
48	1	08/07/2018	18	763	1	9.3	0	23
1 primi 1		H						
48	1	08/08/2018	19	764	1	9.1	0	23.5
1 primi 1		H						
48	1	08/09/2018	20	765	1	8.7	0	23.74

1 primi 1 H
48 1 08/10/2018 21 766 1 8.7 0.21 23.93
1 primi 1 H
48 1 08/11/2018 22 767 1 8.9 0.22 24.11
1 primi 2 H
48 1 08/12/2018 23 768 1 8.9 0 25.17
1 primi 2 H
48 1 08/13/2018 24 769 1 8.9 0 24.33
1 primi 2 H
48 1 08/14/2018 25 770 1 9.1 0 24.51
1 primi 2 H
48 1 08/15/2018 26 771 1 8.8 0 24.96
1 primi 2 H
48 1 08/16/2018 27 772 1 9.3 0.06 24.79
1 primi 2 H
48 1 08/17/2018 28 773 1 8.7 0 22.59
1 primi 2 H
48 1 08/18/2018 29 774 1 8.8 0.24 24.93
1 primi 2 H
48 1 08/19/2018 30 775 1 8.8 0 24.21
1 primi 2 H
48 1 08/20/2018 31 776 1 8.7 0.2 23.8
1 primi 2 H
48 1 08/21/2018 32 777 1 8.6 0.06 24.27
1 primi 2 H
48 1 08/22/2018 33 778 1 9.1 0.19 25.48
1 primi 2 H
48 1 08/23/2018 34 779 1 9.5 0.16 24.37
1 primi 2 H
48 1 08/24/2018 35 780 1 8.9 0 24.08
1 primi 2 H
48 1 08/25/2018 36 781 1 8.7 0 24.68
1 primi 2 H
48 1 08/26/2018 37 782 1 8.8 0.38 25.67
1 primi 2 H
48 1 08/27/2018 38 783 1 8.9 0.31 24.88
1 primi 2 H
48 1 08/28/2018 39 784 1 8.9 0.05 26.21
1 primi 2 H
48 1 08/29/2018 40 785 1 8.9 0.23 25.94
1 primi 2 H
48 1 08/30/2018 41 786 1 8.8 0.3 27.44
1 primi 2 H
48 1 08/31/2018 42 787 1 8.9 0.05 25.96
1 primi 2 H
48 1 09/01/2018 43 788 1 8.8 0.31 28.36
1 primi 2 H
48 1 09/02/2018 44 789 1 8.8 0.25 27.02
1 primi 2 H
48 1 08/19/2019 7 1140 2 9.5 0.3 28.29
2 multi 1 H
48 1 08/20/2019 8 1141 2 9.5 0.06 29.66
2 multi 1 H
48 1 08/21/2019 9 1142 2 9.8 0.04 28.45

2 multi	1	H						
48	1	08/22/2019	10	1143	2	9.6	0.19	26.59
2 multi	1	H						
48	1	08/23/2019	11	1144	2	9.3	0.06	31.47
2 multi	1	H						
48	1	08/24/2019	12	1145	2	9.5	0.16	29.5
2 multi	1	H						
48	1	08/25/2019	13	1146	2	9.3	0	31.3
2 multi	1	H						
48	1	08/26/2019	14	1147	2	9.6	0.19	31.54
2 multi	1	H						
48	1	08/27/2019	15	1148	2	9.3	0.02	31.39
2 multi	1	H						
48	1	08/28/2019	16	1149	2	9.5	0.01	32.03
2 multi	1	H						
48	1	08/29/2019	17	1150	2	9.9	0.01	31.82
2 multi	1	H						
48	1	08/30/2019	18	1151	2	9.6	0.04	31
2 multi	1	H						
48	1	08/31/2019	19	1152	2	9.6	0	32.64
2 multi	1	H						
48	1	09/01/2019	20	1153	2	9.5	0.09	32.31
2 multi	1	H						
48	1	09/02/2019	21	1154	2	9.8	0.21	31.49
2 multi	1	H						
48	1	09/03/2019	22	1155	2	9.5	0.2	32.79
2 multi	2	H						
48	1	09/04/2019	23	1156	2	9.3	0.4	32.92
2 multi	2	H						
48	1	09/05/2019	24	1157	2	9.6	0.03	34.78
2 multi	2	H						
48	1	09/06/2019	25	1158	2	9.6	0.05	30.52
2 multi	2	H						
48	1	09/07/2019	26	1159	2	9.5	0.19	32.01
2 multi	2	H						
48	1	09/08/2019	27	1160	2	9.3	0.02	33.64
2 multi	2	H						
48	1	09/09/2019	28	1161	2	8.8	0.45	30.44
2 multi	2	H						
48	1	09/10/2019	29	1162	2	8.8	0.25	32.45
2 multi	2	H						
48	1	09/11/2019	30	1163	2	9.2	0.22	33.14
2 multi	2	H						
48	1	09/12/2019	31	1164	2	8.9	0.36	31.59
2 multi	2	H						
48	1	09/07/2020	4	1525	3	9.2	0.22	29.64
2 multi	1	H						
48	1	09/08/2020	5	1526	3	9.5	0.35	30.84
2 multi	1	H						
48	1	09/09/2020	6	1527	3	9.2	0.07	32.03
2 multi	1	H						
48	1	09/10/2020	7	1528	3	9.5	0.34	35.33
2 multi	1	H						
48	1	09/11/2020	8	1529	3	9.3	0.06	33.9

2 multi	1	H						
48	1	09/12/2020	9	1530	3	9.3	0.17	36.96
2 multi	1	H						
48	1	09/13/2020	10	1531	3	9.2	0.06	36.92
2 multi	1	H						
48	1	09/14/2020	11	1532	3	9.6	0.06	37.45
2 multi	1	H						
48	1	09/15/2020	12	1533	3	9.2	0.1	38.93
2 multi	1	H						
48	1	09/16/2020	13	1534	3	9.5	0.03	40.55
2 multi	1	H						
48	1	09/17/2020	14	1535	3	9.3	0.11	39.82
2 multi	1	H						
48	1	09/18/2020	15	1536	3	9.2	0.06	40.77
2 multi	1	H						
48	1	09/19/2020	16	1537	3	9.2	0.05	40.05
2 multi	1	H						
48	1	09/20/2020	17	1538	3	9.3	0.03	40.15
2 multi	1	H						
48	1	09/21/2020	18	1539	3	9.1	0.03	42.77
2 multi	1	H						
48	1	09/22/2020	19	1540	3	9.5	0.05	41.67
2 multi	1	H						
48	1	09/23/2020	20	1541	3	8.9	0	42.91
2 multi	1	H						
48	1	09/24/2020	21	1542	3	9.3	0.04	42.08
2 multi	1	H						
48	1	09/25/2020	22	1543	3	9.2	0	39.61
2 multi	2	H						
48	1	09/26/2020	23	1544	3	9.3	0.05	41.69
2 multi	2	H						
48	1	09/27/2020	24	1545	3	9.1	0.63	44.92
2 multi	2	H						
48	1	09/28/2020	25	1546	3	9.1	0.07	40.86
2 multi	2	H						
48	1	09/29/2020	26	1547	3	9.3	0	43.88
2 multi	2	H						
48	1	09/30/2020	27	1548	3	9.2	0.23	40.9
2 multi	2	H						
48	1	10/01/2020	28	1549	3	9.2	0.37	43.03
2 multi	2	H						
48	1	10/02/2020	29	1550	3	9.1	0.09	42.65
2 multi	2	H						
48	1	10/03/2020	30	1551	3	9.2	0.09	41.69
2 multi	2	H						
48	1	10/04/2020	31	1552	3	9.8	0	38.41
2 multi	2	H						
48	1	10/05/2020	32	1553	3	8.7	0.09	41.37
2 multi	2	H						
48	1	10/06/2020	33	1554	3	9.2	0.1	38.71
2 multi	2	H						
48	1	10/07/2020	34	1555	3	8.8	0.01	41.02
2 multi	2	H						
48	1	10/08/2020	35	1556	3	9.2	0.06	39.04

2 multi	2	H						
48	1	10/09/2020	36	1557	3	9.1	0	39.97
2 multi	2	H						
48	1	10/10/2020	37	1558	3	9.2	0.08	40.82
2 multi	2	H						
48	1	10/11/2020	38	1559	3	9.1	0.26	35.79
2 multi	2	H						
48	1	10/12/2020	39	1560	3	9.1	0.33	40.57
2 multi	2	H						
48	1	10/13/2020	40	1561	3	9.3	0.39	38.62
2 multi	2	H						
50	1	05/06/2019	5	659	1	8.9	0.18	28.23
1 primi	1	H						
50	1	05/07/2019	6	660	1	9.3	0	27.01
1 primi	1	H						
50	1	05/08/2019	7	661	1	9.1	0.83	27.37
1 primi	1	H						
50	1	05/09/2019	8	662	1	8.8	0.16	28.56
1 primi	1	H						
50	1	05/10/2019	9	663	1	8.6	0.11	30.33
1 primi	1	H						
50	1	05/11/2019	10	664	1	8.5	0.05	26.94
1 primi	1	H						
50	1	05/12/2019	11	665	1	8.7	0.09	30.64
1 primi	1	H						
50	1	05/13/2019	12	666	1	8.3	0.08	31.54
1 primi	1	H						
50	1	05/14/2019	13	667	1	8.1	0.25	30.57
1 primi	1	H						
50	1	05/15/2019	14	668	1	8.1	0.22	29.3
1 primi	1	H						
50	1	05/16/2019	15	669	1	8.3	0.25	32.28
1 primi	1	H						
50	1	05/17/2019	16	670	1	8.5	0.23	30.91
1 primi	1	H						
50	1	05/18/2019	17	671	1	8.5	0.28	32.53
1 primi	1	H						
50	1	05/19/2019	18	672	1	11.4	0.22	52.13
1 primi	1	H						
50	1	05/20/2019	19	673	1	8.3	0.08	34.35
1 primi	1	H						
50	1	05/21/2019	20	674	1	8.3	0.12	33.48
1 primi	1	H						
50	1	05/22/2019	21	675	1	8.4	0.18	35.48
1 primi	1	H						
50	1	05/23/2019	22	676	1	8.4	0.18	35.94
1 primi	2	H						
50	1	05/24/2019	23	677	1	8.2	0.21	34.09
1 primi	2	H						
50	1	05/25/2019	24	678	1	8.9	0.23	34.36
1 primi	2	H						
50	1	05/26/2019	25	679	1	8.4	0.27	33.08
1 primi	2	H						
50	1	05/27/2019	26	680	1	8.4	0.21	35.08

1 primi	2	H						
50	1	05/28/2019	27	681	1	8.3	0.28	35.73
1 primi	2	H						
50	1	05/29/2019	28	682	1	8.4	0.28	36.32
1 primi	2	H						
50	1	05/30/2019	29	683	1	8.4	0.31	34.88
1 primi	2	H						
50	1	05/31/2019	30	684	1	8.2	0.03	34.93
1 primi	2	H						
50	1	06/01/2019	31	685	1	8.5	0	36.39
1 primi	2	H						
50	1	06/02/2019	32	686	1	8.2	0.1	34.59
1 primi	2	H						
50	1	06/03/2019	33	687	1	8.6	0.12	36.53
1 primi	2	H						
50	1	06/04/2019	34	688	1	8.5	0.15	34.85
1 primi	2	H						
50	1	06/05/2019	35	689	1	8.4	0.25	36.35
1 primi	2	H						
50	1	06/06/2019	36	690	1	8.7	0	35.79
1 primi	2	H						
50	1	06/07/2019	37	691	1	8.7	0	35.83
1 primi	2	H						
50	1	06/08/2019	38	692	1	8.3	0.09	35.21
1 primi	2	H						
50	1	06/09/2019	39	693	1	8.9	0.1	38.44
1 primi	2	H						
50	1	06/10/2019	40	694	1	8.9	0	38.41
1 primi	2	H						
50	1	06/11/2019	41	695	1	8.7	0.22	36.75
1 primi	2	H						
50	1	06/12/2019	42	696	1	8.6	0.24	35.36
1 primi	2	H						
50	1	06/13/2019	43	697	1	8.4	0.06	35.94
1 primi	2	H						
50	1	06/14/2019	44	698	1	8.6	0.14	37.11
1 primi	2	H						
50	1	06/15/2019	45	699	1	8.7	0.1	38.39
1 primi	2	H						
50	1	06/16/2019	46	700	1	8.8	0.28	38.12
1 primi	2	H						
50	1	06/17/2019	47	701	1	8.7	0	36.77
1 primi	2	H						
50	1	06/18/2019	48	702	1	8.8	0	35.79
1 primi	2	H						
50	1	06/19/2019	49	703	1	9.1	0	34.92
1 primi	2	H						
50	1	06/20/2019	50	704	1	8.8	0	35.94
1 primi	2	H						
50	1	06/21/2019	51	705	1	8.9	0	36.22
1 primi	2	H						
50	1	06/22/2019	52	706	1	8.8	0.2	35.06
1 primi	2	H						
50	1	06/23/2019	53	707	1	8.9	0	38.43

1 primi	2	H						
50	1	06/24/2019	54	708	1	8.7	0	36.76
1 primi	2	H						
50	1	06/25/2019	55	709	1	8.7	0.09	38.78
1 primi	2	H						
50	1	06/26/2019	56	710	1	9.1	0	39.39
1 primi	2	H						
50	1	06/27/2019	57	711	1	9.3	0	38.86
1 primi	2	H						
50	1	06/28/2019	58	712	1	8.8	0.25	34.99
1 primi	2	H						
50	1	06/29/2019	59	713	1	8.6	0	34.89
1 primi	2	H						
50	1	06/30/2019	60	714	1	8.7	0.13	35.64
1 primi	2	H						
50	1	07/01/2019	61	715	1	8.9	0	38.52
1 primi	2	H						
50	1	07/02/2019	62	716	1	8.7	0.08	38.46
1 primi	2	H						
50	1	07/03/2019	63	717	1	9.1	0	35.8
1 primi	2	H						
50	1	07/04/2019	64	718	1	8.8	0.02	37.08
1 primi	2	H						
50	1	07/05/2019	65	719	1	8.9	0.07	38.07
1 primi	2	H						
50	1	07/06/2019	66	720	1	8.7	0	34.27
1 primi	2	H						
50	1	07/07/2019	67	721	1	9.2	0.08	35.11
1 primi	2	H						
50	1	07/08/2019	68	722	1	8.5	0	35.6
1 primi	2	H						
50	1	07/09/2019	69	723	1	8.8	0	36.21
1 primi	2	H						
50	1	07/10/2019	70	724	1	9.3	0.04	35.51
1 primi	2	H						
50	1	07/11/2019	71	725	1	8.8	0	34.89
1 primi	2	H						
50	1	07/12/2019	72	726	1	8.5	0.21	34.65
1 primi	2	H						
50	1	07/13/2019	73	727	1	8.8	0	35.82
1 primi	2	H						
50	1	07/14/2019	74	728	1	9.2	0	36.95
1 primi	2	H						
50	1	07/15/2019	75	729	1	8.7	0.01	35.67
1 primi	2	H						
50	1	07/16/2019	76	730	1	8.6	0	37.63
1 primi	2	H						
50	1	07/17/2019	77	731	1	8.8	0.07	36.2
1 primi	2	H						
50	1	07/18/2019	78	732	1	9.1	0	37.54
1 primi	2	H						
50	1	07/19/2019	79	733	1	8.8	0	33.63
1 primi	2	H						
50	1	07/20/2019	80	734	1	8.7	0.05	36.28

1 primi	2	H						
50	1	07/21/2019	81	735	1	9.1	0	37.65
1 primi	2	H						
50	1	07/22/2019	82	736	1	9.9	0	34.67
1 primi	2	H						
50	1	07/23/2019	83	737	1	8.8	0.2	35.72
1 primi	2	H						
50	1	07/24/2019	84	738	1	8.8	0.48	40.13
1 primi	2	H						
50	1	07/25/2019	85	739	1	8.9	0.01	36.26
1 primi	2	H						
50	1	07/26/2019	86	740	1	8.9	0.09	34.03
1 primi	2	H						
50	1	07/27/2019	87	741	1	8.8	0	34.77
1 primi	2	H						
50	1	07/28/2019	88	742	1	8.5	0.02	34
1 primi	2	H						
50	1	07/29/2019	89	743	1	8.5	0	36.53
1 primi	2	H						
50	1	07/30/2019	90	744	1	8.9	0	35.63
1 primi	2	H						
50	1	07/31/2019	91	745	1	8.9	0.02	36.22
1 primi	2	H						
50	1	08/01/2019	92	746	1	9.2	0.21	33.43
1 primi	2	H						
50	1	08/02/2019	93	747	1	9.1	0.02	36.57
1 primi	2	H						
50	1	08/03/2019	94	748	1	8.8	0.02	36.08
1 primi	2	H						
50	1	08/04/2019	95	749	1	8.9	0	35.58
1 primi	2	H						
50	1	08/05/2019	96	750	1	9.1	0	37.31
1 primi	2	H						
50	1	08/06/2019	97	751	1	8.9	0	35.1
1 primi	2	H						
50	1	08/07/2019	98	752	1	9.1	0	35.66
1 primi	2	H						
50	1	08/08/2019	99	753	1	9.1	0	35.04
1 primi	2	H						
50	1	08/09/2019	100	754	1	8.7	0	33.42
1 primi	2	H						
50	1	08/10/2019	101	755	1	8.8	0.05	33.74
1 primi	2	H						
50	1	08/11/2019	102	756	1	8.9	0	34.25
1 primi	2	H						
50	1	07/06/2020	4	1086	2	10.2	0.41	32.42
2 multi	1	H						
50	1	07/07/2020	5	1087	2	9.5	0.3	30.97
2 multi	1	H						
50	1	07/08/2020	6	1088	2	9.8	0.13	33.46
2 multi	1	H						
50	1	07/09/2020	7	1089	2	10.2	0.11	34.44
2 multi	1	H						
50	1	07/10/2020	8	1090	2	10.6	0.32	33.58

2 multi	1	H						
50	1	07/11/2020	9	1091	2	10.2	0.11	37.24
2 multi	1	H						
50	1	07/12/2020	10	1092	2	9.5	0.49	39.24
2 multi	1	H						
50	1	07/13/2020	11	1093	2	9.5	0.14	39.1
2 multi	1	H						
50	1	07/14/2020	12	1094	2	9.2	0	37.65
2 multi	1	H						
50	1	07/15/2020	13	1095	2	8.9	0.2	36.8
2 multi	1	H						
50	1	07/16/2020	14	1096	2	8.9	0.26	38.45
2 multi	1	H						
50	1	07/17/2020	15	1097	2	9.3	0.42	41.81
2 multi	1	H						
50	1	07/18/2020	16	1098	2	9.5	0.24	43.24
2 multi	1	H						
50	1	07/19/2020	17	1099	2	9.3	0.08	42
2 multi	1	H						
50	1	07/20/2020	18	1100	2	8.9	0.09	42.05
2 multi	1	H						
50	1	07/21/2020	19	1101	2	9.2	0.11	43.2
2 multi	1	H						
50	1	07/22/2020	20	1102	2	9.1	0.11	44.71
2 multi	1	H						
50	1	07/23/2020	21	1103	2	8.8	0.24	42.95
2 multi	1	H						
50	1	07/24/2020	22	1104	2	8.9	0.08	43.34
2 multi	2	H						
50	1	07/25/2020	23	1105	2	9.2	0.11	41.33
2 multi	2	H						
50	1	07/26/2020	24	1106	2	8.9	0.16	41.71
2 multi	2	H						
50	1	07/27/2020	25	1107	2	9.1	0	45.12
2 multi	2	H						
50	1	07/28/2020	26	1108	2	8.8	0.09	38.79
2 multi	2	H						
50	1	07/29/2020	27	1109	2	9.1	0.15	34.93
2 multi	2	H						
50	1	07/30/2020	28	1110	2	9.1	0.19	40.05
2 multi	2	H						
50	1	07/31/2020	29	1111	2	9.6	0.22	44.73
2 multi	2	H						
50	1	08/01/2020	30	1112	2	9.6	0.35	45.9
2 multi	2	H						
50	1	08/02/2020	31	1113	2	9.5	0	45.32
2 multi	2	H						
50	1	08/03/2020	32	1114	2	9.3	0.11	41.67
2 multi	2	H						
50	1	08/04/2020	33	1115	2	9.3	0.28	42.31
2 multi	2	H						
50	1	08/05/2020	34	1116	2	9.3	0	44.2
2 multi	2	H						
50	1	08/06/2020	35	1117	2	9.3	0.44	47.05

2 multi	2	H						
50	1	08/07/2020	36	1118	2	9.9	0.08	44.8
2 multi	2	H						
50	1	08/08/2020	37	1119	2	9.5	0.14	45.71
2 multi	2	H						
50	1	08/09/2020	38	1120	2	8.8	0.49	43.27
2 multi	2	H						
50	1	08/10/2020	39	1121	2	9.1	0.22	40.09
2 multi	2	H						
50	1	08/11/2020	40	1122	2	9.3	0.09	37.65
2 multi	2	H						
50	1	08/12/2020	41	1123	2	9.3	0.1	42.68
2 multi	2	H						
50	1	08/13/2020	42	1124	2	9.6	0	46.41
2 multi	2	H						
50	1	08/14/2020	43	1125	2	9.6	0.17	43.32
2 multi	2	H						
50	1	08/15/2020	44	1126	2	9.6	0.01	44.17
2 multi	2	H						
50	1	08/16/2020	45	1127	2	9.8	0	41.98
2 multi	2	H						
50	1	08/17/2020	46	1128	2	10.4	0.26	42.66
2 multi	2	H						
50	1	08/18/2020	47	1129	2	10	0	43.41
2 multi	2	H						
50	1	08/19/2020	48	1130	2	9.8	0	43.45
2 multi	2	H						
50	1	08/20/2020	49	1131	2	10	0.11	44.27
2 multi	2	H						
50	1	08/21/2020	50	1132	2	9.9	0.12	46.28
2 multi	2	H						
50	1	08/22/2020	51	1133	2	9.6	0.07	45.02
2 multi	2	H						
50	1	08/23/2020	52	1134	2	9.1	0.18	44.19
2 multi	2	H						
50	1	08/24/2020	53	1135	2	8.9	0.08	43.37
2 multi	2	H						
50	1	08/25/2020	54	1136	2	9.3	0	43.99
2 multi	2	H						
50	1	08/26/2020	55	1137	2	9.6	0.09	44.62
2 multi	2	H						
50	1	08/27/2020	56	1138	2	9.8	0.01	47.67
2 multi	2	H						
50	1	08/28/2020	57	1139	2	9.5	0	42.09
2 multi	2	H						
50	1	08/29/2020	58	1140	2	9.5	0.16	44.96
2 multi	2	H						
50	1	08/30/2020	59	1141	2	9.6	0	46.77
2 multi	2	H						
50	1	08/31/2020	60	1142	2	9.9	0.09	48.25
2 multi	2	H						
50	1	09/01/2020	61	1143	2	9.6	0	42.27
2 multi	2	H						
50	1	09/02/2020	62	1144	2	9.5	0.03	27.99

2 multi 2		H						
50	1	09/03/2020	63	1145	2	9.3	0	37.64
2 multi 2		H						
50	1	09/04/2020	64	1146	2	9.5	0.08	43.63
2 multi 2		H						
50	1	09/05/2020	65	1147	2	9.2	0	41.18
2 multi 2		H						
50	1	09/06/2020	66	1148	2	9.8	0	40.95
2 multi 2		H						
50	1	09/07/2020	67	1149	2	9.1	0	26.79
2 multi 2		H						
50	1	09/08/2020	68	1150	2	10	0.2	38.12
2 multi 2		H						
50	1	09/09/2020	69	1151	2	8.7	0.04	27.1
2 multi 2		H						
50	1	09/10/2020	70	1152	2	9.2	0.09	34.33
2 multi 2		H						
50	1	09/11/2020	71	1153	2	9.8	0	36.15
2 multi 2		H						
50	1	09/12/2020	72	1154	2	10	0.26	39.55
2 multi 2		H						
50	1	09/13/2020	73	1155	2	10.3	0.05	43.55
2 multi 2		H						
50	1	09/14/2020	74	1156	2	10.2	0.03	42.94
2 multi 2		H						
50	1	09/15/2020	75	1157	2	9.9	0.08	41.8
2 multi 2		H						
50	1	09/16/2020	76	1158	2	9.6	0.02	43.77
2 multi 2		H						
50	1	09/17/2020	77	1159	2	9.6	0.03	42.17
2 multi 2		H						
50	1	09/18/2020	78	1160	2	9.8	0.55	41.33
2 multi 2		H						
50	1	09/19/2020	79	1161	2	9.8	0.67	38.53
2 multi 2		H						
50	1	09/20/2020	80	1162	2	9.3	0.17	38.94
2 multi 2		H						
50	1	09/21/2020	81	1163	2	9.6	0.64	38.72
2 multi 2		H						
50	1	09/22/2020	82	1164	2	9.5	0.1	40.86
2 multi 2		H						
50	1	09/23/2020	83	1165	2	9.8	0	39.52
2 multi 2		H						
50	1	09/24/2020	84	1166	2	9.6	0.02	39.98
2 multi 2		H						
50	1	09/25/2020	85	1167	2	9.8	0.1	37.44
2 multi 2		H						
50	1	09/26/2020	86	1168	2	9.2	0.11	32.75
2 multi 2		H						
50	1	09/27/2020	87	1169	2	9.5	0	39.94
2 multi 2		H						
50	1	09/28/2020	88	1170	2	9.5	0.09	41.44
2 multi 2		H						
50	1	09/29/2020	89	1171	2	9.6	0	42.46

2 multi	2	H						
50	1	09/30/2020	90	1172	2	9.3	0.02	39.51
2 multi	2	H						
50	1	10/01/2020	91	1173	2	9.2	0.09	37.48
2 multi	2	H						
50	1	10/02/2020	92	1174	2	9.5	0	37.75
2 multi	2	H						
50	1	10/03/2020	93	1175	2	9.5	0	39.3
2 multi	2	H						
50	1	10/04/2020	94	1176	2	9.3	0.18	41.28
2 multi	2	H						
50	1	10/05/2020	95	1177	2	9.5	0.42	38.34
2 multi	2	H						
50	1	10/06/2020	96	1178	2	9.3	0.03	25.88
2 multi	2	H						
50	1	10/07/2020	97	1179	2	8.8	0	19.74
2 multi	2	H						
50	1	10/08/2020	98	1180	2	9.3	0.01	21.15
2 multi	2	H						
50	1	10/09/2020	99	1181	2	9.2	0	26.76
2 multi	2	H						
50	1	10/10/2020	100	1182	2	9.2	0.09	29.5
2 multi	2	H						
50	1	10/11/2020	101	1183	2	9.1	0.03	31.89
2 multi	2	H						
50	1	10/12/2020	102	1184	2	9.2	0.16	32.68
2 multi	2	H						
50	1	10/13/2020	103	1185	2	9.5	0	29.44
2 multi	2	H						
50	1	10/14/2020	104	1186	2	9.8	0.17	29.69
2 multi	2	H						
50	1	10/15/2020	105	1187	2	9.8	0.12	29.95
2 multi	2	H						
50	1	10/16/2020	106	1188	2	9.1	0.05	28.2
2 multi	2	H						
50	1	10/17/2020	107	1189	2	9.8	0	32.81
2 multi	2	H						
50	1	10/18/2020	108	1190	2	9.8	0.13	34.66
2 multi	2	H						
50	1	10/19/2020	109	1191	2	9.6	0.13	34.68
2 multi	2	H						
50	1	10/20/2020	110	1192	2	9.2	0.08	34.21
2 multi	2	H						
50	1	10/21/2020	111	1193	2	9.5	0.13	32.66
2 multi	2	H						
50	1	10/22/2020	112	1194	2	9.5	0	31.98
2 multi	2	H						
50	1	10/23/2020	113	1195	2	9.3	0.05	34.14
2 multi	2	H						
50	1	10/24/2020	114	1196	2	9.5	0.05	32.75
2 multi	2	H						
50	1	10/25/2020	115	1197	2	9.2	0.07	35.5
2 multi	2	H						
50	1	10/26/2020	116	1198	2	9.6	0.32	37.75

2 multi	2	H						
50	1	10/27/2020	117	1199	2	9.6	0	35.65
2 multi	2	H						
50	1	10/28/2020	118	1200	2	9.3	0.14	35.56
2 multi	2	H						
50	1	10/29/2020	119	1201	2	8.8	0.03	34.32
2 multi	2	H						
50	1	10/30/2020	120	1202	2	9.5	0.12	33.93
2 multi	2	H						
50	1	10/31/2020	121	1203	2	9.3	0.12	30.32
2 multi	2	H						
50	1	11/01/2020	122	1204	2	9.3	0	29.44
2 multi	2	H						
50	1	11/02/2020	123	1205	2	9.5	0	34.29
2 multi	2	H						
50	1	11/03/2020	124	1206	2	10.3	0.05	33.92
2 multi	2	H						
50	1	11/04/2020	125	1207	2	9.6	0	33.67
2 multi	2	H						
50	1	11/05/2020	126	1208	2	9.8	0	35.65
2 multi	2	H						
50	1	11/06/2020	127	1209	2	10.3	0.17	36.07
2 multi	2	H						
50	1	11/07/2020	128	1210	2	9.6	0.2	37.34
2 multi	2	H						
50	1	11/08/2020	129	1211	2	10.2	0.09	38.12
2 multi	2	H						
50	1	11/09/2020	130	1212	2	10.2	0.19	36.38
2 multi	2	H						
50	1	11/10/2020	131	1213	2	10.2	0.09	36.06
2 multi	2	H						
50	1	11/11/2020	132	1214	2	10.2	0.09	35.72
2 multi	2	H						
50	1	11/12/2020	133	1215	2	10	0.32	36.01
2 multi	2	H						
50	1	11/13/2020	134	1216	2	10.3	0.17	36.97
2 multi	2	H						
50	1	11/14/2020	135	1217	2	10.2	0.09	35.21
2 multi	2	H						
50	1	11/15/2020	136	1218	2	9.6	0.05	32.99
2 multi	2	H						
50	1	11/16/2020	137	1219	2	9.6	0.01	35.63
2 multi	2	H						
50	1	11/17/2020	138	1220	2	9.6	0.05	35.97
2 multi	2	H						
50	1	11/18/2020	139	1221	2	9.9	0.11	34.48
2 multi	2	H						
50	1	11/19/2020	140	1222	2	9.6	0.14	34.89
2 multi	2	H						
50	1	11/20/2020	141	1223	2	9.9	0.61	37.43
2 multi	2	H						
50	1	11/21/2020	142	1224	2	9.5	0.04	35.44
2 multi	2	H						
51	1	11/22/2020	4	754	1	9.9	0.39	21.31

1 primi	1	C						
51	1	11/23/2020	5	755	1	9.2	0.25	23.1
1 primi	1	C						
51	1	11/24/2020	6	756	1	9.6	0.2	21.02
1 primi	1	C						
51	1	11/25/2020	7	757	1	9.3	0.59	21.96
1 primi	1	C						
51	1	11/26/2020	8	758	1	9.5	0.22	23.65
1 primi	1	C						
51	1	11/27/2020	9	759	1	9.2	0.14	24.06
1 primi	1	C						
51	1	11/28/2020	10	760	1	9.2	0.16	24.91
1 primi	1	C						
51	1	11/29/2020	11	761	1	9.2	0.21	25.65
1 primi	1	C						
51	1	11/30/2020	12	762	1	9.2	0.17	26.25
1 primi	1	C						
51	1	12/01/2020	13	763	1	8.8	0.05	28.58
1 primi	1	C						
51	1	12/02/2020	14	764	1	8.9	0.15	27.65
1 primi	1	C						
51	1	12/03/2020	15	765	1	8.9	0.16	24.46
1 primi	1	C						
51	1	12/04/2020	16	766	1	8.9	0.19	28.63
1 primi	1	C						
51	1	12/05/2020	17	767	1	8.7	0.28	27.25
1 primi	1	C						
51	1	12/06/2020	18	768	1	8.5	0.26	30.14
1 primi	1	C						
51	1	12/07/2020	19	769	1	8.6	0.18	27.32
1 primi	1	C						
51	1	12/08/2020	20	770	1	9.1	0.07	30.83
1 primi	1	C						
51	1	12/09/2020	21	771	1	8.8	0.25	29.27
1 primi	1	C						
51	1	12/10/2020	22	772	1	8.8	0.15	29.22
1 primi	2	C						
51	1	12/11/2020	23	773	1	8.8	0.07	31.09
1 primi	2	C						
51	1	12/12/2020	24	774	1	8.6	0.16	29.33
1 primi	2	C						
51	1	12/13/2020	25	775	1	8.8	0.11	30.3
1 primi	2	C						
51	1	12/14/2020	26	776	1	8.5	0.07	29.63
1 primi	2	C						
51	1	12/15/2020	27	777	1	8.6	0	29.48
1 primi	2	C						
51	1	12/16/2020	28	778	1	8.7	0	30.33
1 primi	2	C						
51	1	12/17/2020	29	779	1	8.5	0.2	30.8
1 primi	2	C						
51	1	12/18/2020	30	780	1	8.7	0.14	31.56
1 primi	2	C						
51	1	12/19/2020	31	781	1	8.4	0.24	30.9

1 primi 2 C
51 1 12/20/2020 32 782 1 8.3 0.24 30.48
1 primi 2 C
51 1 12/21/2020 33 783 1 8.7 0 30.77
1 primi 2 C
51 1 12/22/2020 34 784 1 8.9 0.03 31.43
1 primi 2 C
51 1 12/23/2020 35 785 1 8.8 0.07 30.76
1 primi 2 C
51 1 12/24/2020 36 786 1 8.6 0.03 31.54
1 primi 2 C
51 1 12/25/2020 37 787 1 8.8 0 33.82
1 primi 2 C
51 1 12/26/2020 38 788 1 8.7 0 31.86
1 primi 2 C
51 1 12/27/2020 39 789 1 8.8 0.13 31.79
1 primi 2 C
51 1 12/28/2020 40 790 1 8.7 0.15 32.62
1 primi 2 C
51 1 12/29/2020 41 791 1 8.6 0.05 32.96
1 primi 2 C
51 1 12/30/2020 42 792 1 8.7 0.34 31.8
1 primi 2 C
51 1 12/31/2020 43 793 1 8.9 0 32.82
1 primi 2 C
51 1 01/01/2021 44 794 1 8.7 0.04 33.1
1 primi 2 C
51 1 01/02/2021 45 795 1 8.4 0.06 30.89
1 primi 2 C
51 1 01/03/2021 46 796 1 8.7 0.1 32.56
1 primi 2 C
51 1 01/04/2021 47 797 1 9.1 0.12 31.99
1 primi 2 C
51 1 01/05/2021 48 798 1 8.9 0 33.3
1 primi 2 C
51 1 01/06/2021 49 799 1 8.7 0.12 33.77
1 primi 2 C
51 1 01/07/2021 50 800 1 8.7 0.06 33.55
1 primi 2 C
51 1 01/08/2021 51 801 1 8.8 0.12 30.57
1 primi 2 C
51 1 01/09/2021 52 802 1 8.8 0.04 33.32
1 primi 2 C
51 1 01/10/2021 53 803 1 8.6 0.1 31.89
1 primi 2 C
51 1 01/11/2021 54 804 1 8.8 0.03 33.75
1 primi 2 C
51 1 01/12/2021 55 805 1 8.7 0.05 32.9
1 primi 2 C
51 1 01/13/2021 56 806 1 8.7 0 33.78
1 primi 2 C
51 1 01/14/2021 57 807 1 8.4 0.18 31.76
1 primi 2 C
51 1 01/15/2021 58 808 1 8.4 0.24 32.15

1 primi 2 C
51 1 01/16/2021 59 809 1 8.1 0.17 31
1 primi 2 C
51 1 01/17/2021 60 810 1 8.4 0 31.93
1 primi 2 C
51 1 01/18/2021 61 811 1 8.5 0.17 31.58
1 primi 2 C
51 1 01/19/2021 62 812 1 8.7 0.06 30.78
1 primi 2 C
51 1 01/20/2021 63 813 1 8.7 0 28.36
1 primi 2 C
51 1 01/21/2021 64 814 1 8.7 0.11 27.37
1 primi 2 C
51 1 01/22/2021 65 815 1 8.9 0 29.47
1 primi 2 C
51 1 01/23/2021 66 816 1 8.8 0.01 29.65
1 primi 2 C
51 1 01/24/2021 67 817 1 8.7 0.03 29.05
1 primi 2 C
51 1 01/25/2021 68 818 1 8.7 0.14 26.82
1 primi 2 C
51 1 01/26/2021 69 819 1 8.8 0.01 26.85
1 primi 2 C
51 1 01/27/2021 70 820 1 9.1 0.11 28.6
1 primi 2 C
51 1 01/28/2021 71 821 1 8.6 0.06 27.96
1 primi 2 C
51 1 01/29/2021 72 822 1 8.8 0.19 28.83
1 primi 2 C
51 1 01/30/2021 73 823 1 8.6 0.1 29.65
1 primi 2 C
51 1 01/31/2021 74 824 1 8.3 0 28.62
1 primi 2 C
51 1 02/01/2021 75 825 1 8.8 0.35 31.09
1 primi 2 C
51 1 02/02/2021 76 826 1 8.6 0.03 29.91
1 primi 2 C
51 1 02/03/2021 77 827 1 9.2 0 30.21
1 primi 2 C
52 1 06/10/2019 9 725 1 9.8 0.21 24.04
1 primi 1 H
52 1 06/11/2019 10 726 1 11.3 0 23.31
1 primi 1 H
52 1 06/12/2019 11 727 1 10.2 0.04 24.61
1 primi 1 H
52 1 06/13/2019 12 728 1 10.4 0.03 23.86
1 primi 1 H
52 1 06/14/2019 13 729 1 10.4 0.01 24.87
1 primi 1 H
52 1 06/15/2019 14 730 1 11.7 0.01 26.19
1 primi 1 H
52 1 06/16/2019 15 731 1 10.7 0.11 26.93
1 primi 1 H
52 1 06/17/2019 16 732 1 10.2 0.04 27.19

1 primi	1	H						
52	1	06/18/2019	17	733	1	10.7	0.14	26.24
1 primi	1	H						
52	1	06/19/2019	18	734	1	10.2	0.02	28.09
1 primi	1	H						
52	1	06/20/2019	19	735	1	10.3	0.11	29.25
1 primi	1	H						
52	1	06/21/2019	20	736	1	10.2	0.02	28.42
1 primi	1	H						
52	1	06/22/2019	21	737	1	10.3	0.01	29.95
1 primi	1	H						
52	1	06/23/2019	22	738	1	9.6	0.08	29.8
1 primi	2	H						
52	1	06/24/2019	23	739	1	10.2	0.06	30.35
1 primi	2	H						
52	1	06/25/2019	24	740	1	10.3	0	32.24
1 primi	2	H						
52	1	06/26/2019	25	741	1	10.6	0.07	31.6
1 primi	2	H						
52	1	06/27/2019	26	742	1	10.3	0.17	31.82
1 primi	2	H						
52	1	06/28/2019	27	743	1	10.2	0.11	31.67
1 primi	2	H						
52	1	06/29/2019	28	744	1	9.5	0.02	31.61
1 primi	2	H						
52	1	06/30/2019	29	745	1	10.3	0.1	32.08
1 primi	2	H						
52	1	07/01/2019	30	746	1	10.2	0.11	32.99
1 primi	2	H						
52	1	07/02/2019	31	747	1	10.2	0	34.68
1 primi	2	H						
52	1	07/03/2019	32	748	1	10.3	0.15	36.63
1 primi	2	H						
52	1	07/04/2019	33	749	1	10.4	0	32.24
1 primi	2	H						
52	1	07/05/2019	34	750	1	10	0.06	30.2
1 primi	2	H						
52	1	07/06/2019	35	751	1	9.9	0	32.78
1 primi	2	H						
52	1	07/07/2019	36	752	1	10.2	0	34.68
1 primi	2	H						
52	1	07/08/2019	37	753	1	10.3	0	33.11
1 primi	2	H						
52	1	07/09/2019	38	754	1	10.2	0.01	30.75
1 primi	2	H						
52	1	07/10/2019	39	755	1	10.2	0.1	34.4
1 primi	2	H						
52	1	07/11/2019	40	756	1	10.2	0.08	32.12
1 primi	2	H						
52	1	07/12/2019	41	757	1	11.3	0.01	34.42
1 primi	2	H						
52	1	07/13/2019	42	758	1	10.6	0	33.32
1 primi	2	H						
52	1	07/14/2019	43	759	1	10.7	0.13	34.49

1 primi 2 H
52 1 07/15/2019 44 760 1 10.4 0.16 32.85
1 primi 2 H
52 1 07/16/2019 45 761 1 10.4 0.08 36.29
1 primi 2 H
52 1 07/17/2019 46 762 1 10.7 0.09 33.81
1 primi 2 H
52 1 07/18/2019 47 763 1 10.7 0.36 34.95
1 primi 2 H
52 1 07/19/2019 48 764 1 10.6 0.02 34.54
1 primi 2 H
52 1 07/20/2019 49 765 1 10.9 0.04 34.96
1 primi 2 H
52 1 07/21/2019 50 766 1 10.6 0 35.33
1 primi 2 H
52 1 07/22/2019 51 767 1 10.4 0 35.22
1 primi 2 H
52 1 07/23/2019 52 768 1 10.4 0.05 33.87
1 primi 2 H
52 1 07/24/2019 53 769 1 10.3 0.01 35.15
1 primi 2 H
52 1 07/25/2019 54 770 1 10.4 0.01 34.9
1 primi 2 H
52 1 07/26/2019 55 771 1 10.6 0 33.11
1 primi 2 H
52 1 07/27/2019 56 772 1 9.9 0.07 31.33
1 primi 2 H
52 1 07/28/2019 57 773 1 10.7 0 33.57
1 primi 2 H
52 1 07/29/2019 58 774 1 10.6 0.01 32.66
1 primi 2 H
52 1 07/30/2019 59 775 1 10.9 0.05 30.05
1 primi 2 H
52 1 07/31/2019 60 776 1 10.6 0.03 34.86
1 primi 2 H
52 1 08/01/2019 61 777 1 10.9 0.02 32.5
1 primi 2 H
52 1 08/02/2019 62 778 1 10.6 0.04 33.32
1 primi 2 H
52 1 08/03/2019 63 779 1 10.6 0.22 32.69
1 primi 2 H
52 1 08/04/2019 64 780 1 10.6 0.02 32.22
1 primi 2 H
52 1 08/05/2019 65 781 1 10.7 0.01 35.66
1 primi 2 H
52 1 08/06/2019 66 782 1 10.6 0.02 31.67
1 primi 2 H
52 1 08/07/2019 67 783 1 11.1 0.08 33.67
1 primi 2 H
52 1 08/08/2019 68 784 1 10.7 0.02 31.51
1 primi 2 H
52 1 08/09/2019 69 785 1 10.7 0.28 32.69
1 primi 2 H
52 1 08/10/2019 70 786 1 11.1 0.02 33.84

1 primi	2	H						
52	1	08/11/2019	71	787	1	10.6	0.26	31.7
1 primi	2	H						
52	1	08/12/2019	72	788	1	11.3	0.01	34.52
1 primi	2	H						
52	1	08/13/2019	73	789	1	11.1	0.03	33.03
1 primi	2	H						
52	1	08/14/2019	74	790	1	11.3	0.03	28.93
1 primi	2	H						
52	1	08/15/2019	75	791	1	10.7	0.24	27.85
1 primi	2	H						
52	1	08/16/2019	76	792	1	11.3	0.05	32.93
1 primi	2	H						
52	1	08/17/2019	77	793	1	11.1	0.03	33.46
1 primi	2	H						
52	1	08/18/2019	78	794	1	11.1	0.09	32.87
1 primi	2	H						
52	1	08/19/2019	79	795	1	10.7	0.09	32.7
1 primi	2	H						
52	1	08/20/2019	80	796	1	10.9	0.03	32.33
1 primi	2	H						
52	1	08/21/2019	81	797	1	10.3	0.02	29.82
1 primi	2	H						
52	1	08/22/2019	82	798	1	10.9	0.06	31.64
1 primi	2	H						
52	1	08/23/2019	83	799	1	10.7	0.03	32.26
1 primi	2	H						
52	1	08/24/2019	84	800	1	10.9	0.03	34.21
1 primi	2	H						
52	1	08/25/2019	85	801	1	11.1	0.04	33.88
1 primi	2	H						
52	1	08/26/2019	86	802	1	10.6	0.03	31.11
1 primi	2	H						
52	1	08/27/2019	87	803	1	10.9	0.02	32.53
1 primi	2	H						
52	1	08/28/2019	88	804	1	10.6	0	30.55
1 primi	2	H						
52	1	08/29/2019	89	805	1	10.4	0.01	29.97
1 primi	2	H						
52	1	08/30/2019	90	806	1	9.6	0.01	28.79
1 primi	2	H						
52	1	08/31/2019	91	807	1	10.9	0.01	29.35
1 primi	2	H						
52	1	09/01/2019	92	808	1	11.4	0.02	29.18
1 primi	2	H						
52	1	09/02/2019	93	809	1	10.4	0.01	28.26
1 primi	2	H						
52	1	09/03/2019	94	810	1	11.3	0.02	28.93
1 primi	2	H						
52	1	09/04/2019	95	811	1	10.4	0.09	28.38
1 primi	2	H						
52	1	10/05/2020	4	1208	2	9.5	0.32	29.07
2 multi	1	H						
52	1	10/06/2020	5	1209	2	10	0	30.62

2 multi	1	H						
52	1	10/07/2020	6	1210	2	10.4	0.04	28.88
2 multi	1	H						
52	1	10/08/2020	7	1211	2	9.5	0	26.21
2 multi	1	H						
52	1	10/09/2020	8	1212	2	9.5	0	32.67
2 multi	1	H						
52	1	10/10/2020	9	1213	2	9.6	0	33.88
2 multi	1	H						
52	1	10/11/2020	10	1214	2	9.8	0	37.82
2 multi	1	H						
52	1	10/12/2020	11	1215	2	9.9	0	38.85
2 multi	1	H						
52	1	10/13/2020	12	1216	2	10.2	0.07	38.97
2 multi	1	H						
53	1	01/27/2020	5	719	1	9.2	0.23	23.05
1 primi	1	H						
53	1	01/28/2020	6	720	1	9.9	0.03	25.54
1 primi	1	H						
53	1	01/29/2020	7	721	1	10.6	0.13	25.71
1 primi	1	H						
53	1	01/30/2020	8	722	1	9.9	0	25.6
1 primi	1	H						
53	1	01/31/2020	9	723	1	10.3	0.05	28.25
1 primi	1	H						
53	1	02/01/2020	10	724	1	9.5	0	28.82
1 primi	1	H						
53	1	02/02/2020	11	725	1	9.5	0.24	31.8
1 primi	1	H						
53	1	02/03/2020	12	726	1	9.6	0	30.96
1 primi	1	H						
53	1	02/04/2020	13	727	1	9.6	0	32.05
1 primi	1	H						
53	1	02/05/2020	14	728	1	8.8	0.03	31.5
1 primi	1	H						
53	1	02/06/2020	15	729	1	9.8	0	32.26
1 primi	1	H						
53	1	02/07/2020	16	730	1	9.3	0	32.37
1 primi	1	H						
53	1	02/08/2020	17	731	1	9.8	0.82	33.56
1 primi	1	H						
53	1	02/09/2020	18	732	1	9.6	0	33.03
1 primi	1	H						
53	1	02/10/2020	19	733	1	8.8	0.3	33.43
1 primi	1	H						
53	1	02/11/2020	20	734	1	9.5	0.08	33.06
1 primi	1	H						
53	1	02/12/2020	21	735	1	9.6	0.08	34.22
1 primi	1	H						
53	1	02/13/2020	22	736	1	9.3	0.37	35.14
1 primi	2	H						
53	1	02/14/2020	23	737	1	9.3	0	36.25
1 primi	2	H						
53	1	02/15/2020	24	738	1	9.1	0.02	36.34

1 primi 2 H
53 1 02/16/2020 25 739 1 8.7 0 35.23
1 primi 2 H
53 1 02/17/2020 26 740 1 9.2 0 36.6
1 primi 2 H
53 1 02/18/2020 27 741 1 9.5 0.01 37.34
1 primi 2 H
53 1 02/19/2020 28 742 1 9.3 0.01 38.82
1 primi 2 H
53 1 02/20/2020 29 743 1 9.6 0.08 34.6
1 primi 2 H
53 1 02/21/2020 30 744 1 9.2 0.02 38.56
1 primi 2 H
53 1 02/22/2020 31 745 1 9.3 0 36.27
1 primi 2 H
53 1 02/23/2020 32 746 1 9.2 0.03 39.49
1 primi 2 H
53 1 02/24/2020 33 747 1 8.9 0.05 38.56
1 primi 2 H
53 1 02/25/2020 34 748 1 8.6 0.17 40.17
1 primi 2 H
53 1 02/26/2020 35 749 1 8.9 0 41.4
1 primi 2 H
53 1 02/27/2020 36 750 1 9.1 0.01 38.38
1 primi 2 H
53 1 02/28/2020 37 751 1 9.2 0.02 39.01
1 primi 2 H
53 1 02/29/2020 38 752 1 9.2 0.02 39.25
1 primi 2 H
53 1 03/01/2020 39 753 1 9.1 0 38.04
1 primi 2 H
53 1 03/02/2020 40 754 1 9.5 0.24 40.01
1 primi 2 H
53 1 03/03/2020 41 755 1 9.5 0.03 42.59
1 primi 2 H
53 1 03/04/2020 42 756 1 9.6 0.03 39.55
1 primi 2 H
53 1 03/05/2020 43 757 1 9.6 0.04 42.14
1 primi 2 H
53 1 03/06/2020 44 758 1 9.2 0.11 42.79
1 primi 2 H
53 1 03/07/2020 45 759 1 9.6 0 43.56
1 primi 2 H
53 1 03/08/2020 46 760 1 9.2 0.2 40.57
1 primi 2 H
53 1 03/09/2020 47 761 1 9.3 0.27 41.35
1 primi 2 H
53 1 03/10/2020 48 762 1 8.8 0.23 39.7
1 primi 2 H
53 1 03/11/2020 49 763 1 8.9 0.38 40.96
1 primi 2 H
53 1 03/12/2020 50 764 1 8.9 0.4 41.72
1 primi 2 H
53 1 03/13/2020 51 765 1 9.1 0 42.32

1 primi	2	H						
53	1	03/14/2020	52	766	1	8.9	0.37	42.37
1 primi	2	H						
53	1	03/15/2020	53	767	1	9.2	0	43.38
1 primi	2	H						
53	1	03/16/2020	54	768	1	9.2	0.2	39.84
1 primi	2	H						
53	1	03/17/2020	55	769	1	9.2	0	43.2
1 primi	2	H						
53	1	03/18/2020	56	770	1	9.5	0.02	42.66
1 primi	2	H						
53	1	03/19/2020	57	771	1	9.5	0.19	44.59
1 primi	2	H						
53	1	03/20/2020	58	772	1	9.6	0.23	42.38
1 primi	2	H						
53	1	03/21/2020	59	773	1	9.6	0.25	45.25
1 primi	2	H						
53	1	03/22/2020	60	774	1	9.5	0.24	41.18
1 primi	2	H						
53	1	03/23/2020	61	775	1	9.1	0.02	45.99
1 primi	2	H						
53	1	03/24/2020	62	776	1	9.5	0.22	43.4
1 primi	2	H						
53	1	03/25/2020	63	777	1	9.3	0.09	41.9
1 primi	2	H						
53	1	03/26/2020	64	778	1	9.3	0.28	44.07
1 primi	2	H						
53	1	03/27/2020	65	779	1	9.2	0.38	44.65
1 primi	2	H						
53	1	03/28/2020	66	780	1	9.3	0.02	45.92
1 primi	2	H						
53	1	03/29/2020	67	781	1	9.1	0.34	42.34
1 primi	2	H						
53	1	03/30/2020	68	782	1	9.3	0	44.12
1 primi	2	H						
53	1	03/31/2020	69	783	1	9.1	0.1	42.92
1 primi	2	H						
53	1	04/01/2020	70	784	1	9.1	0	44.93
1 primi	2	H						
53	1	04/02/2020	71	785	1	9.3	0.02	43.61
1 primi	2	H						
53	1	04/03/2020	72	786	1	9.1	0.05	43.95
1 primi	2	H						
53	1	04/04/2020	73	787	1	9.3	0	43.86
1 primi	2	H						
53	1	04/05/2020	74	788	1	9.2	0.01	40.71
1 primi	2	H						
53	1	04/06/2020	75	789	1	9.2	0.04	42.3
1 primi	2	H						
53	1	04/07/2020	76	790	1	9.1	0.23	43.04
1 primi	2	H						
53	1	04/08/2020	77	791	1	9.3	0	39.7
1 primi	2	H						
53	1	04/09/2020	78	792	1	9.3	0	38.2

1 primi	2	H						
53	1	04/10/2020	79	793	1	9.6	0.1	43.35
1 primi	2	H						
53	1	04/11/2020	80	794	1	9.9	0.28	41.58
1 primi	2	H						
53	1	04/12/2020	81	795	1	9.5	0	40.51
1 primi	2	H						
53	1	04/13/2020	82	796	1	9.5	0	44.67
1 primi	2	H						
53	1	04/14/2020	83	797	1	9.5	0.07	43.56
1 primi	2	H						
53	1	04/15/2020	84	798	1	9.5	0.2	44.47
1 primi	2	H						
53	1	04/16/2020	85	799	1	9.3	0.03	44.32
1 primi	2	H						
53	1	04/17/2020	86	800	1	9.5	0.03	43.88
1 primi	2	H						
53	1	04/18/2020	87	801	1	9.5	0.48	41.35
1 primi	2	H						
53	1	04/19/2020	88	802	1	9.6	0.01	42.33
1 primi	2	H						
53	1	04/20/2020	89	803	1	9.5	0.02	43.76
1 primi	2	H						
53	1	04/21/2020	90	804	1	9.3	0.01	40.36
1 primi	2	H						
53	1	04/22/2020	91	805	1	9.6	0	40.79
1 primi	2	H						
53	1	04/23/2020	92	806	1	9.3	0	38.17
1 primi	2	H						
53	1	04/24/2020	93	807	1	9.5	0	40.15
1 primi	2	H						
53	1	04/25/2020	94	808	1	9.1	0.05	40.94
1 primi	2	H						
53	1	04/26/2020	95	809	1	9.6	0.04	41.99
1 primi	2	H						
53	1	04/27/2020	96	810	1	9.5	0	43.02
1 primi	2	H						
53	1	04/28/2020	97	811	1	9.5	0	42.59
1 primi	2	H						
53	1	04/29/2020	98	812	1	9.6	0.05	42.7
1 primi	2	H						
53	1	04/30/2020	99	813	1	9.3	0.65	40.67
1 primi	2	H						
53	1	05/01/2020	100	814	1	9.1	0	40.5
1 primi	2	H						
53	1	05/02/2020	101	815	1	9.3	0.31	39.98
1 primi	2	H						
53	1	05/03/2020	102	816	1	9.1	0.22	40.01
1 primi	2	H						
53	1	05/04/2020	103	817	1	9.3	0.37	39.41
1 primi	2	H						
53	1	05/05/2020	104	818	1	9.3	0	41.08
1 primi	2	H						
53	1	05/06/2020	105	819	1	9.5	0.03	39.64

1 primi	2	H						
53	1	05/07/2020	106	820	1	9.6	0	40.38
1 primi	2	H						
53	1	05/08/2020	107	821	1	9.5	0	40.22
1 primi	2	H						
53	1	05/09/2020	108	822	1	9.8	0	38.56
1 primi	2	H						
53	1	05/10/2020	109	823	1	9.8	0.03	41.28
1 primi	2	H						
53	1	05/11/2020	110	824	1	9.8	0.01	37.89
1 primi	2	H						
53	1	05/12/2020	111	825	1	9.6	0	38.98
1 primi	2	H						
53	1	05/13/2020	112	826	1	10	0.03	40.98
1 primi	2	H						
53	1	05/14/2020	113	827	1	9.8	0.02	41.02
1 primi	2	H						
53	1	05/15/2020	114	828	1	9.9	0.08	38.8
1 primi	2	H						
53	1	05/16/2020	115	829	1	9.6	0.19	43.63
1 primi	2	H						
53	1	05/17/2020	116	830	1	9.6	0	41.13
1 primi	2	H						
53	1	05/18/2020	117	831	1	9.3	0.06	41.44
1 primi	2	H						
53	1	05/19/2020	118	832	1	9.6	0.03	41.55
1 primi	2	H						
53	1	05/20/2020	119	833	1	9.6	0	41.51
1 primi	2	H						
53	1	05/21/2020	120	834	1	9.5	0	36.81
1 primi	2	H						
53	1	05/22/2020	121	835	1	9.2	0.33	41.51
1 primi	2	H						
53	1	05/23/2020	122	836	1	9.3	0	37.51
1 primi	2	H						
53	1	05/24/2020	123	837	1	9.6	0.03	40.3
1 primi	2	H						
53	1	05/25/2020	124	838	1	9.9	0.04	45.05
1 primi	2	H						
53	1	05/26/2020	125	839	1	9.5	0.05	42.26
1 primi	2	H						
53	1	05/27/2020	126	840	1	9.5	0.11	42.6
1 primi	2	H						
53	1	05/28/2020	127	841	1	9.5	0.01	43.86
1 primi	2	H						
53	1	05/29/2020	128	842	1	9.6	0.03	41.91
1 primi	2	H						
53	1	05/30/2020	129	843	1	9.5	0.02	43.41
1 primi	2	H						
53	1	05/31/2020	130	844	1	9.2	0	30.43
1 primi	2	H						
53	1	06/01/2020	131	845	1	9.3	0	32.45
1 primi	2	H						
53	1	06/02/2020	132	846	1	9.8	0.01	41.42

1 primi	2	H						
53	1	06/03/2020	133	847	1	9.3	0	37.85
1 primi	2	H						
53	1	06/04/2020	134	848	1	9.3	0.04	40.14
1 primi	2	H						
53	1	06/05/2020	135	849	1	9.3	0	39.51
1 primi	2	H						
53	1	06/06/2020	136	850	1	9.3	0	36.2
1 primi	2	H						
53	1	06/07/2020	137	851	1	9.8	0	37.27
1 primi	2	H						
53	1	06/08/2020	138	852	1	9.6	0.01	42.74
1 primi	2	H						
53	1	06/09/2020	139	853	1	9.8	0.02	43.74
1 primi	2	H						
53	1	06/10/2020	140	854	1	10	0.04	41.7
1 primi	2	H						
53	1	06/11/2020	141	855	1	9.9	0	41.53
1 primi	2	H						
53	1	06/12/2020	142	856	1	9.8	0.1	43.58
1 primi	2	H						
53	1	06/13/2020	143	857	1	9.8	0	43.02
1 primi	2	H						
53	1	06/14/2020	144	858	1	9.5	0	41.05
1 primi	2	H						
53	1	06/15/2020	145	859	1	9.6	0	40.65
1 primi	2	H						
53	1	06/16/2020	146	860	1	9.8	0.03	41.33
1 primi	2	H						
53	1	06/30/2020	160	874	1	9.5	0	38.5
1 primi	2	H						
53	1	07/01/2020	161	875	1	9.9	0	37.05
1 primi	2	H						
53	1	07/02/2020	162	876	1	9.3	0	37.13
1 primi	2	H						
53	1	07/03/2020	163	877	1	9.6	0.24	41.83
1 primi	2	H						
53	1	07/04/2020	164	878	1	10	0	39.52
1 primi	2	H						
53	1	07/05/2020	165	879	1	9.2	0.34	37.69
1 primi	2	H						
53	1	07/06/2020	166	880	1	9.3	0.1	40.96
1 primi	2	H						
53	1	07/07/2020	167	881	1	9.5	0	41.16
1 primi	2	H						
53	1	07/08/2020	168	882	1	9.5	0.1	41.85
1 primi	2	H						
53	1	07/09/2020	169	883	1	9.3	0	38.98
1 primi	2	H						
53	1	07/10/2020	170	884	1	9.6	0.15	43.04
1 primi	2	H						
53	1	07/11/2020	171	885	1	9.3	0.12	33.49
1 primi	2	H						
53	1	07/12/2020	172	886	1	9.9	0.04	41.37

1 primi 2 H
53 1 07/13/2020 173 887 1 10 0.01 39.1
1 primi 2 H
53 1 07/14/2020 174 888 1 9.5 0.01 41.7
1 primi 2 H
53 1 07/15/2020 175 889 1 9.5 0 41.7
1 primi 2 H
53 1 07/16/2020 176 890 1 9.5 0.33 40.18
1 primi 2 H
53 1 07/17/2020 177 891 1 9.8 0 42.68
1 primi 2 H
53 1 07/18/2020 178 892 1 9.6 0.18 39.96
1 primi 2 H
53 1 07/19/2020 179 893 1 9.8 0.14 41.82
1 primi 2 H
53 1 07/20/2020 180 894 1 9.8 0 41.93
1 primi 2 H
53 1 07/21/2020 181 895 1 9.8 0 42.91
1 primi 2 H
53 1 07/22/2020 182 896 1 9.5 0.01 43.01
1 primi 2 H
53 1 07/23/2020 183 897 1 9.6 0.01 36.36
1 primi 2 H
53 1 07/24/2020 184 898 1 9.6 0.02 36.9
1 primi 2 H
53 1 07/25/2020 185 899 1 9.8 0 39.04
1 primi 2 H
53 1 07/26/2020 186 900 1 9.8 0 37.91
1 primi 2 H
53 1 07/27/2020 187 901 1 9.8 0.08 37.56
1 primi 2 H
53 1 07/28/2020 188 902 1 9.3 0.22 34.67
1 primi 2 H
53 1 07/29/2020 189 903 1 9.8 0 39.03
1 primi 2 H
53 1 07/30/2020 190 904 1 9.5 0 38.21
1 primi 2 H
53 1 07/31/2020 191 905 1 9.6 0 39.53
1 primi 2 H
53 1 08/01/2020 192 906 1 9.6 0 36.43
1 primi 2 H
53 1 08/02/2020 193 907 1 9.6 0 40.03
1 primi 2 H
53 1 08/03/2020 194 908 1 9.6 0.24 41.76
1 primi 2 H
53 1 08/04/2020 195 909 1 9.6 0.11 41.15
1 primi 2 H
53 1 08/05/2020 196 910 1 9.8 0.19 41.85
1 primi 2 H
53 1 08/06/2020 197 911 1 9.8 0 40.78
1 primi 2 H
53 1 08/07/2020 198 912 1 9.8 0 37.73
1 primi 2 H
53 1 08/08/2020 199 913 1 9.8 0 40.15

1 primi 2	H
53	1	08/09/2020	200	914	1	9.6	0	40.96
1 primi 2	H
53	1	08/10/2020	201	915	1	9.8	0	39.83
1 primi 2	H
53	1	08/11/2020	202	916	1	9.8	0	37.16
1 primi 2	H
53	1	08/12/2020	203	917	1	9.9	0.08	41.41
1 primi 2	H
53	1	08/13/2020	204	918	1	9.6	0.23	36.8
1 primi 2	H
53	1	08/14/2020	205	919	1	9.6	0.18	38.43
1 primi 2	H
53	1	08/15/2020	206	920	1	9.5	0.08	38.7
1 primi 2	H
53	1	08/16/2020	207	921	1	9.6	0.02	39.36
1 primi 2	H
53	1	08/17/2020	208	922	1	9.6	0.02	38.26
1 primi 2	H
53	1	08/18/2020	209	923	1	9.8	0.12	38.31
1 primi 2	H
53	1	08/19/2020	210	924	1	9.8	0.11	38.32
1 primi 2	H
53	1	08/20/2020	211	925	1	9.6	0.01	36.28
1 primi 2	H
53	1	08/21/2020	212	926	1	10	0.03	39.9
1 primi 2	H
53	1	08/22/2020	213	927	1	10	0.09	40.32
1 primi 2	H
53	1	08/23/2020	214	928	1	9.9	0.2	40.17
1 primi 2	H
53	1	08/24/2020	215	929	1	9.6	0.34	35.92
1 primi 2	H
53	1	08/25/2020	216	930	1	9.6	0.08	37.24
1 primi 2	H
53	1	08/26/2020	217	931	1	9.9	0	38.21
1 primi 2	H
53	1	08/27/2020	218	932	1	9.6	0	37.52
1 primi 2	H
53	1	08/28/2020	219	933	1	9.6	0.11	33.33
1 primi 2	H
53	1	08/29/2020	220	934	1	9.8	0.22	37.67
1 primi 2	H
53	1	08/30/2020	221	935	1	9.3	0.21	37.2
1 primi 2	H
53	1	08/31/2020	222	936	1	9.6	0	37.83
1 primi 2	H
53	1	09/01/2020	223	937	1	9.8	0.06	32.55
1 primi 2	H
53	1	09/02/2020	224	938	1	9.3	0	34.38
1 primi 2	H
53	1	09/03/2020	225	939	1	9.9	0.13	34.72
1 primi 2	H
53	1	09/04/2020	226	940	1	9.5	0.17	17.61

1 primi	2	H						
53	1	09/05/2020	227	941	1	9.9	0.18	28.77
1 primi	2	H						
53	1	09/06/2020	228	942	1	9.6	0.25	32.84
1 primi	2	H						
53	1	09/07/2020	229	943	1	9.6	0.28	30.37
1 primi	2	H						
53	1	09/08/2020	230	944	1	10.2	0	39.24
1 primi	2	H						
53	1	09/09/2020	231	945	1	10	0.01	37.3
1 primi	2	H						
53	1	09/10/2020	232	946	1	9.8	0.01	37.56
1 primi	2	H						
53	1	09/11/2020	233	947	1	9.5	0	34.95
1 primi	2	H						
53	1	09/12/2020	234	948	1	9.8	0	42.1
1 primi	2	H						
53	1	09/13/2020	235	949	1	9.6	0	38.85
1 primi	2	H						
53	1	05/07/2021	4	1185	2	10	0.39	39.75
2 multi	1	H						
53	1	05/08/2021	5	1186	2	9.9	0.17	41.64
2 multi	1	H						
53	1	05/09/2021	6	1187	2	10.2	0	40.66
2 multi	1	H						
53	1	05/10/2021	7	1188	2	10.2	0	40.78
2 multi	1	H						
53	1	05/11/2021	8	1189	2	9.6	0	42.68
2 multi	1	H						
53	1	05/12/2021	9	1190	2	9.3	0.03	41.66
2 multi	1	H						
53	1	05/13/2021	10	1191	2	9.6	0.28	44.98
2 multi	1	H						
53	1	05/14/2021	11	1192	2	9.6	0.03	44.04
2 multi	1	H						
53	1	05/15/2021	12	1193	2	9.3	0	44.7
2 multi	1	H						
53	1	05/16/2021	13	1194	2	9.6	0.1	44.4
2 multi	1	H						
53	1	05/17/2021	14	1195	2	9.5	0.3	46.29
2 multi	1	H						
53	1	05/18/2021	15	1196	2	9.3	0	47.03
2 multi	1	H						
53	1	05/19/2021	16	1197	2	9.5	0.06	49.96
2 multi	1	H						
53	1	05/20/2021	17	1198	2	9.5	0.18	47.77
2 multi	1	H						
53	1	05/21/2021	18	1199	2	9.3	0	50.3
2 multi	1	H						
53	1	05/22/2021	19	1200	2	9.8	0.16	51.02
2 multi	1	H						
53	1	05/23/2021	20	1201	2	9.5	0.16	50.91
2 multi	1	H						
53	1	05/24/2021	21	1202	2	9.6	0	52.65

2 multi	1	H						
53	1	05/25/2021	22	1203	2	9.6	0	52.26
2 multi	2	H						
53	1	05/26/2021	23	1204	2	9.8	0.04	27.33
2 multi	2	H						
54	1	05/30/2018	10	728	1	9.2	0	26.43
1 primi	1	H						
54	1	05/31/2018	11	729	1	9.2	0	25.66
1 primi	1	H						
54	1	06/01/2018	12	730	1	9.2	0	25.35
1 primi	1	H						
54	1	06/02/2018	13	731	1	9.2	0.09	27
1 primi	1	H						
54	1	06/03/2018	14	732	1	9.2	0.02	27.86
1 primi	1	H						
54	1	06/04/2018	15	733	1	9.5	0	26.92
1 primi	1	H						
54	1	06/05/2018	16	734	1	9.6	0.09	28.13
1 primi	1	H						
54	1	06/06/2018	17	735	1	9.3	0.11	26.38
1 primi	1	H						
54	1	06/07/2018	18	736	1	9.1	0.1	28.62
1 primi	1	H						
54	1	06/08/2018	19	737	1	9.1	0.3	29.12
1 primi	1	H						
54	1	06/09/2018	20	738	1	8.9	0.09	30.96
1 primi	1	H						
54	1	06/10/2018	21	739	1	9.2	0	31.25
1 primi	1	H						
54	1	06/11/2018	22	740	1	9.3	0.07	31.61
1 primi	2	H						
54	1	06/12/2018	23	741	1	9.3	0.07	33.31
1 primi	2	H						
54	1	06/13/2018	24	742	1	9.1	0	32.73
1 primi	2	H						
54	1	06/14/2018	25	743	1	9.1	0.16	33.87
1 primi	2	H						
54	1	06/15/2018	26	744	1	8.9	0.08	32.68
1 primi	2	H						
54	1	06/16/2018	27	745	1	8.7	0.16	31.64
1 primi	2	H						
54	1	06/17/2018	28	746	1	9.2	0.06	32.86
1 primi	2	H						
54	1	06/18/2018	29	747	1	8.9	0.05	31.68
1 primi	2	H						
54	1	06/19/2018	30	748	1	8.9	0	32.82
1 primi	2	H						
54	1	06/20/2018	31	749	1	8.9	0	31.64
1 primi	2	H						
54	1	06/21/2018	32	750	1	8.8	0	32.23
1 primi	2	H						
54	1	06/22/2018	33	751	1	8.8	0	30.15
1 primi	2	H						
54	1	06/23/2018	34	752	1	8.8	0	31.69

1 primi 2 H
54 1 06/24/2018 35 753 1 9.1 0.2 31.37
1 primi 2 H
54 1 06/25/2018 36 754 1 9.1 0.08 32.56
1 primi 2 H
54 1 06/26/2018 37 755 1 9.2 0.06 33.73
1 primi 2 H
54 1 06/27/2018 38 756 1 8.9 0.05 31.99
1 primi 2 H
54 1 06/28/2018 39 757 1 8.9 0 33.44
1 primi 2 H
54 1 06/29/2018 40 758 1 11.7 0 34.72
1 primi 2 H
54 1 06/30/2018 41 759 1 8.7 0.1 30.31
1 primi 2 H
54 1 07/01/2018 42 760 1 8.9 0.07 31.07
1 primi 2 H
54 1 07/02/2018 43 761 1 8.7 0.15 29.75
1 primi 2 H
54 1 07/03/2018 44 762 1 8.7 0.11 29.51
1 primi 2 H
54 1 07/04/2018 45 763 1 9.2 0 31.67
1 primi 2 H
54 1 07/05/2018 46 764 1 9.1 0 30.92
1 primi 2 H
54 1 07/06/2018 47 765 1 9.1 0.05 29.26
1 primi 2 H
54 1 07/07/2018 48 766 1 8.8 0 30.46
1 primi 2 H
54 1 07/08/2018 49 767 1 8.9 0 30.58
1 primi 2 H
54 1 07/09/2018 50 768 1 8.8 0.05 26.88
1 primi 2 H
54 1 07/10/2018 51 769 1 8.9 0 26.76
1 primi 2 H
54 1 07/11/2018 52 770 1 9.3 0 29.01
1 primi 2 H
54 1 07/12/2018 53 771 1 9.3 0.07 29.89
1 primi 2 H
54 1 06/28/2019 5 1122 2 9.8 0.04 26.34
2 multi 1 H
54 1 06/29/2019 6 1123 2 9.3 0.17 26.66
2 multi 1 H
54 1 06/30/2019 7 1124 2 9.3 0.13 26.14
2 multi 1 H
54 1 07/01/2019 8 1125 2 9.8 0.07 28.75
2 multi 1 H
54 1 07/02/2019 9 1126 2 9.1 0.23 30.49
2 multi 1 H
54 1 07/03/2019 10 1127 2 9.1 0 31.15
2 multi 1 H
54 1 07/04/2019 11 1128 2 8.9 0.11 30.54
2 multi 1 H
54 1 07/05/2019 12 1129 2 8.9 0.16 31.07

2 multi	1	H						
54	1	07/06/2019	13	1130	2	9.1	0.09	33.02
2 multi	1	H						
54	1	07/07/2019	14	1131	2	9.2	0.21	33.78
2 multi	1	H						
54	1	07/08/2019	15	1132	2	9.3	0.24	34.52
2 multi	1	H						
54	1	07/09/2019	16	1133	2	9.2	0.22	34.06
2 multi	1	H						
54	1	07/10/2019	17	1134	2	8.9	0.2	33.91
2 multi	1	H						
54	1	07/11/2019	18	1135	2	9.1	0.01	34.93
2 multi	1	H						
54	1	07/12/2019	19	1136	2	9.2	0.04	34.66
2 multi	1	H						
54	1	07/13/2019	20	1137	2	9.5	0.25	35.81
2 multi	1	H						
54	1	07/14/2019	21	1138	2	9.3	0.13	34.46
2 multi	1	H						
54	1	07/15/2019	22	1139	2	9.5	0.11	37.74
2 multi	2	H						
54	1	07/16/2019	23	1140	2	9.5	0.27	39.47
2 multi	2	H						
54	1	07/17/2019	24	1141	2	9.5	0.11	38.42
2 multi	2	H						
54	1	07/18/2019	25	1142	2	9.6	0	39.28
2 multi	2	H						
54	1	07/19/2019	26	1143	2	9.6	0.23	40.42
2 multi	2	H						
54	1	07/20/2019	27	1144	2	9.6	0	41.19
2 multi	2	H						
54	1	07/21/2019	28	1145	2	9.6	0.12	39.68
2 multi	2	H						
54	1	07/22/2019	29	1146	2	9.6	0.08	39.09
2 multi	2	H						
54	1	07/23/2019	30	1147	2	9.6	0.07	42.18
2 multi	2	H						
54	1	07/24/2019	31	1148	2	9.3	0.09	40.05
2 multi	2	H						
54	1	07/25/2019	32	1149	2	9.5	0.11	39.53
2 multi	2	H						
54	1	07/26/2019	33	1150	2	9.6	0.01	38.06
2 multi	2	H						
54	1	07/27/2019	34	1151	2	9.5	0	36.64
2 multi	2	H						
54	1	07/28/2019	35	1152	2	9.5	0.09	37.75
2 multi	2	H						
54	1	07/29/2019	36	1153	2	9.5	0	37.2
2 multi	2	H						
54	1	07/30/2019	37	1154	2	9.1	0	35.21
2 multi	2	H						
54	1	07/31/2019	38	1155	2	9.8	0	39.83
2 multi	2	H						
54	1	08/01/2019	39	1156	2	9.8	0.14	39.04

2 multi 2		H						
54	1	08/02/2019	40	1157	2	9.8	0.1	39.28
2 multi 2		H						
54	1	08/03/2019	41	1158	2	9.6	0	38.8
2 multi 2		H						
54	1	08/04/2019	42	1159	2	9.6	0.21	37.78
2 multi 2		H						
54	1	08/05/2019	43	1160	2	9.2	0.15	37.47
2 multi 2		H						
54	1	08/06/2019	44	1161	2	9.5	0	36.64
2 multi 2		H						
54	1	08/07/2019	45	1162	2	9.5	0.15	36.52
2 multi 2		H						
54	1	08/08/2019	46	1163	2	9.3	0	35.34
2 multi 2		H						
54	1	08/09/2019	47	1164	2	9.8	0	36.88
2 multi 2		H						
54	1	08/10/2019	48	1165	2	9.9	0.1	39.62
2 multi 2		H						
54	1	08/11/2019	49	1166	2	9.3	0	36.53
2 multi 2		H						
54	1	08/12/2019	50	1167	2	9.9	0.08	37.86
2 multi 2		H						
54	1	08/13/2019	51	1168	2	9.3	0.09	35.29
2 multi 2		H						
54	1	08/14/2019	52	1169	2	9.3	0	35.3
2 multi 2		H						
54	1	08/15/2019	53	1170	2	9.6	0	33.95
2 multi 2		H						
54	1	08/16/2019	54	1171	2	9.2	0.01	33.22
2 multi 2		H						
54	1	08/17/2019	55	1172	2	9.6	0.22	35.22
2 multi 2		H						
54	1	08/18/2019	56	1173	2	9.6	0.03	35.31
2 multi 2		H						
54	1	08/19/2019	57	1174	2	10	0.13	35.61
2 multi 2		H						
54	1	08/20/2019	58	1175	2	9.2	0.08	33.02
2 multi 2		H						
54	1	08/21/2019	59	1176	2	9.2	0	33.91
2 multi 2		H						
54	1	08/22/2019	60	1177	2	9.2	0	33.4
2 multi 2		H						
54	1	08/23/2019	61	1178	2	9.3	0.01	33.55
2 multi 2		H						
54	1	08/24/2019	62	1179	2	9.6	0.01	34.99
2 multi 2		H						
54	1	08/25/2019	63	1180	2	9.3	0.05	32.76
2 multi 2		H						
54	1	08/26/2019	64	1181	2	9.5	0.01	33.51
2 multi 2		H						
54	1	08/27/2019	65	1182	2	9.8	0	34.12
2 multi 2		H						
54	1	08/28/2019	66	1183	2	9.6	0	33.84

2 multi	2	H						
54	1	08/29/2019	67	1184	2	9.6	0.18	33.34
2 multi	2	H						
54	1	08/30/2019	68	1185	2	9.6	0	32.14
2 multi	2	H						
54	1	08/31/2019	69	1186	2	9.9	0.11	34.36
2 multi	2	H						
54	1	09/01/2019	70	1187	2	9.6	0	31.49
2 multi	2	H						
54	1	09/02/2019	71	1188	2	9.2	0	30.52
2 multi	2	H						
54	1	09/03/2019	72	1189	2	9.5	0	32.22
2 multi	2	H						
54	1	09/04/2019	73	1190	2	9.3	0	28.43
2 multi	2	H						
54	1	11/20/2020	4	1633	3	9.6	0.33	30.94
2 multi	1	H						
54	1	11/21/2020	5	1634	3	9.9	0.24	31.87
2 multi	1	H						
54	1	11/22/2020	6	1635	3	9.9	0.24	30.24
2 multi	1	H						
54	1	11/23/2020	7	1636	3	9.6	0.19	35.18
2 multi	1	H						
54	1	11/24/2020	8	1637	3	9.8	0.13	37.11
2 multi	1	H						
54	1	11/25/2020	9	1638	3	9.6	0.28	38.77
2 multi	1	H						
54	1	11/26/2020	10	1639	3	9.3	0.2	38.54
2 multi	1	H						
54	1	11/27/2020	11	1640	3	9.8	0.22	37.57
2 multi	1	H						
54	1	11/28/2020	12	1641	3	9.2	0.17	40.68
2 multi	1	H						
54	1	11/29/2020	13	1642	3	9.6	0.19	37.01
2 multi	1	H						
54	1	11/30/2020	14	1643	3	9.2	0.19	39.9
2 multi	1	H						
54	1	12/01/2020	15	1644	3	9.1	0.09	41.32
2 multi	1	H						
54	1	12/02/2020	16	1645	3	9.3	0.24	41.95
2 multi	1	H						
54	1	12/03/2020	17	1646	3	9.3	0.13	42.05
2 multi	1	H						
54	1	12/04/2020	18	1647	3	9.5	0.16	43.12
2 multi	1	H						
54	1	12/05/2020	19	1648	3	9.6	0.16	42.9
2 multi	1	H						
54	1	12/06/2020	20	1649	3	9.3	0.14	46.53
2 multi	1	H						
54	1	12/07/2020	21	1650	3	9.8	0.16	43.98
2 multi	1	H						
54	1	12/08/2020	22	1651	3	9.8	0.19	43.16
2 multi	2	H						
54	1	12/09/2020	23	1652	3	9.5	0.16	43.57

2 multi 2		H						
54	1	12/10/2020	24	1653	3	9.5	0.07	42.86
2 multi 2		H						
54	1	12/11/2020	25	1654	3	9.3	0.07	44.27
2 multi 2		H						
54	1	12/12/2020	26	1655	3	9.9	0.07	47.48
2 multi 2		H						
54	1	12/13/2020	27	1656	3	9.8	0.17	46.65
2 multi 2		H						
54	1	12/14/2020	28	1657	3	9.5	0.01	46.54
2 multi 2		H						
54	1	12/15/2020	29	1658	3	9.3	0	43.74
2 multi 2		H						
54	1	12/16/2020	30	1659	3	9.1	0	43.2
2 multi 2		H						
54	1	12/17/2020	31	1660	3	9.1	0.04	42.67
2 multi 2		H						
54	1	12/18/2020	32	1661	3	9.6	0.31	44.09
2 multi 2		H						
54	1	12/19/2020	33	1662	3	9.5	0.34	44.25
2 multi 2		H						
54	1	12/20/2020	34	1663	3	9.6	0.25	44.57
2 multi 2		H						
54	1	12/21/2020	35	1664	3	9.5	0.24	45.12
2 multi 2		H						
54	1	12/22/2020	36	1665	3	10	0.04	44.66
2 multi 2		H						
54	1	12/23/2020	37	1666	3	9.8	0.1	45.01
2 multi 2		H						
54	1	12/24/2020	38	1667	3	9.9	0.19	44.38
2 multi 2		H						
54	1	12/25/2020	39	1668	3	10	0.13	45.86
2 multi 2		H						
54	1	12/26/2020	40	1669	3	9.8	0.05	45.32
2 multi 2		H						
54	1	12/27/2020	41	1670	3	9.8	0.09	44.19
2 multi 2		H						
54	1	12/28/2020	42	1671	3	10.2	0.08	44.72
2 multi 2		H						
54	1	12/29/2020	43	1672	3	10	0.06	48.6
2 multi 2		H						
54	1	12/30/2020	44	1673	3	9.9	0.17	47.76
2 multi 2		H						
54	1	12/31/2020	45	1674	3	10.7	0.11	45.81
2 multi 2		H						
54	1	01/01/2021	46	1675	3	10	0.05	46.53
2 multi 2		H						
54	1	01/02/2021	47	1676	3	10	0.06	42.43
2 multi 2		H						
54	1	01/03/2021	48	1677	3	10.2	0.14	46.13
2 multi 2		H						
54	1	01/04/2021	49	1678	3	10.2	0.02	48.93
2 multi 2		H						
54	1	01/05/2021	50	1679	3	10	0.04	46.49

2 multi 2		H						
54	1	01/06/2021	51	1680	3	10	0.01	46.92
2 multi 2		H						
54	1	01/07/2021	52	1681	3	9.8	0.15	44.9
2 multi 2		H						
54	1	01/08/2021	53	1682	3	10.2	0.27	45.93
2 multi 2		H						
54	1	01/09/2021	54	1683	3	9.9	0.13	45.58
2 multi 2		H						
54	1	01/10/2021	55	1684	3	10.2	0.13	46.36
2 multi 2		H						
54	1	01/11/2021	56	1685	3	10.2	0.12	45.78
2 multi 2		H						
54	1	01/12/2021	57	1686	3	10.3	0.12	45.79
2 multi 2		H						
54	1	01/13/2021	58	1687	3	9.9	0.06	44.85
2 multi 2		H						
54	1	01/14/2021	59	1688	3	10	0.06	45.09
2 multi 2		H						
54	1	01/15/2021	60	1689	3	9.9	0.13	44.42
2 multi 2		H						
54	1	01/16/2021	61	1690	3	9.8	0.05	44.24
2 multi 2		H						
54	1	01/17/2021	62	1691	3	9.9	0.16	45.86
2 multi 2		H						
54	1	01/18/2021	63	1692	3	10.6	0	46.28
2 multi 2		H						
54	1	01/19/2021	64	1693	3	10.2	0.14	45.28
2 multi 2		H						
54	1	01/20/2021	65	1694	3	10.2	0.15	45.13
2 multi 2		H						
54	1	01/21/2021	66	1695	3	10.7	0.05	42.02
2 multi 2		H						
54	1	01/22/2021	67	1696	3	10.9	0.14	43.55
2 multi 2		H						
54	1	01/23/2021	68	1697	3	10.3	0.12	42.56
2 multi 2		H						
54	1	01/24/2021	69	1698	3	10.6	0.12	41.48
2 multi 2		H						
54	1	01/25/2021	70	1699	3	11.1	0.14	39.96
2 multi 2		H						
54	1	01/26/2021	71	1700	3	10.4	0.15	40.2
2 multi 2		H						
54	1	01/27/2021	72	1701	3	10.7	0.03	39.65
2 multi 2		H						
54	1	01/28/2021	73	1702	3	10.2	0.03	40.18
2 multi 2		H						
54	1	01/29/2021	74	1703	3	10.2	0.05	41.81
2 multi 2		H						
54	1	01/30/2021	75	1704	3	10.7	0	43.78
2 multi 2		H						
54	1	01/31/2021	76	1705	3	10.2	0.06	40
2 multi 2		H						
54	1	02/01/2021	77	1706	3	10.2	0.04	39.45

2 multi 2		H						
54	1	02/02/2021	78	1707	3	10.9	0.05	40.14
2 multi 2		H						
54	1	02/03/2021	79	1708	3	11.3	0.12	48.95
2 multi 2		H						
54	1	02/04/2021	80	1709	3	10.2	0.12	46.61
2 multi 2		H						
54	1	02/05/2021	81	1710	3	10.3	0.05	44.05
2 multi 2		H						
54	1	02/06/2021	82	1711	3	10.2	0.04	41.34
2 multi 2		H						
54	1	02/07/2021	83	1712	3	10.4	0.13	47.34
2 multi 2		H						
54	1	02/08/2021	84	1713	3	10.3	0.02	39.68
2 multi 2		H						
54	1	02/09/2021	85	1714	3	10	0.18	40.93
2 multi 2		H						
54	1	02/10/2021	86	1715	3	10.7	0.06	42.38
2 multi 2		H						
54	1	02/11/2021	87	1716	3	10.2	0.07	42.26
2 multi 2		H						
54	1	02/12/2021	88	1717	3	10.6	0	41.95
2 multi 2		H						
54	1	02/13/2021	89	1718	3	10.3	0.03	42.99
2 multi 2		H						
54	1	02/14/2021	90	1719	3	11.4	0.04	41.96
2 multi 2		H						
54	1	02/15/2021	91	1720	3	11.1	0.02	42.16
2 multi 2		H						
54	1	02/16/2021	92	1721	3	10.4	0.03	40.37
2 multi 2		H						
54	1	02/17/2021	93	1722	3	10.2	0.07	41.34
2 multi 2		H						
54	1	02/18/2021	94	1723	3	10.4	0.15	43.33
2 multi 2		H						
54	1	02/19/2021	95	1724	3	10.9	0.16	44.49
2 multi 2		H						
54	1	02/20/2021	96	1725	3	10.6	0.15	44.98
2 multi 2		H						
54	1	02/21/2021	97	1726	3	10.7	0.06	42.44
2 multi 2		H						
54	1	02/22/2021	98	1727	3	10.2	0.05	38.75
2 multi 2		H						
54	1	02/23/2021	99	1728	3	10	0.01	38.58
2 multi 2		H						
54	1	02/24/2021	100	1729	3	10.3	0.13	36.4
2 multi 2		H						
54	1	02/25/2021	101	1730	3	10	0	40.95
2 multi 2		H						
54	1	02/26/2021	102	1731	3	12.3	0.13	34.47
2 multi 2		H						
54	1	02/27/2021	103	1732	3	10.9	0	33.33
2 multi 2		H						
54	1	02/28/2021	104	1733	3	9.9	0.12	36.08

2 multi 2		H						
54	1	03/01/2021	105	1734	3	13.5	0.02	34.69
2 multi 2		H						
54	1	03/02/2021	106	1735	3	11.3	0.04	36.83
2 multi 2		H						
54	1	03/03/2021	107	1736	3	10.2	0.04	36.31
2 multi 2		H						
54	1	03/04/2021	108	1737	3	10.3	0.04	37.73
2 multi 2		H						
54	1	03/05/2021	109	1738	3	10.9	0	38.09
2 multi 2		H						
54	1	03/06/2021	110	1739	3	10.2	0.01	39.89
2 multi 2		H						
54	1	03/07/2021	111	1740	3	10.9	0.15	38.75
2 multi 2		H						
54	1	03/08/2021	112	1741	3	10.7	0.14	40.97
2 multi 2		H						
54	1	03/09/2021	113	1742	3	10.3	0.15	40.54
2 multi 2		H						
54	1	03/10/2021	114	1743	3	10.7	0.05	39.87
2 multi 2		H						
54	1	03/11/2021	115	1744	3	10.7	0.04	36.9
2 multi 2		H						
54	1	03/12/2021	116	1745	3	10.7	0.01	39.69
2 multi 2		H						
54	1	03/13/2021	117	1746	3	11.3	0	37.97
2 multi 2		H						
54	1	03/14/2021	118	1747	3	10.3	0.16	38.23
2 multi 2		H						
54	1	03/15/2021	119	1748	3	11.4	0.01	38.88
2 multi 2		H						
54	1	03/16/2021	120	1749	3	11.3	0.01	40.14
2 multi 2		H						
54	1	03/17/2021	121	1750	3	10.7	0	39.72
2 multi 2		H						
54	1	03/18/2021	122	1751	3	10.6	0	38.66
2 multi 2		H						
54	1	03/19/2021	123	1752	3	10.9	0.23	38
2 multi 2		H						
54	1	03/20/2021	124	1753	3	10.7	0.13	36.31
2 multi 2		H						
54	1	03/21/2021	125	1754	3	10.7	0.33	36.15
2 multi 2		H						
54	1	03/22/2021	126	1755	3	10.7	0.15	37.87
2 multi 2		H						
54	1	03/23/2021	127	1756	3	10.6	0.14	35.25
2 multi 2		H						
54	1	03/24/2021	128	1757	3	10.4	0.15	36.95
2 multi 2		H						
54	1	03/25/2021	129	1758	3	9.9	0.2	39.19
2 multi 2		H						
54	1	03/26/2021	130	1759	3	11.4	0	40.21
2 multi 2		H						
54	1	03/27/2021	131	1760	3	10.4	0.04	40.73

2 multi 2		H						
54	1	03/28/2021	132	1761	3	10.4	0.13	41.08
2 multi 2		H						
54	1	03/29/2021	133	1762	3	10.6	0.14	39.61
2 multi 2		H						
54	1	03/30/2021	134	1763	3	10.3	0	41.3
2 multi 2		H						
54	1	03/31/2021	135	1764	3	10.3	0	41.51
2 multi 2		H						
54	1	04/01/2021	136	1765	3	10.4	0.04	39.86
2 multi 2		H						
54	1	04/02/2021	137	1766	3	10.3	0	41.97
2 multi 2		H						
54	1	04/03/2021	138	1767	3	11.1	0.13	38.33
2 multi 2		H						
54	1	04/04/2021	139	1768	3	10.4	0.15	39.06
2 multi 2		H						
54	1	04/05/2021	140	1769	3	10.7	0.32	39.01
2 multi 2		H						
54	1	04/06/2021	141	1770	3	10.6	0.11	37.39
2 multi 2		H						
54	1	04/07/2021	142	1771	3	10.4	0	39.68
2 multi 2		H						
54	1	04/08/2021	143	1772	3	10.6	0.03	35.59
2 multi 2		H						
54	1	04/09/2021	144	1773	3	10.6	0.05	35.15
2 multi 2		H						
54	1	04/10/2021	145	1774	3	9.9	0	35.47
2 multi 2		H						
54	1	04/11/2021	146	1775	3	10.6	0.23	37.7
2 multi 2		H						
54	1	04/12/2021	147	1776	3	11.1	0.05	40.75
2 multi 2		H						
54	1	04/13/2021	148	1777	3	10.6	0.15	38.66
2 multi 2		H						
54	1	04/14/2021	149	1778	3	10.4	0.32	37.29
2 multi 2		H						
54	1	04/15/2021	150	1779	3	10.7	0	39.51
2 multi 2		H						
54	1	04/16/2021	151	1780	3	11.3	0.12	39.18
2 multi 2		H						
54	1	04/17/2021	152	1781	3	10.7	0.02	39.63
2 multi 2		H						
54	1	04/18/2021	153	1782	3	10.6	0.12	38.94
2 multi 2		H						
54	1	04/19/2021	154	1783	3	10.6	0	41.16
2 multi 2		H						
54	1	04/20/2021	155	1784	3	10.6	0	40.26
2 multi 2		H						
54	1	04/21/2021	156	1785	3	10.4	0.07	39.82
2 multi 2		H						
54	1	04/22/2021	157	1786	3	10.7	0	39.85
2 multi 2		H						
54	1	04/23/2021	158	1787	3	10.3	0.03	39.8

2 multi	2	H						
54	1	04/24/2021	159	1788	3	10.9	0.21	36.46
2 multi	2	H						
54	1	04/25/2021	160	1789	3	10.3	0.13	36.61
2 multi	2	H						
54	1	04/26/2021	161	1790	3	10.4	0	39.63
2 multi	2	H						
54	1	04/27/2021	162	1791	3	10.3	0	36.93
2 multi	2	H						
54	1	04/28/2021	163	1792	3	10.6	0.06	40.76
2 multi	2	H						
54	1	04/29/2021	164	1793	3	11.3	0.05	37.69
2 multi	2	H						
54	1	04/30/2021	165	1794	3	10.7	0.05	38.31
2 multi	2	H						
54	1	05/01/2021	166	1795	3	11.4	0.06	37.84
2 multi	2	H						
54	1	05/02/2021	167	1796	3	10.7	0.01	37.1
2 multi	2	H						
54	1	05/03/2021	168	1797	3	11.3	0.06	35.12
2 multi	2	H						
54	1	05/04/2021	169	1798	3	10.9	0.01	36.56
2 multi	2	H						
54	1	05/05/2021	170	1799	3	11.4	0.14	35.33
2 multi	2	H						
54	1	05/06/2021	171	1800	3	11.1	0.07	33.53
2 multi	2	H						
54	1	05/07/2021	172	1801	3	10.6	0.12	36.12
2 multi	2	H						
54	1	05/08/2021	173	1802	3	11.4	0.05	35.32
2 multi	2	H						
54	1	05/09/2021	174	1803	3	10.7	0.12	36.71
2 multi	2	H						
54	1	05/10/2021	175	1804	3	11.7	0.13	37.85
2 multi	2	H						
54	1	05/11/2021	176	1805	3	11.1	0	37.62
2 multi	2	H						
54	1	05/12/2021	177	1806	3	10.9	0	37.66
2 multi	2	H						
54	1	05/13/2021	178	1807	3	11.3	0	37.23
2 multi	2	H						
54	1	05/14/2021	179	1808	3	11.3	0.13	36.61
2 multi	2	H						
54	1	05/15/2021	180	1809	3	11.4	0.14	35.72
2 multi	2	H						
54	1	05/16/2021	181	1810	3	10.7	0.05	37.06
2 multi	2	H						
54	1	05/17/2021	182	1811	3	11.7	0	37.28
2 multi	2	H						
54	1	05/18/2021	183	1812	3	11.1	0	37.04
2 multi	2	H						
54	1	05/19/2021	184	1813	3	10.9	0.01	33.2
2 multi	2	H						
54	1	05/20/2021	185	1814	3	11.3	0	38.54

2 multi	2	H						
54	1	05/21/2021	186	1815	3	10.7	0.01	37.52
2 multi	2	H						
54	1	05/22/2021	187	1816	3	11.4	0.01	38.18
2 multi	2	H						
54	1	05/23/2021	188	1817	3	10.9	0.06	37.59
2 multi	2	H						
54	1	05/24/2021	189	1818	3	11.3	0.14	36.23
2 multi	2	H						
54	1	05/25/2021	190	1819	3	10.9	0.01	36.73
2 multi	2	H						
54	1	05/26/2021	191	1820	3	10.3	0.15	17.93
2 multi	2	H						
57	1	04/05/2020	6	765	1	9.1	0.16	25.33
1 primi	1	H						
57	1	04/06/2020	7	766	1	9.2	0.16	26.95
1 primi	1	H						
57	1	04/07/2020	8	767	1	8.7	0.12	27.34
1 primi	1	H						
57	1	04/08/2020	9	768	1	8.8	0.16	25.92
1 primi	1	H						
57	1	04/09/2020	10	769	1	8.8	0.17	28.43
1 primi	1	H						
57	1	04/10/2020	11	770	1	9.5	0.34	30.32
1 primi	1	H						
57	1	04/11/2020	12	771	1	9.6	0.17	30.68
1 primi	1	H						
57	1	04/12/2020	13	772	1	9.5	0.15	29.98
1 primi	1	H						
57	1	04/13/2020	14	773	1	9.1	0.22	32.83
1 primi	1	H						
57	1	04/14/2020	15	774	1	8.9	0.06	30.91
1 primi	1	H						
57	1	04/15/2020	16	775	1	9.1	0	32.49
1 primi	1	H						
57	1	04/16/2020	17	776	1	9.5	0.01	31.65
1 primi	1	H						
57	1	04/17/2020	18	777	1	8.9	0.19	31.82
1 primi	1	H						
57	1	04/18/2020	19	778	1	9.1	0.37	32.64
1 primi	1	H						
57	1	04/19/2020	20	779	1	9.2	0.35	32.48
1 primi	1	H						
57	1	04/20/2020	21	780	1	9.1	0	32.3
1 primi	1	H						
57	1	04/21/2020	22	781	1	9.5	0	32.72
1 primi	2	H						
57	1	04/22/2020	23	782	1	8.8	0.25	33.53
1 primi	2	H						
57	1	04/23/2020	24	783	1	8.9	0.3	32.52
1 primi	2	H						
57	1	04/24/2020	25	784	1	8.8	0.19	32.28
1 primi	2	H						
57	1	04/25/2020	26	785	1	8.9	0.32	32.99

1 primi 2		H						
57	1	04/26/2020	27	786	1	8.7	0.33	32.73
1 primi 2		H						
57	1	04/27/2020	28	787	1	8.7	0.33	34.77
1 primi 2		H						
57	1	04/28/2020	29	788	1	8.6	0	35.66
1 primi 2		H						
57	1	04/29/2020	30	789	1	8.5	0.06	33.87
1 primi 2		H						
57	1	04/30/2020	31	790	1	8.9	0.1	33.67
1 primi 2		H						
57	1	05/01/2020	32	791	1	8.8	0.12	35.15
1 primi 2		H						
57	1	05/02/2020	33	792	1	8.6	0.27	32.8
1 primi 2		H						
57	1	05/03/2020	34	793	1	8.8	0.38	34.27
1 primi 2		H						
57	1	05/04/2020	35	794	1	8.8	0.02	34.81
1 primi 2		H						
57	1	05/05/2020	36	795	1	8.7	0.02	33.14
1 primi 2		H						
57	1	05/06/2020	37	796	1	8.7	0.27	35.1
1 primi 2		H						
57	1	05/07/2020	38	797	1	8.7	0.26	33.87
1 primi 2		H						
57	1	05/08/2020	39	798	1	8.9	0.05	35.84
1 primi 2		H						
57	1	05/09/2020	40	799	1	8.9	0.52	35.81
1 primi 2		H						
57	1	05/10/2020	41	800	1	9.3	0	35.81
1 primi 2		H						
57	1	05/11/2020	42	801	1	8.9	0.03	35.78
1 primi 2		H						
57	1	05/12/2020	43	802	1	8.8	0.09	33.63
1 primi 2		H						
57	1	05/13/2020	44	803	1	8.7	0.01	33.93
1 primi 2		H						
57	1	05/14/2020	45	804	1	9.1	0	36.51
1 primi 2		H						
57	1	05/15/2020	46	805	1	8.7	0.28	33.22
1 primi 2		H						
57	1	05/16/2020	47	806	1	9.1	0	34.87
1 primi 2		H						
57	1	05/17/2020	48	807	1	9.1	0.28	33.78
1 primi 2		H						
57	1	05/18/2020	49	808	1	8.9	0.05	35.78
1 primi 2		H						
57	1	05/19/2020	50	809	1	9.3	0.1	35.33
1 primi 2		H						
57	1	05/20/2020	51	810	1	8.7	0	35.07
1 primi 2		H						
57	1	05/21/2020	52	811	1	9.2	0.05	36.55
1 primi 2		H						
57	1	05/22/2020	53	812	1	9.3	0.55	38.79

1 primi	2	H						
57	1	05/23/2020	54	813	1	8.9	0	33.25
1 primi	2	H						
57	1	05/24/2020	55	814	1	8.7	0	34.96
1 primi	2	H						
57	1	05/25/2020	56	815	1	9.5	0	37.08
1 primi	2	H						
57	1	05/26/2020	57	816	1	9.2	0	36.07
1 primi	2	H						
57	1	05/27/2020	58	817	1	9.5	0.23	36.02
1 primi	2	H						
57	1	05/28/2020	59	818	1	9.3	0	37.82
1 primi	2	H						
57	1	05/29/2020	60	819	1	9.1	0.39	38.12
1 primi	2	H						
57	1	05/30/2020	61	820	1	9.1	0.11	37.14
1 primi	2	H						
57	1	05/31/2020	62	821	1	9.2	0.04	34.56
1 primi	2	H						
57	1	06/01/2020	63	822	1	8.9	0.84	34.48
1 primi	2	H						
57	1	06/02/2020	64	823	1	8.9	0.31	32.94
1 primi	2	H						
57	1	02/22/2021	4	1088	2	8.9	0.16	28.44
2 multi	1	H						
57	1	02/23/2021	5	1089	2	9.1	0.02	29.14
2 multi	1	H						
57	1	02/24/2021	6	1090	2	8.8	0.13	28.79
2 multi	1	H						
57	1	02/25/2021	7	1091	2	9.3	0.08	30.74
2 multi	1	H						
57	1	02/26/2021	8	1092	2	8.9	0.08	31.74
2 multi	1	H						
57	1	02/27/2021	9	1093	2	9.1	0.04	34.19
2 multi	1	H						
57	1	02/28/2021	10	1094	2	9.1	0.03	35.43
2 multi	1	H						
57	1	03/01/2021	11	1095	2	8.6	0	33.92
2 multi	1	H						
57	1	03/02/2021	12	1096	2	8.6	0.25	34.65
2 multi	1	H						
57	1	03/03/2021	13	1097	2	8.9	0.07	34.42
2 multi	1	H						
57	1	03/04/2021	14	1098	2	8.5	0	34.12
2 multi	1	H						
57	1	03/05/2021	15	1099	2	8.8	0.25	37.5
2 multi	1	H						
57	1	03/06/2021	16	1100	2	8.7	0.13	40.61
2 multi	1	H						
57	1	03/07/2021	17	1101	2	8.9	0.07	37.22
2 multi	1	H						
57	1	03/08/2021	18	1102	2	8.8	0.05	39.25
2 multi	1	H						
57	1	03/09/2021	19	1103	2	8.8	0.05	37.16

2 multi	1	H						
57	1	03/10/2021	20	1104	2	8.6	0	39.72
2 multi	1	H						
57	1	03/11/2021	21	1105	2	9.1	0.02	38.88
2 multi	1	H						
57	1	03/12/2021	22	1106	2	8.8	0.03	36.17
2 multi	2	H						
57	1	03/13/2021	23	1107	2	8.9	0.02	41.22
2 multi	2	H						
57	1	03/14/2021	24	1108	2	9.2	0.04	41.64
2 multi	2	H						
57	1	03/15/2021	25	1109	2	9.1	0.05	40.26
2 multi	2	H						
57	1	03/16/2021	26	1110	2	8.9	0.03	40.59
2 multi	2	H						
57	1	03/17/2021	27	1111	2	9.1	0.04	39.2
2 multi	2	H						
57	1	03/18/2021	28	1112	2	8.8	0	38.5
2 multi	2	H						
57	1	03/19/2021	29	1113	2	9.1	0	38.27
2 multi	2	H						
57	1	03/20/2021	30	1114	2	9.1	0	39.16
2 multi	2	H						
57	1	03/21/2021	31	1115	2	9.1	0.05	37.51
2 multi	2	H						
57	1	03/22/2021	32	1116	2	8.9	0	36.72
2 multi	2	H						
57	1	03/23/2021	33	1117	2	8.8	0	34.13
2 multi	2	H						
57	1	03/24/2021	34	1118	2	8.9	0.31	33.02
2 multi	2	H						
57	1	03/25/2021	35	1119	2	9.6	0.05	35.92
2 multi	2	H						
57	1	03/26/2021	36	1120	2	9.8	0.04	39.7
2 multi	2	H						
57	1	03/27/2021	37	1121	2	9.5	0.03	39.65
2 multi	2	H						
57	1	03/28/2021	38	1122	2	9.1	0	36.63
2 multi	2	H						
57	1	03/29/2021	39	1123	2	9.5	0	40.54
2 multi	2	H						
57	1	03/30/2021	40	1124	2	9.6	0.02	41.64
2 multi	2	H						
57	1	03/31/2021	41	1125	2	9.5	0	43.99
2 multi	2	H						
57	1	04/01/2021	42	1126	2	9.6	0.03	41.94
2 multi	2	H						
57	1	04/02/2021	43	1127	2	9.6	0.03	41.56
2 multi	2	H						
57	1	04/03/2021	44	1128	2	9.5	0	41.63
2 multi	2	H						
57	1	04/04/2021	45	1129	2	9.5	0	42.2
2 multi	2	H						
57	1	04/05/2021	46	1130	2	9.5	0.23	41.64

2 multi 2		H						
57	1	04/06/2021	47	1131	2	9.3	0.25	41.11
2 multi 2		H						
57	1	04/07/2021	48	1132	2	9.2	0	37.44
2 multi 2		H						
57	1	04/08/2021	49	1133	2	9.5	0.03	38.46
2 multi 2		H						
57	1	04/09/2021	50	1134	2	9.3	0.04	39.42
2 multi 2		H						
57	1	04/10/2021	51	1135	2	9.3	0.02	37.7
2 multi 2		H						
57	1	04/11/2021	52	1136	2	9.5	0.01	44.65
2 multi 2		H						
57	1	04/12/2021	53	1137	2	9.3	0	42.64
2 multi 2		H						
57	1	04/13/2021	54	1138	2	9.5	0.03	42.64
2 multi 2		H						
57	1	04/14/2021	55	1139	2	8.8	0.01	42.24
2 multi 2		H						
57	1	04/15/2021	56	1140	2	9.6	0.02	42.28
2 multi 2		H						
57	1	04/16/2021	57	1141	2	9.2	0	43.22
2 multi 2		H						
57	1	04/17/2021	58	1142	2	9.6	0	42.39
2 multi 2		H						
57	1	04/18/2021	59	1143	2	9.5	0.03	43.22
2 multi 2		H						
57	1	04/19/2021	60	1144	2	9.3	0.03	43.23
2 multi 2		H						
57	1	04/20/2021	61	1145	2	9.5	0.03	42.92
2 multi 2		H						
57	1	04/21/2021	62	1146	2	9.5	0.02	41.58
2 multi 2		H						
57	1	04/22/2021	63	1147	2	9.6	0.02	39.68
2 multi 2		H						
57	1	04/23/2021	64	1148	2	9.2	0	44.22
2 multi 2		H						
57	1	04/24/2021	65	1149	2	9.3	0.02	45.18
2 multi 2		H						
57	1	04/25/2021	66	1150	2	9.3	0.02	44.9
2 multi 2		H						
57	1	04/26/2021	67	1151	2	9.2	0	45.07
2 multi 2		H						
57	1	04/27/2021	68	1152	2	9.5	0	43.17
2 multi 2		H						
57	1	04/28/2021	69	1153	2	9.3	0	43.43
2 multi 2		H						
57	1	04/29/2021	70	1154	2	9.6	0.13	44.33
2 multi 2		H						
57	1	04/30/2021	71	1155	2	9.2	0.15	45.01
2 multi 2		H						
57	1	05/01/2021	72	1156	2	9.5	0.02	41.16
2 multi 2		H						
57	1	05/02/2021	73	1157	2	9.3	0	41.52

2 multi 2		H						
57	1	05/03/2021	74	1158	2	9.8	0	40.98
2 multi 2		H						
57	1	05/04/2021	75	1159	2	9.9	0.03	38.65
2 multi 2		H						
57	1	05/05/2021	76	1160	2	8.9	0	39.99
2 multi 2		H						
57	1	05/06/2021	77	1161	2	9.3	0.05	40.1
2 multi 2		H						
57	1	05/07/2021	78	1162	2	10	0.05	41.23
2 multi 2		H						
57	1	05/08/2021	79	1163	2	9.5	0	38.67
2 multi 2		H						
57	1	05/09/2021	80	1164	2	9.3	0	42.37
2 multi 2		H						
57	1	05/10/2021	81	1165	2	9.3	0.04	42.54
2 multi 2		H						
57	1	05/11/2021	82	1166	2	9.6	0.03	41.92
2 multi 2		H						
57	1	05/12/2021	83	1167	2	9.5	0	44.46
2 multi 2		H						
57	1	05/13/2021	84	1168	2	9.3	0.02	44.24
2 multi 2		H						
57	1	05/14/2021	85	1169	2	9.8	0.02	43.19
2 multi 2		H						
57	1	05/15/2021	86	1170	2	9.9	0.02	42.59
2 multi 2		H						
57	1	05/16/2021	87	1171	2	9.8	0.04	43.82
2 multi 2		H						
57	1	05/17/2021	88	1172	2	9.9	0.04	43.95
2 multi 2		H						
57	1	05/18/2021	89	1173	2	9.6	0	42.91
2 multi 2		H						
57	1	05/19/2021	90	1174	2	9.8	0.03	42.79
2 multi 2		H						
57	1	05/20/2021	91	1175	2	9.3	0.05	43.53
2 multi 2		H						
57	1	05/21/2021	92	1176	2	9.3	0	42.4
2 multi 2		H						
57	1	05/22/2021	93	1177	2	9.5	0.08	42.88
2 multi 2		H						
57	1	05/23/2021	94	1178	2	9.5	0	42.66
2 multi 2		H						
57	1	05/24/2021	95	1179	2	9.5	0	46.35
2 multi 2		H						
57	1	05/25/2021	96	1180	2	9.5	0.03	42.72
2 multi 2		H						
57	1	05/26/2021	97	1181	2	8.9	0.3	20.49
2 multi 2		H						
58	1	01/22/2020	3	823	1	9.3	0.27	16.73
1 primi 1		H						
58	1	01/23/2020	4	824	1	10.2	0	32.72
1 primi 1		H						
58	1	01/24/2020	5	825	1	9.3	0	27.25

1 primi	1	H						
58	1	01/25/2020	6	826	1	9.2	0	28.61
1 primi	1	H						
58	1	01/26/2020	7	827	1	9.2	0	28.26
1 primi	1	H						
58	1	01/27/2020	8	828	1	9.5	0	29.37
1 primi	1	H						
58	1	01/28/2020	9	829	1	9.2	0	30.18
1 primi	1	H						
58	1	01/29/2020	10	830	1	9.3	0	33.8
1 primi	1	H						
58	1	01/30/2020	11	831	1	9.5	0	33.28
1 primi	1	H						
58	1	01/31/2020	12	832	1	9.8	0	36.53
1 primi	1	H						
58	1	02/01/2020	13	833	1	9.9	0.01	36.01
1 primi	1	H						
58	1	02/02/2020	14	834	1	9.8	0	35.11
1 primi	1	H						
58	1	02/03/2020	15	835	1	9.8	0.31	36.53
1 primi	1	H						
58	1	02/04/2020	16	836	1	9.6	0	36.04
1 primi	1	H						
58	1	02/05/2020	17	837	1	9.5	0	36.11
1 primi	1	H						
58	1	02/06/2020	18	838	1	9.6	0	36.16
1 primi	1	H						
58	1	02/07/2020	19	839	1	9.6	0	37.23
1 primi	1	H						
58	1	02/08/2020	20	840	1	9.8	0	38.08
1 primi	1	H						
58	1	02/09/2020	21	841	1	9.8	0	38.19
1 primi	1	H						
58	1	02/10/2020	22	842	1	9.6	0.23	35.9
1 primi	2	H						
58	1	02/11/2020	23	843	1	9.6	0.36	39.03
1 primi	2	H						
58	1	02/12/2020	24	844	1	9.3	0	36.66
1 primi	2	H						
58	1	02/13/2020	25	845	1	9.6	0.03	36.68
1 primi	2	H						
58	1	02/14/2020	26	846	1	9.6	0	36.69
1 primi	2	H						
58	1	02/15/2020	27	847	1	9.6	0.05	36.62
1 primi	2	H						
58	1	02/16/2020	28	848	1	9.6	0.07	35.49
1 primi	2	H						
58	1	02/17/2020	29	849	1	9.2	0.09	35.25
1 primi	2	H						
58	1	02/18/2020	30	850	1	9.2	0.05	35.79
1 primi	2	H						
58	1	02/19/2020	31	851	1	8.9	0.13	36.89
1 primi	2	H						
58	1	02/20/2020	32	852	1	9.3	0.01	34.9

1 primi	2	H						
58	1	02/21/2020	33	853	1	9.2	0.1	35.57
1 primi	2	H						
58	1	02/22/2020	34	854	1	9.5	0.03	35.77
1 primi	2	H						
58	1	02/23/2020	35	855	1	9.5	0.05	37.23
1 primi	2	H						
58	1	02/24/2020	36	856	1	9.3	0	37.4
1 primi	2	H						
58	1	02/25/2020	37	857	1	9.5	0	40.82
1 primi	2	H						
58	1	02/26/2020	38	858	1	9.5	0	38.46
1 primi	2	H						
58	1	02/27/2020	39	859	1	9.3	0	40.77
1 primi	2	H						
58	1	02/28/2020	40	860	1	9.6	0.01	37.72
1 primi	2	H						
58	1	02/29/2020	41	861	1	9.5	0	41.86
1 primi	2	H						
58	1	03/01/2020	42	862	1	9.8	0	37.49
1 primi	2	H						
58	1	03/02/2020	43	863	1	9.5	0.17	40.63
1 primi	2	H						
58	1	03/03/2020	44	864	1	9.6	0	40.65
1 primi	2	H						
58	1	03/04/2020	45	865	1	9.9	0.08	42.08
1 primi	2	H						
58	1	03/05/2020	46	866	1	9.5	0.01	39.37
1 primi	2	H						
58	1	03/06/2020	47	867	1	9.8	0.01	40.56
1 primi	2	H						
58	1	03/07/2020	48	868	1	9.5	0	40.64
1 primi	2	H						
58	1	03/08/2020	49	869	1	9.3	0.25	37.54
1 primi	2	H						
58	1	03/09/2020	50	870	1	9.3	0.39	41.95
1 primi	2	H						
58	1	03/10/2020	51	871	1	0	0	41.46
1 primi	2	H						
58	1	03/11/2020	52	872	1	9.2	0	39.34
1 primi	2	H						
58	1	03/12/2020	53	873	1	9.5	0	39.78
1 primi	2	H						
58	1	03/13/2020	54	874	1	9.2	0	40.64
1 primi	2	H						
58	1	03/14/2020	55	875	1	9.2	0	40.31
1 primi	2	H						
58	1	03/15/2020	56	876	1	9.3	0.02	39.59
1 primi	2	H						
58	1	03/16/2020	57	877	1	9.5	0	40.34
1 primi	2	H						
58	1	03/17/2020	58	878	1	9.5	0	40.4
1 primi	2	H						
58	1	03/18/2020	59	879	1	9.5	0	40.28

1 primi	2	H						
58	1	03/19/2020	60	880	1	9.3	0	40.26
1 primi	2	H						
58	1	03/20/2020	61	881	1	9.3	0	40.44
1 primi	2	H						
58	1	03/21/2020	62	882	1	9.3	0	40.32
1 primi	2	H						
58	1	03/22/2020	63	883	1	9.3	0	39.38
1 primi	2	H						
58	1	03/23/2020	64	884	1	9.2	0	39.98
1 primi	2	H						
58	1	03/24/2020	65	885	1	9.2	0	39.42
1 primi	2	H						
58	1	03/25/2020	66	886	1	9.3	0	38.9
1 primi	2	H						
58	1	03/26/2020	67	887	1	9.3	0	40.45
1 primi	2	H						
58	1	03/27/2020	68	888	1	9.5	0	39.63
1 primi	2	H						
58	1	03/28/2020	69	889	1	9.2	0.12	39.23
1 primi	2	H						
58	1	03/29/2020	70	890	1	9.5	0.01	40.74
1 primi	2	H						
58	1	03/30/2020	71	891	1	9.1	0	39.07
1 primi	2	H						
58	1	03/31/2020	72	892	1	9.5	0	41.53
1 primi	2	H						
58	1	04/01/2020	73	893	1	9.5	0.2	42.64
1 primi	2	H						
58	1	04/02/2020	74	894	1	9.2	0.43	40.56
1 primi	2	H						
58	1	04/03/2020	75	895	1	9.5	0.01	42.61
1 primi	2	H						
58	1	04/04/2020	76	896	1	9.6	0.11	40.48
1 primi	2	H						
58	1	12/22/2020	7	1158	2	10	0.03	34.06
2 multi	1	H						
58	1	12/23/2020	8	1159	2	9.8	0.28	33.28
2 multi	1	H						
58	1	12/24/2020	9	1160	2	10.2	0	35.44
2 multi	1	H						
58	1	12/25/2020	10	1161	2	10.2	0	39.02
2 multi	1	H						
58	1	12/26/2020	11	1162	2	10.2	0.06	38.69
2 multi	1	H						
58	1	12/27/2020	12	1163	2	10	0.05	38.06
2 multi	1	H						
58	1	12/28/2020	13	1164	2	10	0.06	41.26
2 multi	1	H						
58	1	12/29/2020	14	1165	2	9.9	0.05	40.58
2 multi	1	H						
58	1	12/30/2020	15	1166	2	10	0.06	43.06
2 multi	1	H						
58	1	12/31/2020	16	1167	2	10.3	0.33	42.97

2 multi	1	H						
58	1	01/01/2021	17	1168	2	10	0	42.32
2 multi	1	H						
58	1	01/02/2021	18	1169	2	10	0.18	42.76
2 multi	1	H						
58	1	01/03/2021	19	1170	2	10.2	0	46.41
2 multi	1	H						
58	1	01/04/2021	20	1171	2	10.3	0.05	43.63
2 multi	1	H						
58	1	01/05/2021	21	1172	2	10	0.03	48.18
2 multi	1	H						
58	1	01/06/2021	22	1173	2	10	0	43.89
2 multi	2	H						
58	1	01/07/2021	23	1174	2	9.9	0.02	38.18
2 multi	2	H						
58	1	01/08/2021	24	1175	2	10.2	0.05	47.77
2 multi	2	H						
58	1	01/09/2021	25	1176	2	10.2	0.05	50.41
2 multi	2	H						
58	1	01/10/2021	26	1177	2	10.2	0	47.91
2 multi	2	H						
58	1	01/11/2021	27	1178	2	10	0.03	47.75
2 multi	2	H						
58	1	01/12/2021	28	1179	2	10.2	0	52.44
2 multi	2	H						
58	1	01/13/2021	29	1180	2	9.8	0	48.17
2 multi	2	H						
58	1	01/14/2021	30	1181	2	9.9	0.01	48.15
2 multi	2	H						
58	1	01/15/2021	31	1182	2	9.9	0	47.47
2 multi	2	H						
58	1	01/16/2021	32	1183	2	9.9	0.07	46.62
2 multi	2	H						
58	1	01/17/2021	33	1184	2	9.5	0.03	46.71
2 multi	2	H						
58	1	01/18/2021	34	1185	2	9.9	0	47.79
2 multi	2	H						
58	1	01/19/2021	35	1186	2	9.5	0	40.82
2 multi	2	H						
58	1	01/20/2021	36	1187	2	9.5	0.07	38.33
2 multi	2	H						
58	1	01/21/2021	37	1188	2	9.6	0.04	40.75
2 multi	2	H						
58	1	01/22/2021	38	1189	2	9.9	0.13	42.61
2 multi	2	H						
58	1	01/23/2021	39	1190	2	10.3	0.03	45.03
2 multi	2	H						
58	1	01/24/2021	40	1191	2	10.2	0	42.21
2 multi	2	H						
58	1	01/25/2021	41	1192	2	10.2	0.05	43.67
2 multi	2	H						
58	1	01/26/2021	42	1193	2	10	0.07	39.06
2 multi	2	H						
58	1	01/27/2021	43	1194	2	10.2	0.03	40.39

2 multi 2		H						
58	1	01/28/2021	44	1195	2	10.3	0	39.7
2 multi 2		H						
58	1	01/29/2021	45	1196	2	10.3	0.03	39.8
2 multi 2		H						
58	1	01/30/2021	46	1197	2	10	0.03	42
2 multi 2		H						
58	1	01/31/2021	47	1198	2	10	0.04	45.66
2 multi 2		H						
58	1	02/01/2021	48	1199	2	10.2	0.18	45.16
2 multi 2		H						
58	1	02/02/2021	49	1200	2	10.2	0.04	47.61
2 multi 2		H						
58	1	02/03/2021	50	1201	2	10.2	0	43.13
2 multi 2		H						
58	1	02/04/2021	51	1202	2	10.4	0.01	45.75
2 multi 2		H						
58	1	02/05/2021	52	1203	2	9.9	0	45.23
2 multi 2		H						
58	1	02/06/2021	53	1204	2	10.2	0.02	43.59
2 multi 2		H						
58	1	02/07/2021	54	1205	2	10.2	0.02	47.51
2 multi 2		H						
58	1	02/08/2021	55	1206	2	10.3	0	42.42
2 multi 2		H						
58	1	02/09/2021	56	1207	2	9.9	0.03	38.84
2 multi 2		H						
58	1	02/10/2021	57	1208	2	9.9	0	40.84
2 multi 2		H						
58	1	02/11/2021	58	1209	2	10.2	0	45.16
2 multi 2		H						
58	1	02/12/2021	59	1210	2	10.2	0	43.02
2 multi 2		H						
58	1	02/13/2021	60	1211	2	10.3	0.02	44.58
2 multi 2		H						
58	1	02/14/2021	61	1212	2	9.9	0.02	43.18
2 multi 2		H						
58	1	02/15/2021	62	1213	2	10.4	0.01	47.08
2 multi 2		H						
58	1	02/16/2021	63	1214	2	10	0	43.93
2 multi 2		H						
58	1	02/17/2021	64	1215	2	10.2	0.09	46.25
2 multi 2		H						
58	1	02/18/2021	65	1216	2	10.3	0.02	45.24
2 multi 2		H						
58	1	02/19/2021	66	1217	2	10.4	0	42.37
2 multi 2		H						
58	1	02/20/2021	67	1218	2	10.2	0	45.86
2 multi 2		H						
58	1	02/21/2021	68	1219	2	10.3	0	40.65
2 multi 2		H						
58	1	02/22/2021	69	1220	2	10	0.11	45.03
2 multi 2		H						
58	1	02/23/2021	70	1221	2	10.2	0.01	42.77

2 multi 2		H						
58	1	02/24/2021	71	1222	2	10.2	0	43.64
2 multi 2		H						
58	1	02/25/2021	72	1223	2	10	0	43.72
2 multi 2		H						
58	1	02/26/2021	73	1224	2	10	0	42.06
2 multi 2		H						
58	1	02/27/2021	74	1225	2	10	0	41.14
2 multi 2		H						
58	1	02/28/2021	75	1226	2	10	0	41.84
2 multi 2		H						
58	1	03/01/2021	76	1227	2	10.2	0.03	46.28
2 multi 2		H						
58	1	03/02/2021	77	1228	2	10	0.08	42.91
2 multi 2		H						
58	1	03/03/2021	78	1229	2	10.2	0.09	44.78
2 multi 2		H						
58	1	03/04/2021	79	1230	2	9.9	0.16	43.02
2 multi 2		H						
58	1	03/05/2021	80	1231	2	10	0	43.47
2 multi 2		H						
58	1	03/06/2021	81	1232	2	10	0.09	44.59
2 multi 2		H						
58	1	03/07/2021	82	1233	2	10	0.01	44.89
2 multi 2		H						
58	1	03/08/2021	83	1234	2	10.2	0.01	43.6
2 multi 2		H						
58	1	03/09/2021	84	1235	2	9.8	0	42.63
2 multi 2		H						
58	1	03/10/2021	85	1236	2	9.9	0	43.91
2 multi 2		H						
58	1	03/11/2021	86	1237	2	9.9	0	37.47
2 multi 2		H						
58	1	03/12/2021	87	1238	2	10	0.01	47.98
2 multi 2		H						
58	1	03/13/2021	88	1239	2	9.8	0	43.18
2 multi 2		H						
58	1	03/14/2021	89	1240	2	10.2	0.02	40.48
2 multi 2		H						
58	1	03/15/2021	90	1241	2	10	0	46.06
2 multi 2		H						
58	1	03/16/2021	91	1242	2	9.9	0	43.91
2 multi 2		H						
58	1	03/17/2021	92	1243	2	10.2	0.01	42.7
2 multi 2		H						
58	1	03/18/2021	93	1244	2	10.3	0.01	44.61
2 multi 2		H						
58	1	03/19/2021	94	1245	2	9.9	0.1	43.34
2 multi 2		H						
58	1	03/20/2021	95	1246	2	10	0	39.1
2 multi 2		H						
58	1	03/21/2021	96	1247	2	10.3	0.02	44.81
2 multi 2		H						
58	1	03/22/2021	97	1248	2	10.2	0.04	40.84

2 multi	2	H						
58	1	03/23/2021	98	1249	2	10	0	42.97
2 multi	2	H						
58	1	03/24/2021	99	1250	2	10.4	0.02	41.04
2 multi	2	H						
58	1	03/25/2021	100	1251	2	10.2	0.08	33.56
2 multi	2	H						
58	1	03/26/2021	101	1252	2	10.4	0	44.41
2 multi	2	H						
58	1	03/27/2021	102	1253	2	10.3	0.02	42.92
2 multi	2	H						
58	1	03/28/2021	103	1254	2	10	0.1	40.08
2 multi	2	H						
58	1	03/29/2021	104	1255	2	10.2	0	44.19
2 multi	2	H						
58	1	03/30/2021	105	1256	2	10	0	42.64
2 multi	2	H						
58	1	03/31/2021	106	1257	2	10.3	0	42.91
2 multi	2	H						
58	1	04/01/2021	107	1258	2	10.4	0.01	43.44
2 multi	2	H						
58	1	04/02/2021	108	1259	2	10	0	38.27
2 multi	2	H						
58	1	04/03/2021	109	1260	2	10.2	0	44.56
2 multi	2	H						
58	1	04/04/2021	110	1261	2	10.4	0	42.96
2 multi	2	H						
58	1	04/05/2021	111	1262	2	10.3	0.02	43.49
2 multi	2	H						
58	1	04/06/2021	112	1263	2	10.3	0	40.53
2 multi	2	H						
58	1	04/07/2021	113	1264	2	10.3	0.02	45.43
2 multi	2	H						
58	1	04/08/2021	114	1265	2	10.2	0.01	42.42
2 multi	2	H						
58	1	04/09/2021	115	1266	2	10.3	0	44.44
2 multi	2	H						
58	1	04/10/2021	116	1267	2	10	0	46.35
2 multi	2	H						
58	1	04/11/2021	117	1268	2	10.2	0	42.79
2 multi	2	H						
58	1	04/12/2021	118	1269	2	10.2	0	42.9
2 multi	2	H						
58	1	04/13/2021	119	1270	2	9.9	0	42.67
2 multi	2	H						
58	1	04/14/2021	120	1271	2	9.9	0.26	43.75
2 multi	2	H						
58	1	04/15/2021	121	1272	2	9.8	0.01	42.78
2 multi	2	H						
58	1	04/16/2021	122	1273	2	10.2	0.01	44.09
2 multi	2	H						
58	1	04/17/2021	123	1274	2	10.2	0	41.56
2 multi	2	H						
58	1	04/18/2021	124	1275	2	10.2	0	44.41

2 multi 2		H						
58	1	04/19/2021	125	1276	2	9.9	0.02	42.47
2 multi 2		H						
58	1	04/20/2021	126	1277	2	10.2	0	45.01
2 multi 2		H						
58	1	04/21/2021	127	1278	2	10.2	0.01	42.71
2 multi 2		H						
58	1	04/22/2021	128	1279	2	10.2	0.08	47.42
2 multi 2		H						
58	1	04/23/2021	129	1280	2	10.2	0.07	44.98
2 multi 2		H						
58	1	04/24/2021	130	1281	2	9.8	0.37	48.41
2 multi 2		H						
58	1	04/25/2021	131	1282	2	9.9	0	48.91
2 multi 2		H						
58	1	04/26/2021	132	1283	2	10	0	46.4
2 multi 2		H						
58	1	04/27/2021	133	1284	2	9.9	0	46.56
2 multi 2		H						
58	1	04/28/2021	134	1285	2	10	0	43.12
2 multi 2		H						
58	1	04/29/2021	135	1286	2	10	0.01	45.35
2 multi 2		H						
58	1	04/30/2021	136	1287	2	10	0.07	44.34
2 multi 2		H						
58	1	05/01/2021	137	1288	2	10.2	0.01	40.91
2 multi 2		H						
58	1	05/02/2021	138	1289	2	9.9	0.01	38.2
2 multi 2		H						
58	1	05/03/2021	139	1290	2	9.9	0	39.62
2 multi 2		H						
58	1	05/04/2021	140	1291	2	10.3	0	42.45
2 multi 2		H						
58	1	05/05/2021	141	1292	2	10	0	41.25
2 multi 2		H						
58	1	05/06/2021	142	1293	2	10.3	0	45.6
2 multi 2		H						
58	1	05/07/2021	143	1294	2	10	0.01	45.32
2 multi 2		H						
58	1	05/08/2021	144	1295	2	9.9	0.16	39.82
2 multi 2		H						
58	1	05/09/2021	145	1296	2	9.9	0.21	46.68
2 multi 2		H						
58	1	05/10/2021	146	1297	2	10	0.02	42.16
2 multi 2		H						
58	1	05/11/2021	147	1298	2	10.4	0	42.33
2 multi 2		H						
58	1	05/12/2021	148	1299	2	10.3	0.09	39.39
2 multi 2		H						
58	1	05/13/2021	149	1300	2	10.3	0	40.27
2 multi 2		H						
58	1	05/14/2021	150	1301	2	10.3	0.02	44.32
2 multi 2		H						
58	1	05/15/2021	151	1302	2	10.2	0.06	44.58

2 multi 2 H
58 1 05/16/2021 152 1303 2 10.3 0 46.49
2 multi 2 H
58 1 05/17/2021 153 1304 2 10.2 0 46.9
2 multi 2 H
58 1 05/18/2021 154 1305 2 10.2 0.07 44.47
2 multi 2 H
58 1 05/19/2021 155 1306 2 10.3 0 42.96
2 multi 2 H
58 1 05/20/2021 156 1307 2 9.8 0.02 43.64
2 multi 2 H
58 1 05/21/2021 157 1308 2 10.3 0.02 44.53
2 multi 2 H
58 1 05/22/2021 158 1309 2 10.4 0.02 42.16
2 multi 2 H
58 1 05/23/2021 159 1310 2 9.9 0.32 43.6
2 multi 2 H
58 1 05/24/2021 160 1311 2 9.9 0 44.45
2 multi 2 H
58 1 05/25/2021 161 1312 2 10 0 43.61
2 multi 2 H
58 1 05/26/2021 162 1313 2 9.8 0 21.02
2 multi 2 H
59 1 02/15/2021 13 729 1 9.2 0.22 24.61
1 primi 1 H
59 1 02/16/2021 14 730 1 9.8 0.08 26.52
1 primi 1 H
59 1 02/17/2021 15 731 1 9.8 0 25.22
1 primi 1 H
59 1 02/18/2021 16 732 1 9.8 0.27 25.4
1 primi 1 H
59 1 02/19/2021 17 733 1 10 0.18 25.98
1 primi 1 H
59 1 02/20/2021 18 734 1 10.2 0.06 26.07
1 primi 1 H
59 1 02/21/2021 19 735 1 9.9 0.42 25.88
1 primi 1 H
59 1 02/22/2021 20 736 1 9.6 0.14 29.08
1 primi 1 H
59 1 02/23/2021 21 737 1 10 0.27 26.08
1 primi 1 H
59 1 02/24/2021 22 738 1 9.6 0.28 30.03
1 primi 2 H
59 1 02/25/2021 23 739 1 9.9 0.37 29.93
1 primi 2 H
59 1 02/26/2021 24 740 1 9.3 0.47 29.99
1 primi 2 H
59 1 02/27/2021 25 741 1 9.3 0.02 30.37
1 primi 2 H
59 1 02/28/2021 26 742 1 9.5 0.01 30.54
1 primi 2 H
59 1 03/01/2021 27 743 1 9.1 0.1 32.68
1 primi 2 H
59 1 03/02/2021 28 744 1 9.6 0 33.03

1 primi	2	H						
59	1	03/03/2021	29	745	1	9.6	0.07	34.06
1 primi	2	H						
59	1	03/04/2021	30	746	1	9.8	0.45	33.58
1 primi	2	H						
59	1	03/05/2021	31	747	1	9.6	0.46	33.76
1 primi	2	H						
59	1	03/06/2021	32	748	1	10.3	0.28	36.12
1 primi	2	H						
59	1	03/07/2021	33	749	1	10.2	0.28	30.2
1 primi	2	H						
59	1	03/08/2021	34	750	1	9.5	0.11	33.15
1 primi	2	H						
59	1	03/09/2021	35	751	1	9.6	0.01	31.61
1 primi	2	H						
59	1	03/10/2021	36	752	1	9.8	0.11	32.57
1 primi	2	H						
59	1	03/11/2021	37	753	1	10	0.01	33.02
1 primi	2	H						
59	1	03/12/2021	38	754	1	9.9	0	35.25
1 primi	2	H						
59	1	03/13/2021	39	755	1	9.6	0.02	34.99
1 primi	2	H						
59	1	03/14/2021	40	756	1	9.3	0.01	35.11
1 primi	2	H						
59	1	03/15/2021	41	757	1	9.9	0.02	35.02
1 primi	2	H						
59	1	03/16/2021	42	758	1	9.8	0.03	34.61
1 primi	2	H						
59	1	03/17/2021	43	759	1	10	0.11	34.55
1 primi	2	H						
59	1	03/18/2021	44	760	1	10	0.1	33.79
1 primi	2	H						
59	1	03/19/2021	45	761	1	9.9	0.02	34.07
1 primi	2	H						
59	1	03/20/2021	46	762	1	9.9	0.04	34.12
1 primi	2	H						
59	1	03/21/2021	47	763	1	9.8	0.32	33.55
1 primi	2	H						
59	1	03/22/2021	48	764	1	10.2	0	35.52
1 primi	2	H						
59	1	03/23/2021	49	765	1	9.6	0.04	33.88
1 primi	2	H						
59	1	03/24/2021	50	766	1	10.2	0.02	36.67
1 primi	2	H						
59	1	03/25/2021	51	767	1	9.9	0.09	35.2
1 primi	2	H						
59	1	03/26/2021	52	768	1	10.2	0.1	36.87
1 primi	2	H						
59	1	03/27/2021	53	769	1	9.8	0.1	36.27
1 primi	2	H						
59	1	03/28/2021	54	770	1	9.3	0	35.27
1 primi	2	H						
59	1	03/29/2021	55	771	1	9.9	0.01	35.65

1 primi 2 H
59 1 03/30/2021 56 772 1 9.8 0.1 34.49
1 primi 2 H
59 1 03/31/2021 57 773 1 9.8 0 35.71
1 primi 2 H
59 1 04/01/2021 58 774 1 9.9 0.1 28.89
1 primi 2 H
59 1 04/02/2021 59 775 1 9.9 0.04 33.33
1 primi 2 H
59 1 04/03/2021 60 776 1 9.9 0.03 32.95
1 primi 2 H
59 1 04/04/2021 61 777 1 10.3 0.28 33.76
1 primi 2 H
59 1 04/05/2021 62 778 1 10.3 0.37 33.4
1 primi 2 H
59 1 04/06/2021 63 779 1 10.3 0.3 34.09
1 primi 2 H
59 1 04/07/2021 64 780 1 10 0.31 35.5
1 primi 2 H
59 1 04/08/2021 65 781 1 10 0.35 33.69
1 primi 2 H
59 1 04/09/2021 66 782 1 10 0.38 32.31
1 primi 2 H
59 1 04/10/2021 67 783 1 9.9 0 38.05
1 primi 2 H
59 1 04/11/2021 68 784 1 10.3 0.39 36.74
1 primi 2 H
59 1 04/12/2021 69 785 1 9.8 0.38 32.19
1 primi 2 H
59 1 04/13/2021 70 786 1 9.6 0.42 34.05
1 primi 2 H
59 1 04/14/2021 71 787 1 8.9 0.39 33.2
1 primi 2 H
59 1 04/15/2021 72 788 1 9.6 0.24 28.65
1 primi 2 H
65 1 09/09/2019 4 720 1 10.9 0.57 18.36
1 primi 1 H
65 1 09/10/2019 5 721 1 10.9 0.46 22.59
1 primi 1 H
65 1 09/11/2019 6 722 1 11.1 0.47 22.32
1 primi 1 H
65 1 09/12/2019 7 723 1 10.7 0.33 21.35
1 primi 1 H
65 1 09/13/2019 8 724 1 10.2 0.27 21.49
1 primi 1 H
65 1 09/14/2019 9 725 1 10.3 0.26 21.89
1 primi 1 H
65 1 09/15/2019 10 726 1 10.2 0.05 20.39
1 primi 1 H
65 1 09/16/2019 11 727 1 10.3 0.06 19.7
1 primi 1 H
65 1 09/17/2019 12 728 1 9.9 0.06 24.32
1 primi 1 H
65 1 09/18/2019 13 729 1 10 0.16 23.58

1 primi 1		H						
65	1	09/19/2019	14	730	1	9.6	0.17	23.93
1 primi 1		H						
65	1	09/20/2019	15	731	1	9.5	0.05	21.8
1 primi 1		H						
65	1	09/21/2019	16	732	1	9.5	0	25.1
1 primi 1		H						
65	1	09/22/2019	17	733	1	9.8	0.18	27.16
1 primi 1		H						
65	1	09/23/2019	18	734	1	9.6	0.17	26.84
1 primi 1		H						
65	1	09/24/2019	19	735	1	10	0.03	26.46
1 primi 1		H						
65	1	09/25/2019	20	736	1	10	0.07	25.17
1 primi 1		H						
65	1	09/26/2019	21	737	1	9.8	0.14	25.35
1 primi 1		H						
65	1	09/27/2019	22	738	1	9.6	0.01	25.21
1 primi 2		H						
65	1	09/28/2019	23	739	1	9.5	0.01	22.2
1 primi 2		H						
65	1	09/29/2019	24	740	1	9.5	0.13	22.61
1 primi 2		H						
65	1	09/30/2019	25	741	1	9.6	0.11	26.86
1 primi 2		H						
65	1	10/01/2019	26	742	1	9.8	0.05	28.75
1 primi 2		H						
65	1	10/02/2019	27	743	1	10	0.05	28.08
1 primi 2		H						
65	1	10/03/2019	28	744	1	10	0.14	26.22
1 primi 2		H						
65	1	10/04/2019	29	745	1	9.6	0.05	27.66
1 primi 2		H						
65	1	10/05/2019	30	746	1	9.3	0.14	28.7
1 primi 2		H						
65	1	10/06/2019	31	747	1	9.6	0.15	22.42
1 primi 2		H						
65	1	10/07/2019	32	748	1	9.5	0.04	28.3
1 primi 2		H						
65	1	10/08/2019	33	749	1	9.2	0.11	26.07
1 primi 2		H						
65	1	10/09/2019	34	750	1	9.6	0	28.57
1 primi 2		H						
65	1	10/10/2019	35	751	1	9.5	0	26.54
1 primi 2		H						
65	1	10/11/2019	36	752	1	9.6	0.02	25.88
1 primi 2		H						
65	1	10/12/2019	37	753	1	9.8	0.14	27.58
1 primi 2		H						
65	1	10/13/2019	38	754	1	9.5	0	24.21
1 primi 2		H						
65	1	10/14/2019	39	755	1	9.6	0.14	27.1
1 primi 2		H						
65	1	10/15/2019	40	756	1	9.9	0.04	29.09

1 primi	2	H						
65	1	10/16/2019	41	757	1	9.9	0.06	27.58
1 primi	2	H						
65	1	10/17/2019	42	758	1	9.8	0.14	28.91
1 primi	2	H						
65	1	10/18/2019	43	759	1	9.6	0	27.13
1 primi	2	H						
65	1	10/19/2019	44	760	1	9.9	0.02	29.34
1 primi	2	H						
65	1	10/20/2019	45	761	1	9.6	0.03	28.15
1 primi	2	H						
65	1	10/21/2019	46	762	1	9.8	0.04	30.43
1 primi	2	H						
65	1	10/14/2020	6	1121	2	9.9	0.37	22.73
2 multi	1	H						
65	1	10/15/2020	7	1122	2	10	0.21	31.08
2 multi	1	H						
65	1	10/16/2020	8	1123	2	10.2	0.05	33.39
2 multi	1	H						
65	1	10/17/2020	9	1124	2	10.2	0.53	32.83
2 multi	1	H						
65	1	10/18/2020	10	1125	2	10.2	0.07	36.11
2 multi	1	H						
65	1	10/19/2020	11	1126	2	10.4	0.21	35.4
2 multi	1	H						
65	1	10/20/2020	12	1127	2	10.4	0.06	31.52
2 multi	1	H						
65	1	10/21/2020	13	1128	2	10	0.17	31.43
2 multi	1	H						
65	1	10/22/2020	14	1129	2	10.3	0.09	23.09
2 multi	1	H						
65	1	10/23/2020	15	1130	2	10.2	0.16	30.82
2 multi	1	H						
65	1	10/24/2020	16	1131	2	10.3	0.14	24.78
2 multi	1	H						
65	1	10/25/2020	17	1132	2	10.3	0.1	24.36
2 multi	1	H						
65	1	10/26/2020	18	1133	2	10.4	0.02	27.62
2 multi	1	H						
65	1	10/27/2020	19	1134	2	10.3	0.08	25.66
2 multi	1	H						
65	1	10/28/2020	20	1135	2	10.3	0.09	29.46
2 multi	1	H						
65	1	10/29/2020	21	1136	2	10	0.09	23.63
2 multi	1	H						
65	1	10/30/2020	22	1137	2	10.2	0.06	31.96
2 multi	2	H						
65	1	10/31/2020	23	1138	2	10	0.1	33.28
2 multi	2	H						
65	1	11/01/2020	24	1139	2	10.3	0.17	34.28
2 multi	2	H						
65	1	11/02/2020	25	1140	2	10	0.09	29.66
2 multi	2	H						
65	1	11/03/2020	26	1141	2	10.6	0.02	39.76

2 multi	2	H						
65	1	11/04/2020	27	1142	2	10.3	0.12	34.47
2 multi	2	H						
66	1	01/04/2018	8	786	1	10.3	0.07	30.32
1 primi	1	H						
66	1	01/05/2018	9	787	1	9.8	0.04	31.96
1 primi	1	H						
66	1	01/06/2018	10	788	1	9.6	0	30.73
1 primi	1	H						
66	1	01/07/2018	11	789	1	10.3	0	33.48
1 primi	1	H						
66	1	01/08/2018	12	790	1	10	0	33.24
1 primi	1	H						
66	1	01/09/2018	13	791	1	10	0	31.7
1 primi	1	H						
66	1	01/10/2018	14	792	1	9.9	0	34.68
1 primi	1	H						
66	1	01/11/2018	15	793	1	10.2	0.03	34.97
1 primi	1	H						
66	1	01/12/2018	16	794	1	10.3	0.11	33.74
1 primi	1	H						
66	1	01/13/2018	17	795	1	9.9	0	36.99
1 primi	1	H						
66	1	01/14/2018	18	796	1	10	0	38.21
1 primi	1	H						
66	1	01/15/2018	19	797	1	9.6	0.1	35.77
1 primi	1	H						
66	1	01/16/2018	20	798	1	9.9	0.31	40.57
1 primi	1	H						
66	1	01/17/2018	21	799	1	9.6	0.19	36.38
1 primi	1	H						
66	1	01/18/2018	22	800	1	9.5	0.3	39.08
1 primi	2	H						
66	1	01/19/2018	23	801	1	9.5	0.25	38.34
1 primi	2	H						
66	1	01/20/2018	24	802	1	9.3	0.01	36.79
1 primi	2	H						
66	1	01/21/2018	25	803	1	8.9	0.13	33.95
1 primi	2	H						
66	1	01/22/2018	26	804	1	9.3	0.28	36.31
1 primi	2	H						
66	1	01/23/2018	27	805	1	9.6	0.17	35.35
1 primi	2	H						
66	1	01/24/2018	28	806	1	9.6	0.26	40.73
1 primi	2	H						
66	1	01/25/2018	29	807	1	9.9	0	43.21
1 primi	2	H						
66	1	01/26/2018	30	808	1	9.8	0	34.68
1 primi	2	H						
66	1	01/27/2018	31	809	1	9.9	0	39.17
1 primi	2	H						
66	1	01/28/2018	32	810	1	10.2	0	40.6
1 primi	2	H						
66	1	01/29/2018	33	811	1	9.9	0	37.95

1 primi	2	H						
66	1	01/30/2018	34	812	1	10	0	40.99
1 primi	2	H						
66	1	01/31/2018	35	813	1	9.9	0	38.59
1 primi	2	H						
66	1	02/01/2018	36	814	1	9.6	0.05	38.24
1 primi	2	H						
66	1	02/02/2018	37	815	1	9.6	0	38.72
1 primi	2	H						
66	1	02/03/2018	38	816	1	9.9	0	37.78
1 primi	2	H						
66	1	02/04/2018	39	817	1	9.6	0	34.83
1 primi	2	H						
66	1	02/05/2018	40	818	1	9.6	0	36.71
1 primi	2	H						
66	1	02/06/2018	41	819	1	8.8	0	32.52
1 primi	2	H						
66	1	02/07/2018	42	820	1	9.5	0	34.37
1 primi	2	H						
66	1	01/17/2019	5	1164	2	10	0.12	34.22
2 multi	1	H						
66	1	01/18/2019	6	1165	2	10.3	0.21	36.87
2 multi	1	H						
66	1	01/19/2019	7	1166	2	10	0.2	33.98
2 multi	1	H						
66	1	01/20/2019	8	1167	2	10.2	0.01	39.45
2 multi	1	H						
66	1	01/21/2019	9	1168	2	9.8	0	36.36
2 multi	1	H						
66	1	01/22/2019	10	1169	2	9.9	0.18	38.34
2 multi	1	H						
66	1	01/23/2019	11	1170	2	9.5	0	37.32
2 multi	1	H						
66	1	01/24/2019	12	1171	2	9.9	0	44.11
2 multi	1	H						
66	1	01/25/2019	13	1172	2	10.2	0	43.82
2 multi	1	H						
66	1	01/26/2019	14	1173	2	10	0	40.79
2 multi	1	H						
66	1	01/27/2019	15	1174	2	10	0	44.3
2 multi	1	H						
66	1	01/28/2019	16	1175	2	9.9	0	41.15
2 multi	1	H						
66	1	01/29/2019	17	1176	2	9.6	0	43.57
2 multi	1	H						
66	1	01/30/2019	18	1177	2	10	0.18	41.57
2 multi	1	H						
66	1	01/31/2019	19	1178	2	10.2	0	46.84
2 multi	1	H						
66	1	02/01/2019	20	1179	2	10.2	0.13	44.09
2 multi	1	H						
66	1	02/02/2019	21	1180	2	9.6	0	44.61
2 multi	1	H						
66	1	02/03/2019	22	1181	2	9.9	0	50.06

2 multi	2	H						
66	1	02/04/2019	23	1182	2	10.3	0.08	47.63
2 multi	2	H						
66	1	02/05/2019	24	1183	2	10.6	0.21	44.61
2 multi	2	H						
66	1	02/06/2019	25	1184	2	10.2	0	49.89
2 multi	2	H						
66	1	02/07/2019	26	1185	2	10.2	0	44.96
2 multi	2	H						
66	1	02/08/2019	27	1186	2	10.2	0.11	46.38
2 multi	2	H						
66	1	02/09/2019	28	1187	2	10	0	47.13
2 multi	2	H						
66	1	02/10/2019	29	1188	2	10	0	47.61
2 multi	2	H						
66	1	02/11/2019	30	1189	2	10	0	49.62
2 multi	2	H						
66	1	02/12/2019	31	1190	2	9.8	0	46.76
2 multi	2	H						
66	1	02/13/2019	32	1191	2	10.2	0	50.76
2 multi	2	H						
66	1	02/14/2019	33	1192	2	10.4	0	50.97
2 multi	2	H						
66	1	02/15/2019	34	1193	2	10.6	0	47.24
2 multi	2	H						
66	1	02/16/2019	35	1194	2	9.9	0	42.96
2 multi	2	H						
66	1	02/17/2019	36	1195	2	10.3	0	44.98
2 multi	2	H						
66	1	02/18/2019	37	1196	2	10	0.13	45.34
2 multi	2	H						
66	1	02/19/2019	38	1197	2	10.2	0	48.68
2 multi	2	H						
66	1	02/20/2019	39	1198	2	10.3	0	45.77
2 multi	2	H						
66	1	02/21/2019	40	1199	2	10.3	0	52.62
2 multi	2	H						
66	1	02/22/2019	41	1200	2	10.2	0.2	43.09
2 multi	2	H						
66	1	02/23/2019	42	1201	2	10.2	0	44.79
2 multi	2	H						
66	1	02/24/2019	43	1202	2	10.3	0	47.34
2 multi	2	H						
66	1	02/25/2019	44	1203	2	10.3	0	46.25
2 multi	2	H						
66	1	02/26/2019	45	1204	2	10.3	0	47.79
2 multi	2	H						
66	1	02/27/2019	46	1205	2	9.9	0.21	46.65
2 multi	2	H						
66	1	02/28/2019	47	1206	2	10.3	0	49.67
2 multi	2	H						
66	1	03/01/2019	48	1207	2	10.4	0	45.49
2 multi	2	H						
66	1	03/02/2019	49	1208	2	10	0	49.24

2 multi 2		H						
66	1	03/03/2019	50	1209	2	10.3	0	44.89
2 multi 2		H						
66	1	03/04/2019	51	1210	2	10.2	0	46.57
2 multi 2		H						
66	1	03/05/2019	52	1211	2	10.3	0	48.45
2 multi 2		H						
66	1	03/06/2019	53	1212	2	10	0	45.28
2 multi 2		H						
66	1	03/07/2019	54	1213	2	10.3	0.08	45.14
2 multi 2		H						
66	1	03/08/2019	55	1214	2	10.6	0	47.22
2 multi 2		H						
66	1	03/09/2019	56	1215	2	10.7	0	43.6
2 multi 2		H						
66	1	03/10/2019	57	1216	2	10.6	0.03	43.99
2 multi 2		H						
66	1	03/11/2019	58	1217	2	10.6	0	46.74
2 multi 2		H						
66	1	03/12/2019	59	1218	2	10.3	0	45.32
2 multi 2		H						
66	1	03/13/2019	60	1219	2	10.4	0	43.43
2 multi 2		H						
66	1	03/14/2019	61	1220	2	10.4	0.05	45
2 multi 2		H						
66	1	03/15/2019	62	1221	2	10.6	0	41.51
2 multi 2		H						
66	1	03/16/2019	63	1222	2	10.9	0	42.73
2 multi 2		H						
66	1	03/17/2019	64	1223	2	10.4	0	41.96
2 multi 2		H						
66	1	03/18/2019	65	1224	2	10.4	0	44.09
2 multi 2		H						
66	1	03/19/2019	66	1225	2	10.3	0	43.96
2 multi 2		H						
66	1	03/20/2019	67	1226	2	10.4	0	43.92
2 multi 2		H						
66	1	03/21/2019	68	1227	2	10.7	0	43.13
2 multi 2		H						
66	1	03/22/2019	69	1228	2	10.3	0	41.79
2 multi 2		H						
66	1	03/23/2019	70	1229	2	10.4	0	42.55
2 multi 2		H						
66	1	03/24/2019	71	1230	2	10.4	0	42.12
2 multi 2		H						
66	1	03/25/2019	72	1231	2	10.4	0	44.59
2 multi 2		H						
66	1	03/26/2019	73	1232	2	9.9	0	41.98
2 multi 2		H						
66	1	03/27/2019	74	1233	2	10.2	0	41.72
2 multi 2		H						
66	1	06/11/2020	3	1675	3	10.6	0.31	31.83
2 multi 1		H						
66	1	06/12/2020	4	1676	3	11.3	0	33.37

2 multi	1	H						
66	1	06/13/2020	5	1677	3	11.3	0.02	35.58
2 multi	1	H						
66	1	06/14/2020	6	1678	3	11.1	0	39.69
2 multi	1	H						
66	1	06/15/2020	7	1679	3	10.2	0	30.59
2 multi	1	H						
66	1	06/16/2020	8	1680	3	10.6	0.38	42.19
2 multi	1	H						
66	1	06/17/2020	9	1681	3	10.9	0.54	40.93
2 multi	1	H						
66	1	06/18/2020	10	1682	3	10.9	0.09	36.32
2 multi	1	H						
66	1	06/19/2020	11	1683	3	11.1	0	39
2 multi	1	H						
66	1	06/20/2020	12	1684	3	10.4	0.01	40.75
2 multi	1	H						
66	1	06/21/2020	13	1685	3	10.6	0.05	42.6
2 multi	1	H						
66	1	06/22/2020	14	1686	3	10.6	0	45.11
2 multi	1	H						
66	1	06/23/2020	15	1687	3	10.3	0.02	40.23
2 multi	1	H						
66	1	06/24/2020	16	1688	3	10.3	0	40.88
2 multi	1	H						
66	1	06/25/2020	17	1689	3	10.6	0	45.68
2 multi	1	H						
66	1	06/26/2020	18	1690	3	10.3	0	47.76
2 multi	1	H						
66	1	06/27/2020	19	1691	3	10.4	0	46.15
2 multi	1	H						
66	1	06/28/2020	20	1692	3	10.3	0	47.75
2 multi	1	H						
66	1	06/29/2020	21	1693	3	10	0	44.26
2 multi	1	H						
66	1	10/14/2020	128	1800	3	10.4	0.06	43.85
2 multi	2	H						
66	1	10/15/2020	129	1801	3	10.2	0.09	44.32
2 multi	2	H						
66	1	10/16/2020	130	1802	3	10.4	0.06	45.21
2 multi	2	H						
66	1	10/17/2020	131	1803	3	10.6	0.06	42.18
2 multi	2	H						
66	1	10/18/2020	132	1804	3	10.6	0.04	46.02
2 multi	2	H						
66	1	10/19/2020	133	1805	3	10.4	0.06	49.2
2 multi	2	H						
66	1	10/20/2020	134	1806	3	10.6	0	47.14
2 multi	2	H						
66	1	10/21/2020	135	1807	3	10.4	0.8	43.9
2 multi	2	H						
66	1	10/22/2020	136	1808	3	10.2	0.06	41.96
2 multi	2	H						
66	1	10/23/2020	137	1809	3	10.3	0	40.29

2 multi	2	H						
66	1	10/24/2020	138	1810	3	10	0.03	37.77
2 multi	2	H						
66	1	11/01/2020	146	1818	3	10.6	0.02	30.38
2 multi	2	H						
66	1	11/02/2020	147	1819	3	10.6	0.03	29.56
2 multi	2	H						
66	1	11/03/2020	148	1820	3	10.3	0.03	29.19
2 multi	2	H						
66	1	11/04/2020	149	1821	3	10.3	0.03	26.54
2 multi	2	H						
66	1	11/05/2020	150	1822	3	10.2	0.04	25.47
2 multi	2	H						
66	1	11/06/2020	151	1823	3	10.6	0.04	23.88
2 multi	2	H						
66	1	11/07/2020	152	1824	3	10	0.01	23.84
2 multi	2	H						
66	1	11/08/2020	153	1825	3	10.7	0.05	25.21
2 multi	2	H						
66	1	11/09/2020	154	1826	3	10.9	0.05	30.66
2 multi	2	H						
66	1	11/10/2020	155	1827	3	10.7	0.05	29.56
2 multi	2	H						
66	1	11/11/2020	156	1828	3	10.3	0.25	26.98
2 multi	2	H						
66	1	11/12/2020	157	1829	3	10.2	0.07	24.3
2 multi	2	H						
66	1	11/13/2020	158	1830	3	10.6	0.04	26.9
2 multi	2	H						
67	1	08/22/2020	8	798	1	9.3	0.21	17.92
1 primi	1	C						
67	1	08/23/2020	9	799	1	9.3	0.21	19.78
1 primi	1	C						
67	1	08/24/2020	10	800	1	9.2	0.02	19.94
1 primi	1	C						
67	1	08/25/2020	11	801	1	9.2	0.23	19.74
1 primi	1	C						
67	1	08/26/2020	12	802	1	9.3	0.17	21.84
1 primi	1	C						
67	1	08/27/2020	13	803	1	8.6	0.27	22.72
1 primi	1	C						
67	1	08/28/2020	14	804	1	9.3	0.04	20.15
1 primi	1	C						
67	1	08/29/2020	15	805	1	8.7	0	19.54
1 primi	1	C						
67	1	08/30/2020	16	806	1	8.5	0.09	20.33
1 primi	1	C						
67	1	08/31/2020	17	807	1	8.6	0.19	23.89
1 primi	1	C						
67	1	09/01/2020	18	808	1	9.1	0.09	25.57
1 primi	1	C						
67	1	09/02/2020	19	809	1	8.8	0.17	23.45
1 primi	1	C						
67	1	09/03/2020	20	810	1	8.1	0.22	23.2

1 primi	1	C						
67	1	09/04/2020	21	811	1	8.7	0.1	24.54
1 primi	1	C						
67	1	09/05/2020	22	812	1	8.7	0.05	24.66
1 primi	2	C						
67	1	09/06/2020	23	813	1	8.6	0.01	26.02
1 primi	2	C						
67	1	09/07/2020	24	814	1	8.7	0.62	26.11
1 primi	2	C						
67	1	09/08/2020	25	815	1	8.7	0.13	26.73
1 primi	2	C						
67	1	09/09/2020	26	816	1	9.1	0.08	28.94
1 primi	2	C						
67	1	09/10/2020	27	817	1	8.9	0	26.03
1 primi	2	C						
67	1	09/11/2020	28	818	1	8.9	0.21	29.12
1 primi	2	C						
67	1	09/12/2020	29	819	1	9.1	0.04	27.71
1 primi	2	C						
67	1	09/13/2020	30	820	1	9.2	0.03	28.23
1 primi	2	C						
67	1	09/14/2020	31	821	1	9.2	0	27.54
1 primi	2	C						
67	1	09/15/2020	32	822	1	8.9	0.24	28.35
1 primi	2	C						
67	1	09/16/2020	33	823	1	9.2	0.06	27.58
1 primi	2	C						
67	1	09/17/2020	34	824	1	9.3	0.06	28.17
1 primi	2	C						
67	1	09/18/2020	35	825	1	8.8	0.08	29.31
1 primi	2	C						
67	1	09/19/2020	36	826	1	8.9	0.14	28.92
1 primi	2	C						
67	1	09/20/2020	37	827	1	8.4	0.09	29.45
1 primi	2	C						
67	1	09/21/2020	38	828	1	9.2	0.07	30.03
1 primi	2	C						
67	1	09/22/2020	39	829	1	8.7	0.01	29.44
1 primi	2	C						
67	1	09/23/2020	40	830	1	8.5	0.27	28.77
1 primi	2	C						
67	1	09/24/2020	41	831	1	8.7	0.26	29.14
1 primi	2	C						
67	1	09/25/2020	42	832	1	8.6	0.02	29.48
1 primi	2	C						
67	1	09/26/2020	43	833	1	8.5	0.08	27.79
1 primi	2	C						
67	1	09/27/2020	44	834	1	8.7	0.08	29.68
1 primi	2	C						
67	1	09/28/2020	45	835	1	8.4	0	26.99
1 primi	2	C						
68	1	10/26/2019	10	748	1	9.1	0.05	20.23
1 primi	1	H						
68	1	10/27/2019	11	749	1	9.8	0	21.31

1 primi	1	H						
68	1	10/28/2019	12	750	1	9.9	0.12	21.55
1 primi	1	H						
68	1	10/29/2019	13	751	1	8.7	0.13	21.9
1 primi	1	H						
68	1	10/30/2019	14	752	1	9.5	0.14	22.45
1 primi	1	H						
68	1	10/31/2019	15	753	1	9.6	0.15	22.42
1 primi	1	H						
68	1	11/01/2019	16	754	1	9.8	0.13	24.42
1 primi	1	H						
68	1	11/02/2019	17	755	1	9.9	0.03	25.13
1 primi	1	H						
68	1	11/03/2019	18	756	1	9.3	0	24.87
1 primi	1	H						
68	1	11/04/2019	19	757	1	9.2	0.14	25.51
1 primi	1	H						
68	1	11/05/2019	20	758	1	9.2	0.04	25.16
1 primi	1	H						
68	1	11/06/2019	21	759	1	9.6	0.13	25.65
1 primi	1	H						
68	1	11/07/2019	22	760	1	9.1	0.03	25.18
1 primi	2	H						
68	1	11/08/2019	23	761	1	9.5	0.06	27.42
1 primi	2	H						
68	1	11/09/2019	24	762	1	9.3	0.01	27.96
1 primi	2	H						
68	1	11/10/2019	25	763	1	8.8	0	25.46
1 primi	2	H						
68	1	11/11/2019	26	764	1	9.1	0	24.01
1 primi	2	H						
68	1	11/12/2019	27	765	1	9.5	0	26.68
1 primi	2	H						
68	1	11/13/2019	28	766	1	9.3	0.24	26.64
1 primi	2	H						
68	1	11/14/2019	29	767	1	8.6	0.04	27.41
1 primi	2	H						
68	1	11/15/2019	30	768	1	8.9	0.03	26.73
1 primi	2	H						
68	1	11/16/2019	31	769	1	8.7	0.13	25.77
1 primi	2	H						
68	1	11/17/2019	32	770	1	9.1	0.03	26.76
1 primi	2	H						
68	1	11/18/2019	33	771	1	9.5	0	28.24
1 primi	2	H						
68	1	11/19/2019	34	772	1	9.8	0.04	28.9
1 primi	2	H						
68	1	11/20/2019	35	773	1	9.6	0.13	28.72
1 primi	2	H						
68	1	11/21/2019	36	774	1	9.6	0.38	28.11
1 primi	2	H						
68	1	11/22/2019	37	775	1	9.5	0	27.28
1 primi	2	H						
68	1	11/23/2019	38	776	1	9.3	0	28.35

1 primi 2		H						
68	1	11/24/2019	39	777	1	9.8	0.13	28.86
1 primi 2		H						
68	1	11/25/2019	40	778	1	9.6	0	28.61
1 primi 2		H						
68	1	11/26/2019	41	779	1	9.5	0	29.02
1 primi 2		H						
68	1	11/27/2019	42	780	1	9.8	0.13	27.82
1 primi 2		H						
68	1	11/28/2019	43	781	1	9.8	0.01	27.85
1 primi 2		H						
68	1	11/29/2019	44	782	1	9.6	0.01	29.13
1 primi 2		H						
68	1	11/30/2019	45	783	1	9.2	0	28.87
1 primi 2		H						
68	1	12/01/2019	46	784	1	9.3	0.1	26.85
1 primi 2		H						
68	1	12/02/2019	47	785	1	9.1	0	29.24
1 primi 2		H						
68	1	12/03/2019	48	786	1	9.3	0.02	28.43
1 primi 2		H						
68	1	12/04/2019	49	787	1	9.1	0	28.78
1 primi 2		H						
68	1	12/05/2019	50	788	1	9.6	0.09	28.08
1 primi 2		H						
68	1	12/06/2019	51	789	1	9.5	0	25.45
1 primi 2		H						
68	1	12/07/2019	52	790	1	9.3	0.08	27.27
1 primi 2		H						
68	1	12/08/2019	53	791	1	9.8	0.56	28.59
1 primi 2		H						
68	1	12/09/2019	54	792	1	9.6	0.32	29.48
1 primi 2		H						
68	1	12/10/2019	55	793	1	9.3	0.15	26.86
1 primi 2		H						
68	1	12/11/2019	56	794	1	9.5	0.21	27.34
1 primi 2		H						
68	1	12/12/2019	57	795	1	9.8	0	30.01
1 primi 2		H						
68	1	12/13/2019	58	796	1	8.9	0.2	25.45
1 primi 2		H						
68	1	12/14/2019	59	797	1	8.7	0.08	19.05
1 primi 2		H						
68	1	12/15/2019	60	798	1	9.5	0	23.7
1 primi 2		H						
68	1	12/16/2019	61	799	1	9.8	0	25.48
1 primi 2		H						
68	1	12/17/2019	62	800	1	9.8	0	23.48
1 primi 2		H						
68	1	12/18/2019	63	801	1	9.5	0.1	24.13
1 primi 2		H						
68	1	12/19/2019	64	802	1	9.6	0	23.91
1 primi 2		H						
68	1	12/20/2019	65	803	1	9.6	0	26.19

1 primi	2	H						
68	1	12/21/2019	66	804	1	9.8	0.05	25.43
1 primi	2	H						
68	1	12/22/2019	67	805	1	9.6	0.01	25.34
1 primi	2	H						
68	1	12/23/2019	68	806	1	9.8	0.09	25.8
1 primi	2	H						
68	1	12/24/2019	69	807	1	9.3	0.03	26.78
1 primi	2	H						
68	1	12/25/2019	70	808	1	9.3	0	26.83
1 primi	2	H						
69	1	07/18/2017	5	717	1	9.9	0.16	15.04
1 primi	1	H						
69	1	07/19/2017	6	718	1	9.8	0.01	15.1
1 primi	1	H						
69	1	07/20/2017	7	719	1	10	0.02	14.4
1 primi	1	H						
69	1	07/21/2017	8	720	1	9.8	0.05	15.84
1 primi	1	H						
69	1	07/22/2017	9	721	1	10.2	0	14.96
1 primi	1	H						
69	1	07/23/2017	10	722	1	10	0	16.57
1 primi	1	H						
69	1	07/24/2017	11	723	1	10.3	0.04	16.82
1 primi	1	H						
69	1	07/25/2017	12	724	1	10.2	0	17.6
1 primi	1	H						
69	1	07/26/2017	13	725	1	10.2	0.02	18.69
1 primi	1	H						
69	1	07/27/2017	14	726	1	10	0.01	19.59
1 primi	1	H						
69	1	07/28/2017	15	727	1	9.5	0	21.4
1 primi	1	H						
69	1	07/29/2017	16	728	1	9.6	0.09	22.56
1 primi	1	H						
69	1	07/30/2017	17	729	1	9.3	0	21.14
1 primi	1	H						
69	1	07/31/2017	18	730	1	9.3	0	23.98
1 primi	1	H						
69	1	08/01/2017	19	731	1	9.3	0	22.94
1 primi	1	H						
69	1	08/02/2017	20	732	1	9.3	0.03	23.56
1 primi	1	H						
69	1	08/03/2017	21	733	1	9.5	0	26.7
1 primi	1	H						
69	1	08/04/2017	22	734	1	9.3	0	26.31
1 primi	2	H						
69	1	08/05/2017	23	735	1	9.3	0	27.25
1 primi	2	H						
69	1	08/06/2017	24	736	1	9.3	0	28.91
1 primi	2	H						
69	1	08/07/2017	25	737	1	9.2	0.12	28.15
1 primi	2	H						
69	1	08/08/2017	26	738	1	9.3	0.17	29.71

1 primi	2	H						
69	1	08/09/2017	27	739	1	9.5	0.01	31.03
1 primi	2	H						
69	1	08/10/2017	28	740	1	9.3	0	30.78
1 primi	2	H						
69	1	08/11/2017	29	741	1	9.3	0	32.08
1 primi	2	H						
69	1	08/12/2017	30	742	1	9.2	0.16	29.69
1 primi	2	H						
69	1	08/13/2017	31	743	1	9.2	0	33.16
1 primi	2	H						
69	1	08/14/2017	32	744	1	9.5	0.03	32.21
1 primi	2	H						
69	1	08/15/2017	33	745	1	9.5	0.01	33.85
1 primi	2	H						
69	1	08/16/2017	34	746	1	9.2	0	32.82
1 primi	2	H						
69	1	08/17/2017	35	747	1	9.1	0	33.78
1 primi	2	H						
69	1	08/18/2017	36	748	1	9.5	0	34.08
1 primi	2	H						
69	1	08/19/2017	37	749	1	9.2	0.01	32.43
1 primi	2	H						
69	1	08/20/2017	38	750	1	8.6	0	31
1 primi	2	H						
69	1	08/21/2017	39	751	1	9.1	0.02	32.12
1 primi	2	H						
69	1	08/22/2017	40	752	1	9.1	0	31.97
1 primi	2	H						
69	1	08/23/2017	41	753	1	8.9	0	31.5
1 primi	2	H						
69	1	08/24/2017	42	754	1	9.5	0.01	33.61
1 primi	2	H						
69	1	08/25/2017	43	755	1	9.6	0	32.61
1 primi	2	H						
69	1	08/26/2017	44	756	1	9.6	0	33.28
1 primi	2	H						
69	1	08/27/2017	45	757	1	9.8	0	35.47
1 primi	2	H						
69	1	08/28/2017	46	758	1	9.5	0.02	34.71
1 primi	2	H						
69	1	08/29/2017	47	759	1	9.3	0	34.31
1 primi	2	H						
69	1	08/30/2017	48	760	1	8.9	0	35.95
1 primi	2	H						
69	1	08/31/2017	49	761	1	9.2	0	33.74
1 primi	2	H						
69	1	09/01/2017	50	762	1	9.2	0	35.89
1 primi	2	H						
69	1	09/02/2017	51	763	1	9.2	0	37.52
1 primi	2	H						
69	1	09/03/2017	52	764	1	8.9	0	35.04
1 primi	2	H						
69	1	09/04/2017	53	765	1	9.2	0	36.06

1 primi 2 H
69 1 09/05/2017 54 766 1 9.3 0.02 33.59
1 primi 2 H
69 1 09/06/2017 55 767 1 9.1 0.03 34.74
1 primi 2 H
69 1 09/07/2017 56 768 1 9.2 0 35.44
1 primi 2 H
69 1 09/08/2017 57 769 1 9.1 0 31
1 primi 2 H
69 1 09/09/2017 58 770 1 8.5 0.19 31.37
1 primi 2 H
69 1 09/10/2017 59 771 1 9.3 0.11 32.5
1 primi 2 H
69 1 09/11/2017 60 772 1 9.1 0 33.21
1 primi 2 H
69 1 09/12/2017 61 773 1 9.5 0 34.58
1 primi 2 H
69 1 09/13/2017 62 774 1 9.5 0 35.53
1 primi 2 H
69 1 09/14/2017 63 775 1 9.5 0 34.52
1 primi 2 H
69 1 09/15/2017 64 776 1 9.5 0 34.41
1 primi 2 H
69 1 09/16/2017 65 777 1 9.6 0 36.6
1 primi 2 H
69 1 09/17/2017 66 778 1 9.5 0.02 36.43
1 primi 2 H
69 1 09/18/2017 67 779 1 9.5 0 36.72
1 primi 2 H
69 1 09/19/2017 68 780 1 9.2 0.02 37.39
1 primi 2 H
69 1 09/20/2017 69 781 1 9.5 0.24 39.55
1 primi 2 H
69 1 09/21/2017 70 782 1 9.6 0.01 38.77
1 primi 2 H
69 1 09/22/2017 71 783 1 9.6 0.03 36.26
1 primi 2 H
69 1 09/23/2017 72 784 1 9.1 0 37.33
1 primi 2 H
69 1 09/24/2017 73 785 1 9.5 0.04 37.35
1 primi 2 H
69 1 09/25/2017 74 786 1 9.5 0.03 36.58
1 primi 2 H
69 1 09/26/2017 75 787 1 9.6 0 36.41
1 primi 2 H
69 1 09/27/2017 76 788 1 9.1 0 37.62
1 primi 2 H
69 1 09/28/2017 77 789 1 9.8 0.01 37.31
1 primi 2 H
69 1 09/29/2017 78 790 1 9.6 0 40.53
1 primi 2 H
69 1 09/30/2017 79 791 1 9.5 0 37.4
1 primi 2 H
69 1 10/01/2017 80 792 1 9.3 0 38.92

1 primi 2 H
69 1 10/02/2017 81 793 1 9.3 0 35.88
1 primi 2 H
69 1 10/03/2017 82 794 1 9.5 0.01 39.26
1 primi 2 H
69 1 10/04/2017 83 795 1 9.1 0 40.73
1 primi 2 H
69 1 10/05/2017 84 796 1 9.2 0 36.55
1 primi 2 H
69 1 10/06/2017 85 797 1 9.5 0.01 40.41
1 primi 2 H
69 1 10/07/2017 86 798 1 9.6 0 38.7
1 primi 2 H
69 1 10/08/2017 87 799 1 8.9 0 40.09
1 primi 2 H
69 1 10/09/2017 88 800 1 9.5 0 38.11
1 primi 2 H
69 1 10/10/2017 89 801 1 9.1 0 40.03
1 primi 2 H
69 1 10/11/2017 90 802 1 9.6 0 30.82
1 primi 2 H
69 1 10/12/2017 91 803 1 9.8 0.07 36.67
1 primi 2 H
69 1 10/13/2017 92 804 1 9.6 0 39.48
1 primi 2 H
69 1 10/14/2017 93 805 1 8.9 0 37.33
1 primi 2 H
69 1 10/15/2017 94 806 1 9.1 0 38.67
1 primi 2 H
69 1 10/16/2017 95 807 1 9.2 0 38.04
1 primi 2 H
69 1 10/17/2017 96 808 1 9.6 0 40.2
1 primi 2 H
69 1 10/18/2017 97 809 1 8.8 0 40.32
1 primi 2 H
69 1 10/19/2017 98 810 1 9.6 0.01 39.32
1 primi 2 H
69 1 10/20/2017 99 811 1 8.8 0 36.71
1 primi 2 H
69 1 10/21/2017 100 812 1 9.8 0 38.13
1 primi 2 H
69 1 10/22/2017 101 813 1 9.2 0 36.26
1 primi 2 H
69 1 10/23/2017 102 814 1 9.5 0 39.43
1 primi 2 H
69 1 10/24/2017 103 815 1 9.2 0 41.33
1 primi 2 H
69 1 10/25/2017 104 816 1 9.6 0.04 37.7
1 primi 2 H
69 1 10/26/2017 105 817 1 9.3 0.01 39.58
1 primi 2 H
69 1 10/27/2017 106 818 1 9.3 0 31.64
1 primi 2 H
69 1 10/28/2017 107 819 1 8.8 0.02 35.93

1 primi 2	H
69	1	10/29/2017	108	820	1	8.9	0.01	37.67
1 primi 2	H
69	1	10/30/2017	109	821	1	9.2	0	37.59
1 primi 2	H
69	1	10/31/2017	110	822	1	9.3	0	39.18
1 primi 2	H
69	1	11/01/2017	111	823	1	9.5	0	37.72
1 primi 2	H
69	1	11/02/2017	112	824	1	8.8	0.08	40.06
1 primi 2	H
69	1	11/03/2017	113	825	1	9.1	0	38.55
1 primi 2	H
69	1	11/04/2017	114	826	1	9.2	0	39.02
1 primi 2	H
69	1	11/05/2017	115	827	1	9.3	0.06	39.06
1 primi 2	H
69	1	11/06/2017	116	828	1	9.3	0.03	39.66
1 primi 2	H
69	1	11/07/2017	117	829	1	8.9	0	39.8
1 primi 2	H
69	1	11/08/2017	118	830	1	9.1	0.01	41.12
1 primi 2	H
69	1	11/09/2017	119	831	1	9.3	0	40.21
1 primi 2	H
69	1	11/10/2017	120	832	1	8.9	0	39.79
1 primi 2	H
69	1	11/11/2017	121	833	1	8.9	0.02	41.21
1 primi 2	H
69	1	11/12/2017	122	834	1	8.8	0.04	38.04
1 primi 2	H
69	1	11/13/2017	123	835	1	8.6	0.03	37.35
1 primi 2	H
69	1	11/14/2017	124	836	1	9.1	0	38.45
1 primi 2	H
69	1	11/15/2017	125	837	1	9.3	0.02	40.72
1 primi 2	H
69	1	11/16/2017	126	838	1	9.1	0	38.68
1 primi 2	H
69	1	11/17/2017	127	839	1	8.6	0	39.11
1 primi 2	H
69	1	11/18/2017	128	840	1	9.1	0.04	38.53
1 primi 2	H
69	1	11/19/2017	129	841	1	8.9	0.03	38.16
1 primi 2	H
69	1	11/20/2017	130	842	1	9.2	0	39.74
1 primi 2	H
69	1	11/21/2017	131	843	1	9.1	0.13	39.71
1 primi 2	H
69	1	11/22/2017	132	844	1	9.2	0.02	40.17
1 primi 2	H
69	1	11/23/2017	133	845	1	9.2	0.01	38.34
1 primi 2	H
69	1	11/24/2017	134	846	1	9.2	0	40.89

1 primi	2	H						
69	1	11/25/2017	135	847	1	9.5	0.04	38.97
1 primi	2	H						
69	1	11/26/2017	136	848	1	9.1	0.22	39.55
1 primi	2	H						
69	1	11/27/2017	137	849	1	8.9	0.05	40.45
1 primi	2	H						
69	1	11/28/2017	138	850	1	8.7	0.04	40.2
1 primi	2	H						
69	1	11/29/2017	139	851	1	9.2	0	39.24
1 primi	2	H						
69	1	11/30/2017	140	852	1	8.7	0	38.23
1 primi	2	H						
69	1	12/01/2017	141	853	1	8.9	0	36.05
1 primi	2	H						
69	1	12/02/2017	142	854	1	9.3	0	35.34
1 primi	2	H						
69	1	12/03/2017	143	855	1	9.3	0	35.66
1 primi	2	H						
69	1	12/04/2017	144	856	1	8.9	0	33.53
1 primi	2	H						
69	1	06/15/2018	8	1049	2	10	0.62	27.63
2 multi	1	H						
69	1	06/16/2018	9	1050	2	10.2	0.09	29.59
2 multi	1	H						
69	1	06/17/2018	10	1051	2	9.9	0.19	27.94
2 multi	1	H						
69	1	06/18/2018	11	1052	2	9.5	0.01	27.65
2 multi	1	H						
69	1	06/19/2018	12	1053	2	9.6	0.16	27.67
2 multi	1	H						
69	1	06/20/2018	13	1054	2	9.6	0.66	27.28
2 multi	1	H						
69	1	06/21/2018	14	1055	2	10	0	31.21
2 multi	1	H						
69	1	06/22/2018	15	1056	2	9.9	0.25	31.71
2 multi	1	H						
69	1	06/23/2018	16	1057	2	9.5	0.08	29.65
2 multi	1	H						
69	1	06/24/2018	17	1058	2	9.9	0.05	36.33
2 multi	1	H						
69	1	06/25/2018	18	1059	2	10	0.18	33.95
2 multi	1	H						
69	1	06/26/2018	19	1060	2	9.6	0.2	35.38
2 multi	1	H						
69	1	06/27/2018	20	1061	2	9.6	0.09	38.31
2 multi	1	H						
69	1	06/28/2018	21	1062	2	9.8	0	37.8
2 multi	1	H						
69	1	06/29/2018	22	1063	2	9.9	0.11	37.83
2 multi	2	H						
69	1	06/30/2018	23	1064	2	9.6	0.12	35.51
2 multi	2	H						
69	1	07/01/2018	24	1065	2	9.8	0	36.4

2 multi	2	H						
69	1	07/02/2018	25	1066	2	9.9	0.12	39.73
2 multi	2	H						
69	1	07/03/2018	26	1067	2	9.5	0.04	37.38
2 multi	2	H						
69	1	07/04/2018	27	1068	2	10	0.35	39.49
2 multi	2	H						
69	1	07/05/2018	28	1069	2	10.2	0.03	38.76
2 multi	2	H						
69	1	07/06/2018	29	1070	2	9.9	0.01	38.75
2 multi	2	H						
69	1	07/07/2018	30	1071	2	10	0.04	40.25
2 multi	2	H						
69	1	07/08/2018	31	1072	2	9.8	0.19	39.26
2 multi	2	H						
69	1	07/09/2018	32	1073	2	9.9	0.03	40.24
2 multi	2	H						
69	1	07/10/2018	33	1074	2	10.2	0.15	40.59
2 multi	2	H						
69	1	07/11/2018	34	1075	2	10.2	0	40.82
2 multi	2	H						
69	1	07/12/2018	35	1076	2	10.3	0.04	44.51
2 multi	2	H						
69	1	07/13/2018	36	1077	2	10	0	42.15
2 multi	2	H						
69	1	07/14/2018	37	1078	2	9.9	0	37.74
2 multi	2	H						
69	1	07/15/2018	38	1079	2	10.2	0.07	40.29
2 multi	2	H						
69	1	07/16/2018	39	1080	2	10.3	0.05	39.76
2 multi	2	H						
69	1	07/17/2018	40	1081	2	10	0	41.41
2 multi	2	H						
69	1	07/18/2018	41	1082	2	9.8	0.05	39.49
2 multi	2	H						
69	1	07/19/2018	42	1083	2	9.6	0	38.91
2 multi	2	H						
69	1	07/20/2018	43	1084	2	9.8	0.02	39.77
2 multi	2	H						
69	1	07/21/2018	44	1085	2	9.6	0	41.11
2 multi	2	H						
69	1	07/22/2018	45	1086	2	9.8	0	38.44
2 multi	2	H						
69	1	07/23/2018	46	1087	2	9.9	0.23	38.93
2 multi	2	H						
69	1	07/24/2018	47	1088	2	10	0.1	41.83
2 multi	2	H						
69	1	07/25/2018	48	1089	2	9.9	0.51	39.73
2 multi	2	H						
69	1	07/26/2018	49	1090	2	10	0.04	38.45
2 multi	2	H						
69	1	07/27/2018	50	1091	2	10	0	40.67
2 multi	2	H						
69	1	07/28/2018	51	1092	2	10.2	0.11	40.91

2 multi	2	H						
69	1	07/29/2018	52	1093	2	10.2	0.13	40.49
2 multi	2	H						
69	1	07/30/2018	53	1094	2	10.3	0.15	41.54
2 multi	2	H						
69	1	07/31/2018	54	1095	2	9.9	0	39.03
2 multi	2	H						
69	1	08/01/2018	55	1096	2	9.6	0.04	37.96
2 multi	2	H						
69	1	08/02/2018	56	1097	2	9.8	0.04	39.14
2 multi	2	H						
69	1	08/03/2018	57	1098	2	9.5	0.17	37.48
2 multi	2	H						
69	1	08/04/2018	58	1099	2	9.6	0.06	36.99
2 multi	2	H						
69	1	08/05/2018	59	1100	2	9.6	0.19	35.84
2 multi	2	H						
69	1	08/06/2018	60	1101	2	9.9	0	36.36
2 multi	2	H						
69	1	08/07/2018	61	1102	2	9.9	0	38.92
2 multi	2	H						
69	1	08/08/2018	62	1103	2	10	0	37.41
2 multi	2	H						
69	1	08/09/2018	63	1104	2	9.9	0.09	41.04
2 multi	2	H						
69	1	08/10/2018	64	1105	2	10	0.02	38.41
2 multi	2	H						
69	1	08/11/2018	65	1106	2	10	0	42.75
2 multi	2	H						
69	1	08/12/2018	66	1107	2	10.2	0.22	40.78
2 multi	2	H						
69	1	08/13/2018	67	1108	2	9.9	0	42.1
2 multi	2	H						
69	1	08/14/2018	68	1109	2	10.2	0	42.03
2 multi	2	H						
69	1	08/15/2018	69	1110	2	9.8	0.15	36.71
2 multi	2	H						
69	1	08/16/2018	70	1111	2	9.9	0.12	42.49
2 multi	2	H						
69	1	08/17/2018	71	1112	2	9.9	0.15	36.9
2 multi	2	H						
69	1	08/18/2018	72	1113	2	9.8	0.04	40.33
2 multi	2	H						
69	1	08/19/2018	73	1114	2	9.9	0.1	41.36
2 multi	2	H						
69	1	08/20/2018	74	1115	2	10	0.27	44.45
2 multi	2	H						
69	1	08/21/2018	75	1116	2	10.2	0.15	39.64
2 multi	2	H						
69	1	08/22/2018	76	1117	2	10.2	0.18	40.12
2 multi	2	H						
69	1	08/23/2018	77	1118	2	10.2	0.15	38.12
2 multi	2	H						
69	1	08/24/2018	78	1119	2	9.9	0.12	37.9

2 multi 2		H						
69	1	08/25/2018	79	1120	2	10.2	0	38.61
2 multi 2		H						
69	1	08/26/2018	80	1121	2	10.2	0.07	34.73
2 multi 2		H						
69	1	08/27/2018	81	1122	2	10	0.04	39.09
2 multi 2		H						
69	1	08/28/2018	82	1123	2	10.3	0	40.56
2 multi 2		H						
69	1	08/29/2018	83	1124	2	10.3	0.19	40.59
2 multi 2		H						
69	1	08/30/2018	84	1125	2	10.2	0.04	42.63
2 multi 2		H						
69	1	10/01/2018	116	1157	2	10.6	0.06	38.78
2 multi 2		H						
69	1	10/02/2018	117	1158	2	10.2	0.13	36.08
2 multi 2		H						
69	1	10/03/2018	118	1159	2	10.3	0	41.98
2 multi 2		H						
69	1	10/04/2018	119	1160	2	10	0	39.58
2 multi 2		H						
69	1	10/05/2018	120	1161	2	9.9	0.03	37.75
2 multi 2		H						
69	1	10/06/2018	121	1162	2	10.2	0.17	40.14
2 multi 2		H						
69	1	10/07/2018	122	1163	2	10.2	0.06	39.42
2 multi 2		H						
69	1	10/08/2018	123	1164	2	9.9	0	41.11
2 multi 2		H						
69	1	10/09/2018	124	1165	2	9.9	0.05	38.82
2 multi 2		H						
69	1	10/10/2018	125	1166	2	10.2	0	41.83
2 multi 2		H						
69	1	10/11/2018	126	1167	2	10	0.08	40.84
2 multi 2		H						
69	1	10/12/2018	127	1168	2	9.6	0.11	40.23
2 multi 2		H						
69	1	10/13/2018	128	1169	2	9.3	0.07	38.53
2 multi 2		H						
69	1	10/14/2018	129	1170	2	9.2	0	34.88
2 multi 2		H						
69	1	10/15/2018	130	1171	2	9.9	0	42.08
2 multi 2		H						
69	1	10/16/2018	131	1172	2	10	0	40.75
2 multi 2		H						
69	1	10/17/2018	132	1173	2	9.8	0.23	36.51
2 multi 2		H						
69	1	10/18/2018	133	1174	2	9.6	0.17	37.27
2 multi 2		H						
69	1	10/19/2018	134	1175	2	9.6	0.15	34.84
2 multi 2		H						
69	1	10/20/2018	135	1176	2	9.8	0.12	33.52
2 multi 2		H						
69	1	10/21/2018	136	1177	2	9.9	0.2	36.3

2 multi 2 H
69 1 10/22/2018 137 1178 2 9.6 0.24 30.68
2 multi 2 H
69 1 10/23/2018 138 1179 2 9.8 0.06 35.56
2 multi 2 H
69 1 10/24/2018 139 1180 2 9.5 0.09 33.16
2 multi 2 H
69 1 10/25/2018 140 1181 2 9.5 0 38.96
2 multi 2 H
69 1 10/26/2018 141 1182 2 9.5 0.05 35.08
2 multi 2 H
69 1 10/27/2018 142 1183 2 9.5 0.06 39.58
2 multi 2 H
69 1 10/28/2018 143 1184 2 9.5 0 43.59
2 multi 2 H
69 1 10/29/2018 144 1185 2 9.8 0.16 41.06
2 multi 2 H
69 1 10/30/2018 145 1186 2 9.8 0 34.34
2 multi 2 H
69 1 10/31/2018 146 1187 2 9.9 0 39.84
2 multi 2 H
69 1 11/01/2018 147 1188 2 9.5 0.28 41.43
2 multi 2 H
69 1 11/02/2018 148 1189 2 9.2 0 38.8
2 multi 2 H
69 1 11/03/2018 149 1190 2 9.8 0 35.41
2 multi 2 H
69 1 11/04/2018 150 1191 2 9.8 0 41.42
2 multi 2 H
69 1 11/05/2018 151 1192 2 9.2 0 38.21
2 multi 2 H
69 1 11/06/2018 152 1193 2 9.8 0 40.41
2 multi 2 H
69 1 11/07/2018 153 1194 2 9.9 0.06 38.61
2 multi 2 H
69 1 11/08/2018 154 1195 2 9.6 0.05 39.9
2 multi 2 H
69 1 11/09/2018 155 1196 2 9.6 0.09 39.59
2 multi 2 H
69 1 11/10/2018 156 1197 2 9.1 0 36.06
2 multi 2 H
69 1 11/11/2018 157 1198 2 9.3 0 42.19
2 multi 2 H
69 1 11/12/2018 158 1199 2 9.5 0 39.74
2 multi 2 H
69 1 11/13/2018 159 1200 2 9.5 0.08 39.7
2 multi 2 H
69 1 11/14/2018 160 1201 2 9.5 0.07 39.52
2 multi 2 H
69 1 11/15/2018 161 1202 2 9.8 0.06 39.53
2 multi 2 H
69 1 11/16/2018 162 1203 2 9.6 0.03 39.26
2 multi 2 H
69 1 11/17/2018 163 1204 2 9.9 0.07 40.74

2 multi	2	H						
69	1	11/18/2018	164	1205	2	9.9	0	37.74
2 multi	2	H						
69	1	11/19/2018	165	1206	2	10	0.07	37.96
2 multi	2	H						
69	1	11/20/2018	166	1207	2	10.6	0	38.76
2 multi	2	H						
69	1	11/21/2018	167	1208	2	10.4	0	39.17
2 multi	2	H						
69	1	11/22/2018	168	1209	2	10.6	0.06	39.55
2 multi	2	H						
69	1	11/23/2018	169	1210	2	9.9	0	36.57
2 multi	2	H						
69	1	11/24/2018	170	1211	2	10.2	0	38.85
2 multi	2	H						
69	1	11/25/2018	171	1212	2	9.6	0.09	42.15
2 multi	2	H						
69	1	11/26/2018	172	1213	2	10	0	33.21
2 multi	2	H						
69	1	11/27/2018	173	1214	2	10	0	38.83
2 multi	2	H						
69	1	11/28/2018	174	1215	2	10	0	40.54
2 multi	2	H						
69	1	11/29/2018	175	1216	2	10	0.26	40.47
2 multi	2	H						
69	1	11/30/2018	176	1217	2	9.6	0.1	38.4
2 multi	2	H						
69	1	12/01/2018	177	1218	2	10	0.07	34.89
2 multi	2	H						
69	1	12/02/2018	178	1219	2	10.2	0	34.9
2 multi	2	H						
69	1	12/03/2018	179	1220	2	9.3	0	35.64
2 multi	2	H						
69	1	12/04/2018	180	1221	2	10	0	37.67
2 multi	2	H						
69	1	12/05/2018	181	1222	2	9.9	0	39.37
2 multi	2	H						
69	1	12/06/2018	182	1223	2	10.3	0	36.53
2 multi	2	H						
69	1	12/07/2018	183	1224	2	10.3	0	36.61
2 multi	2	H						
69	1	12/08/2018	184	1225	2	8.9	0	37.12
2 multi	2	H						
69	1	12/09/2018	185	1226	2	10	0	37.7
2 multi	2	H						
69	1	12/10/2018	186	1227	2	10	0.11	35.12
2 multi	2	H						
69	1	12/11/2018	187	1228	2	10.3	0.07	39.75
2 multi	2	H						
69	1	12/12/2018	188	1229	2	10.2	0	38.38
2 multi	2	H						
69	1	12/13/2018	189	1230	2	9.8	0.06	36.71
2 multi	2	H						
69	1	12/14/2018	190	1231	2	10.3	0.06	39.93

2 multi	2	H						
69	1	12/15/2018	191	1232	2	9.9	0	33.27
2 multi	2	H						
69	1	12/16/2018	192	1233	2	10.2	0	34.36
2 multi	2	H						
69	1	12/17/2018	193	1234	2	10.2	0.06	39.76
2 multi	2	H						
69	1	05/07/2019	4	1375	3	9.6	0.25	32.5
2 multi	1	H						
69	1	05/08/2019	5	1376	3	9.3	0.13	32.05
2 multi	1	H						
69	1	05/09/2019	6	1377	3	9.5	0.23	32.01
2 multi	1	H						
69	1	05/10/2019	7	1378	3	9.5	0	37.49
2 multi	1	H						
69	1	05/11/2019	8	1379	3	9.3	0.09	40.81
2 multi	1	H						
69	1	05/12/2019	9	1380	3	9.3	0	40.89
2 multi	1	H						
69	1	05/13/2019	10	1381	3	9.3	0.19	42.51
2 multi	1	H						
69	1	05/14/2019	11	1382	3	9.2	0	40.69
2 multi	1	H						
69	1	05/15/2019	12	1383	3	9.5	0	41.23
2 multi	1	H						
69	1	05/16/2019	13	1384	3	9.3	0.52	42.19
2 multi	1	H						
69	1	05/17/2019	14	1385	3	10	0.15	43.41
2 multi	1	H						
69	1	05/18/2019	15	1386	3	9.9	0.13	43.89
2 multi	1	H						
69	1	05/19/2019	16	1387	3	9.6	0.08	46.28
2 multi	1	H						
69	1	05/20/2019	17	1388	3	9.3	0.22	46.14
2 multi	1	H						
69	1	05/21/2019	18	1389	3	10	0	46.21
2 multi	1	H						
69	1	05/22/2019	19	1390	3	10.2	0.23	47.02
2 multi	1	H						
69	1	05/23/2019	20	1391	3	10.4	0.28	46.84
2 multi	1	H						
69	1	05/24/2019	21	1392	3	11.3	0	48.57
2 multi	1	H						
69	1	05/25/2019	22	1393	3	11.1	0	48.16
2 multi	2	H						
69	1	05/26/2019	23	1394	3	11.4	0	49
2 multi	2	H						
69	1	05/27/2019	24	1395	3	12.3	0.16	47.89
2 multi	2	H						
69	1	05/28/2019	25	1396	3	11.1	0.09	48.6
2 multi	2	H						
69	1	05/29/2019	26	1397	3	11.4	0.08	48.4
2 multi	2	H						
69	1	05/30/2019	27	1398	3	11.3	0	48.2

2 multi 2		H						
69	1	05/31/2019	28	1399	3	11.3	0.25	47.89
2 multi 2		H						
69	1	06/01/2019	29	1400	3	10.7	0.1	52.06
2 multi 2		H						
69	1	06/02/2019	30	1401	3	10.9	0	50.79
2 multi 2		H						
69	1	06/03/2019	31	1402	3	11.7	0.26	50.06
2 multi 2		H						
69	1	06/04/2019	32	1403	3	11.3	0.27	55.57
2 multi 2		H						
69	1	06/05/2019	33	1404	3	11.3	0.1	52.75
2 multi 2		H						
69	1	06/06/2019	34	1405	3	10.2	0.01	50.36
2 multi 2		H						
69	1	06/07/2019	35	1406	3	10.6	0.23	51.46
2 multi 2		H						
69	1	06/08/2019	36	1407	3	10.3	0	51.09
2 multi 2		H						
69	1	06/09/2019	37	1408	3	10	0.04	53.06
2 multi 2		H						
69	1	06/10/2019	38	1409	3	10.3	0	51.45
2 multi 2		H						
69	1	06/11/2019	39	1410	3	10.2	0.28	50.59
2 multi 2		H						
69	1	06/12/2019	40	1411	3	10.4	0.15	49.83
2 multi 2		H						
69	1	06/13/2019	41	1412	3	10.6	0	48.28
2 multi 2		H						
69	1	06/14/2019	42	1413	3	10.2	0.26	49.92
2 multi 2		H						
69	1	06/15/2019	43	1414	3	10.4	0.16	48.21
2 multi 2		H						
69	1	06/16/2019	44	1415	3	10.2	0.06	48.36
2 multi 2		H						
69	1	06/17/2019	45	1416	3	10.3	0.11	48.52
2 multi 2		H						
69	1	06/18/2019	46	1417	3	10.4	0	48.42
2 multi 2		H						
69	1	06/19/2019	47	1418	3	11.3	0.08	49.12
2 multi 2		H						
69	1	06/20/2019	48	1419	3	10.9	0	49.26
2 multi 2		H						
69	1	06/21/2019	49	1420	3	10.9	0.09	45.66
2 multi 2		H						
69	1	06/22/2019	50	1421	3	10.6	0	47.15
2 multi 2		H						
69	1	06/23/2019	51	1422	3	10.2	0.06	49.72
2 multi 2		H						
69	1	06/24/2019	52	1423	3	10.4	0.06	50.64
2 multi 2		H						
69	1	06/25/2019	53	1424	3	10.3	0.14	48.56
2 multi 2		H						
69	1	06/26/2019	54	1425	3	10.6	0	51.14

2 multi	2	H						
69	1	06/27/2019	55	1426	3	10.4	0	46.58
2 multi	2	H						
69	1	06/28/2019	56	1427	3	10.4	0.12	49.44
2 multi	2	H						
69	1	06/29/2019	57	1428	3	11.1	0.22	47.39
2 multi	2	H						
69	1	06/30/2019	58	1429	3	10.7	0.22	45.75
2 multi	2	H						
69	1	07/01/2019	59	1430	3	10.4	0.12	48.76
2 multi	2	H						
69	1	07/02/2019	60	1431	3	9.9	0.26	45.56
2 multi	2	H						
69	1	07/03/2019	61	1432	3	10.7	0.06	48.65
2 multi	2	H						
69	1	07/04/2019	62	1433	3	10.2	0.13	48.06
2 multi	2	H						
69	1	07/05/2019	63	1434	3	10.2	0.25	49.75
2 multi	2	H						
69	1	07/06/2019	64	1435	3	10.4	0.23	49.53
2 multi	2	H						
69	1	07/07/2019	65	1436	3	10.3	0	48.29
2 multi	2	H						
69	1	07/08/2019	66	1437	3	10	0.07	47
2 multi	2	H						
69	1	07/09/2019	67	1438	3	10	0.06	47.02
2 multi	2	H						
69	1	07/10/2019	68	1439	3	10	0.24	45.52
2 multi	2	H						
69	1	07/11/2019	69	1440	3	10.7	0.07	48.75
2 multi	2	H						
69	1	07/12/2019	70	1441	3	10.4	0.11	42.45
2 multi	2	H						
69	1	07/13/2019	71	1442	3	11.3	0.17	48.99
2 multi	2	H						
69	1	07/14/2019	72	1443	3	11.4	0.11	50.39
2 multi	2	H						
69	1	07/15/2019	73	1444	3	11.1	0.1	51.93
2 multi	2	H						
69	1	07/16/2019	74	1445	3	10.9	0.14	51.28
2 multi	2	H						
69	1	07/17/2019	75	1446	3	11.1	0	53.24
2 multi	2	H						
69	1	07/18/2019	76	1447	3	11.1	0.15	50.18
2 multi	2	H						
69	1	07/19/2019	77	1448	3	10.6	0.28	48.06
2 multi	2	H						
69	1	07/20/2019	78	1449	3	10.4	0	48.41
2 multi	2	H						
69	1	07/21/2019	79	1450	3	10.6	0.08	49.85
2 multi	2	H						
69	1	07/22/2019	80	1451	3	10.4	0.18	52.03
2 multi	2	H						
69	1	07/23/2019	81	1452	3	9.9	0.1	50.08

2 multi	2	H						
69	1	07/24/2019	82	1453	3	10.4	0.06	46.72
2 multi	2	H						
69	1	07/25/2019	83	1454	3	10.7	0.14	48.75
2 multi	2	H						
69	1	07/26/2019	84	1455	3	9.9	0.16	48.76
2 multi	2	H						
69	1	07/27/2019	85	1456	3	9.9	0.17	43.83
2 multi	2	H						
69	1	07/28/2019	86	1457	3	10.4	0.22	45.68
2 multi	2	H						
69	1	07/29/2019	87	1458	3	10.3	0.1	47.4
2 multi	2	H						
69	1	07/30/2019	88	1459	3	10.4	0.28	45.23
2 multi	2	H						
69	1	07/31/2019	89	1460	3	10.2	0.17	49.67
2 multi	2	H						
69	1	08/01/2019	90	1461	3	10.3	0.02	45.01
2 multi	2	H						
69	1	08/02/2019	91	1462	3	10.2	0.02	45.62
2 multi	2	H						
69	1	08/03/2019	92	1463	3	10.2	0.18	43.73
2 multi	2	H						
69	1	08/04/2019	93	1464	3	10.6	0	44.64
2 multi	2	H						
69	1	08/05/2019	94	1465	3	10	0	38.15
2 multi	2	H						
69	1	08/06/2019	95	1466	3	10.2	0.08	45.44
2 multi	2	H						
69	1	08/07/2019	96	1467	3	10.3	0.07	46.36
2 multi	2	H						
69	1	08/08/2019	97	1468	3	9.8	0.03	40.41
2 multi	2	H						
69	1	08/09/2019	98	1469	3	10.3	0	45.27
2 multi	2	H						
69	1	08/10/2019	99	1470	3	11.3	0	44.22
2 multi	2	H						
69	1	08/11/2019	100	1471	3	10.9	0	43.99
2 multi	2	H						
69	1	08/12/2019	101	1472	3	10.3	0.09	46.93
2 multi	2	H						
69	1	08/13/2019	102	1473	3	10.2	0.08	44.98
2 multi	2	H						
69	1	08/14/2019	103	1474	3	10.4	0	44.82
2 multi	2	H						
69	1	08/15/2019	104	1475	3	10.9	0.23	43.49
2 multi	2	H						
69	1	08/16/2019	105	1476	3	9.8	0	43.07
2 multi	2	H						
69	1	08/17/2019	106	1477	3	10.4	0.08	45.39
2 multi	2	H						
69	1	08/18/2019	107	1478	3	10.4	0.09	47.64
2 multi	2	H						
69	1	08/19/2019	108	1479	3	10.2	0.22	44.3

2 multi 2		H						
69	1	08/20/2019	109	1480	3	10	0	42.99
2 multi 2		H						
69	1	08/21/2019	110	1481	3	10.3	0.24	41.6
2 multi 2		H						
69	1	08/22/2019	111	1482	3	10	0.16	43.62
2 multi 2		H						
69	1	08/23/2019	112	1483	3	10.4	0	45.17
2 multi 2		H						
69	1	08/24/2019	113	1484	3	10	0	45.35
2 multi 2		H						
69	1	08/25/2019	114	1485	3	10	0.13	41.25
2 multi 2		H						
69	1	08/26/2019	115	1486	3	11.1	0.02	43.25
2 multi 2		H						
69	1	08/27/2019	116	1487	3	10.4	0.07	44.55
2 multi 2		H						
69	1	08/28/2019	117	1488	3	10.3	0.27	43.27
2 multi 2		H						
69	1	08/29/2019	118	1489	3	10.3	0.07	41.09
2 multi 2		H						
69	1	08/30/2019	119	1490	3	10	0.11	41.35
2 multi 2		H						
69	1	08/31/2019	120	1491	3	10.3	0	42.95
2 multi 2		H						
69	1	09/01/2019	121	1492	3	10	0	39.45
2 multi 2		H						
69	1	09/02/2019	122	1493	3	10.2	0	42.88
2 multi 2		H						
69	1	09/03/2019	123	1494	3	10.9	0.14	41.63
2 multi 2		H						
69	1	09/04/2019	124	1495	3	10.9	0.46	41.67
2 multi 2		H						
69	1	09/05/2019	125	1496	3	10.3	0.08	42.23
2 multi 2		H						
69	1	09/06/2019	126	1497	3	10.2	0.09	40.91
2 multi 2		H						
69	1	09/07/2019	127	1498	3	10.9	0.07	43.74
2 multi 2		H						
69	1	09/08/2019	128	1499	3	11.1	0	41.91
2 multi 2		H						
69	1	09/09/2019	129	1500	3	10.7	0.05	44.77
2 multi 2		H						
69	1	09/10/2019	130	1501	3	9.9	0.08	40.6
2 multi 2		H						
69	1	09/11/2019	131	1502	3	9.9	0.26	43.17
2 multi 2		H						
69	1	09/12/2019	132	1503	3	10.2	0.02	44.4
2 multi 2		H						
69	1	09/13/2019	133	1504	3	10.4	0.08	44.71
2 multi 2		H						
69	1	09/14/2019	134	1505	3	10.2	0.38	45.83
2 multi 2		H						
69	1	09/15/2019	135	1506	3	10.4	0.21	45.08

2 multi 2		H						
69	1	09/16/2019	136	1507	3	10	0.11	40.76
2 multi 2		H						
69	1	09/17/2019	137	1508	3	10.3	0.36	45.63
2 multi 2		H						
69	1	09/18/2019	138	1509	3	10.9	0.24	46.03
2 multi 2		H						
69	1	09/19/2019	139	1510	3	11.9	0.16	47.82
2 multi 2		H						
69	1	09/20/2019	140	1511	3	10.4	0.11	45.29
2 multi 2		H						
69	1	09/21/2019	141	1512	3	11.3	0.16	46.24
2 multi 2		H						
69	1	09/22/2019	142	1513	3	10.7	0.03	47.06
2 multi 2		H						
69	1	09/23/2019	143	1514	3	10.2	0	44.98
2 multi 2		H						
69	1	09/24/2019	144	1515	3	10.3	0	46.26
2 multi 2		H						
69	1	09/25/2019	145	1516	3	10.9	0.33	48.03
2 multi 2		H						
69	1	09/26/2019	146	1517	3	10.4	0	47.86
2 multi 2		H						
69	1	09/27/2019	147	1518	3	10.3	0	44.37
2 multi 2		H						
69	1	09/28/2019	148	1519	3	10	0.08	47.64
2 multi 2		H						
69	1	09/29/2019	149	1520	3	10	0.19	44.77
2 multi 2		H						
69	1	09/30/2019	150	1521	3	10	0	45.53
2 multi 2		H						
69	1	10/01/2019	151	1522	3	11.1	0.27	45.6
2 multi 2		H						
69	1	10/02/2019	152	1523	3	10.3	0	45.92
2 multi 2		H						
69	1	10/03/2019	153	1524	3	10.9	0.08	44.78
2 multi 2		H						
69	1	10/04/2019	154	1525	3	10.9	0.15	42.98
2 multi 2		H						
69	1	10/05/2019	155	1526	3	10.4	0.2	44.68
2 multi 2		H						
69	1	10/06/2019	156	1527	3	10	0.34	45.62
2 multi 2		H						
69	1	10/07/2019	157	1528	3	9.9	0.04	47.23
2 multi 2		H						
69	1	10/08/2019	158	1529	3	9.8	0.21	45.41
2 multi 2		H						
69	1	10/09/2019	159	1530	3	10	0.04	44.43
2 multi 2		H						
69	1	10/10/2019	160	1531	3	10.6	0.08	45.71
2 multi 2		H						
69	1	10/11/2019	161	1532	3	10.7	0.01	45.96
2 multi 2		H						
69	1	10/12/2019	162	1533	3	10	0	44.38

2 multi	2	H						
69	1	10/13/2019	163	1534	3	10	0.11	43.67
2 multi	2	H						
69	1	10/14/2019	164	1535	3	10.4	0.15	46.28
2 multi	2	H						
69	1	10/15/2019	165	1536	3	10.6	0.16	46.47
2 multi	2	H						
69	1	10/16/2019	166	1537	3	9.9	0.26	45.35
2 multi	2	H						
69	1	10/17/2019	167	1538	3	10.3	0.1	43.81
2 multi	2	H						
69	1	10/18/2019	168	1539	3	10	0.16	45.04
2 multi	2	H						
69	1	10/19/2019	169	1540	3	11.3	0.06	44.16
2 multi	2	H						
69	1	10/20/2019	170	1541	3	10.7	0	46.29
2 multi	2	H						
69	1	10/21/2019	171	1542	3	10	0.11	45.65
2 multi	2	H						
69	1	10/22/2019	172	1543	3	10.4	0.08	43.33
2 multi	2	H						
69	1	10/23/2019	173	1544	3	10.4	0.63	44.07
2 multi	2	H						
69	1	10/24/2019	174	1545	3	10.9	0	41.66
2 multi	2	H						
69	1	10/25/2019	175	1546	3	11.7	0	41.8
2 multi	2	H						
69	1	10/26/2019	176	1547	3	10.9	0.51	42.93
2 multi	2	H						
69	1	10/27/2019	177	1548	3	10.2	0.01	45.96
2 multi	2	H						
69	1	10/28/2019	178	1549	3	10.2	0.1	43.6
2 multi	2	H						
69	1	10/29/2019	179	1550	3	10.2	0.15	41.26
2 multi	2	H						
69	1	10/30/2019	180	1551	3	10.7	0.13	43.29
2 multi	2	H						
69	1	10/31/2019	181	1552	3	10.7	0	43.17
2 multi	2	H						
69	1	11/01/2019	182	1553	3	10.6	0	41.77
2 multi	2	H						
69	1	11/02/2019	183	1554	3	10.2	0.02	41.91
2 multi	2	H						
69	1	11/03/2019	184	1555	3	11.1	0.05	44.58
2 multi	2	H						
69	1	11/04/2019	185	1556	3	9.5	0	36.67
2 multi	2	H						
69	1	11/05/2019	186	1557	3	11.1	0.02	44.54
2 multi	2	H						
69	1	11/06/2019	187	1558	3	10.9	0.25	43.4
2 multi	2	H						
69	1	11/07/2019	188	1559	3	11.7	0.19	40.68
2 multi	2	H						
69	1	11/08/2019	189	1560	3	10.7	0.11	42.54

2 multi 2		H						
69	1	11/09/2019	190	1561	3	10.3	0.1	39.97
2 multi 2		H						
69	1	11/10/2019	191	1562	3	10	0	43.26
2 multi 2		H						
69	1	11/11/2019	192	1563	3	9.9	0	41.2
2 multi 2		H						
69	1	11/12/2019	193	1564	3	10.4	0	43.12
2 multi 2		H						
69	1	11/13/2019	194	1565	3	10.9	0	43.1
2 multi 2		H						
69	1	11/14/2019	195	1566	3	11.1	0	40.19
2 multi 2		H						
69	1	11/15/2019	196	1567	3	11.1	0.08	41.48
2 multi 2		H						
69	1	11/16/2019	197	1568	3	10.4	0.08	41.96
2 multi 2		H						
69	1	11/17/2019	198	1569	3	10.6	0.24	40.91
2 multi 2		H						
69	1	11/18/2019	199	1570	3	9.8	0.05	43.55
2 multi 2		H						
69	1	11/19/2019	200	1571	3	12.9	0.13	36.63
2 multi 2		H						
69	1	11/20/2019	201	1572	3	10.4	0.15	34.25
2 multi 2		H						
69	1	05/19/2020	5	1753	4	10	0.27	26.9
2 multi 1		H						
69	1	05/20/2020	6	1754	4	9.9	0.24	29.26
2 multi 1		H						
69	1	05/21/2020	7	1755	4	10.2	0	29.78
2 multi 1		H						
69	1	05/22/2020	8	1756	4	10.2	0.13	31.23
2 multi 1		H						
69	1	05/23/2020	9	1757	4	10	0.28	31.7
2 multi 1		H						
69	1	05/24/2020	10	1758	4	10.3	0.1	30.34
2 multi 1		H						
69	1	05/25/2020	11	1759	4	10.6	0.09	31.66
2 multi 1		H						
69	1	05/26/2020	12	1760	4	10.6	0	32.16
2 multi 1		H						
69	1	05/27/2020	13	1761	4	10.4	0	32.15
2 multi 1		H						
69	1	05/28/2020	14	1762	4	10.6	0	37.64
2 multi 1		H						
69	1	05/29/2020	15	1763	4	10.2	0.06	36.79
2 multi 1		H						
69	1	05/30/2020	16	1764	4	10.2	0.07	38.74
2 multi 1		H						
69	1	05/31/2020	17	1765	4	10.2	0.22	39.08
2 multi 1		H						
69	1	06/01/2020	18	1766	4	10.3	0.17	40.82
2 multi 1		H						
69	1	06/02/2020	19	1767	4	9.9	0.07	40.67

2 multi	1	H						
69	1	06/03/2020	20	1768	4	10.3	0.1	46.71
2 multi	1	H						
69	1	06/04/2020	21	1769	4	10.2	0.06	45.23
2 multi	1	H						
69	1	06/05/2020	22	1770	4	9.9	0.13	44.34
2 multi	2	H						
69	1	06/06/2020	23	1771	4	9.8	0	42.56
2 multi	2	H						
69	1	06/07/2020	24	1772	4	9.9	0.01	45.11
2 multi	2	H						
69	1	06/08/2020	25	1773	4	9.8	0.24	46.3
2 multi	2	H						
69	1	06/09/2020	26	1774	4	10.2	0.23	52.75
2 multi	2	H						
69	1	06/10/2020	27	1775	4	10.3	0.08	49.53
2 multi	2	H						
69	1	06/11/2020	28	1776	4	10.2	0	53.64
2 multi	2	H						
69	1	06/12/2020	29	1777	4	9.9	0	54.41
2 multi	2	H						
69	1	06/13/2020	30	1778	4	9.8	0.16	56.68
2 multi	2	H						
69	1	06/14/2020	31	1779	4	9.9	0.13	49.92
2 multi	2	H						
69	1	06/15/2020	32	1780	4	9.8	0.19	54.99
2 multi	2	H						
69	1	06/16/2020	33	1781	4	9.6	0.02	52.51
2 multi	2	H						
69	1	06/17/2020	34	1782	4	10	0	58.79
2 multi	2	H						
69	1	06/18/2020	35	1783	4	9.8	0.13	50.95
2 multi	2	H						
69	1	06/19/2020	36	1784	4	10	0.01	56.03
2 multi	2	H						
69	1	06/20/2020	37	1785	4	9.9	0.09	56.87
2 multi	2	H						
69	1	06/21/2020	38	1786	4	9.8	0.08	54.67
2 multi	2	H						
69	1	06/22/2020	39	1787	4	9.8	0.15	53.27
2 multi	2	H						
69	1	06/23/2020	40	1788	4	9.9	0.09	60.18
2 multi	2	H						
69	1	06/24/2020	41	1789	4	9.8	0.26	52.35
2 multi	2	H						
69	1	06/25/2020	42	1790	4	9.8	0	54.59
2 multi	2	H						
69	1	06/26/2020	43	1791	4	9.8	0	57.54
2 multi	2	H						
69	1	06/27/2020	44	1792	4	9.8	0	53.52
2 multi	2	H						
69	1	06/28/2020	45	1793	4	9.6	0	54.21
2 multi	2	H						
69	1	06/29/2020	46	1794	4	9.6	0	50.72

2 multi	2	H						
69	1	10/14/2020	153	1901	4	9.5	0.15	49.83
2 multi	2	H						
69	1	10/15/2020	154	1902	4	9.3	0.11	52.38
2 multi	2	H						
69	1	10/16/2020	155	1903	4	9.8	0.05	52.57
2 multi	2	H						
69	1	10/17/2020	156	1904	4	9.6	0.25	51.19
2 multi	2	H						
69	1	10/18/2020	157	1905	4	10	0.07	50.28
2 multi	2	H						
69	1	10/19/2020	158	1906	4	9.6	0.07	50.19
2 multi	2	H						
69	1	10/20/2020	159	1907	4	9.2	0.15	45.47
2 multi	2	H						
69	1	10/21/2020	160	1908	4	9.8	0.32	51.99
2 multi	2	H						
69	1	10/22/2020	161	1909	4	9.8	0.25	51.98
2 multi	2	H						
69	1	10/23/2020	162	1910	4	10	0.04	54.2
2 multi	2	H						
69	1	10/24/2020	163	1911	4	9.9	0.27	51.49
2 multi	2	H						
69	1	10/25/2020	164	1912	4	9.9	0.24	54.26
2 multi	2	H						
69	1	10/26/2020	165	1913	4	9.8	0.22	55.75
2 multi	2	H						
69	1	10/27/2020	166	1914	4	10.2	0.02	50.91
2 multi	2	H						
69	1	10/28/2020	167	1915	4	9.8	0.28	49.59
2 multi	2	H						
69	1	10/29/2020	168	1916	4	10	0.13	53.56
2 multi	2	H						
69	1	10/30/2020	169	1917	4	9.6	0.12	53.03
2 multi	2	H						
69	1	10/31/2020	170	1918	4	9.8	0.1	53.14
2 multi	2	H						
69	1	11/01/2020	171	1919	4	9.9	0.12	55.51
2 multi	2	H						
69	1	11/02/2020	172	1920	4	9.8	0	55.53
2 multi	2	H						
69	1	11/03/2020	173	1921	4	9.6	0	51.7
2 multi	2	H						
69	1	11/04/2020	174	1922	4	9.5	0.07	55.29
2 multi	2	H						
69	1	11/05/2020	175	1923	4	9.6	0.07	51.41
2 multi	2	H						
69	1	11/06/2020	176	1924	4	9.8	0.25	50.07
2 multi	2	H						
69	1	11/07/2020	177	1925	4	9.8	0.06	52.83
2 multi	2	H						
69	1	11/08/2020	178	1926	4	9.9	0	51.88
2 multi	2	H						
69	1	11/09/2020	179	1927	4	9.8	0.18	50.31

2 multi 2		H						
69	1	11/10/2020	180	1928	4	9.8	0.13	49.94
2 multi 2		H						
69	1	11/11/2020	181	1929	4	9.9	0.63	50
2 multi 2		H						
69	1	11/12/2020	182	1930	4	9.8	0.09	53.8
2 multi 2		H						
69	1	11/13/2020	183	1931	4	10.3	0.03	56.22
2 multi 2		H						
69	1	11/14/2020	184	1932	4	10.3	0	55.02
2 multi 2		H						
69	1	11/15/2020	185	1933	4	10.2	0.12	53.74
2 multi 2		H						
69	1	11/16/2020	186	1934	4	10.3	0.22	56
2 multi 2		H						
69	1	11/17/2020	187	1935	4	10	0.1	53.7
2 multi 2		H						
69	1	11/18/2020	188	1936	4	9.9	0	58.25
2 multi 2		H						
69	1	11/19/2020	189	1937	4	9.9	0.07	52.64
2 multi 2		H						
69	1	11/20/2020	190	1938	4	9.8	0.17	54.21
2 multi 2		H						
69	1	11/21/2020	191	1939	4	9.6	0.1	52.53
2 multi 2		H						
69	1	11/22/2020	192	1940	4	9.8	0.21	53.69
2 multi 2		H						
69	1	11/23/2020	193	1941	4	9.6	0.05	53.88
2 multi 2		H						
69	1	11/24/2020	194	1942	4	9.8	0.12	54.92
2 multi 2		H						
69	1	11/25/2020	195	1943	4	9.8	0.04	51.65
2 multi 2		H						
69	1	11/26/2020	196	1944	4	9.8	0.04	52.93
2 multi 2		H						
69	1	11/27/2020	197	1945	4	9.8	0.23	51.84
2 multi 2		H						
69	1	11/28/2020	198	1946	4	9.6	0.11	54.09
2 multi 2		H						
69	1	11/29/2020	199	1947	4	9.6	0.1	53.57
2 multi 2		H						
69	1	11/30/2020	200	1948	4	9.3	0.01	49.97
2 multi 2		H						
69	1	12/01/2020	201	1949	4	9.8	0.67	52.31
2 multi 2		H						
69	1	12/02/2020	202	1950	4	9.5	0.1	51.54
2 multi 2		H						
69	1	12/03/2020	203	1951	4	9.6	0.1	53.87
2 multi 2		H						
69	1	12/04/2020	204	1952	4	9.6	0.56	51.91
2 multi 2		H						
69	1	12/05/2020	205	1953	4	9.5	0.22	50.21
2 multi 2		H						
69	1	12/06/2020	206	1954	4	9.6	0.05	51.07

2 multi 2		H						
69	1	12/07/2020	207	1955	4	9.9	0.02	54.92
2 multi 2		H						
69	1	12/08/2020	208	1956	4	10	0.09	52.15
2 multi 2		H						
69	1	12/09/2020	209	1957	4	9.9	0	53.75
2 multi 2		H						
69	1	12/10/2020	210	1958	4	10	0.36	49.92
2 multi 2		H						
69	1	12/11/2020	211	1959	4	10	0.1	49.33
2 multi 2		H						
69	1	12/12/2020	212	1960	4	10	0.1	52.01
2 multi 2		H						
69	1	12/13/2020	213	1961	4	10.2	0.07	50.71
2 multi 2		H						
69	1	12/14/2020	214	1962	4	10.2	0.12	52.98
2 multi 2		H						
69	1	12/15/2020	215	1963	4	10.3	0	49.01
2 multi 2		H						
69	1	12/16/2020	216	1964	4	10.2	0	45.31
2 multi 2		H						
69	1	12/17/2020	217	1965	4	10.2	0.05	48.15
2 multi 2		H						
69	1	12/18/2020	218	1966	4	10.2	0.03	50.6
2 multi 2		H						
69	1	12/19/2020	219	1967	4	10.4	0.11	50.43
2 multi 2		H						
69	1	12/20/2020	220	1968	4	10.2	0.24	52.2
2 multi 2		H						
69	1	12/21/2020	221	1969	4	10.2	0.35	52.5
2 multi 2		H						
69	1	12/22/2020	222	1970	4	10.4	0	49.89
2 multi 2		H						
69	1	12/23/2020	223	1971	4	10.2	0.02	49.25
2 multi 2		H						
69	1	12/24/2020	224	1972	4	10.2	0.27	49.81
2 multi 2		H						
69	1	12/25/2020	225	1973	4	10.3	0.02	51.85
2 multi 2		H						
69	1	12/26/2020	226	1974	4	10.2	0.02	52.01
2 multi 2		H						
69	1	12/27/2020	227	1975	4	10.4	0.11	51.01
2 multi 2		H						
69	1	12/28/2020	228	1976	4	10.3	0.06	51.46
2 multi 2		H						
69	1	12/29/2020	229	1977	4	10	0.1	50.11
2 multi 2		H						
69	1	12/30/2020	230	1978	4	10.3	0.09	51.91
2 multi 2		H						
69	1	12/31/2020	231	1979	4	10	0.14	50.18
2 multi 2		H						
69	1	01/01/2021	232	1980	4	10	0.26	49.51
2 multi 2		H						
69	1	01/02/2021	233	1981	4	10	0.55	48.09

2 multi	2	H						
69	1	01/03/2021	234	1982	4	9.2	0.35	53.05
2 multi	2	H						
69	1	01/04/2021	235	1983	4	10	0.11	49.33
2 multi	2	H						
69	1	01/05/2021	236	1984	4	9.9	0.03	53.07
2 multi	2	H						
69	1	01/06/2021	237	1985	4	10.3	0	51.51
2 multi	2	H						
69	1	01/07/2021	238	1986	4	10	0.14	50.05
2 multi	2	H						
69	1	01/08/2021	239	1987	4	10.2	0.02	49.9
2 multi	2	H						
69	1	01/09/2021	240	1988	4	10.3	0.19	53.13
2 multi	2	H						
69	1	01/10/2021	241	1989	4	10.3	0.14	49.17
2 multi	2	H						
69	1	01/11/2021	242	1990	4	10	0.03	49.15
2 multi	2	H						
69	1	01/12/2021	243	1991	4	10.2	0.02	51.58
2 multi	2	H						
69	1	01/13/2021	244	1992	4	10.2	0.02	51.58
2 multi	2	H						
69	1	01/14/2021	245	1993	4	10.2	0.03	48.47
2 multi	2	H						
69	1	01/15/2021	246	1994	4	10.2	0.06	50.76
2 multi	2	H						
69	1	01/16/2021	247	1995	4	10.3	0.01	51.01
2 multi	2	H						
69	1	01/17/2021	248	1996	4	9.9	0.01	49.79
2 multi	2	H						
69	1	01/18/2021	249	1997	4	10.2	0.11	51.12
2 multi	2	H						
69	1	01/19/2021	250	1998	4	10	0.09	50.06
2 multi	2	H						
69	1	01/20/2021	251	1999	4	10.3	0.02	47.91
2 multi	2	H						
69	1	01/21/2021	252	2000	4	10.2	0.01	49.34
2 multi	2	H						
69	1	01/22/2021	253	2001	4	10.2	0	49.79
2 multi	2	H						
69	1	01/23/2021	254	2002	4	10.2	0	49.41
2 multi	2	H						
69	1	01/24/2021	255	2003	4	10.2	0.02	46.76
2 multi	2	H						
69	1	01/25/2021	256	2004	4	10.3	0.05	47.81
2 multi	2	H						
69	1	01/26/2021	257	2005	4	10.3	0.1	47.59
2 multi	2	H						
69	1	01/27/2021	258	2006	4	10.3	0.12	47.48
2 multi	2	H						
69	1	01/28/2021	259	2007	4	10.4	0	46.9
2 multi	2	H						
69	1	01/29/2021	260	2008	4	10	0.13	47.77

2 multi	2	H						
69	1	01/30/2021	261	2009	4	9.3	0.01	48.07
2 multi	2	H						
69	1	01/31/2021	262	2010	4	9.3	0.14	52.69
2 multi	2	H						
69	1	02/01/2021	263	2011	4	10.2	0.1	51.06
2 multi	2	H						
69	1	02/02/2021	264	2012	4	10.2	0.02	49.48
2 multi	2	H						
69	1	02/03/2021	265	2013	4	10.3	0.21	47.6
2 multi	2	H						
69	1	02/04/2021	266	2014	4	10.2	0.12	53.73
2 multi	2	H						
69	1	02/05/2021	267	2015	4	10.3	0	50.23
2 multi	2	H						
69	1	02/06/2021	268	2016	4	10.4	0	49.42
2 multi	2	H						
69	1	02/07/2021	269	2017	4	10.2	0.23	47.96
2 multi	2	H						
69	1	02/08/2021	270	2018	4	10.2	0.01	48.49
2 multi	2	H						
69	1	02/09/2021	271	2019	4	10.2	0.03	48.42
2 multi	2	H						
69	1	02/10/2021	272	2020	4	10.2	0.05	47.04
2 multi	2	H						
69	1	02/11/2021	273	2021	4	10	0	47.49
2 multi	2	H						
69	1	02/12/2021	274	2022	4	10	0.12	51.35
2 multi	2	H						
69	1	02/13/2021	275	2023	4	9.9	0.1	47.24
2 multi	2	H						
69	1	02/14/2021	276	2024	4	8.9	0.14	45.17
2 multi	2	H						
69	1	02/15/2021	277	2025	4	10.2	0	45.71
2 multi	2	H						
69	1	02/16/2021	278	2026	4	9.9	0.09	47.06
2 multi	2	H						
69	1	02/17/2021	279	2027	4	10.2	0	47.05
2 multi	2	H						
69	1	02/18/2021	280	2028	4	10.2	0.17	50.11
2 multi	2	H						
69	1	02/19/2021	281	2029	4	10.2	0.02	45.1
2 multi	2	H						
69	1	02/20/2021	282	2030	4	10	0	44.62
2 multi	2	H						
69	1	02/21/2021	283	2031	4	10.3	0	46.9
2 multi	2	H						
69	1	02/22/2021	284	2032	4	10.2	0.07	45.39
2 multi	2	H						
69	1	02/23/2021	285	2033	4	10.2	0.23	49.76
2 multi	2	H						
69	1	02/24/2021	286	2034	4	10	0.21	47.16
2 multi	2	H						
69	1	02/25/2021	287	2035	4	10.2	0.02	48.12

2 multi	2	H						
69	1	02/26/2021	288	2036	4	10.2	0	45.25
2 multi	2	H						
69	1	02/27/2021	289	2037	4	9.9	0.25	48.74
2 multi	2	H						
69	1	02/28/2021	290	2038	4	9.2	0.02	43.62
2 multi	2	H						
69	1	03/01/2021	291	2039	4	10.3	0.12	43.82
2 multi	2	H						
69	1	03/02/2021	292	2040	4	10.3	0.15	45.38
2 multi	2	H						
69	1	03/03/2021	293	2041	4	10.3	0.1	47.98
2 multi	2	H						
69	1	03/04/2021	294	2042	4	10.3	0	44.42
2 multi	2	H						
69	1	03/05/2021	295	2043	4	10.3	0.1	46.83
2 multi	2	H						
69	1	03/06/2021	296	2044	4	10.3	0.11	45.4
2 multi	2	H						
69	1	03/07/2021	297	2045	4	10.4	0	45.85
2 multi	2	H						
69	1	03/08/2021	298	2046	4	10.4	0.02	46.79
2 multi	2	H						
69	1	03/09/2021	299	2047	4	10.3	0	42.72
2 multi	2	H						
69	1	03/10/2021	300	2048	4	9.9	0.02	44.22
2 multi	2	H						
69	1	03/11/2021	301	2049	4	10.3	0.12	41.97
2 multi	2	H						
69	1	03/12/2021	302	2050	4	10.2	0.02	41.83
2 multi	2	H						
69	1	03/13/2021	303	2051	4	10.2	0	43.02
2 multi	2	H						
69	1	03/14/2021	304	2052	4	10	0	46.73
2 multi	2	H						
69	1	03/15/2021	305	2053	4	10.3	0.03	43.85
2 multi	2	H						
69	1	03/16/2021	306	2054	4	10.3	0	43.14
2 multi	2	H						
69	1	03/17/2021	307	2055	4	10.3	0.17	44.87
2 multi	2	H						
69	1	03/18/2021	308	2056	4	10.4	0	44.54
2 multi	2	H						
69	1	03/19/2021	309	2057	4	10.4	0.01	42.47
2 multi	2	H						
69	1	03/20/2021	310	2058	4	10.4	0.1	42.75
2 multi	2	H						
69	1	03/21/2021	311	2059	4	10.2	0.18	42.98
2 multi	2	H						
69	1	03/22/2021	312	2060	4	10.2	0.09	44.22
2 multi	2	H						
69	1	03/23/2021	313	2061	4	10.3	0	43.46
2 multi	2	H						
69	1	03/24/2021	314	2062	4	10.2	0.3	43.87

2 multi 2		H						
69	1	03/25/2021	315	2063	4	10.3	0.08	44.91
2 multi 2		H						
69	1	03/26/2021	316	2064	4	10.3	0.02	42.28
2 multi 2		H						
69	1	03/27/2021	317	2065	4	10.3	0.01	44.03
2 multi 2		H						
69	1	03/28/2021	318	2066	4	10.4	0	43
2 multi 2		H						
69	1	03/29/2021	319	2067	4	10.4	0	44.06
2 multi 2		H						
69	1	03/30/2021	320	2068	4	10.6	0.06	44.68
2 multi 2		H						
69	1	03/31/2021	321	2069	4	10.4	0.09	43.17
2 multi 2		H						
69	1	04/01/2021	322	2070	4	10.4	0.09	41.47
2 multi 2		H						
69	1	04/02/2021	323	2071	4	10.6	0	42.46
2 multi 2		H						
69	1	04/03/2021	324	2072	4	10.4	0.1	44.26
2 multi 2		H						
69	1	04/04/2021	325	2073	4	10.6	0	41.46
2 multi 2		H						
69	1	04/05/2021	326	2074	4	10.4	0.19	40.5
2 multi 2		H						
69	1	04/06/2021	327	2075	4	10.3	0.09	41.27
2 multi 2		H						
69	1	04/07/2021	328	2076	4	10.3	0.1	39.75
2 multi 2		H						
69	1	04/08/2021	329	2077	4	10.3	0.13	39.46
2 multi 2		H						
69	1	04/09/2021	330	2078	4	10.3	0	43.38
2 multi 2		H						
69	1	04/10/2021	331	2079	4	10.4	0	44.07
2 multi 2		H						
69	1	04/11/2021	332	2080	4	10.3	0.21	42.29
2 multi 2		H						
69	1	04/12/2021	333	2081	4	10.3	0.26	44.27
2 multi 2		H						
69	1	04/13/2021	334	2082	4	10.7	0	43.85
2 multi 2		H						
69	1	04/14/2021	335	2083	4	10.2	0.16	40.39
2 multi 2		H						
69	1	04/15/2021	336	2084	4	10.3	0	40.16
2 multi 2		H						
69	1	04/16/2021	337	2085	4	10.6	0	42.08
2 multi 2		H						
69	1	04/17/2021	338	2086	4	10.7	0.22	43.37
2 multi 2		H						
69	1	04/18/2021	339	2087	4	10.6	0.09	42.47
2 multi 2		H						
69	1	04/19/2021	340	2088	4	10.4	0.01	44.28
2 multi 2		H						
69	1	04/20/2021	341	2089	4	10.4	0	43.14

2 multi	2	H						
69	1	04/21/2021	342	2090	4	10.6	0.09	41.52
2 multi	2	H						
69	1	04/22/2021	343	2091	4	10.4	0.22	41.05
2 multi	2	H						
69	1	04/23/2021	344	2092	4	10.3	0.26	44.41
2 multi	2	H						
69	1	04/24/2021	345	2093	4	10.4	0.19	41.45
2 multi	2	H						
69	1	04/25/2021	346	2094	4	10.4	0.02	42
2 multi	2	H						
69	1	04/26/2021	347	2095	4	10.3	0.08	42.99
2 multi	2	H						
69	1	04/27/2021	348	2096	4	10.4	0	40.34
2 multi	2	H						
69	1	04/28/2021	349	2097	4	10.4	0.21	39.82
2 multi	2	H						
69	1	04/29/2021	350	2098	4	10.4	0	46.42
2 multi	2	H						
69	1	04/30/2021	351	2099	4	10.6	0	42.09
2 multi	2	H						
69	1	05/01/2021	352	2100	4	10.4	0.08	38.89
2 multi	2	H						
69	1	05/02/2021	353	2101	4	10.7	0	40.21
2 multi	2	H						
69	1	05/03/2021	354	2102	4	10.4	0.13	40.86
2 multi	2	H						
69	1	05/04/2021	355	2103	4	10.7	0	38.86
2 multi	2	H						
69	1	05/05/2021	356	2104	4	10.6	0	39.08
2 multi	2	H						
69	1	05/06/2021	357	2105	4	10.6	0.09	38.25
2 multi	2	H						
70	1	06/21/2019	3	747	1	9.2	0.24	22.46
1 primi	1	H						
70	1	06/22/2019	4	748	1	9.9	0.09	24.97
1 primi	1	H						
70	1	06/23/2019	5	749	1	9.9	0.07	25.91
1 primi	1	H						
70	1	06/24/2019	6	750	1	9.5	0	26.23
1 primi	1	H						
70	1	06/25/2019	7	751	1	9.5	0	25.14
1 primi	1	H						
70	1	06/26/2019	8	752	1	9.8	0.06	27.61
1 primi	1	H						
70	1	06/27/2019	9	753	1	9.5	0	29.63
1 primi	1	H						
70	1	06/28/2019	10	754	1	9.5	0	27.44
1 primi	1	H						
70	1	06/29/2019	11	755	1	9.3	0	30.72
1 primi	1	H						
70	1	06/30/2019	12	756	1	9.6	0.1	30.14
1 primi	1	H						
70	1	07/01/2019	13	757	1	9.1	0	28.67

1 primi	1	H						
70	1	07/02/2019	14	758	1	9.5	0	29.82
1 primi	1	H						
70	1	07/03/2019	15	759	1	9.2	0	32.35
1 primi	1	H						
70	1	07/04/2019	16	760	1	9.2	0.27	32.34
1 primi	1	H						
70	1	07/05/2019	17	761	1	9.3	0.11	33.04
1 primi	1	H						
70	1	07/06/2019	18	762	1	9.1	0.01	33.21
1 primi	1	H						
70	1	07/07/2019	19	763	1	9.5	0.1	33.14
1 primi	1	H						
70	1	07/08/2019	20	764	1	9.3	0.28	32.84
1 primi	1	H						
70	1	07/09/2019	21	765	1	9.5	0	31.54
1 primi	1	H						
70	1	07/10/2019	22	766	1	9.3	0.1	33.04
1 primi	2	H						
70	1	07/11/2019	23	767	1	9.3	0.1	34.14
1 primi	2	H						
70	1	07/12/2019	24	768	1	9.3	0	31.94
1 primi	2	H						
70	1	07/13/2019	25	769	1	9.1	0.02	27.16
1 primi	2	H						
70	1	07/14/2019	26	770	1	9.9	0.06	34.48
1 primi	2	H						
70	1	07/15/2019	27	771	1	9.3	0.03	32.61
1 primi	2	H						
70	1	07/16/2019	28	772	1	9.6	0.01	33.37
1 primi	2	H						
70	1	07/17/2019	29	773	1	9.2	0.28	31.53
1 primi	2	H						
70	1	07/18/2019	30	774	1	9.3	0	31.91
1 primi	2	H						
70	1	07/19/2019	31	775	1	9.3	0	32.04
1 primi	2	H						
70	1	07/20/2019	32	776	1	9.5	0	34.78
1 primi	2	H						
70	1	07/21/2019	33	777	1	9.3	0.1	36.43
1 primi	2	H						
70	1	07/22/2019	34	778	1	9.3	0.01	35.38
1 primi	2	H						
70	1	07/23/2019	35	779	1	9.3	0.1	38.92
1 primi	2	H						
70	1	07/24/2019	36	780	1	9.2	0	33.66
1 primi	2	H						
70	1	07/25/2019	37	781	1	9.3	0	36.62
1 primi	2	H						
70	1	07/26/2019	38	782	1	9.2	0.21	36.09
1 primi	2	H						
70	1	07/27/2019	39	783	1	9.2	0.31	36.88
1 primi	2	H						
70	1	07/28/2019	40	784	1	9.1	0	32.82

1 primi 2 H
70 1 07/29/2019 41 785 1 9.2 0.1 35.11
1 primi 2 H
70 1 07/30/2019 42 786 1 9.2 0.1 36.46
1 primi 2 H
70 1 07/31/2019 43 787 1 9.1 0.04 37.38
1 primi 2 H
70 1 08/01/2019 44 788 1 9.3 0.22 36.89
1 primi 2 H
70 1 08/02/2019 45 789 1 9.2 0 36.99
1 primi 2 H
70 1 08/03/2019 46 790 1 9.1 0.24 35.75
1 primi 2 H
70 1 08/04/2019 47 791 1 9.2 0 35.85
1 primi 2 H
70 1 08/05/2019 48 792 1 10.3 0.01 33.75
1 primi 2 H
70 1 08/06/2019 49 793 1 9.5 0.1 33.91
1 primi 2 H
70 1 08/07/2019 50 794 1 9.2 0.11 35.63
1 primi 2 H
70 1 08/08/2019 51 795 1 9.3 0.01 36.4
1 primi 2 H
70 1 08/09/2019 52 796 1 9.2 0 31.62
1 primi 2 H
70 1 08/10/2019 53 797 1 9.3 0 33.24
1 primi 2 H
70 1 08/11/2019 54 798 1 9.2 0.19 33.66
1 primi 2 H
70 1 08/12/2019 55 799 1 10.4 0 32.72
1 primi 2 H
70 1 08/13/2019 56 800 1 10 0.03 30.87
1 primi 2 H
70 1 08/14/2019 57 801 1 11.4 0.25 34.54
1 primi 2 H
70 1 08/15/2019 58 802 1 10 0.12 32.54
1 primi 2 H
70 1 08/16/2019 59 803 1 10.3 0.11 31.71
1 primi 2 H
70 1 08/17/2019 60 804 1 10 0 32.66
1 primi 2 H
70 1 08/18/2019 61 805 1 10 0.12 33.95
1 primi 2 H
70 1 08/19/2019 62 806 1 9.8 0.11 31.11
1 primi 2 H
70 1 08/20/2019 63 807 1 10.3 0 33.51
1 primi 2 H
70 1 08/21/2019 64 808 1 10.4 0.13 22.79
1 primi 2 H
70 1 08/22/2019 65 809 1 9.8 0.13 27.21
1 primi 2 H
70 1 08/23/2019 66 810 1 10 0.12 29.42
1 primi 2 H
70 1 08/24/2019 67 811 1 10.3 0 31.68

1 primi 2 H
70 1 08/25/2019 68 812 1 10 0.15 32.47
1 primi 2 H
70 1 08/26/2019 69 813 1 10 0 30.55
1 primi 2 H
70 1 08/27/2019 70 814 1 10 0.1 34.31
1 primi 2 H
70 1 08/28/2019 71 815 1 9.9 0.1 32.74
1 primi 2 H
70 1 08/29/2019 72 816 1 10 0 32.55
1 primi 2 H
70 1 08/30/2019 73 817 1 10 0 33.09
1 primi 2 H
70 1 08/31/2019 74 818 1 10 0 33.8
1 primi 2 H
70 1 09/01/2019 75 819 1 9.8 0 31.22
1 primi 2 H
70 1 09/02/2019 76 820 1 9.5 0 27.88
1 primi 2 H
70 1 09/03/2019 77 821 1 9.6 0.23 31.85
1 primi 2 H
70 1 09/04/2019 78 822 1 9.8 0.1 27.52
1 primi 2 H
70 1 09/05/2019 79 823 1 9.6 0.07 30.87
1 primi 2 H
70 1 09/06/2019 80 824 1 8.9 0 28.28
1 primi 2 H
70 1 09/07/2019 81 825 1 9.5 0.23 31.87
1 primi 2 H
70 1 09/08/2019 82 826 1 9.8 0 29.91
1 primi 2 H
70 1 09/09/2019 83 827 1 9.6 0.49 31.52
1 primi 2 H
70 1 09/10/2019 84 828 1 9.9 0 32.26
1 primi 2 H
70 1 09/11/2019 85 829 1 10 0.11 34.75
1 primi 2 H
70 1 09/12/2019 86 830 1 9.9 0.1 34.95
1 primi 2 H
70 1 05/14/2020 6 1075 2 9.6 0.21 28.3
2 multi 1 H
70 1 05/15/2020 7 1076 2 9.8 0.14 25.69
2 multi 1 H
70 1 05/16/2020 8 1077 2 10.4 0.07 26.03
2 multi 1 H
70 1 05/17/2020 9 1078 2 9.6 0.13 28.71
2 multi 1 H
70 1 05/18/2020 10 1079 2 9.5 0.16 28.53
2 multi 1 H
70 1 05/19/2020 11 1080 2 10.2 0.14 27.45
2 multi 1 H
70 1 05/20/2020 12 1081 2 10.2 0.22 31.09
2 multi 1 H
70 1 05/21/2020 13 1082 2 10 0.18 29.67

2 multi	1	H						
70	1	05/22/2020	14	1083	2	9.5	0.05	33.27
2 multi	1	H						
70	1	05/23/2020	15	1084	2	9.6	0	33.26
2 multi	1	H						
70	1	05/24/2020	16	1085	2	9.6	0.14	31.59
2 multi	1	H						
70	1	05/25/2020	17	1086	2	9.8	0.65	35.38
2 multi	1	H						
70	1	05/26/2020	18	1087	2	10.2	0.17	27.2
2 multi	1	H						
70	1	05/27/2020	19	1088	2	9.6	0.03	31.36
2 multi	1	H						
70	1	05/28/2020	20	1089	2	9.6	0	36.2
2 multi	1	H						
70	1	05/29/2020	21	1090	2	9.8	0	37.57
2 multi	1	H						
70	1	05/30/2020	22	1091	2	9.6	0.12	32.57
2 multi	2	H						
70	1	05/31/2020	23	1092	2	10.3	0.05	31.98
2 multi	2	H						
70	1	06/01/2020	24	1093	2	9.8	0.12	35.83
2 multi	2	H						
70	1	06/02/2020	25	1094	2	9.9	0.04	38.54
2 multi	2	H						
70	1	06/03/2020	26	1095	2	9.9	0.05	37.77
2 multi	2	H						
70	1	06/04/2020	27	1096	2	9.8	0	33.78
2 multi	2	H						
70	1	06/05/2020	28	1097	2	9.3	0	33.11
2 multi	2	H						
70	1	06/06/2020	29	1098	2	9.2	0	35.41
2 multi	2	H						
70	1	06/07/2020	30	1099	2	9.3	0	35.2
2 multi	2	H						
70	1	06/08/2020	31	1100	2	9.5	0.02	36.13
2 multi	2	H						
70	1	06/09/2020	32	1101	2	9.8	0.15	29.68
2 multi	2	H						
70	1	06/10/2020	33	1102	2	10.3	0.12	35.8
2 multi	2	H						
70	1	06/11/2020	34	1103	2	10.2	0	37.04
2 multi	2	H						
70	1	06/12/2020	35	1104	2	10	0.08	34.08
2 multi	2	H						
70	1	06/13/2020	36	1105	2	9.6	0	34.62
2 multi	2	H						
70	1	06/14/2020	37	1106	2	10	0.76	35.74
2 multi	2	H						
70	1	06/15/2020	38	1107	2	10.2	0.21	37.71
2 multi	2	H						
70	1	06/16/2020	39	1108	2	9.6	0.08	34.76
2 multi	2	H						
70	1	06/17/2020	40	1109	2	9.9	0.28	39.09

2 multi 2		H						
70	1	06/18/2020	41	1110	2	9.9	0.01	38.03
2 multi 2		H						
70	1	06/19/2020	42	1111	2	10.3	0.35	39.88
2 multi 2		H						
70	1	06/20/2020	43	1112	2	10	0.09	34.13
2 multi 2		H						
70	1	06/21/2020	44	1113	2	9.9	0.48	40.34
2 multi 2		H						
70	1	06/22/2020	45	1114	2	9.8	0	37.92
2 multi 2		H						
70	1	06/23/2020	46	1115	2	10	0.1	37.4
2 multi 2		H						
70	1	06/24/2020	47	1116	2	10.4	0	33.62
2 multi 2		H						
70	1	06/25/2020	48	1117	2	10.7	0	38.09
2 multi 2		H						
70	1	06/26/2020	49	1118	2	10.4	0	35.31
2 multi 2		H						
70	1	06/27/2020	50	1119	2	10.4	0	34.84
2 multi 2		H						
70	1	06/28/2020	51	1120	2	10.6	0	32.8
2 multi 2		H						
70	1	06/29/2020	52	1121	2	10.6	0	37.12
2 multi 2		H						
70	1	06/30/2020	53	1122	2	10.2	0	33.92
2 multi 2		H						
70	1	07/01/2020	54	1123	2	9.9	0	32.97
2 multi 2		H						
70	1	07/02/2020	55	1124	2	10	0	39.68
2 multi 2		H						
70	1	07/03/2020	56	1125	2	9.5	0	35.89
2 multi 2		H						
70	1	07/04/2020	57	1126	2	9.8	0.16	39.51
2 multi 2		H						
70	1	07/05/2020	58	1127	2	9.6	0.55	32.07
2 multi 2		H						
70	1	07/06/2020	59	1128	2	10	0.16	36.07
2 multi 2		H						
70	1	07/07/2020	60	1129	2	10	0.14	35.37
2 multi 2		H						
70	1	07/08/2020	61	1130	2	9.8	0.11	35.93
2 multi 2		H						
70	1	07/09/2020	62	1131	2	10	0.02	38.77
2 multi 2		H						
70	1	07/10/2020	63	1132	2	10.2	0	39.08
2 multi 2		H						
70	1	07/11/2020	64	1133	2	10	0.11	36.69
2 multi 2		H						
70	1	07/12/2020	65	1134	2	10.2	0.18	35.95
2 multi 2		H						
70	1	07/13/2020	66	1135	2	9.8	0.1	36.47
2 multi 2		H						
70	1	07/14/2020	67	1136	2	9.3	0	34.3

2 multi	2	H						
70	1	07/15/2020	68	1137	2	9.9	0.14	39.22
2 multi	2	H						
70	1	07/16/2020	69	1138	2	9.8	0	35.94
2 multi	2	H						
70	1	07/17/2020	70	1139	2	9.9	0.48	35.85
2 multi	2	H						
70	1	07/18/2020	71	1140	2	9.3	0.04	35.49
2 multi	2	H						
70	1	07/19/2020	72	1141	2	9.5	0.1	41.04
2 multi	2	H						
70	1	07/20/2020	73	1142	2	10.4	0.1	32.28
2 multi	2	H						
70	1	07/21/2020	74	1143	2	9.9	0.07	21.9
2 multi	2	H						
70	1	07/22/2020	75	1144	2	10.4	0.08	34.45
2 multi	2	H						
70	1	07/23/2020	76	1145	2	10.3	0.15	29.37
2 multi	2	H						
70	1	07/24/2020	77	1146	2	10.3	0.01	30.86
2 multi	2	H						
70	1	07/25/2020	78	1147	2	10.3	0.01	33.13
2 multi	2	H						
70	1	07/26/2020	79	1148	2	10.2	0.28	34.69
2 multi	2	H						
70	1	07/27/2020	80	1149	2	10.4	0.11	35.71
2 multi	2	H						
70	1	07/28/2020	81	1150	2	10.2	0.59	31.66
2 multi	2	H						
70	1	07/29/2020	82	1151	2	10.2	0.02	35.79
2 multi	2	H						
70	1	07/30/2020	83	1152	2	9.9	0.11	33.88
2 multi	2	H						
70	1	07/31/2020	84	1153	2	10.2	0.26	33.32
2 multi	2	H						
70	1	08/01/2020	85	1154	2	10	0.1	36.4
2 multi	2	H						
70	1	08/02/2020	86	1155	2	10.2	0	34.71
2 multi	2	H						
70	1	08/03/2020	87	1156	2	10.3	0	34.15
2 multi	2	H						
70	1	08/04/2020	88	1157	2	10.4	0.01	33.14
2 multi	2	H						
70	1	08/05/2020	89	1158	2	10.7	0.01	32.01
2 multi	2	H						
70	1	08/06/2020	90	1159	2	10.6	0.02	32.76
2 multi	2	H						
70	1	08/07/2020	91	1160	2	11.4	0.03	31.82
2 multi	2	H						
70	1	08/08/2020	92	1161	2	10.4	0.04	34.32
2 multi	2	H						
70	1	08/09/2020	93	1162	2	10.4	0.41	36.73
2 multi	2	H						
71	1	10/29/2018	5	730	1	8.9	0.17	21.38

1 primi	1	H						
71	1	10/30/2018	6	731	1	10	0.11	22.71
1 primi	1	H						
71	1	10/31/2018	7	732	1	9.3	0.07	22.34
1 primi	1	H						
71	1	11/01/2018	8	733	1	9.2	0.07	23.32
1 primi	1	H						
71	1	11/02/2018	9	734	1	9.3	0.05	25.34
1 primi	1	H						
71	1	11/03/2018	10	735	1	9.5	0.03	25.2
1 primi	1	H						
71	1	11/04/2018	11	736	1	8.9	0.01	25.7
1 primi	1	H						
71	1	11/05/2018	12	737	1	9.1	0	26.17
1 primi	1	H						
71	1	11/06/2018	13	738	1	9.1	0	27.19
1 primi	1	H						
71	1	11/07/2018	14	739	1	8.9	0.12	26.57
1 primi	1	H						
71	1	11/08/2018	15	740	1	9.1	0.14	25.15
1 primi	1	H						
71	1	11/09/2018	16	741	1	8.9	0.24	26.9
1 primi	1	H						
71	1	11/10/2018	17	742	1	9.3	0.28	27.53
1 primi	1	H						
71	1	11/11/2018	18	743	1	9.5	0.25	28.42
1 primi	1	H						
71	1	11/12/2018	19	744	1	9.2	0.01	28.55
1 primi	1	H						
71	1	11/13/2018	20	745	1	8.9	0.83	30.68
1 primi	1	H						
71	1	11/14/2018	21	746	1	9.1	0.42	27.68
1 primi	1	H						
71	1	11/15/2018	22	747	1	8.9	0	26.78
1 primi	2	H						
71	1	11/16/2018	23	748	1	9.1	0.15	27.91
1 primi	2	H						
71	1	11/17/2018	24	749	1	8.9	0.04	28.13
1 primi	2	H						
71	1	11/18/2018	25	750	1	8.9	0	29.85
1 primi	2	H						
71	1	11/19/2018	26	751	1	9.5	0	31.23
1 primi	2	H						
71	1	11/20/2018	27	752	1	9.3	0	28.66
1 primi	2	H						
71	1	11/21/2018	28	753	1	9.5	0	31.66
1 primi	2	H						
71	1	11/22/2018	29	754	1	9.5	0	30.68
1 primi	2	H						
71	1	11/23/2018	30	755	1	9.9	0.1	32.25
1 primi	2	H						
71	1	11/24/2018	31	756	1	9.3	0	33.22
1 primi	2	H						
71	1	11/25/2018	32	757	1	9.1	0.13	29.68

1 primi 2		H						
71	1	11/26/2018	33	758	1	9.8	0.12	32.53
1 primi 2		H						
71	1	11/27/2018	34	759	1	9.2	0.09	31.63
1 primi 2		H						
71	1	11/28/2018	35	760	1	9.3	0.42	30.71
1 primi 2		H						
71	1	11/29/2018	36	761	1	9.3	0.27	32.62
1 primi 2		H						
71	1	11/30/2018	37	762	1	9.5	0.16	33.34
1 primi 2		H						
71	1	12/01/2018	38	763	1	9.6	0	31.21
1 primi 2		H						
71	1	12/02/2018	39	764	1	9.6	0	29.93
1 primi 2		H						
71	1	12/03/2018	40	765	1	9.8	0	32.68
1 primi 2		H						
71	1	12/04/2018	41	766	1	9.9	0.43	30.73
1 primi 2		H						
71	1	12/05/2018	42	767	1	9.3	0	29.44
1 primi 2		H						
71	1	10/18/2019	4	1084	2	9.9	0.84	28.75
2 multi 1		H						
71	1	10/19/2019	5	1085	2	10	0.23	32.47
2 multi 1		H						
71	1	10/20/2019	6	1086	2	10	0.33	32.1
2 multi 1		H						
71	1	10/21/2019	7	1087	2	10.3	0.01	34.56
2 multi 1		H						
71	1	10/22/2019	8	1088	2	10.4	0.17	37.89
2 multi 1		H						
71	1	10/23/2019	9	1089	2	10.2	0.28	37.49
2 multi 1		H						
71	1	10/24/2019	10	1090	2	10.3	0.02	35.48
2 multi 1		H						
71	1	10/25/2019	11	1091	2	10.3	0.17	38.9
2 multi 1		H						
71	1	10/26/2019	12	1092	2	9.9	0.03	38.91
2 multi 1		H						
71	1	10/27/2019	13	1093	2	10.2	0.01	38.39
2 multi 1		H						
71	1	10/28/2019	14	1094	2	10.2	0.01	40.49
2 multi 1		H						
71	1	10/29/2019	15	1095	2	9.8	0.3	38.93
2 multi 1		H						
71	1	10/30/2019	16	1096	2	9.8	0.24	39.53
2 multi 1		H						
71	1	10/31/2019	17	1097	2	10.3	0.28	42.92
2 multi 1		H						
71	1	11/01/2019	18	1098	2	10.2	0.06	35.95
2 multi 1		H						
71	1	11/02/2019	19	1099	2	10.6	0.31	40.98
2 multi 1		H						
71	1	11/03/2019	20	1100	2	10.3	0.11	43.54

2 multi	1	H						
71	1	11/04/2019	21	1101	2	10	0.09	40.27
2 multi	1	H						
71	1	11/05/2019	22	1102	2	10.3	0.11	40.31
2 multi	2	H						
71	1	11/06/2019	23	1103	2	10.6	0	39.6
2 multi	2	H						
71	1	11/07/2019	24	1104	2	10.2	0.11	39.45
2 multi	2	H						
71	1	11/08/2019	25	1105	2	10	0	40.33
2 multi	2	H						
71	1	11/09/2019	26	1106	2	10.2	0.01	43.84
2 multi	2	H						
71	1	11/10/2019	27	1107	2	10.3	0	38.18
2 multi	2	H						
71	1	11/11/2019	28	1108	2	10	0	40.9
2 multi	2	H						
71	1	11/12/2019	29	1109	2	10	0	38.81
2 multi	2	H						
71	1	11/13/2019	30	1110	2	10.3	0.1	42.93
2 multi	2	H						
71	1	11/14/2019	31	1111	2	10	0.28	40.04
2 multi	2	H						
71	1	11/15/2019	32	1112	2	9.9	0.24	41.92
2 multi	2	H						
71	1	11/16/2019	33	1113	2	10	0	42.21
2 multi	2	H						
71	1	11/17/2019	34	1114	2	10	0.03	45.63
2 multi	2	H						
71	1	11/18/2019	35	1115	2	10	0	44.44
2 multi	2	H						
71	1	11/19/2019	36	1116	2	9.9	0.1	46.21
2 multi	2	H						
71	1	11/20/2019	37	1117	2	10.2	0.09	41.06
2 multi	2	H						
71	1	11/21/2019	38	1118	2	10	0	43.57
2 multi	2	H						
71	1	11/22/2019	39	1119	2	10.3	0	42.05
2 multi	2	H						
71	1	11/23/2019	40	1120	2	10.2	0.08	38.51
2 multi	2	H						
71	1	11/24/2019	41	1121	2	10.2	0.06	36.35
2 multi	2	H						
71	1	11/25/2019	42	1122	2	10.2	0.08	39.83
2 multi	2	H						
71	1	11/26/2019	43	1123	2	10.2	0.13	40.23
2 multi	2	H						
71	1	11/27/2019	44	1124	2	10.4	0	41.61
2 multi	2	H						
71	1	11/28/2019	45	1125	2	10.6	0.06	40.23
2 multi	2	H						
71	1	11/29/2019	46	1126	2	10	0.21	37.39
2 multi	2	H						
71	1	11/30/2019	47	1127	2	10.4	0.07	38.66

2 multi	2	H						
71	1	12/01/2019	48	1128	2	10.3	0.03	42.75
2 multi	2	H						
71	1	12/02/2019	49	1129	2	10.4	0.08	42.29
2 multi	2	H						
71	1	12/03/2019	50	1130	2	10.3	0	40
2 multi	2	H						
71	1	12/04/2019	51	1131	2	10.4	0	41.69
2 multi	2	H						
71	1	12/05/2019	52	1132	2	10	0	35.47
2 multi	2	H						
71	1	12/06/2019	53	1133	2	10.3	0.24	35.33
2 multi	2	H						
71	1	12/07/2019	54	1134	2	10.3	0.25	38.02
2 multi	2	H						
71	1	12/08/2019	55	1135	2	10.4	0.08	39.94
2 multi	2	H						
71	1	12/09/2019	56	1136	2	10.4	0.07	38.46
2 multi	2	H						
71	1	12/10/2019	57	1137	2	10	0.2	39.08
2 multi	2	H						
71	1	12/11/2019	58	1138	2	9.8	0	36.7
2 multi	2	H						
71	1	12/12/2019	59	1139	2	10.3	0.39	35.91
2 multi	2	H						
71	1	12/13/2019	60	1140	2	10.2	0	36.04
2 multi	2	H						
71	1	12/14/2019	61	1141	2	10.6	0.24	40.44
2 multi	2	H						
71	1	12/15/2019	62	1142	2	10.4	0	38.58
2 multi	2	H						
71	1	12/16/2019	63	1143	2	10.2	0.22	39.3
2 multi	2	H						
71	1	12/17/2019	64	1144	2	10	0.24	37.23
2 multi	2	H						
71	1	12/18/2019	65	1145	2	9.8	0.18	38.57
2 multi	2	H						
71	1	12/19/2019	66	1146	2	10	0.12	35.68
2 multi	2	H						
71	1	12/20/2019	67	1147	2	10.2	0	38.28
2 multi	2	H						
71	1	12/21/2019	68	1148	2	10	0	39.67
2 multi	2	H						
71	1	12/22/2019	69	1149	2	10.6	0.01	38.36
2 multi	2	H						
71	1	12/23/2019	70	1150	2	9.9	0	38.1
2 multi	2	H						
71	1	12/24/2019	71	1151	2	10	0.03	39.52
2 multi	2	H						
71	1	12/25/2019	72	1152	2	10.2	0.13	38.09
2 multi	2	H						
71	1	12/26/2019	73	1153	2	10.3	0	38.82
2 multi	2	H						
71	1	12/27/2019	74	1154	2	9.8	0.27	37.57

2 multi	2	H						
71	1	12/28/2019	75	1155	2	10	0.33	37.9
2 multi	2	H						
71	1	12/29/2019	76	1156	2	10	0.62	36.93
2 multi	2	H						
71	1	12/30/2019	77	1157	2	10	0	29.24
2 multi	2	H						
71	1	12/31/2019	78	1158	2	10.7	0.07	36.74
2 multi	2	H						
72	1	03/08/2020	7	846	1	9.3	0.28	18.88
1 primi	1	H						
72	1	03/09/2020	8	847	1	9.9	0.09	22.12
1 primi	1	H						
72	1	03/10/2020	9	848	1	9.9	0.03	20.87
1 primi	1	H						
72	1	03/11/2020	10	849	1	9.6	0.02	21.01
1 primi	1	H						
72	1	03/12/2020	11	850	1	9.9	0	22.08
1 primi	1	H						
72	1	03/13/2020	12	851	1	9.8	0.08	21.84
1 primi	1	H						
72	1	03/14/2020	13	852	1	9.5	0	22.53
1 primi	1	H						
72	1	03/15/2020	14	853	1	9.5	0	22.87
1 primi	1	H						
72	1	03/16/2020	15	854	1	9.2	0.06	23.38
1 primi	1	H						
72	1	03/17/2020	16	855	1	9.5	0.02	24.18
1 primi	1	H						
72	1	03/18/2020	17	856	1	9.2	0.09	21.32
1 primi	1	H						
72	1	03/19/2020	18	857	1	9.3	0.08	25.39
1 primi	1	H						
72	1	03/20/2020	19	858	1	9.1	0.06	25.74
1 primi	1	H						
72	1	03/21/2020	20	859	1	9.1	0	25.41
1 primi	1	H						
72	1	03/22/2020	21	860	1	8.7	0.05	24.2
1 primi	1	H						
72	1	03/23/2020	22	861	1	9.2	0.06	25.22
1 primi	2	H						
72	1	03/24/2020	23	862	1	8.6	0	26.29
1 primi	2	H						
72	1	03/25/2020	24	863	1	9.3	0.04	26.46
1 primi	2	H						
72	1	03/26/2020	25	864	1	9.1	0.03	25.51
1 primi	2	H						
72	1	03/27/2020	26	865	1	9.2	0.04	27.64
1 primi	2	H						
72	1	03/28/2020	27	866	1	9.1	0.34	27.92
1 primi	2	H						
72	1	03/29/2020	28	867	1	9.1	0	25.88
1 primi	2	H						
72	1	03/30/2020	29	868	1	8.9	0.04	27.56

1 primi	2	H						
72	1	03/31/2020	30	869	1	9.1	0.08	26.03
1 primi	2	H						
72	1	04/01/2020	31	870	1	8.7	0.02	26.11
1 primi	2	H						
72	1	04/02/2020	32	871	1	8.9	0.02	26.08
1 primi	2	H						
72	1	04/03/2020	33	872	1	8.9	0.03	27.62
1 primi	2	H						
72	1	04/04/2020	34	873	1	9.1	0.03	27.97
1 primi	2	H						
72	1	04/05/2020	35	874	1	9.1	0.02	26.36
1 primi	2	H						
72	1	04/06/2020	36	875	1	8.9	0.01	27.98
1 primi	2	H						
72	1	04/07/2020	37	876	1	8.9	0	27.11
1 primi	2	H						
72	1	04/08/2020	38	877	1	8.9	0.01	31.84
1 primi	2	H						
72	1	04/09/2020	39	878	1	9.3	0	25.5
1 primi	2	H						
72	1	04/10/2020	40	879	1	9.3	0	29.59
1 primi	2	H						
72	1	04/11/2020	41	880	1	9.5	0.01	26.42
1 primi	2	H						
72	1	04/12/2020	42	881	1	9.2	0	27.38
1 primi	2	H						
72	1	04/13/2020	43	882	1	8.9	0	28.66
1 primi	2	H						
72	1	04/14/2020	44	883	1	8.8	0	28.39
1 primi	2	H						
72	1	04/15/2020	45	884	1	8.8	0	28.89
1 primi	2	H						
73	1	10/25/2017	4	727	1	9.5	0.11	24.84
1 primi	1	H						
73	1	10/26/2017	5	728	1	9.5	0.12	23.8
1 primi	1	H						
73	1	10/27/2017	6	729	1	8.9	0.21	25.44
1 primi	1	H						
73	1	10/28/2017	7	730	1	9.3	0	28.05
1 primi	1	H						
73	1	10/29/2017	8	731	1	9.5	0	29.3
1 primi	1	H						
73	1	10/30/2017	9	732	1	8.7	0	31.76
1 primi	1	H						
73	1	10/31/2017	10	733	1	8.8	0	32.72
1 primi	1	H						
73	1	11/01/2017	11	734	1	9.3	0	33.18
1 primi	1	H						
73	1	11/02/2017	12	735	1	9.2	0	31.49
1 primi	1	H						
73	1	11/03/2017	13	736	1	8.8	0.14	32.64
1 primi	1	H						
73	1	11/04/2017	14	737	1	9.3	0.03	34.78

1 primi 1 H
73 1 11/05/2017 15 738 1 9.2 0.04 33.45
1 primi 1 H
73 1 11/06/2017 16 739 1 9.2 0.03 34.55
1 primi 1 H
73 1 11/07/2017 17 740 1 9.2 0.62 33.45
1 primi 1 H
73 1 11/08/2017 18 741 1 9.1 0 34.42
1 primi 1 H
73 1 11/09/2017 19 742 1 9.6 0 36.48
1 primi 1 H
73 1 11/10/2017 20 743 1 9.2 0.14 32.43
1 primi 1 H
73 1 11/11/2017 21 744 1 8.9 0 34.15
1 primi 1 H
73 1 11/12/2017 22 745 1 9.5 0.04 36.3
1 primi 2 H
73 1 11/13/2017 23 746 1 9.3 0.11 35.04
1 primi 2 H
73 1 11/14/2017 24 747 1 8.8 0 35.88
1 primi 2 H
73 1 11/15/2017 25 748 1 9.5 0.05 36.19
1 primi 2 H
73 1 11/16/2017 26 749 1 8.9 0.15 35.09
1 primi 2 H
73 1 11/17/2017 27 750 1 8.9 0 34.66
1 primi 2 H
73 1 11/18/2017 28 751 1 8.9 0 38.14
1 primi 2 H
73 1 11/19/2017 29 752 1 9.2 0.1 36.57
1 primi 2 H
73 1 11/20/2017 30 753 1 9.2 0.05 33.47
1 primi 2 H
73 1 11/21/2017 31 754 1 9.5 0.4 34.79
1 primi 2 H
73 1 11/22/2017 32 755 1 9.1 0 34.83
1 primi 2 H
73 1 11/23/2017 33 756 1 9.3 0 34.85
1 primi 2 H
73 1 11/24/2017 34 757 1 8.6 0.01 36.73
1 primi 2 H
73 1 11/25/2017 35 758 1 8.6 0 34.17
1 primi 2 H
73 1 11/26/2017 36 759 1 9.1 0.43 34.21
1 primi 2 H
73 1 11/27/2017 37 760 1 8.9 0.07 33.85
1 primi 2 H
73 1 11/28/2017 38 761 1 8.8 0 32.68
1 primi 2 H
73 1 11/29/2017 39 762 1 8.8 0.07 33.18
1 primi 2 H
73 1 11/30/2017 40 763 1 8.5 0.07 31.68
1 primi 2 H
73 1 12/01/2017 41 764 1 8.9 0 34.9

1 primi 2		H						
73	1	12/02/2017	42	765	1	9.3	0.08	33.63
1 primi 2		H						
73	1	12/03/2017	43	766	1	8.8	0.02	33.83
1 primi 2		H						
73	1	12/04/2017	44	767	1	9.2	0	33.11
1 primi 2		H						
73	1	12/05/2017	45	768	1	9.3	0	30.22
1 primi 2		H						
73	1	12/06/2017	46	769	1	9.5	0	29.62
1 primi 2		H						
73	1	12/07/2017	47	770	1	9.5	0	32.62
1 primi 2		H						
73	1	12/08/2017	48	771	1	9.3	0	31.23
1 primi 2		H						
73	1	12/09/2017	49	772	1	9.2	0	31.49
1 primi 2		H						
73	1	12/10/2017	50	773	1	9.5	0	35.24
1 primi 2		H						
73	1	12/11/2017	51	774	1	9.3	0	32
1 primi 2		H						
73	1	12/12/2017	52	775	1	8.8	0	33.25
1 primi 2		H						
73	1	12/13/2017	53	776	1	9.1	0	35.23
1 primi 2		H						
73	1	12/14/2017	54	777	1	9.5	0	34.5
1 primi 2		H						
73	1	12/15/2017	55	778	1	9.8	0	29.29
1 primi 2		H						
73	1	12/16/2017	56	779	1	9.6	0.02	36.71
1 primi 2		H						
73	1	12/17/2017	57	780	1	9.3	0	30.98
1 primi 2		H						
73	1	12/18/2017	58	781	1	9.8	0.17	32.11
1 primi 2		H						
73	1	12/19/2017	59	782	1	9.1	0.25	28.89
1 primi 2		H						
73	1	12/20/2017	60	783	1	9.6	0.1	34.58
1 primi 2		H						
73	1	12/21/2017	61	784	1	10	0.01	31.75
1 primi 2		H						
73	1	12/22/2017	62	785	1	10.2	0	33.95
1 primi 2		H						
73	1	12/23/2017	63	786	1	9.8	0.02	35.41
1 primi 2		H						
73	1	12/24/2017	64	787	1	9.5	0	33.64
1 primi 2		H						
73	1	12/25/2017	65	788	1	9.8	0.26	36.36
1 primi 2		H						
73	1	12/26/2017	66	789	1	9.5	0.01	35.38
1 primi 2		H						
73	1	12/27/2017	67	790	1	9.3	0	33.99
1 primi 2		H						
73	1	12/28/2017	68	791	1	9.6	0.03	36.15

1 primi 2 H
73 1 12/29/2017 69 792 1 9.9 0.01 33.44
1 primi 2 H
73 1 12/30/2017 70 793 1 9.9 0 34.69
1 primi 2 H
73 1 12/31/2017 71 794 1 9.8 0.01 36.25
1 primi 2 H
73 1 01/01/2018 72 795 1 9.8 0.09 34.66
1 primi 2 H
73 1 01/02/2018 73 796 1 10.2 0.02 35.56
1 primi 2 H
73 1 01/03/2018 74 797 1 9.6 0.01 40.99
1 primi 2 H
73 1 01/04/2018 75 798 1 9.5 0 36.35
1 primi 2 H
73 1 01/05/2018 76 799 1 9.3 0 35.46
1 primi 2 H
73 1 01/06/2018 77 800 1 9.5 0.07 34.7
1 primi 2 H
73 1 01/07/2018 78 801 1 9.6 0.18 30.03
1 primi 2 H
73 1 01/08/2018 79 802 1 9.6 0 28.67
1 primi 2 H
73 1 01/09/2018 80 803 1 9.6 0.01 36.11
1 primi 2 H
73 1 01/10/2018 81 804 1 10.3 0 35.54
1 primi 2 H
73 1 01/11/2018 82 805 1 10 0 37.42
1 primi 2 H
73 1 01/12/2018 83 806 1 10.4 0.14 25.33
1 primi 2 H
73 1 01/13/2018 84 807 1 10.4 0 38.48
1 primi 2 H
73 1 01/14/2018 85 808 1 10.4 0.06 38.18
1 primi 2 H
73 1 01/15/2018 86 809 1 9.5 0 35.45
1 primi 2 H
73 1 01/16/2018 87 810 1 10.3 0 43.21
1 primi 2 H
73 1 01/17/2018 88 811 1 10 0 38.67
1 primi 2 H
73 1 01/18/2018 89 812 1 10 0.02 37.36
1 primi 2 H
73 1 01/19/2018 90 813 1 9.9 0 35.87
1 primi 2 H
73 1 01/20/2018 91 814 1 9.1 0 34.64
1 primi 2 H
73 1 01/21/2018 92 815 1 9.5 0.03 37.15
1 primi 2 H
73 1 01/22/2018 93 816 1 10.2 0.02 36.14
1 primi 2 H
73 1 01/23/2018 94 817 1 9.8 0 35.66
1 primi 2 H
73 1 01/24/2018 95 818 1 10.3 0.01 38.04

1 primi 2 H
73 1 01/25/2018 96 819 1 10.2 0 37.91
1 primi 2 H
73 1 01/26/2018 97 820 1 10.3 0 34.5
1 primi 2 H
73 1 01/27/2018 98 821 1 10.3 0 36.15
1 primi 2 H
73 1 01/28/2018 99 822 1 10 0 39.63
1 primi 2 H
73 1 01/29/2018 100 823 1 9.8 0.06 37.02
1 primi 2 H
73 1 01/30/2018 101 824 1 10.2 0 35.84
1 primi 2 H
73 1 01/31/2018 102 825 1 10.2 0 39.35
1 primi 2 H
73 1 02/01/2018 103 826 1 10 0.01 37.38
1 primi 2 H
73 1 02/02/2018 104 827 1 9.6 0.01 38.65
1 primi 2 H
73 1 02/03/2018 105 828 1 10 0.02 37.41
1 primi 2 H
73 1 02/04/2018 106 829 1 9.9 0 37.8
1 primi 2 H
73 1 02/05/2018 107 830 1 9.8 0 37.4
1 primi 2 H
73 1 02/06/2018 108 831 1 10.3 0.03 38.33
1 primi 2 H
73 1 02/07/2018 109 832 1 10 0.04 37.92
1 primi 2 H
73 1 02/08/2018 110 833 1 9.5 0 38.81
1 primi 2 H
73 1 02/09/2018 111 834 1 9.6 0.18 38.7
1 primi 2 H
73 1 02/10/2018 112 835 1 10.2 0 36.94
1 primi 2 H
73 1 02/11/2018 113 836 1 10.3 0 38.22
1 primi 2 H
73 1 02/12/2018 114 837 1 10.4 0.03 37.96
1 primi 2 H
73 1 02/13/2018 115 838 1 10.4 0.1 37.88
1 primi 2 H
73 1 02/14/2018 116 839 1 9.8 0 43.62
1 primi 2 H
73 1 02/15/2018 117 840 1 10.3 0 42.24
1 primi 2 H
73 1 02/16/2018 118 841 1 10.4 0.01 39.93
1 primi 2 H
73 1 02/17/2018 119 842 1 9.5 0.05 33.97
1 primi 2 H
73 1 02/18/2018 120 843 1 9.8 0.03 40.03
1 primi 2 H
73 1 02/19/2018 121 844 1 10.4 0 38.23
1 primi 2 H
73 1 02/20/2018 122 845 1 10.6 0.05 35.25

1 primi	2	H						
73	1	02/21/2018	123	846	1	10.3	0	37
1 primi	2	H						
73	1	02/22/2018	124	847	1	10.3	0	40.22
1 primi	2	H						
73	1	02/23/2018	125	848	1	10.2	0.03	36.82
1 primi	2	H						
73	1	02/24/2018	126	849	1	10.3	0	36.32
1 primi	2	H						
73	1	02/25/2018	127	850	1	10.4	0	37.97
1 primi	2	H						
73	1	02/26/2018	128	851	1	10.3	0	39.41
1 primi	2	H						
73	1	02/27/2018	129	852	1	10	0	36.03
1 primi	2	H						
73	1	02/28/2018	130	853	1	9.6	0	36.99
1 primi	2	H						
73	1	03/01/2018	131	854	1	9.8	0	35.07
1 primi	2	H						
73	1	03/02/2018	132	855	1	9.8	0	33.66
1 primi	2	H						
73	1	03/03/2018	133	856	1	9.6	0	35.12
1 primi	2	H						
73	1	03/04/2018	134	857	1	9.6	0	33.17
1 primi	2	H						
73	1	03/05/2018	135	858	1	9.9	0	34.32
1 primi	2	H						
73	1	03/06/2018	136	859	1	9.5	0	34.03
1 primi	2	H						
73	1	03/07/2018	137	860	1	9.2	0	33.86
1 primi	2	H						
73	1	03/08/2018	138	861	1	9.9	0	31.52
1 primi	2	H						
73	1	03/09/2018	139	862	1	9.8	0	33.77
1 primi	2	H						
73	1	03/10/2018	140	863	1	10.2	0.22	27.99
1 primi	2	H						
73	1	03/11/2018	141	864	1	10.7	0	30.12
1 primi	2	H						
73	1	11/30/2018	3	1128	2	10	0.67	30.1
2 multi	1	H						
73	1	12/01/2018	4	1129	2	10.2	0.24	32.87
2 multi	1	H						
73	1	12/02/2018	5	1130	2	10	0.17	30.02
2 multi	1	H						
73	1	12/03/2018	6	1131	2	9.8	0.67	32.45
2 multi	1	H						
73	1	12/04/2018	7	1132	2	10	0.44	32.85
2 multi	1	H						
73	1	12/05/2018	8	1133	2	9.8	0.46	35.89
2 multi	1	H						
73	1	12/06/2018	9	1134	2	10.2	0	37.17
2 multi	1	H						
73	1	12/07/2018	10	1135	2	9.8	0	38.14

2 multi	1	H						
73	1	12/08/2018	11	1136	2	10	0	39.35
2 multi	1	H						
73	1	12/09/2018	12	1137	2	10	0.08	38.8
2 multi	1	H						
73	1	12/10/2018	13	1138	2	10.6	0	40.19
2 multi	1	H						
73	1	12/11/2018	14	1139	2	10.6	0	40.04
2 multi	1	H						
73	1	12/12/2018	15	1140	2	10	0.22	40.09
2 multi	1	H						
73	1	12/13/2018	16	1141	2	10.3	0	38.92
2 multi	1	H						
73	1	12/14/2018	17	1142	2	10.3	0	40.72
2 multi	1	H						
73	1	12/15/2018	18	1143	2	10.2	0	42.47
2 multi	1	H						
73	1	12/16/2018	19	1144	2	10.2	0	43.93
2 multi	1	H						
73	1	12/17/2018	20	1145	2	10.3	0	45.45
2 multi	1	H						
73	1	12/18/2018	21	1146	2	10.4	0	43.92
2 multi	1	H						
73	1	12/19/2018	22	1147	2	10.4	0.06	44.21
2 multi	2	H						
73	1	12/20/2018	23	1148	2	10.3	0	45.05
2 multi	2	H						
73	1	12/21/2018	24	1149	2	10.3	0.66	44.1
2 multi	2	H						
73	1	12/22/2018	25	1150	2	10	0	47.02
2 multi	2	H						
73	1	12/23/2018	26	1151	2	9.9	0	46.17
2 multi	2	H						
73	1	12/24/2018	27	1152	2	10.2	0	42.92
2 multi	2	H						
73	1	12/25/2018	28	1153	2	10.3	0	45.75
2 multi	2	H						
73	1	12/26/2018	29	1154	2	10.6	0	42.14
2 multi	2	H						
73	1	12/27/2018	30	1155	2	10.4	0	42.57
2 multi	2	H						
73	1	12/28/2018	31	1156	2	10	0	40.75
2 multi	2	H						
73	1	12/29/2018	32	1157	2	10.2	0	41.89
2 multi	2	H						
73	1	12/30/2018	33	1158	2	10.3	0.16	41.56
2 multi	2	H						
73	1	12/31/2018	34	1159	2	10.2	0	41.88
2 multi	2	H						
73	1	01/01/2019	35	1160	2	10.2	0	39.71
2 multi	2	H						
73	1	01/02/2019	36	1161	2	10.2	0.23	42.37
2 multi	2	H						
73	1	01/03/2019	37	1162	2	10.2	0	42.8

2 multi 2		H						
73	1	01/04/2019	38	1163	2	0	0	40.39
2 multi 2		H						
73	1	01/05/2019	39	1164	2	10	0	38.25
2 multi 2		H						
73	1	01/06/2019	40	1165	2	10	0	38.71
2 multi 2		H						
73	1	01/07/2019	41	1166	2	10.3	0	40.01
2 multi 2		H						
73	1	01/08/2019	42	1167	2	10.3	0	36.04
2 multi 2		H						
73	1	01/09/2019	43	1168	2	10.2	0	37.59
2 multi 2		H						
73	1	01/10/2019	44	1169	2	10.3	0	42.99
2 multi 2		H						
73	1	01/11/2019	45	1170	2	10.4	0	41.35
2 multi 2		H						
73	1	01/12/2019	46	1171	2	10.4	0	34.63
2 multi 2		H						
73	1	01/13/2019	47	1172	2	10.4	0	36.02
2 multi 2		H						
73	1	01/14/2019	48	1173	2	10.3	0	40.36
2 multi 2		H						
73	1	01/15/2019	49	1174	2	10.2	0	35.65
2 multi 2		H						
73	1	01/16/2019	50	1175	2	10.3	0.07	41.61
2 multi 2		H						
73	1	01/17/2019	51	1176	2	10.2	0.14	40.2
2 multi 2		H						
73	1	01/18/2019	52	1177	2	10	0	36.45
2 multi 2		H						
73	1	01/19/2019	53	1178	2	10.4	0.06	39.63
2 multi 2		H						
73	1	01/20/2019	54	1179	2	10.3	0	38.32
2 multi 2		H						
73	1	01/21/2019	55	1180	2	10.3	0	40.91
2 multi 2		H						
73	1	01/22/2019	56	1181	2	10	0	39.07
2 multi 2		H						
73	1	01/23/2019	57	1182	2	10.4	0	41.81
2 multi 2		H						
73	1	01/24/2019	58	1183	2	10.4	0	40.33
2 multi 2		H						
73	1	01/25/2019	59	1184	2	10.2	0	39.59
2 multi 2		H						
73	1	01/26/2019	60	1185	2	10	0	35.31
2 multi 2		H						
73	1	01/27/2019	61	1186	2	10.3	0	41.16
2 multi 2		H						
73	1	01/28/2019	62	1187	2	10.4	0	41.15
2 multi 2		H						
73	1	01/29/2019	63	1188	2	10.2	0	39.58
2 multi 2		H						
73	1	01/30/2019	64	1189	2	10.3	0	38.58

2 multi 2		H						
73	1	01/31/2019	65	1190	2	10.3	0	39.76
2 multi 2		H						
73	1	02/01/2019	66	1191	2	10.4	0	40.21
2 multi 2		H						
73	1	02/02/2019	67	1192	2	10.2	0	41.82
2 multi 2		H						
73	1	02/03/2019	68	1193	2	10.3	0	42.44
2 multi 2		H						
73	1	02/04/2019	69	1194	2	10.3	0.06	39.45
2 multi 2		H						
73	1	02/05/2019	70	1195	2	10	0	38.54
2 multi 2		H						
73	1	02/06/2019	71	1196	2	10.4	0.07	38.46
2 multi 2		H						
73	1	02/07/2019	72	1197	2	10.2	0	38.48
2 multi 2		H						
73	1	02/08/2019	73	1198	2	10.3	0	39.89
2 multi 2		H						
73	1	02/09/2019	74	1199	2	10.4	0	40.96
2 multi 2		H						
73	1	02/10/2019	75	1200	2	10.6	0.07	39.44
2 multi 2		H						
73	1	02/11/2019	76	1201	2	10.6	0	40.81
2 multi 2		H						
73	1	02/12/2019	77	1202	2	10.4	0	39.75
2 multi 2		H						
73	1	02/13/2019	78	1203	2	10.4	0	40.98
2 multi 2		H						
73	1	02/14/2019	79	1204	2	10.2	0	41.6
2 multi 2		H						
73	1	02/15/2019	80	1205	2	10	0	39.52
2 multi 2		H						
73	1	02/16/2019	81	1206	2	10.3	0	40.01
2 multi 2		H						
73	1	02/17/2019	82	1207	2	10.4	0	41.45
2 multi 2		H						
73	1	02/18/2019	83	1208	2	10.6	0	41.36
2 multi 2		H						
73	1	02/19/2019	84	1209	2	10.3	0	37.32
2 multi 2		H						
73	1	02/20/2019	85	1210	2	10.3	0	38.55
2 multi 2		H						
73	1	02/21/2019	86	1211	2	10.4	0	36.01
2 multi 2		H						
73	1	02/22/2019	87	1212	2	10.4	0	41.1
2 multi 2		H						
73	1	02/23/2019	88	1213	2	10.6	0	33.36
2 multi 2		H						
73	1	02/24/2019	89	1214	2	10.9	0	37.51
2 multi 2		H						
73	1	02/25/2019	90	1215	2	10.6	0	36.7
2 multi 2		H						
73	1	12/30/2019	3	1523	3	9.5	0.34	28.66

2 multi	1	H						
73	1	12/31/2019	4	1524	3	10	0.4	32.21
2 multi	1	H						
73	1	01/01/2020	5	1525	3	9.9	0.08	34.15
2 multi	1	H						
73	1	01/02/2020	6	1526	3	10	0.59	31.65
2 multi	1	H						
73	1	01/03/2020	7	1527	3	10	0.21	33.83
2 multi	1	H						
73	1	01/04/2020	8	1528	3	10.3	0	36.07
2 multi	1	H						
73	1	01/05/2020	9	1529	3	10.3	0.14	37.33
2 multi	1	H						
73	1	01/06/2020	10	1530	3	10.2	0.07	36.68
2 multi	1	H						
73	1	01/07/2020	11	1531	3	10.2	0.42	38.31
2 multi	1	H						
73	1	01/08/2020	12	1532	3	10	0	40.23
2 multi	1	H						
73	1	01/09/2020	13	1533	3	10.2	0.14	42.58
2 multi	1	H						
73	1	01/10/2020	14	1534	3	10.4	0.08	42.64
2 multi	1	H						
73	1	01/11/2020	15	1535	3	10.4	0	41.11
2 multi	1	H						
73	1	01/12/2020	16	1536	3	10.4	0.01	44.26
2 multi	1	H						
73	1	01/13/2020	17	1537	3	10.6	0	45.84
2 multi	1	H						
73	1	01/14/2020	18	1538	3	10.4	0	47.59
2 multi	1	H						
73	1	01/15/2020	19	1539	3	10.6	0	48.97
2 multi	1	H						
73	1	01/16/2020	20	1540	3	10.9	0	46.17
2 multi	1	H						
73	1	01/17/2020	21	1541	3	10.6	0	50.16
2 multi	1	H						
73	1	01/18/2020	22	1542	3	10.6	0.09	47.61
2 multi	2	H						
73	1	01/19/2020	23	1543	3	10.6	0	48.61
2 multi	2	H						
73	1	01/20/2020	24	1544	3	10.3	0	50.27
2 multi	2	H						
73	1	01/21/2020	25	1545	3	10.3	0.31	48.69
2 multi	2	H						
73	1	01/22/2020	26	1546	3	10.3	0.02	50.93
2 multi	2	H						
73	1	01/23/2020	27	1547	3	10.6	0	50.65
2 multi	2	H						
73	1	01/24/2020	28	1548	3	10.3	0.54	50.34
2 multi	2	H						
73	1	01/25/2020	29	1549	3	10.4	0.13	50.18
2 multi	2	H						
73	1	01/26/2020	30	1550	3	10.9	0	50.83

2 multi	2	H						
73	1	01/27/2020	31	1551	3	10.9	0	52.43
2 multi	2	H						
73	1	01/28/2020	32	1552	3	10.3	0.14	44.46
2 multi	2	H						
73	1	01/29/2020	33	1553	3	10.2	0.4	49.86
2 multi	2	H						
73	1	01/30/2020	34	1554	3	10.4	0.02	49.08
2 multi	2	H						
73	1	01/31/2020	35	1555	3	10.4	0.02	50.44
2 multi	2	H						
73	1	02/01/2020	36	1556	3	10.4	0	44.82
2 multi	2	H						
73	1	02/02/2020	37	1557	3	10.3	0	45.46
2 multi	2	H						
73	1	02/03/2020	38	1558	3	10.2	0	48.86
2 multi	2	H						
73	1	02/04/2020	39	1559	3	10.2	0	38.98
2 multi	2	H						
73	1	02/05/2020	40	1560	3	10.2	0	50.03
2 multi	2	H						
73	1	02/06/2020	41	1561	3	10.6	0.02	41.83
2 multi	2	H						
73	1	02/07/2020	42	1562	3	10	0	48.75
2 multi	2	H						
73	1	02/08/2020	43	1563	3	10.4	0.52	45.59
2 multi	2	H						
73	1	02/09/2020	44	1564	3	10	0	49.65
2 multi	2	H						
73	1	02/10/2020	45	1565	3	10.2	0	49.8
2 multi	2	H						
73	1	02/11/2020	46	1566	3	9.9	0	48.3
2 multi	2	H						
73	1	02/12/2020	47	1567	3	10.3	0	50.25
2 multi	2	H						
73	1	02/13/2020	48	1568	3	10.3	0.01	50.32
2 multi	2	H						
73	1	02/14/2020	49	1569	3	10.2	0.22	51.36
2 multi	2	H						
73	1	02/15/2020	50	1570	3	10.2	0.03	51.67
2 multi	2	H						
73	1	02/16/2020	51	1571	3	10	0.14	49.21
2 multi	2	H						
73	1	02/17/2020	52	1572	3	10	0.14	51.37
2 multi	2	H						
73	1	02/18/2020	53	1573	3	10.3	0	47.45
2 multi	2	H						
73	1	02/19/2020	54	1574	3	10.4	0.03	52.53
2 multi	2	H						
73	1	02/20/2020	55	1575	3	10.3	0.02	50.97
2 multi	2	H						
73	1	02/21/2020	56	1576	3	9.9	0	52.72
2 multi	2	H						
73	1	02/22/2020	57	1577	3	10.6	0	52.2

2 multi	2	H						
73	1	02/23/2020	58	1578	3	10.4	0.07	50.22
2 multi	2	H						
73	1	02/24/2020	59	1579	3	11.9	0	44.68
2 multi	2	H						
73	1	02/25/2020	60	1580	3	10.7	0.03	50.89
2 multi	2	H						
73	1	02/26/2020	61	1581	3	10.6	0	50.21
2 multi	2	H						
73	1	02/27/2020	62	1582	3	10.4	0	49.8
2 multi	2	H						
73	1	02/28/2020	63	1583	3	10.3	0.03	48.63
2 multi	2	H						
73	1	02/29/2020	64	1584	3	10.3	0.01	52.05
2 multi	2	H						
73	1	03/01/2020	65	1585	3	10.3	0	44.91
2 multi	2	H						
73	1	03/02/2020	66	1586	3	10.9	0.03	52.58
2 multi	2	H						
73	1	03/03/2020	67	1587	3	10.6	0	50.94
2 multi	2	H						
73	1	03/04/2020	68	1588	3	10.6	0	50.8
2 multi	2	H						
73	1	03/05/2020	69	1589	3	10.9	0.01	52.96
2 multi	2	H						
73	1	03/06/2020	70	1590	3	10.7	0.02	52.95
2 multi	2	H						
73	1	03/07/2020	71	1591	3	11.1	0.01	49.88
2 multi	2	H						
73	1	03/08/2020	72	1592	3	10.9	0.05	49.25
2 multi	2	H						
73	1	03/09/2020	73	1593	3	11.4	0.02	48.9
2 multi	2	H						
73	1	03/10/2020	74	1594	3	11.3	0	50.02
2 multi	2	H						
73	1	03/11/2020	75	1595	3	11.1	0	49.7
2 multi	2	H						
73	1	03/12/2020	76	1596	3	11.1	0.02	53.38
2 multi	2	H						
73	1	03/13/2020	77	1597	3	10.9	0	52.34
2 multi	2	H						
73	1	03/14/2020	78	1598	3	10.9	0	53.48
2 multi	2	H						
73	1	03/15/2020	79	1599	3	10.4	0	52.29
2 multi	2	H						
73	1	03/16/2020	80	1600	3	10.7	0.01	52.92
2 multi	2	H						
73	1	03/17/2020	81	1601	3	10.7	0	53.18
2 multi	2	H						
73	1	03/18/2020	82	1602	3	11.3	0	54.03
2 multi	2	H						
73	1	03/19/2020	83	1603	3	10.9	0	54.85
2 multi	2	H						
73	1	03/20/2020	84	1604	3	11.3	0	54.6

2 multi	2	H						
73	1	03/21/2020	85	1605	3	11.7	0.08	50.47
2 multi	2	H						
73	1	03/22/2020	86	1606	3	11.7	0.09	53.6
2 multi	2	H						
73	1	03/23/2020	87	1607	3	11.3	0.08	51.35
2 multi	2	H						
73	1	03/24/2020	88	1608	3	11.1	0	50.73
2 multi	2	H						
73	1	03/25/2020	89	1609	3	11.1	0	53.22
2 multi	2	H						
73	1	03/26/2020	90	1610	3	10.9	0.03	53.22
2 multi	2	H						
73	1	03/27/2020	91	1611	3	11.3	0	52.53
2 multi	2	H						
73	1	03/28/2020	92	1612	3	11.1	0	50.57
2 multi	2	H						
73	1	03/29/2020	93	1613	3	11.1	0	51.64
2 multi	2	H						
73	1	03/30/2020	94	1614	3	11.1	0	51.18
2 multi	2	H						
73	1	03/31/2020	95	1615	3	10.3	0.03	52.83
2 multi	2	H						
73	1	04/01/2020	96	1616	3	10.9	0.02	51.3
2 multi	2	H						
73	1	04/02/2020	97	1617	3	11.3	0.01	48.64
2 multi	2	H						
73	1	04/03/2020	98	1618	3	11.1	0.04	51.57
2 multi	2	H						
73	1	04/04/2020	99	1619	3	11.4	0.02	47.12
2 multi	2	H						
73	1	04/05/2020	100	1620	3	11.7	0.01	49.02
2 multi	2	H						
73	1	04/06/2020	101	1621	3	11.4	0	47.04
2 multi	2	H						
73	1	04/07/2020	102	1622	3	11.1	0	46.54
2 multi	2	H						
73	1	04/08/2020	103	1623	3	11.3	0	51.22
2 multi	2	H						
73	1	04/09/2020	104	1624	3	11.3	0.02	48.65
2 multi	2	H						
73	1	04/10/2020	105	1625	3	10.9	0	48.22
2 multi	2	H						
73	1	04/11/2020	106	1626	3	10.9	0.01	52.37
2 multi	2	H						
73	1	04/12/2020	107	1627	3	11.3	0	49.47
2 multi	2	H						
73	1	04/13/2020	108	1628	3	10.9	0.18	55.31
2 multi	2	H						
73	1	04/14/2020	109	1629	3	11.3	0	49.09
2 multi	2	H						
73	1	04/15/2020	110	1630	3	11.4	0	50.65
2 multi	2	H						
73	1	04/16/2020	111	1631	3	11.7	0	49.55

2 multi	2	H						
73	1	04/17/2020	112	1632	3	11.4	0	48.76
2 multi	2	H						
73	1	04/18/2020	113	1633	3	11.7	0	48.94
2 multi	2	H						
73	1	04/19/2020	114	1634	3	11.1	0.18	47.02
2 multi	2	H						
73	1	04/20/2020	115	1635	3	11.4	0.03	47.24
2 multi	2	H						
73	1	04/21/2020	116	1636	3	11.4	0.02	44.36
2 multi	2	H						
73	1	04/22/2020	117	1637	3	11.7	0	47.44
2 multi	2	H						
73	1	04/23/2020	118	1638	3	11.3	0.23	43.62
2 multi	2	H						
73	1	04/24/2020	119	1639	3	10.9	0	45.06
2 multi	2	H						
73	1	04/25/2020	120	1640	3	10.6	0.01	45.51
2 multi	2	H						
73	1	04/26/2020	121	1641	3	10.7	0	45.86
2 multi	2	H						
73	1	04/27/2020	122	1642	3	11.4	0	47.05
2 multi	2	H						
73	1	04/28/2020	123	1643	3	11.4	0.08	46.62
2 multi	2	H						
73	1	04/29/2020	124	1644	3	11.4	0.01	47.49
2 multi	2	H						
73	1	04/30/2020	125	1645	3	11.9	0	48.83
2 multi	2	H						
73	1	05/01/2020	126	1646	3	11.4	0.07	44.33
2 multi	2	H						
73	1	05/02/2020	127	1647	3	11.7	0.08	45.39
2 multi	2	H						
73	1	05/03/2020	128	1648	3	11.7	0	47.47
2 multi	2	H						
73	1	05/04/2020	129	1649	3	11.7	0.03	48.08
2 multi	2	H						
73	1	05/05/2020	130	1650	3	10.9	0	45.53
2 multi	2	H						
73	1	05/06/2020	131	1651	3	11.3	0	42.03
2 multi	2	H						
73	1	05/07/2020	132	1652	3	11.1	0.27	47.36
2 multi	2	H						
73	1	05/08/2020	133	1653	3	10.4	0.03	44.15
2 multi	2	H						
73	1	05/09/2020	134	1654	3	10.3	0.41	44.85
2 multi	2	H						
73	1	05/10/2020	135	1655	3	11.3	0.02	48.2
2 multi	2	H						
73	1	05/11/2020	136	1656	3	11.3	0.04	49.54
2 multi	2	H						
73	1	05/12/2020	137	1657	3	10.7	0.02	46.93
2 multi	2	H						
73	1	05/13/2020	138	1658	3	10.4	0.23	43.35

2 multi 2		H						
73	1	05/14/2020	139	1659	3	10.7	0.02	49.19
2 multi 2		H						
73	1	05/15/2020	140	1660	3	10.9	0.04	41.5
2 multi 2		H						
73	1	05/16/2020	141	1661	3	10.9	0	46.62
2 multi 2		H						
73	1	05/17/2020	142	1662	3	11.4	0	46.71
2 multi 2		H						
73	1	05/18/2020	143	1663	3	11.1	0.02	47.35
2 multi 2		H						
73	1	05/19/2020	144	1664	3	10.6	0.02	44.66
2 multi 2		H						
73	1	05/20/2020	145	1665	3	11.3	0	47.12
2 multi 2		H						
73	1	05/21/2020	146	1666	3	11.4	0.21	43.55
2 multi 2		H						
73	1	05/22/2020	147	1667	3	11.4	0.01	46.08
2 multi 2		H						
73	1	05/23/2020	148	1668	3	11.1	0	40.19
2 multi 2		H						
73	1	05/24/2020	149	1669	3	11.4	0.02	45.48
2 multi 2		H						
73	1	05/25/2020	150	1670	3	11.4	0.03	48.68
2 multi 2		H						
73	1	05/26/2020	151	1671	3	10.7	0.02	47.45
2 multi 2		H						
73	1	05/27/2020	152	1672	3	10.9	0	39.53
2 multi 2		H						
73	1	05/28/2020	153	1673	3	11.1	0.01	47.14
2 multi 2		H						
73	1	05/29/2020	154	1674	3	11.1	0.05	44.78
2 multi 2		H						
73	1	05/30/2020	155	1675	3	11.3	0	45.51
2 multi 2		H						
73	1	05/31/2020	156	1676	3	11.4	0.04	44.17
2 multi 2		H						
73	1	06/01/2020	157	1677	3	11.7	0	42.84
2 multi 2		H						
73	1	06/02/2020	158	1678	3	11.3	0	40.12
2 multi 2		H						
73	1	06/03/2020	159	1679	3	11.3	0	37.64
2 multi 2		H						
73	1	06/04/2020	160	1680	3	11.3	0	38.77
2 multi 2		H						
73	1	06/05/2020	161	1681	3	11.3	0	35.47
2 multi 2		H						
73	1	06/06/2020	162	1682	3	11.7	0.01	36.73
2 multi 2		H						
73	1	06/07/2020	163	1683	3	11.1	0	37.8
2 multi 2		H						
73	1	06/08/2020	164	1684	3	10.9	0.02	45.73
2 multi 2		H						
73	1	06/09/2020	165	1685	3	11.3	0	41.6

2 multi	2	H						
73	1	06/10/2020	166	1686	3	10.9	0	44.11
2 multi	2	H						
73	1	06/11/2020	167	1687	3	11.1	0	42.97
2 multi	2	H						
73	1	06/12/2020	168	1688	3	11.3	0	44.62
2 multi	2	H						
73	1	06/13/2020	169	1689	3	11.3	0	42.81
2 multi	2	H						
73	1	06/14/2020	170	1690	3	11.7	0	41.49
2 multi	2	H						
73	1	06/15/2020	171	1691	3	11.1	0	40.94
2 multi	2	H						
73	1	06/16/2020	172	1692	3	11.4	0	37.36
2 multi	2	H						
73	1	06/17/2020	173	1693	3	11.3	0	37.8
2 multi	2	H						
73	1	06/18/2020	174	1694	3	11.3	0	41.33
2 multi	2	H						
73	1	06/19/2020	175	1695	3	11.3	0.03	42.4
2 multi	2	H						
73	1	06/20/2020	176	1696	3	11.1	0	43.07
2 multi	2	H						
73	1	06/21/2020	177	1697	3	11.1	0	38.03
2 multi	2	H						
73	1	06/22/2020	178	1698	3	11.3	0.05	42.29
2 multi	2	H						
73	1	06/23/2020	179	1699	3	10.9	0	41.4
2 multi	2	H						
73	1	06/24/2020	180	1700	3	10.9	0	41.71
2 multi	2	H						
73	1	06/25/2020	181	1701	3	11.1	0	42.9
2 multi	2	H						
73	1	06/26/2020	182	1702	3	11.3	0	47.2
2 multi	2	H						
73	1	06/27/2020	183	1703	3	11.3	0	43.6
2 multi	2	H						
73	1	06/28/2020	184	1704	3	11.4	0	42.9
2 multi	2	H						
73	1	06/29/2020	185	1705	3	11.1	0	41.29
2 multi	2	H						
73	1	06/30/2020	186	1706	3	11.4	0	36.22
2 multi	2	H						
73	1	07/01/2020	187	1707	3	11.4	0	41.68
2 multi	2	H						
73	1	07/02/2020	188	1708	3	12.3	0	40.96
2 multi	2	H						
73	1	07/03/2020	189	1709	3	11.9	0.09	44.08
2 multi	2	H						
73	1	07/04/2020	190	1710	3	12.3	0.48	43.94
2 multi	2	H						
73	1	07/05/2020	191	1711	3	12.3	0.01	38.76
2 multi	2	H						
73	1	07/06/2020	192	1712	3	11.7	0.05	42.02

2 multi	2	H						
73	1	07/07/2020	193	1713	3	12.3	0.15	39.26
2 multi	2	H						
73	1	07/08/2020	194	1714	3	11.9	0.03	38.25
2 multi	2	H						
73	1	07/09/2020	195	1715	3	12.3	0.03	39.38
2 multi	2	H						
73	1	07/10/2020	196	1716	3	11.4	0.09	34.79
2 multi	2	H						
73	1	07/11/2020	197	1717	3	12.6	0.02	40.2
2 multi	2	H						
73	1	07/12/2020	198	1718	3	12	0	33.38
2 multi	2	H						
73	1	07/13/2020	199	1719	3	11.7	0.05	40.36
2 multi	2	H						
73	1	07/14/2020	200	1720	3	12.5	0.06	42.4
2 multi	2	H						
73	1	07/15/2020	201	1721	3	12.5	0.03	38.8
2 multi	2	H						
73	1	07/16/2020	202	1722	3	12.6	0.04	37.66
2 multi	2	H						
73	1	07/17/2020	203	1723	3	10.2	0.04	37.72
2 multi	2	H						
73	1	07/18/2020	204	1724	3	10.9	0.03	37.02
2 multi	2	H						
73	1	07/19/2020	205	1725	3	10.2	0	35.42
2 multi	2	H						
73	1	07/20/2020	206	1726	3	12.3	0.07	39.89
2 multi	2	H						
73	1	07/21/2020	207	1727	3	11.9	0.1	37.46
2 multi	2	H						
73	1	07/22/2020	208	1728	3	11.7	0.02	39.84
2 multi	2	H						
73	1	07/23/2020	209	1729	3	11.7	0.21	36.34
2 multi	2	H						
73	1	07/24/2020	210	1730	3	11.7	0.09	33.71
2 multi	2	H						
73	1	07/25/2020	211	1731	3	11.3	0.01	38.19
2 multi	2	H						
73	1	07/26/2020	212	1732	3	11.3	0	34.75
2 multi	2	H						
73	1	07/27/2020	213	1733	3	11.1	0.03	38.02
2 multi	2	H						
73	1	07/28/2020	214	1734	3	10.6	0	33.56
2 multi	2	H						
73	1	07/29/2020	215	1735	3	10.9	0.12	37.41
2 multi	2	H						
73	1	07/30/2020	216	1736	3	11.1	0.09	33.13
2 multi	2	H						
73	1	07/31/2020	217	1737	3	10.7	0.05	28.53
2 multi	2	H						
73	1	08/01/2020	218	1738	3	11.3	0.02	32.74
2 multi	2	H						
73	1	08/02/2020	219	1739	3	10.9	0	30.93

2 multi	2	H						
73	1	08/03/2020	220	1740	3	11.1	0.03	32.58
2 multi	2	H						
73	1	08/04/2020	221	1741	3	11.1	0	36.09
2 multi	2	H						
73	1	08/05/2020	222	1742	3	10.3	0.13	32.27
2 multi	2	H						
73	1	08/06/2020	223	1743	3	10.9	0	34.13
2 multi	2	H						
73	1	08/07/2020	224	1744	3	10.9	0	30.21
2 multi	2	H						
73	1	08/08/2020	225	1745	3	11.1	0.09	34.33
2 multi	2	H						
73	1	08/09/2020	226	1746	3	11.3	0	30.98
2 multi	2	H						
73	1	11/16/2020	4	1845	4	9.8	0.64	27.87
2 multi	1	H						
73	1	11/17/2020	5	1846	4	10.2	0	30.17
2 multi	1	H						
73	1	11/18/2020	6	1847	4	10.6	0.5	31.58
2 multi	1	H						
73	1	11/19/2020	7	1848	4	10.2	0.39	36.19
2 multi	1	H						
73	1	11/20/2020	8	1849	4	10.2	0.76	39.83
2 multi	1	H						
73	1	11/21/2020	9	1850	4	10	0.5	41.12
2 multi	1	H						
73	1	11/22/2020	10	1851	4	10.2	0.13	45.11
2 multi	1	H						
73	1	11/23/2020	11	1852	4	10	0.07	42.04
2 multi	1	H						
73	1	11/24/2020	12	1853	4	10.2	0.07	41.08
2 multi	1	H						
73	1	11/25/2020	13	1854	4	9.8	0.68	39.99
2 multi	1	H						
73	1	11/26/2020	14	1855	4	9.8	0.17	43.98
2 multi	1	H						
73	1	11/27/2020	15	1856	4	9.9	0.06	46.35
2 multi	1	H						
73	1	11/28/2020	16	1857	4	10	0	46.36
2 multi	1	H						
73	1	11/29/2020	17	1858	4	9.6	0.2	45.21
2 multi	1	H						
73	1	11/30/2020	18	1859	4	9.9	0.26	46.23
2 multi	1	H						
73	1	12/01/2020	19	1860	4	10	0.66	44.67
2 multi	1	H						
73	1	12/02/2020	20	1861	4	10.2	0.98	46.44
2 multi	1	H						
73	1	12/03/2020	21	1862	4	10	0.55	43.57
2 multi	1	H						
73	1	12/04/2020	22	1863	4	10.2	0.2	49.37
2 multi	2	H						
73	1	12/05/2020	23	1864	4	10	0.08	48.59

2 multi 2		H						
73	1	12/06/2020	24	1865	4	10.2	0	52.5
2 multi 2		H						
73	1	12/07/2020	25	1866	4	10	0	51.93
2 multi 2		H						
73	1	12/08/2020	26	1867	4	10.3	0.08	50.93
2 multi 2		H						
73	1	12/09/2020	27	1868	4	10	0.1	52
2 multi 2		H						
73	1	12/10/2020	28	1869	4	10	0.11	52.29
2 multi 2		H						
73	1	12/11/2020	29	1870	4	10	0.04	52.35
2 multi 2		H						
73	1	12/12/2020	30	1871	4	10	0	51.47
2 multi 2		H						
73	1	12/13/2020	31	1872	4	10.3	0.41	49.77
2 multi 2		H						
73	1	12/14/2020	32	1873	4	10.2	0.08	51.49
2 multi 2		H						
73	1	12/15/2020	33	1874	4	10.3	0	52.34
2 multi 2		H						
73	1	12/16/2020	34	1875	4	10	0	46.34
2 multi 2		H						
73	1	12/17/2020	35	1876	4	9.9	0.02	48.8
2 multi 2		H						
73	1	12/18/2020	36	1877	4	10	0.07	48.84
2 multi 2		H						
73	1	12/19/2020	37	1878	4	10.3	0.05	49.61
2 multi 2		H						
73	1	12/20/2020	38	1879	4	10.2	0.05	49.04
2 multi 2		H						
73	1	12/21/2020	39	1880	4	10.6	0.1	47.95
2 multi 2		H						
73	1	12/22/2020	40	1881	4	10.6	0.1	48.36
2 multi 2		H						
73	1	12/23/2020	41	1882	4	10.9	0.09	48.49
2 multi 2		H						
73	1	12/24/2020	42	1883	4	10.2	0.02	41.03
2 multi 2		H						
73	1	12/25/2020	43	1884	4	10	0.02	43.22
2 multi 2		H						
73	1	12/26/2020	44	1885	4	10.4	0	47.84
2 multi 2		H						
73	1	12/27/2020	45	1886	4	10.4	0.07	50.38
2 multi 2		H						
73	1	12/28/2020	46	1887	4	10.4	0.51	50.6
2 multi 2		H						
73	1	12/29/2020	47	1888	4	10.4	0.17	47.9
2 multi 2		H						
73	1	12/30/2020	48	1889	4	10.6	0.03	50.75
2 multi 2		H						
73	1	12/31/2020	49	1890	4	10.4	0.02	51.42
2 multi 2		H						
73	1	01/01/2021	50	1891	4	10.4	0	47.81

2 multi 2		H						
73	1	01/02/2021	51	1892	4	10.6	0	46.34
2 multi 2		H						
73	1	01/03/2021	52	1893	4	10.4	0	51.77
2 multi 2		H						
73	1	01/04/2021	53	1894	4	10.7	0.02	52.45
2 multi 2		H						
73	1	01/05/2021	54	1895	4	10.6	0.09	51.22
2 multi 2		H						
73	1	01/06/2021	55	1896	4	10.7	0.48	52.87
2 multi 2		H						
73	1	01/07/2021	56	1897	4	10.9	0.07	46.5
2 multi 2		H						
73	1	01/08/2021	57	1898	4	10.6	0.02	48.62
2 multi 2		H						
73	1	01/09/2021	58	1899	4	10.6	0.06	51.13
2 multi 2		H						
73	1	01/10/2021	59	1900	4	10.4	0.72	50.73
2 multi 2		H						
73	1	01/11/2021	60	1901	4	10.4	0.01	50.39
2 multi 2		H						
73	1	01/12/2021	61	1902	4	10.6	0	47.02
2 multi 2		H						
73	1	01/13/2021	62	1903	4	10.3	0.55	47.58
2 multi 2		H						
73	1	01/14/2021	63	1904	4	10.3	0.03	46.58
2 multi 2		H						
73	1	01/15/2021	64	1905	4	10.6	0.01	40.25
2 multi 2		H						
73	1	01/16/2021	65	1906	4	10.7	0.04	50.07
2 multi 2		H						
73	1	01/17/2021	66	1907	4	10.9	0.73	45.82
2 multi 2		H						
73	1	01/18/2021	67	1908	4	11.1	0.01	45.33
2 multi 2		H						
73	1	01/19/2021	68	1909	4	10.6	0	37.82
2 multi 2		H						
73	1	01/20/2021	69	1910	4	10.9	0	42.3
2 multi 2		H						
73	1	01/21/2021	70	1911	4	10.7	0.03	43.18
2 multi 2		H						
73	1	01/22/2021	71	1912	4	10.7	0.04	44.9
2 multi 2		H						
73	1	01/23/2021	72	1913	4	10.9	0.17	44.99
2 multi 2		H						
73	1	01/24/2021	73	1914	4	10.6	0.01	43.45
2 multi 2		H						
73	1	01/25/2021	74	1915	4	10.3	0	39.03
2 multi 2		H						
73	1	01/26/2021	75	1916	4	10.9	0	45.68
2 multi 2		H						
73	1	01/27/2021	76	1917	4	10.7	0.02	43.98
2 multi 2		H						
73	1	01/28/2021	77	1918	4	11.1	0.05	48.42

2 multi	2	H						
73	1	01/29/2021	78	1919	4	10.7	0.03	47.74
2 multi	2	H						
73	1	01/30/2021	79	1920	4	11.1	0.04	49.59
2 multi	2	H						
73	1	01/31/2021	80	1921	4	10.6	0	47.95
2 multi	2	H						
73	1	02/01/2021	81	1922	4	10.6	0	49.59
2 multi	2	H						
73	1	02/02/2021	82	1923	4	11.3	0	51.48
2 multi	2	H						
73	1	02/03/2021	83	1924	4	10.7	0.25	46.51
2 multi	2	H						
73	1	02/04/2021	84	1925	4	11.7	0.03	52.34
2 multi	2	H						
73	1	02/05/2021	85	1926	4	10.4	0	48.01
2 multi	2	H						
73	1	02/06/2021	86	1927	4	10.9	0	47.89
2 multi	2	H						
73	1	02/07/2021	87	1928	4	10.6	0.05	48.45
2 multi	2	H						
73	1	02/08/2021	88	1929	4	10.4	0.03	46.21
2 multi	2	H						
73	1	02/09/2021	89	1930	4	10.4	0.01	42.99
2 multi	2	H						
73	1	02/10/2021	90	1931	4	10.7	0	48.18
2 multi	2	H						
73	1	02/11/2021	91	1932	4	11.1	0.05	46.22
2 multi	2	H						
73	1	02/12/2021	92	1933	4	11.3	0.03	44.85
2 multi	2	H						
73	1	02/13/2021	93	1934	4	10.4	0.03	44.91
2 multi	2	H						
73	1	02/14/2021	94	1935	4	10.4	0.61	42.82
2 multi	2	H						
73	1	02/15/2021	95	1936	4	11.4	0.03	43.36
2 multi	2	H						
73	1	02/16/2021	96	1937	4	11.4	0.08	44.97
2 multi	2	H						
73	1	02/17/2021	97	1938	4	10.9	0.08	42.83
2 multi	2	H						
73	1	02/18/2021	98	1939	4	11.3	0.03	49.83
2 multi	2	H						
73	1	02/19/2021	99	1940	4	11.1	0.02	44.37
2 multi	2	H						
73	1	02/20/2021	100	1941	4	10.9	0	45.78
2 multi	2	H						
73	1	02/21/2021	101	1942	4	10.7	0.14	46.35
2 multi	2	H						
73	1	02/22/2021	102	1943	4	10.7	0.02	46.33
2 multi	2	H						
73	1	02/23/2021	103	1944	4	11.1	0.35	46.22
2 multi	2	H						
73	1	02/24/2021	104	1945	4	10.9	0.03	47.31

2 multi	2	H						
73	1	02/25/2021	105	1946	4	10.6	0.52	47.41
2 multi	2	H						
73	1	02/26/2021	106	1947	4	10.2	0	41.35
2 multi	2	H						
73	1	02/27/2021	107	1948	4	11.3	0.04	43.84
2 multi	2	H						
73	1	02/28/2021	108	1949	4	10	0.03	43.93
2 multi	2	H						
73	1	03/01/2021	109	1950	4	10.4	0.15	45.55
2 multi	2	H						
73	1	03/02/2021	110	1951	4	10.6	0	47.93
2 multi	2	H						
73	1	03/03/2021	111	1952	4	11.1	0.05	49.76
2 multi	2	H						
73	1	03/04/2021	112	1953	4	11.3	0	47.42
2 multi	2	H						
73	1	03/05/2021	113	1954	4	11.7	0.05	47.69
2 multi	2	H						
73	1	03/06/2021	114	1955	4	11.3	0	48.4
2 multi	2	H						
73	1	03/07/2021	115	1956	4	11.4	0.05	46.13
2 multi	2	H						
73	1	03/08/2021	116	1957	4	11.3	0.11	47.2
2 multi	2	H						
73	1	03/09/2021	117	1958	4	11.1	0.07	45.53
2 multi	2	H						
73	1	03/10/2021	118	1959	4	10.9	0.03	46.6
2 multi	2	H						
73	1	03/11/2021	119	1960	4	10.9	0.1	44.73
2 multi	2	H						
73	1	03/12/2021	120	1961	4	10.7	0	47.37
2 multi	2	H						
73	1	03/13/2021	121	1962	4	11.9	0.09	43.91
2 multi	2	H						
73	1	03/14/2021	122	1963	4	10.6	0	42.32
2 multi	2	H						
73	1	03/15/2021	123	1964	4	11.1	0.05	47.94
2 multi	2	H						
73	1	03/16/2021	124	1965	4	10.9	0.04	46.67
2 multi	2	H						
73	1	03/17/2021	125	1966	4	10.9	0.05	44.5
2 multi	2	H						
73	1	03/18/2021	126	1967	4	10.9	0.04	43.99
2 multi	2	H						
73	1	03/19/2021	127	1968	4	11.3	0	45.69
2 multi	2	H						
73	1	03/20/2021	128	1969	4	11.4	0.02	44.69
2 multi	2	H						
73	1	03/21/2021	129	1970	4	11.4	0.07	43.57
2 multi	2	H						
73	1	03/22/2021	130	1971	4	11.1	0.02	44.67
2 multi	2	H						
73	1	03/23/2021	131	1972	4	11.4	0	45.58

2 multi 2		H						
73	1	03/24/2021	132	1973	4	11.3	0	45.59
2 multi 2		H						
73	1	03/25/2021	133	1974	4	11.7	0.11	45.2
2 multi 2		H						
73	1	03/26/2021	134	1975	4	11.7	0.06	43.78
2 multi 2		H						
73	1	03/27/2021	135	1976	4	11.1	0	41.92
2 multi 2		H						
73	1	03/28/2021	136	1977	4	11.1	0	42.12
2 multi 2		H						
73	1	03/29/2021	137	1978	4	10.7	0.12	42.14
2 multi 2		H						
73	1	03/30/2021	138	1979	4	10.9	0.03	41.7
2 multi 2		H						
73	1	03/31/2021	139	1980	4	11.3	0	42.44
2 multi 2		H						
73	1	04/01/2021	140	1981	4	12	0.11	45.01
2 multi 2		H						
73	1	04/02/2021	141	1982	4	12.3	0.05	41.82
2 multi 2		H						
73	1	04/03/2021	142	1983	4	12.3	0.06	39.54
2 multi 2		H						
73	1	04/04/2021	143	1984	4	11.9	0	41.96
2 multi 2		H						
73	1	04/05/2021	144	1985	4	11.9	0	41
2 multi 2		H						
73	1	04/06/2021	145	1986	4	12	0.01	43.3
2 multi 2		H						
73	1	04/07/2021	146	1987	4	11.7	0	41.31
2 multi 2		H						
73	1	04/08/2021	147	1988	4	11.9	0.04	44.13
2 multi 2		H						
73	1	04/09/2021	148	1989	4	11.1	0	41.52
2 multi 2		H						
73	1	04/10/2021	149	1990	4	11.7	0.05	40.82
2 multi 2		H						
73	1	04/11/2021	150	1991	4	11.4	0.05	38.32
2 multi 2		H						
73	1	04/12/2021	151	1992	4	11.3	0.06	43.76
2 multi 2		H						
73	1	04/13/2021	152	1993	4	11.9	0.05	41.33
2 multi 2		H						
73	1	04/14/2021	153	1994	4	11.4	0.08	42.44
2 multi 2		H						
73	1	04/15/2021	154	1995	4	11.4	0	42.31
2 multi 2		H						
73	1	04/16/2021	155	1996	4	11.7	0.1	41.47
2 multi 2		H						
73	1	04/17/2021	156	1997	4	12	0	43.24
2 multi 2		H						
73	1	04/18/2021	157	1998	4	12	0.02	43.63
2 multi 2		H						
73	1	04/19/2021	158	1999	4	11.9	0.05	41.27

2 multi	2	H						
73	1	04/20/2021	159	2000	4	12.5	0.12	43.37
2 multi	2	H						
73	1	04/21/2021	160	2001	4	12	0	45.76
2 multi	2	H						
73	1	04/22/2021	161	2002	4	12.5	0.02	42.51
2 multi	2	H						
73	1	04/23/2021	162	2003	4	12.3	0.19	45.05
2 multi	2	H						
73	1	04/24/2021	163	2004	4	12.3	0.09	43.32
2 multi	2	H						
73	1	04/25/2021	164	2005	4	12.6	0.09	43.88
2 multi	2	H						
73	1	04/26/2021	165	2006	4	12.3	0	45.43
2 multi	2	H						
73	1	04/27/2021	166	2007	4	12.5	0	43.81
2 multi	2	H						
73	1	04/28/2021	167	2008	4	12.9	0	45.3
2 multi	2	H						
73	1	04/29/2021	168	2009	4	12.9	0.01	46.24
2 multi	2	H						
74	1	09/24/2018	8	723	1	9.3	0.22	19.64
1 primi	1	H						
74	1	09/25/2018	9	724	1	9.8	0.1	20.85
1 primi	1	H						
74	1	09/26/2018	10	725	1	9.6	0.01	19.35
1 primi	1	H						
74	1	09/27/2018	11	726	1	9.8	0	21.59
1 primi	1	H						
74	1	09/28/2018	12	727	1	9.2	0	22.88
1 primi	1	H						
74	1	09/29/2018	13	728	1	9.3	0.1	20.45
1 primi	1	H						
74	1	09/30/2018	14	729	1	8.8	0	21.17
1 primi	1	H						
74	1	10/01/2018	15	730	1	9.1	0	23.72
1 primi	1	H						
74	1	10/02/2018	16	731	1	8.9	0.01	23.36
1 primi	1	H						
74	1	10/03/2018	17	732	1	9.1	0	24.56
1 primi	1	H						
74	1	10/04/2018	18	733	1	8.8	0.21	25.17
1 primi	1	H						
74	1	10/05/2018	19	734	1	9.2	0.01	24.9
1 primi	1	H						
74	1	10/06/2018	20	735	1	9.3	0.01	26.29
1 primi	1	H						
74	1	10/07/2018	21	736	1	9.1	0	27.75
1 primi	1	H						
74	1	10/08/2018	22	737	1	9.2	0	27.3
1 primi	2	H						
74	1	10/09/2018	23	738	1	8.6	0	24.86
1 primi	2	H						
74	1	10/10/2018	24	739	1	8.6	0.35	24.17

1 primi	2	H						
74	1	10/11/2018	25	740	1	8.9	0	24.71
1 primi	2	H						
74	1	10/12/2018	26	741	1	8.8	0	28.64
1 primi	2	H						
74	1	10/13/2018	27	742	1	8.6	0	26.09
1 primi	2	H						
74	1	10/14/2018	28	743	1	8.6	0	25.5
1 primi	2	H						
74	1	10/15/2018	29	744	1	8.6	0	27.44
1 primi	2	H						
74	1	10/16/2018	30	745	1	8.7	0	26.52
1 primi	2	H						
74	1	10/17/2018	31	746	1	8.6	0	29.54
1 primi	2	H						
74	1	10/18/2018	32	747	1	8.5	0	26.37
1 primi	2	H						
74	1	10/19/2018	33	748	1	8.6	0	27.72
1 primi	2	H						
74	1	10/20/2018	34	749	1	8.6	0	28.18
1 primi	2	H						
74	1	10/21/2018	35	750	1	8.7	0	29.54
1 primi	2	H						
74	1	10/22/2018	36	751	1	8.4	0	27.59
1 primi	2	H						
74	1	10/23/2018	37	752	1	8.4	0	26.69
1 primi	2	H						
74	1	10/24/2018	38	753	1	8.6	0	26.75
1 primi	2	H						
74	1	10/25/2018	39	754	1	8.7	0	26.65
1 primi	2	H						
74	1	10/26/2018	40	755	1	9.1	0	23.45
1 primi	2	H						
74	1	10/27/2018	41	756	1	8.4	0	24.33
1 primi	2	H						
74	1	10/28/2018	42	757	1	8.3	0.48	26.09
1 primi	2	H						
74	1	10/29/2018	43	758	1	8.7	0	26.14
1 primi	2	H						
74	1	10/30/2018	44	759	1	8.9	0	27.6
1 primi	2	H						
74	1	10/31/2018	45	760	1	9.2	0	25.03
1 primi	2	H						
74	1	11/01/2018	46	761	1	9.1	0	27.8
1 primi	2	H						
74	1	11/02/2018	47	762	1	9.1	0	27.51
1 primi	2	H						
74	1	11/03/2018	48	763	1	9.1	0	29.45
1 primi	2	H						
74	1	11/04/2018	49	764	1	9.1	0	28.29
1 primi	2	H						
74	1	11/05/2018	50	765	1	9.2	0	27.94
1 primi	2	H						
74	1	08/23/2019	3	1056	2	10.3	0.42	31.12

2 multi	1	H						
74	1	08/24/2019	4	1057	2	10.7	0.08	37.45
2 multi	1	H						
74	1	08/25/2019	5	1058	2	10.7	0.08	34.94
2 multi	1	H						
74	1	08/26/2019	6	1059	2	10.3	0.04	37.89
2 multi	1	H						
74	1	08/27/2019	7	1060	2	10.6	0	37.77
2 multi	1	H						
74	1	08/28/2019	8	1061	2	10.4	0	37.78
2 multi	1	H						
74	1	08/29/2019	9	1062	2	10.2	0.32	38.04
2 multi	1	H						
74	1	08/30/2019	10	1063	2	9.9	0.01	39.35
2 multi	1	H						
74	1	08/31/2019	11	1064	2	10	0.03	40.38
2 multi	1	H						
74	1	09/01/2019	12	1065	2	9.6	0.03	40.24
2 multi	1	H						
74	1	09/02/2019	13	1066	2	10.2	0.05	41.66
2 multi	1	H						
74	1	09/03/2019	14	1067	2	9.8	0.03	38.78
2 multi	1	H						
74	1	09/04/2019	15	1068	2	9.8	0	40.64
2 multi	1	H						
74	1	09/05/2019	16	1069	2	10	0	38.92
2 multi	1	H						
74	1	09/06/2019	17	1070	2	9.8	0	40.54
2 multi	1	H						
74	1	09/07/2019	18	1071	2	9.9	0	39.82
2 multi	1	H						
74	1	09/08/2019	19	1072	2	9.5	0.02	39.01
2 multi	1	H						
74	1	09/09/2019	20	1073	2	9.9	0.54	39.29
2 multi	1	H						
74	1	09/10/2019	21	1074	2	9.8	0.25	40.99
2 multi	1	H						
74	1	09/11/2019	22	1075	2	9.6	0.03	43.21
2 multi	2	H						
74	1	09/12/2019	23	1076	2	9.5	0.14	41.48
2 multi	2	H						
74	1	06/30/2020	315	1368	2	9.6	0	32.42
2 multi	2	H						
74	1	07/01/2020	316	1369	2	9.6	0	33.62
2 multi	2	H						
74	1	07/02/2020	317	1370	2	9.6	0	32.18
2 multi	2	H						
74	1	07/03/2020	318	1371	2	9.6	0.12	31.24
2 multi	2	H						
74	1	07/04/2020	319	1372	2	8.9	0.6	33.99
2 multi	2	H						
74	1	07/05/2020	320	1373	2	9.5	0.19	33.13
2 multi	2	H						
74	1	07/06/2020	321	1374	2	10	0.36	32.94

2 multi	2	H						
74	1	07/07/2020	322	1375	2	9.9	0.05	32.81
2 multi	2	H						
74	1	07/08/2020	323	1376	2	8.9	0.1	32.86
2 multi	2	H						
74	1	07/09/2020	324	1377	2	10	0.06	36.78
2 multi	2	H						
74	1	07/10/2020	325	1378	2	9.5	0	37.1
2 multi	2	H						
74	1	07/11/2020	326	1379	2	9.9	0.14	35.13
2 multi	2	H						
74	1	07/12/2020	327	1380	2	9.8	0	31.67
2 multi	2	H						
74	1	07/13/2020	328	1381	2	9.2	0.32	34.11
2 multi	2	H						
74	1	07/14/2020	329	1382	2	9.1	0.64	33.64
2 multi	2	H						
74	1	07/15/2020	330	1383	2	10.2	0	33.7
2 multi	2	H						
74	1	07/16/2020	331	1384	2	9.2	0	32.59
2 multi	2	H						
74	1	07/17/2020	332	1385	2	9.9	0.55	35.15
2 multi	2	H						
74	1	07/18/2020	333	1386	2	8.8	0.18	33.59
2 multi	2	H						
74	1	07/19/2020	334	1387	2	9.5	0	31.95
2 multi	2	H						
74	1	07/20/2020	335	1388	2	9.8	0.3	31.68
2 multi	2	H						
74	1	07/21/2020	336	1389	2	9.9	0.7	31.52
2 multi	2	H						
74	1	07/22/2020	337	1390	2	9.8	0	29.08
2 multi	2	H						
74	1	07/23/2020	338	1391	2	9.2	0.35	26.89
2 multi	2	H						
74	1	07/24/2020	339	1392	2	9.5	0	22.72
2 multi	2	H						
74	1	07/25/2020	340	1393	2	9.9	0	24.44
2 multi	2	H						
74	1	07/26/2020	341	1394	2	9.8	0	25.93
2 multi	2	H						
74	1	07/27/2020	342	1395	2	9.9	0.01	29.42
2 multi	2	H						
74	1	07/28/2020	343	1396	2	10	0.24	27.58
2 multi	2	H						
74	1	07/29/2020	344	1397	2	10.2	0.11	30.63
2 multi	2	H						
74	1	07/30/2020	345	1398	2	9.9	0.52	30.13
2 multi	2	H						
74	1	07/31/2020	346	1399	2	9.6	0.17	31.75
2 multi	2	H						
74	1	08/01/2020	347	1400	2	9.3	0	29.28
2 multi	2	H						
76	1	10/05/2020	9	811	1	9.5	0.06	28.21

1 primi	1	H						
76	1	10/06/2020	10	812	1	10	0	25.66
1 primi	1	H						
76	1	10/07/2020	11	813	1	9.6	0	24.92
1 primi	1	H						
76	1	10/08/2020	12	814	1	9.5	0.1	26.14
1 primi	1	H						
76	1	10/09/2020	13	815	1	9.6	0.03	28.81
1 primi	1	H						
76	1	10/10/2020	14	816	1	9.6	0	27.83
1 primi	1	H						
76	1	10/11/2020	15	817	1	9.5	0.24	28.28
1 primi	1	H						
76	1	10/12/2020	16	818	1	9.2	0.05	29.41
1 primi	1	H						
76	1	10/13/2020	17	819	1	9.5	0	30.84
1 primi	1	H						
76	1	10/14/2020	18	820	1	9.3	0.01	28.62
1 primi	1	H						
76	1	10/15/2020	19	821	1	10.3	0.03	30.9
1 primi	1	H						
76	1	10/16/2020	20	822	1	10.3	0.04	29.65
1 primi	1	H						
76	1	10/17/2020	21	823	1	9.8	0.07	33.53
1 primi	1	H						
76	1	10/18/2020	22	824	1	9.3	0.04	35.84
1 primi	2	H						
76	1	10/19/2020	23	825	1	9.1	0.04	37.32
1 primi	2	H						
76	1	10/20/2020	24	826	1	9.5	0	36.73
1 primi	2	H						
76	1	10/21/2020	25	827	1	9.2	0.04	37.81
1 primi	2	H						
76	1	10/22/2020	26	828	1	9.1	0	35.51
1 primi	2	H						
76	1	10/23/2020	27	829	1	9.2	0	38.85
1 primi	2	H						
76	1	10/24/2020	28	830	1	8.8	0	36.24
1 primi	2	H						
76	1	10/25/2020	29	831	1	8.7	0.04	38.38
1 primi	2	H						
76	1	10/26/2020	30	832	1	8.9	0.18	37.04
1 primi	2	H						
76	1	10/27/2020	31	833	1	8.9	0	36.05
1 primi	2	H						
76	1	10/28/2020	32	834	1	9.1	0.03	36.17
1 primi	2	H						
76	1	10/29/2020	33	835	1	8.8	0.04	37.31
1 primi	2	H						
76	1	10/30/2020	34	836	1	8.8	0.01	38.09
1 primi	2	H						
76	1	10/31/2020	35	837	1	8.8	0.09	39.71
1 primi	2	H						
76	1	11/01/2020	36	838	1	8.7	0	37.66

1 primi	2	H						
76	1	11/02/2020	37	839	1	8.8	0	39.54
1 primi	2	H						
76	1	11/03/2020	38	840	1	8.8	0.06	38.26
1 primi	2	H						
76	1	11/04/2020	39	841	1	8.7	0.06	40.62
1 primi	2	H						
76	1	11/05/2020	40	842	1	8.9	0.01	39.13
1 primi	2	H						
76	1	11/06/2020	41	843	1	8.9	0	38.63
1 primi	2	H						
76	1	11/07/2020	42	844	1	8.5	0	36.49
1 primi	2	H						
76	1	11/08/2020	43	845	1	8.7	0	39.69
1 primi	2	H						
76	1	11/09/2020	44	846	1	8.9	0	37.63
1 primi	2	H						
76	1	11/10/2020	45	847	1	8.8	0.05	39.15
1 primi	2	H						
76	1	11/11/2020	46	848	1	8.7	0.06	39.91
1 primi	2	H						
76	1	11/12/2020	47	849	1	8.9	0.04	38.36
1 primi	2	H						
76	1	11/13/2020	48	850	1	8.8	0.06	37.71
1 primi	2	H						
76	1	11/14/2020	49	851	1	8.8	0.03	32.73
1 primi	2	H						
76	1	11/15/2020	50	852	1	8.8	0	38.52
1 primi	2	H						
76	1	11/16/2020	51	853	1	8.9	0.05	36.59
1 primi	2	H						
76	1	11/17/2020	52	854	1	9.3	0.06	41.04
1 primi	2	H						
76	1	11/18/2020	53	855	1	9.3	0.12	40.22
1 primi	2	H						
76	1	11/19/2020	54	856	1	9.3	0.05	39.45
1 primi	2	H						
76	1	11/20/2020	55	857	1	9.1	0.05	41.14
1 primi	2	H						
76	1	11/21/2020	56	858	1	8.6	0	38.33
1 primi	2	H						
76	1	11/22/2020	57	859	1	8.8	0	40.24
1 primi	2	H						
76	1	11/23/2020	58	860	1	8.8	0	39.99
1 primi	2	H						
76	1	11/24/2020	59	861	1	8.9	0.04	37.61
1 primi	2	H						
76	1	11/25/2020	60	862	1	8.4	0.02	39.65
1 primi	2	H						
76	1	11/26/2020	61	863	1	8.7	0.06	39.31
1 primi	2	H						
76	1	11/27/2020	62	864	1	9.1	0.06	38.68
1 primi	2	H						
76	1	11/28/2020	63	865	1	9.1	0	38.29

1 primi	2	H						
76	1	11/29/2020	64	866	1	8.8	0.06	38.79
1 primi	2	H						
76	1	11/30/2020	65	867	1	8.8	0.07	36.51
1 primi	2	H						
76	1	12/01/2020	66	868	1	8.8	0.07	37.84
1 primi	2	H						
76	1	12/02/2020	67	869	1	8.8	0.07	38.63
1 primi	2	H						
76	1	12/03/2020	68	870	1	8.9	0.03	37.68
1 primi	2	H						
76	1	12/04/2020	69	871	1	8.9	0.45	38.64
1 primi	2	H						
76	1	12/05/2020	70	872	1	9.1	0.07	40.41
1 primi	2	H						
76	1	12/06/2020	71	873	1	8.7	0.06	40.31
1 primi	2	H						
77	1	02/24/2020	6	793	1	9.2	0	26.09
1 primi	1	H						
77	1	02/25/2020	7	794	1	9.5	0.02	27.53
1 primi	1	H						
77	1	02/26/2020	8	795	1	8.9	0	26.84
1 primi	1	H						
77	1	02/27/2020	9	796	1	9.2	0	28.68
1 primi	1	H						
77	1	02/28/2020	10	797	1	8.7	0.28	27.29
1 primi	1	H						
77	1	02/29/2020	11	798	1	8.8	0	31.41
1 primi	1	H						
77	1	03/01/2020	12	799	1	8.8	0	30.68
1 primi	1	H						
77	1	03/02/2020	13	800	1	8.6	0.38	27.08
1 primi	1	H						
77	1	03/03/2020	14	801	1	8.8	0	32.09
1 primi	1	H						
77	1	03/04/2020	15	802	1	8.8	0.02	29.54
1 primi	1	H						
77	1	03/05/2020	16	803	1	8.6	0.02	30.28
1 primi	1	H						
77	1	03/06/2020	17	804	1	8.9	0	29.18
1 primi	1	H						
77	1	03/07/2020	18	805	1	8.5	0.04	27.84
1 primi	1	H						
77	1	03/08/2020	19	806	1	8.6	0.45	27.65
1 primi	1	H						
77	1	03/09/2020	20	807	1	8.6	0	28.12
1 primi	1	H						
77	1	03/10/2020	21	808	1	8.8	0.01	29.99
1 primi	1	H						
77	1	03/11/2020	22	809	1	8.7	0	27.77
1 primi	2	H						
77	1	03/12/2020	23	810	1	8.9	0.42	27.77
1 primi	2	H						
77	1	03/13/2020	24	811	1	8.7	0	28.38

1 primi 2		H						
77	1	03/14/2020	25	812	1	8.8	0	29.34
1 primi 2		H						
77	1	03/15/2020	26	813	1	8.9	0.28	30.34
1 primi 2		H						
77	1	03/16/2020	27	814	1	8.8	0.3	31.12
1 primi 2		H						
77	1	03/17/2020	28	815	1	8.7	0.37	29.02
1 primi 2		H						
77	1	03/18/2020	29	816	1	8.5	0.09	31.85
1 primi 2		H						
77	1	03/19/2020	30	817	1	8.8	0.27	33.44
1 primi 2		H						
77	1	03/20/2020	31	818	1	8.6	0.06	33.73
1 primi 2		H						
77	1	03/21/2020	32	819	1	8.7	0	33.9
1 primi 2		H						
77	1	03/22/2020	33	820	1	8.8	0.01	33.45
1 primi 2		H						
77	1	03/23/2020	34	821	1	8.7	0	34
1 primi 2		H						
77	1	03/24/2020	35	822	1	8.7	0	34.47
1 primi 2		H						
77	1	03/25/2020	36	823	1	8.5	0	36.47
1 primi 2		H						
77	1	03/26/2020	37	824	1	8.6	0.07	34.62
1 primi 2		H						
77	1	03/27/2020	38	825	1	8.7	0.46	31.39
1 primi 2		H						
77	1	03/28/2020	39	826	1	8.5	0	34.91
1 primi 2		H						
77	1	03/29/2020	40	827	1	8.4	0	35.43
1 primi 2		H						
77	1	03/30/2020	41	828	1	8.4	0.16	33.29
1 primi 2		H						
77	1	03/31/2020	42	829	1	8.3	0	36.67
1 primi 2		H						
77	1	04/01/2020	43	830	1	8.4	0.19	35.72
1 primi 2		H						
77	1	04/02/2020	44	831	1	8.3	0	33.79
1 primi 2		H						
77	1	04/03/2020	45	832	1	8	0.31	31.93
1 primi 2		H						
77	1	04/04/2020	46	833	1	8.8	0.42	33.33
1 primi 2		H						
77	1	04/05/2020	47	834	1	8.2	0	30.74
1 primi 2		H						
77	1	04/06/2020	48	835	1	8.3	0.15	33.5
1 primi 2		H						
77	1	04/07/2020	49	836	1	8.3	0	32.44
1 primi 2		H						
77	1	04/08/2020	50	837	1	8.4	0	32.71
1 primi 2		H						
77	1	04/09/2020	51	838	1	8.4	0.02	34.44

1 primi 2		H						
77	1	04/10/2020	52	839	1	8.6	0	33.84
1 primi 2		H						
77	1	04/11/2020	53	840	1	8.7	0.57	32.74
1 primi 2		H						
77	1	04/12/2020	54	841	1	8.6	0	34.76
1 primi 2		H						
77	1	04/13/2020	55	842	1	8.5	0.04	35.95
1 primi 2		H						
77	1	04/14/2020	56	843	1	8.9	0	34
1 primi 2		H						
77	1	04/15/2020	57	844	1	8.7	0	36.85
1 primi 2		H						
77	1	04/16/2020	58	845	1	8.8	0.31	34.7
1 primi 2		H						
77	1	04/17/2020	59	846	1	8.6	0	36.35
1 primi 2		H						
77	1	04/18/2020	60	847	1	8.5	0	34.01
1 primi 2		H						
77	1	04/19/2020	61	848	1	8.5	0.49	32.31
1 primi 2		H						
77	1	04/20/2020	62	849	1	8.9	0.06	29.75
1 primi 2		H						
77	1	04/21/2020	63	850	1	8.8	1	36
1 primi 2		H						
77	1	04/22/2020	64	851	1	8.7	0.32	38.3
1 primi 2		H						
77	1	04/23/2020	65	852	1	0	0	32.54
1 primi 2		H						
77	1	04/24/2020	66	853	1	8.2	0.41	37.49
1 primi 2		H						
77	1	04/25/2020	67	854	1	9.1	0	35.85
1 primi 2		H						
77	1	04/26/2020	68	855	1	8.8	0	37.25
1 primi 2		H						
77	1	04/27/2020	69	856	1	8.7	0.08	34.97
1 primi 2		H						
77	1	04/28/2020	70	857	1	8.9	0.22	35.69
1 primi 2		H						
77	1	04/29/2020	71	858	1	8.5	0	36.13
1 primi 2		H						
77	1	04/30/2020	72	859	1	8.9	0.07	35.17
1 primi 2		H						
77	1	05/01/2020	73	860	1	8.9	0	37.03
1 primi 2		H						
77	1	05/02/2020	74	861	1	8.5	0	33.99
1 primi 2		H						
77	1	05/03/2020	75	862	1	8.7	0.35	34.08
1 primi 2		H						
77	1	05/04/2020	76	863	1	8.7	0	35.99
1 primi 2		H						
77	1	05/05/2020	77	864	1	8.7	0	35.13
1 primi 2		H						
77	1	05/06/2020	78	865	1	8.9	0	33

1 primi 2 H
77 1 05/07/2020 79 866 1 9.3 0 36.25
1 primi 2 H
77 1 05/08/2020 80 867 1 9.2 0.28 37.87
1 primi 2 H
77 1 05/09/2020 81 868 1 8.9 0 36.51
1 primi 2 H
77 1 05/10/2020 82 869 1 8.9 0.61 38.49
1 primi 2 H
77 1 05/11/2020 83 870 1 9.1 0 35.6
1 primi 2 H
77 1 05/12/2020 84 871 1 8.8 0.28 32.34
1 primi 2 H
77 1 05/13/2020 85 872 1 8.7 0 36.04
1 primi 2 H
77 1 05/14/2020 86 873 1 8.6 0.07 37.91
1 primi 2 H
77 1 05/15/2020 87 874 1 8.8 0 37.61
1 primi 2 H
77 1 05/16/2020 88 875 1 8.7 0.26 35.65
1 primi 2 H
77 1 05/17/2020 89 876 1 8.4 0.2 33.98
1 primi 2 H
77 1 05/18/2020 90 877 1 8.4 0.02 33.79
1 primi 2 H
77 1 05/19/2020 91 878 1 8.4 0.32 34.51
1 primi 2 H
77 1 05/20/2020 92 879 1 8.4 0.32 33.61
1 primi 2 H
77 1 05/21/2020 93 880 1 8.6 0 35.15
1 primi 2 H
77 1 05/22/2020 94 881 1 9.2 0.16 33.11
1 primi 2 H
77 1 05/23/2020 95 882 1 8.8 0 35.64
1 primi 2 H
77 1 05/24/2020 96 883 1 8.8 0.02 33.69
1 primi 2 H
77 1 05/25/2020 97 884 1 9.1 0 36.15
1 primi 2 H
77 1 05/26/2020 98 885 1 8.7 0.21 34.29
1 primi 2 H
77 1 05/27/2020 99 886 1 9.1 0 31.28
1 primi 2 H
77 1 05/28/2020 100 887 1 9.3 0.1 39.32
1 primi 2 H
77 1 06/30/2020 133 920 1 9.1 0 34.58
1 primi 2 H
77 1 07/01/2020 134 921 1 9.3 0 35.1
1 primi 2 H
77 1 07/02/2020 135 922 1 8.9 0 34.98
1 primi 2 H
77 1 07/03/2020 136 923 1 9.1 0.06 35.36
1 primi 2 H
77 1 07/04/2020 137 924 1 9.3 0.04 34.58

1 primi 2		H						
77	1	07/05/2020	138	925	1	8.9	0	35.51
1 primi 2		H						
77	1	07/06/2020	139	926	1	9.2	0.23	37.29
1 primi 2		H						
77	1	07/07/2020	140	927	1	8.6	0.2	33.76
1 primi 2		H						
77	1	07/08/2020	141	928	1	9.5	0.08	36.53
1 primi 2		H						
77	1	07/09/2020	142	929	1	9.5	0.03	35.58
1 primi 2		H						
77	1	07/10/2020	143	930	1	9.2	0.22	38.93
1 primi 2		H						
77	1	07/11/2020	144	931	1	8.8	0.01	37.37
1 primi 2		H						
77	1	07/12/2020	145	932	1	8.9	0	37.23
1 primi 2		H						
77	1	07/13/2020	146	933	1	8.7	0.24	35.36
1 primi 2		H						
77	1	07/14/2020	147	934	1	8.8	0.45	37.13
1 primi 2		H						
77	1	07/15/2020	148	935	1	8.8	0.17	36.05
1 primi 2		H						
77	1	07/16/2020	149	936	1	8.9	0.02	37.34
1 primi 2		H						
77	1	07/17/2020	150	937	1	8.9	0.48	35.05
1 primi 2		H						
77	1	07/18/2020	151	938	1	8.8	0.25	37.76
1 primi 2		H						
77	1	07/19/2020	152	939	1	9.2	0.03	37.78
1 primi 2		H						
77	1	07/20/2020	153	940	1	8.9	0.13	35.29
1 primi 2		H						
77	1	07/21/2020	154	941	1	8.9	0.02	35.95
1 primi 2		H						
77	1	07/22/2020	155	942	1	9.2	0.39	36.94
1 primi 2		H						
77	1	07/23/2020	156	943	1	8.9	0	36.08
1 primi 2		H						
77	1	07/24/2020	157	944	1	9.2	0.06	35.17
1 primi 2		H						
77	1	07/25/2020	158	945	1	9.1	0.49	34.72
1 primi 2		H						
77	1	07/26/2020	159	946	1	9.1	0.19	35.27
1 primi 2		H						
77	1	07/27/2020	160	947	1	9.3	0.01	35.61
1 primi 2		H						
77	1	07/28/2020	161	948	1	9.1	0.01	34.66
1 primi 2		H						
77	1	07/29/2020	162	949	1	9.1	0.06	34.1
1 primi 2		H						
77	1	07/30/2020	163	950	1	9.5	0.11	34.36
1 primi 2		H						
77	1	07/31/2020	164	951	1	9.1	0.15	31.59

1 primi 2 H
77 1 08/01/2020 165 952 1 8.8 0.05 33.67
1 primi 2 H
77 1 08/02/2020 166 953 1 9.1 0 35.75
1 primi 2 H
77 1 08/03/2020 167 954 1 9.1 0.14 30.97
1 primi 2 H
77 1 08/04/2020 168 955 1 9.2 0.01 37.55
1 primi 2 H
77 1 08/05/2020 169 956 1 8.9 0 36.87
1 primi 2 H
77 1 08/06/2020 170 957 1 9.1 0 35.98
1 primi 2 H
77 1 08/07/2020 171 958 1 8.7 0 35.4
1 primi 2 H
77 1 08/08/2020 172 959 1 9.1 0.03 35.46
1 primi 2 H
77 1 08/09/2020 173 960 1 9.2 0.03 33.21
1 primi 2 H
77 1 08/10/2020 174 961 1 8.7 0.22 34.23
1 primi 2 H
77 1 08/11/2020 175 962 1 8.8 0 33.12
1 primi 2 H
77 1 08/12/2020 176 963 1 9.3 0 31.52
1 primi 2 H
77 1 08/13/2020 177 964 1 9.1 0.15 30.49
1 primi 2 H
77 1 08/14/2020 178 965 1 9.2 0.02 30.62
1 primi 2 H
77 1 08/15/2020 179 966 1 8.9 0.04 30.57
1 primi 2 H
77 1 08/16/2020 180 967 1 8.8 0.17 31.16
1 primi 2 H
77 1 03/01/2021 4 1164 2 8.8 0.28 27.26
2 multi 1 H
77 1 03/02/2021 5 1165 2 9.3 0.24 32.73
2 multi 1 H
77 1 03/03/2021 6 1166 2 8.8 0.32 28.98
2 multi 1 H
77 1 03/04/2021 7 1167 2 9.8 0.07 30.96
2 multi 1 H
77 1 03/05/2021 8 1168 2 9.6 0.08 29.84
2 multi 1 H
77 1 03/06/2021 9 1169 2 9.3 0 35.08
2 multi 1 H
77 1 03/07/2021 10 1170 2 9.8 0.08 36.36
2 multi 1 H
77 1 03/08/2021 11 1171 2 9.1 0.06 35.72
2 multi 1 H
77 1 03/09/2021 12 1172 2 8.9 0 35.57
2 multi 1 H
77 1 03/10/2021 13 1173 2 9.1 0.04 40.66
2 multi 1 H
77 1 03/11/2021 14 1174 2 9.2 0 36.89

2 multi	1	H						
77	1	03/12/2021	15	1175	2	9.3	0.07	43.55
2 multi	1	H						
77	1	03/13/2021	16	1176	2	8.9	0	42.24
2 multi	1	H						
77	1	03/14/2021	17	1177	2	8.7	0.04	39.13
2 multi	1	H						
77	1	03/15/2021	18	1178	2	8.9	0.21	45.58
2 multi	1	H						
77	1	03/16/2021	19	1179	2	8.9	0.17	45.32
2 multi	1	H						
77	1	03/17/2021	20	1180	2	9.1	0.28	45.03
2 multi	1	H						
77	1	03/18/2021	21	1181	2	9.1	0.01	48.03
2 multi	1	H						
77	1	03/19/2021	22	1182	2	10	0.22	32.4
2 multi	2	H						
77	1	03/20/2021	23	1183	2	10.3	0.06	39.97
2 multi	2	H						
77	1	03/21/2021	24	1184	2	8.9	0.32	34.29
2 multi	2	H						
77	1	03/22/2021	25	1185	2	8.9	0	39.29
2 multi	2	H						
77	1	03/23/2021	26	1186	2	9.5	0	44.32
2 multi	2	H						
77	1	03/24/2021	27	1187	2	9.2	0.01	43.89
2 multi	2	H						
77	1	03/25/2021	28	1188	2	9.3	0.19	45.21
2 multi	2	H						
77	1	03/26/2021	29	1189	2	9.1	0.21	46.39
2 multi	2	H						
77	1	03/27/2021	30	1190	2	9.1	0.16	41.45
2 multi	2	H						
77	1	03/28/2021	31	1191	2	8.9	0.25	44.13
2 multi	2	H						
77	1	03/29/2021	32	1192	2	9.2	0	45.6
2 multi	2	H						
77	1	03/30/2021	33	1193	2	9.8	0.33	44.75
2 multi	2	H						
77	1	03/31/2021	34	1194	2	9.3	0.02	46.56
2 multi	2	H						
77	1	04/01/2021	35	1195	2	9.3	0.17	45.09
2 multi	2	H						
77	1	04/02/2021	36	1196	2	9.2	0.02	42.65
2 multi	2	H						
77	1	04/03/2021	37	1197	2	9.2	0.03	44.95
2 multi	2	H						
77	1	04/04/2021	38	1198	2	9.2	0.04	46.42
2 multi	2	H						
77	1	04/05/2021	39	1199	2	9.5	0	43.66
2 multi	2	H						
77	1	04/06/2021	40	1200	2	9.2	0	43.9
2 multi	2	H						
77	1	04/07/2021	41	1201	2	9.3	0.02	45.13

2 multi	2	H						
77	1	04/08/2021	42	1202	2	9.3	0.03	44.95
2 multi	2	H						
77	1	04/09/2021	43	1203	2	9.1	0	41.08
2 multi	2	H						
77	1	04/10/2021	44	1204	2	9.1	0.16	48.81
2 multi	2	H						
77	1	04/11/2021	45	1205	2	9.2	0.2	46.88
2 multi	2	H						
77	1	04/12/2021	46	1206	2	9.1	0.24	43.63
2 multi	2	H						
77	1	04/13/2021	47	1207	2	9.3	0.31	46.08
2 multi	2	H						
77	1	04/14/2021	48	1208	2	9.3	0.3	42.06
2 multi	2	H						
77	1	04/15/2021	49	1209	2	9.5	0.05	36.71
2 multi	2	H						
77	1	04/16/2021	50	1210	2	9.5	0.03	40.82
2 multi	2	H						
77	1	04/17/2021	51	1211	2	9.9	0.05	40.97
2 multi	2	H						
77	1	04/18/2021	52	1212	2	10	0.03	45.25
2 multi	2	H						
77	1	04/19/2021	53	1213	2	9.8	0.03	44.88
2 multi	2	H						
77	1	04/20/2021	54	1214	2	9.9	0	39.9
2 multi	2	H						
77	1	04/21/2021	55	1215	2	9.9	0.06	51.3
2 multi	2	H						
77	1	04/22/2021	56	1216	2	10.2	0.25	52.27
2 multi	2	H						
77	1	04/23/2021	57	1217	2	10	0.28	49.51
2 multi	2	H						
77	1	04/24/2021	58	1218	2	9.9	0	50.97
2 multi	2	H						
77	1	04/25/2021	59	1219	2	9.6	0	52.34
2 multi	2	H						
77	1	04/26/2021	60	1220	2	10	0	51.37
2 multi	2	H						
77	1	04/27/2021	61	1221	2	9.6	0	50.74
2 multi	2	H						
77	1	04/28/2021	62	1222	2	9.8	0	49.72
2 multi	2	H						
77	1	04/29/2021	63	1223	2	9.9	0.03	52.74
2 multi	2	H						
77	1	04/30/2021	64	1224	2	9.9	0.08	49.21
2 multi	2	H						
77	1	05/01/2021	65	1225	2	9.3	0.04	42.21
2 multi	2	H						
77	1	05/02/2021	66	1226	2	9.6	0.05	49.13
2 multi	2	H						
77	1	05/03/2021	67	1227	2	10.4	0.36	44.25
2 multi	2	H						
77	1	05/04/2021	68	1228	2	10	0.06	44.74

2 multi	2	H						
77	1	05/05/2021	69	1229	2	9.6	0.23	44.24
2 multi	2	H						
77	1	05/06/2021	70	1230	2	10.2	0.2	45.85
2 multi	2	H						
77	1	05/07/2021	71	1231	2	9.9	0	46.49
2 multi	2	H						
77	1	05/08/2021	72	1232	2	10	0.06	48.66
2 multi	2	H						
77	1	05/09/2021	73	1233	2	9.6	0.16	49.82
2 multi	2	H						
77	1	05/10/2021	74	1234	2	9.6	0.16	48.8
2 multi	2	H						
77	1	05/11/2021	75	1235	2	10	0	44.37
2 multi	2	H						
77	1	05/12/2021	76	1236	2	9.9	0	44.81
2 multi	2	H						
77	1	05/13/2021	77	1237	2	9.6	0.03	45.9
2 multi	2	H						
77	1	05/14/2021	78	1238	2	9.6	0.03	42.52
2 multi	2	H						
77	1	05/15/2021	79	1239	2	9.6	0.22	44.38
2 multi	2	H						
77	1	05/16/2021	80	1240	2	9.8	0.26	46.22
2 multi	2	H						
77	1	05/17/2021	81	1241	2	9.9	0.26	46.72
2 multi	2	H						
77	1	05/18/2021	82	1242	2	10.3	0.28	47
2 multi	2	H						
77	1	05/19/2021	83	1243	2	9.9	0.31	46.61
2 multi	2	H						
77	1	05/20/2021	84	1244	2	10	0.28	36.43
2 multi	2	H						
77	1	05/21/2021	85	1245	2	10.3	0.34	43.86
2 multi	2	H						
77	1	05/22/2021	86	1246	2	10	0.21	46.77
2 multi	2	H						
77	1	05/23/2021	87	1247	2	10.6	0.23	46.9
2 multi	2	H						
77	1	05/24/2021	88	1248	2	10.2	0.28	46.51
2 multi	2	H						
77	1	05/25/2021	89	1249	2	9.8	0.01	45.5
2 multi	2	H						
77	1	05/26/2021	90	1250	2	9.6	0	22.89
2 multi	2	H						
80	1	03/26/2020	3	772	1	9.9	0.4	22.79
1 primi	1	H						
80	1	03/27/2020	4	773	1	11.4	0.02	30.63
1 primi	1	H						
80	1	03/28/2020	5	774	1	11.1	0.06	30.55
1 primi	1	H						
80	1	03/29/2020	6	775	1	10.9	0.05	28.76
1 primi	1	H						
80	1	03/30/2020	7	776	1	9.8	0.11	28.43

1 primi	1	H						
80	1	03/31/2020	8	777	1	9.6	0	28.6
1 primi	1	H						
80	1	04/01/2020	9	778	1	10.6	0.15	31.26
1 primi	1	H						
80	1	04/02/2020	10	779	1	10.9	0.05	31.2
1 primi	1	H						
80	1	04/03/2020	11	780	1	10.9	0	31.81
1 primi	1	H						
80	1	04/04/2020	12	781	1	11.1	0.12	31.17
1 primi	1	H						
80	1	04/05/2020	13	782	1	11.1	0.16	32.56
1 primi	1	H						
80	1	04/06/2020	14	783	1	10.2	0.35	31.46
1 primi	1	H						
80	1	04/07/2020	15	784	1	10.7	0.04	35.18
1 primi	1	H						
80	1	04/08/2020	16	785	1	10.2	0	36.15
1 primi	1	H						
80	1	04/09/2020	17	786	1	10.2	0.37	36.95
1 primi	1	H						
80	1	04/10/2020	18	787	1	10.2	0	34.59
1 primi	1	H						
80	1	04/11/2020	19	788	1	10.2	0.32	35.03
1 primi	1	H						
80	1	04/12/2020	20	789	1	10	0	38.96
1 primi	1	H						
80	1	04/13/2020	21	790	1	10.2	0.14	35.13
1 primi	1	H						
80	1	04/14/2020	22	791	1	10.3	0.1	36.89
1 primi	2	H						
80	1	04/15/2020	23	792	1	10.4	0	38.01
1 primi	2	H						
80	1	04/16/2020	24	793	1	9.5	0	39.43
1 primi	2	H						
80	1	04/17/2020	25	794	1	9.5	0.28	38.62
1 primi	2	H						
80	1	04/18/2020	26	795	1	9.9	0	39.79
1 primi	2	H						
80	1	04/19/2020	27	796	1	9.8	0.23	40.78
1 primi	2	H						
80	1	04/20/2020	28	797	1	9.3	0.16	42.18
1 primi	2	H						
80	1	04/21/2020	29	798	1	9.6	0.19	41.14
1 primi	2	H						
80	1	04/22/2020	30	799	1	9.6	0.08	40.02
1 primi	2	H						
80	1	04/23/2020	31	800	1	9.6	0.08	42.08
1 primi	2	H						
80	1	04/24/2020	32	801	1	9.6	0	43.01
1 primi	2	H						
80	1	04/25/2020	33	802	1	9.3	0.17	41.15
1 primi	2	H						
80	1	04/26/2020	34	803	1	9.5	0	42.76

1 primi 2 H
80 1 04/27/2020 35 804 1 9.5 0.25 41.28
1 primi 2 H
80 1 04/28/2020 36 805 1 10.2 0.03 41.39
1 primi 2 H
80 1 04/29/2020 37 806 1 9.8 0.25 38.55
1 primi 2 H
80 1 04/30/2020 38 807 1 9.6 0.02 40.61
1 primi 2 H
80 1 05/01/2020 39 808 1 9.9 0.01 38.45
1 primi 2 H
80 1 05/02/2020 40 809 1 9.9 0.01 36.66
1 primi 2 H
80 1 05/03/2020 41 810 1 10.3 0.18 38.41
1 primi 2 H
80 1 05/04/2020 42 811 1 10.2 0 37.35
1 primi 2 H
80 1 05/05/2020 43 812 1 9.9 0 32.74
1 primi 2 H
80 1 05/06/2020 44 813 1 9.8 0 31.79
1 primi 2 H
80 1 05/07/2020 45 814 1 11.7 0.09 34.49
1 primi 2 H
80 1 05/08/2020 46 815 1 10.7 0.07 41.59
1 primi 2 H
80 1 05/09/2020 47 816 1 9.3 0 39.44
1 primi 2 H
80 1 05/10/2020 48 817 1 9.3 0.05 39.01
1 primi 2 H
80 1 05/11/2020 49 818 1 10.2 0 39.92
1 primi 2 H
80 1 05/12/2020 50 819 1 10.2 0.09 37.88
1 primi 2 H
80 1 05/13/2020 51 820 1 9.8 0.18 38.45
1 primi 2 H
81 1 06/10/2019 3 721 1 10.2 0.38 22.64
1 primi 1 H
81 1 06/11/2019 4 722 1 10.7 0.05 23.62
1 primi 1 H
81 1 06/12/2019 5 723 1 9.9 0.12 23.07
1 primi 1 H
81 1 06/13/2019 6 724 1 9.6 0.03 24.66
1 primi 1 H
81 1 06/14/2019 7 725 1 9.9 0.02 25.3
1 primi 1 H
81 1 06/15/2019 8 726 1 9.9 0.12 24.13
1 primi 1 H
81 1 06/16/2019 9 727 1 9.8 0.01 26.2
1 primi 1 H
81 1 06/17/2019 10 728 1 9.2 0 29.06
1 primi 1 H
81 1 06/18/2019 11 729 1 9.5 0.02 28.4
1 primi 1 H
81 1 06/19/2019 12 730 1 9.3 0.15 27.5

1 primi	1	H						
81	1	06/20/2019	13	731	1	9.3	0.09	29.64
1 primi	1	H						
81	1	06/21/2019	14	732	1	9.3	0.02	29.33
1 primi	1	H						
81	1	06/22/2019	15	733	1	9.6	0.02	30.24
1 primi	1	H						
81	1	06/23/2019	16	734	1	9.3	0	31.3
1 primi	1	H						
81	1	06/24/2019	17	735	1	9.2	0.01	29.65
1 primi	1	H						
81	1	06/25/2019	18	736	1	9.6	0	32.23
1 primi	1	H						
81	1	06/26/2019	19	737	1	9.5	0.11	31.75
1 primi	1	H						
81	1	06/27/2019	20	738	1	9.6	0.03	32.89
1 primi	1	H						
81	1	06/28/2019	21	739	1	9.5	0.13	31.96
1 primi	1	H						
81	1	06/29/2019	22	740	1	9.6	0.2	30.88
1 primi	2	H						
81	1	06/30/2019	23	741	1	9.6	0.25	31.7
1 primi	2	H						
81	1	07/01/2019	24	742	1	9.5	0.24	32.11
1 primi	2	H						
81	1	07/02/2019	25	743	1	9.3	0.3	33.69
1 primi	2	H						
81	1	07/03/2019	26	744	1	9.8	0.02	33.05
1 primi	2	H						
81	1	07/04/2019	27	745	1	9.3	0.01	34.29
1 primi	2	H						
81	1	07/05/2019	28	746	1	9.9	0.04	34.67
1 primi	2	H						
81	1	07/06/2019	29	747	1	9.8	0.03	33.84
1 primi	2	H						
81	1	07/07/2019	30	748	1	9.2	0.35	31.35
1 primi	2	H						
81	1	07/08/2019	31	749	1	9.2	0.23	31.58
1 primi	2	H						
81	1	07/09/2019	32	750	1	8.7	0	33.56
1 primi	2	H						
81	1	07/10/2019	33	751	1	9.1	0.17	35.18
1 primi	2	H						
81	1	07/11/2019	34	752	1	8.7	0.02	32.33
1 primi	2	H						
81	1	07/12/2019	35	753	1	8.9	0.02	33.46
1 primi	2	H						
81	1	07/13/2019	36	754	1	9.2	0.31	35.46
1 primi	2	H						
81	1	07/14/2019	37	755	1	9.2	0	36.11
1 primi	2	H						
81	1	07/15/2019	38	756	1	9.2	0.05	37.54
1 primi	2	H						
81	1	07/16/2019	39	757	1	10.4	0.08	37.39

1 primi 2		H						
81	1	07/17/2019	40	758	1	9.6	0.19	34.81
1 primi 2		H						
81	1	07/18/2019	41	759	1	8.9	0.1	36.48
1 primi 2		H						
81	1	07/19/2019	42	760	1	9.2	0.15	37.45
1 primi 2		H						
81	1	07/20/2019	43	761	1	10	0.27	37.56
1 primi 2		H						
81	1	07/21/2019	44	762	1	10	0.25	37.77
1 primi 2		H						
81	1	07/22/2019	45	763	1	9.8	0.15	36.87
1 primi 2		H						
81	1	07/23/2019	46	764	1	9.6	0.17	39.49
1 primi 2		H						
81	1	07/24/2019	47	765	1	9.8	0.06	42.2
1 primi 2		H						
81	1	07/25/2019	48	766	1	9.2	0.04	41.96
1 primi 2		H						
81	1	07/26/2019	49	767	1	8.9	0.28	38.93
1 primi 2		H						
81	1	07/27/2019	50	768	1	9.1	0.18	43.31
1 primi 2		H						
81	1	07/28/2019	51	769	1	9.1	0.28	38.81
1 primi 2		H						
81	1	07/29/2019	52	770	1	9.5	0.03	40.51
1 primi 2		H						
81	1	07/30/2019	53	771	1	9.5	0.03	40.08
1 primi 2		H						
81	1	07/31/2019	54	772	1	9.6	0.01	42.05
1 primi 2		H						
81	1	08/01/2019	55	773	1	9.5	0.17	39.09
1 primi 2		H						
81	1	08/02/2019	56	774	1	9.5	0.09	42.19
1 primi 2		H						
81	1	08/03/2019	57	775	1	9.5	0.04	40.56
1 primi 2		H						
81	1	08/04/2019	58	776	1	9.2	0.27	38.86
1 primi 2		H						
81	1	08/05/2019	59	777	1	9.6	0.01	40.52
1 primi 2		H						
81	1	08/06/2019	60	778	1	9.6	0.04	42.04
1 primi 2		H						
81	1	08/07/2019	61	779	1	9.3	0.01	41.44
1 primi 2		H						
81	1	08/08/2019	62	780	1	9.6	0.21	40.92
1 primi 2		H						
81	1	08/09/2019	63	781	1	9.3	0.03	39.6
1 primi 2		H						
81	1	08/10/2019	64	782	1	9.5	0.09	39.31
1 primi 2		H						
81	1	08/11/2019	65	783	1	11.1	0.03	35.01
1 primi 2		H						
81	1	08/12/2019	66	784	1	10	0.33	38.64

1 primi	2	H						
81	1	08/13/2019	67	785	1	10	0.28	38
1 primi	2	H						
81	1	08/14/2019	68	786	1	9.9	0.06	37.64
1 primi	2	H						
81	1	08/15/2019	69	787	1	10	0.07	40.04
1 primi	2	H						
81	1	08/16/2019	70	788	1	9.8	0.2	40.61
1 primi	2	H						
81	1	08/17/2019	71	789	1	9.5	0.06	40.17
1 primi	2	H						
81	1	08/18/2019	72	790	1	9.1	0.04	41.22
1 primi	2	H						
81	1	08/19/2019	73	791	1	9.3	0.03	41.8
1 primi	2	H						
81	1	08/20/2019	74	792	1	9.2	0.34	40.01
1 primi	2	H						
81	1	08/21/2019	75	793	1	8.8	0.03	36.6
1 primi	2	H						
81	1	08/22/2019	76	794	1	8.8	0.17	33.06
1 primi	2	H						
81	1	08/23/2019	77	795	1	9.3	0.11	36.19
1 primi	2	H						
81	1	08/24/2019	78	796	1	9.3	0.03	38.76
1 primi	2	H						
81	1	08/25/2019	79	797	1	9.1	0.22	37.71
1 primi	2	H						
81	1	08/26/2019	80	798	1	9.3	0.17	36.39
1 primi	2	H						
81	1	08/27/2019	81	799	1	9.3	0.05	38.02
1 primi	2	H						
81	1	08/28/2019	82	800	1	9.6	0.4	35.53
1 primi	2	H						
81	1	08/29/2019	83	801	1	9.5	0.04	37.33
1 primi	2	H						
81	1	08/30/2019	84	802	1	9.9	0.19	35.42
1 primi	2	H						
81	1	08/31/2019	85	803	1	9.8	0.45	35.38
1 primi	2	H						
81	1	09/01/2019	86	804	1	9.5	0.27	36.47
1 primi	2	H						
81	1	09/02/2019	87	805	1	9.8	0.18	37.07
1 primi	2	H						
81	1	09/03/2019	88	806	1	9.8	0.03	38.06
1 primi	2	H						
81	1	09/04/2019	89	807	1	10.7	0.07	34.45
1 primi	2	H						
81	1	09/05/2019	90	808	1	9.6	0.1	35.57
1 primi	2	H						
81	1	09/06/2019	91	809	1	9.5	0.1	38.33
1 primi	2	H						
81	1	09/07/2019	92	810	1	9.9	0.17	37.38
1 primi	2	H						
81	1	09/08/2019	93	811	1	9.2	0.01	37.15

1 primi	2	H						
81	1	09/09/2019	94	812	1	9.5	0.02	39.3
1 primi	2	H						
81	1	09/10/2019	95	813	1	9.2	0.15	37.52
1 primi	2	H						
81	1	09/11/2019	96	814	1	9.2	0.05	40.17
1 primi	2	H						
81	1	09/12/2019	97	815	1	9.2	0.04	39.76
1 primi	2	H						
81	1	09/13/2019	98	816	1	9.5	0.19	38.11
1 primi	2	H						
81	1	09/14/2019	99	817	1	9.3	0.33	38.84
1 primi	2	H						
81	1	09/15/2019	100	818	1	9.6	0.07	37.75
1 primi	2	H						
81	1	09/16/2019	101	819	1	9.8	0.03	39.2
1 primi	2	H						
81	1	09/17/2019	102	820	1	9.5	0.05	40.44
1 primi	2	H						
81	1	09/18/2019	103	821	1	9.3	0.05	38.59
1 primi	2	H						
81	1	09/19/2019	104	822	1	9.8	0.18	37.78
1 primi	2	H						
81	1	09/20/2019	105	823	1	9.5	0.01	38.43
1 primi	2	H						
81	1	09/21/2019	106	824	1	9.8	0.28	39.56
1 primi	2	H						
81	1	09/22/2019	107	825	1	9.3	0.15	39.59
1 primi	2	H						
81	1	09/23/2019	108	826	1	9.8	0.16	42.08
1 primi	2	H						
81	1	09/24/2019	109	827	1	9.6	0.03	39.43
1 primi	2	H						
81	1	09/25/2019	110	828	1	9.6	0.15	40.1
1 primi	2	H						
81	1	09/26/2019	111	829	1	9.9	0.28	41.5
1 primi	2	H						
81	1	09/27/2019	112	830	1	9.8	0.04	40.2
1 primi	2	H						
81	1	09/28/2019	113	831	1	9.2	0.21	41.13
1 primi	2	H						
81	1	09/29/2019	114	832	1	9.8	0.03	41.28
1 primi	2	H						
81	1	09/30/2019	115	833	1	9.9	0	42.71
1 primi	2	H						
81	1	10/01/2019	116	834	1	9.5	0.23	42.03
1 primi	2	H						
81	1	10/02/2019	117	835	1	9.8	0.31	38.26
1 primi	2	H						
81	1	10/03/2019	118	836	1	9.5	0.2	38.16
1 primi	2	H						
81	1	10/04/2019	119	837	1	9.5	0.36	36.03
1 primi	2	H						
81	1	10/05/2019	120	838	1	9.5	0.24	41.18

1 primi 2		H						
81	1	10/06/2019	121	839	1	9.5	0.06	40.64
1 primi 2		H						
81	1	10/07/2019	122	840	1	9.8	0	41.81
1 primi 2		H						
81	1	10/08/2019	123	841	1	9.6	0.1	41.65
1 primi 2		H						
81	1	10/09/2019	124	842	1	9.5	0.03	40.9
1 primi 2		H						
81	1	10/10/2019	125	843	1	9.6	0.06	39.14
1 primi 2		H						
81	1	10/11/2019	126	844	1	9.3	0.04	41.05
1 primi 2		H						
81	1	10/12/2019	127	845	1	9.8	0.22	39.69
1 primi 2		H						
81	1	10/13/2019	128	846	1	9.8	0.23	39.32
1 primi 2		H						
81	1	10/14/2019	129	847	1	9.3	0.15	38.47
1 primi 2		H						
81	1	10/15/2019	130	848	1	9.8	0.11	40.31
1 primi 2		H						
81	1	10/16/2019	131	849	1	9.6	0	35.97
1 primi 2		H						
81	1	10/17/2019	132	850	1	9.6	0.04	38.01
1 primi 2		H						
81	1	10/18/2019	133	851	1	10	0	37.77
1 primi 2		H						
81	1	10/19/2019	134	852	1	10	0.05	39.18
1 primi 2		H						
81	1	10/20/2019	135	853	1	10.2	0.03	39.87
1 primi 2		H						
81	1	10/21/2019	136	854	1	10.2	0.03	39.09
1 primi 2		H						
81	1	10/22/2019	137	855	1	9.9	0	37.78
1 primi 2		H						
81	1	10/23/2019	138	856	1	10	0.04	39.24
1 primi 2		H						
81	1	10/24/2019	139	857	1	10	0.05	38.5
1 primi 2		H						
81	1	10/25/2019	140	858	1	9.9	0	38.54
1 primi 2		H						
81	1	10/26/2019	141	859	1	9.8	0.03	37.85
1 primi 2		H						
81	1	10/27/2019	142	860	1	10	0.01	38.7
1 primi 2		H						
81	1	10/28/2019	143	861	1	9.2	0.02	36.52
1 primi 2		H						
81	1	10/29/2019	144	862	1	9.9	0.04	36.86
1 primi 2		H						
81	1	10/30/2019	145	863	1	9.2	0.08	36.31
1 primi 2		H						
81	1	10/31/2019	146	864	1	9.5	0.07	39.18
1 primi 2		H						
81	1	11/01/2019	147	865	1	9.8	0.03	37.94

1 primi 2 H
81 1 11/02/2019 148 866 1 9.3 0 37.67
1 primi 2 H
81 1 11/03/2019 149 867 1 10.2 0 39.63
1 primi 2 H
81 1 11/04/2019 150 868 1 9.3 0.02 35.23
1 primi 2 H
81 1 11/05/2019 151 869 1 9.9 0.01 37.86
1 primi 2 H
81 1 11/06/2019 152 870 1 9.6 0.02 37.65
1 primi 2 H
81 1 11/07/2019 153 871 1 9.6 0 36.71
1 primi 2 H
81 1 11/08/2019 154 872 1 9.9 0.02 38.73
1 primi 2 H
81 1 11/09/2019 155 873 1 9.3 0 35.12
1 primi 2 H
81 1 11/10/2019 156 874 1 9.6 0 37.28
1 primi 2 H
81 1 11/11/2019 157 875 1 9.9 0 32.01
1 primi 2 H
81 1 11/12/2019 158 876 1 9.8 0 36.53
1 primi 2 H
81 1 11/13/2019 159 877 1 10.2 0.03 36.09
1 primi 2 H
81 1 11/14/2019 160 878 1 9.6 0 37.37
1 primi 2 H
81 1 11/15/2019 161 879 1 9.6 0.09 36.42
1 primi 2 H
81 1 11/16/2019 162 880 1 9.6 0.02 36.01
1 primi 2 H
81 1 11/17/2019 163 881 1 9.6 0.02 38.42
1 primi 2 H
81 1 11/18/2019 164 882 1 9.8 0 37.77
1 primi 2 H
81 1 11/19/2019 165 883 1 9.8 0.03 36.93
1 primi 2 H
81 1 11/20/2019 166 884 1 9.9 0.02 37.62
1 primi 2 H
81 1 06/18/2020 3 1095 2 10.2 0.3 28.9
2 multi 1 H
81 1 06/19/2020 4 1096 2 9.8 0.3 28.56
2 multi 1 H
81 1 06/20/2020 5 1097 2 9.9 0.09 32.63
2 multi 1 H
81 1 06/21/2020 6 1098 2 9.3 0.08 28.02
2 multi 1 H
81 1 06/22/2020 7 1099 2 11.4 0.07 35.35
2 multi 1 H
81 1 06/23/2020 8 1100 2 10 0.06 37.37
2 multi 1 H
81 1 06/24/2020 9 1101 2 9.8 0 38.28
2 multi 1 H
81 1 06/25/2020 10 1102 2 9.8 0 43.17

2 multi	1	H						
81	1	06/26/2020	11	1103	2	9.9	0	43.25
2 multi	1	H						
81	1	06/27/2020	12	1104	2	9.8	0	44.89
2 multi	1	H						
81	1	06/28/2020	13	1105	2	9.6	0	43.88
2 multi	1	H						
81	1	06/29/2020	14	1106	2	9.5	0	43.54
2 multi	1	H						
81	1	10/14/2020	121	1213	2	10.4	0.06	41.74
2 multi	2	H						
81	1	10/15/2020	122	1214	2	10.3	0.02	42.6
2 multi	2	H						
81	1	10/16/2020	123	1215	2	10.2	0.07	41
2 multi	2	H						
81	1	10/17/2020	124	1216	2	10.2	0.02	45.02
2 multi	2	H						
81	1	10/18/2020	125	1217	2	10.3	0.02	46.44
2 multi	2	H						
81	1	10/19/2020	126	1218	2	10	0.05	46.85
2 multi	2	H						
81	1	10/20/2020	127	1219	2	10	0.21	45
2 multi	2	H						
81	1	10/21/2020	128	1220	2	9.9	0.06	45.88
2 multi	2	H						
81	1	10/22/2020	129	1221	2	9.8	0	43.96
2 multi	2	H						
81	1	10/23/2020	130	1222	2	10.2	0	45.95
2 multi	2	H						
81	1	10/24/2020	131	1223	2	10	0	41.8
2 multi	2	H						
81	1	10/25/2020	132	1224	2	10.2	0.07	49.99
2 multi	2	H						
81	1	10/26/2020	133	1225	2	10.2	0	42.2
2 multi	2	H						
81	1	10/27/2020	134	1226	2	9.9	0.03	40.74
2 multi	2	H						
81	1	10/28/2020	135	1227	2	10	0.05	42.26
2 multi	2	H						
81	1	10/29/2020	136	1228	2	9.9	0.09	42.2
2 multi	2	H						
81	1	10/30/2020	137	1229	2	10.2	0.08	42.89
2 multi	2	H						
81	1	10/31/2020	138	1230	2	10.4	0.09	44.45
2 multi	2	H						
81	1	11/01/2020	139	1231	2	10.6	0.09	44.11
2 multi	2	H						
81	1	11/02/2020	140	1232	2	10.4	0	40.82
2 multi	2	H						
81	1	11/03/2020	141	1233	2	10	0.07	43.66
2 multi	2	H						
81	1	11/04/2020	142	1234	2	9.6	0.09	45.97
2 multi	2	H						
81	1	11/05/2020	143	1235	2	10.2	0.08	45.67

2 multi 2		H						
81	1	11/06/2020	144	1236	2	10	0.07	45.63
2 multi 2		H						
81	1	11/07/2020	145	1237	2	10.2	0.05	46.18
2 multi 2		H						
81	1	11/08/2020	146	1238	2	9.9	0.09	49.8
2 multi 2		H						
81	1	11/09/2020	147	1239	2	10.3	0.07	44.99
2 multi 2		H						
81	1	11/10/2020	148	1240	2	10.2	0.03	41.31
2 multi 2		H						
81	1	11/11/2020	149	1241	2	10.2	0.05	45.31
2 multi 2		H						
81	1	11/12/2020	150	1242	2	10	0.08	43.2
2 multi 2		H						
81	1	11/13/2020	151	1243	2	10.2	0.1	48.8
2 multi 2		H						
81	1	11/14/2020	152	1244	2	10.3	0.06	46.76
2 multi 2		H						
81	1	11/15/2020	153	1245	2	10.3	0.08	47.35
2 multi 2		H						
81	1	11/16/2020	154	1246	2	10.4	0.09	47.29
2 multi 2		H						
81	1	11/17/2020	155	1247	2	10.2	0.09	49.16
2 multi 2		H						
81	1	11/18/2020	156	1248	2	10.2	0.08	50.09
2 multi 2		H						
81	1	11/19/2020	157	1249	2	10.3	0.01	50.14
2 multi 2		H						
81	1	11/20/2020	158	1250	2	10	0.06	46.57
2 multi 2		H						
81	1	11/21/2020	159	1251	2	9.9	0.03	45.81
2 multi 2		H						
81	1	11/22/2020	160	1252	2	10.2	0.07	49.14
2 multi 2		H						
81	1	11/23/2020	161	1253	2	10	0.08	48.14
2 multi 2		H						
81	1	11/24/2020	162	1254	2	10.4	0.09	47.74
2 multi 2		H						
81	1	11/25/2020	163	1255	2	10.7	0.06	48.35
2 multi 2		H						
81	1	11/26/2020	164	1256	2	10.7	0.1	48.31
2 multi 2		H						
81	1	11/27/2020	165	1257	2	10.4	0.07	47.08
2 multi 2		H						
81	1	11/28/2020	166	1258	2	10	0.08	49.25
2 multi 2		H						
81	1	11/29/2020	167	1259	2	10.3	0.08	48.99
2 multi 2		H						
81	1	11/30/2020	168	1260	2	10.2	0.09	44.58
2 multi 2		H						
81	1	12/01/2020	169	1261	2	10.3	0.04	48.75
2 multi 2		H						
81	1	12/02/2020	170	1262	2	10.2	0.01	48.47

2 multi 2		H						
81	1	12/03/2020	171	1263	2	10.2	0.08	45.09
2 multi 2		H						
81	1	12/04/2020	172	1264	2	10.3	0.1	48.84
2 multi 2		H						
81	1	12/05/2020	173	1265	2	10	0.08	49.07
2 multi 2		H						
81	1	12/06/2020	174	1266	2	10.3	0.08	48.24
2 multi 2		H						
81	1	12/07/2020	175	1267	2	10.2	0.07	45.55
2 multi 2		H						
81	1	12/08/2020	176	1268	2	10.2	0	43.7
2 multi 2		H						
81	1	12/09/2020	177	1269	2	10	0.09	45
2 multi 2		H						
81	1	12/10/2020	178	1270	2	10.3	0.1	43.67
2 multi 2		H						
81	1	12/11/2020	179	1271	2	10.6	0.22	46.84
2 multi 2		H						
81	1	12/12/2020	180	1272	2	10.3	0.4	46.18
2 multi 2		H						
81	1	12/13/2020	181	1273	2	10.6	0.08	45.5
2 multi 2		H						
81	1	12/14/2020	182	1274	2	10.7	0.02	45.08
2 multi 2		H						
81	1	12/15/2020	183	1275	2	10.7	0	46.78
2 multi 2		H						
81	1	12/16/2020	184	1276	2	10.6	0	46.38
2 multi 2		H						
81	1	12/17/2020	185	1277	2	10.7	0.07	45.81
2 multi 2		H						
81	1	12/18/2020	186	1278	2	10.7	0.06	47.3
2 multi 2		H						
81	1	12/19/2020	187	1279	2	10.7	0.02	48.11
2 multi 2		H						
81	1	12/20/2020	188	1280	2	10.6	0.01	47.96
2 multi 2		H						
81	1	12/21/2020	189	1281	2	10.4	0.08	46.6
2 multi 2		H						
81	1	12/22/2020	190	1282	2	10.9	0.03	50.84
2 multi 2		H						
81	1	12/23/2020	191	1283	2	10.7	0.07	45.71
2 multi 2		H						
81	1	12/24/2020	192	1284	2	11.1	0.08	44.5
2 multi 2		H						
81	1	12/25/2020	193	1285	2	10.7	0.07	45.54
2 multi 2		H						
81	1	12/26/2020	194	1286	2	10.9	0.01	45.87
2 multi 2		H						
81	1	12/27/2020	195	1287	2	10.9	0.09	46.83
2 multi 2		H						
81	1	12/28/2020	196	1288	2	10.9	0.07	47.09
2 multi 2		H						
81	1	12/29/2020	197	1289	2	11.1	0.08	48

2 multi 2		H						
81	1	12/30/2020	198	1290	2	10.6	0.08	46.29
2 multi 2		H						
81	1	12/31/2020	199	1291	2	10.9	0.02	48.3
2 multi 2		H						
81	1	01/01/2021	200	1292	2	10.9	0.02	46.84
2 multi 2		H						
81	1	01/02/2021	201	1293	2	10.4	0.07	45.4
2 multi 2		H						
81	1	01/03/2021	202	1294	2	10.9	0.07	48.11
2 multi 2		H						
81	1	01/04/2021	203	1295	2	11.3	0.07	47.89
2 multi 2		H						
81	1	01/05/2021	204	1296	2	11.1	0.08	47.56
2 multi 2		H						
81	1	01/06/2021	205	1297	2	10.9	0.06	46.9
2 multi 2		H						
81	1	01/07/2021	206	1298	2	11.1	0.09	49.04
2 multi 2		H						
81	1	01/08/2021	207	1299	2	10.9	0.01	43.3
2 multi 2		H						
81	1	01/09/2021	208	1300	2	10.9	0.09	47.7
2 multi 2		H						
81	1	01/10/2021	209	1301	2	10.9	0.07	48.61
2 multi 2		H						
81	1	01/11/2021	210	1302	2	10.9	0.07	43.91
2 multi 2		H						
81	1	01/12/2021	211	1303	2	10.9	0.06	47.57
2 multi 2		H						
81	1	01/13/2021	212	1304	2	10.6	0.07	46.37
2 multi 2		H						
81	1	01/14/2021	213	1305	2	10.7	0.06	45.91
2 multi 2		H						
81	1	01/15/2021	214	1306	2	10.3	0	44.3
2 multi 2		H						
81	1	01/16/2021	215	1307	2	11.4	0.07	44.97
2 multi 2		H						
81	1	01/17/2021	216	1308	2	11.1	0.07	45.46
2 multi 2		H						
81	1	01/18/2021	217	1309	2	11.1	0	45.88
2 multi 2		H						
81	1	01/19/2021	218	1310	2	10.7	0.07	45.09
2 multi 2		H						
81	1	01/20/2021	219	1311	2	11.9	0.07	41.66
2 multi 2		H						
81	1	01/21/2021	220	1312	2	11.7	0	41.27
2 multi 2		H						
81	1	01/22/2021	221	1313	2	11.3	0	43.11
2 multi 2		H						
81	1	01/23/2021	222	1314	2	11.3	0	43.39
2 multi 2		H						
81	1	01/24/2021	223	1315	2	10.7	0.11	42.05
2 multi 2		H						
81	1	01/25/2021	224	1316	2	11.3	0	43.21

2 multi 2		H						
81	1	01/26/2021	225	1317	2	11.3	0.09	42.61
2 multi 2		H						
81	1	01/27/2021	226	1318	2	10.7	0	40.43
2 multi 2		H						
81	1	01/28/2021	227	1319	2	11.1	0.17	44
2 multi 2		H						
81	1	01/29/2021	228	1320	2	10.6	0	45.69
2 multi 2		H						
81	1	01/30/2021	229	1321	2	11.3	0.06	45.89
2 multi 2		H						
81	1	01/31/2021	230	1322	2	10.9	0.08	46.51
2 multi 2		H						
81	1	02/01/2021	231	1323	2	11.3	0	44.97
2 multi 2		H						
81	1	02/02/2021	232	1324	2	10.9	0.07	48.2
2 multi 2		H						
81	1	02/03/2021	233	1325	2	11.1	0.06	45.7
2 multi 2		H						
81	1	02/04/2021	234	1326	2	11.3	0.05	45.8
2 multi 2		H						
81	1	02/05/2021	235	1327	2	10.7	0.06	45.92
2 multi 2		H						
81	1	02/06/2021	236	1328	2	10.9	0.08	42.72
2 multi 2		H						
81	1	02/07/2021	237	1329	2	10.9	0	45.83
2 multi 2		H						
81	1	02/08/2021	238	1330	2	10.7	0.08	43.83
2 multi 2		H						
81	1	02/09/2021	239	1331	2	11.1	0.05	45.86
2 multi 2		H						
81	1	02/10/2021	240	1332	2	11.1	0.05	40.68
2 multi 2		H						
81	1	02/11/2021	241	1333	2	11.1	0.06	47.25
2 multi 2		H						
81	1	02/12/2021	242	1334	2	11.4	0.09	46.82
2 multi 2		H						
81	1	02/13/2021	243	1335	2	10.7	0.13	43.54
2 multi 2		H						
81	1	02/14/2021	244	1336	2	11.3	0.07	43.84
2 multi 2		H						
81	1	02/15/2021	245	1337	2	11.3	0	44.3
2 multi 2		H						
81	1	02/16/2021	246	1338	2	11.4	0	45.09
2 multi 2		H						
81	1	02/17/2021	247	1339	2	11.7	0	42.78
2 multi 2		H						
81	1	02/18/2021	248	1340	2	11.9	0.1	45.22
2 multi 2		H						
81	1	02/19/2021	249	1341	2	11.9	0.07	44.49
2 multi 2		H						
81	1	02/20/2021	250	1342	2	11.3	0	41.12
2 multi 2		H						
81	1	02/21/2021	251	1343	2	11.3	0	44.73

2 multi 2		H						
81	1	02/22/2021	252	1344	2	10.9	0	41.6
2 multi 2		H						
81	1	02/23/2021	253	1345	2	11.3	0	41.03
2 multi 2		H						
81	1	02/24/2021	254	1346	2	11.3	0.09	42.26
2 multi 2		H						
81	1	02/25/2021	255	1347	2	11.4	0.1	44.09
2 multi 2		H						
81	1	02/26/2021	256	1348	2	11.3	0.09	42.38
2 multi 2		H						
81	1	02/27/2021	257	1349	2	10.9	0	41.88
2 multi 2		H						
81	1	02/28/2021	258	1350	2	11.3	0.02	42.72
2 multi 2		H						
81	1	03/01/2021	259	1351	2	12	0.09	42.93
2 multi 2		H						
81	1	03/02/2021	260	1352	2	12	0.09	43.18
2 multi 2		H						
81	1	03/03/2021	261	1353	2	11.3	0.07	45.7
2 multi 2		H						
81	1	03/04/2021	262	1354	2	11.4	0.1	44.21
2 multi 2		H						
81	1	03/05/2021	263	1355	2	11.7	0.11	46.37
2 multi 2		H						
81	1	03/06/2021	264	1356	2	11.9	0.02	42.98
2 multi 2		H						
81	1	03/07/2021	265	1357	2	12.3	0.01	44.32
2 multi 2		H						
81	1	03/08/2021	266	1358	2	11.9	0.05	45.34
2 multi 2		H						
81	1	03/09/2021	267	1359	2	12.3	0.09	40.97
2 multi 2		H						
81	1	03/10/2021	268	1360	2	12.3	0.12	46.14
2 multi 2		H						
81	1	03/11/2021	269	1361	2	12	0.09	46.57
2 multi 2		H						
81	1	03/12/2021	270	1362	2	11.1	0.09	39.31
2 multi 2		H						
83	1	08/29/2019	3	681	1	10	0.19	22.75
1 primi 1		H						
83	1	08/30/2019	4	682	1	9.5	0.07	24.64
1 primi 1		H						
83	1	08/31/2019	5	683	1	9.9	0.09	24.35
1 primi 1		H						
83	1	09/01/2019	6	684	1	10.4	0.04	23.71
1 primi 1		H						
83	1	09/02/2019	7	685	1	9.9	0.25	25.76
1 primi 1		H						
83	1	09/03/2019	8	686	1	9.3	0.02	27.26
1 primi 1		H						
83	1	09/04/2019	9	687	1	9.9	0.02	28.49
1 primi 1		H						
83	1	09/05/2019	10	688	1	9.9	0.02	28.63

1 primi 1		H						
83	1	09/06/2019	11	689	1	9.6	0.02	28.2
1 primi 1		H						
83	1	09/07/2019	12	690	1	9.6	0.08	28.26
1 primi 1		H						
83	1	09/08/2019	13	691	1	9.1	0.01	28.39
1 primi 1		H						
83	1	09/09/2019	14	692	1	9.1	0.07	27.26
1 primi 1		H						
83	1	09/10/2019	15	693	1	8.9	0.17	29.16
1 primi 1		H						
83	1	09/11/2019	16	694	1	8.7	0.19	28.38
1 primi 1		H						
83	1	09/12/2019	17	695	1	8.8	0.08	30.19
1 primi 1		H						
83	1	09/13/2019	18	696	1	8.8	0.28	29.19
1 primi 1		H						
83	1	09/14/2019	19	697	1	9.2	0.05	30.34
1 primi 1		H						
83	1	09/15/2019	20	698	1	9.1	0	31.82
1 primi 1		H						
83	1	09/16/2019	21	699	1	9.3	0.33	30.74
1 primi 1		H						
83	1	09/17/2019	22	700	1	9.3	0.02	32.9
1 primi 2		H						
83	1	09/18/2019	23	701	1	8.9	0.31	30.47
1 primi 2		H						
83	1	09/19/2019	24	702	1	8.7	0.31	32.88
1 primi 2		H						
83	1	09/20/2019	25	703	1	9.2	0.04	31.41
1 primi 2		H						
83	1	09/21/2019	26	704	1	8.7	0.02	32.55
1 primi 2		H						
83	1	09/22/2019	27	705	1	8.9	0.23	32.83
1 primi 2		H						
83	1	09/23/2019	28	706	1	9.1	0	32.87
1 primi 2		H						
83	1	09/24/2019	29	707	1	8.7	0.2	33.63
1 primi 2		H						
83	1	09/25/2019	30	708	1	8.8	0	35.43
1 primi 2		H						
83	1	09/26/2019	31	709	1	8.8	0.06	34.38
1 primi 2		H						
83	1	09/27/2019	32	710	1	8.5	0	34.29
1 primi 2		H						
83	1	09/28/2019	33	711	1	8.7	0.25	32.5
1 primi 2		H						
83	1	09/29/2019	34	712	1	8.7	0.22	33.62
1 primi 2		H						
83	1	09/30/2019	35	713	1	8.5	0.17	35.71
1 primi 2		H						
83	1	10/01/2019	36	714	1	8.4	0	34.63
1 primi 2		H						
83	1	10/02/2019	37	715	1	8.9	0.16	35.06

1 primi 2		H						
83	1	10/03/2019	38	716	1	8.7	0.32	33.28
1 primi 2		H						
83	1	10/04/2019	39	717	1	8.6	0	34.79
1 primi 2		H						
83	1	10/05/2019	40	718	1	8.2	1.91	34.68
1 primi 2		H						
83	1	10/06/2019	41	719	1	8.2	1.95	34.94
1 primi 2		H						
83	1	10/07/2019	42	720	1	8.1	1.99	35.71
1 primi 2		H						
83	1	10/08/2019	43	721	1	8.4	1.95	36.2
1 primi 2		H						
83	1	10/09/2019	44	722	1	8.5	0	37.31
1 primi 2		H						
83	1	10/10/2019	45	723	1	8.5	1.95	37.18
1 primi 2		H						
83	1	10/11/2019	46	724	1	8.7	0.17	35.64
1 primi 2		H						
83	1	10/12/2019	47	725	1	8	0.17	34.68
1 primi 2		H						
83	1	10/13/2019	48	726	1	8.5	0.34	35.92
1 primi 2		H						
83	1	10/14/2019	49	727	1	8.4	0.04	35.04
1 primi 2		H						
83	1	10/15/2019	50	728	1	8.4	0.02	35.2
1 primi 2		H						
83	1	10/16/2019	51	729	1	8.3	0.12	34.64
1 primi 2		H						
83	1	10/17/2019	52	730	1	8.5	0.17	35.76
1 primi 2		H						
83	1	10/18/2019	53	731	1	8.5	0.06	35.67
1 primi 2		H						
83	1	10/19/2019	54	732	1	8.8	0.03	34.95
1 primi 2		H						
83	1	10/20/2019	55	733	1	8.8	0.15	35.75
1 primi 2		H						
83	1	10/21/2019	56	734	1	8.6	0.06	36.46
1 primi 2		H						
83	1	10/22/2019	57	735	1	8.8	0.28	35.95
1 primi 2		H						
83	1	10/23/2019	58	736	1	9.1	0	37.96
1 primi 2		H						
83	1	10/24/2019	59	737	1	8.9	0.06	36.15
1 primi 2		H						
83	1	10/25/2019	60	738	1	8.8	0.02	37.34
1 primi 2		H						
83	1	10/26/2019	61	739	1	8.8	0.34	36.51
1 primi 2		H						
83	1	10/27/2019	62	740	1	8.6	0.15	39.07
1 primi 2		H						
83	1	10/28/2019	63	741	1	8.4	0.01	36.9
1 primi 2		H						
83	1	10/29/2019	64	742	1	8.5	0.07	37.38

1 primi 2		H						
83	1	10/30/2019	65	743	1	8.6	0.16	35.76
1 primi 2		H						
83	1	10/31/2019	66	744	1	8.3	0.32	38.29
1 primi 2		H						
83	1	11/01/2019	67	745	1	8.3	0.28	38.07
1 primi 2		H						
83	1	11/02/2019	68	746	1	8.2	0.14	37.57
1 primi 2		H						
83	1	11/03/2019	69	747	1	8.7	0	37.44
1 primi 2		H						
83	1	11/04/2019	70	748	1	8.6	0.01	36.35
1 primi 2		H						
83	1	11/05/2019	71	749	1	8.9	0	37.29
1 primi 2		H						
83	1	11/06/2019	72	750	1	8.5	0.02	34.91
1 primi 2		H						
83	1	11/07/2019	73	751	1	8.4	0.31	32.12
1 primi 2		H						
83	1	11/08/2019	74	752	1	8.1	0.18	31.02
1 primi 2		H						
83	1	11/09/2019	75	753	1	8.5	0.09	33.84
1 primi 2		H						
83	1	11/10/2019	76	754	1	8.8	0	33.65
1 primi 2		H						
83	1	11/11/2019	77	755	1	8.5	0	32.64
1 primi 2		H						
83	1	11/12/2019	78	756	1	8.6	0	33.79
1 primi 2		H						
83	1	11/13/2019	79	757	1	8.4	0.28	36.11
1 primi 2		H						
83	1	11/14/2019	80	758	1	8.6	0	35.98
1 primi 2		H						
83	1	11/15/2019	81	759	1	8.5	0	35.51
1 primi 2		H						
83	1	11/16/2019	82	760	1	8.8	0.02	37.36
1 primi 2		H						
83	1	11/17/2019	83	761	1	8.8	0	37.11
1 primi 2		H						
83	1	11/18/2019	84	762	1	8.7	0	36.48
1 primi 2		H						
83	1	11/19/2019	85	763	1	8.7	0.15	37.54
1 primi 2		H						
83	1	11/20/2019	86	764	1	8.9	0	37.49
1 primi 2		H						
83	1	11/21/2019	87	765	1	8.7	0	37.31
1 primi 2		H						
83	1	11/22/2019	88	766	1	8.8	0.15	36.95
1 primi 2		H						
83	1	11/23/2019	89	767	1	8.7	0.19	37.8
1 primi 2		H						
83	1	11/24/2019	90	768	1	8.8	0.3	38.46
1 primi 2		H						
83	1	11/25/2019	91	769	1	8.6	0	36.3

1 primi 2 H
83 1 11/26/2019 92 770 1 9.1 0 38.35
1 primi 2 H
83 1 11/27/2019 93 771 1 8.8 0.03 35.96
1 primi 2 H
83 1 11/28/2019 94 772 1 8.5 0 37.31
1 primi 2 H
83 1 11/29/2019 95 773 1 8.5 0 38.38
1 primi 2 H
83 1 11/30/2019 96 774 1 8.7 0 37.34
1 primi 2 H
83 1 12/01/2019 97 775 1 8.7 0 36.57
1 primi 2 H
83 1 12/02/2019 98 776 1 8.7 0 36.25
1 primi 2 H
83 1 12/03/2019 99 777 1 8.7 0 37.71
1 primi 2 H
83 1 12/04/2019 100 778 1 8.5 0 35.62
1 primi 2 H
83 1 08/02/2020 4 1020 2 9.9 0.27 35.59
2 multi 1 H
83 1 08/03/2020 5 1021 2 10.2 0.24 32.82
2 multi 1 H
83 1 08/04/2020 6 1022 2 9.5 0.21 38.31
2 multi 1 H
83 1 08/05/2020 7 1023 2 9.3 0.17 38.8
2 multi 1 H
83 1 08/06/2020 8 1024 2 9.5 0.14 39.52
2 multi 1 H
83 1 08/07/2020 9 1025 2 9.9 0.01 41.09
2 multi 1 H
83 1 08/08/2020 10 1026 2 9.6 0.2 42.8
2 multi 1 H
83 1 08/09/2020 11 1027 2 9.2 0.16 44.21
2 multi 1 H
83 1 08/10/2020 12 1028 2 9.3 0.18 44.24
2 multi 1 H
83 1 08/11/2020 13 1029 2 9.2 0.26 45.53
2 multi 1 H
83 1 08/12/2020 14 1030 2 9.2 0.07 46.88
2 multi 1 H
83 1 08/13/2020 15 1031 2 9.2 0.09 46.52
2 multi 1 H
83 1 08/14/2020 16 1032 2 9.3 0.01 44.37
2 multi 1 H
83 1 08/15/2020 17 1033 2 9.6 0.2 44.82
2 multi 1 H
83 1 08/16/2020 18 1034 2 9.6 0.16 42.16
2 multi 1 H
83 1 08/17/2020 19 1035 2 9.5 0.31 46.96
2 multi 1 H
83 1 08/18/2020 20 1036 2 9.2 0.2 43.61
2 multi 1 H
83 1 08/19/2020 21 1037 2 9.6 0.06 43.93

2 multi 1		H						
83	1	08/20/2020	22	1038	2	9.8	0.17	45.16
2 multi 2		H						
83	1	08/21/2020	23	1039	2	9.9	0.05	45.36
2 multi 2		H						
83	1	08/22/2020	24	1040	2	9.9	0.23	44.79
2 multi 2		H						
83	1	08/23/2020	25	1041	2	9.2	0.27	46.43
2 multi 2		H						
83	1	08/24/2020	26	1042	2	9.8	0.24	46.07
2 multi 2		H						
83	1	08/25/2020	27	1043	2	9.5	0.21	47.01
2 multi 2		H						
83	1	08/26/2020	28	1044	2	9.3	0.22	46.09
2 multi 2		H						
83	1	08/27/2020	29	1045	2	9.3	0.28	45.99
2 multi 2		H						
83	1	08/28/2020	30	1046	2	9.5	0.18	44.86
2 multi 2		H						
83	1	08/29/2020	31	1047	2	9.2	0	43.32
2 multi 2		H						
83	1	08/30/2020	32	1048	2	9.6	0.21	47.99
2 multi 2		H						
83	1	08/31/2020	33	1049	2	9.3	0.23	50.42
2 multi 2		H						
83	1	09/01/2020	34	1050	2	9.2	0.24	51.85
2 multi 2		H						
83	1	09/02/2020	35	1051	2	9.3	0.24	48.09
2 multi 2		H						
83	1	09/03/2020	36	1052	2	9.6	0.28	48.26
2 multi 2		H						
83	1	09/04/2020	37	1053	2	9.8	0.04	52.46
2 multi 2		H						
83	1	09/05/2020	38	1054	2	9.5	0.05	46.65
2 multi 2		H						
83	1	09/06/2020	39	1055	2	9.5	0.1	46.01
2 multi 2		H						
83	1	09/07/2020	40	1056	2	9.5	0	44.66
2 multi 2		H						
83	1	09/08/2020	41	1057	2	9.1	0.19	42.47
2 multi 2		H						
83	1	09/09/2020	42	1058	2	8.6	0.26	38.03
2 multi 2		H						
83	1	09/10/2020	43	1059	2	9.3	0.3	43.89
2 multi 2		H						
83	1	09/11/2020	44	1060	2	9.3	0.64	48.77
2 multi 2		H						
83	1	09/12/2020	45	1061	2	9.2	0.22	47.53
2 multi 2		H						
83	1	09/13/2020	46	1062	2	9.5	0.12	50.69
2 multi 2		H						
83	1	09/14/2020	47	1063	2	9.6	0.16	46.55
2 multi 2		H						
83	1	09/15/2020	48	1064	2	8.9	0	43.29

2 multi	2	H						
83	1	09/16/2020	49	1065	2	9.2	0.1	48.32
2 multi	2	H						
83	1	09/17/2020	50	1066	2	8.8	0	43.42
2 multi	2	H						
83	1	09/18/2020	51	1067	2	9.2	0	42.63
2 multi	2	H						
83	1	09/19/2020	52	1068	2	9.8	0.21	47.24
2 multi	2	H						
83	1	09/20/2020	53	1069	2	9.6	0.35	43.39
2 multi	2	H						
83	1	09/21/2020	54	1070	2	9.6	0.02	45.54
2 multi	2	H						
83	1	09/22/2020	55	1071	2	9.6	0.01	49.33
2 multi	2	H						
83	1	09/23/2020	56	1072	2	9.3	0.03	44.12
2 multi	2	H						
83	1	09/24/2020	57	1073	2	9.3	0.05	48.77
2 multi	2	H						
83	1	09/25/2020	58	1074	2	9.5	0	47.5
2 multi	2	H						
83	1	09/26/2020	59	1075	2	9.2	0.28	50.16
2 multi	2	H						
83	1	09/27/2020	60	1076	2	9.5	0.31	50.51
2 multi	2	H						
83	1	09/28/2020	61	1077	2	9.3	0.28	49.59
2 multi	2	H						
83	1	09/29/2020	62	1078	2	9.5	0.01	50.97
2 multi	2	H						
83	1	09/30/2020	63	1079	2	9.5	0.21	48.26
2 multi	2	H						
83	1	10/01/2020	64	1080	2	9.2	0.22	50.97
2 multi	2	H						
83	1	10/02/2020	65	1081	2	9.6	0.3	49.69
2 multi	2	H						
83	1	10/03/2020	66	1082	2	9.3	0.16	52.71
2 multi	2	H						
83	1	10/04/2020	67	1083	2	9.1	0.36	50.39
2 multi	2	H						
83	1	10/05/2020	68	1084	2	9.3	0.01	49.74
2 multi	2	H						
83	1	10/06/2020	69	1085	2	9.3	0	45.86
2 multi	2	H						
83	1	10/07/2020	70	1086	2	8.9	0.12	39.17
2 multi	2	H						
83	1	10/08/2020	71	1087	2	9.2	0.28	35.47
2 multi	2	H						
83	1	10/09/2020	72	1088	2	9.2	0	39.42
2 multi	2	H						
83	1	10/10/2020	73	1089	2	8.8	0	44.18
2 multi	2	H						
83	1	10/11/2020	74	1090	2	9.2	0.02	47.73
2 multi	2	H						
83	1	10/12/2020	75	1091	2	9.3	0.16	47.98

2 multi 2	H
83	1	10/13/2020	76	1092	2	9.3	0.19	48.46
2 multi 2	H
83	1	10/14/2020	77	1093	2	9.6	0.17	48.02
2 multi 2	H
83	1	10/15/2020	78	1094	2	9.5	0.3	49.26
2 multi 2	H
83	1	10/16/2020	79	1095	2	9.3	0.28	48.9
2 multi 2	H
83	1	10/17/2020	80	1096	2	9.3	0	44.24
2 multi 2	H
83	1	10/18/2020	81	1097	2	9.3	0.06	52.43
2 multi 2	H
83	1	10/19/2020	82	1098	2	9.3	0.04	53.58
2 multi 2	H
83	1	10/20/2020	83	1099	2	9.3	0.31	53.78
2 multi 2	H
83	1	10/21/2020	84	1100	2	9.5	0.13	48.31
2 multi 2	H
83	1	10/22/2020	85	1101	2	9.1	0.2	52.56
2 multi 2	H
83	1	10/23/2020	86	1102	2	9.5	0.01	42.51
2 multi 2	H
83	1	10/24/2020	87	1103	2	9.2	0.21	23.94
2 multi 2	H
83	1	10/25/2020	88	1104	2	9.3	0.56	36.71
2 multi 2	H
83	1	10/26/2020	89	1105	2	9.5	0.07	38.98
2 multi 2	H
83	1	10/27/2020	90	1106	2	9.5	0.15	38.82
2 multi 2	H
83	1	10/28/2020	91	1107	2	9.3	0.12	42.4
2 multi 2	H
83	1	10/29/2020	92	1108	2	9.5	0.26	46.71
2 multi 2	H
83	1	10/30/2020	93	1109	2	9.6	0.15	48.8
2 multi 2	H
83	1	10/31/2020	94	1110	2	9.5	0	54.12
2 multi 2	H
83	1	11/01/2020	95	1111	2	9.6	0.25	56.35
2 multi 2	H
83	1	11/02/2020	96	1112	2	9.8	0.03	49.61
2 multi 2	H
83	1	11/03/2020	97	1113	2	9.6	0.17	43.86
2 multi 2	H
83	1	11/04/2020	98	1114	2	9.2	0.18	51.13
2 multi 2	H
83	1	11/05/2020	99	1115	2	9.6	0.33	45.64
2 multi 2	H
83	1	11/06/2020	100	1116	2	9.3	0.25	46.03
2 multi 2	H
83	1	11/07/2020	101	1117	2	9.3	0.28	48.01
2 multi 2	H
83	1	11/08/2020	102	1118	2	9.6	0.09	45.85

2 multi 2		H						
83	1	11/09/2020	103	1119	2	9.8	0.16	45.47
2 multi 2		H						
83	1	11/10/2020	104	1120	2	9.8	0.05	49.09
2 multi 2		H						
83	1	11/11/2020	105	1121	2	9.6	0.24	48.17
2 multi 2		H						
83	1	11/12/2020	106	1122	2	9.8	0.72	49.45
2 multi 2		H						
83	1	11/13/2020	107	1123	2	9.5	0.21	48.46
2 multi 2		H						
83	1	11/14/2020	108	1124	2	9.6	0.01	46.43
2 multi 2		H						
83	1	11/15/2020	109	1125	2	9.3	0.03	48.58
2 multi 2		H						
83	1	11/16/2020	110	1126	2	9.3	0	49.34
2 multi 2		H						
83	1	11/17/2020	111	1127	2	9.3	0.04	47.43
2 multi 2		H						
83	1	11/18/2020	112	1128	2	9.2	0.21	49.14
2 multi 2		H						
83	1	11/19/2020	113	1129	2	9.5	0.13	49.1
2 multi 2		H						
83	1	11/20/2020	114	1130	2	9.3	0.09	47.8
2 multi 2		H						
83	1	11/21/2020	115	1131	2	8.7	0.03	47.69
2 multi 2		H						
83	1	11/22/2020	116	1132	2	9.1	0.09	46.72
2 multi 2		H						
83	1	11/23/2020	117	1133	2	8.8	0.04	47.02
2 multi 2		H						
83	1	11/24/2020	118	1134	2	9.5	0.09	45.5
2 multi 2		H						
83	1	11/25/2020	119	1135	2	9.3	0	44.72
2 multi 2		H						
83	1	11/26/2020	120	1136	2	9.6	0.07	49.16
2 multi 2		H						
83	1	11/27/2020	121	1137	2	9.6	0.01	49.25
2 multi 2		H						
83	1	11/28/2020	122	1138	2	9.3	0.3	42.03
2 multi 2		H						
83	1	11/29/2020	123	1139	2	9.3	0.02	42.81
2 multi 2		H						
83	1	11/30/2020	124	1140	2	9.3	0.31	41.2
2 multi 2		H						
83	1	12/01/2020	125	1141	2	9.5	0.04	47.04
2 multi 2		H						
83	1	12/02/2020	126	1142	2	9.3	0.14	48.62
2 multi 2		H						
83	1	12/03/2020	127	1143	2	9.5	0.03	48.43
2 multi 2		H						
83	1	12/04/2020	128	1144	2	9.5	0.04	48.97
2 multi 2		H						
83	1	12/05/2020	129	1145	2	9.6	0.05	47.05

2 multi	2	H						
83	1	12/06/2020	130	1146	2	9.1	0.01	45.67
2 multi	2	H						
83	1	12/07/2020	131	1147	2	9.6	0.05	50.8
2 multi	2	H						
83	1	12/08/2020	132	1148	2	9.5	0.28	46.71
2 multi	2	H						
83	1	12/09/2020	133	1149	2	9.3	0.27	46.62
2 multi	2	H						
83	1	12/10/2020	134	1150	2	9.8	0.26	47.79
2 multi	2	H						
83	1	12/11/2020	135	1151	2	9.3	0.32	45.29
2 multi	2	H						
83	1	12/12/2020	136	1152	2	9.5	0.19	43.59
2 multi	2	H						
83	1	12/13/2020	137	1153	2	9.6	0.31	47.74
2 multi	2	H						
83	1	12/14/2020	138	1154	2	9.6	0.03	48.22
2 multi	2	H						
83	1	12/15/2020	139	1155	2	9.5	0	47.01
2 multi	2	H						
83	1	12/16/2020	140	1156	2	9.5	0	46.95
2 multi	2	H						
83	1	12/17/2020	141	1157	2	9.5	0.04	47.87
2 multi	2	H						
83	1	12/18/2020	142	1158	2	9.6	0	47.76
2 multi	2	H						
83	1	12/19/2020	143	1159	2	9.6	0.15	47.78
2 multi	2	H						
83	1	12/20/2020	144	1160	2	9.8	0	48.65
2 multi	2	H						
83	1	12/21/2020	145	1161	2	9.8	0.04	42.72
2 multi	2	H						
83	1	12/22/2020	146	1162	2	9.3	0.28	44.88
2 multi	2	H						
83	1	12/23/2020	147	1163	2	9.9	0.2	45.66
2 multi	2	H						
83	1	12/24/2020	148	1164	2	9.5	0.3	45.86
2 multi	2	H						
83	1	12/25/2020	149	1165	2	9.6	0.2	46.62
2 multi	2	H						
83	1	12/26/2020	150	1166	2	9.5	0.28	47.55
2 multi	2	H						
83	1	12/27/2020	151	1167	2	9.8	0.04	45.91
2 multi	2	H						
83	1	12/28/2020	152	1168	2	9.5	0.04	47.68
2 multi	2	H						
83	1	12/29/2020	153	1169	2	9.8	0.3	46.42
2 multi	2	H						
83	1	12/30/2020	154	1170	2	9.5	0.08	46
2 multi	2	H						
83	1	12/31/2020	155	1171	2	9.8	0.3	45.67
2 multi	2	H						
83	1	01/01/2021	156	1172	2	9.8	0.35	44.59

2 multi 2		H						
83	1	01/02/2021	157	1173	2	9.8	0.01	49.68
2 multi 2		H						
83	1	01/03/2021	158	1174	2	10	0.31	47.15
2 multi 2		H						
83	1	01/04/2021	159	1175	2	10	0	47.47
2 multi 2		H						
83	1	01/05/2021	160	1176	2	10	0.06	46.7
2 multi 2		H						
83	1	01/06/2021	161	1177	2	9.6	0.28	40.55
2 multi 2		H						
83	1	01/07/2021	162	1178	2	11.1	0.05	29.53
2 multi 2		H						
83	1	01/08/2021	163	1179	2	10.7	0.08	46.45
2 multi 2		H						
83	1	01/09/2021	164	1180	2	10.7	0.12	41.76
2 multi 2		H						
83	1	01/10/2021	165	1181	2	10.6	0	44.21
2 multi 2		H						
83	1	01/11/2021	166	1182	2	10.4	0.1	45.61
2 multi 2		H						
83	1	01/12/2021	167	1183	2	9.9	0.14	49.61
2 multi 2		H						
83	1	01/13/2021	168	1184	2	10.2	0.27	45.7
2 multi 2		H						
83	1	01/14/2021	169	1185	2	10.2	0.02	47.15
2 multi 2		H						
83	1	01/15/2021	170	1186	2	10	0	42.65
2 multi 2		H						
83	1	01/16/2021	171	1187	2	9.9	0.04	46.61
2 multi 2		H						
83	1	01/17/2021	172	1188	2	9.2	0.25	43.36
2 multi 2		H						
83	1	01/18/2021	173	1189	2	10.3	0.09	48.46
2 multi 2		H						
83	1	01/19/2021	174	1190	2	10.2	0.21	47.98
2 multi 2		H						
83	1	01/20/2021	175	1191	2	10.2	0	42.39
2 multi 2		H						
83	1	01/21/2021	176	1192	2	10.2	0	47.51
2 multi 2		H						
83	1	01/22/2021	177	1193	2	10.3	0	47.3
2 multi 2		H						
83	1	01/23/2021	178	1194	2	10.3	0	46.02
2 multi 2		H						
83	1	01/24/2021	179	1195	2	10.3	0	43.25
2 multi 2		H						
83	1	01/25/2021	180	1196	2	10.7	0	41.3
2 multi 2		H						
83	1	01/26/2021	181	1197	2	10.4	0	44.73
2 multi 2		H						
83	1	01/27/2021	182	1198	2	10.3	0.46	42.9
2 multi 2		H						
83	1	01/28/2021	183	1199	2	10.4	0.04	43.92

2 multi 2		H						
83	1	01/29/2021	184	1200	2	10.2	0.12	43.85
2 multi 2		H						
83	1	01/30/2021	185	1201	2	10.2	0.05	47.51
2 multi 2		H						
83	1	01/31/2021	186	1202	2	10.7	0.23	38.88
2 multi 2		H						
83	1	02/01/2021	187	1203	2	10.4	0.35	37.81
2 multi 2		H						
83	1	02/02/2021	188	1204	2	10.2	0.37	44.64
2 multi 2		H						
83	1	02/03/2021	189	1205	2	10.4	0.18	44.95
2 multi 2		H						
83	1	02/04/2021	190	1206	2	11.1	0.07	47.11
2 multi 2		H						
83	1	02/05/2021	191	1207	2	10.6	0.05	43.28
2 multi 2		H						
83	1	02/06/2021	192	1208	2	10.2	0.05	44.73
2 multi 2		H						
83	1	02/07/2021	193	1209	2	10.2	0.01	24.32
2 multi 2		H						
83	1	02/08/2021	194	1210	2	10.3	0	43.86
2 multi 2		H						
83	1	02/09/2021	195	1211	2	10.3	0.06	44.28
2 multi 2		H						
83	1	02/10/2021	196	1212	2	10.4	0	38.82
2 multi 2		H						
83	1	02/11/2021	197	1213	2	11.3	0.05	43.24
2 multi 2		H						
83	1	02/12/2021	198	1214	2	10.9	0.03	34.62
2 multi 2		H						
83	1	02/13/2021	199	1215	2	10.3	0.1	40.01
2 multi 2		H						
83	1	02/14/2021	200	1216	2	10.3	0.49	41.92
2 multi 2		H						
83	1	02/15/2021	201	1217	2	10.4	0.02	39.33
2 multi 2		H						
83	1	02/16/2021	202	1218	2	10.6	0.06	35.27
2 multi 2		H						
83	1	02/17/2021	203	1219	2	10.7	0.11	41.3
2 multi 2		H						
83	1	02/18/2021	204	1220	2	10	0.11	44.75
2 multi 2		H						
83	1	02/19/2021	205	1221	2	10.4	0.02	42.04
2 multi 2		H						
83	1	02/20/2021	206	1222	2	10.6	0.04	43.11
2 multi 2		H						
83	1	02/21/2021	207	1223	2	10.3	0	40.32
2 multi 2		H						
83	1	02/22/2021	208	1224	2	10.4	0.03	46.27
2 multi 2		H						
83	1	02/23/2021	209	1225	2	10.6	0.02	41.71
2 multi 2		H						
83	1	02/24/2021	210	1226	2	10.7	0	40.22

2 multi 2		H						
83	1	02/25/2021	211	1227	2	10.6	0.04	45.36
2 multi 2		H						
83	1	02/26/2021	212	1228	2	11.3	0.04	45.59
2 multi 2		H						
83	1	02/27/2021	213	1229	2	10.7	0	37.38
2 multi 2		H						
83	1	02/28/2021	214	1230	2	10.7	0	42.06
2 multi 2		H						
83	1	03/01/2021	215	1231	2	11.1	0.02	44.35
2 multi 2		H						
83	1	03/02/2021	216	1232	2	11.3	0.03	44.23
2 multi 2		H						
83	1	03/03/2021	217	1233	2	10.9	0	45.15
2 multi 2		H						
83	1	03/04/2021	218	1234	2	11.1	0.32	43.96
2 multi 2		H						
83	1	03/05/2021	219	1235	2	10.9	0	41.5
2 multi 2		H						
83	1	03/06/2021	220	1236	2	10.9	0.03	43.02
2 multi 2		H						
83	1	03/07/2021	221	1237	2	10.4	0.13	43.71
2 multi 2		H						
83	1	03/08/2021	222	1238	2	10.9	0.04	45.04
2 multi 2		H						
83	1	03/09/2021	223	1239	2	10.6	0.04	44.72
2 multi 2		H						
83	1	03/10/2021	224	1240	2	10.6	0.05	46.49
2 multi 2		H						
83	1	03/11/2021	225	1241	2	10.6	0.05	39.02
2 multi 2		H						
83	1	03/12/2021	226	1242	2	10.4	0.03	48.71
2 multi 2		H						
83	1	03/13/2021	227	1243	2	11.3	0.02	47.9
2 multi 2		H						
83	1	03/14/2021	228	1244	2	10.6	0.34	46.6
2 multi 2		H						
83	1	03/15/2021	229	1245	2	10.7	0.32	44.62
2 multi 2		H						
83	1	03/16/2021	230	1246	2	10.6	0.03	44.71
2 multi 2		H						
83	1	03/17/2021	231	1247	2	10.7	0	41.25
2 multi 2		H						
83	1	03/18/2021	232	1248	2	10.6	0	39.29
2 multi 2		H						
83	1	03/19/2021	233	1249	2	11.1	0.04	43.36
2 multi 2		H						
83	1	03/20/2021	234	1250	2	10.3	0.32	41.89
2 multi 2		H						
83	1	03/21/2021	235	1251	2	10.9	0.27	45.01
2 multi 2		H						
83	1	03/22/2021	236	1252	2	11.1	0.04	42.91
2 multi 2		H						
83	1	03/23/2021	237	1253	2	10.9	0	45.02

2 multi	2	H						
83	1	03/24/2021	238	1254	2	10.2	0.31	45.98
2 multi	2	H						
83	1	03/25/2021	239	1255	2	11.3	0.03	45.11
2 multi	2	H						
83	1	03/26/2021	240	1256	2	10.7	0.32	45.21
2 multi	2	H						
83	1	03/27/2021	241	1257	2	10.4	0.26	45.73
2 multi	2	H						
83	1	03/28/2021	242	1258	2	10	0.38	40.73
2 multi	2	H						
83	1	03/29/2021	243	1259	2	10.2	0.3	45.82
2 multi	2	H						
83	1	03/30/2021	244	1260	2	10.6	0.01	43.9
2 multi	2	H						
83	1	03/31/2021	245	1261	2	10.4	0.41	43.25
2 multi	2	H						
83	1	04/01/2021	246	1262	2	10.6	0.02	45.18
2 multi	2	H						
83	1	04/02/2021	247	1263	2	10.6	0	41.64
2 multi	2	H						
83	1	04/03/2021	248	1264	2	10.7	0.42	43.71
2 multi	2	H						
83	1	04/04/2021	249	1265	2	10.9	0.03	43.2
2 multi	2	H						
83	1	04/05/2021	250	1266	2	10.7	0.02	43.53
2 multi	2	H						
83	1	04/06/2021	251	1267	2	10.4	0.02	38.49
2 multi	2	H						
83	1	04/07/2021	252	1268	2	10.6	0.01	39.97
2 multi	2	H						
83	1	04/08/2021	253	1269	2	10.9	0.01	41.26
2 multi	2	H						
83	1	04/09/2021	254	1270	2	10.9	0.13	42.84
2 multi	2	H						
83	1	04/10/2021	255	1271	2	11.4	0.02	45.8
2 multi	2	H						
83	1	04/11/2021	256	1272	2	10.6	0.33	41.41
2 multi	2	H						
83	1	04/12/2021	257	1273	2	10.9	0	42.8
2 multi	2	H						
83	1	04/13/2021	258	1274	2	11.1	0.01	43.73
2 multi	2	H						
83	1	04/14/2021	259	1275	2	10.7	0.03	44.29
2 multi	2	H						
83	1	04/15/2021	260	1276	2	10.3	0.03	45.26
2 multi	2	H						
83	1	04/16/2021	261	1277	2	11.1	0.04	45.01
2 multi	2	H						
83	1	04/17/2021	262	1278	2	10.3	0.35	42.9
2 multi	2	H						
83	1	04/18/2021	263	1279	2	11.3	0.03	41.44
2 multi	2	H						
83	1	04/19/2021	264	1280	2	10.7	0.19	46.35

2 multi 2		H						
83	1	04/20/2021	265	1281	2	10.6	0	41.49
2 multi 2		H						
83	1	04/21/2021	266	1282	2	10.4	0.04	43.4
2 multi 2		H						
83	1	04/22/2021	267	1283	2	10.4	0.05	43.6
2 multi 2		H						
83	1	04/23/2021	268	1284	2	10.6	0.02	44.73
2 multi 2		H						
83	1	04/24/2021	269	1285	2	11.3	0.02	44.45
2 multi 2		H						
83	1	04/25/2021	270	1286	2	10.4	0.04	43.85
2 multi 2		H						
83	1	04/26/2021	271	1287	2	10.3	0	43.73
2 multi 2		H						
83	1	04/27/2021	272	1288	2	10.6	0	43.95
2 multi 2		H						
83	1	04/28/2021	273	1289	2	10.6	0	43.14
2 multi 2		H						
83	1	04/29/2021	274	1290	2	11.1	0.08	43.65
2 multi 2		H						
83	1	04/30/2021	275	1291	2	10.7	0	44.63
2 multi 2		H						
83	1	05/01/2021	276	1292	2	11.1	0.04	41.53
2 multi 2		H						
83	1	05/02/2021	277	1293	2	11.1	0.05	37.79
2 multi 2		H						
83	1	05/03/2021	278	1294	2	11.4	0.04	39.73
2 multi 2		H						
83	1	05/04/2021	279	1295	2	11.1	0.04	37.13
2 multi 2		H						
83	1	05/05/2021	280	1296	2	10.4	0.04	40.45
2 multi 2		H						
83	1	05/06/2021	281	1297	2	10.4	0.04	41.86
2 multi 2		H						
84	1	08/08/2020	7	825	1	9.9	0.18	23.15
1 primi 1		H						
84	1	08/09/2020	8	826	1	9.5	0.02	21.95
1 primi 1		H						
84	1	08/10/2020	9	827	1	9.3	0.01	23.24
1 primi 1		H						
84	1	08/11/2020	10	828	1	9.3	0	25.04
1 primi 1		H						
84	1	08/12/2020	11	829	1	9.6	0.12	26.42
1 primi 1		H						
84	1	08/13/2020	12	830	1	9.5	0.01	25.57
1 primi 1		H						
84	1	08/14/2020	13	831	1	9.5	0.14	25.69
1 primi 1		H						
84	1	08/15/2020	14	832	1	8.8	0.01	25.45
1 primi 1		H						
84	1	08/16/2020	15	833	1	9.1	0.44	24.81
1 primi 1		H						
84	1	08/17/2020	16	834	1	9.6	0.34	28.38

1 primi 1 H
84 1 08/18/2020 17 835 1 9.5 0.44 26.23
1 primi 1 H
84 1 08/19/2020 18 836 1 9.8 0.36 28.55
1 primi 1 H
84 1 08/20/2020 19 837 1 9.6 0.05 27.19
1 primi 1 H
84 1 08/21/2020 20 838 1 9.3 0.19 29.84
1 primi 1 H
84 1 08/22/2020 21 839 1 9.3 0.07 29.33
1 primi 1 H
84 1 08/23/2020 22 840 1 9.1 0.32 28.95
1 primi 2 H
84 1 08/24/2020 23 841 1 9.1 0.26 29.17
1 primi 2 H
84 1 08/25/2020 24 842 1 8.9 0.39 30.64
1 primi 2 H
84 1 08/26/2020 25 843 1 9.1 0.38 30.59
1 primi 2 H
84 1 08/27/2020 26 844 1 8.8 0.14 29.34
1 primi 2 H
84 1 08/28/2020 27 845 1 8.9 0.21 32.4
1 primi 2 H
84 1 08/29/2020 28 846 1 8.4 0.04 32.09
1 primi 2 H
84 1 08/30/2020 29 847 1 8.6 0 31.95
1 primi 2 H
84 1 08/31/2020 30 848 1 8.8 0.15 33.42
1 primi 2 H
84 1 09/01/2020 31 849 1 9.1 0.05 32.81
1 primi 2 H
84 1 09/02/2020 32 850 1 8.9 0.21 32.6
1 primi 2 H
84 1 09/03/2020 33 851 1 9.1 0.07 30.9
1 primi 2 H
84 1 09/04/2020 34 852 1 9.1 0 31.94
1 primi 2 H
84 1 09/05/2020 35 853 1 9.1 0.33 31.7
1 primi 2 H
84 1 09/06/2020 36 854 1 9.1 0 29.76
1 primi 2 H
84 1 09/07/2020 37 855 1 8.9 0.19 32.15
1 primi 2 H
84 1 09/08/2020 38 856 1 9.1 0 30.69
1 primi 2 H
84 1 09/09/2020 39 857 1 8.9 0.76 31.25
1 primi 2 H
84 1 09/10/2020 40 858 1 9.3 0.06 31.15
1 primi 2 H
84 1 09/11/2020 41 859 1 9.3 0.06 31.94
1 primi 2 H
84 1 09/12/2020 42 860 1 9.1 0.01 29.74
1 primi 2 H
84 1 09/13/2020 43 861 1 9.2 0.13 33.87

1 primi 2		H						
84	1	09/14/2020	44	862	1	9.3	0	30.14
1 primi 2		H						
84	1	09/15/2020	45	863	1	9.3	0.35	33.49
1 primi 2		H						
84	1	09/16/2020	46	864	1	9.5	0.27	36.18
1 primi 2		H						
84	1	09/17/2020	47	865	1	9.6	0	34.62
1 primi 2		H						
84	1	09/18/2020	48	866	1	9.1	0	35.74
1 primi 2		H						
84	1	09/19/2020	49	867	1	9.3	0	32.66
1 primi 2		H						
84	1	09/20/2020	50	868	1	9.3	0.06	35.3
1 primi 2		H						
84	1	09/21/2020	51	869	1	9.2	0.1	35.19
1 primi 2		H						
84	1	09/22/2020	52	870	1	9.6	0	35.01
1 primi 2		H						
84	1	09/23/2020	53	871	1	9.3	0.51	32.86
1 primi 2		H						
84	1	09/24/2020	54	872	1	9.8	0.05	34.33
1 primi 2		H						
84	1	09/25/2020	55	873	1	9.1	0.26	36.26
1 primi 2		H						
84	1	09/26/2020	56	874	1	9.1	0.11	36.5
1 primi 2		H						
84	1	09/27/2020	57	875	1	9.2	0.65	36.58
1 primi 2		H						
84	1	09/28/2020	58	876	1	9.3	0.05	33.84
1 primi 2		H						
84	1	09/29/2020	59	877	1	8.8	0.44	35.06
1 primi 2		H						
84	1	09/30/2020	60	878	1	9.6	0	36.39
1 primi 2		H						
84	1	10/01/2020	61	879	1	9.5	0.03	35.25
1 primi 2		H						
84	1	10/02/2020	62	880	1	9.9	0	36.04
1 primi 2		H						
84	1	10/03/2020	63	881	1	9.6	0.05	35.19
1 primi 2		H						
84	1	10/04/2020	64	882	1	9.9	0	36.28
1 primi 2		H						
84	1	10/05/2020	65	883	1	9.5	0.12	34.74
1 primi 2		H						
84	1	10/06/2020	66	884	1	9.8	0.05	36.06
1 primi 2		H						
84	1	10/07/2020	67	885	1	9.6	0.14	34.71
1 primi 2		H						
84	1	10/08/2020	68	886	1	9.6	0.05	33.33
1 primi 2		H						
84	1	10/09/2020	69	887	1	9.8	0.15	34.7
1 primi 2		H						
84	1	10/10/2020	70	888	1	10	0.13	34.05

1 primi	2	H						
84	1	10/11/2020	71	889	1	9.6	0.13	33.51
1 primi	2	H						
84	1	10/12/2020	72	890	1	9.8	0.01	34.32
1 primi	2	H						
84	1	10/13/2020	73	891	1	9.2	0.05	33.86
1 primi	2	H						
85	1	10/11/2020	9	778	1	9.5	0.2	20.05
1 primi	1	C						
85	1	10/12/2020	10	779	1	9.9	0.06	19.79
1 primi	1	C						
85	1	10/13/2020	11	780	1	9.9	0.04	19.68
1 primi	1	C						
85	1	10/14/2020	12	781	1	10	0.13	20.53
1 primi	1	C						
85	1	10/15/2020	13	782	1	9.9	0.06	20.31
1 primi	1	C						
85	1	10/16/2020	14	783	1	9.9	0.18	22.35
1 primi	1	C						
85	1	10/17/2020	15	784	1	9.2	0.18	22.89
1 primi	1	C						
85	1	10/18/2020	16	785	1	9.5	0.12	23.77
1 primi	1	C						
85	1	10/19/2020	17	786	1	9.6	0.18	25.6
1 primi	1	C						
85	1	10/20/2020	18	787	1	10	0.11	24.27
1 primi	1	C						
85	1	10/21/2020	19	788	1	9.5	0.18	24.88
1 primi	1	C						
85	1	10/22/2020	20	789	1	9.6	0.15	25.36
1 primi	1	C						
85	1	10/23/2020	21	790	1	9.3	0.24	27.25
1 primi	1	C						
85	1	10/24/2020	22	791	1	9.6	0.15	25.32
1 primi	2	C						
85	1	10/25/2020	23	792	1	9.5	0.07	27.15
1 primi	2	C						
85	1	10/26/2020	24	793	1	9.5	0.03	25.78
1 primi	2	C						
85	1	10/27/2020	25	794	1	9.3	0.08	25.28
1 primi	2	C						
85	1	10/28/2020	26	795	1	9.3	0.17	25.77
1 primi	2	C						
85	1	10/29/2020	27	796	1	9.2	0.09	25.64
1 primi	2	C						
85	1	10/30/2020	28	797	1	9.2	0.1	27.15
1 primi	2	C						
85	1	10/31/2020	29	798	1	0	0.21	33.17
1 primi	2	C						
85	1	11/01/2020	30	799	1	10.2	0.05	27.98
1 primi	2	C						
85	1	11/02/2020	31	800	1	10	0.02	26.81
1 primi	2	C						
85	1	11/03/2020	32	801	1	9.6	0.18	26.46

1 primi	2	C						
85	1	11/04/2020	33	802	1	9.2	0.12	25.43
1 primi	2	C						
85	1	11/05/2020	34	803	1	9.6	0.08	27.55
1 primi	2	C						
85	1	11/06/2020	35	804	1	9.5	0.17	27
1 primi	2	C						
85	1	11/07/2020	36	805	1	9.5	0.09	27.05
1 primi	2	C						
85	1	11/08/2020	37	806	1	9.2	0.14	25.57
1 primi	2	C						
85	1	11/09/2020	38	807	1	9.3	0.19	26.43
1 primi	2	C						
85	1	11/10/2020	39	808	1	9.6	0.16	27.77
1 primi	2	C						
85	1	11/11/2020	40	809	1	9.2	0.09	26.26
1 primi	2	C						
85	1	11/12/2020	41	810	1	9.3	0.04	27.42
1 primi	2	C						
85	1	11/13/2020	42	811	1	9.6	0.15	28.56
1 primi	2	C						
86	1	08/11/2017	8	709	1	9.9	0	21.18
1 primi	1	H						
86	1	08/12/2017	9	710	1	8.8	0.1	21.28
1 primi	1	H						
86	1	08/13/2017	10	711	1	9.2	0.03	25.01
1 primi	1	H						
86	1	08/14/2017	11	712	1	9.3	0	22.48
1 primi	1	H						
86	1	08/15/2017	12	713	1	8.9	0	26.55
1 primi	1	H						
86	1	08/16/2017	13	714	1	9.5	0	26.62
1 primi	1	H						
86	1	08/17/2017	14	715	1	9.5	0	27.31
1 primi	1	H						
86	1	08/18/2017	15	716	1	9.6	0.19	27.25
1 primi	1	H						
86	1	08/19/2017	16	717	1	8.9	0.07	26.54
1 primi	1	H						
86	1	08/20/2017	17	718	1	9.1	0.02	25.91
1 primi	1	H						
86	1	08/21/2017	18	719	1	9.8	0.05	29.06
1 primi	1	H						
86	1	08/22/2017	19	720	1	9.8	0.08	26.98
1 primi	1	H						
86	1	08/23/2017	20	721	1	9.2	0	26.73
1 primi	1	H						
86	1	08/24/2017	21	722	1	9.1	0	25.39
1 primi	1	H						
86	1	08/25/2017	22	723	1	8.6	0	26.44
1 primi	2	H						
86	1	08/26/2017	23	724	1	9.2	0	25.72
1 primi	2	H						
86	1	08/27/2017	24	725	1	9.6	0.09	26.58

1 primi 2 H
86 1 08/28/2017 25 726 1 8.9 0 27.43
1 primi 2 H
86 1 08/29/2017 26 727 1 9.3 0 28.95
1 primi 2 H
86 1 08/30/2017 27 728 1 9.6 0 28.19
1 primi 2 H
86 1 08/31/2017 28 729 1 9.6 0 29.09
1 primi 2 H
86 1 09/01/2017 29 730 1 9.2 0 27.29
1 primi 2 H
86 1 09/02/2017 30 731 1 8.9 0 28.73
1 primi 2 H
86 1 09/03/2017 31 732 1 9.8 0 28.8
1 primi 2 H
86 1 09/04/2017 32 733 1 9.2 0.2 30.78
1 primi 2 H
86 1 09/05/2017 33 734 1 9.2 0 29.7
1 primi 2 H
86 1 09/06/2017 34 735 1 9.1 0 26.49
1 primi 2 H
86 1 09/07/2017 35 736 1 9.8 0.14 33.19
1 primi 2 H
86 1 09/08/2017 36 737 1 9.3 0.05 27.6
1 primi 2 H
86 1 09/09/2017 37 738 1 8.8 0.17 31.88
1 primi 2 H
86 1 09/10/2017 38 739 1 9.2 0.18 29.11
1 primi 2 H
86 1 09/11/2017 39 740 1 9.5 0 31.83
1 primi 2 H
86 1 09/12/2017 40 741 1 9.1 0.04 30.4
1 primi 2 H
86 1 09/13/2017 41 742 1 9.3 0.55 31.99
1 primi 2 H
86 1 09/14/2017 42 743 1 8.8 0 31.49
1 primi 2 H
86 1 09/15/2017 43 744 1 9.5 0 32.59
1 primi 2 H
86 1 09/16/2017 44 745 1 9.6 0.01 28.41
1 primi 2 H
86 1 09/17/2017 45 746 1 9.9 0.1 33.41
1 primi 2 H
86 1 09/18/2017 46 747 1 8.9 0.25 28.03
1 primi 2 H
86 1 09/19/2017 47 748 1 9.2 0 34.51
1 primi 2 H
86 1 09/20/2017 48 749 1 8.9 0 29.71
1 primi 2 H
86 1 09/21/2017 49 750 1 9.3 0 29.91
1 primi 2 H
86 1 09/22/2017 50 751 1 9.1 0.17 34.84
1 primi 2 H
86 1 09/23/2017 51 752 1 9.5 0 29.82

1 primi 2		H						
86	1	09/24/2017	52	753	1	9.2	0	32.39
1 primi 2		H						
86	1	09/25/2017	53	754	1	8.8	0.17	32.59
1 primi 2		H						
86	1	09/26/2017	54	755	1	9.3	0.1	29.24
1 primi 2		H						
86	1	09/27/2017	55	756	1	8.8	0.05	34.79
1 primi 2		H						
86	1	09/28/2017	56	757	1	8.9	0.1	29.51
1 primi 2		H						
86	1	09/29/2017	57	758	1	8.8	0.05	27.99
1 primi 2		H						
86	1	09/30/2017	58	759	1	8.7	0.71	32.53
1 primi 2		H						
86	1	10/01/2017	59	760	1	9.1	0.01	29.01
1 primi 2		H						
86	1	10/02/2017	60	761	1	9.8	0.02	34.38
1 primi 2		H						
86	1	10/03/2017	61	762	1	9.2	0.06	34.26
1 primi 2		H						
86	1	10/04/2017	62	763	1	9.3	0.16	36.91
1 primi 2		H						
86	1	10/05/2017	63	764	1	9.6	0	33.23
1 primi 2		H						
86	1	10/06/2017	64	765	1	9.5	0.17	33.03
1 primi 2		H						
86	1	10/07/2017	65	766	1	9.2	0	34.55
1 primi 2		H						
86	1	10/08/2017	66	767	1	8.8	0	29.4
1 primi 2		H						
86	1	10/09/2017	67	768	1	9.1	0.18	34.93
1 primi 2		H						
86	1	10/10/2017	68	769	1	9.3	0	32.92
1 primi 2		H						
86	1	10/11/2017	69	770	1	9.6	0.08	32.57
1 primi 2		H						
86	1	10/12/2017	70	771	1	9.3	0	32.62
1 primi 2		H						
86	1	10/13/2017	71	772	1	9.1	0	28.65
1 primi 2		H						
86	1	10/14/2017	72	773	1	9.2	0.03	33.22
1 primi 2		H						
86	1	10/15/2017	73	774	1	9.1	0.01	30.64
1 primi 2		H						
86	1	10/16/2017	74	775	1	9.1	0.04	33.88
1 primi 2		H						
86	1	10/17/2017	75	776	1	8.9	0	33.59
1 primi 2		H						
86	1	10/18/2017	76	777	1	8.9	0.34	32.7
1 primi 2		H						
86	1	10/19/2017	77	778	1	8.9	0	30.61
1 primi 2		H						
86	1	10/20/2017	78	779	1	8.8	0.1	36.61

1 primi	2	H						
86	1	10/21/2017	79	780	1	9.1	0.03	31.07
1 primi	2	H						
86	1	10/22/2017	80	781	1	9.1	0	34.75
1 primi	2	H						
86	1	10/23/2017	81	782	1	9.2	0	34.07
1 primi	2	H						
86	1	10/24/2017	82	783	1	9.1	0	33.53
1 primi	2	H						
86	1	10/25/2017	83	784	1	9.3	0.1	29.65
1 primi	2	H						
86	1	10/26/2017	84	785	1	9.5	0	33.15
1 primi	2	H						
86	1	10/27/2017	85	786	1	9.2	0	30.5
1 primi	2	H						
86	1	10/28/2017	86	787	1	9.6	0.04	31.04
1 primi	2	H						
86	1	10/29/2017	87	788	1	9.2	0.16	33.78
1 primi	2	H						
86	1	10/30/2017	88	789	1	9.3	0	34.2
1 primi	2	H						
86	1	10/31/2017	89	790	1	9.5	0	32.58
1 primi	2	H						
86	1	11/01/2017	90	791	1	8.9	0	32.23
1 primi	2	H						
86	1	11/02/2017	91	792	1	8.9	0	30.48
1 primi	2	H						
86	1	11/03/2017	92	793	1	9.5	0	36.79
1 primi	2	H						
86	1	11/04/2017	93	794	1	9.8	0.04	34.55
1 primi	2	H						
86	1	11/05/2017	94	795	1	9.1	0.03	31.66
1 primi	2	H						
86	1	11/06/2017	95	796	1	9.3	0	34.7
1 primi	2	H						
86	1	11/07/2017	96	797	1	9.6	0	33.01
1 primi	2	H						
86	1	11/08/2017	97	798	1	9.2	0	32.92
1 primi	2	H						
86	1	11/09/2017	98	799	1	9.1	0.03	31.47
1 primi	2	H						
86	1	11/10/2017	99	800	1	9.1	0	31.29
1 primi	2	H						
86	1	11/11/2017	100	801	1	8.7	0.52	31.21
1 primi	2	H						
86	1	11/12/2017	101	802	1	8.9	0.2	32.25
1 primi	2	H						
86	1	11/13/2017	102	803	1	9.3	0	30.66
1 primi	2	H						
86	1	11/14/2017	103	804	1	8.6	0.07	32.66
1 primi	2	H						
86	1	11/15/2017	104	805	1	8.7	0.06	31.21
1 primi	2	H						
86	1	11/16/2017	105	806	1	9.1	0	32.31

1 primi 2		H						
86	1	11/17/2017	106	807	1	9.1	0	31.75
1 primi 2		H						
86	1	11/18/2017	107	808	1	9.3	0.01	31.69
1 primi 2		H						
86	1	11/19/2017	108	809	1	8.9	0	32.18
1 primi 2		H						
86	1	11/20/2017	109	810	1	9.5	0.14	31.84
1 primi 2		H						
86	1	11/21/2017	110	811	1	9.1	0	31.51
1 primi 2		H						
86	1	11/22/2017	111	812	1	9.5	0.18	33.35
1 primi 2		H						
86	1	11/23/2017	112	813	1	9.9	0.05	32.58
1 primi 2		H						
86	1	11/24/2017	113	814	1	9.6	0	33.27
1 primi 2		H						
86	1	11/25/2017	114	815	1	9.3	0.11	32.79
1 primi 2		H						
86	1	11/26/2017	115	816	1	10.9	0.1	34.45
1 primi 2		H						
86	1	11/27/2017	116	817	1	9.3	0.08	32.8
1 primi 2		H						
86	1	11/28/2017	117	818	1	9.9	0	32.85
1 primi 2		H						
86	1	11/29/2017	118	819	1	9.1	0.12	32.13
1 primi 2		H						
86	1	11/30/2017	119	820	1	9.5	0	31.16
1 primi 2		H						
86	1	12/01/2017	120	821	1	9.1	0	32.24
1 primi 2		H						
86	1	12/02/2017	121	822	1	9.3	0.01	31.17
1 primi 2		H						
86	1	12/03/2017	122	823	1	10	0	34.19
1 primi 2		H						
86	1	12/04/2017	123	824	1	8.9	0.02	31.36
1 primi 2		H						
86	1	12/05/2017	124	825	1	9.5	0.09	32.27
1 primi 2		H						
86	1	12/06/2017	125	826	1	9.1	0	34.13
1 primi 2		H						
86	1	12/07/2017	126	827	1	9.6	0.19	31.86
1 primi 2		H						
86	1	12/08/2017	127	828	1	9.5	0.08	30.91
1 primi 2		H						
86	1	12/09/2017	128	829	1	8.8	0	29.97
1 primi 2		H						
86	1	12/10/2017	129	830	1	8.7	0.07	29.3
1 primi 2		H						
86	1	12/11/2017	130	831	1	9.8	0.02	29.61
1 primi 2		H						
86	1	12/12/2017	131	832	1	9.3	0	30.16
1 primi 2		H						
86	1	12/13/2017	132	833	1	10.3	0.07	32.94

1 primi 2		H						
86	1	10/19/2018	7	1143	2	8.7	0.17	23.65
2 multi 1		H						
86	1	10/20/2018	8	1144	2	8.7	0.18	24.85
2 multi 1		H						
86	1	10/21/2018	9	1145	2	8.6	0.03	26.18
2 multi 1		H						
86	1	10/22/2018	10	1146	2	8.8	0.03	28.03
2 multi 1		H						
86	1	10/23/2018	11	1147	2	8.6	0	31.26
2 multi 1		H						
86	1	10/24/2018	12	1148	2	8.6	0.04	29.69
2 multi 1		H						
86	1	10/25/2018	13	1149	2	8.8	0.13	30.69
2 multi 1		H						
86	1	10/26/2018	14	1150	2	8.4	0	31.6
2 multi 1		H						
86	1	10/27/2018	15	1151	2	8.8	0.03	30.97
2 multi 1		H						
86	1	10/28/2018	16	1152	2	8.7	0	32.64
2 multi 1		H						
86	1	10/29/2018	17	1153	2	8.5	0.21	31.08
2 multi 1		H						
86	1	10/30/2018	18	1154	2	9.1	0.14	29.6
2 multi 1		H						
86	1	10/31/2018	19	1155	2	8.9	0.16	33.89
2 multi 1		H						
86	1	11/01/2018	20	1156	2	8.8	0.05	34.49
2 multi 1		H						
86	1	11/02/2018	21	1157	2	8.7	0.01	35.22
2 multi 1		H						
86	1	11/03/2018	22	1158	2	8.8	0.02	34.98
2 multi 2		H						
86	1	11/04/2018	23	1159	2	8.8	0.22	34.94
2 multi 2		H						
86	1	11/05/2018	24	1160	2	9.1	0.03	35.57
2 multi 2		H						
86	1	11/06/2018	25	1161	2	8.8	0.05	35.01
2 multi 2		H						
86	1	11/07/2018	26	1162	2	8.8	0.34	34.89
2 multi 2		H						
86	1	11/08/2018	27	1163	2	9.1	0	37.1
2 multi 2		H						
86	1	11/09/2018	28	1164	2	8.9	0	35.36
2 multi 2		H						
86	1	11/10/2018	29	1165	2	9.1	0.13	35.03
2 multi 2		H						
86	1	11/11/2018	30	1166	2	8.7	0	37.75
2 multi 2		H						
86	1	11/12/2018	31	1167	2	8.7	0	37.9
2 multi 2		H						
86	1	11/13/2018	32	1168	2	9.5	0	37.83
2 multi 2		H						
86	1	11/14/2018	33	1169	2	8.8	0.08	38.32

2 multi	2	H						
86	1	11/15/2018	34	1170	2	8.8	0.11	34.53
2 multi	2	H						
86	1	11/16/2018	35	1171	2	8.7	0.02	37.44
2 multi	2	H						
86	1	11/17/2018	36	1172	2	9.1	0.12	36.84
2 multi	2	H						
86	1	11/18/2018	37	1173	2	9.1	0.1	36.61
2 multi	2	H						
86	1	11/19/2018	38	1174	2	9.1	0	38.56
2 multi	2	H						
86	1	11/20/2018	39	1175	2	8.9	0.1	38.13
2 multi	2	H						
86	1	11/21/2018	40	1176	2	9.1	0.06	39.05
2 multi	2	H						
86	1	11/22/2018	41	1177	2	8.9	0.21	40.38
2 multi	2	H						
86	1	11/23/2018	42	1178	2	9.2	0	38.82
2 multi	2	H						
86	1	11/24/2018	43	1179	2	8.9	0	40.36
2 multi	2	H						
86	1	11/25/2018	44	1180	2	9.1	0	41.55
2 multi	2	H						
86	1	11/26/2018	45	1181	2	9.5	0.12	40.52
2 multi	2	H						
86	1	11/27/2018	46	1182	2	9.3	0	34.57
2 multi	2	H						
86	1	11/28/2018	47	1183	2	9.3	0.1	39.13
2 multi	2	H						
86	1	11/29/2018	48	1184	2	9.5	0	36.68
2 multi	2	H						
86	1	11/30/2018	49	1185	2	8.7	0.2	35.36
2 multi	2	H						
86	1	12/01/2018	50	1186	2	8.7	0.19	35.15
2 multi	2	H						
86	1	12/02/2018	51	1187	2	9.1	0	35.7
2 multi	2	H						
86	1	12/03/2018	52	1188	2	8.7	0	36.68
2 multi	2	H						
86	1	12/04/2018	53	1189	2	9.1	0	36.75
2 multi	2	H						
86	1	12/05/2018	54	1190	2	8.7	0.08	35.9
2 multi	2	H						
86	1	12/06/2018	55	1191	2	9.2	0	38.23
2 multi	2	H						
86	1	12/07/2018	56	1192	2	9.2	0	38.56
2 multi	2	H						
86	1	12/08/2018	57	1193	2	8.8	0.49	42.15
2 multi	2	H						
86	1	12/09/2018	58	1194	2	9.5	0	36.23
2 multi	2	H						
86	1	12/10/2018	59	1195	2	9.3	0.23	38.06
2 multi	2	H						
86	1	12/11/2018	60	1196	2	8.9	0.01	35.91

2 multi	2	H							
86	1	12/12/2018	61	1197	2	9.2	0.09	41	
2 multi	2	H							
86	1	12/13/2018	62	1198	2	8.8	0	34.02	
2 multi	2	H							
86	1	12/14/2018	63	1199	2	8.7	0.16	37.63	
2 multi	2	H							
86	1	12/15/2018	64	1200	2	8.9	0	40.08	
2 multi	2	H							
86	1	12/16/2018	65	1201	2	9.3	0	37.96	
2 multi	2	H							
86	1	12/17/2018	66	1202	2	9.5	0	38.08	
2 multi	2	H							
86	1	12/18/2018	67	1203	2	9.5	0.1	36.61	
2 multi	2	H							
86	1	12/19/2018	68	1204	2	9.3	0	42.51	
2 multi	2	H							
86	1	12/20/2018	69	1205	2	9.6	0.15	37.25	
2 multi	2	H							
86	1	12/21/2018	70	1206	2	9.1	0.2	40.24	
2 multi	2	H							
86	1	12/22/2018	71	1207	2	9.8	0	38.83	
2 multi	2	H							
86	1	12/23/2018	72	1208	2	9.1	0.01	40.7	
2 multi	2	H							
86	1	12/24/2018	73	1209	2	9.5	0	40.66	
2 multi	2	H							
86	1	12/25/2018	74	1210	2	9.5	0	39.86	
2 multi	2	H							
86	1	12/26/2018	75	1211	2	9.2	0	40.96	
2 multi	2	H							
86	1	12/27/2018	76	1212	2	9.6	0	38.36	
2 multi	2	H							
86	1	12/28/2018	77	1213	2	9.2	0.33	39.74	
2 multi	2	H							
86	1	12/29/2018	78	1214	2	8.7	0.04	35.57	
2 multi	2	H							
86	1	12/30/2018	79	1215	2	8.8	0.1	36.86	
2 multi	2	H							
86	1	12/31/2018	80	1216	2	9.1	0.17	41.37	
2 multi	2	H							
86	1	01/01/2019	81	1217	2	8.9	0	39.68	
2 multi	2	H							
86	1	01/02/2019	82	1218	2	9.3	0	32.04	
2 multi	2	H							
86	1	01/03/2019	83	1219	2	8.4	0.27	45.99	
2 multi	2	H							
86	1	01/04/2019	84	1220	2	9.3	0	39.01	
2 multi	2	H							
86	1	01/05/2019	85	1221	2	8.6	0.01	34.59	
2 multi	2	H							
86	1	01/06/2019	86	1222	2	9.1	0	38.49	
2 multi	2	H							
86	1	01/07/2019	87	1223	2	8.9	0	35.62	

2 multi	2	H						
86	1	01/08/2019	88	1224	2	9.1	0.01	37.28
2 multi	2	H						
86	1	01/09/2019	89	1225	2	8.5	0.1	38.99
2 multi	2	H						
86	1	01/10/2019	90	1226	2	9.2	0.07	40
2 multi	2	H						
86	1	01/11/2019	91	1227	2	9.2	0.11	37.86
2 multi	2	H						
86	1	01/12/2019	92	1228	2	9.2	0	36.07
2 multi	2	H						
86	1	01/13/2019	93	1229	2	8.9	0.1	34.96
2 multi	2	H						
86	1	01/14/2019	94	1230	2	8.6	0.22	39.56
2 multi	2	H						
86	1	01/15/2019	95	1231	2	9.5	0	37.52
2 multi	2	H						
86	1	01/16/2019	96	1232	2	9.6	0.1	37.73
2 multi	2	H						
86	1	03/28/2019	167	1303	2	8.9	0	25.25
2 multi	2	H						
86	1	03/29/2019	168	1304	2	9.3	0.11	25.19
2 multi	2	H						
86	1	03/30/2019	169	1305	2	9.3	0.1	28.1
2 multi	2	H						
86	1	03/31/2019	170	1306	2	9.3	0.21	25.93
2 multi	2	H						
86	1	04/01/2019	171	1307	2	9.1	0.06	29.08
2 multi	2	H						
86	1	04/02/2019	172	1308	2	8.9	0.19	27.36
2 multi	2	H						
86	1	04/03/2019	173	1309	2	9.8	0	31.08
2 multi	2	H						
86	1	04/04/2019	174	1310	2	9.6	0.25	29.27
2 multi	2	H						
86	1	04/05/2019	175	1311	2	9.6	0.14	29.39
2 multi	2	H						
86	1	04/06/2019	176	1312	2	8.7	0.16	29.05
2 multi	2	H						
86	1	04/07/2019	177	1313	2	9.1	0	31
2 multi	2	H						
86	1	04/08/2019	178	1314	2	9.2	0	30.09
2 multi	2	H						
86	1	04/09/2019	179	1315	2	9.6	0.24	32.6
2 multi	2	H						
86	1	04/10/2019	180	1316	2	8.9	0.31	28.93
2 multi	2	H						
86	1	04/11/2019	181	1317	2	9.5	0.25	31.88
2 multi	2	H						
86	1	04/12/2019	182	1318	2	9.9	0.23	25.29
2 multi	2	H						
86	1	04/13/2019	183	1319	2	8.8	0.25	36.2
2 multi	2	H						
86	1	04/14/2019	184	1320	2	9.5	0	26.16

2 multi	2	H						
86	1	04/15/2019	185	1321	2	9.5	0	27.93
2 multi	2	H						
86	1	04/16/2019	186	1322	2	9.2	0.01	28.03
2 multi	2	H						
86	1	04/17/2019	187	1323	2	9.3	0	29.82
2 multi	2	H						
86	1	05/29/2020	5	1731	3	9.2	0.26	34.8
2 multi	1	H						
86	1	05/30/2020	6	1732	3	9.6	0.06	33.29
2 multi	1	H						
86	1	05/31/2020	7	1733	3	9.3	0.36	32.75
2 multi	1	H						
86	1	06/01/2020	8	1734	3	9.1	0.12	35.85
2 multi	1	H						
86	1	06/02/2020	9	1735	3	9.2	0	37.04
2 multi	1	H						
86	1	06/03/2020	10	1736	3	9.1	0.25	37.09
2 multi	1	H						
86	1	06/04/2020	11	1737	3	9.2	0.03	39.32
2 multi	1	H						
86	1	06/05/2020	12	1738	3	8.6	0.24	35.41
2 multi	1	H						
86	1	06/06/2020	13	1739	3	8.4	0.22	37.81
2 multi	1	H						
86	1	06/07/2020	14	1740	3	8.6	0.28	42.24
2 multi	1	H						
86	1	06/08/2020	15	1741	3	8.7	0.03	42.92
2 multi	1	H						
86	1	06/09/2020	16	1742	3	8.7	0.26	44.06
2 multi	1	H						
86	1	06/10/2020	17	1743	3	8.4	0.18	39.94
2 multi	1	H						
86	1	06/11/2020	18	1744	3	8.8	0.18	43.29
2 multi	1	H						
86	1	06/12/2020	19	1745	3	8.6	0.09	42.97
2 multi	1	H						
86	1	06/13/2020	20	1746	3	8.8	0.03	45.36
2 multi	1	H						
86	1	06/14/2020	21	1747	3	8.7	0.24	44.1
2 multi	1	H						
86	1	06/15/2020	22	1748	3	8.8	0.06	42.29
2 multi	2	H						
86	1	06/16/2020	23	1749	3	8.7	0	46.76
2 multi	2	H						
86	1	06/17/2020	24	1750	3	8.9	0.16	41.08
2 multi	2	H						
86	1	06/18/2020	25	1751	3	8.8	0	47.64
2 multi	2	H						
86	1	06/19/2020	26	1752	3	8.8	0	44.56
2 multi	2	H						
86	1	06/20/2020	27	1753	3	8.5	0	40.51
2 multi	2	H						
86	1	06/21/2020	28	1754	3	8.8	0	47.68

2 multi	2	H						
86	1	06/22/2020	29	1755	3	8.8	0	45.42
2 multi	2	H						
86	1	06/23/2020	30	1756	3	9.1	0	44
2 multi	2	H						
86	1	06/24/2020	31	1757	3	8.9	0	49.15
2 multi	2	H						
86	1	06/25/2020	32	1758	3	8.8	0	49.65
2 multi	2	H						
86	1	06/26/2020	33	1759	3	8.8	0	45.75
2 multi	2	H						
86	1	06/27/2020	34	1760	3	8.7	0	49.12
2 multi	2	H						
86	1	06/28/2020	35	1761	3	8.8	0	44.6
2 multi	2	H						
86	1	06/29/2020	36	1762	3	8.9	0	52.15
2 multi	2	H						
86	1	10/14/2020	143	1869	3	9.9	0.31	36.73
2 multi	2	H						
86	1	10/15/2020	144	1870	3	9.2	0.2	40.56
2 multi	2	H						
86	1	10/16/2020	145	1871	3	10	0.08	41.14
2 multi	2	H						
86	1	10/17/2020	146	1872	3	9.8	0.09	41.18
2 multi	2	H						
86	1	10/18/2020	147	1873	3	10.2	0.12	41.48
2 multi	2	H						
86	1	10/19/2020	148	1874	3	10.2	0.15	44.12
2 multi	2	H						
86	1	10/20/2020	149	1875	3	10.3	0.04	45.49
2 multi	2	H						
86	1	10/21/2020	150	1876	3	10.2	0.09	44.84
2 multi	2	H						
86	1	10/22/2020	151	1877	3	9.8	0.19	40.32
2 multi	2	H						
86	1	10/23/2020	152	1878	3	9.3	0.07	47.04
2 multi	2	H						
86	1	10/24/2020	153	1879	3	9.5	0.24	40.73
2 multi	2	H						
86	1	10/25/2020	154	1880	3	9.9	0.08	48.36
2 multi	2	H						
86	1	10/26/2020	155	1881	3	10.2	0.07	41.97
2 multi	2	H						
86	1	10/27/2020	156	1882	3	10.4	0.06	38.03
2 multi	2	H						
86	1	10/28/2020	157	1883	3	10.7	0	35.45
2 multi	2	H						
86	1	10/29/2020	158	1884	3	10.6	0.01	41.23
2 multi	2	H						
86	1	10/30/2020	159	1885	3	10.6	0.08	40.12
2 multi	2	H						
86	1	10/31/2020	160	1886	3	10.4	0.13	41.21
2 multi	2	H						
86	1	11/01/2020	161	1887	3	10.4	0.09	39.06

2 multi	2	H						
86	1	11/02/2020	162	1888	3	10.3	0.01	48.77
2 multi	2	H						
86	1	11/03/2020	163	1889	3	10.7	0.13	43.07
2 multi	2	H						
86	1	11/04/2020	164	1890	3	10.3	0.02	42.46
2 multi	2	H						
86	1	11/05/2020	165	1891	3	10.2	0.07	40.63
2 multi	2	H						
86	1	11/06/2020	166	1892	3	10.6	0.03	42.79
2 multi	2	H						
86	1	11/07/2020	167	1893	3	10	0.3	42.73
2 multi	2	H						
86	1	11/08/2020	168	1894	3	10.3	0.01	41.01
2 multi	2	H						
86	1	11/09/2020	169	1895	3	10.6	0.02	38.99
2 multi	2	H						
86	1	11/10/2020	170	1896	3	10.4	0	36.15
2 multi	2	H						
86	1	11/11/2020	171	1897	3	10.7	0.26	40.61
2 multi	2	H						
86	1	11/12/2020	172	1898	3	10.6	0.13	39.02
2 multi	2	H						
86	1	11/13/2020	173	1899	3	10.7	0.11	37.18
2 multi	2	H						
87	1	10/19/2018	7	755	1	9.8	0	30.28
1 primi	1	H						
87	1	10/20/2018	8	756	1	10.3	0	29.12
1 primi	1	H						
87	1	10/21/2018	9	757	1	10	0	29.75
1 primi	1	H						
87	1	10/22/2018	10	758	1	8.9	0.15	28.91
1 primi	1	H						
87	1	10/23/2018	11	759	1	9.6	0.05	32.03
1 primi	1	H						
87	1	10/24/2018	12	760	1	9.8	0.13	30.75
1 primi	1	H						
87	1	10/25/2018	13	761	1	9.6	0.02	32.08
1 primi	1	H						
87	1	10/26/2018	14	762	1	9.2	0.02	30.86
1 primi	1	H						
87	1	10/27/2018	15	763	1	9.5	0.02	30.74
1 primi	1	H						
87	1	10/28/2018	16	764	1	9.8	0.02	31
1 primi	1	H						
87	1	10/29/2018	17	765	1	9.3	0.21	33.74
1 primi	1	H						
87	1	10/30/2018	18	766	1	9.8	0	34.59
1 primi	1	H						
87	1	10/31/2018	19	767	1	9.3	0.05	34.33
1 primi	1	H						
87	1	11/01/2018	20	768	1	9.6	0	36.31
1 primi	1	H						
87	1	11/02/2018	21	769	1	9.6	0	36.18

1 primi	1	H						
87	1	11/03/2018	22	770	1	9.9	0	36.28
1 primi	2	H						
87	1	11/04/2018	23	771	1	9.5	0.01	34.9
1 primi	2	H						
87	1	11/05/2018	24	772	1	9.8	0.01	34.91
1 primi	2	H						
87	1	11/06/2018	25	773	1	9.5	0	35.95
1 primi	2	H						
87	1	11/07/2018	26	774	1	8.9	0.18	35.33
1 primi	2	H						
87	1	11/08/2018	27	775	1	9.3	0.26	36.45
1 primi	2	H						
87	1	11/09/2018	28	776	1	8.9	0.34	35.06
1 primi	2	H						
87	1	11/10/2018	29	777	1	9.1	0	36.26
1 primi	2	H						
87	1	11/11/2018	30	778	1	0	0.34	36.26
1 primi	2	H						
87	1	11/12/2018	31	779	1	9.2	0.02	37.66
1 primi	2	H						
87	1	11/13/2018	32	780	1	9.6	0.06	34.89
1 primi	2	H						
87	1	11/14/2018	33	781	1	9.9	0.28	37.59
1 primi	2	H						
87	1	11/15/2018	34	782	1	9.9	0.07	37.18
1 primi	2	H						
87	1	11/16/2018	35	783	1	9.2	0	36.7
1 primi	2	H						
87	1	11/17/2018	36	784	1	9.6	0	36.77
1 primi	2	H						
87	1	11/18/2018	37	785	1	10.2	0	39.49
1 primi	2	H						
87	1	11/19/2018	38	786	1	9.3	0	37.72
1 primi	2	H						
87	1	11/20/2018	39	787	1	9.3	0.13	37.38
1 primi	2	H						
87	1	11/21/2018	40	788	1	9.8	0.04	39.59
1 primi	2	H						
87	1	11/22/2018	41	789	1	9.1	0.28	38.68
1 primi	2	H						
87	1	11/23/2018	42	790	1	10.3	0	38.15
1 primi	2	H						
87	1	11/24/2018	43	791	1	11.3	0.01	24.9
1 primi	2	H						
87	1	11/25/2018	44	792	1	9.8	0	32.68
1 primi	2	H						
87	1	11/26/2018	45	793	1	10.3	0.03	34.8
1 primi	2	H						
87	1	11/27/2018	46	794	1	9.5	0.23	36.45
1 primi	2	H						
87	1	11/28/2018	47	795	1	10.9	0.24	26.56
1 primi	2	H						
87	1	11/29/2018	48	796	1	11.1	0.25	12.9

1 primi	2	H						
87	1	11/30/2018	49	797	1	12.3	0.14	26.92
1 primi	2	H						
87	1	12/01/2018	50	798	1	11.1	0.03	30.45
1 primi	2	H						
87	1	12/02/2018	51	799	1	10.9	0.05	29.78
1 primi	2	H						
87	1	12/03/2018	52	800	1	11.1	0	30.56
1 primi	2	H						
87	1	12/04/2018	53	801	1	10.4	0.07	29.23
1 primi	2	H						
87	1	12/05/2018	54	802	1	9.3	0.02	29.46
1 primi	2	H						
87	1	10/16/2019	3	1117	2	10	0.41	28.59
2 multi	1	H						
87	1	10/17/2019	4	1118	2	9.9	0.18	31.11
2 multi	1	H						
87	1	10/18/2019	5	1119	2	9.8	0.08	31.65
2 multi	1	H						
87	1	10/19/2019	6	1120	2	9.6	0.36	33.08
2 multi	1	H						
87	1	10/20/2019	7	1121	2	9.5	0.12	37.42
2 multi	1	H						
87	1	10/21/2019	8	1122	2	9.8	0.07	35.05
2 multi	1	H						
87	1	10/22/2019	9	1123	2	9.6	0.27	37.07
2 multi	1	H						
87	1	10/23/2019	10	1124	2	9.9	0.06	37.19
2 multi	1	H						
87	1	10/24/2019	11	1125	2	9.8	0.17	39.44
2 multi	1	H						
87	1	10/25/2019	12	1126	2	10.3	0.26	38.37
2 multi	1	H						
87	1	10/26/2019	13	1127	2	11.3	0.11	30.75
2 multi	1	H						
87	1	10/27/2019	14	1128	2	10.6	0.35	35.28
2 multi	1	H						
87	1	10/28/2019	15	1129	2	9.9	0.07	36.11
2 multi	1	H						
87	1	10/29/2019	16	1130	2	9.8	0.2	35.09
2 multi	1	H						
87	1	10/30/2019	17	1131	2	10	0.31	31.43
2 multi	1	H						
87	1	10/31/2019	18	1132	2	9.9	0.21	35.93
2 multi	1	H						
87	1	11/01/2019	19	1133	2	9.3	0.13	35.29
2 multi	1	H						
87	1	11/02/2019	20	1134	2	10	0.2	35.58
2 multi	1	H						
87	1	11/03/2019	21	1135	2	10.4	0.06	36.63
2 multi	1	H						
87	1	11/04/2019	22	1136	2	10	0.16	32.92
2 multi	2	H						
87	1	11/05/2019	23	1137	2	10.4	0.13	37.1

2 multi	2	H						
87	1	11/06/2019	24	1138	2	10	0.25	35.55
2 multi	2	H						
87	1	11/07/2019	25	1139	2	10	0.16	34.18
2 multi	2	H						
87	1	11/08/2019	26	1140	2	10	0.05	35.24
2 multi	2	H						
87	1	11/09/2019	27	1141	2	9.6	0.13	35.38
2 multi	2	H						
87	1	11/10/2019	28	1142	2	9.2	0	36.98
2 multi	2	H						
87	1	11/11/2019	29	1143	2	9.5	0	36.44
2 multi	2	H						
87	1	11/12/2019	30	1144	2	9.6	0	37.07
2 multi	2	H						
87	1	11/13/2019	31	1145	2	9.5	0.06	37.91
2 multi	2	H						
87	1	11/14/2019	32	1146	2	9.6	0.11	37.31
2 multi	2	H						
87	1	11/15/2019	33	1147	2	9.6	0.1	37.51
2 multi	2	H						
87	1	11/16/2019	34	1148	2	9.8	0.07	40.28
2 multi	2	H						
87	1	11/17/2019	35	1149	2	9.5	0.16	40.85
2 multi	2	H						
87	1	11/18/2019	36	1150	2	9.6	0.05	39.27
2 multi	2	H						
87	1	11/19/2019	37	1151	2	9.3	0	36.57
2 multi	2	H						
87	1	11/20/2019	38	1152	2	9.5	0.04	38.81
2 multi	2	H						
87	1	11/21/2019	39	1153	2	9.6	0.07	37.88
2 multi	2	H						
87	1	11/22/2019	40	1154	2	9.8	0.07	41.64
2 multi	2	H						
87	1	11/23/2019	41	1155	2	10.2	0.09	38.44
2 multi	2	H						
87	1	11/24/2019	42	1156	2	10	0.15	38.75
2 multi	2	H						
87	1	11/25/2019	43	1157	2	10	0.3	39.45
2 multi	2	H						
87	1	11/26/2019	44	1158	2	10	0.06	38.51
2 multi	2	H						
87	1	11/27/2019	45	1159	2	9.9	0.06	38.93
2 multi	2	H						
87	1	11/28/2019	46	1160	2	9.9	0.13	39.97
2 multi	2	H						
87	1	11/29/2019	47	1161	2	10.2	0.07	38.08
2 multi	2	H						
87	1	11/30/2019	48	1162	2	9.6	0.03	38.5
2 multi	2	H						
87	1	12/01/2019	49	1163	2	9.5	0.09	30.88
2 multi	2	H						
87	1	12/02/2019	50	1164	2	9.8	0.04	36.35

2 multi 2		H						
87	1	12/03/2019	51	1165	2	9.8	0.27	37.8
2 multi 2		H						
87	1	12/04/2019	52	1166	2	10	0.06	36.41
2 multi 2		H						
87	1	12/05/2019	53	1167	2	10	0.22	36.92
2 multi 2		H						
87	1	12/06/2019	54	1168	2	9.9	0.01	37.45
2 multi 2		H						
87	1	12/07/2019	55	1169	2	9.9	0.25	37.13
2 multi 2		H						
87	1	12/08/2019	56	1170	2	10	0	34.82
2 multi 2		H						
87	1	12/09/2019	57	1171	2	10.9	0.25	35.28
2 multi 2		H						
87	1	12/10/2019	58	1172	2	10.3	0.01	38.01
2 multi 2		H						
87	1	12/11/2019	59	1173	2	10	0.28	36.28
2 multi 2		H						
87	1	12/12/2019	60	1174	2	9.9	0.21	36.04
2 multi 2		H						
87	1	12/13/2019	61	1175	2	10	0.01	37.17
2 multi 2		H						
87	1	12/14/2019	62	1176	2	9.9	0.09	32.77
2 multi 2		H						
87	1	12/15/2019	63	1177	2	9.6	0.02	34.97
2 multi 2		H						
87	1	12/16/2019	64	1178	2	10	0.26	38.11
2 multi 2		H						
87	1	12/17/2019	65	1179	2	9.6	0.38	37.96
2 multi 2		H						
87	1	12/18/2019	66	1180	2	9.9	0.26	37.71
2 multi 2		H						
87	1	12/19/2019	67	1181	2	9.5	0	37.07
2 multi 2		H						
87	1	12/20/2019	68	1182	2	10.3	0	37.56
2 multi 2		H						
87	1	12/21/2019	69	1183	2	10.4	0.08	38.67
2 multi 2		H						
87	1	12/22/2019	70	1184	2	9.9	0.03	39.58
2 multi 2		H						
87	1	12/23/2019	71	1185	2	10	0.02	34.71
2 multi 2		H						
87	1	12/24/2019	72	1186	2	9.8	0.12	33.52
2 multi 2		H						
87	1	12/25/2019	73	1187	2	9.6	0.09	36.39
2 multi 2		H						
87	1	12/26/2019	74	1188	2	10.2	0.02	38.66
2 multi 2		H						
87	1	12/27/2019	75	1189	2	9.9	0.02	36.72
2 multi 2		H						
87	1	12/28/2019	76	1190	2	10.2	0.03	36.76
2 multi 2		H						
87	1	12/29/2019	77	1191	2	10	0.02	39.75

2 multi	2	H						
87	1	12/30/2019	78	1192	2	10.2	0.02	39.78
2 multi	2	H						
87	1	12/31/2019	79	1193	2	10.4	0	39.91
2 multi	2	H						
87	1	01/01/2020	80	1194	2	10	0.02	39.59
2 multi	2	H						
87	1	01/02/2020	81	1195	2	10.2	0.08	35.97
2 multi	2	H						
87	1	01/03/2020	82	1196	2	10.3	0.02	35.9
2 multi	2	H						
87	1	01/04/2020	83	1197	2	10.3	0.04	32.05
2 multi	2	H						
87	1	01/05/2020	84	1198	2	9.9	0.03	37.92
2 multi	2	H						
89	1	12/12/2018	5	745	1	9.6	0.07	29.45
1 primi	1	H						
89	1	12/13/2018	6	746	1	9.1	0	31.03
1 primi	1	H						
89	1	12/14/2018	7	747	1	8.7	0	30.14
1 primi	1	H						
89	1	12/15/2018	8	748	1	8.9	0	29.39
1 primi	1	H						
89	1	12/16/2018	9	749	1	8.9	0	31.11
1 primi	1	H						
89	1	12/17/2018	10	750	1	8.9	0	33.03
1 primi	1	H						
89	1	12/18/2018	11	751	1	8.9	0.01	35.05
1 primi	1	H						
89	1	12/19/2018	12	752	1	8.8	0.02	34.43
1 primi	1	H						
89	1	12/20/2018	13	753	1	8.8	0	33.21
1 primi	1	H						
89	1	12/21/2018	14	754	1	8.7	0	34.2
1 primi	1	H						
89	1	12/22/2018	15	755	1	8.7	0	34.9
1 primi	1	H						
89	1	12/23/2018	16	756	1	8.8	0	36.36
1 primi	1	H						
89	1	12/24/2018	17	757	1	8.8	0	34.69
1 primi	1	H						
89	1	12/25/2018	18	758	1	8.6	0	38.14
1 primi	1	H						
89	1	12/26/2018	19	759	1	8.7	0.15	35.26
1 primi	1	H						
89	1	12/27/2018	20	760	1	8.9	0	36.76
1 primi	1	H						
89	1	12/28/2018	21	761	1	8.9	0	36.35
1 primi	1	H						
89	1	12/29/2018	22	762	1	8.8	0	34.55
1 primi	2	H						
89	1	12/30/2018	23	763	1	8.7	0	35.25
1 primi	2	H						
89	1	12/31/2018	24	764	1	8.9	0	34.4

1 primi	2	H						
89	1	01/01/2019	25	765	1	8.9	0	36.66
1 primi	2	H						
89	1	01/02/2019	26	766	1	8.9	0	35.54
1 primi	2	H						
89	1	01/03/2019	27	767	1	8.8	0	36.87
1 primi	2	H						
89	1	01/04/2019	28	768	1	8.9	0	34.69
1 primi	2	H						
89	1	01/05/2019	29	769	1	8.5	0	35.45
1 primi	2	H						
89	1	01/06/2019	30	770	1	8.6	0	37.55
1 primi	2	H						
89	1	01/07/2019	31	771	1	9.1	0	38.52
1 primi	2	H						
89	1	01/08/2019	32	772	1	8.8	0	38.1
1 primi	2	H						
89	1	01/09/2019	33	773	1	8.8	0	36.9
1 primi	2	H						
89	1	01/10/2019	34	774	1	8.8	0	38
1 primi	2	H						
89	1	01/11/2019	35	775	1	9.1	0	37.92
1 primi	2	H						
89	1	01/12/2019	36	776	1	9.1	0	38.17
1 primi	2	H						
89	1	01/13/2019	37	777	1	8.9	0	34.91
1 primi	2	H						
89	1	01/14/2019	38	778	1	9.1	0	36.91
1 primi	2	H						
89	1	01/15/2019	39	779	1	9.1	0	38.72
1 primi	2	H						
89	1	01/16/2019	40	780	1	8.7	0	33.82
1 primi	2	H						
89	1	01/17/2019	41	781	1	9.3	0	35.76
1 primi	2	H						
89	1	01/18/2019	42	782	1	9.2	0	38.01
1 primi	2	H						
89	1	01/19/2019	43	783	1	9.3	0	38.75
1 primi	2	H						
89	1	01/20/2019	44	784	1	9.2	0	38.28
1 primi	2	H						
89	1	01/21/2019	45	785	1	9.1	0	39.63
1 primi	2	H						
89	1	01/22/2019	46	786	1	8.9	0.08	38.03
1 primi	2	H						
89	1	01/23/2019	47	787	1	8.9	0	38.22
1 primi	2	H						
89	1	01/24/2019	48	788	1	9.3	0	39.77
1 primi	2	H						
89	1	01/25/2019	49	789	1	9.3	0.22	39.73
1 primi	2	H						
89	1	01/26/2019	50	790	1	9.1	0	41.25
1 primi	2	H						
89	1	01/27/2019	51	791	1	9.2	0	40.09

1 primi	2	H						
89	1	01/28/2019	52	792	1	9.3	0	40.28
1 primi	2	H						
89	1	01/29/2019	53	793	1	9.2	0	36.49
1 primi	2	H						
89	1	01/30/2019	54	794	1	9.1	0	41.33
1 primi	2	H						
89	1	01/31/2019	55	795	1	9.3	0	38.92
1 primi	2	H						
89	1	02/01/2019	56	796	1	9.1	0	40.29
1 primi	2	H						
89	1	02/02/2019	57	797	1	9.2	0	39.47
1 primi	2	H						
89	1	02/03/2019	58	798	1	9.1	0	39
1 primi	2	H						
89	1	02/04/2019	59	799	1	9.2	0.4	39.99
1 primi	2	H						
89	1	02/05/2019	60	800	1	8.8	0.09	37.15
1 primi	2	H						
89	1	02/06/2019	61	801	1	9.2	0	38.56
1 primi	2	H						
89	1	02/07/2019	62	802	1	9.1	0	38.53
1 primi	2	H						
89	1	02/08/2019	63	803	1	9.3	0	40.83
1 primi	2	H						
89	1	02/09/2019	64	804	1	9.5	0	40.76
1 primi	2	H						
89	1	02/10/2019	65	805	1	9.5	0.25	39.84
1 primi	2	H						
89	1	02/11/2019	66	806	1	9.3	0	37.96
1 primi	2	H						
89	1	02/12/2019	67	807	1	9.3	0	40.75
1 primi	2	H						
89	1	02/13/2019	68	808	1	9.3	0	42.19
1 primi	2	H						
89	1	02/14/2019	69	809	1	9.6	0	40.78
1 primi	2	H						
89	1	02/15/2019	70	810	1	9.6	0.01	41.09
1 primi	2	H						
89	1	02/16/2019	71	811	1	9.3	0.21	39.73
1 primi	2	H						
89	1	02/17/2019	72	812	1	9.3	0	39.34
1 primi	2	H						
89	1	02/18/2019	73	813	1	9.6	0	40.68
1 primi	2	H						
89	1	02/19/2019	74	814	1	9.6	0	38.79
1 primi	2	H						
89	1	02/20/2019	75	815	1	9.6	0	40.17
1 primi	2	H						
89	1	02/21/2019	76	816	1	9.6	0	39.89
1 primi	2	H						
89	1	02/22/2019	77	817	1	9.5	0	40.6
1 primi	2	H						
89	1	02/23/2019	78	818	1	9.3	0.25	39.33

1 primi	2	H						
89	1	02/24/2019	79	819	1	9.8	0.13	39.8
1 primi	2	H						
89	1	02/25/2019	80	820	1	9.8	0	39.36
1 primi	2	H						
89	1	02/26/2019	81	821	1	9.9	0.11	38.96
1 primi	2	H						
89	1	02/27/2019	82	822	1	9.6	0.14	38.95
1 primi	2	H						
89	1	02/28/2019	83	823	1	9.8	0	42.47
1 primi	2	H						
89	1	03/01/2019	84	824	1	9.8	0	41.52
1 primi	2	H						
89	1	03/02/2019	85	825	1	9.6	0	41.1
1 primi	2	H						
89	1	03/03/2019	86	826	1	9.6	0.21	38.71
1 primi	2	H						
89	1	03/04/2019	87	827	1	9.8	0	40.83
1 primi	2	H						
89	1	03/05/2019	88	828	1	9.8	0.07	41.79
1 primi	2	H						
89	1	03/06/2019	89	829	1	9.8	0	43.47
1 primi	2	H						
89	1	03/07/2019	90	830	1	9.5	0	39.79
1 primi	2	H						
89	1	03/08/2019	91	831	1	9.8	0	40.42
1 primi	2	H						
89	1	03/09/2019	92	832	1	9.9	0	39.73
1 primi	2	H						
89	1	03/10/2019	93	833	1	9.9	0	39.2
1 primi	2	H						
89	1	03/11/2019	94	834	1	9.8	0	38.91
1 primi	2	H						
89	1	03/12/2019	95	835	1	9.9	0	39.05
1 primi	2	H						
89	1	03/13/2019	96	836	1	9.8	0.23	40.6
1 primi	2	H						
89	1	03/14/2019	97	837	1	9.8	0.09	39.2
1 primi	2	H						
89	1	03/15/2019	98	838	1	9.8	0	40.35
1 primi	2	H						
89	1	03/16/2019	99	839	1	9.8	0	41.64
1 primi	2	H						
89	1	03/17/2019	100	840	1	9.8	0	39.43
1 primi	2	H						
89	1	03/18/2019	101	841	1	9.3	0	39.76
1 primi	2	H						
89	1	03/19/2019	102	842	1	9.9	0	39.89
1 primi	2	H						
89	1	03/20/2019	103	843	1	9.9	0	43.93
1 primi	2	H						
89	1	03/21/2019	104	844	1	9.8	0	38.47
1 primi	2	H						
89	1	03/22/2019	105	845	1	9.8	0	40.31

1 primi	2	H						
89	1	03/23/2019	106	846	1	9.8	0	40.05
1 primi	2	H						
89	1	03/24/2019	107	847	1	9.6	0	39.04
1 primi	2	H						
89	1	03/25/2019	108	848	1	9.8	0.09	39.61
1 primi	2	H						
89	1	03/26/2019	109	849	1	9.6	0.17	37.6
1 primi	2	H						
89	1	03/27/2019	110	850	1	9.8	0.17	41.03
1 primi	2	H						
89	1	03/28/2019	111	851	1	10	0	41.33
1 primi	2	H						
89	1	03/29/2019	112	852	1	9.8	0.13	39.53
1 primi	2	H						
89	1	03/30/2019	113	853	1	9.8	0.01	36.85
1 primi	2	H						
89	1	03/31/2019	114	854	1	9.9	0	37.42
1 primi	2	H						
89	1	04/01/2019	115	855	1	9.9	0	42.43
1 primi	2	H						
89	1	04/02/2019	116	856	1	10.2	0	37.43
1 primi	2	H						
89	1	04/03/2019	117	857	1	9.9	0.09	39.58
1 primi	2	H						
89	1	04/04/2019	118	858	1	9.9	0	40.05
1 primi	2	H						
89	1	04/05/2019	119	859	1	10	0	38.95
1 primi	2	H						
89	1	04/06/2019	120	860	1	10.3	0.16	38.53
1 primi	2	H						
89	1	04/07/2019	121	861	1	9.9	0.1	40.28
1 primi	2	H						
89	1	04/08/2019	122	862	1	9.8	0	37.59
1 primi	2	H						
89	1	04/09/2019	123	863	1	9.9	0	37.22
1 primi	2	H						
89	1	04/10/2019	124	864	1	9.5	0	38.48
1 primi	2	H						
89	1	04/11/2019	125	865	1	9.8	0	38.75
1 primi	2	H						
89	1	04/12/2019	126	866	1	10	0	38.14
1 primi	2	H						
89	1	04/13/2019	127	867	1	9.8	0.1	38.69
1 primi	2	H						
89	1	04/14/2019	128	868	1	10	0	41.51
1 primi	2	H						
89	1	04/15/2019	129	869	1	10	0	41.73
1 primi	2	H						
89	1	04/16/2019	130	870	1	10.2	0	41.71
1 primi	2	H						
89	1	04/17/2019	131	871	1	10.3	0	37.84
1 primi	2	H						
89	1	04/18/2019	132	872	1	10.4	0	41.7

1 primi 2		H						
89	1	04/19/2019	133	873	1	9.9	0	38.85
1 primi 2		H						
89	1	04/20/2019	134	874	1	10	0	42.39
1 primi 2		H						
89	1	04/21/2019	135	875	1	10	0	40.82
1 primi 2		H						
89	1	04/22/2019	136	876	1	10	0	39.57
1 primi 2		H						
89	1	04/23/2019	137	877	1	10.6	0	36.62
1 primi 2		H						
89	1	04/24/2019	138	878	1	9.8	0	39.37
1 primi 2		H						
89	1	04/25/2019	139	879	1	10.2	0	42.4
1 primi 2		H						
89	1	04/26/2019	140	880	1	9.8	0	38.87
1 primi 2		H						
89	1	04/27/2019	141	881	1	9.8	0	39.74
1 primi 2		H						
89	1	04/28/2019	142	882	1	9.9	0	39.2
1 primi 2		H						
89	1	04/29/2019	143	883	1	10	0	41.18
1 primi 2		H						
89	1	04/30/2019	144	884	1	10	0	41.84
1 primi 2		H						
89	1	05/01/2019	145	885	1	10.2	0	40.29
1 primi 2		H						
89	1	05/02/2019	146	886	1	10.2	0	41.72
1 primi 2		H						
89	1	05/03/2019	147	887	1	10.6	0.08	39.63
1 primi 2		H						
89	1	05/04/2019	148	888	1	10	0	38.8
1 primi 2		H						
89	1	05/05/2019	149	889	1	10	0	40.39
1 primi 2		H						
89	1	05/06/2019	150	890	1	10	0.13	38.73
1 primi 2		H						
89	1	05/07/2019	151	891	1	9.6	0.23	39.95
1 primi 2		H						
89	1	05/08/2019	152	892	1	9.9	0	39.16
1 primi 2		H						
89	1	05/09/2019	153	893	1	10.2	0	39.16
1 primi 2		H						
89	1	05/10/2019	154	894	1	9.9	0	40.64
1 primi 2		H						
89	1	05/11/2019	155	895	1	9.9	0	42.04
1 primi 2		H						
89	1	05/12/2019	156	896	1	9.9	0	40.52
1 primi 2		H						
89	1	05/13/2019	157	897	1	9.9	0	42.36
1 primi 2		H						
89	1	05/14/2019	158	898	1	9.9	0	42.72
1 primi 2		H						
89	1	05/15/2019	159	899	1	9.9	0.11	39.23

1 primi	2	H						
89	1	05/16/2019	160	900	1	10.3	0.25	40.22
1 primi	2	H						
89	1	05/17/2019	161	901	1	10.7	0	38.6
1 primi	2	H						
89	1	05/18/2019	162	902	1	9.8	0.15	42.65
1 primi	2	H						
89	1	05/19/2019	163	903	1	10.3	0	45.76
1 primi	2	H						
89	1	05/20/2019	164	904	1	10.2	0.16	34.88
1 primi	2	H						
89	1	05/21/2019	165	905	1	10	0	38.35
1 primi	2	H						
89	1	05/22/2019	166	906	1	9.9	0.08	38.6
1 primi	2	H						
89	1	05/23/2019	167	907	1	9.9	0	42.2
1 primi	2	H						
89	1	05/24/2019	168	908	1	9.9	0.19	38.57
1 primi	2	H						
89	1	05/25/2019	169	909	1	9.9	0	40.08
1 primi	2	H						
89	1	05/26/2019	170	910	1	9.9	0	37.62
1 primi	2	H						
89	1	05/27/2019	171	911	1	10.2	0	36.98
1 primi	2	H						
89	1	05/28/2019	172	912	1	10	0	38.86
1 primi	2	H						
89	1	05/29/2019	173	913	1	10	0	39.15
1 primi	2	H						
89	1	05/30/2019	174	914	1	10	0	41.42
1 primi	2	H						
89	1	05/31/2019	175	915	1	10.2	0	39.21
1 primi	2	H						
89	1	06/01/2019	176	916	1	10	0.06	36.76
1 primi	2	H						
89	1	06/02/2019	177	917	1	9.9	0	38.55
1 primi	2	H						
89	1	06/03/2019	178	918	1	10	0.32	37.09
1 primi	2	H						
89	1	06/04/2019	179	919	1	10	0	38.69
1 primi	2	H						
89	1	06/05/2019	180	920	1	10	0.19	38.03
1 primi	2	H						
89	1	06/06/2019	181	921	1	10.3	0	39.24
1 primi	2	H						
89	1	06/07/2019	182	922	1	10.2	0	38.62
1 primi	2	H						
89	1	06/08/2019	183	923	1	10.3	0.56	37.73
1 primi	2	H						
89	1	06/09/2019	184	924	1	9.8	0	39.83
1 primi	2	H						
89	1	06/10/2019	185	925	1	10	0	36.21
1 primi	2	H						
89	1	06/11/2019	186	926	1	10	0	38.43

1 primi	2	H						
89	1	06/12/2019	187	927	1	10.3	0.1	40.77
1 primi	2	H						
89	1	06/13/2019	188	928	1	10.3	0.16	38.05
1 primi	2	H						
89	1	06/14/2019	189	929	1	10.2	0.07	37.41
1 primi	2	H						
89	1	06/15/2019	190	930	1	10.2	0.02	39.55
1 primi	2	H						
89	1	06/16/2019	191	931	1	10.4	0	31.07
1 primi	2	H						
89	1	06/17/2019	192	932	1	10	0	32.96
1 primi	2	H						
89	1	06/18/2019	193	933	1	10.6	0	35.28
1 primi	2	H						
89	1	06/19/2019	194	934	1	10.4	0	35.81
1 primi	2	H						
89	1	06/20/2019	195	935	1	10.4	0.14	36.11
1 primi	2	H						
89	1	06/21/2019	196	936	1	10.4	0	35.59
1 primi	2	H						
89	1	06/22/2019	197	937	1	10.3	0.08	35.84
1 primi	2	H						
89	1	06/23/2019	198	938	1	11.3	0	38.19
1 primi	2	H						
89	1	06/24/2019	199	939	1	10.7	0.14	37.4
1 primi	2	H						
89	1	06/25/2019	200	940	1	9.5	0.2	28.01
1 primi	2	H						
89	1	06/26/2019	201	941	1	11.1	0	35.67
1 primi	2	H						
89	1	03/08/2020	4	1197	2	9.1	0.56	40.52
2 multi	1	H						
89	1	03/09/2020	5	1198	2	9.2	0.19	40.87
2 multi	1	H						
89	1	03/10/2020	6	1199	2	8.9	0.19	38.21
2 multi	1	H						
89	1	03/11/2020	7	1200	2	8.9	0.04	43.75
2 multi	1	H						
89	1	03/12/2020	8	1201	2	9.2	0.21	44.8
2 multi	1	H						
89	1	03/13/2020	9	1202	2	9.5	0.09	47.04
2 multi	1	H						
89	1	03/14/2020	10	1203	2	9.9	0.16	45.17
2 multi	1	H						
89	1	03/15/2020	11	1204	2	10	0.28	48.69
2 multi	1	H						
89	1	03/16/2020	12	1205	2	9.8	0.05	49.19
2 multi	1	H						
89	1	03/17/2020	13	1206	2	9.8	0.53	51.15
2 multi	1	H						
89	1	03/18/2020	14	1207	2	9.9	0.36	53.47
2 multi	1	H						
89	1	03/19/2020	15	1208	2	10.3	0.09	53.49

2 multi	1	H						
89	1	03/20/2020	16	1209	2	10	0.28	51.9
2 multi	1	H						
89	1	03/21/2020	17	1210	2	10.3	0.28	52.99
2 multi	1	H						
89	1	03/22/2020	18	1211	2	10.2	0.2	52.5
2 multi	1	H						
89	1	03/23/2020	19	1212	2	10.4	0.08	57.07
2 multi	1	H						
89	1	03/24/2020	20	1213	2	10.4	0.01	51.39
2 multi	1	H						
89	1	03/25/2020	21	1214	2	10.3	0	56.2
2 multi	1	H						
89	1	03/26/2020	22	1215	2	10.6	0.01	53.7
2 multi	2	H						
89	1	03/27/2020	23	1216	2	10.6	0.07	57.36
2 multi	2	H						
89	1	03/28/2020	24	1217	2	10.4	0	56.03
2 multi	2	H						
89	1	03/29/2020	25	1218	2	10.3	0	52.39
2 multi	2	H						
89	1	03/30/2020	26	1219	2	10.4	0	54.47
2 multi	2	H						
89	1	03/31/2020	27	1220	2	10.4	0	56.54
2 multi	2	H						
89	1	04/01/2020	28	1221	2	10.9	0	54.22
2 multi	2	H						
89	1	04/02/2020	29	1222	2	10.7	0.02	52.88
2 multi	2	H						
89	1	04/03/2020	30	1223	2	10.7	0	57.91
2 multi	2	H						
89	1	04/04/2020	31	1224	2	10.4	0	58.8
2 multi	2	H						
89	1	04/05/2020	32	1225	2	11.1	0.19	56.08
2 multi	2	H						
89	1	04/06/2020	33	1226	2	11.7	0.54	41.94
2 multi	2	H						
89	1	04/07/2020	34	1227	2	9.9	0	40.84
2 multi	2	H						
89	1	04/08/2020	35	1228	2	9.9	0.01	45.75
2 multi	2	H						
89	1	04/09/2020	36	1229	2	10.4	0.15	49.21
2 multi	2	H						
89	1	04/10/2020	37	1230	2	10.6	0	49.29
2 multi	2	H						
89	1	04/11/2020	38	1231	2	9.9	0	49.64
2 multi	2	H						
89	1	04/12/2020	39	1232	2	10.3	0	50.51
2 multi	2	H						
89	1	04/13/2020	40	1233	2	10.7	0	53.09
2 multi	2	H						
89	1	04/14/2020	41	1234	2	10.4	0	54.11
2 multi	2	H						
89	1	04/15/2020	42	1235	2	10.4	0	56.26

2 multi	2	H						
89	1	04/16/2020	43	1236	2	10.7	0	54.7
2 multi	2	H						
89	1	04/17/2020	44	1237	2	11.3	0	56.27
2 multi	2	H						
89	1	04/18/2020	45	1238	2	10.7	0	52
2 multi	2	H						
89	1	04/19/2020	46	1239	2	11.1	0	57.52
2 multi	2	H						
89	1	04/20/2020	47	1240	2	10.6	0	54.96
2 multi	2	H						
89	1	04/21/2020	48	1241	2	10.9	0	55.98
2 multi	2	H						
89	1	04/22/2020	49	1242	2	11.1	0	53.49
2 multi	2	H						
89	1	04/23/2020	50	1243	2	11.3	0.15	49.41
2 multi	2	H						
89	1	04/24/2020	51	1244	2	10.4	0.33	52.13
2 multi	2	H						
89	1	04/25/2020	52	1245	2	10.7	0	54.11
2 multi	2	H						
89	1	04/26/2020	53	1246	2	10.4	0	55.03
2 multi	2	H						
89	1	04/27/2020	54	1247	2	10.9	0	56.3
2 multi	2	H						
89	1	04/28/2020	55	1248	2	10.9	0	57.92
2 multi	2	H						
89	1	04/29/2020	56	1249	2	10.9	0	52.88
2 multi	2	H						
89	1	04/30/2020	57	1250	2	10.7	0.04	55.72
2 multi	2	H						
89	1	05/01/2020	58	1251	2	11.1	0.07	55.1
2 multi	2	H						
89	1	05/02/2020	59	1252	2	11.1	0	55.46
2 multi	2	H						
89	1	05/03/2020	60	1253	2	11.1	0.22	52.81
2 multi	2	H						
89	1	05/04/2020	61	1254	2	10.9	0	55.12
2 multi	2	H						
89	1	05/05/2020	62	1255	2	11.1	0.18	54.57
2 multi	2	H						
89	1	05/06/2020	63	1256	2	10.7	0.72	53.89
2 multi	2	H						
89	1	05/07/2020	64	1257	2	10.9	0.37	53.26
2 multi	2	H						
89	1	05/08/2020	65	1258	2	10.9	0	55.53
2 multi	2	H						
89	1	05/09/2020	66	1259	2	10.4	0	53.05
2 multi	2	H						
89	1	05/10/2020	67	1260	2	10.9	0	56.98
2 multi	2	H						
89	1	05/11/2020	68	1261	2	10.9	0.15	54.94
2 multi	2	H						
89	1	05/12/2020	69	1262	2	10.9	0.01	52.59

2 multi	2	H						
89	1	05/13/2020	70	1263	2	10.9	0	48.64
2 multi	2	H						
89	1	05/14/2020	71	1264	2	11.1	0	53.84
2 multi	2	H						
89	1	05/15/2020	72	1265	2	11.1	0	54.31
2 multi	2	H						
89	1	05/16/2020	73	1266	2	10.6	0.02	52.62
2 multi	2	H						
89	1	05/17/2020	74	1267	2	11.4	0	56.22
2 multi	2	H						
89	1	05/18/2020	75	1268	2	11.1	0	55.38
2 multi	2	H						
89	1	05/19/2020	76	1269	2	11.3	0.04	54.47
2 multi	2	H						
89	1	05/20/2020	77	1270	2	11.1	0.22	54.66
2 multi	2	H						
89	1	05/21/2020	78	1271	2	10.9	0.01	54.87
2 multi	2	H						
89	1	05/22/2020	79	1272	2	10.7	0	57.23
2 multi	2	H						
89	1	05/23/2020	80	1273	2	10.7	0	55.95
2 multi	2	H						
89	1	05/24/2020	81	1274	2	10.7	0	54.94
2 multi	2	H						
89	1	05/25/2020	82	1275	2	10.6	0	55.06
2 multi	2	H						
89	1	05/26/2020	83	1276	2	11.1	0	53.29
2 multi	2	H						
89	1	05/27/2020	84	1277	2	10.9	0	53.63
2 multi	2	H						
89	1	05/28/2020	85	1278	2	10.7	0	55.48
2 multi	2	H						
89	1	05/29/2020	86	1279	2	10.9	0	54.93
2 multi	2	H						
89	1	05/30/2020	87	1280	2	10.9	0.05	55.91
2 multi	2	H						
89	1	05/31/2020	88	1281	2	11.1	0	56.89
2 multi	2	H						
89	1	06/01/2020	89	1282	2	11.1	0.06	53.47
2 multi	2	H						
89	1	06/02/2020	90	1283	2	11.3	0.42	54.28
2 multi	2	H						
89	1	06/03/2020	91	1284	2	11.3	0	54.43
2 multi	2	H						
89	1	06/04/2020	92	1285	2	11.1	0	53.65
2 multi	2	H						
89	1	06/05/2020	93	1286	2	10.9	0	52.92
2 multi	2	H						
89	1	06/06/2020	94	1287	2	10.2	0	55.66
2 multi	2	H						
89	1	06/07/2020	95	1288	2	10.3	0	58.14
2 multi	2	H						
89	1	06/08/2020	96	1289	2	10.7	0	55.19

2 multi 2		H						
89	1	06/09/2020	97	1290	2	10.6	0	58.49
2 multi 2		H						
89	1	06/10/2020	98	1291	2	10.7	0	51.38
2 multi 2		H						
89	1	06/11/2020	99	1292	2	10.6	0	53.54
2 multi 2		H						
89	1	06/12/2020	100	1293	2	10.6	0.18	50.1
2 multi 2		H						
89	1	06/13/2020	101	1294	2	11.1	0.43	55.83
2 multi 2		H						
89	1	06/14/2020	102	1295	2	10.6	0.01	55.39
2 multi 2		H						
89	1	06/15/2020	103	1296	2	10.9	0	52.62
2 multi 2		H						
89	1	06/16/2020	104	1297	2	10.7	0.09	53.82
2 multi 2		H						
89	1	06/17/2020	105	1298	2	10.6	0.09	52.94
2 multi 2		H						
89	1	06/18/2020	106	1299	2	10.6	0	53.71
2 multi 2		H						
89	1	06/19/2020	107	1300	2	11.1	0	54.16
2 multi 2		H						
89	1	06/20/2020	108	1301	2	10.7	0	54
2 multi 2		H						
89	1	06/21/2020	109	1302	2	10.9	0	51.4
2 multi 2		H						
89	1	06/22/2020	110	1303	2	10.9	0.02	52.35
2 multi 2		H						
89	1	06/23/2020	111	1304	2	10.9	0	52.57
2 multi 2		H						
89	1	06/24/2020	112	1305	2	10.7	0	49.7
2 multi 2		H						
89	1	06/25/2020	113	1306	2	10.6	0	51.79
2 multi 2		H						
89	1	06/26/2020	114	1307	2	10.6	0	48.95
2 multi 2		H						
89	1	06/27/2020	115	1308	2	10.7	0	54.33
2 multi 2		H						
89	1	06/28/2020	116	1309	2	11.3	0	50.08
2 multi 2		H						
89	1	06/29/2020	117	1310	2	11.3	0	47.51
2 multi 2		H						
89	1	06/30/2020	118	1311	2	11.7	0	47.59
2 multi 2		H						
89	1	07/01/2020	119	1312	2	11.3	0	53.57
2 multi 2		H						
89	1	07/02/2020	120	1313	2	10.9	0	48.53
2 multi 2		H						
89	1	07/03/2020	121	1314	2	11.1	0.08	49.55
2 multi 2		H						
89	1	07/04/2020	122	1315	2	10.9	0	52.52
2 multi 2		H						
89	1	07/05/2020	123	1316	2	10.9	0.24	44

2 multi	2	H						
89	1	07/06/2020	124	1317	2	10.9	0.1	48.97
2 multi	2	H						
89	1	07/07/2020	125	1318	2	10.7	0	49.69
2 multi	2	H						
89	1	07/08/2020	126	1319	2	10.7	0.19	50.21
2 multi	2	H						
89	1	07/09/2020	127	1320	2	10.6	0.42	49.39
2 multi	2	H						
89	1	07/10/2020	128	1321	2	10.6	0	55.2
2 multi	2	H						
89	1	07/11/2020	129	1322	2	10.6	0.06	48.06
2 multi	2	H						
89	1	07/12/2020	130	1323	2	10.7	0	48.82
2 multi	2	H						
89	1	07/13/2020	131	1324	2	10.7	0.41	47.54
2 multi	2	H						
89	1	07/14/2020	132	1325	2	10.6	0.32	48.28
2 multi	2	H						
89	1	07/15/2020	133	1326	2	10.9	0.15	48.11
2 multi	2	H						
89	1	07/16/2020	134	1327	2	10.9	0	51
2 multi	2	H						
89	1	07/17/2020	135	1328	2	11.3	0	48.03
2 multi	2	H						
89	1	07/18/2020	136	1329	2	11.1	0	49.31
2 multi	2	H						
89	1	07/19/2020	137	1330	2	11.1	0.21	49.24
2 multi	2	H						
89	1	07/20/2020	138	1331	2	11.3	0.14	49.07
2 multi	2	H						
89	1	07/21/2020	139	1332	2	11.3	0	48.19
2 multi	2	H						
89	1	07/22/2020	140	1333	2	10.9	0.09	49.68
2 multi	2	H						
89	1	07/23/2020	141	1334	2	11.1	0	49.69
2 multi	2	H						
89	1	07/24/2020	142	1335	2	10.9	0.3	49.27
2 multi	2	H						
89	1	07/25/2020	143	1336	2	11.3	0	46.33
2 multi	2	H						
89	1	07/26/2020	144	1337	2	11.3	0	48.01
2 multi	2	H						
89	1	07/27/2020	145	1338	2	11.3	0	46.4
2 multi	2	H						
89	1	07/28/2020	146	1339	2	11.3	0	43.73
2 multi	2	H						
89	1	07/29/2020	147	1340	2	11.3	0	47.72
2 multi	2	H						
89	1	07/30/2020	148	1341	2	11.3	0	47.74
2 multi	2	H						
89	1	07/31/2020	149	1342	2	11.1	0.02	44.98
2 multi	2	H						
89	1	08/01/2020	150	1343	2	10.9	0	45.98

2 multi	2	H						
89	1	08/02/2020	151	1344	2	11.1	0	48.35
2 multi	2	H						
89	1	08/03/2020	152	1345	2	11.1	0	47.49
2 multi	2	H						
89	1	08/04/2020	153	1346	2	11.3	0.01	51.24
2 multi	2	H						
89	1	08/05/2020	154	1347	2	11.7	0	49.6
2 multi	2	H						
89	1	08/06/2020	155	1348	2	11.4	0.12	50.4
2 multi	2	H						
89	1	08/07/2020	156	1349	2	12	0.19	48.86
2 multi	2	H						
89	1	08/08/2020	157	1350	2	12.6	0.23	48.31
2 multi	2	H						
89	1	08/09/2020	158	1351	2	12	0	44.71
2 multi	2	H						
89	1	08/10/2020	159	1352	2	12	0	46.49
2 multi	2	H						
89	1	08/11/2020	160	1353	2	12.9	0	42.97
2 multi	2	H						
89	1	08/12/2020	161	1354	2	12.3	0.26	41.1
2 multi	2	H						
89	1	08/13/2020	162	1355	2	13.6	0.35	28.16
2 multi	2	H						
89	1	08/14/2020	163	1356	2	12.5	0	40.62
2 multi	2	H						
89	1	08/15/2020	164	1357	2	10.7	0.06	40.87
2 multi	2	H						
89	1	08/16/2020	165	1358	2	10.9	0.04	40.45
2 multi	2	H						
89	1	08/17/2020	166	1359	2	11.9	0.09	41
2 multi	2	H						
89	1	08/18/2020	167	1360	2	11.7	0.02	41.1
2 multi	2	H						
89	1	08/19/2020	168	1361	2	11.9	0	44.41
2 multi	2	H						
89	1	08/20/2020	169	1362	2	11.4	0	43.31
2 multi	2	H						
89	1	08/21/2020	170	1363	2	12	0.1	44.71
2 multi	2	H						
89	1	08/22/2020	171	1364	2	11.7	0	44.47
2 multi	2	H						
89	1	08/23/2020	172	1365	2	11.9	0.08	44.03
2 multi	2	H						
89	1	08/24/2020	173	1366	2	12.3	0.01	43.43
2 multi	2	H						
89	1	08/25/2020	174	1367	2	11.9	0	43.82
2 multi	2	H						
89	1	08/26/2020	175	1368	2	12	0	42.23
2 multi	2	H						
89	1	08/27/2020	176	1369	2	12.3	0	39.69
2 multi	2	H						
89	1	08/28/2020	177	1370	2	12.3	0.01	41.03

2 multi	2	H						
89	1	08/29/2020	178	1371	2	11.7	0.01	42.82
2 multi	2	H						
89	1	08/30/2020	179	1372	2	11.7	0	43.22
2 multi	2	H						
89	1	08/31/2020	180	1373	2	11.9	0	41.04
2 multi	2	H						
89	1	09/01/2020	181	1374	2	11.7	0	43.08
2 multi	2	H						
89	1	09/02/2020	182	1375	2	11.7	0	41.23
2 multi	2	H						
89	1	09/03/2020	183	1376	2	11.9	0.02	44.03
2 multi	2	H						
89	1	09/04/2020	184	1377	2	11.7	0	41.92
2 multi	2	H						
89	1	09/05/2020	185	1378	2	11.7	0.02	42.24
2 multi	2	H						
89	1	09/06/2020	186	1379	2	11.7	0	41.14
2 multi	2	H						
89	1	09/07/2020	187	1380	2	11.9	0	39.88
2 multi	2	H						
89	1	09/08/2020	188	1381	2	12	0	36.85
2 multi	2	H						
89	1	09/09/2020	189	1382	2	11.9	0	39.73
2 multi	2	H						
89	1	09/10/2020	190	1383	2	12	0	39.9
2 multi	2	H						
89	1	09/11/2020	191	1384	2	11.7	0	43.75
2 multi	2	H						
89	1	09/12/2020	192	1385	2	11.7	0	41.01
2 multi	2	H						
89	1	09/13/2020	193	1386	2	11.7	0	39.45
2 multi	2	H						
89	1	09/14/2020	194	1387	2	11.7	0	42.14
2 multi	2	H						
89	1	09/15/2020	195	1388	2	11.4	0	41.39
2 multi	2	H						
89	1	09/16/2020	196	1389	2	11.9	0.01	43.62
2 multi	2	H						
89	1	09/17/2020	197	1390	2	11.3	0	39.37
2 multi	2	H						
89	1	09/18/2020	198	1391	2	11.7	0	41.61
2 multi	2	H						
89	1	09/19/2020	199	1392	2	11.3	0.01	43.5
2 multi	2	H						
89	1	09/20/2020	200	1393	2	11.7	0.04	40.99
2 multi	2	H						
89	1	09/21/2020	201	1394	2	11.1	0	44.01
2 multi	2	H						
89	1	09/22/2020	202	1395	2	11.4	0	43.79
2 multi	2	H						
89	1	09/23/2020	203	1396	2	11.4	0	41.46
2 multi	2	H						
89	1	09/24/2020	204	1397	2	11.7	0	44.01

2 multi	2	H						
89	1	09/25/2020	205	1398	2	11.3	0	42.54
2 multi	2	H						
89	1	09/26/2020	206	1399	2	11.4	0	43.61
2 multi	2	H						
89	1	09/27/2020	207	1400	2	11.9	0.06	42.33
2 multi	2	H						
89	1	09/28/2020	208	1401	2	11.1	0	41.45
2 multi	2	H						
89	1	09/29/2020	209	1402	2	11.4	0	43.18
2 multi	2	H						
89	1	09/30/2020	210	1403	2	11.1	0.05	40.55
2 multi	2	H						
89	1	10/01/2020	211	1404	2	11.3	0	41.32
2 multi	2	H						
89	1	10/02/2020	212	1405	2	11.7	0	38.44
2 multi	2	H						
89	1	10/03/2020	213	1406	2	11.4	0	41.1
2 multi	2	H						
89	1	10/04/2020	214	1407	2	11.4	0	37.87
2 multi	2	H						
89	1	10/05/2020	215	1408	2	11.7	0.02	38.33
2 multi	2	H						
89	1	10/06/2020	216	1409	2	11.3	0	38.34
2 multi	2	H						
89	1	10/07/2020	217	1410	2	11.1	0.01	42.76
2 multi	2	H						
89	1	10/08/2020	218	1411	2	11.4	0	24.19
2 multi	2	H						
89	1	10/09/2020	219	1412	2	11.1	0.09	44.93
2 multi	2	H						
89	1	10/10/2020	220	1413	2	12.5	0.05	38.18
2 multi	2	H						
89	1	10/11/2020	221	1414	2	11.9	0	40.2
2 multi	2	H						
89	1	10/12/2020	222	1415	2	11.4	0.22	35.67
2 multi	2	H						
89	1	10/13/2020	223	1416	2	11.9	0	39.75
2 multi	2	H						
89	1	10/14/2020	224	1417	2	12	0.04	39.24
2 multi	2	H						
89	1	10/15/2020	225	1418	2	11.7	0	41.41
2 multi	2	H						
89	1	10/16/2020	226	1419	2	12.3	0.03	39.23
2 multi	2	H						
89	1	10/17/2020	227	1420	2	12.3	0.01	39.88
2 multi	2	H						
89	1	10/18/2020	228	1421	2	12.6	0.28	36.83
2 multi	2	H						
89	1	10/19/2020	229	1422	2	12	0.01	38.92
2 multi	2	H						
89	1	10/20/2020	230	1423	2	12	0.21	33.91
2 multi	2	H						
89	1	10/21/2020	231	1424	2	11.4	0.21	39.54

2 multi 2		H						
89	1	10/22/2020	232	1425	2	11.9	0.01	37.49
2 multi 2		H						
89	1	10/23/2020	233	1426	2	12.3	0.13	40.6
2 multi 2		H						
89	1	10/24/2020	234	1427	2	10.6	0.28	41.3
2 multi 2		H						
89	1	10/25/2020	235	1428	2	12	0	38.05
2 multi 2		H						
89	1	10/26/2020	236	1429	2	11.7	0.03	39.63
2 multi 2		H						
89	1	10/27/2020	237	1430	2	11.9	0	33.28
2 multi 2		H						
89	1	10/28/2020	238	1431	2	12.3	0.03	41.4
2 multi 2		H						
89	1	10/29/2020	239	1432	2	11.7	0.02	38.28
2 multi 2		H						
89	1	10/30/2020	240	1433	2	12	0.05	42.11
2 multi 2		H						
89	1	10/31/2020	241	1434	2	12.5	0.03	41.52
2 multi 2		H						
89	1	11/01/2020	242	1435	2	12.5	0.04	39.74
2 multi 2		H						
89	1	11/02/2020	243	1436	2	12	0.04	43.24
2 multi 2		H						
89	1	11/03/2020	244	1437	2	11.9	0	41.88
2 multi 2		H						
89	1	11/04/2020	245	1438	2	11.3	0.05	40.29
2 multi 2		H						
89	1	11/05/2020	246	1439	2	11.7	0.02	44.56
2 multi 2		H						
89	1	11/06/2020	247	1440	2	12.3	0.05	40.8
2 multi 2		H						
89	1	11/07/2020	248	1441	2	11.9	0	39.01
2 multi 2		H						
89	1	11/08/2020	249	1442	2	11.3	0.05	40.84
2 multi 2		H						
89	1	11/09/2020	250	1443	2	12	0.04	40
2 multi 2		H						
89	1	11/10/2020	251	1444	2	11.7	0.05	36.65
2 multi 2		H						
89	1	11/11/2020	252	1445	2	12.3	0	38.59
2 multi 2		H						
89	1	11/12/2020	253	1446	2	12	0.01	39.75
2 multi 2		H						
89	1	11/13/2020	254	1447	2	11.4	0.03	41.75
2 multi 2		H						
89	1	11/14/2020	255	1448	2	11.9	0.06	40.13
2 multi 2		H						
89	1	11/15/2020	256	1449	2	12	0.05	39.56
2 multi 2		H						
89	1	11/16/2020	257	1450	2	11.4	0	43.1
2 multi 2		H						
89	1	11/17/2020	258	1451	2	11.9	0	42.49

2 multi	2	H						
89	1	11/18/2020	259	1452	2	11.4	0.03	44.03
2 multi	2	H						
89	1	11/19/2020	260	1453	2	11.9	0.38	41.84
2 multi	2	H						
89	1	11/20/2020	261	1454	2	11.7	0.03	45.36
2 multi	2	H						
89	1	11/21/2020	262	1455	2	10.9	0.06	43.13
2 multi	2	H						
89	1	11/22/2020	263	1456	2	10.2	0.09	43.06
2 multi	2	H						
89	1	11/23/2020	264	1457	2	11.4	0	45.19
2 multi	2	H						
89	1	11/24/2020	265	1458	2	11.7	0.03	43.74
2 multi	2	H						
89	1	11/25/2020	266	1459	2	11.7	0.03	27.8
2 multi	2	H						
89	1	11/26/2020	267	1460	2	11.9	0.01	51.57
2 multi	2	H						
89	1	11/27/2020	268	1461	2	11.7	0.03	40.33
2 multi	2	H						
89	1	11/28/2020	269	1462	2	11.7	0.03	43.95
2 multi	2	H						
89	1	11/29/2020	270	1463	2	11.7	0	42.85
2 multi	2	H						
89	1	11/30/2020	271	1464	2	11.4	0.17	41.04
2 multi	2	H						
89	1	12/01/2020	272	1465	2	11.4	0.05	45.32
2 multi	2	H						
89	1	12/02/2020	273	1466	2	11.4	0.04	44.73
2 multi	2	H						
89	1	12/03/2020	274	1467	2	11.7	0.43	45.01
2 multi	2	H						
89	1	12/04/2020	275	1468	2	11.9	0.06	43.38
2 multi	2	H						
89	1	12/05/2020	276	1469	2	11.4	0.07	41.89
2 multi	2	H						
89	1	12/06/2020	277	1470	2	10.6	0.05	46.33
2 multi	2	H						
89	1	12/07/2020	278	1471	2	11.7	0.02	42.95
2 multi	2	H						
89	1	12/08/2020	279	1472	2	11.7	0.02	41.5
2 multi	2	H						
89	1	12/09/2020	280	1473	2	12	0.38	41.06
2 multi	2	H						
89	1	12/10/2020	281	1474	2	12	0	39.3
2 multi	2	H						
89	1	12/11/2020	282	1475	2	12	0	36.84
2 multi	2	H						
89	1	12/12/2020	283	1476	2	11.7	0	40.81
2 multi	2	H						
89	1	12/13/2020	284	1477	2	11.4	0	39.57
2 multi	2	H						
89	1	12/14/2020	285	1478	2	11.7	0	40.73

2 multi	2	H						
89	1	12/15/2020	286	1479	2	11.4	0	34.97
2 multi	2	H						
89	1	12/16/2020	287	1480	2	11.7	0	41.49
2 multi	2	H						
89	1	04/04/2021	5	1589	3	9.6	0.09	37.06
2 multi	1	H						
89	1	04/05/2021	6	1590	3	9.6	0.07	34.5
2 multi	1	H						
89	1	04/06/2021	7	1591	3	9.9	0	34.82
2 multi	1	H						
89	1	04/07/2021	8	1592	3	9.8	0	42.11
2 multi	1	H						
89	1	04/08/2021	9	1593	3	9.6	0.05	43.49
2 multi	1	H						
89	1	04/09/2021	10	1594	3	9.5	0	42.92
2 multi	1	H						
89	1	04/10/2021	11	1595	3	9.2	0.28	48.54
2 multi	1	H						
89	1	04/11/2021	12	1596	3	9.3	0.24	46.81
2 multi	1	H						
89	1	04/12/2021	13	1597	3	9.5	0.22	48.64
2 multi	1	H						
89	1	04/13/2021	14	1598	3	9.8	0.04	51.08
2 multi	1	H						
89	1	04/14/2021	15	1599	3	10	0	51.67
2 multi	1	H						
89	1	04/15/2021	16	1600	3	9.9	0.26	54.59
2 multi	1	H						
89	1	04/16/2021	17	1601	3	9.9	0.05	51.09
2 multi	1	H						
89	1	04/17/2021	18	1602	3	10	0.04	52.01
2 multi	1	H						
89	1	04/18/2021	19	1603	3	10	0.04	52.31
2 multi	1	H						
89	1	04/19/2021	20	1604	3	9.9	0.04	53.8
2 multi	1	H						
89	1	04/20/2021	21	1605	3	10.2	0.04	53.68
2 multi	1	H						
89	1	04/21/2021	22	1606	3	10	0	54.1
2 multi	2	H						
89	1	04/22/2021	23	1607	3	10.4	0	54.34
2 multi	2	H						
89	1	04/23/2021	24	1608	3	10	0.02	55.11
2 multi	2	H						
89	1	04/24/2021	25	1609	3	10	0.01	53.64
2 multi	2	H						
89	1	04/25/2021	26	1610	3	9.8	0	48.99
2 multi	2	H						
89	1	04/26/2021	27	1611	3	10	0.01	57.81
2 multi	2	H						
89	1	04/27/2021	28	1612	3	10.2	0	56.53
2 multi	2	H						
89	1	04/28/2021	29	1613	3	10.3	0	54.86

2 multi 2		H						
89	1	04/29/2021	30	1614	3	9.9	0	55.85
2 multi 2		H						
89	1	04/30/2021	31	1615	3	9.8	0.02	54.12
2 multi 2		H						
89	1	05/01/2021	32	1616	3	9.8	0.02	56.27
2 multi 2		H						
89	1	05/02/2021	33	1617	3	10	0	55.2
2 multi 2		H						
89	1	05/03/2021	34	1618	3	9.9	0.01	55.97
2 multi 2		H						
89	1	05/04/2021	35	1619	3	9.6	0	54.27
2 multi 2		H						
89	1	05/05/2021	36	1620	3	10	0.03	53.5
2 multi 2		H						
89	1	05/06/2021	37	1621	3	9.9	0	54.2
2 multi 2		H						
89	1	05/07/2021	38	1622	3	9.9	0.03	54.96
2 multi 2		H						
89	1	05/08/2021	39	1623	3	10	0	56.3
2 multi 2		H						
89	1	05/09/2021	40	1624	3	10	0	59.19
2 multi 2		H						
89	1	05/10/2021	41	1625	3	9.9	0	56.18
2 multi 2		H						
89	1	05/11/2021	42	1626	3	9.9	0	56.48
2 multi 2		H						
89	1	05/12/2021	43	1627	3	10	0.02	55.3
2 multi 2		H						
89	1	05/13/2021	44	1628	3	10.2	0.01	54.56
2 multi 2		H						
89	1	05/14/2021	45	1629	3	10	0	57.26
2 multi 2		H						
89	1	05/15/2021	46	1630	3	0	0.04	56.37
2 multi 2		H						
89	1	05/16/2021	47	1631	3	11.3	0.27	58.83
2 multi 2		H						
89	1	05/17/2021	48	1632	3	10.7	0.04	43.15
2 multi 2		H						
89	1	05/18/2021	49	1633	3	10.4	0.05	50.58
2 multi 2		H						
89	1	05/19/2021	50	1634	3	10.3	0.07	53.12
2 multi 2		H						
89	1	05/20/2021	51	1635	3	10.2	0.04	52.06
2 multi 2		H						
89	1	05/21/2021	52	1636	3	9.9	0.02	51.37
2 multi 2		H						
89	1	05/22/2021	53	1637	3	10.2	0.01	54.13
2 multi 2		H						
89	1	05/23/2021	54	1638	3	10	0	56.41
2 multi 2		H						
89	1	05/24/2021	55	1639	3	10.3	0.02	51.29
2 multi 2		H						
89	1	05/25/2021	56	1640	3	10.3	0.47	54.87

2 multi	2	H						
89	1	05/26/2021	57	1641	3	10.3	0	26.54
2 multi	2	H						
90	1	10/08/2019	7	721	1	12.9	0.36	9.27
1 primi	1	H						
90	1	10/09/2019	8	722	1	11.7	0.15	20.73
1 primi	1	H						
90	1	10/10/2019	9	723	1	11.3	0.14	23.05
1 primi	1	H						
90	1	10/11/2019	10	724	1	10.9	0.18	20.61
1 primi	1	H						
90	1	10/12/2019	11	725	1	10.3	0.14	21.25
1 primi	1	H						
90	1	10/13/2019	12	726	1	10.4	0.1	23.26
1 primi	1	H						
90	1	10/14/2019	13	727	1	10.3	0	16.18
1 primi	1	H						
90	1	10/15/2019	14	728	1	10.6	0.17	28.17
1 primi	1	H						
90	1	10/16/2019	15	729	1	9.8	0.05	18.02
1 primi	1	H						
90	1	10/17/2019	16	730	1	10.2	0.06	29.12
1 primi	1	H						
90	1	10/18/2019	17	731	1	10	0.01	25.56
1 primi	1	H						
90	1	10/19/2019	18	732	1	10	0.15	25.88
1 primi	1	H						
90	1	10/20/2019	19	733	1	9.6	0	25.57
1 primi	1	H						
90	1	10/21/2019	20	734	1	9.5	0.2	28.38
1 primi	1	H						
90	1	10/22/2019	21	735	1	9.6	0	24.27
1 primi	1	H						
90	1	10/23/2019	22	736	1	9.9	0.2	28.7
1 primi	2	H						
90	1	10/24/2019	23	737	1	9.8	0.07	26.01
1 primi	2	H						
90	1	10/25/2019	24	738	1	9.9	0.04	27.02
1 primi	2	H						
90	1	10/26/2019	25	739	1	9.6	0.15	24.91
1 primi	2	H						
90	1	10/27/2019	26	740	1	9.6	0.05	29.46
1 primi	2	H						
90	1	10/28/2019	27	741	1	9.5	0.07	28.94
1 primi	2	H						
90	1	10/29/2019	28	742	1	9.3	0.06	29.02
1 primi	2	H						
90	1	10/30/2019	29	743	1	8.9	0.02	29.35
1 primi	2	H						
90	1	10/31/2019	30	744	1	9.3	0.02	29.68
1 primi	2	H						
90	1	11/01/2019	31	745	1	9.8	0.05	29.7
1 primi	2	H						
90	1	11/02/2019	32	746	1	9.3	0	27.62

1 primi 2		H						
90	1	11/03/2019	33	747	1	9.6	0.06	34.27
1 primi 2		H						
90	1	11/04/2019	34	748	1	9.1	0.12	29.91
1 primi 2		H						
90	1	11/05/2019	35	749	1	9.5	0.15	31.93
1 primi 2		H						
90	1	11/06/2019	36	750	1	9.2	0.06	31.64
1 primi 2		H						
90	1	11/07/2019	37	751	1	9.3	0.05	29.21
1 primi 2		H						
90	1	11/08/2019	38	752	1	8.9	0	28.85
1 primi 2		H						
90	1	11/09/2019	39	753	1	9.1	0.12	30.57
1 primi 2		H						
90	1	11/10/2019	40	754	1	8.8	0	30.87
1 primi 2		H						
90	1	11/11/2019	41	755	1	8.7	0	30.29
1 primi 2		H						
90	1	11/12/2019	42	756	1	8.9	0	31.96
1 primi 2		H						
90	1	11/13/2019	43	757	1	8.6	0.13	29.66
1 primi 2		H						
90	1	11/14/2019	44	758	1	9.1	0	31.42
1 primi 2		H						
90	1	11/15/2019	45	759	1	8.9	0	29.72
1 primi 2		H						
90	1	11/16/2019	46	760	1	8.8	0.3	32.6
1 primi 2		H						
90	1	11/17/2019	47	761	1	9.1	0	33.3
1 primi 2		H						
90	1	11/18/2019	48	762	1	8.8	0.04	28.95
1 primi 2		H						
90	1	11/19/2019	49	763	1	9.3	0.21	32.17
1 primi 2		H						
90	1	11/20/2019	50	764	1	9.2	0	33.25
1 primi 2		H						
90	1	11/21/2019	51	765	1	9.1	0.01	34.91
1 primi 2		H						
90	1	11/22/2019	52	766	1	9.1	0.01	31.48
1 primi 2		H						
90	1	11/23/2019	53	767	1	9.2	0.07	33.5
1 primi 2		H						
90	1	11/24/2019	54	768	1	9.3	0	33.29
1 primi 2		H						
90	1	11/25/2019	55	769	1	9.3	0.15	33.03
1 primi 2		H						
90	1	11/26/2019	56	770	1	8.9	0	33.46
1 primi 2		H						
90	1	11/27/2019	57	771	1	8.7	0.01	31.99
1 primi 2		H						
90	1	11/28/2019	58	772	1	9.1	0.16	32.85
1 primi 2		H						
90	1	11/29/2019	59	773	1	9.1	0	32.31

1 primi 2		H						
90	1	11/30/2019	60	774	1	8.9	0	32.63
1 primi 2		H						
90	1	12/01/2019	61	775	1	9.3	0.03	33.08
1 primi 2		H						
90	1	12/02/2019	62	776	1	8.7	0.12	30.8
1 primi 2		H						
90	1	12/03/2019	63	777	1	8.8	0	31.78
1 primi 2		H						
90	1	12/04/2019	64	778	1	8.8	0.05	34.08
1 primi 2		H						
90	1	12/05/2019	65	779	1	8.9	0	32
1 primi 2		H						
90	1	12/06/2019	66	780	1	9.2	0.13	32.22
1 primi 2		H						
90	1	12/07/2019	67	781	1	9.2	0	33.34
1 primi 2		H						
90	1	12/08/2019	68	782	1	9.2	0.01	34.41
1 primi 2		H						
90	1	12/09/2019	69	783	1	9.6	0.14	33.74
1 primi 2		H						
90	1	12/10/2019	70	784	1	9.2	0.12	32.09
1 primi 2		H						
90	1	12/11/2019	71	785	1	9.1	0	32.33
1 primi 2		H						
90	1	12/12/2019	72	786	1	8.8	0	29.54
1 primi 2		H						
90	1	12/13/2019	73	787	1	9.3	0.13	31.92
1 primi 2		H						
90	1	12/14/2019	74	788	1	9.2	0.02	31.39
1 primi 2		H						
90	1	12/15/2019	75	789	1	9.2	0.12	33.41
1 primi 2		H						
90	1	12/16/2019	76	790	1	9.1	0.13	32.95
1 primi 2		H						
90	1	12/17/2019	77	791	1	9.1	0.11	31.81
1 primi 2		H						
90	1	12/18/2019	78	792	1	9.1	0.03	32.62
1 primi 2		H						
90	1	12/19/2019	79	793	1	9.1	0.03	30.73
1 primi 2		H						
90	1	12/20/2019	80	794	1	9.8	0.01	35.36
1 primi 2		H						
90	1	12/21/2019	81	795	1	9.2	0	34.57
1 primi 2		H						
90	1	12/22/2019	82	796	1	9.3	0.15	32.43
1 primi 2		H						
90	1	12/23/2019	83	797	1	9.5	0	33.81
1 primi 2		H						
90	1	12/24/2019	84	798	1	9.5	0.04	33.07
1 primi 2		H						
90	1	08/31/2020	8	1049	2	10	0.34	25.6
2 multi 1		H						
90	1	09/01/2020	9	1050	2	10.3	0	30.09

2 multi	1	H						
90	1	09/02/2020	10	1051	2	10.3	0	29.71
2 multi	1	H						
90	1	09/03/2020	11	1052	2	10.2	0.56	33.18
2 multi	1	H						
90	1	09/04/2020	12	1053	2	10.3	0	32.25
2 multi	1	H						
90	1	09/05/2020	13	1054	2	10.3	0.22	37.61
2 multi	1	H						
90	1	09/06/2020	14	1055	2	10.3	0	37.24
2 multi	1	H						
90	1	09/07/2020	15	1056	2	10	0.12	37.73
2 multi	1	H						
90	1	09/08/2020	16	1057	2	10.2	0.04	39.91
2 multi	1	H						
90	1	09/09/2020	17	1058	2	10	0	38.54
2 multi	1	H						
90	1	09/10/2020	18	1059	2	9.8	0.04	36.17
2 multi	1	H						
90	1	09/11/2020	19	1060	2	9.9	0.04	41.79
2 multi	1	H						
90	1	09/12/2020	20	1061	2	9.9	0	40.18
2 multi	1	H						
90	1	09/13/2020	21	1062	2	9.8	0.06	38.4
2 multi	1	H						
90	1	09/14/2020	22	1063	2	9.6	0.14	43.33
2 multi	2	H						
90	1	09/15/2020	23	1064	2	9.8	0.01	40.07
2 multi	2	H						
90	1	09/16/2020	24	1065	2	9.6	0.06	41.54
2 multi	2	H						
90	1	09/17/2020	25	1066	2	9.8	0	38.83
2 multi	2	H						
90	1	09/18/2020	26	1067	2	9.8	0.09	40.31
2 multi	2	H						
90	1	09/19/2020	27	1068	2	9.5	0.06	41.67
2 multi	2	H						
90	1	09/20/2020	28	1069	2	9.6	0.01	41.9
2 multi	2	H						
90	1	09/21/2020	29	1070	2	9.8	0.14	44.88
2 multi	2	H						
90	1	09/22/2020	30	1071	2	9.5	0.12	31.8
2 multi	2	H						
90	1	09/23/2020	31	1072	2	9.5	0.48	43.96
2 multi	2	H						
90	1	09/24/2020	32	1073	2	9.6	0.05	39.76
2 multi	2	H						
90	1	09/25/2020	33	1074	2	9.8	0.19	44.05
2 multi	2	H						
90	1	09/26/2020	34	1075	2	10	0.03	42.09
2 multi	2	H						
90	1	09/27/2020	35	1076	2	10.3	0	40.38
2 multi	2	H						
90	1	09/28/2020	36	1077	2	9.8	0.01	42.53

2 multi	2	H						
90	1	09/29/2020	37	1078	2	9.8	0.25	45.94
2 multi	2	H						
90	1	09/30/2020	38	1079	2	9.6	0.13	39.75
2 multi	2	H						
90	1	10/01/2020	39	1080	2	9.6	0.07	46.05
2 multi	2	H						
90	1	10/02/2020	40	1081	2	9.6	0.48	43.72
2 multi	2	H						
90	1	10/03/2020	41	1082	2	9.5	0.25	42.3
2 multi	2	H						
90	1	10/04/2020	42	1083	2	9.8	0.3	43.79
2 multi	2	H						
90	1	10/05/2020	43	1084	2	9.2	0.13	43.81
2 multi	2	H						
90	1	10/06/2020	44	1085	2	9.5	0.14	40.11
2 multi	2	H						
90	1	10/07/2020	45	1086	2	9.6	0.25	38.81
2 multi	2	H						
90	1	10/08/2020	46	1087	2	9.6	0	42.64
2 multi	2	H						
90	1	10/09/2020	47	1088	2	9.6	0	43.84
2 multi	2	H						
90	1	10/10/2020	48	1089	2	9.6	0.14	44.5
2 multi	2	H						
90	1	10/11/2020	49	1090	2	9.8	0.13	42.42
2 multi	2	H						
90	1	10/12/2020	50	1091	2	9.5	0.14	43.22
2 multi	2	H						
90	1	10/13/2020	51	1092	2	9.8	0.15	43.92
2 multi	2	H						
92	1	10/06/2014	3	707	1	11.1	0.12	21.82
1 primi	1	H						
92	1	10/07/2014	4	708	1	10.2	0.06	23.11
1 primi	1	H						
92	1	10/08/2014	5	709	1	10.4	0	24.73
1 primi	1	H						
92	1	10/09/2014	6	710	1	10.2	0.05	20.72
1 primi	1	H						
92	1	10/10/2014	7	711	1	10.4	0	22.62
1 primi	1	H						
92	1	10/11/2014	8	712	1	10.3	0	21
1 primi	1	H						
92	1	10/12/2014	9	713	1	10.2	0	19.77
1 primi	1	H						
92	1	10/13/2014	10	714	1	10.2	0	21.72
1 primi	1	H						
92	1	10/14/2014	11	715	1	9.9	0	23.38
1 primi	1	H						
92	1	10/15/2014	12	716	1	11.4	0	20.37
1 primi	1	H						
92	1	10/16/2014	13	717	1	11.7	0	24.29
1 primi	1	H						
92	1	10/17/2014	14	718	1	11.4	0	28.06

1 primi	1	H						
92	1	10/18/2014	15	719	1	10.7	0	26.85
1 primi	1	H						
92	1	10/19/2014	16	720	1	10.3	0	30.41
1 primi	1	H						
92	1	10/20/2014	17	721	1	10.4	0	29.96
1 primi	1	H						
92	1	10/21/2014	18	722	1	10	0	29.15
1 primi	1	H						
92	1	10/22/2014	19	723	1	10.2	0	30.83
1 primi	1	H						
92	1	10/23/2014	20	724	1	9.8	0	29.57
1 primi	1	H						
92	1	10/24/2014	21	725	1	10	0	33.06
1 primi	1	H						
92	1	10/25/2014	22	726	1	9.8	0	31.05
1 primi	2	H						
92	1	10/26/2014	23	727	1	13.5	0.21	28.85
1 primi	2	H						
92	1	10/27/2014	24	728	1	9.8	0	31.16
1 primi	2	H						
92	1	10/28/2014	25	729	1	9.8	0	30.37
1 primi	2	H						
92	1	10/29/2014	26	730	1	9.5	0	17.74
1 primi	2	H						
92	1	10/30/2014	27	731	1	10.2	0	32.9
1 primi	2	H						
92	1	10/31/2014	28	732	1	10.4	0	34.44
1 primi	2	H						
92	1	11/01/2014	29	733	1	9.9	0	34.34
1 primi	2	H						
92	1	11/02/2014	30	734	1	9.8	0	33.02
1 primi	2	H						
92	1	11/03/2014	31	735	1	10	0	34.67
1 primi	2	H						
92	1	11/04/2014	32	736	1	10.2	0	34.66
1 primi	2	H						
92	1	11/05/2014	33	737	1	10	0	36.01
1 primi	2	H						
92	1	11/06/2014	34	738	1	9.8	0	34.31
1 primi	2	H						
92	1	11/07/2014	35	739	1	9.9	0	35.52
1 primi	2	H						
92	1	11/08/2014	36	740	1	9.9	0	36.19
1 primi	2	H						
92	1	11/09/2014	37	741	1	9.8	0	35.62
1 primi	2	H						
92	1	11/10/2014	38	742	1	10.2	0	38.94
1 primi	2	H						
92	1	11/11/2014	39	743	1	10	0	35.8
1 primi	2	H						
92	1	11/12/2014	40	744	1	9.9	0	36.43
1 primi	2	H						
92	1	11/13/2014	41	745	1	9.6	0	34.69

1 primi 2		H						
92	1	11/14/2014	42	746	1	10	0	36.17
1 primi 2		H						
92	1	11/15/2014	43	747	1	9.5	0	37.24
1 primi 2		H						
92	1	11/16/2014	44	748	1	9.5	0	37.11
1 primi 2		H						
92	1	11/17/2014	45	749	1	9.8	0	37.53
1 primi 2		H						
92	1	11/18/2014	46	750	1	9.3	0	38.38
1 primi 2		H						
92	1	11/19/2014	47	751	1	9.8	0	38.34
1 primi 2		H						
92	1	11/20/2014	48	752	1	9.9	0	37.13
1 primi 2		H						
92	1	11/21/2014	49	753	1	9.6	0	39.49
1 primi 2		H						
92	1	11/22/2014	50	754	1	9.6	0	38.97
1 primi 2		H						
92	1	11/23/2014	51	755	1	9.5	0	36.97
1 primi 2		H						
92	1	11/24/2014	52	756	1	9.2	0	34.38
1 primi 2		H						
92	1	11/25/2014	53	757	1	9.5	0	38.06
1 primi 2		H						
92	1	11/26/2014	54	758	1	9.2	0	33.32
1 primi 2		H						
92	1	11/27/2014	55	759	1	9.5	0	39.48
1 primi 2		H						
92	1	11/28/2014	56	760	1	9.1	0	35.55
1 primi 2		H						
92	1	11/29/2014	57	761	1	9.3	0	34.1
1 primi 2		H						
92	1	11/30/2014	58	762	1	9.6	0	37.07
1 primi 2		H						
92	1	12/01/2014	59	763	1	9.6	0	36.33
1 primi 2		H						
92	1	12/02/2014	60	764	1	9.2	0	34.45
1 primi 2		H						
92	1	12/03/2014	61	765	1	9.3	0	36.58
1 primi 2		H						
92	1	12/04/2014	62	766	1	9.3	0	33.66
1 primi 2		H						
92	1	12/05/2014	63	767	1	9.8	0	40.38
1 primi 2		H						
92	1	12/06/2014	64	768	1	9.6	0	37.25
1 primi 2		H						
92	1	12/07/2014	65	769	1	9.3	0	39.62
1 primi 2		H						
92	1	12/08/2014	66	770	1	9.5	0	39.28
1 primi 2		H						
92	1	12/09/2014	67	771	1	9.2	0	35.53
1 primi 2		H						
92	1	12/10/2014	68	772	1	9.5	0	36.99

1 primi 2 H
92 1 12/11/2014 69 773 1 9.3 0 38.61
1 primi 2 H
92 1 12/12/2014 70 774 1 9.9 0 38.42
1 primi 2 H
92 1 12/13/2014 71 775 1 9.8 0 39.64
1 primi 2 H
92 1 12/14/2014 72 776 1 9.6 0 38.16
1 primi 2 H
92 1 12/15/2014 73 777 1 9.3 0 36.43
1 primi 2 H
92 1 12/16/2014 74 778 1 9.2 0 34.81
1 primi 2 H
92 1 12/17/2014 75 779 1 9.3 0 39.77
1 primi 2 H
92 1 12/18/2014 76 780 1 9.6 0 38.67
1 primi 2 H
92 1 12/19/2014 77 781 1 9.8 0 37.88
1 primi 2 H
92 1 12/20/2014 78 782 1 9.5 0.05 36.47
1 primi 2 H
92 1 12/21/2014 79 783 1 9.6 0 37.95
1 primi 2 H
92 1 12/22/2014 80 784 1 9.3 0 33.41
1 primi 2 H
92 1 12/23/2014 81 785 1 9.3 0 37.76
1 primi 2 H
92 1 12/24/2014 82 786 1 9.3 0 35.04
1 primi 2 H
92 1 12/25/2014 83 787 1 9.5 0 39.55
1 primi 2 H
92 1 12/26/2014 84 788 1 9.5 0.03 35.55
1 primi 2 H
92 1 12/27/2014 85 789 1 9.1 0 37.05
1 primi 2 H
92 1 12/28/2014 86 790 1 9.1 0 37.72
1 primi 2 H
92 1 12/29/2014 87 791 1 9.3 0.1 36.46
1 primi 2 H
92 1 12/30/2014 88 792 1 9.6 0 34.14
1 primi 2 H
92 1 12/31/2014 89 793 1 9.3 0 35.61
1 primi 2 H
92 1 01/01/2015 90 794 1 9.5 0 40.25
1 primi 2 H
92 1 01/02/2015 91 795 1 9.1 0 35.55
1 primi 2 H
92 1 01/03/2015 92 796 1 9.2 0 36.75
1 primi 2 H
92 1 01/04/2015 93 797 1 9.2 0 38.87
1 primi 2 H
92 1 01/05/2015 94 798 1 9.5 0 40.35
1 primi 2 H
92 1 01/06/2015 95 799 1 9.3 0 39.85

1 primi 2		H						
92	1	01/07/2015	96	800	1	9.5	0	41.99
1 primi 2		H						
92	1	01/08/2015	97	801	1	9.6	0	42.74
1 primi 2		H						
92	1	01/09/2015	98	802	1	9.3	0	36.34
1 primi 2		H						
92	1	01/10/2015	99	803	1	9.5	0	37.15
1 primi 2		H						
92	1	01/11/2015	100	804	1	9.2	0	39.63
1 primi 2		H						
92	1	01/12/2015	101	805	1	9.1	0	37.88
1 primi 2		H						
92	1	01/13/2015	102	806	1	9.3	0	41.95
1 primi 2		H						
92	1	01/14/2015	103	807	1	9.2	0.04	40.67
1 primi 2		H						
92	1	01/15/2015	104	808	1	8.9	0	36.77
1 primi 2		H						
92	1	01/16/2015	105	809	1	9.3	0	40.75
1 primi 2		H						
92	1	01/17/2015	106	810	1	9.6	0	39.15
1 primi 2		H						
92	1	01/18/2015	107	811	1	9.5	0	38.89
1 primi 2		H						
92	1	01/19/2015	108	812	1	9.8	0	40.54
1 primi 2		H						
92	1	01/20/2015	109	813	1	9.5	0	41.22
1 primi 2		H						
92	1	01/21/2015	110	814	1	9.6	0	39.42
1 primi 2		H						
92	1	01/22/2015	111	815	1	9.6	0	39.75
1 primi 2		H						
92	1	01/23/2015	112	816	1	9.6	0	38.36
1 primi 2		H						
92	1	01/24/2015	113	817	1	9.3	0	40
1 primi 2		H						
92	1	01/25/2015	114	818	1	9.5	0	39.74
1 primi 2		H						
92	1	01/26/2015	115	819	1	9.5	0	35.83
1 primi 2		H						
92	1	01/27/2015	116	820	1	9.5	0	37.65
1 primi 2		H						
92	1	01/28/2015	117	821	1	9.5	0	37.55
1 primi 2		H						
92	1	01/29/2015	118	822	1	9.3	0	36.57
1 primi 2		H						
92	1	01/30/2015	119	823	1	9.3	0	38.96
1 primi 2		H						
92	1	01/31/2015	120	824	1	9.5	0	36.34
1 primi 2		H						
92	1	02/01/2015	121	825	1	9.3	0	38.47
1 primi 2		H						
92	1	02/02/2015	122	826	1	9.6	0.05	38.94

1 primi 2 H
92 1 02/03/2015 123 827 1 9.6 0 40.53
1 primi 2 H
92 1 02/04/2015 124 828 1 9.5 0 38.38
1 primi 2 H
92 1 02/05/2015 125 829 1 9.5 0 38.86
1 primi 2 H
92 1 02/06/2015 126 830 1 9.6 0 40.1
1 primi 2 H
92 1 02/07/2015 127 831 1 9.5 0 41.76
1 primi 2 H
92 1 02/08/2015 128 832 1 9.2 0 37.37
1 primi 2 H
92 1 02/09/2015 129 833 1 9.6 0.33 39.4
1 primi 2 H
92 1 02/10/2015 130 834 1 9.2 0 36.35
1 primi 2 H
92 1 02/11/2015 131 835 1 9.2 0 39.68
1 primi 2 H
92 1 02/12/2015 132 836 1 9.5 0 38.16
1 primi 2 H
92 1 02/13/2015 133 837 1 9.5 0 38.89
1 primi 2 H
92 1 02/14/2015 134 838 1 9.5 0 39.27
1 primi 2 H
92 1 02/15/2015 135 839 1 9.6 0 43.68
1 primi 2 H
92 1 02/16/2015 136 840 1 9.8 0 40.42
1 primi 2 H
92 1 02/17/2015 137 841 1 9.6 0 40.61
1 primi 2 H
92 1 02/18/2015 138 842 1 9.5 0 39.01
1 primi 2 H
92 1 02/19/2015 139 843 1 9.3 0 38.86
1 primi 2 H
92 1 02/20/2015 140 844 1 9.3 0 37.6
1 primi 2 H
92 1 02/21/2015 141 845 1 9.6 0 41.93
1 primi 2 H
92 1 02/22/2015 142 846 1 9.6 0 39.69
1 primi 2 H
92 1 02/23/2015 143 847 1 9.5 0 39.59
1 primi 2 H
92 1 02/24/2015 144 848 1 9.8 0 39.2
1 primi 2 H
92 1 02/25/2015 145 849 1 9.6 0 37.93
1 primi 2 H
92 1 02/26/2015 146 850 1 9.5 0 40.48
1 primi 2 H
92 1 02/27/2015 147 851 1 9.5 0 39.66
1 primi 2 H
92 1 02/28/2015 148 852 1 9.5 0 38.66
1 primi 2 H
92 1 03/01/2015 149 853 1 9.6 0 39.8

1 primi 2 H
92 1 03/02/2015 150 854 1 9.6 0 41.22
1 primi 2 H
92 1 03/03/2015 151 855 1 9.5 0 39.58
1 primi 2 H
92 1 03/04/2015 152 856 1 9.5 0 41.04
1 primi 2 H
92 1 03/05/2015 153 857 1 9.8 0 39.51
1 primi 2 H
92 1 03/06/2015 154 858 1 9.6 0 39.49
1 primi 2 H
92 1 03/07/2015 155 859 1 9.6 0 42.72
1 primi 2 H
92 1 03/08/2015 156 860 1 9.2 0 37.74
1 primi 2 H
92 1 03/09/2015 157 861 1 9.5 0 38.26
1 primi 2 H
92 1 03/10/2015 158 862 1 9.3 0 36.39
1 primi 2 H
92 1 03/11/2015 159 863 1 10.2 0 44.46
1 primi 2 H
92 1 03/12/2015 160 864 1 9.8 0 41.1
1 primi 2 H
92 1 03/13/2015 161 865 1 10.2 0 38.1
1 primi 2 H
92 1 03/14/2015 162 866 1 9.5 0 40.8
1 primi 2 H
92 1 03/15/2015 163 867 1 9.3 0 35.98
1 primi 2 H
92 1 03/16/2015 164 868 1 9.9 0 40.29
1 primi 2 H
92 1 03/17/2015 165 869 1 9.8 0 40.22
1 primi 2 H
92 1 03/18/2015 166 870 1 9.8 0 41.34
1 primi 2 H
92 1 03/19/2015 167 871 1 9.5 0 36.61
1 primi 2 H
92 1 03/20/2015 168 872 1 9.9 0 39.9
1 primi 2 H
92 1 03/21/2015 169 873 1 9.6 0 40.78
1 primi 2 H
92 1 03/22/2015 170 874 1 9.3 0 36.8
1 primi 2 H
92 1 03/23/2015 171 875 1 9.6 0 38.05
1 primi 2 H
92 1 03/24/2015 172 876 1 9.5 0 39.95
1 primi 2 H
92 1 03/25/2015 173 877 1 9.3 0 38.51
1 primi 2 H
92 1 03/26/2015 174 878 1 9.6 0 37.86
1 primi 2 H
92 1 03/27/2015 175 879 1 9.8 0 43.01
1 primi 2 H
92 1 03/28/2015 176 880 1 9.5 0 39.51

1 primi 2 H
92 1 03/29/2015 177 881 1 9.6 0 36.58
1 primi 2 H
92 1 03/30/2015 178 882 1 9.8 0.04 40.21
1 primi 2 H
92 1 03/31/2015 179 883 1 9.8 0 41.1
1 primi 2 H
92 1 10/07/2015 6 1073 2 10.9 0.03 33.73
2 multi 1 H
92 1 10/08/2015 7 1074 2 10.6 0.05 30.88
2 multi 1 H
92 1 10/09/2015 8 1075 2 10.9 0.03 38.96
2 multi 1 H
92 1 10/10/2015 9 1076 2 11.7 0 35.49
2 multi 1 H
92 1 10/11/2015 10 1077 2 12.5 0.03 38.58
2 multi 1 H
92 1 10/12/2015 11 1078 2 10.6 0 35.95
2 multi 1 H
92 1 10/13/2015 12 1079 2 10.4 0.12 37.78
2 multi 1 H
92 1 10/14/2015 13 1080 2 10.6 0 26.76
2 multi 1 H
92 1 10/15/2015 14 1081 2 12.9 0.04 49.42
2 multi 1 H
92 1 10/16/2015 15 1082 2 10.9 0 37.92
2 multi 1 H
92 1 10/17/2015 16 1083 2 10.9 0 41.4
2 multi 1 H
92 1 10/18/2015 17 1084 2 11.1 0 41.86
2 multi 1 H
92 1 10/19/2015 18 1085 2 10.9 0 47.35
2 multi 1 H
92 1 10/20/2015 19 1086 2 10.4 0 46.43
2 multi 1 H
92 1 10/21/2015 20 1087 2 10.9 0 29.33
2 multi 1 H
92 1 10/22/2015 21 1088 2 10.4 0 41.93
2 multi 1 H
92 1 10/23/2015 22 1089 2 11.3 0 46.27
2 multi 2 H
92 1 10/24/2015 23 1090 2 10.9 0 44.69
2 multi 2 H
92 1 10/25/2015 24 1091 2 11.1 0 44.75
2 multi 2 H
92 1 10/26/2015 25 1092 2 11.1 0 45.29
2 multi 2 H
92 1 10/27/2015 26 1093 2 11.4 0 44.21
2 multi 2 H
92 1 10/28/2015 27 1094 2 11.4 0 45.83
2 multi 2 H
92 1 10/29/2015 28 1095 2 11.9 0.04 48.58
2 multi 2 H
92 1 10/30/2015 29 1096 2 11.3 0 47.46

2 multi 2	H
92	1	10/31/2015	30	1097	2	11.1	0	48.67
2 multi 2	H
92	1	11/01/2015	31	1098	2	10.3	0	48.7
2 multi 2	H
92	1	11/02/2015	32	1099	2	10.4	0	49.01
2 multi 2	H
92	1	11/03/2015	33	1100	2	9.8	0	47.86
2 multi 2	H
92	1	11/04/2015	34	1101	2	10.2	0	49.72
2 multi 2	H
92	1	11/05/2015	35	1102	2	10.3	0	49.12
2 multi 2	H
92	1	11/06/2015	36	1103	2	10.3	0	44.25
2 multi 2	H
92	1	11/07/2015	37	1104	2	9.9	0	44.37
2 multi 2	H
92	1	11/08/2015	38	1105	2	10	0	43.74
2 multi 2	H
92	1	11/09/2015	39	1106	2	10.3	0	44.12
2 multi 2	H
92	1	11/10/2015	40	1107	2	9.9	0	43.52
2 multi 2	H
92	1	11/11/2015	41	1108	2	10.3	0	41.27
2 multi 2	H
92	1	11/12/2015	42	1109	2	10.2	0	43.06
2 multi 2	H
92	1	11/13/2015	43	1110	2	10.3	0	40.55
2 multi 2	H
92	1	11/14/2015	44	1111	2	10.3	0	44.51
2 multi 2	H
92	1	11/15/2015	45	1112	2	10.2	0	44.46
2 multi 2	H
92	1	11/16/2015	46	1113	2	10.2	0	43.39
2 multi 2	H
92	1	11/17/2015	47	1114	2	9.9	0.03	46.51
2 multi 2	H
92	1	11/18/2015	48	1115	2	10.2	0	47.02
2 multi 2	H
92	1	11/19/2015	49	1116	2	9.9	0	45.43
2 multi 2	H
92	1	11/20/2015	50	1117	2	10.3	0	49.08
2 multi 2	H
92	1	11/21/2015	51	1118	2	10.2	0	46.79
2 multi 2	H
92	1	11/22/2015	52	1119	2	10.3	0	48.71
2 multi 2	H
92	1	11/23/2015	53	1120	2	10.3	0	45.93
2 multi 2	H
92	1	11/24/2015	54	1121	2	10	0	25.86
2 multi 2	H
92	1	11/25/2015	55	1122	2	10.3	0	49.07
2 multi 2	H
92	1	11/26/2015	56	1123	2	10.3	0	47.85

2 multi	2	H						
92	1	11/27/2015	57	1124	2	10.2	0	44.35
2 multi	2	H						
92	1	11/28/2015	58	1125	2	10.2	0	46.64
2 multi	2	H						
92	1	11/29/2015	59	1126	2	10.3	0	46.89
2 multi	2	H						
92	1	11/30/2015	60	1127	2	10.2	0	46.78
2 multi	2	H						
92	1	12/01/2015	61	1128	2	10.6	0	36.16
2 multi	2	H						
92	1	12/02/2015	62	1129	2	10	0	39.41
2 multi	2	H						
92	1	12/03/2015	63	1130	2	11.3	0	34.75
2 multi	2	H						
92	1	12/04/2015	64	1131	2	10	0	43.99
2 multi	2	H						
92	1	12/05/2015	65	1132	2	10.6	0	42.48
2 multi	2	H						
92	1	12/06/2015	66	1133	2	10.4	0	44.08
2 multi	2	H						
92	1	12/07/2015	67	1134	2	10.3	0	42.63
2 multi	2	H						
92	1	12/08/2015	68	1135	2	10.3	0	51.18
2 multi	2	H						
92	1	12/09/2015	69	1136	2	10	0.02	45.21
2 multi	2	H						
92	1	12/10/2015	70	1137	2	10	0	45.32
2 multi	2	H						
92	1	12/11/2015	71	1138	2	9.8	0	45.33
2 multi	2	H						
92	1	12/12/2015	72	1139	2	10	0	46.28
2 multi	2	H						
92	1	12/13/2015	73	1140	2	10.2	0	44.04
2 multi	2	H						
92	1	12/14/2015	74	1141	2	10.6	0	45.78
2 multi	2	H						
92	1	12/15/2015	75	1142	2	10.2	0	42.14
2 multi	2	H						
92	1	12/16/2015	76	1143	2	10.2	0	43.54
2 multi	2	H						
92	1	12/17/2015	77	1144	2	10	0	44.33
2 multi	2	H						
92	1	12/18/2015	78	1145	2	10.3	0	45.41
2 multi	2	H						
92	1	12/19/2015	79	1146	2	10.4	0	43.83
2 multi	2	H						
92	1	12/20/2015	80	1147	2	10.4	0	46.47
2 multi	2	H						
92	1	12/21/2015	81	1148	2	10.2	0	46.49
2 multi	2	H						
92	1	12/22/2015	82	1149	2	10	0	43.57
2 multi	2	H						
92	1	12/23/2015	83	1150	2	10.2	0	47.46

2 multi	2	H						
92	1	12/24/2015	84	1151	2	10.6	0	42.23
2 multi	2	H						
92	1	12/25/2015	85	1152	2	10.9	0	44.42
2 multi	2	H						
92	1	12/26/2015	86	1153	2	10.3	0	38.22
2 multi	2	H						
92	1	12/27/2015	87	1154	2	10	0	36.99
2 multi	2	H						
92	1	12/28/2015	88	1155	2	9.9	0	39
2 multi	2	H						
92	1	12/29/2015	89	1156	2	9.8	0	42.34
2 multi	2	H						
92	1	12/30/2015	90	1157	2	10.2	0	42.26
2 multi	2	H						
92	1	12/31/2015	91	1158	2	10	0	38.13
2 multi	2	H						
92	1	01/01/2016	92	1159	2	10.3	0	41.74
2 multi	2	H						
92	1	01/02/2016	93	1160	2	10.3	0	44.33
2 multi	2	H						
92	1	01/03/2016	94	1161	2	9.9	0	37.76
2 multi	2	H						
92	1	01/04/2016	95	1162	2	10	0	32.89
2 multi	2	H						
92	1	01/05/2016	96	1163	2	10.2	0	29.15
2 multi	2	H						
92	1	01/06/2016	97	1164	2	9.5	0	28.36
2 multi	2	H						
92	1	01/07/2016	98	1165	2	9.8	0	33.47
2 multi	2	H						
92	1	01/08/2016	99	1166	2	10.4	0	33.31
2 multi	2	H						
92	1	01/09/2016	100	1167	2	10.3	0	35.6
2 multi	2	H						
92	1	01/10/2016	101	1168	2	10.4	0	34.96
2 multi	2	H						
92	1	01/11/2016	102	1169	2	10.3	0	35.4
2 multi	2	H						
92	1	01/12/2016	103	1170	2	10.3	0	34.86
2 multi	2	H						
92	1	01/13/2016	104	1171	2	10.4	0	38.69
2 multi	2	H						
92	1	01/14/2016	105	1172	2	10.6	0	38.41
2 multi	2	H						
92	1	01/15/2016	106	1173	2	10.2	0	35.92
2 multi	2	H						
92	1	01/16/2016	107	1174	2	10.3	0	38.82
2 multi	2	H						
92	1	01/17/2016	108	1175	2	10	0	37.36
2 multi	2	H						
92	1	01/18/2016	109	1176	2	10.4	0	37.04
2 multi	2	H						
92	1	01/19/2016	110	1177	2	10.2	0	39.51

2 multi 2		H						
92	1	01/20/2016	111	1178	2	10.3	0	37.84
2 multi 2		H						
92	1	01/21/2016	112	1179	2	10.3	0	38.31
2 multi 2		H						
92	1	01/22/2016	113	1180	2	9.9	0	36.62
2 multi 2		H						
92	1	01/23/2016	114	1181	2	10.3	0	40.52
2 multi 2		H						
92	1	01/24/2016	115	1182	2	10.2	0	40.76
2 multi 2		H						
92	1	01/25/2016	116	1183	2	10.2	0	37.06
2 multi 2		H						
92	1	01/26/2016	117	1184	2	10.3	0	39.18
2 multi 2		H						
92	1	01/27/2016	118	1185	2	10.3	0	40.42
2 multi 2		H						
92	1	01/28/2016	119	1186	2	10.6	0	38.2
2 multi 2		H						
92	1	01/29/2016	120	1187	2	10.6	0	38.31
2 multi 2		H						
92	1	01/30/2016	121	1188	2	10	0	35.29
2 multi 2		H						
92	1	01/31/2016	122	1189	2	10.2	0	35.93
2 multi 2		H						
92	1	02/01/2016	123	1190	2	10.3	0	33.02
2 multi 2		H						
92	1	02/02/2016	124	1191	2	10.7	0	40.2
2 multi 2		H						
92	1	02/03/2016	125	1192	2	10.4	0	35.55
2 multi 2		H						
92	1	02/04/2016	126	1193	2	10.3	0	35.29
2 multi 2		H						
92	1	02/05/2016	127	1194	2	10.6	0	38.1
2 multi 2		H						
92	1	02/06/2016	128	1195	2	10.2	0	40.38
2 multi 2		H						
92	1	02/07/2016	129	1196	2	10.6	0	38.8
2 multi 2		H						
92	1	02/08/2016	130	1197	2	10.3	0	39.75
2 multi 2		H						
92	1	02/09/2016	131	1198	2	10.4	0	37.15
2 multi 2		H						
92	1	02/10/2016	132	1199	2	10.6	0	39.52
2 multi 2		H						
92	1	02/11/2016	133	1200	2	10	0	40.5
2 multi 2		H						
92	1	02/12/2016	134	1201	2	10.4	0	39.59
2 multi 2		H						
92	1	02/13/2016	135	1202	2	12.3	0	38.28
2 multi 2		H						
92	1	02/14/2016	136	1203	2	10.7	0	37.04
2 multi 2		H						
92	1	02/15/2016	137	1204	2	11.1	0	42.51

2 multi 2		H						
92	1	02/16/2016	138	1205	2	11.1	0	39.12
2 multi 2		H						
92	1	02/17/2016	139	1206	2	10.9	0	37.76
2 multi 2		H						
92	1	01/26/2017	117	1550	3	10	0	43.55
2 multi 2		H						
92	1	01/27/2017	118	1551	3	10.7	0	45.27
2 multi 2		H						
92	1	01/28/2017	119	1552	3	9.9	0	43.8
2 multi 2		H						
92	1	01/29/2017	120	1553	3	10.4	0	45.59
2 multi 2		H						
92	1	01/30/2017	121	1554	3	10.7	0	42.64
2 multi 2		H						
92	1	01/31/2017	122	1555	3	10.6	0	44.11
2 multi 2		H						
92	1	02/01/2017	123	1556	3	10.4	0	44.77
2 multi 2		H						
92	1	02/02/2017	124	1557	3	10.7	0	45.4
2 multi 2		H						
92	1	02/03/2017	125	1558	3	0	0	41.98
2 multi 2		H						
92	1	02/04/2017	126	1559	3	10.6	0	38.71
2 multi 2		H						
92	1	02/05/2017	127	1560	3	10.9	0	40.65
2 multi 2		H						
92	1	02/06/2017	128	1561	3	10.4	0	41.67
2 multi 2		H						
92	1	02/07/2017	129	1562	3	10.4	0	40.9
2 multi 2		H						
92	1	02/08/2017	130	1563	3	10.4	0	41.11
2 multi 2		H						
92	1	02/09/2017	131	1564	3	10.6	0	44.33
2 multi 2		H						
92	1	02/10/2017	132	1565	3	10.3	0	41.35
2 multi 2		H						
92	1	02/11/2017	133	1566	3	10.4	0	43.29
2 multi 2		H						
92	1	02/12/2017	134	1567	3	10.3	0	40.34
2 multi 2		H						
92	1	02/13/2017	135	1568	3	10	0	40.69
2 multi 2		H						
92	1	02/14/2017	136	1569	3	10.6	0	44.01
2 multi 2		H						
92	1	02/15/2017	137	1570	3	10.4	0.13	39.89
2 multi 2		H						
92	1	02/16/2017	138	1571	3	10.3	0	42.59
2 multi 2		H						
92	1	02/17/2017	139	1572	3	10.3	0.26	42.6
2 multi 2		H						
92	1	02/18/2017	140	1573	3	10.4	0	40.27
2 multi 2		H						
92	1	02/19/2017	141	1574	3	10.3	0	41

2 multi	2	H						
92	1	02/20/2017	142	1575	3	10.4	0	45.02
2 multi	2	H						
92	1	02/21/2017	143	1576	3	10.3	0	40.42
2 multi	2	H						
92	1	02/22/2017	144	1577	3	10.4	0	45.65
2 multi	2	H						
92	1	02/23/2017	145	1578	3	10.7	0	41.93
2 multi	2	H						
92	1	02/24/2017	146	1579	3	10.4	0	40.24
2 multi	2	H						
92	1	02/25/2017	147	1580	3	10.2	0.12	43.55
2 multi	2	H						
92	1	02/26/2017	148	1581	3	10	0	41.26
2 multi	2	H						
92	1	02/27/2017	149	1582	3	10.4	0	44.95
2 multi	2	H						
92	1	02/28/2017	150	1583	3	10.7	0	43.56
2 multi	2	H						
92	1	03/01/2017	151	1584	3	10.7	0	40.51
2 multi	2	H						
92	1	03/02/2017	152	1585	3	10.9	0	42.93
2 multi	2	H						
92	1	03/03/2017	153	1586	3	10.7	0.15	38.45
2 multi	2	H						
92	1	03/04/2017	154	1587	3	10.7	0	42.15
2 multi	2	H						
92	1	03/05/2017	155	1588	3	10.9	0	38.79
2 multi	2	H						
92	1	03/06/2017	156	1589	3	10.2	0	40.62
2 multi	2	H						
92	1	03/07/2017	157	1590	3	10.6	0	41.71
2 multi	2	H						
92	1	03/08/2017	158	1591	3	10	0	41.21
2 multi	2	H						
92	1	03/09/2017	159	1592	3	10.3	0	41.84
2 multi	2	H						
92	1	03/10/2017	160	1593	3	9.8	0	41.52
2 multi	2	H						
92	1	03/11/2017	161	1594	3	10.6	0	43.47
2 multi	2	H						
92	1	03/12/2017	162	1595	3	10	0	37.44
2 multi	2	H						
92	1	03/13/2017	163	1596	3	10.6	0	41.49
2 multi	2	H						
92	1	03/14/2017	164	1597	3	10.7	0	40.78
2 multi	2	H						
92	1	03/15/2017	165	1598	3	10.3	0	41.54
2 multi	2	H						
92	1	03/16/2017	166	1599	3	10.3	0	43.44
2 multi	2	H						
92	1	03/17/2017	167	1600	3	10.6	0	40.81
2 multi	2	H						
92	1	03/18/2017	168	1601	3	10.6	0	42.35

2 multi 2		H						
92	1	03/19/2017	169	1602	3	10.2	0	35.72
2 multi 2		H						
92	1	03/20/2017	170	1603	3	9.8	0	41.05
2 multi 2		H						
92	1	03/21/2017	171	1604	3	10.2	0	44.85
2 multi 2		H						
92	1	03/22/2017	172	1605	3	10.4	0	41.31
2 multi 2		H						
92	1	03/23/2017	173	1606	3	10.3	0	39.07
2 multi 2		H						
92	1	03/24/2017	174	1607	3	9.9	0	42.16
2 multi 2		H						
92	1	03/25/2017	175	1608	3	10.2	0	40.74
2 multi 2		H						
92	1	03/26/2017	176	1609	3	9.6	0	39.96
2 multi 2		H						
92	1	03/27/2017	177	1610	3	9.6	0.37	37.9
2 multi 2		H						
92	1	03/28/2017	178	1611	3	10.3	0.82	39.01
2 multi 2		H						
92	1	03/29/2017	179	1612	3	10.4	0.08	39.53
2 multi 2		H						
92	1	03/30/2017	180	1613	3	10.4	0	37.53
2 multi 2		H						
92	1	03/31/2017	181	1614	3	10.6	0	40.38
2 multi 2		H						
92	1	04/01/2017	182	1615	3	11.1	0	38.92
2 multi 2		H						
92	1	04/02/2017	183	1616	3	10.4	0	38.1
2 multi 2		H						
92	1	04/03/2017	184	1617	3	10.7	0	40.98
2 multi 2		H						
92	1	04/04/2017	185	1618	3	10.7	0	41.37
2 multi 2		H						
92	1	04/05/2017	186	1619	3	10.6	0	39.78
2 multi 2		H						
92	1	04/06/2017	187	1620	3	10.7	0.26	39.23
2 multi 2		H						
92	1	04/07/2017	188	1621	3	10.6	0	39.99
2 multi 2		H						
92	1	04/08/2017	189	1622	3	9.9	0	40.08
2 multi 2		H						
92	1	04/09/2017	190	1623	3	10.3	0	39.04
2 multi 2		H						
92	1	04/10/2017	191	1624	3	11.3	0	37.35
2 multi 2		H						
92	1	04/11/2017	192	1625	3	10.9	0	39.88
2 multi 2		H						
92	1	04/12/2017	193	1626	3	10.7	0	37.48
2 multi 2		H						
92	1	04/13/2017	194	1627	3	9.9	0	42.15
2 multi 2		H						
92	1	04/14/2017	195	1628	3	11.4	0	35.44

2 multi	2	H						
92	1	04/15/2017	196	1629	3	10.6	0.68	39.75
2 multi	2	H						
92	1	04/16/2017	197	1630	3	10.7	0	39.36
2 multi	2	H						
92	1	04/17/2017	198	1631	3	10.7	0	39.62
2 multi	2	H						
92	1	04/18/2017	199	1632	3	10.7	0	42.2
2 multi	2	H						
92	1	04/19/2017	200	1633	3	10.4	0	37.97
2 multi	2	H						
92	1	04/20/2017	201	1634	3	10.3	0	37.09
2 multi	2	H						
92	1	04/21/2017	202	1635	3	10.4	0	35.46
2 multi	2	H						
92	1	04/22/2017	203	1636	3	10.9	0	37.07
2 multi	2	H						
92	1	04/23/2017	204	1637	3	10.2	0	39.28
2 multi	2	H						
92	1	04/24/2017	205	1638	3	10.6	0	36.56
2 multi	2	H						
92	1	04/25/2017	206	1639	3	10.7	0	38.51
2 multi	2	H						
92	1	04/26/2017	207	1640	3	10.7	0	37.84
2 multi	2	H						
92	1	04/27/2017	208	1641	3	10.4	0	38.38
2 multi	2	H						
92	1	04/28/2017	209	1642	3	11.1	0	39.73
2 multi	2	H						
92	1	04/29/2017	210	1643	3	10.7	0	40.5
2 multi	2	H						
92	1	04/30/2017	211	1644	3	11.3	0	38.06
2 multi	2	H						
92	1	05/01/2017	212	1645	3	10.4	0	41.18
2 multi	2	H						
92	1	05/02/2017	213	1646	3	10.7	0	39.89
2 multi	2	H						
92	1	05/03/2017	214	1647	3	10.4	0	37.73
2 multi	2	H						
92	1	05/04/2017	215	1648	3	10.6	0	40.38
2 multi	2	H						
92	1	05/05/2017	216	1649	3	10.9	0	40.64
2 multi	2	H						
92	1	05/06/2017	217	1650	3	11.1	0	37.23
2 multi	2	H						
92	1	05/07/2017	218	1651	3	10.7	0	36.52
2 multi	2	H						
92	1	05/08/2017	219	1652	3	11.1	0	38.06
2 multi	2	H						
92	1	05/09/2017	220	1653	3	10.4	0	40
2 multi	2	H						
92	1	05/10/2017	221	1654	3	10.6	0	39.74
2 multi	2	H						
92	1	05/11/2017	222	1655	3	10.7	0.08	37.08

2 multi	2	H						
92	1	05/12/2017	223	1656	3	11.1	0	39.15
2 multi	2	H						
92	1	05/13/2017	224	1657	3	10.9	0	39.05
2 multi	2	H						
92	1	05/14/2017	225	1658	3	10.7	0	39.8
2 multi	2	H						
92	1	05/15/2017	226	1659	3	10.4	0	39.23
2 multi	2	H						
92	1	05/16/2017	227	1660	3	10.6	0	37.93
2 multi	2	H						
92	1	05/17/2017	228	1661	3	10.6	0	41.41
2 multi	2	H						
92	1	10/14/2018	6	2176	5	10.2	0.12	29.34
2 multi	1	H						
92	1	10/15/2018	7	2177	5	10.2	0.09	36.34
2 multi	1	H						
92	1	10/16/2018	8	2178	5	10.4	0	37.41
2 multi	1	H						
92	1	10/17/2018	9	2179	5	10.3	0	39.12
2 multi	1	H						
92	1	10/18/2018	10	2180	5	10.6	0	36.79
2 multi	1	H						
92	1	10/19/2018	11	2181	5	10.2	0	41.45
2 multi	1	H						
92	1	10/20/2018	12	2182	5	10.3	0	40.02
2 multi	1	H						
92	1	10/21/2018	13	2183	5	10	0	39.32
2 multi	1	H						
92	1	10/22/2018	14	2184	5	10.3	0	39.28
2 multi	1	H						
92	1	10/23/2018	15	2185	5	10.4	0	38.65
2 multi	1	H						
92	1	10/24/2018	16	2186	5	10.4	0	37.47
2 multi	1	H						
92	1	10/25/2018	17	2187	5	10	0.01	41.56
2 multi	1	H						
92	1	10/26/2018	18	2188	5	10.2	0	41.66
2 multi	1	H						
92	1	10/27/2018	19	2189	5	10.2	0.06	41.14
2 multi	1	H						
92	1	10/28/2018	20	2190	5	9.8	0	41.18
2 multi	1	H						
92	1	10/29/2018	21	2191	5	9.8	0	41.83
2 multi	1	H						
92	1	10/30/2018	22	2192	5	10.2	0	38.29
2 multi	2	H						
92	1	10/31/2018	23	2193	5	10.3	0	46.16
2 multi	2	H						
92	1	11/01/2018	24	2194	5	10.2	0	44.33
2 multi	2	H						
92	1	11/02/2018	25	2195	5	10.2	0	43.51
2 multi	2	H						
92	1	11/03/2018	26	2196	5	10.3	0.12	42.06

2 multi 2		H						
92	1	11/04/2018	27	2197	5	9.9	0.02	44.35
2 multi 2		H						
92	1	11/05/2018	28	2198	5	9.8	0.24	40.65
2 multi 2		H						
92	1	11/06/2018	29	2199	5	10.2	0	41.98
2 multi 2		H						
92	1	11/07/2018	30	2200	5	10.2	0	40.63
2 multi 2		H						
92	1	11/08/2018	31	2201	5	10	0	44.36
2 multi 2		H						
92	1	11/09/2018	32	2202	5	10	0	41.98
2 multi 2		H						
92	1	11/10/2018	33	2203	5	10	0	40.15
2 multi 2		H						
92	1	11/11/2018	34	2204	5	10	0	42.84
2 multi 2		H						
92	1	11/12/2018	35	2205	5	10	0	44.68
2 multi 2		H						
92	1	11/13/2018	36	2206	5	10	0	41.66
2 multi 2		H						
92	1	11/14/2018	37	2207	5	10	0	43.72
2 multi 2		H						
92	1	11/15/2018	38	2208	5	10	0.01	42.25
2 multi 2		H						
92	1	11/16/2018	39	2209	5	9.8	0	39.58
2 multi 2		H						
92	1	11/17/2018	40	2210	5	10.2	0	37
2 multi 2		H						
92	1	11/18/2018	41	2211	5	10.3	0	39.61
2 multi 2		H						
92	1	11/19/2018	42	2212	5	10.3	0	40.58
2 multi 2		H						
92	1	11/20/2018	43	2213	5	10	0	40.33
2 multi 2		H						
92	1	11/21/2018	44	2214	5	10	0	41.27
2 multi 2		H						
92	1	11/22/2018	45	2215	5	10	0	41.94
2 multi 2		H						
92	1	11/23/2018	46	2216	5	10.2	0	42.84
2 multi 2		H						
92	1	11/24/2018	47	2217	5	9.9	0	42.82
2 multi 2		H						
92	1	11/25/2018	48	2218	5	9.8	0	40.42
2 multi 2		H						
92	1	11/26/2018	49	2219	5	10.2	0	43.53
2 multi 2		H						
92	1	11/27/2018	50	2220	5	9.9	0	39.11
2 multi 2		H						
92	1	11/28/2018	51	2221	5	9.9	0	42.88
2 multi 2		H						
92	1	11/29/2018	52	2222	5	9.9	0	38.06
2 multi 2		H						
92	1	11/30/2018	53	2223	5	9.6	0	42.74

2 multi	2	H						
92	1	12/01/2018	54	2224	5	10.2	0	41.11
2 multi	2	H						
92	1	12/02/2018	55	2225	5	10	0.01	39.92
2 multi	2	H						
92	1	12/03/2018	56	2226	5	10	0	43.34
2 multi	2	H						
92	1	12/04/2018	57	2227	5	10.2	0	39.43
2 multi	2	H						
92	1	12/05/2018	58	2228	5	9.9	0	41.94
2 multi	2	H						
92	1	12/06/2018	59	2229	5	9.9	0.01	41.27
2 multi	2	H						
92	1	12/07/2018	60	2230	5	10.2	0	41.46
2 multi	2	H						
92	1	12/08/2018	61	2231	5	10.2	0	35.6
2 multi	2	H						
92	1	12/09/2018	62	2232	5	9.9	0	48.24
2 multi	2	H						
92	1	12/10/2018	63	2233	5	10	0	43.82
2 multi	2	H						
92	1	12/11/2018	64	2234	5	10	0.21	32.99
2 multi	2	H						
92	1	12/12/2018	65	2235	5	9.6	0	42.44
2 multi	2	H						
92	1	12/13/2018	66	2236	5	9.9	0	38.37
2 multi	2	H						
92	1	12/14/2018	67	2237	5	9.8	0	40.58
2 multi	2	H						
92	1	12/15/2018	68	2238	5	10.3	0	39.77
2 multi	2	H						
92	1	12/16/2018	69	2239	5	10.2	0.01	39.75
2 multi	2	H						
92	1	12/17/2018	70	2240	5	10.2	0	38.87
2 multi	2	H						
92	1	12/18/2018	71	2241	5	10.2	0	42.03
2 multi	2	H						
92	1	12/19/2018	72	2242	5	10	0	39.73
2 multi	2	H						
92	1	12/20/2018	73	2243	5	10	0	40.18
2 multi	2	H						
92	1	12/21/2018	74	2244	5	10.3	0	38.79
2 multi	2	H						
92	1	12/22/2018	75	2245	5	9.8	0	35.53
2 multi	2	H						
92	1	12/23/2018	76	2246	5	10	0	36.03
2 multi	2	H						
92	1	12/24/2018	77	2247	5	10.2	0	38.22
2 multi	2	H						
92	1	01/05/2020	7	2624	6	10.2	0.01	39.84
2 multi	1	H						
92	1	01/06/2020	8	2625	6	10	0.04	38.26
2 multi	1	H						
92	1	01/07/2020	9	2626	6	10	0	38.59

2 multi	1	H						
92	1	01/08/2020	10	2627	6	10	0.05	31.24
2 multi	1	H						
92	1	01/09/2020	11	2628	6	10.4	0	38.42
2 multi	1	H						
92	1	01/10/2020	12	2629	6	10.2	0	39.02
2 multi	1	H						
92	1	01/11/2020	13	2630	6	10.3	0	42.79
2 multi	1	H						
92	1	01/12/2020	14	2631	6	10.4	0.01	43.71
2 multi	1	H						
92	1	01/13/2020	15	2632	6	10.3	0	43.94
2 multi	1	H						
92	1	01/14/2020	16	2633	6	10.2	0	41.78
2 multi	1	H						
92	1	01/15/2020	17	2634	6	10.2	0	45.29
2 multi	1	H						
92	1	01/16/2020	18	2635	6	10	0	41.06
2 multi	1	H						
92	1	01/17/2020	19	2636	6	10	0	43.87
2 multi	1	H						
92	1	01/18/2020	20	2637	6	10.2	0	45.36
2 multi	1	H						
92	1	01/19/2020	21	2638	6	9.9	0.08	45.69
2 multi	1	H						
92	1	01/20/2020	22	2639	6	9.8	0	42.32
2 multi	2	H						
92	1	01/21/2020	23	2640	6	9.9	0	48.76
2 multi	2	H						
92	1	01/22/2020	24	2641	6	9.9	0	47.15
2 multi	2	H						
92	1	01/23/2020	25	2642	6	10	0	47.56
2 multi	2	H						
92	1	01/24/2020	26	2643	6	9.9	0.05	47.9
2 multi	2	H						
92	1	01/25/2020	27	2644	6	9.8	0	49.33
2 multi	2	H						
92	1	01/26/2020	28	2645	6	9.8	0	45.79
2 multi	2	H						
92	1	01/27/2020	29	2646	6	9.8	0	47.38
2 multi	2	H						
92	1	01/28/2020	30	2647	6	10.2	0	43.48
2 multi	2	H						
92	1	01/29/2020	31	2648	6	9.5	0	51.91
2 multi	2	H						
92	1	01/30/2020	32	2649	6	10	0	43.77
2 multi	2	H						
92	1	01/31/2020	33	2650	6	10	0.03	47.19
2 multi	2	H						
92	1	02/01/2020	34	2651	6	9.9	0	49.72
2 multi	2	H						
92	1	02/02/2020	35	2652	6	9.9	0	49.1
2 multi	2	H						
92	1	02/03/2020	36	2653	6	10	0	49.28

2 multi	2	H						
92	1	02/04/2020	37	2654	6	10	0	49.46
2 multi	2	H						
92	1	02/05/2020	38	2655	6	11.7	0	46.34
2 multi	2	H						
92	1	02/06/2020	39	2656	6	10	0.12	52.22
2 multi	2	H						
92	1	02/07/2020	40	2657	6	10.3	0	51.08
2 multi	2	H						
92	1	02/08/2020	41	2658	6	10.4	0.01	46.2
2 multi	2	H						
92	1	02/09/2020	42	2659	6	9.6	0.03	46.57
2 multi	2	H						
92	1	02/10/2020	43	2660	6	10	0	49.42
2 multi	2	H						
92	1	02/11/2020	44	2661	6	9.8	0	43.91
2 multi	2	H						
92	1	02/12/2020	45	2662	6	9.9	0	46.61
2 multi	2	H						
92	1	02/13/2020	46	2663	6	9.8	0	47.99
2 multi	2	H						
92	1	02/14/2020	47	2664	6	9.5	0	45.06
2 multi	2	H						
92	1	02/15/2020	48	2665	6	9.8	0.42	45.83
2 multi	2	H						
92	1	02/16/2020	49	2666	6	10	0	50.7
2 multi	2	H						
92	1	02/17/2020	50	2667	6	9.9	0	47.5
2 multi	2	H						
92	1	02/18/2020	51	2668	6	9.9	0.06	51.89
2 multi	2	H						
92	1	02/19/2020	52	2669	6	10.2	0.63	46.29
2 multi	2	H						
92	1	02/20/2020	53	2670	6	10.2	0	47.84
2 multi	2	H						
92	1	02/21/2020	54	2671	6	9.9	0	49.27
2 multi	2	H						
92	1	02/22/2020	55	2672	6	10	0	46.64
2 multi	2	H						
92	1	02/23/2020	56	2673	6	10.2	0	48.95
2 multi	2	H						
92	1	02/24/2020	57	2674	6	10.3	0	45.27
2 multi	2	H						
92	1	02/25/2020	58	2675	6	10	0	48.26
2 multi	2	H						
92	1	02/26/2020	59	2676	6	10	0.08	48.96
2 multi	2	H						
92	1	02/27/2020	60	2677	6	10.3	0	49.67
2 multi	2	H						
92	1	02/28/2020	61	2678	6	10.2	0	45.58
2 multi	2	H						
92	1	02/29/2020	62	2679	6	10	0.32	48.98
2 multi	2	H						
92	1	03/01/2020	63	2680	6	10.6	0	46.64

2 multi	2	H						
92	1	03/02/2020	64	2681	6	10.3	0	47.47
2 multi	2	H						
92	1	03/03/2020	65	2682	6	10.3	0	51.33
2 multi	2	H						
92	1	03/04/2020	66	2683	6	10.2	0	47.54
2 multi	2	H						
92	1	03/05/2020	67	2684	6	10.4	0.02	48.44
2 multi	2	H						
92	1	03/06/2020	68	2685	6	10.2	0	47.1
2 multi	2	H						
92	1	03/07/2020	69	2686	6	10.2	0	50.09
2 multi	2	H						
92	1	03/08/2020	70	2687	6	10.4	0	46.38
2 multi	2	H						
92	1	03/09/2020	71	2688	6	10.2	0	48.21
2 multi	2	H						
92	1	03/10/2020	72	2689	6	10.2	0	46.65
2 multi	2	H						
92	1	03/11/2020	73	2690	6	10.3	0	48.6
2 multi	2	H						
92	1	03/12/2020	74	2691	6	10.3	0	43.82
2 multi	2	H						
92	1	03/13/2020	75	2692	6	10	0	47.37
2 multi	2	H						
92	1	03/14/2020	76	2693	6	10.4	0	47.07
2 multi	2	H						
92	1	03/15/2020	77	2694	6	10.2	0	47.46
2 multi	2	H						
92	1	03/16/2020	78	2695	6	10	0	47.8
2 multi	2	H						
92	1	03/17/2020	79	2696	6	10.3	0	46.1
2 multi	2	H						
92	1	03/18/2020	80	2697	6	10.2	0	47.09
2 multi	2	H						
92	1	03/19/2020	81	2698	6	10.2	0.15	45.38
2 multi	2	H						
92	1	03/20/2020	82	2699	6	10.3	0	46.58
2 multi	2	H						
92	1	03/21/2020	83	2700	6	10.4	0	48.29
2 multi	2	H						
92	1	03/22/2020	84	2701	6	10.2	0	44.87
2 multi	2	H						
92	1	03/23/2020	85	2702	6	10.3	0	47.67
2 multi	2	H						
92	1	03/24/2020	86	2703	6	10.4	0	49.09
2 multi	2	H						
92	1	03/25/2020	87	2704	6	10.6	0	47.54
2 multi	2	H						
92	1	03/26/2020	88	2705	6	10.3	0	43.82
2 multi	2	H						
92	1	03/27/2020	89	2706	6	10.2	0	49.98
2 multi	2	H						
92	1	03/28/2020	90	2707	6	10.4	0.45	44.64

2 multi	2	H						
92	1	03/29/2020	91	2708	6	10.3	0	42.5
2 multi	2	H						
92	1	03/30/2020	92	2709	6	10.4	0	42.15
2 multi	2	H						
92	1	03/31/2020	93	2710	6	10.3	0	45.11
2 multi	2	H						
92	1	04/01/2020	94	2711	6	10	0	44.99
2 multi	2	H						
92	1	04/02/2020	95	2712	6	10.4	0	46.03
2 multi	2	H						
92	1	04/03/2020	96	2713	6	10.6	0	44.15
2 multi	2	H						
92	1	04/04/2020	97	2714	6	10.4	0	46.06
2 multi	2	H						
92	1	11/18/2020	3	2942	7	10.3	0.41	30.09
2 multi	1	H						
92	1	11/19/2020	4	2943	7	11.1	0.12	33.51
2 multi	1	H						
92	1	11/20/2020	5	2944	7	10.4	0.45	37.84
2 multi	1	H						
92	1	11/21/2020	6	2945	7	10.4	0	40.32
2 multi	1	H						
92	1	11/22/2020	7	2946	7	10.4	0.05	41.92
2 multi	1	H						
92	1	11/23/2020	8	2947	7	10.6	0.06	44.35
2 multi	1	H						
92	1	11/24/2020	9	2948	7	10.3	0.05	42.22
2 multi	1	H						
92	1	11/25/2020	10	2949	7	10.6	0.05	42.46
2 multi	1	H						
92	1	11/26/2020	11	2950	7	10.2	0.47	45.88
2 multi	1	H						
92	1	11/27/2020	12	2951	7	10.2	0.04	45.46
2 multi	1	H						
92	1	11/28/2020	13	2952	7	10	0.05	46.29
2 multi	1	H						
92	1	11/29/2020	14	2953	7	10.3	0.01	44.52
2 multi	1	H						
92	1	11/30/2020	15	2954	7	10.2	0	45.08
2 multi	1	H						
92	1	12/01/2020	16	2955	7	10	0.03	49.7
2 multi	1	H						
92	1	12/02/2020	17	2956	7	10.2	0.11	49.8
2 multi	1	H						
92	1	12/03/2020	18	2957	7	10.2	0	47.81
2 multi	1	H						
92	1	12/04/2020	19	2958	7	10.2	0.05	50.84
2 multi	1	H						
92	1	12/05/2020	20	2959	7	10.2	0	49.37
2 multi	1	H						
92	1	12/06/2020	21	2960	7	10.2	0.08	49.79
2 multi	1	H						
92	1	12/07/2020	22	2961	7	10	0.04	52.1

2 multi	2	H						
92	1	12/08/2020	23	2962	7	10.3	0.03	49.94
2 multi	2	H						
92	1	12/09/2020	24	2963	7	10.4	0	46.56
2 multi	2	H						
92	1	12/10/2020	25	2964	7	10.3	0.11	46.27
2 multi	2	H						
92	1	12/11/2020	26	2965	7	10.3	0	50.57
2 multi	2	H						
92	1	12/12/2020	27	2966	7	10.3	0.03	49.32
2 multi	2	H						
92	1	12/13/2020	28	2967	7	10.4	0.07	49.15
2 multi	2	H						
92	1	12/14/2020	29	2968	7	10.2	0.05	46.06
2 multi	2	H						
92	1	12/15/2020	30	2969	7	10.2	0	48.83
2 multi	2	H						
92	1	12/16/2020	31	2970	7	10	0	49.15
2 multi	2	H						
92	1	12/17/2020	32	2971	7	10.2	0	41.07
2 multi	2	H						
92	1	12/18/2020	33	2972	7	10.6	0	48.03
2 multi	2	H						
92	1	12/19/2020	34	2973	7	10.3	0.06	46.43
2 multi	2	H						
92	1	12/20/2020	35	2974	7	9.9	0.01	43.59
2 multi	2	H						
92	1	12/21/2020	36	2975	7	10.3	0.02	39.13
2 multi	2	H						
92	1	12/22/2020	37	2976	7	10.3	0.02	46.09
2 multi	2	H						
92	1	12/23/2020	38	2977	7	10.4	0.03	41.42
2 multi	2	H						
92	1	12/24/2020	39	2978	7	10.2	0.08	42.28
2 multi	2	H						
92	1	12/25/2020	40	2979	7	10.3	0.02	43.4
2 multi	2	H						
92	1	12/26/2020	41	2980	7	10.3	0.01	46.01
2 multi	2	H						
92	1	12/27/2020	42	2981	7	10.4	0.01	45.2
2 multi	2	H						
92	1	12/28/2020	43	2982	7	10.2	0	45.55
2 multi	2	H						
92	1	12/29/2020	44	2983	7	10.6	0.06	47.77
2 multi	2	H						
92	1	12/30/2020	45	2984	7	10.4	0	45.33
2 multi	2	H						
92	1	12/31/2020	46	2985	7	10.2	0.05	44.96
2 multi	2	H						
92	1	01/01/2021	47	2986	7	10.2	0.55	50.31
2 multi	2	H						
92	1	01/02/2021	48	2987	7	10	0.03	49
2 multi	2	H						
92	1	01/03/2021	49	2988	7	10.2	0	47.78

2 multi	2	H						
92	1	01/04/2021	50	2989	7	10	0.04	46.21
2 multi	2	H						
92	1	01/05/2021	51	2990	7	9.9	0.23	47.73
2 multi	2	H						
92	1	01/06/2021	52	2991	7	9.8	0.3	42.04
2 multi	2	H						
92	1	01/07/2021	53	2992	7	10	0.4	45.55
2 multi	2	H						
92	1	01/08/2021	54	2993	7	9.6	0.03	41.15
2 multi	2	H						
92	1	01/09/2021	55	2994	7	10	0	43.87
2 multi	2	H						
92	1	01/10/2021	56	2995	7	10	0	42.18
2 multi	2	H						
92	1	01/11/2021	57	2996	7	9.8	0.03	41.12
2 multi	2	H						
92	1	01/12/2021	58	2997	7	9.9	0	43.39
2 multi	2	H						
92	1	01/13/2021	59	2998	7	9.8	0.24	41.35
2 multi	2	H						
92	1	01/14/2021	60	2999	7	10.3	0.02	46.86
2 multi	2	H						
92	1	01/15/2021	61	3000	7	10.3	0	39.73
2 multi	2	H						
92	1	01/16/2021	62	3001	7	9.9	0.05	47.29
2 multi	2	H						
92	1	01/17/2021	63	3002	7	9.9	0	43.8
2 multi	2	H						
92	1	01/18/2021	64	3003	7	10.3	0.1	46.41
2 multi	2	H						
92	1	01/19/2021	65	3004	7	10.3	0.02	43.57
2 multi	2	H						
92	1	01/20/2021	66	3005	7	10.2	0.02	43.1
2 multi	2	H						
92	1	01/21/2021	67	3006	7	10.2	0	42.4
2 multi	2	H						
92	1	01/22/2021	68	3007	7	10	0.01	38.39
2 multi	2	H						
92	1	01/23/2021	69	3008	7	10	0	35.43
2 multi	2	H						
92	1	01/24/2021	70	3009	7	10	0.01	37.67
2 multi	2	H						
92	1	01/25/2021	71	3010	7	10.2	0.01	42.77
2 multi	2	H						
92	1	01/26/2021	72	3011	7	10	0.01	42.22
2 multi	2	H						
92	1	01/27/2021	73	3012	7	10.3	0.05	40.43
2 multi	2	H						
92	1	01/28/2021	74	3013	7	10.3	0	42.43
2 multi	2	H						
92	1	01/29/2021	75	3014	7	10	0	39.12
2 multi	2	H						
92	1	01/30/2021	76	3015	7	10	0.01	39.72

2 multi	2	H						
92	1	01/31/2021	77	3016	7	10.2	0.02	39.2
2 multi	2	H						
92	1	02/01/2021	78	3017	7	10	0.46	39.28
2 multi	2	H						
92	1	02/02/2021	79	3018	7	9.8	0	40.84
2 multi	2	H						
92	1	02/03/2021	80	3019	7	10.3	0.07	40.09
2 multi	2	H						
92	1	02/04/2021	81	3020	7	10.2	0.02	40.01
2 multi	2	H						
92	1	02/05/2021	82	3021	7	10.3	0.06	48.12
2 multi	2	H						
92	1	02/06/2021	83	3022	7	10.4	0.01	42.69
2 multi	2	H						
92	1	02/07/2021	84	3023	7	10.4	0.01	42.3
2 multi	2	H						
92	1	02/08/2021	85	3024	7	10.3	0.02	40.55
2 multi	2	H						
92	1	02/09/2021	86	3025	7	10	0	43.15
2 multi	2	H						
92	1	02/10/2021	87	3026	7	10.3	0.23	40.94
2 multi	2	H						
92	1	02/11/2021	88	3027	7	10.3	0	42.94
2 multi	2	H						
92	1	02/12/2021	89	3028	7	10.2	0	41.23
2 multi	2	H						
92	1	02/13/2021	90	3029	7	10.2	0.15	42.58
2 multi	2	H						
92	1	02/14/2021	91	3030	7	9.9	0.05	47.99
2 multi	2	H						
92	1	02/15/2021	92	3031	7	10.6	0	39.61
2 multi	2	H						
92	1	02/16/2021	93	3032	7	10.2	0.07	42.32
2 multi	2	H						
92	1	02/17/2021	94	3033	7	10.3	0.05	44.85
2 multi	2	H						
92	1	02/18/2021	95	3034	7	10.2	0.03	41.75
2 multi	2	H						
92	1	02/19/2021	96	3035	7	10.3	0.03	43.23
2 multi	2	H						
92	1	02/20/2021	97	3036	7	10.4	0.03	43.2
2 multi	2	H						
92	1	02/21/2021	98	3037	7	10.2	0.37	42.96
2 multi	2	H						
92	1	02/22/2021	99	3038	7	10.3	0.03	40.91
2 multi	2	H						
92	1	02/23/2021	100	3039	7	10.3	0	42.93
2 multi	2	H						
92	1	02/24/2021	101	3040	7	10.3	0.27	45.15
2 multi	2	H						
92	1	02/25/2021	102	3041	7	10.3	0	38.9
2 multi	2	H						
92	1	02/26/2021	103	3042	7	10.3	0	45.48

2 multi	2	H						
92	1	02/27/2021	104	3043	7	10.3	0.02	42.79
2 multi	2	H						
92	1	02/28/2021	105	3044	7	10	0.02	46.42
2 multi	2	H						
92	1	03/01/2021	106	3045	7	10.7	0.09	34.53
2 multi	2	H						
92	1	03/02/2021	107	3046	7	10.3	0.02	43.22
2 multi	2	H						
92	1	03/03/2021	108	3047	7	10.3	0.01	37.51
2 multi	2	H						
92	1	03/04/2021	109	3048	7	11.1	0.04	41.98
2 multi	2	H						
92	1	03/05/2021	110	3049	7	11.7	0.06	45.28
2 multi	2	H						
92	1	03/06/2021	111	3050	7	11.7	0.07	40.3
2 multi	2	H						
92	1	03/07/2021	112	3051	7	12.3	0.12	40.95
2 multi	2	H						
92	1	03/08/2021	113	3052	7	10.9	0	38.9
2 multi	2	H						
92	1	03/09/2021	114	3053	7	10.9	0.09	38.89
2 multi	2	H						
92	1	03/10/2021	115	3054	7	10.3	0.04	42.34
2 multi	2	H						
92	1	03/11/2021	116	3055	7	10.3	0	36.6
2 multi	2	H						
92	1	03/12/2021	117	3056	7	10.3	0	37.38
2 multi	2	H						
92	1	03/13/2021	118	3057	7	10.3	0	37.89
2 multi	2	H						
92	1	03/14/2021	119	3058	7	10.3	0.01	41.17
2 multi	2	H						
92	1	03/15/2021	120	3059	7	10.3	0	36.54
2 multi	2	H						
92	1	03/16/2021	121	3060	7	10.4	0	38.2
2 multi	2	H						
92	1	03/17/2021	122	3061	7	10.3	0	38.14
2 multi	2	H						
92	1	03/18/2021	123	3062	7	10.3	0.03	46.36
2 multi	2	H						
92	1	03/19/2021	124	3063	7	10.4	0	41.97
2 multi	2	H						
92	1	03/20/2021	125	3064	7	10.6	0.02	42.03
2 multi	2	H						
92	1	03/21/2021	126	3065	7	10.4	0	37.5
2 multi	2	H						
92	1	03/22/2021	127	3066	7	10.3	0	43.4
2 multi	2	H						
92	1	03/23/2021	128	3067	7	10.2	0.01	38.94
2 multi	2	H						
92	1	03/24/2021	129	3068	7	10.3	0	39.66
2 multi	2	H						
92	1	03/25/2021	130	3069	7	10.2	0.01	35.29

2 multi 2		H						
92	1	03/26/2021	131	3070	7	10.2	0	45.35
2 multi 2		H						
92	1	03/27/2021	132	3071	7	10.2	0	40.06
2 multi 2		H						
92	1	03/28/2021	133	3072	7	10.3	0.01	39.13
2 multi 2		H						
92	1	03/29/2021	134	3073	7	10.6	0.01	37.29
2 multi 2		H						
92	1	03/30/2021	135	3074	7	10.4	0.13	46.27
2 multi 2		H						
92	1	03/31/2021	136	3075	7	10.2	0	40.93
2 multi 2		H						
92	1	04/01/2021	137	3076	7	10.4	0	40.32
2 multi 2		H						
92	1	04/02/2021	138	3077	7	10.3	0.01	39.88
2 multi 2		H						
92	1	04/03/2021	139	3078	7	10.3	0	41.21
2 multi 2		H						
92	1	04/04/2021	140	3079	7	10.3	0.02	38.86
2 multi 2		H						
92	1	04/05/2021	141	3080	7	10.3	0.04	39.86
2 multi 2		H						
92	1	04/06/2021	142	3081	7	10.3	0.02	40.75
2 multi 2		H						
92	1	04/07/2021	143	3082	7	10.4	0.07	39.09
2 multi 2		H						
92	1	04/08/2021	144	3083	7	10.6	0	41.08
2 multi 2		H						
92	1	04/09/2021	145	3084	7	10.4	0	37.36
2 multi 2		H						
92	1	04/10/2021	146	3085	7	10.4	0	42.26
2 multi 2		H						
92	1	04/11/2021	147	3086	7	11.1	0	41.76
2 multi 2		H						
92	1	04/12/2021	148	3087	7	10.6	0.01	42.77
2 multi 2		H						
92	1	04/13/2021	149	3088	7	10.4	0	40.93
2 multi 2		H						
92	1	04/14/2021	150	3089	7	10.6	0	44.17
2 multi 2		H						
92	1	04/15/2021	151	3090	7	10.4	0	43.97
2 multi 2		H						
92	1	04/16/2021	152	3091	7	10.6	0.02	41.56
2 multi 2		H						
92	1	04/17/2021	153	3092	7	10.4	0	43.06
2 multi 2		H						
92	1	04/18/2021	154	3093	7	10.4	0.02	41.58
2 multi 2		H						
92	1	04/19/2021	155	3094	7	10.4	0	44.33
2 multi 2		H						
92	1	04/20/2021	156	3095	7	10.6	0.03	44.57
2 multi 2		H						
92	1	04/21/2021	157	3096	7	10.4	0	37.59

2 multi	2	H						
92	1	04/22/2021	158	3097	7	10.3	0.02	43.77
2 multi	2	H						
92	1	04/23/2021	159	3098	7	10.6	0.03	40.42
2 multi	2	H						
92	1	04/24/2021	160	3099	7	10.3	0.02	43.53
2 multi	2	H						
92	1	04/25/2021	161	3100	7	10.4	0.02	43.1
2 multi	2	H						
92	1	04/26/2021	162	3101	7	10.4	0.02	44.68
2 multi	2	H						
92	1	04/27/2021	163	3102	7	10.4	0	39.62
2 multi	2	H						
92	1	04/28/2021	164	3103	7	10.6	0	44.17
2 multi	2	H						
92	1	04/29/2021	165	3104	7	10.6	0	42.36
2 multi	2	H						
92	1	04/30/2021	166	3105	7	10.4	0.11	43.36
2 multi	2	H						
92	1	05/01/2021	167	3106	7	10.4	0.02	40.58
2 multi	2	H						
92	1	05/02/2021	168	3107	7	10.7	0	42.01
2 multi	2	H						
92	1	05/03/2021	169	3108	7	10.7	0.01	39.15
2 multi	2	H						
92	1	05/04/2021	170	3109	7	10.9	0.03	39.8
2 multi	2	H						
92	1	05/05/2021	171	3110	7	10.6	0.03	41.93
2 multi	2	H						
92	1	05/06/2021	172	3111	7	10.7	0.03	42.68
2 multi	2	H						
92	1	05/07/2021	173	3112	7	10.6	0	38.97
2 multi	2	H						
92	1	05/08/2021	174	3113	7	10.7	0	38.41
2 multi	2	H						
92	1	05/09/2021	175	3114	7	11.1	0.02	36.57
2 multi	2	H						
92	1	05/10/2021	176	3115	7	10.9	0	37.57
2 multi	2	H						
93	1	06/14/2014	7	757	1	8.4	0.06	23
1 primi	1	H						
93	1	06/15/2014	8	758	1	8.7	0	22.44
1 primi	1	H						
93	1	06/16/2014	9	759	1	8.8	0.07	24.62
1 primi	1	H						
93	1	06/17/2014	10	760	1	8.7	0.06	27.83
1 primi	1	H						
93	1	06/18/2014	11	761	1	8.8	0	27.84
1 primi	1	H						
93	1	06/19/2014	12	762	1	8.6	0.05	27.8
1 primi	1	H						
93	1	06/20/2014	13	763	1	8.8	0.04	28.59
1 primi	1	H						
93	1	06/21/2014	14	764	1	8.8	0.05	28.07

1 primi	1	H						
93	1	06/22/2014	15	765	1	8.5	0.1	27.96
1 primi	1	H						
93	1	06/23/2014	16	766	1	8.7	0	31.32
1 primi	1	H						
93	1	06/24/2014	17	767	1	8.7	0.02	31.53
1 primi	1	H						
93	1	06/25/2014	18	768	1	8.6	0	31.93
1 primi	1	H						
93	1	06/26/2014	19	769	1	8.8	0.03	32.09
1 primi	1	H						
93	1	06/27/2014	20	770	1	8.8	0	33.61
1 primi	1	H						
93	1	06/28/2014	21	771	1	8.9	0	32.35
1 primi	1	H						
93	1	06/29/2014	22	772	1	8.7	0	34.42
1 primi	2	H						
93	1	06/30/2014	23	773	1	8.7	0	31.7
1 primi	2	H						
93	1	07/01/2014	24	774	1	8.5	0.11	33.78
1 primi	2	H						
93	1	07/02/2014	25	775	1	8.6	0.09	33.13
1 primi	2	H						
93	1	07/03/2014	26	776	1	8.4	0.17	34.09
1 primi	2	H						
93	1	07/04/2014	27	777	1	8.5	0.24	34.44
1 primi	2	H						
93	1	07/05/2014	28	778	1	8.3	0	33.96
1 primi	2	H						
93	1	07/06/2014	29	779	1	8.5	0.12	34.64
1 primi	2	H						
93	1	07/07/2014	30	780	1	8.6	0.12	33.94
1 primi	2	H						
93	1	07/08/2014	31	781	1	8.4	0.1	33.39
1 primi	2	H						
93	1	07/09/2014	32	782	1	8.4	0.1	31.98
1 primi	2	H						
93	1	07/10/2014	33	783	1	8.5	0	34.89
1 primi	2	H						
93	1	07/11/2014	34	784	1	8.5	0.11	34.64
1 primi	2	H						
93	1	07/12/2014	35	785	1	8.6	0	35.42
1 primi	2	H						
93	1	07/13/2014	36	786	1	8.8	0	33.22
1 primi	2	H						
93	1	07/14/2014	37	787	1	8.4	0.01	34.9
1 primi	2	H						
93	1	07/15/2014	38	788	1	8.7	0.03	35.23
1 primi	2	H						
93	1	07/16/2014	39	789	1	8.6	0.1	33.78
1 primi	2	H						
93	1	07/17/2014	40	790	1	8.9	0	36.77
1 primi	2	H						
93	1	07/18/2014	41	791	1	8.6	0.03	34.79

1 primi 2		H						
93	1	07/19/2014	42	792	1	8.7	0	35.25
1 primi 2		H						
93	1	07/20/2014	43	793	1	8.8	0	36.77
1 primi 2		H						
93	1	07/21/2014	44	794	1	8.9	0.12	36.28
1 primi 2		H						
93	1	07/22/2014	45	795	1	8.9	0.07	37.11
1 primi 2		H						
93	1	07/23/2014	46	796	1	8.6	0.1	36.01
1 primi 2		H						
93	1	07/24/2014	47	797	1	8.9	0	33.61
1 primi 2		H						
93	1	07/25/2014	48	798	1	8.8	0.09	34.85
1 primi 2		H						
93	1	07/26/2014	49	799	1	8.7	0.03	34.72
1 primi 2		H						
93	1	07/27/2014	50	800	1	8.8	0.02	37.44
1 primi 2		H						
93	1	07/28/2014	51	801	1	8.7	0.03	36.16
1 primi 2		H						
93	1	07/29/2014	52	802	1	8.8	0.08	35.38
1 primi 2		H						
93	1	07/30/2014	53	803	1	9.2	0.07	36.65
1 primi 2		H						
93	1	07/31/2014	54	804	1	9.1	0.09	35.01
1 primi 2		H						
93	1	08/01/2014	55	805	1	8.8	0.12	37.61
1 primi 2		H						
93	1	08/02/2014	56	806	1	8.9	0.03	37.77
1 primi 2		H						
93	1	08/03/2014	57	807	1	8.5	0.11	35.74
1 primi 2		H						
93	1	08/04/2014	58	808	1	8.8	0.08	35.14
1 primi 2		H						
93	1	08/05/2014	59	809	1	8.6	0.09	35.69
1 primi 2		H						
93	1	08/06/2014	60	810	1	8.7	0.09	37.01
1 primi 2		H						
93	1	08/07/2014	61	811	1	8.9	0	34.8
1 primi 2		H						
93	1	08/08/2014	62	812	1	8.9	0	34.64
1 primi 2		H						
93	1	08/09/2014	63	813	1	9.1	0	37.17
1 primi 2		H						
93	1	08/10/2014	64	814	1	9.2	0	36.02
1 primi 2		H						
93	1	08/11/2014	65	815	1	8.8	0	39.15
1 primi 2		H						
93	1	08/12/2014	66	816	1	8.8	0	39
1 primi 2		H						
93	1	08/13/2014	67	817	1	8.8	0	36.92
1 primi 2		H						
93	1	08/14/2014	68	818	1	8.8	0	36.05

1 primi	2	H						
93	1	08/15/2014	69	819	1	8.9	0	37.73
1 primi	2	H						
93	1	08/16/2014	70	820	1	8.8	0.17	36.36
1 primi	2	H						
93	1	08/17/2014	71	821	1	8.8	0.09	36.16
1 primi	2	H						
93	1	08/18/2014	72	822	1	9.1	0	37.43
1 primi	2	H						
93	1	08/19/2014	73	823	1	9.2	0	35.41
1 primi	2	H						
93	1	08/20/2014	74	824	1	8.9	0.05	32.48
1 primi	2	H						
93	1	08/21/2014	75	825	1	8.7	0.08	36.24
1 primi	2	H						
93	1	08/22/2014	76	826	1	8.7	0	37.79
1 primi	2	H						
93	1	08/23/2014	77	827	1	8.9	0	36.05
1 primi	2	H						
93	1	08/24/2014	78	828	1	8.8	0.04	32.62
1 primi	2	H						
93	1	08/25/2014	79	829	1	8.7	0	37.19
1 primi	2	H						
93	1	08/26/2014	80	830	1	8.6	0	35.74
1 primi	2	H						
93	1	08/27/2014	81	831	1	8.7	0.04	36.84
1 primi	2	H						
93	1	08/28/2014	82	832	1	8.9	0	38.67
1 primi	2	H						
93	1	08/29/2014	83	833	1	9.2	0	34.89
1 primi	2	H						
93	1	08/30/2014	84	834	1	9.5	0	38.08
1 primi	2	H						
93	1	08/31/2014	85	835	1	8.7	0	39.84
1 primi	2	H						
93	1	09/01/2014	86	836	1	8.8	0	39
1 primi	2	H						
93	1	09/02/2014	87	837	1	8.9	0	36.28
1 primi	2	H						
93	1	09/03/2014	88	838	1	8.8	0.06	33.88
1 primi	2	H						
93	1	09/04/2014	89	839	1	9.2	0	40.21
1 primi	2	H						
93	1	09/05/2014	90	840	1	8.7	0	37.04
1 primi	2	H						
93	1	09/06/2014	91	841	1	8.9	0	38.12
1 primi	2	H						
93	1	09/07/2014	92	842	1	8.7	0	33.47
1 primi	2	H						
93	1	09/08/2014	93	843	1	8.7	0	32.92
1 primi	2	H						
93	1	09/09/2014	94	844	1	8.9	0	31.69
1 primi	2	H						
93	1	09/10/2014	95	845	1	8.9	0	33.69

1 primi	2	H						
93	1	09/11/2014	96	846	1	9.2	0	33.48
1 primi	2	H						
93	1	09/12/2014	97	847	1	8.7	0	33.83
1 primi	2	H						
93	1	09/13/2014	98	848	1	9.2	0.06	32.75
1 primi	2	H						
93	1	09/14/2014	99	849	1	8.8	0	34.72
1 primi	2	H						
93	1	09/15/2014	100	850	1	8.8	0	32.47
1 primi	2	H						
93	1	06/05/2015	5	1113	2	8.8	0.05	35.28
2 multi	1	H						
93	1	06/06/2015	6	1114	2	9.2	0	34.15
2 multi	1	H						
93	1	06/07/2015	7	1115	2	9.6	0.1	34.02
2 multi	1	H						
93	1	06/08/2015	8	1116	2	9.3	0	38.23
2 multi	1	H						
93	1	06/09/2015	9	1117	2	9.3	0	38.7
2 multi	1	H						
93	1	06/10/2015	10	1118	2	9.6	0	40.11
2 multi	1	H						
93	1	06/11/2015	11	1119	2	9.3	0	42.63
2 multi	1	H						
93	1	06/12/2015	12	1120	2	9.3	0	38.89
2 multi	1	H						
93	1	06/13/2015	13	1121	2	9.2	0	42.01
2 multi	1	H						
93	1	06/14/2015	14	1122	2	9.3	0	41.13
2 multi	1	H						
93	1	06/15/2015	15	1123	2	9.2	0	41.58
2 multi	1	H						
93	1	06/16/2015	16	1124	2	9.6	0	46.23
2 multi	1	H						
93	1	06/17/2015	17	1125	2	9.5	0.02	44.81
2 multi	1	H						
93	1	06/18/2015	18	1126	2	9.9	0.07	49.32
2 multi	1	H						
93	1	06/19/2015	19	1127	2	9.8	0	48.33
2 multi	1	H						
93	1	06/20/2015	20	1128	2	10	0	49.43
2 multi	1	H						
93	1	06/21/2015	21	1129	2	9.8	0	50.13
2 multi	1	H						
93	1	06/22/2015	22	1130	2	9.9	0	51.98
2 multi	2	H						
93	1	06/23/2015	23	1131	2	9.8	0	50.71
2 multi	2	H						
93	1	06/24/2015	24	1132	2	9.3	0.17	48.39
2 multi	2	H						
93	1	06/25/2015	25	1133	2	9.6	0	48.01
2 multi	2	H						
93	1	06/26/2015	26	1134	2	9.5	0.01	51.2

2 multi	2	H						
93	1	06/27/2015	27	1135	2	9.6	0	55.37
2 multi	2	H						
93	1	06/28/2015	28	1136	2	9.6	0	50.18
2 multi	2	H						
93	1	06/29/2015	29	1137	2	9.1	0.09	52.03
2 multi	2	H						
93	1	06/30/2015	30	1138	2	9.3	0	51.66
2 multi	2	H						
93	1	07/01/2015	31	1139	2	9.3	0	51.59
2 multi	2	H						
93	1	07/02/2015	32	1140	2	9.3	0	49.31
2 multi	2	H						
93	1	07/03/2015	33	1141	2	9.2	0	49.11
2 multi	2	H						
93	1	07/04/2015	34	1142	2	9.2	0	48.17
2 multi	2	H						
93	1	07/05/2015	35	1143	2	9.1	0	46.59
2 multi	2	H						
93	1	07/06/2015	36	1144	2	9.2	0	45.74
2 multi	2	H						
93	1	07/07/2015	37	1145	2	9.5	0	44.48
2 multi	2	H						
93	1	07/08/2015	38	1146	2	9.3	0	43.7
2 multi	2	H						
93	1	07/09/2015	39	1147	2	9.3	0	44.81
2 multi	2	H						
93	1	07/10/2015	40	1148	2	9.9	0	41.86
2 multi	2	H						
93	1	07/11/2015	41	1149	2	9.6	0	46.13
2 multi	2	H						
93	1	07/12/2015	42	1150	2	9.3	0	48
2 multi	2	H						
93	1	07/13/2015	43	1151	2	9.9	0.01	51.87
2 multi	2	H						
93	1	07/14/2015	44	1152	2	9.3	0	44.06
2 multi	2	H						
93	1	07/15/2015	45	1153	2	9.6	0	54.82
2 multi	2	H						
93	1	07/16/2015	46	1154	2	9.5	0	49.28
2 multi	2	H						
93	1	07/17/2015	47	1155	2	9.6	0.08	47.5
2 multi	2	H						
93	1	07/18/2015	48	1156	2	9.5	0	46.95
2 multi	2	H						
93	1	07/19/2015	49	1157	2	9.3	0	42.98
2 multi	2	H						
93	1	07/20/2015	50	1158	2	9.5	0	41.23
2 multi	2	H						
93	1	07/21/2015	51	1159	2	10	0	50.91
2 multi	2	H						
93	1	07/22/2015	52	1160	2	9.6	0	46.04
2 multi	2	H						
93	1	07/23/2015	53	1161	2	9.6	0	45.35

2 multi	2	H						
93	1	07/24/2015	54	1162	2	9.9	0	44.79
2 multi	2	H						
93	1	07/25/2015	55	1163	2	9.6	0.13	44.1
2 multi	2	H						
93	1	07/26/2015	56	1164	2	9.8	0.13	48.68
2 multi	2	H						
93	1	07/27/2015	57	1165	2	9.8	0	48.07
2 multi	2	H						
93	1	07/28/2015	58	1166	2	9.6	0.23	48.76
2 multi	2	H						
93	1	07/29/2015	59	1167	2	9.6	0	47.44
2 multi	2	H						
93	1	07/30/2015	60	1168	2	9.3	0.04	44.09
2 multi	2	H						
93	1	07/31/2015	61	1169	2	9.8	0	52.77
2 multi	2	H						
93	1	08/01/2015	62	1170	2	9.8	0	51.11
2 multi	2	H						
93	1	08/02/2015	63	1171	2	9.9	0	48.31
2 multi	2	H						
93	1	08/03/2015	64	1172	2	9.9	0	45.88
2 multi	2	H						
93	1	08/04/2015	65	1173	2	9.8	0	45.46
2 multi	2	H						
93	1	08/05/2015	66	1174	2	9.8	0.08	45.64
2 multi	2	H						
93	1	08/06/2015	67	1175	2	9.8	0.09	46.92
2 multi	2	H						
93	1	08/07/2015	68	1176	2	9.8	0.09	46.66
2 multi	2	H						
93	1	08/08/2015	69	1177	2	9.6	0.04	42.93
2 multi	2	H						
93	1	08/09/2015	70	1178	2	9.5	0.04	44.18
2 multi	2	H						
93	1	08/10/2015	71	1179	2	9.6	0.06	41.69
2 multi	2	H						
93	1	08/11/2015	72	1180	2	9.5	0	45.63
2 multi	2	H						
93	1	08/12/2015	73	1181	2	9.3	0	43.4
2 multi	2	H						
93	1	08/13/2015	74	1182	2	9.6	0.11	43.8
2 multi	2	H						
93	1	08/14/2015	75	1183	2	9.9	0	43.36
2 multi	2	H						
93	1	08/15/2015	76	1184	2	9.9	0	47.05
2 multi	2	H						
93	1	08/16/2015	77	1185	2	10	0	66.28
2 multi	2	H						
93	1	08/17/2015	78	1186	2	10	0	46.49
2 multi	2	H						
93	1	08/18/2015	79	1187	2	9.8	0	43.93
2 multi	2	H						
93	1	08/19/2015	80	1188	2	9.8	0.19	41.03

2 multi	2	H						
93	1	08/20/2015	81	1189	2	9.9	0	44.5
2 multi	2	H						
93	1	08/21/2015	82	1190	2	9.8	0.02	43.98
2 multi	2	H						
93	1	08/22/2015	83	1191	2	10.2	0	46.92
2 multi	2	H						
93	1	08/23/2015	84	1192	2	9.9	0	48.71
2 multi	2	H						
93	1	08/24/2015	85	1193	2	10.2	0	44.23
2 multi	2	H						
93	1	08/25/2015	86	1194	2	10	0	41.37
2 multi	2	H						
93	1	08/26/2015	87	1195	2	9.9	0.09	45.88
2 multi	2	H						
93	1	08/27/2015	88	1196	2	10	0	53.72
2 multi	2	H						
93	1	08/28/2015	89	1197	2	9.9	0	51.56
2 multi	2	H						
93	1	08/29/2015	90	1198	2	10	0	48.8
2 multi	2	H						
93	1	08/30/2015	91	1199	2	9.8	0	47.55
2 multi	2	H						
93	1	08/31/2015	92	1200	2	9.6	0	49
2 multi	2	H						
93	1	09/01/2015	93	1201	2	9.8	0	46.54
2 multi	2	H						
93	1	09/02/2015	94	1202	2	9.8	0	43.8
2 multi	2	H						
93	1	09/03/2015	95	1203	2	9.9	0	49.23
2 multi	2	H						
93	1	09/04/2015	96	1204	2	9.8	0	48.63
2 multi	2	H						
93	1	09/05/2015	97	1205	2	9.5	0	47.06
2 multi	2	H						
93	1	09/06/2015	98	1206	2	9.8	0.08	43.88
2 multi	2	H						
93	1	09/07/2015	99	1207	2	9.9	0	51.39
2 multi	2	H						
93	1	09/08/2015	100	1208	2	9.2	0	49.24
2 multi	2	H						
93	1	09/09/2015	101	1209	2	9.8	0	48.11
2 multi	2	H						
93	1	09/10/2015	102	1210	2	9.2	0	41.77
2 multi	2	H						
93	1	09/11/2015	103	1211	2	10.2	0	42.43
2 multi	2	H						
93	1	09/12/2015	104	1212	2	9.9	0	38.77
2 multi	2	H						
93	1	09/13/2015	105	1213	2	10	0	45.29
2 multi	2	H						
93	1	09/14/2015	106	1214	2	10.4	0	44.66
2 multi	2	H						
93	1	09/15/2015	107	1215	2	9.8	0	53.03

2 multi 2		H						
93	1	09/16/2015	108	1216	2	10	0	42.56
2 multi 2		H						
93	1	09/17/2015	109	1217	2	9.9	0	43.19
2 multi 2		H						
93	1	09/18/2015	110	1218	2	10	0	42.15
2 multi 2		H						
93	1	09/19/2015	111	1219	2	9.8	0	38.59
2 multi 2		H						
93	1	09/20/2015	112	1220	2	9.5	0	40.17
2 multi 2		H						
93	1	09/21/2015	113	1221	2	9.1	0.01	37.88
2 multi 2		H						
93	1	09/22/2015	114	1222	2	9.1	0	38.57
2 multi 2		H						
93	1	09/23/2015	115	1223	2	9.8	0	40.79
2 multi 2		H						
93	1	09/24/2015	116	1224	2	9.6	0.01	53.67
2 multi 2		H						
93	1	09/25/2015	117	1225	2	9.8	0.13	41.98
2 multi 2		H						
93	1	09/26/2015	118	1226	2	9.3	0.1	48.68
2 multi 2		H						
93	1	09/27/2015	119	1227	2	9.1	0.01	43.43
2 multi 2		H						
93	1	09/28/2015	120	1228	2	9.9	0	50.18
2 multi 2		H						
93	1	09/29/2015	121	1229	2	9.6	0.01	45.09
2 multi 2		H						
93	1	09/30/2015	122	1230	2	9.6	0.03	43.57
2 multi 2		H						
93	1	10/01/2015	123	1231	2	9.6	0	46.49
2 multi 2		H						
93	1	10/02/2015	124	1232	2	9.2	0.04	40.31
2 multi 2		H						
93	1	10/03/2015	125	1233	2	9.3	0	46.62
2 multi 2		H						
93	1	10/04/2015	126	1234	2	9.6	0	39.88
2 multi 2		H						
93	1	10/05/2015	127	1235	2	9.8	0.05	41.7
2 multi 2		H						
93	1	10/06/2015	128	1236	2	9.8	0	48.95
2 multi 2		H						
93	1	10/07/2015	129	1237	2	9.8	0	40.42
2 multi 2		H						
93	1	10/08/2015	130	1238	2	9.5	0.11	41.54
2 multi 2		H						
93	1	10/09/2015	131	1239	2	9.5	0.12	48.92
2 multi 2		H						
93	1	10/10/2015	132	1240	2	9.6	0	46.19
2 multi 2		H						
93	1	10/11/2015	133	1241	2	9.3	0	37.66
2 multi 2		H						
93	1	10/12/2015	134	1242	2	9.6	0	38.24

2 multi 2		H						
93	1	10/13/2015	135	1243	2	9.8	0	40.79
2 multi 2		H						
93	1	10/14/2015	136	1244	2	9.6	0	43.28
2 multi 2		H						
93	1	10/15/2015	137	1245	2	9.3	0	43.95
2 multi 2		H						
93	1	10/16/2015	138	1246	2	9.6	0	42.65
2 multi 2		H						
93	1	10/17/2015	139	1247	2	9.9	0	41.69
2 multi 2		H						
93	1	10/18/2015	140	1248	2	10.2	0.13	45.64
2 multi 2		H						
93	1	10/19/2015	141	1249	2	10	0	43.11
2 multi 2		H						
93	1	10/20/2015	142	1250	2	9.8	0	38.08
2 multi 2		H						
93	1	10/21/2015	143	1251	2	9.3	0	36.81
2 multi 2		H						
93	1	10/22/2015	144	1252	2	9.6	0	36.41
2 multi 2		H						
93	1	10/23/2015	145	1253	2	8.6	0	39.8
2 multi 2		H						
93	1	10/24/2015	146	1254	2	9.2	0	37.22
2 multi 2		H						
93	1	10/25/2015	147	1255	2	10	0	42.93
2 multi 2		H						
93	1	10/26/2015	148	1256	2	9.1	0	37.66
2 multi 2		H						
93	1	10/27/2015	149	1257	2	9.6	0	39.81
2 multi 2		H						
93	1	10/28/2015	150	1258	2	9.3	0	42.15
2 multi 2		H						
93	1	10/29/2015	151	1259	2	9.8	0	41.39
2 multi 2		H						
93	1	10/30/2015	152	1260	2	9.3	0.04	38.55
2 multi 2		H						
93	1	10/31/2015	153	1261	2	9.9	0.01	43.15
2 multi 2		H						
93	1	11/01/2015	154	1262	2	10.2	0	40.12
2 multi 2		H						
93	1	11/02/2015	155	1263	2	9.6	0.08	39.91
2 multi 2		H						
93	1	11/03/2015	156	1264	2	10	0	43.18
2 multi 2		H						
93	1	11/04/2015	157	1265	2	9.2	0.08	38.31
2 multi 2		H						
93	1	11/05/2015	158	1266	2	9.9	0	43.64
2 multi 2		H						
93	1	11/06/2015	159	1267	2	8.8	0	39.6
2 multi 2		H						
93	1	11/07/2015	160	1268	2	10	0.15	37.49
2 multi 2		H						
93	1	11/08/2015	161	1269	2	9.8	0.15	38.93

2 multi 2 H
93 1 11/09/2015 162 1270 2 10.2 0 37.57
2 multi 2 H
93 1 11/10/2015 163 1271 2 8.8 0.03 36.91
2 multi 2 H
93 1 11/11/2015 164 1272 2 9.8 0.12 35.09
2 multi 2 H
93 1 11/12/2015 165 1273 2 9.2 0 38.21
2 multi 2 H
93 1 11/13/2015 166 1274 2 9.8 0 33.84
2 multi 2 H
93 1 11/14/2015 167 1275 2 9.6 0 38.93
2 multi 2 H
93 1 11/15/2015 168 1276 2 9.8 0.01 38.77
2 multi 2 H
93 1 11/16/2015 169 1277 2 9.5 0 42.62
2 multi 2 H
93 1 11/17/2015 170 1278 2 10 0 39.35
2 multi 2 H
93 1 11/18/2015 171 1279 2 9.6 0 43.91
2 multi 2 H
93 1 11/19/2015 172 1280 2 9.9 0.05 40.21
2 multi 2 H
93 1 11/20/2015 173 1281 2 10.2 0 41.99
2 multi 2 H
93 1 11/21/2015 174 1282 2 10.2 0.05 41.4
2 multi 2 H
93 1 11/22/2015 175 1283 2 9.5 0 36.19
2 multi 2 H
93 1 11/23/2015 176 1284 2 9.8 0 40.07
2 multi 2 H
93 1 11/24/2015 177 1285 2 9.6 0 20.29
2 multi 2 H
93 1 11/25/2015 178 1286 2 10 0.21 38.81
2 multi 2 H
93 1 11/26/2015 179 1287 2 10.2 0 41.72
2 multi 2 H
93 1 11/27/2015 180 1288 2 9.3 0.02 39.41
2 multi 2 H
93 1 11/28/2015 181 1289 2 10.3 0 38.88
2 multi 2 H
93 1 11/29/2015 182 1290 2 9.8 0 39.54
2 multi 2 H
93 1 11/30/2015 183 1291 2 10 0 41.82
2 multi 2 H
93 1 12/01/2015 184 1292 2 9.8 0 38.33
2 multi 2 H
93 1 12/02/2015 185 1293 2 9.6 0 38.05
2 multi 2 H
93 1 12/03/2015 186 1294 2 10 0 37.92
2 multi 2 H
93 1 12/04/2015 187 1295 2 9.9 0.05 27.81
2 multi 2 H
93 1 12/19/2016 9 1676 3 8.6 0 37.15

2 multi	1	H						
93	1	12/20/2016	10	1677	3	8.7	0.11	38.8
2 multi	1	H						
93	1	12/21/2016	11	1678	3	9.1	0	39.82
2 multi	1	H						
93	1	12/22/2016	12	1679	3	8.9	0	42.48
2 multi	1	H						
93	1	12/23/2016	13	1680	3	8.6	0	39.55
2 multi	1	H						
93	1	12/24/2016	14	1681	3	8.8	0.17	40.46
2 multi	1	H						
93	1	12/25/2016	15	1682	3	9.1	0	43.08
2 multi	1	H						
93	1	12/26/2016	16	1683	3	8.9	0.11	41.88
2 multi	1	H						
93	1	12/27/2016	17	1684	3	9.1	0	44.63
2 multi	1	H						
93	1	12/28/2016	18	1685	3	8.9	0.13	46.22
2 multi	1	H						
93	1	12/29/2016	19	1686	3	9.2	0.13	44.23
2 multi	1	H						
93	1	12/30/2016	20	1687	3	8.9	0.07	45.47
2 multi	1	H						
93	1	12/31/2016	21	1688	3	8.9	0.08	43.36
2 multi	1	H						
93	1	01/01/2017	22	1689	3	9.6	0	54.73
2 multi	2	H						
93	1	01/02/2017	23	1690	3	9.8	0.03	49.89
2 multi	2	H						
93	1	01/03/2017	24	1691	3	8.8	0	49.43
2 multi	2	H						
93	1	01/04/2017	25	1692	3	9.3	0.17	46.91
2 multi	2	H						
93	1	01/05/2017	26	1693	3	9.3	0	48.88
2 multi	2	H						
93	1	01/06/2017	27	1694	3	9.1	0.16	49.88
2 multi	2	H						
93	1	01/07/2017	28	1695	3	9.1	0.11	50.49
2 multi	2	H						
93	1	01/08/2017	29	1696	3	9.1	0	48.7
2 multi	2	H						
93	1	01/09/2017	30	1697	3	8.9	0	48.73
2 multi	2	H						
93	1	01/10/2017	31	1698	3	8.8	0	48.29
2 multi	2	H						
93	1	01/11/2017	32	1699	3	8.6	0	49.47
2 multi	2	H						
93	1	01/12/2017	33	1700	3	8.8	0	50.1
2 multi	2	H						
93	1	01/13/2017	34	1701	3	8.7	0.14	49.59
2 multi	2	H						
93	1	01/14/2017	35	1702	3	8.6	0.16	46.77
2 multi	2	H						
93	1	01/15/2017	36	1703	3	8.8	0	47.98

2 multi	2	H						
93	1	01/16/2017	37	1704	3	8.5	0.08	46.14
2 multi	2	H						
93	1	01/17/2017	38	1705	3	8.8	0.02	47.49
2 multi	2	H						
93	1	01/18/2017	39	1706	3	8.6	0	45.39
2 multi	2	H						
93	1	01/19/2017	40	1707	3	8.6	0.12	45.36
2 multi	2	H						
93	1	01/20/2017	41	1708	3	8.9	0.02	42.99
2 multi	2	H						
93	1	01/21/2017	42	1709	3	9.3	0.03	42.57
2 multi	2	H						
93	1	01/22/2017	43	1710	3	8.4	0.03	39.28
2 multi	2	H						
93	1	01/23/2017	44	1711	3	8.9	0	44.77
2 multi	2	H						
93	1	01/24/2017	45	1712	3	9.3	0	45.55
2 multi	2	H						
93	1	01/25/2017	46	1713	3	8.8	0	43.66
2 multi	2	H						
93	1	01/04/2018	7	2057	4	9.5	0.22	36.65
2 multi	1	H						
93	1	01/05/2018	8	2058	4	9.3	0.02	40.45
2 multi	1	H						
93	1	01/06/2018	9	2059	4	9.3	0.01	38.79
2 multi	1	H						
93	1	01/07/2018	10	2060	4	9.6	0	35.96
2 multi	1	H						
93	1	01/08/2018	11	2061	4	9.1	0.02	42.52
2 multi	1	H						
93	1	01/09/2018	12	2062	4	9.6	0.08	43.4
2 multi	1	H						
93	1	01/10/2018	13	2063	4	9.3	0	40.63
2 multi	1	H						
93	1	01/11/2018	14	2064	4	9.5	0.07	42.29
2 multi	1	H						
93	1	01/12/2018	15	2065	4	9.3	0	41.96
2 multi	1	H						
93	1	01/13/2018	16	2066	4	9.1	0.07	39.31
2 multi	1	H						
93	1	01/14/2018	17	2067	4	9.3	0.16	44.95
2 multi	1	H						
93	1	01/15/2018	18	2068	4	8.9	0.06	34.38
2 multi	1	H						
93	1	01/16/2018	19	2069	4	9.2	0.07	45.4
2 multi	1	H						
93	1	01/17/2018	20	2070	4	9.2	0.25	44.24
2 multi	1	H						
93	1	01/18/2018	21	2071	4	8.8	0.08	43.08
2 multi	1	H						
93	1	01/19/2018	22	2072	4	8.7	0	39.83
2 multi	2	H						
93	1	01/20/2018	23	2073	4	8.7	0	43.07

2 multi	2	H						
93	1	01/21/2018	24	2074	4	8.9	0.08	42.93
2 multi	2	H						
93	1	01/22/2018	25	2075	4	8.8	0.2	44.11
2 multi	2	H						
93	1	01/23/2018	26	2076	4	8.8	0	44.11
2 multi	2	H						
93	1	01/24/2018	27	2077	4	9.2	0.06	55.1
2 multi	2	H						
93	1	01/25/2018	28	2078	4	9.2	0.09	50.06
2 multi	2	H						
93	1	01/26/2018	29	2079	4	9.1	0.25	49.64
2 multi	2	H						
93	1	01/27/2018	30	2080	4	9.1	0	43.92
2 multi	2	H						
93	1	01/28/2018	31	2081	4	9.2	0	49.43
2 multi	2	H						
93	1	01/29/2018	32	2082	4	9.3	0	46.49
2 multi	2	H						
93	1	01/30/2018	33	2083	4	8.9	0.07	48.14
2 multi	2	H						
93	1	01/31/2018	34	2084	4	10	0	44.52
2 multi	2	H						
93	1	02/01/2018	35	2085	4	9.9	0.09	35.62
2 multi	2	H						
93	1	02/02/2018	36	2086	4	12	0	40.72
2 multi	2	H						
93	1	02/03/2018	37	2087	4	9.1	0	35.19
2 multi	2	H						
93	1	02/04/2018	38	2088	4	8.9	0.21	40.75
2 multi	2	H						
93	1	02/05/2018	39	2089	4	9.1	0.07	45.56
2 multi	2	H						
93	1	02/06/2018	40	2090	4	9.1	0.09	48.46
2 multi	2	H						
93	1	02/07/2018	41	2091	4	10.2	0.03	43.38
2 multi	2	H						
93	1	02/08/2018	42	2092	4	9.1	0	42.07
2 multi	2	H						
93	1	02/09/2018	43	2093	4	9.9	0.08	46.78
2 multi	2	H						
93	1	02/10/2018	44	2094	4	9.3	0.09	47.17
2 multi	2	H						
93	1	02/11/2018	45	2095	4	9.5	0.13	47.55
2 multi	2	H						
93	1	02/12/2018	46	2096	4	9.2	0.08	44.51
2 multi	2	H						
93	1	02/13/2018	47	2097	4	9.3	0.09	46.11
2 multi	2	H						
93	1	02/14/2018	48	2098	4	9.2	0.08	44.41
2 multi	2	H						
93	1	02/15/2018	49	2099	4	8.9	0.05	42.67
2 multi	2	H						
93	1	02/16/2018	50	2100	4	9.3	0.01	45.36

2 multi 2		H						
93	1	02/17/2018	51	2101	4	9.5	0	44.61
2 multi 2		H						
93	1	02/18/2018	52	2102	4	9.6	0	46.62
2 multi 2		H						
93	1	02/19/2018	53	2103	4	9.8	0.09	46.76
2 multi 2		H						
93	1	02/20/2018	54	2104	4	9.2	0.11	42.93
2 multi 2		H						
93	1	02/21/2018	55	2105	4	9.5	0.1	49.3
2 multi 2		H						
93	1	02/22/2018	56	2106	4	9.2	0.09	42.74
2 multi 2		H						
93	1	02/23/2018	57	2107	4	9.3	0	43.92
2 multi 2		H						
93	1	02/24/2018	58	2108	4	9.3	0	45.2
2 multi 2		H						
93	1	02/25/2018	59	2109	4	9.5	0	45.01
2 multi 2		H						
93	1	02/26/2018	60	2110	4	9.8	0.1	46.79
2 multi 2		H						
93	1	02/27/2018	61	2111	4	10.2	0.09	41.51
2 multi 2		H						
93	1	02/28/2018	62	2112	4	9.3	0	39.84
2 multi 2		H						
93	1	03/01/2018	63	2113	4	9.6	0	45.55
2 multi 2		H						
93	1	03/02/2018	64	2114	4	9.5	0	42.94
2 multi 2		H						
93	1	03/03/2018	65	2115	4	9.3	0.11	41.74
2 multi 2		H						
93	1	03/04/2018	66	2116	4	9.5	0.1	47.22
2 multi 2		H						
93	1	03/05/2018	67	2117	4	8.9	0	48.29
2 multi 2		H						
93	1	03/06/2018	68	2118	4	9.1	0	43.37
2 multi 2		H						
93	1	03/07/2018	69	2119	4	8.8	0	44.48
2 multi 2		H						
93	1	03/08/2018	70	2120	4	8.7	0.09	41.76
2 multi 2		H						
93	1	03/09/2018	71	2121	4	9.8	0.09	43.41
2 multi 2		H						
93	1	03/10/2018	72	2122	4	8.8	0.01	44.86
2 multi 2		H						
93	1	03/11/2018	73	2123	4	9.5	0.1	42.49
2 multi 2		H						
93	1	03/12/2018	74	2124	4	9.3	0	44.9
2 multi 2		H						
93	1	03/13/2018	75	2125	4	9.1	0.17	40.05
2 multi 2		H						
93	1	03/14/2018	76	2126	4	9.3	0	48.95
2 multi 2		H						
93	1	03/15/2018	77	2127	4	9.2	0.19	43.33

2 multi 2		H						
93	1	03/16/2018	78	2128	4	9.9	0.11	43.35
2 multi 2		H						
93	1	03/17/2018	79	2129	4	9.5	0.1	41.57
2 multi 2		H						
93	1	03/18/2018	80	2130	4	9.8	0.02	45.6
2 multi 2		H						
93	1	08/31/2018	246	2296	4	9.3	0.15	31.33
2 multi 2		H						
93	1	09/01/2018	247	2297	4	9.9	0	36.39
2 multi 2		H						
93	1	09/02/2018	248	2298	4	10	0.1	37.35
2 multi 2		H						
93	1	09/03/2018	249	2299	4	10.2	0.24	38.75
2 multi 2		H						
93	1	09/04/2018	250	2300	4	9.2	0.23	36.26
2 multi 2		H						
93	1	09/05/2018	251	2301	4	9.6	0.23	36.71
2 multi 2		H						
93	1	09/06/2018	252	2302	4	9.8	0	34.47
2 multi 2		H						
93	1	09/07/2018	253	2303	4	9.6	0.03	37.65
2 multi 2		H						
93	1	09/08/2018	254	2304	4	9.2	0.11	35.54
2 multi 2		H						
93	1	09/09/2018	255	2305	4	9.8	0.08	36.61
2 multi 2		H						
93	1	09/10/2018	256	2306	4	9.9	0	35.66
2 multi 2		H						
93	1	09/11/2018	257	2307	4	9.8	0.07	29.26
2 multi 2		H						
93	1	09/12/2018	258	2308	4	9.9	0	33.42
2 multi 2		H						
93	1	09/13/2018	259	2309	4	10.2	0	33.47
2 multi 2		H						
93	1	09/14/2018	260	2310	4	9.8	0.09	31.48
2 multi 2		H						
93	1	09/15/2018	261	2311	4	10	0.12	32.74
2 multi 2		H						
93	1	09/16/2018	262	2312	4	9.6	0.22	31.92
2 multi 2		H						
93	1	09/17/2018	263	2313	4	10.2	0.16	31.8
2 multi 2		H						
93	1	09/18/2018	264	2314	4	10.3	0	35.16
2 multi 2		H						
93	1	09/19/2018	265	2315	4	10	0.08	29.44
2 multi 2		H						
93	1	09/20/2018	266	2316	4	10	0.03	31.57
2 multi 2		H						
93	1	09/21/2018	267	2317	4	10.3	0	30.86
2 multi 2		H						
93	1	09/22/2018	268	2318	4	9.9	0	30.56
2 multi 2		H						
93	1	09/23/2018	269	2319	4	10.2	0.09	32.35

2 multi	2	H						
93	1	09/24/2018	270	2320	4	10.3	0	32.83
2 multi	2	H						
93	1	09/25/2018	271	2321	4	10.3	0	28.29
2 multi	2	H						
93	1	09/26/2018	272	2322	4	9.8	0.06	31.69
2 multi	2	H						
93	1	09/27/2018	273	2323	4	10.3	0	34.37
2 multi	2	H						
93	1	01/07/2019	8	2425	5	9.2	0.16	33.27
2 multi	1	H						
93	1	01/08/2019	9	2426	5	9.2	0.04	32.35
2 multi	1	H						
93	1	01/09/2019	10	2427	5	9.6	0.04	33.33
2 multi	1	H						
93	1	01/10/2019	11	2428	5	9.3	0.04	33.69
2 multi	1	H						
93	1	01/11/2019	12	2429	5	9.6	0	37.07
2 multi	1	H						
93	1	01/12/2019	13	2430	5	9.5	0.15	34.38
2 multi	1	H						
93	1	01/13/2019	14	2431	5	9.3	0.04	33.91
2 multi	1	H						
93	1	01/14/2019	15	2432	5	9.2	0.13	35.56
2 multi	1	H						
93	1	01/15/2019	16	2433	5	9.2	0.13	36.19
2 multi	1	H						
93	1	01/16/2019	17	2434	5	9.3	0.03	34.81
2 multi	1	H						
93	1	01/17/2019	18	2435	5	9.1	0.01	38.86
2 multi	1	H						
93	1	01/18/2019	19	2436	5	9.3	0	38
2 multi	1	H						
93	1	01/19/2019	20	2437	5	9.3	0.14	39.75
2 multi	1	H						
93	1	01/20/2019	21	2438	5	9.1	0	19.24
2 multi	1	H						
93	1	01/21/2019	22	2439	5	9.1	0.02	41.7
2 multi	2	H						
93	1	01/22/2019	23	2440	5	9.1	0.14	37.43
2 multi	2	H						
93	1	01/23/2019	24	2441	5	9.3	0	40.86
2 multi	2	H						
93	1	01/24/2019	25	2442	5	9.2	0.13	41.14
2 multi	2	H						
93	1	01/25/2019	26	2443	5	9.2	0.17	40.81
2 multi	2	H						
93	1	01/26/2019	27	2444	5	8.9	0	40.98
2 multi	2	H						
93	1	01/27/2019	28	2445	5	8.9	0	42.35
2 multi	2	H						
93	1	01/28/2019	29	2446	5	9.2	0	36.47
2 multi	2	H						
93	1	01/29/2019	30	2447	5	9.1	0.01	36.98

2 multi	2	H						
93	1	01/30/2019	31	2448	5	9.1	0	37.38
2 multi	2	H						
93	1	01/31/2019	32	2449	5	8.9	0	37.32
2 multi	2	H						
93	1	02/01/2019	33	2450	5	9.2	0.13	41.1
2 multi	2	H						
93	1	02/02/2019	34	2451	5	9.2	0	38.14
2 multi	2	H						
93	1	02/03/2019	35	2452	5	9.1	0.06	36.09
2 multi	2	H						
93	1	02/04/2019	36	2453	5	9.3	0	40.05
2 multi	2	H						
93	1	02/05/2019	37	2454	5	9.2	0.02	38.43
2 multi	2	H						
93	1	02/06/2019	38	2455	5	9.3	0	40.29
2 multi	2	H						
93	1	02/07/2019	39	2456	5	9.8	0	40.13
2 multi	2	H						
93	1	02/08/2019	40	2457	5	8.9	0	37.46
2 multi	2	H						
93	1	02/09/2019	41	2458	5	9.3	0.15	39.41
2 multi	2	H						
93	1	02/10/2019	42	2459	5	9.5	0.13	38.8
2 multi	2	H						
93	1	02/11/2019	43	2460	5	9.5	0.14	38.25
2 multi	2	H						
93	1	02/12/2019	44	2461	5	9.2	0.13	36.7
2 multi	2	H						
93	1	02/13/2019	45	2462	5	9.5	0.12	38.8
2 multi	2	H						
93	1	02/14/2019	46	2463	5	9.3	0.01	37.85
2 multi	2	H						
93	1	02/15/2019	47	2464	5	9.3	0	38
2 multi	2	H						
93	1	02/16/2019	48	2465	5	9.1	0.2	40.01
2 multi	2	H						
93	1	02/17/2019	49	2466	5	9.3	0	40.49
2 multi	2	H						
93	1	02/18/2019	50	2467	5	9.5	0	42.03
2 multi	2	H						
93	1	02/19/2019	51	2468	5	9.2	0	38.55
2 multi	2	H						
93	1	02/20/2019	52	2469	5	9.2	0	40.39
2 multi	2	H						
93	1	02/21/2019	53	2470	5	9.2	0	36.91
2 multi	2	H						
93	1	02/22/2019	54	2471	5	9.3	0.12	37.35
2 multi	2	H						
93	1	02/23/2019	55	2472	5	8.9	0.1	37.98
2 multi	2	H						
93	1	02/24/2019	56	2473	5	9.1	0.05	38.33
2 multi	2	H						
93	1	02/25/2019	57	2474	5	9.5	0.28	37.19

2 multi 2		H						
93	1	02/26/2019	58	2475	5	9.5	0.12	40.04
2 multi 2		H						
93	1	02/27/2019	59	2476	5	9.5	0.01	36.37
2 multi 2		H						
93	1	02/28/2019	60	2477	5	9.1	0.06	35.25
2 multi 2		H						
93	1	03/01/2019	61	2478	5	9.6	0	40.16
2 multi 2		H						
93	1	03/02/2019	62	2479	5	9.8	0.07	36.27
2 multi 2		H						
93	1	03/03/2019	63	2480	5	9.5	0.19	36.41
2 multi 2		H						
93	1	03/04/2019	64	2481	5	9.5	0	36.21
2 multi 2		H						
93	1	03/05/2019	65	2482	5	9.6	0.15	36.47
2 multi 2		H						
93	1	03/06/2019	66	2483	5	9.8	0.11	34.83
2 multi 2		H						
93	1	03/07/2019	67	2484	5	9.6	0	38.31
2 multi 2		H						
93	1	03/28/2019	88	2505	5	9.5	0.11	33.32
2 multi 2		H						
93	1	03/29/2019	89	2506	5	9.3	0	36.8
2 multi 2		H						
93	1	03/30/2019	90	2507	5	9.3	0	37.89
2 multi 2		H						
93	1	03/31/2019	91	2508	5	9.9	0.11	35.32
2 multi 2		H						
93	1	04/01/2019	92	2509	5	9.8	0.13	38.97
2 multi 2		H						
93	1	04/02/2019	93	2510	5	9.8	0.01	36.06
2 multi 2		H						
93	1	04/03/2019	94	2511	5	9.6	0.01	30.02
2 multi 2		H						
93	1	04/04/2019	95	2512	5	9.8	0	34.25
2 multi 2		H						
93	1	04/05/2019	96	2513	5	8.9	0.24	34.09
2 multi 2		H						
93	1	04/06/2019	97	2514	5	9.8	0.11	35.62
2 multi 2		H						
93	1	04/07/2019	98	2515	5	9.6	0.18	31.54
2 multi 2		H						
93	1	04/08/2019	99	2516	5	9.5	0.33	30.5
2 multi 2		H						
93	1	04/09/2019	100	2517	5	9.6	0.17	35.34
2 multi 2		H						
93	1	04/10/2019	101	2518	5	10	0	34.61
2 multi 2		H						
93	1	04/11/2019	102	2519	5	9.8	0	33.37
2 multi 2		H						
93	1	04/12/2019	103	2520	5	9.6	0	32.16
2 multi 2		H						
93	1	04/13/2019	104	2521	5	9.8	0.12	35.49

2 multi	2	H						
93	1	04/14/2019	105	2522	5	9.8	0	34.36
2 multi	2	H						
93	1	04/15/2019	106	2523	5	9.6	0.11	33.47
2 multi	2	H						
93	1	04/16/2019	107	2524	5	9.5	0	33.02
2 multi	2	H						
93	1	04/17/2019	108	2525	5	9.6	0.11	35.12
2 multi	2	H						
93	1	04/18/2019	109	2526	5	9.5	0.27	34.37
2 multi	2	H						
93	1	04/19/2019	110	2527	5	9.3	0.09	34.32
2 multi	2	H						
93	1	04/20/2019	111	2528	5	10	0	35.95
2 multi	2	H						
93	1	04/21/2019	112	2529	5	9.8	0.12	35.42
2 multi	2	H						
93	1	04/22/2019	113	2530	5	9.2	0.13	37.11
2 multi	2	H						
93	1	04/23/2019	114	2531	5	9.9	0	34.69
2 multi	2	H						
93	1	04/24/2019	115	2532	5	8.9	0	26.23
2 multi	2	H						
93	1	04/25/2019	116	2533	5	8.8	0.11	31.76
2 multi	2	H						
93	1	04/26/2019	117	2534	5	9.3	0.4	33.94
2 multi	2	H						
93	1	04/27/2019	118	2535	5	9.2	0.16	34.55
2 multi	2	H						
93	1	04/28/2019	119	2536	5	9.3	0.3	29.84
2 multi	2	H						
93	1	04/29/2019	120	2537	5	9.6	0.19	30.64
2 multi	2	H						
93	1	04/30/2019	121	2538	5	9.5	0.12	30.98
2 multi	2	H						
93	1	05/01/2019	122	2539	5	9.9	0.11	31.12
2 multi	2	H						
93	1	05/02/2019	123	2540	5	9.5	0	32.1
2 multi	2	H						
93	1	05/03/2019	124	2541	5	9.5	0	33.58
2 multi	2	H						
93	1	01/16/2020	7	2799	6	9.5	0.22	34.99
2 multi	1	H						
93	1	01/17/2020	8	2800	6	9.5	0.24	31.01
2 multi	1	H						
93	1	01/18/2020	9	2801	6	9.8	0.14	32.58
2 multi	1	H						
93	1	01/19/2020	10	2802	6	9.8	0.05	35.93
2 multi	1	H						
93	1	01/20/2020	11	2803	6	9.3	0.13	36.02
2 multi	1	H						
93	1	01/21/2020	12	2804	6	9.8	0.03	36.6
2 multi	1	H						
93	1	01/22/2020	13	2805	6	9.5	0	37.42

2 multi	1	H						
93	1	01/23/2020	14	2806	6	9.6	0.13	37.9
2 multi	1	H						
93	1	01/24/2020	15	2807	6	9.5	0.15	39.33
2 multi	1	H						
93	1	01/25/2020	16	2808	6	9.3	0.14	37.03
2 multi	1	H						
93	1	01/26/2020	17	2809	6	9.5	0.07	42.32
2 multi	1	H						
93	1	01/27/2020	18	2810	6	9.6	0.05	45.12
2 multi	1	H						
93	1	01/28/2020	19	2811	6	9.6	0.13	41.4
2 multi	1	H						
93	1	01/29/2020	20	2812	6	9.6	0	40.32
2 multi	1	H						
93	1	01/30/2020	21	2813	6	9.8	0	43.05
2 multi	1	H						
93	1	01/31/2020	22	2814	6	9.5	0	46.52
2 multi	2	H						
93	1	02/01/2020	23	2815	6	9.8	0	44.7
2 multi	2	H						
93	1	02/02/2020	24	2816	6	9.5	0.1	44.97
2 multi	2	H						
93	1	02/03/2020	25	2817	6	9.3	0	46.84
2 multi	2	H						
93	1	02/04/2020	26	2818	6	9.5	0.07	46.23
2 multi	2	H						
93	1	02/05/2020	27	2819	6	9.2	0.17	45.99
2 multi	2	H						
93	1	02/06/2020	28	2820	6	9.5	0.02	45.8
2 multi	2	H						
93	1	02/07/2020	29	2821	6	9.5	0.12	44.21
2 multi	2	H						
93	1	02/08/2020	30	2822	6	9.1	0.1	44.46
2 multi	2	H						
93	1	02/09/2020	31	2823	6	9.5	0.03	46.76
2 multi	2	H						
93	1	02/10/2020	32	2824	6	9.6	0.02	44.03
2 multi	2	H						
93	1	02/11/2020	33	2825	6	9.8	0	46.25
2 multi	2	H						
93	1	02/12/2020	34	2826	6	9.6	0.03	46.05
2 multi	2	H						
93	1	02/13/2020	35	2827	6	9.6	0.09	45.4
2 multi	2	H						
93	1	02/14/2020	36	2828	6	9.5	0	46
2 multi	2	H						
93	1	02/15/2020	37	2829	6	9.8	0.09	43.87
2 multi	2	H						
93	1	02/16/2020	38	2830	6	9.6	0.1	47.55
2 multi	2	H						
93	1	02/17/2020	39	2831	6	9.8	0.01	44.36
2 multi	2	H						
93	1	02/18/2020	40	2832	6	9.5	0.01	46.88

2 multi	2	H						
93	1	02/19/2020	41	2833	6	9.3	0	47.32
2 multi	2	H						
93	1	02/20/2020	42	2834	6	9.2	0	42.95
2 multi	2	H						
93	1	02/21/2020	43	2835	6	9.5	0	50.71
2 multi	2	H						
93	1	02/22/2020	44	2836	6	9.5	0	39.3
2 multi	2	H						
93	1	02/23/2020	45	2837	6	9.3	0	40.71
2 multi	2	H						
93	1	02/24/2020	46	2838	6	9.6	0.11	42.3
2 multi	2	H						
93	1	02/25/2020	47	2839	6	9.3	0.05	44.82
2 multi	2	H						
93	1	02/26/2020	48	2840	6	9.5	0.11	42.06
2 multi	2	H						
93	1	02/27/2020	49	2841	6	9.5	0	45.24
2 multi	2	H						
93	1	02/28/2020	50	2842	6	9.5	0	44.21
2 multi	2	H						
93	1	02/29/2020	51	2843	6	9.5	0.01	43.08
2 multi	2	H						
93	1	03/01/2020	52	2844	6	9.6	0.05	45.3
2 multi	2	H						
93	1	03/02/2020	53	2845	6	9.2	0	42.11
2 multi	2	H						
93	1	03/03/2020	54	2846	6	9.5	0.01	41.81
2 multi	2	H						
93	1	03/04/2020	55	2847	6	9.3	0	40.44
2 multi	2	H						
93	1	12/13/2020	5	3131	7	9.5	0.36	33.37
2 multi	1	H						
93	1	12/14/2020	6	3132	7	9.6	0.09	36.18
2 multi	1	H						
93	1	12/15/2020	7	3133	7	9.6	0	36.34
2 multi	1	H						
93	1	12/16/2020	8	3134	7	9.5	0	36.81
2 multi	1	H						
93	1	12/17/2020	9	3135	7	9.8	0.3	38.95
2 multi	1	H						
93	1	12/18/2020	10	3136	7	9.8	0.55	41.87
2 multi	1	H						
93	1	12/19/2020	11	3137	7	9.1	0.23	41
2 multi	1	H						
93	1	12/20/2020	12	3138	7	9.6	0.34	40.66
2 multi	1	H						
93	1	12/21/2020	13	3139	7	9.5	0.1	40.54
2 multi	1	H						
93	1	12/22/2020	14	3140	7	10	0.1	43.45
2 multi	1	H						
93	1	12/23/2020	15	3141	7	9.8	0.23	43.21
2 multi	1	H						
93	1	12/24/2020	16	3142	7	10	0.08	45.37

2 multi	1	H						
93	1	12/25/2020	17	3143	7	10.2	0.13	45.35
2 multi	1	H						
93	1	12/26/2020	18	3144	7	10.2	0.06	46.56
2 multi	1	H						
93	1	12/27/2020	19	3145	7	10.2	0.18	45.41
2 multi	1	H						
93	1	12/28/2020	20	3146	7	9.5	0.19	45.28
2 multi	1	H						
93	1	12/29/2020	21	3147	7	10	0.08	47.11
2 multi	1	H						
93	1	12/30/2020	22	3148	7	9.9	0.19	47.37
2 multi	2	H						
93	1	12/31/2020	23	3149	7	9.9	0.09	46.34
2 multi	2	H						
93	1	01/01/2021	24	3150	7	9.6	0.08	47.16
2 multi	2	H						
93	1	01/02/2021	25	3151	7	9.8	0.05	45.74
2 multi	2	H						
93	1	01/03/2021	26	3152	7	9.6	0.18	47.18
2 multi	2	H						
93	1	01/04/2021	27	3153	7	9.6	0.09	46.23
2 multi	2	H						
93	1	01/05/2021	28	3154	7	9.3	0.15	48.01
2 multi	2	H						
93	1	01/06/2021	29	3155	7	9.5	0.08	48.11
2 multi	2	H						
93	1	01/07/2021	30	3156	7	9.6	0.07	47.13
2 multi	2	H						
93	1	01/08/2021	31	3157	7	9.5	0.16	47.94
2 multi	2	H						
93	1	01/09/2021	32	3158	7	9.3	0.13	46.99
2 multi	2	H						
93	1	01/10/2021	33	3159	7	9.3	0.08	48.09
2 multi	2	H						
93	1	01/11/2021	34	3160	7	9.5	0	48.21
2 multi	2	H						
93	1	01/12/2021	35	3161	7	9.3	0.07	45.95
2 multi	2	H						
93	1	01/13/2021	36	3162	7	9.3	0.16	48.7
2 multi	2	H						
93	1	01/14/2021	37	3163	7	9.6	0.15	45.55
2 multi	2	H						
93	1	01/15/2021	38	3164	7	9.6	0.06	47.31
2 multi	2	H						
93	1	01/16/2021	39	3165	7	9.3	0.06	48.21
2 multi	2	H						
93	1	01/17/2021	40	3166	7	9.3	0.06	44.36
2 multi	2	H						
93	1	01/18/2021	41	3167	7	9.6	0	47.44
2 multi	2	H						
93	1	01/19/2021	42	3168	7	9.8	0.12	43.01
2 multi	2	H						
93	1	01/20/2021	43	3169	7	9.9	0.12	43.39

2 multi 2		H						
93	1	01/21/2021	44	3170	7	9.8	0.06	42.8
2 multi 2		H						
93	1	01/22/2021	45	3171	7	9.6	0.04	46.49
2 multi 2		H						
93	1	01/23/2021	46	3172	7	9.8	0.07	45.6
2 multi 2		H						
93	1	01/24/2021	47	3173	7	9.6	0.13	40.85
2 multi 2		H						
93	1	01/25/2021	48	3174	7	9.6	0.14	44.96
2 multi 2		H						
93	1	01/26/2021	49	3175	7	9.8	0.01	45.99
2 multi 2		H						
93	1	01/27/2021	50	3176	7	9.6	0.07	44.05
2 multi 2		H						
93	1	01/28/2021	51	3177	7	10	0	44.14
2 multi 2		H						
93	1	01/29/2021	52	3178	7	10	0.15	45.6
2 multi 2		H						
93	1	01/30/2021	53	3179	7	9.8	0	44.47
2 multi 2		H						
93	1	01/31/2021	54	3180	7	9.8	0.13	49.03
2 multi 2		H						
93	1	02/01/2021	55	3181	7	9.8	0.05	45.91
2 multi 2		H						
93	1	02/02/2021	56	3182	7	9.8	0.13	46.56
2 multi 2		H						
93	1	02/03/2021	57	3183	7	9.9	0.12	47.82
2 multi 2		H						
93	1	02/04/2021	58	3184	7	9.8	0	46.99
2 multi 2		H						
93	1	02/05/2021	59	3185	7	9.8	0.12	47.87
2 multi 2		H						
93	1	02/06/2021	60	3186	7	9.5	0.04	44.47
2 multi 2		H						
93	1	02/07/2021	61	3187	7	9.5	0.07	41.31
2 multi 2		H						
93	1	02/08/2021	62	3188	7	9.6	0.15	47.12
2 multi 2		H						
93	1	02/09/2021	63	3189	7	9.6	0	43.67
2 multi 2		H						
93	1	02/10/2021	64	3190	7	9.5	0.06	42.52
2 multi 2		H						
93	1	02/11/2021	65	3191	7	9.5	0.15	42.04
2 multi 2		H						
93	1	02/12/2021	66	3192	7	9.8	0.12	42.98
2 multi 2		H						
93	1	02/13/2021	67	3193	7	9.8	0.13	43.01
2 multi 2		H						
93	1	02/14/2021	68	3194	7	9.8	0	42.53
2 multi 2		H						
93	1	02/15/2021	69	3195	7	9.8	0.06	43.61
2 multi 2		H						
93	1	02/16/2021	70	3196	7	9.8	0.07	42.42

2 multi 2		H						
93	1	02/17/2021	71	3197	7	10.2	0.16	46.62
2 multi 2		H						
93	1	02/18/2021	72	3198	7	10.3	0	46.77
2 multi 2		H						
93	1	02/19/2021	73	3199	7	9.8	0.07	43.19
2 multi 2		H						
93	1	02/20/2021	74	3200	7	9.9	0.12	46.56
2 multi 2		H						
93	1	02/21/2021	75	3201	7	9.9	0.15	46.41
2 multi 2		H						
93	1	02/22/2021	76	3202	7	9.9	0	41.47
2 multi 2		H						
93	1	02/23/2021	77	3203	7	9.8	0.1	42.22
2 multi 2		H						
93	1	02/24/2021	78	3204	7	10	0.14	44.21
2 multi 2		H						
93	1	02/25/2021	79	3205	7	10	0.04	44.92
2 multi 2		H						
93	1	02/26/2021	80	3206	7	10.2	0	43.79
2 multi 2		H						
93	1	02/27/2021	81	3207	7	10	0	42.75
2 multi 2		H						
93	1	02/28/2021	82	3208	7	9.9	0.05	43.93
2 multi 2		H						
93	1	03/01/2021	83	3209	7	9.9	0.06	43.1
2 multi 2		H						
93	1	03/02/2021	84	3210	7	10.2	0.16	45.94
2 multi 2		H						
93	1	03/03/2021	85	3211	7	10	0.16	45.22
2 multi 2		H						
93	1	03/04/2021	86	3212	7	9.9	0.04	43.3
2 multi 2		H						
93	1	03/05/2021	87	3213	7	9.8	0	44.44
2 multi 2		H						
93	1	03/06/2021	88	3214	7	9.9	0	44.26
2 multi 2		H						
93	1	03/07/2021	89	3215	7	9.8	0.13	41.23
2 multi 2		H						
93	1	03/08/2021	90	3216	7	9.9	0	43.26
2 multi 2		H						
93	1	03/09/2021	91	3217	7	9.6	0.05	42.15
2 multi 2		H						
93	1	03/10/2021	92	3218	7	9.9	0.05	43.74
2 multi 2		H						
93	1	03/11/2021	93	3219	7	9.9	0.13	39.84
2 multi 2		H						
93	1	03/12/2021	94	3220	7	10.2	0	45.49
2 multi 2		H						
93	1	03/13/2021	95	3221	7	10.3	0.06	43.58
2 multi 2		H						
93	1	03/14/2021	96	3222	7	9.9	0.14	39.48
2 multi 2		H						
93	1	03/15/2021	97	3223	7	10.2	0.13	44.21

2 multi	2	H						
93	1	03/16/2021	98	3224	7	10.2	0.13	40.34
2 multi	2	H						
93	1	03/17/2021	99	3225	7	10.3	0.01	42.16
2 multi	2	H						
93	1	03/18/2021	100	3226	7	10.3	0.02	41.03
2 multi	2	H						
93	1	03/19/2021	101	3227	7	10	0	39.95
2 multi	2	H						
93	1	03/20/2021	102	3228	7	10	0	40.59
2 multi	2	H						
93	1	03/21/2021	103	3229	7	10	0	41.06
2 multi	2	H						
93	1	03/22/2021	104	3230	7	9.9	0.08	40.83
2 multi	2	H						
93	1	03/23/2021	105	3231	7	10	0.13	40.19
2 multi	2	H						
93	1	03/24/2021	106	3232	7	10.3	0.12	41.25
2 multi	2	H						
93	1	03/25/2021	107	3233	7	10	0.02	39.98
2 multi	2	H						
93	1	03/26/2021	108	3234	7	10.2	0.04	41.21
2 multi	2	H						
93	1	03/27/2021	109	3235	7	10	0.02	41.51
2 multi	2	H						
93	1	03/28/2021	110	3236	7	10.2	0.07	38.17
2 multi	2	H						
93	1	03/29/2021	111	3237	7	10.2	0	42.31
2 multi	2	H						
93	1	03/30/2021	112	3238	7	10.3	0	42.08
2 multi	2	H						
93	1	03/31/2021	113	3239	7	10.3	0.05	42.38
2 multi	2	H						
93	1	04/01/2021	114	3240	7	10.2	0	40.21
2 multi	2	H						
93	1	04/02/2021	115	3241	7	10.2	0.12	40.46
2 multi	2	H						
93	1	04/03/2021	116	3242	7	10.2	0.05	42.31
2 multi	2	H						
93	1	04/04/2021	117	3243	7	10.3	0	40.53
2 multi	2	H						
93	1	04/05/2021	118	3244	7	10.2	0.02	38.61
2 multi	2	H						
93	1	04/06/2021	119	3245	7	10.2	0.13	37.8
2 multi	2	H						
93	1	04/07/2021	120	3246	7	9.9	0.01	38.15
2 multi	2	H						
93	1	04/08/2021	121	3247	7	9.9	0	40.15
2 multi	2	H						
93	1	04/09/2021	122	3248	7	10.3	0.04	39.29
2 multi	2	H						
93	1	04/10/2021	123	3249	7	10.3	0.24	39.4
2 multi	2	H						
93	1	04/11/2021	124	3250	7	10.2	0.01	37.63

2 multi	2	H						
93	1	04/12/2021	125	3251	7	10.3	0.12	40.82
2 multi	2	H						
93	1	04/13/2021	126	3252	7	10.4	0.11	39.19
2 multi	2	H						
93	1	04/14/2021	127	3253	7	10.3	0	40.94
2 multi	2	H						
93	1	04/15/2021	128	3254	7	10.4	0	39.61
2 multi	2	H						
93	1	04/16/2021	129	3255	7	10.3	0.05	40.06
2 multi	2	H						
93	1	04/17/2021	130	3256	7	10.3	0.02	39.63
2 multi	2	H						
93	1	04/18/2021	131	3257	7	10.6	0	41.36
2 multi	2	H						
93	1	04/19/2021	132	3258	7	10.4	0	40.22
2 multi	2	H						
93	1	04/20/2021	133	3259	7	10.2	0.14	37.74
2 multi	2	H						
93	1	04/21/2021	134	3260	7	10.6	0.12	43.14
2 multi	2	H						
93	1	04/22/2021	135	3261	7	10.7	0.02	42.82
2 multi	2	H						
93	1	04/23/2021	136	3262	7	10.4	0.06	41.32
2 multi	2	H						
93	1	04/24/2021	137	3263	7	10.3	0.12	39.56
2 multi	2	H						
93	1	04/25/2021	138	3264	7	10.7	0	41.28
2 multi	2	H						
93	1	04/26/2021	139	3265	7	10.7	0	42.97
2 multi	2	H						
93	1	04/27/2021	140	3266	7	10.4	0	37.95
2 multi	2	H						
93	1	04/28/2021	141	3267	7	10.3	0	39.22
2 multi	2	H						
93	1	04/29/2021	142	3268	7	10.7	0	39.2
2 multi	2	H						
93	1	04/30/2021	143	3269	7	10.6	0	40.68
2 multi	2	H						
93	1	05/01/2021	144	3270	7	10.7	0.13	37.28
2 multi	2	H						
93	1	05/02/2021	145	3271	7	10.4	0.13	37.96
2 multi	2	H						
93	1	05/03/2021	146	3272	7	10	0.06	38.13
2 multi	2	H						
93	1	05/04/2021	147	3273	7	10.4	0.12	36.81
2 multi	2	H						
93	1	05/05/2021	148	3274	7	10.7	0.13	37.1
2 multi	2	H						
93	1	05/06/2021	149	3275	7	10.7	0.13	37.93
2 multi	2	H						
93	1	05/07/2021	150	3276	7	10.6	0.08	36.31
2 multi	2	H						
93	1	05/08/2021	151	3277	7	10.4	0.06	36.54

2 multi	2	H						
93	1	05/09/2021	152	3278	7	10.9	0.11	37.93
2 multi	2	H						
93	1	05/10/2021	153	3279	7	10.6	0.15	39.81
2 multi	2	H						
95	1	01/27/2020	5	722	1	9.2	0.25	20.23
1 primi	1	H						
95	1	01/28/2020	6	723	1	9.2	0.01	21.11
1 primi	1	H						
95	1	01/29/2020	7	724	1	9.1	0.28	21.13
1 primi	1	H						
95	1	01/30/2020	8	725	1	9.5	0.01	21.52
1 primi	1	H						
95	1	01/31/2020	9	726	1	9.2	0.11	21.38
1 primi	1	H						
95	1	02/01/2020	10	727	1	8.9	0	23.14
1 primi	1	H						
95	1	02/02/2020	11	728	1	8.9	0.1	22.78
1 primi	1	H						
95	1	02/03/2020	12	729	1	8.8	0	23.82
1 primi	1	H						
95	1	02/04/2020	13	730	1	8.9	0	24.37
1 primi	1	H						
95	1	02/05/2020	14	731	1	8.9	0	24.3
1 primi	1	H						
95	1	02/06/2020	15	732	1	8.9	0.08	25.09
1 primi	1	H						
95	1	02/07/2020	16	733	1	8.7	0.1	25.6
1 primi	1	H						
95	1	02/08/2020	17	734	1	8.9	0.07	25.44
1 primi	1	H						
95	1	02/09/2020	18	735	1	8.6	0	25.69
1 primi	1	H						
95	1	02/10/2020	19	736	1	8.7	0	26.25
1 primi	1	H						
95	1	02/11/2020	20	737	1	8.7	0.07	25.66
1 primi	1	H						
95	1	02/12/2020	21	738	1	8.7	0	26.63
1 primi	1	H						
95	1	02/13/2020	22	739	1	8.7	0.21	26.38
1 primi	2	H						
95	1	02/14/2020	23	740	1	9.1	0	27.23
1 primi	2	H						
95	1	02/15/2020	24	741	1	8.9	0	25.79
1 primi	2	H						
95	1	02/16/2020	25	742	1	8.8	0.08	24.94
1 primi	2	H						
95	1	02/17/2020	26	743	1	8.7	0	25.88
1 primi	2	H						
95	1	02/18/2020	27	744	1	8.7	0.07	26.1
1 primi	2	H						
95	1	02/19/2020	28	745	1	8.8	0.07	26.56
1 primi	2	H						
95	1	02/20/2020	29	746	1	9.2	0	26.45

1 primi	2	H						
95	1	02/21/2020	30	747	1	8.9	0	27.89
1 primi	2	H						
95	1	02/22/2020	31	748	1	8.8	0.05	24.99
1 primi	2	H						
95	1	02/23/2020	32	749	1	8.9	0	27.76
1 primi	2	H						
95	1	02/24/2020	33	750	1	8.8	0	27.55
1 primi	2	H						
95	1	02/25/2020	34	751	1	8.4	0	27.12
1 primi	2	H						
95	1	02/26/2020	35	752	1	8.9	0	28.11
1 primi	2	H						
95	1	02/27/2020	36	753	1	8.7	0.05	25.07
1 primi	2	H						
95	1	02/28/2020	37	754	1	9.1	0	24.62
1 primi	2	H						
95	1	02/29/2020	38	755	1	9.1	0	25.02
1 primi	2	H						
95	1	03/01/2020	39	756	1	9.2	0	26.13
1 primi	2	H						
95	1	03/02/2020	40	757	1	8.8	0	24.91
1 primi	2	H						
95	1	03/03/2020	41	758	1	9.3	0	26.72
1 primi	2	H						
95	1	03/04/2020	42	759	1	9.1	0.05	27.32
1 primi	2	H						
95	1	03/05/2020	43	760	1	9.2	0.06	26.67
1 primi	2	H						
95	1	03/06/2020	44	761	1	9.1	0.05	27.44
1 primi	2	H						
95	1	03/07/2020	45	762	1	9.1	0	27.16
1 primi	2	H						
95	1	03/08/2020	46	763	1	9.1	0	25.24
1 primi	2	H						
95	1	03/09/2020	47	764	1	9.1	0	27.09
1 primi	2	H						
95	1	03/10/2020	48	765	1	9.1	0	25.25
1 primi	2	H						
95	1	03/11/2020	49	766	1	8.8	0	27.32
1 primi	2	H						
95	1	03/12/2020	50	767	1	8.7	0	26.95
1 primi	2	H						
95	1	03/13/2020	51	768	1	9.1	0	26.79
1 primi	2	H						
95	1	03/14/2020	52	769	1	8.9	0.05	27.61
1 primi	2	H						
95	1	03/15/2020	53	770	1	8.9	0	28.85
1 primi	2	H						
95	1	03/16/2020	54	771	1	9.1	0.04	27.93
1 primi	2	H						
95	1	03/17/2020	55	772	1	9.2	0	29.36
1 primi	2	H						
95	1	03/18/2020	56	773	1	9.1	0	28.09

1 primi	2	H						
95	1	01/01/2021	8	1062	2	9.8	0.13	22.13
2 multi	1	H						
95	1	01/02/2021	9	1063	2	10.4	0.12	23.92
2 multi	1	H						
95	1	01/03/2021	10	1064	2	10	0.2	23.38
2 multi	1	H						
95	1	01/04/2021	11	1065	2	9.5	0.2	24.58
2 multi	1	H						
95	1	01/05/2021	12	1066	2	10.2	0.1	27.72
2 multi	1	H						
95	1	01/06/2021	13	1067	2	9.9	0.18	27.75
2 multi	1	H						
95	1	01/07/2021	14	1068	2	9.9	0.04	27.76
2 multi	1	H						
95	1	01/08/2021	15	1069	2	9.9	0.09	26.05
2 multi	1	H						
95	1	01/09/2021	16	1070	2	9.8	0.08	26.82
2 multi	1	H						
95	1	01/10/2021	17	1071	2	9.6	0.31	28.32
2 multi	1	H						
95	1	01/11/2021	18	1072	2	9.5	0.08	29.73
2 multi	1	H						
95	1	01/12/2021	19	1073	2	9.6	0.56	30.37
2 multi	1	H						
95	1	01/13/2021	20	1074	2	9.9	0.09	30.14
2 multi	1	H						
95	1	01/14/2021	21	1075	2	9.6	0.14	28.87
2 multi	1	H						
95	1	01/15/2021	22	1076	2	9.8	0	27.84
2 multi	2	H						
95	1	01/16/2021	23	1077	2	10	0.07	29.02
2 multi	2	H						
95	1	01/17/2021	24	1078	2	9.5	0.15	25.47
2 multi	2	H						
95	1	01/18/2021	25	1079	2	10.3	0.03	27.45
2 multi	2	H						
95	1	01/19/2021	26	1080	2	9.9	0.12	29.52
2 multi	2	H						
95	1	01/20/2021	27	1081	2	10.3	0.12	28.41
2 multi	2	H						
95	1	01/21/2021	28	1082	2	9.6	0.04	30.47
2 multi	2	H						
95	1	01/22/2021	29	1083	2	9.6	0.04	30.35
2 multi	2	H						
95	1	01/23/2021	30	1084	2	10.2	0.06	30.88
2 multi	2	H						
95	1	01/24/2021	31	1085	2	10.2	0	31.51
2 multi	2	H						
95	1	01/25/2021	32	1086	2	9.6	0.05	33.24
2 multi	2	H						
95	1	01/26/2021	33	1087	2	10.4	0.13	31.1
2 multi	2	H						
95	1	01/27/2021	34	1088	2	9.9	0.06	32.25

2 multi	2	H						
95	1	01/28/2021	35	1089	2	10	0.04	32.36
2 multi	2	H						
95	1	01/29/2021	36	1090	2	10.2	0.16	32.66
2 multi	2	H						
95	1	01/30/2021	37	1091	2	9.9	0	32.48
2 multi	2	H						
95	1	01/31/2021	38	1092	2	9.3	0.07	31.71
2 multi	2	H						
95	1	02/01/2021	39	1093	2	9.8	0	31.93
2 multi	2	H						
95	1	02/02/2021	40	1094	2	9.9	0.01	31.81
2 multi	2	H						
95	1	02/03/2021	41	1095	2	10.2	0.02	32.1
2 multi	2	H						
96	1	05/11/2021	12	681	1	9.5	0.34	23.2
1 primi	1	C						
96	1	05/12/2021	13	682	1	9.8	0.02	23.14
1 primi	1	C						
96	1	05/13/2021	14	683	1	9.8	0.01	24.39
1 primi	1	C						
96	1	05/14/2021	15	684	1	9.6	0.08	25.9
1 primi	1	C						
96	1	05/15/2021	16	685	1	9.5	0	26.82
1 primi	1	C						
96	1	05/16/2021	17	686	1	9.6	0	27.85
1 primi	1	C						
96	1	05/17/2021	18	687	1	9.5	0.02	28.73
1 primi	1	C						
96	1	05/18/2021	19	688	1	9.5	0	28.72
1 primi	1	C						
96	1	05/19/2021	20	689	1	9.2	0	27.03
1 primi	1	C						
96	1	05/20/2021	21	690	1	9.5	0.01	29.75
1 primi	1	C						
96	1	05/21/2021	22	691	1	9.3	0.01	29.94
1 primi	2	C						
96	1	05/22/2021	23	692	1	9.8	0.01	31.46
1 primi	2	C						
96	1	05/23/2021	24	693	1	9.5	0	32.18
1 primi	2	C						
96	1	05/24/2021	25	694	1	9.5	0.01	33.21
1 primi	2	C						
96	1	05/25/2021	26	695	1	9.6	0.01	33.42
1 primi	2	C						
96	1	05/26/2021	27	696	1	9.1	0	16.08
1 primi	2	C						
97	1	08/03/2014	4	720	1	12.3	0.08	22.34
1 primi	1	H						
97	1	08/04/2014	5	721	1	9.5	0	25.81
1 primi	1	H						
97	1	08/05/2014	6	722	1	9.8	0	24.72
1 primi	1	H						
97	1	08/06/2014	7	723	1	9.5	0.04	25.58

1 primi	1	H						
97	1	08/07/2014	8	724	1	9.3	0.01	22.85
1 primi	1	H						
97	1	08/08/2014	9	725	1	9.5	0	25.75
1 primi	1	H						
97	1	08/09/2014	10	726	1	9.8	0	27.95
1 primi	1	H						
97	1	08/10/2014	11	727	1	9.5	0	25.8
1 primi	1	H						
97	1	08/11/2014	12	728	1	9.5	0	28.18
1 primi	1	H						
97	1	08/12/2014	13	729	1	10	0.13	26.77
1 primi	1	H						
97	1	08/13/2014	14	730	1	10	0	28.1
1 primi	1	H						
97	1	08/14/2014	15	731	1	10.4	0	28.48
1 primi	1	H						
97	1	08/15/2014	16	732	1	9.5	0	28.87
1 primi	1	H						
97	1	08/16/2014	17	733	1	10.2	0.02	31.31
1 primi	1	H						
97	1	08/17/2014	18	734	1	10.2	0	32.87
1 primi	1	H						
97	1	08/18/2014	19	735	1	10.2	0	31.68
1 primi	1	H						
97	1	08/19/2014	20	736	1	9.8	0.21	30.38
1 primi	1	H						
97	1	08/20/2014	21	737	1	10.2	0	33.23
1 primi	1	H						
97	1	08/21/2014	22	738	1	10	0	33.55
1 primi	2	H						
97	1	08/22/2014	23	739	1	9.8	0.2	31.26
1 primi	2	H						
97	1	08/23/2014	24	740	1	9.6	0	34.49
1 primi	2	H						
97	1	08/24/2014	25	741	1	9.2	0.02	33.34
1 primi	2	H						
97	1	08/25/2014	26	742	1	9.8	0.03	32
1 primi	2	H						
97	1	08/26/2014	27	743	1	9.2	0.15	34.83
1 primi	2	H						
97	1	08/27/2014	28	744	1	8.6	0	34.15
1 primi	2	H						
97	1	08/28/2014	29	745	1	10.3	0	33.05
1 primi	2	H						
97	1	08/29/2014	30	746	1	9.1	0.25	33.56
1 primi	2	H						
97	1	08/30/2014	31	747	1	9.5	0	34.35
1 primi	2	H						
97	1	08/31/2014	32	748	1	9.8	0.05	36.63
1 primi	2	H						
97	1	09/01/2014	33	749	1	10	0	33.21
1 primi	2	H						
97	1	09/02/2014	34	750	1	9.6	0.12	34.02

1 primi	2	H						
97	1	09/03/2014	35	751	1	10	0.17	32.85
1 primi	2	H						
97	1	09/04/2014	36	752	1	9.9	0.05	30.92
1 primi	2	H						
97	1	09/05/2014	37	753	1	11.1	0	27.66
1 primi	2	H						
97	1	09/06/2014	38	754	1	9.8	0	31.75
1 primi	2	H						
97	1	09/07/2014	39	755	1	9.2	0	31.25
1 primi	2	H						
97	1	09/08/2014	40	756	1	9.5	0	33.07
1 primi	2	H						
97	1	09/09/2014	41	757	1	9.3	0	34.11
1 primi	2	H						
97	1	09/10/2014	42	758	1	9.9	0	33.58
1 primi	2	H						
97	1	09/11/2014	43	759	1	10.3	0	33.42
1 primi	2	H						
97	1	09/12/2014	44	760	1	9.5	0	32.7
1 primi	2	H						
97	1	09/13/2014	45	761	1	9.3	0	31.75
1 primi	2	H						
97	1	09/14/2014	46	762	1	9.1	0	33.97
1 primi	2	H						
97	1	09/15/2014	47	763	1	8.8	0	32.26
1 primi	2	H						
97	1	09/16/2014	48	764	1	9.5	0	29.27
1 primi	2	H						
97	1	09/17/2014	49	765	1	9.2	0.12	30.15
1 primi	2	H						
97	1	09/18/2014	50	766	1	9.5	0.06	30.84
1 primi	2	H						
97	1	09/19/2014	51	767	1	9.8	0	32.39
1 primi	2	H						
97	1	09/20/2014	52	768	1	9.2	0.05	30.24
1 primi	2	H						
97	1	09/21/2014	53	769	1	9.2	0.08	30.17
1 primi	2	H						
97	1	09/22/2014	54	770	1	9.8	0.1	31.3
1 primi	2	H						
97	1	09/23/2014	55	771	1	9.6	0.04	33.19
1 primi	2	H						
97	1	09/24/2014	56	772	1	9.3	0	33.3
1 primi	2	H						
97	1	09/25/2014	57	773	1	9.3	0.01	34.31
1 primi	2	H						
97	1	09/26/2014	58	774	1	9.1	0	33.75
1 primi	2	H						
97	1	09/27/2014	59	775	1	9.9	0	37.03
1 primi	2	H						
97	1	09/28/2014	60	776	1	9.5	0	36.32
1 primi	2	H						
97	1	09/29/2014	61	777	1	9.8	0	34.24

1 primi	2	H						
97	1	09/30/2014	62	778	1	8.9	0	31.27
1 primi	2	H						
97	1	10/01/2014	63	779	1	9.6	0.22	32.38
1 primi	2	H						
97	1	10/02/2014	64	780	1	9.1	0.38	33.13
1 primi	2	H						
97	1	10/03/2014	65	781	1	9.8	0.09	34.46
1 primi	2	H						
97	1	10/04/2014	66	782	1	9.8	0	33.17
1 primi	2	H						
97	1	10/05/2014	67	783	1	9.5	0.53	31.8
1 primi	2	H						
97	1	10/06/2014	68	784	1	9.6	0.18	34.79
1 primi	2	H						
97	1	10/07/2014	69	785	1	9.8	0.06	34.81
1 primi	2	H						
97	1	10/08/2014	70	786	1	9.8	0	33.97
1 primi	2	H						
97	1	10/09/2014	71	787	1	9.8	0	35.01
1 primi	2	H						
97	1	10/10/2014	72	788	1	10	0.18	34.24
1 primi	2	H						
97	1	10/11/2014	73	789	1	10.3	0	35.46
1 primi	2	H						
97	1	10/12/2014	74	790	1	9.8	0	36.03
1 primi	2	H						
97	1	10/13/2014	75	791	1	9.3	0	35.37
1 primi	2	H						
97	1	10/14/2014	76	792	1	9.3	0	32.1
1 primi	2	H						
97	1	10/15/2014	77	793	1	9.3	0.02	35.77
1 primi	2	H						
97	1	10/16/2014	78	794	1	9.1	0	32.92
1 primi	2	H						
97	1	10/17/2014	79	795	1	9.1	0	33.77
1 primi	2	H						
97	1	10/18/2014	80	796	1	9.2	0.07	32.82
1 primi	2	H						
97	1	10/19/2014	81	797	1	8.7	0	32.71
1 primi	2	H						
97	1	10/20/2014	82	798	1	8.9	0	34.78
1 primi	2	H						
97	1	10/21/2014	83	799	1	9.2	0	32.51
1 primi	2	H						
97	1	08/26/2015	6	1108	2	8.7	0.13	26.15
2 multi	1	H						
97	1	08/27/2015	7	1109	2	9.1	0.4	29.77
2 multi	1	H						
97	1	08/28/2015	8	1110	2	9.8	0.03	33.04
2 multi	1	H						
97	1	08/29/2015	9	1111	2	9.2	0.05	36.83
2 multi	1	H						
97	1	08/30/2015	10	1112	2	9.2	0.12	38.98

2 multi 1		H						
97	1	08/31/2015	11	1113	2	9.6	0	42.84
2 multi 1		H						
97	1	09/01/2015	12	1114	2	9.8	0.08	41.41
2 multi 1		H						
97	1	09/02/2015	13	1115	2	9.5	0.39	40.72
2 multi 1		H						
97	1	09/03/2015	14	1116	2	9.6	0.12	38.79
2 multi 1		H						
97	1	09/04/2015	15	1117	2	9.6	0	42.41
2 multi 1		H						
97	1	09/05/2015	16	1118	2	9.6	0.09	39.12
2 multi 1		H						
97	1	09/06/2015	17	1119	2	9.3	0.07	39.37
2 multi 1		H						
97	1	09/07/2015	18	1120	2	9.3	0	44.81
2 multi 1		H						
97	1	09/08/2015	19	1121	2	9.5	0.1	40.61
2 multi 1		H						
97	1	09/09/2015	20	1122	2	9.3	0.05	44.8
2 multi 1		H						
97	1	09/10/2015	21	1123	2	9.6	0.05	44.05
2 multi 1		H						
97	1	09/11/2015	22	1124	2	9.8	0	44.85
2 multi 2		H						
97	1	09/12/2015	23	1125	2	9.8	0.25	47.63
2 multi 2		H						
97	1	09/13/2015	24	1126	2	9.6	0	45.03
2 multi 2		H						
97	1	09/14/2015	25	1127	2	9.6	0	47.11
2 multi 2		H						
97	1	09/15/2015	26	1128	2	9.5	0	46.37
2 multi 2		H						
97	1	09/16/2015	27	1129	2	9.6	0	50.21
2 multi 2		H						
97	1	09/17/2015	28	1130	2	9.5	0.04	51.3
2 multi 2		H						
97	1	09/18/2015	29	1131	2	9.2	0.11	48.66
2 multi 2		H						
97	1	09/19/2015	30	1132	2	9.2	0.33	45.5
2 multi 2		H						
97	1	09/20/2015	31	1133	2	9.1	0.32	47.04
2 multi 2		H						
97	1	09/21/2015	32	1134	2	8.8	0.19	44.8
2 multi 2		H						
97	1	09/22/2015	33	1135	2	9.1	0.37	43.78
2 multi 2		H						
97	1	09/23/2015	34	1136	2	9.6	0.07	48.91
2 multi 2		H						
97	1	09/24/2015	35	1137	2	9.8	0.01	49.3
2 multi 2		H						
97	1	09/25/2015	36	1138	2	9.8	0	49.29
2 multi 2		H						
97	1	09/26/2015	37	1139	2	9.5	0	50.61

2 multi	2	H						
97	1	09/27/2015	38	1140	2	9.5	0.12	50.8
2 multi	2	H						
97	1	09/28/2015	39	1141	2	9.8	0.06	50.56
2 multi	2	H						
97	1	09/29/2015	40	1142	2	9.6	0.01	52.5
2 multi	2	H						
97	1	09/30/2015	41	1143	2	9.5	0.02	49.03
2 multi	2	H						
97	1	10/01/2015	42	1144	2	9.2	0.28	50.26
2 multi	2	H						
97	1	10/02/2015	43	1145	2	9.5	0.2	47.57
2 multi	2	H						
97	1	10/03/2015	44	1146	2	9.3	0.38	47.27
2 multi	2	H						
97	1	10/04/2015	45	1147	2	9.8	0.24	41.36
2 multi	2	H						
97	1	10/05/2015	46	1148	2	9.3	0.19	55.24
2 multi	2	H						
97	1	10/06/2015	47	1149	2	9.3	0.18	49.04
2 multi	2	H						
97	1	10/07/2015	48	1150	2	9.6	0.07	47.76
2 multi	2	H						
97	1	10/08/2015	49	1151	2	9.5	0.07	50.17
2 multi	2	H						
97	1	10/09/2015	50	1152	2	9.5	0.23	47.91
2 multi	2	H						
97	1	10/10/2015	51	1153	2	9.3	0.4	53.3
2 multi	2	H						
97	1	10/11/2015	52	1154	2	9.6	0.02	48.23
2 multi	2	H						
97	1	10/12/2015	53	1155	2	9.3	0.11	48.9
2 multi	2	H						
97	1	10/13/2015	54	1156	2	9.1	0.18	45.8
2 multi	2	H						
97	1	10/14/2015	55	1157	2	8.9	0.28	46.57
2 multi	2	H						
97	1	10/15/2015	56	1158	2	9.2	0.01	50.31
2 multi	2	H						
97	1	10/16/2015	57	1159	2	9.6	0.08	52.42
2 multi	2	H						
97	1	10/17/2015	58	1160	2	9.5	0.02	44.63
2 multi	2	H						
97	1	10/18/2015	59	1161	2	9.2	0.05	52.22
2 multi	2	H						
97	1	10/19/2015	60	1162	2	9.5	0.17	48.07
2 multi	2	H						
97	1	10/20/2015	61	1163	2	9.3	0.02	50
2 multi	2	H						
97	1	10/21/2015	62	1164	2	9.1	0	48.73
2 multi	2	H						
97	1	10/22/2015	63	1165	2	8.8	0.15	42.72
2 multi	2	H						
97	1	10/23/2015	64	1166	2	9.3	0	47.78

2 multi	2	H						
97	1	10/24/2015	65	1167	2	9.6	0.16	42.29
2 multi	2	H						
97	1	10/25/2015	66	1168	2	8.9	0	49.54
2 multi	2	H						
97	1	10/26/2015	67	1169	2	9.2	0.04	47.91
2 multi	2	H						
97	1	10/27/2015	68	1170	2	9.2	0.12	44.28
2 multi	2	H						
97	1	10/28/2015	69	1171	2	9.6	0.1	51.61
2 multi	2	H						
97	1	10/29/2015	70	1172	2	9.2	0.02	45.54
2 multi	2	H						
97	1	10/30/2015	71	1173	2	9.6	0.02	45.12
2 multi	2	H						
97	1	10/31/2015	72	1174	2	9.3	0.4	45.62
2 multi	2	H						
97	1	11/01/2015	73	1175	2	9.6	0.26	46.43
2 multi	2	H						
97	1	11/02/2015	74	1176	2	9.8	0.33	50.73
2 multi	2	H						
97	1	11/03/2015	75	1177	2	9.8	0.21	50.38
2 multi	2	H						
97	1	11/04/2015	76	1178	2	9.2	0	48.09
2 multi	2	H						
97	1	11/05/2015	77	1179	2	9.5	0	50.11
2 multi	2	H						
97	1	11/06/2015	78	1180	2	9.2	0.18	45.1
2 multi	2	H						
97	1	11/07/2015	79	1181	2	9.5	0	48.7
2 multi	2	H						
97	1	11/08/2015	80	1182	2	9.5	0.11	42.91
2 multi	2	H						
97	1	11/09/2015	81	1183	2	9.1	0.13	41.58
2 multi	2	H						
97	1	11/10/2015	82	1184	2	9.6	0.01	42.73
2 multi	2	H						
97	1	11/11/2015	83	1185	2	9.5	0	43.19
2 multi	2	H						
97	1	11/12/2015	84	1186	2	9.6	0.03	42.58
2 multi	2	H						
97	1	11/13/2015	85	1187	2	9.8	0.05	41.23
2 multi	2	H						
97	1	11/14/2015	86	1188	2	9.6	0.23	41.44
2 multi	2	H						
97	1	11/15/2015	87	1189	2	9.9	0	43.82
2 multi	2	H						
97	1	11/16/2015	88	1190	2	9.6	0.13	47.58
2 multi	2	H						
97	1	11/17/2015	89	1191	2	9.6	0.2	45.98
2 multi	2	H						
97	1	11/18/2015	90	1192	2	9.9	0.14	48.13
2 multi	2	H						
97	1	11/19/2015	91	1193	2	9.6	0	46.99

2 multi 2		H						
97	1	11/20/2015	92	1194	2	9.8	0.13	46.35
2 multi 2		H						
97	1	11/21/2015	93	1195	2	9.6	0	47.75
2 multi 2		H						
97	1	11/22/2015	94	1196	2	9.8	0	53.71
2 multi 2		H						
97	1	11/23/2015	95	1197	2	9.8	0.11	47.4
2 multi 2		H						
97	1	11/24/2015	96	1198	2	9.5	0	24.92
2 multi 2		H						
97	1	11/25/2015	97	1199	2	9.2	0	45.41
2 multi 2		H						
97	1	11/26/2015	98	1200	2	9.2	0	44.13
2 multi 2		H						
97	1	11/27/2015	99	1201	2	9.3	0	46.22
2 multi 2		H						
97	1	11/28/2015	100	1202	2	9.3	0.06	44.96
2 multi 2		H						
97	1	11/29/2015	101	1203	2	9.5	0	45.71
2 multi 2		H						
97	1	11/30/2015	102	1204	2	9.3	0	45.86
2 multi 2		H						
97	1	12/01/2015	103	1205	2	9.3	0.11	49.1
2 multi 2		H						
97	1	12/02/2015	104	1206	2	9.1	0	42.46
2 multi 2		H						
97	1	12/03/2015	105	1207	2	9.1	0.1	39.66
2 multi 2		H						
97	1	12/04/2015	106	1208	2	8.7	0	30.12
2 multi 2		H						
97	1	12/05/2015	107	1209	2	9.1	0	39.72
2 multi 2		H						
97	1	12/06/2015	108	1210	2	9.2	0	37.58
2 multi 2		H						
97	1	12/07/2015	109	1211	2	9.3	0	39.27
2 multi 2		H						
97	1	12/08/2015	110	1212	2	9.3	0	43.1
2 multi 2		H						
97	1	12/09/2015	111	1213	2	9.3	0	42.4
2 multi 2		H						
97	1	12/10/2015	112	1214	2	9.3	0	42.42
2 multi 2		H						
97	1	12/11/2015	113	1215	2	9.3	0	42.88
2 multi 2		H						
97	1	12/12/2015	114	1216	2	9.2	0	43.36
2 multi 2		H						
97	1	12/13/2015	115	1217	2	9.1	0.27	38.91
2 multi 2		H						
97	1	12/14/2015	116	1218	2	9.3	0	43.6
2 multi 2		H						
97	1	12/15/2015	117	1219	2	9.3	0	42.43
2 multi 2		H						
97	1	12/16/2015	118	1220	2	9.1	0	38.81

2 multi	2	H						
97	1	12/17/2015	119	1221	2	9.2	0	41.37
2 multi	2	H						
97	1	12/18/2015	120	1222	2	9.3	0	44.65
2 multi	2	H						
97	1	12/19/2015	121	1223	2	9.2	0.11	44.83
2 multi	2	H						
97	1	12/20/2015	122	1224	2	9.5	0	41.95
2 multi	2	H						
97	1	12/21/2015	123	1225	2	9.6	0.06	29.87
2 multi	2	H						
97	1	12/22/2015	124	1226	2	9.5	0	41.19
2 multi	2	H						
97	1	12/23/2015	125	1227	2	8.9	0	39.49
2 multi	2	H						
97	1	12/24/2015	126	1228	2	9.2	0.02	38.42
2 multi	2	H						
97	1	12/25/2015	127	1229	2	9.1	0	36.73
2 multi	2	H						
97	1	12/26/2015	128	1230	2	8.7	0	34.9
2 multi	2	H						
97	1	12/27/2015	129	1231	2	9.3	0	38
2 multi	2	H						
97	1	12/28/2015	130	1232	2	9.2	0	39.94
2 multi	2	H						
97	1	12/29/2015	131	1233	2	9.5	0	43.06
2 multi	2	H						
97	1	12/30/2015	132	1234	2	9.1	0	40.84
2 multi	2	H						
97	1	12/31/2015	133	1235	2	9.2	0	38.23
2 multi	2	H						
97	1	01/01/2016	134	1236	2	9.3	0.05	43.9
2 multi	2	H						
97	1	01/02/2016	135	1237	2	9.5	0	41.07
2 multi	2	H						
97	1	01/03/2016	136	1238	2	9.3	0.09	36.67
2 multi	2	H						
97	1	01/04/2016	137	1239	2	8.6	0.08	30.14
2 multi	2	H						
97	1	01/26/2017	140	1627	3	9.8	0	40.56
2 multi	2	H						
97	1	01/27/2017	141	1628	3	9.5	0.19	45.58
2 multi	2	H						
97	1	01/28/2017	142	1629	3	9.5	0.28	43.78
2 multi	2	H						
97	1	01/29/2017	143	1630	3	10.4	0	41.28
2 multi	2	H						
97	1	01/30/2017	144	1631	3	9.6	0.18	42.48
2 multi	2	H						
97	1	01/31/2017	145	1632	3	9.5	0	45.09
2 multi	2	H						
97	1	02/01/2017	146	1633	3	10	0.02	42.87
2 multi	2	H						
97	1	02/02/2017	147	1634	3	10.2	0.18	41.92

2 multi 2		H						
97	1	02/03/2017	148	1635	3	9.9	0.1	41.15
2 multi 2		H						
97	1	02/04/2017	149	1636	3	9.9	0	40.96
2 multi 2		H						
97	1	02/05/2017	150	1637	3	9.6	0.12	41.82
2 multi 2		H						
97	1	02/06/2017	151	1638	3	9.6	0.04	40.38
2 multi 2		H						
97	1	02/07/2017	152	1639	3	9.3	0.11	40.41
2 multi 2		H						
97	1	02/08/2017	153	1640	3	9.8	0.14	41.86
2 multi 2		H						
97	1	02/09/2017	154	1641	3	9.2	0	43.05
2 multi 2		H						
97	1	02/10/2017	155	1642	3	9.6	0.07	41.9
2 multi 2		H						
97	1	02/11/2017	156	1643	3	10.2	0	39.38
2 multi 2		H						
97	1	02/12/2017	157	1644	3	10	0	36.56
2 multi 2		H						
97	1	02/13/2017	158	1645	3	9.3	0	38.75
2 multi 2		H						
97	1	02/14/2017	159	1646	3	9.8	0	42.6
2 multi 2		H						
97	1	02/15/2017	160	1647	3	9.6	0	41.53
2 multi 2		H						
97	1	02/16/2017	161	1648	3	9.1	0	44.14
2 multi 2		H						
97	1	02/17/2017	162	1649	3	9.9	0.04	40.69
2 multi 2		H						
97	1	02/18/2017	163	1650	3	9.8	0.03	39.22
2 multi 2		H						
97	1	02/19/2017	164	1651	3	9.8	0.88	44.25
2 multi 2		H						
97	1	02/20/2017	165	1652	3	10	0	42.52
2 multi 2		H						
97	1	02/21/2017	166	1653	3	10	0.03	39.21
2 multi 2		H						
97	1	02/22/2017	167	1654	3	9.9	0	42.58
2 multi 2		H						
97	1	02/23/2017	168	1655	3	9.9	0	37.99
2 multi 2		H						
97	1	02/24/2017	169	1656	3	9.2	0.07	40.4
2 multi 2		H						
97	1	02/25/2017	170	1657	3	9.2	0	39.42
2 multi 2		H						
97	1	02/26/2017	171	1658	3	10.2	0	42.2
2 multi 2		H						
97	1	02/27/2017	172	1659	3	9.3	0	40.51
2 multi 2		H						
97	1	02/28/2017	173	1660	3	9.2	0.02	42.35
2 multi 2		H						
97	1	03/01/2017	174	1661	3	9.5	0	42.64

2 multi 2		H						
97	1	03/02/2017	175	1662	3	9.9	0.26	40.08
2 multi 2		H						
97	1	03/03/2017	176	1663	3	9.6	0	38.65
2 multi 2		H						
97	1	03/04/2017	177	1664	3	9.6	0	40.45
2 multi 2		H						
97	1	03/05/2017	178	1665	3	9.1	0.07	38.47
2 multi 2		H						
97	1	03/06/2017	179	1666	3	9.9	0	39.8
2 multi 2		H						
97	1	03/07/2017	180	1667	3	9.8	0	39.6
2 multi 2		H						
97	1	03/08/2017	181	1668	3	9.3	0.09	40.47
2 multi 2		H						
97	1	03/09/2017	182	1669	3	9.6	0.1	40.92
2 multi 2		H						
97	1	03/10/2017	183	1670	3	9.5	0.15	39.61
2 multi 2		H						
97	1	03/11/2017	184	1671	3	10.2	0	38.55
2 multi 2		H						
97	1	03/12/2017	185	1672	3	9.8	0.14	33.42
2 multi 2		H						
97	1	03/13/2017	186	1673	3	10	0	33.83
2 multi 2		H						
97	1	03/14/2017	187	1674	3	9.6	0.04	37.04
2 multi 2		H						
97	1	03/15/2017	188	1675	3	9.8	0	37.43
2 multi 2		H						
97	1	03/16/2017	189	1676	3	9.9	0	42.28
2 multi 2		H						
97	1	03/17/2017	190	1677	3	9.3	0	38.93
2 multi 2		H						
97	1	03/18/2017	191	1678	3	9.9	0	39.04
2 multi 2		H						
97	1	03/19/2017	192	1679	3	9.8	0	36.99
2 multi 2		H						
97	1	03/20/2017	193	1680	3	9.5	0.3	34.95
2 multi 2		H						
97	1	03/21/2017	194	1681	3	9.6	0	39.58
2 multi 2		H						
97	1	03/22/2017	195	1682	3	9.8	0	43.05
2 multi 2		H						
97	1	03/23/2017	196	1683	3	9.9	0	37.52
2 multi 2		H						
97	1	03/24/2017	197	1684	3	10	0.2	43.32
2 multi 2		H						
97	1	03/25/2017	198	1685	3	9.9	0.05	38.43
2 multi 2		H						
97	1	03/26/2017	199	1686	3	9.9	0	37.3
2 multi 2		H						
97	1	03/27/2017	200	1687	3	9.5	0	40.48
2 multi 2		H						
97	1	03/28/2017	201	1688	3	9.8	0	39.91

2 multi	2	H						
97	1	03/29/2017	202	1689	3	9.8	0	38.04
2 multi	2	H						
97	1	03/30/2017	203	1690	3	10.2	0	37.44
2 multi	2	H						
97	1	03/31/2017	204	1691	3	9.9	0	42.04
2 multi	2	H						
97	1	04/01/2017	205	1692	3	10.2	0	37.2
2 multi	2	H						
97	1	04/02/2017	206	1693	3	10.2	0	39.1
2 multi	2	H						
97	1	04/03/2017	207	1694	3	10.2	0	39.58
2 multi	2	H						
97	1	04/04/2017	208	1695	3	10.2	0	40.11
2 multi	2	H						
97	1	04/05/2017	209	1696	3	10.2	0.01	41.51
2 multi	2	H						
97	1	04/06/2017	210	1697	3	9.8	0	37.35
2 multi	2	H						
97	1	04/07/2017	211	1698	3	9.9	0.12	35.76
2 multi	2	H						
97	1	04/08/2017	212	1699	3	11.3	0.04	37.21
2 multi	2	H						
97	1	04/09/2017	213	1700	3	9.3	0	32.51
2 multi	2	H						
97	1	01/12/2018	4	1978	4	9.5	0.32	35.44
2 multi	1	H						
97	1	01/13/2018	5	1979	4	9.5	0.66	36.38
2 multi	1	H						
97	1	01/14/2018	6	1980	4	9.5	0.03	40.35
2 multi	1	H						
97	1	01/15/2018	7	1981	4	9.5	0.35	40.23
2 multi	1	H						
97	1	01/16/2018	8	1982	4	9.8	0.08	46.56
2 multi	1	H						
97	1	01/17/2018	9	1983	4	9.9	0.26	46.25
2 multi	1	H						
97	1	01/18/2018	10	1984	4	9.1	0.12	46.53
2 multi	1	H						
97	1	01/19/2018	11	1985	4	8.5	0.16	47.1
2 multi	1	H						
97	1	01/20/2018	12	1986	4	9.2	0.1	46.17
2 multi	1	H						
97	1	01/21/2018	13	1987	4	9.6	0.17	49.66
2 multi	1	H						
97	1	01/22/2018	14	1988	4	9.3	0.2	47.07
2 multi	1	H						
97	1	01/23/2018	15	1989	4	9.5	0.19	48.49
2 multi	1	H						
97	1	01/24/2018	16	1990	4	9.9	0.26	49.52
2 multi	1	H						
97	1	01/25/2018	17	1991	4	9.6	0.31	50.84
2 multi	1	H						
97	1	01/26/2018	18	1992	4	9.6	0.04	48.55

2 multi	1	H						
97	1	01/27/2018	19	1993	4	9.5	0.13	48.51
2 multi	1	H						
97	1	01/28/2018	20	1994	4	9.9	0.45	49.73
2 multi	1	H						
97	1	01/29/2018	21	1995	4	9.8	0.18	56.25
2 multi	1	H						
97	1	01/30/2018	22	1996	4	9.6	0.36	51.74
2 multi	2	H						
97	1	01/31/2018	23	1997	4	10	0.71	58.35
2 multi	2	H						
97	1	02/01/2018	24	1998	4	9.8	0.14	52.92
2 multi	2	H						
97	1	02/02/2018	25	1999	4	9.5	0.09	56.13
2 multi	2	H						
97	1	02/03/2018	26	2000	4	9.8	0.15	55.7
2 multi	2	H						
97	1	02/04/2018	27	2001	4	9.5	0.36	56.59
2 multi	2	H						
97	1	02/05/2018	28	2002	4	9.8	0.37	57.92
2 multi	2	H						
97	1	02/06/2018	29	2003	4	9.6	0.15	57.6
2 multi	2	H						
97	1	02/07/2018	30	2004	4	9.6	0.18	57.75
2 multi	2	H						
97	1	02/08/2018	31	2005	4	9.9	0.23	59.71
2 multi	2	H						
97	1	02/09/2018	32	2006	4	9.9	0.06	59.79
2 multi	2	H						
97	1	02/10/2018	33	2007	4	9.8	0.04	61.21
2 multi	2	H						
97	1	02/11/2018	34	2008	4	9.5	0.07	58.37
2 multi	2	H						
97	1	02/12/2018	35	2009	4	9.5	0	55.87
2 multi	2	H						
97	1	02/13/2018	36	2010	4	9.5	0.22	54.34
2 multi	2	H						
97	1	02/14/2018	37	2011	4	9.8	0.11	60.26
2 multi	2	H						
97	1	02/15/2018	38	2012	4	9.6	0	57.79
2 multi	2	H						
97	1	02/16/2018	39	2013	4	9.6	0.07	59.4
2 multi	2	H						
97	1	02/17/2018	40	2014	4	9.9	0.08	59.46
2 multi	2	H						
97	1	02/18/2018	41	2015	4	9.8	0	59.55
2 multi	2	H						
97	1	02/19/2018	42	2016	4	9.5	0	57.55
2 multi	2	H						
97	1	02/20/2018	43	2017	4	9.8	0.23	56.03
2 multi	2	H						
97	1	02/21/2018	44	2018	4	9.9	0.2	60.5
2 multi	2	H						
97	1	02/22/2018	45	2019	4	9.5	0.16	61.01

2 multi 2		H						
97	1	02/23/2018	46	2020	4	9.1	0.16	53.13
2 multi 2		H						
97	1	02/24/2018	47	2021	4	9.6	0.37	57.06
2 multi 2		H						
97	1	02/25/2018	48	2022	4	9.3	0.5	56.88
2 multi 2		H						
97	1	02/26/2018	49	2023	4	9.3	0.51	59.51
2 multi 2		H						
97	1	02/27/2018	50	2024	4	9.6	0.22	58.17
2 multi 2		H						
97	1	02/28/2018	51	2025	4	9.1	0.05	58.61
2 multi 2		H						
97	1	03/01/2018	52	2026	4	9.3	0.19	55.91
2 multi 2		H						
97	1	03/02/2018	53	2027	4	9.3	0.15	57.09
2 multi 2		H						
97	1	03/03/2018	54	2028	4	9.6	0.04	57.65
2 multi 2		H						
97	1	03/04/2018	55	2029	4	9.8	0	61.32
2 multi 2		H						
97	1	03/05/2018	56	2030	4	9.6	0.17	54.66
2 multi 2		H						
97	1	03/06/2018	57	2031	4	10.4	0.25	52.79
2 multi 2		H						
97	1	03/07/2018	58	2032	4	9.2	0.23	37.81
2 multi 2		H						
97	1	03/08/2018	59	2033	4	10	0	39.94
2 multi 2		H						
97	1	03/09/2018	60	2034	4	9.8	0.4	40.27
2 multi 2		H						
97	1	03/10/2018	61	2035	4	9.8	0.18	46.83
2 multi 2		H						
97	1	03/11/2018	62	2036	4	10.2	0.08	53.8
2 multi 2		H						
97	1	03/12/2018	63	2037	4	10.4	0.02	51.52
2 multi 2		H						
97	1	03/13/2018	64	2038	4	11.9	0.24	46.9
2 multi 2		H						
97	1	03/14/2018	65	2039	4	9.8	0.17	48.16
2 multi 2		H						
97	1	03/15/2018	66	2040	4	10.6	0	52.42
2 multi 2		H						
97	1	03/16/2018	67	2041	4	10.6	0.19	48.73
2 multi 2		H						
97	1	03/17/2018	68	2042	4	10.3	0.18	48.25
2 multi 2		H						
97	1	03/18/2018	69	2043	4	10	0.05	54.14
2 multi 2		H						
97	1	07/29/2018	202	2176	4	10.2	0.08	38.76
2 multi 2		H						
97	1	07/30/2018	203	2177	4	10	0.26	34.58
2 multi 2		H						
97	1	07/31/2018	204	2178	4	9.9	0.17	38.14

2 multi	2	H						
97	1	08/01/2018	205	2179	4	10.3	0.13	38.66
2 multi	2	H						
97	1	08/02/2018	206	2180	4	10.3	0.05	41.6
2 multi	2	H						
97	1	08/03/2018	207	2181	4	10.3	0	37.15
2 multi	2	H						
97	1	08/04/2018	208	2182	4	9.8	0.05	37.7
2 multi	2	H						
97	1	08/05/2018	209	2183	4	10	0.19	39.62
2 multi	2	H						
97	1	08/06/2018	210	2184	4	10.2	0.13	40.8
2 multi	2	H						
97	1	08/07/2018	211	2185	4	10.3	0.05	41.17
2 multi	2	H						
97	1	08/08/2018	212	2186	4	10	0.3	38.8
2 multi	2	H						
97	1	08/09/2018	213	2187	4	10.2	0.13	45.39
2 multi	2	H						
97	1	08/10/2018	214	2188	4	10	0	42.95
2 multi	2	H						
97	1	08/11/2018	215	2189	4	10.2	0.06	42.37
2 multi	2	H						
97	1	08/12/2018	216	2190	4	10.2	0.24	40.42
2 multi	2	H						
97	1	08/13/2018	217	2191	4	10.3	0	40.8
2 multi	2	H						
97	1	08/14/2018	218	2192	4	10.2	0.13	42.47
2 multi	2	H						
97	1	08/15/2018	219	2193	4	10.2	0.06	41.05
2 multi	2	H						
97	1	08/16/2018	220	2194	4	10.2	0.21	43.29
2 multi	2	H						
97	1	08/17/2018	221	2195	4	10	0.05	36.19
2 multi	2	H						
97	1	08/18/2018	222	2196	4	9.9	0.11	39.51
2 multi	2	H						
97	1	08/19/2018	223	2197	4	10	0	43.06
2 multi	2	H						
97	1	08/20/2018	224	2198	4	9.9	0.32	38.9
2 multi	2	H						
97	1	08/21/2018	225	2199	4	10.2	0	39.34
2 multi	2	H						
97	1	08/22/2018	226	2200	4	10.3	0.07	40.64
2 multi	2	H						
97	1	08/23/2018	227	2201	4	10	0	35.7
2 multi	2	H						
97	1	08/24/2018	228	2202	4	10	0	40.06
2 multi	2	H						
97	1	08/25/2018	229	2203	4	10.4	0.24	36.77
2 multi	2	H						
97	1	08/26/2018	230	2204	4	10.3	0.07	40.36
2 multi	2	H						
97	1	08/27/2018	231	2205	4	10.2	0	37.22

2 multi 2		H						
97	1	08/28/2018	232	2206	4	10.3	0	41.59
2 multi 2		H						
97	1	08/29/2018	233	2207	4	10.3	0.05	40.79
2 multi 2		H						
97	1	08/30/2018	234	2208	4	10.2	0.06	41.06
2 multi 2		H						
97	1	08/31/2018	235	2209	4	10.2	0.13	39.31
2 multi 2		H						
97	1	09/01/2018	236	2210	4	10	0	41.68
2 multi 2		H						
97	1	09/02/2018	237	2211	4	10.2	0.53	45.46
2 multi 2		H						
97	1	09/03/2018	238	2212	4	10	0.2	41.74
2 multi 2		H						
97	1	09/04/2018	239	2213	4	10	0.23	41.26
2 multi 2		H						
97	1	09/05/2018	240	2214	4	10.2	0.08	43.55
2 multi 2		H						
97	1	09/06/2018	241	2215	4	10.2	0	40.94
2 multi 2		H						
97	1	09/07/2018	242	2216	4	10	0	42.36
2 multi 2		H						
97	1	09/08/2018	243	2217	4	10.2	0	38.33
2 multi 2		H						
97	1	09/09/2018	244	2218	4	10.2	0.26	38.48
2 multi 2		H						
97	1	09/10/2018	245	2219	4	9.8	0.15	36.06
2 multi 2		H						
97	1	09/11/2018	246	2220	4	10	0.06	34.62
2 multi 2		H						
97	1	09/12/2018	247	2221	4	10	0.11	36.32
2 multi 2		H						
97	1	09/13/2018	248	2222	4	10.3	0.25	37.42
2 multi 2		H						
97	1	09/14/2018	249	2223	4	10.2	0.23	36.01
2 multi 2		H						
97	1	09/15/2018	250	2224	4	10.2	0	37.04
2 multi 2		H						
97	1	09/16/2018	251	2225	4	9.9	0	35.8
2 multi 2		H						
97	1	09/17/2018	252	2226	4	10.2	0.02	37.31
2 multi 2		H						
97	1	09/18/2018	253	2227	4	10.4	0.13	35.56
2 multi 2		H						
97	1	09/19/2018	254	2228	4	10.7	0.01	37.12
2 multi 2		H						
97	1	09/20/2018	255	2229	4	10.9	0.2	35.73
2 multi 2		H						
97	1	09/21/2018	256	2230	4	11.7	0.24	36.32
2 multi 2		H						
97	1	09/22/2018	257	2231	4	10.2	0.08	34.4
2 multi 2		H						
97	1	09/23/2018	258	2232	4	10.4	0.08	34.2

2 multi 2		H						
97	1	09/24/2018	259	2233	4	10.6	0.11	32.25
2 multi 2		H						
97	1	09/25/2018	260	2234	4	10.9	0.14	38.1
2 multi 2		H						
97	1	09/26/2018	261	2235	4	10.6	0.03	36.03
2 multi 2		H						
97	1	09/27/2018	262	2236	4	10.4	0.09	37.56
2 multi 2		H						
97	1	12/08/2018	7	2308	5	9.5	0.2	39.07
2 multi 1		H						
97	1	12/09/2018	8	2309	5	9.1	0.04	38.29
2 multi 1		H						
97	1	12/10/2018	9	2310	5	9.9	0.22	48.77
2 multi 1		H						
97	1	12/11/2018	10	2311	5	10	0	48.42
2 multi 1		H						
97	1	12/12/2018	11	2312	5	9.8	0.12	47.2
2 multi 1		H						
97	1	12/13/2018	12	2313	5	9.5	0.22	47.16
2 multi 1		H						
97	1	12/14/2018	13	2314	5	9.2	0	45.33
2 multi 1		H						
97	1	12/15/2018	14	2315	5	9.6	0.11	52.16
2 multi 1		H						
97	1	12/16/2018	15	2316	5	9.5	0.23	46.05
2 multi 1		H						
97	1	12/17/2018	16	2317	5	9.6	0.14	46.82
2 multi 1		H						
97	1	12/18/2018	17	2318	5	9.6	0.12	49.1
2 multi 1		H						
97	1	12/19/2018	18	2319	5	9.6	0.13	48.2
2 multi 1		H						
97	1	12/20/2018	19	2320	5	9.8	0.05	52.18
2 multi 1		H						
97	1	12/21/2018	20	2321	5	9.8	0.1	48.77
2 multi 1		H						
97	1	12/22/2018	21	2322	5	9.9	0	51.4
2 multi 1		H						
97	1	12/23/2018	22	2323	5	9.8	0.08	52.28
2 multi 2		H						
97	1	12/24/2018	23	2324	5	9.6	0.1	45.39
2 multi 2		H						
97	1	12/25/2018	24	2325	5	9.9	0.11	56.7
2 multi 2		H						
97	1	12/26/2018	25	2326	5	9.9	0.1	50.21
2 multi 2		H						
97	1	12/27/2018	26	2327	5	9.8	0.11	50.64
2 multi 2		H						
97	1	12/28/2018	27	2328	5	9.8	0.19	52.49
2 multi 2		H						
97	1	12/29/2018	28	2329	5	9.6	0.13	52.4
2 multi 2		H						
97	1	12/30/2018	29	2330	5	9.5	0.08	48.87

2 multi 2		H						
97	1	12/31/2018	30	2331	5	9.6	0.1	53.12
2 multi 2		H						
97	1	01/01/2019	31	2332	5	9.8	0.09	53.93
2 multi 2		H						
97	1	01/02/2019	32	2333	5	9.6	0	51.08
2 multi 2		H						
97	1	01/03/2019	33	2334	5	9.5	0.05	51.82
2 multi 2		H						
97	1	01/04/2019	34	2335	5	9.8	0	49.5
2 multi 2		H						
97	1	01/05/2019	35	2336	5	9.8	0.02	50.27
2 multi 2		H						
97	1	01/06/2019	36	2337	5	9.3	0.09	46.7
2 multi 2		H						
97	1	01/07/2019	37	2338	5	9.9	0.31	54.04
2 multi 2		H						
97	1	01/08/2019	38	2339	5	9.9	0	53.86
2 multi 2		H						
97	1	01/09/2019	39	2340	5	9.8	0	51.59
2 multi 2		H						
97	1	01/10/2019	40	2341	5	9.9	0.18	51.65
2 multi 2		H						
97	1	01/11/2019	41	2342	5	9.5	0	48.44
2 multi 2		H						
97	1	01/12/2019	42	2343	5	9.8	0	51.59
2 multi 2		H						
97	1	01/13/2019	43	2344	5	9.5	0.1	49.6
2 multi 2		H						
97	1	01/14/2019	44	2345	5	9.5	0.07	49.83
2 multi 2		H						
97	1	01/15/2019	45	2346	5	9.2	0	44.93
2 multi 2		H						
97	1	01/16/2019	46	2347	5	9.9	0.05	53.45
2 multi 2		H						
97	1	01/17/2019	47	2348	5	10.2	0.09	52.08
2 multi 2		H						
97	1	01/18/2019	48	2349	5	9.9	0.28	52.35
2 multi 2		H						
97	1	01/19/2019	49	2350	5	9.8	0.16	47.98
2 multi 2		H						
97	1	01/20/2019	50	2351	5	9.8	0	49.09
2 multi 2		H						
97	1	01/21/2019	51	2352	5	9.8	0.21	54.48
2 multi 2		H						
97	1	01/22/2019	52	2353	5	9.5	0.12	48.17
2 multi 2		H						
97	1	01/23/2019	53	2354	5	9.5	0.07	50.33
2 multi 2		H						
97	1	01/24/2019	54	2355	5	9.5	0.26	49.94
2 multi 2		H						
97	1	01/25/2019	55	2356	5	9.8	0	53.77
2 multi 2		H						
97	1	01/26/2019	56	2357	5	9.3	0	46.28

2 multi	2	H						
97	1	01/27/2019	57	2358	5	9.6	0	54.77
2 multi	2	H						
97	1	01/28/2019	58	2359	5	9.6	0.12	49.25
2 multi	2	H						
97	1	01/29/2019	59	2360	5	9.6	0.1	54.52
2 multi	2	H						
97	1	01/30/2019	60	2361	5	9.6	0	53.54
2 multi	2	H						
97	1	01/31/2019	61	2362	5	9.5	0.15	52.06
2 multi	2	H						
97	1	02/01/2019	62	2363	5	9.3	0	52.49
2 multi	2	H						
97	1	02/02/2019	63	2364	5	9.2	0.01	43.46
2 multi	2	H						
97	1	02/03/2019	64	2365	5	9.5	0.1	48.7
2 multi	2	H						
97	1	02/04/2019	65	2366	5	9.5	0.08	50.53
2 multi	2	H						
97	1	02/05/2019	66	2367	5	9.6	0	49.2
2 multi	2	H						
97	1	02/06/2019	67	2368	5	9.8	0.11	49.42
2 multi	2	H						
97	1	02/07/2019	68	2369	5	9.5	0.24	45.56
2 multi	2	H						
97	1	02/08/2019	69	2370	5	9.6	0.15	49.58
2 multi	2	H						
97	1	02/09/2019	70	2371	5	9.5	0.48	49.3
2 multi	2	H						
97	1	02/10/2019	71	2372	5	9.9	0.33	51.87
2 multi	2	H						
97	1	02/11/2019	72	2373	5	9.6	0.32	51.17
2 multi	2	H						
97	1	02/12/2019	73	2374	5	9.5	0.17	50.93
2 multi	2	H						
97	1	02/13/2019	74	2375	5	9.5	0.11	53.09
2 multi	2	H						
97	1	02/14/2019	75	2376	5	9.6	0.03	52.68
2 multi	2	H						
97	1	02/15/2019	76	2377	5	9.6	0.01	52.1
2 multi	2	H						
97	1	02/16/2019	77	2378	5	9.3	0.09	50.51
2 multi	2	H						
97	1	02/17/2019	78	2379	5	9.3	0	52
2 multi	2	H						
97	1	02/18/2019	79	2380	5	9.3	0.22	48.02
2 multi	2	H						
97	1	02/19/2019	80	2381	5	9.6	0.1	51.58
2 multi	2	H						
97	1	02/20/2019	81	2382	5	9.5	0	49.92
2 multi	2	H						
97	1	02/21/2019	82	2383	5	9.8	0.01	47.36
2 multi	2	H						
97	1	02/22/2019	83	2384	5	9.5	0.03	52.32

2 multi	2	H						
97	1	02/23/2019	84	2385	5	9.5	0	47.52
2 multi	2	H						
97	1	02/24/2019	85	2386	5	9.8	0.23	50.86
2 multi	2	H						
97	1	02/25/2019	86	2387	5	9.6	0.1	46.02
2 multi	2	H						
97	1	02/26/2019	87	2388	5	9.6	0.36	47.67
2 multi	2	H						
97	1	02/27/2019	88	2389	5	9.6	0.14	45.27
2 multi	2	H						
97	1	02/28/2019	89	2390	5	9.6	0.14	52.52
2 multi	2	H						
97	1	03/01/2019	90	2391	5	9.6	0.23	49.13
2 multi	2	H						
97	1	03/02/2019	91	2392	5	9.1	0.18	41.86
2 multi	2	H						
97	1	03/03/2019	92	2393	5	9.3	0.13	47.27
2 multi	2	H						
97	1	03/04/2019	93	2394	5	9.6	0.16	51.31
2 multi	2	H						
97	1	03/05/2019	94	2395	5	9.8	0	46.97
2 multi	2	H						
97	1	03/06/2019	95	2396	5	9.6	0	47.24
2 multi	2	H						
97	1	03/07/2019	96	2397	5	9.6	0	48.72
2 multi	2	H						
97	1	03/08/2019	97	2398	5	10.2	0.16	52.05
2 multi	2	H						
97	1	03/09/2019	98	2399	5	9.8	0.15	49.48
2 multi	2	H						
97	1	03/10/2019	99	2400	5	9.3	0	45.12
2 multi	2	H						
97	1	03/11/2019	100	2401	5	9.1	0.21	43.39
2 multi	2	H						
97	1	03/12/2019	101	2402	5	9.5	0.07	47.3
2 multi	2	H						
97	1	03/13/2019	102	2403	5	9.6	0	47.24
2 multi	2	H						
97	1	03/14/2019	103	2404	5	9.6	0.06	49.82
2 multi	2	H						
97	1	03/15/2019	104	2405	5	9.8	0.09	50.73
2 multi	2	H						
97	1	03/16/2019	105	2406	5	9.5	0.09	47.44
2 multi	2	H						
97	1	03/17/2019	106	2407	5	9.8	0	50.79
2 multi	2	H						
97	1	03/18/2019	107	2408	5	9.8	0	52.17
2 multi	2	H						
97	1	03/19/2019	108	2409	5	9.8	0	51.46
2 multi	2	H						
97	1	03/20/2019	109	2410	5	9.9	0.03	53.74
2 multi	2	H						
97	1	03/21/2019	110	2411	5	9.5	0.15	46.49

2 multi	2	H						
97	1	03/22/2019	111	2412	5	9.6	0.08	51.53
2 multi	2	H						
97	1	03/23/2019	112	2413	5	9.8	0	51.19
2 multi	2	H						
97	1	03/24/2019	113	2414	5	9.5	0.16	47.95
2 multi	2	H						
97	1	03/25/2019	114	2415	5	9.6	0.18	50.14
2 multi	2	H						
97	1	03/26/2019	115	2416	5	9.5	0.22	48.98
2 multi	2	H						
97	1	03/27/2019	116	2417	5	9.1	0.23	42.39
2 multi	2	H						
97	1	03/28/2019	117	2418	5	8.9	0.1	37.93
2 multi	2	H						
97	1	03/29/2019	118	2419	5	9.6	0.08	41.38
2 multi	2	H						
97	1	03/30/2019	119	2420	5	9.5	0.14	44.24
2 multi	2	H						
97	1	03/31/2019	120	2421	5	9.9	0.03	47.83
2 multi	2	H						
97	1	04/01/2019	121	2422	5	9.8	0.24	50.42
2 multi	2	H						
97	1	04/02/2019	122	2423	5	9.9	0.24	48.78
2 multi	2	H						
97	1	04/03/2019	123	2424	5	9.9	0.08	48.4
2 multi	2	H						
97	1	04/04/2019	124	2425	5	9.9	0	46.51
2 multi	2	H						
97	1	04/05/2019	125	2426	5	9.9	0.16	47.71
2 multi	2	H						
97	1	04/06/2019	126	2427	5	9.6	0	44.08
2 multi	2	H						
97	1	04/07/2019	127	2428	5	9.6	0.1	42.85
2 multi	2	H						
97	1	04/08/2019	128	2429	5	9.8	0.25	43.4
2 multi	2	H						
97	1	04/09/2019	129	2430	5	9.8	0.26	42.7
2 multi	2	H						
97	1	04/10/2019	130	2431	5	9.6	0	42.12
2 multi	2	H						
97	1	04/11/2019	131	2432	5	9.8	0.09	44.7
2 multi	2	H						
97	1	04/12/2019	132	2433	5	9.9	0.15	44.12
2 multi	2	H						
97	1	04/13/2019	133	2434	5	9.8	0	45.03
2 multi	2	H						
97	1	04/14/2019	134	2435	5	9.8	0	46.26
2 multi	2	H						
97	1	04/15/2019	135	2436	5	9.9	0	47.11
2 multi	2	H						
97	1	04/16/2019	136	2437	5	9.2	0.25	44.24
2 multi	2	H						
97	1	04/17/2019	137	2438	5	9.8	0	46.28

2 multi 2		H						
97	1	04/18/2019	138	2439	5	9.6	0.09	48.2
2 multi 2		H						
97	1	04/19/2019	139	2440	5	9.6	0.24	46.66
2 multi 2		H						
97	1	04/20/2019	140	2441	5	9.8	0	48.32
2 multi 2		H						
97	1	04/21/2019	141	2442	5	9.9	0.23	50.88
2 multi 2		H						
97	1	04/22/2019	142	2443	5	9.9	0	49.21
2 multi 2		H						
97	1	04/23/2019	143	2444	5	9.6	0.28	47.15
2 multi 2		H						
97	1	04/24/2019	144	2445	5	9.9	0	50.18
2 multi 2		H						
97	1	04/25/2019	145	2446	5	9.6	0	48.72
2 multi 2		H						
97	1	04/26/2019	146	2447	5	9.9	0.07	50.86
2 multi 2		H						
97	1	04/27/2019	147	2448	5	9.9	0	49.14
2 multi 2		H						
97	1	04/28/2019	148	2449	5	9.8	0.28	48.54
2 multi 2		H						
97	1	04/29/2019	149	2450	5	9.8	0	48.92
2 multi 2		H						
97	1	04/30/2019	150	2451	5	9.9	0	49.34
2 multi 2		H						
97	1	05/01/2019	151	2452	5	9.9	0.23	46.23
2 multi 2		H						
97	1	05/02/2019	152	2453	5	9.9	0	49.09
2 multi 2		H						
97	1	05/03/2019	153	2454	5	9.8	0.01	47.92
2 multi 2		H						
97	1	05/04/2019	154	2455	5	9.6	0	46.02
2 multi 2		H						
97	1	05/05/2019	155	2456	5	9.9	0.08	47.99
2 multi 2		H						
97	1	05/06/2019	156	2457	5	10	0.25	46.05
2 multi 2		H						
97	1	05/07/2019	157	2458	5	10	0.25	47.74
2 multi 2		H						
97	1	05/08/2019	158	2459	5	9.9	0.09	45.69
2 multi 2		H						
97	1	05/09/2019	159	2460	5	10.2	0	50.41
2 multi 2		H						
97	1	05/10/2019	160	2461	5	10.2	0	43.96
2 multi 2		H						
97	1	05/11/2019	161	2462	5	9.2	0	46.44
2 multi 2		H						
97	1	05/12/2019	162	2463	5	9.3	0.09	45.1
2 multi 2		H						
97	1	05/13/2019	163	2464	5	9.8	0.13	45.55
2 multi 2		H						
97	1	05/14/2019	164	2465	5	9.6	0.16	44.11

2 multi	2	H						
97	1	05/15/2019	165	2466	5	9.8	0.08	47.98
2 multi	2	H						
97	1	05/16/2019	166	2467	5	9.5	0.06	40.8
2 multi	2	H						
97	1	05/17/2019	167	2468	5	9.6	0	43.94
2 multi	2	H						
97	1	05/18/2019	168	2469	5	9.6	0.13	45.58
2 multi	2	H						
97	1	05/19/2019	169	2470	5	9.9	0.08	63.12
2 multi	2	H						
97	1	05/20/2019	170	2471	5	9.6	0	47.63
2 multi	2	H						
97	1	05/21/2019	171	2472	5	9.3	0.15	44.75
2 multi	2	H						
97	1	05/22/2019	172	2473	5	9.5	0.15	46.51
2 multi	2	H						
97	1	05/23/2019	173	2474	5	9.9	0.2	47.09
2 multi	2	H						
97	1	05/24/2019	174	2475	5	9.5	0.24	45.34
2 multi	2	H						
97	1	05/25/2019	175	2476	5	9.5	0.09	46.06
2 multi	2	H						
97	1	05/26/2019	176	2477	5	9.9	0	45.02
2 multi	2	H						
97	1	05/27/2019	177	2478	5	9.9	0.25	46.01
2 multi	2	H						
97	1	05/28/2019	178	2479	5	9.8	0.23	44.07
2 multi	2	H						
97	1	05/29/2019	179	2480	5	9.6	0	44.61
2 multi	2	H						
97	1	05/30/2019	180	2481	5	9.6	0.22	42.51
2 multi	2	H						
97	1	05/31/2019	181	2482	5	9.8	0	44.69
2 multi	2	H						
97	1	06/01/2019	182	2483	5	9.6	0.1	42.29
2 multi	2	H						
97	1	06/02/2019	183	2484	5	9.8	0	42.28
2 multi	2	H						
97	1	06/03/2019	184	2485	5	9.6	0	42.04
2 multi	2	H						
97	1	06/04/2019	185	2486	5	9.9	0.12	39.17
2 multi	2	H						
97	1	06/05/2019	186	2487	5	9.6	0.31	40.89
2 multi	2	H						
97	1	06/06/2019	187	2488	5	9.9	0.26	38.12
2 multi	2	H						
97	1	06/07/2019	188	2489	5	9.8	0	41.89
2 multi	2	H						
97	1	06/08/2019	189	2490	5	9.9	0.28	40.89
2 multi	2	H						
97	1	06/09/2019	190	2491	5	9.9	0	43.6
2 multi	2	H						
97	1	06/10/2019	191	2492	5	9.9	0	42.19

2 multi	2	H						
97	1	06/11/2019	192	2493	5	9.8	0.3	42.38
2 multi	2	H						
97	1	06/12/2019	193	2494	5	9.9	0	42.64
2 multi	2	H						
97	1	06/13/2019	194	2495	5	9.9	0.27	41.55
2 multi	2	H						
97	1	06/14/2019	195	2496	5	9.8	0.07	40.33
2 multi	2	H						
97	1	06/15/2019	196	2497	5	10.2	0.24	41.04
2 multi	2	H						
97	1	06/16/2019	197	2498	5	9.9	0.17	40.38
2 multi	2	H						
97	1	06/17/2019	198	2499	5	10	0.25	40.79
2 multi	2	H						
97	1	06/18/2019	199	2500	5	10	0.13	41.82
2 multi	2	H						
97	1	06/19/2019	200	2501	5	10.2	0	41.2
2 multi	2	H						
97	1	06/20/2019	201	2502	5	10	0	43.86
2 multi	2	H						
97	1	06/21/2019	202	2503	5	9.8	0	39.15
2 multi	2	H						
97	1	06/22/2019	203	2504	5	9.5	0	40.37
2 multi	2	H						
97	1	06/23/2019	204	2505	5	9.8	0.22	40.81
2 multi	2	H						
97	1	06/24/2019	205	2506	5	9.9	0.12	42.97
2 multi	2	H						
97	1	06/25/2019	206	2507	5	9.9	0	40.68
2 multi	2	H						
97	1	06/26/2019	207	2508	5	10	0.24	43.11
2 multi	2	H						
97	1	06/27/2019	208	2509	5	9.9	0	37.22
2 multi	2	H						
97	1	06/28/2019	209	2510	5	9.8	0.16	41.3
2 multi	2	H						
97	1	06/29/2019	210	2511	5	10.2	0.08	43.41
2 multi	2	H						
97	1	06/30/2019	211	2512	5	10.2	0.19	45.44
2 multi	2	H						
97	1	07/01/2019	212	2513	5	10	0.14	39.8
2 multi	2	H						
97	1	07/02/2019	213	2514	5	9.8	0.06	40.26
2 multi	2	H						
97	1	07/03/2019	214	2515	5	10	0	40.98
2 multi	2	H						
97	1	07/04/2019	215	2516	5	9.8	0	40.19
2 multi	2	H						
97	1	07/05/2019	216	2517	5	10	0.07	38.96
2 multi	2	H						
97	1	07/06/2019	217	2518	5	10.2	0	39.48
2 multi	2	H						
97	1	07/07/2019	218	2519	5	9.3	0	37.97

2 multi 2		H						
97	1	07/08/2019	219	2520	5	9.9	0.83	39.82
2 multi 2		H						
97	1	07/09/2019	220	2521	5	9.8	0.15	39.92
2 multi 2		H						
97	1	07/10/2019	221	2522	5	10	0.09	42.45
2 multi 2		H						
97	1	07/11/2019	222	2523	5	9.9	0.08	41.13
2 multi 2		H						
97	1	07/12/2019	223	2524	5	10	0.12	41.81
2 multi 2		H						
97	1	07/13/2019	224	2525	5	10.4	0.1	45.09
2 multi 2		H						
97	1	07/14/2019	225	2526	5	10.2	0.08	44.04
2 multi 2		H						
97	1	07/15/2019	226	2527	5	10.3	0.1	37.86
2 multi 2		H						
97	1	07/16/2019	227	2528	5	10	0.09	45.06
2 multi 2		H						
97	1	07/17/2019	228	2529	5	10	0	39.18
2 multi 2		H						
97	1	07/18/2019	229	2530	5	10.3	0.1	46.04
2 multi 2		H						
97	1	07/19/2019	230	2531	5	10.2	0.08	41.11
2 multi 2		H						
97	1	07/20/2019	231	2532	5	9.8	0.07	42.88
2 multi 2		H						
97	1	07/21/2019	232	2533	5	9.8	0	40.02
2 multi 2		H						
97	1	07/22/2019	233	2534	5	10.3	0.23	43.25
2 multi 2		H						
97	1	07/23/2019	234	2535	5	10	0.26	42.16
2 multi 2		H						
97	1	07/24/2019	235	2536	5	10.3	0.16	34.18
2 multi 2		H						
97	1	07/25/2019	236	2537	5	10	0.07	37.04
2 multi 2		H						
97	1	07/26/2019	237	2538	5	9.6	0.19	33.05
2 multi 2		H						
97	1	07/27/2019	238	2539	5	10.3	0.07	34.31
2 multi 2		H						
97	1	07/28/2019	239	2540	5	10.4	0	36.41
2 multi 2		H						
97	1	07/29/2019	240	2541	5	10.4	0.04	38.34
2 multi 2		H						
97	1	07/30/2019	241	2542	5	10.3	0.24	37.85
2 multi 2		H						
97	1	07/31/2019	242	2543	5	10.3	0.12	38.41
2 multi 2		H						
97	1	08/01/2019	243	2544	5	10.2	0.08	37.42
2 multi 2		H						
97	1	08/02/2019	244	2545	5	10.3	0	39.48
2 multi 2		H						
97	1	08/03/2019	245	2546	5	10.2	0.03	38.88

2 multi 2 H
97 1 08/04/2019 246 2547 5 9.9 0.02 35.12
2 multi 2 H
97 1 08/05/2019 247 2548 5 9.9 0.19 37.13
2 multi 2 H
97 1 08/06/2019 248 2549 5 10.2 0 35.3
2 multi 2 H
97 1 08/07/2019 249 2550 5 9.9 0 39.87
2 multi 2 H
97 1 08/08/2019 250 2551 5 10 0 38.96
2 multi 2 H
97 1 08/09/2019 251 2552 5 10 0.06 38.8
2 multi 2 H
97 1 08/10/2019 252 2553 5 9.8 0 36.55
2 multi 2 H
97 1 08/11/2019 253 2554 5 10 0.2 38.66
2 multi 2 H
97 1 05/29/2020 5 2846 6 10.2 0.38 34.27
2 multi 1 H
97 1 05/30/2020 6 2847 6 10.3 0.13 40.32
2 multi 1 H
97 1 05/31/2020 7 2848 6 10.4 0 40.86
2 multi 1 H
97 1 06/01/2020 8 2849 6 10.2 0.36 43.7
2 multi 1 H
97 1 06/02/2020 9 2850 6 10.2 0 38.08
2 multi 1 H
97 1 06/03/2020 10 2851 6 10.2 0.09 41.8
2 multi 1 H
97 1 06/04/2020 11 2852 6 9.6 0.32 46.11
2 multi 1 H
97 1 06/05/2020 12 2853 6 10.4 0 48.09
2 multi 1 H
97 1 06/06/2020 13 2854 6 10.3 0.01 52.89
2 multi 1 H
97 1 06/07/2020 14 2855 6 9.9 0.09 46.81
2 multi 1 H
97 1 06/08/2020 15 2856 6 9.9 0.56 45.63
2 multi 1 H
97 1 06/09/2020 16 2857 6 10.3 0 54.34
2 multi 1 H
97 1 06/10/2020 17 2858 6 10.2 0.11 51.44
2 multi 1 H
97 1 06/11/2020 18 2859 6 10.2 0.31 48.62
2 multi 1 H
97 1 06/12/2020 19 2860 6 10.4 0.07 53.89
2 multi 1 H
97 1 06/13/2020 20 2861 6 10 0 50.41
2 multi 1 H
97 1 06/14/2020 21 2862 6 10.3 0.36 56.68
2 multi 1 H
97 1 06/15/2020 22 2863 6 10.3 0.01 54.39
2 multi 2 H
97 1 06/16/2020 23 2864 6 9.9 0.2 47.94

2 multi	2	H						
97	1	06/17/2020	24	2865	6	10.2	0.02	51.74
2 multi	2	H						
97	1	06/18/2020	25	2866	6	9.8	0.08	46.13
2 multi	2	H						
97	1	06/19/2020	26	2867	6	9.9	0	48.69
2 multi	2	H						
97	1	06/20/2020	27	2868	6	10.2	0.08	45.72
2 multi	2	H						
97	1	06/21/2020	28	2869	6	10	0.2	46.29
2 multi	2	H						
97	1	06/22/2020	29	2870	6	10.2	0.26	43.98
2 multi	2	H						
97	1	06/23/2020	30	2871	6	10.9	0.26	40.34
2 multi	2	H						
97	1	06/24/2020	31	2872	6	10.7	0.15	44.01
2 multi	2	H						
97	1	06/25/2020	32	2873	6	10.7	0	48.36
2 multi	2	H						
97	1	06/26/2020	33	2874	6	11.1	0	48.62
2 multi	2	H						
97	1	06/27/2020	34	2875	6	10.9	0	54.96
2 multi	2	H						
97	1	06/28/2020	35	2876	6	10.9	0	54.49
2 multi	2	H						
97	1	06/29/2020	36	2877	6	10.4	0	48.24
2 multi	2	H						
97	1	06/30/2020	37	2878	6	10.4	0	56.93
2 multi	2	H						
97	1	07/01/2020	38	2879	6	10.4	0	56.81
2 multi	2	H						
97	1	07/02/2020	39	2880	6	10	0	47.44
2 multi	2	H						
97	1	07/03/2020	40	2881	6	9.9	0	46.78
2 multi	2	H						
97	1	07/04/2020	41	2882	6	9.9	0.23	53.39
2 multi	2	H						
97	1	07/05/2020	42	2883	6	10	0.28	46.93
2 multi	2	H						
97	1	07/06/2020	43	2884	6	10.3	0.01	57.42
2 multi	2	H						
97	1	07/07/2020	44	2885	6	10.2	0.13	55.67
2 multi	2	H						
97	1	07/08/2020	45	2886	6	10.2	0.16	52.9
2 multi	2	H						
97	1	07/09/2020	46	2887	6	10.2	0.19	52.14
2 multi	2	H						
97	1	07/10/2020	47	2888	6	10.3	0.15	55.8
2 multi	2	H						
97	1	07/11/2020	48	2889	6	10.2	0.2	52.14
2 multi	2	H						
97	1	07/12/2020	49	2890	6	10.2	0.25	54.59
2 multi	2	H						
97	1	07/13/2020	50	2891	6	10.3	0.18	52.6

2 multi 2		H						
97	1	07/14/2020	51	2892	6	10.2	0.34	52.78
2 multi 2		H						
97	1	07/15/2020	52	2893	6	10.6	0.32	57.65
2 multi 2		H						
97	1	07/16/2020	53	2894	6	10.6	0.15	56.82
2 multi 2		H						
97	1	07/17/2020	54	2895	6	10.4	0.2	58.28
2 multi 2		H						
97	1	07/18/2020	55	2896	6	10.2	0.21	55.4
2 multi 2		H						
97	1	07/19/2020	56	2897	6	10	0	50.06
2 multi 2		H						
97	1	07/20/2020	57	2898	6	10.2	0.28	58.09
2 multi 2		H						
97	1	07/21/2020	58	2899	6	10	0.17	55.52
2 multi 2		H						
97	1	07/22/2020	59	2900	6	10.2	0.23	53.49
2 multi 2		H						
97	1	07/23/2020	60	2901	6	10	0.3	54.5
2 multi 2		H						
97	1	07/24/2020	61	2902	6	9.9	0.26	60.22
2 multi 2		H						
97	1	07/25/2020	62	2903	6	9.9	0.28	58.56
2 multi 2		H						
97	1	07/26/2020	63	2904	6	10.2	0.26	54.84
2 multi 2		H						
97	1	07/27/2020	64	2905	6	10.2	0.22	55.51
2 multi 2		H						
97	1	07/28/2020	65	2906	6	10	0.28	51.74
2 multi 2		H						
97	1	07/29/2020	66	2907	6	9.9	0.17	52.3
2 multi 2		H						
97	1	07/30/2020	67	2908	6	10	0.32	53.83
2 multi 2		H						
97	1	07/31/2020	68	2909	6	10.3	0	49.61
2 multi 2		H						
97	1	08/01/2020	69	2910	6	10.2	0.02	49.52
2 multi 2		H						
97	1	08/02/2020	70	2911	6	10	0.22	57.3
2 multi 2		H						
97	1	08/03/2020	71	2912	6	10.2	0.17	52.4
2 multi 2		H						
97	1	08/04/2020	72	2913	6	10.2	0.2	49.65
2 multi 2		H						
97	1	08/05/2020	73	2914	6	10.7	0.2	43.84
2 multi 2		H						
97	1	08/06/2020	74	2915	6	10.3	0.13	51.09
2 multi 2		H						
97	1	08/07/2020	75	2916	6	10	0.2	50.36
2 multi 2		H						
97	1	08/08/2020	76	2917	6	10	0.23	52.81
2 multi 2		H						
97	1	08/09/2020	77	2918	6	9.9	0.25	50.23

2 multi	2	H						
97	1	08/10/2020	78	2919	6	10	0.05	51.19
2 multi	2	H						
97	1	08/11/2020	79	2920	6	10	0.31	44.84
2 multi	2	H						
97	1	08/12/2020	80	2921	6	9.9	0.32	43.08
2 multi	2	H						
97	1	08/13/2020	81	2922	6	10.2	0.28	47.72
2 multi	2	H						
97	1	08/14/2020	82	2923	6	10	0.03	43.02
2 multi	2	H						
97	1	08/15/2020	83	2924	6	10.4	0.11	47.96
2 multi	2	H						
97	1	08/16/2020	84	2925	6	10.6	0.14	47.41
2 multi	2	H						
97	1	08/17/2020	85	2926	6	10.4	0.24	46.16
2 multi	2	H						
97	1	08/18/2020	86	2927	6	10.4	0.19	44.48
2 multi	2	H						
97	1	08/19/2020	87	2928	6	10.2	0.23	40.79
2 multi	2	H						
97	1	08/20/2020	88	2929	6	10.4	0.21	49.74
2 multi	2	H						
97	1	08/21/2020	89	2930	6	10.2	0	48.14
2 multi	2	H						
97	1	08/22/2020	90	2931	6	10.3	0.04	47.41
2 multi	2	H						
97	1	08/23/2020	91	2932	6	9.8	0.22	43.34
2 multi	2	H						
97	1	08/24/2020	92	2933	6	10.2	0.17	48.69
2 multi	2	H						
97	1	08/25/2020	93	2934	6	10	0.28	45.37
2 multi	2	H						
97	1	08/26/2020	94	2935	6	10.3	0.12	51.31
2 multi	2	H						
97	1	08/27/2020	95	2936	6	10.3	0.27	47.4
2 multi	2	H						
97	1	08/28/2020	96	2937	6	10.2	0.05	51.56
2 multi	2	H						
97	1	08/29/2020	97	2938	6	10.2	0	47.63
2 multi	2	H						
97	1	08/30/2020	98	2939	6	10.4	0.06	50.32
2 multi	2	H						
97	1	08/31/2020	99	2940	6	10.4	0.01	49.84
2 multi	2	H						
97	1	09/01/2020	100	2941	6	10.9	0.05	53.37
2 multi	2	H						
97	1	09/02/2020	101	2942	6	10.9	0.12	51.42
2 multi	2	H						
97	1	09/03/2020	102	2943	6	10.9	0.04	49.81
2 multi	2	H						
97	1	09/04/2020	103	2944	6	10.4	0.32	52.53
2 multi	2	H						
97	1	09/05/2020	104	2945	6	10	0	46.58

2 multi	2	H						
97	1	09/06/2020	105	2946	6	10	0.1	52.79
2 multi	2	H						
97	1	09/07/2020	106	2947	6	9.9	0.17	47.72
2 multi	2	H						
97	1	09/08/2020	107	2948	6	10	0.05	42.48
2 multi	2	H						
97	1	09/09/2020	108	2949	6	10	0.12	38.56
2 multi	2	H						
97	1	09/10/2020	109	2950	6	10	0	46.7
2 multi	2	H						
97	1	09/11/2020	110	2951	6	9.9	0	49.66
2 multi	2	H						
97	1	09/12/2020	111	2952	6	10.2	0	51.57
2 multi	2	H						
97	1	09/13/2020	112	2953	6	10.3	0.02	49.25
2 multi	2	H						
97	1	09/14/2020	113	2954	6	10.3	0.04	51.3
2 multi	2	H						
97	1	09/15/2020	114	2955	6	10.2	0.09	53.08
2 multi	2	H						
97	1	09/16/2020	115	2956	6	10.3	0	49.36
2 multi	2	H						
97	1	09/17/2020	116	2957	6	10.2	0	41.38
2 multi	2	H						
97	1	09/18/2020	117	2958	6	10.6	0	47.46
2 multi	2	H						
97	1	09/19/2020	118	2959	6	10.6	0	47.71
2 multi	2	H						
97	1	09/20/2020	119	2960	6	10.3	0.11	50.98
2 multi	2	H						
97	1	09/21/2020	120	2961	6	10.4	0.09	50.23
2 multi	2	H						
97	1	09/22/2020	121	2962	6	10.4	0.03	52.22
2 multi	2	H						
97	1	09/23/2020	122	2963	6	10.2	0.13	48.5
2 multi	2	H						
97	1	09/24/2020	123	2964	6	10	0.21	55.18
2 multi	2	H						
97	1	09/25/2020	124	2965	6	10	0.22	46.4
2 multi	2	H						
97	1	09/26/2020	125	2966	6	10	0.26	53.63
2 multi	2	H						
97	1	09/27/2020	126	2967	6	10.2	0.12	50.11
2 multi	2	H						
97	1	09/28/2020	127	2968	6	10	0.37	52.37
2 multi	2	H						
97	1	09/29/2020	128	2969	6	10.2	0.45	50.17
2 multi	2	H						
97	1	09/30/2020	129	2970	6	10.2	0	51.85
2 multi	2	H						
97	1	10/01/2020	130	2971	6	10.2	0.12	51.57
2 multi	2	H						
97	1	10/02/2020	131	2972	6	10	0.12	53.39

2 multi	2	H						
97	1	10/03/2020	132	2973	6	9.8	0.02	46.23
2 multi	2	H						
97	1	10/04/2020	133	2974	6	9.8	0.03	49.93
2 multi	2	H						
97	1	10/05/2020	134	2975	6	9.9	0.12	51.02
2 multi	2	H						
97	1	10/06/2020	135	2976	6	10.2	0.2	50.16
2 multi	2	H						
97	1	10/07/2020	136	2977	6	10.3	0.06	51.7
2 multi	2	H						
97	1	10/08/2020	137	2978	6	10.3	0.14	52.78
2 multi	2	H						
97	1	10/09/2020	138	2979	6	10.3	0.25	51.86
2 multi	2	H						
97	1	10/10/2020	139	2980	6	10.3	0	49.3
2 multi	2	H						
97	1	10/11/2020	140	2981	6	10	0.01	52.28
2 multi	2	H						
97	1	10/12/2020	141	2982	6	9.8	0	51.33
2 multi	2	H						
97	1	10/13/2020	142	2983	6	10	0.04	47.62
2 multi	2	H						
97	1	10/14/2020	143	2984	6	9.9	0	52.59
2 multi	2	H						
97	1	10/15/2020	144	2985	6	10.2	0.17	49.71
2 multi	2	H						
97	1	10/16/2020	145	2986	6	9.9	0	52.01
2 multi	2	H						
97	1	10/17/2020	146	2987	6	10.2	0.03	50.48
2 multi	2	H						
97	1	10/18/2020	147	2988	6	10	0.05	44.56
2 multi	2	H						
97	1	10/19/2020	148	2989	6	0	0	53.17
2 multi	2	H						
97	1	10/20/2020	149	2990	6	10.3	0.14	55.57
2 multi	2	H						
97	1	10/21/2020	150	2991	6	10.3	0.24	53.68
2 multi	2	H						
97	1	10/22/2020	151	2992	6	10.3	0	53.52
2 multi	2	H						
97	1	10/23/2020	152	2993	6	10.2	0.05	52.91
2 multi	2	H						
97	1	10/24/2020	153	2994	6	10.2	0.05	50.22
2 multi	2	H						
97	1	10/25/2020	154	2995	6	10.2	0.01	49.66
2 multi	2	H						
97	1	10/26/2020	155	2996	6	9.9	0.22	56.36
2 multi	2	H						
97	1	10/27/2020	156	2997	6	10	0.14	49.48
2 multi	2	H						
97	1	10/28/2020	157	2998	6	10.2	0.07	50.83
2 multi	2	H						
97	1	10/29/2020	158	2999	6	10.2	0.13	49.87

2 multi	2	H						
97	1	10/30/2020	159	3000	6	10	0.06	52.45
2 multi	2	H						
97	1	10/31/2020	160	3001	6	10.2	0.01	51.42
2 multi	2	H						
97	1	11/01/2020	161	3002	6	10.2	0.06	49.16
2 multi	2	H						
97	1	11/02/2020	162	3003	6	10.2	0.07	50.16
2 multi	2	H						
97	1	11/03/2020	163	3004	6	10	0	50.12
2 multi	2	H						
97	1	11/04/2020	164	3005	6	9.9	0.15	53.57
2 multi	2	H						
97	1	11/05/2020	165	3006	6	10	0	50.32
2 multi	2	H						
97	1	11/06/2020	166	3007	6	10	0.31	48.19
2 multi	2	H						
97	1	11/07/2020	167	3008	6	9.9	0.1	51.91
2 multi	2	H						
97	1	11/08/2020	168	3009	6	10.2	0.16	50.94
2 multi	2	H						
97	1	11/09/2020	169	3010	6	9.8	0.15	49.49
2 multi	2	H						
97	1	11/10/2020	170	3011	6	9.9	0.01	50.77
2 multi	2	H						
97	1	11/11/2020	171	3012	6	9.9	0.07	50.45
2 multi	2	H						
97	1	11/12/2020	172	3013	6	9.9	0.21	48.07
2 multi	2	H						
97	1	11/13/2020	173	3014	6	9.8	0.12	48.62
2 multi	2	H						
97	1	11/14/2020	174	3015	6	10	0.05	50.28
2 multi	2	H						
97	1	11/15/2020	175	3016	6	9.9	0.09	46.36
2 multi	2	H						
97	1	11/16/2020	176	3017	6	9.9	0.1	50.88
2 multi	2	H						
97	1	11/17/2020	177	3018	6	9.8	0.08	50.87
2 multi	2	H						
97	1	11/18/2020	178	3019	6	9.9	0.09	47.76
2 multi	2	H						
97	1	11/19/2020	179	3020	6	10	0.12	51.98
2 multi	2	H						
97	1	11/20/2020	180	3021	6	9.9	0.11	50.74
2 multi	2	H						
97	1	11/21/2020	181	3022	6	10	0.04	49.1
2 multi	2	H						
97	1	11/22/2020	182	3023	6	10	0.04	48.58
2 multi	2	H						
97	1	11/23/2020	183	3024	6	9.9	0.1	45.05
2 multi	2	H						
97	1	11/24/2020	184	3025	6	9.9	0.05	51.2
2 multi	2	H						
97	1	11/25/2020	185	3026	6	10	0.1	44.11

2 multi 2		H						
97	1	11/26/2020	186	3027	6	9.9	0.14	49.12
2 multi 2		H						
97	1	11/27/2020	187	3028	6	9.8	0.04	46.3
2 multi 2		H						
97	1	11/28/2020	188	3029	6	9.8	0.06	47.54
2 multi 2		H						
97	1	11/29/2020	189	3030	6	10	0.04	48.5
2 multi 2		H						
97	1	11/30/2020	190	3031	6	9.8	0.05	46.69
2 multi 2		H						
97	1	12/01/2020	191	3032	6	9.9	0.01	51.07
2 multi 2		H						
97	1	12/02/2020	192	3033	6	10	0.05	51.9
2 multi 2		H						
97	1	12/03/2020	193	3034	6	9.9	0.13	49.52
2 multi 2		H						
97	1	12/04/2020	194	3035	6	9.8	0.17	49.67
2 multi 2		H						
97	1	12/05/2020	195	3036	6	9.9	0.1	48.76
2 multi 2		H						
97	1	12/06/2020	196	3037	6	10	0.04	47.54
2 multi 2		H						
97	1	12/07/2020	197	3038	6	10	0.12	46.07
2 multi 2		H						
97	1	12/08/2020	198	3039	6	9.9	0.3	51.88
2 multi 2		H						
97	1	12/09/2020	199	3040	6	9.8	0.36	45.05
2 multi 2		H						
97	1	12/10/2020	200	3041	6	9.9	0.12	49.59
2 multi 2		H						
97	1	12/11/2020	201	3042	6	10	0.13	50.39
2 multi 2		H						
97	1	12/12/2020	202	3043	6	10.2	0.13	45.42
2 multi 2		H						
97	1	12/13/2020	203	3044	6	10.2	0.12	50.6
2 multi 2		H						
97	1	12/14/2020	204	3045	6	10.2	0.13	49.55
2 multi 2		H						
97	1	12/15/2020	205	3046	6	10.2	0	49.38
2 multi 2		H						
97	1	12/16/2020	206	3047	6	10	0	45.67
2 multi 2		H						
97	1	12/17/2020	207	3048	6	10	0.11	51.8
2 multi 2		H						
97	1	12/18/2020	208	3049	6	10.2	0.07	48.04
2 multi 2		H						
97	1	12/19/2020	209	3050	6	10.2	0.03	50.21
2 multi 2		H						
97	1	12/20/2020	210	3051	6	9.8	0.13	47.14
2 multi 2		H						
97	1	12/21/2020	211	3052	6	10	0.37	45.12
2 multi 2		H						
97	1	12/22/2020	212	3053	6	10	0.24	51.77

2 multi	2	H						
97	1	12/23/2020	213	3054	6	10	0.07	44.74
2 multi	2	H						
97	1	12/24/2020	214	3055	6	9.9	0.07	46.09
2 multi	2	H						
97	1	12/25/2020	215	3056	6	10	0	50.28
2 multi	2	H						
97	1	12/26/2020	216	3057	6	10.2	0.11	47.69
2 multi	2	H						
97	1	12/27/2020	217	3058	6	10	0.11	48
2 multi	2	H						
97	1	12/28/2020	218	3059	6	10	0.12	47.13
2 multi	2	H						
97	1	12/29/2020	219	3060	6	10.2	0.17	50.3
2 multi	2	H						
97	1	12/30/2020	220	3061	6	10	0.01	48.34
2 multi	2	H						
97	1	12/31/2020	221	3062	6	10	0.03	47.09
2 multi	2	H						
97	1	01/01/2021	222	3063	6	10	0.04	45.99
2 multi	2	H						
97	1	01/02/2021	223	3064	6	10	0.13	49.24
2 multi	2	H						
97	1	01/03/2021	224	3065	6	10	0.05	47.72
2 multi	2	H						
97	1	01/04/2021	225	3066	6	10.2	0.13	47.75
2 multi	2	H						
97	1	01/05/2021	226	3067	6	10.2	0.13	47.39
2 multi	2	H						
97	1	01/06/2021	227	3068	6	10.2	0.06	47.59
2 multi	2	H						
97	1	01/07/2021	228	3069	6	10	0.12	47.08
2 multi	2	H						
97	1	01/08/2021	229	3070	6	10.2	0.04	47.26
2 multi	2	H						
97	1	01/09/2021	230	3071	6	10.2	0.13	48.41
2 multi	2	H						
97	1	01/10/2021	231	3072	6	10	0.06	47.46
2 multi	2	H						
97	1	01/11/2021	232	3073	6	10	0.04	45.33
2 multi	2	H						
97	1	01/12/2021	233	3074	6	10	0.1	46.5
2 multi	2	H						
97	1	01/13/2021	234	3075	6	9.9	0.04	45.47
2 multi	2	H						
97	1	01/14/2021	235	3076	6	10	0.1	47.19
2 multi	2	H						
97	1	01/15/2021	236	3077	6	10.2	0.05	44.28
2 multi	2	H						
97	1	01/16/2021	237	3078	6	9.9	0.04	47.79
2 multi	2	H						
97	1	01/17/2021	238	3079	6	10.2	0.02	46.22
2 multi	2	H						
97	1	01/18/2021	239	3080	6	9.9	0.14	47.54

2 multi	2	H						
97	1	01/19/2021	240	3081	6	9.9	0.1	43.23
2 multi	2	H						
97	1	01/20/2021	241	3082	6	9.8	0.11	38.37
2 multi	2	H						
97	1	01/21/2021	242	3083	6	9.6	0.02	37.25
2 multi	2	H						
97	1	01/22/2021	243	3084	6	9.9	0	39.53
2 multi	2	H						
97	1	01/23/2021	244	3085	6	9.8	0.04	41.46
2 multi	2	H						
97	1	01/24/2021	245	3086	6	9.9	0.01	38.09
2 multi	2	H						
97	1	01/25/2021	246	3087	6	10.2	0.03	43.89
2 multi	2	H						
97	1	01/26/2021	247	3088	6	10.2	0.11	43.96
2 multi	2	H						
97	1	01/27/2021	248	3089	6	10.4	0.03	44.84
2 multi	2	H						
97	1	01/28/2021	249	3090	6	10.3	0.1	46.14
2 multi	2	H						
97	1	01/29/2021	250	3091	6	10.2	0.03	44.52
2 multi	2	H						
97	1	01/30/2021	251	3092	6	10	0.11	43.21
2 multi	2	H						
97	1	01/31/2021	252	3093	6	9.9	0.11	45.82
2 multi	2	H						
97	1	02/01/2021	253	3094	6	9.9	0.01	41.5
2 multi	2	H						
97	1	02/02/2021	254	3095	6	10	0.16	44.49
2 multi	2	H						
97	1	02/03/2021	255	3096	6	9.9	0.04	42.49
2 multi	2	H						
97	1	02/04/2021	256	3097	6	10	0.03	42.34
2 multi	2	H						
97	1	02/05/2021	257	3098	6	9.8	0.05	40.63
2 multi	2	H						
97	1	02/06/2021	258	3099	6	9.9	0.11	39.98
2 multi	2	H						
97	1	02/07/2021	259	3100	6	10.3	0.13	45.07
2 multi	2	H						
97	1	02/08/2021	260	3101	6	9.8	0.03	39.37
2 multi	2	H						
97	1	02/09/2021	261	3102	6	9.9	0.01	40.46
2 multi	2	H						
97	1	02/10/2021	262	3103	6	10	0.13	43.35
2 multi	2	H						
97	1	02/11/2021	263	3104	6	10.2	0.02	42.13
2 multi	2	H						
97	1	02/12/2021	264	3105	6	9.9	0.04	40.28
2 multi	2	H						
97	1	02/13/2021	265	3106	6	9.9	0.13	46.21
2 multi	2	H						
97	1	02/14/2021	266	3107	6	9.8	0.26	39.61

2 multi 2		H						
97	1	05/04/2021	4	3186	7	10.2	0.36	39.25
2 multi 1		H						
97	1	05/05/2021	5	3187	7	10.2	0.39	40.94
2 multi 1		H						
97	1	05/06/2021	6	3188	7	10.9	0.15	45.68
2 multi 1		H						
97	1	05/07/2021	7	3189	7	10.3	0.08	45.15
2 multi 1		H						
97	1	05/08/2021	8	3190	7	9.9	0.11	45.33
2 multi 1		H						
97	1	05/09/2021	9	3191	7	10.2	0.11	49.75
2 multi 1		H						
97	1	05/10/2021	10	3192	7	10.3	0.14	51.1
2 multi 1		H						
97	1	05/11/2021	11	3193	7	10	0.02	50.93
2 multi 1		H						
97	1	05/12/2021	12	3194	7	10	0.23	50.52
2 multi 1		H						
97	1	05/13/2021	13	3195	7	10	0.02	51.33
2 multi 1		H						
97	1	05/14/2021	14	3196	7	9.9	0.24	51.97
2 multi 1		H						
97	1	05/15/2021	15	3197	7	9.9	0.16	51.26
2 multi 1		H						
97	1	05/16/2021	16	3198	7	9.9	0.32	53.62
2 multi 1		H						
97	1	05/17/2021	17	3199	7	10	0	57.7
2 multi 1		H						
97	1	05/18/2021	18	3200	7	10	0	56.35
2 multi 1		H						
97	1	05/19/2021	19	3201	7	10	0.14	56.66
2 multi 1		H						
97	1	05/20/2021	20	3202	7	9.6	0.18	56.63
2 multi 1		H						
97	1	05/21/2021	21	3203	7	9.9	0.25	58.68
2 multi 1		H						
97	1	05/22/2021	22	3204	7	9.9	0	58.38
2 multi 2		H						
97	1	05/23/2021	23	3205	7	9.8	0.41	61.09
2 multi 2		H						
97	1	05/24/2021	24	3206	7	9.9	0.28	62.68
2 multi 2		H						
97	1	05/25/2021	25	3207	7	10	0.13	58.6
2 multi 2		H						
97	1	05/26/2021	26	3208	7	10	0	29.95
2 multi 2		H						
99	1	05/14/2020	4	736	1	9.2	0.48	19.24
1 primi 1		H						
99	1	05/15/2020	5	737	1	9.8	0.07	18.98
1 primi 1		H						
99	1	05/16/2020	6	738	1	10.2	0.05	19.68
1 primi 1		H						
99	1	05/17/2020	7	739	1	9.2	0.01	20.88

1 primi	1	H						
99	1	05/18/2020	8	740	1	9.5	0.05	22.61
1 primi	1	H						
99	1	05/19/2020	9	741	1	9.5	0.08	23.92
1 primi	1	H						
99	1	05/20/2020	10	742	1	9.1	0	24.32
1 primi	1	H						
99	1	05/21/2020	11	743	1	9.2	0.14	24.77
1 primi	1	H						
99	1	05/22/2020	12	744	1	9.1	0.62	24.94
1 primi	1	H						
99	1	05/23/2020	13	745	1	9.6	0.42	24.59
1 primi	1	H						
99	1	05/24/2020	14	746	1	9.2	0.35	27.35
1 primi	1	H						
99	1	05/25/2020	15	747	1	9.1	0.32	27.77
1 primi	1	H						
99	1	05/26/2020	16	748	1	9.1	0.16	26.87
1 primi	1	H						
99	1	05/27/2020	17	749	1	9.2	0.11	28.77
1 primi	1	H						
99	1	05/28/2020	18	750	1	9.2	0.19	29.33
1 primi	1	H						
99	1	05/29/2020	19	751	1	9.1	0.21	28.18
1 primi	1	H						
99	1	05/30/2020	20	752	1	8.9	0	30.78
1 primi	1	H						
99	1	05/31/2020	21	753	1	8.9	0	31.63
1 primi	1	H						
99	1	06/01/2020	22	754	1	9.2	0.02	29.72
1 primi	2	H						
99	1	06/02/2020	23	755	1	8.7	0	31.18
1 primi	2	H						
99	1	06/03/2020	24	756	1	8.8	0.24	31.84
1 primi	2	H						
99	1	06/04/2020	25	757	1	9.1	0.02	31.72
1 primi	2	H						
99	1	06/05/2020	26	758	1	8.8	0.17	31.78
1 primi	2	H						
99	1	06/06/2020	27	759	1	8.6	0	33.43
1 primi	2	H						
99	1	06/07/2020	28	760	1	8.6	0.47	31.81
1 primi	2	H						
99	1	06/08/2020	29	761	1	8.6	0.08	30.46
1 primi	2	H						
99	1	06/09/2020	30	762	1	8.6	0	32.35
1 primi	2	H						
99	1	06/10/2020	31	763	1	8.7	0.09	30.82
1 primi	2	H						
99	1	06/11/2020	32	764	1	8.8	0	33.02
1 primi	2	H						
99	1	06/12/2020	33	765	1	8.9	0.09	30.8
1 primi	2	H						
99	1	06/13/2020	34	766	1	8.8	0	34.02

1 primi 2		H						
99	1	06/14/2020	35	767	1	8.9	0.12	31.42
1 primi 2		H						
99	1	06/15/2020	36	768	1	8.6	0	32.83
1 primi 2		H						
99	1	06/16/2020	37	769	1	8.7	0	32.2
1 primi 2		H						
99	1	06/17/2020	38	770	1	8.7	0.14	33.77
1 primi 2		H						
99	1	06/18/2020	39	771	1	8.9	0.09	30.1
1 primi 2		H						
99	1	06/19/2020	40	772	1	8.8	0	33.95
1 primi 2		H						
99	1	06/20/2020	41	773	1	9.1	0.13	35.74
1 primi 2		H						
99	1	06/21/2020	42	774	1	8.7	0.02	34.82
1 primi 2		H						
99	1	06/22/2020	43	775	1	9.1	0	33.68
1 primi 2		H						
99	1	06/23/2020	44	776	1	9.1	0	35.47
1 primi 2		H						
99	1	06/24/2020	45	777	1	8.7	0.25	33.04
1 primi 2		H						
99	1	06/25/2020	46	778	1	8.8	0	31.61
1 primi 2		H						
99	1	06/26/2020	47	779	1	8.9	0	32.31
1 primi 2		H						
99	1	06/27/2020	48	780	1	8.8	0	34.27
1 primi 2		H						
99	1	06/28/2020	49	781	1	8.8	0	33.37
1 primi 2		H						
99	1	06/29/2020	50	782	1	9.2	0	34.32
1 primi 2		H						
100	1	02/25/2020	4	825	1	9.9	0.21	23.82
1 primi 1		H						
100	1	02/26/2020	5	826	1	10	0.01	18.43
1 primi 1		H						
100	1	02/27/2020	6	827	1	9.9	0.16	23.81
1 primi 1		H						
100	1	02/28/2020	7	828	1	9.3	0.13	19.11
1 primi 1		H						
100	1	02/29/2020	8	829	1	9.3	0	23.55
1 primi 1		H						
100	1	03/01/2020	9	830	1	9.6	0.01	25
1 primi 1		H						
100	1	03/02/2020	10	831	1	9.3	0	24.49
1 primi 1		H						
100	1	03/03/2020	11	832	1	9.9	0	24.1
1 primi 1		H						
100	1	03/04/2020	12	833	1	10	0.12	30.02
1 primi 1		H						
100	1	03/05/2020	13	834	1	9.8	0.25	26.35
1 primi 1		H						
100	1	03/06/2020	14	835	1	9.9	0	27.9

1 primi	1	H						
100	1	03/07/2020	15	836	1	9.8	0.03	22.54
1 primi	1	H						
100	1	03/08/2020	16	837	1	9.6	0	31.05
1 primi	1	H						
100	1	03/09/2020	17	838	1	9.8	0.02	30.23
1 primi	1	H						
100	1	03/10/2020	18	839	1	9.8	0	28.76
1 primi	1	H						
100	1	03/11/2020	19	840	1	9.8	0	31.1
1 primi	1	H						
100	1	03/12/2020	20	841	1	9.6	0	31.37
1 primi	1	H						
100	1	03/13/2020	21	842	1	9.3	0	31.58
1 primi	1	H						
100	1	03/14/2020	22	843	1	9.2	0	31.08
1 primi	2	H						
100	1	03/15/2020	23	844	1	9.1	0	31.71
1 primi	2	H						
100	1	03/16/2020	24	845	1	9.1	0	31.67
1 primi	2	H						
100	1	03/17/2020	25	846	1	9.3	0	29.25
1 primi	2	H						
100	1	03/18/2020	26	847	1	8.9	0	34.77
1 primi	2	H						
100	1	03/19/2020	27	848	1	9.1	0.26	31.67
1 primi	2	H						
100	1	03/20/2020	28	849	1	9.1	0	32.68
1 primi	2	H						
100	1	03/21/2020	29	850	1	9.1	0.05	32.94
1 primi	2	H						
100	1	03/22/2020	30	851	1	9.1	0	30.2
1 primi	2	H						
100	1	03/23/2020	31	852	1	8.9	0	31.39
1 primi	2	H						
100	1	03/24/2020	32	853	1	8.9	0	33.85
1 primi	2	H						
100	1	03/25/2020	33	854	1	9.3	0	31.71
1 primi	2	H						
100	1	03/26/2020	34	855	1	8.7	0.01	32.33
1 primi	2	H						
100	1	03/27/2020	35	856	1	8.7	0.09	33.38
1 primi	2	H						
100	1	03/28/2020	36	857	1	8.9	0	34.01
1 primi	2	H						
100	1	03/29/2020	37	858	1	8.6	0	33.1
1 primi	2	H						
100	1	03/30/2020	38	859	1	8.6	0.02	33.9
1 primi	2	H						
100	1	03/31/2020	39	860	1	8.9	0	32.18
1 primi	2	H						
100	1	04/01/2020	40	861	1	8.7	0.12	35.18
1 primi	2	H						
100	1	04/02/2020	41	862	1	8.7	0.1	33.14

1 primi 2		H						
100	1	04/03/2020	42	863	1	9.1	0	34.64
1 primi 2		H						
100	1	04/04/2020	43	864	1	9.1	0	31.35
1 primi 2		H						
100	1	04/05/2020	44	865	1	8.9	0.04	34.33
1 primi 2		H						
100	1	04/06/2020	45	866	1	9.1	0.2	31.69
1 primi 2		H						
100	1	04/07/2020	46	867	1	8.8	0.24	31.21
1 primi 2		H						
100	1	04/08/2020	47	868	1	8.9	0.48	32.77
1 primi 2		H						
100	1	04/09/2020	48	869	1	9.1	0.39	32.71
1 primi 2		H						
100	1	04/10/2020	49	870	1	9.3	0.16	36.64
1 primi 2		H						
100	1	04/11/2020	50	871	1	9.6	0	33.77
1 primi 2		H						
100	1	04/12/2020	51	872	1	9.2	0	33.65
1 primi 2		H						
100	1	04/13/2020	52	873	1	8.9	0	36.41
1 primi 2		H						
100	1	04/14/2020	53	874	1	8.9	0	33.97
1 primi 2		H						
100	1	04/15/2020	54	875	1	8.8	0	31.94
1 primi 2		H						
100	1	04/16/2020	55	876	1	8.9	0	32.34
1 primi 2		H						
100	1	04/17/2020	56	877	1	8.9	0	32.75
1 primi 2		H						
100	1	04/18/2020	57	878	1	8.9	0	33.44
1 primi 2		H						
100	1	04/19/2020	58	879	1	9.2	0	34.41
1 primi 2		H						
100	1	04/20/2020	59	880	1	9.1	0.01	34.78
1 primi 2		H						
100	1	04/21/2020	60	881	1	9.3	0	33.94
1 primi 2		H						
100	1	04/22/2020	61	882	1	8.9	0.27	33.27
1 primi 2		H						
100	1	04/23/2020	62	883	1	9.3	0	32.65
1 primi 2		H						
100	1	04/24/2020	63	884	1	9.2	0	34.54
1 primi 2		H						
100	1	04/25/2020	64	885	1	8.9	0.13	34.16
1 primi 2		H						
100	1	04/26/2020	65	886	1	8.9	0	34.34
1 primi 2		H						
100	1	04/27/2020	66	887	1	9.1	0	35.16
1 primi 2		H						
100	1	04/28/2020	67	888	1	9.1	0.17	34.08
1 primi 2		H						
100	1	04/29/2020	68	889	1	9.1	0.25	32.86

1 primi	2	H						
100	1	04/30/2020	69	890	1	9.2	0	33.43
1 primi	2	H						
100	1	05/01/2020	70	891	1	9.3	0	33.58
1 primi	2	H						
100	1	05/02/2020	71	892	1	9.1	0	33.48
1 primi	2	H						
100	1	05/03/2020	72	893	1	9.3	0.14	34.85
1 primi	2	H						
100	1	05/04/2020	73	894	1	8.9	0	32.88
1 primi	2	H						
100	1	05/05/2020	74	895	1	9.2	0	35.05
1 primi	2	H						
100	1	05/06/2020	75	896	1	9.3	0	31.53
1 primi	2	H						
100	1	05/07/2020	76	897	1	9.3	0	35.22
1 primi	2	H						
100	1	05/08/2020	77	898	1	9.3	0.65	32.34
1 primi	2	H						
100	1	05/09/2020	78	899	1	9.2	0	33.38
1 primi	2	H						
100	1	05/10/2020	79	900	1	9.1	0.33	34.25
1 primi	2	H						
100	1	05/11/2020	80	901	1	9.2	0.07	33.4
1 primi	2	H						
100	1	05/12/2020	81	902	1	9.1	0	32.1
1 primi	2	H						
100	1	05/13/2020	82	903	1	9.3	0	31.57
1 primi	2	H						
100	1	03/12/2021	7	1206	2	9.2	0.2	29.72
2 multi	1	H						
100	1	03/13/2021	8	1207	2	9.3	0.1	30.93
2 multi	1	H						
100	1	03/14/2021	9	1208	2	10.4	0.04	30.33
2 multi	1	H						
100	1	03/15/2021	10	1209	2	10.7	0.04	34.99
2 multi	1	H						
100	1	03/16/2021	11	1210	2	10.3	0	34.27
2 multi	1	H						
100	1	03/17/2021	12	1211	2	9.8	0.03	37.83
2 multi	1	H						
100	1	03/18/2021	13	1212	2	9.8	0.03	35.92
2 multi	1	H						
100	1	03/19/2021	14	1213	2	10.3	0.04	38.44
2 multi	1	H						
100	1	03/20/2021	15	1214	2	9.9	0.03	39.27
2 multi	1	H						
100	1	03/21/2021	16	1215	2	9.9	0.04	39.12
2 multi	1	H						
100	1	03/22/2021	17	1216	2	9.9	0	39.58
2 multi	1	H						
100	1	03/23/2021	18	1217	2	10.3	0	39.65
2 multi	1	H						
100	1	03/24/2021	19	1218	2	10.7	0.06	41.16

2 multi	1	H						
100	1	03/25/2021	20	1219	2	9.9	0	43.04
2 multi	1	H						
100	1	03/26/2021	21	1220	2	9.8	0	42.47
2 multi	1	H						
100	1	03/27/2021	22	1221	2	10.7	0.15	39.48
2 multi	2	H						
100	1	03/28/2021	23	1222	2	9.8	0.02	41.68
2 multi	2	H						
100	1	03/29/2021	24	1223	2	10	0	45.45
2 multi	2	H						
100	1	03/30/2021	25	1224	2	10.6	0.03	41
2 multi	2	H						
100	1	03/31/2021	26	1225	2	10	0.02	42.04
2 multi	2	H						
100	1	04/01/2021	27	1226	2	10	0	40.83
2 multi	2	H						
100	1	04/02/2021	28	1227	2	9.8	0	40.56
2 multi	2	H						
100	1	04/03/2021	29	1228	2	9.9	0.01	44.72
2 multi	2	H						
100	1	04/04/2021	30	1229	2	10.2	0.01	41.36
2 multi	2	H						
100	1	04/05/2021	31	1230	2	10.2	0.17	44.4
2 multi	2	H						
100	1	04/06/2021	32	1231	2	10.4	0.02	44.01
2 multi	2	H						
100	1	04/07/2021	33	1232	2	10.2	0.02	43.41
2 multi	2	H						
100	1	04/08/2021	34	1233	2	10	0.01	42.56
2 multi	2	H						
100	1	04/09/2021	35	1234	2	10.7	0.02	42.78
2 multi	2	H						
100	1	04/10/2021	36	1235	2	10.2	0	46.51
2 multi	2	H						
100	1	04/11/2021	37	1236	2	10.3	0.01	42.05
2 multi	2	H						
100	1	04/12/2021	38	1237	2	10.6	0.03	44.23
2 multi	2	H						
100	1	04/13/2021	39	1238	2	10.9	0.02	48.29
2 multi	2	H						
100	1	04/14/2021	40	1239	2	11.1	0	45.1
2 multi	2	H						
100	1	04/15/2021	41	1240	2	10.7	0	45.54
2 multi	2	H						
100	1	04/16/2021	42	1241	2	10.4	0.05	44.89
2 multi	2	H						
100	1	04/17/2021	43	1242	2	10.4	0.02	43.8
2 multi	2	H						
100	1	04/18/2021	44	1243	2	10.6	0	45.4
2 multi	2	H						
100	1	04/19/2021	45	1244	2	10.7	0	43.57
2 multi	2	H						
100	1	04/20/2021	46	1245	2	10.2	0	42.53

2 multi 2		H						
100	1	04/21/2021	47	1246	2	10.4	0	43.43
2 multi 2		H						
100	1	04/22/2021	48	1247	2	10.7	0	46.7
2 multi 2		H						
100	1	04/23/2021	49	1248	2	10.7	0	46.61
2 multi 2		H						
100	1	04/24/2021	50	1249	2	10.4	0	46.86
2 multi 2		H						
100	1	04/25/2021	51	1250	2	10.9	0.04	47.85
2 multi 2		H						
100	1	04/26/2021	52	1251	2	10.4	0.03	45.3
2 multi 2		H						
100	1	04/27/2021	53	1252	2	11.3	0	44.64
2 multi 2		H						
100	1	04/28/2021	54	1253	2	10.6	0	44.71
2 multi 2		H						
100	1	04/29/2021	55	1254	2	11.1	0.02	47.37
2 multi 2		H						
100	1	04/30/2021	56	1255	2	10.7	0.11	46.07
2 multi 2		H						
100	1	05/01/2021	57	1256	2	10.3	0	43.17
2 multi 2		H						
100	1	05/02/2021	58	1257	2	12.6	0.16	47.98
2 multi 2		H						
100	1	05/03/2021	59	1258	2	11.7	0.06	43.31
2 multi 2		H						
100	1	05/04/2021	60	1259	2	10.6	0.03	40.58
2 multi 2		H						
100	1	05/05/2021	61	1260	2	10.6	0	44.69
2 multi 2		H						
100	1	05/06/2021	62	1261	2	10.7	0	40.83
2 multi 2		H						
100	1	05/07/2021	63	1262	2	11.3	0	45.29
2 multi 2		H						
100	1	05/08/2021	64	1263	2	10.6	0.05	44.37
2 multi 2		H						
100	1	05/09/2021	65	1264	2	10.9	0.04	46.43
2 multi 2		H						
100	1	05/10/2021	66	1265	2	11.9	0.19	43.37
2 multi 2		H						
100	1	05/11/2021	67	1266	2	10.9	0	44.81
2 multi 2		H						
100	1	05/12/2021	68	1267	2	10.4	0.03	43.06
2 multi 2		H						
100	1	05/13/2021	69	1268	2	11.1	0.07	44.15
2 multi 2		H						
100	1	05/14/2021	70	1269	2	11.4	0.05	43.81
2 multi 2		H						
100	1	05/15/2021	71	1270	2	10.6	0	45.39
2 multi 2		H						
100	1	05/16/2021	72	1271	2	10.4	0.02	43.87
2 multi 2		H						
100	1	05/17/2021	73	1272	2	11.1	0	44.5

2 multi	2	H						
100	1	05/18/2021	74	1273	2	11.3	0.01	42.88
2 multi	2	H						
100	1	05/19/2021	75	1274	2	10.4	0.08	43.87
2 multi	2	H						
100	1	05/20/2021	76	1275	2	10.4	0.1	41.15
2 multi	2	H						
100	1	05/21/2021	77	1276	2	11.3	0.04	48.23
2 multi	2	H						
100	1	05/22/2021	78	1277	2	11.3	0.04	45.33
2 multi	2	H						
100	1	05/23/2021	79	1278	2	10.6	0.01	42.8
2 multi	2	H						
100	1	05/24/2021	80	1279	2	10.7	0.04	43.89
2 multi	2	H						
100	1	05/25/2021	81	1280	2	11.1	0.04	44.02
2 multi	2	H						
100	1	05/26/2021	82	1281	2	10.4	0.04	21.96
2 multi	2	H						
101	1	05/15/2019	4	668	1	9.8	0.25	20.75
1 primi	1	H						
101	1	05/16/2019	5	669	1	10.2	0	20.08
1 primi	1	H						
101	1	05/17/2019	6	670	1	9.9	0.11	22.38
1 primi	1	H						
101	1	05/18/2019	7	671	1	9.5	0.1	24.09
1 primi	1	H						
101	1	05/19/2019	8	672	1	10	0.08	22.81
1 primi	1	H						
101	1	05/20/2019	9	673	1	9.9	0.12	23.26
1 primi	1	H						
101	1	05/21/2019	10	674	1	9.9	0	24.06
1 primi	1	H						
101	1	05/22/2019	11	675	1	9.8	0.05	22.33
1 primi	1	H						
101	1	05/23/2019	12	676	1	9.8	0.09	23.57
1 primi	1	H						
101	1	05/24/2019	13	677	1	9.8	0	22.13
1 primi	1	H						
101	1	05/25/2019	14	678	1	10.7	0.09	23.37
1 primi	1	H						
101	1	05/26/2019	15	679	1	10.4	0	23.07
1 primi	1	H						
101	1	05/27/2019	16	680	1	10.7	0.08	25.5
1 primi	1	H						
101	1	05/28/2019	17	681	1	10.6	0	23.85
1 primi	1	H						
101	1	05/29/2019	18	682	1	10.2	0	22.87
1 primi	1	H						
101	1	05/30/2019	19	683	1	9.9	0.09	24.02
1 primi	1	H						
101	1	05/31/2019	20	684	1	9.9	0	23.48
1 primi	1	H						
101	1	06/01/2019	21	685	1	10	0.09	21.76

1 primi 1		H						
101	1	06/02/2019	22	686	1	9.9	0	21.84
1 primi 2		H						
101	1	06/03/2019	23	687	1	10	0.09	24.41
1 primi 2		H						
101	1	06/04/2019	24	688	1	10.7	0	24.86
1 primi 2		H						
101	1	06/05/2019	25	689	1	9.9	0	26.03
1 primi 2		H						
101	1	06/06/2019	26	690	1	10.2	0.08	24.97
1 primi 2		H						
101	1	06/07/2019	27	691	1	10	0	22.38
1 primi 2		H						
101	1	06/08/2019	28	692	1	10	0.08	24.5
1 primi 2		H						
101	1	06/09/2019	29	693	1	10.2	0	27.66
1 primi 2		H						
101	1	06/10/2019	30	694	1	10.6	0	25.31
1 primi 2		H						
101	1	06/11/2019	31	695	1	10	0	25.93
1 primi 2		H						
101	1	06/12/2019	32	696	1	10	0.07	28.8
1 primi 2		H						
101	1	06/13/2019	33	697	1	9.8	0	25.41
1 primi 2		H						
101	1	06/14/2019	34	698	1	9.6	0.07	26.77
1 primi 2		H						
101	1	06/15/2019	35	699	1	10	0	26.96
1 primi 2		H						
101	1	06/16/2019	36	700	1	9.8	0	25.56
1 primi 2		H						
101	1	06/17/2019	37	701	1	10	0	27.62
1 primi 2		H						
101	1	06/18/2019	38	702	1	10	0.08	28.01
1 primi 2		H						
101	1	06/19/2019	39	703	1	10	0	27.65
1 primi 2		H						
101	1	06/20/2019	40	704	1	9.9	0.08	29.74
1 primi 2		H						
101	1	06/21/2019	41	705	1	9.9	0	27.4
1 primi 2		H						
101	1	06/22/2019	42	706	1	9.3	0	28.17
1 primi 2		H						
101	1	06/23/2019	43	707	1	9.2	0.06	28.86
1 primi 2		H						
101	1	06/24/2019	44	708	1	9.6	0.06	29.82
1 primi 2		H						
101	1	06/25/2019	45	709	1	9.6	0	29.4
1 primi 2		H						
101	1	06/26/2019	46	710	1	9.8	0	29.39
1 primi 2		H						
101	1	06/27/2019	47	711	1	9.5	0.04	25.95
1 primi 2		H						
101	1	06/28/2019	48	712	1	9.8	0.07	28.75

1 primi 2		H						
101	1	06/29/2019	49	713	1	9.8	0.07	26.14
1 primi 2		H						
101	1	06/30/2019	50	714	1	9.9	0	27.74
1 primi 2		H						
101	1	07/01/2019	51	715	1	9.1	0.06	29.09
1 primi 2		H						
101	1	07/02/2019	52	716	1	9.2	0.16	29.05
1 primi 2		H						
101	1	07/03/2019	53	717	1	9.6	0.15	29.86
1 primi 2		H						
101	1	07/04/2019	54	718	1	9.5	0	28.35
1 primi 2		H						
101	1	07/05/2019	55	719	1	9.8	0	27.63
1 primi 2		H						
101	1	07/06/2019	56	720	1	9.6	0.08	28.71
1 primi 2		H						
101	1	07/07/2019	57	721	1	9.2	0.01	29.63
1 primi 2		H						
101	1	07/08/2019	58	722	1	9.8	0	29.14
1 primi 2		H						
101	1	07/09/2019	59	723	1	9.6	0	27.89
1 primi 2		H						
101	1	07/10/2019	60	724	1	9.2	0.06	29.06
1 primi 2		H						
101	1	07/11/2019	61	725	1	9.3	0	27.91
1 primi 2		H						
101	1	07/12/2019	62	726	1	9.2	0.1	29.97
1 primi 2		H						
101	1	07/13/2019	63	727	1	9.6	0	28.1
1 primi 2		H						
101	1	07/14/2019	64	728	1	9.8	0	29.06
1 primi 2		H						
101	1	07/15/2019	65	729	1	9.3	0	27.75
1 primi 2		H						
101	1	07/16/2019	66	730	1	9.5	0	29.94
1 primi 2		H						
101	1	07/17/2019	67	731	1	9.6	0.07	30.15
1 primi 2		H						
101	1	07/18/2019	68	732	1	9.6	0	27.11
1 primi 2		H						
101	1	07/19/2019	69	733	1	9.8	0.08	31.85
1 primi 2		H						
101	1	07/20/2019	70	734	1	9.6	0.07	27.15
1 primi 2		H						
101	1	07/21/2019	71	735	1	9.9	0.09	28.92
1 primi 2		H						
101	1	07/22/2019	72	736	1	8.9	0	27.63
1 primi 2		H						
101	1	07/23/2019	73	737	1	9.5	0.24	27.72
1 primi 2		H						
101	1	07/24/2019	74	738	1	9.8	0.15	30.06
1 primi 2		H						
101	1	07/25/2019	75	739	1	9.2	0.11	29.83

1 primi	2	H						
101	1	07/26/2019	76	740	1	9.3	0	27.99
1 primi	2	H						
101	1	07/27/2019	77	741	1	9.3	0.03	28.26
1 primi	2	H						
101	1	07/28/2019	78	742	1	9.6	0.08	28.81
1 primi	2	H						
101	1	07/29/2019	79	743	1	9.8	0.08	29.89
1 primi	2	H						
101	1	07/30/2019	80	744	1	9.8	0	30.4
1 primi	2	H						
101	1	07/31/2019	81	745	1	10	0	29.26
1 primi	2	H						
101	1	06/23/2020	6	1073	2	10	0.28	34.48
2 multi	1	H						
101	1	06/24/2020	7	1074	2	9.9	0.03	32.14
2 multi	1	H						
101	1	06/25/2020	8	1075	2	9.5	0	33.9
2 multi	1	H						
101	1	06/26/2020	9	1076	2	9.5	0	36.25
2 multi	1	H						
101	1	06/27/2020	10	1077	2	9.3	0	38.57
2 multi	1	H						
101	1	06/28/2020	11	1078	2	9.3	0	35.03
2 multi	1	H						
101	1	06/29/2020	12	1079	2	9.3	0	36.24
2 multi	1	H						
101	1	10/14/2020	119	1186	2	9.5	0.17	35.83
2 multi	2	H						
101	1	10/15/2020	120	1187	2	9.8	0.04	37.94
2 multi	2	H						
101	1	10/16/2020	121	1188	2	9.9	0.04	39.01
2 multi	2	H						
101	1	10/17/2020	122	1189	2	9.9	0.17	37.16
2 multi	2	H						
101	1	10/18/2020	123	1190	2	10	0.1	39.97
2 multi	2	H						
101	1	10/19/2020	124	1191	2	9.9	0.12	38.96
2 multi	2	H						
101	1	10/20/2020	125	1192	2	10.2	0.13	39.11
2 multi	2	H						
101	1	10/21/2020	126	1193	2	9.2	0.06	39.59
2 multi	2	H						
101	1	10/22/2020	127	1194	2	9.9	0.13	39.28
2 multi	2	H						
101	1	10/23/2020	128	1195	2	10	0.18	40.1
2 multi	2	H						
101	1	10/24/2020	129	1196	2	10.2	0.07	39.87
2 multi	2	H						
101	1	10/25/2020	130	1197	2	10.2	0.12	39.61
2 multi	2	H						
101	1	10/26/2020	131	1198	2	9.9	0.19	41.23
2 multi	2	H						
101	1	10/27/2020	132	1199	2	9.6	0.18	37.09

2 multi	2	H						
101	1	10/28/2020	133	1200	2	9.5	0.1	38.02
2 multi	2	H						
101	1	10/29/2020	134	1201	2	9.8	0.1	39.2
2 multi	2	H						
101	1	10/30/2020	135	1202	2	9.6	0.11	37.28
2 multi	2	H						
101	1	10/31/2020	136	1203	2	10	0.12	31.29
2 multi	2	H						
101	1	11/01/2020	137	1204	2	10	0.03	33.17
2 multi	2	H						
101	1	11/02/2020	138	1205	2	10.2	0.1	34.92
2 multi	2	H						
101	1	11/03/2020	139	1206	2	9.5	0.09	33.37
2 multi	2	H						
101	1	11/04/2020	140	1207	2	9.9	0.19	35.77
2 multi	2	H						
101	1	11/05/2020	141	1208	2	9.8	0.03	35.4
2 multi	2	H						
101	1	11/06/2020	142	1209	2	9.6	0.05	35.19
2 multi	2	H						
101	1	11/07/2020	143	1210	2	9.8	0.2	34.03
2 multi	2	H						
101	1	11/08/2020	144	1211	2	9.8	0.24	34.47
2 multi	2	H						
101	1	11/09/2020	145	1212	2	9.8	0.13	33.44
2 multi	2	H						
101	1	11/10/2020	146	1213	2	9.9	0.16	34.89
2 multi	2	H						
101	1	11/11/2020	147	1214	2	9.9	0.12	37.02
2 multi	2	H						
101	1	11/12/2020	148	1215	2	10.2	0.17	36.65
2 multi	2	H						
101	1	11/13/2020	149	1216	2	10.2	0.08	36.41
2 multi	2	H						
101	1	11/14/2020	150	1217	2	10	0.03	38.6
2 multi	2	H						
101	1	11/15/2020	151	1218	2	10	0.06	35.26
2 multi	2	H						
101	1	11/16/2020	152	1219	2	10	0.15	35.37
2 multi	2	H						
101	1	11/17/2020	153	1220	2	10.2	0.15	38.81
2 multi	2	H						
101	1	11/18/2020	154	1221	2	10	0.16	38.04
2 multi	2	H						
101	1	11/19/2020	155	1222	2	9.8	0.15	37.81
2 multi	2	H						
101	1	11/20/2020	156	1223	2	9.9	0.16	37.88
2 multi	2	H						
101	1	11/21/2020	157	1224	2	9.5	0.11	35.24
2 multi	2	H						
101	1	11/22/2020	158	1225	2	9.6	0.17	38.53
2 multi	2	H						
101	1	11/23/2020	159	1226	2	9.3	0.08	39.27

2 multi	2	H						
101	1	11/24/2020	160	1227	2	9.6	0.14	35.36
2 multi	2	H						
102	1	02/04/2021	6	792	1	8.6	0.24	24.05
1 primi	1	H						
102	1	02/05/2021	7	793	1	8.6	0.09	26.94
1 primi	1	H						
102	1	02/06/2021	8	794	1	8.7	0.14	25.82
1 primi	1	H						
102	1	02/07/2021	9	795	1	9.5	0.17	25.72
1 primi	1	H						
102	1	02/08/2021	10	796	1	9.1	0	25.94
1 primi	1	H						
102	1	02/09/2021	11	797	1	8.8	0.1	25.27
1 primi	1	H						
102	1	02/10/2021	12	798	1	9.9	0.1	24.94
1 primi	1	H						
102	1	02/11/2021	13	799	1	9.6	0.08	26.47
1 primi	1	H						
102	1	02/12/2021	14	800	1	9.2	0.06	25.99
1 primi	1	H						
102	1	02/13/2021	15	801	1	9.2	0	28.07
1 primi	1	H						
102	1	02/14/2021	16	802	1	8.8	0.06	26.99
1 primi	1	H						
102	1	02/15/2021	17	803	1	9.8	0.06	24.91
1 primi	1	H						
102	1	02/16/2021	18	804	1	9.8	0	22.49
1 primi	1	H						
102	1	02/17/2021	19	805	1	9.6	0	25.6
1 primi	1	H						
102	1	02/18/2021	20	806	1	10	0.05	23.57
1 primi	1	H						
102	1	02/19/2021	21	807	1	9.6	0	26.79
1 primi	1	H						
102	1	02/20/2021	22	808	1	10.2	0.05	27.34
1 primi	2	H						
102	1	02/21/2021	23	809	1	10	0.03	27.02
1 primi	2	H						
102	1	02/22/2021	24	810	1	10.2	0.04	27.73
1 primi	2	H						
102	1	02/23/2021	25	811	1	9.3	0.28	31.83
1 primi	2	H						
102	1	02/24/2021	26	812	1	10	0	32.03
1 primi	2	H						
102	1	02/25/2021	27	813	1	9.6	0.14	32.19
1 primi	2	H						
102	1	02/26/2021	28	814	1	8.7	0.04	31.61
1 primi	2	H						
102	1	02/27/2021	29	815	1	9.2	0	30.76
1 primi	2	H						
102	1	02/28/2021	30	816	1	9.2	0	33.11
1 primi	2	H						
102	1	03/01/2021	31	817	1	9.3	0.05	32.03

1 primi	2	H						
102	1	03/02/2021	32	818	1	9.5	0	32.23
1 primi	2	H						
102	1	03/03/2021	33	819	1	9.2	0.08	30.52
1 primi	2	H						
102	1	03/04/2021	34	820	1	8.9	0	33.2
1 primi	2	H						
102	1	03/05/2021	35	821	1	9.2	0.05	32.78
1 primi	2	H						
102	1	03/06/2021	36	822	1	9.1	0.06	33.21
1 primi	2	H						
102	1	03/07/2021	37	823	1	8.9	0	32.07
1 primi	2	H						
102	1	03/08/2021	38	824	1	9.2	0.05	31.3
1 primi	2	H						
102	1	03/09/2021	39	825	1	9.2	0.18	32.12
1 primi	2	H						
102	1	03/10/2021	40	826	1	9.3	0.08	31.71
1 primi	2	H						
102	1	03/11/2021	41	827	1	9.6	0.05	29.76
1 primi	2	H						
102	1	03/12/2021	42	828	1	9.3	0.07	33.08
1 primi	2	H						
102	1	03/13/2021	43	829	1	9.3	0.24	31.08
1 primi	2	H						
102	1	03/14/2021	44	830	1	9.2	0.34	33.38
1 primi	2	H						
102	1	03/15/2021	45	831	1	8.9	0.25	31.61
1 primi	2	H						
102	1	03/16/2021	46	832	1	9.1	0.24	32.83
1 primi	2	H						
102	1	03/17/2021	47	833	1	9.6	0.05	32.6
1 primi	2	H						
102	1	03/18/2021	48	834	1	9.8	0.15	31.08
1 primi	2	H						
102	1	03/19/2021	49	835	1	9.3	0	32.67
1 primi	2	H						
102	1	03/20/2021	50	836	1	9.5	0.25	32.16
1 primi	2	H						
102	1	03/21/2021	51	837	1	8.9	0.41	32.12
1 primi	2	H						
102	1	03/22/2021	52	838	1	9.3	0.24	32.73
1 primi	2	H						
102	1	03/23/2021	53	839	1	9.1	0.25	33.33
1 primi	2	H						
102	1	03/24/2021	54	840	1	9.3	0.28	33.44
1 primi	2	H						
102	1	03/25/2021	55	841	1	9.2	0	33.48
1 primi	2	H						
102	1	03/26/2021	56	842	1	9.2	0.41	34.49
1 primi	2	H						
102	1	03/27/2021	57	843	1	9.6	0.05	33.07
1 primi	2	H						
102	1	03/28/2021	58	844	1	9.5	0	33.77

1 primi 2		H						
102	1	03/29/2021	59	845	1	9.5	0.28	34.1
1 primi 2		H						
102	1	03/30/2021	60	846	1	9.2	0.37	35.81
1 primi 2		H						
102	1	03/31/2021	61	847	1	9.5	0.36	35.32
1 primi 2		H						
102	1	04/01/2021	62	848	1	10.6	0.06	34.49
1 primi 2		H						
102	1	04/02/2021	63	849	1	8.7	0.36	34.59
1 primi 2		H						
102	1	04/03/2021	64	850	1	9.2	0.15	35.51
1 primi 2		H						
102	1	04/04/2021	65	851	1	9.2	0.32	33.14
1 primi 2		H						
102	1	04/05/2021	66	852	1	9.3	0.27	32.68
1 primi 2		H						
102	1	04/06/2021	67	853	1	9.8	0	31.63
1 primi 2		H						
102	1	04/07/2021	68	854	1	9.3	0.12	29.62
1 primi 2		H						
102	1	04/08/2021	69	855	1	9.2	0.03	29.89
1 primi 2		H						
102	1	04/09/2021	70	856	1	8.8	0.19	30.56
1 primi 2		H						
102	1	04/10/2021	71	857	1	9.1	0	32.83
1 primi 2		H						
102	1	04/11/2021	72	858	1	9.3	0.35	33.62
1 primi 2		H						
102	1	04/12/2021	73	859	1	8.9	0.25	32.98
1 primi 2		H						
102	1	04/13/2021	74	860	1	9.6	0.34	33.18
1 primi 2		H						
102	1	04/14/2021	75	861	1	9.3	0.03	32.45
1 primi 2		H						
102	1	04/15/2021	76	862	1	9.2	0	32.33
1 primi 2		H						
102	1	04/16/2021	77	863	1	9.8	0.04	35.19
1 primi 2		H						
102	1	04/17/2021	78	864	1	9.3	0.04	33.99
1 primi 2		H						
102	1	04/18/2021	79	865	1	9.8	0.38	37.19
1 primi 2		H						
102	1	04/19/2021	80	866	1	9.6	0	35.88
1 primi 2		H						
102	1	04/20/2021	81	867	1	10.2	0.28	36.27
1 primi 2		H						
102	1	04/21/2021	82	868	1	10	0.11	35.87
1 primi 2		H						
102	1	04/22/2021	83	869	1	9.3	0.04	36.42
1 primi 2		H						
102	1	04/23/2021	84	870	1	10.4	0.04	38.62
1 primi 2		H						
102	1	04/24/2021	85	871	1	10	0.04	34.43

1 primi	2	H						
102	1	04/25/2021	86	872	1	9.8	0.03	38.36
1 primi	2	H						
102	1	04/26/2021	87	873	1	10.4	0.09	34.81
1 primi	2	H						
102	1	04/27/2021	88	874	1	9.3	0	33.64
1 primi	2	H						
102	1	04/28/2021	89	875	1	9.6	0	37.44
1 primi	2	H						
102	1	04/29/2021	90	876	1	10.3	0.04	37.74
1 primi	2	H						
102	1	04/30/2021	91	877	1	10.6	0.19	36.73
1 primi	2	H						
102	1	05/01/2021	92	878	1	10.9	0.06	36.27
1 primi	2	H						
102	1	05/02/2021	93	879	1	9.8	0	33.56
1 primi	2	H						
102	1	05/03/2021	94	880	1	9.9	0.03	33.54
1 primi	2	H						
102	1	05/04/2021	95	881	1	10.4	0	35.35
1 primi	2	H						
102	1	05/05/2021	96	882	1	11.1	0.21	35.53
1 primi	2	H						
102	1	05/06/2021	97	883	1	10	0	34.25
1 primi	2	H						
102	1	05/07/2021	98	884	1	10	0	36.18
1 primi	2	H						
102	1	05/08/2021	99	885	1	10.6	0.19	33.86
1 primi	2	H						
102	1	05/09/2021	100	886	1	10.4	0	36.94
1 primi	2	H						
102	1	05/10/2021	101	887	1	10.4	0.05	38.09
1 primi	2	H						
102	1	05/11/2021	102	888	1	10.4	0.03	36.97
1 primi	2	H						
102	1	05/12/2021	103	889	1	9.3	0.37	36.95
1 primi	2	H						
102	1	05/13/2021	104	890	1	9.8	0.38	37.71
1 primi	2	H						
102	1	05/14/2021	105	891	1	9.5	0.31	37.82
1 primi	2	H						
102	1	05/15/2021	106	892	1	10	0.3	38.6
1 primi	2	H						
102	1	05/16/2021	107	893	1	9.8	0.03	37.76
1 primi	2	H						
102	1	05/17/2021	108	894	1	9.8	0.31	37.76
1 primi	2	H						
102	1	05/18/2021	109	895	1	9.8	0.33	37.11
1 primi	2	H						
102	1	05/19/2021	110	896	1	9.3	0.14	35.15
1 primi	2	H						
102	1	05/20/2021	111	897	1	9.8	0.02	37.04
1 primi	2	H						
102	1	05/21/2021	112	898	1	9.5	0.03	37.71

1 primi	2	H						
102	1	05/22/2021	113	899	1	9.3	0.36	37.66
1 primi	2	H						
102	1	05/23/2021	114	900	1	10.4	0.04	39.28
1 primi	2	H						
102	1	05/24/2021	115	901	1	10.9	0.05	39.26
1 primi	2	H						
102	1	05/25/2021	116	902	1	10.7	0	38.26
1 primi	2	H						
102	1	05/26/2021	117	903	1	9.5	0	18.59
1 primi	2	H						
103	1	04/20/2018	10	778	1	10	0.02	22.15
1 primi	1	H						
103	1	04/21/2018	11	779	1	10.3	0	23.93
1 primi	1	H						
103	1	04/22/2018	12	780	1	9.9	0	24.15
1 primi	1	H						
103	1	04/23/2018	13	781	1	9.8	0	25.09
1 primi	1	H						
103	1	04/24/2018	14	782	1	10	0.02	25.63
1 primi	1	H						
103	1	04/25/2018	15	783	1	10	0	24.04
1 primi	1	H						
103	1	04/26/2018	16	784	1	9.5	0.05	23.72
1 primi	1	H						
103	1	04/27/2018	17	785	1	9.6	0.02	24.86
1 primi	1	H						
103	1	04/28/2018	18	786	1	10	0.01	25.06
1 primi	1	H						
103	1	04/29/2018	19	787	1	10	0.02	24.93
1 primi	1	H						
103	1	04/30/2018	20	788	1	9.8	0	23.81
1 primi	1	H						
103	1	05/01/2018	21	789	1	9.8	0	24.19
1 primi	1	H						
103	1	05/02/2018	22	790	1	9.9	0	8.36
1 primi	2	H						
103	1	05/03/2018	23	791	1	10.2	0	22.87
1 primi	2	H						
103	1	05/04/2018	24	792	1	10.4	0	22.04
1 primi	2	H						
103	1	05/05/2018	25	793	1	10.2	0	24.63
1 primi	2	H						
103	1	05/06/2018	26	794	1	10.2	0	24.36
1 primi	2	H						
103	1	05/07/2018	27	795	1	10.2	0	24.24
1 primi	2	H						
103	1	05/08/2018	28	796	1	10.4	0	22.5
1 primi	2	H						
103	1	05/09/2018	29	797	1	10.2	0	23.85
1 primi	2	H						
103	1	05/10/2018	30	798	1	10.4	0	24.61
1 primi	2	H						
103	1	05/11/2018	31	799	1	10.3	0	24.18

1 primi 2 H
103 1 05/12/2018 32 800 1 9.8 0 23.14
1 primi 2 H
103 1 05/13/2018 33 801 1 10.3 0 23.43
1 primi 2 H
103 1 05/14/2018 34 802 1 10.2 0 25.72
1 primi 2 H
103 1 05/15/2018 35 803 1 9.9 0 25.26
1 primi 2 H
103 1 05/16/2018 36 804 1 9.8 0 23.56
1 primi 2 H
103 1 05/17/2018 37 805 1 9.8 0 24.28
1 primi 2 H
103 1 05/18/2018 38 806 1 10 0 24.17
1 primi 2 H
103 1 05/19/2018 39 807 1 9.8 0 24.3
1 primi 2 H
103 1 05/20/2018 40 808 1 9.8 0 21.11
1 primi 2 H
103 1 05/21/2018 41 809 1 9.3 0.01 25.81
1 primi 2 H
103 1 05/22/2018 42 810 1 10.3 0 25
1 primi 2 H
103 1 05/23/2018 43 811 1 10 0 26.44
1 primi 2 H
103 1 05/24/2018 44 812 1 9.8 0 27.32
1 primi 2 H
103 1 05/25/2018 45 813 1 10 0 27.04
1 primi 2 H
103 1 05/26/2018 46 814 1 10 0.12 24.47
1 primi 2 H
103 1 05/27/2018 47 815 1 9.9 0 26.78
1 primi 2 H
103 1 05/28/2018 48 816 1 10.3 0 26.37
1 primi 2 H
103 1 05/29/2018 49 817 1 10.4 0 25.17
1 primi 2 H
103 1 05/30/2018 50 818 1 10.6 0 26.41
1 primi 2 H
103 1 05/31/2018 51 819 1 10.3 0 24.82
1 primi 2 H
103 1 06/01/2018 52 820 1 10.4 0 25.93
1 primi 2 H
103 1 06/02/2018 53 821 1 10.9 0.05 17.39
1 primi 2 H
103 1 06/03/2018 54 822 1 11.9 0.17 24.68
1 primi 2 H
103 1 06/04/2018 55 823 1 11.4 0 26.08
1 primi 2 H
103 1 06/05/2018 56 824 1 9.6 0 22.34
1 primi 2 H
103 1 06/06/2018 57 825 1 11.3 0.09 25.6
1 primi 2 H
103 1 06/07/2018 58 826 1 10.9 0.02 27.99

1 primi	2	H						
103	1	06/08/2018	59	827	1	11.1	0	25.56
1 primi	2	H						
103	1	06/09/2018	60	828	1	11.3	0	24.32
1 primi	2	H						
103	1	06/10/2018	61	829	1	10.7	0	25.72
1 primi	2	H						
103	1	06/11/2018	62	830	1	11.1	0	25.37
1 primi	2	H						
103	1	06/12/2018	63	831	1	10.6	0	24.96
1 primi	2	H						
103	1	06/13/2018	64	832	1	10.7	0	24.89
1 primi	2	H						
103	1	06/14/2018	65	833	1	10.6	0	26.62
1 primi	2	H						
103	1	06/15/2018	66	834	1	10.7	0.05	26.5
1 primi	2	H						
103	1	06/16/2018	67	835	1	10.6	0	25.46
1 primi	2	H						
103	1	06/17/2018	68	836	1	10.7	0	26.62
1 primi	2	H						
103	1	06/18/2018	69	837	1	10.9	0	26.7
1 primi	2	H						
103	1	06/19/2018	70	838	1	10.9	0.01	25.17
1 primi	2	H						
103	1	06/20/2018	71	839	1	10.4	0	26.53
1 primi	2	H						
103	1	06/21/2018	72	840	1	11.4	0.07	24.4
1 primi	2	H						
103	1	06/22/2018	73	841	1	11.9	0.03	23.55
1 primi	2	H						
103	1	06/23/2018	74	842	1	11.4	0	22.77
1 primi	2	H						
103	1	06/24/2018	75	843	1	11.1	0	22.92
1 primi	2	H						
103	1	06/25/2018	76	844	1	11.3	0	23.51
1 primi	2	H						
103	1	06/26/2018	77	845	1	10.7	0	22.13
1 primi	2	H						
103	1	06/27/2018	78	846	1	11.1	0	23.65
1 primi	2	H						
103	1	06/28/2018	79	847	1	10.7	0.07	23.53
1 primi	2	H						
103	1	06/29/2018	80	848	1	11.1	0	21.07
1 primi	2	H						
103	1	06/30/2018	81	849	1	11.3	0	25.13
1 primi	2	H						
103	1	07/01/2018	82	850	1	10.7	0.03	24.14
1 primi	2	H						
103	1	07/02/2018	83	851	1	10.7	0	23.5
1 primi	2	H						
103	1	07/03/2018	84	852	1	11.1	0.2	23.99
1 primi	2	H						
103	1	07/04/2018	85	853	1	11.1	0.23	25.04

1 primi	2	H						
103	1	07/05/2018	86	854	1	11.7	0	27.09
1 primi	2	H						
103	1	07/06/2018	87	855	1	10.9	0	25.49
1 primi	2	H						
103	1	07/07/2018	88	856	1	11.3	0	25.5
1 primi	2	H						
103	1	07/08/2018	89	857	1	11.1	0	24.83
1 primi	2	H						
103	1	07/09/2018	90	858	1	10.6	0	23.8
1 primi	2	H						
103	1	07/10/2018	91	859	1	10.4	0	20.18
1 primi	2	H						
103	1	07/11/2018	92	860	1	10.9	0	20.49
1 primi	2	H						
103	1	07/12/2018	93	861	1	10.6	0	23.23
1 primi	2	H						
103	1	08/31/2019	3	1276	2	10.3	0.21	27.78
2 multi	1	H						
103	1	09/01/2019	4	1277	2	10.2	0.24	29.13
2 multi	1	H						
103	1	09/02/2019	5	1278	2	9.9	0.03	30.23
2 multi	1	H						
103	1	09/03/2019	6	1279	2	9.8	0.06	32.56
2 multi	1	H						
103	1	09/04/2019	7	1280	2	9.6	0	32.66
2 multi	1	H						
103	1	09/05/2019	8	1281	2	9.1	0.02	36.32
2 multi	1	H						
103	1	09/06/2019	9	1282	2	9.6	0	34.03
2 multi	1	H						
103	1	09/07/2019	10	1283	2	9.5	0.01	34.39
2 multi	1	H						
103	1	09/08/2019	11	1284	2	9.5	0.02	35.29
2 multi	1	H						
103	1	09/09/2019	12	1285	2	8.8	0.08	36.34
2 multi	1	H						
103	1	09/10/2019	13	1286	2	8.9	0.01	35.99
2 multi	1	H						
103	1	09/11/2019	14	1287	2	9.1	0	37.51
2 multi	1	H						
103	1	09/12/2019	15	1288	2	8.9	0.12	37.49
2 multi	1	H						
103	1	12/17/2019	111	1384	2	10	0.18	29.57
2 multi	2	H						
103	1	12/18/2019	112	1385	2	10.2	0	33.36
2 multi	2	H						
103	1	12/19/2019	113	1386	2	10.3	0	30.81
2 multi	2	H						
103	1	12/20/2019	114	1387	2	10.6	0	31.68
2 multi	2	H						
103	1	12/21/2019	115	1388	2	10	0	33.73
2 multi	2	H						
103	1	12/22/2019	116	1389	2	10.3	0	31.16

2 multi	2	H						
103	1	12/23/2019	117	1390	2	10.2	0	31.53
2 multi	2	H						
103	1	12/24/2019	118	1391	2	10.4	0	33.72
2 multi	2	H						
103	1	12/25/2019	119	1392	2	10.2	0.01	30.97
2 multi	2	H						
103	1	11/02/2020	4	1705	3	10.2	0.27	30.35
2 multi	1	H						
103	1	11/03/2020	5	1706	3	10.4	0.13	35.64
2 multi	1	H						
103	1	11/04/2020	6	1707	3	10.4	0.24	33.52
2 multi	1	H						
103	1	11/05/2020	7	1708	3	11.1	0.08	35.1
2 multi	1	H						
103	1	11/06/2020	8	1709	3	12	0.02	32.02
2 multi	1	H						
103	1	11/07/2020	9	1710	3	11.3	0.33	32.78
2 multi	1	H						
103	1	11/08/2020	10	1711	3	10.7	0.1	39.49
2 multi	1	H						
103	1	11/09/2020	11	1712	3	10.6	0.11	37.16
2 multi	1	H						
103	1	11/10/2020	12	1713	3	11.7	0.11	39.12
2 multi	1	H						
103	1	11/11/2020	13	1714	3	10.4	0.02	37.57
2 multi	1	H						
103	1	11/12/2020	14	1715	3	10.6	0.07	40.15
2 multi	1	H						
103	1	11/13/2020	15	1716	3	10.4	0.18	43.72
2 multi	1	H						
103	1	11/14/2020	16	1717	3	10.4	0.07	40.23
2 multi	1	H						
103	1	11/15/2020	17	1718	3	10.3	0.02	43.44
2 multi	1	H						
103	1	11/16/2020	18	1719	3	10.3	0.06	44.85
2 multi	1	H						
103	1	11/17/2020	19	1720	3	9.8	0.06	42.02
2 multi	1	H						
103	1	11/18/2020	20	1721	3	10.6	0.06	44.4
2 multi	1	H						
103	1	11/19/2020	21	1722	3	10.4	0.12	43.82
2 multi	1	H						
103	1	11/20/2020	22	1723	3	10.9	0.07	44.47
2 multi	2	H						
103	1	11/21/2020	23	1724	3	11.7	0.04	43
2 multi	2	H						
103	1	11/22/2020	24	1725	3	9.2	0.07	42.69
2 multi	2	H						
103	1	11/23/2020	25	1726	3	10.3	0	46.05
2 multi	2	H						
103	1	11/24/2020	26	1727	3	10.3	0	40.04
2 multi	2	H						
103	1	11/25/2020	27	1728	3	10.3	0.05	34.29

2 multi	2	H						
103	1	11/26/2020	28	1729	3	10.6	0.05	34.58
2 multi	2	H						
103	1	11/27/2020	29	1730	3	10.6	0.01	35.97
2 multi	2	H						
103	1	11/28/2020	30	1731	3	10.9	0.07	39.25
2 multi	2	H						
103	1	11/29/2020	31	1732	3	10.7	0	36.08
2 multi	2	H						
103	1	11/30/2020	32	1733	3	11.3	0	36.08
2 multi	2	H						
103	1	12/01/2020	33	1734	3	10.4	0.05	38.9
2 multi	2	H						
103	1	12/02/2020	34	1735	3	10.9	0.25	39.55
2 multi	2	H						
103	1	12/03/2020	35	1736	3	10.6	0	38.19
2 multi	2	H						
103	1	12/04/2020	36	1737	3	10.4	0.14	39.99
2 multi	2	H						
103	1	12/05/2020	37	1738	3	10.7	0.07	40.76
2 multi	2	H						
103	1	12/06/2020	38	1739	3	10.7	0.05	41.01
2 multi	2	H						
103	1	12/07/2020	39	1740	3	10.9	0.65	42.86
2 multi	2	H						
103	1	12/08/2020	40	1741	3	11.7	0	37.61
2 multi	2	H						
103	1	12/09/2020	41	1742	3	10.7	0.13	37.39
2 multi	2	H						
103	1	12/10/2020	42	1743	3	11.3	0.39	36.42
2 multi	2	H						
103	1	12/11/2020	43	1744	3	10.9	0.05	41.92
2 multi	2	H						
103	1	12/12/2020	44	1745	3	10.7	0	39.12
2 multi	2	H						
103	1	12/13/2020	45	1746	3	10.2	0.03	39.24
2 multi	2	H						
103	1	12/14/2020	46	1747	3	10.3	0.02	41.34
2 multi	2	H						
103	1	12/15/2020	47	1748	3	10.7	0	39.55
2 multi	2	H						
103	1	12/16/2020	48	1749	3	10.4	0	40.14
2 multi	2	H						
103	1	12/17/2020	49	1750	3	10.6	0.02	40.24
2 multi	2	H						
103	1	12/18/2020	50	1751	3	10.7	0.02	39.21
2 multi	2	H						
103	1	12/19/2020	51	1752	3	10.6	0.04	40.76
2 multi	2	H						
103	1	12/20/2020	52	1753	3	11.1	0	38.93
2 multi	2	H						
103	1	12/21/2020	53	1754	3	10.9	0.06	39.16
2 multi	2	H						
103	1	12/22/2020	54	1755	3	11.3	0.05	39.57

2 multi 2		H						
103	1	12/23/2020	55	1756	3	11.3	0.1	37.4
2 multi 2		H						
103	1	12/24/2020	56	1757	3	11.1	0.04	38.06
2 multi 2		H						
103	1	12/25/2020	57	1758	3	10.9	0.04	40.36
2 multi 2		H						
103	1	12/26/2020	58	1759	3	10.7	0.04	39.02
2 multi 2		H						
103	1	12/27/2020	59	1760	3	10.9	0.05	39.77
2 multi 2		H						
103	1	12/28/2020	60	1761	3	10.7	0.04	42.99
2 multi 2		H						
103	1	12/29/2020	61	1762	3	11.1	0.05	42.65
2 multi 2		H						
103	1	12/30/2020	62	1763	3	11.1	0.84	38.11
2 multi 2		H						
103	1	12/31/2020	63	1764	3	10.6	0.1	38.55
2 multi 2		H						
103	1	01/01/2021	64	1765	3	10	0.12	38.29
2 multi 2		H						
103	1	01/02/2021	65	1766	3	9.8	0.25	37.6
2 multi 2		H						
103	1	01/03/2021	66	1767	3	9.8	0.43	41.16
2 multi 2		H						
103	1	01/04/2021	67	1768	3	10.9	0.03	38.33
2 multi 2		H						
103	1	01/05/2021	68	1769	3	10.4	0.07	37.81
2 multi 2		H						
103	1	01/06/2021	69	1770	3	10.7	0.05	40.32
2 multi 2		H						
103	1	01/07/2021	70	1771	3	10.6	0.06	37.73
2 multi 2		H						
103	1	01/08/2021	71	1772	3	10.7	0	34.87
2 multi 2		H						
103	1	01/09/2021	72	1773	3	10.7	0	38.29
2 multi 2		H						
103	1	01/10/2021	73	1774	3	10.7	0	38.31
2 multi 2		H						
103	1	01/11/2021	74	1775	3	10.3	0.04	38.73
2 multi 2		H						
103	1	01/12/2021	75	1776	3	10.6	0.05	40.22
2 multi 2		H						
103	1	01/13/2021	76	1777	3	10.9	0.04	39.62
2 multi 2		H						
103	1	01/14/2021	77	1778	3	10.9	0.66	36.83
2 multi 2		H						
103	1	01/15/2021	78	1779	3	10.3	0.05	36.08
2 multi 2		H						
103	1	01/16/2021	79	1780	3	9.3	0	36.66
2 multi 2		H						
103	1	01/17/2021	80	1781	3	9.8	0.02	39.63
2 multi 2		H						
103	1	01/18/2021	81	1782	3	10.3	0	37.71

2 multi 2		H						
103	1	01/19/2021	82	1783	3	10.3	0.26	37.28
2 multi 2		H						
103	1	01/20/2021	83	1784	3	11.1	0.05	39.95
2 multi 2		H						
103	1	01/21/2021	84	1785	3	10.6	0	36.84
2 multi 2		H						
103	1	01/22/2021	85	1786	3	10.6	0.07	40.75
2 multi 2		H						
103	1	01/23/2021	86	1787	3	10.6	0.03	34.8
2 multi 2		H						
103	1	01/24/2021	87	1788	3	11.1	0.03	36.54
2 multi 2		H						
103	1	01/25/2021	88	1789	3	10.9	0.04	36.57
2 multi 2		H						
103	1	01/26/2021	89	1790	3	10.7	0.04	36.56
2 multi 2		H						
103	1	01/27/2021	90	1791	3	10.4	0.03	37.1
2 multi 2		H						
103	1	01/28/2021	91	1792	3	10.7	0.13	37.78
2 multi 2		H						
103	1	01/29/2021	92	1793	3	10.6	0.45	38.63
2 multi 2		H						
103	1	01/30/2021	93	1794	3	9.9	0.17	34.73
2 multi 2		H						
103	1	01/31/2021	94	1795	3	9.8	0.05	37.62
2 multi 2		H						
103	1	02/01/2021	95	1796	3	10.3	0.05	34.61
2 multi 2		H						
103	1	02/02/2021	96	1797	3	10.7	0.05	37.96
2 multi 2		H						
103	1	02/03/2021	97	1798	3	11.1	0.07	38.53
2 multi 2		H						
103	1	02/04/2021	98	1799	3	11.3	0.04	36.34
2 multi 2		H						
103	1	02/05/2021	99	1800	3	10.3	0.12	37.28
2 multi 2		H						
103	1	02/06/2021	100	1801	3	10.2	0.02	35.86
2 multi 2		H						
103	1	02/07/2021	101	1802	3	10.6	0.03	36.5
2 multi 2		H						
103	1	02/08/2021	102	1803	3	10.4	0.06	37.44
2 multi 2		H						
103	1	02/09/2021	103	1804	3	10.4	0.05	35.55
2 multi 2		H						
103	1	02/10/2021	104	1805	3	10.6	0.05	37.73
2 multi 2		H						
103	1	02/11/2021	105	1806	3	10.4	0	35.81
2 multi 2		H						
103	1	02/12/2021	106	1807	3	11.3	0.05	33.72
2 multi 2		H						
103	1	02/13/2021	107	1808	3	11.1	0	35.59
2 multi 2		H						
103	1	02/14/2021	108	1809	3	11.1	0	36.04

2 multi 2		H						
103	1	02/15/2021	109	1810	3	10.3	0	32.94
2 multi 2		H						
103	1	02/16/2021	110	1811	3	10.4	0.04	35.6
2 multi 2		H						
103	1	02/17/2021	111	1812	3	10.7	0.04	35.94
2 multi 2		H						
103	1	02/18/2021	112	1813	3	10.6	0	37.67
2 multi 2		H						
103	1	02/19/2021	113	1814	3	10.6	0.03	32.3
2 multi 2		H						
103	1	02/20/2021	114	1815	3	10.6	0.02	35.8
2 multi 2		H						
103	1	02/21/2021	115	1816	3	10.6	0.03	36.04
2 multi 2		H						
103	1	02/22/2021	116	1817	3	10.6	0.14	36.29
2 multi 2		H						
103	1	02/23/2021	117	1818	3	10.7	0	32.64
2 multi 2		H						
103	1	02/24/2021	118	1819	3	10.3	0.05	34.14
2 multi 2		H						
103	1	02/25/2021	119	1820	3	10.4	0.03	35.74
2 multi 2		H						
103	1	02/26/2021	120	1821	3	10.7	0	38.59
2 multi 2		H						
103	1	02/27/2021	121	1822	3	10.4	0.18	34.17
2 multi 2		H						
103	1	02/28/2021	122	1823	3	10.4	0.26	36.28
2 multi 2		H						
103	1	03/01/2021	123	1824	3	10.3	0.28	37.11
2 multi 2		H						
103	1	03/02/2021	124	1825	3	10.7	0.04	32.43
2 multi 2		H						
103	1	03/03/2021	125	1826	3	10.2	0.03	35.55
2 multi 2		H						
103	1	03/04/2021	126	1827	3	10	0	32.8
2 multi 2		H						
103	1	03/05/2021	127	1828	3	10.3	0.24	34.47
2 multi 2		H						
103	1	03/06/2021	128	1829	3	10.6	0.04	36.32
2 multi 2		H						
104	1	05/11/2020	4	805	1	9.8	0.33	25.15
1 primi 1		H						
104	1	05/12/2020	5	806	1	0	0.04	29.15
1 primi 1		H						
104	1	05/13/2020	6	807	1	10.2	0.14	26
1 primi 1		H						
104	1	05/14/2020	7	808	1	9.3	0	29.53
1 primi 1		H						
104	1	05/15/2020	8	809	1	9.5	0.31	29.65
1 primi 1		H						
104	1	05/16/2020	9	810	1	9.9	0.02	29.47
1 primi 1		H						
104	1	05/17/2020	10	811	1	9.3	0	27.55

1 primi 1		H						
104	1	05/18/2020	11	812	1	9.3	0.08	26.71
1 primi 1		H						
104	1	05/19/2020	12	813	1	9.5	0	31.19
1 primi 1		H						
104	1	05/20/2020	13	814	1	9.2	0.08	30.84
1 primi 1		H						
104	1	05/21/2020	14	815	1	9.5	0	30.74
1 primi 1		H						
104	1	05/22/2020	15	816	1	9.6	0.09	33.07
1 primi 1		H						
104	1	05/23/2020	16	817	1	9.6	0.1	31.87
1 primi 1		H						
104	1	05/24/2020	17	818	1	9.5	0.08	33.74
1 primi 1		H						
104	1	05/25/2020	18	819	1	9.5	0	32.52
1 primi 1		H						
104	1	05/26/2020	19	820	1	9.5	0.02	35.65
1 primi 1		H						
104	1	05/27/2020	20	821	1	9.2	0.02	37.14
1 primi 1		H						
104	1	05/28/2020	21	822	1	9.3	0.1	33.84
1 primi 1		H						
104	1	05/29/2020	22	823	1	9.2	0.02	36.95
1 primi 2		H						
104	1	05/30/2020	23	824	1	9.5	0	34.94
1 primi 2		H						
104	1	05/31/2020	24	825	1	9.2	0.09	33.58
1 primi 2		H						
104	1	06/01/2020	25	826	1	9.1	0.08	33.83
1 primi 2		H						
104	1	06/02/2020	26	827	1	9.2	0	35.92
1 primi 2		H						
104	1	06/03/2020	27	828	1	9.2	0	36.38
1 primi 2		H						
104	1	06/04/2020	28	829	1	9.6	0.07	36.62
1 primi 2		H						
104	1	06/05/2020	29	830	1	9.5	0	37.69
1 primi 2		H						
104	1	06/06/2020	30	831	1	9.5	0.08	34.58
1 primi 2		H						
104	1	06/07/2020	31	832	1	9.3	0.09	38.1
1 primi 2		H						
104	1	06/08/2020	32	833	1	9.6	0.01	40.82
1 primi 2		H						
104	1	06/09/2020	33	834	1	9.2	0.09	36.97
1 primi 2		H						
104	1	06/10/2020	34	835	1	9.8	0	42.05
1 primi 2		H						
104	1	06/11/2020	35	836	1	9.3	0.07	39.7
1 primi 2		H						
104	1	06/12/2020	36	837	1	9.3	0	40.99
1 primi 2		H						
104	1	06/13/2020	37	838	1	9.5	0.08	40.57

1 primi 2		H						
104	1	06/14/2020	38	839	1	9.6	0	40.15
1 primi 2		H						
104	1	06/15/2020	39	840	1	9.5	0	41.51
1 primi 2		H						
104	1	06/16/2020	40	841	1	9.5	0.08	40.73
1 primi 2		H						
104	1	06/17/2020	41	842	1	9.2	0	42.87
1 primi 2		H						
104	1	06/18/2020	42	843	1	9.5	0	41.49
1 primi 2		H						
104	1	06/19/2020	43	844	1	9.6	0.05	42.8
1 primi 2		H						
104	1	06/20/2020	44	845	1	9.6	0.02	42.03
1 primi 2		H						
104	1	06/21/2020	45	846	1	9.5	0.07	40.92
1 primi 2		H						
104	1	06/22/2020	46	847	1	9.5	0.09	40.84
1 primi 2		H						
104	1	06/23/2020	47	848	1	9.5	0.02	45.62
1 primi 2		H						
104	1	06/24/2020	48	849	1	9.3	0	41.28
1 primi 2		H						
104	1	06/25/2020	49	850	1	9.5	0	42.33
1 primi 2		H						
104	1	06/26/2020	50	851	1	9.6	0	43.06
1 primi 2		H						
104	1	06/27/2020	51	852	1	9.3	0	41.87
1 primi 2		H						
104	1	06/28/2020	52	853	1	9.2	0	39.74
1 primi 2		H						
104	1	06/29/2020	53	854	1	9.5	0	40.44
1 primi 2		H						
104	1	06/30/2020	54	855	1	9.8	0	39.72
1 primi 2		H						
104	1	07/01/2020	55	856	1	9.6	0	43.21
1 primi 2		H						
104	1	07/02/2020	56	857	1	9.1	0	38.33
1 primi 2		H						
104	1	07/03/2020	57	858	1	9.5	0.2	46.97
1 primi 2		H						
104	1	07/04/2020	58	859	1	9.5	0.18	41.11
1 primi 2		H						
104	1	07/05/2020	59	860	1	9.5	0.23	39.35
1 primi 2		H						
104	1	07/06/2020	60	861	1	9.6	0	40.64
1 primi 2		H						
104	1	07/07/2020	61	862	1	9.2	0.2	40.55
1 primi 2		H						
104	1	07/08/2020	62	863	1	9.6	0.2	38.91
1 primi 2		H						
104	1	07/09/2020	63	864	1	9.5	0.14	40.15
1 primi 2		H						
104	1	07/10/2020	64	865	1	9.6	0.03	45.84

1 primi 2		H						
104	1	07/11/2020	65	866	1	9.2	0.13	38.22
1 primi 2		H						
104	1	07/12/2020	66	867	1	9.8	0	43.75
1 primi 2		H						
104	1	07/13/2020	67	868	1	9.1	0.12	39.77
1 primi 2		H						
104	1	07/14/2020	68	869	1	9.5	0.02	42.48
1 primi 2		H						
104	1	07/15/2020	69	870	1	9.3	0	34.38
1 primi 2		H						
104	1	07/16/2020	70	871	1	9.3	0	22.68
1 primi 2		H						
104	1	07/17/2020	71	872	1	8.8	0.11	27.87
1 primi 2		H						
104	1	07/18/2020	72	873	1	9.2	0.17	30.1
1 primi 2		H						
104	1	07/19/2020	73	874	1	9.8	0.04	31.5
1 primi 2		H						
104	1	07/20/2020	74	875	1	9.9	0.1	32.98
1 primi 2		H						
104	1	07/21/2020	75	876	1	9.8	0	33.55
1 primi 2		H						
104	1	07/22/2020	76	877	1	9.8	0	34.84
1 primi 2		H						
104	1	07/23/2020	77	878	1	9.8	0.03	34.69
1 primi 2		H						
104	1	07/24/2020	78	879	1	9.8	0.03	36.34
1 primi 2		H						
104	1	07/25/2020	79	880	1	9.6	0	34.51
1 primi 2		H						
104	1	07/26/2020	80	881	1	9.9	0.12	36.16
1 primi 2		H						
104	1	07/27/2020	81	882	1	9.8	0.2	33.98
1 primi 2		H						
104	1	07/28/2020	82	883	1	9.9	0	36.07
1 primi 2		H						
104	1	07/29/2020	83	884	1	10.2	0	39.06
1 primi 2		H						
104	1	07/30/2020	84	885	1	9.8	0.12	36.09
1 primi 2		H						
104	1	07/31/2020	85	886	1	9.8	0	37.31
1 primi 2		H						
104	1	08/01/2020	86	887	1	9.9	0.03	37.31
1 primi 2		H						
104	1	08/02/2020	87	888	1	9.8	0.1	42.45
1 primi 2		H						
104	1	08/03/2020	88	889	1	9.6	0.04	38.85
1 primi 2		H						
104	1	08/04/2020	89	890	1	9.6	0.12	37.41
1 primi 2		H						
104	1	08/05/2020	90	891	1	9.2	0.13	30.42
1 primi 2		H						
104	1	08/06/2020	91	892	1	9.6	0	34.81

1 primi 2	H
104	1	08/07/2020	92	893	1	9.9	0.11	36.65
1 primi 2	H
104	1	08/08/2020	93	894	1	9.5	0.13	37.96
1 primi 2	H
104	1	08/09/2020	94	895	1	9.5	0	37.25
1 primi 2	H
104	1	08/10/2020	95	896	1	9.9	0.13	40.21
1 primi 2	H
104	1	08/11/2020	96	897	1	10	0.13	36.85
1 primi 2	H
104	1	08/12/2020	97	898	1	10	0	37.82
1 primi 2	H
104	1	08/13/2020	98	899	1	10	0.13	37.44
1 primi 2	H
104	1	08/14/2020	99	900	1	10	0	35.48
1 primi 2	H
104	1	08/15/2020	100	901	1	10	0	35.95
1 primi 2	H
104	1	08/16/2020	101	902	1	10	0.12	34.95
1 primi 2	H
104	1	08/17/2020	102	903	1	10.2	0	33.62
1 primi 2	H
104	1	08/18/2020	103	904	1	10	0.04	34.12
1 primi 2	H
104	1	08/19/2020	104	905	1	10	0.13	38.3
1 primi 2	H
104	1	08/20/2020	105	906	1	10.2	0.12	36.22
1 primi 2	H
104	1	08/21/2020	106	907	1	10.2	0.06	37.64
1 primi 2	H
104	1	08/22/2020	107	908	1	10.3	0.04	37.25
1 primi 2	H
104	1	08/23/2020	108	909	1	10.2	0.05	40.13
1 primi 2	H
104	1	08/24/2020	109	910	1	10	0.03	34.89
1 primi 2	H
104	1	08/25/2020	110	911	1	9.9	0.13	35.55
1 primi 2	H
104	1	08/26/2020	111	912	1	10	0.05	32.65
1 primi 2	H
104	1	08/27/2020	112	913	1	9.8	0	31.27
1 primi 2	H
104	1	08/28/2020	113	914	1	9.8	0.04	34.38
1 primi 2	H
104	1	08/29/2020	114	915	1	9.9	0.23	34.52
1 primi 2	H
104	1	08/30/2020	115	916	1	9.9	0.14	33.56
1 primi 2	H
104	1	08/31/2020	116	917	1	9.6	0.04	32.71
1 primi 2	H
104	1	09/01/2020	117	918	1	9.9	0.03	32.21
1 primi 2	H
104	1	09/02/2020	118	919	1	10.4	0	37.69

1 primi	2	H						
104	1	09/03/2020	119	920	1	10	0	38.21
1 primi	2	H						
104	1	09/04/2020	120	921	1	10.2	0	40.47
1 primi	2	H						
104	1	09/05/2020	121	922	1	9.9	0.03	39.91
1 primi	2	H						
104	1	09/06/2020	122	923	1	9.6	0.11	36.22
1 primi	2	H						
104	1	09/07/2020	123	924	1	9.5	0.13	37.87
1 primi	2	H						
104	1	09/08/2020	124	925	1	9.8	0.14	41.22
1 primi	2	H						
104	1	09/09/2020	125	926	1	9.6	0.16	37.52
1 primi	2	H						
104	1	09/10/2020	126	927	1	9.6	0.04	37.08
1 primi	2	H						
104	1	09/11/2020	127	928	1	9.6	0.12	45.72
1 primi	2	H						
104	1	09/12/2020	128	929	1	9.8	0.04	37.37
1 primi	2	H						
104	1	09/13/2020	129	930	1	9.8	0.15	41.01
1 primi	2	H						
104	1	09/14/2020	130	931	1	9.6	0	38.56
1 primi	2	H						
104	1	09/15/2020	131	932	1	9.5	0	32.74
1 primi	2	H						
104	1	09/16/2020	132	933	1	9.8	0.12	36.08
1 primi	2	H						
104	1	09/17/2020	133	934	1	9.6	0.05	35.44
1 primi	2	H						
105	1	11/01/2020	7	775	1	9.1	0.28	26.54
1 primi	1	H						
105	1	11/02/2020	8	776	1	8.9	0.24	28.7
1 primi	1	H						
105	1	11/03/2020	9	777	1	9.2	0.07	27.69
1 primi	1	H						
105	1	11/04/2020	10	778	1	9.3	0.06	26.84
1 primi	1	H						
105	1	11/05/2020	11	779	1	9.2	0.28	27.72
1 primi	1	H						
105	1	11/06/2020	12	780	1	8.9	0.34	29.21
1 primi	1	H						
105	1	11/07/2020	13	781	1	8.7	0.17	29.48
1 primi	1	H						
105	1	11/08/2020	14	782	1	9.1	0.02	30.45
1 primi	1	H						
105	1	11/09/2020	15	783	1	9.3	0.11	31.41
1 primi	1	H						
105	1	11/10/2020	16	784	1	9.2	0.31	30.92
1 primi	1	H						
105	1	11/11/2020	17	785	1	9.3	0.06	30.85
1 primi	1	H						
105	1	11/12/2020	18	786	1	8.4	0.11	31.54

1 primi	1	H						
105	1	11/13/2020	19	787	1	9.1	0.17	34.01
1 primi	1	H						
105	1	11/14/2020	20	788	1	9.3	0.19	34.72
1 primi	1	H						
105	1	11/15/2020	21	789	1	9.5	0.28	34.98
1 primi	1	H						
105	1	11/16/2020	22	790	1	0	0.19	36.81
1 primi	2	H						
105	1	11/17/2020	23	791	1	10.3	0.12	32.5
1 primi	2	H						
105	1	11/18/2020	24	792	1	10.2	0.05	32.27
1 primi	2	H						
105	1	11/19/2020	25	793	1	9.2	0.17	33.65
1 primi	2	H						
105	1	11/20/2020	26	794	1	8.9	0.21	36.1
1 primi	2	H						
105	1	11/21/2020	27	795	1	8.8	0.19	36.32
1 primi	2	H						
105	1	11/22/2020	28	796	1	9.3	0.04	35.77
1 primi	2	H						
105	1	11/23/2020	29	797	1	8.7	0.06	35.83
1 primi	2	H						
105	1	11/24/2020	30	798	1	9.2	0.13	33.68
1 primi	2	H						
105	1	11/25/2020	31	799	1	9.2	0.1	35.29
1 primi	2	H						
105	1	11/26/2020	32	800	1	8.4	0.25	32.86
1 primi	2	H						
105	1	11/27/2020	33	801	1	8.7	0.05	36.86
1 primi	2	H						
105	1	11/28/2020	34	802	1	9.1	0.13	37.07
1 primi	2	H						
105	1	11/29/2020	35	803	1	8.7	0.28	35.26
1 primi	2	H						
105	1	11/30/2020	36	804	1	8.6	0.21	34.27
1 primi	2	H						
105	1	12/01/2020	37	805	1	8.8	0.25	34.88
1 primi	2	H						
105	1	12/02/2020	38	806	1	0	0.03	35.24
1 primi	2	H						
105	1	12/03/2020	39	807	1	8.7	0.05	35.51
1 primi	2	H						
105	1	12/04/2020	40	808	1	8.9	0.14	32.46
1 primi	2	H						
105	1	12/05/2020	41	809	1	9.2	0.12	38.46
1 primi	2	H						
105	1	12/06/2020	42	810	1	8.8	0.12	37.58
1 primi	2	H						
106	1	11/26/2019	9	851	1	8.6	0.23	26.26
1 primi	1	H						
106	1	11/27/2019	10	852	1	8.8	0.05	26.02
1 primi	1	H						
106	1	11/28/2019	11	853	1	8.8	0	26.17

1 primi	1	H						
106	1	11/29/2019	12	854	1	8.7	0.13	26.73
1 primi	1	H						
106	1	11/30/2019	13	855	1	8.6	0.12	26.18
1 primi	1	H						
106	1	12/01/2019	14	856	1	8.6	0	26.76
1 primi	1	H						
106	1	12/02/2019	15	857	1	8.5	0.03	27.47
1 primi	1	H						
106	1	12/03/2019	16	858	1	8.8	0.11	27.1
1 primi	1	H						
106	1	12/04/2019	17	859	1	8.9	0.28	28.3
1 primi	1	H						
106	1	12/05/2019	18	860	1	9.1	0.02	29.1
1 primi	1	H						
106	1	12/06/2019	19	861	1	8.9	0.17	29.8
1 primi	1	H						
106	1	12/07/2019	20	862	1	9.1	0.03	29.33
1 primi	1	H						
106	1	12/08/2019	21	863	1	8.8	0	27.97
1 primi	1	H						
106	1	12/09/2019	22	864	1	9.2	0	31.52
1 primi	2	H						
106	1	12/10/2019	23	865	1	8.8	0.13	30.39
1 primi	2	H						
106	1	12/11/2019	24	866	1	8.7	0.03	30.41
1 primi	2	H						
106	1	12/12/2019	25	867	1	8.9	0.33	30.43
1 primi	2	H						
106	1	12/13/2019	26	868	1	8.9	0.06	31.84
1 primi	2	H						
106	1	12/14/2019	27	869	1	8.6	0.03	29.91
1 primi	2	H						
106	1	12/15/2019	28	870	1	8.7	0.01	31.05
1 primi	2	H						
106	1	12/16/2019	29	871	1	8.8	0	32.71
1 primi	2	H						
106	1	12/17/2019	30	872	1	8.8	0	31.23
1 primi	2	H						
106	1	12/18/2019	31	873	1	8.8	0	32.41
1 primi	2	H						
106	1	12/19/2019	32	874	1	8.8	0.01	29.52
1 primi	2	H						
106	1	12/20/2019	33	875	1	8.8	0.02	31.36
1 primi	2	H						
106	1	12/21/2019	34	876	1	8.9	0	31.45
1 primi	2	H						
106	1	12/22/2019	35	877	1	9.1	0	32.18
1 primi	2	H						
106	1	12/23/2019	36	878	1	9.2	0.02	31.59
1 primi	2	H						
106	1	12/24/2019	37	879	1	8.8	0.02	31.58
1 primi	2	H						
106	1	12/25/2019	38	880	1	9.2	0	31.68

1 primi 2 H
106 1 12/26/2019 39 881 1 8.6 0.1 30.6
1 primi 2 H
106 1 12/27/2019 40 882 1 8.6 0.11 30
1 primi 2 H
106 1 12/28/2019 41 883 1 8.6 0.12 31.29
1 primi 2 H
106 1 12/29/2019 42 884 1 8.9 0 32.2
1 primi 2 H
106 1 12/30/2019 43 885 1 8.8 0.03 32.12
1 primi 2 H
106 1 12/31/2019 44 886 1 9.2 0 30.74
1 primi 2 H
106 1 01/01/2020 45 887 1 8.8 0 30.37
1 primi 2 H
106 1 01/02/2020 46 888 1 8.7 0.01 31.23
1 primi 2 H
106 1 01/03/2020 47 889 1 8.4 0.16 30.2
1 primi 2 H
106 1 01/04/2020 48 890 1 8.7 0 29.05
1 primi 2 H
106 1 01/05/2020 49 891 1 8.5 0.08 30.11
1 primi 2 H
106 1 01/06/2020 50 892 1 8.9 0 31.28
1 primi 2 H
106 1 01/07/2020 51 893 1 8.9 0.08 30.19
1 primi 2 H
106 1 01/08/2020 52 894 1 9.1 0 30.39
1 primi 2 H
106 1 01/09/2020 53 895 1 9.1 0.01 31.1
1 primi 2 H
106 1 01/10/2020 54 896 1 9.9 0.12 27.78
1 primi 2 H
106 1 11/16/2020 6 1207 2 9.6 0.16 26.95
2 multi 1 H
106 1 11/17/2020 7 1208 2 9.8 0.23 28.88
2 multi 1 H
106 1 11/18/2020 8 1209 2 10.2 0.02 28.23
2 multi 1 H
106 1 11/19/2020 9 1210 2 9.9 0.22 29.35
2 multi 1 H
106 1 11/20/2020 10 1211 2 9.5 0.08 30.17
2 multi 1 H
106 1 11/21/2020 11 1212 2 8.9 0.34 29.43
2 multi 1 H
106 1 11/22/2020 12 1213 2 9.2 0.17 30.43
2 multi 1 H
106 1 11/23/2020 13 1214 2 9.6 0.13 31.47
2 multi 1 H
106 1 11/24/2020 14 1215 2 9.8 0.08 31.42
2 multi 1 H
106 1 11/25/2020 15 1216 2 9.6 0.07 30.58
2 multi 1 H
106 1 11/26/2020 16 1217 2 9.6 0.04 30.8

```
2 multi 1       H
106     1       11/27/2020      17      1218    2       9.5     0.05    33.69
2 multi 1       H
106     1       11/28/2020      18      1219    2       9.5     0.31    31.25
2 multi 1       H
106     1       11/29/2020      19      1220    2       9.5     0.05    32.51
2 multi 1       H
106     1       11/30/2020      20      1221    2       9.9     0.08    31.68
2 multi 1       H
106     1       12/01/2020      21      1222    2       9.8     0.12    33.4
2 multi 1       H
106     1       12/02/2020      22      1223    2       9.8     0.07    33.2
2 multi 2       H
106     1       12/03/2020      23      1224    2       9.8     0.04    34.24
2 multi 2       H
106     1       12/04/2020      24      1225    2       9.8     0.06    33.15
2 multi 2       H
106     1       12/05/2020      25      1226    2       9.9     0.08    33.5
2 multi 2       H
106     1       12/06/2020      26      1227    2       9.8     0.01    35.1
2 multi 2       H
106     1       12/07/2020      27      1228    2       9.8     0.14    34.12
2 multi 2       H
106     1       12/08/2020      28      1229    2       9.9     0.86    34.82
2 multi 2       H
106     1       12/09/2020      29      1230    2       0       0       33.87
2 multi 2       H
106     1       12/10/2020      30      1231    2       10.2    0.14    34.24
2 multi 2       H
106     1       12/11/2020      31      1232    2       10.2    0.14    34.64
2 multi 2       H
106     1       12/12/2020      32      1233    2       10      0.07    33.7
2 multi 2       H
106     1       12/13/2020      33      1234    2       10      0.02    35.71
2 multi 2       H
106     1       12/14/2020      34      1235    2       10.6    0       34.58
2 multi 2       H
106     1       12/15/2020      35      1236    2       9.9     0       35.1
2 multi 2       H
106     1       12/16/2020      36      1237    2       10.2    0       35.36
2 multi 2       H
106     1       12/17/2020      37      1238    2       10.2    0       33.37
2 multi 2       H
106     1       12/18/2020      38      1239    2       10      0.06    34.26
2 multi 2       H
106     1       12/19/2020      39      1240    2       10.7    0.05    32.4
2 multi 2       H
106     1       12/20/2020      40      1241    2       10.7    0.13    33.67
2 multi 2       H
106     1       12/21/2020      41      1242    2       11.1    0.08    34.66
2 multi 2       H
106     1       12/22/2020      42      1243    2       10.7    0.06    33.81
2 multi 2       H
106     1       12/23/2020      43      1244    2       10.6    0.08    34.85
```

2 multi 2		H						
106	1	12/24/2020	44	1245	2	10.3	0.04	34.97
2 multi 2		H						
106	1	12/25/2020	45	1246	2	10.6	0.05	36.46
2 multi 2		H						
106	1	12/26/2020	46	1247	2	10.4	0.13	35.58
2 multi 2		H						
107	1	05/07/2019	4	756	1	9.5	0.28	19.83
1 primi 1		H						
107	1	05/08/2019	5	757	1	9.8	0	22.89
1 primi 1		H						
107	1	05/09/2019	6	758	1	9.5	0	23.18
1 primi 1		H						
107	1	05/10/2019	7	759	1	9.6	0.1	24.52
1 primi 1		H						
107	1	05/11/2019	8	760	1	9.5	0	24.32
1 primi 1		H						
107	1	05/12/2019	9	761	1	9.3	0	26.11
1 primi 1		H						
107	1	05/13/2019	10	762	1	9.1	0	25.75
1 primi 1		H						
107	1	05/14/2019	11	763	1	9.2	0.1	27.84
1 primi 1		H						
107	1	05/15/2019	12	764	1	9.2	0	28.2
1 primi 1		H						
107	1	05/16/2019	13	765	1	9.3	0	26.79
1 primi 1		H						
107	1	05/17/2019	14	766	1	9.6	0	27.05
1 primi 1		H						
107	1	05/18/2019	15	767	1	8.7	0	29.39
1 primi 1		H						
107	1	05/19/2019	16	768	1	8.8	0.15	26.94
1 primi 1		H						
107	1	05/20/2019	17	769	1	9.2	0	27.5
1 primi 1		H						
107	1	05/21/2019	18	770	1	9.2	0	31.11
1 primi 1		H						
107	1	05/22/2019	19	771	1	9.2	0	29.16
1 primi 1		H						
107	1	05/23/2019	20	772	1	9.1	0	29.43
1 primi 1		H						
107	1	05/24/2019	21	773	1	9.2	0	30.07
1 primi 1		H						
107	1	05/25/2019	22	774	1	9.1	0	30.61
1 primi 2		H						
107	1	05/26/2019	23	775	1	9.5	0	26.64
1 primi 2		H						
107	1	05/27/2019	24	776	1	9.2	0	25.55
1 primi 2		H						
107	1	05/28/2019	25	777	1	9.1	0	23.28
1 primi 2		H						
107	1	05/29/2019	26	778	1	8.9	0.02	23.96
1 primi 2		H						
107	1	05/30/2019	27	779	1	9.3	0.03	27.44

1 primi	2	H						
107	1	05/31/2019	28	780	1	9.5	0.02	26.97
1 primi	2	H						
107	1	06/01/2019	29	781	1	9.6	0.03	28.9
1 primi	2	H						
107	1	06/02/2019	30	782	1	9.2	0.33	26.23
1 primi	2	H						
107	1	06/03/2019	31	783	1	9.6	0.02	29.33
1 primi	2	H						
107	1	06/04/2019	32	784	1	9.5	0.01	27.6
1 primi	2	H						
107	1	06/05/2019	33	785	1	9.3	0	29.13
1 primi	2	H						
107	1	06/06/2019	34	786	1	9.5	0	29.41
1 primi	2	H						
107	1	06/07/2019	35	787	1	9.5	0	29.5
1 primi	2	H						
107	1	06/08/2019	36	788	1	9.5	0.01	29.24
1 primi	2	H						
107	1	06/09/2019	37	789	1	9.6	0	30.96
1 primi	2	H						
107	1	06/10/2019	38	790	1	9.3	0.01	31.34
1 primi	2	H						
107	1	06/11/2019	39	791	1	9.6	0	32.01
1 primi	2	H						
107	1	06/12/2019	40	792	1	9.3	0	31.28
1 primi	2	H						
107	1	06/13/2019	41	793	1	9.1	0.06	30.9
1 primi	2	H						
107	1	06/14/2019	42	794	1	8.9	0	32.01
1 primi	2	H						
107	1	06/15/2019	43	795	1	9.5	0	30.28
1 primi	2	H						
107	1	06/16/2019	44	796	1	9.3	0	29
1 primi	2	H						
107	1	06/17/2019	45	797	1	9.2	0	30.73
1 primi	2	H						
107	1	06/18/2019	46	798	1	8.6	0.18	28.45
1 primi	2	H						
107	1	06/19/2019	47	799	1	8.8	0	29.72
1 primi	2	H						
107	1	06/20/2019	48	800	1	9.1	0	30.27
1 primi	2	H						
107	1	06/21/2019	49	801	1	8.9	0.11	30.14
1 primi	2	H						
107	1	06/22/2019	50	802	1	9.2	0	29.95
1 primi	2	H						
107	1	06/23/2019	51	803	1	9.2	0	31.17
1 primi	2	H						
107	1	06/24/2019	52	804	1	9.1	0	32.03
1 primi	2	H						
107	1	06/25/2019	53	805	1	9.1	0	33.1
1 primi	2	H						
107	1	06/26/2019	54	806	1	9.5	0	32.62

1 primi 2		H						
107	1	06/27/2019	55	807	1	9.2	0	33.56
1 primi 2		H						
107	1	06/28/2019	56	808	1	9.1	0	31.25
1 primi 2		H						
107	1	06/29/2019	57	809	1	8.9	0	31.25
1 primi 2		H						
107	1	06/30/2019	58	810	1	9.2	0	31.17
1 primi 2		H						
107	1	07/01/2019	59	811	1	9.2	0	31.37
1 primi 2		H						
107	1	07/02/2019	60	812	1	9.5	0.26	32.87
1 primi 2		H						
107	1	07/03/2019	61	813	1	9.6	0	32.84
1 primi 2		H						
107	1	07/04/2019	62	814	1	9.2	0	30.3
1 primi 2		H						
107	1	07/05/2019	63	815	1	9.2	0	29.79
1 primi 2		H						
107	1	07/06/2019	64	816	1	9.2	0.11	30.8
1 primi 2		H						
107	1	07/07/2019	65	817	1	9.3	0	31.59
1 primi 2		H						
107	1	07/08/2019	66	818	1	9.1	0	31.01
1 primi 2		H						
107	1	07/09/2019	67	819	1	8.9	0.12	29.71
1 primi 2		H						
107	1	07/10/2019	68	820	1	8.7	0	30.32
1 primi 2		H						
107	1	07/11/2019	69	821	1	8.6	0.14	29.2
1 primi 2		H						
107	1	07/12/2019	70	822	1	8.8	0	29.52
1 primi 2		H						
107	1	07/13/2019	71	823	1	8.9	0	29.23
1 primi 2		H						
107	1	07/14/2019	72	824	1	9.3	0	30.89
1 primi 2		H						
107	1	07/15/2019	73	825	1	9.1	0	31.6
1 primi 2		H						
107	1	07/16/2019	74	826	1	8.9	0.27	30.03
1 primi 2		H						
107	1	07/17/2019	75	827	1	9.1	0.05	29.4
1 primi 2		H						
107	1	07/18/2019	76	828	1	9.2	0.21	31.01
1 primi 2		H						
107	1	07/19/2019	77	829	1	8.8	0	30.07
1 primi 2		H						
107	1	07/20/2019	78	830	1	9.2	0	30.13
1 primi 2		H						
107	1	07/21/2019	79	831	1	9.3	0	30.68
1 primi 2		H						
107	1	07/22/2019	80	832	1	9.3	0.01	29.09
1 primi 2		H						
107	1	07/23/2019	81	833	1	9.5	0	30.49

1 primi 2		H						
107	1	07/24/2019	82	834	1	9.9	0.31	32.02
1 primi 2		H						
107	1	07/25/2019	83	835	1	9.5	0	30.18
1 primi 2		H						
107	1	07/26/2019	84	836	1	9.6	0	30.21
1 primi 2		H						
107	1	07/27/2019	85	837	1	9.2	0.02	29.08
1 primi 2		H						
107	1	07/28/2019	86	838	1	9.2	0	28.69
1 primi 2		H						
107	1	07/29/2019	87	839	1	9.3	0	30.05
1 primi 2		H						
107	1	07/30/2019	88	840	1	9.2	0	28.17
1 primi 2		H						
107	1	07/31/2019	89	841	1	9.6	0	29.56
1 primi 2		H						
107	1	05/29/2020	4	1144	2	9.9	0.26	31.15
2 multi 1		H						
107	1	05/30/2020	5	1145	2	9.9	0.23	33.36
2 multi 1		H						
107	1	05/31/2020	6	1146	2	10	0.03	39.27
2 multi 1		H						
107	1	06/01/2020	7	1147	2	10.2	0.03	38.99
2 multi 1		H						
107	1	06/02/2020	8	1148	2	10	0.04	39.81
2 multi 1		H						
107	1	06/03/2020	9	1149	2	10.3	0.03	37.71
2 multi 1		H						
107	1	06/04/2020	10	1150	2	10	0.28	38.77
2 multi 1		H						
107	1	06/05/2020	11	1151	2	10	0.26	38.53
2 multi 1		H						
107	1	06/06/2020	12	1152	2	9.8	0	39.23
2 multi 1		H						
107	1	06/07/2020	13	1153	2	10	0	41.05
2 multi 1		H						
107	1	06/08/2020	14	1154	2	10.2	0.02	41.84
2 multi 1		H						
107	1	06/09/2020	15	1155	2	9.9	0.17	40.05
2 multi 1		H						
107	1	06/10/2020	16	1156	2	9.8	0.27	41.65
2 multi 1		H						
107	1	06/11/2020	17	1157	2	9.8	0.02	42.64
2 multi 1		H						
107	1	06/12/2020	18	1158	2	9.6	0.3	41.71
2 multi 1		H						
107	1	06/13/2020	19	1159	2	9.9	0.02	44.37
2 multi 1		H						
107	1	06/14/2020	20	1160	2	9.9	0	42.04
2 multi 1		H						
107	1	06/15/2020	21	1161	2	9.8	0.1	41.58
2 multi 1		H						
107	1	06/16/2020	22	1162	2	9.8	0	40.3

2 multi	2	H						
107	1	06/17/2020	23	1163	2	9.9	0.13	42.24
2 multi	2	H						
107	1	06/18/2020	24	1164	2	9.8	0.2	42.78
2 multi	2	H						
107	1	06/19/2020	25	1165	2	9.8	0.17	38.24
2 multi	2	H						
107	1	06/20/2020	26	1166	2	10.2	0	42.32
2 multi	2	H						
107	1	06/21/2020	27	1167	2	9.9	0.48	43.22
2 multi	2	H						
107	1	06/22/2020	28	1168	2	10.2	0.02	42.11
2 multi	2	H						
107	1	06/23/2020	29	1169	2	10	0.32	42.1
2 multi	2	H						
107	1	06/24/2020	30	1170	2	9.8	0.19	43.27
2 multi	2	H						
107	1	06/25/2020	31	1171	2	10.2	0	42.62
2 multi	2	H						
107	1	06/26/2020	32	1172	2	10.2	0	42.92
2 multi	2	H						
107	1	06/27/2020	33	1173	2	10.2	0	42.43
2 multi	2	H						
107	1	06/28/2020	34	1174	2	10.2	0	43.22
2 multi	2	H						
107	1	06/29/2020	35	1175	2	10	0	40.33
2 multi	2	H						
109	1	03/30/2019	8	763	1	9.8	0.02	21.88
1 primi	1	H						
109	1	03/31/2019	9	764	1	10.2	0.01	23.07
1 primi	1	H						
109	1	04/01/2019	10	765	1	10.2	0	23.58
1 primi	1	H						
109	1	04/02/2019	11	766	1	9.6	0.01	24.76
1 primi	1	H						
109	1	04/03/2019	12	767	1	9.6	0.04	22.81
1 primi	1	H						
109	1	04/04/2019	13	768	1	9.8	0.02	22.65
1 primi	1	H						
109	1	04/05/2019	14	769	1	9.5	0.02	22.23
1 primi	1	H						
109	1	04/06/2019	15	770	1	9.5	0.26	22.16
1 primi	1	H						
109	1	04/07/2019	16	771	1	9.9	0.46	24.53
1 primi	1	H						
109	1	04/08/2019	17	772	1	9.8	0.09	25.46
1 primi	1	H						
109	1	04/09/2019	18	773	1	9.6	0	26.25
1 primi	1	H						
109	1	04/10/2019	19	774	1	9.5	0	26.44
1 primi	1	H						
109	1	04/11/2019	20	775	1	9.6	0	25.61
1 primi	1	H						
109	1	04/12/2019	21	776	1	9.3	0	26.36

1 primi 1		H						
109	1	04/13/2019	22	777	1	9.5	0	27.7
1 primi 2		H						
109	1	04/14/2019	23	778	1	9.6	0	27.88
1 primi 2		H						
109	1	04/15/2019	24	779	1	9.5	0	27.05
1 primi 2		H						
109	1	04/16/2019	25	780	1	9.3	0	28.25
1 primi 2		H						
109	1	04/17/2019	26	781	1	9.6	0	29.79
1 primi 2		H						
109	1	04/18/2019	27	782	1	9.5	0	29.42
1 primi 2		H						
109	1	04/19/2019	28	783	1	9.3	0	28.31
1 primi 2		H						
109	1	04/20/2019	29	784	1	9.1	0.02	29.01
1 primi 2		H						
109	1	04/21/2019	30	785	1	9.1	0.3	29.87
1 primi 2		H						
109	1	04/22/2019	31	786	1	9.6	0	31.4
1 primi 2		H						
109	1	04/23/2019	32	787	1	9.2	0.01	29.69
1 primi 2		H						
109	1	04/24/2019	33	788	1	9.2	0	29.78
1 primi 2		H						
109	1	04/25/2019	34	789	1	9.2	0	28.91
1 primi 2		H						
109	1	04/26/2019	35	790	1	9.3	0.12	29.72
1 primi 2		H						
109	1	04/27/2019	36	791	1	9.2	0.15	29.13
1 primi 2		H						
109	1	04/28/2019	37	792	1	8.9	0.14	31.02
1 primi 2		H						
109	1	04/29/2019	38	793	1	9.5	0	31.06
1 primi 2		H						
109	1	04/30/2019	39	794	1	9.3	0.39	32.11
1 primi 2		H						
109	1	05/01/2019	40	795	1	9.2	0.19	31.77
1 primi 2		H						
109	1	05/02/2019	41	796	1	9.3	0	32.99
1 primi 2		H						
109	1	05/03/2019	42	797	1	9.3	0.1	31.57
1 primi 2		H						
109	1	05/04/2019	43	798	1	9.5	0.14	28.83
1 primi 2		H						
109	1	05/05/2019	44	799	1	9.3	0.07	31.2
1 primi 2		H						
109	1	05/06/2019	45	800	1	11.7	0	29.61
1 primi 2		H						
109	1	05/07/2019	46	801	1	9.2	0	33.69
1 primi 2		H						
109	1	05/08/2019	47	802	1	9.3	0	32.93
1 primi 2		H						
109	1	05/09/2019	48	803	1	9.2	0	33.07

1 primi 2 H
109 1 05/10/2019 49 804 1 9.5 0 34.32
1 primi 2 H
109 1 05/11/2019 50 805 1 9.2 0 33.93
1 primi 2 H
109 1 05/12/2019 51 806 1 9.3 0.22 33.15
1 primi 2 H
109 1 05/13/2019 52 807 1 9.3 0 33
1 primi 2 H
109 1 05/14/2019 53 808 1 8.9 0 30.5
1 primi 2 H
109 1 05/15/2019 54 809 1 8.9 0 31.68
1 primi 2 H
109 1 05/16/2019 55 810 1 9.3 0 31.69
1 primi 2 H
109 1 05/17/2019 56 811 1 8.9 0.06 31.33
1 primi 2 H
109 1 05/18/2019 57 812 1 9.2 0.04 37.79
1 primi 2 H
109 1 05/19/2019 58 813 1 9.3 0 40.11
1 primi 2 H
109 1 05/20/2019 59 814 1 9.2 0.03 34.19
1 primi 2 H
109 1 05/21/2019 60 815 1 9.3 0 34.98
1 primi 2 H
109 1 05/22/2019 61 816 1 9.2 0 36.98
1 primi 2 H
109 1 05/23/2019 62 817 1 9.2 0.51 33.11
1 primi 2 H
109 1 05/24/2019 63 818 1 9.5 0.12 35.13
1 primi 2 H
109 1 05/25/2019 64 819 1 9.6 0.26 36.86
1 primi 2 H
109 1 05/26/2019 65 820 1 9.2 0.23 33.14
1 primi 2 H
109 1 05/27/2019 66 821 1 9.6 0.1 36.35
1 primi 2 H
109 1 05/28/2019 67 822 1 9.3 0.11 34.79
1 primi 2 H
109 1 05/29/2019 68 823 1 9.3 0 36.05
1 primi 2 H
109 1 05/30/2019 69 824 1 9.2 0.21 34.55
1 primi 2 H
109 1 05/31/2019 70 825 1 9.2 0 35.23
1 primi 2 H
109 1 06/01/2019 71 826 1 9.6 0.56 32.68
1 primi 2 H
109 1 06/02/2019 72 827 1 8.9 0.25 32.32
1 primi 2 H
109 1 06/03/2019 73 828 1 9.6 0 33.71
1 primi 2 H
109 1 06/04/2019 74 829 1 9.2 0 34.3
1 primi 2 H
109 1 06/05/2019 75 830 1 9.6 0 34.03

1 primi 2 H
109 1 06/06/2019 76 831 1 9.9 0.01 34.48
1 primi 2 H
109 1 06/07/2019 77 832 1 10.7 0.02 30.46
1 primi 2 H
109 1 06/08/2019 78 833 1 10.6 0 30.97
1 primi 2 H
109 1 06/09/2019 79 834 1 10.3 0 29.7
1 primi 2 H
109 1 06/10/2019 80 835 1 10.6 0.03 31.39
1 primi 2 H
109 1 06/11/2019 81 836 1 10.6 0.14 31.7
1 primi 2 H
109 1 06/12/2019 82 837 1 10.4 0 30.16
1 primi 2 H
109 1 06/13/2019 83 838 1 10 0 30.92
1 primi 2 H
109 1 06/14/2019 84 839 1 10.4 0.02 32.37
1 primi 2 H
109 1 06/15/2019 85 840 1 10 0 31.44
1 primi 2 H
109 1 06/16/2019 86 841 1 10.2 0.28 30.06
1 primi 2 H
109 1 06/17/2019 87 842 1 9.9 0 32.21
1 primi 2 H
109 1 06/18/2019 88 843 1 9.8 0 32.37
1 primi 2 H
109 1 06/19/2019 89 844 1 9.8 0 31.35
1 primi 2 H
109 1 06/20/2019 90 845 1 9.6 0 30.82
1 primi 2 H
109 1 06/21/2019 91 846 1 10 0.43 31.22
1 primi 2 H
109 1 06/22/2019 92 847 1 9.8 0 29
1 primi 2 H
109 1 06/23/2019 93 848 1 9.8 0 28.51
1 primi 2 H
109 1 06/24/2019 94 849 1 9.9 0.34 28.68
1 primi 2 H
109 1 06/25/2019 95 850 1 10 0.45 32.53
1 primi 2 H
109 1 06/26/2019 96 851 1 9.9 0.21 33.28
1 primi 2 H
109 1 06/27/2019 97 852 1 9.9 0.08 32.78
1 primi 2 H
109 1 06/28/2019 98 853 1 9.9 0 31.29
1 primi 2 H
109 1 06/29/2019 99 854 1 10.4 0 33.19
1 primi 2 H
109 1 06/30/2019 100 855 1 9.8 0 30.04
1 primi 2 H
109 1 07/01/2019 101 856 1 10.2 0.05 32.74
1 primi 2 H
109 1 07/02/2019 102 857 1 10 0 33.56

1 primi	2	H						
109	1	07/03/2019	103	858	1	10	0	33.33
1 primi	2	H						
109	1	07/04/2019	104	859	1	9.9	0	34.51
1 primi	2	H						
109	1	07/05/2019	105	860	1	9.8	0.25	34.21
1 primi	2	H						
109	1	07/06/2019	106	861	1	9.8	0	34.16
1 primi	2	H						
109	1	07/07/2019	107	862	1	9.9	0	33
1 primi	2	H						
109	1	07/08/2019	108	863	1	10	0.11	32.75
1 primi	2	H						
109	1	07/09/2019	109	864	1	9.9	0	31.8
1 primi	2	H						
109	1	07/10/2019	110	865	1	9.6	0	33.09
1 primi	2	H						
109	1	07/11/2019	111	866	1	9.5	0.07	32.14
1 primi	2	H						
109	1	07/12/2019	112	867	1	9.9	0.1	32.6
1 primi	2	H						
109	1	07/13/2019	113	868	1	9.3	0.04	33.17
1 primi	2	H						
109	1	07/14/2019	114	869	1	9.9	0	33.06
1 primi	2	H						
109	1	07/15/2019	115	870	1	9.8	0.23	34.32
1 primi	2	H						
109	1	07/16/2019	116	871	1	9.9	0.01	32.66
1 primi	2	H						
109	1	07/17/2019	117	872	1	10.2	0	35.16
1 primi	2	H						
109	1	02/25/2020	4	1095	2	9.5	0.3	28.81
2 multi	1	H						
109	1	02/26/2020	5	1096	2	9.5	0.26	30.89
2 multi	1	H						
109	1	02/27/2020	6	1097	2	10	0.05	32.4
2 multi	1	H						
109	1	02/28/2020	7	1098	2	9.5	0.03	33.01
2 multi	1	H						
109	1	02/29/2020	8	1099	2	9.9	0	36.1
2 multi	1	H						
109	1	03/01/2020	9	1100	2	9.5	0.02	32.57
2 multi	1	H						
109	1	03/02/2020	10	1101	2	9.6	0.01	38.9
2 multi	1	H						
109	1	03/03/2020	11	1102	2	9.8	0.02	37.3
2 multi	1	H						
109	1	03/04/2020	12	1103	2	9.1	0	38.06
2 multi	1	H						
109	1	03/05/2020	13	1104	2	9.3	0.1	39.32
2 multi	1	H						
109	1	03/06/2020	14	1105	2	9.2	0.15	38.52
2 multi	1	H						
109	1	03/07/2020	15	1106	2	9.5	0.66	41.25

2 multi	1	H						
109	1	03/08/2020	16	1107	2	9.3	0	38.87
2 multi	1	H						
109	1	03/09/2020	17	1108	2	9.6	0.01	42.27
2 multi	1	H						
109	1	03/10/2020	18	1109	2	9.3	0.01	41.99
2 multi	1	H						
109	1	03/11/2020	19	1110	2	9.5	0	42.36
2 multi	1	H						
109	1	03/12/2020	20	1111	2	9.5	0.62	44.6
2 multi	1	H						
109	1	03/13/2020	21	1112	2	9.5	0.3	42.91
2 multi	1	H						
109	1	03/14/2020	22	1113	2	9.3	0.21	44.26
2 multi	2	H						
109	1	03/15/2020	23	1114	2	9.8	0	44.63
2 multi	2	H						
109	1	03/16/2020	24	1115	2	9.3	0.28	42.17
2 multi	2	H						
109	1	03/17/2020	25	1116	2	9.5	0.08	44.92
2 multi	2	H						
109	1	03/18/2020	26	1117	2	9.5	0.62	45.3
2 multi	2	H						
109	1	03/19/2020	27	1118	2	9.3	0.01	41.02
2 multi	2	H						
109	1	03/20/2020	28	1119	2	9.3	0	30.53
2 multi	2	H						
109	1	03/21/2020	29	1120	2	9.3	0.04	44.55
2 multi	2	H						
109	1	03/22/2020	30	1121	2	9.3	0.02	41.52
2 multi	2	H						
109	1	03/23/2020	31	1122	2	9.6	0.69	42.04
2 multi	2	H						
109	1	03/24/2020	32	1123	2	9.6	0.19	45.15
2 multi	2	H						
109	1	03/25/2020	33	1124	2	9.6	0.2	43.93
2 multi	2	H						
109	1	03/26/2020	34	1125	2	9.6	0	44.09
2 multi	2	H						
109	1	03/27/2020	35	1126	2	9.8	0	45.72
2 multi	2	H						
109	1	03/28/2020	36	1127	2	9.6	0.19	41.99
2 multi	2	H						
109	1	03/29/2020	37	1128	2	9.5	0	45.24
2 multi	2	H						
109	1	03/30/2020	38	1129	2	9.2	0.08	44.22
2 multi	2	H						
109	1	03/31/2020	39	1130	2	9.3	0.05	42.29
2 multi	2	H						
109	1	04/01/2020	40	1131	2	9.2	0	45.78
2 multi	2	H						
109	1	04/02/2020	41	1132	2	9.3	0	42.83
2 multi	2	H						
109	1	04/03/2020	42	1133	2	9.8	0.18	43.6

2 multi	2	H						
109	1	04/04/2020	43	1134	2	9.5	0.25	43.36
2 multi	2	H						
109	1	04/05/2020	44	1135	2	9.6	0.03	40.81
2 multi	2	H						
109	1	04/06/2020	45	1136	2	9.5	0.12	43.47
2 multi	2	H						
109	1	04/07/2020	46	1137	2	9.3	0.51	42.67
2 multi	2	H						
109	1	04/08/2020	47	1138	2	9.8	0.33	46.37
2 multi	2	H						
109	1	04/09/2020	48	1139	2	9.8	0.24	43.37
2 multi	2	H						
109	1	04/10/2020	49	1140	2	9.6	0.28	44.94
2 multi	2	H						
109	1	04/11/2020	50	1141	2	9.5	0.26	43.04
2 multi	2	H						
109	1	04/12/2020	51	1142	2	9.9	0.25	42.38
2 multi	2	H						
109	1	04/13/2020	52	1143	2	9.6	0.18	38.8
2 multi	2	H						
109	1	04/14/2020	53	1144	2	9.5	0.04	43.26
2 multi	2	H						
109	1	04/15/2020	54	1145	2	9.8	0.12	38.79
2 multi	2	H						
109	1	04/16/2020	55	1146	2	9.8	0.55	42.98
2 multi	2	H						
109	1	04/17/2020	56	1147	2	9.3	0	40.78
2 multi	2	H						
109	1	04/18/2020	57	1148	2	9.8	0.51	39.3
2 multi	2	H						
109	1	04/19/2020	58	1149	2	9.9	0.06	39.66
2 multi	2	H						
109	1	04/20/2020	59	1150	2	9.2	0.31	38.56
2 multi	2	H						
109	1	04/21/2020	60	1151	2	9.2	0.57	39.1
2 multi	2	H						
109	1	04/22/2020	61	1152	2	9.5	0	42.68
2 multi	2	H						
109	1	04/23/2020	62	1153	2	9.3	0.03	37.72
2 multi	2	H						
109	1	04/24/2020	63	1154	2	9.8	0.37	39.47
2 multi	2	H						
109	1	04/25/2020	64	1155	2	9.6	0	38.22
2 multi	2	H						
109	1	04/26/2020	65	1156	2	9.8	0.34	42.4
2 multi	2	H						
109	1	04/27/2020	66	1157	2	10	0.19	42.62
2 multi	2	H						
109	1	04/28/2020	67	1158	2	10.2	0.41	41.56
2 multi	2	H						
109	1	04/29/2020	68	1159	2	9.8	0.32	39.05
2 multi	2	H						
109	1	04/30/2020	69	1160	2	10	0.32	43.58

2 multi 2		H						
109	1	05/01/2020	70	1161	2	9.9	0	40.23
2 multi 2		H						
109	1	05/02/2020	71	1162	2	9.6	0.01	44.39
2 multi 2		H						
109	1	05/03/2020	72	1163	2	9.9	0	41.82
2 multi 2		H						
109	1	05/04/2020	73	1164	2	9.9	0	43.93
2 multi 2		H						
109	1	05/05/2020	74	1165	2	9.6	0.03	41.39
2 multi 2		H						
109	1	05/06/2020	75	1166	2	9.9	0.16	41.65
2 multi 2		H						
109	1	05/07/2020	76	1167	2	10.2	0.05	41.77
2 multi 2		H						
109	1	05/08/2020	77	1168	2	10.2	0	41.08
2 multi 2		H						
109	1	05/09/2020	78	1169	2	10	0.42	42.29
2 multi 2		H						
109	1	05/10/2020	79	1170	2	10	0.38	42.66
2 multi 2		H						
109	1	05/11/2020	80	1171	2	10.3	0.11	37.19
2 multi 2		H						
109	1	05/12/2020	81	1172	2	9.3	0	34.23
2 multi 2		H						
109	1	05/13/2020	82	1173	2	10.4	0.01	38.16
2 multi 2		H						
109	1	05/14/2020	83	1174	2	10.3	0.52	39.85
2 multi 2		H						
109	1	05/15/2020	84	1175	2	10.2	0.01	40.31
2 multi 2		H						
109	1	05/16/2020	85	1176	2	10.3	0.08	41.68
2 multi 2		H						
109	1	05/17/2020	86	1177	2	10.6	0	40.57
2 multi 2		H						
109	1	05/18/2020	87	1178	2	10.3	0.16	41.77
2 multi 2		H						
109	1	05/19/2020	88	1179	2	10.4	0.06	39.2
2 multi 2		H						
109	1	05/20/2020	89	1180	2	10.3	0	39.53
2 multi 2		H						
109	1	05/21/2020	90	1181	2	10.6	0	39.69
2 multi 2		H						
109	1	05/22/2020	91	1182	2	10.3	0.17	42.8
2 multi 2		H						
109	1	05/23/2020	92	1183	2	10.6	0.44	40.4
2 multi 2		H						
109	1	05/24/2020	93	1184	2	10.7	0.37	40.92
2 multi 2		H						
109	1	05/25/2020	94	1185	2	10.4	0	43.36
2 multi 2		H						
109	1	05/26/2020	95	1186	2	10.2	0.4	41.38
2 multi 2		H						
109	1	05/27/2020	96	1187	2	10.9	0	38.47

2 multi	2	H						
109	1	05/28/2020	97	1188	2	10.2	0.61	38.13
2 multi	2	H						
109	1	05/29/2020	98	1189	2	10.7	0.01	44.45
2 multi	2	H						
109	1	05/30/2020	99	1190	2	10.9	0.38	39
2 multi	2	H						
109	1	05/31/2020	100	1191	2	10.7	0.08	40.64
2 multi	2	H						
109	1	06/01/2020	101	1192	2	10.4	0.34	40.9
2 multi	2	H						
109	1	06/02/2020	102	1193	2	10.2	0.02	43.32
2 multi	2	H						
109	1	06/03/2020	103	1194	2	10.6	0.44	37.36
2 multi	2	H						
109	1	06/04/2020	104	1195	2	10.4	0.03	40.66
2 multi	2	H						
109	1	06/05/2020	105	1196	2	10.4	0.16	41.25
2 multi	2	H						
109	1	06/06/2020	106	1197	2	10.4	0.19	40.92
2 multi	2	H						
109	1	06/07/2020	107	1198	2	10.9	0.01	40.68
2 multi	2	H						
109	1	06/08/2020	108	1199	2	10.4	0	44.7
2 multi	2	H						
109	1	06/09/2020	109	1200	2	10.4	0.8	41.6
2 multi	2	H						
109	1	06/10/2020	110	1201	2	10.2	0.37	41.41
2 multi	2	H						
109	1	06/11/2020	111	1202	2	10.3	0.18	40.31
2 multi	2	H						
109	1	06/12/2020	112	1203	2	10.2	0.01	41.01
2 multi	2	H						
109	1	06/13/2020	113	1204	2	10.3	0.1	43.11
2 multi	2	H						
109	1	06/14/2020	114	1205	2	10.6	0.04	42.94
2 multi	2	H						
109	1	06/15/2020	115	1206	2	10.3	0.47	39.37
2 multi	2	H						
109	1	06/16/2020	116	1207	2	9.9	0	37.44
2 multi	2	H						
109	1	06/17/2020	117	1208	2	9.8	0	27.81
2 multi	2	H						
109	1	06/18/2020	118	1209	2	10.2	0.02	30.35
2 multi	2	H						
109	1	06/19/2020	119	1210	2	9.5	0.26	31.31
2 multi	2	H						
109	1	06/20/2020	120	1211	2	9.9	0.03	34.46
2 multi	2	H						
109	1	06/21/2020	121	1212	2	9.9	0.03	34.86
2 multi	2	H						
109	1	06/22/2020	122	1213	2	10.2	0	35.94
2 multi	2	H						
109	1	06/23/2020	123	1214	2	10.3	0.12	35.93

2 multi 2		H						
109	1	06/24/2020	124	1215	2	10.2	0	37.61
2 multi 2		H						
109	1	06/30/2020	130	1221	2	9.9	0	34.44
2 multi 2		H						
109	1	07/01/2020	131	1222	2	10	0	35.42
2 multi 2		H						
109	1	07/02/2020	132	1223	2	10	0	36.05
2 multi 2		H						
109	1	07/03/2020	133	1224	2	10.2	0.23	40.55
2 multi 2		H						
109	1	07/04/2020	134	1225	2	10.4	0.07	38.13
2 multi 2		H						
109	1	07/05/2020	135	1226	2	9.6	0.21	38.15
2 multi 2		H						
109	1	07/06/2020	136	1227	2	10.3	0.03	39.44
2 multi 2		H						
109	1	07/07/2020	137	1228	2	10.2	0.04	37.64
2 multi 2		H						
109	1	07/08/2020	138	1229	2	10.6	0.01	37.51
2 multi 2		H						
109	1	07/09/2020	139	1230	2	10.3	0.01	38.43
2 multi 2		H						
109	1	07/10/2020	140	1231	2	10.4	0.15	40.94
2 multi 2		H						
109	1	07/11/2020	141	1232	2	10	0.19	35.81
2 multi 2		H						
109	1	07/12/2020	142	1233	2	10.2	0.4	35.6
2 multi 2		H						
109	1	07/13/2020	143	1234	2	10.4	0.18	37.26
2 multi 2		H						
109	1	07/14/2020	144	1235	2	9.9	0.7	38.06
2 multi 2		H						
109	1	07/15/2020	145	1236	2	10.3	0.34	37.35
2 multi 2		H						
109	1	07/16/2020	146	1237	2	10.3	0.28	38.34
2 multi 2		H						
109	1	07/17/2020	147	1238	2	9.9	0.2	38.39
2 multi 2		H						
109	1	07/18/2020	148	1239	2	9.9	0	38.28
2 multi 2		H						
109	1	07/19/2020	149	1240	2	9.8	0.5	37.14
2 multi 2		H						
109	1	07/20/2020	150	1241	2	10.2	0.06	39.14
2 multi 2		H						
109	1	07/21/2020	151	1242	2	10.2	0.59	38
2 multi 2		H						
109	1	07/22/2020	152	1243	2	9.8	0.57	39.1
2 multi 2		H						
109	1	07/23/2020	153	1244	2	9.8	0.41	35.77
2 multi 2		H						
109	1	07/24/2020	154	1245	2	10.2	0.49	37.51
2 multi 2		H						
109	1	07/25/2020	155	1246	2	10.6	0.45	36.96

2 multi 2 H
109 1 07/26/2020 156 1247 2 10.3 0.53 37.37
2 multi 2 H
109 1 07/27/2020 157 1248 2 10.3 0.39 35.62
2 multi 2 H
109 1 07/28/2020 158 1249 2 10.3 0 34.77
2 multi 2 H
109 1 07/29/2020 159 1250 2 10.7 0 38.22
2 multi 2 H
109 1 07/30/2020 160 1251 2 9.5 0.21 34.6
2 multi 2 H
109 1 07/31/2020 161 1252 2 10.4 0.01 36.33
2 multi 2 H
109 1 08/01/2020 162 1253 2 9.9 0.52 34.9
2 multi 2 H
109 1 08/02/2020 163 1254 2 10 0.19 36.17
2 multi 2 H
109 1 08/03/2020 164 1255 2 9.9 0 36.27
2 multi 2 H
109 1 08/04/2020 165 1256 2 10.2 0.31 38.12
2 multi 2 H
109 1 08/05/2020 166 1257 2 9.6 0.67 34.68
2 multi 2 H
109 1 08/06/2020 167 1258 2 10.3 0.6 37.26
2 multi 2 H
109 1 08/07/2020 168 1259 2 10.6 0.17 37.5
2 multi 2 H
109 1 08/08/2020 169 1260 2 10.3 0.27 34.23
2 multi 2 H
109 1 08/09/2020 170 1261 2 10.6 0.15 36.96
2 multi 2 H
109 1 08/10/2020 171 1262 2 10.2 0.05 34.28
2 multi 2 H
109 1 08/11/2020 172 1263 2 10 0.56 33.13
2 multi 2 H
109 1 08/12/2020 173 1264 2 10.3 0 33.73
2 multi 2 H
109 1 08/13/2020 174 1265 2 9.9 0 31.62
2 multi 2 H
109 1 08/14/2020 175 1266 2 10.3 0.02 31.84
2 multi 2 H
109 1 08/15/2020 176 1267 2 10.2 0 29.8
2 multi 2 H
109 1 08/16/2020 177 1268 2 9.9 0.16 29.56
2 multi 2 H
109 1 04/30/2021 17 1525 3 9.8 0.27 30.83
2 multi 1 H
109 1 05/01/2021 18 1526 3 10.4 0.36 33.35
2 multi 1 H
109 1 05/02/2021 19 1527 3 10.2 0 32.15
2 multi 1 H
109 1 05/03/2021 20 1528 3 10.3 0.08 31.78
2 multi 1 H
109 1 05/04/2021 21 1529 3 10.3 0.08 32.85

2 multi 1		H						
109	1	05/05/2021	22	1530	3	10	0.01	33.44
2 multi 2		H						
109	1	05/06/2021	23	1531	3	9.9	0.01	29.56
2 multi 2		H						
109	1	05/07/2021	24	1532	3	10.2	0.09	32.42
2 multi 2		H						
109	1	05/08/2021	25	1533	3	10.6	0.08	31.41
2 multi 2		H						
109	1	05/09/2021	26	1534	3	10.6	0	36.02
2 multi 2		H						
109	1	05/10/2021	27	1535	3	10.7	0.07	36.41
2 multi 2		H						
109	1	05/11/2021	28	1536	3	10.7	0.07	36.19
2 multi 2		H						
109	1	05/12/2021	29	1537	3	10.7	0.07	38.29
2 multi 2		H						
109	1	05/13/2021	30	1538	3	10.3	0.06	38.73
2 multi 2		H						
109	1	05/14/2021	31	1539	3	10.3	0.05	40.2
2 multi 2		H						
109	1	05/15/2021	32	1540	3	10.2	0.05	40.35
2 multi 2		H						
109	1	05/16/2021	33	1541	3	10	0.06	37.68
2 multi 2		H						
109	1	05/17/2021	34	1542	3	9.9	0.28	40.38
2 multi 2		H						
109	1	05/18/2021	35	1543	3	10.3	0.13	42.7
2 multi 2		H						
109	1	05/19/2021	36	1544	3	10	0.06	41.84
2 multi 2		H						
109	1	05/20/2021	37	1545	3	10	0	41.02
2 multi 2		H						
109	1	05/21/2021	38	1546	3	10.2	0	41.64
2 multi 2		H						
109	1	05/22/2021	39	1547	3	10	0	43.88
2 multi 2		H						
109	1	05/23/2021	40	1548	3	9.9	0.06	44.57
2 multi 2		H						
109	1	05/24/2021	41	1549	3	10	0	46.68
2 multi 2		H						
109	1	05/25/2021	42	1550	3	9.6	0	39.4
2 multi 2		H						
109	1	05/26/2021	43	1551	3	10	0.07	24.04
2 multi 2		H						
110	1	03/31/2020	5	792	1	8.9	0.17	23.19
1 primi 1		H						
110	1	04/01/2020	6	793	1	8.8	0.01	25.76
1 primi 1		H						
110	1	04/02/2020	7	794	1	8.8	0.02	25.97
1 primi 1		H						
110	1	04/03/2020	8	795	1	8.7	0	27.63
1 primi 1		H						
110	1	04/04/2020	9	796	1	8.7	0	29.17

1 primi	1	H						
110	1	04/05/2020	10	797	1	8.8	0	30.4
1 primi	1	H						
110	1	04/06/2020	11	798	1	10.2	0	32.7
1 primi	1	H						
110	1	04/07/2020	12	799	1	9.9	0	32.23
1 primi	1	H						
110	1	04/08/2020	13	800	1	9.5	0.31	32.37
1 primi	1	H						
110	1	04/09/2020	14	801	1	8.9	0.09	33.61
1 primi	1	H						
110	1	04/10/2020	15	802	1	8.8	0.19	33.42
1 primi	1	H						
110	1	04/11/2020	16	803	1	8.8	0.17	35.51
1 primi	1	H						
110	1	04/12/2020	17	804	1	8.8	0.08	33.46
1 primi	1	H						
110	1	04/13/2020	18	805	1	9.3	0	37.54
1 primi	1	H						
110	1	04/14/2020	19	806	1	8.9	0.14	37.22
1 primi	1	H						
110	1	04/15/2020	20	807	1	9.2	0	38.9
1 primi	1	H						
110	1	04/16/2020	21	808	1	9.3	0	37.7
1 primi	1	H						
110	1	04/17/2020	22	809	1	9.1	0.31	35.3
1 primi	2	H						
110	1	04/18/2020	23	810	1	9.2	0.03	38.8
1 primi	2	H						
110	1	04/19/2020	24	811	1	9.3	0.03	38.55
1 primi	2	H						
110	1	04/20/2020	25	812	1	9.1	0.08	38.08
1 primi	2	H						
110	1	04/21/2020	26	813	1	8.9	0	36.33
1 primi	2	H						
110	1	04/22/2020	27	814	1	8.9	0.28	38.08
1 primi	2	H						
110	1	04/23/2020	28	815	1	9.1	0.22	37.76
1 primi	2	H						
110	1	04/24/2020	29	816	1	9.2	0	37.21
1 primi	2	H						
110	1	04/25/2020	30	817	1	8.9	0.42	36.42
1 primi	2	H						
110	1	04/26/2020	31	818	1	9.3	0	38.3
1 primi	2	H						
110	1	04/27/2020	32	819	1	9.2	0	38.72
1 primi	2	H						
110	1	04/28/2020	33	820	1	9.5	0	40.54
1 primi	2	H						
110	1	04/29/2020	34	821	1	8.9	0	40.6
1 primi	2	H						
110	1	04/30/2020	35	822	1	9.3	0	40.96
1 primi	2	H						
110	1	05/01/2020	36	823	1	9.1	0	38.09

1 primi 2		H						
110	1	05/02/2020	37	824	1	8.6	0	34.94
1 primi 2		H						
110	1	05/03/2020	38	825	1	8.9	0	37.18
1 primi 2		H						
110	1	05/04/2020	39	826	1	9.1	0.01	37.56
1 primi 2		H						
110	1	05/05/2020	40	827	1	9.2	0.22	33.86
1 primi 2		H						
110	1	05/06/2020	41	828	1	10.3	0	35.94
1 primi 2		H						
110	1	05/07/2020	42	829	1	9.6	0	38.19
1 primi 2		H						
110	1	05/08/2020	43	830	1	9.1	0	39.47
1 primi 2		H						
110	1	05/09/2020	44	831	1	8.9	0	36.71
1 primi 2		H						
110	1	05/10/2020	45	832	1	9.3	0	37.78
1 primi 2		H						
110	1	05/11/2020	46	833	1	9.1	0	39.51
1 primi 2		H						
110	1	05/12/2020	47	834	1	8.8	0	32.52
1 primi 2		H						
110	1	05/13/2020	48	835	1	9.6	0	37.01
1 primi 2		H						
110	1	05/14/2020	49	836	1	9.6	0	38.57
1 primi 2		H						
110	1	05/15/2020	50	837	1	9.1	0.2	34.25
1 primi 2		H						
110	1	05/16/2020	51	838	1	9.5	0.01	36.28
1 primi 2		H						
110	1	05/17/2020	52	839	1	9.3	0	34.45
1 primi 2		H						
110	1	05/18/2020	53	840	1	9.5	0.01	36.52
1 primi 2		H						
110	1	05/19/2020	54	841	1	9.1	0	38.54
1 primi 2		H						
110	1	05/20/2020	55	842	1	9.2	0	35.61
1 primi 2		H						
110	1	05/21/2020	56	843	1	9.3	0.24	34.9
1 primi 2		H						
111	1	12/02/2018	6	805	1	9.5	0.18	17.71
1 primi 1		H						
111	1	12/03/2018	7	806	1	9.6	0.08	18.65
1 primi 1		H						
111	1	12/04/2018	8	807	1	9.3	0	17.87
1 primi 1		H						
111	1	12/05/2018	9	808	1	9.6	0	18.32
1 primi 1		H						
111	1	12/06/2018	10	809	1	9.8	0	20
1 primi 1		H						
111	1	12/07/2018	11	810	1	9.2	0	20.47
1 primi 1		H						
111	1	12/08/2018	12	811	1	9.5	0	22.16

1 primi	1	H							
111	1	12/09/2018	13	812	1	9.6	0	22.38	
1 primi	1	H							
111	1	12/10/2018	14	813	1	9.3	0	21.95	
1 primi	1	H							
111	1	12/11/2018	15	814	1	9.5	0	22.03	
1 primi	1	H							
111	1	12/12/2018	16	815	1	9.2	0	23.64	
1 primi	1	H							
111	1	12/13/2018	17	816	1	9.2	0	22.93	
1 primi	1	H							
111	1	12/14/2018	18	817	1	9.5	0	24.03	
1 primi	1	H							
111	1	12/15/2018	19	818	1	9.8	0.15	24.32	
1 primi	1	H							
111	1	12/16/2018	20	819	1	9.8	0	23.3	
1 primi	1	H							
111	1	12/17/2018	21	820	1	9.2	0	23.16	
1 primi	1	H							
111	1	12/18/2018	22	821	1	9.2	0	24.76	
1 primi	2	H							
111	1	12/19/2018	23	822	1	9.1	0	23.42	
1 primi	2	H							
111	1	12/20/2018	24	823	1	9.1	0.2	24.37	
1 primi	2	H							
111	1	12/21/2018	25	824	1	9.6	0	23.26	
1 primi	2	H							
111	1	12/22/2018	26	825	1	9.5	0	24.58	
1 primi	2	H							
111	1	12/23/2018	27	826	1	8.9	0.02	23.51	
1 primi	2	H							
111	1	12/24/2018	28	827	1	9.1	0	25.34	
1 primi	2	H							
111	1	12/25/2018	29	828	1	9.1	0	25.05	
1 primi	2	H							
111	1	12/26/2018	30	829	1	9.5	0	24.46	
1 primi	2	H							
111	1	12/27/2018	31	830	1	9.2	0	24.47	
1 primi	2	H							
111	1	12/28/2018	32	831	1	9.5	0	24.84	
1 primi	2	H							
111	1	12/29/2018	33	832	1	9.1	0	25.24	
1 primi	2	H							
111	1	12/30/2018	34	833	1	8.9	0.15	24.58	
1 primi	2	H							
111	1	12/31/2018	35	834	1	8.8	0.08	23.15	
1 primi	2	H							
111	1	01/01/2019	36	835	1	8.8	0.05	24.94	
1 primi	2	H							
111	1	01/02/2019	37	836	1	8.9	0.04	23.92	
1 primi	2	H							
111	1	01/03/2019	38	837	1	8.9	0.08	23.88	
1 primi	2	H							
111	1	01/04/2019	39	838	1	8.9	0	23.95	

1 primi 2 H
111 1 01/05/2019 40 839 1 8.4 0 22.2
1 primi 2 H
111 1 01/06/2019 41 840 1 8.7 0 23.4
1 primi 2 H
111 1 01/07/2019 42 841 1 8.8 0 22.7
1 primi 2 H
111 1 01/08/2019 43 842 1 8.5 0.22 20.82
1 primi 2 H
111 1 01/09/2019 44 843 1 8.6 0.25 22.6
1 primi 2 H
111 1 01/10/2019 45 844 1 8.8 0 24.31
1 primi 2 H
111 1 01/11/2019 46 845 1 9.1 0 22.31
1 primi 2 H
111 1 01/12/2019 47 846 1 8.7 0.23 21.79
1 primi 2 H
111 1 01/13/2019 48 847 1 8.8 0.13 22.46
1 primi 2 H
111 1 01/14/2019 49 848 1 8.7 0 23.17
1 primi 2 H
111 1 01/15/2019 50 849 1 8.8 0 23.69
1 primi 2 H
111 1 01/16/2019 51 850 1 9.2 0.06 23.86
1 primi 2 H
111 1 11/14/2019 6 1152 2 8.9 0.27 24.94
2 multi 1 H
111 1 11/15/2019 7 1153 2 9.1 0.16 26.31
2 multi 1 H
111 1 11/16/2019 8 1154 2 9.1 0 25.41
2 multi 1 H
111 1 11/17/2019 9 1155 2 10 0 25.9
2 multi 1 H
111 1 11/18/2019 10 1156 2 9.8 0.02 26.24
2 multi 1 H
111 1 11/19/2019 11 1157 2 10 0 27.74
2 multi 1 H
111 1 11/20/2019 12 1158 2 10.3 0.08 28.48
2 multi 1 H
111 1 11/21/2019 13 1159 2 9.6 0 28.75
2 multi 1 H
111 1 11/22/2019 14 1160 2 9.5 0.07 30.47
2 multi 1 H
111 1 11/23/2019 15 1161 2 9.3 0.08 27.02
2 multi 1 H
111 1 11/24/2019 16 1162 2 9.5 0.1 29.22
2 multi 1 H
111 1 11/25/2019 17 1163 2 9.3 0.17 28.56
2 multi 1 H
111 1 11/26/2019 18 1164 2 9.6 0 29.77
2 multi 1 H
111 1 11/27/2019 19 1165 2 9.8 0.26 29.63
2 multi 1 H
111 1 11/28/2019 20 1166 2 9.6 0 30.01

2 multi	1	H						
111	1	11/29/2019	21	1167	2	9.5	0	29.07
2 multi	1	H						
111	1	11/30/2019	22	1168	2	9.6	0	30.29
2 multi	2	H						
111	1	12/01/2019	23	1169	2	9.1	0.02	27.39
2 multi	2	H						
111	1	12/02/2019	24	1170	2	9.5	0.06	29.29
2 multi	2	H						
111	1	12/03/2019	25	1171	2	9.5	0	28.64
2 multi	2	H						
111	1	12/04/2019	26	1172	2	9.5	0	30.55
2 multi	2	H						
111	1	12/05/2019	27	1173	2	9.3	0	28.28
2 multi	2	H						
111	1	12/06/2019	28	1174	2	9.5	0	30.61
2 multi	2	H						
111	1	12/07/2019	29	1175	2	9.9	0.04	30.23
2 multi	2	H						
111	1	12/08/2019	30	1176	2	10	0	27.99
2 multi	2	H						
111	1	12/09/2019	31	1177	2	9.8	0.16	30.16
2 multi	2	H						
111	1	12/10/2019	32	1178	2	9.8	0.05	30.15
2 multi	2	H						
111	1	12/11/2019	33	1179	2	9.8	0	29.65
2 multi	2	H						
111	1	12/12/2019	34	1180	2	9.5	0.22	25.92
2 multi	2	H						
111	1	12/13/2019	35	1181	2	9.3	0	23.41
2 multi	2	H						
111	1	12/14/2019	36	1182	2	9.3	0.23	23.08
2 multi	2	H						
111	1	12/15/2019	37	1183	2	9.5	0.01	26.31
2 multi	2	H						
111	1	12/16/2019	38	1184	2	9.1	0.22	26.69
2 multi	2	H						
111	1	12/17/2019	39	1185	2	9.3	0	23.98
2 multi	2	H						
111	1	12/18/2019	40	1186	2	9.5	0.16	31.13
2 multi	2	H						
111	1	12/19/2019	41	1187	2	9.6	0.01	25.88
2 multi	2	H						
111	1	12/20/2019	42	1188	2	9.9	0	28.52
2 multi	2	H						
111	1	12/21/2019	43	1189	2	10.4	0	28.53
2 multi	2	H						
111	1	12/22/2019	44	1190	2	9.3	0	26.74
2 multi	2	H						
111	1	12/23/2019	45	1191	2	9.8	0.04	28.67
2 multi	2	H						
111	1	12/24/2019	46	1192	2	9.8	0	29.32
2 multi	2	H						
111	1	12/25/2019	47	1193	2	9.1	0	29.75

2 multi	2	H						
111	1	12/26/2019	48	1194	2	9.5	0	28.65
2 multi	2	H						
111	1	12/27/2019	49	1195	2	9.6	0.02	28.31
2 multi	2	H						
111	1	12/28/2019	50	1196	2	9.5	0	28.91
2 multi	2	H						
111	1	12/29/2019	51	1197	2	9.9	0	29.96
2 multi	2	H						
111	1	12/30/2019	52	1198	2	9.9	0	30.61
2 multi	2	H						
111	1	12/31/2019	53	1199	2	9.9	0	30.42
2 multi	2	H						
111	1	10/26/2020	5	1499	3	9.1	0.24	24.84
2 multi	1	H						
111	1	10/27/2020	6	1500	3	9.1	0.07	19.8
2 multi	1	H						
111	1	10/28/2020	7	1501	3	9.1	0.1	23.04
2 multi	1	H						
111	1	10/29/2020	8	1502	3	9.1	0.06	24.7
2 multi	1	H						
111	1	10/30/2020	9	1503	3	8.8	0.04	23.96
2 multi	1	H						
111	1	10/31/2020	10	1504	3	8.8	0.06	26.87
2 multi	1	H						
111	1	11/01/2020	11	1505	3	9.1	0.05	25.33
2 multi	1	H						
111	1	11/02/2020	12	1506	3	8.9	0.06	27.98
2 multi	1	H						
111	1	11/03/2020	13	1507	3	9.1	0	27.23
2 multi	1	H						
111	1	11/04/2020	14	1508	3	9.2	0.04	27.55
2 multi	1	H						
111	1	11/05/2020	15	1509	3	8.9	0.04	28.01
2 multi	1	H						
111	1	11/06/2020	16	1510	3	8.8	0.05	27.45
2 multi	1	H						
111	1	11/07/2020	17	1511	3	8.7	0.07	27.85
2 multi	1	H						
111	1	11/08/2020	18	1512	3	8.9	0.08	28.69
2 multi	1	H						
111	1	11/09/2020	19	1513	3	9.1	0.33	29.24
2 multi	1	H						
111	1	11/10/2020	20	1514	3	9.1	0.28	30.82
2 multi	1	H						
111	1	11/11/2020	21	1515	3	8.9	0.1	31.64
2 multi	1	H						
111	1	11/12/2020	22	1516	3	8.9	0.05	31.64
2 multi	2	H						
111	1	11/13/2020	23	1517	3	9.1	0.1	31.4
2 multi	2	H						
111	1	11/14/2020	24	1518	3	9.5	0.05	34.18
2 multi	2	H						
111	1	11/15/2020	25	1519	3	9.1	0.02	33.05

```
2 multi 2       H
111     1       11/16/2020      26      1520    3       9.3     0.15    32.33
2 multi 2       H
111     1       11/17/2020      27      1521    3       9.5     0       34.4
2 multi 2       H
111     1       11/18/2020      28      1522    3       9.2     0.02    33.06
2 multi 2       H
111     1       11/19/2020      29      1523    3       9.1     0       31.24
2 multi 2       H
111     1       11/20/2020      30      1524    3       9.1     0.08    33.5
2 multi 2       H
111     1       11/21/2020      31      1525    3       9.3     0.04    33.95
2 multi 2       H
112     1       08/02/2020      7       761     1       10      0       20.04
1 primi 1       C
112     1       08/03/2020      8       762     1       10      0.11    20
1 primi 1       C
112     1       08/04/2020      9       763     1       9.9     0.11    21.63
1 primi 1       C
112     1       08/05/2020      10      764     1       9.3     0.04    21.45
1 primi 1       C
112     1       08/06/2020      11      765     1       9.1     0.03    20.49
1 primi 1       C
112     1       08/07/2020      12      766     1       9.5     0       21.57
1 primi 1       C
112     1       08/08/2020      13      767     1       9.5     0.12    22.35
1 primi 1       C
112     1       08/09/2020      14      768     1       9.5     0.05    23.58
1 primi 1       C
112     1       08/10/2020      15      769     1       9.3     0.03    24.87
1 primi 1       C
112     1       08/11/2020      16      770     1       9.3     0.04    22.43
1 primi 1       C
112     1       08/12/2020      17      771     1       9.3     0       22.47
1 primi 1       C
112     1       08/13/2020      18      772     1       8.9     0.15    22.3
1 primi 1       C
112     1       08/14/2020      19      773     1       9.8     0.11    25.3
1 primi 1       C
112     1       08/15/2020      20      774     1       9.6     0       24.54
1 primi 1       C
112     1       08/16/2020      21      775     1       9.5     0       26.53
1 primi 1       C
112     1       08/17/2020      22      776     1       9.2     0.02    24.37
1 primi 2       C
112     1       08/18/2020      23      777     1       9.3     0.02    26.46
1 primi 2       C
112     1       08/19/2020      24      778     1       9.2     0.03    26.63
1 primi 2       C
112     1       08/20/2020      25      779     1       9.2     0       26.72
1 primi 2       C
112     1       08/21/2020      26      780     1       9.3     0.03    27.84
1 primi 2       C
112     1       08/22/2020      27      781     1       9.3     0.11    28.35
```

1 primi 2 C
112 1 08/23/2020 28 782 1 9.3 0.06 27.72
1 primi 2 C
112 1 08/24/2020 29 783 1 9.5 0.03 27.57
1 primi 2 C
112 1 08/25/2020 30 784 1 9.2 0 27.35
1 primi 2 C
112 1 08/26/2020 31 785 1 9.2 0.01 27.55
1 primi 2 C
112 1 08/27/2020 32 786 1 9.1 0.12 27.08
1 primi 2 C
112 1 08/28/2020 33 787 1 9.3 0.05 26.76
1 primi 2 C
112 1 08/29/2020 34 788 1 9.1 0.04 26.48
1 primi 2 C
112 1 08/30/2020 35 789 1 9.1 0.04 26.98
1 primi 2 C
112 1 08/31/2020 36 790 1 9.3 0 25.4
1 primi 2 C
112 1 09/01/2020 37 791 1 9.2 0.06 27.46
1 primi 2 C
112 1 09/02/2020 38 792 1 9.2 0 26.4
1 primi 2 C
112 1 09/03/2020 39 793 1 9.2 0 25.64
1 primi 2 C
112 1 09/04/2020 40 794 1 9.3 0.12 28.12
1 primi 2 C
112 1 09/05/2020 41 795 1 9.3 0 27.45
1 primi 2 C
112 1 09/06/2020 42 796 1 9.5 0.18 28.83
1 primi 2 C
112 1 09/07/2020 43 797 1 9.5 0.19 29.15
1 primi 2 C
112 1 09/08/2020 44 798 1 10.4 0.04 27.58
1 primi 2 C
112 1 09/09/2020 45 799 1 9.6 0.12 29.9
1 primi 2 C
112 1 09/10/2020 46 800 1 9.3 0 28.59
1 primi 2 C
113 1 08/16/2020 6 798 1 11.4 0.17 5.27
1 primi 1 H
113 1 08/17/2020 7 799 1 11.1 0.16 5.58
1 primi 1 H
113 1 08/18/2020 8 800 1 10.3 0.08 3.41
1 primi 1 H
113 1 08/19/2020 9 801 1 10.2 0.33 3.78
1 primi 1 H
113 1 08/20/2020 10 802 1 9.2 0.24 7.52
1 primi 1 H
113 1 08/21/2020 11 803 1 10.4 0.12 3.68
1 primi 1 H
113 1 08/22/2020 12 804 1 11.7 0.24 10.7
1 primi 1 H
113 1 08/23/2020 13 805 1 10 0.43 8.74

1 primi	1	H						
113	1	08/24/2020	14	806	1	10.2	0.3	10.08
1 primi	1	H						
113	1	08/25/2020	15	807	1	9.1	0.26	12.51
1 primi	1	H						
113	1	08/26/2020	16	808	1	9.1	0.23	16.12
1 primi	1	H						
113	1	08/27/2020	17	809	1	9.8	0.19	16.93
1 primi	1	H						
113	1	08/28/2020	18	810	1	9.1	0.23	17.75
1 primi	1	H						
113	1	08/29/2020	19	811	1	8.8	0.11	20.5
1 primi	1	H						
113	1	08/30/2020	20	812	1	8.6	0.05	20.4
1 primi	1	H						
113	1	08/31/2020	21	813	1	8.6	0.07	22.57
1 primi	1	H						
113	1	09/01/2020	22	814	1	8.5	0.06	22.58
1 primi	2	H						
113	1	09/02/2020	23	815	1	9.1	0.07	23.14
1 primi	2	H						
113	1	09/03/2020	24	816	1	9.1	0.07	25.11
1 primi	2	H						
113	1	09/04/2020	25	817	1	8.8	0.04	26.19
1 primi	2	H						
113	1	09/05/2020	26	818	1	8.7	0.05	25.93
1 primi	2	H						
113	1	09/06/2020	27	819	1	8.7	0.06	26.39
1 primi	2	H						
113	1	09/07/2020	28	820	1	8.6	0.07	27.55
1 primi	2	H						
113	1	09/08/2020	29	821	1	8.6	0.01	27.17
1 primi	2	H						
113	1	09/09/2020	30	822	1	8.4	0.53	28.21
1 primi	2	H						
113	1	09/10/2020	31	823	1	8.4	0.06	27.72
1 primi	2	H						
113	1	09/11/2020	32	824	1	8.6	0.06	29.07
1 primi	2	H						
113	1	09/12/2020	33	825	1	8.2	0.13	29.69
1 primi	2	H						
113	1	09/13/2020	34	826	1	8.6	0.06	29.47
1 primi	2	H						
113	1	09/14/2020	35	827	1	8.4	0.06	30.86
1 primi	2	H						
113	1	09/15/2020	36	828	1	8.5	0.05	30.52
1 primi	2	H						
113	1	09/16/2020	37	829	1	8.7	0	30.05
1 primi	2	H						
113	1	09/17/2020	38	830	1	8.5	0.25	30.96
1 primi	2	H						
113	1	09/18/2020	39	831	1	8.6	0.68	29.09
1 primi	2	H						
113	1	09/19/2020	40	832	1	8.4	0	29.08

1 primi 2 H
113 1 09/20/2020 41 833 1 8.3 0.13 27.77
1 primi 2 H
113 1 09/21/2020 42 834 1 8.6 0 32.54
1 primi 2 H
113 1 09/22/2020 43 835 1 8.6 0.19 26.98
1 primi 2 H
113 1 09/23/2020 44 836 1 8.7 0.12 32.99
1 primi 2 H
113 1 09/24/2020 45 837 1 9.2 0.06 30.96
1 primi 2 H
113 1 09/25/2020 46 838 1 9.3 0.06 32.86
1 primi 2 H
113 1 09/26/2020 47 839 1 8.6 0.06 31.28
1 primi 2 H
113 1 09/27/2020 48 840 1 8.2 0.05 32.61
1 primi 2 H
113 1 09/28/2020 49 841 1 8.5 0.04 33.43
1 primi 2 H
114 1 05/11/2020 4 794 1 8.8 0.46 17.88
1 primi 1 C
114 1 05/12/2020 5 795 1 9.2 0.11 20.26
1 primi 1 C
114 1 05/13/2020 6 796 1 9.3 0.15 21.93
1 primi 1 C
114 1 05/14/2020 7 797 1 8.9 0.22 21.61
1 primi 1 C
114 1 05/15/2020 8 798 1 8.5 0.05 22.19
1 primi 1 C
114 1 05/16/2020 9 799 1 9.1 0.18 23.31
1 primi 1 C
114 1 05/17/2020 10 800 1 9.1 0.2 24.29
1 primi 1 C
114 1 05/18/2020 11 801 1 8.9 0.07 25.45
1 primi 1 C
114 1 05/19/2020 12 802 1 9.3 0.17 24.96
1 primi 1 C
114 1 05/20/2020 13 803 1 9.2 0.03 25.47
1 primi 1 C
114 1 05/21/2020 14 804 1 9.1 0.12 24.62
1 primi 1 C
114 1 05/22/2020 15 805 1 9.2 0.09 27.94
1 primi 1 C
114 1 05/23/2020 16 806 1 9.3 0.17 25.62
1 primi 1 C
114 1 05/24/2020 17 807 1 8.8 0.02 25.77
1 primi 1 C
114 1 05/25/2020 18 808 1 8.8 0.12 27.11
1 primi 1 C
114 1 05/26/2020 19 809 1 8.8 0.39 24.33
1 primi 1 C
114 1 05/27/2020 20 810 1 9.2 0.3 24.78
1 primi 1 C
114 1 05/28/2020 21 811 1 9.6 0.08 27.11

1 primi	1	C						
114	1	05/29/2020	22	812	1	9.5	0.01	26.54
1 primi	2	C						
114	1	05/30/2020	23	813	1	9.8	0.05	25.96
1 primi	2	C						
114	1	05/31/2020	24	814	1	9.3	0.06	27.47
1 primi	2	C						
114	1	06/01/2020	25	815	1	9.2	0.12	26.17
1 primi	2	C						
114	1	06/02/2020	26	816	1	8.8	0.12	24.96
1 primi	2	C						
114	1	06/03/2020	27	817	1	8.8	0.14	26.15
1 primi	2	C						
114	1	06/04/2020	28	818	1	8.8	0.08	24.13
1 primi	2	C						
114	1	06/05/2020	29	819	1	8.8	0	27.1
1 primi	2	C						
114	1	06/06/2020	30	820	1	9.1	0.11	26.99
1 primi	2	C						
114	1	06/07/2020	31	821	1	9.2	0.04	29.66
1 primi	2	C						
114	1	06/08/2020	32	822	1	8.7	0	27.59
1 primi	2	C						
114	1	06/09/2020	33	823	1	8.9	0	25.09
1 primi	2	C						
114	1	06/10/2020	34	824	1	8.6	0.38	24.02
1 primi	2	C						
114	1	06/11/2020	35	825	1	9.2	0.27	25.27
1 primi	2	C						
114	1	06/12/2020	36	826	1	8.8	0.04	26.93
1 primi	2	C						
114	1	06/13/2020	37	827	1	8.9	0.24	26.32
1 primi	2	C						
114	1	06/14/2020	38	828	1	9.1	0.14	29.36
1 primi	2	C						
114	1	06/15/2020	39	829	1	8.7	0.22	27.52
1 primi	2	C						
114	1	06/16/2020	40	830	1	8.7	0.02	28.58
1 primi	2	C						
114	1	06/17/2020	41	831	1	9.3	0.1	28.55
1 primi	2	C						
114	1	06/18/2020	42	832	1	9.2	0.1	28.27
1 primi	2	C						
114	1	06/19/2020	43	833	1	9.2	0.42	30.04
1 primi	2	C						
114	1	06/20/2020	44	834	1	9.2	0	30.82
1 primi	2	C						
114	1	06/21/2020	45	835	1	8.9	0	30.02
1 primi	2	C						
114	1	06/22/2020	46	836	1	8.8	0.02	29.22
1 primi	2	C						
114	1	06/23/2020	47	837	1	8.9	0.04	31.24
1 primi	2	C						
114	1	06/24/2020	48	838	1	8.9	0	26.86

1 primi 2		C						
115	1	03/26/2020	9	809	1	9.2	0.28	20.85
1 primi 1		H						
115	1	03/27/2020	10	810	1	10.2	0.1	21.95
1 primi 1		H						
115	1	03/28/2020	11	811	1	10.2	0.18	22.11
1 primi 1		H						
115	1	03/29/2020	12	812	1	9.8	0.18	22.23
1 primi 1		H						
115	1	03/30/2020	13	813	1	9.9	0.24	23.46
1 primi 1		H						
115	1	03/31/2020	14	814	1	9.9	0.18	23.63
1 primi 1		H						
115	1	04/01/2020	15	815	1	9.8	0.18	23.33
1 primi 1		H						
115	1	04/02/2020	16	816	1	9.2	0.2	23.81
1 primi 1		H						
115	1	04/03/2020	17	817	1	9.5	0.2	25
1 primi 1		H						
115	1	04/04/2020	18	818	1	10.2	0.02	25.81
1 primi 1		H						
115	1	04/05/2020	19	819	1	9.8	0.01	26.32
1 primi 1		H						
115	1	04/06/2020	20	820	1	9.8	0.07	26.8
1 primi 1		H						
115	1	04/07/2020	21	821	1	9.3	0.14	26.52
1 primi 1		H						
115	1	04/08/2020	22	822	1	10.2	0.11	28.74
1 primi 2		H						
115	1	04/09/2020	23	823	1	0	0.01	26.82
1 primi 2		H						
115	1	04/10/2020	24	824	1	9.2	0.28	28.44
1 primi 2		H						
115	1	04/11/2020	25	825	1	9.5	0.31	28.46
1 primi 2		H						
115	1	04/12/2020	26	826	1	9.5	0.33	29.39
1 primi 2		H						
115	1	04/13/2020	27	827	1	9.9	0.2	31.09
1 primi 2		H						
115	1	04/14/2020	28	828	1	9.6	0.15	29.86
1 primi 2		H						
115	1	04/15/2020	29	829	1	9.6	0.04	31.55
1 primi 2		H						
115	1	04/16/2020	30	830	1	9.5	0.12	31.08
1 primi 2		H						
115	1	04/17/2020	31	831	1	9.3	0.3	29.97
1 primi 2		H						
115	1	04/18/2020	32	832	1	9.5	0.28	23.65
1 primi 2		H						
115	1	04/19/2020	33	833	1	9.2	0.35	28.44
1 primi 2		H						
115	1	04/20/2020	34	834	1	9.3	0.35	29.79
1 primi 2		H						
115	1	04/21/2020	35	835	1	9.2	0.27	28.57

1 primi	2	H						
115	1	04/22/2020	36	836	1	9.5	0.42	30.8
1 primi	2	H						
115	1	04/23/2020	37	837	1	9.9	0.28	24.66
1 primi	2	H						
115	1	04/24/2020	38	838	1	9.5	0.28	34.05
1 primi	2	H						
115	1	04/25/2020	39	839	1	9.8	0.33	31.08
1 primi	2	H						
115	1	04/26/2020	40	840	1	9.6	0.39	31.41
1 primi	2	H						
115	1	04/27/2020	41	841	1	9.8	0.01	33.19
1 primi	2	H						
115	1	04/28/2020	42	842	1	10	0.02	31.08
1 primi	2	H						
116	1	10/14/2017	6	824	1	9.1	0.37	22.95
1 primi	1	H						
116	1	10/15/2017	7	825	1	9.3	0	25.1
1 primi	1	H						
116	1	10/16/2017	8	826	1	8.6	0.06	24.42
1 primi	1	H						
116	1	10/17/2017	9	827	1	9.2	0.16	24.46
1 primi	1	H						
116	1	10/18/2017	10	828	1	9.1	0	26.3
1 primi	1	H						
116	1	10/19/2017	11	829	1	9.2	0.04	26.95
1 primi	1	H						
116	1	10/20/2017	12	830	1	9.1	0	29.82
1 primi	1	H						
116	1	10/21/2017	13	831	1	8.9	0	30.95
1 primi	1	H						
116	1	10/22/2017	14	832	1	8.9	0	31.11
1 primi	1	H						
116	1	10/23/2017	15	833	1	9.2	0.19	31.55
1 primi	1	H						
116	1	10/24/2017	16	834	1	9.3	0.08	31.8
1 primi	1	H						
116	1	10/25/2017	17	835	1	9.5	0.03	32.88
1 primi	1	H						
116	1	10/26/2017	18	836	1	9.8	0.42	35.28
1 primi	1	H						
116	1	10/27/2017	19	837	1	9.2	0.06	35.18
1 primi	1	H						
116	1	10/28/2017	20	838	1	9.2	0	37.66
1 primi	1	H						
116	1	10/29/2017	21	839	1	8.9	0	38.29
1 primi	1	H						
116	1	10/30/2017	22	840	1	9.1	0	31.8
1 primi	2	H						
116	1	10/31/2017	23	841	1	9.2	0	31.84
1 primi	2	H						
116	1	11/01/2017	24	842	1	9.1	0.59	35.09
1 primi	2	H						
116	1	11/02/2017	25	843	1	9.2	0.14	38.22

1 primi 2		H						
116	1	11/03/2017	26	844	1	9.1	0.07	37.69
1 primi 2		H						
116	1	11/04/2017	27	845	1	9.2	0	41.04
1 primi 2		H						
116	1	11/05/2017	28	846	1	9.1	0.08	39.86
1 primi 2		H						
116	1	11/06/2017	29	847	1	9.1	0.08	42.01
1 primi 2		H						
116	1	11/07/2017	30	848	1	8.9	0.16	39.55
1 primi 2		H						
116	1	11/08/2017	31	849	1	9.2	0	39.31
1 primi 2		H						
116	1	11/09/2017	32	850	1	8.9	0.18	40.57
1 primi 2		H						
116	1	11/10/2017	33	851	1	9.1	0	40.59
1 primi 2		H						
116	1	11/11/2017	34	852	1	9.2	0	38.64
1 primi 2		H						
116	1	11/12/2017	35	853	1	9.2	0	39.52
1 primi 2		H						
116	1	11/13/2017	36	854	1	9.1	0.17	38.98
1 primi 2		H						
116	1	11/14/2017	37	855	1	9.2	0	38.24
1 primi 2		H						
116	1	11/15/2017	38	856	1	9.1	0	44.35
1 primi 2		H						
116	1	11/16/2017	39	857	1	9.5	0.22	44.24
1 primi 2		H						
116	1	11/17/2017	40	858	1	9.2	0	41.28
1 primi 2		H						
116	1	11/18/2017	41	859	1	9.2	0.1	40.95
1 primi 2		H						
116	1	11/19/2017	42	860	1	9.1	0	41.51
1 primi 2		H						
116	1	11/20/2017	43	861	1	9.1	0.04	45.65
1 primi 2		H						
116	1	11/21/2017	44	862	1	9.1	0	43.59
1 primi 2		H						
116	1	11/22/2017	45	863	1	8.6	0	40.89
1 primi 2		H						
116	1	11/23/2017	46	864	1	9.1	0.28	40.16
1 primi 2		H						
116	1	11/24/2017	47	865	1	8.8	0.05	42.46
1 primi 2		H						
116	1	11/25/2017	48	866	1	9.1	0	40.75
1 primi 2		H						
116	1	11/26/2017	49	867	1	9.1	0	42.21
1 primi 2		H						
116	1	11/27/2017	50	868	1	9.3	0.02	41.76
1 primi 2		H						
116	1	11/28/2017	51	869	1	9.3	0.22	40.91
1 primi 2		H						
116	1	11/29/2017	52	870	1	9.3	0.01	40.9

1 primi 2		H						
116	1	11/30/2017	53	871	1	9.1	0.01	41.32
1 primi 2		H						
116	1	12/01/2017	54	872	1	9.3	0	39.85
1 primi 2		H						
116	1	12/02/2017	55	873	1	9.3	0.02	43.23
1 primi 2		H						
116	1	12/03/2017	56	874	1	9.6	0	45.28
1 primi 2		H						
116	1	12/04/2017	57	875	1	8.9	0.01	42.38
1 primi 2		H						
116	1	12/05/2017	58	876	1	9.1	0.02	37.76
1 primi 2		H						
116	1	12/06/2017	59	877	1	8.9	0.01	41.95
1 primi 2		H						
116	1	12/07/2017	60	878	1	9.1	0.13	43.07
1 primi 2		H						
116	1	12/08/2017	61	879	1	9.3	0	41.72
1 primi 2		H						
116	1	12/09/2017	62	880	1	9.2	0	43.08
1 primi 2		H						
116	1	12/10/2017	63	881	1	9.2	0	44.2
1 primi 2		H						
116	1	12/11/2017	64	882	1	9.5	0	46.22
1 primi 2		H						
116	1	12/12/2017	65	883	1	9.8	0	47.89
1 primi 2		H						
116	1	12/13/2017	66	884	1	9.3	0	39.75
1 primi 2		H						
116	1	12/14/2017	67	885	1	9.2	0.01	40.93
1 primi 2		H						
116	1	12/15/2017	68	886	1	9.2	0.19	40.37
1 primi 2		H						
116	1	12/16/2017	69	887	1	9.2	0.2	44.9
1 primi 2		H						
116	1	12/17/2017	70	888	1	9.2	0	47.94
1 primi 2		H						
116	1	12/18/2017	71	889	1	9.3	0.14	43.95
1 primi 2		H						
116	1	12/19/2017	72	890	1	9.3	0	43.58
1 primi 2		H						
116	1	12/20/2017	73	891	1	9.5	0.26	43.51
1 primi 2		H						
116	1	12/21/2017	74	892	1	10.2	0.09	43.64
1 primi 2		H						
116	1	12/22/2017	75	893	1	9.6	0.15	48.58
1 primi 2		H						
116	1	12/23/2017	76	894	1	9.5	0	44.81
1 primi 2		H						
116	1	12/24/2017	77	895	1	9.6	0.12	48.66
1 primi 2		H						
116	1	12/25/2017	78	896	1	9.3	0.11	46.69
1 primi 2		H						
116	1	12/26/2017	79	897	1	9.5	0.03	42.6

1 primi 2 H
116 1 12/27/2017 80 898 1 9.3 0.39 44.33
1 primi 2 H
116 1 12/28/2017 81 899 1 9.5 0 47.09
1 primi 2 H
116 1 12/29/2017 82 900 1 9.6 0 45.24
1 primi 2 H
116 1 12/30/2017 83 901 1 9.5 0.13 41.11
1 primi 2 H
116 1 12/31/2017 84 902 1 9.6 0 44.23
1 primi 2 H
116 1 01/01/2018 85 903 1 9.2 0.09 40.42
1 primi 2 H
116 1 01/02/2018 86 904 1 9.5 0.47 42.69
1 primi 2 H
116 1 01/03/2018 87 905 1 9.5 0.2 46.13
1 primi 2 H
116 1 01/04/2018 88 906 1 9.8 0 45.57
1 primi 2 H
116 1 01/05/2018 89 907 1 9.8 0.05 44.18
1 primi 2 H
116 1 01/06/2018 90 908 1 9.2 0.28 41.78
1 primi 2 H
116 1 01/07/2018 91 909 1 9.5 0 43.86
1 primi 2 H
116 1 01/08/2018 92 910 1 9.6 0 48.16
1 primi 2 H
116 1 01/09/2018 93 911 1 9.6 0 44.77
1 primi 2 H
116 1 01/10/2018 94 912 1 9.8 0.08 45.79
1 primi 2 H
116 1 01/11/2018 95 913 1 9.8 0.07 45.86
1 primi 2 H
116 1 01/12/2018 96 914 1 9.9 0 45.62
1 primi 2 H
116 1 01/13/2018 97 915 1 10 0 44.96
1 primi 2 H
116 1 01/14/2018 98 916 1 10 0 43.19
1 primi 2 H
116 1 01/15/2018 99 917 1 9.6 0.1 44.06
1 primi 2 H
116 1 01/16/2018 100 918 1 9.6 0.1 42.53
1 primi 2 H
116 1 01/17/2018 101 919 1 9.8 0 45.43
1 primi 2 H
116 1 01/18/2018 102 920 1 9.6 0 45.69
1 primi 2 H
116 1 01/19/2018 103 921 1 9.8 0 45.98
1 primi 2 H
116 1 01/20/2018 104 922 1 10 0 45.73
1 primi 2 H
116 1 01/21/2018 105 923 1 9.8 0 45.63
1 primi 2 H
116 1 01/22/2018 106 924 1 9.8 0 45.64

1 primi 2		H						
116	1	01/23/2018	107	925	1	9.6	0	52.39
1 primi 2		H						
116	1	01/24/2018	108	926	1	9.9	0	48.79
1 primi 2		H						
116	1	01/25/2018	109	927	1	9.6	0	44.17
1 primi 2		H						
116	1	01/26/2018	110	928	1	9.6	0.14	48.53
1 primi 2		H						
116	1	01/27/2018	111	929	1	9.5	0.28	44.71
1 primi 2		H						
116	1	01/28/2018	112	930	1	9.8	0.33	44.53
1 primi 2		H						
116	1	01/29/2018	113	931	1	9.8	0.05	50.1
1 primi 2		H						
116	1	01/30/2018	114	932	1	9.9	0.05	48.13
1 primi 2		H						
116	1	01/31/2018	115	933	1	9.9	0	44.48
1 primi 2		H						
116	1	02/01/2018	116	934	1	9.6	0.63	47.33
1 primi 2		H						
116	1	02/02/2018	117	935	1	9.5	0.23	45.36
1 primi 2		H						
116	1	02/03/2018	118	936	1	9.6	0	41.52
1 primi 2		H						
116	1	02/04/2018	119	937	1	9.6	0.3	45.3
1 primi 2		H						
116	1	02/05/2018	120	938	1	9.6	0	41.83
1 primi 2		H						
116	1	02/06/2018	121	939	1	9.6	0	48.71
1 primi 2		H						
116	1	02/07/2018	122	940	1	9.8	0.21	42.07
1 primi 2		H						
116	1	02/08/2018	123	941	1	10	0.12	47.51
1 primi 2		H						
116	1	02/09/2018	124	942	1	9.6	0	47.14
1 primi 2		H						
116	1	02/10/2018	125	943	1	9.9	0.16	55.44
1 primi 2		H						
116	1	02/11/2018	126	944	1	9.6	0	49.84
1 primi 2		H						
116	1	02/12/2018	127	945	1	9.5	0	44.74
1 primi 2		H						
116	1	02/13/2018	128	946	1	9.6	0	46.64
1 primi 2		H						
116	1	02/14/2018	129	947	1	9.3	0	45.51
1 primi 2		H						
116	1	02/15/2018	130	948	1	8.9	0	44.13
1 primi 2		H						
116	1	02/16/2018	131	949	1	9.3	0	45.24
1 primi 2		H						
116	1	02/17/2018	132	950	1	9.3	0	43.91
1 primi 2		H						
116	1	02/18/2018	133	951	1	9.6	0	42.18

1 primi 2		H						
116	1	02/19/2018	134	952	1	9.5	0.23	42.11
1 primi 2		H						
116	1	02/20/2018	135	953	1	9.5	0	44.2
1 primi 2		H						
116	1	02/21/2018	136	954	1	9.5	0.32	43.21
1 primi 2		H						
116	1	02/22/2018	137	955	1	9.2	0.17	47.94
1 primi 2		H						
116	1	02/23/2018	138	956	1	8.9	0	43.3
1 primi 2		H						
116	1	02/24/2018	139	957	1	8.8	0.2	42.17
1 primi 2		H						
116	1	02/25/2018	140	958	1	8.8	0	41.48
1 primi 2		H						
116	1	02/26/2018	141	959	1	9.3	0.3	41.44
1 primi 2		H						
116	1	02/27/2018	142	960	1	9.2	0	42.08
1 primi 2		H						
116	1	02/28/2018	143	961	1	8.8	0	40.06
1 primi 2		H						
116	1	03/01/2018	144	962	1	9.3	0	41.81
1 primi 2		H						
116	1	03/02/2018	145	963	1	9.2	0.15	42.52
1 primi 2		H						
116	1	03/03/2018	146	964	1	9.3	0.06	45.4
1 primi 2		H						
116	1	03/04/2018	147	965	1	9.3	0	42.01
1 primi 2		H						
116	1	03/05/2018	148	966	1	9.5	0	41.14
1 primi 2		H						
116	1	03/06/2018	149	967	1	9.3	0	47.9
1 primi 2		H						
116	1	03/07/2018	150	968	1	9.5	0	43.79
1 primi 2		H						
116	1	03/08/2018	151	969	1	9.6	0	40.43
1 primi 2		H						
116	1	03/09/2018	152	970	1	9.6	0.22	45.79
1 primi 2		H						
116	1	03/10/2018	153	971	1	9.2	0.32	40.66
1 primi 2		H						
116	1	03/11/2018	154	972	1	9.2	0.28	39.37
1 primi 2		H						
116	1	03/12/2018	155	973	1	9.6	0	42.35
1 primi 2		H						
116	1	03/13/2018	156	974	1	9.3	0	40.69
1 primi 2		H						
116	1	03/14/2018	157	975	1	9.6	0.26	42.94
1 primi 2		H						
116	1	03/15/2018	158	976	1	9.6	0	45.49
1 primi 2		H						
116	1	03/16/2018	159	977	1	8.9	0	44.65
1 primi 2		H						
116	1	03/17/2018	160	978	1	9.2	0	40.44

1 primi 2		H						
116	1	03/18/2018	161	979	1	9.3	0	41.24
1 primi 2		H						
116	1	03/19/2018	162	980	1	9.6	0	46.84
1 primi 2		H						
116	1	03/20/2018	163	981	1	9.9	0	47.01
1 primi 2		H						
116	1	03/21/2018	164	982	1	9.6	0	47.79
1 primi 2		H						
116	1	03/22/2018	165	983	1	9.3	0	43.18
1 primi 2		H						
116	1	03/23/2018	166	984	1	9.2	0.18	43.02
1 primi 2		H						
116	1	03/24/2018	167	985	1	9.5	0.39	42.2
1 primi 2		H						
116	1	03/25/2018	168	986	1	9.5	0	46.62
1 primi 2		H						
116	1	03/26/2018	169	987	1	9.5	0.04	43.47
1 primi 2		H						
116	1	03/27/2018	170	988	1	9.5	0.39	44.14
1 primi 2		H						
116	1	03/28/2018	171	989	1	9.6	0	45.79
1 primi 2		H						
116	1	03/29/2018	172	990	1	9.2	0.25	40.29
1 primi 2		H						
116	1	03/30/2018	173	991	1	9.3	0	41.03
1 primi 2		H						
116	1	03/31/2018	174	992	1	9.3	0.05	39.68
1 primi 2		H						
116	1	04/01/2018	175	993	1	9.6	0	40.69
1 primi 2		H						
116	1	04/02/2018	176	994	1	9.5	0	42.09
1 primi 2		H						
116	1	04/03/2018	177	995	1	9.9	0.12	51.05
1 primi 2		H						
116	1	04/04/2018	178	996	1	10	0	52.81
1 primi 2		H						
116	1	04/05/2018	179	997	1	9.8	0	41.94
1 primi 2		H						
116	1	04/06/2018	180	998	1	9.8	0.04	42.28
1 primi 2		H						
116	1	04/07/2018	181	999	1	9.3	0	40.99
1 primi 2		H						
116	1	04/08/2018	182	1000	1	9.3	0	40.11
1 primi 2		H						
116	1	04/09/2018	183	1001	1	9.3	0	41.88
1 primi 2		H						
116	1	04/10/2018	184	1002	1	9.2	0	40.67
1 primi 2		H						
116	1	04/11/2018	185	1003	1	9.5	0.03	39.61
1 primi 2		H						
116	1	04/12/2018	186	1004	1	9.3	0.04	41.17
1 primi 2		H						
116	1	04/13/2018	187	1005	1	9.6	0	37.8

1 primi 2 H
116 1 04/14/2018 188 1006 1 9.6 0 40.76
1 primi 2 H
116 1 04/15/2018 189 1007 1 9.8 0 39.28
1 primi 2 H
116 1 04/16/2018 190 1008 1 9.8 0 39.51
1 primi 2 H
116 1 04/17/2018 191 1009 1 9.5 0.08 39.84
1 primi 2 H
116 1 04/18/2018 192 1010 1 0 0 40.27
1 primi 2 H
116 1 04/19/2018 193 1011 1 9.3 0 38.57
1 primi 2 H
116 1 04/20/2018 194 1012 1 9.5 0 37.06
1 primi 2 H
116 1 04/21/2018 195 1013 1 9.6 0 37.95
1 primi 2 H
116 1 04/22/2018 196 1014 1 9.6 0.47 37.75
1 primi 2 H
116 1 04/23/2018 197 1015 1 9.3 0 39.53
1 primi 2 H
116 1 04/24/2018 198 1016 1 9.6 0 40.65
1 primi 2 H
116 1 04/25/2018 199 1017 1 9.6 0 39.73
1 primi 2 H
116 1 04/26/2018 200 1018 1 9.6 0.28 38.85
1 primi 2 H
116 1 04/27/2018 201 1019 1 9.3 0 38.97
1 primi 2 H
116 1 04/28/2018 202 1020 1 9.1 0 39.99
1 primi 2 H
116 1 04/29/2018 203 1021 1 9.3 0.34 38.1
1 primi 2 H
116 1 04/30/2018 204 1022 1 9.5 0 40.02
1 primi 2 H
116 1 05/01/2018 205 1023 1 9.5 0 34.45
1 primi 2 H
116 1 05/02/2018 206 1024 1 9.5 0 38.05
1 primi 2 H
116 1 05/03/2018 207 1025 1 9.6 0 40.56
1 primi 2 H
116 1 05/04/2018 208 1026 1 9.5 0 38.28
1 primi 2 H
116 1 05/05/2018 209 1027 1 9.6 0 37.74
1 primi 2 H
116 1 05/06/2018 210 1028 1 9.5 0 40.2
1 primi 2 H
116 1 05/07/2018 211 1029 1 9.5 0 36.28
1 primi 2 H
116 1 05/08/2018 212 1030 1 9.6 0 38.13
1 primi 2 H
116 1 05/09/2018 213 1031 1 9.8 0 37.95
1 primi 2 H
116 1 05/10/2018 214 1032 1 9.6 0 39.96

1 primi	2	H						
116	1	05/11/2018	215	1033	1	9.8	0	39.07
1 primi	2	H						
116	1	05/12/2018	216	1034	1	9.6	0	39.46
1 primi	2	H						
116	1	05/13/2018	217	1035	1	9.8	0	38.55
1 primi	2	H						
116	1	05/14/2018	218	1036	1	9.6	0	39.62
1 primi	2	H						
116	1	05/15/2018	219	1037	1	9.3	0	35.58
1 primi	2	H						
116	1	05/16/2018	220	1038	1	9.6	0	38.12
1 primi	2	H						
116	1	05/17/2018	221	1039	1	10.6	0.05	36.66
1 primi	2	H						
116	1	05/18/2018	222	1040	1	10.2	0	36.85
1 primi	2	H						
116	1	05/19/2018	223	1041	1	9.8	0	32.95
1 primi	2	H						
116	1	05/20/2018	224	1042	1	9.8	0.12	32.21
1 primi	2	H						
116	1	09/09/2018	10	1154	2	9.5	0	28.22
2 multi	1	H						
116	1	09/10/2018	11	1155	2	9.3	0.11	28.54
2 multi	1	H						
116	1	09/11/2018	12	1156	2	9.5	0.04	28.95
2 multi	1	H						
116	1	09/12/2018	13	1157	2	9.8	0.52	33.53
2 multi	1	H						
116	1	09/13/2018	14	1158	2	9.8	0.15	34.6
2 multi	1	H						
116	1	09/14/2018	15	1159	2	9.9	0.04	34.18
2 multi	1	H						
116	1	09/15/2018	16	1160	2	9.8	0.01	35.04
2 multi	1	H						
116	1	09/16/2018	17	1161	2	9.6	0	35.2
2 multi	1	H						
116	1	09/17/2018	18	1162	2	9.5	0	38.07
2 multi	1	H						
116	1	09/18/2018	19	1163	2	9.9	0	36.68
2 multi	1	H						
116	1	09/19/2018	20	1164	2	9.8	0.01	39.24
2 multi	1	H						
116	1	09/20/2018	21	1165	2	9.8	0	38.56
2 multi	1	H						
116	1	09/21/2018	22	1166	2	9.6	0	40.72
2 multi	2	H						
116	1	09/22/2018	23	1167	2	9.8	0	37.76
2 multi	2	H						
116	1	09/23/2018	24	1168	2	9.6	0	38.79
2 multi	2	H						
116	1	09/24/2018	25	1169	2	9.5	0	38.76
2 multi	2	H						
116	1	09/25/2018	26	1170	2	9.5	0.01	39.29

2 multi 2		H						
116	1	09/26/2018	27	1171	2	9.3	0.05	37.96
2 multi 2		H						
116	1	09/27/2018	28	1172	2	9.5	0	39.2
2 multi 2		H						
116	1	09/28/2018	29	1173	2	9.5	0.13	36.97
2 multi 2		H						
116	1	09/29/2018	30	1174	2	9.5	0.08	39.91
2 multi 2		H						
116	1	09/30/2018	31	1175	2	9.6	0.09	38.64
2 multi 2		H						
116	1	10/01/2018	32	1176	2	9.5	0.01	39.77
2 multi 2		H						
116	1	10/02/2018	33	1177	2	9.6	0.01	41.63
2 multi 2		H						
116	1	10/03/2018	34	1178	2	9.5	0.16	38.94
2 multi 2		H						
116	1	10/04/2018	35	1179	2	9.2	0.09	37.14
2 multi 2		H						
116	1	10/05/2018	36	1180	2	9.3	0.13	37.16
2 multi 2		H						
116	1	10/06/2018	37	1181	2	9.3	0.2	36.42
2 multi 2		H						
116	1	10/07/2018	38	1182	2	9.5	0	39.05
2 multi 2		H						
116	1	10/08/2018	39	1183	2	9.5	0.27	38.55
2 multi 2		H						
116	1	10/09/2018	40	1184	2	9.3	0.04	35.23
2 multi 2		H						
116	1	10/10/2018	41	1185	2	9.8	0.21	37.14
2 multi 2		H						
116	1	10/11/2018	42	1186	2	9.6	0.03	37.17
2 multi 2		H						
116	1	10/12/2018	43	1187	2	9.8	0.06	39.33
2 multi 2		H						
116	1	10/13/2018	44	1188	2	9.9	0	39.89
2 multi 2		H						
116	1	10/14/2018	45	1189	2	9.3	0	38.57
2 multi 2		H						
116	1	10/15/2018	46	1190	2	9.6	0	36.74
2 multi 2		H						
116	1	10/16/2018	47	1191	2	9.5	0	36.17
2 multi 2		H						
116	1	10/17/2018	48	1192	2	9.5	0	36.56
2 multi 2		H						
116	1	10/18/2018	49	1193	2	9.6	0.14	37.23
2 multi 2		H						
116	1	10/19/2018	50	1194	2	9.6	0.16	33.22
2 multi 2		H						
116	1	10/20/2018	51	1195	2	9.8	0	35.34
2 multi 2		H						
116	1	10/21/2018	52	1196	2	9.8	0	36.67
2 multi 2		H						
116	1	10/22/2018	53	1197	2	9.6	0	36.51

2 multi	2	H						
116	1	10/23/2018	54	1198	2	9.6	0	35.47
2 multi	2	H						
116	1	10/24/2018	55	1199	2	9.9	0	36.51
2 multi	2	H						
116	1	10/25/2018	56	1200	2	9.8	0.08	37.9
2 multi	2	H						
116	1	10/26/2018	57	1201	2	9.8	0	38.74
2 multi	2	H						
116	1	10/27/2018	58	1202	2	9.8	0	39.34
2 multi	2	H						
116	1	10/28/2018	59	1203	2	9.5	0.01	39
2 multi	2	H						
116	1	10/29/2018	60	1204	2	9.8	0	37.19
2 multi	2	H						
116	1	10/30/2018	61	1205	2	9.9	0.02	39.6
2 multi	2	H						
116	1	10/31/2018	62	1206	2	9.9	0	37.87
2 multi	2	H						
116	1	11/01/2018	63	1207	2	10	0.19	39.12
2 multi	2	H						
116	1	11/02/2018	64	1208	2	10	0	38.88
2 multi	2	H						
116	1	11/03/2018	65	1209	2	10	0.1	39.6
2 multi	2	H						
116	1	11/04/2018	66	1210	2	10.2	0	39.47
2 multi	2	H						
116	1	11/05/2018	67	1211	2	10.2	0	42.05
2 multi	2	H						
116	1	11/06/2018	68	1212	2	10.2	0	40.42
2 multi	2	H						
116	1	11/07/2018	69	1213	2	9.8	0	39.13
2 multi	2	H						
116	1	11/08/2018	70	1214	2	10.2	0	40.15
2 multi	2	H						
116	1	11/09/2018	71	1215	2	9.9	0	39.8
2 multi	2	H						
116	1	11/10/2018	72	1216	2	9.8	0	41.79
2 multi	2	H						
116	1	11/11/2018	73	1217	2	10.3	0.22	43.16
2 multi	2	H						
116	1	11/12/2018	74	1218	2	9.8	0	40.71
2 multi	2	H						
116	1	11/13/2018	75	1219	2	10	0	41.35
2 multi	2	H						
116	1	11/14/2018	76	1220	2	9.8	0	42.93
2 multi	2	H						
116	1	11/15/2018	77	1221	2	9.6	0	41.47
2 multi	2	H						
116	1	11/16/2018	78	1222	2	9.8	0	41.88
2 multi	2	H						
116	1	11/17/2018	79	1223	2	9.8	0	41.44
2 multi	2	H						
116	1	11/18/2018	80	1224	2	9.8	0	41.05

2 multi	2	H						
116	1	11/19/2018	81	1225	2	9.5	0.53	40.99
2 multi	2	H						
116	1	11/20/2018	82	1226	2	9.6	0	40.42
2 multi	2	H						
116	1	11/21/2018	83	1227	2	9.6	0	39.82
2 multi	2	H						
116	1	11/22/2018	84	1228	2	9.8	0	41.34
2 multi	2	H						
116	1	11/23/2018	85	1229	2	9.8	0	46.05
2 multi	2	H						
116	1	11/24/2018	86	1230	2	9.5	0	42.75
2 multi	2	H						
116	1	11/25/2018	87	1231	2	9.8	0	41.86
2 multi	2	H						
116	1	11/26/2018	88	1232	2	9.8	0	43.36
2 multi	2	H						
116	1	11/27/2018	89	1233	2	9.9	0	43.5
2 multi	2	H						
116	1	11/28/2018	90	1234	2	9.8	0	42.5
2 multi	2	H						
116	1	11/29/2018	91	1235	2	9.8	0.04	41.7
2 multi	2	H						
116	1	11/30/2018	92	1236	2	9.8	0.04	42.82
2 multi	2	H						
116	1	12/01/2018	93	1237	2	9.5	0.04	42.73
2 multi	2	H						
116	1	12/02/2018	94	1238	2	9.6	0	40.26
2 multi	2	H						
116	1	12/03/2018	95	1239	2	9.9	0	42.4
2 multi	2	H						
116	1	12/04/2018	96	1240	2	9.9	0	41.21
2 multi	2	H						
116	1	12/05/2018	97	1241	2	9.6	0	42.58
2 multi	2	H						
116	1	12/06/2018	98	1242	2	9.5	0	39.9
2 multi	2	H						
116	1	12/07/2018	99	1243	2	9.6	0	41.7
2 multi	2	H						
116	1	12/08/2018	100	1244	2	9.6	0.19	41.35
2 multi	2	H						
116	1	12/09/2018	101	1245	2	9.6	0	42.12
2 multi	2	H						
116	1	12/10/2018	102	1246	2	9.8	0	39.83
2 multi	2	H						
116	1	12/11/2018	103	1247	2	9.6	0	39.87
2 multi	2	H						
116	1	12/12/2018	104	1248	2	9.9	0	41.88
2 multi	2	H						
116	1	12/13/2018	105	1249	2	9.9	0	40.37
2 multi	2	H						
116	1	12/14/2018	106	1250	2	9.8	0	43.27
2 multi	2	H						
116	1	12/15/2018	107	1251	2	9.8	0	44.22

2 multi	2	H						
116	1	12/16/2018	108	1252	2	9.8	0	42.85
2 multi	2	H						
116	1	12/17/2018	109	1253	2	9.9	0	39.79
2 multi	2	H						
116	1	12/18/2018	110	1254	2	9.6	0	41.07
2 multi	2	H						
116	1	12/19/2018	111	1255	2	9.8	0	40.38
2 multi	2	H						
116	1	12/20/2018	112	1256	2	9.6	0	42.1
2 multi	2	H						
116	1	12/21/2018	113	1257	2	9.8	0.07	38.94
2 multi	2	H						
116	1	12/22/2018	114	1258	2	9.5	0	37.7
2 multi	2	H						
116	1	12/23/2018	115	1259	2	9.5	0.03	38.36
2 multi	2	H						
116	1	12/24/2018	116	1260	2	9.6	0.04	40.25
2 multi	2	H						
116	1	12/25/2018	117	1261	2	9.6	0.22	38.49
2 multi	2	H						
116	1	12/26/2018	118	1262	2	9.8	0	36.91
2 multi	2	H						
116	1	12/27/2018	119	1263	2	9.6	0	39.82
2 multi	2	H						
116	1	12/28/2018	120	1264	2	9.9	0	38.71
2 multi	2	H						
116	1	12/29/2018	121	1265	2	10	0	36.4
2 multi	2	H						
116	1	12/30/2018	122	1266	2	9.8	0	38.54
2 multi	2	H						
116	1	12/31/2018	123	1267	2	9.6	0	36.77
2 multi	2	H						
116	1	01/01/2019	124	1268	2	9.6	0	36.83
2 multi	2	H						
116	1	01/02/2019	125	1269	2	9.6	0	38.73
2 multi	2	H						
116	1	01/03/2019	126	1270	2	9.5	0	34.57
2 multi	2	H						
116	1	01/04/2019	127	1271	2	9.3	0	33.74
2 multi	2	H						
116	1	01/05/2019	128	1272	2	9.5	0	31.84
2 multi	2	H						
116	1	01/06/2019	129	1273	2	9.6	0	35.03
2 multi	2	H						
116	1	01/07/2019	130	1274	2	10	0	35.31
2 multi	2	H						
116	1	01/08/2019	131	1275	2	9.9	0	33.96
2 multi	2	H						
116	1	12/23/2019	4	1624	3	9.9	0.28	30.21
2 multi	1	H						
116	1	12/24/2019	5	1625	3	10.2	0.1	33.39
2 multi	1	H						
116	1	12/25/2019	6	1626	3	10	0.62	30.54

2 multi	1	H						
116	1	12/26/2019	7	1627	3	9.6	0.02	32.65
2 multi	1	H						
116	1	12/27/2019	8	1628	3	9.2	0.11	29.75
2 multi	1	H						
116	1	12/28/2019	9	1629	3	9.5	0.07	31.44
2 multi	1	H						
116	1	12/29/2019	10	1630	3	9.3	0.34	32.72
2 multi	1	H						
116	1	12/30/2019	11	1631	3	9.3	0	32.62
2 multi	1	H						
116	1	12/31/2019	12	1632	3	13.5	0.61	23.89
2 multi	1	H						
116	1	01/01/2020	13	1633	3	12.9	0.04	32.39
2 multi	1	H						
116	1	01/02/2020	14	1634	3	11.9	0.06	26.91
2 multi	1	H						
116	1	01/03/2020	15	1635	3	12.6	0.14	32.15
2 multi	1	H						
116	1	01/04/2020	16	1636	3	10.6	0.26	31.15
2 multi	1	H						
116	1	01/05/2020	17	1637	3	10.3	0.04	34.39
2 multi	1	H						
116	1	01/06/2020	18	1638	3	12.3	0.02	35.22
2 multi	1	H						
116	1	01/07/2020	19	1639	3	12.3	0.05	26.73
2 multi	1	H						
116	1	01/08/2020	20	1640	3	12	0.09	28.83
2 multi	1	H						
116	1	01/09/2020	21	1641	3	11.1	0.02	28.79
2 multi	1	H						
116	1	01/10/2020	22	1642	3	11.1	0.2	31.71
2 multi	2	H						
116	1	01/11/2020	23	1643	3	11.4	0.03	33.17
2 multi	2	H						
116	1	01/12/2020	24	1644	3	12	0.04	31.55
2 multi	2	H						
116	1	01/13/2020	25	1645	3	12	0.46	36.25
2 multi	2	H						
116	1	01/14/2020	26	1646	3	12	0.05	33.54
2 multi	2	H						
116	1	01/15/2020	27	1647	3	11.7	0.05	36.48
2 multi	2	H						
116	1	01/16/2020	28	1648	3	11.4	0.16	35.54
2 multi	2	H						
116	1	01/17/2020	29	1649	3	11.3	0.04	40.24
2 multi	2	H						
116	1	01/18/2020	30	1650	3	11.7	0.28	36.34
2 multi	2	H						
116	1	01/19/2020	31	1651	3	11.4	0.01	41.59
2 multi	2	H						
116	1	01/20/2020	32	1652	3	11.7	0	40.92
2 multi	2	H						
116	1	01/21/2020	33	1653	3	11.1	0.04	41.24

2 multi 2		H						
116	1	01/22/2020	34	1654	3	12.5	0.08	40.3
2 multi 2		H						
116	1	01/23/2020	35	1655	3	12	0.04	41.63
2 multi 2		H						
116	1	01/24/2020	36	1656	3	11.4	0.75	40.46
2 multi 2		H						
116	1	01/25/2020	37	1657	3	12.3	0.07	42.06
2 multi 2		H						
116	1	01/26/2020	38	1658	3	11.9	0.02	40.54
2 multi 2		H						
116	1	01/27/2020	39	1659	3	12.6	0.03	42.28
2 multi 2		H						
116	1	01/28/2020	40	1660	3	11.4	0.05	39.86
2 multi 2		H						
116	1	01/29/2020	41	1661	3	12.9	0.1	41.07
2 multi 2		H						
116	1	01/30/2020	42	1662	3	11.9	0.07	37.56
2 multi 2		H						
116	1	01/31/2020	43	1663	3	11.7	0.28	40.79
2 multi 2		H						
116	1	02/01/2020	44	1664	3	13.1	0.17	42.12
2 multi 2		H						
116	1	02/02/2020	45	1665	3	12	0.08	44.94
2 multi 2		H						
116	1	02/03/2020	46	1666	3	12.5	0.07	44.22
2 multi 2		H						
116	1	02/04/2020	47	1667	3	12	0.07	42.87
2 multi 2		H						
116	1	02/05/2020	48	1668	3	12	0.41	42.76
2 multi 2		H						
116	1	02/06/2020	49	1669	3	11.3	0.05	43.31
2 multi 2		H						
116	1	02/07/2020	50	1670	3	12.3	0	45.5
2 multi 2		H						
116	1	02/08/2020	51	1671	3	12	0.59	46.24
2 multi 2		H						
116	1	02/09/2020	52	1672	3	12.5	0.02	42.88
2 multi 2		H						
116	1	02/10/2020	53	1673	3	12.6	0	42.05
2 multi 2		H						
116	1	02/11/2020	54	1674	3	12.3	0.07	47.06
2 multi 2		H						
116	1	02/12/2020	55	1675	3	11.7	0.03	44.4
2 multi 2		H						
116	1	02/13/2020	56	1676	3	12	0	45.95
2 multi 2		H						
116	1	02/14/2020	57	1677	3	12.3	0.02	44.91
2 multi 2		H						
116	1	02/15/2020	58	1678	3	12.3	0.12	49
2 multi 2		H						
116	1	02/16/2020	59	1679	3	12.3	0.04	47.45
2 multi 2		H						
116	1	02/17/2020	60	1680	3	11.4	0.05	47.09

2 multi	2	H						
116	1	02/18/2020	61	1681	3	11.7	0.03	47.33
2 multi	2	H						
116	1	02/19/2020	62	1682	3	11.3	0	45.94
2 multi	2	H						
116	1	02/20/2020	63	1683	3	11.4	0.03	46.97
2 multi	2	H						
116	1	02/21/2020	64	1684	3	11.9	0.05	46.57
2 multi	2	H						
116	1	02/22/2020	65	1685	3	11.9	0.05	46.37
2 multi	2	H						
116	1	02/23/2020	66	1686	3	12.3	0.04	45.79
2 multi	2	H						
116	1	02/24/2020	67	1687	3	11.7	0	49.44
2 multi	2	H						
116	1	02/25/2020	68	1688	3	12.3	0.05	48.29
2 multi	2	H						
116	1	02/26/2020	69	1689	3	11.9	0	44.07
2 multi	2	H						
116	1	02/27/2020	70	1690	3	12.3	0.21	44.05
2 multi	2	H						
116	1	02/28/2020	71	1691	3	12	0.18	48.38
2 multi	2	H						
116	1	02/29/2020	72	1692	3	12.3	0.04	46.49
2 multi	2	H						
116	1	03/01/2020	73	1693	3	12	0.04	49.31
2 multi	2	H						
116	1	03/02/2020	74	1694	3	11.4	0.02	48.48
2 multi	2	H						
116	1	03/03/2020	75	1695	3	12.3	0	47.43
2 multi	2	H						
116	1	03/04/2020	76	1696	3	12.3	0.16	49.05
2 multi	2	H						
116	1	03/05/2020	77	1697	3	12.5	0.31	48.18
2 multi	2	H						
116	1	03/06/2020	78	1698	3	12.6	0.03	46.41
2 multi	2	H						
116	1	03/07/2020	79	1699	3	12.3	0.04	46.54
2 multi	2	H						
116	1	03/08/2020	80	1700	3	12.5	0.02	45.98
2 multi	2	H						
116	1	03/09/2020	81	1701	3	12.5	0.2	45.51
2 multi	2	H						
116	1	03/10/2020	82	1702	3	11.9	0.02	46
2 multi	2	H						
116	1	03/11/2020	83	1703	3	12.5	0.04	44.83
2 multi	2	H						
116	1	03/12/2020	84	1704	3	11.9	0	47.89
2 multi	2	H						
116	1	03/13/2020	85	1705	3	11.7	0	45.14
2 multi	2	H						
116	1	03/14/2020	86	1706	3	11.7	0.03	46.54
2 multi	2	H						
116	1	03/15/2020	87	1707	3	11.4	0.02	45.68

2 multi 2		H						
116	1	03/16/2020	88	1708	3	11.4	0.04	46.8
2 multi 2		H						
116	1	03/17/2020	89	1709	3	12.3	0.04	48.98
2 multi 2		H						
116	1	03/18/2020	90	1710	3	12.3	0.04	46.24
2 multi 2		H						
116	1	03/19/2020	91	1711	3	12.3	0.06	48.06
2 multi 2		H						
116	1	03/20/2020	92	1712	3	11.9	0.05	47.27
2 multi 2		H						
116	1	03/21/2020	93	1713	3	12	0.05	44.89
2 multi 2		H						
116	1	03/22/2020	94	1714	3	12.3	0	44.98
2 multi 2		H						
116	1	03/23/2020	95	1715	3	11.7	0.02	45.78
2 multi 2		H						
116	1	03/24/2020	96	1716	3	11.7	0.44	42.22
2 multi 2		H						
116	1	03/25/2020	97	1717	3	12.3	0.02	42.08
2 multi 2		H						
116	1	03/26/2020	98	1718	3	12.5	0.06	43.98
2 multi 2		H						
116	1	03/27/2020	99	1719	3	11.9	0	44.25
2 multi 2		H						
116	1	03/28/2020	100	1720	3	11.9	0.02	44.3
2 multi 2		H						
116	1	03/29/2020	101	1721	3	11.7	0.03	43.65
2 multi 2		H						
116	1	03/30/2020	102	1722	3	11.3	0.18	45.37
2 multi 2		H						
116	1	03/31/2020	103	1723	3	11.1	0	44.19
2 multi 2		H						
116	1	04/01/2020	104	1724	3	11.3	0.04	44.24
2 multi 2		H						
116	1	04/02/2020	105	1725	3	11.1	0	43.46
2 multi 2		H						
116	1	04/03/2020	106	1726	3	11.1	0.03	42.4
2 multi 2		H						
116	1	04/04/2020	107	1727	3	11.4	0.01	40.99
2 multi 2		H						
116	1	04/05/2020	108	1728	3	11.3	0	42.65
2 multi 2		H						
116	1	04/06/2020	109	1729	3	10.9	0.03	41.32
2 multi 2		H						
116	1	04/07/2020	110	1730	3	10.9	0	41.74
2 multi 2		H						
116	1	04/08/2020	111	1731	3	11.1	0	39.4
2 multi 2		H						
116	1	04/09/2020	112	1732	3	11.1	0.01	40.02
2 multi 2		H						
116	1	04/10/2020	113	1733	3	11.1	0.02	47.23
2 multi 2		H						
116	1	04/11/2020	114	1734	3	11.3	0	44.25

2 multi	2	H						
116	1	04/12/2020	115	1735	3	10.9	0.01	42.39
2 multi	2	H						
116	1	04/13/2020	116	1736	3	11.1	0	45.13
2 multi	2	H						
116	1	04/14/2020	117	1737	3	11.3	0	42.74
2 multi	2	H						
116	1	04/15/2020	118	1738	3	11.1	0.05	46.73
2 multi	2	H						
116	1	04/16/2020	119	1739	3	11.1	0	42.93
2 multi	2	H						
116	1	04/17/2020	120	1740	3	10.9	0.11	42.38
2 multi	2	H						
116	1	04/18/2020	121	1741	3	11.1	0	40.73
2 multi	2	H						
116	1	04/19/2020	122	1742	3	11.4	0	44.43
2 multi	2	H						
116	1	04/20/2020	123	1743	3	11.3	0.02	41.32
2 multi	2	H						
116	1	04/21/2020	124	1744	3	11.1	0.06	43.18
2 multi	2	H						
116	1	04/22/2020	125	1745	3	10.9	0.02	41.97
2 multi	2	H						
116	1	04/23/2020	126	1746	3	12	0	40.61
2 multi	2	H						
116	1	04/24/2020	127	1747	3	11.3	0.02	39.46
2 multi	2	H						
116	1	04/25/2020	128	1748	3	10.9	0.02	41.3
2 multi	2	H						
116	1	04/26/2020	129	1749	3	10.7	0	42.56
2 multi	2	H						
116	1	04/27/2020	130	1750	3	10.9	0.02	41.1
2 multi	2	H						
116	1	04/28/2020	131	1751	3	10.9	0	41.83
2 multi	2	H						
116	1	04/29/2020	132	1752	3	10.7	0	43.86
2 multi	2	H						
116	1	04/30/2020	133	1753	3	10.3	0.01	42.87
2 multi	2	H						
116	1	05/01/2020	134	1754	3	10.7	0.01	40.97
2 multi	2	H						
116	1	05/02/2020	135	1755	3	10.4	0.02	42.51
2 multi	2	H						
116	1	05/03/2020	136	1756	3	10.6	0	43.12
2 multi	2	H						
116	1	05/04/2020	137	1757	3	10.9	0.03	44.03
2 multi	2	H						
116	1	05/05/2020	138	1758	3	10.6	0	43.78
2 multi	2	H						
116	1	05/06/2020	139	1759	3	11.3	0.01	42.67
2 multi	2	H						
116	1	05/07/2020	140	1760	3	11.4	0	44.14
2 multi	2	H						
116	1	05/08/2020	141	1761	3	11.4	0	43.82

2 multi	2	H						
116	1	05/09/2020	142	1762	3	11.3	0.01	43.41
2 multi	2	H						
116	1	05/10/2020	143	1763	3	10.9	0	42.73
2 multi	2	H						
116	1	06/30/2020	194	1814	3	11.4	0	36.5
2 multi	2	H						
116	1	07/01/2020	195	1815	3	11.4	0	34.94
2 multi	2	H						
116	1	07/02/2020	196	1816	3	10.9	0	36.44
2 multi	2	H						
116	1	07/03/2020	197	1817	3	11.3	0	39.31
2 multi	2	H						
116	1	07/04/2020	198	1818	3	11.4	0.24	36.34
2 multi	2	H						
116	1	07/05/2020	199	1819	3	11.7	0.02	34.68
2 multi	2	H						
116	1	07/06/2020	200	1820	3	12	0.19	37.61
2 multi	2	H						
116	1	07/07/2020	201	1821	3	11.3	0.18	38.88
2 multi	2	H						
116	1	07/08/2020	202	1822	3	11.4	0.06	35.57
2 multi	2	H						
116	1	07/09/2020	203	1823	3	11.1	0.06	39.61
2 multi	2	H						
116	1	07/10/2020	204	1824	3	12	0.05	40
2 multi	2	H						
116	1	07/11/2020	205	1825	3	11.9	0	37.76
2 multi	2	H						
116	1	07/12/2020	206	1826	3	11.4	0.07	38.27
2 multi	2	H						
116	1	07/13/2020	207	1827	3	11.7	0.04	38.59
2 multi	2	H						
116	1	07/14/2020	208	1828	3	11.4	0	38.89
2 multi	2	H						
116	1	07/15/2020	209	1829	3	11.4	0.05	37.76
2 multi	2	H						
116	1	07/16/2020	210	1830	3	11.3	0.05	38.97
2 multi	2	H						
116	1	07/17/2020	211	1831	3	11.1	0	38.66
2 multi	2	H						
116	1	07/18/2020	212	1832	3	11.4	0.2	38.08
2 multi	2	H						
116	1	07/19/2020	213	1833	3	11.7	0.04	36.27
2 multi	2	H						
116	1	07/20/2020	214	1834	3	11.4	0.06	35.47
2 multi	2	H						
116	1	07/21/2020	215	1835	3	11.4	0	36.25
2 multi	2	H						
116	1	07/22/2020	216	1836	3	11.7	0.03	34.1
2 multi	2	H						
116	1	07/23/2020	217	1837	3	11.9	0.03	33.39
2 multi	2	H						
116	1	07/24/2020	218	1838	3	12.5	0	32.79

2 multi 2		H						
116	1	07/25/2020	219	1839	3	11.9	0.05	34.48
2 multi 2		H						
116	1	07/26/2020	220	1840	3	11.7	0	31.86
2 multi 2		H						
116	1	07/27/2020	221	1841	3	11.9	0.15	30.35
2 multi 2		H						
116	1	07/28/2020	222	1842	3	12	0.11	27.6
2 multi 2		H						
116	1	07/29/2020	223	1843	3	12.5	0.03	32.18
2 multi 2		H						
116	1	07/30/2020	224	1844	3	12	0.04	31.55
2 multi 2		H						
116	1	07/31/2020	225	1845	3	12.3	0	29.01
2 multi 2		H						
116	1	08/01/2020	226	1846	3	11.9	0.06	30.82
2 multi 2		H						
116	1	08/02/2020	227	1847	3	11.7	0.07	29.87
2 multi 2		H						
116	1	08/03/2020	228	1848	3	11.9	0	30.54
2 multi 2		H						
116	1	08/04/2020	229	1849	3	11.7	0.07	30.43
2 multi 2		H						
116	1	08/05/2020	230	1850	3	11.7	0	32.63
2 multi 2		H						
116	1	08/06/2020	231	1851	3	11.7	0.07	34.7
2 multi 2		H						
116	1	08/07/2020	232	1852	3	12.6	0.07	32.95
2 multi 2		H						
116	1	08/08/2020	233	1853	3	12.9	0.03	30.2
2 multi 2		H						
116	1	08/09/2020	234	1854	3	11.9	0	30.55
2 multi 2		H						
117	1	03/31/2020	4	867	1	10.2	0.23	20.1
1 primi 1		H						
117	1	04/01/2020	5	868	1	10.3	0.28	21.43
1 primi 1		H						
117	1	04/02/2020	6	869	1	10.3	0.09	22.34
1 primi 1		H						
117	1	04/03/2020	7	870	1	9.9	0.16	25.64
1 primi 1		H						
117	1	04/04/2020	8	871	1	9.9	0.07	27.51
1 primi 1		H						
117	1	04/05/2020	9	872	1	10	0.02	28.57
1 primi 1		H						
117	1	04/06/2020	10	873	1	10	0.33	31.02
1 primi 1		H						
117	1	04/07/2020	11	874	1	10	0.02	30.92
1 primi 1		H						
117	1	04/08/2020	12	875	1	10	0	30.23
1 primi 1		H						
117	1	04/09/2020	13	876	1	9.9	0	30.94
1 primi 1		H						
117	1	04/10/2020	14	877	1	9.9	0.09	33.54

1 primi	1	H						
117	1	04/11/2020	15	878	1	9.9	0	32.53
1 primi	1	H						
117	1	04/12/2020	16	879	1	9.9	0.09	34.57
1 primi	1	H						
117	1	04/13/2020	17	880	1	10	0.48	36.49
1 primi	1	H						
117	1	04/14/2020	18	881	1	10	0	37.75
1 primi	1	H						
117	1	04/15/2020	19	882	1	10.3	0.08	35.11
1 primi	1	H						
117	1	04/16/2020	20	883	1	10.3	0.11	39.36
1 primi	1	H						
117	1	04/17/2020	21	884	1	10.3	0.21	38.36
1 primi	1	H						
117	1	04/18/2020	22	885	1	10	0.2	38.75
1 primi	2	H						
117	1	04/19/2020	23	886	1	10	0.31	38.2
1 primi	2	H						
117	1	04/20/2020	24	887	1	10	0.28	40.18
1 primi	2	H						
117	1	04/21/2020	25	888	1	10	0.19	40.01
1 primi	2	H						
117	1	04/22/2020	26	889	1	10	0.18	41.47
1 primi	2	H						
117	1	04/23/2020	27	890	1	10	0	39.72
1 primi	2	H						
117	1	04/24/2020	28	891	1	9.6	0.32	37.9
1 primi	2	H						
117	1	04/25/2020	29	892	1	10.2	0.31	38.43
1 primi	2	H						
117	1	04/26/2020	30	893	1	9.6	0.18	39.61
1 primi	2	H						
117	1	04/27/2020	31	894	1	9.9	0	39.22
1 primi	2	H						
117	1	04/28/2020	32	895	1	9.8	0.26	39.25
1 primi	2	H						
117	1	04/29/2020	33	896	1	10	0.06	39.52
1 primi	2	H						
117	1	04/30/2020	34	897	1	10	0	40.32
1 primi	2	H						
117	1	05/01/2020	35	898	1	9.8	0.26	39.81
1 primi	2	H						
117	1	05/02/2020	36	899	1	9.9	0	38.71
1 primi	2	H						
117	1	05/03/2020	37	900	1	10	0	40.87
1 primi	2	H						
117	1	05/04/2020	38	901	1	10	0.26	40.04
1 primi	2	H						
117	1	05/05/2020	39	902	1	10	0.14	39.67
1 primi	2	H						
117	1	05/06/2020	40	903	1	9.8	0.2	39.26
1 primi	2	H						
117	1	05/07/2020	41	904	1	10.2	0.22	41.04

1 primi	2	H						
117	1	05/08/2020	42	905	1	10.2	0.24	40.96
1 primi	2	H						
117	1	05/09/2020	43	906	1	10	0.11	39.38
1 primi	2	H						
117	1	05/10/2020	44	907	1	10	0.02	40.7
1 primi	2	H						
117	1	05/11/2020	45	908	1	9.9	0	39.04
1 primi	2	H						
117	1	05/12/2020	46	909	1	10	0.26	38.27
1 primi	2	H						
117	1	05/13/2020	47	910	1	9.9	0.02	39.83
1 primi	2	H						
117	1	10/14/2020	201	1064	1	9.8	0.13	38.04
1 primi	2	H						
117	1	10/15/2020	202	1065	1	9.5	0.22	39.56
1 primi	2	H						
117	1	10/16/2020	203	1066	1	10	0.07	41.58
1 primi	2	H						
117	1	10/17/2020	204	1067	1	9.6	0.59	39.67
1 primi	2	H						
117	1	10/18/2020	205	1068	1	10	0.1	41.21
1 primi	2	H						
117	1	10/19/2020	206	1069	1	9.8	0.08	41.36
1 primi	2	H						
117	1	10/20/2020	207	1070	1	9.5	0.15	40.03
1 primi	2	H						
117	1	10/21/2020	208	1071	1	9.8	0.15	42.27
1 primi	2	H						
117	1	10/22/2020	209	1072	1	9.8	0.49	40.98
1 primi	2	H						
117	1	10/23/2020	210	1073	1	9.8	0.08	42.22
1 primi	2	H						
117	1	10/24/2020	211	1074	1	9.6	0.06	37.95
1 primi	2	H						
117	1	10/25/2020	212	1075	1	9.6	0.15	40.15
1 primi	2	H						
117	1	10/26/2020	213	1076	1	9.5	0.23	37.82
1 primi	2	H						
117	1	10/27/2020	214	1077	1	9.9	0.05	37.24
1 primi	2	H						
117	1	10/28/2020	215	1078	1	9.8	0.14	36.75
1 primi	2	H						
117	1	10/29/2020	216	1079	1	9.9	0.07	36.85
1 primi	2	H						
117	1	10/30/2020	217	1080	1	9.9	0.15	37.59
1 primi	2	H						
117	1	10/31/2020	218	1081	1	9.9	0.08	37.18
1 primi	2	H						
117	1	11/01/2020	219	1082	1	9.8	0.14	36.06
1 primi	2	H						
117	1	11/02/2020	220	1083	1	9.9	0.11	36.44
1 primi	2	H						
117	1	11/03/2020	221	1084	1	9.8	0.08	35.4

1 primi	2	H						
117	1	11/04/2020	222	1085	1	9.9	0.19	37.94
1 primi	2	H						
120	1	06/10/2019	5	701	1	8.9	0.19	25.81
1 primi	1	H						
120	1	06/11/2019	6	702	1	9.2	0.17	25.94
1 primi	1	H						
120	1	06/12/2019	7	703	1	9.6	0	26.51
1 primi	1	H						
120	1	06/13/2019	8	704	1	9.8	0	27.69
1 primi	1	H						
120	1	06/14/2019	9	705	1	11.3	0	29.82
1 primi	1	H						
120	1	06/15/2019	10	706	1	10.7	0	26.7
1 primi	1	H						
120	1	06/16/2019	11	707	1	10.2	0	32.05
1 primi	1	H						
120	1	06/17/2019	12	708	1	9.8	0.09	30.82
1 primi	1	H						
120	1	06/18/2019	13	709	1	9.3	0.24	29.75
1 primi	1	H						
120	1	06/19/2019	14	710	1	10	0	31.13
1 primi	1	H						
120	1	06/20/2019	15	711	1	9.9	0	32.54
1 primi	1	H						
120	1	06/21/2019	16	712	1	9.8	0	32.08
1 primi	1	H						
120	1	06/22/2019	17	713	1	9.8	0	31.94
1 primi	1	H						
120	1	06/23/2019	18	714	1	9.8	0.2	33.82
1 primi	1	H						
120	1	06/24/2019	19	715	1	9.9	0	33.61
1 primi	1	H						
120	1	06/25/2019	20	716	1	9.6	0	35.14
1 primi	1	H						
120	1	06/26/2019	21	717	1	9.9	0.16	36.03
1 primi	1	H						
120	1	06/27/2019	22	718	1	9.6	0	35.14
1 primi	2	H						
120	1	06/28/2019	23	719	1	9.5	0.09	35.31
1 primi	2	H						
120	1	06/29/2019	24	720	1	9.9	0.28	34.43
1 primi	2	H						
120	1	06/30/2019	25	721	1	9.6	0	37.36
1 primi	2	H						
120	1	07/01/2019	26	722	1	9.5	0	36.73
1 primi	2	H						
120	1	07/02/2019	27	723	1	9.6	0	36.61
1 primi	2	H						
120	1	07/03/2019	28	724	1	9.2	0	37.37
1 primi	2	H						
120	1	07/04/2019	29	725	1	9.5	0	36.42
1 primi	2	H						
120	1	07/05/2019	30	726	1	9.3	0	36.9

1 primi 2 H
120 1 07/06/2019 31 727 1 8.8 0 37
1 primi 2 H
120 1 07/07/2019 32 728 1 9.2 0 35.84
1 primi 2 H
120 1 07/08/2019 33 729 1 9.2 0 36.38
1 primi 2 H
120 1 07/09/2019 34 730 1 9.1 0.04 35.42
1 primi 2 H
120 1 07/10/2019 35 731 1 9.5 0 36.25
1 primi 2 H
120 1 07/11/2019 36 732 1 8.8 0.01 37.68
1 primi 2 H
120 1 07/12/2019 37 733 1 9.2 0.01 36.6
1 primi 2 H
120 1 07/13/2019 38 734 1 9.1 0.03 37.49
1 primi 2 H
120 1 07/14/2019 39 735 1 8.9 0.15 37.52
1 primi 2 H
120 1 07/15/2019 40 736 1 9.2 0.14 37.13
1 primi 2 H
120 1 07/16/2019 41 737 1 8.8 0 37.61
1 primi 2 H
120 1 07/17/2019 42 738 1 9.2 0.27 36.32
1 primi 2 H
120 1 07/18/2019 43 739 1 8.9 0 40.84
1 primi 2 H
120 1 07/19/2019 44 740 1 9.3 0.14 39.13
1 primi 2 H
120 1 07/20/2019 45 741 1 8.7 0 39.88
1 primi 2 H
120 1 07/21/2019 46 742 1 8.9 0 41.85
1 primi 2 H
120 1 07/22/2019 47 743 1 9.1 0 40.15
1 primi 2 H
120 1 07/23/2019 48 744 1 8.9 0 41.71
1 primi 2 H
120 1 07/24/2019 49 745 1 8.8 0 38.61
1 primi 2 H
120 1 07/25/2019 50 746 1 9.1 0 36.07
1 primi 2 H
120 1 07/26/2019 51 747 1 9.1 0 41.51
1 primi 2 H
120 1 07/27/2019 52 748 1 9.2 0 39.09
1 primi 2 H
120 1 07/28/2019 53 749 1 9.2 0 39.37
1 primi 2 H
120 1 07/29/2019 54 750 1 9.1 0 39.67
1 primi 2 H
120 1 07/30/2019 55 751 1 8.9 0 39.04
1 primi 2 H
120 1 07/31/2019 56 752 1 8.9 0 40.8
1 primi 2 H
120 1 08/01/2019 57 753 1 8.9 0 39.02

1 primi 2 H
120 1 08/02/2019 58 754 1 8.9 0.12 38.7
1 primi 2 H
120 1 08/03/2019 59 755 1 8.9 0 39.72
1 primi 2 H
120 1 08/04/2019 60 756 1 8.9 0 38.69
1 primi 2 H
120 1 08/05/2019 61 757 1 8.8 0.25 40.29
1 primi 2 H
120 1 08/06/2019 62 758 1 9.1 0 39.53
1 primi 2 H
120 1 08/07/2019 63 759 1 8.7 0.01 38.27
1 primi 2 H
120 1 08/08/2019 64 760 1 8.9 0 37.97
1 primi 2 H
120 1 08/09/2019 65 761 1 9.2 0 39.69
1 primi 2 H
120 1 08/10/2019 66 762 1 8.9 0 37.99
1 primi 2 H
120 1 08/11/2019 67 763 1 9.1 0 38.22
1 primi 2 H
120 1 08/12/2019 68 764 1 9.3 0.01 39.98
1 primi 2 H
120 1 08/13/2019 69 765 1 9.1 0.23 34.26
1 primi 2 H
120 1 08/14/2019 70 766 1 8.9 0.28 37.45
1 primi 2 H
120 1 08/15/2019 71 767 1 8.7 0.2 37.17
1 primi 2 H
120 1 08/16/2019 72 768 1 8.9 0.14 36.92
1 primi 2 H
120 1 08/17/2019 73 769 1 8.6 0 37.29
1 primi 2 H
120 1 08/18/2019 74 770 1 8.9 0 37.07
1 primi 2 H
120 1 08/19/2019 75 771 1 9.1 0.02 37.26
1 primi 2 H
120 1 08/20/2019 76 772 1 8.8 0 36.64
1 primi 2 H
120 1 08/21/2019 77 773 1 8.7 0.01 34.32
1 primi 2 H
120 1 08/22/2019 78 774 1 8.7 0.01 34.04
1 primi 2 H
120 1 08/23/2019 79 775 1 8.9 0.08 33.87
1 primi 2 H
120 1 08/24/2019 80 776 1 8.7 0.01 35.65
1 primi 2 H
120 1 08/25/2019 81 777 1 8.8 0 34.39
1 primi 2 H
120 1 08/26/2019 82 778 1 8.8 0.27 33.64
1 primi 2 H
120 1 08/27/2019 83 779 1 9.1 0 37.68
1 primi 2 H
120 1 08/28/2019 84 780 1 9.2 0 33.2

1 primi 2		H						
120	1	08/29/2019	85	781	1	9.2	0	35.61
1 primi 2		H						
120	1	08/30/2019	86	782	1	9.1	0	33.2
1 primi 2		H						
120	1	08/31/2019	87	783	1	9.2	0.27	37.44
1 primi 2		H						
120	1	09/01/2019	88	784	1	9.3	0	36.51
1 primi 2		H						
120	1	09/02/2019	89	785	1	9.9	0.05	31.13
1 primi 2		H						
120	1	09/03/2019	90	786	1	9.3	0.05	32.29
1 primi 2		H						
120	1	09/04/2019	91	787	1	9.6	0.18	38.21
1 primi 2		H						
120	1	09/05/2019	92	788	1	9.2	0.01	36.17
1 primi 2		H						
120	1	09/06/2019	93	789	1	8.9	0.04	31.71
1 primi 2		H						
120	1	09/07/2019	94	790	1	8.5	0.02	34.63
1 primi 2		H						
120	1	09/08/2019	95	791	1	8.7	0.16	33.67
1 primi 2		H						
120	1	09/09/2019	96	792	1	8.5	0	33.04
1 primi 2		H						
120	1	09/10/2019	97	793	1	8.6	0	30.71
1 primi 2		H						
120	1	09/11/2019	98	794	1	8.6	0.06	32.99
1 primi 2		H						
120	1	09/12/2019	99	795	1	8.7	0.01	33.61
1 primi 2		H						
120	1	09/13/2019	100	796	1	8.6	0	34.35
1 primi 2		H						
120	1	09/14/2019	101	797	1	9.2	0	33.28
1 primi 2		H						
120	1	09/15/2019	102	798	1	9.1	0.19	34.34
1 primi 2		H						
120	1	09/16/2019	103	799	1	8.9	0.01	33.71
1 primi 2		H						
120	1	09/17/2019	104	800	1	10.9	0.1	31.11
1 primi 2		H						
120	1	09/18/2019	105	801	1	9.1	0.08	35.13
1 primi 2		H						
120	1	09/19/2019	106	802	1	9.2	0.24	37.04
1 primi 2		H						
120	1	09/20/2019	107	803	1	9.2	0.23	35.87
1 primi 2		H						
120	1	09/21/2019	108	804	1	9.1	0	37.61
1 primi 2		H						
120	1	09/22/2019	109	805	1	9.1	0	38.09
1 primi 2		H						
120	1	09/23/2019	110	806	1	8.8	0.02	36.45
1 primi 2		H						
120	1	09/24/2019	111	807	1	8.9	0.02	37.08

1 primi 2 H
120 1 09/25/2019 112 808 1 8.9 0.18 38.83
1 primi 2 H
120 1 09/26/2019 113 809 1 8.8 0.01 37.83
1 primi 2 H
120 1 09/27/2019 114 810 1 8.7 0.24 38.31
1 primi 2 H
120 1 09/28/2019 115 811 1 8.7 0 37.11
1 primi 2 H
120 1 09/29/2019 116 812 1 8.6 0.06 37.97
1 primi 2 H
120 1 09/30/2019 117 813 1 8.6 0 20.8
1 primi 2 H
120 1 10/01/2019 118 814 1 9.2 0.01 38.98
1 primi 2 H
120 1 10/02/2019 119 815 1 8.8 0.28 37.56
1 primi 2 H
120 1 10/03/2019 120 816 1 9.2 0 38.42
1 primi 2 H
120 1 10/04/2019 121 817 1 8.8 0.01 35.38
1 primi 2 H
120 1 10/05/2019 122 818 1 8.8 0 38.51
1 primi 2 H
120 1 10/06/2019 123 819 1 8.8 0.05 38.05
1 primi 2 H
120 1 10/07/2019 124 820 1 8.8 0.09 38.54
1 primi 2 H
120 1 10/08/2019 125 821 1 8.8 0 38.4
1 primi 2 H
120 1 10/09/2019 126 822 1 8.6 0.08 38.19
1 primi 2 H
120 1 10/10/2019 127 823 1 9.1 0 39.71
1 primi 2 H
120 1 10/11/2019 128 824 1 8.6 0.25 38.7
1 primi 2 H
120 1 10/12/2019 129 825 1 8.7 0 36.75
1 primi 2 H
120 1 10/13/2019 130 826 1 8.7 0 40.45
1 primi 2 H
120 1 10/14/2019 131 827 1 8.8 0.12 38.13
1 primi 2 H
120 1 10/15/2019 132 828 1 9.1 0 42.45
1 primi 2 H
120 1 10/16/2019 133 829 1 9.1 0 39.45
1 primi 2 H
120 1 10/17/2019 134 830 1 8.6 0 37.91
1 primi 2 H
120 1 10/18/2019 135 831 1 8.5 0 36.5
1 primi 2 H
120 1 10/19/2019 136 832 1 8.7 0.02 37.18
1 primi 2 H
120 1 10/20/2019 137 833 1 8.9 0 40.03
1 primi 2 H
120 1 10/21/2019 138 834 1 8.7 0 39.88

1 primi 2 H
120 1 10/22/2019 139 835 1 8.7 0 39.15
1 primi 2 H
120 1 10/23/2019 140 836 1 8.9 0 37.62
1 primi 2 H
120 1 10/24/2019 141 837 1 8.9 0 38.53
1 primi 2 H
120 1 10/25/2019 142 838 1 8.7 0.15 37.4
1 primi 2 H
120 1 10/26/2019 143 839 1 8.5 0.28 38.56
1 primi 2 H
120 1 10/27/2019 144 840 1 8.8 0.11 39.95
1 primi 2 H
120 1 10/28/2019 145 841 1 8.8 0.11 38.43
1 primi 2 H
120 1 10/29/2019 146 842 1 8.7 0 39.04
1 primi 2 H
120 1 10/30/2019 147 843 1 8.6 0 37.26
1 primi 2 H
120 1 10/31/2019 148 844 1 8.9 0 39.03
1 primi 2 H
120 1 11/01/2019 149 845 1 8.6 0.01 37.62
1 primi 2 H
120 1 11/02/2019 150 846 1 8.5 0 37
1 primi 2 H
120 1 11/03/2019 151 847 1 8.7 0.77 37.15
1 primi 2 H
120 1 11/04/2019 152 848 1 8.9 0.11 37.17
1 primi 2 H
120 1 11/05/2019 153 849 1 8.8 0.09 37.35
1 primi 2 H
120 1 11/06/2019 154 850 1 8.6 0 37.63
1 primi 2 H
120 1 09/07/2020 4 1156 2 9.9 0.27 28.67
2 multi 1 H
120 1 09/08/2020 5 1157 2 9.8 0.24 33.81
2 multi 1 H
120 1 09/09/2020 6 1158 2 9.6 0.13 33.18
2 multi 1 H
120 1 09/10/2020 7 1159 2 9.8 0.23 36.25
2 multi 1 H
120 1 09/11/2020 8 1160 2 9.8 0.04 36.73
2 multi 1 H
120 1 09/12/2020 9 1161 2 9.5 0.01 36.44
2 multi 1 H
120 1 09/13/2020 10 1162 2 9.3 0.05 37.06
2 multi 1 H
120 1 09/14/2020 11 1163 2 9.6 0.02 36.3
2 multi 1 H
120 1 09/15/2020 12 1164 2 9.3 0.07 37.37
2 multi 1 H
120 1 09/16/2020 13 1165 2 9.5 0.02 42.03
2 multi 1 H
120 1 09/17/2020 14 1166 2 9.2 0.07 37.7

2 multi 1	H
120	1	09/18/2020	15	1167	2	9.5	0.1	40.13
2 multi 1	H
120	1	09/19/2020	16	1168	2	9.2	0.03	39.09
2 multi 1	H
120	1	09/20/2020	17	1169	2	9.5	0.02	40.82
2 multi 1	H
120	1	09/21/2020	18	1170	2	9.2	0.01	40.91
2 multi 1	H
120	1	09/22/2020	19	1171	2	9.6	0.26	43.45
2 multi 1	H
120	1	09/23/2020	20	1172	2	9.3	0.17	43.42
2 multi 1	H
120	1	09/24/2020	21	1173	2	9.6	0.01	41.5
2 multi 1	H
120	1	09/25/2020	22	1174	2	9.5	0.11	47.21
2 multi 2	H
120	1	09/26/2020	23	1175	2	9.6	0.02	46.27
2 multi 2	H
120	1	09/27/2020	24	1176	2	9.5	0.18	47.15
2 multi 2	H
120	1	09/28/2020	25	1177	2	9.5	0.04	47.2
2 multi 2	H
120	1	09/29/2020	26	1178	2	9.6	0.06	47.41
2 multi 2	H
120	1	09/30/2020	27	1179	2	9.5	0.17	47.18
2 multi 2	H
120	1	10/01/2020	28	1180	2	9.3	0.16	49.66
2 multi 2	H
120	1	10/02/2020	29	1181	2	9.3	0.08	45.05
2 multi 2	H
120	1	10/03/2020	30	1182	2	8.9	0.06	38.02
2 multi 2	H
120	1	10/04/2020	31	1183	2	9.5	0.02	44.35
2 multi 2	H
120	1	10/05/2020	32	1184	2	9.3	0.03	46.31
2 multi 2	H
120	1	10/06/2020	33	1185	2	9.5	0.07	45.81
2 multi 2	H
120	1	10/07/2020	34	1186	2	9.2	0.14	44.71
2 multi 2	H
120	1	10/08/2020	35	1187	2	9.3	0.02	46.68
2 multi 2	H
120	1	10/09/2020	36	1188	2	9.6	0.04	40.28
2 multi 2	H
120	1	10/10/2020	37	1189	2	9.5	0.13	48.34
2 multi 2	H
120	1	10/11/2020	38	1190	2	9.5	0.03	43.07
2 multi 2	H
120	1	10/12/2020	39	1191	2	9.1	0.2	50.21
2 multi 2	H
120	1	10/13/2020	40	1192	2	9.5	0.16	46.79
2 multi 2	H
122	1	03/31/2020	4	798	1	9.5	0.51	19.37

1 primi	1	H						
122	1	04/01/2020	5	799	1	9.9	0.2	22.98
1 primi	1	H						
122	1	04/02/2020	6	800	1	10	0.19	22.08
1 primi	1	H						
122	1	04/03/2020	7	801	1	10.6	0.13	26.28
1 primi	1	H						
122	1	04/04/2020	8	802	1	10.6	0.08	24.15
1 primi	1	H						
122	1	04/05/2020	9	803	1	10.2	0.41	27.32
1 primi	1	H						
122	1	04/06/2020	10	804	1	10.6	0.04	30.21
1 primi	1	H						
122	1	04/07/2020	11	805	1	10.7	0.11	27.36
1 primi	1	H						
122	1	04/08/2020	12	806	1	11.3	0.02	24.02
1 primi	1	H						
122	1	04/09/2020	13	807	1	10.2	0.01	28.13
1 primi	1	H						
122	1	04/10/2020	14	808	1	10.2	0.13	26.82
1 primi	1	H						
122	1	04/11/2020	15	809	1	9.1	0.06	24.46
1 primi	1	H						
122	1	04/12/2020	16	810	1	9.2	0.08	27.84
1 primi	1	H						
122	1	04/13/2020	17	811	1	10.2	0.04	28.73
1 primi	1	H						
122	1	04/14/2020	18	812	1	10.2	0	29.49
1 primi	1	H						
122	1	04/15/2020	19	813	1	10.2	0.05	29.93
1 primi	1	H						
122	1	04/16/2020	20	814	1	10	0.04	32.5
1 primi	1	H						
122	1	04/17/2020	21	815	1	9.9	0.02	30.53
1 primi	1	H						
122	1	04/18/2020	22	816	1	9.9	0	32.24
1 primi	2	H						
122	1	04/19/2020	23	817	1	9.9	0	34.59
1 primi	2	H						
122	1	04/20/2020	24	818	1	10	0.01	33.67
1 primi	2	H						
122	1	04/21/2020	25	819	1	9.8	0	32.58
1 primi	2	H						
122	1	04/22/2020	26	820	1	9.1	0.36	33.51
1 primi	2	H						
122	1	04/23/2020	27	821	1	9.9	0.03	34.53
1 primi	2	H						
122	1	04/24/2020	28	822	1	9.9	0	33.14
1 primi	2	H						
122	1	04/25/2020	29	823	1	9.6	0.03	33.81
1 primi	2	H						
122	1	04/26/2020	30	824	1	9.5	0.06	36.89
1 primi	2	H						
122	1	04/27/2020	31	825	1	9.8	0.03	35.68

1 primi	2	H						
122	1	04/28/2020	32	826	1	10	0.02	33.51
1 primi	2	H						
122	1	04/29/2020	33	827	1	10.2	0.01	36.78
1 primi	2	H						
122	1	04/30/2020	34	828	1	10.3	0.03	34.63
1 primi	2	H						
122	1	05/01/2020	35	829	1	9.9	0	32.04
1 primi	2	H						
122	1	05/02/2020	36	830	1	9.2	0.01	35.63
1 primi	2	H						
122	1	05/03/2020	37	831	1	9.9	0.3	35.46
1 primi	2	H						
122	1	05/04/2020	38	832	1	9.5	0	37.1
1 primi	2	H						
122	1	05/05/2020	39	833	1	9.1	0.04	36.34
1 primi	2	H						
122	1	05/06/2020	40	834	1	9.6	0.04	36.71
1 primi	2	H						
122	1	05/07/2020	41	835	1	9.9	0.04	34.76
1 primi	2	H						
122	1	05/08/2020	42	836	1	9.9	0.05	37.67
1 primi	2	H						
122	1	05/09/2020	43	837	1	9.1	0.18	35.02
1 primi	2	H						
122	1	05/10/2020	44	838	1	9.3	0.03	37.12
1 primi	2	H						
122	1	05/11/2020	45	839	1	9.2	0	35.81
1 primi	2	H						
122	1	05/12/2020	46	840	1	9.2	0.02	35.03
1 primi	2	H						
122	1	05/13/2020	47	841	1	9.2	0.02	36.49
1 primi	2	H						
122	1	05/14/2020	48	842	1	9.5	0	36.03
1 primi	2	H						
122	1	05/15/2020	49	843	1	9.3	0.01	31.35
1 primi	2	H						
122	1	05/16/2020	50	844	1	9.3	0.01	36.46
1 primi	2	H						
122	1	05/17/2020	51	845	1	9.3	0	34.51
1 primi	2	H						
122	1	05/18/2020	52	846	1	8.9	0	35.19
1 primi	2	H						
122	1	05/19/2020	53	847	1	9.9	0.48	32.58
1 primi	2	H						
122	1	05/20/2020	54	848	1	8.9	0	35.23
1 primi	2	H						
122	1	05/21/2020	55	849	1	9.5	0	36.36
1 primi	2	H						
123	1	02/06/2018	7	777	1	8.8	0.11	24.89
1 primi	1	H						
123	1	02/07/2018	8	778	1	9.2	0.23	23.16
1 primi	1	H						
123	1	02/08/2018	9	779	1	9.5	0.04	28.72

```
1 primi 1       H
123     1       02/09/2018      10      780     1       8.6     0.03    26.52
1 primi 1       H
123     1       02/10/2018      11      781     1       9.3     0.13    29.08
1 primi 1       H
123     1       02/11/2018      12      782     1       8.6     0.09    27.74
1 primi 1       H
123     1       02/12/2018      13      783     1       9.3     0.06    28.57
1 primi 1       H
123     1       02/13/2018      14      784     1       9.2     0       29.62
1 primi 1       H
123     1       02/14/2018      15      785     1       8.7     0.04    30.18
1 primi 1       H
123     1       02/15/2018      16      786     1       8.5     0.09    28.32
1 primi 1       H
123     1       02/16/2018      17      787     1       8.9     0.12    33.69
1 primi 1       H
123     1       02/17/2018      18      788     1       8.8     0.06    33.35
1 primi 1       H
123     1       02/18/2018      19      789     1       9.2     0.04    34.04
1 primi 1       H
123     1       02/19/2018      20      790     1       8.4     0.19    32.75
1 primi 1       H
123     1       02/20/2018      21      791     1       8.3     0.18    33.56
1 primi 1       H
123     1       02/21/2018      22      792     1       9.2     0.03    30.82
1 primi 2       H
123     1       02/22/2018      23      793     1       8.7     0.04    29.85
1 primi 2       H
123     1       02/23/2018      24      794     1       8.8     0.01    29.62
1 primi 2       H
123     1       02/24/2018      25      795     1       8.8     0.01    29.91
1 primi 2       H
123     1       02/25/2018      26      796     1       8.6     0       29.89
1 primi 2       H
123     1       02/26/2018      27      797     1       9.5     0       33.52
1 primi 2       H
123     1       02/27/2018      28      798     1       9.1     0.01    32.28
1 primi 2       H
123     1       02/28/2018      29      799     1       9.1     0.02    33.26
1 primi 2       H
123     1       03/01/2018      30      800     1       9.1     0       34.62
1 primi 2       H
123     1       03/02/2018      31      801     1       9.2     0.01    35.6
1 primi 2       H
123     1       03/03/2018      32      802     1       8.9     0       38.89
1 primi 2       H
123     1       03/04/2018      33      803     1       9.3     0       38.07
1 primi 2       H
123     1       03/05/2018      34      804     1       9.1     0       38.53
1 primi 2       H
123     1       03/06/2018      35      805     1       9.2     0       38.87
1 primi 2       H
123     1       03/07/2018      36      806     1       8.8     0.02    38.95
```

1 primi	2	H						
123	1	03/08/2018	37	807	1	8.9	0	38.5
1 primi	2	H						
123	1	03/09/2018	38	808	1	8.7	0.02	35.17
1 primi	2	H						
123	1	03/10/2018	39	809	1	9.3	0.01	38.03
1 primi	2	H						
123	1	03/11/2018	40	810	1	9.3	0.01	37.52
1 primi	2	H						
123	1	03/12/2018	41	811	1	9.2	0	33.57
1 primi	2	H						
123	1	03/13/2018	42	812	1	9.5	0.2	38.75
1 primi	2	H						
123	1	03/14/2018	43	813	1	8.8	0.05	36.95
1 primi	2	H						
123	1	03/15/2018	44	814	1	9.2	0	39.54
1 primi	2	H						
123	1	03/16/2018	45	815	1	9.1	0.01	41.4
1 primi	2	H						
123	1	03/17/2018	46	816	1	9.3	0.08	40.25
1 primi	2	H						
123	1	03/18/2018	47	817	1	9.9	0	37.25
1 primi	2	H						
123	1	09/13/2019	3	1361	2	9.3	0.25	33.71
2 multi	1	H						
123	1	09/14/2019	4	1362	2	10	0.13	37.18
2 multi	1	H						
123	1	09/15/2019	5	1363	2	9.5	0.12	38.6
2 multi	1	H						
123	1	09/16/2019	6	1364	2	9.5	0.08	36.36
2 multi	1	H						
123	1	09/17/2019	7	1365	2	9.9	0.21	41.77
2 multi	1	H						
123	1	09/18/2019	8	1366	2	9.3	0.19	39.65
2 multi	1	H						
123	1	09/19/2019	9	1367	2	9.6	0.06	41.93
2 multi	1	H						
123	1	09/20/2019	10	1368	2	9.6	0.32	40.02
2 multi	1	H						
123	1	09/21/2019	11	1369	2	9.6	0.03	44.13
2 multi	1	H						
123	1	09/22/2019	12	1370	2	9.6	0.12	44.87
2 multi	1	H						
123	1	09/23/2019	13	1371	2	9.9	0.06	45.95
2 multi	1	H						
123	1	09/24/2019	14	1372	2	9.6	0.11	43.18
2 multi	1	H						
123	1	09/25/2019	15	1373	2	9.8	0	46.46
2 multi	1	H						
123	1	09/26/2019	16	1374	2	9.5	0.03	44.33
2 multi	1	H						
123	1	09/27/2019	17	1375	2	9.5	0.08	45.45
2 multi	1	H						
123	1	09/28/2019	18	1376	2	9.3	0.2	43.42

2 multi	1	H						
123	1	09/29/2019	19	1377	2	8.8	0.26	44.11
2 multi	1	H						
123	1	09/30/2019	20	1378	2	9.1	0	45.75
2 multi	1	H						
123	1	10/01/2019	21	1379	2	9.3	0.03	50.77
2 multi	1	H						
123	1	10/02/2019	22	1380	2	9.3	0	47.15
2 multi	2	H						
123	1	10/03/2019	23	1381	2	9.2	0.26	48.28
2 multi	2	H						
123	1	10/04/2019	24	1382	2	9.2	1.93	47.89
2 multi	2	H						
123	1	10/05/2019	25	1383	2	10	0.05	44.41
2 multi	2	H						
123	1	10/06/2019	26	1384	2	9.3	0.28	46.92
2 multi	2	H						
123	1	10/07/2019	27	1385	2	9.2	0.14	51.37
2 multi	2	H						
123	1	10/08/2019	28	1386	2	9.3	0	47.85
2 multi	2	H						
123	1	10/09/2019	29	1387	2	9.5	0.14	46
2 multi	2	H						
123	1	10/10/2019	30	1388	2	8.8	0.21	44.54
2 multi	2	H						
123	1	10/11/2019	31	1389	2	9.5	0	45.49
2 multi	2	H						
123	1	10/12/2019	32	1390	2	10.2	0.28	47.94
2 multi	2	H						
123	1	10/13/2019	33	1391	2	10	0.28	48.36
2 multi	2	H						
123	1	10/14/2019	34	1392	2	10.4	0.18	46.71
2 multi	2	H						
123	1	10/15/2019	35	1393	2	10	0.1	48.55
2 multi	2	H						
123	1	10/16/2019	36	1394	2	10.3	0.05	50.62
2 multi	2	H						
123	1	10/17/2019	37	1395	2	9.9	0.42	50.69
2 multi	2	H						
123	1	10/18/2019	38	1396	2	10.7	0.12	45.41
2 multi	2	H						
123	1	10/19/2019	39	1397	2	10.2	0	43.5
2 multi	2	H						
123	1	10/20/2019	40	1398	2	10.3	0.05	44.44
2 multi	2	H						
123	1	10/21/2019	41	1399	2	9.5	0.06	47.27
2 multi	2	H						
123	1	10/22/2019	42	1400	2	10	0.06	45.75
2 multi	2	H						
123	1	10/23/2019	43	1401	2	10.4	0.07	48.88
2 multi	2	H						
123	1	10/24/2019	44	1402	2	9.3	0.07	45.36
2 multi	2	H						
123	1	10/25/2019	45	1403	2	11.3	0	45.67

2 multi	2	H						
123	1	10/26/2019	46	1404	2	10.3	0.21	44.21
2 multi	2	H						
123	1	10/27/2019	47	1405	2	10.2	0.32	47.1
2 multi	2	H						
123	1	10/28/2019	48	1406	2	10.2	0.3	47
2 multi	2	H						
123	1	10/29/2019	49	1407	2	10.2	0.02	45.11
2 multi	2	H						
123	1	10/30/2019	50	1408	2	9.8	0.06	49.64
2 multi	2	H						
123	1	10/31/2019	51	1409	2	10.4	0.41	47.41
2 multi	2	H						
123	1	11/01/2019	52	1410	2	9.9	0.05	41.91
2 multi	2	H						
123	1	11/02/2019	53	1411	2	9.9	0.03	45.3
2 multi	2	H						
123	1	11/03/2019	54	1412	2	10.2	0	48.6
2 multi	2	H						
123	1	11/04/2019	55	1413	2	10.4	0.11	44.39
2 multi	2	H						
123	1	11/05/2019	56	1414	2	10.2	0.18	47.65
2 multi	2	H						
123	1	11/06/2019	57	1415	2	10	0	43.56
2 multi	2	H						
123	1	11/07/2019	58	1416	2	9.9	0.04	47.26
2 multi	2	H						
123	1	11/08/2019	59	1417	2	10.3	0.03	46.69
2 multi	2	H						
123	1	11/09/2019	60	1418	2	10.3	0	42.78
2 multi	2	H						
123	1	11/10/2019	61	1419	2	9.8	0	43.49
2 multi	2	H						
123	1	11/11/2019	62	1420	2	10.9	0	45.83
2 multi	2	H						
123	1	11/12/2019	63	1421	2	11.1	0	41.76
2 multi	2	H						
123	1	11/13/2019	64	1422	2	10.2	0.11	39.99
2 multi	2	H						
123	1	11/14/2019	65	1423	2	10	0.06	48.21
2 multi	2	H						
123	1	11/15/2019	66	1424	2	10.7	0.04	42.04
2 multi	2	H						
123	1	11/16/2019	67	1425	2	9.9	0.15	43.57
2 multi	2	H						
123	1	11/17/2019	68	1426	2	10	0.05	43.09
2 multi	2	H						
123	1	11/18/2019	69	1427	2	10.2	0	43.63
2 multi	2	H						
123	1	11/19/2019	70	1428	2	9.9	0	43.64
2 multi	2	H						
123	1	11/20/2019	71	1429	2	10.3	0.01	44.84
2 multi	2	H						
123	1	11/21/2019	72	1430	2	10.3	0.02	43.73

2 multi 2		H						
123	1	11/22/2019	73	1431	2	10.2	0.24	46.19
2 multi 2		H						
123	1	11/23/2019	74	1432	2	10	0.21	44.97
2 multi 2		H						
123	1	11/24/2019	75	1433	2	9.8	0.11	45.11
2 multi 2		H						
123	1	11/25/2019	76	1434	2	10.4	0	43.45
2 multi 2		H						
123	1	11/26/2019	77	1435	2	10.2	0.02	44.38
2 multi 2		H						
123	1	11/27/2019	78	1436	2	10.6	0	43.69
2 multi 2		H						
123	1	11/28/2019	79	1437	2	10.6	0	45.62
2 multi 2		H						
123	1	11/29/2019	80	1438	2	10.6	0.04	45.86
2 multi 2		H						
123	1	11/30/2019	81	1439	2	10.4	0.03	43.99
2 multi 2		H						
123	1	12/01/2019	82	1440	2	10.2	0.28	47.71
2 multi 2		H						
123	1	12/02/2019	83	1441	2	9.9	0.45	44.78
2 multi 2		H						
123	1	12/03/2019	84	1442	2	10.6	0.04	47.21
2 multi 2		H						
123	1	12/04/2019	85	1443	2	10.3	0.02	47.7
2 multi 2		H						
123	1	12/05/2019	86	1444	2	10.3	0.04	46.55
2 multi 2		H						
123	1	12/06/2019	87	1445	2	10.2	0.01	46.18
2 multi 2		H						
123	1	12/07/2019	88	1446	2	10.2	0.06	45.01
2 multi 2		H						
123	1	12/08/2019	89	1447	2	10.3	0.02	44.64
2 multi 2		H						
123	1	12/09/2019	90	1448	2	12	0.01	48.11
2 multi 2		H						
123	1	12/10/2019	91	1449	2	10.3	0.03	46.35
2 multi 2		H						
123	1	12/11/2019	92	1450	2	10.9	0	45.91
2 multi 2		H						
123	1	12/12/2019	93	1451	2	10.9	0.02	42
2 multi 2		H						
123	1	12/13/2019	94	1452	2	10.4	0.03	40.14
2 multi 2		H						
123	1	12/14/2019	95	1453	2	10.6	0.04	40
2 multi 2		H						
123	1	12/15/2019	96	1454	2	9.9	0	40.73
2 multi 2		H						
123	1	12/16/2019	97	1455	2	10.6	0	43.75
2 multi 2		H						
123	1	12/17/2019	98	1456	2	10.2	0.01	42.61
2 multi 2		H						
123	1	12/18/2019	99	1457	2	10.4	0.24	43.12

2 multi	2	H						
123	1	12/19/2019	100	1458	2	10.3	0.06	42.26
2 multi	2	H						
123	1	12/20/2019	101	1459	2	10.6	0.02	45.37
2 multi	2	H						
123	1	12/21/2019	102	1460	2	10.4	0.09	46.89
2 multi	2	H						
123	1	12/22/2019	103	1461	2	10.9	0.01	48.51
2 multi	2	H						
123	1	12/23/2019	104	1462	2	10.4	0	38.69
2 multi	2	H						
123	1	12/24/2019	105	1463	2	10.3	0	38.11
2 multi	2	H						
123	1	12/25/2019	106	1464	2	11.4	0.24	34.76
2 multi	2	H						
123	1	12/17/2020	4	1822	3	10	0	29.11
2 multi	1	H						
123	1	12/18/2020	5	1823	3	9.9	0.15	31.25
2 multi	1	H						
123	1	12/19/2020	6	1824	3	10.2	0.19	33.97
2 multi	1	H						
123	1	12/20/2020	7	1825	3	9.9	0.37	34.73
2 multi	1	H						
123	1	12/21/2020	8	1826	3	9.6	0.06	34.22
2 multi	1	H						
123	1	12/22/2020	9	1827	3	9.8	0.19	34.76
2 multi	1	H						
123	1	12/23/2020	10	1828	3	9.9	0.2	36.69
2 multi	1	H						
123	1	12/24/2020	11	1829	3	9.8	0.09	36.85
2 multi	1	H						
123	1	12/25/2020	12	1830	3	9.6	0.05	40.51
2 multi	1	H						
123	1	12/26/2020	13	1831	3	9.8	0.11	42.79
2 multi	1	H						
123	1	12/27/2020	14	1832	3	9.3	0.17	40.65
2 multi	1	H						
123	1	12/28/2020	15	1833	3	9.8	0.11	42.81
2 multi	1	H						
123	1	12/29/2020	16	1834	3	9.5	0.28	43.17
2 multi	1	H						
123	1	12/30/2020	17	1835	3	9.5	0.02	38.66
2 multi	1	H						
123	1	12/31/2020	18	1836	3	9.3	0.09	43.57
2 multi	1	H						
123	1	01/01/2021	19	1837	3	9.2	0.07	42.55
2 multi	1	H						
123	1	01/02/2021	20	1838	3	8.9	0.3	39.98
2 multi	1	H						
123	1	01/03/2021	21	1839	3	9.2	0.2	38.36
2 multi	1	H						
123	1	01/04/2021	22	1840	3	9.5	0.1	45.67
2 multi	2	H						
123	1	01/05/2021	23	1841	3	9.8	0.02	40.83

2 multi	2	H						
123	1	01/06/2021	24	1842	3	9.6	0.03	45.87
2 multi	2	H						
123	1	01/07/2021	25	1843	3	9.3	0.08	40.4
2 multi	2	H						
123	1	01/08/2021	26	1844	3	9.6	0.07	41.81
2 multi	2	H						
123	1	01/09/2021	27	1845	3	9.3	0.06	42.54
2 multi	2	H						
123	1	01/10/2021	28	1846	3	9.5	0.73	41.98
2 multi	2	H						
123	1	01/11/2021	29	1847	3	9.6	0.09	46.17
2 multi	2	H						
123	1	01/12/2021	30	1848	3	9.6	0.08	42.69
2 multi	2	H						
123	1	01/13/2021	31	1849	3	9.6	0.1	44.57
2 multi	2	H						
123	1	01/14/2021	32	1850	3	9.6	0.1	44
2 multi	2	H						
123	1	01/15/2021	33	1851	3	9.3	0.07	44.55
2 multi	2	H						
123	1	01/16/2021	34	1852	3	9.8	0.1	43.73
2 multi	2	H						
123	1	01/17/2021	35	1853	3	9.8	0.08	46.64
2 multi	2	H						
123	1	01/18/2021	36	1854	3	9.6	0	42.98
2 multi	2	H						
123	1	01/19/2021	37	1855	3	9.9	0.1	45.29
2 multi	2	H						
123	1	01/20/2021	38	1856	3	10	0	44.81
2 multi	2	H						
123	1	01/21/2021	39	1857	3	9.6	0.07	42.19
2 multi	2	H						
123	1	01/22/2021	40	1858	3	9.5	0.06	43.39
2 multi	2	H						
123	1	01/23/2021	41	1859	3	9.5	0.11	41.61
2 multi	2	H						
123	1	01/24/2021	42	1860	3	9.6	0.07	46.39
2 multi	2	H						
123	1	01/25/2021	43	1861	3	9.9	0.08	44.42
2 multi	2	H						
123	1	01/26/2021	44	1862	3	9.8	0.13	45.73
2 multi	2	H						
123	1	01/27/2021	45	1863	3	9.8	0.01	44.95
2 multi	2	H						
123	1	01/28/2021	46	1864	3	10	0.1	46.55
2 multi	2	H						
123	1	01/29/2021	47	1865	3	9.5	0.11	44.34
2 multi	2	H						
123	1	01/30/2021	48	1866	3	9.6	0.02	47.58
2 multi	2	H						
123	1	01/31/2021	49	1867	3	9.8	0.06	43.25
2 multi	2	H						
123	1	02/01/2021	50	1868	3	9.6	0.07	41.36

2 multi 2		H						
123	1	02/02/2021	51	1869	3	9.9	0.07	43.65
2 multi 2		H						
123	1	02/03/2021	52	1870	3	9.8	0.07	45.65
2 multi 2		H						
123	1	02/04/2021	53	1871	3	9.8	0.06	47.45
2 multi 2		H						
123	1	02/05/2021	54	1872	3	9.9	0.08	45.41
2 multi 2		H						
123	1	02/06/2021	55	1873	3	0	0	51.44
2 multi 2		H						
123	1	02/07/2021	56	1874	3	10.2	0.24	43.13
2 multi 2		H						
123	1	02/08/2021	57	1875	3	10.3	0.22	42.23
2 multi 2		H						
123	1	02/09/2021	58	1876	3	10	0.08	46.15
2 multi 2		H						
123	1	02/10/2021	59	1877	3	9.8	0.02	46.38
2 multi 2		H						
123	1	02/11/2021	60	1878	3	9.6	0.07	38.94
2 multi 2		H						
123	1	02/12/2021	61	1879	3	9.3	0.07	41.4
2 multi 2		H						
123	1	02/13/2021	62	1880	3	9.6	0.07	45.89
2 multi 2		H						
123	1	02/14/2021	63	1881	3	9.3	0.06	41.26
2 multi 2		H						
123	1	02/15/2021	64	1882	3	9.6	0	41.3
2 multi 2		H						
123	1	02/16/2021	65	1883	3	10	0.12	44.5
2 multi 2		H						
123	1	02/17/2021	66	1884	3	10	0	44.07
2 multi 2		H						
123	1	02/18/2021	67	1885	3	10.2	0	42.58
2 multi 2		H						
123	1	02/19/2021	68	1886	3	10.2	0.07	40.71
2 multi 2		H						
123	1	02/20/2021	69	1887	3	10.2	0	41.88
2 multi 2		H						
123	1	02/21/2021	70	1888	3	10.4	0.07	41.06
2 multi 2		H						
123	1	02/22/2021	71	1889	3	10.2	0	39.92
2 multi 2		H						
123	1	02/23/2021	72	1890	3	10.2	0	40.81
2 multi 2		H						
123	1	02/24/2021	73	1891	3	10.6	0.08	36.68
2 multi 2		H						
123	1	02/25/2021	74	1892	3	10.2	0.07	40.69
2 multi 2		H						
123	1	02/26/2021	75	1893	3	10	0	35.39
2 multi 2		H						
123	1	02/27/2021	76	1894	3	10.4	0.08	39.22
2 multi 2		H						
123	1	02/28/2021	77	1895	3	10	0.08	38.64

2 multi	2	H						
123	1	03/01/2021	78	1896	3	10.4	0.08	37.16
2 multi	2	H						
123	1	03/02/2021	79	1897	3	10.3	0.14	39.24
2 multi	2	H						
123	1	03/03/2021	80	1898	3	9.8	0	40.02
2 multi	2	H						
123	1	03/04/2021	81	1899	3	10	0.06	41.57
2 multi	2	H						
123	1	03/05/2021	82	1900	3	9.9	0.06	38.79
2 multi	2	H						
123	1	03/06/2021	83	1901	3	10.2	0.06	44.55
2 multi	2	H						
123	1	03/07/2021	84	1902	3	9.9	0	39.45
2 multi	2	H						
123	1	03/08/2021	85	1903	3	10.2	0.53	42.52
2 multi	2	H						
123	1	03/09/2021	86	1904	3	9.9	0.06	39.04
2 multi	2	H						
123	1	03/10/2021	87	1905	3	10	0.07	44.02
2 multi	2	H						
123	1	03/11/2021	88	1906	3	10	0	38.92
2 multi	2	H						
123	1	03/12/2021	89	1907	3	10	0.05	42.96
2 multi	2	H						
123	1	03/13/2021	90	1908	3	10	0.05	42.58
2 multi	2	H						
123	1	03/14/2021	91	1909	3	9.8	0	38.25
2 multi	2	H						
123	1	03/15/2021	92	1910	3	10	0.06	43.15
2 multi	2	H						
123	1	03/16/2021	93	1911	3	10.2	0.07	41.54
2 multi	2	H						
123	1	03/17/2021	94	1912	3	10.4	0.07	41.03
2 multi	2	H						
123	1	03/18/2021	95	1913	3	10.4	0.08	38.14
2 multi	2	H						
123	1	03/19/2021	96	1914	3	10.3	0.05	37.38
2 multi	2	H						
123	1	03/20/2021	97	1915	3	10.2	0	42.95
2 multi	2	H						
123	1	03/21/2021	98	1916	3	10.2	0.07	40.82
2 multi	2	H						
123	1	03/22/2021	99	1917	3	9.8	0.33	42.55
2 multi	2	H						
123	1	03/23/2021	100	1918	3	10.2	0.06	41.76
2 multi	2	H						
123	1	03/24/2021	101	1919	3	10.3	0	43.6
2 multi	2	H						
123	1	03/25/2021	102	1920	3	10.4	0.07	38.36
2 multi	2	H						
123	1	03/26/2021	103	1921	3	10.3	0.06	39.07
2 multi	2	H						
123	1	03/27/2021	104	1922	3	10.3	0.06	38.63

2 multi	2	H						
123	1	03/28/2021	105	1923	3	10	0.05	37.78
2 multi	2	H						
123	1	03/29/2021	106	1924	3	10.4	0.05	41.14
2 multi	2	H						
123	1	03/30/2021	107	1925	3	11.3	0.07	39.71
2 multi	2	H						
123	1	03/31/2021	108	1926	3	10.9	0.08	41.88
2 multi	2	H						
123	1	04/01/2021	109	1927	3	10.7	0.1	37.5
2 multi	2	H						
123	1	04/02/2021	110	1928	3	10.4	0.11	39.58
2 multi	2	H						
123	1	04/03/2021	111	1929	3	10.7	0.33	36.52
2 multi	2	H						
123	1	04/04/2021	112	1930	3	10.4	0	38.67
2 multi	2	H						
123	1	04/05/2021	113	1931	3	10.4	0.19	38.15
2 multi	2	H						
123	1	04/06/2021	114	1932	3	10.3	0.05	38.12
2 multi	2	H						
123	1	04/07/2021	115	1933	3	10.7	0.18	36.21
2 multi	2	H						
123	1	04/08/2021	116	1934	3	10.9	0.05	36.16
2 multi	2	H						
123	1	04/09/2021	117	1935	3	10.6	0	40.04
2 multi	2	H						
123	1	04/10/2021	118	1936	3	10.2	0.05	40.36
2 multi	2	H						
123	1	04/11/2021	119	1937	3	10.7	0	40.13
2 multi	2	H						
123	1	04/12/2021	120	1938	3	10.7	0	38.75
2 multi	2	H						
123	1	04/13/2021	121	1939	3	10.6	0	39.78
2 multi	2	H						
123	1	04/14/2021	122	1940	3	10.7	0.04	41.27
2 multi	2	H						
123	1	04/15/2021	123	1941	3	10.3	0.08	37.1
2 multi	2	H						
123	1	04/16/2021	124	1942	3	10.4	0.06	40.63
2 multi	2	H						
123	1	04/17/2021	125	1943	3	10.4	0.07	39.37
2 multi	2	H						
123	1	04/18/2021	126	1944	3	10.6	0	39.62
2 multi	2	H						
123	1	04/19/2021	127	1945	3	10.9	0.07	39.41
2 multi	2	H						
123	1	04/20/2021	128	1946	3	10.7	0	41.35
2 multi	2	H						
123	1	04/21/2021	129	1947	3	10.7	0.21	38.32
2 multi	2	H						
123	1	04/22/2021	130	1948	3	10.3	0	39.42
2 multi	2	H						
123	1	04/23/2021	131	1949	3	10.4	0	37.53

2 multi 2		H						
123	1	04/24/2021	132	1950	3	10.4	0.07	39.07
2 multi 2		H						
123	1	04/25/2021	133	1951	3	10.4	0.13	41.49
2 multi 2		H						
123	1	04/26/2021	134	1952	3	10.4	0	41.02
2 multi 2		H						
123	1	04/27/2021	135	1953	3	10.4	0	38.93
2 multi 2		H						
123	1	04/28/2021	136	1954	3	10.4	0.16	38.69
2 multi 2		H						
123	1	04/29/2021	137	1955	3	10.4	0.06	41.59
2 multi 2		H						
123	1	04/30/2021	138	1956	3	10.6	0.07	37.6
2 multi 2		H						
123	1	05/01/2021	139	1957	3	10.6	0.06	37.66
2 multi 2		H						
123	1	05/02/2021	140	1958	3	10.4	0	40.96
2 multi 2		H						
123	1	05/03/2021	141	1959	3	10.4	0	43.12
2 multi 2		H						
123	1	05/04/2021	142	1960	3	10.4	0.06	42.71
2 multi 2		H						
123	1	05/05/2021	143	1961	3	10.7	0.07	39.81
2 multi 2		H						
123	1	05/06/2021	144	1962	3	10.4	0	41.17
2 multi 2		H						
123	1	05/07/2021	145	1963	3	10.7	0.07	41.39
2 multi 2		H						
123	1	05/08/2021	146	1964	3	10.4	0.07	38.61
2 multi 2		H						
123	1	05/09/2021	147	1965	3	10.6	0.07	39.86
2 multi 2		H						
123	1	05/10/2021	148	1966	3	10.6	0.25	41.59
2 multi 2		H						
123	1	05/11/2021	149	1967	3	10.7	0.06	41.3
2 multi 2		H						
123	1	05/12/2021	150	1968	3	10.6	0	43.21
2 multi 2		H						
123	1	05/13/2021	151	1969	3	10.4	0	41.3
2 multi 2		H						
123	1	05/14/2021	152	1970	3	10.4	0.06	43.15
2 multi 2		H						
123	1	05/15/2021	153	1971	3	10.9	0.06	40.73
2 multi 2		H						
123	1	05/16/2021	154	1972	3	10.9	0.07	40.68
2 multi 2		H						
123	1	05/17/2021	155	1973	3	10.7	0	39.9
2 multi 2		H						
123	1	05/18/2021	156	1974	3	10.6	0.25	42.21
2 multi 2		H						
123	1	05/19/2021	157	1975	3	10.7	0.07	42.94
2 multi 2		H						
123	1	05/20/2021	158	1976	3	10.4	0.06	40.38

2 multi	2	H						
123	1	05/21/2021	159	1977	3	10.7	0.07	42.46
2 multi	2	H						
123	1	05/22/2021	160	1978	3	10.3	0.05	43.75
2 multi	2	H						
123	1	05/23/2021	161	1979	3	10.7	0.07	43.74
2 multi	2	H						
123	1	05/24/2021	162	1980	3	10.9	0.06	43.01
2 multi	2	H						
123	1	05/25/2021	163	1981	3	10	0.07	39.45
2 multi	2	H						
123	1	05/26/2021	164	1982	3	10.6	0.07	19.42
2 multi	2	H						
126	1	06/15/2018	7	696	1	9.8	0.19	24.47
1 primi	1	H						
126	1	06/16/2018	8	697	1	9.8	0	23.65
1 primi	1	H						
126	1	06/17/2018	9	698	1	10	0.03	25.47
1 primi	1	H						
126	1	06/18/2018	10	699	1	9.3	0.09	21.2
1 primi	1	H						
126	1	06/19/2018	11	700	1	9.5	0.15	23.45
1 primi	1	H						
126	1	06/20/2018	12	701	1	9.6	0	23.02
1 primi	1	H						
126	1	06/21/2018	13	702	1	10	0.04	25.06
1 primi	1	H						
126	1	06/22/2018	14	703	1	9.3	0.11	18.44
1 primi	1	H						
126	1	06/23/2018	15	704	1	9.3	0.03	24.11
1 primi	1	H						
126	1	06/24/2018	16	705	1	9.8	0.04	25.69
1 primi	1	H						
126	1	06/25/2018	17	706	1	9.3	0.05	25.63
1 primi	1	H						
126	1	06/26/2018	18	707	1	9.3	0.13	26.05
1 primi	1	H						
126	1	06/27/2018	19	708	1	8.9	0.08	27.36
1 primi	1	H						
126	1	06/28/2018	20	709	1	8.9	0.11	26.27
1 primi	1	H						
126	1	06/29/2018	21	710	1	9.2	0	27.11
1 primi	1	H						
126	1	06/30/2018	22	711	1	9.1	0.06	27.82
1 primi	2	H						
126	1	07/01/2018	23	712	1	9.3	0.17	26.22
1 primi	2	H						
126	1	07/02/2018	24	713	1	9.2	0	27.91
1 primi	2	H						
126	1	07/03/2018	25	714	1	8.9	0	26.63
1 primi	2	H						
126	1	07/04/2018	26	715	1	9.3	0.14	28.24
1 primi	2	H						
126	1	07/05/2018	27	716	1	9.3	0.03	30.22

1 primi	2	H						
126	1	07/06/2018	28	717	1	9.2	0.02	29.72
1 primi	2	H						
126	1	07/07/2018	29	718	1	9.1	0	28.32
1 primi	2	H						
126	1	07/08/2018	30	719	1	9.1	0.09	29.13
1 primi	2	H						
126	1	07/09/2018	31	720	1	9.3	0.01	30.32
1 primi	2	H						
126	1	07/10/2018	32	721	1	9.3	0	30.68
1 primi	2	H						
126	1	07/11/2018	33	722	1	9.5	0	30.86
1 primi	2	H						
126	1	07/12/2018	34	723	1	9.3	0	31.74
1 primi	2	H						
126	1	07/13/2018	35	724	1	9.8	0.02	30.41
1 primi	2	H						
126	1	07/14/2018	36	725	1	9.5	0.06	28.17
1 primi	2	H						
126	1	07/15/2018	37	726	1	9.3	0.18	27.49
1 primi	2	H						
126	1	07/16/2018	38	727	1	9.2	0	27.58
1 primi	2	H						
126	1	07/17/2018	39	728	1	9.1	0.21	27.41
1 primi	2	H						
126	1	07/18/2018	40	729	1	9.2	0.61	27.95
1 primi	2	H						
126	1	07/19/2018	41	730	1	9.5	0	28.96
1 primi	2	H						
126	1	07/20/2018	42	731	1	9.3	0	28.34
1 primi	2	H						
126	1	07/21/2018	43	732	1	9.5	0.04	28.1
1 primi	2	H						
126	1	07/22/2018	44	733	1	9.6	0	29.43
1 primi	2	H						
126	1	07/23/2018	45	734	1	9.9	0.04	30.55
1 primi	2	H						
126	1	07/24/2018	46	735	1	9.8	0	30.24
1 primi	2	H						
126	1	07/25/2018	47	736	1	9.8	0.21	32.81
1 primi	2	H						
126	1	07/26/2018	48	737	1	9.8	0	32.42
1 primi	2	H						
126	1	07/27/2018	49	738	1	10.9	0.02	29.98
1 primi	2	H						
126	1	07/28/2018	50	739	1	9.9	0	32.43
1 primi	2	H						
126	1	07/29/2018	51	740	1	10	0	29.37
1 primi	2	H						
126	1	07/30/2018	52	741	1	9.6	0.04	28.3
1 primi	2	H						
126	1	07/31/2018	53	742	1	9.9	0.03	31.11
1 primi	2	H						
126	1	08/01/2018	54	743	1	9.9	0.05	33.57

1 primi	2	H						
126	1	08/02/2018	55	744	1	9.6	0	30.69
1 primi	2	H						
126	1	08/03/2018	56	745	1	10	0	29.95
1 primi	2	H						
126	1	08/04/2018	57	746	1	9.5	0.14	29.43
1 primi	2	H						
126	1	08/05/2018	58	747	1	9.9	0.03	29.48
1 primi	2	H						
126	1	08/06/2018	59	748	1	9.8	0	31.73
1 primi	2	H						
126	1	08/07/2018	60	749	1	10	0	31.54
1 primi	2	H						
126	1	08/08/2018	61	750	1	10.2	0.02	31.5
1 primi	2	H						
126	1	08/09/2018	62	751	1	10	0.02	32.42
1 primi	2	H						
126	1	08/10/2018	63	752	1	9.8	0.11	34.74
1 primi	2	H						
126	1	08/11/2018	64	753	1	9.9	0.09	31.65
1 primi	2	H						
126	1	08/12/2018	65	754	1	9.8	0	32.94
1 primi	2	H						
126	1	08/13/2018	66	755	1	9.8	0	32.79
1 primi	2	H						
126	1	08/14/2018	67	756	1	9.6	0	31.43
1 primi	2	H						
126	1	08/15/2018	68	757	1	9.5	0	31.34
1 primi	2	H						
126	1	08/16/2018	69	758	1	9.3	0	31.19
1 primi	2	H						
126	1	08/17/2018	70	759	1	9.6	0.01	28.64
1 primi	2	H						
126	1	08/18/2018	71	760	1	9.6	0.14	28.11
1 primi	2	H						
126	1	08/19/2018	72	761	1	9.8	0.12	31.9
1 primi	2	H						
126	1	08/20/2018	73	762	1	9.8	0	32.91
1 primi	2	H						
126	1	08/21/2018	74	763	1	10	0	31.97
1 primi	2	H						
126	1	08/22/2018	75	764	1	9.8	0.11	31.71
1 primi	2	H						
126	1	08/23/2018	76	765	1	9.8	0	33.83
1 primi	2	H						
126	1	08/24/2018	77	766	1	9.8	0	34.38
1 primi	2	H						
126	1	08/25/2018	78	767	1	9.8	0.12	29.98
1 primi	2	H						
126	1	08/26/2018	79	768	1	10	0.03	32.76
1 primi	2	H						
126	1	08/27/2018	80	769	1	9.8	0	32.85
1 primi	2	H						
126	1	08/28/2018	81	770	1	9.8	0	32.73

1 primi 2		H						
126	1	08/29/2018	82	771	1	9.9	0	29.26
1 primi 2		H						
126	1	08/30/2018	83	772	1	9.6	0.18	32.49
1 primi 2		H						
126	1	08/31/2018	84	773	1	9.2	0	31.27
1 primi 2		H						
126	1	09/01/2018	85	774	1	9.6	0.17	32
1 primi 2		H						
126	1	09/02/2018	86	775	1	9.8	0	32.69
1 primi 2		H						
126	1	09/03/2018	87	776	1	9.6	0.08	31.65
1 primi 2		H						
126	1	09/04/2018	88	777	1	9.6	0	34.23
1 primi 2		H						
126	1	09/05/2018	89	778	1	9.6	0.08	32.11
1 primi 2		H						
126	1	09/06/2018	90	779	1	9.6	0.12	31.3
1 primi 2		H						
126	1	09/07/2018	91	780	1	9.5	0.01	31.22
1 primi 2		H						
126	1	09/08/2018	92	781	1	9.8	0.12	30.96
1 primi 2		H						
126	1	09/09/2018	93	782	1	9.5	0	32.44
1 primi 2		H						
126	1	09/10/2018	94	783	1	10	0.05	32.32
1 primi 2		H						
126	1	09/11/2018	95	784	1	9.9	0.18	32.54
1 primi 2		H						
126	1	09/12/2018	96	785	1	9.8	0	30.85
1 primi 2		H						
126	1	09/13/2018	97	786	1	9.8	0.09	28.5
1 primi 2		H						
126	1	09/14/2018	98	787	1	9.9	0.04	23.1
1 primi 2		H						
126	1	09/15/2018	99	788	1	9.8	0.13	25.22
1 primi 2		H						
126	1	09/16/2018	100	789	1	9.5	0.01	24.06
1 primi 2		H						
126	1	09/17/2018	101	790	1	8.9	0.07	24.77
1 primi 2		H						
126	1	09/18/2018	102	791	1	9.8	0.02	27.49
1 primi 2		H						
126	1	09/19/2018	103	792	1	10	0.04	27.94
1 primi 2		H						
126	1	09/20/2018	104	793	1	10.2	0	30.97
1 primi 2		H						
126	1	09/21/2018	105	794	1	10	0	29.96
1 primi 2		H						
126	1	09/22/2018	106	795	1	10	0	30.83
1 primi 2		H						
126	1	09/23/2018	107	796	1	9.8	0	30.79
1 primi 2		H						
126	1	09/24/2018	108	797	1	9.9	0.05	28.36

1 primi	2	H						
126	1	09/25/2018	109	798	1	10	0.07	27.49
1 primi	2	H						
126	1	09/26/2018	110	799	1	9.6	0.08	29.7
1 primi	2	H						
126	1	09/27/2018	111	800	1	10	0.01	32.08
1 primi	2	H						
126	1	06/17/2019	6	1063	2	9.5	0.22	28.8
2 multi	1	H						
126	1	06/18/2019	7	1064	2	9.6	0.2	32.06
2 multi	1	H						
126	1	06/19/2019	8	1065	2	9.8	0.2	31.07
2 multi	1	H						
126	1	06/20/2019	9	1066	2	9.6	0.09	32.52
2 multi	1	H						
126	1	06/21/2019	10	1067	2	9.6	0.11	32
2 multi	1	H						
126	1	06/22/2019	11	1068	2	9.5	0	34.43
2 multi	1	H						
126	1	06/23/2019	12	1069	2	9.6	0	37.69
2 multi	1	H						
126	1	06/24/2019	13	1070	2	9.3	0	37.57
2 multi	1	H						
126	1	06/25/2019	14	1071	2	9.5	0.06	36.25
2 multi	1	H						
126	1	06/26/2019	15	1072	2	9.6	0.04	36.96
2 multi	1	H						
126	1	06/27/2019	16	1073	2	9.6	0	39.1
2 multi	1	H						
126	1	06/28/2019	17	1074	2	9.3	0	34.82
2 multi	1	H						
126	1	06/29/2019	18	1075	2	9.5	0.21	38.24
2 multi	1	H						
126	1	06/30/2019	19	1076	2	9.9	0	38.48
2 multi	1	H						
126	1	07/01/2019	20	1077	2	9.8	0	41.92
2 multi	1	H						
126	1	07/02/2019	21	1078	2	9.6	0	42.34
2 multi	1	H						
126	1	07/03/2019	22	1079	2	9.6	0	39.69
2 multi	2	H						
126	1	07/04/2019	23	1080	2	9.5	0.05	40.18
2 multi	2	H						
126	1	07/05/2019	24	1081	2	9.6	0.05	41.88
2 multi	2	H						
126	1	07/06/2019	25	1082	2	9.6	0.05	41.77
2 multi	2	H						
126	1	07/07/2019	26	1083	2	9.6	0	40.62
2 multi	2	H						
126	1	07/08/2019	27	1084	2	9.3	0.17	40.83
2 multi	2	H						
126	1	07/09/2019	28	1085	2	9.5	0	38.46
2 multi	2	H						
126	1	07/10/2019	29	1086	2	9.1	0.01	35.45

2 multi	2	H						
126	1	07/11/2019	30	1087	2	9.3	0	40.77
2 multi	2	H						
126	1	07/12/2019	31	1088	2	9.5	0.08	39.15
2 multi	2	H						
126	1	07/13/2019	32	1089	2	9.5	0.06	38.93
2 multi	2	H						
126	1	07/14/2019	33	1090	2	9.5	0.02	40.8
2 multi	2	H						
126	1	07/15/2019	34	1091	2	9.8	0	43.6
2 multi	2	H						
126	1	07/16/2019	35	1092	2	9.6	0.05	43.86
2 multi	2	H						
126	1	07/17/2019	36	1093	2	9.6	0	41.76
2 multi	2	H						
126	1	07/18/2019	37	1094	2	9.5	0	39.47
2 multi	2	H						
126	1	07/19/2019	38	1095	2	9.3	0.06	40.3
2 multi	2	H						
126	1	07/20/2019	39	1096	2	9.8	0	44.01
2 multi	2	H						
126	1	07/21/2019	40	1097	2	9.5	0	41.55
2 multi	2	H						
126	1	07/22/2019	41	1098	2	9.9	0	41.23
2 multi	2	H						
126	1	07/23/2019	42	1099	2	9.8	0	45.95
2 multi	2	H						
126	1	07/24/2019	43	1100	2	9.9	0.51	44.58
2 multi	2	H						
126	1	07/25/2019	44	1101	2	9.9	0	41.08
2 multi	2	H						
126	1	07/26/2019	45	1102	2	9.8	0.04	36.98
2 multi	2	H						
126	1	07/27/2019	46	1103	2	9.8	0.1	39.22
2 multi	2	H						
126	1	07/28/2019	47	1104	2	9.6	0.36	39.74
2 multi	2	H						
126	1	07/29/2019	48	1105	2	9.5	0	38.41
2 multi	2	H						
126	1	07/30/2019	49	1106	2	9.2	0	37.14
2 multi	2	H						
126	1	07/31/2019	50	1107	2	9.6	0.06	36.28
2 multi	2	H						
126	1	08/01/2019	51	1108	2	9.9	0.06	39.41
2 multi	2	H						
126	1	08/02/2019	52	1109	2	9.8	0	38.32
2 multi	2	H						
126	1	08/03/2019	53	1110	2	10.2	0	37.19
2 multi	2	H						
126	1	08/04/2019	54	1111	2	10.3	0	42.24
2 multi	2	H						
126	1	08/05/2019	55	1112	2	10	0.07	40.35
2 multi	2	H						
126	1	08/06/2019	56	1113	2	10.4	0.06	41.18

2 multi 2		H						
126	1	08/07/2019	57	1114	2	10.2	0	45.02
2 multi 2		H						
126	1	08/08/2019	58	1115	2	9.8	0	41.96
2 multi 2		H						
126	1	08/09/2019	59	1116	2	9.6	0	40.4
2 multi 2		H						
126	1	08/10/2019	60	1117	2	9.5	0	36.62
2 multi 2		H						
126	1	08/11/2019	61	1118	2	9.6	0	39.35
2 multi 2		H						
126	1	08/12/2019	62	1119	2	9.9	0.1	41.62
2 multi 2		H						
126	1	08/13/2019	63	1120	2	9.3	0	34.67
2 multi 2		H						
126	1	08/14/2019	64	1121	2	9.8	0	39.53
2 multi 2		H						
126	1	08/15/2019	65	1122	2	9.6	0.18	36.97
2 multi 2		H						
126	1	08/16/2019	66	1123	2	9.6	0.02	36.38
2 multi 2		H						
126	1	08/17/2019	67	1124	2	10	0	39.13
2 multi 2		H						
126	1	08/18/2019	68	1125	2	10	0.05	38.34
2 multi 2		H						
126	1	08/19/2019	69	1126	2	9.9	0.57	37.08
2 multi 2		H						
126	1	08/20/2019	70	1127	2	9.6	0	39.13
2 multi 2		H						
126	1	08/21/2019	71	1128	2	9.5	0	35.46
2 multi 2		H						
126	1	08/22/2019	72	1129	2	9.5	0	36.39
2 multi 2		H						
126	1	08/23/2019	73	1130	2	9.8	0.05	35.95
2 multi 2		H						
126	1	08/24/2019	74	1131	2	9.9	0.14	38.42
2 multi 2		H						
126	1	08/25/2019	75	1132	2	9.9	0	37.61
2 multi 2		H						
126	1	08/26/2019	76	1133	2	9.8	0.06	34.75
2 multi 2		H						
126	1	08/27/2019	77	1134	2	9.8	0	37.6
2 multi 2		H						
126	1	08/28/2019	78	1135	2	10	0.06	36.06
2 multi 2		H						
126	1	08/29/2019	79	1136	2	9.9	0	36.75
2 multi 2		H						
126	1	08/30/2019	80	1137	2	9.8	0.04	36.25
2 multi 2		H						
126	1	08/31/2019	81	1138	2	10.3	0.03	36.87
2 multi 2		H						
126	1	09/01/2019	82	1139	2	10	0.08	35.31
2 multi 2		H						
126	1	09/02/2019	83	1140	2	10.2	0.05	35.3

2 multi	2	H						
126	1	09/03/2019	84	1141	2	10	0	35.48
2 multi	2	H						
126	1	09/04/2019	85	1142	2	9.6	0	28.95
2 multi	2	H						
126	1	09/05/2019	86	1143	2	10	0.07	32.8
2 multi	2	H						
126	1	09/06/2019	87	1144	2	9.8	0	31.54
2 multi	2	H						
126	1	09/07/2019	88	1145	2	9.8	0	33.2
2 multi	2	H						
126	1	09/08/2019	89	1146	2	9.9	0	31.42
2 multi	2	H						
126	1	09/09/2019	90	1147	2	9.9	0	34.76
2 multi	2	H						
126	1	09/10/2019	91	1148	2	9.9	0.15	35.53
2 multi	2	H						
126	1	09/11/2019	92	1149	2	9.6	0	34.13
2 multi	2	H						
126	1	09/12/2019	93	1150	2	9.9	0	36.59
2 multi	2	H						
126	1	05/22/2020	3	1403	3	9.9	0.34	27.08
2 multi	1	H						
126	1	05/23/2020	4	1404	3	9.8	0.12	29.06
2 multi	1	H						
126	1	05/24/2020	5	1405	3	9.6	0.09	30.36
2 multi	1	H						
126	1	05/25/2020	6	1406	3	9.6	0.18	35.74
2 multi	1	H						
126	1	05/26/2020	7	1407	3	9.5	0	33.04
2 multi	1	H						
126	1	05/27/2020	8	1408	3	9.3	0.09	33.74
2 multi	1	H						
126	1	05/28/2020	9	1409	3	9.5	0	34.56
2 multi	1	H						
126	1	05/29/2020	10	1410	3	9.8	0.06	35.92
2 multi	1	H						
126	1	05/30/2020	11	1411	3	9.8	0	37.38
2 multi	1	H						
126	1	05/31/2020	12	1412	3	9.3	0.06	36.4
2 multi	1	H						
126	1	06/01/2020	13	1413	3	9.5	0	39.07
2 multi	1	H						
126	1	06/02/2020	14	1414	3	9.3	0.37	35.02
2 multi	1	H						
126	1	06/03/2020	15	1415	3	9.6	0	40.67
2 multi	1	H						
126	1	06/04/2020	16	1416	3	9.8	0.46	40.51
2 multi	1	H						
126	1	06/05/2020	17	1417	3	9.5	0.05	35.73
2 multi	1	H						
126	1	06/06/2020	18	1418	3	9.5	0.05	38.48
2 multi	1	H						
126	1	06/07/2020	19	1419	3	9.6	0.04	40.58

2 multi	1	H						
126	1	06/08/2020	20	1420	3	9.8	0.36	44.43
2 multi	1	H						
126	1	06/09/2020	21	1421	3	9.6	0.04	42.46
2 multi	1	H						
126	1	06/10/2020	22	1422	3	9.5	0.33	44.43
2 multi	2	H						
126	1	06/11/2020	23	1423	3	9.3	0.05	43.31
2 multi	2	H						
126	1	06/12/2020	24	1424	3	9.6	0	47.49
2 multi	2	H						
126	1	06/13/2020	25	1425	3	9.6	0	47.75
2 multi	2	H						
126	1	06/14/2020	26	1426	3	9.8	0	48.2
2 multi	2	H						
126	1	06/15/2020	27	1427	3	9.6	0	48.28
2 multi	2	H						
126	1	06/16/2020	28	1428	3	9.8	0	47.12
2 multi	2	H						
126	1	06/17/2020	29	1429	3	9.6	0.13	45.63
2 multi	2	H						
126	1	06/18/2020	30	1430	3	9.8	0	44.16
2 multi	2	H						
126	1	06/19/2020	31	1431	3	9.5	0.05	42.05
2 multi	2	H						
126	1	06/20/2020	32	1432	3	9.9	0	48.58
2 multi	2	H						
126	1	06/21/2020	33	1433	3	9.3	0	40.41
2 multi	2	H						
126	1	06/22/2020	34	1434	3	9.5	0	43.44
2 multi	2	H						
126	1	06/23/2020	35	1435	3	9.6	0	43.93
2 multi	2	H						
126	1	06/24/2020	36	1436	3	9.6	0	42.34
2 multi	2	H						
126	1	06/25/2020	37	1437	3	9.6	0	43.83
2 multi	2	H						
126	1	06/26/2020	38	1438	3	9.9	0	47.18
2 multi	2	H						
126	1	06/27/2020	39	1439	3	9.6	0	45.15
2 multi	2	H						
126	1	06/28/2020	40	1440	3	9.9	0	44.48
2 multi	2	H						
126	1	06/29/2020	41	1441	3	9.5	0	38.21
2 multi	2	H						
126	1	10/14/2020	148	1548	3	9.3	0	33.41
2 multi	2	H						
126	1	10/15/2020	149	1549	3	9.3	0.01	36.41
2 multi	2	H						
126	1	10/16/2020	150	1550	3	9.5	0.01	36.53
2 multi	2	H						
126	1	10/17/2020	151	1551	3	9.5	0	36.49
2 multi	2	H						
126	1	10/18/2020	152	1552	3	9.8	0.06	37.28

2 multi 2		H						
126	1	10/19/2020	153	1553	3	9.8	0.03	37.94
2 multi 2		H						
126	1	10/20/2020	154	1554	3	9.5	0	39.29
2 multi 2		H						
126	1	10/21/2020	155	1555	3	9.5	0.04	38.79
2 multi 2		H						
126	1	10/22/2020	156	1556	3	9.6	0.12	38.31
2 multi 2		H						
126	1	10/23/2020	157	1557	3	9.8	0.08	39.18
2 multi 2		H						
126	1	10/24/2020	158	1558	3	9.8	0.07	35.96
2 multi 2		H						
126	1	10/25/2020	159	1559	3	9.8	0.07	38.42
2 multi 2		H						
126	1	10/26/2020	160	1560	3	10	0.05	38.71
2 multi 2		H						
126	1	10/27/2020	161	1561	3	9.6	0.04	36.29
2 multi 2		H						
126	1	10/28/2020	162	1562	3	9.8	0.03	39.18
2 multi 2		H						
126	1	10/29/2020	163	1563	3	9.8	0	38.2
2 multi 2		H						
126	1	10/30/2020	164	1564	3	9.8	0	38.9
2 multi 2		H						
126	1	10/31/2020	165	1565	3	9.6	0.07	38.12
2 multi 2		H						
126	1	11/01/2020	166	1566	3	9.5	0.02	37.16
2 multi 2		H						
126	1	11/02/2020	167	1567	3	9.8	0.11	41.59
2 multi 2		H						
126	1	11/03/2020	168	1568	3	9.6	0.08	39.55
2 multi 2		H						
126	1	11/04/2020	169	1569	3	9.5	0.1	36.49
2 multi 2		H						
126	1	11/05/2020	170	1570	3	9.8	0.11	38.11
2 multi 2		H						
126	1	11/06/2020	171	1571	3	9.5	0.05	35.05
2 multi 2		H						
126	1	11/07/2020	172	1572	3	9.6	0	37.26
2 multi 2		H						
126	1	11/08/2020	173	1573	3	9.5	0.06	38.35
2 multi 2		H						
126	1	11/09/2020	174	1574	3	9.6	0.06	38.98
2 multi 2		H						
126	1	11/10/2020	175	1575	3	9.6	0.01	38.17
2 multi 2		H						
126	1	11/11/2020	176	1576	3	9.5	0	38.29
2 multi 2		H						
126	1	11/12/2020	177	1577	3	9.6	0.08	40.87
2 multi 2		H						
126	1	11/13/2020	178	1578	3	9.5	0.13	38.53
2 multi 2		H						
126	1	11/14/2020	179	1579	3	9.6	0.11	41.34

2 multi	2	H						
126	1	11/15/2020	180	1580	3	9.6	0.07	39.38
2 multi	2	H						
126	1	11/16/2020	181	1581	3	9.6	0.06	37.78
2 multi	2	H						
126	1	11/17/2020	182	1582	3	9.6	0.05	37.74
2 multi	2	H						
126	1	11/18/2020	183	1583	3	9.5	0	40.08
2 multi	2	H						
126	1	11/19/2020	184	1584	3	9.5	0	37.75
2 multi	2	H						
126	1	11/20/2020	185	1585	3	9.6	0.11	38.62
2 multi	2	H						
126	1	11/21/2020	186	1586	3	9.6	0.01	38.43
2 multi	2	H						
126	1	05/24/2021	9	1770	4	9.3	0.28	35.84
2 multi	1	H						
126	1	05/25/2021	10	1771	4	9.9	0.09	35.52
2 multi	1	H						
126	1	05/26/2021	11	1772	4	10	0	18.12
2 multi	1	H						
128	1	11/17/2019	8	711	1	9.5	0.16	24.33
1 primi	1	H						
128	1	11/18/2019	9	712	1	9.8	0.14	27.83
1 primi	1	H						
128	1	11/19/2019	10	713	1	9.6	0.04	27.02
1 primi	1	H						
128	1	11/20/2019	11	714	1	9.6	0.02	28.02
1 primi	1	H						
128	1	11/21/2019	12	715	1	9.9	0.05	27.3
1 primi	1	H						
128	1	11/22/2019	13	716	1	9.8	0	27.4
1 primi	1	H						
128	1	11/23/2019	14	717	1	9.6	0	27.09
1 primi	1	H						
128	1	11/24/2019	15	718	1	9.8	0	28.07
1 primi	1	H						
128	1	11/25/2019	16	719	1	9.8	0.01	26.87
1 primi	1	H						
128	1	11/26/2019	17	720	1	9.9	0	28.73
1 primi	1	H						
128	1	11/27/2019	18	721	1	10.2	0	25.29
1 primi	1	H						
128	1	11/28/2019	19	722	1	9.9	0	25.34
1 primi	1	H						
128	1	11/29/2019	20	723	1	10	0	27.57
1 primi	1	H						
128	1	11/30/2019	21	724	1	10	0	28.39
1 primi	1	H						
128	1	12/01/2019	22	725	1	10	0	27.77
1 primi	2	H						
128	1	12/02/2019	23	726	1	10	0	28.97
1 primi	2	H						
128	1	12/03/2019	24	727	1	10	0	27.96

1 primi	2	H						
128	1	12/04/2019	25	728	1	9.8	0	30.09
1 primi	2	H						
128	1	12/05/2019	26	729	1	10	0	25.67
1 primi	2	H						
128	1	12/06/2019	27	730	1	10	0	24.47
1 primi	2	H						
128	1	12/07/2019	28	731	1	10.2	0	28.48
1 primi	2	H						
128	1	12/08/2019	29	732	1	10.2	0.01	32.13
1 primi	2	H						
128	1	12/09/2019	30	733	1	10.2	0	32.99
1 primi	2	H						
128	1	12/10/2019	31	734	1	10	0	24.66
1 primi	2	H						
128	1	12/11/2019	32	735	1	9.6	0.06	33.42
1 primi	2	H						
128	1	12/12/2019	33	736	1	9.8	0	31.37
1 primi	2	H						
128	1	12/13/2019	34	737	1	9.6	0	32.03
1 primi	2	H						
128	1	12/14/2019	35	738	1	9.9	0	29.92
1 primi	2	H						
128	1	12/15/2019	36	739	1	9.8	0.01	24.85
1 primi	2	H						
128	1	12/16/2019	37	740	1	9.3	0	31.57
1 primi	2	H						
128	1	12/17/2019	38	741	1	9.8	0	30.73
1 primi	2	H						
128	1	12/18/2019	39	742	1	9.9	0	33.89
1 primi	2	H						
128	1	12/19/2019	40	743	1	9.6	0	27.96
1 primi	2	H						
128	1	12/20/2019	41	744	1	9.6	0.03	29.7
1 primi	2	H						
128	1	12/21/2019	42	745	1	9.5	0.04	31.28
1 primi	2	H						
128	1	12/22/2019	43	746	1	9.6	0	28.16
1 primi	2	H						
128	1	12/23/2019	44	747	1	9.9	0	33.66
1 primi	2	H						
128	1	12/24/2019	45	748	1	9.8	0	27.45
1 primi	2	H						
128	1	12/25/2019	46	749	1	9.6	0	29.83
1 primi	2	H						
128	1	12/26/2019	47	750	1	10.2	0	28.1
1 primi	2	H						
128	1	12/27/2019	48	751	1	9.8	0.24	32.09
1 primi	2	H						
128	1	12/28/2019	49	752	1	10.2	0	30.69
1 primi	2	H						
128	1	12/29/2019	50	753	1	9.9	0	36.34
1 primi	2	H						
128	1	12/30/2019	51	754	1	9.8	0	31.57

1 primi 2		H						
128	1	12/31/2019	52	755	1	9.9	0	31.57
1 primi 2		H						
128	1	01/01/2020	53	756	1	9.8	0	30.89
1 primi 2		H						
128	1	01/02/2020	54	757	1	9.3	0	29.11
1 primi 2		H						
128	1	01/03/2020	55	758	1	9.6	0.34	31.04
1 primi 2		H						
128	1	01/04/2020	56	759	1	9.6	0	27.84
1 primi 2		H						
128	1	01/05/2020	57	760	1	9.8	0	30.31
1 primi 2		H						
128	1	01/06/2020	58	761	1	9.6	0	31
1 primi 2		H						
128	1	01/07/2020	59	762	1	9.9	0.93	32.16
1 primi 2		H						
128	1	01/08/2020	60	763	1	9.9	0.22	33.63
1 primi 2		H						
128	1	01/09/2020	61	764	1	10	0.17	31.07
1 primi 2		H						
128	1	01/10/2020	62	765	1	9.8	0	29.28
1 primi 2		H						
128	1	10/19/2020	15	1048	2	9.8	0.21	32.98
2 multi 1		H						
128	1	10/20/2020	16	1049	2	9.6	0.06	34.63
2 multi 1		H						
128	1	10/21/2020	17	1050	2	9.6	0.02	34.4
2 multi 1		H						
128	1	10/22/2020	18	1051	2	9.6	0.23	34
2 multi 1		H						
128	1	10/23/2020	19	1052	2	9.8	0.03	36.47
2 multi 1		H						
128	1	10/24/2020	20	1053	2	9.3	0.05	35.71
2 multi 1		H						
128	1	10/25/2020	21	1054	2	10.2	0.05	39.27
2 multi 1		H						
128	1	10/26/2020	22	1055	2	9.9	0.02	38.32
2 multi 2		H						
128	1	10/27/2020	23	1056	2	9.6	0	37.87
2 multi 2		H						
128	1	10/28/2020	24	1057	2	9.3	0.1	37.59
2 multi 2		H						
128	1	10/29/2020	25	1058	2	9.3	0	37.64
2 multi 2		H						
128	1	10/30/2020	26	1059	2	9.3	0.02	34.91
2 multi 2		H						
128	1	10/31/2020	27	1060	2	9.6	0	36.81
2 multi 2		H						
128	1	11/01/2020	28	1061	2	9.5	0.05	35.73
2 multi 2		H						
128	1	11/02/2020	29	1062	2	9.8	0.28	37.84
2 multi 2		H						
128	1	11/03/2020	30	1063	2	9.9	0	33.86

2 multi	2	H						
128	1	11/04/2020	31	1064	2	9.9	0.25	36.53
2 multi	2	H						
128	1	11/05/2020	32	1065	2	9.8	0.16	35.37
2 multi	2	H						
128	1	11/06/2020	33	1066	2	9.8	0	35.36
2 multi	2	H						
128	1	11/07/2020	34	1067	2	9.6	0.14	36.77
2 multi	2	H						
128	1	11/08/2020	35	1068	2	9.9	0.07	37.36
2 multi	2	H						
128	1	11/09/2020	36	1069	2	9.9	0.12	36.06
2 multi	2	H						
128	1	11/10/2020	37	1070	2	9.3	0.19	35.83
2 multi	2	H						
128	1	11/11/2020	38	1071	2	9.8	0.07	39.26
2 multi	2	H						
128	1	11/12/2020	39	1072	2	9.8	0.04	34.61
2 multi	2	H						
128	1	11/13/2020	40	1073	2	9.6	0.03	38.76
2 multi	2	H						
128	1	11/14/2020	41	1074	2	9.6	0	35.48
2 multi	2	H						
128	1	11/15/2020	42	1075	2	9.6	0.07	36.9
2 multi	2	H						
128	1	11/16/2020	43	1076	2	9.8	0.03	38.87
2 multi	2	H						
128	1	11/17/2020	44	1077	2	9.8	0.02	36.35
2 multi	2	H						
128	1	11/18/2020	45	1078	2	9.6	0	37.28
2 multi	2	H						
128	1	11/19/2020	46	1079	2	9.8	0.03	38.13
2 multi	2	H						
128	1	11/20/2020	47	1080	2	9.3	0.09	40.05
2 multi	2	H						
128	1	11/21/2020	48	1081	2	9.6	0.18	36.54
2 multi	2	H						
128	1	11/22/2020	49	1082	2	9.5	0	39.36
2 multi	2	H						
128	1	11/23/2020	50	1083	2	9.8	0.01	43
2 multi	2	H						
128	1	11/24/2020	51	1084	2	9.8	0.35	41.27
2 multi	2	H						
128	1	11/25/2020	52	1085	2	9.5	0.08	36.48
2 multi	2	H						
128	1	11/26/2020	53	1086	2	9.5	0.09	42.06
2 multi	2	H						
128	1	11/27/2020	54	1087	2	9.8	0.08	40.46
2 multi	2	H						
128	1	11/28/2020	55	1088	2	9.6	0.08	39.46
2 multi	2	H						
128	1	11/29/2020	56	1089	2	9.6	0.07	40.32
2 multi	2	H						
128	1	11/30/2020	57	1090	2	9.5	0.1	40.54

2 multi 2 H
128 1 12/01/2020 58 1091 2 9.6 0.26 41.52
2 multi 2 H
128 1 12/02/2020 59 1092 2 9.5 0.01 39.07
2 multi 2 H
128 1 12/03/2020 60 1093 2 9.5 0.08 38.21
2 multi 2 H
128 1 12/04/2020 61 1094 2 9.5 0.09 40.21
2 multi 2 H
128 1 12/05/2020 62 1095 2 10.2 0.02 37.07
2 multi 2 H
128 1 12/06/2020 63 1096 2 10.2 0.11 34.93
2 multi 2 H
129 1 09/24/2018 5 757 1 10.4 0.33 25.18
1 primi 1 H
129 1 09/25/2018 6 758 1 10 0.12 28.07
1 primi 1 H
129 1 09/26/2018 7 759 1 10.2 0.22 29.24
1 primi 1 H
129 1 09/27/2018 8 760 1 9.9 0.15 29.4
1 primi 1 H
129 1 09/28/2018 9 761 1 10.6 0.06 29.58
1 primi 1 H
129 1 09/29/2018 10 762 1 9.8 0.06 30.98
1 primi 1 H
129 1 09/30/2018 11 763 1 10 0.11 32.81
1 primi 1 H
129 1 10/01/2018 12 764 1 10.3 0.15 30.87
1 primi 1 H
129 1 10/02/2018 13 765 1 10.4 0.03 32.27
1 primi 1 H
129 1 10/03/2018 14 766 1 9.9 0.04 34.28
1 primi 1 H
129 1 10/04/2018 15 767 1 10 0.03 34.58
1 primi 1 H
129 1 10/05/2018 16 768 1 10.2 0.04 34.83
1 primi 1 H
129 1 10/06/2018 17 769 1 10.2 0.03 33.81
1 primi 1 H
129 1 10/07/2018 18 770 1 10.4 0.03 35.81
1 primi 1 H
129 1 10/08/2018 19 771 1 9.6 0.08 34.11
1 primi 1 H
129 1 10/09/2018 20 772 1 9.6 0.14 35.83
1 primi 1 H
129 1 10/10/2018 21 773 1 9.9 0.08 34.66
1 primi 1 H
129 1 10/11/2018 22 774 1 10.3 0.16 37.3
1 primi 2 H
129 1 10/12/2018 23 775 1 9.8 0 36.36
1 primi 2 H
129 1 10/13/2018 24 776 1 9.6 0.13 35.54
1 primi 2 H
129 1 10/14/2018 25 777 1 9.9 0 35.71

1 primi	2	H						
129	1	10/15/2018	26	778	1	10.3	0	33.59
1 primi	2	H						
129	1	10/16/2018	27	779	1	9.8	0	31.79
1 primi	2	H						
129	1	10/17/2018	28	780	1	9.3	0.07	32.83
1 primi	2	H						
129	1	10/18/2018	29	781	1	9.5	0.02	30.34
1 primi	2	H						
129	1	10/19/2018	30	782	1	9.9	0.03	32.13
1 primi	2	H						
129	1	10/20/2018	31	783	1	9.6	0.37	32.92
1 primi	2	H						
129	1	10/21/2018	32	784	1	9.8	0.17	34.26
1 primi	2	H						
129	1	10/22/2018	33	785	1	9.5	0.1	33.77
1 primi	2	H						
129	1	10/23/2018	34	786	1	9.9	0.09	34.39
1 primi	2	H						
129	1	10/24/2018	35	787	1	9.9	0.24	34.21
1 primi	2	H						
129	1	10/25/2018	36	788	1	9.5	0.45	34.75
1 primi	2	H						
129	1	10/26/2018	37	789	1	9.6	0	34.54
1 primi	2	H						
129	1	10/27/2018	38	790	1	9.8	0.18	34.14
1 primi	2	H						
129	1	10/28/2018	39	791	1	10.3	0	37.27
1 primi	2	H						
129	1	10/29/2018	40	792	1	10.2	0	37.6
1 primi	2	H						
129	1	10/30/2018	41	793	1	10.3	0.23	36.61
1 primi	2	H						
129	1	10/31/2018	42	794	1	10.3	0.15	35.16
1 primi	2	H						
129	1	11/01/2018	43	795	1	10	0	37.23
1 primi	2	H						
129	1	11/02/2018	44	796	1	9.3	0	37
1 primi	2	H						
129	1	11/03/2018	45	797	1	9.8	0.06	37.65
1 primi	2	H						
129	1	11/04/2018	46	798	1	9.9	0	38.03
1 primi	2	H						
129	1	11/05/2018	47	799	1	10.2	0.03	38.48
1 primi	2	H						
129	1	11/06/2018	48	800	1	9.9	0.09	39.01
1 primi	2	H						
129	1	11/07/2018	49	801	1	9.9	0.06	38.82
1 primi	2	H						
129	1	11/08/2018	50	802	1	9.9	0.06	38.66
1 primi	2	H						
129	1	11/09/2018	51	803	1	9.9	0	33.98
1 primi	2	H						
129	1	11/10/2018	52	804	1	10.3	0	33.95

1 primi	2	H						
129	1	11/11/2018	53	805	1	9.5	0.25	34.03
1 primi	2	H						
129	1	11/12/2018	54	806	1	9.8	0.17	35.45
1 primi	2	H						
129	1	11/13/2018	55	807	1	9.9	0	37.11
1 primi	2	H						
129	1	11/14/2018	56	808	1	10.6	0	37.56
1 primi	2	H						
129	1	11/15/2018	57	809	1	9.9	0.05	37.28
1 primi	2	H						
129	1	11/16/2018	58	810	1	9.9	0.01	36.57
1 primi	2	H						
129	1	11/17/2018	59	811	1	9.9	0	38.26
1 primi	2	H						
129	1	11/18/2018	60	812	1	9.8	0	38.59
1 primi	2	H						
129	1	11/19/2018	61	813	1	9.6	0	37.18
1 primi	2	H						
129	1	11/20/2018	62	814	1	9.6	0	37.81
1 primi	2	H						
129	1	11/21/2018	63	815	1	9.9	0	37.01
1 primi	2	H						
129	1	11/22/2018	64	816	1	9.8	0	38.69
1 primi	2	H						
129	1	11/23/2018	65	817	1	9.9	0	37.56
1 primi	2	H						
129	1	11/24/2018	66	818	1	9.9	0	38.35
1 primi	2	H						
129	1	11/25/2018	67	819	1	9.8	0	39.28
1 primi	2	H						
129	1	11/26/2018	68	820	1	10.3	0	37.98
1 primi	2	H						
129	1	11/27/2018	69	821	1	10.4	0	36.06
1 primi	2	H						
129	1	11/28/2018	70	822	1	10	0	37.3
1 primi	2	H						
129	1	11/29/2018	71	823	1	9.8	0	39.01
1 primi	2	H						
129	1	11/30/2018	72	824	1	9.6	0	35.51
1 primi	2	H						
129	1	12/01/2018	73	825	1	9.9	0	37.01
1 primi	2	H						
129	1	12/02/2018	74	826	1	9.9	0	35.32
1 primi	2	H						
129	1	12/03/2018	75	827	1	10.4	0	37.31
1 primi	2	H						
129	1	12/04/2018	76	828	1	10.2	0	35.85
1 primi	2	H						
129	1	12/05/2018	77	829	1	9.8	0	36.76
1 primi	2	H						
129	1	12/06/2018	78	830	1	10.4	0	35.39
1 primi	2	H						
129	1	12/07/2018	79	831	1	10.2	0	36.74

1 primi 2		H						
129	1	12/08/2018	80	832	1	10.2	0	39.56
1 primi 2		H						
129	1	12/09/2018	81	833	1	10.2	0	36.14
1 primi 2		H						
129	1	12/10/2018	82	834	1	10.3	0	39.34
1 primi 2		H						
129	1	12/11/2018	83	835	1	10.3	0.1	37.11
1 primi 2		H						
129	1	12/12/2018	84	836	1	10.2	0	38.76
1 primi 2		H						
129	1	12/13/2018	85	837	1	10	0	34.77
1 primi 2		H						
129	1	12/14/2018	86	838	1	10.2	0	40.44
1 primi 2		H						
129	1	12/15/2018	87	839	1	10.4	0	39.39
1 primi 2		H						
129	1	12/16/2018	88	840	1	10.7	0	37.88
1 primi 2		H						
129	1	12/17/2018	89	841	1	10.7	0	37.08
1 primi 2		H						
129	1	08/19/2019	5	1086	2	9.8	0.18	24.31
2 multi 1		H						
129	1	08/20/2019	6	1087	2	9.5	0.24	29.94
2 multi 1		H						
129	1	08/21/2019	7	1088	2	9.6	0.16	27.87
2 multi 1		H						
129	1	08/22/2019	8	1089	2	9.8	0.11	25.53
2 multi 1		H						
129	1	08/23/2019	9	1090	2	9.9	0.3	27.59
2 multi 1		H						
129	1	08/24/2019	10	1091	2	9.6	0.03	28.77
2 multi 1		H						
129	1	08/25/2019	11	1092	2	9.9	0.04	30.76
2 multi 1		H						
129	1	08/26/2019	12	1093	2	9.9	0.24	28.34
2 multi 1		H						
129	1	08/27/2019	13	1094	2	9.9	0.05	32.7
2 multi 1		H						
129	1	08/28/2019	14	1095	2	10.2	0.21	31.66
2 multi 1		H						
129	1	08/29/2019	15	1096	2	10.2	0.16	33.32
2 multi 1		H						
129	1	08/30/2019	16	1097	2	9.9	0.15	34.03
2 multi 1		H						
129	1	08/31/2019	17	1098	2	9.6	0.01	34.75
2 multi 1		H						
129	1	09/01/2019	18	1099	2	9.8	0	35.04
2 multi 1		H						
129	1	09/02/2019	19	1100	2	9.8	0.06	34.78
2 multi 1		H						
129	1	09/03/2019	20	1101	2	10	0.18	35.75
2 multi 1		H						
129	1	09/04/2019	21	1102	2	9.6	0.16	35.04

2 multi	1	H						
129	1	09/05/2019	22	1103	2	9.8	0.27	35.71
2 multi	2	H						
129	1	09/06/2019	23	1104	2	9.5	0.34	36.47
2 multi	2	H						
129	1	09/07/2019	24	1105	2	9.5	0.14	36.14
2 multi	2	H						
129	1	09/08/2019	25	1106	2	9.3	0	35.04
2 multi	2	H						
129	1	09/09/2019	26	1107	2	9.6	0.16	38.38
2 multi	2	H						
129	1	09/10/2019	27	1108	2	9.5	0.48	38.71
2 multi	2	H						
129	1	09/11/2019	28	1109	2	9.6	0	40.51
2 multi	2	H						
129	1	09/12/2019	29	1110	2	9.8	0.02	39.22
2 multi	2	H						
129	1	12/17/2019	125	1206	2	9.8	0.19	35.26
2 multi	2	H						
129	1	12/18/2019	126	1207	2	9.5	0	36.41
2 multi	2	H						
129	1	12/19/2019	127	1208	2	9.9	0	30.11
2 multi	2	H						
129	1	12/20/2019	128	1209	2	9.9	0	35.22
2 multi	2	H						
129	1	12/21/2019	129	1210	2	9.2	0	34.63
2 multi	2	H						
129	1	12/22/2019	130	1211	2	9.3	0.02	31.84
2 multi	2	H						
129	1	12/23/2019	131	1212	2	9.5	0	33.32
2 multi	2	H						
129	1	12/24/2019	132	1213	2	10	0	36.35
2 multi	2	H						
129	1	12/25/2019	133	1214	2	10	0	35.09
2 multi	2	H						
129	1	12/26/2019	134	1215	2	10.3	0.12	31.76
2 multi	2	H						
129	1	12/27/2019	135	1216	2	9.6	0	33.82
2 multi	2	H						
129	1	12/28/2019	136	1217	2	9.9	0.02	33.8
2 multi	2	H						
129	1	12/29/2019	137	1218	2	9.9	0	36.85
2 multi	2	H						
129	1	12/30/2019	138	1219	2	9.1	0	38.84
2 multi	2	H						
129	1	12/31/2019	139	1220	2	9.8	0	37.09
2 multi	2	H						
129	1	01/01/2020	140	1221	2	9.6	0.06	37.83
2 multi	2	H						
129	1	01/02/2020	141	1222	2	9.8	0	35.29
2 multi	2	H						
129	1	01/03/2020	142	1223	2	9.8	0.54	34.36
2 multi	2	H						
129	1	01/04/2020	143	1224	2	9.6	0	34.28

2 multi	2	H						
129	1	01/05/2020	144	1225	2	9.6	0	35.4
2 multi	2	H						
129	1	01/06/2020	145	1226	2	10	0	31.98
2 multi	2	H						
129	1	01/07/2020	146	1227	2	9.8	0	33.36
2 multi	2	H						
129	1	01/08/2020	147	1228	2	9.9	0	37.33
2 multi	2	H						
129	1	01/09/2020	148	1229	2	10.3	0	35.93
2 multi	2	H						
129	1	01/10/2020	149	1230	2	10	0	34.72
2 multi	2	H						
129	1	10/11/2020	6	1505	3	9.6	0.15	36.99
2 multi	1	H						
129	1	10/12/2020	7	1506	3	10.4	0.07	33.72
2 multi	1	H						
129	1	10/13/2020	8	1507	3	10.3	0.05	38.69
2 multi	1	H						
130	1	10/17/2016	8	818	1	9.3	0	25.24
1 primi	1	H						
130	1	10/18/2016	9	819	1	9.5	0	24.69
1 primi	1	H						
130	1	10/19/2016	10	820	1	9.3	0	25.82
1 primi	1	H						
130	1	10/20/2016	11	821	1	9.3	0.56	25.7
1 primi	1	H						
130	1	10/21/2016	12	822	1	9.2	0	25.99
1 primi	1	H						
130	1	10/22/2016	13	823	1	9.2	0	25.45
1 primi	1	H						
130	1	10/23/2016	14	824	1	9.3	0.06	28.47
1 primi	1	H						
130	1	10/24/2016	15	825	1	9.5	0	26.28
1 primi	1	H						
130	1	10/25/2016	16	826	1	9.5	0	28.93
1 primi	1	H						
130	1	10/26/2016	17	827	1	9.5	0.05	29.72
1 primi	1	H						
130	1	10/27/2016	18	828	1	9.5	0.15	31.2
1 primi	1	H						
130	1	10/28/2016	19	829	1	9.6	0	31.56
1 primi	1	H						
130	1	11/01/2016	23	833	1	9.3	0.02	32.37
1 primi	2	H						
130	1	11/02/2016	24	834	1	9.1	0.11	32.09
1 primi	2	H						
130	1	11/03/2016	25	835	1	9.2	0	32.9
1 primi	2	H						
130	1	11/04/2016	26	836	1	9.2	0.13	31.52
1 primi	2	H						
130	1	11/05/2016	27	837	1	9.3	0.12	34.33
1 primi	2	H						
130	1	11/06/2016	28	838	1	9.3	0	33.79

1 primi 2 H
130 1 11/07/2016 29 839 1 9.3 0.09 35.21
1 primi 2 H
130 1 11/08/2016 30 840 1 9.2 0 32.53
1 primi 2 H
130 1 11/09/2016 31 841 1 9.3 0 31.92
1 primi 2 H
130 1 11/10/2016 32 842 1 9.5 0 34.6
1 primi 2 H
130 1 11/11/2016 33 843 1 9.2 0 34.34
1 primi 2 H
130 1 11/12/2016 34 844 1 9.2 0.12 33.12
1 primi 2 H
130 1 11/13/2016 35 845 1 9.5 0.09 32.96
1 primi 2 H
130 1 11/14/2016 36 846 1 9.6 0.08 33.36
1 primi 2 H
130 1 11/15/2016 37 847 1 9.5 0 32.27
1 primi 2 H
130 1 11/16/2016 38 848 1 9.6 0 34.32
1 primi 2 H
130 1 11/17/2016 39 849 1 9.6 0 33.35
1 primi 2 H
130 1 11/18/2016 40 850 1 9.5 0.16 30.9
1 primi 2 H
130 1 11/19/2016 41 851 1 9.3 0.12 33.21
1 primi 2 H
130 1 11/20/2016 42 852 1 9.3 0 33.15
1 primi 2 H
130 1 11/21/2016 43 853 1 9.3 0 33.06
1 primi 2 H
130 1 11/22/2016 44 854 1 9.5 0 34.59
1 primi 2 H
130 1 11/23/2016 45 855 1 9.3 0 32.41
1 primi 2 H
130 1 11/24/2016 46 856 1 9.8 0.12 32.83
1 primi 2 H
130 1 11/25/2016 47 857 1 9.5 0 33.96
1 primi 2 H
130 1 11/26/2016 48 858 1 9.6 0.01 34.56
1 primi 2 H
130 1 11/27/2016 49 859 1 9.6 0 33.9
1 primi 2 H
130 1 11/28/2016 50 860 1 9.6 0 33.37
1 primi 2 H
130 1 11/29/2016 51 861 1 9.8 0 34.59
1 primi 2 H
130 1 11/30/2016 52 862 1 9.6 0 34.33
1 primi 2 H
130 1 12/01/2016 53 863 1 9.3 0 32.03
1 primi 2 H
130 1 12/02/2016 54 864 1 9.8 0 32.44
1 primi 2 H
130 1 12/03/2016 55 865 1 9.8 0 33.48

1 primi	2	H						
130	1	12/04/2016	56	866	1	9.6	0	35.26
1 primi	2	H						
130	1	12/05/2016	57	867	1	9.5	0	32.99
1 primi	2	H						
130	1	12/06/2016	58	868	1	9.6	0	34.1
1 primi	2	H						
130	1	12/07/2016	59	869	1	10	0	33.8
1 primi	2	H						
130	1	12/08/2016	60	870	1	9.8	0.11	35.09
1 primi	2	H						
130	1	12/09/2016	61	871	1	9.8	0	34.72
1 primi	2	H						
130	1	12/10/2016	62	872	1	9.6	0	34.01
1 primi	2	H						
130	1	12/11/2016	63	873	1	9.8	0	34.89
1 primi	2	H						
130	1	12/12/2016	64	874	1	9.6	0	32.34
1 primi	2	H						
130	1	12/13/2016	65	875	1	9.6	0	32.72
1 primi	2	H						
130	1	12/14/2016	66	876	1	10.2	0	32.41
1 primi	2	H						
130	1	12/15/2016	67	877	1	9.9	0	32.55
1 primi	2	H						
130	1	12/16/2016	68	878	1	9.9	0	31.37
1 primi	2	H						
130	1	12/17/2016	69	879	1	9.9	0	32.85
1 primi	2	H						
130	1	12/18/2016	70	880	1	10.2	0	33.42
1 primi	2	H						
130	1	12/19/2016	71	881	1	10.2	0	33.39
1 primi	2	H						
130	1	12/20/2016	72	882	1	9.9	0.01	34.17
1 primi	2	H						
130	1	12/21/2016	73	883	1	10	0	33.59
1 primi	2	H						
130	1	12/22/2016	74	884	1	10	0	34.24
1 primi	2	H						
130	1	12/23/2016	75	885	1	9.9	0	35.15
1 primi	2	H						
130	1	12/24/2016	76	886	1	9.9	0	33.61
1 primi	2	H						
130	1	12/25/2016	77	887	1	10	0	36.76
1 primi	2	H						
130	1	12/26/2016	78	888	1	9.9	0	35.2
1 primi	2	H						
130	1	12/27/2016	79	889	1	9.8	0	36.88
1 primi	2	H						
130	1	12/28/2016	80	890	1	9.8	0	36.04
1 primi	2	H						
130	1	12/29/2016	81	891	1	9.8	0	33.96
1 primi	2	H						
130	1	12/30/2016	82	892	1	9.6	0	34.6

1 primi	2	H						
130	1	12/31/2016	83	893	1	9.5	0	34.22
1 primi	2	H						
130	1	01/01/2017	84	894	1	9.6	0	36.04
1 primi	2	H						
130	1	01/02/2017	85	895	1	9.6	0	35.65
1 primi	2	H						
130	1	01/03/2017	86	896	1	9.6	0	33.47
1 primi	2	H						
130	1	01/04/2017	87	897	1	9.5	0	34.19
1 primi	2	H						
130	1	01/05/2017	88	898	1	9.6	0.07	35.85
1 primi	2	H						
130	1	01/06/2017	89	899	1	9.5	0.23	35.32
1 primi	2	H						
130	1	01/07/2017	90	900	1	9.3	0.1	34.7
1 primi	2	H						
130	1	01/08/2017	91	901	1	9.5	0.01	33.96
1 primi	2	H						
130	1	01/09/2017	92	902	1	9.5	0	36.29
1 primi	2	H						
130	1	01/10/2017	93	903	1	9.5	0	36.83
1 primi	2	H						
130	1	01/11/2017	94	904	1	9.3	0	37.22
1 primi	2	H						
130	1	01/12/2017	95	905	1	9.5	0	38.49
1 primi	2	H						
130	1	01/13/2017	96	906	1	9.2	0	35.89
1 primi	2	H						
130	1	01/14/2017	97	907	1	9.5	0	37.34
1 primi	2	H						
130	1	01/15/2017	98	908	1	9.6	0	35.87
1 primi	2	H						
130	1	01/16/2017	99	909	1	9.5	0	36.7
1 primi	2	H						
130	1	01/17/2017	100	910	1	9.5	0	40.37
1 primi	2	H						
130	1	01/18/2017	101	911	1	9.3	0	37.03
1 primi	2	H						
130	1	01/19/2017	102	912	1	9.5	0	37.7
1 primi	2	H						
130	1	01/20/2017	103	913	1	9.3	0.06	38.02
1 primi	2	H						
130	1	01/21/2017	104	914	1	9.6	0	38.37
1 primi	2	H						
130	1	01/22/2017	105	915	1	8.9	0	36.45
1 primi	2	H						
130	1	01/23/2017	106	916	1	9.1	0	38.48
1 primi	2	H						
130	1	01/24/2017	107	917	1	9.2	0	37.95
1 primi	2	H						
130	1	01/25/2017	108	918	1	9.5	0	37.53
1 primi	2	H						
130	1	01/26/2017	109	919	1	9.6	0	38.63

1 primi 2 H
130 1 01/27/2017 110 920 1 9.6 0 38.66
1 primi 2 H
130 1 01/28/2017 111 921 1 9.3 0 37.47
1 primi 2 H
130 1 01/29/2017 112 922 1 9.5 0 37.71
1 primi 2 H
130 1 01/30/2017 113 923 1 9.5 0 37.36
1 primi 2 H
130 1 01/31/2017 114 924 1 9.3 0 38.96
1 primi 2 H
130 1 02/01/2017 115 925 1 9.5 0 37.75
1 primi 2 H
130 1 02/02/2017 116 926 1 9.5 0.05 37.95
1 primi 2 H
130 1 02/03/2017 117 927 1 9.5 0 38.14
1 primi 2 H
130 1 02/04/2017 118 928 1 9.5 0 36.49
1 primi 2 H
130 1 02/05/2017 119 929 1 9.3 0 38.64
1 primi 2 H
130 1 02/06/2017 120 930 1 9.6 0 36.68
1 primi 2 H
130 1 02/07/2017 121 931 1 9.2 0 38.11
1 primi 2 H
130 1 02/08/2017 122 932 1 9.5 0 38.88
1 primi 2 H
130 1 02/09/2017 123 933 1 9.5 0.05 36.69
1 primi 2 H
130 1 02/10/2017 124 934 1 9.8 0 36.75
1 primi 2 H
130 1 02/11/2017 125 935 1 9.6 0 36.19
1 primi 2 H
130 1 02/12/2017 126 936 1 9.5 0.12 36.61
1 primi 2 H
130 1 02/13/2017 127 937 1 9.1 0 34.68
1 primi 2 H
130 1 02/14/2017 128 938 1 8.9 0 34.39
1 primi 2 H
130 1 02/15/2017 129 939 1 9.3 0.12 36.89
1 primi 2 H
130 1 02/16/2017 130 940 1 9.6 0.09 37.86
1 primi 2 H
130 1 02/17/2017 131 941 1 9.3 0.04 36.06
1 primi 2 H
130 1 02/18/2017 132 942 1 9.6 0.2 35.02
1 primi 2 H
130 1 02/19/2017 133 943 1 9.8 0.17 35.63
1 primi 2 H
130 1 02/20/2017 134 944 1 9.6 0.04 35.27
1 primi 2 H
130 1 02/21/2017 135 945 1 9.8 0 35.69
1 primi 2 H
130 1 02/22/2017 136 946 1 9.6 0.22 35.32

1 primi 2 H
130 1 02/23/2017 137 947 1 9.8 0.05 35.88
1 primi 2 H
130 1 02/24/2017 138 948 1 9.8 0.22 36.25
1 primi 2 H
130 1 02/25/2017 139 949 1 9.5 0.02 35.53
1 primi 2 H
130 1 02/26/2017 140 950 1 9.6 0.13 38.2
1 primi 2 H
130 1 02/27/2017 141 951 1 9.8 0.03 39.1
1 primi 2 H
130 1 02/28/2017 142 952 1 9.8 0.15 36.36
1 primi 2 H
130 1 03/01/2017 143 953 1 9.6 0.1 38.31
1 primi 2 H
130 1 03/02/2017 144 954 1 9.6 0 33.35
1 primi 2 H
130 1 03/03/2017 145 955 1 9.8 0 36.3
1 primi 2 H
130 1 03/04/2017 146 956 1 9.6 0 33.22
1 primi 2 H
130 1 03/05/2017 147 957 1 9.8 0 37.43
1 primi 2 H
130 1 03/06/2017 148 958 1 9.8 0 37.04
1 primi 2 H
130 1 03/07/2017 149 959 1 9.5 0 33.62
1 primi 2 H
130 1 03/08/2017 150 960 1 9.9 0 33.63
1 primi 2 H
130 1 03/09/2017 151 961 1 9.9 0 34.27
1 primi 2 H
130 1 03/19/2018 199 1336 2 10.9 0 46.72
2 multi 2 H
130 1 03/20/2018 200 1337 2 10.6 0 44.94
2 multi 2 H
130 1 03/21/2018 201 1338 2 10.9 0 45.06
2 multi 2 H
130 1 03/22/2018 202 1339 2 11.1 0 44.64
2 multi 2 H
130 1 03/23/2018 203 1340 2 11.4 0 45.98
2 multi 2 H
130 1 03/24/2018 204 1341 2 11.3 0 43.54
2 multi 2 H
130 1 03/25/2018 205 1342 2 11.1 0 39.55
2 multi 2 H
130 1 03/26/2018 206 1343 2 10.7 0 45.92
2 multi 2 H
130 1 03/27/2018 207 1344 2 11.4 0 40.85
2 multi 2 H
130 1 03/28/2018 208 1345 2 11.3 0.17 45.39
2 multi 2 H
130 1 03/29/2018 209 1346 2 10.6 0.5 47
2 multi 2 H
130 1 03/30/2018 210 1347 2 10.7 0 46

2 multi	2	H						
130	1	03/31/2018	211	1348	2	10	0	42.72
2 multi	2	H						
130	1	04/01/2018	212	1349	2	10.4	0	40.38
2 multi	2	H						
130	1	04/02/2018	213	1350	2	11.7	0.01	41.82
2 multi	2	H						
130	1	04/03/2018	214	1351	2	11.1	0	43.07
2 multi	2	H						
130	1	04/04/2018	215	1352	2	10.2	0	44.82
2 multi	2	H						
130	1	04/05/2018	216	1353	2	10.3	0	40.46
2 multi	2	H						
130	1	04/06/2018	217	1354	2	10.7	0	46.94
2 multi	2	H						
130	1	04/07/2018	218	1355	2	11.4	0.08	41.66
2 multi	2	H						
130	1	04/08/2018	219	1356	2	10.6	0	42.68
2 multi	2	H						
130	1	04/09/2018	220	1357	2	10.6	0	42.25
2 multi	2	H						
130	1	04/10/2018	221	1358	2	11.7	0	42.47
2 multi	2	H						
130	1	04/11/2018	222	1359	2	10.9	0	43.03
2 multi	2	H						
130	1	04/12/2018	223	1360	2	11.4	0	40.44
2 multi	2	H						
130	1	04/13/2018	224	1361	2	11.1	0	41.23
2 multi	2	H						
130	1	04/14/2018	225	1362	2	10.6	0	38.81
2 multi	2	H						
130	1	04/15/2018	226	1363	2	10	0	42.89
2 multi	2	H						
130	1	04/16/2018	227	1364	2	10.9	0	35.89
2 multi	2	H						
130	1	04/17/2018	228	1365	2	10.3	0.53	34.33
2 multi	2	H						
130	1	04/18/2018	229	1366	2	0	0	36.81
2 multi	2	H						
130	1	04/19/2018	230	1367	2	11.1	0.16	38.55
2 multi	2	H						
130	1	04/20/2018	231	1368	2	11.4	0	37.46
2 multi	2	H						
130	1	04/21/2018	232	1369	2	11.3	0	38.89
2 multi	2	H						
130	1	04/22/2018	233	1370	2	10.9	0	39.83
2 multi	2	H						
130	1	04/23/2018	234	1371	2	10.9	0.37	40.47
2 multi	2	H						
130	1	04/24/2018	235	1372	2	11.3	0	38.89
2 multi	2	H						
130	1	04/25/2018	236	1373	2	11.3	0	40.21
2 multi	2	H						
130	1	04/26/2018	237	1374	2	11.4	0	37.16

2 multi 2 H
130 1 04/27/2018 238 1375 2 10.7 0 38.93
2 multi 2 H
130 1 04/28/2018 239 1376 2 11.4 0 41.45
2 multi 2 H
130 1 04/29/2018 240 1377 2 11.4 0 42.03
2 multi 2 H
130 1 04/30/2018 241 1378 2 12 0.1 42.4
2 multi 2 H
130 1 05/01/2018 242 1379 2 11.3 0 41.68
2 multi 2 H
130 1 05/02/2018 243 1380 2 11.1 0 42.75
2 multi 2 H
130 1 05/03/2018 244 1381 2 11.7 0 41.56
2 multi 2 H
130 1 05/04/2018 245 1382 2 11.3 0 37.52
2 multi 2 H
130 1 05/05/2018 246 1383 2 11.3 0.21 38.56
2 multi 2 H
130 1 05/06/2018 247 1384 2 11.1 0 37.79
2 multi 2 H
130 1 08/16/2018 5 1486 3 9.8 0.14 26.9
2 multi 1 H
130 1 08/17/2018 6 1487 3 9.8 0.19 33.12
2 multi 1 H
130 1 08/18/2018 7 1488 3 9.6 0.01 33.45
2 multi 1 H
130 1 08/19/2018 8 1489 3 9.3 0.02 30.23
2 multi 1 H
130 1 08/20/2018 9 1490 3 9.8 0 30.41
2 multi 1 H
130 1 08/21/2018 10 1491 3 10 0.05 30.25
2 multi 1 H
130 1 08/22/2018 11 1492 3 10.2 0.25 32.18
2 multi 1 H
130 1 08/23/2018 12 1493 3 10.3 0.25 39.17
2 multi 1 H
130 1 08/24/2018 13 1494 3 10.2 0 35.7
2 multi 1 H
130 1 08/25/2018 14 1495 3 10.2 0 40.11
2 multi 1 H
130 1 08/26/2018 15 1496 3 10.2 0 40.93
2 multi 1 H
130 1 08/27/2018 16 1497 3 10.3 0 40.03
2 multi 1 H
130 1 08/28/2018 17 1498 3 10.3 0 41.29
2 multi 1 H
130 1 08/29/2018 18 1499 3 10.2 0 45.5
2 multi 1 H
130 1 08/30/2018 19 1500 3 10.2 0.09 44.99
2 multi 1 H
130 1 08/31/2018 20 1501 3 10 0.05 44.42
2 multi 1 H
130 1 09/01/2018 21 1502 3 10 0 46.82

2 multi	1	H						
130	1	09/02/2018	22	1503	3	10.4	0	46.32
2 multi	2	H						
130	1	09/03/2018	23	1504	3	10	0.07	46.51
2 multi	2	H						
130	1	09/04/2018	24	1505	3	10.3	0	48.62
2 multi	2	H						
130	1	09/05/2018	25	1506	3	10.2	0	45.15
2 multi	2	H						
130	1	09/06/2018	26	1507	3	10.2	0.27	48.31
2 multi	2	H						
130	1	09/07/2018	27	1508	3	10.2	0.31	47.36
2 multi	2	H						
130	1	09/08/2018	28	1509	3	10.3	0	49.43
2 multi	2	H						
130	1	09/09/2018	29	1510	3	10.3	0	47.89
2 multi	2	H						
130	1	09/10/2018	30	1511	3	10.2	0	46.94
2 multi	2	H						
130	1	09/11/2018	31	1512	3	10.2	0	45.04
2 multi	2	H						
130	1	09/12/2018	32	1513	3	9.9	0	51.26
2 multi	2	H						
130	1	09/13/2018	33	1514	3	10.2	0	45.11
2 multi	2	H						
130	1	09/14/2018	34	1515	3	9.9	0	45.81
2 multi	2	H						
130	1	09/15/2018	35	1516	3	10.2	0	46.34
2 multi	2	H						
130	1	09/16/2018	36	1517	3	10	0	45.81
2 multi	2	H						
130	1	09/17/2018	37	1518	3	10	0	44.94
2 multi	2	H						
130	1	09/18/2018	38	1519	3	10.2	0	46.52
2 multi	2	H						
130	1	09/19/2018	39	1520	3	10.2	0	48.5
2 multi	2	H						
130	1	09/20/2018	40	1521	3	10.3	0	46.64
2 multi	2	H						
130	1	09/21/2018	41	1522	3	10.2	0	42.99
2 multi	2	H						
130	1	09/22/2018	42	1523	3	10.2	0.05	45.95
2 multi	2	H						
130	1	09/23/2018	43	1524	3	10.2	0	44.41
2 multi	2	H						
130	1	09/24/2018	44	1525	3	10.2	0	43.36
2 multi	2	H						
130	1	09/25/2018	45	1526	3	10.2	0	41.22
2 multi	2	H						
130	1	09/26/2018	46	1527	3	10	0	39.12
2 multi	2	H						
130	1	09/27/2018	47	1528	3	10.4	0.68	43.35
2 multi	2	H						
130	1	09/28/2018	48	1529	3	10.3	0	45.47

2 multi	2	H						
130	1	09/29/2018	49	1530	3	10.3	0	39.49
2 multi	2	H						
130	1	09/30/2018	50	1531	3	9.5	0	41.24
2 multi	2	H						
130	1	07/14/2019	10	1818	4	10.2	0.05	32.19
2 multi	1	H						
130	1	07/15/2019	11	1819	4	10.2	0.01	35.97
2 multi	1	H						
130	1	07/16/2019	12	1820	4	10.6	0.15	36.56
2 multi	1	H						
130	1	07/17/2019	13	1821	4	10.4	0	40.35
2 multi	1	H						
130	1	07/18/2019	14	1822	4	10.3	0.02	41.73
2 multi	1	H						
130	1	07/19/2019	15	1823	4	10.4	0	40.29
2 multi	1	H						
130	1	07/20/2019	16	1824	4	10	0.01	43.69
2 multi	1	H						
130	1	07/21/2019	17	1825	4	9.9	0.02	45.49
2 multi	1	H						
130	1	07/22/2019	18	1826	4	10	0	47.38
2 multi	1	H						
130	1	07/23/2019	19	1827	4	10	0	49.87
2 multi	1	H						
130	1	07/24/2019	20	1828	4	10	0	47.35
2 multi	1	H						
130	1	07/25/2019	21	1829	4	9.9	0	50.69
2 multi	1	H						
130	1	07/26/2019	22	1830	4	10.2	0.22	49.87
2 multi	2	H						
130	1	07/27/2019	23	1831	4	10.2	0	50.09
2 multi	2	H						
130	1	07/28/2019	24	1832	4	10	0.06	49
2 multi	2	H						
130	1	07/29/2019	25	1833	4	10.2	0	50.13
2 multi	2	H						
130	1	07/30/2019	26	1834	4	10	0.16	49.21
2 multi	2	H						
130	1	07/31/2019	27	1835	4	10	0	50.49
2 multi	2	H						
130	1	08/01/2019	28	1836	4	10	0	50.64
2 multi	2	H						
130	1	08/02/2019	29	1837	4	10.2	0	48.73
2 multi	2	H						
130	1	08/03/2019	30	1838	4	9.9	0.01	51.8
2 multi	2	H						
130	1	08/04/2019	31	1839	4	9.9	0	49.42
2 multi	2	H						
130	1	08/05/2019	32	1840	4	10	0	52.89
2 multi	2	H						
130	1	08/06/2019	33	1841	4	10	0	48.38
2 multi	2	H						
130	1	08/07/2019	34	1842	4	9.9	0	52.33

2 multi	2	H						
130	1	08/08/2019	35	1843	4	9.9	0	48.58
2 multi	2	H						
130	1	08/09/2019	36	1844	4	10.2	0	51.41
2 multi	2	H						
130	1	08/10/2019	37	1845	4	10	0	51.66
2 multi	2	H						
130	1	08/11/2019	38	1846	4	10.2	0	47.3
2 multi	2	H						
130	1	08/12/2019	39	1847	4	10	0.01	54.39
2 multi	2	H						
130	1	08/13/2019	40	1848	4	10.2	0	46.02
2 multi	2	H						
130	1	08/14/2019	41	1849	4	10	0	49.4
2 multi	2	H						
130	1	08/15/2019	42	1850	4	9.9	0.01	47.36
2 multi	2	H						
130	1	08/16/2019	43	1851	4	10.3	0.01	51.14
2 multi	2	H						
130	1	08/17/2019	44	1852	4	10	0.01	47.51
2 multi	2	H						
130	1	08/18/2019	45	1853	4	10.2	0.14	46.57
2 multi	2	H						
130	1	08/19/2019	46	1854	4	10.2	0.3	48.03
2 multi	2	H						
130	1	08/20/2019	47	1855	4	10.4	0.28	49.05
2 multi	2	H						
130	1	08/21/2019	48	1856	4	9.8	0.24	45.5
2 multi	2	H						
130	1	08/22/2019	49	1857	4	10	0.12	43.56
2 multi	2	H						
130	1	08/23/2019	50	1858	4	10	0.11	47.46
2 multi	2	H						
130	1	08/24/2019	51	1859	4	10.2	0	47.71
2 multi	2	H						
130	1	08/25/2019	52	1860	4	10	0.59	46.85
2 multi	2	H						
130	1	08/26/2019	53	1861	4	10.2	0	47.29
2 multi	2	H						
130	1	08/27/2019	54	1862	4	10.3	0.53	47.51
2 multi	2	H						
130	1	08/28/2019	55	1863	4	10.3	0.41	44.16
2 multi	2	H						
130	1	08/29/2019	56	1864	4	10.4	0	47.35
2 multi	2	H						
130	1	08/30/2019	57	1865	4	10.3	0	43.67
2 multi	2	H						
130	1	08/31/2019	58	1866	4	10.3	0.03	45.96
2 multi	2	H						
130	1	09/01/2019	59	1867	4	10.2	0.18	44.72
2 multi	2	H						
130	1	09/02/2019	60	1868	4	10.3	0	42.93
2 multi	2	H						
130	1	09/03/2019	61	1869	4	10.2	0.36	44.87

2 multi 2		H						
130	1	09/04/2019	62	1870	4	9.9	0.02	44.21
2 multi 2		H						
130	1	09/05/2019	63	1871	4	10	0	44.5
2 multi 2		H						
130	1	09/06/2019	64	1872	4	10.3	0.27	45.05
2 multi 2		H						
130	1	09/07/2019	65	1873	4	10.3	0	43.58
2 multi 2		H						
130	1	09/08/2019	66	1874	4	10.3	0	43.8
2 multi 2		H						
130	1	09/09/2019	67	1875	4	10.3	0	40.34
2 multi 2		H						
130	1	09/10/2019	68	1876	4	10.3	0.01	41.21
2 multi 2		H						
130	1	09/11/2019	69	1877	4	9.9	0	43.4
2 multi 2		H						
130	1	09/12/2019	70	1878	4	10.2	0	41.66
2 multi 2		H						
132	1	06/17/2020	10	764	1	9.5	0.28	16.2
1 primi 1		C						
132	1	06/18/2020	11	765	1	9.8	0.09	17.49
1 primi 1		C						
132	1	06/19/2020	12	766	1	10	0.18	16.78
1 primi 1		C						
132	1	06/20/2020	13	767	1	9.8	0.16	17.45
1 primi 1		C						
132	1	06/21/2020	14	768	1	9.6	0	17.47
1 primi 1		C						
132	1	06/22/2020	15	769	1	9.5	0.01	16.35
1 primi 1		C						
132	1	06/23/2020	16	770	1	9.8	0.07	19.72
1 primi 1		C						
132	1	06/24/2020	17	771	1	10	0.04	20.47
1 primi 1		C						
132	1	06/25/2020	18	772	1	9.9	0	20.86
1 primi 1		C						
132	1	06/26/2020	19	773	1	9.8	0	19.03
1 primi 1		C						
132	1	06/27/2020	20	774	1	9.8	0	20.38
1 primi 1		C						
132	1	06/28/2020	21	775	1	9.8	0	20.22
1 primi 1		C						
132	1	06/29/2020	22	776	1	9.8	0	20.16
1 primi 2		C						
132	1	06/30/2020	23	777	1	9.8	0	21.58
1 primi 2		C						
132	1	07/01/2020	24	778	1	9.5	0	20.05
1 primi 2		C						
132	1	07/02/2020	25	779	1	9.8	0	23.08
1 primi 2		C						
132	1	07/03/2020	26	780	1	9.6	0.18	22.79
1 primi 2		C						
132	1	07/04/2020	27	781	1	9.6	0.14	23

1 primi	2	C						
132	1	07/05/2020	28	782	1	9.5	0.22	20.75
1 primi	2	C						
132	1	07/06/2020	29	783	1	9.6	0.21	22.45
1 primi	2	C						
132	1	07/07/2020	30	784	1	9.6	0.15	22.08
1 primi	2	C						
132	1	07/08/2020	31	785	1	10	0.1	22.46
1 primi	2	C						
132	1	07/09/2020	32	786	1	9.8	0	24.3
1 primi	2	C						
132	1	07/10/2020	33	787	1	9.9	0.04	24.04
1 primi	2	C						
132	1	07/11/2020	34	788	1	9.9	0.23	24.98
1 primi	2	C						
132	1	07/12/2020	35	789	1	9.8	0.06	23.01
1 primi	2	C						
132	1	07/13/2020	36	790	1	9.9	0.17	22.28
1 primi	2	C						
132	1	07/14/2020	37	791	1	9.9	0	24.39
1 primi	2	C						
132	1	07/15/2020	38	792	1	9.8	0	23.7
1 primi	2	C						
132	1	07/16/2020	39	793	1	9.6	0	22.56
1 primi	2	C						
132	1	07/17/2020	40	794	1	9.9	0.25	26.97
1 primi	2	C						
132	1	07/18/2020	41	795	1	10	0.11	25.09
1 primi	2	C						
132	1	07/19/2020	42	796	1	9.6	0.27	25.48
1 primi	2	C						
132	1	07/20/2020	43	797	1	9.8	0	25.91
1 primi	2	C						
132	1	07/21/2020	44	798	1	9.9	0.04	25.67
1 primi	2	C						
132	1	07/22/2020	45	799	1	9.6	0.2	25.07
1 primi	2	C						
132	1	07/23/2020	46	800	1	9.9	0.11	25.24
1 primi	2	C						
132	1	07/24/2020	47	801	1	9.6	0.28	24.75
1 primi	2	C						
132	1	07/25/2020	48	802	1	9.9	0.26	26.78
1 primi	2	C						
132	1	07/26/2020	49	803	1	9.8	0.3	26.34
1 primi	2	C						
132	1	07/27/2020	50	804	1	9.9	0.27	25.85
1 primi	2	C						
132	1	07/28/2020	51	805	1	9.8	0.11	24.7
1 primi	2	C						
132	1	07/29/2020	52	806	1	10	0.09	26.71
1 primi	2	C						
132	1	07/30/2020	53	807	1	9.9	0.15	24.37
1 primi	2	C						
132	1	07/31/2020	54	808	1	9.8	0.04	25.74

1 primi 2		C						
132	1	08/01/2020	55	809	1	9.9	0.13	24.78
1 primi 2		C						
132	1	08/02/2020	56	810	1	9.9	0.18	25
1 primi 2		C						
132	1	08/03/2020	57	811	1	10	0.05	25.93
1 primi 2		C						
132	1	08/04/2020	58	812	1	9.8	0.34	23.97
1 primi 2		C						
132	1	08/05/2020	59	813	1	9.9	0.04	27.06
1 primi 2		C						
132	1	08/06/2020	60	814	1	9.9	0.06	27.59
1 primi 2		C						
132	1	08/07/2020	61	815	1	9.9	0.11	27.69
1 primi 2		C						
132	1	08/08/2020	62	816	1	10.2	0.13	27.08
1 primi 2		C						
132	1	08/09/2020	63	817	1	10.2	0.05	27.26
1 primi 2		C						
133	1	03/01/2020	5	751	1	9.9	0.25	18.16
1 primi 1		H						
133	1	03/02/2020	6	752	1	10	0.13	20.88
1 primi 1		H						
133	1	03/03/2020	7	753	1	9.8	0.12	22.03
1 primi 1		H						
133	1	03/04/2020	8	754	1	9.6	0.03	20.97
1 primi 1		H						
133	1	03/05/2020	9	755	1	9.6	0.04	21.27
1 primi 1		H						
133	1	03/06/2020	10	756	1	9.3	0.12	22.94
1 primi 1		H						
133	1	03/07/2020	11	757	1	9.8	0.05	24.17
1 primi 1		H						
133	1	03/08/2020	12	758	1	9.9	0	25.11
1 primi 1		H						
133	1	03/09/2020	13	759	1	9.6	0.03	25.64
1 primi 1		H						
133	1	03/10/2020	14	760	1	9.2	0.12	26.69
1 primi 1		H						
133	1	03/11/2020	15	761	1	9.2	0.12	25.76
1 primi 1		H						
133	1	03/12/2020	16	762	1	9.1	0	28.68
1 primi 1		H						
133	1	03/13/2020	17	763	1	9.3	0.12	27.88
1 primi 1		H						
133	1	03/14/2020	18	764	1	9.5	0.02	27.62
1 primi 1		H						
133	1	03/15/2020	19	765	1	9.2	0	28.77
1 primi 1		H						
133	1	03/16/2020	20	766	1	9.6	0.12	28.3
1 primi 1		H						
133	1	03/17/2020	21	767	1	9.5	0	30.34
1 primi 1		H						
133	1	03/18/2020	22	768	1	9.5	0	30.01

1 primi	2	H						
133	1	03/19/2020	23	769	1	9.2	0.13	27.36
1 primi	2	H						
133	1	03/20/2020	24	770	1	9.6	0.11	29.73
1 primi	2	H						
133	1	03/21/2020	25	771	1	9.3	0	30.39
1 primi	2	H						
133	1	03/22/2020	26	772	1	9.1	0.11	28.93
1 primi	2	H						
133	1	03/23/2020	27	773	1	9.3	0.11	31.71
1 primi	2	H						
133	1	03/24/2020	28	774	1	9.2	0	32.01
1 primi	2	H						
133	1	03/25/2020	29	775	1	9.2	0.02	31.77
1 primi	2	H						
133	1	03/26/2020	30	776	1	9.2	0	34.36
1 primi	2	H						
133	1	03/27/2020	31	777	1	9.5	0	33.51
1 primi	2	H						
133	1	03/28/2020	32	778	1	8.9	0.01	32.38
1 primi	2	H						
133	1	03/29/2020	33	779	1	8.8	0.08	31.94
1 primi	2	H						
133	1	03/30/2020	34	780	1	8.8	0	33.12
1 primi	2	H						
133	1	03/31/2020	35	781	1	8.8	0.07	34.11
1 primi	2	H						
133	1	04/01/2020	36	782	1	8.9	0.19	33.96
1 primi	2	H						
133	1	04/02/2020	37	783	1	8.6	0.09	28.57
1 primi	2	H						
133	1	04/03/2020	38	784	1	8.7	0	28.71
1 primi	2	H						
133	1	04/04/2020	39	785	1	8.9	0	31.65
1 primi	2	H						
133	1	04/05/2020	40	786	1	8.9	0	33.46
1 primi	2	H						
133	1	04/06/2020	41	787	1	9.2	0	33.29
1 primi	2	H						
133	1	04/07/2020	42	788	1	9.2	0.07	32.63
1 primi	2	H						
133	1	04/08/2020	43	789	1	9.3	0	32.86
1 primi	2	H						
133	1	04/09/2020	44	790	1	9.6	0.08	34.55
1 primi	2	H						
133	1	04/10/2020	45	791	1	9.5	0.08	36.66
1 primi	2	H						
133	1	04/11/2020	46	792	1	9.2	0	35.94
1 primi	2	H						
133	1	04/12/2020	47	793	1	9.1	0	36.34
1 primi	2	H						
133	1	04/13/2020	48	794	1	9.3	0.07	36.31
1 primi	2	H						
133	1	04/14/2020	49	795	1	8.8	0	33.98

1 primi 2		H						
133	1	04/15/2020	50	796	1	9.3	0	34.79
1 primi 2		H						
133	1	04/16/2020	51	797	1	9.3	0.09	31.64
1 primi 2		H						
133	1	04/17/2020	52	798	1	8.9	0	32.28
1 primi 2		H						
133	1	04/18/2020	53	799	1	9.5	0.2	31.18
1 primi 2		H						
133	1	04/19/2020	54	800	1	8.7	0	32.56
1 primi 2		H						
133	1	04/20/2020	55	801	1	8.9	0	33.96
1 primi 2		H						
133	1	04/21/2020	56	802	1	8.9	0.06	33.8
1 primi 2		H						
133	1	04/22/2020	57	803	1	9.1	0.06	34.64
1 primi 2		H						
133	1	04/23/2020	58	804	1	9.1	0.07	35.92
1 primi 2		H						
133	1	04/24/2020	59	805	1	9.1	0.07	34.79
1 primi 2		H						
133	1	04/25/2020	60	806	1	8.9	0.07	34.12
1 primi 2		H						
133	1	04/26/2020	61	807	1	8.9	0.07	35.92
1 primi 2		H						
133	1	04/27/2020	62	808	1	9.3	0	34.63
1 primi 2		H						
133	1	04/28/2020	63	809	1	9.2	0	34.18
1 primi 2		H						
133	1	06/30/2020	126	872	1	9.8	0	36.56
1 primi 2		H						
133	1	07/01/2020	127	873	1	9.3	0	34.37
1 primi 2		H						
133	1	07/02/2020	128	874	1	9.3	0	33.48
1 primi 2		H						
133	1	07/03/2020	129	875	1	8.9	0	29.26
1 primi 2		H						
133	1	07/04/2020	130	876	1	9.8	0.21	33.25
1 primi 2		H						
133	1	07/05/2020	131	877	1	9.9	0.19	34.86
1 primi 2		H						
133	1	07/06/2020	132	878	1	9.8	0.17	35.49
1 primi 2		H						
133	1	07/07/2020	133	879	1	9.6	0.23	32.92
1 primi 2		H						
133	1	07/08/2020	134	880	1	9.5	0.08	32.2
1 primi 2		H						
133	1	07/09/2020	135	881	1	9.2	0.12	27.59
1 primi 2		H						
133	1	07/10/2020	136	882	1	9.8	0	37.12
1 primi 2		H						
133	1	07/11/2020	137	883	1	9.6	0.1	34.98
1 primi 2		H						
133	1	07/12/2020	138	884	1	9.8	0	34.4

1 primi 2		H						
133	1	07/13/2020	139	885	1	9.5	0.12	33.9
1 primi 2		H						
133	1	07/14/2020	140	886	1	9.6	0.03	38.12
1 primi 2		H						
133	1	07/15/2020	141	887	1	9.8	0.1	37.42
1 primi 2		H						
133	1	07/16/2020	142	888	1	9.8	0.02	35.9
1 primi 2		H						
133	1	07/17/2020	143	889	1	9.6	0.11	37.69
1 primi 2		H						
133	1	07/18/2020	144	890	1	9.6	0.07	36.04
1 primi 2		H						
133	1	07/19/2020	145	891	1	10	0.15	39.48
1 primi 2		H						
133	1	07/20/2020	146	892	1	9.8	0.04	39.36
1 primi 2		H						
133	1	07/21/2020	147	893	1	9.6	0	36.98
1 primi 2		H						
133	1	07/22/2020	148	894	1	9.5	0.11	38.08
1 primi 2		H						
133	1	07/23/2020	149	895	1	9.3	0.16	33.91
1 primi 2		H						
133	1	07/24/2020	150	896	1	9.3	0.2	34.19
1 primi 2		H						
133	1	07/25/2020	151	897	1	10.2	0.03	38.81
1 primi 2		H						
133	1	07/26/2020	152	898	1	10.7	0	36.49
1 primi 2		H						
133	1	07/27/2020	153	899	1	10.2	0.05	37.04
1 primi 2		H						
133	1	07/28/2020	154	900	1	9.6	0	35.52
1 primi 2		H						
133	1	07/29/2020	155	901	1	9.8	0.1	37.38
1 primi 2		H						
133	1	07/30/2020	156	902	1	9.9	0.18	38.21
1 primi 2		H						
133	1	07/31/2020	157	903	1	9.6	0.11	36.6
1 primi 2		H						
133	1	08/01/2020	158	904	1	9.6	0.11	38.9
1 primi 2		H						
133	1	08/02/2020	159	905	1	9.2	0	37.79
1 primi 2		H						
133	1	08/03/2020	160	906	1	9.5	0	36.55
1 primi 2		H						
133	1	08/04/2020	161	907	1	9.6	0	36.92
1 primi 2		H						
133	1	08/05/2020	162	908	1	9.5	0.13	38.01
1 primi 2		H						
133	1	08/06/2020	163	909	1	9.6	0.04	38.91
1 primi 2		H						
133	1	08/07/2020	164	910	1	9.6	0.03	39.93
1 primi 2		H						
133	1	08/08/2020	165	911	1	9.9	0.1	40.8

1 primi 2		H						
133	1	08/09/2020	166	912	1	9.8	0.04	40.91
1 primi 2		H						
133	1	08/10/2020	167	913	1	9.8	0.11	40.59
1 primi 2		H						
133	1	08/11/2020	168	914	1	9.2	0.11	37.26
1 primi 2		H						
133	1	08/12/2020	169	915	1	9.8	0.01	37.27
1 primi 2		H						
133	1	08/13/2020	170	916	1	9.8	0.04	37.12
1 primi 2		H						
133	1	08/14/2020	171	917	1	9.9	0.1	37.6
1 primi 2		H						
133	1	08/15/2020	172	918	1	9.9	0.03	37.76
1 primi 2		H						
133	1	08/16/2020	173	919	1	9.8	0.37	36.19
1 primi 2		H						
133	1	08/17/2020	174	920	1	9.9	0	37.1
1 primi 2		H						
133	1	08/18/2020	175	921	1	9.9	0.12	39
1 primi 2		H						
133	1	08/19/2020	176	922	1	9.8	0.08	37.91
1 primi 2		H						
133	1	08/20/2020	177	923	1	9.6	0.1	35.84
1 primi 2		H						
133	1	08/21/2020	178	924	1	10	0.1	39.14
1 primi 2		H						
133	1	08/22/2020	179	925	1	9.6	0.07	35.8
1 primi 2		H						
133	1	08/23/2020	180	926	1	10	0	36.41
1 primi 2		H						
133	1	08/24/2020	181	927	1	9.6	0.03	33.2
1 primi 2		H						
133	1	08/25/2020	182	928	1	9.8	0	35.88
1 primi 2		H						
133	1	08/26/2020	183	929	1	9.6	0	34.51
1 primi 2		H						
133	1	08/27/2020	184	930	1	9.8	0.06	33.58
1 primi 2		H						
133	1	08/28/2020	185	931	1	9.8	0.13	35.45
1 primi 2		H						
133	1	08/29/2020	186	932	1	9.8	0	35.32
1 primi 2		H						
133	1	08/30/2020	187	933	1	9.6	0	34.84
1 primi 2		H						
133	1	08/31/2020	188	934	1	9.8	0.24	37.13
1 primi 2		H						
133	1	09/01/2020	189	935	1	9.8	0	36.5
1 primi 2		H						
133	1	09/02/2020	190	936	1	9.8	0.04	36.03
1 primi 2		H						
133	1	09/03/2020	191	937	1	9.8	0.18	36.85
1 primi 2		H						
133	1	09/04/2020	192	938	1	9.9	0	40.72

1 primi 2		H						
133	1	09/05/2020	193	939	1	9.8	0.17	38.48
1 primi 2		H						
133	1	09/06/2020	194	940	1	9.6	0.2	38.36
1 primi 2		H						
133	1	09/07/2020	195	941	1	9.5	0.04	37.32
1 primi 2		H						
133	1	09/08/2020	196	942	1	9.6	0.13	39.4
1 primi 2		H						
133	1	09/09/2020	197	943	1	9.3	0.23	43.71
1 primi 2		H						
133	1	09/10/2020	198	944	1	9.6	0.05	35.95
1 primi 2		H						
133	1	09/11/2020	199	945	1	9.8	0.12	37.47
1 primi 2		H						
133	1	09/12/2020	200	946	1	9.6	0	35.11
1 primi 2		H						
133	1	09/13/2020	201	947	1	9.6	0.13	37.3
1 primi 2		H						
133	1	09/14/2020	202	948	1	9.8	0.04	36.43
1 primi 2		H						
133	1	09/15/2020	203	949	1	9.2	0.05	37.68
1 primi 2		H						
133	1	09/16/2020	204	950	1	9.6	0	34.49
1 primi 2		H						
133	1	09/17/2020	205	951	1	9.6	0.07	26.49
1 primi 2		H						
133	1	09/18/2020	206	952	1	9.6	0	28.67
1 primi 2		H						
133	1	09/19/2020	207	953	1	9.6	0.12	33.61
1 primi 2		H						
133	1	09/20/2020	208	954	1	9.6	0.12	33.12
1 primi 2		H						
133	1	09/21/2020	209	955	1	9.3	0.12	35.56
1 primi 2		H						
133	1	09/22/2020	210	956	1	9.8	0.04	37.1
1 primi 2		H						
133	1	09/23/2020	211	957	1	9.8	0.05	34.47
1 primi 2		H						
133	1	09/24/2020	212	958	1	9.9	0	37.19
1 primi 2		H						
133	1	09/25/2020	213	959	1	9.6	0	37.44
1 primi 2		H						
133	1	09/26/2020	214	960	1	9.8	0.14	37.28
1 primi 2		H						
133	1	09/27/2020	215	961	1	9.3	0.08	38.91
1 primi 2		H						
133	1	09/28/2020	216	962	1	9.5	0.06	37.83
1 primi 2		H						
133	1	09/29/2020	217	963	1	9.6	0.13	37.45
1 primi 2		H						
133	1	09/30/2020	218	964	1	9.5	0.14	37.68
1 primi 2		H						
133	1	10/01/2020	219	965	1	9.6	0.01	38.32

1 primi 2		H						
133	1	10/02/2020	220	966	1	9.6	0.04	38.12
1 primi 2		H						
133	1	10/03/2020	221	967	1	9.9	0	38.92
1 primi 2		H						
133	1	10/04/2020	222	968	1	9.9	0	37.17
1 primi 2		H						
133	1	10/05/2020	223	969	1	9.6	0.05	37.42
1 primi 2		H						
133	1	10/06/2020	224	970	1	9.6	0.05	37.05
1 primi 2		H						
133	1	10/07/2020	225	971	1	9.9	0.05	36.69
1 primi 2		H						
133	1	10/08/2020	226	972	1	9.6	0.13	37.44
1 primi 2		H						
133	1	10/09/2020	227	973	1	9.5	0.01	32.18
1 primi 2		H						
133	1	10/10/2020	228	974	1	10	0.01	37.58
1 primi 2		H						
133	1	10/11/2020	229	975	1	9.6	0.02	33.3
1 primi 2		H						
133	1	10/12/2020	230	976	1	9.9	0.01	34.62
1 primi 2		H						
133	1	10/13/2020	231	977	1	10.6	0.14	36.13
1 primi 2		H						
133	1	10/14/2020	232	978	1	10	0.14	35.12
1 primi 2		H						
133	1	10/15/2020	233	979	1	10	0.07	38.25
1 primi 2		H						
133	1	10/16/2020	234	980	1	10	0.06	36.12
1 primi 2		H						
133	1	10/17/2020	235	981	1	9.6	0.07	38.41
1 primi 2		H						
133	1	10/18/2020	236	982	1	9.8	0.07	37.43
1 primi 2		H						
133	1	10/19/2020	237	983	1	9.8	0.14	37.65
1 primi 2		H						
133	1	10/20/2020	238	984	1	9.8	0.08	37.93
1 primi 2		H						
133	1	10/21/2020	239	985	1	9.9	0.02	39.65
1 primi 2		H						
133	1	10/22/2020	240	986	1	9.8	0.05	36.82
1 primi 2		H						
133	1	10/23/2020	241	987	1	9.8	0.07	35.27
1 primi 2		H						
133	1	10/24/2020	242	988	1	9.8	0.07	32.29
1 primi 2		H						
133	1	10/25/2020	243	989	1	9.8	0.15	33.15
1 primi 2		H						
133	1	10/26/2020	244	990	1	9.9	0.06	34.13
1 primi 2		H						
133	1	10/27/2020	245	991	1	9.8	0	30.2
1 primi 2		H						
133	1	10/28/2020	246	992	1	9.9	0.13	24.45

1 primi 2 H
133 1 10/29/2020 247 993 1 9.9 0.07 30.04
1 primi 2 H
133 1 10/30/2020 248 994 1 9.9 0.12 29.35
1 primi 2 H
133 1 10/31/2020 249 995 1 10 0.13 31.07
1 primi 2 H
133 1 11/01/2020 250 996 1 10 0.08 31.22
1 primi 2 H
133 1 11/02/2020 251 997 1 9.9 0.14 33.62
1 primi 2 H
133 1 11/03/2020 252 998 1 10.3 0.16 34.51
1 primi 2 H
133 1 11/04/2020 253 999 1 9.8 0.17 34.93
1 primi 2 H
134 1 02/26/2019 3 799 1 10 0.49 21.56
1 primi 1 H
134 1 02/27/2019 4 800 1 10.3 0.14 23.89
1 primi 1 H
134 1 02/28/2019 5 801 1 10 0.16 23.93
1 primi 1 H
134 1 03/01/2019 6 802 1 9.9 0 27.96
1 primi 1 H
134 1 03/02/2019 7 803 1 9.9 0 28.84
1 primi 1 H
134 1 03/03/2019 8 804 1 9.8 0 30.42
1 primi 1 H
134 1 03/04/2019 9 805 1 9.5 0 28.73
1 primi 1 H
134 1 03/05/2019 10 806 1 9.3 0 29.65
1 primi 1 H
134 1 03/06/2019 11 807 1 9.6 0 31.13
1 primi 1 H
134 1 03/07/2019 12 808 1 9.6 0 30.9
1 primi 1 H
134 1 03/08/2019 13 809 1 9.8 0 29.54
1 primi 1 H
134 1 03/09/2019 14 810 1 9.3 0.2 31.39
1 primi 1 H
134 1 03/10/2019 15 811 1 9.6 0 32.27
1 primi 1 H
134 1 03/11/2019 16 812 1 9.5 0 33.12
1 primi 1 H
134 1 03/12/2019 17 813 1 9.3 0.07 29.63
1 primi 1 H
134 1 03/13/2019 18 814 1 9.6 0 32.03
1 primi 1 H
134 1 03/14/2019 19 815 1 10 0 34.2
1 primi 1 H
134 1 03/15/2019 20 816 1 9.5 0.07 32.72
1 primi 1 H
134 1 03/16/2019 21 817 1 9.9 0 34.55
1 primi 1 H
134 1 03/17/2019 22 818 1 9.6 0 33.78

1 primi	2	H						
134	1	03/18/2019	23	819	1	9.6	0	35.06
1 primi	2	H						
134	1	03/19/2019	24	820	1	9.8	0.19	33.8
1 primi	2	H						
134	1	03/20/2019	25	821	1	9.6	0	36.24
1 primi	2	H						
134	1	03/21/2019	26	822	1	9.8	0	33.57
1 primi	2	H						
134	1	03/22/2019	27	823	1	9.2	0.14	36.32
1 primi	2	H						
134	1	03/23/2019	28	824	1	9.6	0	36.33
1 primi	2	H						
134	1	03/24/2019	29	825	1	9.6	0.08	35.29
1 primi	2	H						
134	1	03/25/2019	30	826	1	9.8	0	34.87
1 primi	2	H						
134	1	03/26/2019	31	827	1	9.6	0	36.66
1 primi	2	H						
134	1	03/27/2019	32	828	1	9.5	0	35.21
1 primi	2	H						
134	1	02/10/2020	4	1148	2	10.2	0.17	25.96
2 multi	1	H						
134	1	02/11/2020	5	1149	2	10.4	0	26.29
2 multi	1	H						
134	1	02/12/2020	6	1150	2	10.9	0.02	27.24
2 multi	1	H						
134	1	02/13/2020	7	1151	2	10	0.07	29.99
2 multi	1	H						
134	1	02/14/2020	8	1152	2	10.3	0.05	30.68
2 multi	1	H						
134	1	02/15/2020	9	1153	2	10	0.28	30.74
2 multi	1	H						
134	1	02/16/2020	10	1154	2	10.3	0.14	32.62
2 multi	1	H						
134	1	02/17/2020	11	1155	2	10.3	0.15	33.15
2 multi	1	H						
134	1	02/18/2020	12	1156	2	10	0.03	34.05
2 multi	1	H						
134	1	02/19/2020	13	1157	2	10.3	0.02	35.9
2 multi	1	H						
134	1	02/20/2020	14	1158	2	10.3	0.06	36.99
2 multi	1	H						
134	1	02/21/2020	15	1159	2	10.3	0.01	41.55
2 multi	1	H						
134	1	02/22/2020	16	1160	2	10.6	0.2	40.41
2 multi	1	H						
134	1	02/23/2020	17	1161	2	10.3	0	42.29
2 multi	1	H						
134	1	02/24/2020	18	1162	2	10	0	42.6
2 multi	1	H						
134	1	02/25/2020	19	1163	2	10.2	0	46.23
2 multi	1	H						
134	1	02/26/2020	20	1164	2	10.2	0	48.26

2 multi	1	H						
134	1	02/27/2020	21	1165	2	10.2	0	46.17
2 multi	1	H						
134	1	02/28/2020	22	1166	2	9.9	0.19	46.97
2 multi	2	H						
134	1	02/29/2020	23	1167	2	9.9	0.26	46.7
2 multi	2	H						
134	1	03/01/2020	24	1168	2	9.6	0.24	45.68
2 multi	2	H						
134	1	03/02/2020	25	1169	2	9.9	0.2	47.23
2 multi	2	H						
134	1	03/03/2020	26	1170	2	10.3	0	44.89
2 multi	2	H						
134	1	03/04/2020	27	1171	2	10.2	0.07	48.56
2 multi	2	H						
134	1	03/05/2020	28	1172	2	9.9	0.01	48.48
2 multi	2	H						
134	1	03/06/2020	29	1173	2	10	0	47.2
2 multi	2	H						
134	1	03/07/2020	30	1174	2	9.8	0	46.04
2 multi	2	H						
134	1	03/08/2020	31	1175	2	9.9	0	45.77
2 multi	2	H						
134	1	03/09/2020	32	1176	2	9.9	0	44.5
2 multi	2	H						
134	1	03/10/2020	33	1177	2	9.9	0	47.4
2 multi	2	H						
134	1	03/11/2020	34	1178	2	9.9	0	47.43
2 multi	2	H						
134	1	03/12/2020	35	1179	2	9.8	0.01	45.28
2 multi	2	H						
134	1	03/13/2020	36	1180	2	9.9	0	46.67
2 multi	2	H						
134	1	03/14/2020	37	1181	2	9.9	0	46.67
2 multi	2	H						
134	1	03/15/2020	38	1182	2	9.9	0	46.71
2 multi	2	H						
134	1	03/16/2020	39	1183	2	9.9	0.35	47.09
2 multi	2	H						
134	1	03/17/2020	40	1184	2	10	0	46.75
2 multi	2	H						
134	1	03/18/2020	41	1185	2	10	0	47.51
2 multi	2	H						
134	1	03/19/2020	42	1186	2	10.2	0.32	46.64
2 multi	2	H						
134	1	03/20/2020	43	1187	2	10	0	45.93
2 multi	2	H						
134	1	03/21/2020	44	1188	2	10.2	0.01	44.78
2 multi	2	H						
134	1	03/22/2020	45	1189	2	9.6	0	43.39
2 multi	2	H						
134	1	03/23/2020	46	1190	2	9.8	0	41.75
2 multi	2	H						
134	1	03/24/2020	47	1191	2	9.6	0	46.87

2 multi 2		H							
134	1	03/25/2020	48	1192	2	9.6	0.04	43.26	
2 multi 2		H							
134	1	03/26/2020	49	1193	2	9.9	0	45.61	
2 multi 2		H							
134	1	03/27/2020	50	1194	2	9.9	0	42.86	
2 multi 2		H							
134	1	03/28/2020	51	1195	2	10	0	44.45	
2 multi 2		H							
134	1	03/29/2020	52	1196	2	10.2	0.06	43.32	
2 multi 2		H							
134	1	03/30/2020	53	1197	2	9.8	0	45.88	
2 multi 2		H							
134	1	03/31/2020	54	1198	2	10	0.01	45.26	
2 multi 2		H							
134	1	04/01/2020	55	1199	2	10	0.14	44.25	
2 multi 2		H							
134	1	04/02/2020	56	1200	2	10	0	44	
2 multi 2		H							
134	1	04/03/2020	57	1201	2	9.5	0	45.45	
2 multi 2		H							
134	1	04/04/2020	58	1202	2	10.3	0	42.82	
2 multi 2		H							
134	1	04/05/2020	59	1203	2	10.3	0	44.92	
2 multi 2		H							
134	1	04/06/2020	60	1204	2	10.2	0	43.53	
2 multi 2		H							
134	1	04/07/2020	61	1205	2	10.2	0.3	42.8	
2 multi 2		H							
134	1	04/08/2020	62	1206	2	10.7	0	44.17	
2 multi 2		H							
134	1	04/09/2020	63	1207	2	10.4	0	42.31	
2 multi 2		H							
134	1	04/10/2020	64	1208	2	11.1	0	43.68	
2 multi 2		H							
134	1	04/11/2020	65	1209	2	10.9	0.01	43.53	
2 multi 2		H							
134	1	04/12/2020	66	1210	2	10.9	0	40.83	
2 multi 2		H							
134	1	04/13/2020	67	1211	2	11.1	0	43.65	
2 multi 2		H							
134	1	04/14/2020	68	1212	2	10.4	0.12	42.03	
2 multi 2		H							
134	1	04/15/2020	69	1213	2	11.3	0	41.63	
2 multi 2		H							
134	1	04/16/2020	70	1214	2	10.6	0	43.48	
2 multi 2		H							
134	1	04/17/2020	71	1215	2	11.1	0.01	41.67	
2 multi 2		H							
134	1	04/18/2020	72	1216	2	11.4	0	41.34	
2 multi 2		H							
134	1	04/19/2020	73	1217	2	11.1	0	41.93	
2 multi 2		H							
134	1	04/20/2020	74	1218	2	11.1	0	41.3	

2 multi 2		H						
134	1	04/21/2020	75	1219	2	10.7	0	37.43
2 multi 2		H						
134	1	04/22/2020	76	1220	2	10.3	0.2	36.25
2 multi 2		H						
134	1	04/23/2020	77	1221	2	10.6	0.03	40.7
2 multi 2		H						
134	1	04/24/2020	78	1222	2	10.7	0.05	41.85
2 multi 2		H						
134	1	04/25/2020	79	1223	2	10.6	0	40.65
2 multi 2		H						
134	1	04/26/2020	80	1224	2	10.4	0	41.51
2 multi 2		H						
134	1	04/27/2020	81	1225	2	10	0.3	41.07
2 multi 2		H						
134	1	04/28/2020	82	1226	2	10.6	0	42.27
2 multi 2		H						
134	1	04/29/2020	83	1227	2	10.4	0	39.78
2 multi 2		H						
134	1	04/30/2020	84	1228	2	10.9	0	41.05
2 multi 2		H						
134	1	05/01/2020	85	1229	2	10.4	0	40.75
2 multi 2		H						
134	1	05/02/2020	86	1230	2	10.7	0	39.73
2 multi 2		H						
134	1	05/03/2020	87	1231	2	10.3	0	40.52
2 multi 2		H						
134	1	05/04/2020	88	1232	2	10.4	0	39.96
2 multi 2		H						
134	1	05/05/2020	89	1233	2	10.3	0.15	39.44
2 multi 2		H						
134	1	05/06/2020	90	1234	2	10.2	0.3	37.45
2 multi 2		H						
134	1	05/07/2020	91	1235	2	10.4	0	39.07
2 multi 2		H						
134	1	05/08/2020	92	1236	2	10.6	0	37.6
2 multi 2		H						
134	1	05/09/2020	93	1237	2	10.4	0	40.75
2 multi 2		H						
134	1	05/10/2020	94	1238	2	10.6	0	38.34
2 multi 2		H						
134	1	05/11/2020	95	1239	2	10.9	0	38.66
2 multi 2		H						
134	1	05/12/2020	96	1240	2	10.7	0.01	36.06
2 multi 2		H						
134	1	05/13/2020	97	1241	2	10	0.03	38.46
2 multi 2		H						
134	1	05/14/2020	98	1242	2	10.6	0.27	36.25
2 multi 2		H						
134	1	05/15/2020	99	1243	2	10.3	0	32.77
2 multi 2		H						
134	1	05/16/2020	100	1244	2	10.3	0	36.82
2 multi 2		H						
134	1	05/17/2020	101	1245	2	10.4	0	37.41

2 multi 2 H
134 1 05/18/2020 102 1246 2 10.4 0 36.44
2 multi 2 H
134 1 05/19/2020 103 1247 2 10.3 0 37.36
2 multi 2 H
134 1 05/20/2020 104 1248 2 10 0.03 35.96
2 multi 2 H
134 1 05/21/2020 105 1249 2 10.3 0.01 37.44
2 multi 2 H
134 1 06/30/2020 145 1289 2 10.6 0 29.06
2 multi 2 H
134 1 07/01/2020 146 1290 2 10.6 0 31.44
2 multi 2 H
134 1 07/02/2020 147 1291 2 10.4 0 31.78
2 multi 2 H
134 1 07/03/2020 148 1292 2 10.7 0.04 31.39
2 multi 2 H
134 1 07/04/2020 149 1293 2 10.9 0 29.61
2 multi 2 H
134 1 07/05/2020 150 1294 2 10.6 0.03 32.72
2 multi 2 H
134 1 07/06/2020 151 1295 2 10.4 0.02 32.95
2 multi 2 H
134 1 07/07/2020 152 1296 2 10.2 0 30.65
2 multi 2 H
134 1 07/08/2020 153 1297 2 10.6 0 33.3
2 multi 2 H
134 1 07/09/2020 154 1298 2 10.6 0 33.11
2 multi 2 H
134 1 07/10/2020 155 1299 2 10.4 0.01 34.77
2 multi 2 H
134 1 07/11/2020 156 1300 2 10.4 0.3 32.89
2 multi 2 H
134 1 07/12/2020 157 1301 2 10.4 0.12 32.23
2 multi 2 H
134 1 07/13/2020 158 1302 2 10.6 0.01 32.55
2 multi 2 H
134 1 07/14/2020 159 1303 2 10.3 0.01 32.32
2 multi 2 H
134 1 07/15/2020 160 1304 2 10.7 0 32.79
2 multi 2 H
134 1 07/16/2020 161 1305 2 11.3 0.3 31.5
2 multi 2 H
134 1 07/17/2020 162 1306 2 0 0.19 46.59
2 multi 2 H
134 1 07/18/2020 163 1307 2 11.9 0.16 28.24
2 multi 2 H
134 1 07/19/2020 164 1308 2 11.7 0 29.26
2 multi 2 H
134 1 07/20/2020 165 1309 2 10.9 0 31.09
2 multi 2 H
134 1 07/21/2020 166 1310 2 11.7 0.01 27.64
2 multi 2 H
134 1 07/22/2020 167 1311 2 11.7 0 28.06

2 multi	2	H						
134	1	07/23/2020	168	1312	2	11.3	0	30.37
2 multi	2	H						
134	1	07/24/2020	169	1313	2	11.1	0	29.09
2 multi	2	H						
134	1	07/25/2020	170	1314	2	11.4	0	32.63
2 multi	2	H						
134	1	07/26/2020	171	1315	2	11.3	0	31.49
2 multi	2	H						
134	1	07/27/2020	172	1316	2	11.1	0.17	33.52
2 multi	2	H						
134	1	07/28/2020	173	1317	2	10.9	0.01	30.77
2 multi	2	H						
134	1	07/29/2020	174	1318	2	10.7	0	33.04
2 multi	2	H						
134	1	07/30/2020	175	1319	2	10.9	0.04	30.38
2 multi	2	H						
134	1	07/31/2020	176	1320	2	11.1	0	31.3
2 multi	2	H						
134	1	08/01/2020	177	1321	2	11.3	0	29.55
2 multi	2	H						
134	1	08/02/2020	178	1322	2	11.1	0.02	31.11
2 multi	2	H						
134	1	08/03/2020	179	1323	2	10.7	0.01	29.71
2 multi	2	H						
134	1	08/04/2020	180	1324	2	10.7	0	31.23
2 multi	2	H						
134	1	08/05/2020	181	1325	2	10.7	0	33.29
2 multi	2	H						
134	1	08/06/2020	182	1326	2	10.7	0.02	32.4
2 multi	2	H						
134	1	08/07/2020	183	1327	2	10.6	0	28.33
2 multi	2	H						
134	1	08/08/2020	184	1328	2	10.9	0	33.77
2 multi	2	H						
134	1	08/09/2020	185	1329	2	11.1	0.05	32.78
2 multi	2	H						
134	1	08/10/2020	186	1330	2	11.1	0.11	32.08
2 multi	2	H						
134	1	08/11/2020	187	1331	2	10.7	0.01	28.84
2 multi	2	H						
134	1	08/12/2020	188	1332	2	10.9	0.03	31.99
2 multi	2	H						
134	1	08/13/2020	189	1333	2	10.9	0.04	30.03
2 multi	2	H						
134	1	08/14/2020	190	1334	2	10.9	0.02	31.27
2 multi	2	H						
134	1	08/15/2020	191	1335	2	10.9	0.14	30
2 multi	2	H						
134	1	08/16/2020	192	1336	2	10.3	0	29.09
2 multi	2	H						
136	1	12/09/2017	7	821	1	9.9	0	19.51
1 primi	1	H						
136	1	12/10/2017	8	822	1	8.9	0	18.24

1 primi	1	H						
136	1	12/11/2017	9	823	1	10.2	0.05	18.79
1 primi	1	H						
136	1	12/12/2017	10	824	1	9.2	0.07	18.31
1 primi	1	H						
136	1	12/13/2017	11	825	1	9.1	0.01	17
1 primi	1	H						
136	1	12/14/2017	12	826	1	8.8	0	17.59
1 primi	1	H						
136	1	12/15/2017	13	827	1	10.2	0.13	18.81
1 primi	1	H						
136	1	12/16/2017	14	828	1	10.3	0.13	20.54
1 primi	1	H						
136	1	12/17/2017	15	829	1	10.2	0.13	19.6
1 primi	1	H						
136	1	12/18/2017	16	830	1	8.9	0	17.84
1 primi	1	H						
136	1	12/19/2017	17	831	1	9.9	0.05	19.29
1 primi	1	H						
136	1	12/20/2017	18	832	1	10	0.01	21.98
1 primi	1	H						
136	1	12/21/2017	19	833	1	9.9	0.14	21.01
1 primi	1	H						
136	1	12/22/2017	20	834	1	9.8	0.04	20.98
1 primi	1	H						
136	1	12/23/2017	21	835	1	9.6	0.13	21.51
1 primi	1	H						
136	1	12/24/2017	22	836	1	9.2	0.02	23.2
1 primi	2	H						
136	1	12/25/2017	23	837	1	9.6	0.02	22.63
1 primi	2	H						
136	1	12/26/2017	24	838	1	9.6	0.02	21.71
1 primi	2	H						
136	1	12/27/2017	25	839	1	9.1	0	24.61
1 primi	2	H						
136	1	12/28/2017	26	840	1	10.4	0.01	22.61
1 primi	2	H						
136	1	12/29/2017	27	841	1	10.4	0	18.93
1 primi	2	H						
136	1	12/30/2017	28	842	1	10.2	0.01	20.95
1 primi	2	H						
136	1	12/31/2017	29	843	1	10.2	0	22.44
1 primi	2	H						
136	1	01/01/2018	30	844	1	9.8	0	23.82
1 primi	2	H						
136	1	01/02/2018	31	845	1	9.6	0.07	24.58
1 primi	2	H						
136	1	01/03/2018	32	846	1	9.2	0.21	25.45
1 primi	2	H						
136	1	01/04/2018	33	847	1	9.6	0.28	24.74
1 primi	2	H						
136	1	01/05/2018	34	848	1	10.3	0.02	25.14
1 primi	2	H						
136	1	01/06/2018	35	849	1	9.8	0	23.07

1 primi 2 H
136 1 01/07/2018 36 850 1 9.3 0 27.17
1 primi 2 H
136 1 01/08/2018 37 851 1 9.9 0 27.01
1 primi 2 H
136 1 01/09/2018 38 852 1 9.3 0 25.87
1 primi 2 H
136 1 01/10/2018 39 853 1 9.6 0 25.96
1 primi 2 H
136 1 01/11/2018 40 854 1 9.9 0.71 27.72
1 primi 2 H
136 1 01/12/2018 41 855 1 9.5 0 27.4
1 primi 2 H
136 1 01/13/2018 42 856 1 9.6 0.08 27.39
1 primi 2 H
136 1 01/14/2018 43 857 1 9.8 0 27.89
1 primi 2 H
136 1 01/15/2018 44 858 1 9.2 0 26.86
1 primi 2 H
136 1 01/16/2018 45 859 1 9.5 0.01 28.38
1 primi 2 H
136 1 01/17/2018 46 860 1 9.8 0.12 27.28
1 primi 2 H
136 1 01/18/2018 47 861 1 9.3 0 28.45
1 primi 2 H
136 1 01/19/2018 48 862 1 9.2 0 29.2
1 primi 2 H
136 1 01/20/2018 49 863 1 8.8 0.07 29.18
1 primi 2 H
136 1 01/21/2018 50 864 1 8.9 0 29.2
1 primi 2 H
136 1 01/22/2018 51 865 1 9.2 0 30.03
1 primi 2 H
136 1 01/23/2018 52 866 1 8.8 0 28.14
1 primi 2 H
136 1 01/24/2018 53 867 1 9.2 0 31.29
1 primi 2 H
136 1 01/25/2018 54 868 1 9.1 0.08 29.89
1 primi 2 H
136 1 01/26/2018 55 869 1 9.6 0 31.97
1 primi 2 H
136 1 01/27/2018 56 870 1 9.5 0 30.72
1 primi 2 H
136 1 01/28/2018 57 871 1 9.6 0 32.75
1 primi 2 H
136 1 01/29/2018 58 872 1 9.1 0 32.42
1 primi 2 H
136 1 01/30/2018 59 873 1 9.8 0.21 30.39
1 primi 2 H
136 1 01/31/2018 60 874 1 9.6 0.06 32.67
1 primi 2 H
136 1 02/01/2018 61 875 1 9.5 0.06 32.22
1 primi 2 H
136 1 02/02/2018 62 876 1 9.3 0.06 29.56

1 primi 2		H						
136	1	02/03/2018	63	877	1	9.5	0	32.22
1 primi 2		H						
136	1	02/04/2018	64	878	1	9.5	0	31.27
1 primi 2		H						
136	1	02/05/2018	65	879	1	9.6	0	31.83
1 primi 2		H						
136	1	02/06/2018	66	880	1	9.3	0	31.13
1 primi 2		H						
136	1	02/07/2018	67	881	1	9.3	0	32.44
1 primi 2		H						
136	1	10/25/2018	4	1141	2	9.2	0.26	22.28
2 multi 1		H						
136	1	10/26/2018	5	1142	2	9.5	0.2	22.93
2 multi 1		H						
136	1	10/27/2018	6	1143	2	9.3	0.07	24.47
2 multi 1		H						
136	1	10/28/2018	7	1144	2	9.6	0.04	30.7
2 multi 1		H						
136	1	10/29/2018	8	1145	2	9.8	0	28.9
2 multi 1		H						
136	1	10/30/2018	9	1146	2	9.5	0.02	27.56
2 multi 1		H						
136	1	10/31/2018	10	1147	2	9.6	0.02	29.31
2 multi 1		H						
136	1	11/01/2018	11	1148	2	9.9	0.04	32.77
2 multi 1		H						
136	1	11/02/2018	12	1149	2	9.6	0	33.42
2 multi 1		H						
136	1	11/03/2018	13	1150	2	9.8	0.19	33.7
2 multi 1		H						
136	1	11/04/2018	14	1151	2	9.6	0	31.99
2 multi 1		H						
136	1	11/05/2018	15	1152	2	9.8	0	32.8
2 multi 1		H						
136	1	11/06/2018	16	1153	2	9.6	0	36.37
2 multi 1		H						
136	1	11/07/2018	17	1154	2	9.6	0.09	35.76
2 multi 1		H						
136	1	11/08/2018	18	1155	2	9.9	0.09	37.09
2 multi 1		H						
136	1	11/09/2018	19	1156	2	9.9	0.2	35.37
2 multi 1		H						
136	1	11/10/2018	20	1157	2	9.9	0.04	38.88
2 multi 1		H						
136	1	11/11/2018	21	1158	2	9.9	0.03	41.12
2 multi 1		H						
136	1	11/12/2018	22	1159	2	10	0.08	40.98
2 multi 2		H						
136	1	11/13/2018	23	1160	2	9.8	0.07	39.95
2 multi 2		H						
136	1	11/14/2018	24	1161	2	9.8	0	42.87
2 multi 2		H						
136	1	11/15/2018	25	1162	2	9.9	0	40.34

2 multi 2		H						
136	1	11/16/2018	26	1163	2	9.9	0.1	39.37
2 multi 2		H						
136	1	11/17/2018	27	1164	2	9.6	0.07	40.13
2 multi 2		H						
136	1	11/18/2018	28	1165	2	9.6	0.07	42.36
2 multi 2		H						
136	1	11/19/2018	29	1166	2	9.9	0	43.17
2 multi 2		H						
136	1	11/20/2018	30	1167	2	9.8	0.07	39.55
2 multi 2		H						
136	1	11/21/2018	31	1168	2	9.6	0.02	40.49
2 multi 2		H						
136	1	11/22/2018	32	1169	2	9.6	0	39.07
2 multi 2		H						
136	1	11/23/2018	33	1170	2	9.6	0	42.39
2 multi 2		H						
136	1	11/24/2018	34	1171	2	9.9	0.11	45.67
2 multi 2		H						
136	1	11/25/2018	35	1172	2	9.8	0	46.38
2 multi 2		H						
136	1	11/26/2018	36	1173	2	9.9	0.07	40.9
2 multi 2		H						
136	1	11/27/2018	37	1174	2	9.8	0	40.86
2 multi 2		H						
136	1	11/28/2018	38	1175	2	9.8	0	41.96
2 multi 2		H						
136	1	11/29/2018	39	1176	2	9.9	0	43.43
2 multi 2		H						
136	1	11/30/2018	40	1177	2	9.6	0	39.81
2 multi 2		H						
136	1	12/01/2018	41	1178	2	9.8	0	39.32
2 multi 2		H						
136	1	12/02/2018	42	1179	2	9.8	0	39.32
2 multi 2		H						
136	1	12/03/2018	43	1180	2	9.8	0.07	43.89
2 multi 2		H						
136	1	12/04/2018	44	1181	2	10	0.07	39.89
2 multi 2		H						
136	1	12/05/2018	45	1182	2	9.3	0.07	38.08
2 multi 2		H						
136	1	12/06/2018	46	1183	2	9.5	0	40.83
2 multi 2		H						
136	1	12/07/2018	47	1184	2	9.6	0.06	43.88
2 multi 2		H						
136	1	12/08/2018	48	1185	2	9.2	0	39.43
2 multi 2		H						
136	1	12/09/2018	49	1186	2	9.6	0.07	47.1
2 multi 2		H						
136	1	12/10/2018	50	1187	2	9.5	0.05	41.96
2 multi 2		H						
136	1	12/11/2018	51	1188	2	9.5	0.05	43.96
2 multi 2		H						
136	1	12/12/2018	52	1189	2	9.5	0.07	42.74

2 multi 2		H						
136	1	12/13/2018	53	1190	2	9.6	0.25	42.57
2 multi 2		H						
136	1	12/14/2018	54	1191	2	9.1	0	39.6
2 multi 2		H						
136	1	12/15/2018	55	1192	2	9.6	0	44.86
2 multi 2		H						
136	1	12/16/2018	56	1193	2	9.5	0	42.95
2 multi 2		H						
136	1	12/17/2018	57	1194	2	9.6	0	37.67
2 multi 2		H						
136	1	12/18/2018	58	1195	2	9.5	0.04	40.94
2 multi 2		H						
136	1	12/19/2018	59	1196	2	9.9	0	43.66
2 multi 2		H						
136	1	12/20/2018	60	1197	2	9.5	0	41.88
2 multi 2		H						
136	1	12/21/2018	61	1198	2	9.8	0	41.49
2 multi 2		H						
136	1	12/22/2018	62	1199	2	9.3	0.06	36.22
2 multi 2		H						
136	1	12/23/2018	63	1200	2	9.8	0	43.78
2 multi 2		H						
136	1	12/24/2018	64	1201	2	9.5	0	40.85
2 multi 2		H						
136	1	12/25/2018	65	1202	2	9.8	0.05	46.49
2 multi 2		H						
136	1	12/26/2018	66	1203	2	9.8	0.05	43.41
2 multi 2		H						
136	1	12/27/2018	67	1204	2	9.5	0	39.43
2 multi 2		H						
136	1	12/28/2018	68	1205	2	9.6	0	37.14
2 multi 2		H						
136	1	12/29/2018	69	1206	2	9.8	0.05	42.59
2 multi 2		H						
136	1	12/30/2018	70	1207	2	9.8	0	42.17
2 multi 2		H						
136	1	12/31/2018	71	1208	2	9.6	0	42.3
2 multi 2		H						
136	1	01/01/2019	72	1209	2	9.3	0	36.64
2 multi 2		H						
136	1	01/02/2019	73	1210	2	9.5	0	39.16
2 multi 2		H						
136	1	01/03/2019	74	1211	2	9.5	0	36.94
2 multi 2		H						
136	1	01/04/2019	75	1212	2	9.3	0	35.8
2 multi 2		H						
136	1	01/05/2019	76	1213	2	9.6	0.3	39.59
2 multi 2		H						
136	1	01/06/2019	77	1214	2	9.5	0	38.22
2 multi 2		H						
136	1	01/07/2019	78	1215	2	9.5	0	37.03
2 multi 2		H						
136	1	01/08/2019	79	1216	2	9.3	0.06	39.26

2 multi	2	H						
136	1	01/09/2019	80	1217	2	9.6	0	40.1
2 multi	2	H						
136	1	01/10/2019	81	1218	2	9.6	0	39.2
2 multi	2	H						
136	1	01/11/2019	82	1219	2	9.8	0	38.37
2 multi	2	H						
136	1	01/12/2019	83	1220	2	9.8	0	37.96
2 multi	2	H						
136	1	01/13/2019	84	1221	2	9.6	0	39.65
2 multi	2	H						
136	1	01/14/2019	85	1222	2	9.6	0.12	35.48
2 multi	2	H						
136	1	01/15/2019	86	1223	2	9.9	0.22	41.69
2 multi	2	H						
136	1	01/16/2019	87	1224	2	10	0.07	42.64
2 multi	2	H						
136	1	01/17/2019	88	1225	2	9.6	0	36.99
2 multi	2	H						
136	1	01/18/2019	89	1226	2	9.6	0.1	36.75
2 multi	2	H						
136	1	01/19/2019	90	1227	2	9.9	0	38.07
2 multi	2	H						
136	1	01/20/2019	91	1228	2	9.6	0	41.21
2 multi	2	H						
136	1	01/21/2019	92	1229	2	9.9	0.06	43.73
2 multi	2	H						
136	1	01/22/2019	93	1230	2	9.8	0.06	40.57
2 multi	2	H						
136	1	01/23/2019	94	1231	2	9.8	0.19	43.03
2 multi	2	H						
136	1	01/24/2019	95	1232	2	9.8	0.18	42.22
2 multi	2	H						
136	1	01/25/2019	96	1233	2	9.9	0	40.24
2 multi	2	H						
136	1	01/26/2019	97	1234	2	9.8	0.13	42.08
2 multi	2	H						
136	1	01/27/2019	98	1235	2	10	0.06	43.51
2 multi	2	H						
136	1	01/28/2019	99	1236	2	9.9	0.06	43.17
2 multi	2	H						
136	1	01/29/2019	100	1237	2	9.9	0.07	39.92
2 multi	2	H						
136	1	01/30/2019	101	1238	2	9.9	0.06	41.32
2 multi	2	H						
136	1	01/31/2019	102	1239	2	9.8	0.12	42.62
2 multi	2	H						
136	1	02/01/2019	103	1240	2	9.6	0	38.05
2 multi	2	H						
136	1	02/02/2019	104	1241	2	9.9	0.05	40.48
2 multi	2	H						
136	1	02/03/2019	105	1242	2	9.8	0	38.01
2 multi	2	H						
136	1	02/04/2019	106	1243	2	9.6	0	42.64

2 multi	2	H						
136	1	02/05/2019	107	1244	2	9.5	0	35.86
2 multi	2	H						
136	1	02/06/2019	108	1245	2	9.6	0.07	38.76
2 multi	2	H						
136	1	02/07/2019	109	1246	2	10	0	41.38
2 multi	2	H						
136	1	02/08/2019	110	1247	2	9.9	0.09	39.09
2 multi	2	H						
136	1	02/09/2019	111	1248	2	9.9	0	41.58
2 multi	2	H						
136	1	02/10/2019	112	1249	2	9.9	0	41.82
2 multi	2	H						
136	1	02/11/2019	113	1250	2	9.8	0.08	40.43
2 multi	2	H						
136	1	02/12/2019	114	1251	2	9.8	0.08	42.25
2 multi	2	H						
136	1	02/13/2019	115	1252	2	9.8	0	39.5
2 multi	2	H						
136	1	02/14/2019	116	1253	2	10	0.07	44.48
2 multi	2	H						
136	1	02/15/2019	117	1254	2	9.8	0	38.83
2 multi	2	H						
136	1	02/16/2019	118	1255	2	9.9	0	42.95
2 multi	2	H						
136	1	02/17/2019	119	1256	2	9.9	0.06	40.29
2 multi	2	H						
136	1	02/18/2019	120	1257	2	9.6	0	36.76
2 multi	2	H						
136	1	02/19/2019	121	1258	2	9.9	0.07	42.31
2 multi	2	H						
136	1	02/20/2019	122	1259	2	9.9	0.07	38.53
2 multi	2	H						
136	1	02/21/2019	123	1260	2	9.9	0.12	39.87
2 multi	2	H						
136	1	02/22/2019	124	1261	2	9.6	0.1	34.57
2 multi	2	H						
136	1	02/23/2019	125	1262	2	9.8	0.07	38.25
2 multi	2	H						
136	1	02/24/2019	126	1263	2	10	0	38.95
2 multi	2	H						
136	1	02/25/2019	127	1264	2	9.9	0	37.83
2 multi	2	H						
136	1	02/26/2019	128	1265	2	9.8	0.03	39.07
2 multi	2	H						
136	1	02/27/2019	129	1266	2	10.2	0	41.73
2 multi	2	H						
136	1	02/28/2019	130	1267	2	9.9	0.17	42.34
2 multi	2	H						
136	1	03/01/2019	131	1268	2	9.9	0.01	39.5
2 multi	2	H						
136	1	03/02/2019	132	1269	2	9.9	0	42.57
2 multi	2	H						
136	1	03/03/2019	133	1270	2	9.9	0	41.23

2 multi	2	H						
136	1	03/04/2019	134	1271	2	9.8	0.22	42.04
2 multi	2	H						
136	1	03/05/2019	135	1272	2	10	0	39.35
2 multi	2	H						
136	1	03/06/2019	136	1273	2	9.9	0	38.18
2 multi	2	H						
136	1	03/07/2019	137	1274	2	9.9	0.05	38.26
2 multi	2	H						
136	1	03/28/2019	158	1295	2	10.3	0	34.68
2 multi	2	H						
136	1	03/29/2019	159	1296	2	10.2	0.09	38.66
2 multi	2	H						
136	1	03/30/2019	160	1297	2	9.9	0	37.22
2 multi	2	H						
136	1	03/31/2019	161	1298	2	10	0.4	41.54
2 multi	2	H						
136	1	04/01/2019	162	1299	2	9.9	0	38.21
2 multi	2	H						
136	1	04/02/2019	163	1300	2	9.5	0.11	35.07
2 multi	2	H						
136	1	04/03/2019	164	1301	2	9.9	0	37.95
2 multi	2	H						
136	1	04/04/2019	165	1302	2	10.2	0.06	39.56
2 multi	2	H						
136	1	04/05/2019	166	1303	2	10	0	33.37
2 multi	2	H						
136	1	04/06/2019	167	1304	2	10	0.09	38.21
2 multi	2	H						
136	1	04/07/2019	168	1305	2	10	0	34.22
2 multi	2	H						
136	1	04/08/2019	169	1306	2	9.8	0	33.93
2 multi	2	H						
136	1	04/09/2019	170	1307	2	10.2	0.08	38.3
2 multi	2	H						
136	1	04/10/2019	171	1308	2	10	0.06	37.35
2 multi	2	H						
136	1	04/11/2019	172	1309	2	10	0.06	37.74
2 multi	2	H						
136	1	04/12/2019	173	1310	2	10	0	34.17
2 multi	2	H						
136	1	04/13/2019	174	1311	2	10	0.07	41.05
2 multi	2	H						
136	1	04/14/2019	175	1312	2	9.9	0	40.5
2 multi	2	H						
136	1	04/15/2019	176	1313	2	9.8	0.06	40.44
2 multi	2	H						
136	1	04/16/2019	177	1314	2	9.9	0	36.99
2 multi	2	H						
136	1	04/17/2019	178	1315	2	9.9	0	38.15
2 multi	2	H						
136	1	04/18/2019	179	1316	2	10	0	39.61
2 multi	2	H						
136	1	04/19/2019	180	1317	2	10	0.06	41.5

2 multi	2	H						
136	1	04/20/2019	181	1318	2	9.9	0	39.69
2 multi	2	H						
136	1	04/21/2019	182	1319	2	9.9	0	40.4
2 multi	2	H						
136	1	04/22/2019	183	1320	2	9.9	0	41.8
2 multi	2	H						
136	1	04/23/2019	184	1321	2	9.8	0	40.75
2 multi	2	H						
136	1	04/24/2019	185	1322	2	9.9	0.08	38.32
2 multi	2	H						
136	1	04/25/2019	186	1323	2	9.9	0	39.73
2 multi	2	H						
136	1	04/26/2019	187	1324	2	10.2	0.06	41.77
2 multi	2	H						
136	1	04/27/2019	188	1325	2	9.9	0.05	42.72
2 multi	2	H						
136	1	04/28/2019	189	1326	2	10	0	39.62
2 multi	2	H						
136	1	04/29/2019	190	1327	2	9.9	0	37.47
2 multi	2	H						
136	1	04/30/2019	191	1328	2	10	0.06	40.18
2 multi	2	H						
136	1	05/01/2019	192	1329	2	10	0	40.67
2 multi	2	H						
136	1	05/02/2019	193	1330	2	10	0	39.63
2 multi	2	H						
136	1	05/03/2019	194	1331	2	9.9	0	40.02
2 multi	2	H						
136	1	05/04/2019	195	1332	2	10	0	41.14
2 multi	2	H						
136	1	05/05/2019	196	1333	2	9.9	0	36.54
2 multi	2	H						
136	1	05/06/2019	197	1334	2	9.9	0.36	36.99
2 multi	2	H						
136	1	05/07/2019	198	1335	2	10	0	41.49
2 multi	2	H						
136	1	05/08/2019	199	1336	2	9.9	0.06	39.34
2 multi	2	H						
136	1	05/09/2019	200	1337	2	9.8	0	38.39
2 multi	2	H						
136	1	05/10/2019	201	1338	2	9.9	0	38.39
2 multi	2	H						
136	1	05/11/2019	202	1339	2	9.9	0	38.71
2 multi	2	H						
136	1	05/12/2019	203	1340	2	9.9	0	38.32
2 multi	2	H						
136	1	05/13/2019	204	1341	2	9.8	0	35.59
2 multi	2	H						
136	1	05/14/2019	205	1342	2	9.8	0.13	38.7
2 multi	2	H						
136	1	05/15/2019	206	1343	2	9.8	0	38.69
2 multi	2	H						
136	1	05/16/2019	207	1344	2	9.8	0	36.82

2 multi 2		H						
136	1	05/17/2019	208	1345	2	9.6	0	37.48
2 multi 2		H						
136	1	05/18/2019	209	1346	2	9.8	0	37.74
2 multi 2		H						
136	1	05/19/2019	210	1347	2	10.2	0	58.45
2 multi 2		H						
136	1	05/20/2019	211	1348	2	9.9	0	35.71
2 multi 2		H						
136	1	05/21/2019	212	1349	2	9.9	0.23	39.2
2 multi 2		H						
136	1	05/22/2019	213	1350	2	9.9	0	39.82
2 multi 2		H						
136	1	05/23/2019	214	1351	2	9.8	0.12	33.37
2 multi 2		H						
136	1	05/24/2019	215	1352	2	9.9	0	38.86
2 multi 2		H						
136	1	05/25/2019	216	1353	2	9.9	0	39.56
2 multi 2		H						
136	1	05/26/2019	217	1354	2	9.9	0	37.77
2 multi 2		H						
136	1	05/27/2019	218	1355	2	9.9	0	35.77
2 multi 2		H						
136	1	05/28/2019	219	1356	2	9.8	0	36.28
2 multi 2		H						
136	1	05/29/2019	220	1357	2	9.9	0	35.88
2 multi 2		H						
136	1	05/30/2019	221	1358	2	10	0	36.27
2 multi 2		H						
136	1	05/31/2019	222	1359	2	10	0	38.37
2 multi 2		H						
136	1	06/01/2019	223	1360	2	9.9	0.08	37.77
2 multi 2		H						
136	1	06/02/2019	224	1361	2	9.8	0	36.21
2 multi 2		H						
136	1	06/03/2019	225	1362	2	9.8	0	34.9
2 multi 2		H						
136	1	06/04/2019	226	1363	2	9.8	0.08	35.83
2 multi 2		H						
136	1	06/05/2019	227	1364	2	10	0	37.26
2 multi 2		H						
136	1	06/06/2019	228	1365	2	10	0.15	36.79
2 multi 2		H						
136	1	06/07/2019	229	1366	2	10	0	35.81
2 multi 2		H						
136	1	06/08/2019	230	1367	2	10	0	35.49
2 multi 2		H						
136	1	06/09/2019	231	1368	2	10.2	0.05	38.76
2 multi 2		H						
136	1	06/10/2019	232	1369	2	10.3	0.04	38.05
2 multi 2		H						
136	1	06/11/2019	233	1370	2	10.2	0	40.44
2 multi 2		H						
136	1	06/12/2019	234	1371	2	9.5	0	34.09

2 multi	2	H						
136	1	06/13/2019	235	1372	2	9.8	0.05	35.28
2 multi	2	H						
136	1	06/14/2019	236	1373	2	10	0.06	36.28
2 multi	2	H						
136	1	06/15/2019	237	1374	2	10.2	0.2	33.19
2 multi	2	H						
136	1	06/16/2019	238	1375	2	10.2	0	35.14
2 multi	2	H						
136	1	06/17/2019	239	1376	2	10	0	34.73
2 multi	2	H						
136	1	06/18/2019	240	1377	2	10.3	0	36.51
2 multi	2	H						
136	1	06/19/2019	241	1378	2	10.3	0	37.11
2 multi	2	H						
136	1	06/20/2019	242	1379	2	10	0	34.81
2 multi	2	H						
136	1	06/21/2019	243	1380	2	10.2	0.07	35.83
2 multi	2	H						
136	1	06/22/2019	244	1381	2	10	0	35.87
2 multi	2	H						
136	1	06/23/2019	245	1382	2	10	0	37.06
2 multi	2	H						
136	1	06/24/2019	246	1383	2	9.8	0	32.89
2 multi	2	H						
136	1	06/25/2019	247	1384	2	9.5	0	16.14
2 multi	2	H						
136	1	06/26/2019	248	1385	2	10.4	0	12.29
2 multi	2	H						
136	1	09/18/2019	17	1469	3	9.6	0.12	22.33
2 multi	1	H						
136	1	09/19/2019	18	1470	3	9.5	0.05	21.49
2 multi	1	H						
136	1	09/20/2019	19	1471	3	9.8	0	22.33
2 multi	1	H						
136	1	09/21/2019	20	1472	3	9.8	0	23.81
2 multi	1	H						
136	1	09/22/2019	21	1473	3	9.8	0	24.76
2 multi	1	H						
136	1	09/23/2019	22	1474	3	9.8	0.12	24.55
2 multi	2	H						
136	1	09/24/2019	23	1475	3	9.9	0.11	25.87
2 multi	2	H						
136	1	09/25/2019	24	1476	3	10.3	0	27.21
2 multi	2	H						
136	1	09/26/2019	25	1477	3	10.2	0.13	26.33
2 multi	2	H						
136	1	09/27/2019	26	1478	3	10.2	0	26.18
2 multi	2	H						
136	1	09/28/2019	27	1479	3	10	0.01	26.46
2 multi	2	H						
136	1	09/29/2019	28	1480	3	9.9	0.12	28.76
2 multi	2	H						
136	1	09/30/2019	29	1481	3	10	0	27.61

2 multi 2		H						
136	1	10/01/2019	30	1482	3	10	0	29.17
2 multi 2		H						
136	1	10/02/2019	31	1483	3	10	0.05	29.18
2 multi 2		H						
136	1	10/03/2019	32	1484	3	9.9	0.14	28.05
2 multi 2		H						
136	1	10/04/2019	33	1485	3	9.5	0.2	27.36
2 multi 2		H						
136	1	10/05/2019	34	1486	3	9.8	0	29.26
2 multi 2		H						
136	1	10/06/2019	35	1487	3	9.9	0.05	29.74
2 multi 2		H						
136	1	10/07/2019	36	1488	3	9.5	0.08	28.06
2 multi 2		H						
136	1	10/08/2019	37	1489	3	9.8	0.15	29.88
2 multi 2		H						
136	1	10/09/2019	38	1490	3	9.8	0.13	30.38
2 multi 2		H						
136	1	10/10/2019	39	1491	3	9.6	0	31.56
2 multi 2		H						
136	1	10/11/2019	40	1492	3	9.6	0.01	31.03
2 multi 2		H						
136	1	10/12/2019	41	1493	3	9.6	0.01	31.09
2 multi 2		H						
136	1	10/13/2019	42	1494	3	9.6	0.11	31.53
2 multi 2		H						
136	1	10/14/2019	43	1495	3	9.6	0	30.31
2 multi 2		H						
136	1	10/15/2019	44	1496	3	9.8	0.2	31.31
2 multi 2		H						
136	1	10/16/2019	45	1497	3	9.8	0.16	31.45
2 multi 2		H						
136	1	10/17/2019	46	1498	3	9.6	0.12	32.37
2 multi 2		H						
136	1	10/18/2019	47	1499	3	9.5	0.06	32.39
2 multi 2		H						
136	1	10/19/2019	48	1500	3	9.8	0.11	32.47
2 multi 2		H						
136	1	10/20/2019	49	1501	3	9.9	0.03	34.36
2 multi 2		H						
136	1	10/21/2019	50	1502	3	9.9	0.15	34.08
2 multi 2		H						
136	1	10/22/2019	51	1503	3	9.5	0	32.9
2 multi 2		H						
136	1	10/23/2019	52	1504	3	9.6	0	32.88
2 multi 2		H						
136	1	10/24/2019	53	1505	3	9.6	0.04	32.83
2 multi 2		H						
136	1	10/25/2019	54	1506	3	9.8	0	33.95
2 multi 2		H						
136	1	10/26/2019	55	1507	3	9.8	0	34.42
2 multi 2		H						
136	1	10/27/2019	56	1508	3	9.6	0.03	34.08

2 multi	2	H						
136	1	10/28/2019	57	1509	3	9.5	0.04	29.98
2 multi	2	H						
136	1	10/29/2019	58	1510	3	9.6	0.12	28.31
2 multi	2	H						
136	1	10/30/2019	59	1511	3	9.8	0.12	31.42
2 multi	2	H						
136	1	10/31/2019	60	1512	3	10	0.17	32.34
2 multi	2	H						
136	1	11/01/2019	61	1513	3	9.9	0.13	32.13
2 multi	2	H						
136	1	11/02/2019	62	1514	3	9.8	0.13	33.32
2 multi	2	H						
136	1	11/03/2019	63	1515	3	9.9	0	33.91
2 multi	2	H						
136	1	11/04/2019	64	1516	3	9.9	0.01	33.77
2 multi	2	H						
136	1	11/05/2019	65	1517	3	9.6	0.13	32.4
2 multi	2	H						
136	1	11/06/2019	66	1518	3	9.6	0	32.16
2 multi	2	H						
136	1	11/07/2019	67	1519	3	9.3	0.05	32.25
2 multi	2	H						
136	1	11/08/2019	68	1520	3	9.8	0.02	29.88
2 multi	2	H						
136	1	11/09/2019	69	1521	3	9.8	0.03	32.31
2 multi	2	H						
136	1	11/10/2019	70	1522	3	9.5	0	33.24
2 multi	2	H						
136	1	09/21/2020	5	1838	4	10.4	0.28	31.39
2 multi	1	H						
136	1	09/22/2020	6	1839	4	10.3	0.11	35.77
2 multi	1	H						
136	1	09/23/2020	7	1840	4	10.3	0.13	37.18
2 multi	1	H						
136	1	09/24/2020	8	1841	4	10.3	0.74	37.32
2 multi	1	H						
136	1	09/25/2020	9	1842	4	10.3	0.04	39.98
2 multi	1	H						
136	1	09/26/2020	10	1843	4	10.3	0.12	40.16
2 multi	1	H						
136	1	09/27/2020	11	1844	4	10.2	0.05	41.7
2 multi	1	H						
136	1	09/28/2020	12	1845	4	10.2	0.05	40.3
2 multi	1	H						
136	1	09/29/2020	13	1846	4	10	0.06	40.99
2 multi	1	H						
136	1	09/30/2020	14	1847	4	9.9	0	43.04
2 multi	1	H						
136	1	10/01/2020	15	1848	4	10	0.05	45.48
2 multi	1	H						
136	1	10/02/2020	16	1849	4	9.9	0	44.86
2 multi	1	H						
136	1	10/03/2020	17	1850	4	9.8	0.12	48.08

2 multi	1	H						
136	1	10/04/2020	18	1851	4	9.8	0.23	46.5
2 multi	1	H						
136	1	10/05/2020	19	1852	4	9.9	0.03	47.53
2 multi	1	H						
136	1	10/06/2020	20	1853	4	9.8	0.07	44.93
2 multi	1	H						
136	1	10/07/2020	21	1854	4	9.6	0.31	43.36
2 multi	1	H						
136	1	10/08/2020	22	1855	4	9.9	0.05	45.61
2 multi	2	H						
136	1	10/09/2020	23	1856	4	10	0.19	46
2 multi	2	H						
136	1	10/10/2020	24	1857	4	10.2	0.11	49.02
2 multi	2	H						
136	1	10/11/2020	25	1858	4	9.9	0.11	49.08
2 multi	2	H						
136	1	10/12/2020	26	1859	4	9.8	0.11	50.35
2 multi	2	H						
136	1	10/13/2020	27	1860	4	9.5	0.03	44.82
2 multi	2	H						
137	1	09/03/2019	3	682	1	9.8	0.23	16.14
1 primi	1	H						
137	1	09/04/2019	4	683	1	10.3	0.05	19.3
1 primi	1	H						
137	1	09/05/2019	5	684	1	10.3	0.04	19.22
1 primi	1	H						
137	1	09/06/2019	6	685	1	10.3	0.04	19.49
1 primi	1	H						
137	1	09/07/2019	7	686	1	10.6	0.02	19.74
1 primi	1	H						
137	1	09/08/2019	8	687	1	10.2	0	20.47
1 primi	1	H						
137	1	09/09/2019	9	688	1	9.9	0	20.68
1 primi	1	H						
137	1	09/10/2019	10	689	1	9.9	0	22.23
1 primi	1	H						
137	1	09/11/2019	11	690	1	10.2	0	22.77
1 primi	1	H						
137	1	09/12/2019	12	691	1	10.2	0.11	22.35
1 primi	1	H						
137	1	09/13/2019	13	692	1	9.8	0	22.53
1 primi	1	H						
137	1	09/14/2019	14	693	1	9.6	0.02	24.28
1 primi	1	H						
137	1	09/15/2019	15	694	1	9.6	0	23.78
1 primi	1	H						
137	1	09/16/2019	16	695	1	9.8	0.07	24.45
1 primi	1	H						
137	1	09/17/2019	17	696	1	10	0	26.31
1 primi	1	H						
137	1	09/18/2019	18	697	1	9.8	0	26.38
1 primi	1	H						
137	1	09/19/2019	19	698	1	9.6	0	25.53

1 primi	1	H						
137	1	09/20/2019	20	699	1	9.8	0.08	26.75
1 primi	1	H						
137	1	09/21/2019	21	700	1	9.8	0	26.5
1 primi	1	H						
137	1	09/22/2019	22	701	1	9.9	0	26.55
1 primi	2	H						
137	1	09/23/2019	23	702	1	9.9	0	26.81
1 primi	2	H						
137	1	09/24/2019	24	703	1	9.8	0	25.43
1 primi	2	H						
137	1	09/25/2019	25	704	1	10	0	28.38
1 primi	2	H						
137	1	09/26/2019	26	705	1	9.9	0.11	25.83
1 primi	2	H						
137	1	09/27/2019	27	706	1	9.8	0.05	27.58
1 primi	2	H						
137	1	09/28/2019	28	707	1	9.9	0	26.44
1 primi	2	H						
137	1	09/29/2019	29	708	1	9.8	0.07	29.27
1 primi	2	H						
137	1	09/30/2019	30	709	1	9.8	0.06	27.45
1 primi	2	H						
137	1	10/01/2019	31	710	1	9.9	0.08	28.56
1 primi	2	H						
137	1	10/02/2019	32	711	1	9.9	0	27.47
1 primi	2	H						
137	1	10/03/2019	33	712	1	9.6	0.15	27.52
1 primi	2	H						
137	1	10/04/2019	34	713	1	9.6	0.07	27.47
1 primi	2	H						
137	1	10/05/2019	35	714	1	9.8	0.07	28.31
1 primi	2	H						
137	1	10/06/2019	36	715	1	9.5	0.08	28.39
1 primi	2	H						
137	1	10/07/2019	37	716	1	9.3	0.08	29.16
1 primi	2	H						
137	1	10/08/2019	38	717	1	9.2	0.02	26.88
1 primi	2	H						
137	1	10/09/2019	39	718	1	9.9	0.06	28.3
1 primi	2	H						
137	1	10/10/2019	40	719	1	9.8	0.07	27.49
1 primi	2	H						
137	1	10/11/2019	41	720	1	9.6	0	29.03
1 primi	2	H						
137	1	10/12/2019	42	721	1	9.3	0	27.66
1 primi	2	H						
137	1	10/13/2019	43	722	1	9.6	0	26.43
1 primi	2	H						
137	1	10/14/2019	44	723	1	9.5	0	27.46
1 primi	2	H						
137	1	10/15/2019	45	724	1	9.5	0.08	28.18
1 primi	2	H						
137	1	10/16/2019	46	725	1	9.5	0	26.82

1 primi	2	H						
137	1	10/17/2019	47	726	1	9.5	0.04	27.41
1 primi	2	H						
137	1	10/18/2019	48	727	1	9.6	0	27.56
1 primi	2	H						
137	1	10/19/2019	49	728	1	9.5	0	28.19
1 primi	2	H						
137	1	10/20/2019	50	729	1	9.6	0	29.06
1 primi	2	H						
137	1	10/21/2019	51	730	1	9.2	0	28.77
1 primi	2	H						
137	1	10/23/2020	4	1098	2	9.6	0.07	33.22
2 multi	1	H						
137	1	10/24/2020	5	1099	2	10.2	0.17	38.82
2 multi	1	H						
137	1	10/25/2020	6	1100	2	10	0.17	40.54
2 multi	1	H						
137	1	10/26/2020	7	1101	2	9.9	0.1	40.89
2 multi	1	H						
137	1	10/27/2020	8	1102	2	10.2	0.09	39.61
2 multi	1	H						
137	1	10/28/2020	9	1103	2	10	0.15	39.11
2 multi	1	H						
137	1	10/29/2020	10	1104	2	9.9	0.05	40.21
2 multi	1	H						
137	1	10/30/2020	11	1105	2	9.9	0	39.53
2 multi	1	H						
137	1	10/31/2020	12	1106	2	9.9	0.05	41.88
2 multi	1	H						
137	1	11/01/2020	13	1107	2	9.6	0.06	41.32
2 multi	1	H						
137	1	11/02/2020	14	1108	2	9.9	0.04	41.55
2 multi	1	H						
137	1	11/03/2020	15	1109	2	9.9	0.14	42.02
2 multi	1	H						
137	1	11/04/2020	16	1110	2	9.8	0.04	45.45
2 multi	1	H						
137	1	11/05/2020	17	1111	2	10	0.01	45.01
2 multi	1	H						
137	1	11/06/2020	18	1112	2	9.8	0.16	41.93
2 multi	1	H						
137	1	11/07/2020	19	1113	2	9.9	0	44.6
2 multi	1	H						
137	1	11/08/2020	20	1114	2	9.8	0.04	43.43
2 multi	1	H						
137	1	11/09/2020	21	1115	2	9.5	0.15	45.04
2 multi	1	H						
137	1	11/10/2020	22	1116	2	9.8	0.03	45.35
2 multi	2	H						
137	1	11/11/2020	23	1117	2	9.9	0.01	47.93
2 multi	2	H						
137	1	11/12/2020	24	1118	2	9.6	0	44.42
2 multi	2	H						
137	1	11/13/2020	25	1119	2	9.5	0.07	45.52

2 multi	2	H						
137	1	11/14/2020	26	1120	2	9.8	0	45.14
2 multi	2	H						
137	1	11/15/2020	27	1121	2	9.6	0.02	44.26
2 multi	2	H						
137	1	11/16/2020	28	1122	2	9.6	0.1	48.58
2 multi	2	H						
137	1	11/17/2020	29	1123	2	9.8	0.01	48.75
2 multi	2	H						
137	1	11/18/2020	30	1124	2	9.3	0.02	48.6
2 multi	2	H						
137	1	11/19/2020	31	1125	2	9.3	0	46.02
2 multi	2	H						
137	1	11/20/2020	32	1126	2	9.5	0	45.15
2 multi	2	H						
137	1	11/21/2020	33	1127	2	9.5	0.55	48.37
2 multi	2	H						
137	1	11/22/2020	34	1128	2	9.6	0.09	49.45
2 multi	2	H						
137	1	11/23/2020	35	1129	2	9.8	0.09	50.14
2 multi	2	H						
137	1	11/24/2020	36	1130	2	9.5	0.11	47.24
2 multi	2	H						
137	1	11/25/2020	37	1131	2	9.6	0.08	48.03
2 multi	2	H						
137	1	11/26/2020	38	1132	2	9.3	0.11	46.23
2 multi	2	H						
137	1	11/27/2020	39	1133	2	9.5	0.02	41.53
2 multi	2	H						
137	1	11/28/2020	40	1134	2	9.5	0	51.21
2 multi	2	H						
137	1	11/29/2020	41	1135	2	9.3	0.1	47.21
2 multi	2	H						
137	1	11/30/2020	42	1136	2	9.3	0.02	46.71
2 multi	2	H						
137	1	12/01/2020	43	1137	2	9.3	0.09	49.24
2 multi	2	H						
137	1	12/02/2020	44	1138	2	9.5	0.11	47.31
2 multi	2	H						
137	1	12/03/2020	45	1139	2	9.6	0.12	45.51
2 multi	2	H						
137	1	12/04/2020	46	1140	2	9.6	0.12	46.04
2 multi	2	H						
137	1	12/05/2020	47	1141	2	9.9	0.1	50.51
2 multi	2	H						
137	1	12/06/2020	48	1142	2	9.8	0.1	51.38
2 multi	2	H						
137	1	12/07/2020	49	1143	2	9.6	0	50.01
2 multi	2	H						
137	1	12/08/2020	50	1144	2	9.6	0.04	47.13
2 multi	2	H						
137	1	12/09/2020	51	1145	2	9.8	0.03	48.46
2 multi	2	H						
137	1	12/10/2020	52	1146	2	9.8	0	47.04

2 multi	2	H						
137	1	12/11/2020	53	1147	2	9.6	0.1	47.47
2 multi	2	H						
137	1	12/12/2020	54	1148	2	9.8	0.11	48.8
2 multi	2	H						
137	1	12/13/2020	55	1149	2	9.9	0.08	47.08
2 multi	2	H						
137	1	12/14/2020	56	1150	2	9.8	0	48.54
2 multi	2	H						
137	1	12/15/2020	57	1151	2	9.8	0	46.31
2 multi	2	H						
137	1	12/16/2020	58	1152	2	9.6	0	47.84
2 multi	2	H						
137	1	12/17/2020	59	1153	2	9.8	0	44.84
2 multi	2	H						
137	1	12/18/2020	60	1154	2	9.8	0.1	44.51
2 multi	2	H						
137	1	12/19/2020	61	1155	2	9.5	0.05	43.77
2 multi	2	H						
137	1	12/20/2020	62	1156	2	9.5	0	45.35
2 multi	2	H						
137	1	12/21/2020	63	1157	2	9.6	0.4	44.23
2 multi	2	H						
137	1	12/22/2020	64	1158	2	9.2	0.06	42.7
2 multi	2	H						
137	1	12/23/2020	65	1159	2	9.3	0.03	43.05
2 multi	2	H						
137	1	12/24/2020	66	1160	2	9.1	0.1	40.07
2 multi	2	H						
137	1	12/25/2020	67	1161	2	9.5	0.02	41.03
2 multi	2	H						
137	1	12/26/2020	68	1162	2	8.9	0.1	40.62
2 multi	2	H						
139	1	09/18/2020	15	712	1	8.4	0.28	24.86
1 primi	1	H						
139	1	09/19/2020	16	713	1	8.8	0.06	28.33
1 primi	1	H						
139	1	09/20/2020	17	714	1	8.6	0.23	28.07
1 primi	1	H						
139	1	09/21/2020	18	715	1	8.9	0.14	29.08
1 primi	1	H						
139	1	09/22/2020	19	716	1	8.9	0.11	30.58
1 primi	1	H						
139	1	09/23/2020	20	717	1	8.8	0.04	30.94
1 primi	1	H						
139	1	09/24/2020	21	718	1	9.1	0.04	30.93
1 primi	1	H						
139	1	09/25/2020	22	719	1	8.9	0	32.55
1 primi	2	H						
139	1	09/26/2020	23	720	1	8.9	0.11	32.69
1 primi	2	H						
139	1	09/27/2020	24	721	1	8.9	0	32.51
1 primi	2	H						
139	1	09/28/2020	25	722	1	8.7	0.11	34.21

1 primi 2 H
139 1 09/29/2020 26 723 1 8.8 0.28 35.07
1 primi 2 H
139 1 09/30/2020 27 724 1 8.4 0.3 35.16
1 primi 2 H
139 1 10/01/2020 28 725 1 8.6 0.09 34.39
1 primi 2 H
139 1 10/02/2020 29 726 1 8.9 0.07 34.12
1 primi 2 H
139 1 10/03/2020 30 727 1 8.8 0.11 33.6
1 primi 2 H
139 1 10/04/2020 31 728 1 8.5 0.03 34.99
1 primi 2 H
139 1 10/05/2020 32 729 1 8.4 0.11 34.43
1 primi 2 H
139 1 10/06/2020 33 730 1 8.5 0.02 34.27
1 primi 2 H
139 1 10/07/2020 34 731 1 8.4 0.02 32.59
1 primi 2 H
139 1 10/08/2020 35 732 1 8.5 0.03 32.84
1 primi 2 H
139 1 10/09/2020 36 733 1 8.4 0.35 35.62
1 primi 2 H
139 1 10/10/2020 37 734 1 8.5 0.04 33.31
1 primi 2 H
139 1 10/11/2020 38 735 1 8.8 0 36.12
1 primi 2 H
139 1 10/12/2020 39 736 1 8.6 0.01 35.05
1 primi 2 H
139 1 10/13/2020 40 737 1 8.5 0.05 35.75
1 primi 2 H
140 1 01/22/2020 3 747 1 9.8 0.28 16.88
1 primi 1 H
140 1 01/23/2020 4 748 1 10.7 0.15 26.59
1 primi 1 H
140 1 01/24/2020 5 749 1 9.9 0.07 21.4
1 primi 1 H
140 1 01/25/2020 6 750 1 9.6 0.05 24.97
1 primi 1 H
140 1 01/26/2020 7 751 1 9.6 0 24.72
1 primi 1 H
140 1 01/27/2020 8 752 1 9.2 0.24 27.53
1 primi 1 H
140 1 01/28/2020 9 753 1 9.3 0.06 25.81
1 primi 1 H
140 1 01/29/2020 10 754 1 10.2 0.04 39.09
1 primi 1 H
140 1 01/30/2020 11 755 1 8.9 0 25.08
1 primi 1 H
140 1 01/31/2020 12 756 1 9.1 0.23 29.81
1 primi 1 H
140 1 02/01/2020 13 757 1 8.6 0 30.53
1 primi 1 H
140 1 02/02/2020 14 758 1 8.8 0.25 31.29

1 primi 1 H
140 1 02/03/2020 15 759 1 8.7 0.08 30.71
1 primi 1 H
140 1 02/04/2020 16 760 1 10.3 0.08 28.02
1 primi 1 H
140 1 02/05/2020 17 761 1 8.8 0.1 26.41
1 primi 1 H
140 1 02/06/2020 18 762 1 8.8 0.01 30
1 primi 1 H
140 1 02/07/2020 19 763 1 8.8 0.38 31.7
1 primi 1 H
140 1 02/08/2020 20 764 1 8.9 0.1 28.85
1 primi 1 H
140 1 02/09/2020 21 765 1 9.1 0 36.07
1 primi 1 H
140 1 02/10/2020 22 766 1 8.6 0 32.17
1 primi 2 H
140 1 02/11/2020 23 767 1 8.3 0 35.57
1 primi 2 H
140 1 02/12/2020 24 768 1 8.6 0.35 36.21
1 primi 2 H
140 1 02/13/2020 25 769 1 8.4 0 36.18
1 primi 2 H
140 1 02/14/2020 26 770 1 8.7 0 36.82
1 primi 2 H
140 1 02/15/2020 27 771 1 8.3 0.22 35.64
1 primi 2 H
140 1 02/16/2020 28 772 1 8.5 0.07 35.54
1 primi 2 H
140 1 02/17/2020 29 773 1 8.6 0 37.62
1 primi 2 H
140 1 02/18/2020 30 774 1 8.4 0.01 35.46
1 primi 2 H
140 1 02/19/2020 31 775 1 8.5 0.19 35.86
1 primi 2 H
140 1 02/20/2020 32 776 1 8.8 0 32.64
1 primi 2 H
140 1 02/21/2020 33 777 1 8.7 0 30.39
1 primi 2 H
140 1 02/22/2020 34 778 1 8.7 0.01 30.21
1 primi 2 H
140 1 02/23/2020 35 779 1 8.3 0.1 34.85
1 primi 2 H
140 1 02/24/2020 36 780 1 8.5 0.14 34.76
1 primi 2 H
140 1 02/25/2020 37 781 1 8.5 0.1 35.43
1 primi 2 H
140 1 02/26/2020 38 782 1 8.8 0.08 34.01
1 primi 2 H
140 1 02/27/2020 39 783 1 8.6 0.32 36.17
1 primi 2 H
140 1 02/28/2020 40 784 1 8.5 0.31 35.91
1 primi 2 H
140 1 02/29/2020 41 785 1 8.5 0.07 33.71

1 primi	2	H						
140	1	03/01/2020	42	786	1	8.5	0.07	37.69
1 primi	2	H						
140	1	03/02/2020	43	787	1	8.8	0.05	38.09
1 primi	2	H						
140	1	03/03/2020	44	788	1	8.9	0.07	37.24
1 primi	2	H						
140	1	03/04/2020	45	789	1	9.1	0.03	36.82
1 primi	2	H						
140	1	03/05/2020	46	790	1	8.8	0.15	37.02
1 primi	2	H						
140	1	03/06/2020	47	791	1	8.7	0	37.36
1 primi	2	H						
140	1	03/07/2020	48	792	1	8.8	0	35.4
1 primi	2	H						
140	1	03/08/2020	49	793	1	9.1	0.13	34.3
1 primi	2	H						
140	1	03/09/2020	50	794	1	8.8	0.43	36.82
1 primi	2	H						
140	1	03/10/2020	51	795	1	8.7	0	35.74
1 primi	2	H						
140	1	03/11/2020	52	796	1	8.7	0.17	34.35
1 primi	2	H						
140	1	03/12/2020	53	797	1	9.2	0.22	33.48
1 primi	2	H						
140	1	03/13/2020	54	798	1	8.9	0	35.18
1 primi	2	H						
140	1	03/14/2020	55	799	1	8.4	0	34.73
1 primi	2	H						
140	1	03/15/2020	56	800	1	8.3	0.09	33.94
1 primi	2	H						
140	1	03/16/2020	57	801	1	9.1	0	34.34
1 primi	2	H						
140	1	03/17/2020	58	802	1	8.7	0	37.34
1 primi	2	H						
140	1	03/18/2020	59	803	1	8.7	0.18	35.59
1 primi	2	H						
140	1	03/19/2020	60	804	1	9.3	0.01	38.09
1 primi	2	H						
140	1	03/20/2020	61	805	1	9.3	0.08	36.89
1 primi	2	H						
140	1	03/21/2020	62	806	1	9.1	0	36.34
1 primi	2	H						
140	1	03/22/2020	63	807	1	8.8	0	36.23
1 primi	2	H						
140	1	03/23/2020	64	808	1	8.5	0	34.2
1 primi	2	H						
140	1	03/24/2020	65	809	1	8.2	0.26	35.13
1 primi	2	H						
140	1	03/25/2020	66	810	1	9.1	0	33.88
1 primi	2	H						
140	1	03/26/2020	67	811	1	8.8	0	38.21
1 primi	2	H						
140	1	03/27/2020	68	812	1	8.5	0.1	35.29

1 primi	2	H						
140	1	03/28/2020	69	813	1	8.5	0	31.65
1 primi	2	H						
140	1	03/29/2020	70	814	1	8.4	0	32.58
1 primi	2	H						
140	1	03/30/2020	71	815	1	8.5	0	34.46
1 primi	2	H						
142	1	01/28/2019	14	870	1	8.2	0.18	18.88
1 primi	1	H						
142	1	01/29/2019	15	871	1	8.8	0	20.32
1 primi	1	H						
142	1	01/30/2019	16	872	1	8.9	0	16.94
1 primi	1	H						
142	1	01/31/2019	17	873	1	8.5	0	20.02
1 primi	1	H						
142	1	02/01/2019	18	874	1	8.5	0.06	18.25
1 primi	1	H						
142	1	02/02/2019	19	875	1	8.8	0.06	19.53
1 primi	1	H						
142	1	02/03/2019	20	876	1	8.4	0.21	20.51
1 primi	1	H						
142	1	02/04/2019	21	877	1	8.7	0.06	21.34
1 primi	1	H						
142	1	02/05/2019	22	878	1	8.7	0.05	20.09
1 primi	2	H						
142	1	02/06/2019	23	879	1	8.7	0.06	20.94
1 primi	2	H						
142	1	02/07/2019	24	880	1	8.7	0.26	20.82
1 primi	2	H						
142	1	02/08/2019	25	881	1	8.3	0.21	23.29
1 primi	2	H						
142	1	02/09/2019	26	882	1	8.7	0.09	22.02
1 primi	2	H						
142	1	02/10/2019	27	883	1	8.6	0	22.14
1 primi	2	H						
142	1	02/11/2019	28	884	1	8.4	0.17	21.4
1 primi	2	H						
142	1	02/12/2019	29	885	1	9.1	0.07	23.23
1 primi	2	H						
142	1	02/13/2019	30	886	1	9.1	0.05	22.79
1 primi	2	H						
142	1	02/14/2019	31	887	1	8.8	0.23	22.09
1 primi	2	H						
142	1	02/15/2019	32	888	1	8.7	0.2	21.59
1 primi	2	H						
142	1	02/16/2019	33	889	1	8.5	0.09	21.4
1 primi	2	H						
142	1	02/17/2019	34	890	1	8.7	0.06	24.76
1 primi	2	H						
142	1	02/18/2019	35	891	1	8.3	0.12	21.76
1 primi	2	H						
142	1	02/19/2019	36	892	1	8.6	0	23.46
1 primi	2	H						
142	1	02/20/2019	37	893	1	8.1	0	20.62

1 primi 2		H						
142	1	01/20/2020	6	1227	2	9.6	0.25	16.6
2 multi 1		H						
142	1	01/21/2020	7	1228	2	9.6	0.23	17.82
2 multi 1		H						
142	1	01/22/2020	8	1229	2	9.9	0.14	16.52
2 multi 1		H						
142	1	01/23/2020	9	1230	2	9.8	0.22	17.36
2 multi 1		H						
142	1	01/24/2020	10	1231	2	10	0.11	18.14
2 multi 1		H						
142	1	01/25/2020	11	1232	2	9.8	0	18.41
2 multi 1		H						
142	1	01/26/2020	12	1233	2	9.3	0	20.44
2 multi 1		H						
142	1	01/27/2020	13	1234	2	9.5	0.03	20.86
2 multi 1		H						
142	1	01/28/2020	14	1235	2	9.1	0.18	20.64
2 multi 1		H						
142	1	01/29/2020	15	1236	2	9.1	0.22	20.29
2 multi 1		H						
142	1	01/30/2020	16	1237	2	9.5	0.16	21.78
2 multi 1		H						
142	1	01/31/2020	17	1238	2	9.8	0.2	21.53
2 multi 1		H						
142	1	02/01/2020	18	1239	2	9.3	0.09	23.23
2 multi 1		H						
142	1	02/02/2020	19	1240	2	9.2	0.19	23.27
2 multi 1		H						
142	1	02/03/2020	20	1241	2	9.5	0.23	24.34
2 multi 1		H						
142	1	02/04/2020	21	1242	2	9.8	0.16	25.2
2 multi 1		H						
142	1	02/05/2020	22	1243	2	9.2	0.2	23.8
2 multi 2		H						
142	1	02/06/2020	23	1244	2	9.2	0.13	24.67
2 multi 2		H						
142	1	02/07/2020	24	1245	2	9.3	0.22	25.89
2 multi 2		H						
142	1	02/08/2020	25	1246	2	9.3	0.11	24.99
2 multi 2		H						
142	1	02/09/2020	26	1247	2	9.2	0.14	25.97
2 multi 2		H						
142	1	02/10/2020	27	1248	2	9.2	0.16	23.27
2 multi 2		H						
142	1	02/11/2020	28	1249	2	9.3	0.26	23.13
2 multi 2		H						
142	1	02/12/2020	29	1250	2	9.3	0.07	22.85
2 multi 2		H						
142	1	02/13/2020	30	1251	2	9.2	0	22.59
2 multi 2		H						
142	1	02/14/2020	31	1252	2	9.2	0.25	21.96
2 multi 2		H						
142	1	02/15/2020	32	1253	2	9.1	0.34	23.62

2 multi	2	H						
142	1	02/16/2020	33	1254	2	9.2	0.27	24.9
2 multi	2	H						
142	1	02/17/2020	34	1255	2	9.1	0.32	25.43
2 multi	2	H						
142	1	02/18/2020	35	1256	2	8.8	0.21	24.38
2 multi	2	H						
142	1	02/19/2020	36	1257	2	9.6	0.13	25.5
2 multi	2	H						
142	1	02/20/2020	37	1258	2	9.2	0.12	23.45
2 multi	2	H						
142	1	02/21/2020	38	1259	2	9.2	0.03	25.65
2 multi	2	H						
142	1	02/22/2020	39	1260	2	9.2	0	25.67
2 multi	2	H						
142	1	02/23/2020	40	1261	2	9.6	0	23.97
2 multi	2	H						
142	1	02/24/2020	41	1262	2	9.6	0	28.04
2 multi	2	H						
142	1	02/25/2020	42	1263	2	9.6	0.06	26.34
2 multi	2	H						
142	1	02/26/2020	43	1264	2	9.8	0.07	26.43
2 multi	2	H						
142	1	02/27/2020	44	1265	2	9.6	0.09	25.87
2 multi	2	H						
142	1	02/28/2020	45	1266	2	9.1	0.23	25.18
2 multi	2	H						
142	1	02/29/2020	46	1267	2	9.2	0.01	26.14
2 multi	2	H						
142	1	03/01/2020	47	1268	2	8.7	0.38	25.26
2 multi	2	H						
142	1	03/02/2020	48	1269	2	8.8	0.19	25.95
2 multi	2	H						
142	1	03/03/2020	49	1270	2	8.9	0.17	22.97
2 multi	2	H						
142	1	03/04/2020	50	1271	2	8.9	0.15	22.8
2 multi	2	H						
142	1	01/02/2021	4	1575	3	9.8	0.22	22.72
2 multi	1	H						
142	1	01/03/2021	5	1576	3	9.5	0.2	25.5
2 multi	1	H						
142	1	01/04/2021	6	1577	3	9.1	0.15	27.42
2 multi	1	H						
142	1	01/05/2021	7	1578	3	9.5	0.19	29.01
2 multi	1	H						
142	1	01/06/2021	8	1579	3	8.8	0.23	29.52
2 multi	1	H						
142	1	01/07/2021	9	1580	3	8.8	0.09	31.59
2 multi	1	H						
142	1	01/08/2021	10	1581	3	9.3	0.02	30.08
2 multi	1	H						
142	1	01/09/2021	11	1582	3	9.1	0.02	30.95
2 multi	1	H						
142	1	01/10/2021	12	1583	3	9.2	0.08	31.18

2 multi	1	H						
142	1	01/11/2021	13	1584	3	8.9	0.19	32.18
2 multi	1	H						
142	1	01/12/2021	14	1585	3	9.2	0.1	31.53
2 multi	1	H						
142	1	01/13/2021	15	1586	3	8.9	0.14	31.5
2 multi	1	H						
142	1	01/14/2021	16	1587	3	9.3	0.08	33.01
2 multi	1	H						
142	1	01/15/2021	17	1588	3	8.8	0.22	29.55
2 multi	1	H						
142	1	01/16/2021	18	1589	3	8.8	0.11	31.24
2 multi	1	H						
142	1	01/17/2021	19	1590	3	9.1	0.04	32.89
2 multi	1	H						
142	1	01/18/2021	20	1591	3	9.1	0.06	34.45
2 multi	1	H						
142	1	01/19/2021	21	1592	3	9.1	0.03	31.43
2 multi	1	H						
142	1	01/20/2021	22	1593	3	8.8	0.04	32.24
2 multi	2	H						
142	1	01/21/2021	23	1594	3	9.3	0.21	35.24
2 multi	2	H						
142	1	01/22/2021	24	1595	3	9.2	0.14	35.35
2 multi	2	H						
142	1	01/23/2021	25	1596	3	9.1	0.08	33.48
2 multi	2	H						
142	1	01/24/2021	26	1597	3	9.2	0.03	34.72
2 multi	2	H						
142	1	01/25/2021	27	1598	3	9.2	0.18	34.93
2 multi	2	H						
142	1	01/26/2021	28	1599	3	8.7	0	35.86
2 multi	2	H						
142	1	01/27/2021	29	1600	3	9.2	0.07	34.58
2 multi	2	H						
142	1	01/28/2021	30	1601	3	9.2	0.03	34.3
2 multi	2	H						
142	1	01/29/2021	31	1602	3	9.1	0.02	33.7
2 multi	2	H						
142	1	01/30/2021	32	1603	3	8.7	0.08	34.59
2 multi	2	H						
142	1	01/31/2021	33	1604	3	8.8	0	35.78
2 multi	2	H						
142	1	02/01/2021	34	1605	3	9.2	0.11	36.47
2 multi	2	H						
142	1	02/02/2021	35	1606	3	9.1	0.02	34.83
2 multi	2	H						
142	1	02/03/2021	36	1607	3	9.2	0.04	34.93
2 multi	2	H						
144	1	05/06/2019	5	696	1	9.2	0.13	21.07
1 primi	1	H						
144	1	05/07/2019	6	697	1	8.8	0.02	21.82
1 primi	1	H						
144	1	05/08/2019	7	698	1	9.6	0.04	23.27

1 primi 1 H
144 1 05/09/2019 8 699 1 9.5 0.06 22.76
1 primi 1 H
144 1 05/10/2019 9 700 1 9.2 0.01 23.18
1 primi 1 H
144 1 05/11/2019 10 701 1 9.9 0 24.51
1 primi 1 H
144 1 05/12/2019 11 702 1 9.5 0 23.22
1 primi 1 H
144 1 05/13/2019 12 703 1 9.1 0 24.64
1 primi 1 H
144 1 05/14/2019 13 704 1 8.9 0 24.05
1 primi 1 H
144 1 05/15/2019 14 705 1 9.2 0 23.41
1 primi 1 H
144 1 05/16/2019 15 706 1 8.9 0 23.92
1 primi 1 H
144 1 05/17/2019 16 707 1 9.1 0 22.51
1 primi 1 H
144 1 05/18/2019 17 708 1 8.9 0 27.15
1 primi 1 H
144 1 05/19/2019 18 709 1 9.3 0 27.25
1 primi 1 H
144 1 05/20/2019 19 710 1 9.2 0 26.28
1 primi 1 H
144 1 05/21/2019 20 711 1 9.2 0 25.79
1 primi 1 H
144 1 05/22/2019 21 712 1 9.1 0.11 27.87
1 primi 1 H
144 1 05/23/2019 22 713 1 9.1 0 28.74
1 primi 2 H
144 1 05/24/2019 23 714 1 8.9 0 27.07
1 primi 2 H
144 1 05/25/2019 24 715 1 8.8 0 27.25
1 primi 2 H
144 1 05/26/2019 25 716 1 8.8 0 28.25
1 primi 2 H
144 1 05/27/2019 26 717 1 8.8 0 27.97
1 primi 2 H
144 1 05/28/2019 27 718 1 9.1 0 26.08
1 primi 2 H
144 1 05/29/2019 28 719 1 9.1 0.01 27.02
1 primi 2 H
144 1 05/30/2019 29 720 1 8.8 0.01 27.91
1 primi 2 H
144 1 05/31/2019 30 721 1 8.7 0.31 31.04
1 primi 2 H
144 1 06/01/2019 31 722 1 8.8 0.01 28.74
1 primi 2 H
144 1 06/02/2019 32 723 1 8.7 0.02 26.5
1 primi 2 H
144 1 06/03/2019 33 724 1 8.8 0.03 29.69
1 primi 2 H
144 1 06/04/2019 34 725 1 8.8 0.14 29.22

1 primi	2	H						
144	1	06/05/2019	35	726	1	8.8	0.02	28.65
1 primi	2	H						
144	1	06/06/2019	36	727	1	8.7	0.03	29.04
1 primi	2	H						
144	1	06/07/2019	37	728	1	8.9	0	22.44
1 primi	2	H						
144	1	06/08/2019	38	729	1	8.8	0.01	27.18
1 primi	2	H						
144	1	06/09/2019	39	730	1	9.1	0	26.54
1 primi	2	H						
144	1	06/10/2019	40	731	1	9.5	0	29.03
1 primi	2	H						
144	1	06/11/2019	41	732	1	9.2	0.24	29.63
1 primi	2	H						
144	1	06/12/2019	42	733	1	9.2	0.01	31.6
1 primi	2	H						
144	1	06/13/2019	43	734	1	9.2	0.26	27.9
1 primi	2	H						
144	1	06/14/2019	44	735	1	8.8	0.08	29.3
1 primi	2	H						
144	1	06/15/2019	45	736	1	8.9	0.09	28.02
1 primi	2	H						
144	1	06/16/2019	46	737	1	8.9	0.09	30.05
1 primi	2	H						
144	1	06/17/2019	47	738	1	8.8	0	29.28
1 primi	2	H						
144	1	06/18/2019	48	739	1	8.9	0.02	28.9
1 primi	2	H						
144	1	06/19/2019	49	740	1	9.1	0.01	28.79
1 primi	2	H						
144	1	06/20/2019	50	741	1	9.1	0.07	29.16
1 primi	2	H						
144	1	06/21/2019	51	742	1	9.1	0	29.62
1 primi	2	H						
144	1	06/22/2019	52	743	1	8.9	0.01	29.38
1 primi	2	H						
144	1	06/23/2019	53	744	1	8.7	0.3	28.81
1 primi	2	H						
144	1	06/24/2019	54	745	1	8.8	0.04	30.68
1 primi	2	H						
144	1	06/25/2019	55	746	1	9.1	0.03	30.59
1 primi	2	H						
144	1	06/26/2019	56	747	1	9.1	0	31.39
1 primi	2	H						
144	1	06/27/2019	57	748	1	9.2	0.1	31.12
1 primi	2	H						
144	1	06/28/2019	58	749	1	9.2	0.02	26.51
1 primi	2	H						
144	1	06/29/2019	59	750	1	9.2	0.02	29.94
1 primi	2	H						
144	1	06/30/2019	60	751	1	9.2	0.06	31.09
1 primi	2	H						
144	1	07/01/2019	61	752	1	9.2	0.01	30.76

1 primi 2		H						
144	1	07/02/2019	62	753	1	9.2	0.04	30.04
1 primi 2		H						
144	1	07/03/2019	63	754	1	9.1	0	32.01
1 primi 2		H						
144	1	07/04/2019	64	755	1	9.2	0	31.85
1 primi 2		H						
144	1	07/05/2019	65	756	1	9.3	0	30.96
1 primi 2		H						
144	1	07/06/2019	66	757	1	9.6	0.05	30.48
1 primi 2		H						
144	1	07/07/2019	67	758	1	9.6	0	27.19
1 primi 2		H						
144	1	07/08/2019	68	759	1	8.6	0.01	27.62
1 primi 2		H						
144	1	07/09/2019	69	760	1	8.9	0.03	27.94
1 primi 2		H						
144	1	07/10/2019	70	761	1	9.2	0.06	28.96
1 primi 2		H						
144	1	07/11/2019	71	762	1	8.8	0.22	27.35
1 primi 2		H						
144	1	07/12/2019	72	763	1	9.6	0.02	29.45
1 primi 2		H						
144	1	07/13/2019	73	764	1	9.1	0.02	29.29
1 primi 2		H						
144	1	07/14/2019	74	765	1	9.2	0.01	32.06
1 primi 2		H						
144	1	07/15/2019	75	766	1	8.9	0.09	34.31
1 primi 2		H						
144	1	07/16/2019	76	767	1	8.9	0.13	32.26
1 primi 2		H						
144	1	07/17/2019	77	768	1	9.1	0	30.64
1 primi 2		H						
144	1	07/18/2019	78	769	1	9.5	0.01	28.4
1 primi 2		H						
144	1	07/19/2019	79	770	1	9.8	0.19	30.1
1 primi 2		H						
144	1	07/20/2019	80	771	1	9.3	0	30.92
1 primi 2		H						
144	1	07/21/2019	81	772	1	8.9	0.19	30.55
1 primi 2		H						
144	1	07/22/2019	82	773	1	9.3	0.02	30.02
1 primi 2		H						
144	1	07/23/2019	83	774	1	9.3	0.01	30.58
1 primi 2		H						
144	1	07/24/2019	84	775	1	9.1	0.06	29.39
1 primi 2		H						
144	1	07/25/2019	85	776	1	9.3	0.04	29.75
1 primi 2		H						
144	1	07/26/2019	86	777	1	9.8	0.03	27.55
1 primi 2		H						
144	1	07/27/2019	87	778	1	9.8	0	29.24
1 primi 2		H						
144	1	07/28/2019	88	779	1	9.3	0.15	29.48

1 primi 2 H
144 1 07/29/2019 89 780 1 9.5 0.01 29.56
1 primi 2 H
144 1 07/30/2019 90 781 1 9.3 0 30.55
1 primi 2 H
144 1 07/31/2019 91 782 1 9.5 0 31
1 primi 2 H
144 1 04/22/2020 5 1048 2 9.3 0.12 25.91
2 multi 1 H
144 1 04/23/2020 6 1049 2 8.8 0.12 24.67
2 multi 1 H
144 1 04/24/2020 7 1050 2 9.2 0 27.23
2 multi 1 H
144 1 04/25/2020 8 1051 2 9.1 0.06 29.92
2 multi 1 H
144 1 04/26/2020 9 1052 2 9.2 0.06 30.59
2 multi 1 H
144 1 04/27/2020 10 1053 2 9.3 0.01 31.54
2 multi 1 H
144 1 04/28/2020 11 1054 2 9.3 0.07 36.4
2 multi 1 H
144 1 04/29/2020 12 1055 2 9.6 0 34.05
2 multi 1 H
144 1 04/30/2020 13 1056 2 9.5 0 31.18
2 multi 1 H
144 1 05/01/2020 14 1057 2 9.8 0 36.74
2 multi 1 H
144 1 05/02/2020 15 1058 2 9.5 0.27 36.11
2 multi 1 H
144 1 05/03/2020 16 1059 2 9.5 0.03 38.96
2 multi 1 H
144 1 05/04/2020 17 1060 2 9.6 0.02 38.46
2 multi 1 H
144 1 05/05/2020 18 1061 2 9.5 0 36.34
2 multi 1 H
144 1 05/06/2020 19 1062 2 9.3 0.02 39.82
2 multi 1 H
144 1 05/07/2020 20 1063 2 9.6 0 39.6
2 multi 1 H
144 1 05/08/2020 21 1064 2 9.6 0.02 41.98
2 multi 1 H
144 1 05/09/2020 22 1065 2 9.2 0.02 38.64
2 multi 2 H
144 1 05/10/2020 23 1066 2 9.3 0.15 38.95
2 multi 2 H
144 1 05/11/2020 24 1067 2 9.2 0 39.36
2 multi 2 H
144 1 05/12/2020 25 1068 2 9.6 0.02 39.07
2 multi 2 H
144 1 05/13/2020 26 1069 2 8.9 0 41.62
2 multi 2 H
144 1 05/14/2020 27 1070 2 9.1 0 40.22
2 multi 2 H
144 1 05/15/2020 28 1071 2 9.1 0.03 38.95

2 multi	2	H						
144	1	05/16/2020	29	1072	2	9.2	0.5	42.25
2 multi	2	H						
144	1	05/17/2020	30	1073	2	9.2	0.12	40.94
2 multi	2	H						
144	1	05/18/2020	31	1074	2	8.9	0.06	37.69
2 multi	2	H						
144	1	05/19/2020	32	1075	2	8.9	0	37.99
2 multi	2	H						
144	1	05/20/2020	33	1076	2	8.8	0.07	40.03
2 multi	2	H						
144	1	05/21/2020	34	1077	2	9.2	0.06	40.73
2 multi	2	H						
144	1	05/22/2020	35	1078	2	9.2	0.02	39.39
2 multi	2	H						
144	1	05/23/2020	36	1079	2	9.3	0.05	38.39
2 multi	2	H						
144	1	05/24/2020	37	1080	2	9.3	0.06	39.62
2 multi	2	H						
144	1	05/25/2020	38	1081	2	9.5	0	36.25
2 multi	2	H						
144	1	05/26/2020	39	1082	2	9.6	0.02	39.43
2 multi	2	H						
144	1	05/27/2020	40	1083	2	9.5	0.03	39.31
2 multi	2	H						
144	1	05/28/2020	41	1084	2	9.5	0.08	39.21
2 multi	2	H						
144	1	05/07/2021	10	1428	3	8.7	0.21	28.66
2 multi	1	H						
144	1	05/08/2021	11	1429	3	9.2	0.23	35.55
2 multi	1	H						
144	1	05/09/2021	12	1430	3	9.2	0.08	33.79
2 multi	1	H						
144	1	05/10/2021	13	1431	3	9.6	0.07	36.84
2 multi	1	H						
144	1	05/11/2021	14	1432	3	9.6	0	35.77
2 multi	1	H						
144	1	05/12/2021	15	1433	3	9.8	0.06	37.67
2 multi	1	H						
144	1	05/13/2021	16	1434	3	9.8	0	38.03
2 multi	1	H						
144	1	05/14/2021	17	1435	3	9.8	0.07	40.63
2 multi	1	H						
144	1	05/15/2021	18	1436	3	9.9	0.06	42.94
2 multi	1	H						
144	1	05/16/2021	19	1437	3	9.8	0.06	41.56
2 multi	1	H						
144	1	05/17/2021	20	1438	3	9.9	0	39.32
2 multi	1	H						
144	1	05/18/2021	21	1439	3	9.5	0	39.2
2 multi	1	H						
144	1	05/19/2021	22	1440	3	9.2	0.05	37.72
2 multi	2	H						
144	1	05/20/2021	23	1441	3	9.3	0.39	42.1

2 multi	2	H						
144	1	05/21/2021	24	1442	3	9.5	0.05	44.36
2 multi	2	H						
144	1	05/22/2021	25	1443	3	9.6	0	45.09
2 multi	2	H						
144	1	05/23/2021	26	1444	3	9.8	0	42.45
2 multi	2	H						
144	1	05/24/2021	27	1445	3	9.5	0.06	45.14
2 multi	2	H						
144	1	05/25/2021	28	1446	3	9.3	0.79	39.89
2 multi	2	H						
144	1	05/26/2021	29	1447	3	9.2	0	19.81
2 multi	2	H						
145	1	07/13/2020	9	943	1	9.6	0.24	22
1 primi	1	H						
145	1	07/14/2020	10	944	1	9.3	0	21.21
1 primi	1	H						
145	1	07/15/2020	11	945	1	11.9	0.04	26.43
1 primi	1	H						
145	1	07/16/2020	12	946	1	10.4	0.5	26.51
1 primi	1	H						
145	1	07/17/2020	13	947	1	9.9	0	27.05
1 primi	1	H						
145	1	07/18/2020	14	948	1	10	0.13	32.55
1 primi	1	H						
145	1	07/19/2020	15	949	1	10.2	0.11	33.5
1 primi	1	H						
145	1	07/20/2020	16	950	1	9.9	0.15	31.9
1 primi	1	H						
145	1	07/21/2020	17	951	1	10.9	0.03	33.28
1 primi	1	H						
145	1	07/22/2020	18	952	1	10.2	0	30.03
1 primi	1	H						
145	1	07/23/2020	19	953	1	10	0.05	33.13
1 primi	1	H						
145	1	07/24/2020	20	954	1	10.2	0.01	34.12
1 primi	1	H						
145	1	07/25/2020	21	955	1	9.8	0.11	32.36
1 primi	1	H						
145	1	07/26/2020	22	956	1	10	0.1	30.78
1 primi	2	H						
145	1	07/27/2020	23	957	1	10.2	0.23	30.76
1 primi	2	H						
145	1	07/28/2020	24	958	1	9.9	0.09	30.4
1 primi	2	H						
145	1	07/29/2020	25	959	1	9.9	0.22	31.64
1 primi	2	H						
145	1	07/30/2020	26	960	1	9.9	0.2	32.88
1 primi	2	H						
145	1	07/31/2020	27	961	1	9.9	0.03	31.83
1 primi	2	H						
145	1	08/01/2020	28	962	1	9.8	0.11	31.34
1 primi	2	H						
145	1	08/02/2020	29	963	1	10	0.11	37.16

1 primi	2	H						
145	1	08/03/2020	30	964	1	9.9	0.03	35.64
1 primi	2	H						
145	1	08/04/2020	31	965	1	10	0	34.68
1 primi	2	H						
145	1	08/05/2020	32	966	1	9.9	0.04	36.29
1 primi	2	H						
145	1	08/06/2020	33	967	1	9.8	0.11	35.1
1 primi	2	H						
145	1	08/07/2020	34	968	1	9.6	0.1	33.14
1 primi	2	H						
145	1	08/08/2020	35	969	1	9.8	0	29.46
1 primi	2	H						
145	1	08/09/2020	36	970	1	9.8	0.1	32.42
1 primi	2	H						
145	1	08/10/2020	37	971	1	10	0.09	34.66
1 primi	2	H						
145	1	08/11/2020	38	972	1	9.9	0.36	37.51
1 primi	2	H						
145	1	08/12/2020	39	973	1	10	0.1	36.29
1 primi	2	H						
145	1	08/13/2020	40	974	1	10	0	37.22
1 primi	2	H						
145	1	08/14/2020	41	975	1	10	0.1	35.54
1 primi	2	H						
145	1	08/15/2020	42	976	1	10	0	34.52
1 primi	2	H						
145	1	08/16/2020	43	977	1	10	0.03	33.1
1 primi	2	H						
145	1	08/17/2020	44	978	1	9.8	0.02	33.32
1 primi	2	H						
145	1	08/18/2020	45	979	1	9.8	0.23	33.7
1 primi	2	H						
145	1	08/19/2020	46	980	1	10	0.03	34.05
1 primi	2	H						
145	1	08/20/2020	47	981	1	10	0.1	37.57
1 primi	2	H						
145	1	08/21/2020	48	982	1	9.9	0.03	37.89
1 primi	2	H						
145	1	08/22/2020	49	983	1	9.9	0.1	35.67
1 primi	2	H						
145	1	08/23/2020	50	984	1	10	0.03	34.34
1 primi	2	H						
145	1	08/24/2020	51	985	1	10	0.14	35.82
1 primi	2	H						
145	1	08/25/2020	52	986	1	9.8	0.25	34.29
1 primi	2	H						
145	1	08/26/2020	53	987	1	10.2	0.12	35.65
1 primi	2	H						
145	1	08/27/2020	54	988	1	10	0	33.01
1 primi	2	H						
145	1	08/28/2020	55	989	1	9.8	0	34.34
1 primi	2	H						
145	1	08/29/2020	56	990	1	10	0.03	36.55

1 primi 2		H						
145	1	08/30/2020	57	991	1	9.9	0.11	34.58
1 primi 2		H						
145	1	08/31/2020	58	992	1	9.9	0.02	35.13
1 primi 2		H						
145	1	09/01/2020	59	993	1	9.9	0	36.17
1 primi 2		H						
145	1	09/02/2020	60	994	1	10	0.26	35.23
1 primi 2		H						
145	1	09/03/2020	61	995	1	9.8	0	33.59
1 primi 2		H						
145	1	09/04/2020	62	996	1	10	0.02	37.92
1 primi 2		H						
145	1	09/05/2020	63	997	1	9.8	0	35.18
1 primi 2		H						
145	1	09/06/2020	64	998	1	9.8	0.02	34.18
1 primi 2		H						
145	1	09/07/2020	65	999	1	9.6	0	34.29
1 primi 2		H						
145	1	09/08/2020	66	1000	1	9.9	0	35.98
1 primi 2		H						
145	1	09/09/2020	67	1001	1	9.5	0.01	33.79
1 primi 2		H						
145	1	09/10/2020	68	1002	1	9.8	0	30.77
1 primi 2		H						
145	1	09/11/2020	69	1003	1	10	0.03	38.92
1 primi 2		H						
145	1	09/12/2020	70	1004	1	10	0	36.04
1 primi 2		H						
145	1	09/13/2020	71	1005	1	10	0	34.56
1 primi 2		H						
145	1	09/14/2020	72	1006	1	9.9	0.01	33.66
1 primi 2		H						
145	1	09/15/2020	73	1007	1	9.8	0	37.37
1 primi 2		H						
145	1	09/16/2020	74	1008	1	10	0.05	38.03
1 primi 2		H						
145	1	09/17/2020	75	1009	1	9.9	0	36.17
1 primi 2		H						
146	1	07/25/2018	5	781	1	10.7	0	21.09
1 primi 1		H						
146	1	07/26/2018	6	782	1	10.6	0.22	19.49
1 primi 1		H						
146	1	07/27/2018	7	783	1	11.4	0.04	20.63
1 primi 1		H						
146	1	07/28/2018	8	784	1	10.7	0	22.9
1 primi 1		H						
146	1	07/29/2018	9	785	1	10.6	0	20.81
1 primi 1		H						
146	1	07/30/2018	10	786	1	10.2	0.15	21.32
1 primi 1		H						
146	1	07/31/2018	11	787	1	10.7	0	24.1
1 primi 1		H						
146	1	08/01/2018	12	788	1	10	0	22.2

1 primi	1	H						
146	1	08/02/2018	13	789	1	10.2	0.23	24.34
1 primi	1	H						
146	1	08/03/2018	14	790	1	10.2	0.1	25.35
1 primi	1	H						
146	1	08/04/2018	15	791	1	9.9	0.13	26.23
1 primi	1	H						
146	1	08/05/2018	16	792	1	9.8	0	24.15
1 primi	1	H						
146	1	08/06/2018	17	793	1	9.8	0.24	26.42
1 primi	1	H						
146	1	08/07/2018	18	794	1	9.8	0	27.05
1 primi	1	H						
146	1	08/08/2018	19	795	1	10	0	28.22
1 primi	1	H						
146	1	08/09/2018	20	796	1	9.8	0	29.27
1 primi	1	H						
146	1	08/10/2018	21	797	1	9.6	0.2	29.61
1 primi	1	H						
146	1	08/11/2018	22	798	1	9.9	0.19	21.69
1 primi	2	H						
146	1	08/12/2018	23	799	1	10.2	0	29.75
1 primi	2	H						
146	1	08/13/2018	24	800	1	10	0	26.84
1 primi	2	H						
146	1	08/14/2018	25	801	1	9.5	0.24	29.87
1 primi	2	H						
146	1	08/15/2018	26	802	1	9.5	0	29.07
1 primi	2	H						
146	1	08/16/2018	27	803	1	9.3	0	29.68
1 primi	2	H						
146	1	08/17/2018	28	804	1	9.6	0	27.16
1 primi	2	H						
146	1	08/18/2018	29	805	1	9.6	0.16	29.78
1 primi	2	H						
146	1	08/19/2018	30	806	1	10	0.02	28.58
1 primi	2	H						
146	1	08/20/2018	31	807	1	9.8	0.26	26.54
1 primi	2	H						
146	1	08/21/2018	32	808	1	9.5	0	26.58
1 primi	2	H						
146	1	08/22/2018	33	809	1	9.5	0	28.16
1 primi	2	H						
146	1	08/23/2018	34	810	1	9.6	0.05	28.69
1 primi	2	H						
146	1	08/24/2018	35	811	1	9.6	0	28.02
1 primi	2	H						
146	1	08/25/2018	36	812	1	9.8	0.02	29.88
1 primi	2	H						
146	1	08/26/2018	37	813	1	10	0.01	29.55
1 primi	2	H						
146	1	08/27/2018	38	814	1	9.8	0	30.25
1 primi	2	H						
146	1	08/28/2018	39	815	1	9.9	0	32.49

1 primi	2	H						
146	1	08/29/2018	40	816	1	10	0	32.35
1 primi	2	H						
146	1	08/30/2018	41	817	1	9.9	0.18	33.7
1 primi	2	H						
146	1	08/31/2018	42	818	1	10	0	33.46
1 primi	2	H						
146	1	09/01/2018	43	819	1	9.6	0.32	31.22
1 primi	2	H						
146	1	09/02/2018	44	820	1	9.3	0	33.88
1 primi	2	H						
146	1	06/17/2019	6	1108	2	10.6	0.25	27.09
2 multi	1	H						
146	1	06/18/2019	7	1109	2	10.4	0	32.96
2 multi	1	H						
146	1	06/19/2019	8	1110	2	10.7	0	32.71
2 multi	1	H						
146	1	06/20/2019	9	1111	2	10.7	0	25.55
2 multi	1	H						
146	1	06/21/2019	10	1112	2	10.6	0.38	25.85
2 multi	1	H						
146	1	06/22/2019	11	1113	2	10.2	0	31.59
2 multi	1	H						
146	1	06/23/2019	12	1114	2	9.5	0	29.7
2 multi	1	H						
146	1	06/24/2019	13	1115	2	10.2	0	31.07
2 multi	1	H						
146	1	06/25/2019	14	1116	2	9.9	0	34.15
2 multi	1	H						
146	1	06/26/2019	15	1117	2	10.2	0	30.03
2 multi	1	H						
146	1	06/27/2019	16	1118	2	10.3	0	35.33
2 multi	1	H						
146	1	06/28/2019	17	1119	2	10	0	32.58
2 multi	1	H						
146	1	06/29/2019	18	1120	2	10	0	33.82
2 multi	1	H						
146	1	06/30/2019	19	1121	2	10	0	35.32
2 multi	1	H						
146	1	07/01/2019	20	1122	2	10.3	0	37.02
2 multi	1	H						
146	1	07/02/2019	21	1123	2	10.2	0	39.7
2 multi	1	H						
146	1	07/03/2019	22	1124	2	10.2	0.09	37.9
2 multi	2	H						
146	1	07/04/2019	23	1125	2	9.9	0	37.76
2 multi	2	H						
146	1	07/05/2019	24	1126	2	10	0	37.2
2 multi	2	H						
146	1	07/06/2019	25	1127	2	10	0	38.3
2 multi	2	H						
146	1	07/07/2019	26	1128	2	10	0	38.38
2 multi	2	H						
146	1	07/08/2019	27	1129	2	10.2	0.01	39.92

2 multi 2 H
146 1 07/09/2019 28 1130 2 9.9 0 39.09
2 multi 2 H
146 1 07/10/2019 29 1131 2 9.9 0 37.97
2 multi 2 H
146 1 07/11/2019 30 1132 2 9.8 0 38.33
2 multi 2 H
146 1 07/12/2019 31 1133 2 10 0 39.47
2 multi 2 H
146 1 07/13/2019 32 1134 2 9.9 0.02 40.1
2 multi 2 H
146 1 07/14/2019 33 1135 2 10.2 0.01 40.44
2 multi 2 H
146 1 07/15/2019 34 1136 2 9.9 0.01 42.4
2 multi 2 H
146 1 07/16/2019 35 1137 2 10 0.05 40.51
2 multi 2 H
146 1 07/17/2019 36 1138 2 10 0.19 43.37
2 multi 2 H
146 1 07/18/2019 37 1139 2 10 0.21 41.99
2 multi 2 H
146 1 07/19/2019 38 1140 2 10.2 0 40.76
2 multi 2 H
146 1 07/20/2019 39 1141 2 9.9 0 44.79
2 multi 2 H
146 1 07/21/2019 40 1142 2 10 0 43.84
2 multi 2 H
146 1 07/22/2019 41 1143 2 10.2 0 47.01
2 multi 2 H
146 1 07/23/2019 42 1144 2 10 0 45.17
2 multi 2 H
146 1 07/24/2019 43 1145 2 10 0 41.61
2 multi 2 H
146 1 07/25/2019 44 1146 2 10.2 0 41.87
2 multi 2 H
146 1 07/26/2019 45 1147 2 9.9 0 44.88
2 multi 2 H
146 1 07/27/2019 46 1148 2 9.8 0.17 44.05
2 multi 2 H
146 1 07/28/2019 47 1149 2 10 0 41.08
2 multi 2 H
146 1 07/29/2019 48 1150 2 10.2 0.01 43.02
2 multi 2 H
146 1 07/30/2019 49 1151 2 10 0 44.69
2 multi 2 H
146 1 07/31/2019 50 1152 2 10 0 45.5
2 multi 2 H
146 1 08/01/2019 51 1153 2 9.9 0.06 43.26
2 multi 2 H
146 1 08/02/2019 52 1154 2 10.4 0 43.55
2 multi 2 H
146 1 08/03/2019 53 1155 2 10 0.06 44.21
2 multi 2 H
146 1 08/04/2019 54 1156 2 10.2 0 43.31

2 multi	2	H						
146	1	08/05/2019	55	1157	2	10.3	0	45.36
2 multi	2	H						
146	1	08/06/2019	56	1158	2	10.2	0	42.59
2 multi	2	H						
146	1	08/07/2019	57	1159	2	10	0.05	43.72
2 multi	2	H						
146	1	08/08/2019	58	1160	2	10.2	0	43
2 multi	2	H						
146	1	08/09/2019	59	1161	2	10.3	0	42.11
2 multi	2	H						
146	1	08/10/2019	60	1162	2	10.2	0	43.14
2 multi	2	H						
146	1	08/11/2019	61	1163	2	10	0.31	41.88
2 multi	2	H						
146	1	08/12/2019	62	1164	2	10.2	0	43.14
2 multi	2	H						
146	1	08/13/2019	63	1165	2	10.2	0	42.8
2 multi	2	H						
146	1	08/14/2019	64	1166	2	10.4	0	43.8
2 multi	2	H						
146	1	08/15/2019	65	1167	2	10.2	0	42.66
2 multi	2	H						
146	1	08/16/2019	66	1168	2	10.2	0	41.01
2 multi	2	H						
146	1	08/17/2019	67	1169	2	9.9	0	39.81
2 multi	2	H						
146	1	08/18/2019	68	1170	2	10.2	0.02	45.64
2 multi	2	H						
146	1	08/19/2019	69	1171	2	10.3	0	43.93
2 multi	2	H						
146	1	08/20/2019	70	1172	2	10	0	40.79
2 multi	2	H						
146	1	08/21/2019	71	1173	2	9.8	0.03	40.73
2 multi	2	H						
146	1	08/22/2019	72	1174	2	9.9	0	38.49
2 multi	2	H						
146	1	08/23/2019	73	1175	2	10.2	0	41.32
2 multi	2	H						
146	1	08/24/2019	74	1176	2	10	0	41.38
2 multi	2	H						
146	1	08/25/2019	75	1177	2	9.9	0.02	40.26
2 multi	2	H						
146	1	08/26/2019	76	1178	2	10	0	41.81
2 multi	2	H						
146	1	08/27/2019	77	1179	2	10	0	39.94
2 multi	2	H						
146	1	08/28/2019	78	1180	2	10	0	40.07
2 multi	2	H						
146	1	08/29/2019	79	1181	2	10.2	0	39.17
2 multi	2	H						
146	1	08/30/2019	80	1182	2	10	0.05	38.49
2 multi	2	H						
146	1	08/31/2019	81	1183	2	10	0	38.3

2 multi 2		H						
146	1	09/01/2019	82	1184	2	10	0.03	37.26
2 multi 2		H						
146	1	09/02/2019	83	1185	2	9.9	0	37.86
2 multi 2		H						
146	1	09/03/2019	84	1186	2	9.9	0	36.78
2 multi 2		H						
146	1	09/04/2019	85	1187	2	10	0	37.48
2 multi 2		H						
146	1	09/05/2019	86	1188	2	10	0.1	38.75
2 multi 2		H						
146	1	09/06/2019	87	1189	2	9.6	0	34.39
2 multi 2		H						
146	1	09/07/2019	88	1190	2	9.9	0	40.23
2 multi 2		H						
146	1	09/08/2019	89	1191	2	10	0.17	35.65
2 multi 2		H						
146	1	09/09/2019	90	1192	2	10	0.36	28.28
2 multi 2		H						
146	1	09/10/2019	91	1193	2	10	0.3	31
2 multi 2		H						
146	1	09/11/2019	92	1194	2	9.9	0.09	33.24
2 multi 2		H						
146	1	09/12/2019	93	1195	2	9.9	0	33.66
2 multi 2		H						
146	1	08/16/2020	5	1534	3	10.9	0.06	38.65
2 multi 1		H						
146	1	08/17/2020	6	1535	3	10.7	0.03	38.23
2 multi 1		H						
146	1	08/18/2020	7	1536	3	10.6	0.02	37.87
2 multi 1		H						
146	1	08/19/2020	8	1537	3	10.4	0.6	38.4
2 multi 1		H						
146	1	08/20/2020	9	1538	3	10.4	0	43.8
2 multi 1		H						
146	1	08/21/2020	10	1539	3	10	0	45.86
2 multi 1		H						
146	1	08/22/2020	11	1540	3	10.3	0.07	45.18
2 multi 1		H						
146	1	08/23/2020	12	1541	3	10.4	0.01	45.43
2 multi 1		H						
146	1	08/24/2020	13	1542	3	10.2	0.03	45.83
2 multi 1		H						
146	1	08/25/2020	14	1543	3	10.3	0	42.08
2 multi 1		H						
146	1	08/26/2020	15	1544	3	10.4	0	43.08
2 multi 1		H						
146	1	08/27/2020	16	1545	3	10.3	0	48.33
2 multi 1		H						
146	1	08/28/2020	17	1546	3	10.6	0.02	47.7
2 multi 1		H						
146	1	08/29/2020	18	1547	3	10	0	46.4
2 multi 1		H						
146	1	08/30/2020	19	1548	3	10.4	0.04	47.71

2 multi	1	H						
146	1	08/31/2020	20	1549	3	10.4	0.03	48.82
2 multi	1	H						
146	1	09/01/2020	21	1550	3	10.3	0.03	47.89
2 multi	1	H						
146	1	09/02/2020	22	1551	3	10.4	0.03	49.89
2 multi	2	H						
146	1	09/03/2020	23	1552	3	10.6	0.01	47.67
2 multi	2	H						
146	1	09/04/2020	24	1553	3	10.7	0.01	52.37
2 multi	2	H						
146	1	09/05/2020	25	1554	3	10.4	0	48.3
2 multi	2	H						
146	1	09/06/2020	26	1555	3	10.3	0.15	49
2 multi	2	H						
146	1	09/07/2020	27	1556	3	11.1	0	50.93
2 multi	2	H						
146	1	09/08/2020	28	1557	3	10.7	0.04	51.52
2 multi	2	H						
146	1	09/09/2020	29	1558	3	10.7	0.08	46.7
2 multi	2	H						
146	1	09/10/2020	30	1559	3	10.4	0.01	46.38
2 multi	2	H						
146	1	09/11/2020	31	1560	3	10.6	0.01	52.46
2 multi	2	H						
146	1	09/12/2020	32	1561	3	10.7	0.04	49.79
2 multi	2	H						
146	1	09/13/2020	33	1562	3	10.7	0.06	51.01
2 multi	2	H						
146	1	09/14/2020	34	1563	3	10.7	0	50.48
2 multi	2	H						
146	1	09/15/2020	35	1564	3	10.6	0	50.12
2 multi	2	H						
146	1	09/16/2020	36	1565	3	10.9	0	56.58
2 multi	2	H						
146	1	09/17/2020	37	1566	3	10.7	0.06	50.32
2 multi	2	H						
146	1	09/18/2020	38	1567	3	10.4	0.07	51.2
2 multi	2	H						
146	1	09/19/2020	39	1568	3	10.6	0.05	48.63
2 multi	2	H						
146	1	09/20/2020	40	1569	3	10.4	0	49.1
2 multi	2	H						
146	1	09/21/2020	41	1570	3	10.9	0	50.18
2 multi	2	H						
146	1	09/22/2020	42	1571	3	11.1	0.02	52.15
2 multi	2	H						
146	1	09/23/2020	43	1572	3	10.7	0	53.54
2 multi	2	H						
146	1	09/24/2020	44	1573	3	10.7	0	50.53
2 multi	2	H						
146	1	09/25/2020	45	1574	3	10.6	0	57.06
2 multi	2	H						
146	1	09/26/2020	46	1575	3	10.4	0	47.97

2 multi	2	H						
146	1	09/27/2020	47	1576	3	10.6	0.32	52.62
2 multi	2	H						
146	1	09/28/2020	48	1577	3	10.7	0	50.15
2 multi	2	H						
146	1	09/29/2020	49	1578	3	10.6	0.02	47.73
2 multi	2	H						
146	1	09/30/2020	50	1579	3	10.7	0.02	50.7
2 multi	2	H						
146	1	10/01/2020	51	1580	3	10.9	0	56.98
2 multi	2	H						
146	1	10/02/2020	52	1581	3	10.7	0.06	49.59
2 multi	2	H						
146	1	10/03/2020	53	1582	3	10.6	0	54.4
2 multi	2	H						
146	1	10/04/2020	54	1583	3	10.9	0.02	53.05
2 multi	2	H						
146	1	10/05/2020	55	1584	3	10.4	0	45.45
2 multi	2	H						
146	1	10/06/2020	56	1585	3	10.3	0.05	53.04
2 multi	2	H						
146	1	10/07/2020	57	1586	3	10.3	0.03	50.8
2 multi	2	H						
146	1	10/08/2020	58	1587	3	10.4	0.02	47.31
2 multi	2	H						
146	1	10/09/2020	59	1588	3	10.7	0.04	50.06
2 multi	2	H						
146	1	10/10/2020	60	1589	3	10.4	0.03	48.02
2 multi	2	H						
146	1	10/11/2020	61	1590	3	10.7	0.04	52.52
2 multi	2	H						
146	1	10/12/2020	62	1591	3	10.4	0	51.42
2 multi	2	H						
146	1	10/13/2020	63	1592	3	10.4	0	50.25
2 multi	2	H						
146	1	10/14/2020	64	1593	3	10.4	0.05	50.36
2 multi	2	H						
146	1	10/15/2020	65	1594	3	10.6	0.07	54.38
2 multi	2	H						
146	1	10/16/2020	66	1595	3	10.6	0.05	50.95
2 multi	2	H						
146	1	10/17/2020	67	1596	3	10.3	0.03	49.13
2 multi	2	H						
146	1	10/18/2020	68	1597	3	10.6	0.02	52.14
2 multi	2	H						
146	1	10/19/2020	69	1598	3	10.7	0.02	53.05
2 multi	2	H						
146	1	10/20/2020	70	1599	3	10.6	0.05	56.44
2 multi	2	H						
146	1	10/21/2020	71	1600	3	10.7	0.05	55.22
2 multi	2	H						
146	1	10/22/2020	72	1601	3	10.9	0	49.29
2 multi	2	H						
146	1	10/23/2020	73	1602	3	10.6	0.03	59.45

2 multi	2	H						
146	1	10/24/2020	74	1603	3	10.3	0	49.98
2 multi	2	H						
146	1	10/25/2020	75	1604	3	10.2	0.06	48.8
2 multi	2	H						
146	1	10/26/2020	76	1605	3	10.4	0	52.58
2 multi	2	H						
146	1	10/27/2020	77	1606	3	10.6	0.03	49.7
2 multi	2	H						
146	1	10/28/2020	78	1607	3	10.4	0.05	50.1
2 multi	2	H						
146	1	10/29/2020	79	1608	3	10.4	0	49.95
2 multi	2	H						
146	1	10/30/2020	80	1609	3	10.9	0.09	49.95
2 multi	2	H						
146	1	10/31/2020	81	1610	3	10.7	0	56.11
2 multi	2	H						
146	1	11/01/2020	82	1611	3	10.9	0.01	53.92
2 multi	2	H						
146	1	11/02/2020	83	1612	3	10.6	0	53.24
2 multi	2	H						
146	1	11/03/2020	84	1613	3	10.6	0.06	52.16
2 multi	2	H						
146	1	11/04/2020	85	1614	3	10.7	0	55.71
2 multi	2	H						
146	1	11/05/2020	86	1615	3	10.4	0.06	51.79
2 multi	2	H						
146	1	11/06/2020	87	1616	3	10.3	0	54.76
2 multi	2	H						
146	1	11/07/2020	88	1617	3	10.3	0.06	51.14
2 multi	2	H						
146	1	11/08/2020	89	1618	3	10.4	0	51.74
2 multi	2	H						
146	1	11/09/2020	90	1619	3	10.4	0.1	47.25
2 multi	2	H						
146	1	11/10/2020	91	1620	3	10.9	0.03	47.81
2 multi	2	H						
146	1	11/11/2020	92	1621	3	10.4	0.05	48.94
2 multi	2	H						
146	1	11/12/2020	93	1622	3	10.7	0.08	50.07
2 multi	2	H						
146	1	11/13/2020	94	1623	3	10.6	0	49.5
2 multi	2	H						
146	1	11/14/2020	95	1624	3	10.6	0.04	52.2
2 multi	2	H						
146	1	11/15/2020	96	1625	3	10.7	0.03	51.43
2 multi	2	H						
146	1	11/16/2020	97	1626	3	10.3	0	50.63
2 multi	2	H						
146	1	11/17/2020	98	1627	3	10.4	0.05	52.85
2 multi	2	H						
146	1	11/18/2020	99	1628	3	10.6	0.2	54.81
2 multi	2	H						
146	1	11/19/2020	100	1629	3	10.9	0.05	48.66

2 multi 2		H						
146	1	11/20/2020	101	1630	3	10.7	0.06	53.67
2 multi 2		H						
146	1	11/21/2020	102	1631	3	10.7	0.05	51.53
2 multi 2		H						
146	1	11/22/2020	103	1632	3	10.2	0.03	50.48
2 multi 2		H						
146	1	11/23/2020	104	1633	3	10.3	0.05	54.09
2 multi 2		H						
146	1	11/24/2020	105	1634	3	10.4	0	51.34
2 multi 2		H						
146	1	11/25/2020	106	1635	3	10.4	0.13	49.91
2 multi 2		H						
146	1	11/26/2020	107	1636	3	10.3	0	50.27
2 multi 2		H						
146	1	11/27/2020	108	1637	3	10.3	0.05	49.03
2 multi 2		H						
146	1	11/28/2020	109	1638	3	10.6	0.1	48.6
2 multi 2		H						
146	1	11/29/2020	110	1639	3	10.4	0.43	51.51
2 multi 2		H						
146	1	11/30/2020	111	1640	3	10.3	0.04	49.48
2 multi 2		H						
146	1	12/01/2020	112	1641	3	10.4	0.03	50.95
2 multi 2		H						
146	1	12/02/2020	113	1642	3	10.3	0.02	50.69
2 multi 2		H						
146	1	12/03/2020	114	1643	3	10.4	0.06	52.43
2 multi 2		H						
146	1	12/04/2020	115	1644	3	10.4	0.07	51.37
2 multi 2		H						
146	1	12/05/2020	116	1645	3	10.4	0.04	51.06
2 multi 2		H						
146	1	12/06/2020	117	1646	3	10.4	0	52.56
2 multi 2		H						
146	1	12/07/2020	118	1647	3	10.4	0.09	50.29
2 multi 2		H						
146	1	12/08/2020	119	1648	3	10.4	0.03	48.57
2 multi 2		H						
146	1	12/09/2020	120	1649	3	10.7	0.08	45.6
2 multi 2		H						
146	1	12/10/2020	121	1650	3	10.7	0.03	47.56
2 multi 2		H						
146	1	12/11/2020	122	1651	3	10.7	0.04	49.89
2 multi 2		H						
146	1	12/12/2020	123	1652	3	10.7	0.03	47.55
2 multi 2		H						
146	1	12/13/2020	124	1653	3	10.9	0.03	49.56
2 multi 2		H						
146	1	12/14/2020	125	1654	3	10.7	0.04	48.97
2 multi 2		H						
146	1	12/15/2020	126	1655	3	10.6	0	45.57
2 multi 2		H						
146	1	12/16/2020	127	1656	3	10.6	0	47.84

2 multi	2	H						
146	1	12/17/2020	128	1657	3	10.7	0.03	48.17
2 multi	2	H						
146	1	12/18/2020	129	1658	3	10.9	0.1	47.57
2 multi	2	H						
146	1	12/19/2020	130	1659	3	10.9	0	48.6
2 multi	2	H						
146	1	12/20/2020	131	1660	3	10.7	0.03	49.59
2 multi	2	H						
146	1	12/21/2020	132	1661	3	10.9	0.03	46.36
2 multi	2	H						
146	1	12/22/2020	133	1662	3	10.6	0.04	46.07
2 multi	2	H						
146	1	12/23/2020	134	1663	3	11.1	0.04	47.19
2 multi	2	H						
146	1	12/24/2020	135	1664	3	11.1	0.05	47.25
2 multi	2	H						
146	1	12/25/2020	136	1665	3	10.6	0	44.65
2 multi	2	H						
146	1	12/26/2020	137	1666	3	10.6	0.04	46.69
2 multi	2	H						
146	1	12/27/2020	138	1667	3	10.7	0	49.16
2 multi	2	H						
146	1	12/28/2020	139	1668	3	10.7	0	47.86
2 multi	2	H						
146	1	12/29/2020	140	1669	3	10.7	0	49.09
2 multi	2	H						
146	1	12/30/2020	141	1670	3	10.9	0.03	45.98
2 multi	2	H						
146	1	12/31/2020	142	1671	3	10.7	0.05	47.3
2 multi	2	H						
146	1	01/01/2021	143	1672	3	10.6	0	43.04
2 multi	2	H						
146	1	01/02/2021	144	1673	3	11.1	0.04	39.59
2 multi	2	H						
146	1	01/03/2021	145	1674	3	10.4	0.06	44.96
2 multi	2	H						
146	1	01/04/2021	146	1675	3	10.6	0	46.29
2 multi	2	H						
146	1	01/05/2021	147	1676	3	10.4	0.16	45.96
2 multi	2	H						
146	1	01/06/2021	148	1677	3	10.6	0.03	44.39
2 multi	2	H						
146	1	01/07/2021	149	1678	3	10.4	0	41.59
2 multi	2	H						
146	1	01/08/2021	150	1679	3	10.6	0.04	45.96
2 multi	2	H						
146	1	01/09/2021	151	1680	3	10.6	0	45.4
2 multi	2	H						
146	1	01/10/2021	152	1681	3	10.4	0.04	45.4
2 multi	2	H						
146	1	01/11/2021	153	1682	3	10.7	0.41	45.51
2 multi	2	H						
146	1	01/12/2021	154	1683	3	10.6	0	40.27

2 multi 2 H
146 1 01/13/2021 155 1684 3 10.6 0.07 47.18
2 multi 2 H
146 1 01/14/2021 156 1685 3 10.6 0.04 41.85
2 multi 2 H
146 1 01/15/2021 157 1686 3 10.6 0.03 45.61
2 multi 2 H
146 1 01/16/2021 158 1687 3 10.7 0.05 44.79
2 multi 2 H
146 1 01/17/2021 159 1688 3 10.9 0.04 41.67
2 multi 2 H
146 1 01/18/2021 160 1689 3 11.1 0.05 44.82
2 multi 2 H
146 1 01/19/2021 161 1690 3 10.9 0 45.08
2 multi 2 H
146 1 01/20/2021 162 1691 3 10.9 0.05 43.52
2 multi 2 H
146 1 01/21/2021 163 1692 3 10.9 0.04 43.62
2 multi 2 H
146 1 01/22/2021 164 1693 3 11.3 0.03 42.07
2 multi 2 H
146 1 01/23/2021 165 1694 3 11.3 0.06 42.31
2 multi 2 H
146 1 01/24/2021 166 1695 3 11.3 0.05 42.25
2 multi 2 H
146 1 01/25/2021 167 1696 3 11.1 0.04 43.68
2 multi 2 H
146 1 01/26/2021 168 1697 3 11.3 0 45.07
2 multi 2 H
146 1 01/27/2021 169 1698 3 10.9 0.03 42.74
2 multi 2 H
146 1 01/28/2021 170 1699 3 10.6 0 39.93
2 multi 2 H
146 1 01/29/2021 171 1700 3 10.9 0.2 45.64
2 multi 2 H
146 1 01/30/2021 172 1701 3 10.9 0 40.43
2 multi 2 H
146 1 01/31/2021 173 1702 3 10.7 0 43
2 multi 2 H
146 1 02/01/2021 174 1703 3 10.7 0.06 44.32
2 multi 2 H
146 1 02/02/2021 175 1704 3 10.9 0 41.18
2 multi 2 H
146 1 02/03/2021 176 1705 3 11.1 0.04 42.13
2 multi 2 H
146 1 02/04/2021 177 1706 3 10.9 0.05 44.13
2 multi 2 H
146 1 02/05/2021 178 1707 3 10.6 0.18 43.91
2 multi 2 H
146 1 02/06/2021 179 1708 3 10.9 0 41.59
2 multi 2 H
146 1 02/07/2021 180 1709 3 10.9 0.04 44.64
2 multi 2 H
146 1 02/08/2021 181 1710 3 10.9 0.03 41.83

2 multi 2		H						
146	1	02/09/2021	182	1711	3	10.7	0	45.55
2 multi 2		H						
146	1	02/10/2021	183	1712	3	10.7	0	41.44
2 multi 2		H						
146	1	02/11/2021	184	1713	3	10.7	0.05	43.35
2 multi 2		H						
146	1	02/12/2021	185	1714	3	10.7	0.05	41.81
2 multi 2		H						
146	1	02/13/2021	186	1715	3	10.9	0.03	42.37
2 multi 2		H						
146	1	02/14/2021	187	1716	3	10.9	0.03	40.63
2 multi 2		H						
146	1	02/15/2021	188	1717	3	11.1	0	42.73
2 multi 2		H						
146	1	02/16/2021	189	1718	3	10.9	0.05	43.2
2 multi 2		H						
146	1	02/17/2021	190	1719	3	11.4	0.04	40.24
2 multi 2		H						
146	1	02/18/2021	191	1720	3	11.3	0.04	42.93
2 multi 2		H						
146	1	02/19/2021	192	1721	3	11.3	0.05	41.77
2 multi 2		H						
146	1	02/20/2021	193	1722	3	11.3	0.05	41.05
2 multi 2		H						
146	1	02/21/2021	194	1723	3	11.4	0.05	41.13
2 multi 2		H						
146	1	02/22/2021	195	1724	3	11.4	0	40.27
2 multi 2		H						
146	1	02/23/2021	196	1725	3	11.4	0	38.69
2 multi 2		H						
146	1	02/24/2021	197	1726	3	11.3	0.05	42.8
2 multi 2		H						
146	1	02/25/2021	198	1727	3	11.3	0.03	39.73
2 multi 2		H						
146	1	02/26/2021	199	1728	3	11.1	0.05	44.42
2 multi 2		H						
146	1	02/27/2021	200	1729	3	11.1	0.05	40.83
2 multi 2		H						
146	1	02/28/2021	201	1730	3	11.1	0	37.85
2 multi 2		H						
146	1	03/01/2021	202	1731	3	11.1	0.05	41.58
2 multi 2		H						
146	1	03/02/2021	203	1732	3	11.4	0.05	41.6
2 multi 2		H						
146	1	03/03/2021	204	1733	3	11.3	0.05	41.37
2 multi 2		H						
146	1	03/04/2021	205	1734	3	11.7	0.06	41.1
2 multi 2		H						
146	1	03/05/2021	206	1735	3	11.3	0.07	40.86
2 multi 2		H						
146	1	03/06/2021	207	1736	3	11.9	0	41.23
2 multi 2		H						
146	1	03/07/2021	208	1737	3	11.3	0.09	40.88

2 multi	2	H						
146	1	03/08/2021	209	1738	3	11.3	0	43.63
2 multi	2	H						
146	1	03/09/2021	210	1739	3	11.1	0.04	39.52
2 multi	2	H						
146	1	03/10/2021	211	1740	3	11.4	0.04	40.9
2 multi	2	H						
146	1	03/11/2021	212	1741	3	11.1	0.07	39.92
2 multi	2	H						
146	1	03/12/2021	213	1742	3	11.1	0	40.11
2 multi	2	H						
148	1	11/02/2020	6	758	1	10	0.17	19.62
1 primi	1	C						
148	1	11/03/2020	7	759	1	11.1	0.13	24.34
1 primi	1	C						
148	1	11/04/2020	8	760	1	10.6	0.04	21.75
1 primi	1	C						
148	1	11/05/2020	9	761	1	10.4	0.07	23.2
1 primi	1	C						
148	1	11/06/2020	10	762	1	10.2	0.1	24.46
1 primi	1	C						
148	1	11/07/2020	11	763	1	9.9	0.09	25.66
1 primi	1	C						
148	1	11/08/2020	12	764	1	10	0.15	25.95
1 primi	1	C						
148	1	11/09/2020	13	765	1	10.3	0.04	27.43
1 primi	1	C						
148	1	11/10/2020	14	766	1	10.6	0.12	27.25
1 primi	1	C						
148	1	11/11/2020	15	767	1	11.4	0.09	27.24
1 primi	1	C						
148	1	11/12/2020	16	768	1	10.6	0.12	29.06
1 primi	1	C						
148	1	11/13/2020	17	769	1	10.4	0.12	28.72
1 primi	1	C						
148	1	11/14/2020	18	770	1	10.4	0.12	28.82
1 primi	1	C						
148	1	11/15/2020	19	771	1	10	0.09	28.73
1 primi	1	C						
148	1	11/16/2020	20	772	1	10.4	0.1	30.88
1 primi	1	C						
148	1	11/17/2020	21	773	1	10.2	0.11	31.37
1 primi	1	C						
148	1	11/18/2020	22	774	1	10.2	0.11	30.57
1 primi	2	C						
148	1	11/19/2020	23	775	1	10	0.07	31.32
1 primi	2	C						
148	1	11/20/2020	24	776	1	9.8	0.09	31.26
1 primi	2	C						
148	1	11/21/2020	25	777	1	9.9	0.1	31.38
1 primi	2	C						
148	1	11/22/2020	26	778	1	9.8	0.18	31.98
1 primi	2	C						
148	1	11/23/2020	27	779	1	9.5	0.18	31.73

1 primi 2		C						
148	1	11/24/2020	28	780	1	10	0.1	32.54
1 primi 2		C						
149	1	08/22/2020	4	742	1	10.9	0.06	21.5
1 primi 1		H						
149	1	08/23/2020	5	743	1	10.3	0	24.2
1 primi 1		H						
149	1	08/24/2020	6	744	1	10.3	0	22.35
1 primi 1		H						
149	1	08/25/2020	7	745	1	10.2	0	25.55
1 primi 1		H						
149	1	08/26/2020	8	746	1	10.2	0.09	26.58
1 primi 1		H						
149	1	08/27/2020	9	747	1	10.3	0.06	26.41
1 primi 1		H						
149	1	08/28/2020	10	748	1	10	0.07	26.44
1 primi 1		H						
149	1	08/29/2020	11	749	1	9.8	0.06	28.43
1 primi 1		H						
149	1	08/30/2020	12	750	1	10.2	0.01	27.96
1 primi 1		H						
149	1	08/31/2020	13	751	1	9.9	0.06	28.54
1 primi 1		H						
149	1	09/01/2020	14	752	1	10	0	26.7
1 primi 1		H						
149	1	09/02/2020	15	753	1	9.9	0.02	29.41
1 primi 1		H						
149	1	09/03/2020	16	754	1	9.8	0.02	28.01
1 primi 1		H						
149	1	09/04/2020	17	755	1	9.6	0.02	30.69
1 primi 1		H						
149	1	09/05/2020	18	756	1	9.8	0	30.28
1 primi 1		H						
149	1	09/06/2020	19	757	1	9.5	0.04	30.12
1 primi 1		H						
149	1	09/07/2020	20	758	1	10	0	32.56
1 primi 1		H						
149	1	09/08/2020	21	759	1	9.6	0.04	30.93
1 primi 1		H						
149	1	09/09/2020	22	760	1	9.3	0	31.67
1 primi 2		H						
149	1	09/10/2020	23	761	1	9.3	0.03	32.42
1 primi 2		H						
149	1	09/11/2020	24	762	1	9.6	0.02	32.56
1 primi 2		H						
149	1	09/12/2020	25	763	1	9.8	0	35.01
1 primi 2		H						
149	1	09/13/2020	26	764	1	9.6	0.03	30.97
1 primi 2		H						
149	1	09/14/2020	27	765	1	9.3	0	30.68
1 primi 2		H						
149	1	09/15/2020	28	766	1	9.5	0	34.49
1 primi 2		H						
149	1	09/16/2020	29	767	1	9.8	0.01	33.22

1 primi 2 H
149 1 09/17/2020 30 768 1 9.5 0 32.93
1 primi 2 H
149 1 09/18/2020 31 769 1 9.3 0.08 33.42
1 primi 2 H
149 1 09/19/2020 32 770 1 9.3 0 34.43
1 primi 2 H
149 1 09/20/2020 33 771 1 9.6 0 34.57
1 primi 2 H
149 1 09/21/2020 34 772 1 9.8 0.01 33.53
1 primi 2 H
149 1 09/22/2020 35 773 1 9.3 0.02 32.49
1 primi 2 H
149 1 09/23/2020 36 774 1 9.5 0.32 32.38
1 primi 2 H
149 1 09/24/2020 37 775 1 9.6 0.03 34.37
1 primi 2 H
149 1 09/25/2020 38 776 1 9.6 0.27 33
1 primi 2 H
149 1 09/26/2020 39 777 1 9.6 0.31 35.78
1 primi 2 H
149 1 09/27/2020 40 778 1 9.3 0.3 32.86
1 primi 2 H
149 1 09/28/2020 41 779 1 9.3 0.36 35.34
1 primi 2 H
149 1 09/29/2020 42 780 1 9.2 0.21 37.44
1 primi 2 H
149 1 09/30/2020 43 781 1 9.6 0.12 36.42
1 primi 2 H
149 1 10/01/2020 44 782 1 9.5 0.11 38.17
1 primi 2 H
149 1 10/02/2020 45 783 1 9.6 0.19 36.99
1 primi 2 H
149 1 10/03/2020 46 784 1 9.6 0.31 36.2
1 primi 2 H
149 1 10/04/2020 47 785 1 9.6 0 38.3
1 primi 2 H
149 1 10/05/2020 48 786 1 9.6 0 40.5
1 primi 2 H
149 1 10/06/2020 49 787 1 9.2 0 34.7
1 primi 2 H
149 1 10/07/2020 50 788 1 9.8 0 38.64
1 primi 2 H
149 1 10/08/2020 51 789 1 9.5 0 33.42
1 primi 2 H
149 1 10/09/2020 52 790 1 9.6 0.07 39
1 primi 2 H
149 1 10/10/2020 53 791 1 9.6 0.13 36.75
1 primi 2 H
149 1 10/11/2020 54 792 1 9.6 0.12 36.81
1 primi 2 H
149 1 10/12/2020 55 793 1 9.8 0.26 32.41
1 primi 2 H
149 1 10/13/2020 56 794 1 9.9 0.03 35.88

1 primi 2		H						
150	1	07/13/2017	4	693	1	9.6	0.22	15.99
1 primi 1		H						
150	1	07/14/2017	5	694	1	9.2	0.09	17.43
1 primi 1		H						
150	1	07/15/2017	6	695	1	9.5	0	19.37
1 primi 1		H						
150	1	07/16/2017	7	696	1	9.6	0.06	20.75
1 primi 1		H						
150	1	07/17/2017	8	697	1	9.5	0	21.65
1 primi 1		H						
150	1	07/18/2017	9	698	1	9.1	0.09	23.47
1 primi 1		H						
150	1	07/19/2017	10	699	1	8.9	0.02	22.82
1 primi 1		H						
150	1	07/20/2017	11	700	1	9.1	0.07	24.52
1 primi 1		H						
150	1	07/21/2017	12	701	1	9.3	0.35	25.29
1 primi 1		H						
150	1	07/22/2017	13	702	1	9.6	0.01	25.1
1 primi 1		H						
150	1	07/23/2017	14	703	1	8.8	0.12	24.61
1 primi 1		H						
150	1	07/24/2017	15	704	1	8.7	0.08	25.49
1 primi 1		H						
150	1	07/25/2017	16	705	1	8.7	0.59	26.5
1 primi 1		H						
150	1	07/26/2017	17	706	1	8.6	0.11	25.2
1 primi 1		H						
150	1	07/27/2017	18	707	1	8.9	0.13	27.67
1 primi 1		H						
150	1	07/28/2017	19	708	1	9.1	0.13	29.71
1 primi 1		H						
150	1	07/29/2017	20	709	1	8.8	0.1	27.73
1 primi 1		H						
150	1	07/30/2017	21	710	1	8.9	0.08	30.42
1 primi 1		H						
150	1	07/31/2017	22	711	1	9.2	0.04	31.06
1 primi 2		H						
150	1	08/01/2017	23	712	1	8.7	0	29.16
1 primi 2		H						
150	1	08/02/2017	24	713	1	8.8	0	27.87
1 primi 2		H						
150	1	08/03/2017	25	714	1	8.7	0.16	30.22
1 primi 2		H						
150	1	08/04/2017	26	715	1	8.7	0.13	28.3
1 primi 2		H						
150	1	08/05/2017	27	716	1	8.6	0.01	28.01
1 primi 2		H						
150	1	08/06/2017	28	717	1	8.6	0.1	29.55
1 primi 2		H						
150	1	08/07/2017	29	718	1	8.4	0.25	28.89
1 primi 2		H						
150	1	08/08/2017	30	719	1	8.5	0.26	29.97

1 primi	2	H						
150	1	08/09/2017	31	720	1	8.8	0.23	31.01
1 primi	2	H						
150	1	08/10/2017	32	721	1	8.9	0.15	32.36
1 primi	2	H						
150	1	08/11/2017	33	722	1	8.7	0.09	32.08
1 primi	2	H						
150	1	08/12/2017	34	723	1	8.8	0.21	31.29
1 primi	2	H						
150	1	08/13/2017	35	724	1	8.9	0.24	33.62
1 primi	2	H						
150	1	08/14/2017	36	725	1	8.5	0.11	31.52
1 primi	2	H						
150	1	08/15/2017	37	726	1	8.5	0.16	33.62
1 primi	2	H						
150	1	08/16/2017	38	727	1	8.6	0	32.89
1 primi	2	H						
150	1	08/17/2017	39	728	1	8.6	0.12	31.55
1 primi	2	H						
150	1	08/18/2017	40	729	1	8.8	0.14	30.49
1 primi	2	H						
150	1	08/19/2017	41	730	1	8.6	0	29.21
1 primi	2	H						
150	1	08/20/2017	42	731	1	8.6	0.07	28.47
1 primi	2	H						
150	1	08/21/2017	43	732	1	9.1	0.06	32.18
1 primi	2	H						
150	1	08/22/2017	44	733	1	8.8	0.16	33.13
1 primi	2	H						
150	1	08/23/2017	45	734	1	8.4	0.11	27.93
1 primi	2	H						
150	1	08/24/2017	46	735	1	8.4	0.14	29.94
1 primi	2	H						
150	1	08/25/2017	47	736	1	8.3	0	30.26
1 primi	2	H						
150	1	08/26/2017	48	737	1	8.7	0.18	30.52
1 primi	2	H						
150	1	08/27/2017	49	738	1	8.9	0.06	30.97
1 primi	2	H						
150	1	08/28/2017	50	739	1	8.8	0.04	29.85
1 primi	2	H						
150	1	08/29/2017	51	740	1	8.5	0.05	29.84
1 primi	2	H						
150	1	08/30/2017	52	741	1	8.8	0.01	27.37
1 primi	2	H						
150	1	08/31/2017	53	742	1	8.6	0.08	24.39
1 primi	2	H						
150	1	09/01/2017	54	743	1	8.9	0	28.95
1 primi	2	H						
150	1	09/02/2017	55	744	1	8.6	0.06	27.62
1 primi	2	H						
150	1	09/03/2017	56	745	1	8.4	0	25.22
1 primi	2	H						
150	1	09/04/2017	57	746	1	8.9	0.02	29.04

1 primi 2		H							
150	1	09/05/2017	58	747	1	8.8	0.07	28.16	
1 primi 2		H							
150	1	09/06/2017	59	748	1	8.5	0.01	28.88	
1 primi 2		H							
150	1	09/07/2017	60	749	1	8.6	0.08	27.25	
1 primi 2		H							
150	1	09/08/2017	61	750	1	8.7	0.55	27.45	
1 primi 2		H							
150	1	09/09/2017	62	751	1	8.5	0.11	30.14	
1 primi 2		H							
150	1	09/10/2017	63	752	1	8.7	0.09	28.65	
1 primi 2		H							
150	1	09/11/2017	64	753	1	8.8	0.02	28.79	
1 primi 2		H							
150	1	09/12/2017	65	754	1	8.9	0	28.92	
1 primi 2		H							
150	1	09/13/2017	66	755	1	9.1	0.09	30.2	
1 primi 2		H							
150	1	09/14/2017	67	756	1	9.2	0.01	29.37	
1 primi 2		H							
150	1	09/15/2017	68	757	1	8.9	0.01	30.13	
1 primi 2		H							
150	1	09/16/2017	69	758	1	8.7	0.1	29.67	
1 primi 2		H							
150	1	09/17/2017	70	759	1	8.6	0.17	30.25	
1 primi 2		H							
150	1	09/18/2017	71	760	1	8.6	0.09	28.83	
1 primi 2		H							
150	1	09/19/2017	72	761	1	8.7	0.22	30.87	
1 primi 2		H							
150	1	09/20/2017	73	762	1	9.1	0.09	31.21	
1 primi 2		H							
150	1	09/21/2017	74	763	1	8.9	0.1	30.92	
1 primi 2		H							
150	1	09/22/2017	75	764	1	8.5	0.04	28.53	
1 primi 2		H							
150	1	09/23/2017	76	765	1	8.8	0.01	29.97	
1 primi 2		H							
150	1	09/24/2017	77	766	1	8.7	0.1	31.28	
1 primi 2		H							
150	1	09/25/2017	78	767	1	8.8	0.02	32.94	
1 primi 2		H							
150	1	09/26/2017	79	768	1	8.8	0	31.4	
1 primi 2		H							
150	1	09/27/2017	80	769	1	8.7	0.02	30.25	
1 primi 2		H							
150	1	09/28/2017	81	770	1	8.7	0.11	31.93	
1 primi 2		H							
150	1	09/29/2017	82	771	1	8.8	0.11	31.04	
1 primi 2		H							
150	1	09/30/2017	83	772	1	8.8	0.02	29.45	
1 primi 2		H							
150	1	10/01/2017	84	773	1	8.8	0	29.91	

1 primi	2	H						
150	1	10/02/2017	85	774	1	9.3	0	33.46
1 primi	2	H						
150	1	10/03/2017	86	775	1	9.3	0.09	33.53
1 primi	2	H						
150	1	10/04/2017	87	776	1	8.9	0.08	32.85
1 primi	2	H						
150	1	10/05/2017	88	777	1	8.9	0.07	33.77
1 primi	2	H						
150	1	10/06/2017	89	778	1	8.9	0.07	33.34
1 primi	2	H						
150	1	10/07/2017	90	779	1	8.9	0.01	31.49
1 primi	2	H						
150	1	10/08/2017	91	780	1	8.8	0	33.29
1 primi	2	H						
150	1	10/09/2017	92	781	1	8.9	0.07	30.1
1 primi	2	H						
150	1	10/10/2017	93	782	1	8.9	0	33.4
1 primi	2	H						
150	1	10/11/2017	94	783	1	0	0.01	32.01
1 primi	2	H						
150	1	10/12/2017	95	784	1	8.4	0.28	11.81
1 primi	2	H						
150	1	11/03/2017	117	806	1	8.8	0.2	26.45
1 primi	2	H						
150	1	11/04/2017	118	807	1	8.2	0	32.26
1 primi	2	H						
150	1	11/05/2017	119	808	1	9.2	0.06	30.7
1 primi	2	H						
150	1	11/06/2017	120	809	1	9.1	0.04	31.76
1 primi	2	H						
150	1	11/07/2017	121	810	1	8.9	0	32.24
1 primi	2	H						
150	1	11/08/2017	122	811	1	8.9	0.1	32.78
1 primi	2	H						
150	1	11/09/2017	123	812	1	8.9	0.23	33.83
1 primi	2	H						
150	1	11/10/2017	124	813	1	8.6	0	34.94
1 primi	2	H						
150	1	11/11/2017	125	814	1	8.9	0.07	32.75
1 primi	2	H						
150	1	11/12/2017	126	815	1	8.8	0.05	29.83
1 primi	2	H						
150	1	11/13/2017	127	816	1	8.4	0	31.49
1 primi	2	H						
150	1	11/14/2017	128	817	1	8.7	0.07	31.49
1 primi	2	H						
150	1	11/15/2017	129	818	1	8.5	0	32.13
1 primi	2	H						
150	1	11/16/2017	130	819	1	8.6	0	31.24
1 primi	2	H						
150	1	11/17/2017	131	820	1	8.1	0	31.78
1 primi	2	H						
150	1	11/18/2017	132	821	1	8.5	0	32.14

1 primi 2 H
150 1 11/19/2017 133 822 1 8.6 0 33.32
1 primi 2 H
150 1 11/20/2017 134 823 1 8.8 0.08 30.65
1 primi 2 H
150 1 11/21/2017 135 824 1 9.2 0.11 32.99
1 primi 2 H
150 1 11/22/2017 136 825 1 8.6 0 30.83
1 primi 2 H
150 1 11/23/2017 137 826 1 8.7 0 32.27
1 primi 2 H
150 1 11/24/2017 138 827 1 8.7 0.08 32.41
1 primi 2 H
150 1 11/25/2017 139 828 1 8.8 0.08 32.98
1 primi 2 H
150 1 11/26/2017 140 829 1 8.6 0.11 32.15
1 primi 2 H
150 1 11/27/2017 141 830 1 8.5 0.01 32.06
1 primi 2 H
150 1 11/28/2017 142 831 1 8.5 0 32.72
1 primi 2 H
150 1 11/29/2017 143 832 1 8.8 0.11 34.26
1 primi 2 H
150 1 11/30/2017 144 833 1 8.7 0.03 31.37
1 primi 2 H
150 1 12/01/2017 145 834 1 8.9 0.11 33.68
1 primi 2 H
150 1 12/02/2017 146 835 1 8.9 0.03 33.13
1 primi 2 H
150 1 12/03/2017 147 836 1 8.7 0.02 33.35
1 primi 2 H
150 1 12/04/2017 148 837 1 8.9 0.12 32.53
1 primi 2 H
150 1 12/05/2017 149 838 1 8.8 0.03 32.38
1 primi 2 H
150 1 12/06/2017 150 839 1 8.7 0.11 32.75
1 primi 2 H
150 1 12/07/2017 151 840 1 8.8 0 31.14
1 primi 2 H
150 1 09/13/2018 5 1120 2 9.2 0.23 36.98
2 multi 1 H
150 1 09/14/2018 6 1121 2 9.3 0.04 39.62
2 multi 1 H
150 1 09/15/2018 7 1122 2 9.5 0.19 37.82
2 multi 1 H
150 1 09/16/2018 8 1123 2 9.5 0.13 37.68
2 multi 1 H
150 1 09/17/2018 9 1124 2 9.5 0.22 37.59
2 multi 1 H
150 1 09/18/2018 10 1125 2 9.2 0.03 40.77
2 multi 1 H
150 1 09/19/2018 11 1126 2 9.1 0.04 41.22
2 multi 1 H
150 1 09/20/2018 12 1127 2 9.2 0.12 41.32

2 multi	1	H						
150	1	09/21/2018	13	1128	2	8.9	0.17	42.9
2 multi	1	H						
150	1	09/22/2018	14	1129	2	9.1	0.1	38.97
2 multi	1	H						
150	1	09/23/2018	15	1130	2	9.3	0.06	42.1
2 multi	1	H						
150	1	09/24/2018	16	1131	2	9.2	0.12	41.81
2 multi	1	H						
150	1	09/25/2018	17	1132	2	8.8	0.23	40.83
2 multi	1	H						
150	1	09/26/2018	18	1133	2	9.2	0.16	42.38
2 multi	1	H						
150	1	09/27/2018	19	1134	2	9.2	0.04	42.23
2 multi	1	H						
150	1	09/28/2018	20	1135	2	8.9	0.02	46.08
2 multi	1	H						
150	1	09/29/2018	21	1136	2	8.9	0.01	47.38
2 multi	1	H						
150	1	09/30/2018	22	1137	2	9.1	0.05	43.36
2 multi	2	H						
150	1	10/01/2018	23	1138	2	9.2	0.13	43.29
2 multi	2	H						
150	1	10/02/2018	24	1139	2	9.1	0.06	45.18
2 multi	2	H						
150	1	10/03/2018	25	1140	2	9.2	0.13	41.81
2 multi	2	H						
150	1	10/04/2018	26	1141	2	8.8	0.12	41.15
2 multi	2	H						
150	1	10/05/2018	27	1142	2	9.1	0	43.1
2 multi	2	H						
150	1	10/06/2018	28	1143	2	9.2	0.01	42.95
2 multi	2	H						
150	1	10/07/2018	29	1144	2	9.2	0.15	41.26
2 multi	2	H						
150	1	10/08/2018	30	1145	2	9.1	0.03	40.77
2 multi	2	H						
150	1	10/09/2018	31	1146	2	9.1	0	45.8
2 multi	2	H						
150	1	10/10/2018	32	1147	2	9.1	0.03	42.21
2 multi	2	H						
150	1	10/11/2018	33	1148	2	9.1	0.14	40.82
2 multi	2	H						
150	1	10/12/2018	34	1149	2	9.1	0.04	40.87
2 multi	2	H						
150	1	10/13/2018	35	1150	2	9.3	0	46.21
2 multi	2	H						
150	1	10/14/2018	36	1151	2	8.9	0	39.64
2 multi	2	H						
150	1	10/15/2018	37	1152	2	8.8	0.02	39.24
2 multi	2	H						
150	1	10/16/2018	38	1153	2	8.9	0.12	42.15
2 multi	2	H						
150	1	10/17/2018	39	1154	2	8.9	0	41.56

2 multi	2	H						
150	1	10/18/2018	40	1155	2	8.8	0.01	41.61
2 multi	2	H						
150	1	10/19/2018	41	1156	2	9.2	0.01	41.3
2 multi	2	H						
150	1	10/20/2018	42	1157	2	9.3	0.02	37.06
2 multi	2	H						
150	1	10/21/2018	43	1158	2	8.9	0.18	37.16
2 multi	2	H						
150	1	10/22/2018	44	1159	2	9.1	0.12	37.42
2 multi	2	H						
150	1	10/23/2018	45	1160	2	9.3	0.16	40.24
2 multi	2	H						
150	1	10/24/2018	46	1161	2	9.3	0.25	42.09
2 multi	2	H						
150	1	10/25/2018	47	1162	2	9.2	0	39.41
2 multi	2	H						
150	1	10/26/2018	48	1163	2	9.1	0	37.51
2 multi	2	H						
150	1	10/27/2018	49	1164	2	9.2	0.12	42.42
2 multi	2	H						
150	1	10/28/2018	50	1165	2	9.3	0.17	43.69
2 multi	2	H						
150	1	10/29/2018	51	1166	2	9.5	0.13	45
2 multi	2	H						
150	1	10/30/2018	52	1167	2	9.3	0	41.11
2 multi	2	H						
150	1	10/31/2018	53	1168	2	9.2	0.02	43.06
2 multi	2	H						
150	1	11/01/2018	54	1169	2	9.3	0.13	40.52
2 multi	2	H						
150	1	11/02/2018	55	1170	2	9.1	0	42.71
2 multi	2	H						
150	1	11/03/2018	56	1171	2	9.2	0.25	41.87
2 multi	2	H						
150	1	11/04/2018	57	1172	2	9.2	0.01	43.03
2 multi	2	H						
150	1	11/05/2018	58	1173	2	9.8	0.06	38.24
2 multi	2	H						
150	1	11/06/2018	59	1174	2	9.8	0.18	28.74
2 multi	2	H						
150	1	11/07/2018	60	1175	2	9.9	0.28	34.89
2 multi	2	H						
150	1	11/08/2018	61	1176	2	9.8	0.03	39.98
2 multi	2	H						
150	1	11/09/2018	62	1177	2	9.9	0.17	36.09
2 multi	2	H						
150	1	11/10/2018	63	1178	2	9.3	0.14	35.86
2 multi	2	H						
150	1	11/11/2018	64	1179	2	8.8	0.18	34.89
2 multi	2	H						
150	1	11/12/2018	65	1180	2	10	0.1	39.95
2 multi	2	H						
150	1	11/13/2018	66	1181	2	9.2	0	35.51

2 multi 2 H
150 1 11/14/2018 67 1182 2 9.3 0 40.93
2 multi 2 H
150 1 11/15/2018 68 1183 2 9.6 0.13 37.16
2 multi 2 H
150 1 11/16/2018 69 1184 2 9.2 0.01 38.29
2 multi 2 H
150 1 11/17/2018 70 1185 2 9.2 0.04 38.41
2 multi 2 H
150 1 11/18/2018 71 1186 2 9.5 0.05 41.24
2 multi 2 H
150 1 11/19/2018 72 1187 2 9.5 0.14 38.96
2 multi 2 H
150 1 11/20/2018 73 1188 2 9.2 0.01 42.65
2 multi 2 H
150 1 11/21/2018 74 1189 2 9.5 0 39.11
2 multi 2 H
150 1 11/22/2018 75 1190 2 9.5 0 39.07
2 multi 2 H
150 1 11/23/2018 76 1191 2 9.2 0.02 36.64
2 multi 2 H
150 1 11/24/2018 77 1192 2 8.9 0 39.88
2 multi 2 H
150 1 11/25/2018 78 1193 2 8.6 0.02 35.33
2 multi 2 H
150 1 11/26/2018 79 1194 2 9.3 0 39.36
2 multi 2 H
150 1 11/27/2018 80 1195 2 9.3 0.02 34.46
2 multi 2 H
150 1 11/28/2018 81 1196 2 9.1 0.14 39.66
2 multi 2 H
150 1 11/29/2018 82 1197 2 8.9 0 38.39
2 multi 2 H
150 1 10/26/2019 9 1528 3 9.3 0.05 40.99
2 multi 1 H
150 1 10/27/2019 10 1529 3 9.1 0.03 39.58
2 multi 1 H
150 1 10/28/2019 11 1530 3 8.9 0 39.02
2 multi 1 H
150 1 10/29/2019 12 1531 3 8.9 0.01 39.79
2 multi 1 H
150 1 10/30/2019 13 1532 3 8.8 0.03 41.06
2 multi 1 H
150 1 10/31/2019 14 1533 3 9.2 0.07 41.24
2 multi 1 H
150 1 11/01/2019 15 1534 3 9.1 0.08 41.51
2 multi 1 H
150 1 11/02/2019 16 1535 3 9.1 0.07 40.77
2 multi 1 H
150 1 11/03/2019 17 1536 3 9.2 0.04 22.44
2 multi 1 H
150 1 11/04/2019 18 1537 3 9.2 0.17 43.09
2 multi 1 H
150 1 11/05/2019 19 1538 3 9.2 0.18 43.38

2 multi 1		H						
150	1	11/06/2019	20	1539	3	9.2	0.06	42.83
2 multi 1		H						
150	1	11/07/2019	21	1540	3	9.1	0.06	42.87
2 multi 1		H						
150	1	11/08/2019	22	1541	3	9.1	0.01	43.82
2 multi 2		H						
150	1	11/09/2019	23	1542	3	8.8	0	41.67
2 multi 2		H						
150	1	11/10/2019	24	1543	3	9.1	0	44.66
2 multi 2		H						
150	1	11/11/2019	25	1544	3	9.1	0	42.67
2 multi 2		H						
150	1	11/12/2019	26	1545	3	9.2	0	44.81
2 multi 2		H						
150	1	11/13/2019	27	1546	3	9.1	0.03	45.53
2 multi 2		H						
150	1	11/14/2019	28	1547	3	9.1	0.05	44.36
2 multi 2		H						
150	1	11/15/2019	29	1548	3	9.1	0.07	45.97
2 multi 2		H						
150	1	11/16/2019	30	1549	3	9.1	0.16	45.34
2 multi 2		H						
150	1	11/17/2019	31	1550	3	9.1	0.07	47.53
2 multi 2		H						
150	1	11/18/2019	32	1551	3	8.8	0.16	42.23
2 multi 2		H						
150	1	11/19/2019	33	1552	3	8.8	0.13	45.82
2 multi 2		H						
150	1	11/20/2019	34	1553	3	9.5	0.07	45.99
2 multi 2		H						
150	1	11/21/2019	35	1554	3	9.3	0.05	39.22
2 multi 2		H						
150	1	11/22/2019	36	1555	3	9.5	0.38	40.76
2 multi 2		H						
150	1	11/23/2019	37	1556	3	8.9	0.03	46.9
2 multi 2		H						
150	1	11/24/2019	38	1557	3	9.1	0.16	46.38
2 multi 2		H						
150	1	11/25/2019	39	1558	3	8.9	0.17	44.1
2 multi 2		H						
150	1	11/26/2019	40	1559	3	9.2	0.17	44.94
2 multi 2		H						
150	1	11/27/2019	41	1560	3	9.1	0.16	39.7
2 multi 2		H						
150	1	11/28/2019	42	1561	3	8.8	0.03	42.29
2 multi 2		H						
150	1	11/29/2019	43	1562	3	9.1	0	40.81
2 multi 2		H						
150	1	11/30/2019	44	1563	3	9.2	0.03	41.08
2 multi 2		H						
150	1	12/01/2019	45	1564	3	9.2	0.15	42.46
2 multi 2		H						
150	1	12/02/2019	46	1565	3	8.8	0.05	41.47

2 multi 2		H						
150	1	12/03/2019	47	1566	3	9.1	0.05	42.68
2 multi 2		H						
150	1	12/04/2019	48	1567	3	9.3	0.08	45.58
2 multi 2		H						
150	1	12/05/2019	49	1568	3	9.5	0.16	42.97
2 multi 2		H						
150	1	12/06/2019	50	1569	3	9.2	0.02	43.22
2 multi 2		H						
150	1	12/07/2019	51	1570	3	9.5	0.05	39.74
2 multi 2		H						
150	1	12/08/2019	52	1571	3	9.3	0.01	34.3
2 multi 2		H						
150	1	12/09/2019	53	1572	3	9.5	0.09	33.63
2 multi 2		H						
150	1	12/10/2019	54	1573	3	9.3	0.05	28.1
2 multi 2		H						
150	1	12/11/2019	55	1574	3	9.2	0.11	27.9
2 multi 2		H						
150	1	12/12/2019	56	1575	3	8.8	0	30.21
2 multi 2		H						
150	1	12/13/2019	57	1576	3	8.9	0.12	26.44
2 multi 2		H						
150	1	12/14/2019	58	1577	3	9.2	0.04	27.48
2 multi 2		H						
150	1	12/15/2019	59	1578	3	9.3	0.12	26.73
2 multi 2		H						
150	1	12/16/2019	60	1579	3	9.5	0	31.76
2 multi 2		H						
150	1	12/17/2019	61	1580	3	9.5	0.02	30.17
2 multi 2		H						
150	1	12/18/2019	62	1581	3	9.3	0.02	32.39
2 multi 2		H						
150	1	12/19/2019	63	1582	3	9.6	0.15	32.38
2 multi 2		H						
150	1	12/20/2019	64	1583	3	9.5	0.04	31.29
2 multi 2		H						
150	1	12/21/2019	65	1584	3	9.3	0.15	31.63
2 multi 2		H						
150	1	12/22/2019	66	1585	3	9.5	0.02	35.87
2 multi 2		H						
150	1	12/23/2019	67	1586	3	9.8	0.13	32.41
2 multi 2		H						
150	1	12/24/2019	68	1587	3	9.3	0.01	32.46
2 multi 2		H						
150	1	12/25/2019	69	1588	3	9.6	0.05	36.1
2 multi 2		H						
150	1	12/26/2019	70	1589	3	9.6	0.09	38.69
2 multi 2		H						
150	1	12/27/2019	71	1590	3	9.8	0.04	38.22
2 multi 2		H						
150	1	12/28/2019	72	1591	3	9.5	0.18	31.39
2 multi 2		H						
150	1	12/29/2019	73	1592	3	9.5	0.07	35.83

2 multi	2	H						
150	1	12/30/2019	74	1593	3	9.1	0.13	34.56
2 multi	2	H						
150	1	12/31/2019	75	1594	3	8.8	0.06	34.63
2 multi	2	H						
150	1	03/01/2021	8	2020	4	8.8	0.28	38.36
2 multi	1	H						
150	1	03/02/2021	9	2021	4	9.3	0.23	47.03
2 multi	1	H						
150	1	03/03/2021	10	2022	4	8.9	0.15	43.33
2 multi	1	H						
150	1	03/04/2021	11	2023	4	9.8	0.05	40.49
2 multi	1	H						
150	1	03/05/2021	12	2024	4	9.8	0.12	44.76
2 multi	1	H						
150	1	03/06/2021	13	2025	4	9.3	0.06	44.55
2 multi	1	H						
150	1	03/07/2021	14	2026	4	9.2	0.24	45.06
2 multi	1	H						
150	1	03/08/2021	15	2027	4	9.1	0.07	44.34
2 multi	1	H						
150	1	03/09/2021	16	2028	4	9.1	0.16	47.37
2 multi	1	H						
150	1	03/10/2021	17	2029	4	9.1	0.03	45.78
2 multi	1	H						
150	1	03/11/2021	18	2030	4	9.3	0.08	45.29
2 multi	1	H						
150	1	03/12/2021	19	2031	4	9.2	0.08	49.36
2 multi	1	H						
150	1	03/13/2021	20	2032	4	9.5	0.13	51.1
2 multi	1	H						
150	1	03/14/2021	21	2033	4	9.1	0.09	50.66
2 multi	1	H						
150	1	03/15/2021	22	2034	4	8.9	0.17	49.87
2 multi	2	H						
150	1	03/16/2021	23	2035	4	8.6	0.18	49.25
2 multi	2	H						
150	1	03/17/2021	24	2036	4	8.8	0.19	48.17
2 multi	2	H						
150	1	03/18/2021	25	2037	4	8.9	0.08	49.01
2 multi	2	H						
150	1	03/19/2021	26	2038	4	8.9	0.17	49.89
2 multi	2	H						
150	1	03/20/2021	27	2039	4	9.2	0.14	49.07
2 multi	2	H						
150	1	03/21/2021	28	2040	4	9.2	0.17	50.96
2 multi	2	H						
150	1	03/22/2021	29	2041	4	9.1	0.15	50.6
2 multi	2	H						
150	1	03/23/2021	30	2042	4	9.5	0.09	52.5
2 multi	2	H						
150	1	03/24/2021	31	2043	4	9.2	0.17	49.61
2 multi	2	H						
150	1	03/25/2021	32	2044	4	9.1	0.19	53.5

2 multi	2	H						
150	1	03/26/2021	33	2045	4	9.3	0.11	49.85
2 multi	2	H						
150	1	03/27/2021	34	2046	4	9.2	0.17	51.23
2 multi	2	H						
150	1	03/28/2021	35	2047	4	9.2	0.07	49.79
2 multi	2	H						
150	1	03/29/2021	36	2048	4	9.2	0.17	50.67
2 multi	2	H						
150	1	03/30/2021	37	2049	4	9.3	0.14	52.44
2 multi	2	H						
150	1	03/31/2021	38	2050	4	9.5	0.18	51.96
2 multi	2	H						
150	1	04/01/2021	39	2051	4	9.2	0	49.11
2 multi	2	H						
150	1	04/02/2021	40	2052	4	8.9	0.17	51.14
2 multi	2	H						
150	1	04/03/2021	41	2053	4	9.6	0.08	49.01
2 multi	2	H						
150	1	04/04/2021	42	2054	4	9.2	0.07	48.41
2 multi	2	H						
150	1	04/05/2021	43	2055	4	9.3	0.02	50.02
2 multi	2	H						
150	1	04/06/2021	44	2056	4	9.1	0.01	51.51
2 multi	2	H						
150	1	04/07/2021	45	2057	4	9.1	0.28	47.32
2 multi	2	H						
150	1	04/08/2021	46	2058	4	9.1	0.3	49.27
2 multi	2	H						
150	1	04/09/2021	47	2059	4	9.3	0.17	50.85
2 multi	2	H						
150	1	04/10/2021	48	2060	4	9.5	0.05	51.5
2 multi	2	H						
150	1	04/11/2021	49	2061	4	9.3	0.21	50.14
2 multi	2	H						
150	1	04/12/2021	50	2062	4	9.5	0.07	51.64
2 multi	2	H						
150	1	04/13/2021	51	2063	4	9.2	0.02	49.67
2 multi	2	H						
150	1	04/14/2021	52	2064	4	9.5	0.04	49.34
2 multi	2	H						
150	1	04/15/2021	53	2065	4	9.1	0.25	48.6
2 multi	2	H						
150	1	04/16/2021	54	2066	4	9.1	0.27	48.72
2 multi	2	H						
150	1	04/17/2021	55	2067	4	9.6	0.03	52.14
2 multi	2	H						
150	1	04/18/2021	56	2068	4	9.3	0.4	51.74
2 multi	2	H						
150	1	04/19/2021	57	2069	4	9.5	0.15	50.48
2 multi	2	H						
150	1	04/20/2021	58	2070	4	9.6	0.03	50.05
2 multi	2	H						
150	1	04/21/2021	59	2071	4	9.2	0.38	50.52

2 multi 2		H						
150	1	04/22/2021	60	2072	4	9.6	0.25	53.02
2 multi 2		H						
150	1	04/23/2021	61	2073	4	9.6	0.11	53.8
2 multi 2		H						
150	1	04/24/2021	62	2074	4	9.9	0.03	49.83
2 multi 2		H						
150	1	04/25/2021	63	2075	4	9.5	0.15	51.55
2 multi 2		H						
150	1	04/26/2021	64	2076	4	9.6	0.04	54.17
2 multi 2		H						
150	1	04/27/2021	65	2077	4	9.6	0	48.09
2 multi 2		H						
150	1	04/28/2021	66	2078	4	9.6	0	52.79
2 multi 2		H						
150	1	04/29/2021	67	2079	4	9.6	0	55.13
2 multi 2		H						
150	1	04/30/2021	68	2080	4	9.6	0.27	53.04
2 multi 2		H						
150	1	05/01/2021	69	2081	4	9.5	0.14	49.99
2 multi 2		H						
150	1	05/02/2021	70	2082	4	9.5	0.13	49.93
2 multi 2		H						
150	1	05/03/2021	71	2083	4	9.6	0.11	53.69
2 multi 2		H						
150	1	05/04/2021	72	2084	4	9.6	0.04	50.62
2 multi 2		H						
150	1	05/05/2021	73	2085	4	9.5	0.04	50.56
2 multi 2		H						
150	1	05/06/2021	74	2086	4	9.3	0.14	47.39
2 multi 2		H						
150	1	05/07/2021	75	2087	4	9.3	0.16	53.17
2 multi 2		H						
150	1	05/08/2021	76	2088	4	9.2	0.25	46.81
2 multi 2		H						
150	1	05/09/2021	77	2089	4	9.8	0.11	53.74
2 multi 2		H						
150	1	05/10/2021	78	2090	4	9.9	0.01	50.58
2 multi 2		H						
150	1	05/11/2021	79	2091	4	9.8	0.09	54.11
2 multi 2		H						
150	1	05/12/2021	80	2092	4	9.2	0.18	47.17
2 multi 2		H						
150	1	05/13/2021	81	2093	4	9.5	0.07	50.36
2 multi 2		H						
150	1	05/14/2021	82	2094	4	9.8	0.03	52.32
2 multi 2		H						
150	1	05/15/2021	83	2095	4	9.5	0.06	50.23
2 multi 2		H						
150	1	05/16/2021	84	2096	4	9.5	0.02	49.84
2 multi 2		H						
150	1	05/17/2021	85	2097	4	9.2	0.13	46.65
2 multi 2		H						
150	1	05/18/2021	86	2098	4	9.3	0.24	49.32

2 multi	2	H						
150	1	05/19/2021	87	2099	4	9.8	0.12	49.5
2 multi	2	H						
150	1	05/20/2021	88	2100	4	9.6	0.01	50.03
2 multi	2	H						
150	1	05/21/2021	89	2101	4	9.5	0.16	49.54
2 multi	2	H						
150	1	05/22/2021	90	2102	4	9.6	0.05	50
2 multi	2	H						
150	1	05/23/2021	91	2103	4	9.3	0.11	48.12
2 multi	2	H						
150	1	05/24/2021	92	2104	4	9.6	0.1	51.04
2 multi	2	H						
150	1	05/25/2021	93	2105	4	9.8	0.26	51.25
2 multi	2	H						
150	1	05/26/2021	94	2106	4	9.5	0	23.5
2 multi	2	H						
151	1	07/02/2018	6	839	1	9.2	0.14	18.57
1 primi	1	H						
151	1	07/03/2018	7	840	1	9.6	0	20.38
1 primi	1	H						
151	1	07/04/2018	8	841	1	10.4	0	17.04
1 primi	1	H						
151	1	07/05/2018	9	842	1	11.1	0.1	22.18
1 primi	1	H						
151	1	07/06/2018	10	843	1	11.7	0	24.08
1 primi	1	H						
151	1	07/07/2018	11	844	1	10.4	0	23.65
1 primi	1	H						
151	1	07/08/2018	12	845	1	9.9	0	24.54
1 primi	1	H						
151	1	07/09/2018	13	846	1	10.2	0	25.95
1 primi	1	H						
151	1	07/10/2018	14	847	1	10.6	0	24.29
1 primi	1	H						
151	1	07/11/2018	15	848	1	10.9	0	27.95
1 primi	1	H						
151	1	07/12/2018	16	849	1	10	0	26.46
1 primi	1	H						
151	1	07/13/2018	17	850	1	11.1	0	20.05
1 primi	1	H						
151	1	07/14/2018	18	851	1	11.3	0	21.28
1 primi	1	H						
151	1	07/15/2018	19	852	1	11.1	0.33	28.95
1 primi	1	H						
151	1	07/16/2018	20	853	1	9.5	0	24.79
1 primi	1	H						
151	1	07/17/2018	21	854	1	9.3	0	24.7
1 primi	1	H						
151	1	07/18/2018	22	855	1	10.2	0.02	28.56
1 primi	2	H						
151	1	07/19/2018	23	856	1	9.9	0	25.92
1 primi	2	H						
151	1	07/20/2018	24	857	1	10.6	0.01	30.48

1 primi 2		H						
151	1	07/21/2018	25	858	1	10	0	28.09
1 primi 2		H						
151	1	07/22/2018	26	859	1	9.9	0.01	29.88
1 primi 2		H						
151	1	07/23/2018	27	860	1	9.8	0	27.03
1 primi 2		H						
151	1	07/24/2018	28	861	1	10	0	30.63
1 primi 2		H						
151	1	07/25/2018	29	862	1	10.6	0	29.94
1 primi 2		H						
151	1	07/26/2018	30	863	1	10.3	0	27.34
1 primi 2		H						
151	1	07/27/2018	31	864	1	10.3	0	26.56
1 primi 2		H						
151	1	07/28/2018	32	865	1	10.4	0	27.41
1 primi 2		H						
151	1	07/29/2018	33	866	1	11.3	0	29.22
1 primi 2		H						
151	1	07/30/2018	34	867	1	10.3	0	26.94
1 primi 2		H						
151	1	07/31/2018	35	868	1	10.4	0	26.98
1 primi 2		H						
151	1	08/01/2018	36	869	1	11.3	0	32.73
1 primi 2		H						
151	1	08/02/2018	37	870	1	10.6	0	26.52
1 primi 2		H						
151	1	08/03/2018	38	871	1	10.2	0.03	27.96
1 primi 2		H						
151	1	08/04/2018	39	872	1	10	0.24	30.18
1 primi 2		H						
151	1	08/05/2018	40	873	1	9.9	0	30.65
1 primi 2		H						
151	1	08/06/2018	41	874	1	10.6	0.02	28.48
1 primi 2		H						
151	1	08/07/2018	42	875	1	10.6	0.01	32.36
1 primi 2		H						
151	1	08/08/2018	43	876	1	10.7	0.01	30.31
1 primi 2		H						
151	1	08/09/2018	44	877	1	10.3	0	33.42
1 primi 2		H						
151	1	08/10/2018	45	878	1	10.4	0.02	32.03
1 primi 2		H						
151	1	08/11/2018	46	879	1	10.4	0	30.27
1 primi 2		H						
151	1	08/12/2018	47	880	1	11.3	0.05	32.01
1 primi 2		H						
151	1	08/13/2018	48	881	1	11.1	0.03	30.29
1 primi 2		H						
151	1	08/14/2018	49	882	1	10.9	0.01	32.33
1 primi 2		H						
151	1	08/15/2018	50	883	1	10.4	0.01	31.46
1 primi 2		H						
151	1	08/16/2018	51	884	1	9.9	0	31.1

1 primi 2		H						
151	1	08/17/2018	52	885	1	10	0	30.6
1 primi 2		H						
151	1	08/18/2018	53	886	1	9.9	0	32.33
1 primi 2		H						
151	1	08/19/2018	54	887	1	9.9	0	31.98
1 primi 2		H						
151	1	08/20/2018	55	888	1	10.4	0	30.69
1 primi 2		H						
151	1	08/21/2018	56	889	1	10.7	0	29.38
1 primi 2		H						
151	1	08/22/2018	57	890	1	10.9	0	30.9
1 primi 2		H						
151	1	08/23/2018	58	891	1	11.1	0	30.71
1 primi 2		H						
151	1	08/24/2018	59	892	1	10.3	0	32.49
1 primi 2		H						
151	1	08/25/2018	60	893	1	10.4	0	30.52
1 primi 2		H						
151	1	08/26/2018	61	894	1	11.1	0	32.97
1 primi 2		H						
151	1	08/27/2018	62	895	1	11.1	0	30.89
1 primi 2		H						
151	1	08/28/2018	63	896	1	10.3	0	34.72
1 primi 2		H						
151	1	08/29/2018	64	897	1	10.7	0	33.61
1 primi 2		H						
151	1	08/30/2018	65	898	1	10.3	0.16	32.84
1 primi 2		H						
151	1	08/31/2018	66	899	1	10.4	0	30.48
1 primi 2		H						
151	1	09/01/2018	67	900	1	9.2	0	34.45
1 primi 2		H						
151	1	09/02/2018	68	901	1	10.2	0	35.44
1 primi 2		H						
151	1	09/03/2018	69	902	1	10.9	0	34.84
1 primi 2		H						
151	1	09/04/2018	70	903	1	10.4	0	33.58
1 primi 2		H						
151	1	09/05/2018	71	904	1	10.9	0.14	34.36
1 primi 2		H						
151	1	09/06/2018	72	905	1	10.4	0	34.83
1 primi 2		H						
151	1	09/07/2018	73	906	1	10.2	0	28.17
1 primi 2		H						
151	1	09/08/2018	74	907	1	14	0.36	26.89
1 primi 2		H						
151	1	09/09/2018	75	908	1	10.2	0	25.52
1 primi 2		H						
151	1	09/10/2018	76	909	1	10.6	0.01	25.89
1 primi 2		H						
151	1	09/11/2018	77	910	1	10.4	0	26.27
1 primi 2		H						
151	1	09/12/2018	78	911	1	10.2	0	27.98

1 primi	2	H						
151	1	09/13/2018	79	912	1	11.1	0	30.17
1 primi	2	H						
151	1	09/14/2018	80	913	1	10.7	0	30.22
1 primi	2	H						
151	1	09/15/2018	81	914	1	9.5	0	29.32
1 primi	2	H						
151	1	09/16/2018	82	915	1	9.6	0	29.81
1 primi	2	H						
151	1	09/17/2018	83	916	1	10.7	0	26.18
1 primi	2	H						
151	1	09/18/2018	84	917	1	11.3	0	33.02
1 primi	2	H						
151	1	09/19/2018	85	918	1	11.1	0	28.57
1 primi	2	H						
151	1	09/20/2018	86	919	1	10.6	0	33.29
1 primi	2	H						
151	1	09/21/2018	87	920	1	11.4	0	32.43
1 primi	2	H						
151	1	09/22/2018	88	921	1	11.1	0	28.9
1 primi	2	H						
151	1	09/23/2018	89	922	1	11.3	0	29.68
1 primi	2	H						
151	1	09/24/2018	90	923	1	10.9	0.01	31.21
1 primi	2	H						
151	1	09/25/2018	91	924	1	10.4	0.36	28.87
1 primi	2	H						
151	1	09/26/2018	92	925	1	10.4	0	34.16
1 primi	2	H						
151	1	09/27/2018	93	926	1	11.1	0	30.44
1 primi	2	H						
151	1	09/28/2018	94	927	1	10.7	0	30.9
1 primi	2	H						
151	1	09/29/2018	95	928	1	11.1	0	30.93
1 primi	2	H						
151	1	09/30/2018	96	929	1	10	0	32.33
1 primi	2	H						
151	1	10/01/2018	97	930	1	10.7	0	30.2
1 primi	2	H						
151	1	10/02/2018	98	931	1	11.1	0	31.35
1 primi	2	H						
151	1	10/03/2018	99	932	1	12	0.01	30.78
1 primi	2	H						
151	1	10/04/2018	100	933	1	11.9	0.06	29.58
1 primi	2	H						
151	1	10/05/2018	101	934	1	10.6	0	28.44
1 primi	2	H						
151	1	10/06/2018	102	935	1	11.1	0	26.88
1 primi	2	H						
151	1	10/07/2018	103	936	1	11.4	0.01	30.71
1 primi	2	H						
151	1	12/02/2019	6	1357	2	9.2	0.11	38.39
2 multi	1	H						
151	1	12/03/2019	7	1358	2	9.5	0.07	39.48

2 multi	1	H						
151	1	12/04/2019	8	1359	2	9.5	0.03	39.28
2 multi	1	H						
151	1	12/05/2019	9	1360	2	9.3	0.01	39.94
2 multi	1	H						
151	1	12/06/2019	10	1361	2	9.5	0.32	40.15
2 multi	1	H						
151	1	12/07/2019	11	1362	2	8.7	0.03	41.21
2 multi	1	H						
151	1	12/08/2019	12	1363	2	8.8	0.02	41
2 multi	1	H						
151	1	12/09/2019	13	1364	2	9.2	0.01	40.72
2 multi	1	H						
151	1	12/10/2019	14	1365	2	8.8	0.01	41.57
2 multi	1	H						
151	1	12/11/2019	15	1366	2	8.9	0.03	41.54
2 multi	1	H						
151	1	12/12/2019	16	1367	2	9.1	0.01	44.02
2 multi	1	H						
151	1	12/13/2019	17	1368	2	8.8	0.04	45.55
2 multi	1	H						
151	1	12/14/2019	18	1369	2	9.1	0.04	46.21
2 multi	1	H						
151	1	12/15/2019	19	1370	2	9.1	0.02	44.24
2 multi	1	H						
151	1	12/16/2019	20	1371	2	9.1	0.08	48.01
2 multi	1	H						
151	1	12/17/2019	21	1372	2	9.1	0	46.2
2 multi	1	H						
151	1	12/18/2019	22	1373	2	8.9	0.02	46.62
2 multi	2	H						
151	1	12/19/2019	23	1374	2	8.9	0.02	43.47
2 multi	2	H						
151	1	12/20/2019	24	1375	2	9.1	0	46.57
2 multi	2	H						
151	1	12/21/2019	25	1376	2	9.3	0	46.21
2 multi	2	H						
151	1	12/22/2019	26	1377	2	8.9	0.01	46.67
2 multi	2	H						
151	1	12/23/2019	27	1378	2	9.1	0.25	46.34
2 multi	2	H						
151	1	12/24/2019	28	1379	2	9.2	0	45.03
2 multi	2	H						
151	1	12/25/2019	29	1380	2	9.1	0	46.71
2 multi	2	H						
151	1	12/26/2019	30	1381	2	9.1	0.01	49.81
2 multi	2	H						
151	1	12/27/2019	31	1382	2	9.1	0.01	45.12
2 multi	2	H						
151	1	12/28/2019	32	1383	2	9.2	0	45.97
2 multi	2	H						
151	1	12/29/2019	33	1384	2	9.3	0	45.55
2 multi	2	H						
151	1	12/30/2019	34	1385	2	9.3	0	50.72

2 multi 2		H						
151	1	12/31/2019	35	1386	2	9.5	0.02	43
2 multi 2		H						
151	1	01/01/2020	36	1387	2	9.2	0.01	46.85
2 multi 2		H						
151	1	01/02/2020	37	1388	2	9.5	0	48.01
2 multi 2		H						
151	1	01/03/2020	38	1389	2	9.3	0.04	45.46
2 multi 2		H						
151	1	01/04/2020	39	1390	2	9.5	0	47.11
2 multi 2		H						
151	1	01/05/2020	40	1391	2	9.6	0.02	46.8
2 multi 2		H						
151	1	01/06/2020	41	1392	2	9.8	0	47.22
2 multi 2		H						
151	1	01/07/2020	42	1393	2	9.6	0.02	44.23
2 multi 2		H						
151	1	01/08/2020	43	1394	2	9.5	0	46.54
2 multi 2		H						
151	1	01/09/2020	44	1395	2	10	0.01	43.71
2 multi 2		H						
151	1	01/10/2020	45	1396	2	10	0	49.05
2 multi 2		H						
151	1	01/11/2020	46	1397	2	10.2	0	46.87
2 multi 2		H						
151	1	01/12/2020	47	1398	2	10	0	45.73
2 multi 2		H						
151	1	01/13/2020	48	1399	2	10	0.01	48.91
2 multi 2		H						
151	1	01/14/2020	49	1400	2	9.9	0.01	48.21
2 multi 2		H						
151	1	01/15/2020	50	1401	2	9.8	0.14	49.4
2 multi 2		H						
151	1	01/16/2020	51	1402	2	9.6	0.02	46.28
2 multi 2		H						
151	1	01/17/2020	52	1403	2	9.1	0	44.26
2 multi 2		H						
151	1	01/18/2020	53	1404	2	9.5	0	48.08
2 multi 2		H						
151	1	01/19/2020	54	1405	2	8.8	0	46.08
2 multi 2		H						
151	1	01/20/2020	55	1406	2	9.2	0	46.07
2 multi 2		H						
151	1	01/21/2020	56	1407	2	9.2	0	44.28
2 multi 2		H						
151	1	01/22/2020	57	1408	2	9.6	0	47.83
2 multi 2		H						
151	1	01/23/2020	58	1409	2	9.1	0	45.66
2 multi 2		H						
151	1	01/24/2020	59	1410	2	9.3	0	44.69
2 multi 2		H						
151	1	01/25/2020	60	1411	2	9.3	0	44.81
2 multi 2		H						
151	1	01/26/2020	61	1412	2	9.6	0.01	46.45

2 multi 2		H						
151	1	01/27/2020	62	1413	2	9.5	0.12	47.19
2 multi 2		H						
151	1	01/28/2020	63	1414	2	9.5	0	45.46
2 multi 2		H						
151	1	01/29/2020	64	1415	2	10.6	0.02	44.07
2 multi 2		H						
151	1	01/30/2020	65	1416	2	9.6	0.01	44.62
2 multi 2		H						
151	1	01/31/2020	66	1417	2	10.3	0	51.49
2 multi 2		H						
151	1	02/01/2020	67	1418	2	10	0	46.8
2 multi 2		H						
151	1	02/02/2020	68	1419	2	11.3	0.03	43.34
2 multi 2		H						
151	1	02/03/2020	69	1420	2	11.1	0.03	48.38
2 multi 2		H						
151	1	02/04/2020	70	1421	2	9.9	0.01	46.85
2 multi 2		H						
151	1	02/05/2020	71	1422	2	10.3	0	48.04
2 multi 2		H						
151	1	02/06/2020	72	1423	2	11.3	0.06	41.7
2 multi 2		H						
151	1	02/07/2020	73	1424	2	9.3	0.03	43.59
2 multi 2		H						
151	1	02/08/2020	74	1425	2	10	0	45.74
2 multi 2		H						
151	1	02/09/2020	75	1426	2	9.8	0.01	44.73
2 multi 2		H						
151	1	02/10/2020	76	1427	2	10.3	0	42.62
2 multi 2		H						
151	1	02/11/2020	77	1428	2	9.6	0	46.8
2 multi 2		H						
151	1	02/12/2020	78	1429	2	9.6	0.01	46.42
2 multi 2		H						
151	1	02/13/2020	79	1430	2	9.6	0.27	43.24
2 multi 2		H						
151	1	02/14/2020	80	1431	2	10.3	0	44.93
2 multi 2		H						
151	1	02/15/2020	81	1432	2	9.1	0	44.88
2 multi 2		H						
151	1	02/16/2020	82	1433	2	9.3	0	43.28
2 multi 2		H						
151	1	02/17/2020	83	1434	2	9.6	0	47.95
2 multi 2		H						
151	1	02/18/2020	84	1435	2	9.6	0	40.43
2 multi 2		H						
151	1	02/19/2020	85	1436	2	9.5	0.19	47.59
2 multi 2		H						
151	1	02/20/2020	86	1437	2	9.9	0	43.58
2 multi 2		H						
151	1	02/21/2020	87	1438	2	9.6	0	45.85
2 multi 2		H						
151	1	02/22/2020	88	1439	2	9.8	0	45.08

2 multi	2	H						
151	1	02/23/2020	89	1440	2	9.8	0.01	42.22
2 multi	2	H						
151	1	02/24/2020	90	1441	2	9.8	0.01	43.92
2 multi	2	H						
151	1	02/25/2020	91	1442	2	9.6	0	46.69
2 multi	2	H						
151	1	02/26/2020	92	1443	2	10	0	44.44
2 multi	2	H						
151	1	06/30/2020	217	1568	2	9.3	0	35.78
2 multi	2	H						
151	1	07/01/2020	218	1569	2	10.3	0	38.62
2 multi	2	H						
151	1	07/02/2020	219	1570	2	9.5	0	35.94
2 multi	2	H						
151	1	07/03/2020	220	1571	2	10	0	36.97
2 multi	2	H						
151	1	07/04/2020	221	1572	2	9.8	0.12	38.2
2 multi	2	H						
151	1	07/05/2020	222	1573	2	9.9	0.1	38.75
2 multi	2	H						
151	1	07/06/2020	223	1574	2	9.9	0.12	37.56
2 multi	2	H						
151	1	07/07/2020	224	1575	2	10	0	36.72
2 multi	2	H						
151	1	07/08/2020	225	1576	2	9.9	0	39.16
2 multi	2	H						
151	1	07/09/2020	226	1577	2	10.4	0.02	38.45
2 multi	2	H						
151	1	07/10/2020	227	1578	2	10.3	0.04	38.61
2 multi	2	H						
151	1	07/11/2020	228	1579	2	9.9	0.18	36.06
2 multi	2	H						
151	1	07/12/2020	229	1580	2	10.3	0.11	34.77
2 multi	2	H						
151	1	07/13/2020	230	1581	2	9.9	0.09	35.65
2 multi	2	H						
151	1	07/14/2020	231	1582	2	10	0	39.41
2 multi	2	H						
151	1	07/15/2020	232	1583	2	9.8	0.04	35.53
2 multi	2	H						
151	1	07/16/2020	233	1584	2	9.6	0	36.33
2 multi	2	H						
151	1	07/17/2020	234	1585	2	9.8	0	36.11
2 multi	2	H						
151	1	07/18/2020	235	1586	2	9.2	0.03	37.35
2 multi	2	H						
151	1	07/19/2020	236	1587	2	10.4	0.03	39.59
2 multi	2	H						
151	1	07/20/2020	237	1588	2	10	0.04	34.47
2 multi	2	H						
151	1	07/21/2020	238	1589	2	9.9	0.03	33.99
2 multi	2	H						
151	1	07/22/2020	239	1590	2	9.3	0.01	34.35

2 multi	2	H							
151	1	07/23/2020	240	1591	2	10	0.03	35.33	
2 multi	2	H							
151	1	07/24/2020	241	1592	2	9.8	0	34.93	
2 multi	2	H							
151	1	07/25/2020	242	1593	2	9.9	0	31.71	
2 multi	2	H							
151	1	07/26/2020	243	1594	2	9.2	0.1	29.87	
2 multi	2	H							
151	1	07/27/2020	244	1595	2	9.8	0	33.05	
2 multi	2	H							
151	1	07/28/2020	245	1596	2	10.3	0.3	28.65	
2 multi	2	H							
151	1	07/29/2020	246	1597	2	10.6	0.17	28	
2 multi	2	H							
151	1	07/30/2020	247	1598	2	10.2	0.02	35.27	
2 multi	2	H							
151	1	07/31/2020	248	1599	2	9.6	0.19	35.6	
2 multi	2	H							
151	1	08/01/2020	249	1600	2	9.6	0.04	34.97	
2 multi	2	H							
151	1	08/02/2020	250	1601	2	9.5	0.14	34.99	
2 multi	2	H							
151	1	08/03/2020	251	1602	2	9.8	0	35.65	
2 multi	2	H							
151	1	08/04/2020	252	1603	2	10	0.05	32.98	
2 multi	2	H							
151	1	08/05/2020	253	1604	2	10.2	0.05	33.25	
2 multi	2	H							
151	1	08/06/2020	254	1605	2	10.3	0.04	35.19	
2 multi	2	H							
151	1	08/07/2020	255	1606	2	9.8	0.01	34.99	
2 multi	2	H							
151	1	08/08/2020	256	1607	2	10	0.23	30.8	
2 multi	2	H							
151	1	08/09/2020	257	1608	2	10.3	0.03	34.07	
2 multi	2	H							
151	1	11/20/2020	4	1711	3	9.5	0.23	38.82	
2 multi	1	H							
151	1	11/21/2020	5	1712	3	9.5	0.16	44.55	
2 multi	1	H							
151	1	11/22/2020	6	1713	3	9.3	0.13	45.02	
2 multi	1	H							
151	1	11/23/2020	7	1714	3	8.9	0.1	43.47	
2 multi	1	H							
151	1	11/24/2020	8	1715	3	9.5	0.1	41.79	
2 multi	1	H							
151	1	11/25/2020	9	1716	3	9.2	0.08	46.96	
2 multi	1	H							
151	1	11/26/2020	10	1717	3	9.1	0.15	44.95	
2 multi	1	H							
151	1	11/27/2020	11	1718	3	8.9	0.04	48.85	
2 multi	1	H							
151	1	11/28/2020	12	1719	3	9.1	0.59	47.78	

2 multi	1	H						
151	1	11/29/2020	13	1720	3	8.9	0.64	47.27
2 multi	1	H						
151	1	11/30/2020	14	1721	3	8.7	0.01	43.62
2 multi	1	H						
151	1	12/01/2020	15	1722	3	9.1	0.85	49.34
2 multi	1	H						
151	1	12/02/2020	16	1723	3	9.1	0.08	44.51
2 multi	1	H						
151	1	12/03/2020	17	1724	3	8.9	0.09	52.41
2 multi	1	H						
151	1	12/04/2020	18	1725	3	8.8	0.07	48.63
2 multi	1	H						
151	1	12/05/2020	19	1726	3	8.9	0.07	53.75
2 multi	1	H						
151	1	12/06/2020	20	1727	3	8.9	0.1	52.15
2 multi	1	H						
151	1	12/07/2020	21	1728	3	8.9	0.08	53.99
2 multi	1	H						
151	1	12/08/2020	22	1729	3	9.1	0.06	50.4
2 multi	2	H						
151	1	12/09/2020	23	1730	3	8.8	0.07	50.74
2 multi	2	H						
151	1	12/10/2020	24	1731	3	8.9	0.08	52.67
2 multi	2	H						
151	1	12/11/2020	25	1732	3	8.9	0.07	56.68
2 multi	2	H						
151	1	12/12/2020	26	1733	3	8.9	0.05	52.43
2 multi	2	H						
151	1	12/13/2020	27	1734	3	8.9	0.04	54.64
2 multi	2	H						
151	1	12/14/2020	28	1735	3	9.2	0.07	53.59
2 multi	2	H						
151	1	12/15/2020	29	1736	3	10.3	0	43.23
2 multi	2	H						
151	1	12/16/2020	30	1737	3	10.6	0	40.45
2 multi	2	H						
151	1	12/17/2020	31	1738	3	10.2	0.03	45.73
2 multi	2	H						
151	1	12/18/2020	32	1739	3	9.9	0.09	46.11
2 multi	2	H						
151	1	12/19/2020	33	1740	3	9.6	0.07	49.97
2 multi	2	H						
151	1	12/20/2020	34	1741	3	10	0.07	51.75
2 multi	2	H						
151	1	12/21/2020	35	1742	3	11.7	0	43.5
2 multi	2	H						
151	1	12/22/2020	36	1743	3	11.9	0	35.41
2 multi	2	H						
151	1	12/23/2020	37	1744	3	11.7	0	41.9
2 multi	2	H						
151	1	12/24/2020	38	1745	3	10.6	0.03	42.02
2 multi	2	H						
151	1	12/25/2020	39	1746	3	10.9	0.11	51.19

2 multi	2	H						
151	1	12/26/2020	40	1747	3	10.9	0.03	48.49
2 multi	2	H						
151	1	12/27/2020	41	1748	3	10.6	0.34	45.92
2 multi	2	H						
151	1	12/28/2020	42	1749	3	9.9	0.11	44.96
2 multi	2	H						
151	1	12/29/2020	43	1750	3	10.4	0.11	49.5
2 multi	2	H						
151	1	12/30/2020	44	1751	3	10.6	0.04	47.2
2 multi	2	H						
151	1	12/31/2020	45	1752	3	10.3	0.07	50.06
2 multi	2	H						
151	1	01/01/2021	46	1753	3	10.4	0.09	46.69
2 multi	2	H						
151	1	01/02/2021	47	1754	3	9.6	0.06	48
2 multi	2	H						
151	1	01/03/2021	48	1755	3	9.9	0.18	47.16
2 multi	2	H						
151	1	01/04/2021	49	1756	3	11.1	0.04	47.77
2 multi	2	H						
151	1	01/05/2021	50	1757	3	10.7	0	42.84
2 multi	2	H						
151	1	01/06/2021	51	1758	3	10.2	0.09	48.39
2 multi	2	H						
151	1	01/07/2021	52	1759	3	10.7	0.09	45.5
2 multi	2	H						
151	1	01/08/2021	53	1760	3	12.3	0.09	44.2
2 multi	2	H						
151	1	01/09/2021	54	1761	3	11.7	0.3	45.08
2 multi	2	H						
151	1	01/10/2021	55	1762	3	12.6	0.16	41.31
2 multi	2	H						
151	1	01/11/2021	56	1763	3	12	0.27	37.15
2 multi	2	H						
151	1	01/12/2021	57	1764	3	12.6	0.31	35.53
2 multi	2	H						
151	1	01/13/2021	58	1765	3	11.7	0.25	31.69
2 multi	2	H						
151	1	01/14/2021	59	1766	3	12	0.24	30.25
2 multi	2	H						
151	1	01/15/2021	60	1767	3	12.3	0.4	36.69
2 multi	2	H						
151	1	01/16/2021	61	1768	3	11.1	0.06	37.82
2 multi	2	H						
151	1	01/17/2021	62	1769	3	10.7	0.13	37.13
2 multi	2	H						
151	1	01/18/2021	63	1770	3	10.6	0.24	38.06
2 multi	2	H						
151	1	01/19/2021	64	1771	3	10.4	0.13	36.67
2 multi	2	H						
151	1	01/20/2021	65	1772	3	10.9	0.01	38.17
2 multi	2	H						
151	1	01/21/2021	66	1773	3	10.9	0.12	33.77

2 multi 2		H						
151	1	01/22/2021	67	1774	3	11.1	0	37.52
2 multi 2		H						
151	1	01/23/2021	68	1775	3	10.4	0.07	32.94
2 multi 2		H						
151	1	01/24/2021	69	1776	3	9.6	0.07	34.12
2 multi 2		H						
[truncated: 428,069 more chars]
